# Circulating proteins as key genetically proxied mediators in the pathway from smoking to intracranial aneurysm disease: A mediation Mendelian randomization analysis

**DOI:** 10.18332/tid/226836

**Published:** 2026-09-21

**Authors:** Yaqi Liu, Hao Yuan, Chi Huang, Jiwan Huang, Can Li, Runze Ge, Jiancheng Lin, Xiru Zhang, Mengshi Huang, Xin Feng, Chuan Zhi Duan

**Affiliations:** 1 Department of Cerebrovascular Surgery, The Third Affiliated Hospital of Sun Yat-sen University, Guangzhou, China; 2 Neurosurgery Center, Department of Cerebrovascular Surgery, Engineering Technology Research Center of Education Ministry of China on Diagnosis and Treatment of Cerebrovascular Disease, Zhujiang Hospital, Southern Medical University, Guangzhou, China

**Keywords:** intracranial aneurysm, smoking, Mendelian randomization, proteomics

## Abstract

**INTRODUCTION:**

The molecular mechanisms linking smoking to intracranial aneurysmal disease remain elusive. In this study, we aimed to employ Mendelian randomization (MR) analysis to identify proteomic pathways that mediate this relationship.

**METHODS:**

We conducted a two-step, two-sample MR study using summary-level GWAS data from the GSCAN (GWAS & Sequencing Consortium of Alcohol and Nicotine Use, release up to 2019) and the FinnGen study (release R12, 2024). In the first step, we estimated the effects of genetically proxied smoking, using the Lifetime Smoking Index (LSI) as the primary exposure, on circulating protein levels. In the second step, we assessed the associations of smoking-related proteins with the risk of unruptured intracranial aneurysms (uIAs) and aneurysmal subarachnoid hemorrhage (aSAH). Proteins that showed significant associations in both steps were considered potential mediators, and we calculated their mediation proportions using the product-of-coefficients method.

**RESULTS:**

Genetically proxied smoking was associated with increased risks of uIAs and aSAH. Each one standard deviation increase in LSI was associated with a 2.99-fold higher risk of uIAs (OR=2.99; 95% CI: 1.77–5.06) and a 2.51-fold higher risk of aSAH (OR=2.51; 95% CI: 1.72–3.66). After multiple-testing correction, LSI was causally associated with 427 circulating proteins; 22 correlated with aSAH and 22 with uIAs, with eight shared. Mediation analysis confirmed six mediators (CD74, HEG1, YAP1, FGF23, SFRP1, PGF) for the LSI-aSAH pathway and four (CD74, YAP1, ANGPT2, RTN4R) for the LSI-uIA pathway. CD74 and YAP1 were shared mediators with the highest mediation ratios.

**CONCLUSIONS:**

Eight distinct circulating proteins mediate smoking-induced intracranial aneurysm development and rupture via independent biological cascades. CD74 and YAP1 may serve as priority candidate mediators for future mechanistic and translational investigations aimed at refining risk stratification in smoking-related intracranial aneurysms.

## INTRODUCTION

The two hallmark manifestations of intracranial aneurysmal (IA) disease are unruptured intracranial aneurysms (uIAs) and aneurysmal subarachnoid hemorrhage (aSAH). uIAs, characterized by permanent localized protuberance of the cerebral arteries, are a highly prevalent cerebrovascular disorder with a global prevalence of 3–5%, carrying substantial morbidity risk^1,2^. Evidence from the International Study Group of Unruptured Intracranial Aneurysm (ISUIA) study indicates that most aneurysms remain asymptomatic, with the risk of rupture primarily influenced by aneurysm location and size^3^. As the most catastrophic outcome of uIAs, the collapse of aneurysm wall stability – resulting from the interplay of multiple factors – culminates in aSAH with a case-fatality rate approaching 50%. The high morbidity and mortality associated with this disease are partly due to the absence of effective preventive measures. Although previous studies have proposed various hypotheses for the formation and progression of aSAH and uIAs – such as hemodynamic stress, inflammatory storms, immune injury, and metabolic burden – no consensus has yet been reached^4-7^.

Smoking, as a modifiable adverse lifestyle habit, is strongly associated with both the development and progression of uIAs and aSAH. Data show that individuals with a smoking history have a fourfold higher risk of IA than non-smokers. Current smokers have a 2.5-fold higher risk of rupture than non-smoking patients with IA^8^. One previous study found that the global incidence of SAH is declining as smoking prevalence decreases^9^. Clinical studies have also highlighted a correlation between smoking and an increased incidence of IA and its rupture^10^. The mechanisms underlying the association between smoking and uIA/aSAH development center on vascular inflammation, oxidative stress, and endothelial dysfunction. These pathological changes collectively promote the initiation and growth of intracranial aneurysms, with nicotine serving as the key causative constituent^11^. However, the precise molecular pathways through which smoking predisposes to uIAs and aSAH remain unclear and warrant deeper investigation. Notably, most previous studies have been observational, and establishing causal links between risk factors and outcomes has consistently been challenging due to residual confounding (e.g. hypertension, alcohol consumption). Although some studies have provided strong evidence supporting causal effects of modifiable lifestyle factors, such as smoking, hypertension, and insomnia, on the risk of uIAs and aSAH, and others have suggested that circulating plasma proteins may serve as therapeutic targets for IAs, there remains a lack of robust evidence that integrates smoking, plasma proteins, and the potential pathogenic mechanisms connecting them to uIAs and aSAH^12,13^. A clearer understanding of these mechanisms could unveil novel therapeutic targets or biomarkers – particularly for individuals with a smoking history.

Traditional clinical investigation protocols often fail to capture the full complexity of smoking-induced intracranial aneurysm disease due to their limited data scale and observational dimensions, thereby creating a key barrier to identifying robust biomarkers and therapeutic targets that can guide clinical intervention. Substantial progress in omics technologies – including Genome-Wide Association Studies (GWAS), Proteome-Wide Association Studies (PWAS), and TranscriptomeWide Association Studies (TWAS) – has been made in recent years, offering novel insights into disease mechanisms and targeted therapy^14^. Here, we applied an integrative two-step network MR framework combining genomics and proteomics to address three key unmet needs: first, to examine the possible causal relationship between genetically proxied smoking behavior and the risk of uIAs and aSAH; second, to identify plasma proteins associated with disease risk; and third, to determine the circulating proteins that possibly mediate the causal effects of smoking on uIAs and aSAH risk, thereby providing a basis for the precise prevention and treatment of intracranial aneurysm disease in high-risk smokers.

## METHODS

### Study design

This study adopted a two-step MR design based on summary-level GWAS data from the GWAS & Sequencing Consortium of Alcohol and Nicotine Use (GSCAN) (2019 release) and the FinnGen database (R12 release, 2024). Strictly adhering to the STROBE-MR guidelines, we adapted the analytical protocol of Yuan et al.^15^ to investigate the causal relationship between genetically predicted smoking exposure and the genetically proxied risk of aSAH and uIAs. Our network MR analysis involved three key steps. First, we quantified the overall effect of genetically proxied smoking exposure on the risk of aSAH and uIAs. Next, we assessed which circulating protein levels are influenced by such exposure, and finally we examined the relationship between these genetically proxied smoking-related proteins and the risk of aSAH and uIAs. Within this analytical framework, we aligned the directions of effect and quantified the proportion of the effect mediated by each protein. For candidate proteins with potential mediating effects, we performed a protein association network investigation to delineate their synergistic patterns. For candidate proteins with completely independent pathological effects, we conducted a Phenome-Wide Association Study (PheWAS) to screen for the full phenome-wide profile associated with their expression levels. Those candidate circulating proteins without extensive potential phenotypic associations were identified as key proteins with independent pathogenic roles and are recommended as potential therapeutic targets for intracranial aneurysm disease in future studies.

### Summary data of smoking traits

The Lifetime Smoking Index (LSI) served as the primary measure of smoking exposure, while smoking initiation and cigarettes per day were used as secondary measures in validation analyses. The LSI is a composite measure that captures an individual’s overall smoking exposure, incorporating both the intensity and duration of smoking. Summary-level data were derived from the GWAS meta-analysis conducted by Wootton et al.^16^ in 462690 individuals from the UK Biobank (2019 release). Information on smoking initiation, duration, cigarettes per day, and cessation was combined with body weight in a linear mixed model that accounted for population stratification and relatedness; the LSI was calculated using the formula referenced in Rachet et al.^17^. GWAS data for smoking initiation and cigarettes per day included 805431 and 326497 individuals, respectively, from the GSCAN (2022 release)^18^.

### Summary data of uIAs and aSAH

To determine how smoking exposure influences the risk of uIAs and aSAH, we conducted multiple replication analyses using summary-level outcome data from two principal sources. First, for the primary analysis, we accessed the latest FinnGen R12 release and extracted aSAH cases from ‘finngen_R12_I9_SAHANEUR.gz’ (6849 patients) and uIAs cases from ‘finngen_R12_I9_ANEURYSM.gz’ (3310 patients)^19^. The data of FinnGen R12 have been made publicly available to the entire network on 4 November 2024. Any researcher can freely download and use it without any restrictions. In the replication analysis, summary-level data for uIAs and aSAH were derived from the meta-analysis of 23 European cohorts by Bakker et al.^20^, comprising 79429 participants (7495 cases: 69% ruptured IAs, 28% unruptured, 3.8% unknown rupture status; 71934 controls). It has been made public to the entire network in December 2020.

### Summary data of circulating proteins (pQTL)

Protein Quantitative Trait Loci (pQTL) are genetic variants associated with inter-individual differences in circulating protein abundance. Summary-level data on circulating protein levels were obtained from the two largest and most authoritative proteomic resources currently available: the UK Biobank Pharma Proteomics Project (UKB-PPP) and the deCODE study^21,22^. The UKB-PPP quantified 2923 unique circulating proteins using the Olink platform in 54219 participants (2023 release), while the deCODE study detected over 4970 plasma proteins via the SomaScan platform in 35559 Icelandic individuals (2021 release). For different peptides derived from the same coding gene, both databases annotated them as distinct protein entities, and we only retained the one with a stronger association with the lifetime smoking index. Given the larger sample size of UKB-PPP, the overlapping proteins between the two datasets were derived from UKB-PPP. We categorized the proteins measured across cohorts into three distinct datasets: Set 1 comprised proteins unique to UKB-PPP, Set 2 comprised proteins exclusive to deCODE, and Set 3 comprised proteins available in both UKB-PPP and deCODE. This classification scheme enabled the application of tailored sensitivity and replication analyses specific to each protein set. The final analysis included 5764 proteins. Supplementary file Table S1 presents the names and corresponding coding genes of these unique proteins. Notably, FinnGen proteomics data were not incorporated herein. Consistent with deCODE, the study profiled 3892 proteins using SomaScan but included only 10708 subjects, which limited its sample size^23^.

### Phenome framework and PheWAS analysis

To ensure alignment with the most widely adopted contemporary standards, our PheWAS analyzed key candidate proteins using the AstraZeneca PheWAS portal (https://azphewas.com/) to evaluate the pleiotropic effects and potential adverse reactions of putative therapeutic targets (the default suggestive significance threshold of 1×10^-6^ and genome-wide significance threshold of 1×10^-8^). This portal captures the largest phenotypic landscape from the UK Biobank exome-sequencing subgroup, encompassing approximately 17361 binary phenotypes and 1419 continuous phenotypes. These data provide a broad perspective for large-scale exploration of protein–phenotype associations and enable comprehensive analysis of cross-phenotype relationships.

### Statistical analysis

All MR analyses were conducted using the *TwoSampleMR* package (version 0.5.7)^24^. To enhance statistical power, we established a uniform set of criteria for instrumental variables (IVs) selection in this study: 1) only single nucleotide polymorphisms (SNPs) demonstrating genome-wide significant associations with smoking exposure (p<5×10^-8^) were included; 2) all selected IVs exhibited F-statistics >10 to minimize weak instrument bias; and 3) SNPs in linkage disequilibrium (LD) were pruned using an R² threshold of <0.001 within a 10000 kb (10 Mb) window. Following the initial quality screening, SNPs retained were subsequently checked against the GWAS Catalog, and any with reported links to uIAs and aSAH or to potential confounding conditions (e.g. obesity, hyperlipidemia, diabetes, hypertension) were removed. We ultimately included 118 SNPs for the LSI, 234 SNPs for smoking initiation, and 53 SNPs for cigarettes per day as IVs to evaluate the causal associations between genetically proxied smoking exposure and the risks of uIAs and aSAH (Supplementary file Tables S2–S4).

In the two-sample MR analyses, inverse-variance-weighted (IVW) regression was used as the primary method to estimate: 1) the overall associations between genetically proxied smoking exposure and genetically proxied uIAs/aSAH (βtotal); and 2) the associations between smoking exposure and circulating protein levels (β1). Random-effects IVW models were used when significant heterogeneity among IVs was detected; otherwise, fixed-effects IVW models were employed^25^. The Steiger directionality test was used to confirm that the causal direction runs from exposure to outcome, rather than reverse causation or an association attributable solely to confounding factors. In addition, we incorporated MR with Robust Adjusted Profile Score (MR-RAPS), a recently developed refinement designed to mitigate biases that can arise in conventional two-sample MR analyses^26^. Estimates were further corroborated by four complementary approaches: weighted-median, MR-Egger, weighted-mode, and simple-mode. The Cochran Q test was used to measure the heterogeneity of the association, and the MR-Egger intercept test was used to examine the horizontal pleiotropy. Scatter plots were employed to visualize the data, and the leave-one-out method was utilized to sequentially exclude individual SNPs to assess their influence on the results.

Summary-data-based MR (SMR) was employed to estimate the associations between genetically proxied smoking-influenced circulating proteins and the risks of uIAs and aSAH (β2)^27^. The analysis was based on genetic variants in the cis gene region (±1 MB) for each protein. Following SMR analysis, we conducted the Heterogeneity In Dependent Instruments (HEIDI) test to explore potential heterogeneity in SMR association statistics^28^. This approach tests whether multiple SNPs in linkage disequilibrium exhibit consistent effects, rejecting the causal hypothesis if significant heterogeneity is detected. For proteins without genome-wide significant cis-pQTL, the strongest cis signal was used as the instrumental variable.

To define a formal mediation pathway, we required directional concordance between the total effect of smoking on intracranial aneurysms (βtotal) and the product of the two step-specific effects (β1 × β2). Specifically, a pathway was considered to be a positive mediator if both βtotal and β1 × β2 were positive (i.e. smoking increased the protein, which in turn increased disease risk), and a negative mediator if both were negative (i.e. smoking decreased the protein, which in turn decreased disease risk). This directional alignment is a critical assumption in two-step MR mediation analyses, as it ensures that the indirect effect operates in the same direction as the overall relationship, thereby supporting a biologically coherent mediating pathway^29^. For proteins where the direction of β1 × β2 was discordant with βtotal (i.e. one positive and the other negative), we did not classify them as formal mediators. In such cases, the indirect effect would counteract the total effect, suggesting either that the protein does not mediate the relationship or that the observed association is influenced by pleiotropy or reverse causation. These discordant findings are nevertheless reported in the Supplementary file for transparency. A protein-protein interaction (PPI) network among the proteins involved in these pathways was visualized using the STRING database (https://cn.string-db.org/). To account for potential pleiotropy, we exclusively employed non-pleiotropic pQTL (i.e. pQTL not associated with any other proteins) as genetic instruments for the protein mediators. The standard errors (SEs) and 95% confidence intervals (CIs) for the indirect effects were calculated using the delta method.

Through knowledge extraction from the AstraZeneca online portal, we disclosed any possible protein–phenotype association networks. Finally, mutually independent mediator proteins were confirmed as potential targets for smoking intervention, excluding any potentially pleiotropic proteins and protein cascades. We also performed Gene Ontology (GO) and Kyoto Encyclopedia of Genes and Genomes (KEGG) enrichment analyses on the potential candidate mediator proteins to explore the possible signaling pathways and biological functions of the key molecules.

The validity of our two-sample MR analysis rests on three core instrumental variable assumptions. First, the relevance assumption requires that genetic instruments are strongly associated with the exposure; we ensured this by selecting genome-wide significant SNPs (p<5×10^-8^) and confirmed instrument strength using F-statistics, with all values >10. Second, the independence assumption requires that instruments are not associated with confounders of the exposure-outcome relationship; given the random assortment of alleles at conception, germline variants are generally independent of environmental and lifestyle confounders, and we further cross-referenced all selected SNPs against the GWAS Catalog to exclude variants linked to uIAs, aSAH, or potential confounding conditions (e.g. obesity, hyperlipidemia, diabetes, hypertension). Third, the exclusion restriction assumption requires that instruments affect the outcome only through the exposure and not via independent pathways (i.e. absence of horizontal pleiotropy); we assessed this using multiple complementary approaches, including the MR-Egger intercept test, Cochran’s Q test for heterogeneity, HEIDI test, and corroboration of estimates across different sensitivity estimators (weighted-median, MR-RAPS, weighted-mode, and simple-mode). In addition, for the protein mediation step, we exclusively used non-pleiotropic pQTL to further safeguard against pleiotropic bias.

## RESULTS

### Smoking exposure and risk of disease

**Figure 1:**
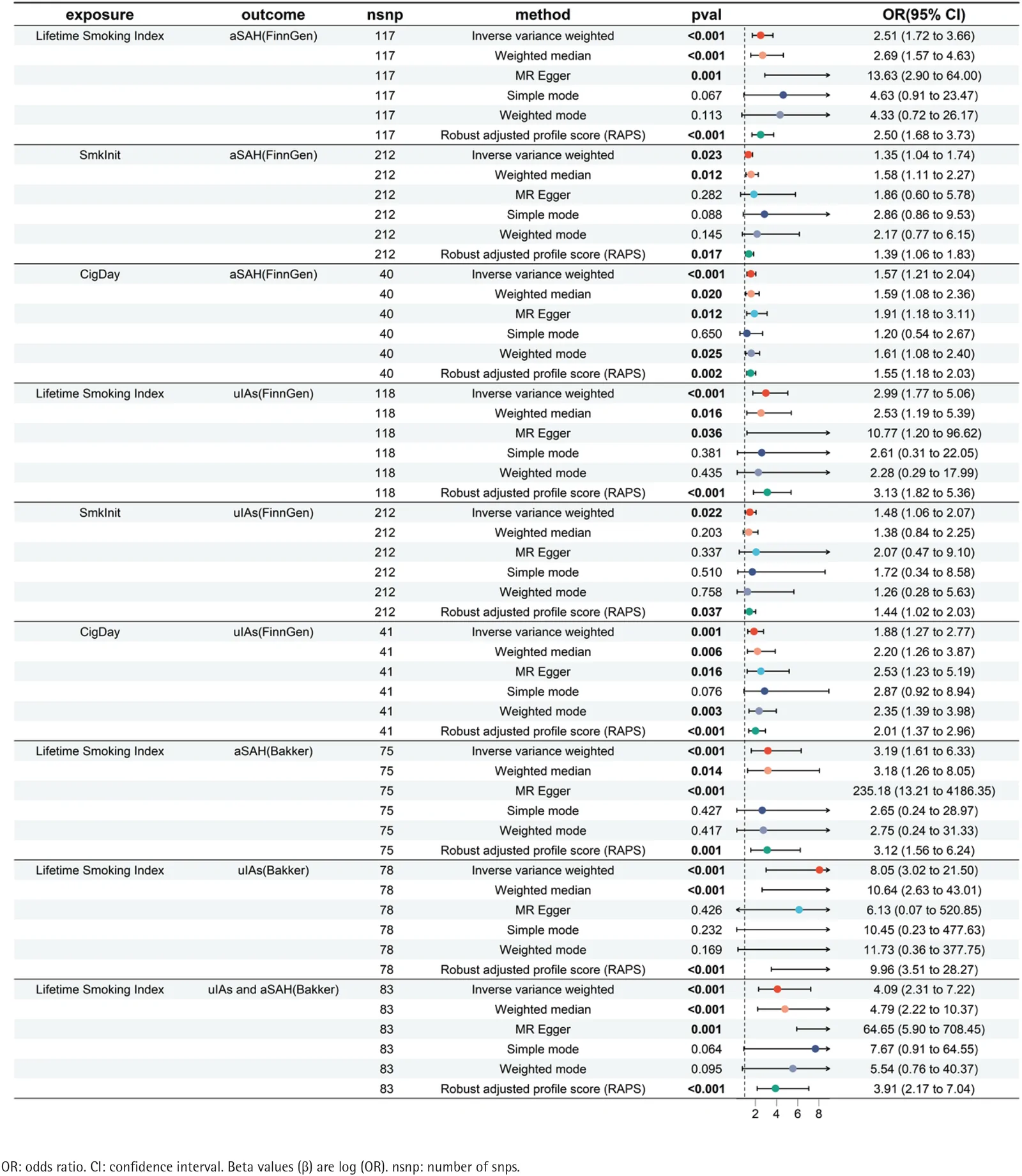
Forest plot of the causal associations between genetically proxied smoking exposure and risk of intracranial aneurysmal disease (unruptured intracranial aneurysms and aneurysmal subarachnoid hemorrhage), evaluated using six Mendelian randomization methods. The inverse variance weighted and MRRobust adjusted profile score methods were used as the primary analyses

Genetically proxied LSI was significantly associated with increased risks of both uIAs and aSAH. Each 1-SD increase in the genetically proxied lifetime smoking index was associated with a 2.51-fold higher risk of aSAH (OR=2.51; 95% CI: 1.72–3.66, p<0.001) and a 2.99-fold higher risk of uIAs (OR=2.99; 95% CI: 1.77–5.06, p<0.001). These associations remained consistent across sensitivity analyses and replication analyses using Smoking Initiation (SmkInit) and Cigarettes per Day (CigDay) as exposures. In the external validation cohort provided by Bakker et al.^20^, even greater risk increases were observed: 3.19-fold for aSAH (OR=3.19; 95% CI: 1.61–6.33, p<0.001), 8.05-fold for uIAs (OR=8.05; 95% CI: 3.02–21.50, p<0.001), and 4.09-fold for overall aneurysmal disease (OR=4.09; 95% CI: 2.31–7.22, p<0.001). We summarize key results in Figure 1. Detailed results of the MR analyses are provided in Supplementary file Table S5. Although sensitivity analyses indicated potential horizontal pleiotropy in the causal associations between the genetically proxied LSI and aSAH risk (MR-Egger intercept = -0.02, p=0.029 for the FinnGen GWAS; intercept= -0.05, p=0.004 for the Bakker et al.^20^ GWAS), and between LSI and overall intracranial aneurysm risk (intercept = -0.03, p=0.023), the MR-PRESSO outlier test did not identify any significant outliers (global test p>0.05 for all three outcomes). While the absence of outliers suggests that the observed pleiotropy is not driven by a few influential variants, the significant MR-Egger intercepts indicate the presence of directional pleiotropy at the instrument-level. To address this, we additionally applied MR-RAPS, which is robust to weak instruments and pleiotropy, and the results remained consistent with the IVW estimates. Detailed results of the sensitivity analyses are presented in Table 1. The consistency of effect directions across multiple sensitivity methods, together with the robustness of MR-RAPS estimates, suggests that despite the evidence of pleiotropy, the overall causal conclusions are not substantially altered.

**Table 1:** Sensitivity analysis of the causal relationship between lifetime smoking index and unruptured intracranial aneurysms and aneurysmal subarachnoid hemorrhage

*Exposure*	*Outcome*	*Methods*	*nSNP*	*Pleiotropy test*				*Heterogeneity test*		
				*MR-EGGER*			*MR-PRESSO*	*Q_value*	*Q_df*	*Q p*
				*Intercept*	*SE*	*p*	*Global test p*			
LSI	aSAH(FinnGen)	MR-EGGER	117	-0.018	0.008	0.029	0.073	133.103	115	0.119
		IVW						138.753	116	0.074
SmkInit	aSAH(FinnGen)	MR-EGGER	212	-0.004	0.007	0.563	< 0.001	287.841	210	<0.001
		IVW						288.302	211	<0.001
CigDay	aSAH(FinnGen)	MR-EGGER	40	-0.006	0.006	0.349	0.369	40.672	38	0.354
		IVW						41.634	39	0.357
LSI	uIAs(FinnGen)	MR-EGGER	118	-0.014	0.012	0.241	0.141	132.108	116	0.146
		IVW						133.690	117	0.139
SmkInit	uIAs(FinnGen)	MR-EGGER	212	-0.004	0.009	0.649	0.065	241.186	210	0.069
		IVW						241.425	211	0.074
CigDay	uIAs(FinnGen)	MR-EGGER	41	-0.009	0.010	0.341	0.246	45.594	39	0.217
		IVW						46.679	40	0.217
LSI	aSAH(Bakker)	MR-EGGER	75	-0.045	0.015	0.004	0.116	78.614	73	0.306
		IVW						88.343	74	0.122
LSI	uIAs(Bakker)	MR-EGGER	78	0.003	0.023	0.902	0.239	85.790	76	0.207
		IVW						85.807	77	0.230
LSI	uIAs and aSAH(Bakker)	MR-EGGER	83	-0.029	0.013	0.023	0.073	95.860	81	0.124
		IVW						102.253	82	0.064

nSNP: number of SNPs.

### Smoking exposure and circulating proteins

**Figure 2:**
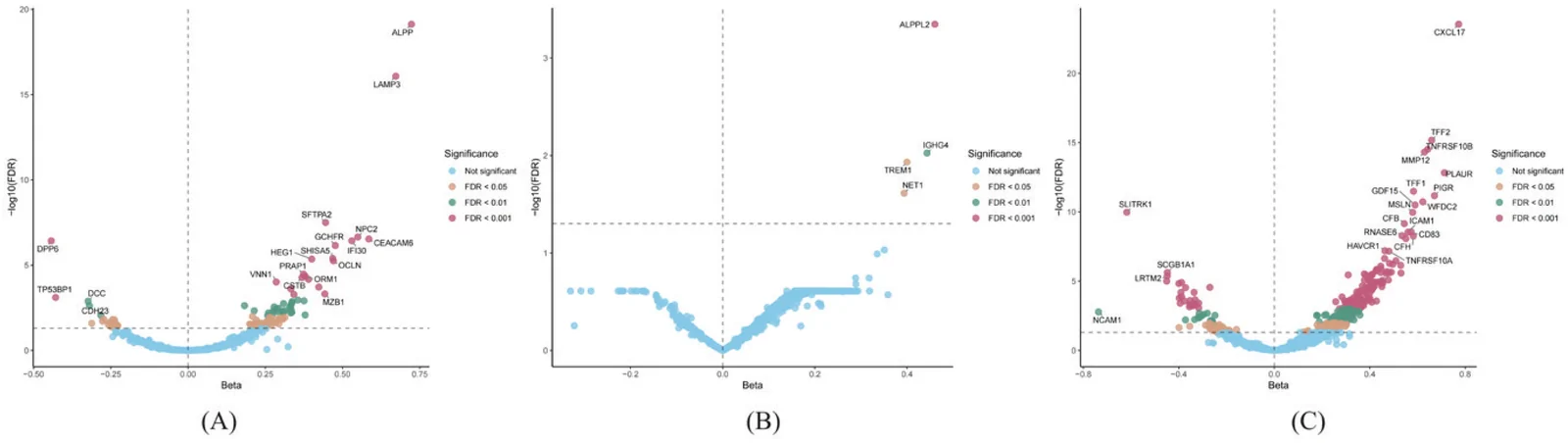
Genetically proxied lifetime smoking index is associated with genetically proxied circulating protein levels: (A) The associations for unique proteins in UKB-PPP; (B) The associations for unique proteins in deCODE; (C) The associations for overlapped proteins between UKB-PPP and deCODE. The associations were based on data from UKB-PPP

In Set 1, which comprised proteins unique to UKB-PPP, genetically proxied lifetime smoking index showed significant associations with 105 plasma proteins after False-Discovery-Rate (FDR, Benjamini-Hochberg method) correction (Figure 2A, Supplementary file Table S6). Among these proteins, 88 showed nominal significance in validation analyses that used genetically proxied smoking initiation and cigarettes per day as exposures (Supplementary file Table S7). In Set 2, which exclusively contained unique plasma proteins from the deCODE database, genetically proxied lifetime smoking index demonstrated significant associations with four distinct proteins after FDR correction (Figure 2B; and Supplementary file Table S8). These four proteins remained nominally significant in the validation analyses and were therefore included in the subsequent analyses (Supplementary file Table S9). For proteins jointly available in UKB-PPP and deCODE (Set 3), FDR-corrected analysis identified 390 plasma proteins significantly associated with genetically proxied lifetime smoking index (Figure 2C; and Supplementary file Table S10). Validation analyses ultimately confirmed that 335 of these proteins maintain a consistent causal association with smoking exposure (Supplementary file Table S11). The FDR was controlled at 0.05. Integrating our multi-layer validation evidence, we ultimately identified 423 circulating proteins in the UKB-PPP project and 4 in the deCODE project that showed robust associations with the genetically proxied lifetime smoking index. Functional and pathway enrichment analysis (GO/KEGG) revealed that the smoking-associated proteins were significantly enriched in multiple biological processes, including those related to vascular inflammation, endothelial function, and angiogenesis, among others (Supplementary file Table S12).

### Smoking-related proteins and risk of disease

**Figure 3:**
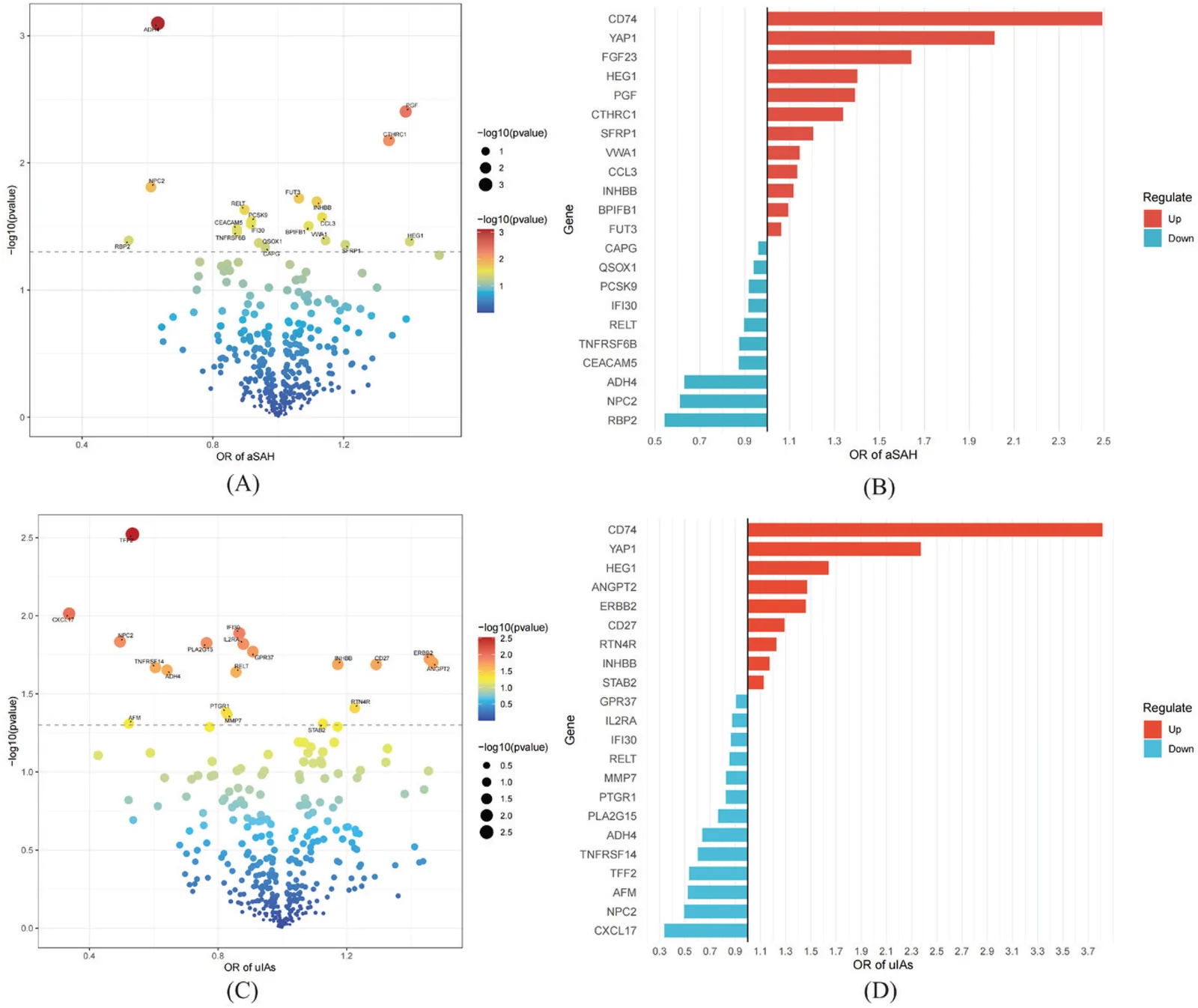
Associations of smoking-associated plasma proteins with intracranial aneurysmal disease risk: (A) Plasma proteins significantly associated with aneurysmal subarachnoid hemorrhage risk (UKB-PPP); (B) Forest plot of the odds ratio ranges for plasma proteins significantly associated with aneurysmal subarachnoid hemorrhage risk; (C) Plasma proteins significantly associated with unruptured intracranial aneurysms risk (UKB-PPP); (D) Forest plot of the odds ratio ranges for plasma proteins significantly associated with unruptured intracranial aneurysms risk

Given that 423 plasma proteins in UKB-PPP and 4 in deCODE were found to be strongly influenced by the genetically proxied lifetime smoking index, we next assessed their associations with the risks of uIAs and aSAH. Among the 423 UKB-PPP plasma proteins strongly linked to smoking exposure, 22 showed nominally significant associations with the risk of developing aSAH (Figure 3A; and Supplementary file Table S13). The odds ratio for aSAH ranged from 0.54 (95% CI: 0.30–0.97) per SD increase in genetically proxied levels of RBP2 (Retinol Binding Protein 2) to 2.49 (95% CI: 1.08–5.76) per SD increase in genetically proxied levels of CD74 (CD74 Molecule) (Figure 3B). However, we observed that TNFRSF6B (TNF Receptor Superfamily Member 6b), IFI30 (IFI30 Lysosomal Thiol Reductase), and NPC2 (NPC Intracellular Cholesterol Transporter 2) had HEIDI test p<0.05, indicating potential pleiotropy in these associations (Supplementary file Table S14). We did not observe any smoking-associated circulating proteins in the deCODE dataset that remained associated with aSAH risk (Supplementary file Table S15). Similarly, among the 423 genetically proxied, smoking-associated circulating proteins provided by the UKB-PPP project, we identified 22 proteins that showed nominally significant associations with uIAs (Figure 3C; and Supplementary file Table S16). The odds ratio for uIAs ranged from 0.34 (95% CI: 0.15–0.77) per SD increase in genetically proxied levels of CXCL17 (C-X-C Motif Chemokine Ligand 17) to 3.82 (95% CI: 1.15–12.71) per SD increase in genetically proxied levels of CD74 (Figure 3D). TFF2 (Trefoil Factor 2) and PTGR1 (Prostaglandin Reductase 1) were considered potentially pleiotropic because their HEIDI test p<0.05 (Supplementary file Table S17). We likewise observed no association between genetically proxied smoking-associated circulating proteins and uIAs risk in the deCODE dataset (Supplementary file Table S18). Interestingly, among the circulating proteins strongly linked to genetically proxied smoking exposure, eight – ADH4 (Alcohol Dehydrogenase 4 (Class II), Pi Polypeptide), CD74, HEG1 (Heart Development Protein With EGF Like Domains 1), IFI30, INHBB (Inhibin Subunit Beta B), NPC2, RELT (RELT TNF Receptor) and YAP1 (Yes1 Associated Transcriptional Regulator) – were simultaneously associated with altered risk of developing aSAH or uIAs. Notably, eight proteins were jointly associated with both uIAs and aSAH, showing a concordant effect pattern: CD74, HEG1, INHBB, and YAP1 promoted disease risk, while ADH4, IFI30, NPC2, and RELT exerted protective effects. This suggests shared molecular pathways underlying aneurysm formation and rupture.

### Mediating effect analysis

To prioritize proteins with genuine mediating roles, we integrated evidence from both smoking-protein and protein-aneurysm associations. Integrating the significant SMR findings from the UKB-PPP and deCODE plasma-protein panels, we ultimately retained 19 unique plasma proteins as candidate biomarkers for evaluating how the lifetime smoking index modulates aSAH risk, and 20 unique plasma proteins for evaluating its modulation of uIAs risk. By aligning the direction of the overall effect with the direction of effect mediated by each protein, mediation analysis identified six plasma proteins – CD74, HEG1, YAP1, FGF23 (Fibroblast Growth Factor 23), SFRP1 (Secreted Frizzled Related Protein 1), and PGF (Placental Growth Factor) – as key candidate mediators in the association between LSI and aSAH risk, and four plasma proteins – CD74, YAP1, ANGPT2 (Angiopoietin 2), and RTN4R (Reticulon 4 Receptor) – as key candidate mediators in the pathway linking LSI to uIAs risk (Table 2; and Supplementary file Material 2 and Figure S1). Further details on the mediation analysis can be found in Supplementary file Tables S19 and S20. According to predictions from an online PPI analysis portal, these plasma proteins exhibited minimal interactions within known protein-protein interaction networks (at a highest confidence threshold of 0.900), suggesting that they may function through independent or non‑overlapping biological pathways (Supplementary file Material 2 and Figure S2). It is noteworthy that both YAP1 and CD74 exerted positive mediation effects on the risk alteration of uIAs and aSAH, suggesting a shared molecular mechanism. Functional and signaling pathway enrichment analyses of the key candidate mediator proteins revealed that these mediators are primarily involved in biological processes such as regulation of vascular endothelial function, inflammatory response, cell proliferation and differentiation, and oxidative stress response. They were significantly enriched in classic vascular signaling pathways, including Rap1, Ras, MAPK, and PI3K-Akt, and were also implicated in vitamin D response, chemokine regulation, and extracellular matrix remodeling. These findings further reveal that the aforementioned mediator proteins may participate in the pathophysiological process of smoking cumulative exposure-induced aneurysm formation and rupture by regulating vascular wall homeostasis, mediating chronic inflammation and vascular remodeling, and serving as molecular bridges linking LSI to the risk of uIAs and aSAH. This conclusion provides important biological evidence for elucidating the molecular mechanisms underlying aneurysm onset and for screening potential intervention targets. Additional details of the mediation analysis are given in Table 2, while detailed information on biological functions and signaling pathway enrichment is presented in Supplementary file Material 2 and Figures S3 and Figure S4.

**Table 2:** Comprehensive mediation analysis of lifetime smoking index influencing aneurysmal subarachnoid hemorrhage and unruptured intracranial aneurysms risk via modulation of plasma protein expression

*Exposure*	*Mediating* *factors*	*Outcome*	*Total effect*	*Beta1*	*Beta2*	*Mediation* *effect*	*Direct effect*	*p*	*Proportion* *of* *mediating* *effect*
LSI	CD74	aSAH	0.920	0.357	0.914	0.326	0.594	1.59E-24	0.355
LSI	YAP1	aSAH	0.920	0.306	0.700	0.214	0.706	5.81E-16	0.233
LSI	FGF23	aSAH	0.920	0.246	0.496	0.122	0.798	2.94E-08	0.133
LSI	PGF	aSAH	0.920	0.299	0.329	0.098	0.821	3.07E-04	0.107
LSI	HEG1	aSAH	0.920	0.400	0.338	0.135	0.784	1.58E-03	0.147
LSI	SFRP1	aSAH	0.920	0.305	0.187	0.057	0.863	3.46E-02	0.062
LSI	CD74	uIAs	1.095	0.357	1.339	0.478	0.617	2.16E-40	0.436
LSI	ANGPT2	uIAs	1.095	0.451	0.384	0.173	0.923	3.82E-05	0.158
LSI	RTN4R	uIAs	1.095	0.243	0.203	0.049	1.046	1.56E-02	0.045
LSI	YAP1	uIAs	1.095	0.306	0.864	0.264	0.831	1.90E-02	0.241

Beta values (β) are log (OR). Total effect: log (OR) of lifetime smoking index on uIAs/aSAH. Mediation effect: β1 (smoking-protein) × β2 (protein-disease).

### PheWAS analysis of the proteome

Plasma proteins with potential mediating effects in the mediation analysis remained independent even at the highest confidence threshold; therefore, all eight unique proteins were included in the phenotypic association exploration. The query was performed with the population set to European ancestry and using the portal’s default suggestive significance threshold of 1×10^-6^ and genome-wide significance threshold of 1×10^-8^. The results showed that, except for FGF23, which exhibited a suggestive association with the continuous phenotype ‘Volumetric scaling from T1 head image to standard space’ and a significant association with plasma phosphate concentration, and HEG1, which showed a significant association with the continuous phenotype ‘plasma concentration of inflammatory factor 2 (AstraZeneca PheWAS portal phenotype ID: 30579)’, the remaining six proteins had no substantial associations with either binary or continuous phenotypes in the database. Further details of the PheWAS analysis are available in Supplementary file Material 2 and Figures S5 and S6. Therefore, integrating our proteomics findings, we ultimately recommend CD74, YAP1, PGF, SFRP1, ANGPT2, and RTN4R as therapeutic targets for intervening in the persistent cerebrovascular injury induced by tobacco exposure. Notably, CD74 and YAP1 exhibit the most pronounced pathogenic effects in both adverse cerebrovascular outcomes; further investigation targeting these two molecules is warranted and would be of benefit to patients.

## DISCUSSION

In this mediation MR study, our findings provide genetic evidence supporting a positive association between genetically predicted LSI and the risk of uIAs and aSAH, suggesting that smoking may increase susceptibility to these outcomes. We firstly established a positive causal association between genetically proxied LSI and the risk of both uIAs and aSAH, with each SD increase in LSI corresponding to approximately 2.5 to 3.0-fold higher odds of these outcomes. Through systematic screening of over 5700 circulating proteins, we identified 427 proteins whose levels were significantly altered by genetically proxied smoking exposure. Among these, 22 were nominally associated with aSAH risk and 22 with uIAs risk, with eight proteins overlapping between the two phenotypes. Formal mediation analysis further distilled these candidates into six mediators (CD74, HEG1, YAP1, FGF23, SFRP1, PGF) for the LSI to aSAH pathway and four mediators (CD74, YAP1, ANGPT2, RTN4R) for the LSI to uIAs pathway, with CD74 and YAP1 consistently exhibiting the highest mediation proportions in both outcomes. Importantly, the largely non-overlapping nature of these mediators across the two disease endpoints, coupled with their minimal protein-protein interactions, suggests that smoking may exert its deleterious effects through multiple independent biological cascades rather than a single unified pathway. These findings deepen our understanding of the molecular basis of smoking-related cerebrovascular disease and nominate several candidate proteins that may serve as biomarkers or prioritized targets for future functional and translational studies in high-risk populations.

In the primary FinnGen analysis, LSI was associated with 2.5-fold (aSAH) and 3.0-fold (uIAs) increased risks; in the Bakker validation cohort, associations were stronger (3.2-fold and 8.1-fold, respectively), which is consistent with previous epidemiological reports and further corroborates these observations^7,8^. Critically, the use of MR analysis minimized confounding factors and reverse causation, thereby strengthening causal inference. The consistency of findings from the combined validation of multiple exposure measures (LSI, SmkInit, and CigDay) and independent outcome cohorts (FinnGen and the Bakker meta-analysis) further reinforced the robustness of this causal inference approach. However, as with all MR analyses, our estimates are conditional on the validity of the instrumental variable assumptions; thus, these results contribute to the accumulating genetic evidence implicating smoking in aneurysm pathology, but do not constitute definitive proof of a causal relationship. At the mechanistic level, among the 105 LSI-associated plasma proteins identified in Set 1, 83.8% (88/105) retained nominal significance in validation analyses using genetically proxied SmkInit and CigDay as exposures. In Set 3, the validation consistency reached 85.7% (335/391) among the 391 proteins. These high validation rates indicate that the LSI-associated proteins identified in this study are not chance discoveries, but rather reflect a systematic and reproducible regulation of the plasma proteome by the cumulative effect of lifetime smoking exposure. From a biological function perspective, the 427 LSI-associated plasma proteins identified in this study (423 from UKB and 4 from deCODE) encompass multiple pathways including inflammatory regulation, oxidative stress, extracellular matrix remodeling, lipid metabolism, angiogenesis, and apoptosis, which are highly consistent with the known pathophysiological effects of smoking exposure. This suggests that the cumulative effect of lifetime smoking, as captured by LSI, may influence aneurysm formation and stability through multi-system, multi-level molecular networks rather than through the specific action of a single target. This pleiotropic nature not only increases the complexity of mechanistic investigations but also provides a rich repertoire of candidate molecules for the development of composite biomarkers.

Through SMR analysis, this study identified 22 proteins nominally significantly associated with aSAH risk among the total 427 LSI-related proteins, and another 22 proteins nominally significantly associated with uIAs risk. Among these, eight proteins (ADH4, CD74, HEG1, IFI30, INHBB, NPC2, RELT, and YAP1) were associated with both disease phenotypes, with highly consistent effect directions. Specifically, CD74, HEG1, INHBB, and YAP1 were associated with increased disease risk, whereas ADH4, IFI30, NPC2, and RELT were associated with decreased disease risk. The shared proteins identified here raise the possibility of common pathophysiological processes linking aneurysm initiation to stability loss. Nevertheless, given the inherent limitations of MR mediation analyses, these findings should be interpreted as suggestive, rather than conclusive, evidence that LSI influences disease progression via modulation of these specific proteins. Mediation analysis further identified CD74, HEG1, YAP1, FGF23, SFRP1, and PGF as mediators of aSAH risk, and CD74, YAP1, ANGPT2, and RTN4R as mediators of uIAs risk, with CD74 and YAP1 being common mediators for both. These findings deepen our understanding of the molecular basis of smoking-related cerebrovascular disease and nominate several candidate proteins that may serve as biomarkers or prioritized targets for future functional and translational studies in high-risk populations. Moreover, these proteins do not exhibit close interconnections in known protein-protein interaction networks, suggesting that smoking exposure may influence clinical subtypes of intracranial aneurysm through multiple independent biological pathways. Notably, the key roles of some potential candidate mediator proteins have already been implicated in vascular homeostasis biology involving inflammatory responses, endothelial dysfunction, and inflammatory cell migration.

HEG1 is an endothelium-specific adhesion molecule and a calcium-binding EGF-like protein that primarily regulates the Rho signaling pathway, thereby contributing to the maintenance of vascular integrity and the promotion of angiogenesis^30^. Its upregulation induced by tobacco exposure may disrupt endothelial barrier function, facilitate inflammatory infiltration, and promote vascular wall injury – a critical step in aneurysm formation. PGF, a member of the VEGF family, promotes angiogenesis and vascular remodeling. It has been implicated in smoking exposure-induced vascular inflammation and atherosclerosis, and may therefore accelerate the progression of aSAH^31^. Despite their biological relevance, HEG1 and FGF23 were excluded from therapeutic target recommendations due to their pleiotropic associations identified in PheWAS, which may increase the risk of off-target effects.

Of particular note is the CD74 protein, which exhibited the strongest risk-mediating effect in both aSAH and uIAs analyses. As an invariant chain of MHC class II molecules, CD74 is involved in antigen presentation and immune regulation. Its high expression in aneurysms likely reflects a smoking-induced chronic inflammatory state of the vessel wall. Previous studies have shown that CD74 plays a central role in macrophage activation, T cell regulation, and inflammatory signal transduction. In turn, inflammatory cell infiltration and immune activation in the aneurysm wall are key pathological features driving wall degeneration and rupture^32^. The genetic evidence from this study supports that CD74 is not only a circulating marker associated with smoking exposure but also a causal mediator driving the pathological progression of aneurysms.

Additionally, YAP1, a core effector of the Hippo signaling pathway, exhibited a strong risk-mediating effect in both aSAH and uIAs analyses, offering a novel perspective on smoking-induced vascular remodeling. YAP1 is a key transcriptional coactivator regulating cell proliferation, apoptosis, and organ size, and plays an important role in mechanosensing and extracellular matrix remodeling. Previous studies have extensively documented its critical involvement in Vascular Smooth Muscle Cell (VSMC) phenotype switching, mediating the vascular wall’s response to hemodynamic stress and promoting abdominal aortic aneurysm formation^33,34^. Genetically proxied elevation of YAP1 levels was significantly associated with both aneurysm subtypes. We therefore hypothesize that smoking burden activates YAP1-dependent mechanotransduction pathways, thereby promoting VSMC phenotype switching and degenerative changes in the aneurysm wall. This mechanistic hypothesis aligns with the hemodynamically driven pathogenesis of IAs, in which the cumulative effect of lifelong smoking and local hemodynamic stress may synergistically accelerate aneurysm formation and structural instability through the YAP1 pathway^35^.

Notably, PPI network analysis revealed that the interactions among these key candidate mediator proteins are remarkably weak, suggesting that smoking exposure may exert its pathogenic effects through multiple independent biological pathways, including endothelial dysfunction (HEG1, PGF), and other smoking-related proteins involved in immune cell recruitment and chemotactic regulation. This system-level complexity underscores why single-target interventions have consistently failed in preventing aneurysm formation and rupture.

### Strengths and limitations

Our study has several strengths. First, methodological innovation: by employing a two-step network MR analysis that integrated GWAS and proteomics data, we were able to minimize confounding interference and provide genetic evidence for causal relationships and mediation effects. Second, through comprehensive sensitivity analyses including IVW, MR-RAPS, Steiger direction tests, and replication analyses in the FinnGen and Bakker cohorts, we obtained highly consistent core results, demonstrating the robustness of our findings. Furthermore, owing to a fully transparent IV selection process, a well-documented sensitivity analysis pipeline, and strict adherence to the STROBE-MR guideline-recommended reporting framework, our study achieves a high degree of reproducibility.

Of course, several limitations of our study merit consideration. First, although MR analysis reduces confounding and we performed sensitivity analyses (including MR-Egger, MR-RAPS, and HEIDI tests), residual horizontal pleiotropy cannot be completely excluded. Second, the proteomics data were primarily derived from cohorts of European ancestry; therefore, the generalizability to other ethnic groups requires further validation, and targeted validation in East Asian populations is particularly needed in the future. Third, the proteomics assays in UKB-PPP and deCODE studies cover only a small fraction of the human plasma proteome; integrating deeper proteomics GWAS data in future research may identify additional effector mediator proteins. Fourth, we used plasma protein data for mediation MR analysis, which may not fully reflect protein expression levels in the intracranial vessel wall – the primary site of aneurysm pathogenesis. Future studies should use human cerebrovascular tissue samples to validate the local expression levels and functional mechanisms of these mediator proteins. Fifth, the findings of this study are primarily based on statistical screening and lack validation from independent plasma sample testing, aneurysm wall tissue examination, or *in vivo* and *in vitro* functional experiments. Therefore, the mediating roles of the relevant proteins should be regarded as statistical hypotheses that await experimental validation. Sixth, the LSI GWAS (UK Biobank, n=462690) and UKB-PPP protein data (UK Biobank subset, n=54219) share participants. Although two-sample MR is robust to moderate sample overlap, this may still introduce weak bias toward observational estimates. However, the consistent findings using independent deCODE proteomic data and replication in the Bakker cohort mitigate this concern. Seventh, the two-step design may introduce winner’s curse, where proteins selected in the first step (smoking to protein) may show inflated effect estimates in the second step (protein to disease), potentially leading to overestimation of mediation proportions. Eighth, in the protein-to-disease association step (the SMR analysis), we used a nominal p<0.05 threshold and did not apply FDR correction for multiple testing. Consequently, these proteins should be regarded as an initial screening pool of potential mediator candidates rather than as definitive mediators. It is also important to note that proteins not nominated at this step should still be considered as possible mediators, given the permissive screening threshold. Future studies using independent discovery and validation cohorts could mitigate this bias.

## CONCLUSIONS

Our findings are consistent with a potential causal relationship between LSI and intracranial aneurysmal disease, and suggest that eight unique plasma proteins may mediate this association through distinct biological pathways. Among them, CD74, HEG1, YAP1, FGF23, SFRP1, and PGF mediate the risk of aSAH; CD74, YAP1, ANGPT2, and RTN4R mediate the risk of uIAs; and CD74 and YAP1 are common mediators for both. These findings provide insights into the molecular basis of smoking-induced cerebrovascular injury and nominate candidate protein mediators that may inform future prevention strategies. However, given the inherent assumptions of MR analyses, further experimental and clinical studies are required to validate these observations before any translational application can be considered. Furthermore, these results are consistent with the established importance of smoking cessation in preventing aneurysm-related diseases. The identified plasma protein alterations may provide a basis for future studies evaluating their potential utility in risk stratification among high-risk smokers.

## Supplementary Material



## Data Availability

The datasets supporting the conclusions of this article are included within the article and its additional files. The analysis code is available from the corresponding authors upon reasonable request.
