# Supplementary material for: Circulating proteins as key genetically proxied mediators in the pathway from smoking to intracranial aneurysm disease: A mediation Mendelian randomization analysis
Source: Tob Induc Dis. 2026 Sep 21;24:10.18332/tid/226836. doi: 10.18332/tid/226836 (PMC13595440; doi:10.18332/tid/226836)
Supplement: Supplementary file 1 [file TID-24-171-s1.pdf]

Tobacco Induced Diseases

Supplementary file

© 2026 Liu Y. et al.

DOI:
10.18332/tid/226836

The content has been provided by the author(s) and has not been reviewed, verified, or endorsed by European Publishing. It may not have undergone peer review. The views, opinions, and recommendations expressed are solely those of the author(s) and do not necessarily reflect the position of European Publishing. European Publishing accepts no responsibility or liability for any consequences arising from the use of, or reliance on, this content.

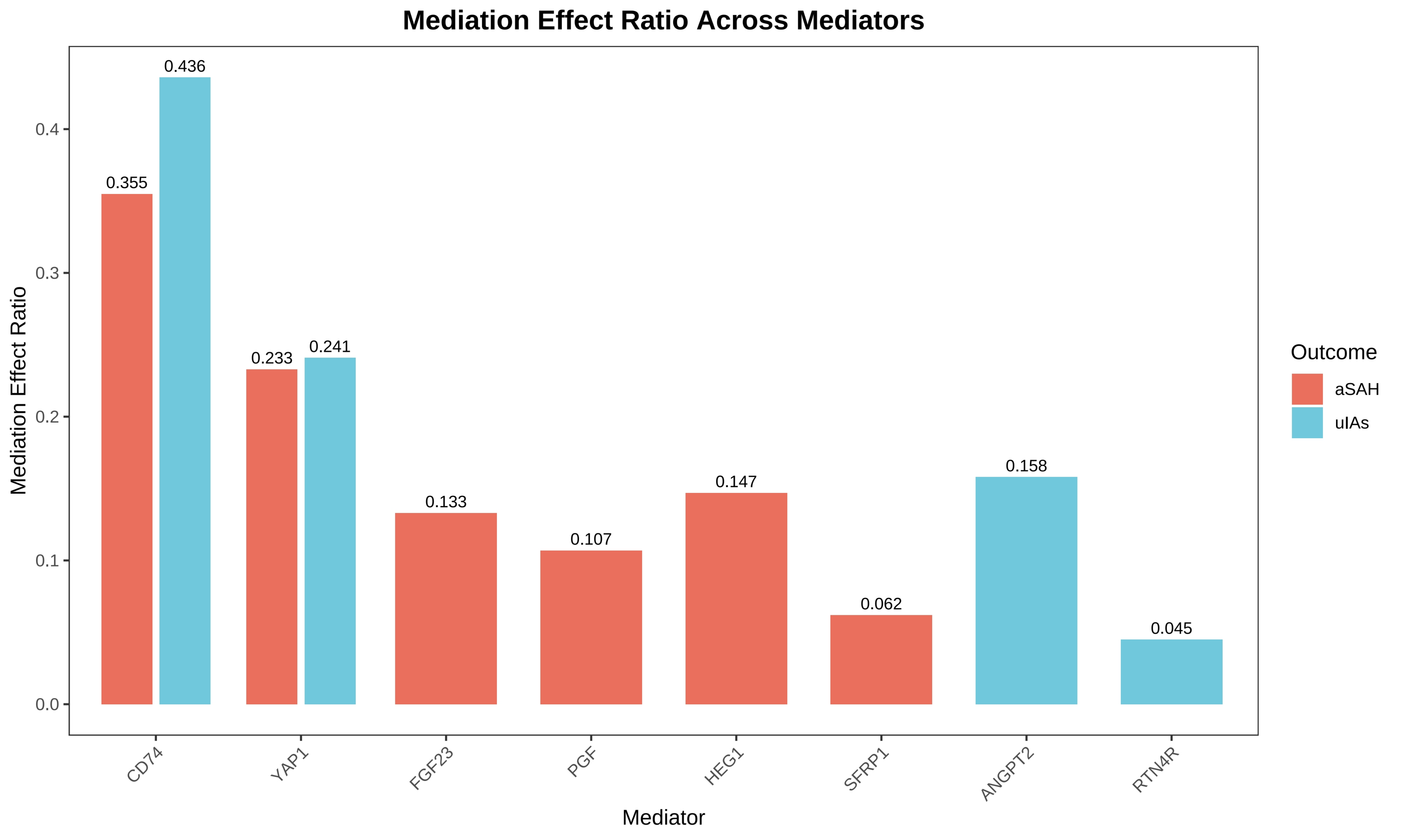
Mediation Effect Ratio Across Mediators
Mediation Effect Ratio
0.0
0.1
0.2
0.3
0.4
0.355
0.436
0.233
0.241
0.133
0.107
0.147
0.062
0.158
0.045
CD74
YAP1
FGF23
PGF
HEG1
SFRP1
ANGPT2
RTN4R
Mediator
Outcome
aSAH
uIAs

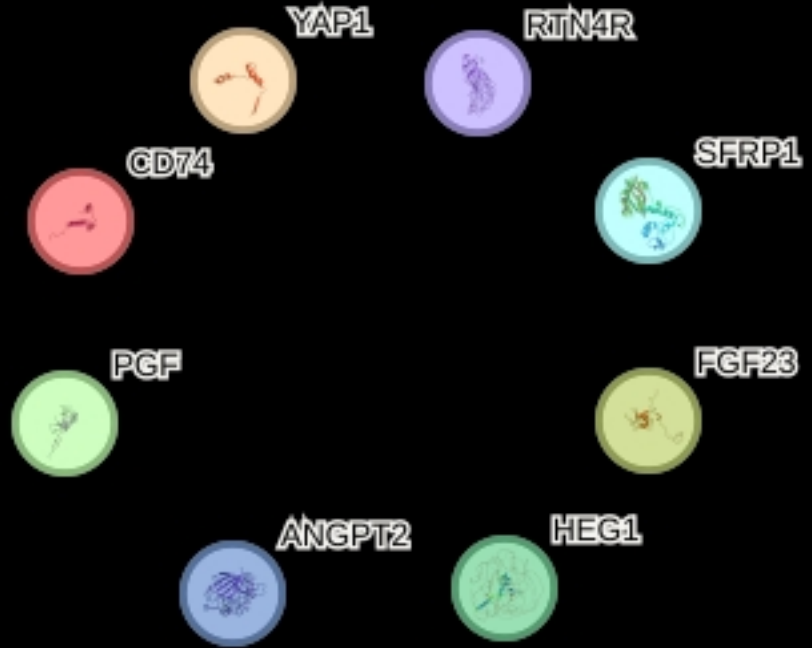
YAP1
RTN4R
CD74
SFRP1
PGF
FGF23
ANGPT2
HEG1

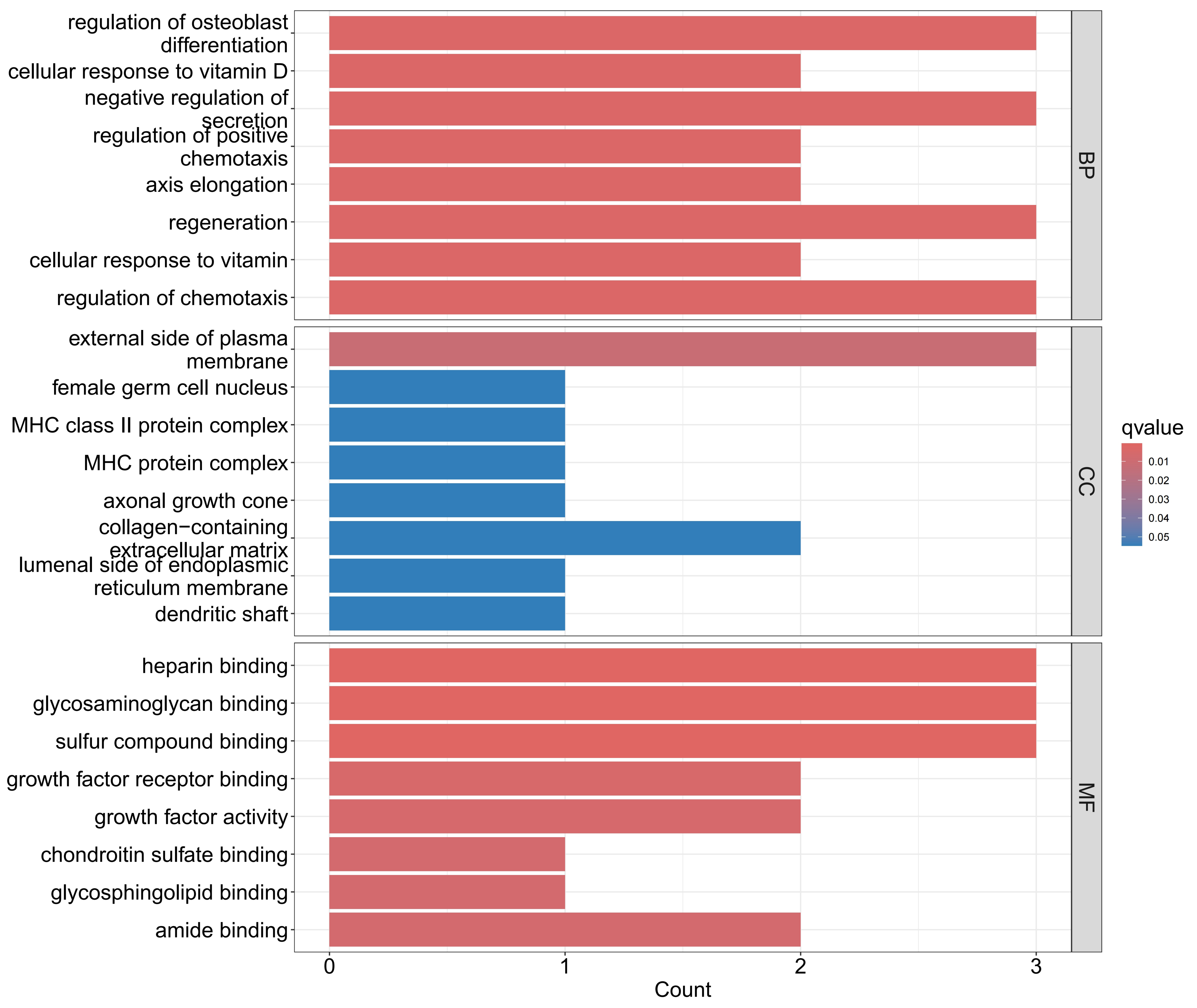

regulation of osteoblast differentiation
cellular response to vitamin D
negative regulation of secretion
regulation of positive chemotaxis
axis elongation
regeneration
cellular response to vitamin
regulation of chemotaxis
external side of plasma membrane
female germ cell nucleus
MHC class II protein complex
MHC protein complex
axonal growth cone
collagen−containing extracellular matrix
lumenal side of endoplasmic reticulum membrane
dendritic shaft
heparin binding
glycosaminoglycan binding
sulfur compound binding
growth factor receptor binding
growth factor activity
chondroitin sulfate binding
glycosphingolipid binding
amide binding
BP
CC
MF
qvalue
0.01
0.02
0.03
0.04
0.05
0
1
2
3
Count

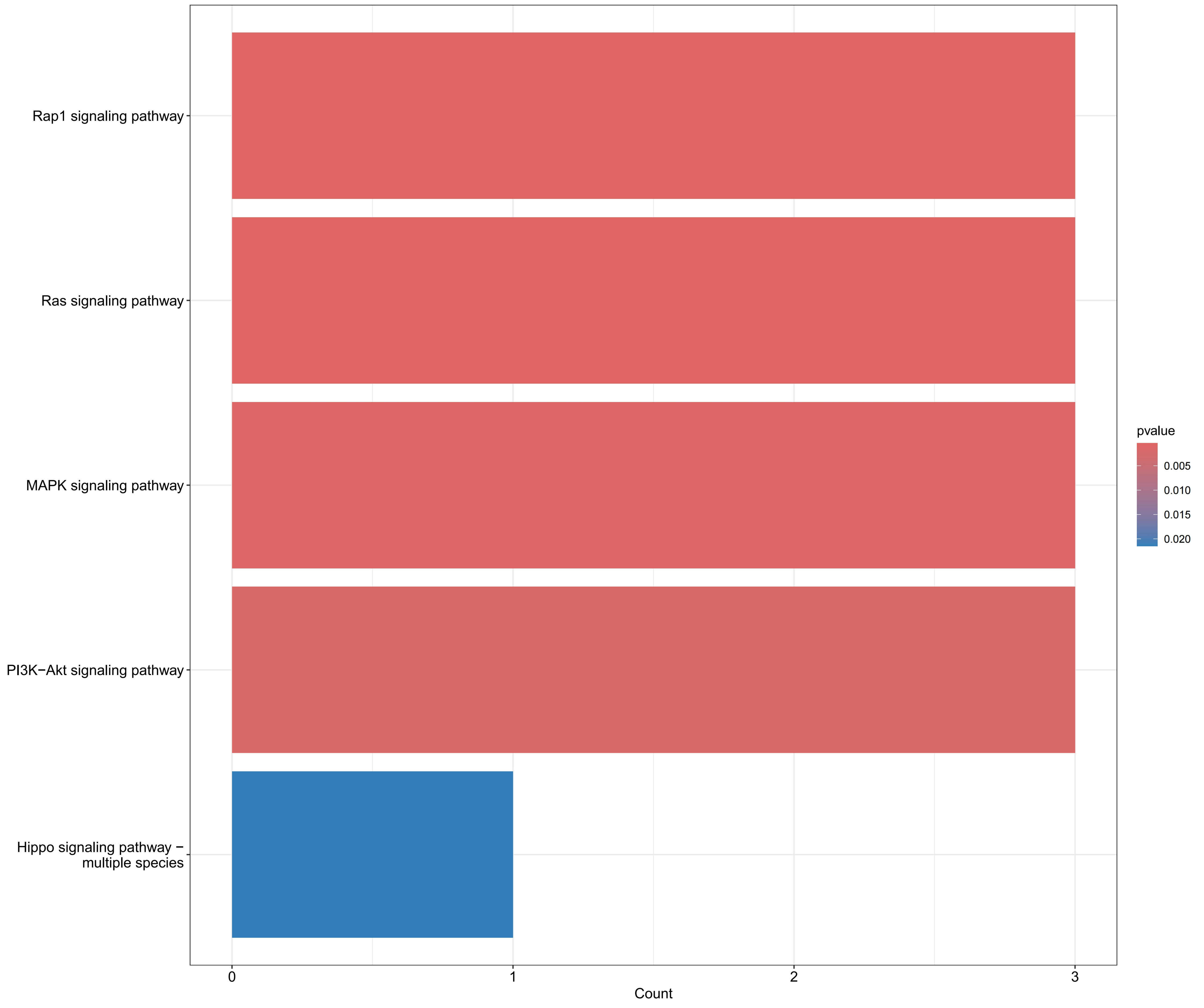
Rap1 signaling pathway
Ras signaling pathway
MAPK signaling pathway
PI3K−Akt signaling pathway
Hippo signaling pathway − multiple species
0
1
2
3
Count
pvalue
0.005
0.010
0.015
0.020

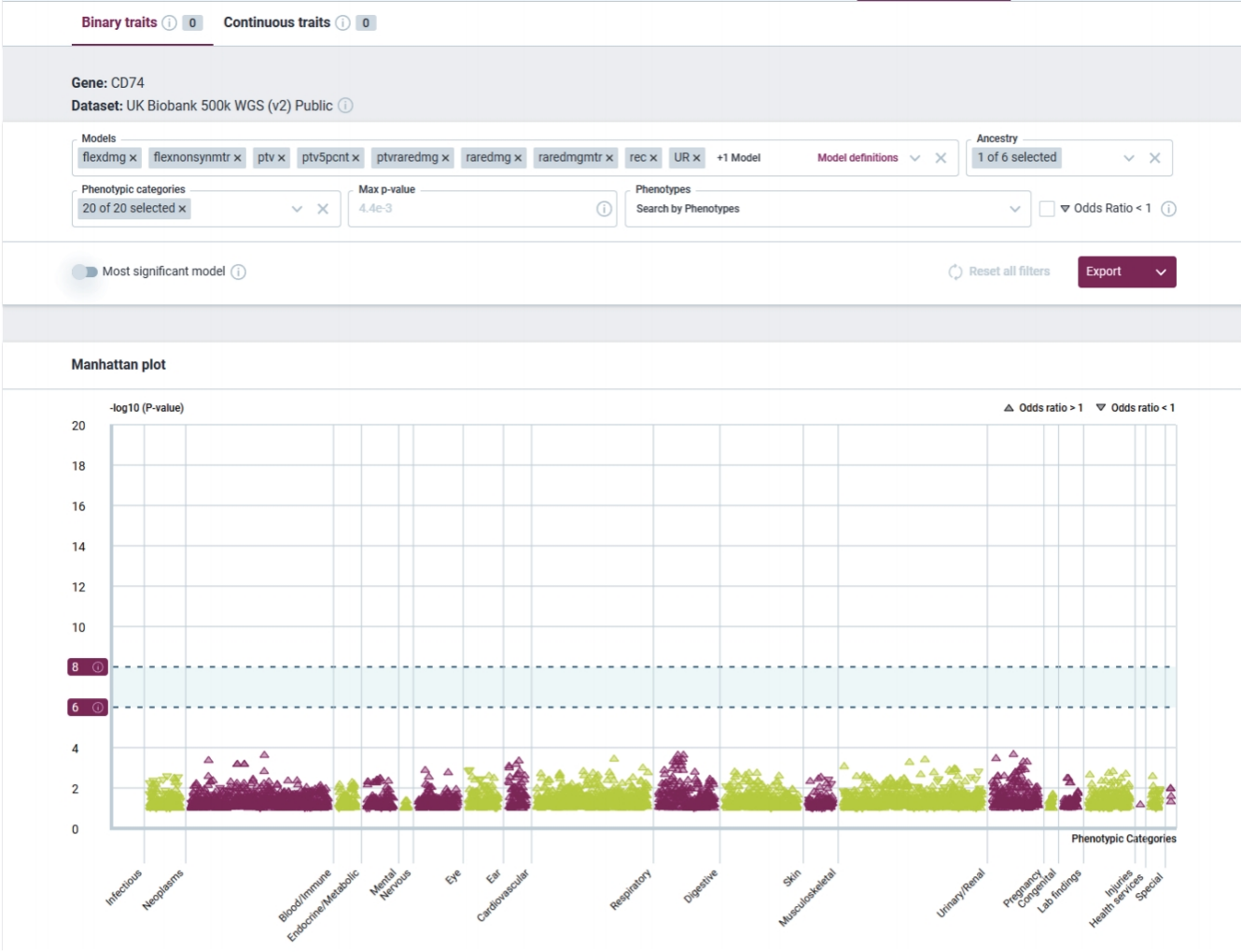
Gene: CD74
Dataset: UK Biobank 500k WGS (v2) Public
Manhattan plot

(A)

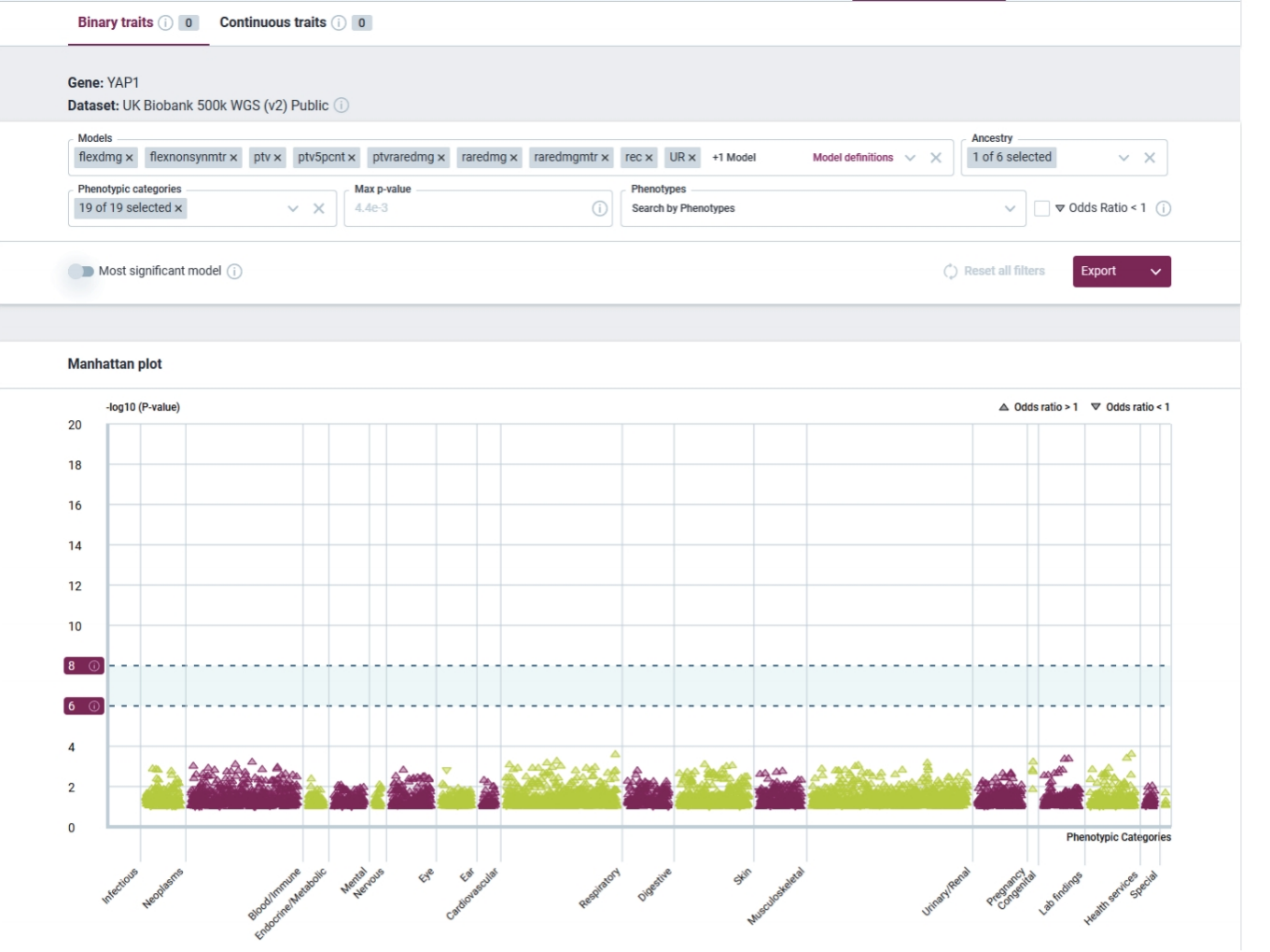
Gene: YAP1
Dataset: UK Biobank 500k WGS (v2) Public
Manhattan plot

(B)

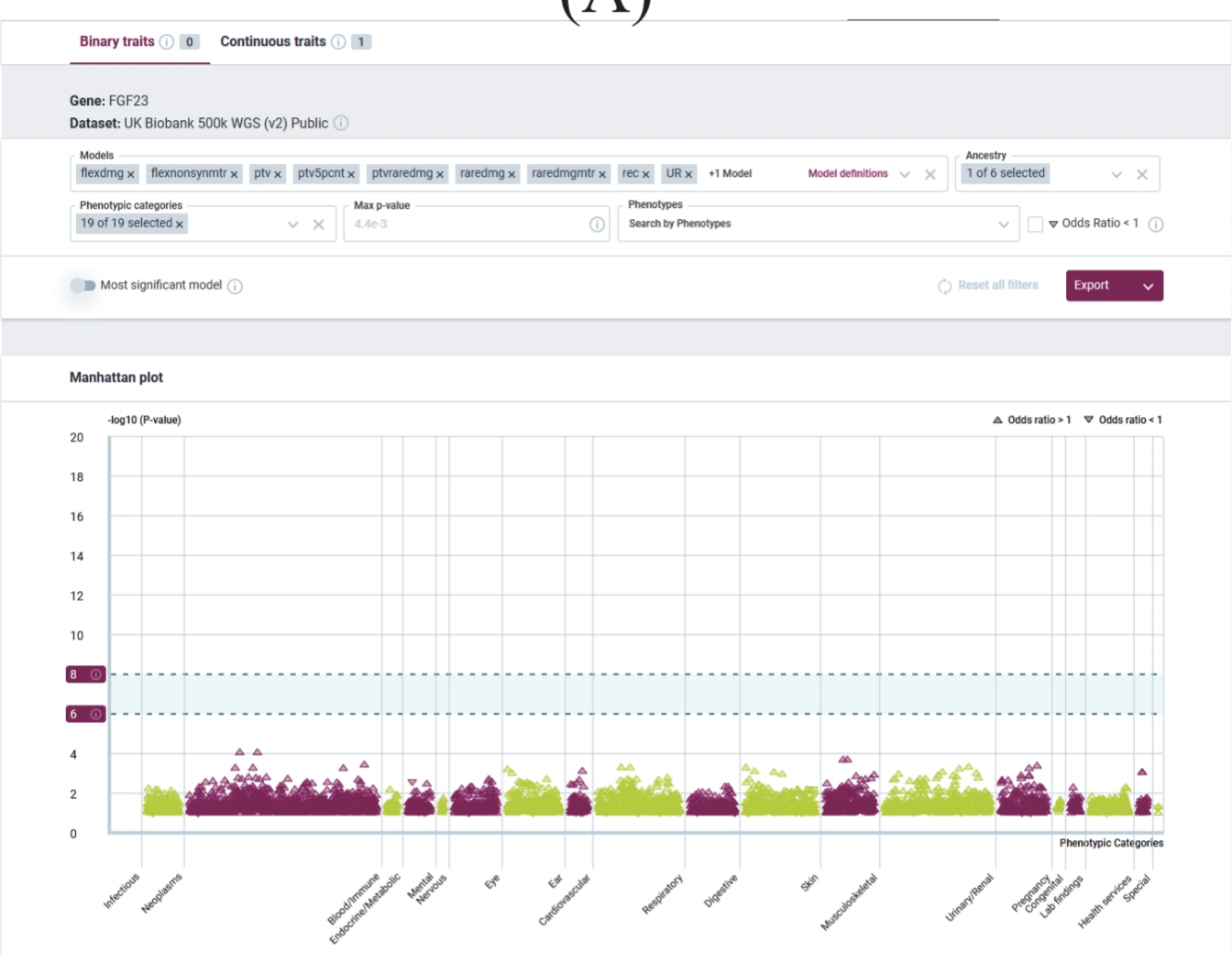
Gene: FGF23
Dataset: UK Biobank 500k WGS (v2) Public
Manhattan plot

(C)

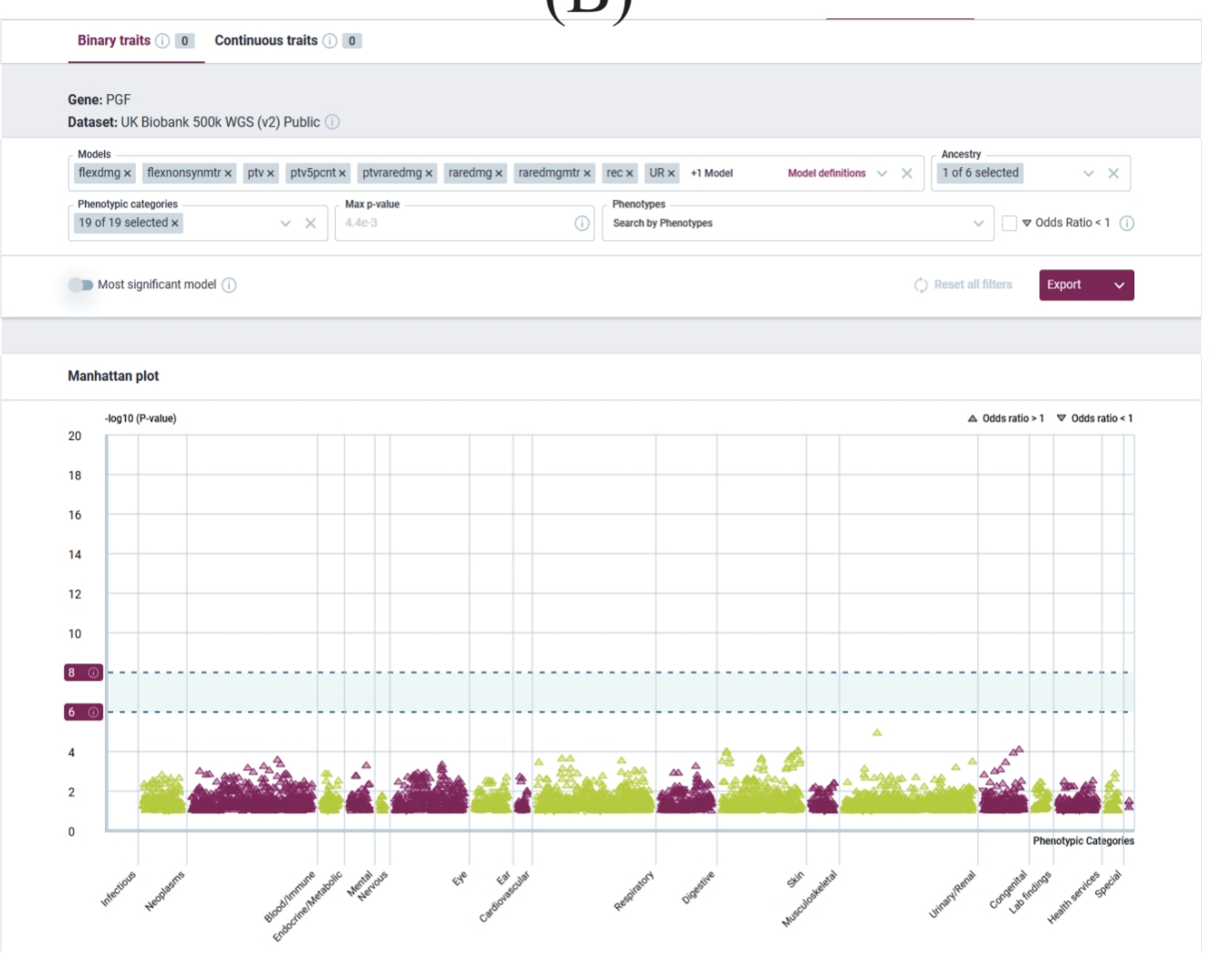
Gene: PGF
Dataset: UK Biobank 500k WGS (v2) Public
Manhattan plot

(D)

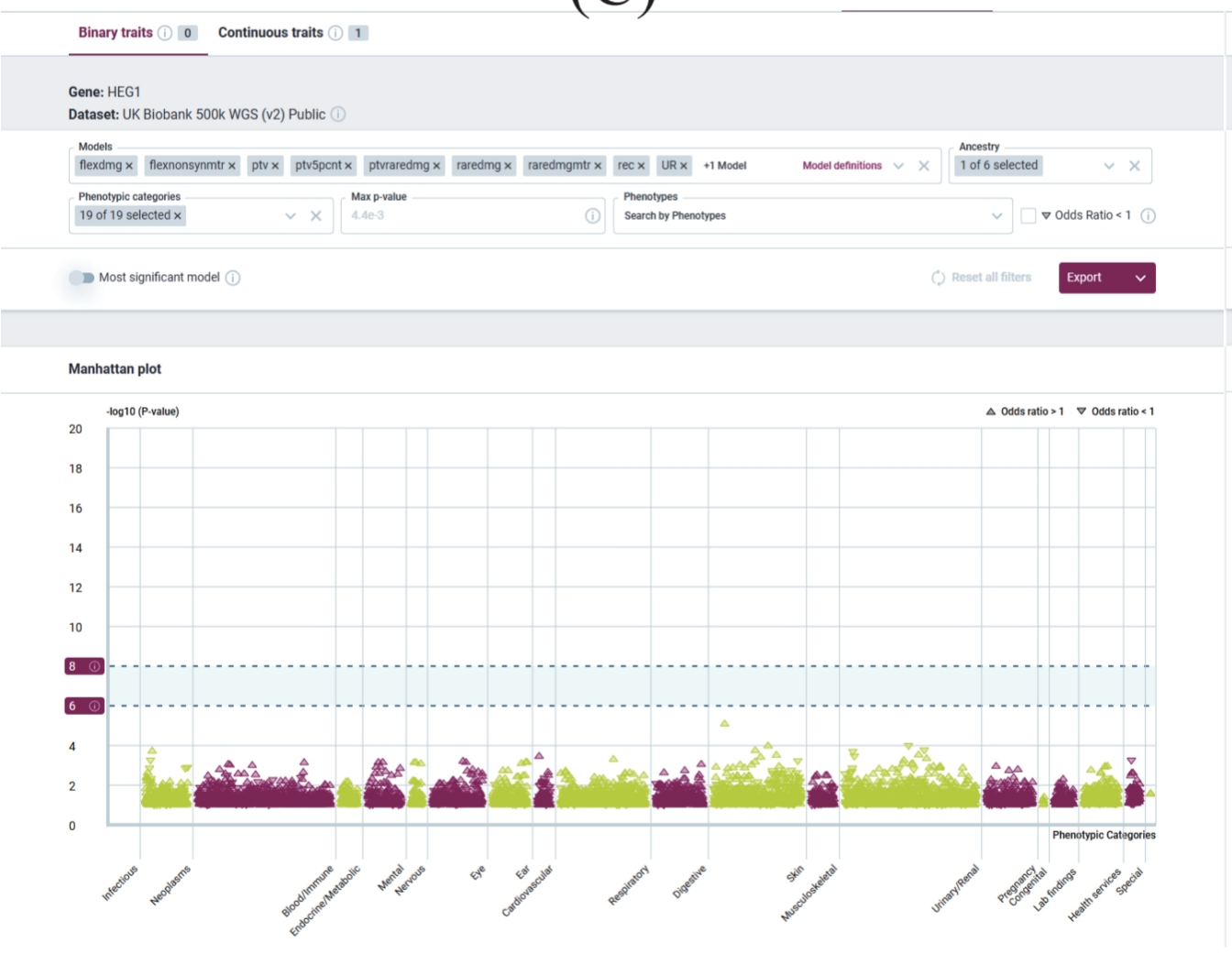
Gene: HEG1
Dataset: UK Biobank 500k WGS (v2) Public
Manhattan plot

(E)

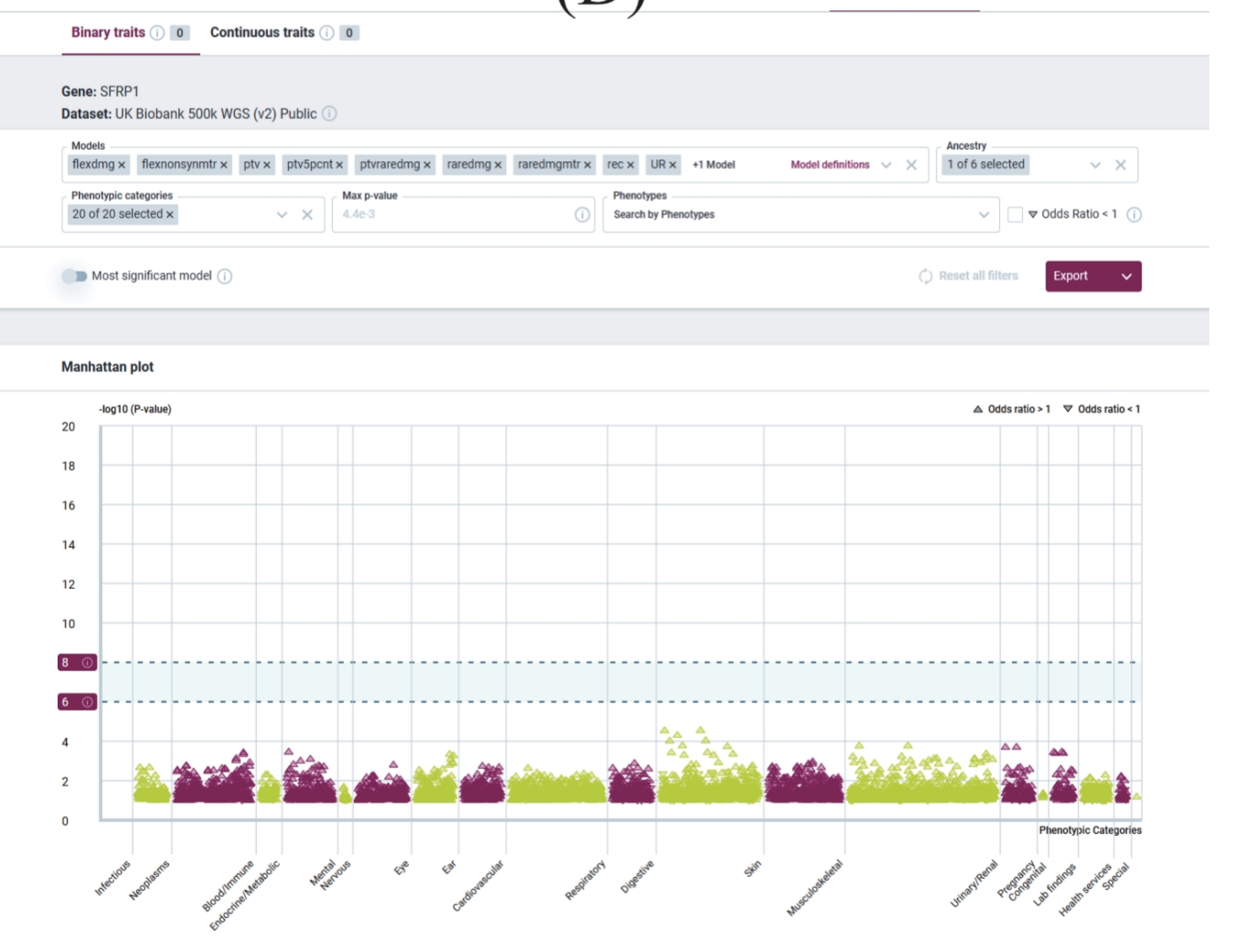
Gene: SFRP1
Dataset: UK Biobank 500k WGS (v2) Public
Manhattan plot

(F)

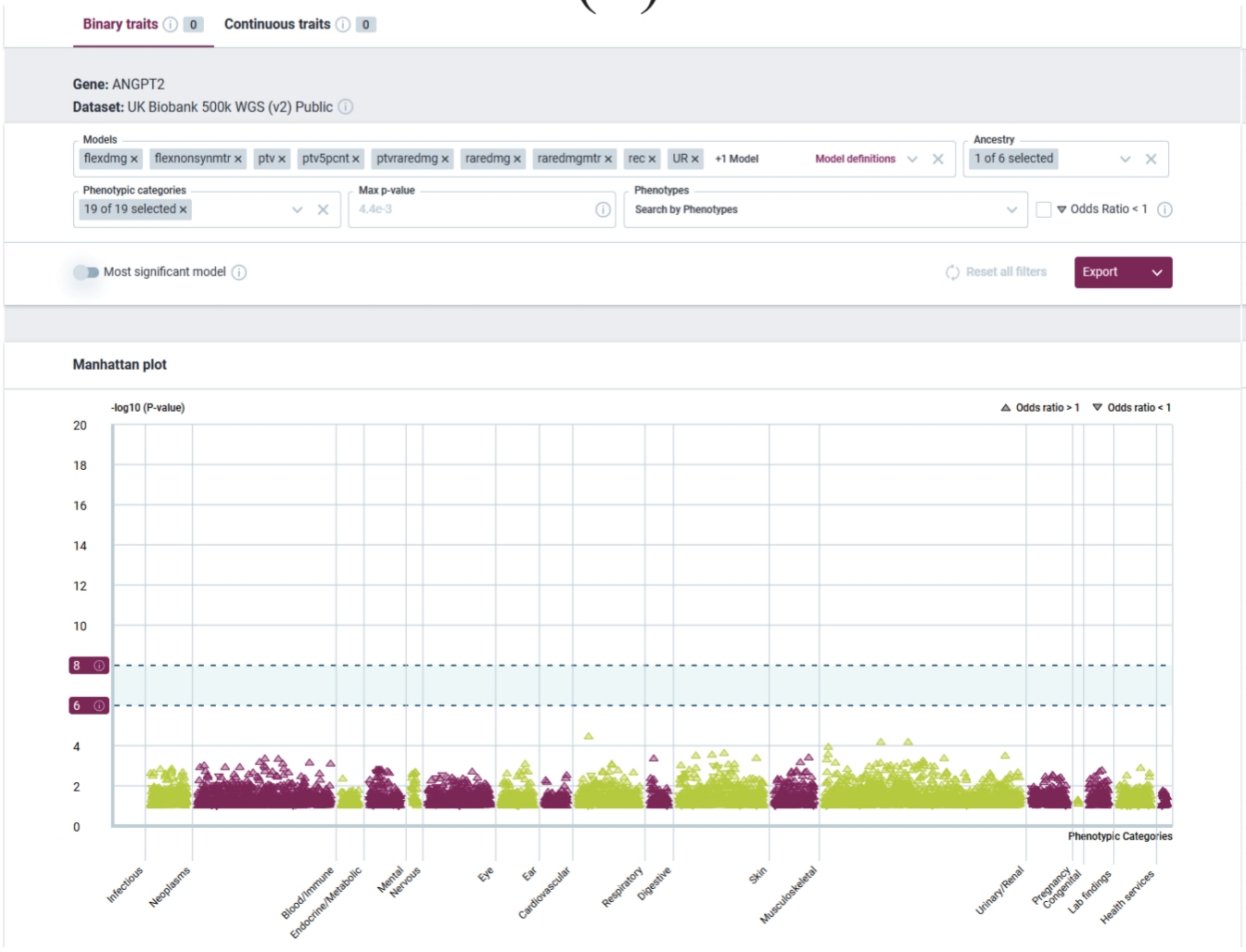
Gene: ANGPT2
Dataset: UK Biobank 500k WGS (v2) Public
Manhattan plot

(G)

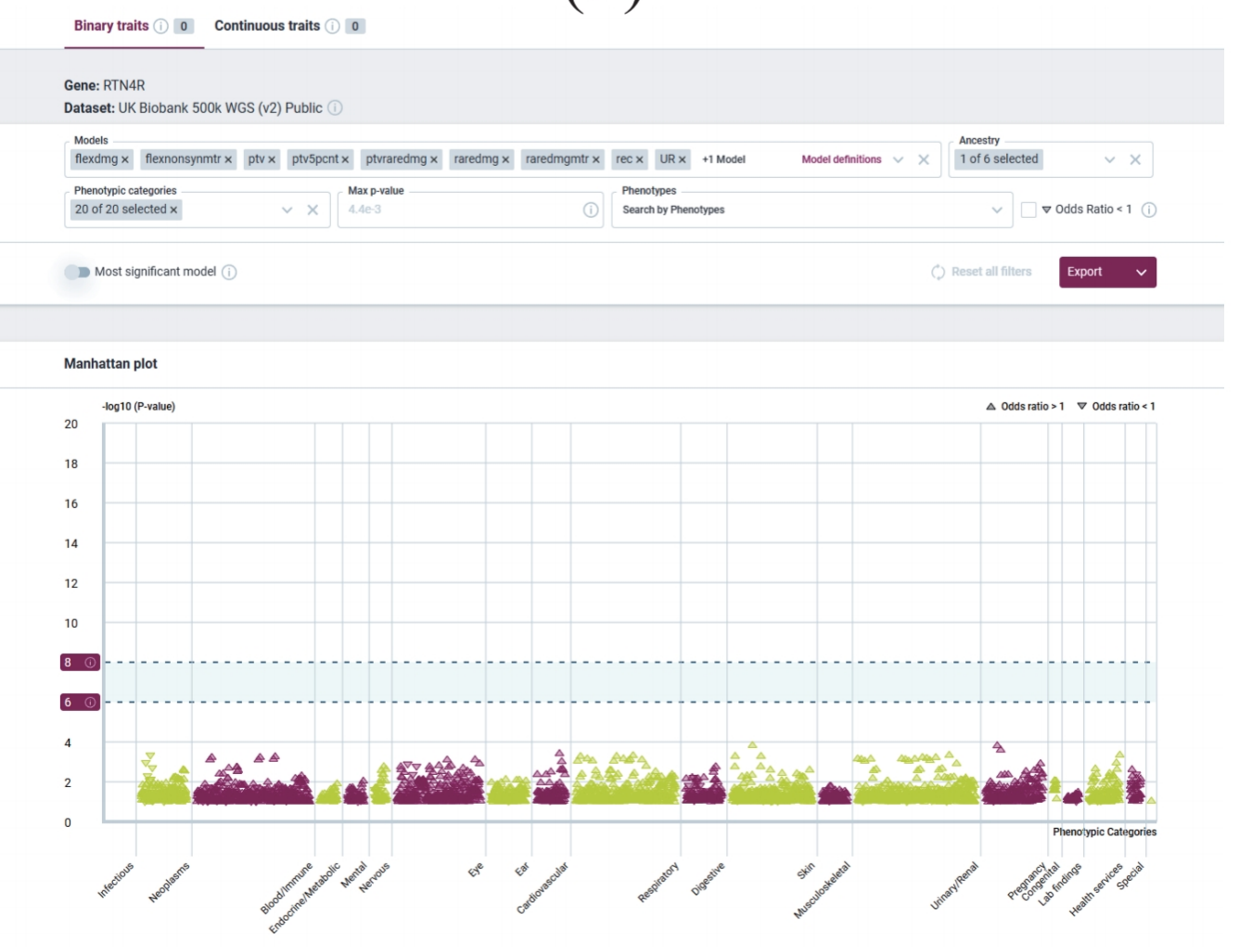
Gene: RTN4R
Dataset: UK Biobank 500k WGS (v2) Public
Manhattan plot

(H)

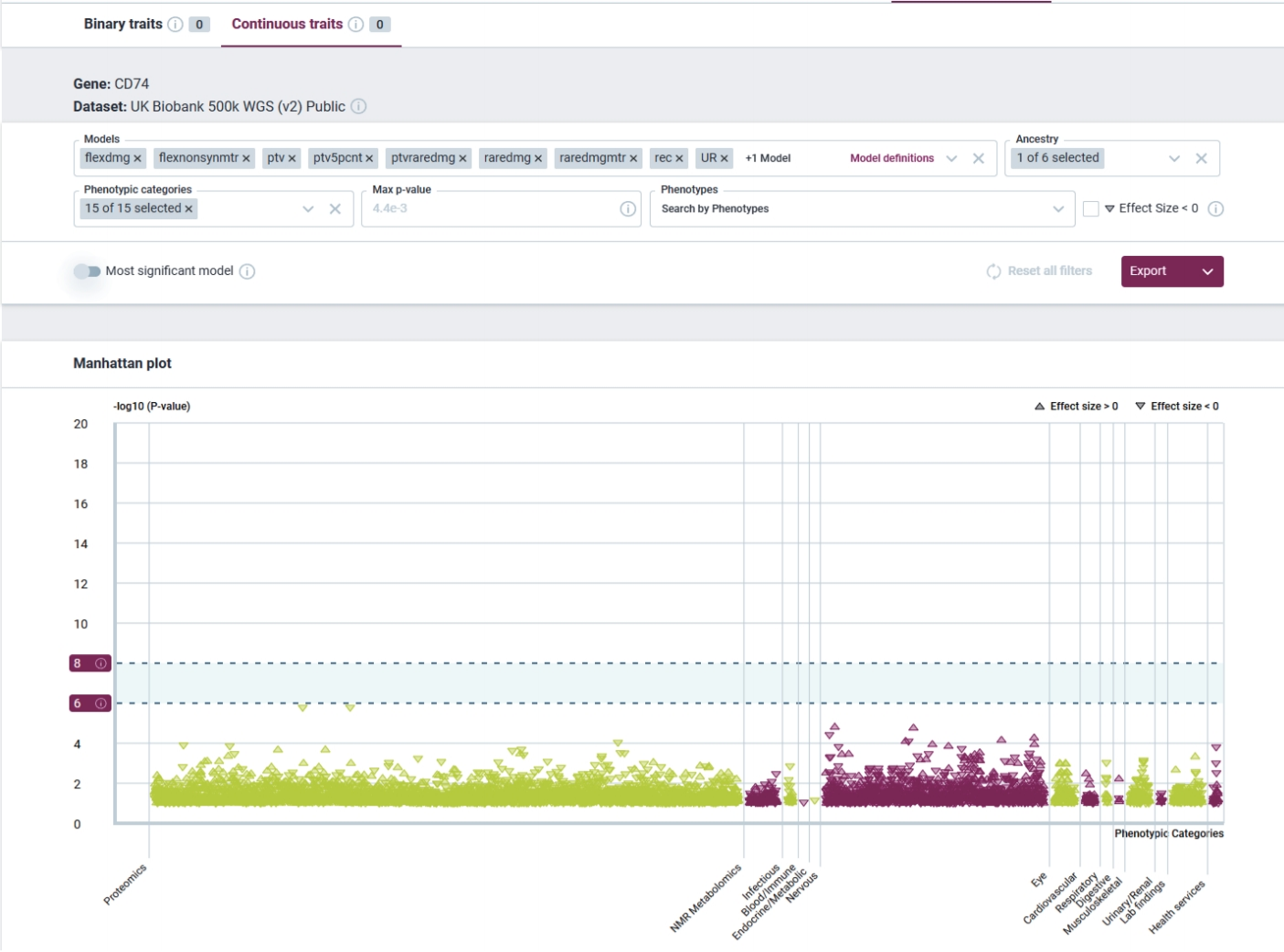
Binary traits
Continuous traits
Gene: CD74
Dataset: UK Biobank 500k WGS (v2) Public
Manhattan plot
-log10 (P-value)
Effect size > 0
Effect size < 0
Phenotypic Categories

(A)

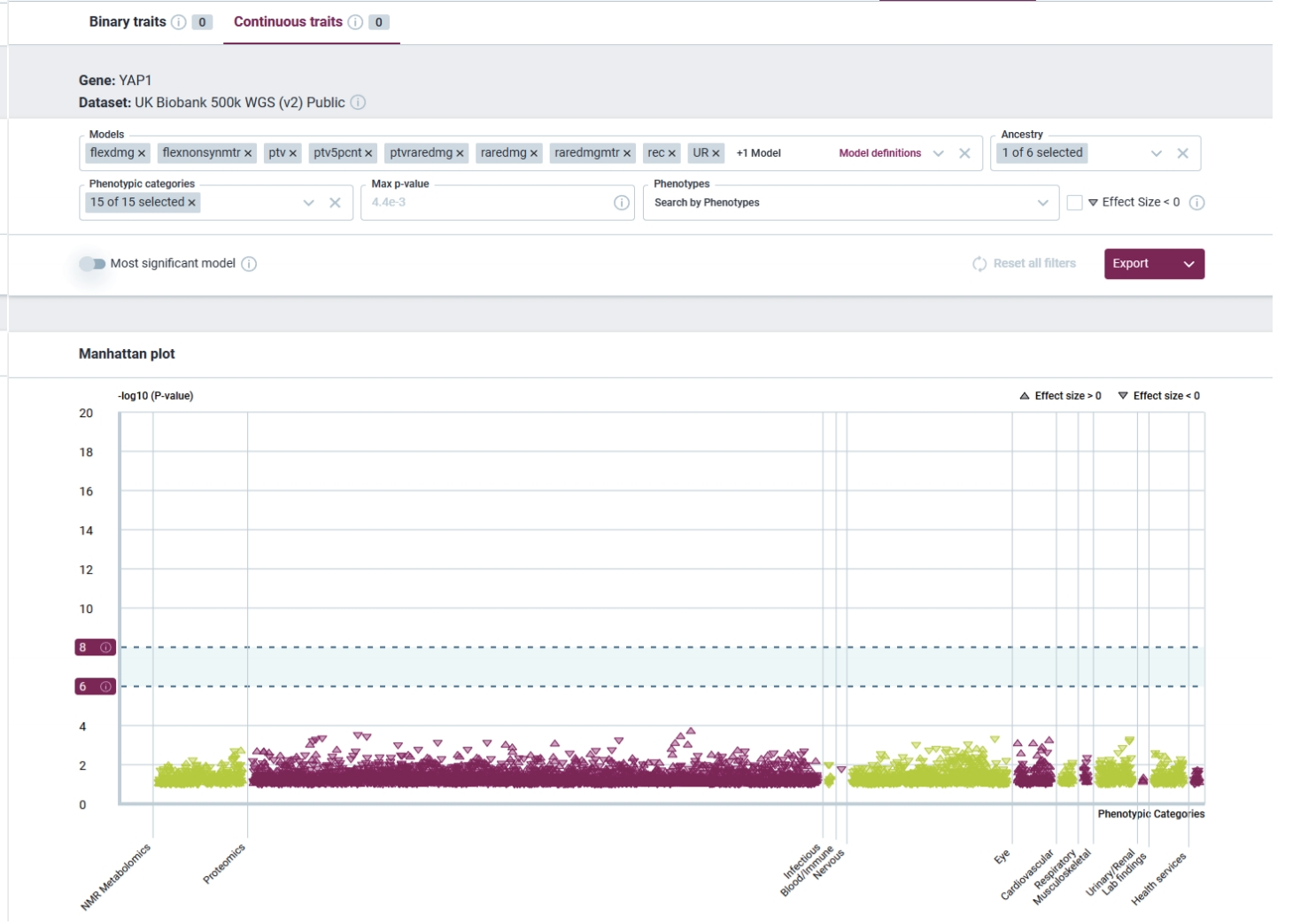
Binary traits
Continuous traits
Gene: YAP1
Dataset: UK Biobank 500k WGS (v2) Public
Manhattan plot
-log10 (P-value)
Effect size > 0
Effect size < 0
Phenotypic Categories

(B)

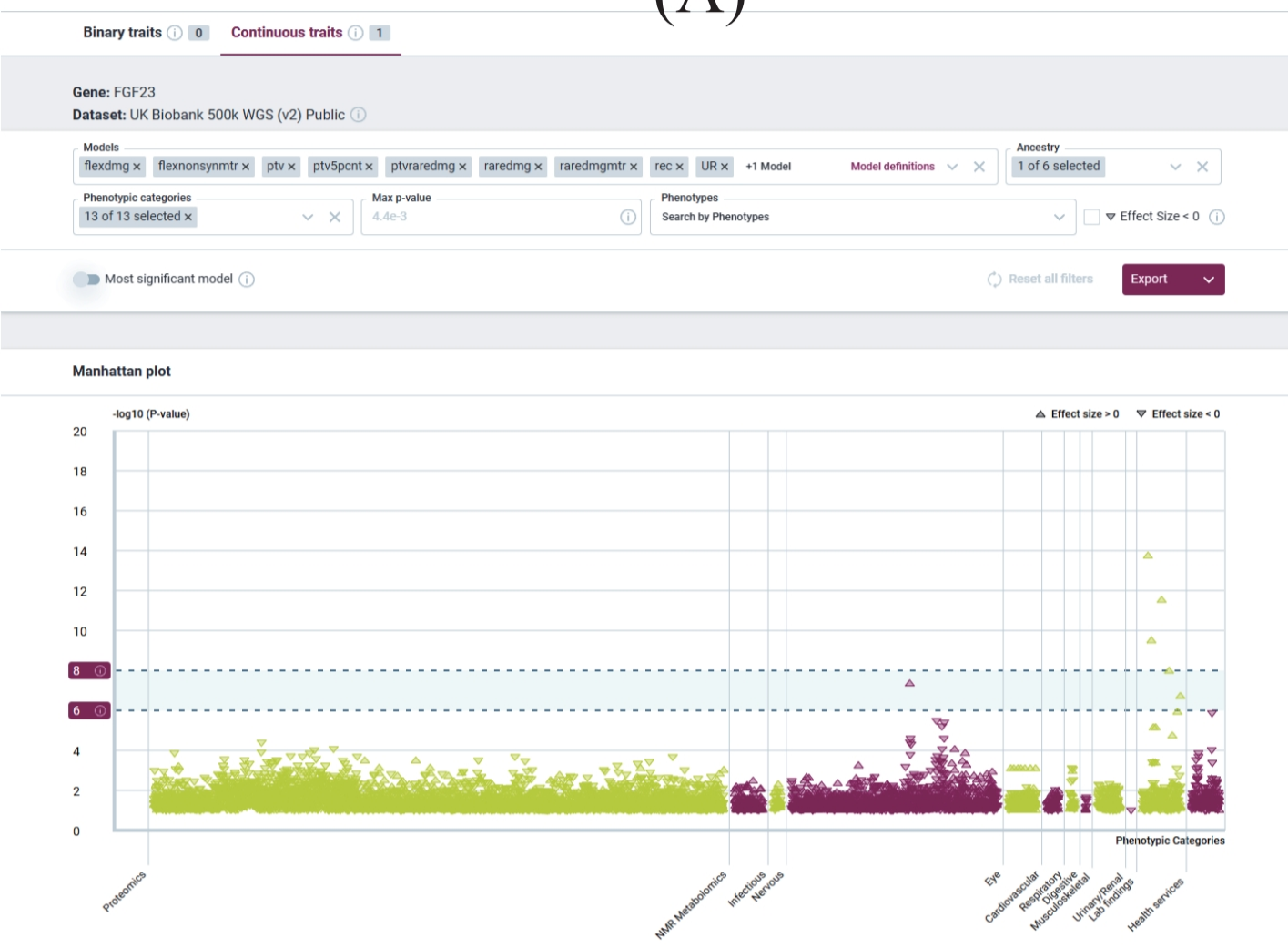
Binary traits
Continuous traits
Gene: FGF23
Dataset: UK Biobank 500k WGS (v2) Public
Manhattan plot
-log10 (P-value)
Effect size > 0
Effect size < 0
Phenotypic Categories

(C)

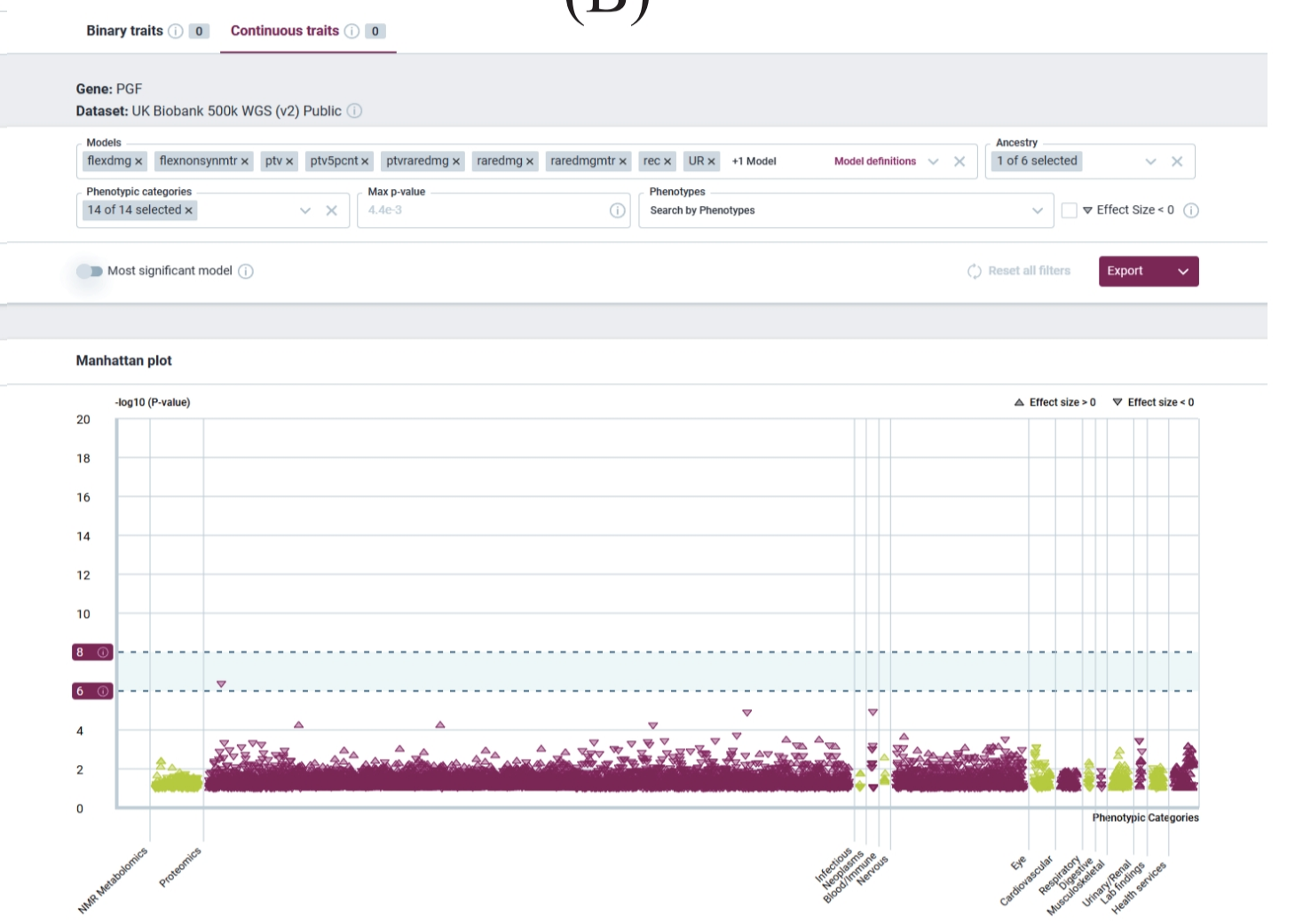
Binary traits
Continuous traits
Gene: PGF
Dataset: UK Biobank 500k WGS (v2) Public
Manhattan plot
-log10 (P-value)
Effect size > 0
Effect size < 0
Phenotypic Categories

(D)

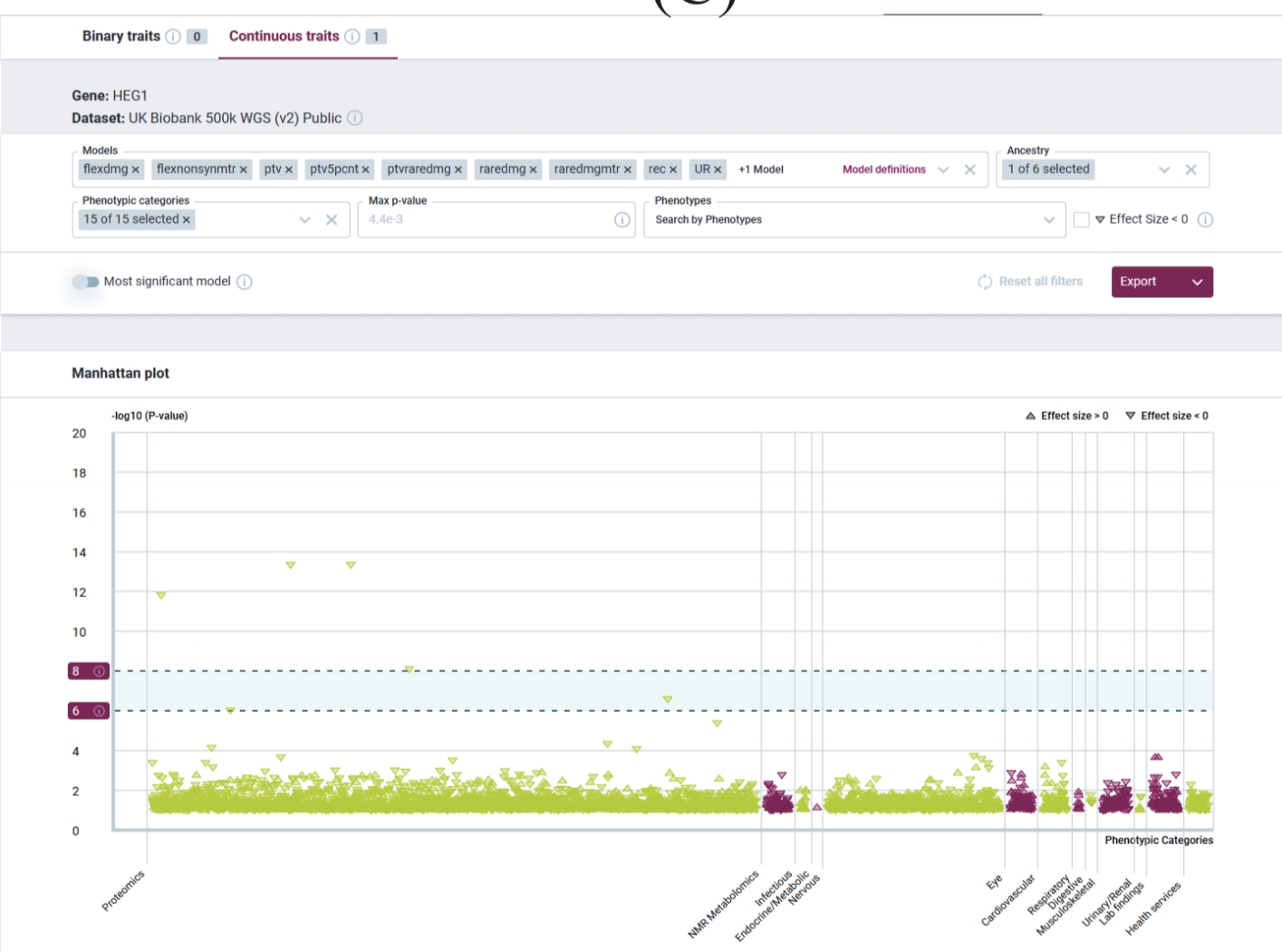
Binary traits
Continuous traits
Gene: HEG1
Dataset: UK Biobank 500k WGS (v2) Public
Manhattan plot
-log10 (P-value)
Effect size > 0
Effect size < 0
Phenotypic Categories

(E)

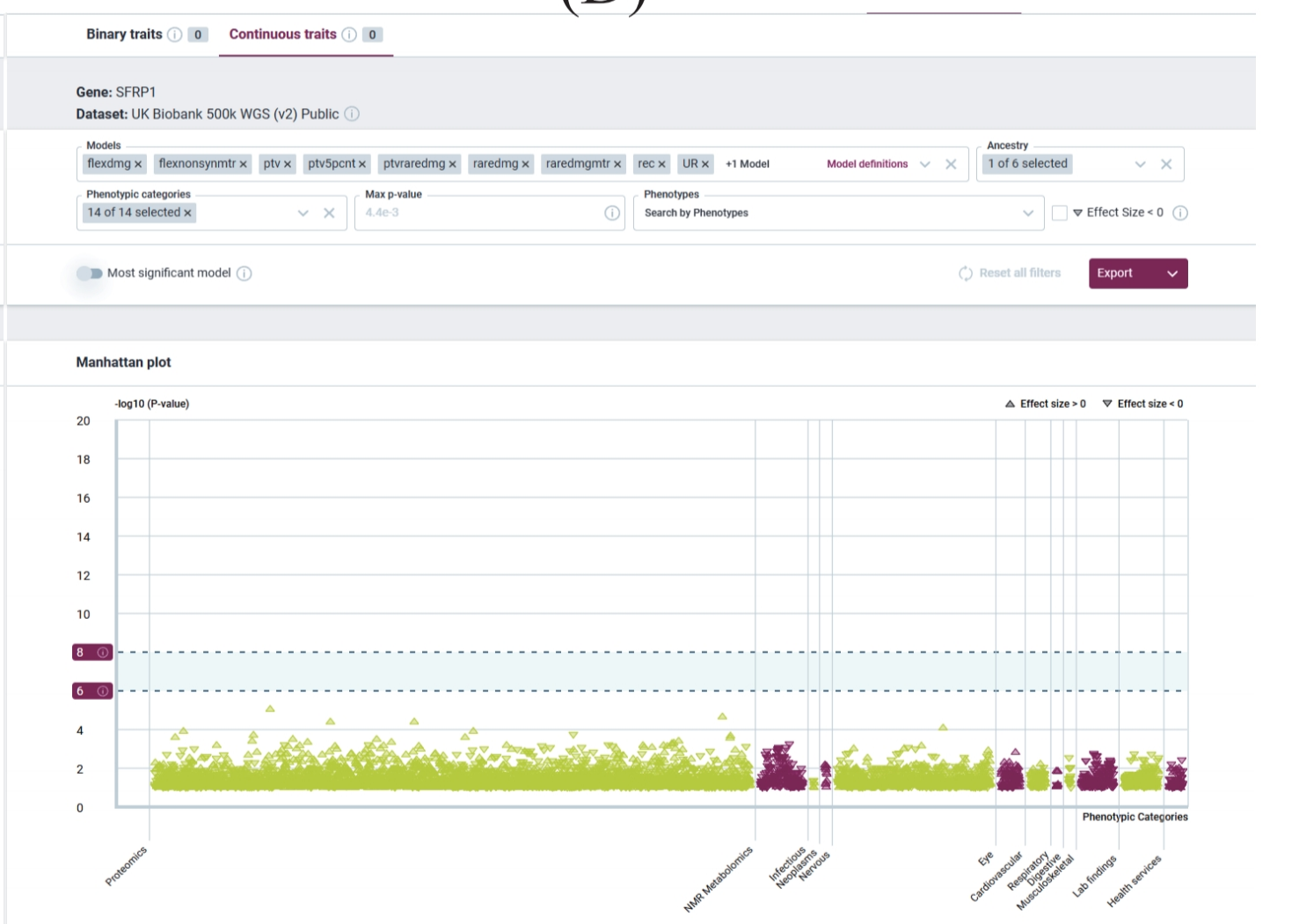
Binary traits
Continuous traits
Gene: SFRP1
Dataset: UK Biobank 500k WGS (v2) Public
Manhattan plot
-log10 (P-value)
Effect size > 0
Effect size < 0
Phenotypic Categories

(F)

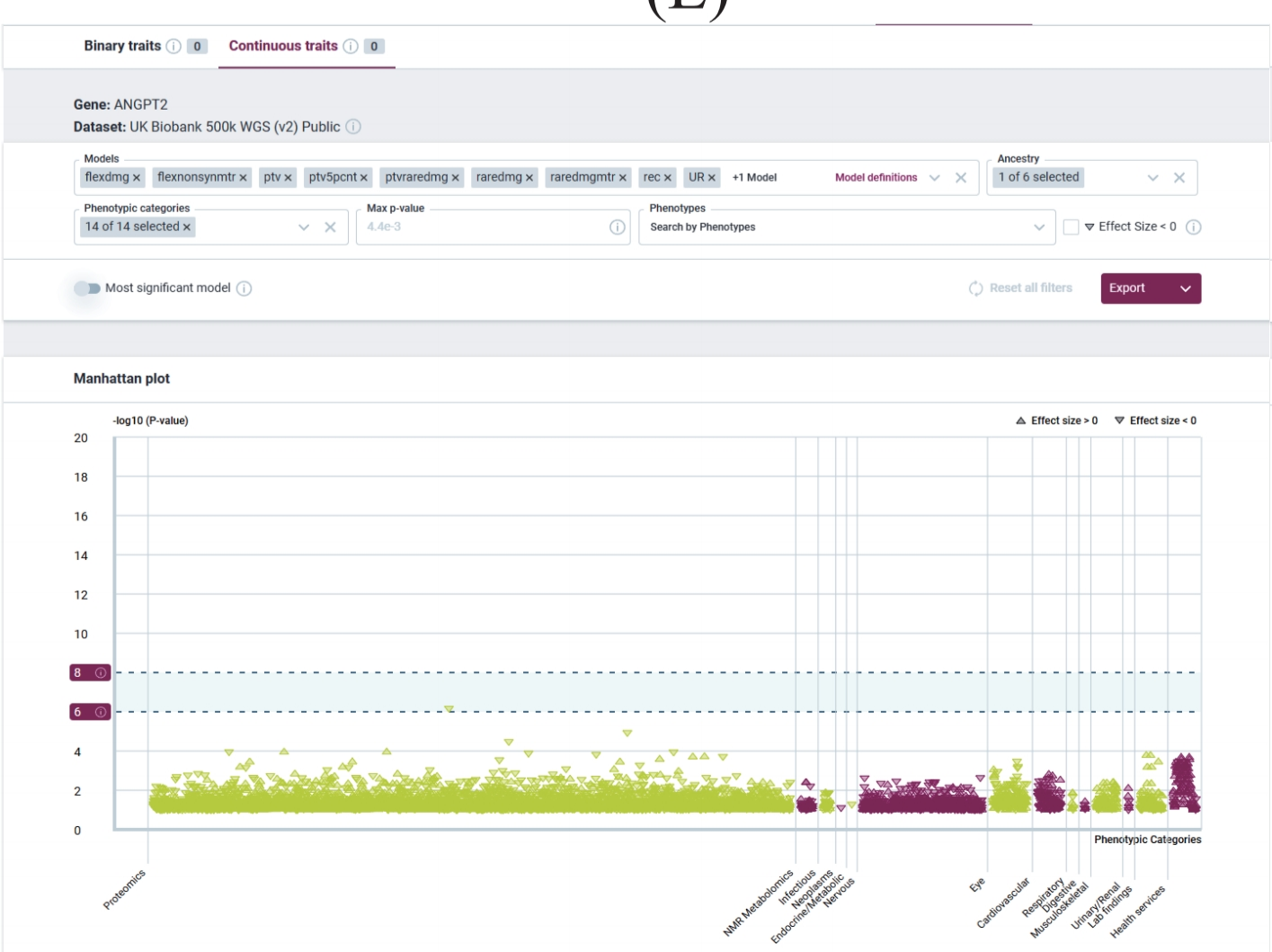
Binary traits
Continuous traits
Gene: ANGPT2
Dataset: UK Biobank 500k WGS (v2) Public
Manhattan plot
-log10 (P-value)
Effect size > 0
Effect size < 0
Phenotypic Categories

(G)

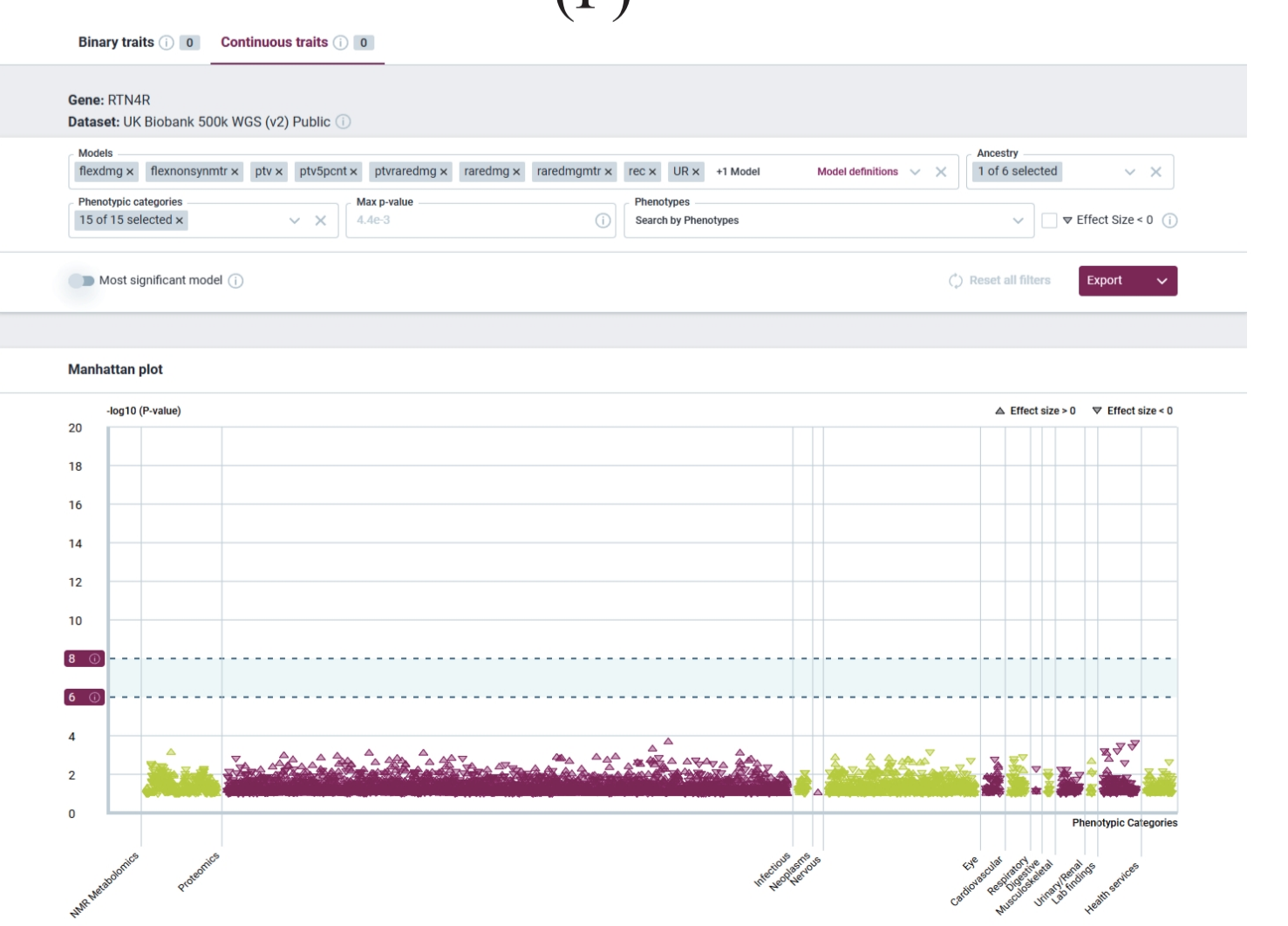
Binary traits
Continuous traits
Gene: RTN4R
Dataset: UK Biobank 500k WGS (v2) Public
Manhattan plot
-log10 (P-value)
Effect size > 0
Effect size < 0
Phenotypic Categories

(H)

Supplementary Table S1: Protein sources and their coding genes

Set 1

Protein	Gene	From
AAMDC	AAMDC	UKB-PPP
AARSD	AARSD	UKB-PPP
ABCA2_	ABCA2	UKB-PPP
ABRAX	ABRAX	UKB-PPP
ACOX1_	ACOX1	UKB-PPP
ACRBP_	ACRBP	UKB-PPP
ACSL1_	ACSL1	UKB-PPP
ACTA2_	ACTA2	UKB-PPP
ACTN4_	ACTN4	UKB-PPP
ACY3_(	ACY3	UKB-PPP
ACYP1_	ACYP1	UKB-PPP
ADA_P(	ADA	UKB-PPP
ADA2_(	ADA2	UKB-PPP
ADAM8	ADAM8	UKB-PPP
ADAMT	ADAMT	UKB-PPP
ADAMT	ADAMT	UKB-PPP
ADAMT	ADAMT	UKB-PPP
ADAMT	ADAMT	UKB-PPP
ADCYA	ADCYA	UKB-PPP
ADD1_I	ADD1	UKB-PPP
ADGRB	ADGRB	UKB-PPP
ADGRD	ADGRD	UKB-PPP
ADGRE	ADGRE	UKB-PPP
ADGRE	ADGRE	UKB-PPP
ADGRG	ADGRG	UKB-PPP
ADGRG	ADGRG	UKB-PPP
ADGRV	ADGRV	UKB-PPP
ADRA2.	ADRA2.	UKB-PPP
AFAP1_	AFAP1	UKB-PPP
AGBL2_	AGBL2	UKB-PPP
AGXT_]	AGXT	UKB-PPP
AHCY_	AHCY	UKB-PPP
AHNAK	AHNAK	UKB-PPP
AHNAK	AHNAK	UKB-PPP
AKAP1:	AKAP1:	UKB-PPP

Set 2

Protein	Gene	From
12423_3	A1CF	deCODE
3708_62	A2M	deCODE
15482_1	A2ML1	deCODE
8759_29	A4GAL'	deCODE
10835_2	A4GNT	deCODE
11926_2	AAGAB	deCODE
12340_1	AARS	deCODE
17320_1	AASDH	deCODE
8935_22	ABCC6	deCODE
17370_1	ABHD1(	deCODE
7825_7_	ABHD1:	deCODE
5715_4_	ABHD1.	deCODE
3342_76	ABL2	deCODE
5261_13	ABL2	deCODE
13578_9	ABLIM:	deCODE
11406_8	ACAD8	deCODE
17857_6	ACADL	deCODE
17393_1	ACADS	deCODE
19262_2	ACADV	deCODE
12343_1	ACAP2	deCODE
19197_9	ACAT1	deCODE
17341_8	ACAT2	deCODE
10075_7	ACBD6	deCODE
19341_3	ACBD6	deCODE
13563_2	ACBD7	deCODE
12700_9	ACLY	deCODE
17400_7	ACOT8	deCODE
9237_54	ACP2	deCODE
6079_59	ACPL2	deCODE
19317_1	ACPP	deCODE
17466_7	ACSF2	deCODE
9843_5_	ACTN1	deCODE
2806_49	ACVR1I	deCODE
19208_8	ACVR2.	deCODE
9412_52	ACVR2I	deCODE

Protein
A1BG_I
ABHD1.
ABL1_P
ABO_P1
ACAA1_
ACADM
ACADS
ACAN_
ACE_P1
ACE2_C
ACHE_I
ACOT1:
ACP1_P
ACP5_P
ACP6_C
ACRV1_
ACTN2_
ACVRL
ACY1_C
ADAM1
ADAM1
ADAM2
ADAM2
ADAM9
ADAMT
ADAMT
ADAMT
ADAMT
ADAMT
ADGRE
ADGRF
ADH1B_
ADH4_I
ADIPOC
ADM_P

AKR7L_	AKR7L	UKB-PPP
AKT1S1	AKT1S1	UKB-PPP
AKT3_(	AKT3	UKB-PPP
ALMS1_	ALMS1	UKB-PPP
ALPP_P	ALPP	UKB-PPP
AMBN_	AMBN	UKB-PPP
AMDHI	AMDHI	UKB-PPP
AMFR_(	AMFR	UKB-PPP
AMOT_	AMOT	UKB-PPP
AMOTL	AMOTL	UKB-PPP
AMPD3_	AMPD3	UKB-PPP
ANGPT	ANGPT	UKB-PPP
ANKMY	ANKMY	UKB-PPP
ANKRA	ANKRA	UKB-PPP
ANP32C	ANP32C	UKB-PPP
AOC3_(	AOC3	UKB-PPP
AP2B1_	AP2B1	UKB-PPP
AP3B1_	AP3B1	UKB-PPP
AP3S2_	AP3S2	UKB-PPP
APBB1I	APBB1I	UKB-PPP
APOBR_	APOBR	UKB-PPP
APPL2_	APPL2	UKB-PPP
ARHGA	ARHGA	UKB-PPP
ARHGE	ARHGE	UKB-PPP
ARHGE	ARHGE	UKB-PPP
ARID4E	ARID4E	UKB-PPP
ARL13E	ARL13E	UKB-PPP
ARMCX	ARMCX	UKB-PPP
ARNT_I	ARNT	UKB-PPP
ARNTL_	ARNTL	UKB-PPP
ART5_(	ART5	UKB-PPP
ASPSCF	ASPSCF	UKB-PPP
ASS1_P	ASS1	UKB-PPP
ATF2_P	ATF2	UKB-PPP
ATF4_P	ATF4	UKB-PPP
ATG16I	ATG16I	UKB-PPP
ATG4A_	ATG4A	UKB-PPP
ATP2B4	ATP2B4	UKB-PPP

12812_2	ACYP2	deCODE
8405_10	ADAM1	deCODE
8654_13	ADAM1	deCODE
6586_19	ADAM1	deCODE
8882_1_	ADAM1	deCODE
8959_61	ADAM1	deCODE
8948_13	ADAM1	deCODE
13549_1	ADAM2	deCODE
8520_8_	ADAM3	deCODE
7827_20	ADAM3	deCODE
10753_3	ADAM7	deCODE
9115_78	ADAMI	deCODE
8845_2_	ADAMT	deCODE
3168_8_	ADAMT	deCODE
6441_62	ADAMT	deCODE
16890_3	ADAMT	deCODE
9794_17	ADCK4	deCODE
8285_64	ADCYA	deCODE
8446_4_	ADCYA	deCODE
8450_36	ADCYA	deCODE
11243_9	ADGRF	deCODE
4551_72	ADGRG	deCODE
17396_2	ADH1A	deCODE
15525_2	ADH1C	deCODE
11369_2	ADH5	deCODE
18206_1	ADH6	deCODE
11377_1	ADH7	deCODE
12457_1	ADI1	deCODE
19207_1	ADK	deCODE
8100_15	ADM2	deCODE
6221_1_	ADPGK	deCODE
18395_5	ADPRH	deCODE
17332_3	ADPRH	deCODE
3347_9_	ADRBK	deCODE
9057_19	ADRM1	deCODE
5023_23	ADSL	deCODE
12644_6	ADSS	deCODE
13998_2	ADSSL1	deCODE

AFM_P4
AFP_P0
AGER_(
AGR2_(
AGR3_(
AGRN_(
AGRP_(
AGT_P(
AHSA1_
AHSG_I
AHSP_(
AIDA_(
AIF1_P5
AIF1L_(
AIFM1_
AK1_P0
AK2_P5
AKR1B1
AKR1B1
AKR1C4
AKT2_P
ALCAM
ALDH1A
ALDH2_
ALDH3A
ALDH5A
ALPI_P(
AMBP_I
AMIGO
AMIGO
AMN_Q
AMY1A
AMY2A
AMY2B
ANG_P(
ANGPT
ANGPT
ANGPT

ATP5F1	ATP5F1	UKB-PPP	18396_1	AES	deCODE	ANGPT
ATP5IF	ATP5IF	UKB-PPP	9889_42	AFAP1L	deCODE	ANGPT
ATP5PC	ATP5PC	UKB-PPP	10435_2	AGA	deCODE	ANGPT
ATP6AI	ATP6AI	UKB-PPP	14291_5	AGAP2	deCODE	ANK2_(
ATP6V1	ATP6V1	UKB-PPP	13960_1	AGAP3	deCODE	ANKRD
ATP6V1	ATP6V1	UKB-PPP	11681_8	AGFG1	deCODE	ANPEP_
ATP6V1	ATP6V1	UKB-PPP	8051_10	AGGF1	deCODE	ANXA1_
ATP6V1	ATP6V1	UKB-PPP	15312_1	AGO1	deCODE	ANXA1
ATRAIL	ATRAIL	UKB-PPP	15323_1	AGO3	deCODE	ANXA1
ATXN1(	ATXN1(	UKB-PPP	12652_3	AHCYL	deCODE	ANXA2_
ATXN2_	ATXN2	UKB-PPP	2714_78	AIMP1	deCODE	ANXA3_
ATXN2I	ATXN2I	UKB-PPP	3839_60	AIP	deCODE	ANXA4_
AXIN1_	AXIN1	UKB-PPP	17432_2	AK4	deCODE	ANXA5_
AZI2_Q	AZI2	UKB-PPP	13613_2	AK5	deCODE	AOC1_I
B3GNT	B3GNT	UKB-PPP	18399_1	AKAP7	deCODE	AP1G2_
B4GAT	B4GAT	UKB-PPP	4192_10	AKR1A	deCODE	APCS_P
BABAM	BABAM	UKB-PPP	12618_5	AKR1C	deCODE	APEX1_
BAG6_I	BAG6	UKB-PPP	17377_1	AKR1C	deCODE	APLP1_
BANK1_	BANK1	UKB-PPP	18398_1	AKR1D	deCODE	APOA1_
BAP18_	BAP18	UKB-PPP	4188_1_	AKR7A	deCODE	APOA2_
BAX_Q	BAX	UKB-PPP	18173_1	AKR7A	deCODE	APOA4_
BCAT1_	BCAT1	UKB-PPP	15627_8	AKT1	deCODE	APOB_I
BCL2L1	BCL2L1	UKB-PPP	15523_9	ALAD	deCODE	APOC1_
BCL7A_	BCL7A	UKB-PPP	18380_7	ALB	deCODE	APOD_I
BCL7B_	BCL7B	UKB-PPP	9835_16	ALDH1	deCODE	APOE_I
BCR_P1	BCR	UKB-PPP	12940_3	ALDH3I	deCODE	APOF_(
BEX3_(	BEX3	UKB-PPP	19136_2	ALDH6	deCODE	APOH_I
BHLHE	BHLHE	UKB-PPP	5864_10	ALDOA	deCODE	APOL1_
BIN2_Q	BIN2	UKB-PPP	18185_1	ALDOB	deCODE	APOM_
BLOC1S	BLOC1S	UKB-PPP	9876_20	ALDOC	deCODE	APP_P0
BLOC1S	BLOC1S	UKB-PPP	8249_12	ALK	deCODE	APRT_I
BNIP2_(	BNIP2	UKB-PPP	18400_5	ALKBH	deCODE	ARAF_I
BPIFB2_	BPIFB2	UKB-PPP	18401_1	ALKBH	deCODE	AREG_I
BRAP_(	BRAP	UKB-PPP	12422_1	ALOX1	deCODE	ARF6_P
BRD3_(	BRD3	UKB-PPP	16926_4	ALPL	deCODE	ARFIP1_
BRK1_(	BRK1	UKB-PPP	6706_18	ALPPL2	deCODE	ARG1_I
BRME1_	BRME1	UKB-PPP	6715_63	ALPPL2	deCODE	ARG2_I
BSND_(	BSND	UKB-PPP	4923_79	AMH	deCODE	ARHGA

BTN1A1	BTN1A1	UKB-PPP
BTN3A2	BTN3A2	UKB-PPP
BTNL10	BTNL10	UKB-PPP
C19orf1	C19orf1	UKB-PPP
C1QA_I	C1QA	UKB-PPP
C1QTNI	C1QTNI	UKB-PPP
C2CD2I	C2CD2I	UKB-PPP
C2orf69_	C2orf69	UKB-PPP
C7orf50_	C7orf50	UKB-PPP
C8B_P0	C8B	UKB-PPP
C9orf40_	C9orf40	UKB-PPP
CA14_Q	CA14	UKB-PPP
CABP2_	CABP2	UKB-PPP
CACNA	CACNA	UKB-PPP
CACNA	CACNA	UKB-PPP
CACNB	CACNB	UKB-PPP
CACNB	CACNB	UKB-PPP
CADPS_	CADPS	UKB-PPP
CALCA_	CALCA	UKB-PPP
CALCO	CALCO	UKB-PPP
CAMLG	CAMLG	UKB-PPP
CAMSA	CAMSA	UKB-PPP
CAPS_C	CAPS	UKB-PPP
CASC3_	CASC3	UKB-PPP
CASP1_	CASP1	UKB-PPP
CASP4_	CASP4	UKB-PPP
CASP7_	CASP7	UKB-PPP
CASP9_	CASP9	UKB-PPP
CBLIF_	CBLIF	UKB-PPP
CBX2_C	CBX2	UKB-PPP
CC2D1A	CC2D1A	UKB-PPP
CCAR2_	CCAR2	UKB-PPP
CCDC28	CCDC28	UKB-PPP
CCER2_	CCER2	UKB-PPP
CCL4_P	CCL4	UKB-PPP
CCN1_C	CCN1	UKB-PPP
CCN2_F	CCN2	UKB-PPP
CCN3_F	CCN3	UKB-PPP

5094_62	AMICA	deCODE
18402_1	AMMEC	deCODE
18403_2	AMPD2	deCODE
12345_4	ANAPC	deCODE
11690_4	ANAPC	deCODE
2500_2_	ANGPT	deCODE
15361_3	ANKRD	deCODE
12445_5	ANKRD	deCODE
7851_30	ANKRD	deCODE
13073_1	ANP32A	deCODE
4194_26	ANP32E	deCODE
10464_6	ANTXR	deCODE
15559_5	ANTXR	deCODE
17835_2	ANXA1	deCODE
5335_73	ANXA6	deCODE
14203_3	ANXA7	deCODE
18244_1	ANXA7	deCODE
18290_6	ANXA8	deCODE
13588_1	ANXA9	deCODE
6434_18	AOC2	deCODE
18405_1	AP1S2	deCODE
13621_3	AP2A2	deCODE
10076_1	AP4M1	deCODE
13583_1	APAF1	deCODE
14206_2	APBB1	deCODE
12822_3	APBB1	deCODE
12753_6	APBB2	deCODE
12761_1	APBB2	deCODE
13589_1	APBB3	deCODE
12784_1	APBB3	deCODE
6599_5_	APCDD	deCODE
6622_90	APLN	deCODE
10627_8	APLP2	deCODE
10605_2	APMAP	deCODE
16621_7	APOA1I	deCODE
15363_3	APOA5	deCODE
13930_3	APOBE	deCODE
6350_43	APOC2	deCODE

ARHGA
ARHGA
ARHGA
ARHGE
ARHGE
ARID3A
ARL2BI
ARSA_I
ARSB_I
ART3_C
ARTN_
ASAH1_
ASAH2_
ASGR1_
ASGR2_
ASPN_C
ASRGL
ATOX1_
ATP1B1
ATP1B2
ATP1B3
ATP1B4
ATRN_
ATXN3_
AXL_P3
AZU1_F
B2M_P6
B3GAT
B4GAL
BACH1_
BAG3_C
BAG4_C
BAIAP2
BAMBI_
BATF_C
BCAM_
BCAN_
BCAT2_

CCN4_	CCN4	UKB-PPP
CCN5_	CCN5	UKB-PPP
CCND2_	CCND2	UKB-PPP
CCNE1_	CCNE1	UKB-PPP
CD101_	CD101	UKB-PPP
CD164_	CD164	UKB-PPP
CD1C_	CD1C	UKB-PPP
CD22_P	CD22	UKB-PPP
CD276_	CD276	UKB-PPP
CD28_P	CD28	UKB-PPP
CD2AP_	CD2AP	UKB-PPP
CD300L	CD300L	UKB-PPP
CD302_	CD302	UKB-PPP
CD34_P	CD34	UKB-PPP
CD3D_	CD3D	UKB-PPP
CD40_P	CD40	UKB-PPP
CD6_P3	CD6	UKB-PPP
CD82_P	CD82	UKB-PPP
CD84_	CD84	UKB-PPP
CD99_P	CD99	UKB-PPP
CDA_P	CDA	UKB-PPP
CDAN1_	CDAN1	UKB-PPP
CDC12	CDC12	UKB-PPP
CDC26_	CDC26	UKB-PPP
CDC27_	CDC27	UKB-PPP
CDH22_	CDH22	UKB-PPP
CDH23_	CDH23	UKB-PPP
CDH4_	CDH4	UKB-PPP
CDHR2_	CDHR2	UKB-PPP
CDK1_	CDK1	UKB-PPP
CDK5R	CDK5R	UKB-PPP
CDKL5_	CDKL5	UKB-PPP
CEACA	CEACA	UKB-PPP
CEACA	CEACA	UKB-PPP
CEACA	CEACA	UKB-PPP
CEACA	CEACA	UKB-PPP
CEACA	CEACA	UKB-PPP
CELA3	CELA3	UKB-PPP

6461_54	APOC3	deCODE
16823_7	APOL3	deCODE
12825_1	APPL1	deCODE
17736_1	APTX	deCODE
11363_5	AQP4	deCODE
19239_5	ARF1	deCODE
12578_1	ARF3	deCODE
18408_2	ARF4	deCODE
18409_6	ARF5	deCODE
11556_1	ARFGA	deCODE
11664_3	ARFGA	deCODE
12630_8	ARFIP2	deCODE
6289_78	ARHGA	deCODE
6454_38	ARHGD	deCODE
9846_32	ARHGD	deCODE
12848_9	ARHGE	deCODE
13519_1	ARHGE	deCODE
13932_4	ARHGE	deCODE
9898_16	ARID1A	deCODE
12392_3	ARL1	deCODE
12433_8	ARL11	deCODE
18410_2	ARL14	deCODE
18411_8	ARL15	deCODE
12587_6	ARL2	deCODE
12571_1	ARL3	deCODE
18413_2	ARL4D	deCODE
18414_2	ARL5A	deCODE
17404_5	ARL5B	deCODE
18415_1	ARL6	deCODE
13503_1	ARL6IP	deCODE
8569_14	ARL8B	deCODE
18416_3	ARL9	deCODE
9107_59	ARMC1	deCODE
8785_1_	ARMC5	deCODE
12689_5	ARPC1	deCODE
13573_5	ARPC3	deCODE
18419_2	ARPC5	deCODE
4963_19	ARPP19	deCODE

BCHE_
BCL2_P
BCL2L1
BCL2L1
BDNF_
BECN1_
BGLAP_
BGN_P
BHMT2_
BID_P5
BIRC2_
BLMH_
BLNK_
BLVRB_
BMP10_
BMP4_
BMP6_
BMPER_
BNIP3L_
BOC_Q
BOLA1_
BOLA2_
BPIFA2_
BPIFB1_
BRD1_
BRD2_
BRDT_
BRSK2_
BSG_P3
BST1_Q
BST2_Q
BTC_P3
BTD_P4
BTLA_
BTN2A
C1GAL
C1QBP_
C1QL2_

CELSR2	CELSR2	UKB-PPP	12860_7	ARPP21	deCODE	C1QTNI
CEMIP2	CEMIP2	UKB-PPP	12643_4	ARRB1	deCODE	C1QTNI
CEND1_	CEND1	UKB-PPP	12352_7	ARRDC	deCODE	C1QTNI
CENPF_	CENPF	UKB-PPP	8269_32	ARSK	deCODE	C1R_P0
CENPJ_	CENPJ	UKB-PPP	6576_1_	ART4	deCODE	C1RL_C
CEP112_	CEP112	UKB-PPP	18417_3	AS3MT	deCODE	C1S_P0
CEP152_	CEP152	UKB-PPP	13518_5	ASAP2	deCODE	C2_P06
CEP164_	CEP164	UKB-PPP	19601_1	ASB9	deCODE	C3_P01
CEP170_	CEP170	UKB-PPP	10647_1	ASCC1	deCODE	C4BPB_
CEP20_	CEP20	UKB-PPP	18172_7	ASF1A	deCODE	C5_P01
CEP290_	CEP290	UKB-PPP	12622_9	ASH1L	deCODE	C7_P10
CEP350_	CEP350	UKB-PPP	12832_1	ASH2L	deCODE	C9_P02
CEP43_	CEP43	UKB-PPP	6951_26	ASIC4	deCODE	CA1_P0
CEP85_	CEP85	UKB-PPP	5676_54	ASIP	deCODE	CA11_C
CERT_C	CERT	UKB-PPP	11241_8	ASL	deCODE	CA12_C
CES2_O	CES2	UKB-PPP	10089_7	ASMTL	deCODE	CA13_C
CES3_Q	CES3	UKB-PPP	13620_1	ASNA1	deCODE	CA2_P0
CGB3_C	CGB3	UKB-PPP	18187_1	ASPA	deCODE	CA3_P0
CGN_Q	CGN	UKB-PPP	6998_10	ASPH	deCODE	CA4_P2
CHAC2_	CHAC2	UKB-PPP	10902_5	ASPRV1	deCODE	CA5A_I
CHCHD	CHCHD	UKB-PPP	7993_23	ASTL	deCODE	CA6_P2
CHM_P	CHM	UKB-PPP	6625_31	ATAD1	deCODE	CA7_P4
CHMP1	CHMP1	UKB-PPP	13043_1	ATAD2	deCODE	CA8_P3
CHMP6_	CHMP6	UKB-PPP	11277_2	ATF6	deCODE	CA9_Q1
CHRM1	CHRM1	UKB-PPP	11387_3	ATF6B	deCODE	CACYB
CIAPIN	CIAPIN	UKB-PPP	18156_7	ATG3	deCODE	CALB1_
CIT_O1	CIT	UKB-PPP	13629_2	ATG4B	deCODE	CALB2_
CLASP1	CLASP1	UKB-PPP	12597_6	ATG5	deCODE	CALCB_
CLEC14	CLEC14	UKB-PPP	12627_9	ATG7	deCODE	CALCO
CLEC1A	CLEC1A	UKB-PPP	13659_3	ATP13A	deCODE	CALY_
CLEC5A	CLEC5A	UKB-PPP	13510_7	ATP2A3	deCODE	CAMKK
CLIP2_	CLIP2	UKB-PPP	9994_21	ATP4B	deCODE	CANT1_
CLNS1A	CLNS1A	UKB-PPP	4965_27	ATP5B	deCODE	CAPG_I
CLSPN_	CLSPN	UKB-PPP	7788_1_	ATP5J	deCODE	CAPN3_
CLUL1_	CLUL1	UKB-PPP	11539_4	ATP5J2	deCODE	CARHS
CMC1_	CMC1	UKB-PPP	9202_30	ATP5O	deCODE	CASP10
CMIP_C	CMIP	UKB-PPP	3091_70	AURKA	deCODE	CASP2_
CNGB3_	CNGB3	UKB-PPP	3346_72	AURKB	deCODE	CASP3_

CNPY2_	CNPY2	UKB-PPP
CNST_(	CNST	UKB-PPP
CNTN3_	CNTN3	UKB-PPP
CNTNA	CNTNA	UKB-PPP
COL24A	COL24A	UKB-PPP
COL4A	COL4A	UKB-PPP
COL4A	COL4A	UKB-PPP
COL5A	COL5A	UKB-PPP
COL9A	COL9A	UKB-PPP
COPB2_	COPB2	UKB-PPP
COQ7_(	COQ7	UKB-PPP
CORO1	CORO1	UKB-PPP
CORO6_	CORO6	UKB-PPP
COX6B	COX6B	UKB-PPP
CPA1_P	CPA1	UKB-PPP
CPPED1	CPPED1	UKB-PPP
CPTP_Q	CPTP	UKB-PPP
CPXM2_	CPXM2	UKB-PPP
CRACR	CRACR	UKB-PPP
CREB3_	CREB3	UKB-PPP
CREBZI	CREBZI	UKB-PPP
CRELD	CRELD	UKB-PPP
CRHR1_	CRHR1	UKB-PPP
CRTAP_	CRTAP	UKB-PPP
CRX_O	CRX	UKB-PPP
CRYM_	CRYM	UKB-PPP
CSH1_P	CSH1	UKB-PPP
CSPG5_	CSPG5	UKB-PPP
CSTB_P	CSTB	UKB-PPP
CTAG1	CTAG1	UKB-PPP
CTBS_(	CTBS	UKB-PPP
CTNNA	CTNNA	UKB-PPP
CWC15_	CWC15	UKB-PPP
CXCL3_	CXCL3	UKB-PPP
CYP24A	CYP24A	UKB-PPP
CYTH3_	CYTH3	UKB-PPP
DAAM1	DAAM1	UKB-PPP
DAB2_I	DAB2	UKB-PPP

12925_1	AXIN2	deCODE
8429_16	AXIN2	deCODE
9312_8_	AZGP1	deCODE
9541_15	B3GAL	deCODE
11638_4	B3GAL	deCODE
7981_23	B3GAL	deCODE
5727_35	B3GAL	deCODE
7980_72	B3GNT	deCODE
16585_1	B3GNT	deCODE
7082_2_	B3GNT	deCODE
9297_12	B3GNT	deCODE
8976_13	B4GALI	deCODE
9595_11	B4GAL	deCODE
6921_24	B4GAL	deCODE
10425_3	B4GAL	deCODE
10832_2	B4GAL	deCODE
7806_33	B4GAL	deCODE
12756_3	BACH2	deCODE
5870_23	BAD	deCODE
17195_4	BAG1	deCODE
19228_1	BAG2	deCODE
12743_1	BAG5	deCODE
6294_11	BAGE2	deCODE
6442_6_	BAGE3	deCODE
15365_4	BAI3	deCODE
13977_2	BARD1	deCODE
8858_21	BATF3	deCODE
11570_9	BCAP2	deCODE
5262_57	BCAR3	deCODE
12634_7	BCAR3	deCODE
18304_1	BCCIP	deCODE
8768_4_	BCL10	deCODE
3413_50	BCL2A1	deCODE
7249_30	BCL2L1	deCODE
13097_1	BCL2L2	deCODE
13111_7	BCL6	deCODE
17329_2	BDH2	deCODE
8929_7_	BDP1	deCODE

CASP8_
CASQ2_
CAT_P(
CBLN1_
CBLN4_
CBS_P3
CCDC1
CCDC5(
CCDC8(
CCL11_
CCL13_
CCL14_
CCL15_
CCL16_
CCL17_
CCL18_
CCL19_
CCL2_P
CCL20_
CCL21_
CCL22_
CCL23_
CCL24_
CCL25_
CCL26_
CCL27_
CCL28_
CCL3_P
CCL5_P
CCL7_P
CCL8_P
CCS_O1
CCT5_P
CD109_
CD14_P
CD160_
CD163_
CD164L

DAND5_	DAND5	UKB-PPP	10959_1	BET1L	deCODE	CD177_
DARS1_	DARS1	UKB-PPP	9574_11	BIN1	deCODE	CD2_P0
DBH_P(	DBH	UKB-PPP	4973_18	BIRC3	deCODE	CD200_
DBN1_(	DBN1	UKB-PPP	3472_40	BIRC5	deCODE	CD200R
DCBLD	DCBLD	UKB-PPP	15412_4	BIRC7	deCODE	CD207_
DCC_P4	DCC	UKB-PPP	15504_3	BIRC7	deCODE	CD209_
DCDC2(	DCDC2(	UKB-PPP	11338_4	BLK	deCODE	CD226_
DCLRE1	DCLRE1	UKB-PPP	11382_5	BLVRA	deCODE	CD244_
DCTN1_	DCTN1	UKB-PPP	3348_49	BMP1	deCODE	CD248_
DDA1_(	DDA1	UKB-PPP	11129_6	BMP15	deCODE	CD27_P
DDHD2_	DDHD2	UKB-PPP	15666_2	BMP2	deCODE	CD274_
DDI2_Q	DDI2	UKB-PPP	16748_1	BMP3	deCODE	CD300A
DDX1_(	DDX1	UKB-PPP	2972_57	BMP7	deCODE	CD300C
DDX39A	DDX39A	UKB-PPP	15668_1	BMP8B	deCODE	CD300E
DDX4_(	DDX4	UKB-PPP	10550_3	BMPR1B	deCODE	CD300L
DDX53_	DDX53	UKB-PPP	4862_63	BMPR2	deCODE	CD33_P
DENND	DENND	UKB-PPP	3414_40	BMX	deCODE	CD36_P
DFFA_(	DFFA	UKB-PPP	7045_4_	BNIP3	deCODE	CD38_P
DGKA_	DGKA	UKB-PPP	5980_55	BOLA3	deCODE	CD3E_P
DGKZ_(	DGKZ	UKB-PPP	12020_3	BPGM	deCODE	CD3G_P
DHODH	DHODH	UKB-PPP	17813_2	BPHL	deCODE	CD4_P0
DHRS4L	DHRS4L	UKB-PPP	4126_22	BPI	deCODE	CD40LG
DIPK1C	DIPK1C	UKB-PPP	6473_55	BPIFA1	deCODE	CD46_P
DIPK2B	DIPK2B	UKB-PPP	17814_8	BPNT1	deCODE	CD48_P
DLGAP5	DLGAP5	UKB-PPP	12482_5	BPTF	deCODE	CD5_P0
DMD_P	DMD	UKB-PPP	15669_7	BRAF	deCODE	CD55_P
DMP1_(	DMP1	UKB-PPP	10876_3	BRAT1	deCODE	CD58_P
DNAJA1	DNAJA1	UKB-PPP	10043_3	BRD4	deCODE	CD59_P
DNAJB8	DNAJB8	UKB-PPP	19596_1	BRF1	deCODE	CD5L_Q
DNAJC2	DNAJC2	UKB-PPP	6612_90	BRICD5	deCODE	CD63_P
DNAJC6	DNAJC6	UKB-PPP	11671_1	BRPF1	deCODE	CD69_Q
DNAJC9	DNAJC9	UKB-PPP	11325_8	BTG2	deCODE	CD7_P0
DNLZ_(	DNLZ	UKB-PPP	19202_1	BTK	deCODE	CD70_P
DNM1_(	DNM1	UKB-PPP	7081_2_	BTN3A1	deCODE	CD72_P
DNM3_(	DNM3	UKB-PPP	17692_2	BTN3A3	deCODE	CD74_P
DNMBP	DNMBP	UKB-PPP	10848_1	BTNL3	deCODE	CD79B_
DNPEP_	DNPEP	UKB-PPP	9026_40	BTNL8	deCODE	CD80_P
DNPH1_	DNPH1	UKB-PPP	7950_14	BTNL9	deCODE	CD83_Q

DPP6_P	DPP6	UKB-PPP	12033_3	BUB1	deCODE	CD86_P
DRG2_	DRG2	UKB-PPP	11220_5	C10orf1	deCODE	CD8A_
DSG4_C	DSG4	UKB-PPP	8220_15	C10orf3	deCODE	CD93_C
DTNB_	DTNB	UKB-PPP	14123_3	C10orf5	deCODE	CD99L2
DTX2_C	DTX2	UKB-PPP	11531_2	C10orf5	deCODE	CDC25
DTX3_C	DTX3	UKB-PPP	8307_47	C11orf6	deCODE	CDC37_
DUOX2	DUOX2	UKB-PPP	8686_34	C11orf7	deCODE	CDC42
DUSP29	DUSP29	UKB-PPP	11116_1	C11orf8	deCODE	CDCP1_
DXO_O	DXO	UKB-PPP	8383_20	C11orf9	deCODE	CDH1_
DYNC1	DYNC1	UKB-PPP	8303_10	C12orf1	deCODE	CDH15_
ECHDC	ECHDC	UKB-PPP	6439_59	C14orf9	deCODE	CDH17_
ECSCR_	ECSCR	UKB-PPP	12394_5	C16orf5	deCODE	CDH2_
EDEM2_	EDEM2	UKB-PPP	9070_1_	C17orf6	deCODE	CDH3_
EDNRB	EDNRB	UKB-PPP	6571_75	C17orf7	deCODE	CDH5_
EFCAB	EFCAB	UKB-PPP	9030_56	C17orf8	deCODE	CDH6_
EFNA1_	EFNA1	UKB-PPP	8236_8_	C18orf3	deCODE	CDHR1_
EGFL7_	EGFL7	UKB-PPP	9445_44	C19orf1	deCODE	CDHR5_
EHD3_C	EHD3	UKB-PPP	7183_10	C19orf8	deCODE	CDKN1
EIF2AK	EIF2AK	UKB-PPP	7821_6_	C1D	deCODE	CDKN2
EIF2AK	EIF2AK	UKB-PPP	8366_19	C1orf11	deCODE	CDNF_
EIF2S2_	EIF2S2	UKB-PPP	6896_3_	C1orf16	deCODE	CDON_
EIF4G1_	EIF4G1	UKB-PPP	10667_7	C1orf18	deCODE	CDSN_
ELAC1_	ELAC1	UKB-PPP	8102_23	C1orf18	deCODE	CEACA
ELAVL	ELAVL	UKB-PPP	8035_6_	C1orf19	deCODE	CEACA
ELN_P1	ELN	UKB-PPP	8088_56	C1orf21	deCODE	CEACA
ELOA_	ELOA	UKB-PPP	7989_5_	C1orf22	deCODE	CEACA
ELOB_	ELOB	UKB-PPP	6937_25	C1orf43	deCODE	CEACA
ENOPH	ENOPH	UKB-PPP	14100_6	C1QC	deCODE	CEBPA_
ENTR1_	ENTR1	UKB-PPP	6404_20	C1QL1	deCODE	CEBPB_
EP300_	EP300	UKB-PPP	7132_55	C1QL4	deCODE	CELA2
EPB41L	EPB41L	UKB-PPP	7251_64	C1QTN	deCODE	CES1_P
EPCAM	EPCAM	UKB-PPP	7046_6_	C20orf1	deCODE	CETN2_
EPGN_	EPGN	UKB-PPP	5981_6_	C21orf3	deCODE	CETN3_
EPHX2_	EPHX2	UKB-PPP	7073_69	C22orf1	deCODE	CFB_P0
EPN1_	EPN1	UKB-PPP	6362_6_	C2orf40	deCODE	CFC1_P
EPPK1_	EPPK1	UKB-PPP	5677_15	C2orf66	deCODE	CFD_P0
EPS8L2	EPS8L2	UKB-PPP	6994_19	C3orf18	deCODE	CFH_P0
ERBIN_	ERBIN	UKB-PPP	9449_15	C4BPA	deCODE	CFHR2_

ERC2_C	ERC2	UKB-PPP	9490_3_	C4orf32	deCODE	CFHR4_
ERI1_Q	ERI1	UKB-PPP	6378_2_	C5orf38	deCODE	CFHR5_
ERVV-1	ERVV-1	UKB-PPP	9100_32	C5orf46	deCODE	CFI_P05
ESPL1_	ESPL1	UKB-PPP	13378_8	C5orf63	deCODE	CFP_P2
ESR1_P	ESR1	UKB-PPP	4127_75	C6	deCODE	CGA_P0
ESYT2_	ESYT2	UKB-PPP	10885_3	C6orf89	deCODE	CGREF
EVI2B_	EVI2B	UKB-PPP	9544_24	C7orf69	deCODE	CHAD_
EVI5_O	EVI5	UKB-PPP	8927_6_	C7orf73	deCODE	CHCHD
EXOSC	EXOSC	UKB-PPP	14708_5	C8G	deCODE	CHEK2_
EXTL1_	EXTL1	UKB-PPP	7778_10	C9orf89	deCODE	CHGA_
F11R_Q	F11R	UKB-PPP	13666_2	CA10	deCODE	CHGB_
F12_P0	F12	UKB-PPP	17673_3	CA5B	deCODE	CHI3L1_
F2R_P2	F2R	UKB-PPP	17757_8	CAB39	deCODE	CHIT1_
FAM13	FAM13	UKB-PPP	7105_7_	CABLE	deCODE	CHL1_C
FAM3C_	FAM3C	UKB-PPP	7104_71	CABP7	deCODE	CHP1_C
FARSA_	FARSA	UKB-PPP	8885_6_	CACNA	deCODE	CHRDL
FBLN2_	FBLN2	UKB-PPP	11130_1	CACNB	deCODE	CHRDL
FBN2_P	FBN2	UKB-PPP	3326_58	CADM1	deCODE	CILP_O
FCRL2_	FCRL2	UKB-PPP	16907_3	CADM2	deCODE	CINP_Q
FDX1_P	FDX1	UKB-PPP	3630_27	CADM3	deCODE	CIRBP_
FDX2_C	FDX2	UKB-PPP	10933_1	CALN1	deCODE	CKAP4_
FES_P0	FES	UKB-PPP	5264_65	CALR	deCODE	CKB_P1
FGA_P0	FGA	UKB-PPP	3592_4_	CAMK1	deCODE	CKMT1
FGD3_C	FGD3	UKB-PPP	3418_12	CAMK1	deCODE	CLC_Q0
FGF21_	FGF21	UKB-PPP	3350_53	CAMK2	deCODE	CLEC10
FGFBP2	FGFBP2	UKB-PPP	3351_1_	CAMK2	deCODE	CLEC11
FHIP2A	FHIP2A	UKB-PPP	3419_49	CAMK2	deCODE	CLEC12
FKBP5_	FKBP5	UKB-PPP	18904_2	CAMK4	deCODE	CLEC1B
FKBPL_	FKBPL	UKB-PPP	15481_4	CAMP	deCODE	CLEC2D
FLI1_Q	FLI1	UKB-PPP	9384_17	CAMP	deCODE	CLEC3B
FMNL1_	FMNL1	UKB-PPP	13937_7	CAND1	deCODE	CLEC4A
FNDC1_	FNDC1	UKB-PPP	8834_58	CANX	deCODE	CLEC4C
FNTA_P	FNTA	UKB-PPP	12034_2	CAP1	deCODE	CLEC4D
FOS_P0	FOS	UKB-PPP	14684_1	CAPN2	deCODE	CLEC4G
FOSB_P	FOSB	UKB-PPP	18299_1	CAPNS1	deCODE	CLEC4M
FOXJ3_	FOXJ3	UKB-PPP	13688_2	CAPSL	deCODE	CLEC6A
FOXO1_	FOXO1	UKB-PPP	11449_2	CAPZA	deCODE	CLEC7A
FRMD4	FRMD4	UKB-PPP	18267_7	CARD1	deCODE	CLGN_

FRMD7_	FRMD7	UKB-PPP	18190_1	CARD18	deCODE	CLIC5_(
FUCA1_	FUCA1	UKB-PPP	11405_1	CARD9	deCODE	CLINT1
FUOM_	FUOM	UKB-PPP	14098_2	CARS	deCODE	CLMP_(
FUS_P3	FUS	UKB-PPP	5743_82	CARTPT	deCODE	CLPP_Q
FUT1_P	FUT1	UKB-PPP	10613_3	CASC4	deCODE	CLPS_P
FXYD5_	FXYD5	UKB-PPP	8838_10	CASC4	deCODE	CLSTN1
FYB1_C	FYB1	UKB-PPP	16754_4	CASK	deCODE	CLSTN2
FZD8_Q	FZD8	UKB-PPP	16617_1	CASP14	deCODE	CLSTN3
GABRA	GABRA	UKB-PPP	11263_5	CASQ1	deCODE	CLTA_P
GAD2_(	GAD2	UKB-PPP	12855_1	CASS4	deCODE	CLU_P1
GADD4	GADD4	UKB-PPP	3026_5_	CAST	deCODE	CNDP1_
GALNT	GALNT	UKB-PPP	9457_3_	CAV2	deCODE	CNP_P0
GALNT	GALNT	UKB-PPP	8690_25	CAV3	deCODE	CNPY4_
GAMT_	GAMT	UKB-PPP	10048_7	CBFB	deCODE	CNTF_P
GART_I	GART	UKB-PPP	12016_6	CBL	deCODE	CNTN1_
GAS2_C	GAS2	UKB-PPP	12381_2	CBR1	deCODE	CNTN2_
GASK1A	GASK1A	UKB-PPP	14091_4	CBR3	deCODE	CNTN4_
GAST_P	GAST	UKB-PPP	18817_5	CBX1	deCODE	CNTN5_
GATA3_	GATA3	UKB-PPP	18868_7	CBX3	deCODE	CNTNA
GATD3_	GATD3	UKB-PPP	4540_11	CBX5	deCODE	COCH_(
GBA_P(	GBA	UKB-PPP	13027_2	CBX7	deCODE	COL15A
GBP4_Q	GBP4	UKB-PPP	17365_7	CCBL1	deCODE	COL18A
GCC1_(	GCC1	UKB-PPP	12682_5	CCBL2	deCODE	COL1A1
GCHFR_	GCHFR	UKB-PPP	12788_6	CCDC10	deCODE	COL28A
GET3_C	GET3	UKB-PPP	6388_21	CCDC12	deCODE	COL2A1
GFOD2_	GFOD2	UKB-PPP	7797_11	CCDC16	deCODE	COL3A1
GGACT	GGACT	UKB-PPP	18264_1	CCDC25	deCODE	COL6A3
GGCT_(	GGCT	UKB-PPP	12790_1	CCDC51	deCODE	COL9A1
GGT1_P	GGT1	UKB-PPP	11158_4	CCDC64	deCODE	COLEC1
GGT5_P	GGT5	UKB-PPP	7792_58	CCDC90	deCODE	COMMI
GHRHR	GHRHR	UKB-PPP	6918_18	CCK	deCODE	COMMI
GID8_Q	GID8	UKB-PPP	2770_51	CCL1	deCODE	COMP_
GIGYF2	GIGYF2	UKB-PPP	2783_18	CCL3L1	deCODE	COMT_
GIMAP7	GIMAP7	UKB-PPP	2781_63	CCL4L1	deCODE	COPE_C
GIMAP8	GIMAP8	UKB-PPP	12347_2	CCM2	deCODE	COX5B_
GIPC2_(	GIPC2	UKB-PPP	15319_2	CCNA1	deCODE	CPA2_P
GIPC3_(	GIPC3	UKB-PPP	15574_3	CCNA2	deCODE	CPA4_Q
GIPR_P	GIPR	UKB-PPP	5347_59	CCNB1	deCODE	CPB1_P

GIT1_Q	GIT1	UKB-PPP	9728_4_	CCNB1I	deCODE	CPB2_Q
GLI2_P1	GLI2	UKB-PPP	18208_3	CCNB2	deCODE	CPE_P1
GLOD4_	GLOD4	UKB-PPP	9848_22	CCNH	deCODE	CPLX2_
GLT8D2	GLT8D2	UKB-PPP	8889_5_	CCPG1	deCODE	CPM_P1
GLYR1_	GLYR1	UKB-PPP	13673_2	CCT7	deCODE	CPOX_I
GNE_Q9	GNE	UKB-PPP	12699_2	CCT8	deCODE	CPQ_Q9
GNGT1_	GNGT1	UKB-PPP	10814_7	CD1A	deCODE	CPVL_C
GNPDA	GNPDA	UKB-PPP	8749_19	CD1D	deCODE	CPXM1_
GOLGA	GOLGA	UKB-PPP	8980_19	CD200R	deCODE	CR1_P1
GOLM2	GOLM2	UKB-PPP	15567_2	CD247	deCODE	CR2_P2
GOPC_(	GOPC	UKB-PPP	10713_1	CD300L	deCODE	CRADD
GORAS	GORAS	UKB-PPP	16320_1	CD320	deCODE	CREG1_
GP1BB_	GP1BB	UKB-PPP	18202_2	CD37	deCODE	CRELD1
GP2_P5	GP2	UKB-PPP	9283_8_	CD44	deCODE	CRH_P0
GPA33_	GPA33	UKB-PPP	6653_58	CD47	deCODE	CRHBP_
GPIHBP	GPIHBP	UKB-PPP	18922_2	CD68	deCODE	CRIM1_
GPKOW	GPKOW	UKB-PPP	15674_3	CD79A	deCODE	CRIP2_I
GPR158	GPR158	UKB-PPP	7796_10	CD79A	deCODE	CRISP2_
GPR15L	GPR15L	UKB-PPP	19612_3	CD81	deCODE	CRISP3_
GPR37_	GPR37	UKB-PPP	9310_2_	CD8B	deCODE	CRKL_I
GPRC5C	GPRC5C	UKB-PPP	17449_2	CD9	deCODE	CRLF1_
GRIK2_	GRIK2	UKB-PPP	9735_44	CD96	deCODE	CRNN_(
GRIN2B	GRIN2B	UKB-PPP	18881_7	CD97	deCODE	CRTAC
GRK5_I	GRK5	UKB-PPP	18913_3	CDC2	deCODE	CRTAM
GRSF1_	GRSF1	UKB-PPP	12427_8	CDC25B	deCODE	CRYBB
GSAP_A	GSAP	UKB-PPP	12532_2	CDC34	deCODE	CRYBB
GTF2IR	GTF2IR	UKB-PPP	9840_2_	CDC42	deCODE	CRYGD
GTPBP2	GTPBP2	UKB-PPP	12706_2	CDC42E	deCODE	CRYZL1
GUCA2	GUCA2	UKB-PPP	16305_1	CDH11	deCODE	CSDE1_
GUCY2	GUCY2	UKB-PPP	7763_25	CDH11	deCODE	CSF1_P
GYS1_P	GYS1	UKB-PPP	19388_2	CDH12	deCODE	CSF1R_
H2AP_C	H2AP	UKB-PPP	7959_34	CDH7	deCODE	CSF2_P
HARS1_	HARS1	UKB-PPP	16021_3	CDH8	deCODE	CSF2RA
HCG22_	HCG22	UKB-PPP	8222_49	CDHR3	deCODE	CSF2RB
HCLS1_	HCLS1	UKB-PPP	9450_18	CDK2A1	deCODE	CSF3_P
HDAC9_	HDAC9	UKB-PPP	18227_3	CDK2A1	deCODE	CSF3R_
HDDC2_	HDDC2	UKB-PPP	3719_2_	CDKN1	deCODE	CSNK1I
HDGFL	HDGFL	UKB-PPP	9874_28	CDKN2	deCODE	CSNK2A

HEG1_(	HEG1	UKB-PPP	12521_3	CDKN2(	deCODE	CSPG4_
HEPH_(	HEPH	UKB-PPP	14178_1	CDKN3	deCODE	CSRP3_
HHEX_(	HHEX	UKB-PPP	7097_8_	CDY1	deCODE	CST1_P
HIP1_O	HIP1	UKB-PPP	10752_8	CDYL2	deCODE	CST3_P
HIP1R_(	HIP1R	UKB-PPP	10910_6	CEACA	deCODE	CST5_P
HJV_Q6	HJV	UKB-PPP	7184_13	CEACA	deCODE	CST6_Q
HLA-A_	HLA-A	UKB-PPP	19572_1	CEBPG	deCODE	CST7_O
HLA-DI	HLA-DI	UKB-PPP	6077_63	CECR1	deCODE	CTF1_Q
HLA-E_	HLA-E	UKB-PPP	9796_4_	CEL	deCODE	CTHRC
HMCN2	HMCN2	UKB-PPP	6107_3_	CELA1	deCODE	CTLA4_
HMGCL	HMGCL	UKB-PPP	6357_83	CELA3I	deCODE	CTRB1_
HMMR_	HMMR	UKB-PPP	7245_2_	CELF2	deCODE	CTRC_(
HNMT_	HNMT	UKB-PPP	8817_29	CENPV	deCODE	CTRL_F
HNRNP	HNRNP	UKB-PPP	8864_59	CENPW	deCODE	CTSB_P
HS1BP3	HS1BP3	UKB-PPP	6230_56	CER1	deCODE	CTSC_P
HSBP1_	HSBP1	UKB-PPP	13494_6	CERS5	deCODE	CTSD_F
HSD11E	HSD11E	UKB-PPP	6553_68	CFAP45	deCODE	CTSE_P
HSD17E	HSD17E	UKB-PPP	15468_1	CFHR1	deCODE	CTSF_Q
HSDL2_	HSDL2	UKB-PPP	16836_1	CFHR3	deCODE	CTSH_F
HSPA2_	HSPA2	UKB-PPP	4203_50	CFL1	deCODE	CTSL_P
HTR1A_	HTR1A	UKB-PPP	15339_3	CFL2	deCODE	CTSO_F
HTR1B_	HTR1B	UKB-PPP	16837_2	CFLAR	deCODE	CTSS_P
HYOU1_	HYOU1	UKB-PPP	7717_95	CFTR	deCODE	CTSV_(
ICA1_Q	ICA1	UKB-PPP	6213_10	CGB2	deCODE	CTSZ_(
ID4_P47	ID4	UKB-PPP	14005_2	CHD7	deCODE	CUZD1_
IFI30_P	IFI30	UKB-PPP	2853_68	CHEK1	deCODE	CX3CL1
IFIT1_P	IFIT1	UKB-PPP	11320_2	CHFR	deCODE	CXADR
IFT20_(	IFT20	UKB-PPP	9383_24	CHI3L2	deCODE	CXCL1_
IGF2BP	IGF2BP	UKB-PPP	16591_7	CHIA	deCODE	CXCL10
IGFL4_(	IGFL4	UKB-PPP	7761_12	CHKB	deCODE	CXCL11
IGHMB	IGHMB	UKB-PPP	17728_6	CHMP2	deCODE	CXCL12
IGLC2_	IGLC2	UKB-PPP	17350_1	CHMP2	deCODE	CXCL13
IGSF21_	IGSF21	UKB-PPP	13438_1	CHRD	deCODE	CXCL14
IGSF9_(	IGSF9	UKB-PPP	7803_4_	CHST1	deCODE	CXCL16
IKBKG_	IKBKG	UKB-PPP	7779_86	CHST11	deCODE	CXCL17
IKZF2_(	IKZF2	UKB-PPP	6626_81	CHST12	deCODE	CXCL5_
IL12A_I	IL12A	UKB-PPP	7262_19	CHST14	deCODE	CXCL6_
IL13RA	IL13RA	UKB-PPP	4469_78	CHST15	deCODE	CXCL8_

IL33_O9	IL33	UKB-PPP	18863_1	CHST2	deCODE	CXCL8_
ILKAP_	ILKAP	UKB-PPP	7189_55	CHST3	deCODE	CXCL8_
IMMT_(	IMMT	UKB-PPP	18876_7	CHST4	deCODE	CXCL8_
IMPACT	IMPACT	UKB-PPP	7020_13	CHST5	deCODE	CXCL9_
IMPG1_	IMPG1	UKB-PPP	4429_51	CHST6	deCODE	CYB5A_
INPP1_I	INPP1	UKB-PPP	11646_4	CHST9	deCODE	CYB5R2
INPP5D	INPP5D	UKB-PPP	8841_65	CILP2	deCODE	CYTL1_
INPP5J_	INPP5J	UKB-PPP	7745_3_	CISD1	deCODE	DAG1_(
INPPL1_	INPPL1	UKB-PPP	8094_20	CISD2	deCODE	DAPK2_
IPCEF1_	IPCEF1	UKB-PPP	5345_51	CKAP2	deCODE	DAPP1_
IQGAP2	IQGAP2	UKB-PPP	18197_9	CKMT2	deCODE	DBI_P0
IRAG2_	IRAG2	UKB-PPP	12530_1	CKS1B	deCODE	DBNL_(
IRAK1_	IRAK1	UKB-PPP	10496_1	CLCA1	deCODE	DCN_P(
ISM2_Q	ISM2	UKB-PPP	8950_4_	CLCA2	deCODE	DCTD_I
ITGA11	ITGA11	UKB-PPP	13683_1	CLCNK	deCODE	DCTN2_
ITGA6_	ITGA6	UKB-PPP	10953_1	CLEC2A	deCODE	DCTN6_
ITGAM_	ITGAM	UKB-PPP	7786_83	CLEC2B	deCODE	DCTPP1
ITGAV_	ITGAV	UKB-PPP	7054_87	CLEC2D	deCODE	DCUN1
ITGAX_	ITGAX	UKB-PPP	5013_2_	CLIC1	deCODE	DCUN1
ITGB1_	ITGB1	UKB-PPP	17837_5	CLIC2	deCODE	DCXR_(
ITGB1B	ITGB1B	UKB-PPP	15314_4	CLIC4	deCODE	DDAH1_
ITGB1B	ITGB1B	UKB-PPP	11327_5	CLK2	deCODE	DDC_P2
ITGBL1	ITGBL1	UKB-PPP	8874_53	CLN5	deCODE	DDR1_(
ITPR1_(	ITPR1	UKB-PPP	9526_3_	CLPSL1	deCODE	DDT_P3
ITPRIP_	ITPRIP	UKB-PPP	7767_1_	CLPSL2	deCODE	DDX25_
IZUMO1	IZUMO1	UKB-PPP	13711_1	CLTC	deCODE	DDX58_
JCHAIN	JCHAIN	UKB-PPP	3423_59	CMA1	deCODE	DECR1_
JMJD1C	JMJD1C	UKB-PPP	19115_1	CMBL	deCODE	DEFA1_
JPT2_Q9	JPT2	UKB-PPP	18243_9	CMPK1	deCODE	DEFB10
KAZN_(	KAZN	UKB-PPP	3192_3_	CNDP2	deCODE	DEFB10
KCNC4_	KCNC4	UKB-PPP	13532_2	CNEP1R	deCODE	DEFB11
KCNH2_	KCNH2	UKB-PPP	12872_3	CNGA2	deCODE	DEFB11
KDM3A	KDM3A	UKB-PPP	15566_1	CNN1	deCODE	DEFB4A
KHDC3	KHDC3	UKB-PPP	18877_1	CNN2	deCODE	DENR_(
KHK_P5	KHK	UKB-PPP	13482_1	CNOT1	deCODE	DGCR6_
KIAA03	KIAA03	UKB-PPP	17327_3	CNPY3	deCODE	DHPS_F
KIAA15	KIAA15	UKB-PPP	18218_4	CNRIP1	deCODE	DIABLO
KIF20B_	KIF20B	UKB-PPP	14101_2	CNTFR	deCODE	DKK1_(

KIFBP_(	KIFBP	UKB-PPP	9044_1_	CNTNA	deCODE	DKK3_(
KIRREL	KIRREL	UKB-PPP	7888_58	COA3	deCODE	DKK4_(
KLHL41	KLHL41	UKB-PPP	9543_13	COG8	deCODE	DKKL1_
KLK1_F	KLK1	UKB-PPP	15653_9	COL10A	deCODE	DLG4_F
KLRD1_	KLRD1	UKB-PPP	11278_4	COL11A	deCODE	DLK1_F
KRT14_	KRT14	UKB-PPP	6570_1_	COL13A	deCODE	DLL1_C
KRT6C_	KRT6C	UKB-PPP	8804_39	COL20A	deCODE	DLL4_C
KRT8_F	KRT8	UKB-PPP	4543_65	COL23A	deCODE	DNAJA
KYAT1_	KYAT1	UKB-PPP	7006_4_	COL25A	deCODE	DNAJA
L3HYPI	L3HYPI	UKB-PPP	13535_2	COL4A	deCODE	DNAJB
LACTB	LACTB	UKB-PPP	13950_9	COL4A	deCODE	DNAJB
LAMA1	LAMA1	UKB-PPP	16828_8	COL6A	deCODE	DNAJB
LAMB1	LAMB1	UKB-PPP	16753_4	COL6A	deCODE	DNAJB
LAMP2_	LAMP2	UKB-PPP	11155_1	COL6A	deCODE	DNER_(
LAMP3_	LAMP3	UKB-PPP	4807_13	COL8A	deCODE	DOC2B_
LAMTO	LAMTO	UKB-PPP	6558_5_	COLEC	deCODE	DOCK9_
LARP1_	LARP1	UKB-PPP	4430_44	COLEC	deCODE	DOK1_(
LATS1_	LATS1	UKB-PPP	5638_23	COLGA	deCODE	DOK2_(
LBR_Q1	LBR	UKB-PPP	7234_12	COLGA	deCODE	DPEP1_
LCN15_	LCN15	UKB-PPP	2823_7_	COMMI	deCODE	DPEP2_
LELP1_	LELP1	UKB-PPP	14029_4	COPS2	deCODE	DPP10_
LEO1_C	LEO1	UKB-PPP	12384_9	COPS7E	deCODE	DPP4_P
LETM1_	LETM1	UKB-PPP	4905_63	COTL1	deCODE	DPP7_Q
LHPP_C	LHPP	UKB-PPP	7850_1_	COX4I2	deCODE	DPT_Q0
LIPF_P(	LIPF	UKB-PPP	18226_1	COX5A	deCODE	DPY30_
LMNB2	LMNB2	UKB-PPP	10535_2	COX6A	deCODE	DRAXIN
LMOD2	LMOD2	UKB-PPP	8903_1_	COX6C	deCODE	DSC2_C
LPA_P0	LPA	UKB-PPP	8390_25	COX7A	deCODE	DSCAM
LPL_P0	LPL	UKB-PPP	9590_10	COX7A	deCODE	DSG2_C
LPP_Q9	LPP	UKB-PPP	17453_3	CP	deCODE	DSG3_F
LRG1_F	LRG1	UKB-PPP	7917_17	CPD	deCODE	DTD1_(
LRP2BF	LRP2BF	UKB-PPP	18332_1	CPLX1	deCODE	DTYMK
LRRC38	LRRC38	UKB-PPP	7142_5_	CPN1	deCODE	DUSP13
LRRFIP	LRRFIP	UKB-PPP	6415_90	CPN2	deCODE	DUSP3_
LSM8_(	LSM8	UKB-PPP	5346_24	CPNE1	deCODE	DUT_P3
LSP1_P	LSP1	UKB-PPP	11709_2	CPT1B	deCODE	DYNLT
LTB_Q0	LTB	UKB-PPP	6493_9_	CPZ	deCODE	DYNLT
LTBP2_	LTBP2	UKB-PPP	11967_2	CRABP	deCODE	EBAG9_

LTBP3_	LTBP3	UKB-PPP	11696_7	CRABP	deCODE	EBI3_IL
LTO1_	LTO1	UKB-PPP	12637_7	CRAT	deCODE	ECE1_P
LUZP2_	LUZP2	UKB-PPP	17771_3	CRCP	deCODE	ECHS1_
LXN_Q	LXN	UKB-PPP	11198_3	CREB3	deCODE	ECI2_O
LY6D_	LY6D	UKB-PPP	17336_5	CREB3	deCODE	ECM1_
LY96_Q	LY96	UKB-PPP	11308_8	CREB3	deCODE	EDA2R_
LYAR_	LYAR	UKB-PPP	13614_6	CREBB	deCODE	EDAR_
LYPD8_	LYPD8	UKB-PPP	18275_5	CRIP1	deCODE	EDDM3
LYPLA	LYPLA	UKB-PPP	19294_2	CRIPT	deCODE	EDF1_C
LZTFL1	LZTFL1	UKB-PPP	5691_2_	CRISPL	deCODE	EDIL3_
M6PR_	M6PR	UKB-PPP	4976_57	CRK	deCODE	EDN1_
MAEA_	MAEA	UKB-PPP	13694_2	CRLF2	deCODE	EEF1D_
MAG_P	MAG	UKB-PPP	18323_3	CRMP1	deCODE	EFCAB
MAGE	MAGE	UKB-PPP	13929_2	CROT	deCODE	EFEMP
MAMD	MAMD	UKB-PPP	4337_49	CRP	deCODE	EFHD1_
MANEA	MANEA	UKB-PPP	10087_1	CRYAA	deCODE	EFNA4_
MAP1L	MAP1L	UKB-PPP	19311_1	CRYGS	deCODE	EFNB2_
MAP2_	MAP2	UKB-PPP	13983_2	CRYZ	deCODE	EGF_P0
MAP3K	MAP3K	UKB-PPP	18309_1	CS	deCODE	EGFLA
MAP4K	MAP4K	UKB-PPP	5716_49	CSAG1	deCODE	EGFR_
MARS1	MARS1	UKB-PPP	12754_1	CSDC2	deCODE	EGLN1_
MAVS_	MAVS	UKB-PPP	10772_2	CSGAL	deCODE	EHBP1_
MCAM_	MCAM	UKB-PPP	3363_31	CSK	deCODE	EIF1AX
MCFD2	MCFD2	UKB-PPP	9598_23	CSMD1	deCODE	EIF4B_
MECR_	MECR	UKB-PPP	9971_5_	CSMD2	deCODE	EIF4E_
MED18_	MED18	UKB-PPP	16587_1	CSN2	deCODE	EIF4EB
MED21_	MED21	UKB-PPP	17436_1	CSNK1	deCODE	EIF4G3_
MEGF1	MEGF1	UKB-PPP	12653_1	CSNK1	deCODE	EIF5_P5
MEGF9_	MEGF9	UKB-PPP	13681_1	CSNK2	deCODE	EIF5A_
MELTF_	MELTF	UKB-PPP	11330_1	CSNK2	deCODE	ENAH_
MEP1A_	MEP1A	UKB-PPP	15529_3	CSRP1	deCODE	ENDOU
MEP1B_	MEP1B	UKB-PPP	12968_2	CSRP2	deCODE	ENG_P1
MERTK	MERTK	UKB-PPP	4324_33	CST2	deCODE	ENO1_
MESD_	MESD	UKB-PPP	14076_7	CST4	deCODE	ENO2_
METAP	METAP	UKB-PPP	10572_6	CST8	deCODE	ENO3_
MFAP3_	MFAP3	UKB-PPP	6368_9_	CSTL1	deCODE	ENOX2_
MGLL_	MGLL	UKB-PPP	9363_11	CT55	deCODE	ENPEP_
MGMT_	MGMT	UKB-PPP	11217_1	CTBP1	deCODE	ENPP2_

MINDY	MINDY	UKB-PPP
MINK1_	MINK1	UKB-PPP
MITD1_	MITD1	UKB-PPP
MKI67_	MKI67	UKB-PPP
MLLT1_	MLLT1	UKB-PPP
MME_P	MME	UKB-PPP
MMP15_	MMP15	UKB-PPP
MMUT_	MMUT	UKB-PPP
MN1_Q	MN1	UKB-PPP
MNAT1	MNAT1	UKB-PPP
MNDA_	MNDA	UKB-PPP
MOCS2_	MOCS2	UKB-PPP
MORC3	MORC3	UKB-PPP
MORF4	MORF4	UKB-PPP
MORN4	MORN4	UKB-PPP
MPHOS	MPHOS	UKB-PPP
MPI_P3	MPI	UKB-PPP
MPIG6B	MPIG6B	UKB-PPP
MPRIP_	MPRIP	UKB-PPP
MRI1_Q	MRI1	UKB-PPP
MRPL24	MRPL24	UKB-PPP
MRPL28	MRPL28	UKB-PPP
MRPL46	MRPL46	UKB-PPP
MRPL58	MRPL58	UKB-PPP
MRPS16	MRPS16	UKB-PPP
MSLNL_	MSLNL	UKB-PPP
MTDH_	MTDH	UKB-PPP
MTPN_	MTPN	UKB-PPP
MTR_Q	MTR	UKB-PPP
MTSS1_	MTSS1	UKB-PPP
MTSS2_	MTSS2	UKB-PPP
MTUS1_	MTUS1	UKB-PPP
MUC13_	MUC13	UKB-PPP
MUC2_	MUC2	UKB-PPP
MUCL3_	MUCL3	UKB-PPP
MYBPC	MYBPC	UKB-PPP
MYCBP	MYCBP	UKB-PPP
MYH4_	MYH4	UKB-PPP

14624_5	CTCF	deCODE
2975_19	CTGF	deCODE
9872_23	CTNNA	deCODE
8424_26	CTNNB	deCODE
9842_2_	CTNNB	deCODE
5648_28	CTRB2	deCODE
3179_51	CTSA	deCODE
2431_17	CTSG	deCODE
9443_13	CTSK	deCODE
12904_1	CUBN	deCODE
17797_1	CUL1	deCODE
10045_4	CUL3	deCODE
13743_5	CUL4B	deCODE
12991_4	CUL9	deCODE
15315_6	CUTC	deCODE
13377_3	CYB561	deCODE
10924_2	CYB5D2	deCODE
18255_6	CYB5R1	deCODE
7215_18	CYB5R3	deCODE
2942_50	CYCS	deCODE
11546_7	CYGB	deCODE
2943_5_	CYP3A4	deCODE
7879_12	CYP3A4	deCODE
6264_9_	CYR61	deCODE
12533_1	CYTH2	deCODE
12746_4	CYTH4	deCODE
13612_7	CYTIP	deCODE
19120_3	DAO	deCODE
19141_2	DAP	deCODE
13955_3	DAPK1	deCODE
12675_1	DARS	deCODE
12395_8	DARS2	deCODE
11283_1	DCAF5	deCODE
9402_18	DCBLD	deCODE
19367_3	DCI	deCODE
9836_20	DCK	deCODE
17156_7	DCLK1	deCODE
7826_1_	DCLK3	deCODE

ENPP5_
ENPP6_
ENPP7_
ENSA_
ENTPD2
ENTPD5
ENTPD6
EPHA1_
EPHA10
EPHA2_
EPHA4_
EPHB4_
EPHB6_
EPO_P0
ERBB2_
ERBB3_
ERBB4_
ERCC1_
EREG_
ERMAP
ERN1_
ERP29_
ERP44_
ESAM_
ESM1_
EVPL_
EZR_P1
F10_P00
F11_P03
F13B_P
F2_P007
F3_P137
F7_P087
F9_P007
FABP1_
FABP2_
FABP3_
FABP4_

MYH7B	MYH7B	UKB-PPP
MYH9_:	MYH9	UKB-PPP
MYL1_I	MYL1	UKB-PPP
MYL4_I	MYL4	UKB-PPP
MYLPF_	MYLPF	UKB-PPP
MYO9B	MYO9B	UKB-PPP
MYOM:	MYOM:	UKB-PPP
MZB1_(	MZB1	UKB-PPP
MZT1_(	MZT1	UKB-PPP
NAA80_	NAA80	UKB-PPP
NACC1_	NACC1	UKB-PPP
NAGA_:	NAGA	UKB-PPP
NAPRT_	NAPRT	UKB-PPP
NARS1_	NARS1	UKB-PPP
NBN_O(	NBN	UKB-PPP
NCLN_(	NCLN	UKB-PPP
NCS1_P	NCS1	UKB-PPP
NDUFB	NDUFB	UKB-PPP
NDUFS(	NDUFS(	UKB-PPP
NEB_P2	NEB	UKB-PPP
NECAP:	NECAP:	UKB-PPP
NECTIN	NECTIN	UKB-PPP
NECTIN	NECTIN	UKB-PPP
NECTIN	NECTIN	UKB-PPP
NEDD4I	NEDD4I	UKB-PPP
NEXN_(	NEXN	UKB-PPP
NFAT5_	NFAT5	UKB-PPP
NFATC:	NFATC:	UKB-PPP
NFE2_Q	NFE2	UKB-PPP
NFIC_P(	NFIC	UKB-PPP
NFKB2_	NFKB2	UKB-PPP
NFKBIE	NFKBIE	UKB-PPP
NFX1_C	NFX1	UKB-PPP
NFYA_I	NFYA	UKB-PPP
NINJ1_(	NINJ1	UKB-PPP
NIT1_Q	NIT1	UKB-PPP
NME3_(	NME3	UKB-PPP
NMRK2	NMRK2	UKB-PPP

14008_2	DCP1A	deCODE
12715_3	DCP1B	deCODE
13553_4	DCUN1:	deCODE
8760_10	DCUN1:	deCODE
19119_1	DDIT3	deCODE
17817_2	DDIT4	deCODE
17161_1	DDOST	deCODE
15381_4	DDR2	deCODE
18813_1	DDX19/	deCODE
5460_60	DDX19I	deCODE
13946_8	DDX23	deCODE
9742_59	DDX39I	deCODE
14216_3	DDX46	deCODE
12869_6	DDX6	deCODE
6369_82	DEAF1	deCODE
18343_1	DECR2	deCODE
14090_2	DEF6	deCODE
16785_4	DEFA5	deCODE
6629_3_	DEFB1	deCODE
5664_57	DEFB10	deCODE
6399_52	DEFB10	deCODE
5611_56	DEFB10	deCODE
8340_9_	DEFB11	deCODE
5689_1_	DEFB11	deCODE
13374_4	DEFB11	deCODE
8391_12	DEFB11	deCODE
8315_5_	DEFB11	deCODE
5765_53	DEFB12	deCODE
9486_13	DEFB12	deCODE
6360_7_	DEFB12	deCODE
8347_22	DEFB12	deCODE
9022_49	DEFB13	deCODE
6411_58	DEFB13	deCODE
9332_6_	DEFB13	deCODE
7178_59	DEPP	deCODE
13393_4	DERL1	deCODE
12030_8	DES	deCODE
11356_1	DGCR14	deCODE

FABP5_
FABP6_
FABP9_
FADD_(
FAM171
FAM171
FAM172
FAM20/
FAM3B_
FAM3D_
FAP_Q1
FAS_P2
FASLG_
FBP1_P(
FCAMR
FCAR_I
FCER1/
FCER2_
FCGR2/
FCGR2I
FCGR3I
FCN1_C
FCN2_Q
FCRL1_
FCRL3_
FCRL5_
FCRL6_
FCRLB_
FEN1_P
FETUB_
FGF12_
FGF16_(
FGF19_(
FGF2_P
FGF20_(
FGF23_(
FGF3_P
FGF5_P

NOMO1	NOMO1	UKB-PPP	8055_33	DGCR2	deCODE	FGF6_P
NOP56_	NOP56	UKB-PPP	12895_2	DGKB	deCODE	FGF7_P
NOS1_P	NOS1	UKB-PPP	9823_2_	DHFR	deCODE	FGF9_P
NOS2_P	NOS2	UKB-PPP	4389_2_	DHH	deCODE	FGFBP1
NOS3_P	NOS3	UKB-PPP	17467_1	DHRS9	deCODE	FGFBP3
NPC2_P	NPC2	UKB-PPP	13645_1	DHX38	deCODE	FGFR2_
NPHS1_	NPHS1	UKB-PPP	14012_1	DHX58	deCODE	FGFR4_
NPHS2_	NPHS2	UKB-PPP	11601_2	DHX8	deCODE	FGL1_Q
NPPC_P	NPPC	UKB-PPP	10527_2	DHX9	deCODE	FGR_P0
NPR1_P	NPR1	UKB-PPP	14329_4	DIDO1	deCODE	FH_P07
NPY_P0	NPY	UKB-PPP	12694_2	DIMT1	deCODE	FHIT_P
NRN1_(	NRN1	UKB-PPP	17793_4	DIRAS1	deCODE	FIS1_Q
NRTN_(	NRTN	UKB-PPP	12406_1	DIRAS3	deCODE	FKBP14
NT5C1A	NT5C1A	UKB-PPP	13441_3	DIXDC1	deCODE	FKBP1B
NTproB	NTproB	UKB-PPP	15678_7	DKK2	deCODE	FKBP4_
NUB1_(	NUB1	UKB-PPP	15527_9	DLD	deCODE	FKBP7_
NUBP1_	NUBP1	UKB-PPP	19620_1	DLG2	deCODE	FLRT2_
NUCB2_	NUCB2	UKB-PPP	7897_75	DLG3	deCODE	FLT1_P
NUDT1(	NUDT1(	UKB-PPP	9359_9_	DLK2	deCODE	FLT3_P
NUDT1	NUDT1	UKB-PPP	9974_8_	DLL3	deCODE	FLT3LG
NUDT1(	NUDT1(	UKB-PPP	11422_2	DLX3	deCODE	FLT4_P
NUMB_	NUMB	UKB-PPP	11910_2	DLX4	deCODE	FMR1_(
NUP50_	NUP50	UKB-PPP	19212_4	DMBT1	deCODE	FN1_P0
NXPE4_	NXPE4	UKB-PPP	5963_9_	DMKN	deCODE	FOLH1_
OCLN_(	OCLN	UKB-PPP	8535_10	DMKN	deCODE	FOLR1_
ODAM_	ODAM	UKB-PPP	11396_3	DNAI1	deCODE	FOLR2_
OFD1_(	OFD1	UKB-PPP	7110_2_	DNAJB	deCODE	FOLR3_
OGA_O	OGA	UKB-PPP	8006_12	DNAJB	deCODE	FOXO3_
OGFR_(	OGFR	UKB-PPP	18884_2	DNAJB4	deCODE	FRZB_(
OGT_O	OGT	UKB-PPP	11214_4	DNAJB9	deCODE	FSHB_P
OPLAH_	OPLAH	UKB-PPP	8297_8_	DNAJC	deCODE	FST_P1
ORM1_	ORM1	UKB-PPP	9783_75	DNAJC	deCODE	FSTL1_
OSBPL2	OSBPL2	UKB-PPP	7197_2_	DNAJC	deCODE	FSTL3_
OTOA_(	OTOA	UKB-PPP	10454_9	DNAJC	deCODE	FTCD_(
OTUD6	OTUD6	UKB-PPP	14655_1	DNAJC	deCODE	FURIN_
OTUD7	OTUD7	UKB-PPP	8033_1_	DNAJC	deCODE	FUT3_F
OXCT1_	OXCT1	UKB-PPP	4545_53	DNAJC	deCODE	FUT8_Q
PACS2_	PACS2	UKB-PPP	12799_6	DNAJC2	deCODE	FXN_Q

PADI2_(	PADI2	UKB-PPP	7866_11	DNAJC3	deCODE	FZD10_
PADI4_(	PADI4	UKB-PPP	8016_19	DNAJC4	deCODE	GABAR
PAG1_C	PAG1	UKB-PPP	8653_13	DNAJC4	deCODE	GABAR
PAGR1_	PAGR1	UKB-PPP	6324_11	DNASE	deCODE	GAD1_(
PAIP2B_	PAIP2B	UKB-PPP	6533_20	DNASE1	deCODE	GADD4
PALLD_	PALLD	UKB-PPP	11572_4	DNM2	deCODE	GAGE2
PALM2_	PALM2	UKB-PPP	19142_3	DNMT3	deCODE	GAL_P2
PALM3_	PALM3	UKB-PPP	9123_18	DOS	deCODE	GALNT
PAMR1_	PAMR1	UKB-PPP	12692_5	DOT1L	deCODE	GALNT
PARD3_	PARD3	UKB-PPP	13567_1	DPYSL2	deCODE	GALNT
PAXX_(	PAXX	UKB-PPP	12707_2	DPYSL3	deCODE	GAPDH
PBLD_F	PBLD	UKB-PPP	12683_1	DPYSL5	deCODE	GAS6_C
PBXIP1_	PBXIP1	UKB-PPP	17849_6	DRG1	deCODE	GBP1_P
PCARE_	PCARE	UKB-PPP	8034_6_	DRGX	deCODE	GBP2_P
PCBP2_	PCBP2	UKB-PPP	10882_1	DSC1	deCODE	GBP6_C
PCDH1_	PCDH1	UKB-PPP	4981_6_	DSC3	deCODE	GC_P02
PCDH12	PCDH12	UKB-PPP	18886_2	DSCAM	deCODE	GCG_P(
PCDH17	PCDH17	UKB-PPP	2976_58	DSG1	deCODE	GCLM_
PCDH7_	PCDH7	UKB-PPP	18883_4	DSTN	deCODE	GCNT1_
PCDHB	PCDHB	UKB-PPP	18280_2	DTD2	deCODE	GDF15_
PCYT2_	PCYT2	UKB-PPP	11430_4	DTX1	deCODE	GDF2_C
PDE1C_	PDE1C	UKB-PPP	11643_7	DTX3L	deCODE	GDNF_I
PDIA2_(	PDIA2	UKB-PPP	13506_1	DUS2	deCODE	GFAP_F
PDLIM5	PDLIM5	UKB-PPP	12838_2	DUSP15	deCODE	GFER_F
PDLIM7	PDLIM7	UKB-PPP	14631_2	DUSP16	deCODE	GFRA1_
PDP1_Q	PDP1	UKB-PPP	17326_4	DUSP23	deCODE	GFRA2_
PDXDC	PDXDC	UKB-PPP	8967_6_	DUSP26	deCODE	GFRA3_
PDZD2_	PDZD2	UKB-PPP	9940_35	DUSP28	deCODE	GFRAL_
PEPD_P	PEPD	UKB-PPP	10035_6	DUSP4	deCODE	GGA1_(
PER3_P	PER3	UKB-PPP	12341_8	DUSP6	deCODE	GGH_Q
PFDN4_	PFDN4	UKB-PPP	13575_4	DVL2	deCODE	GH1_P0
PFDN6_	PFDN6	UKB-PPP	3881_49	DYNLL	deCODE	GH2_P0
PFKFB2	PFKFB2	UKB-PPP	11493_1	DYNLL	deCODE	GHR_P1
PGA4_F	PGA4	UKB-PPP	3845_51	DYNLR	deCODE	GHRL_(
PGLYRI	PGLYRI	UKB-PPP	17827_5	DYNLR	deCODE	GIP_P0
PHACTI	PHACTI	UKB-PPP	4359_87	DYRK3	deCODE	GJA8_P
PHLDB	PHLDB	UKB-PPP	7076_17	ECEL1	deCODE	GKN1_(
PHLDB2	PHLDB2	UKB-PPP	12662_8	ECH1	deCODE	GLA_P(

PHOSPH	PHOSPH	UKB-PPP	14024_1	EDA	deCODE	GLB1_P
PHYKPL	PHYKPL	UKB-PPP	2826_53	EDA	deCODE	GLO1_
PI16_Q6	PI16	UKB-PPP	13066_4	EDC4	deCODE	GLP1R_
PIBF1_	PIBF1	UKB-PPP	9536_16	EDDM3	deCODE	GLRX_
PIK3AP	PIK3AP	UKB-PPP	12574_3	EDN2	deCODE	GLRX5_
PIK3IP1	PIK3IP1	UKB-PPP	15383_2	EDN3	deCODE	GM2A_
PIKFYV	PIKFYV	UKB-PPP	14043_1	EEA1	deCODE	GMFG_
PILRB_	PILRB	UKB-PPP	5882_34	EEF1B2	deCODE	GMPR_
PINLYP	PINLYP	UKB-PPP	12332_7	EEF2K	deCODE	GMPR2_
PITHD1	PITHD1	UKB-PPP	6226_69	EFHA1	deCODE	GNAS_
PKD1_P	PKD1	UKB-PPP	6912_6_	EFHA2	deCODE	GNLY_
PKN3_	PKN3	UKB-PPP	14124_6	EFNA2	deCODE	GNPDA
PLA2G1	PLA2G1	UKB-PPP	14153_8	EFNA3	deCODE	GOT1_P
PLA2G4	PLA2G4	UKB-PPP	2615_60	EFNA5	deCODE	GP1BA_
PLB1_Q	PLB1	UKB-PPP	13104_3	EFNB1	deCODE	GP5_P4
PLCB2_	PLCB2	UKB-PPP	2514_65	EFNB3	deCODE	GP6_Q9
PLEKH	PLEKH	UKB-PPP	7785_1_	EFNB3	deCODE	GPC1_P
PLIN1_	PLIN1	UKB-PPP	12572_2	EFS	deCODE	GPC5_P
PLPBP_	PLPBP	UKB-PPP	17454_1	EGFL6	deCODE	GPD1_P
PM20D1	PM20D1	UKB-PPP	11421_1	EHD4	deCODE	GPHA2_
PMCH_	PMCH	UKB-PPP	13387_5	EHF	deCODE	GPI_P06
PMS1_P	PMS1	UKB-PPP	5843_60	EHMT2	deCODE	GPNMB
PNMA1	PNMA1	UKB-PPP	8079_39	EID3	deCODE	GPR101
PNMA2	PNMA2	UKB-PPP	13545_9	EIF1AD	deCODE	GRAP2_
PNPT1_	PNPT1	UKB-PPP	19224_5	EIF1AY	deCODE	GRHPR
PODXL	PODXL	UKB-PPP	12701_1	EIF1B	deCODE	GRN_P2
POF1B_	POF1B	UKB-PPP	14284_2	EIF2AK	deCODE	GRP_P0
POLR2A	POLR2A	UKB-PPP	10080_9	EIF2B1	deCODE	GRPEL1
POLR2F	POLR2F	UKB-PPP	19259_1	EIF2S1	deCODE	GSN_P0
PON2_	PON2	UKB-PPP	11454_8	EIF3G	deCODE	GSR_P0
PON3_	PON3	UKB-PPP	13497_3	EIF3J	deCODE	GSTA1_
PPL_O6	PPL	UKB-PPP	18829_4	EIF4A1	deCODE	GSTA3_
PPM1B_	PPM1B	UKB-PPP	18824_7	EIF4A2	deCODE	GSTM4_
PPM1F_	PPM1F	UKB-PPP	4997_19	EIF4A3	deCODE	GSTP1_
PPP1R1	PPP1R1	UKB-PPP	9745_20	EIF4E2	deCODE	GSTT2B
PPP1R1	PPP1R1	UKB-PPP	4184_43	EIF4EB	deCODE	GUK1_
PPP1R1	PPP1R1	UKB-PPP	18859_7	EIF4EB	deCODE	GUSB_
PPP1R9	PPP1R9	UKB-PPP	4230_1_	EIF4G2	deCODE	GZMA_

PRAME	PRAME	UKB-PPP
PRAP1_	PRAP1	UKB-PPP
PRDX2_	PRDX2	UKB-PPP
PREB_C	PREB	UKB-PPP
PRELP_	PRELP	UKB-PPP
PRKAB	PRKAB	UKB-PPP
PRKAG	PRKAG	UKB-PPP
PRKAR	PRKAR	UKB-PPP
PRKD2_	PRKD2	UKB-PPP
PRKRA_	PRKRA	UKB-PPP
PROCR_	PROCR	UKB-PPP
PRR4_C	PRR4	UKB-PPP
PRR5_P	PRR5	UKB-PPP
PRRT3_	PRRT3	UKB-PPP
PRSS53_	PRSS53	UKB-PPP
PRTFD(	PRTFD(	UKB-PPP
PRTG_(	PRTG	UKB-PPP
PRUNE:	PRUNE:	UKB-PPP
PSCA_C	PSCA	UKB-PPP
PSMC3_	PSMC3	UKB-PPP
PSMD1_	PSMD1	UKB-PPP
PSMG4_	PSMG4	UKB-PPP
PSRC1_	PSRC1	UKB-PPP
PSTPIP:	PSTPIP:	UKB-PPP
PTPRB_	PTPRB	UKB-PPP
PTPRC_	PTPRC	UKB-PPP
PTPRF_	PTPRF	UKB-PPP
PTPRK_	PTPRK	UKB-PPP
PTPRM_	PTPRM	UKB-PPP
PTPRN2	PTPRN2	UKB-PPP
PTPRZ1	PTPRZ1	UKB-PPP
PTRHD:	PTRHD:	UKB-PPP
PVALB_	PVALB	UKB-PPP
PXN_P4	PXN	UKB-PPP
QSOX1_	QSOX1	UKB-PPP
RAB10_	RAB10	UKB-PPP
RAB11F	RAB11F	UKB-PPP
RAB33A	RAB33A	UKB-PPP

5885_55	EIF4H	deCODE
11355_1	EIF5A2	deCODE
13671_4	ELANE	deCODE
11592_1	ELAVL:	deCODE
13457_3	ELF5	deCODE
10006_2	ELK1	deCODE
5707_55	ELK3	deCODE
11459_8	ELL	deCODE
11494_4	ELL2	deCODE
12764_3	ELMO1	deCODE
11989_3	EMC1	deCODE
13516_4	EMC4	deCODE
17770_4	EMC8	deCODE
9547_29	EMID1	deCODE
8773_17	EMILIN	deCODE
16781_2	ENGAS:	deCODE
12933_1	ENOX1	deCODE
7947_19	ENTHD.	deCODE
3182_38	ENTPD:	deCODE
7999_23	ENTPD:	deCODE
4436_1_	ENTPD:	deCODE
4706_17	EPB41	deCODE
13993_2	EPB41L	deCODE
9040_14	EPDR1	deCODE
3432_21	EPHA3	deCODE
3806_55	EPHA5	deCODE
15580_2	EPHA7	deCODE
17680_1	EPHB1	deCODE
8225_86	EPHB2	deCODE
9220_7_	EPHB3	deCODE
19575_4	EPOR	deCODE
4212_5_	EPS15L	deCODE
9278_9_	EPYC	deCODE
11162_3	EQTN	deCODE
4964_67	ERAP1	deCODE
8960_3_	ERAP2	deCODE
9895_77	ERCC4	deCODE
11614_2	ERH	deCODE

GZMB_
GZMH_
HADH_
HAGH_
HAO1_(
HAVCR
HAVCR
HBEGF_
HBQ1_F
HBZ_P(
HDAC8_
HDGF_I
HEBP1_
HEPAC.
HEXIM:
HGF_P1
HGFAC_
HGS_O:
HIF1A_(
HK2_P5
HMBS_
HMGCS
HMOX1
HMOX2
HNF1A_
HNRNP:
HPCAL:
HPGDS_
HPSE_C
HRAS_F
HRC_P2
HRG_P(
HS3ST3
HS6ST1
HS6ST2
HSD17E
HSP90B
HSPA1A

RAB37_	RAB37	UKB-PPP	8957_72	ERLEC1	deCODE	HSPB1_
RAB3G.	RAB3G.	UKB-PPP	8776_10	ERLIN1	deCODE	HSPB6_
RAB44_	RAB44	UKB-PPP	7060_2_	ERO1L	deCODE	HSPG2_
RAB6A_	RAB6A	UKB-PPP	7994_41	ERO1LI	deCODE	HTRA2_
RABEP1	RABEP1	UKB-PPP	9333_59	ERP27	deCODE	HYAL1_
RABGA	RABGA	UKB-PPP	12531_5	ERVV	deCODE	ICAM1_
RALY_(	RALY	UKB-PPP	4984_83	ESD	deCODE	ICAM2_
RANBP2	RANBP2	UKB-PPP	8828_21	ESRP1	deCODE	ICAM3_
RANGA	RANGA	UKB-PPP	9873_17	ESRRA	deCODE	ICAM4_
RAP1A_	RAP1A	UKB-PPP	17435_4	ETFA	deCODE	ICAM5_
RAPGEI	RAPGEI	UKB-PPP	3847_56	ETHE1	deCODE	ICOSLG
RASGR	RASGR	UKB-PPP	8328_9_	ETNK1	deCODE	IDI2_Q9
RASSF2	RASSF2	UKB-PPP	12350_8	ETS2	deCODE	IDO1_P
RBFOX	RBFOX	UKB-PPP	13403_5	ETV5	deCODE	IDO1_P
RBM25_	RBM25	UKB-PPP	8062_15	EVA1B	deCODE	IDO1_P
RBP2_P	RBP2	UKB-PPP	8877_22	EVA1C	deCODE	IDO1_P
RBPMS_	RBPMS	UKB-PPP	11656_1	EVL	deCODE	IDS_P22
RBPMS2	RBPMS2	UKB-PPP	12988_4	EWSR1	deCODE	IDUA_P
RCC1_P	RCC1	UKB-PPP	7930_3_	EXOSC1	deCODE	IFIT3_O
RCOR1_	RCOR1	UKB-PPP	12605_1	EXOSC3	deCODE	IFNAR1
RECK_(	RECK	UKB-PPP	18909_1	EXOSC8	deCODE	IFNG_P
REEP4_	REEP4	UKB-PPP	6528_95	EXTL2	deCODE	IFNGR1
REPS1_	REPS1	UKB-PPP	9365_91	EYS	deCODE	IFNGR2
REST_C	REST	UKB-PPP	4906_35	F5	deCODE	IFNL1_(
RFC4_P	RFC4	UKB-PPP	13499_3	F8	deCODE	IFNL2_(
RGCC_(	RGCC	UKB-PPP	8396_42	FAAH2	deCODE	IFNLR1
RGL2_C	RGL2	UKB-PPP	18888_3	FABP12	deCODE	IFNW1_
RICTOF	RICTOF	UKB-PPP	9738_7_	FAF2	deCODE	IGBP1_
RIDA_P	RIDA	UKB-PPP	11424_4	FAH	deCODE	IGDCC3
RILP_Q	RILP	UKB-PPP	17819_3	FAHD1	deCODE	IGDCC4
RILPL2_	RILPL2	UKB-PPP	16594_4	FAIM	deCODE	IGF1R_
RIPK4_	RIPK4	UKB-PPP	6574_11	FAIM3	deCODE	IGF2R_
RLN1_P	RLN1	UKB-PPP	2760_2_	FAM107	deCODE	IGFBP1_
RNASEI	RNASEI	UKB-PPP	8556_5_	FAM134	deCODE	IGFBP2_
RNF168	RNF168	UKB-PPP	6284_7_	FAM150	deCODE	IGFBP3_
RNF31_	RNF31	UKB-PPP	7856_51	FAM151	deCODE	IGFBP4_
RNF4_P	RNF4	UKB-PPP	13431_7	FAM159	deCODE	IGFBP6_
RNF5_C	RNF5	UKB-PPP	6531_29	FAM162	deCODE	IGFBP7_

RP2_O7	RP2	UKB-PPP	11163_7	FAM162	deCODE	IGFBPL
RPGR_(	RPGR	UKB-PPP	10880_3	FAM163	deCODE	IGLON5
RPL14_	RPL14	UKB-PPP	5610_32	FAM171	deCODE	IGSF3_(
RRP15_	RRP15	UKB-PPP	7148_42	FAM173	deCODE	IGSF8_(
RTBDN_	RTBDN	UKB-PPP	6597_24	FAM174	deCODE	IL10_P2
RTKN2_	RTKN2	UKB-PPP	8784_7_	FAM175	deCODE	IL10RA_
RWDD1	RWDD1	UKB-PPP	8945_7_	FAM175	deCODE	IL10RB_
RYR1_F	RYR1	UKB-PPP	8039_41	FAM177	deCODE	IL11_P2
S100G_	S100G	UKB-PPP	5719_66	FAM189	deCODE	IL12B_F
SAFB2_	SAFB2	UKB-PPP	5753_21	FAM19A	deCODE	IL12RB1
SAG_P1	SAG	UKB-PPP	6430_36	FAM19A	deCODE	IL12RB2
SAMD9	SAMD9	UKB-PPP	7839_99	FAM19A	deCODE	IL13_P3
SARG_(	SARG	UKB-PPP	6511_17	FAM19A	deCODE	IL13RA
SART1_	SART1	UKB-PPP	5609_92	FAM19A	deCODE	IL15_P4
SCAMP	SCAMP	UKB-PPP	7066_19	FAM20C	deCODE	IL15RA_
SCARB1	SCARB1	UKB-PPP	7198_19	FAM20I	deCODE	IL16_Q1
SCG2_P	SCG2	UKB-PPP	14593_1	FAM210	deCODE	IL17A_(
SCGB2A	SCGB2A	UKB-PPP	13423_9	FAM213	deCODE	IL17C_(
SCGB3A	SCGB3A	UKB-PPP	8775_61	FAM24I	deCODE	IL17D_(
SCLY_(	SCLY	UKB-PPP	19176_2	FAM49I	deCODE	IL17F_(
SCN2A_	SCN2A	UKB-PPP	14341_8	FAM69C	deCODE	IL17RA_
SCN3A_	SCN3A	UKB-PPP	11336_9	FANCF	deCODE	IL17RB_
SCN3B_	SCN3B	UKB-PPP	10063_1	FANCL	deCODE	IL18_Q1
SCP2_P	SCP2	UKB-PPP	15581_1	FANK1	deCODE	IL18BP_
SCPEP1	SCPEP1	UKB-PPP	13941_8	FARS2	deCODE	IL18R1_
SCRG1_	SCRG1	UKB-PPP	8403_18	FASN	deCODE	IL18RA
SCRIB_	SCRIB	UKB-PPP	7740_33	FATE1	deCODE	IL19_Q9
SCRIB_	SCRIB	UKB-PPP	9074_6_	FAXDC	deCODE	IL1A_P(
SCRIB_	SCRIB	UKB-PPP	13994_1	FBL	deCODE	IL1B_P(
SCRIB_	SCRIB	UKB-PPP	6470_19	FBLN1	deCODE	IL1R1_F
SCRN1_	SCRN1	UKB-PPP	15585_3	FBLN5	deCODE	IL1R2_F
SDC1_P	SDC1	UKB-PPP	9867_23	FBP2	deCODE	IL1RAP_
SDC4_P	SDC4	UKB-PPP	9951_36	FBXL4	deCODE	IL1RL1_
SDCCA(	SDCCA(	UKB-PPP	11416_2	FBXL4	deCODE	IL1RL2_
SDHB_I	SDHB	UKB-PPP	12846_3	FBXL5	deCODE	IL1RN_
SDK2_(	SDK2	UKB-PPP	14628_7	FBXO3	deCODE	IL2_P60
SEC31A	SEC31A	UKB-PPP	3312_64	FCGR1A	deCODE	IL20_Q9
SEL1L_	SEL1L	UKB-PPP	15388_2	FCGR3A	deCODE	IL20RA_

SELEN(	SELEN(	UKB-PPP	5462_62	FCN3	deCODE	IL20RB_
SEMA3I	SEMA3I	UKB-PPP	8973_23	FCRL4	deCODE	IL21R_(
SEPTIN	SEPTIN	UKB-PPP	8991_11	FCRL4	deCODE	IL22_Q9
SEPTIN	SEPTIN	UKB-PPP	17346_6	FDCSP	deCODE	IL22RA
SEPTIN	SEPTIN	UKB-PPP	11231_1	FDX1L	deCODE	IL24_Q1
SEPTIN	SEPTIN	UKB-PPP	4220_39	FER	deCODE	IL25_Q9
SERPIN	SERPIN	UKB-PPP	12599_1	FERMT	deCODE	IL2RA_
SERPIN	SERPIN	UKB-PPP	12740_5	FEV	deCODE	IL2RB_
SERPIN	SERPIN	UKB-PPP	18890_2	FGB	deCODE	IL2RG_
SESTD1	SESTD1	UKB-PPP	3486_58	FGF1	deCODE	IL3_P08
SEZ6_Q	SEZ6	UKB-PPP	2441_2_	FGF10	deCODE	IL31_Q6
SFTPA1	SFTPA1	UKB-PPP	3494_71	FGF17	deCODE	IL31RA_
SFTPA2	SFTPA2	UKB-PPP	2761_49	FGF18	deCODE	IL32_P2
SGSH_I	SGSH	UKB-PPP	9513_9_	FGF22	deCODE	IL34_Q6
SH2B3_	SH2B3	UKB-PPP	4123_60	FGF4	deCODE	IL36A_(
SH3BP1	SH3BP1	UKB-PPP	17166_4	FGF8	deCODE	IL36G_(
SHD_Q9	SHD	UKB-PPP	19570_1	FGF8	deCODE	IL3RA_
SHISA5_	SHISA5	UKB-PPP	2443_10	FGF8	deCODE	IL4_P05
SHPK_(	SHPK	UKB-PPP	4394_71	FGF8	deCODE	IL4R_P2
SIGLEC	SIGLEC	UKB-PPP	5532_53	FGFR1	deCODE	IL5_P05
SIL1_Q9	SIL1	UKB-PPP	13669_6	FGFR3	deCODE	IL5RA_(
SIRT1_(	SIRT1	UKB-PPP	3809_1_	FGFR3	deCODE	IL6_P05
SKAP1_	SKAP1	UKB-PPP	6237_11	FGFRL1	deCODE	IL6_P05
SKAP2_	SKAP2	UKB-PPP	4989_7_	FGG	deCODE	IL6_P05
SKIV2L	SKIV2L	UKB-PPP	19635_6	FGL2	deCODE	IL6_P05
SLA2_Q	SLA2	UKB-PPP	16814_1	FHL1	deCODE	IL6R_P(
SLC12A	SLC12A	UKB-PPP	9378_6_	FIBCD1	deCODE	IL6ST_I
SLC13A	SLC13A	UKB-PPP	19169_8	FIBP	deCODE	IL7_P13
SLC16A	SLC16A	UKB-PPP	6948_82	FIG4	deCODE	IL7R_P1
SLC1A4	SLC1A4	UKB-PPP	13098_9	FIGF	deCODE	IL9_P15
SLC27A	SLC27A	UKB-PPP	14705_1	FIGF	deCODE	IMPA1_
SLC28A	SLC28A	UKB-PPP	7921_65	FJX1	deCODE	ING1_Q
SLC34A	SLC34A	UKB-PPP	9339_20	FKBP2	deCODE	INHBB_
SLC39A	SLC39A	UKB-PPP	18383_9	FKBP3	deCODE	INHBC_
SLC39A	SLC39A	UKB-PPP	12529_3	FKBP6	deCODE	INSL3_I
SLC44A	SLC44A	UKB-PPP	12677_1	FLII	deCODE	INSL4_(
SLC4A1	SLC4A1	UKB-PPP	11171_2	FLNA	deCODE	INSL5_(
SLC51B	SLC51B	UKB-PPP	4547_59	FLRT1	deCODE	INSR_P(

SLC9A3	SLC9A3	UKB-PPP
SLC9A3	SLC9A3	UKB-PPP
SLIRP_(	SLIRP	UKB-PPP
SLITRK	SLITRK	UKB-PPP
SLK_Q9	SLK	UKB-PPP
SLURP1	SLURP1	UKB-PPP
SMAD5_	SMAD5	UKB-PPP
SMARC	SMARC	UKB-PPP
SMPD3_	SMPD3	UKB-PPP
SMPDL3	SMPDL3	UKB-PPP
SNAP23	SNAP23	UKB-PPP
SNCG_(	SNCG	UKB-PPP
SNED1_	SNED1	UKB-PPP
SNU13_	SNU13	UKB-PPP
SNX15_	SNX15	UKB-PPP
SNX18_	SNX18	UKB-PPP
SNX2_C	SNX2	UKB-PPP
SNX9_C	SNX9	UKB-PPP
SORBS1	SORBS1	UKB-PPP
SOWAH	SOWAH	UKB-PPP
SOX9_P	SOX9	UKB-PPP
SPAG1_	SPAG1	UKB-PPP
SPART_	SPART	UKB-PPP
SPESP1_	SPESP1	UKB-PPP
SPRED2	SPRED2	UKB-PPP
SPRING	SPRING	UKB-PPP
SPRR1B	SPRR1B	UKB-PPP
SPRR3_	SPRR3	UKB-PPP
SPRY2_	SPRY2	UKB-PPP
SPTBN2	SPTBN2	UKB-PPP
SRPK2_	SRPK2	UKB-PPP
SRPX_P	SRPX	UKB-PPP
SSBP1_(	SSBP1	UKB-PPP
SSC4D_	SSC4D	UKB-PPP
SSC5D_	SSC5D	UKB-PPP
SSH3_Q	SSH3	UKB-PPP
SSNA1_	SSNA1	UKB-PPP
ST8SIA1	ST8SIA1	UKB-PPP

13123_3	FLRT3	deCODE
9128_34	FLRT3	deCODE
6367_66	FMOD	deCODE
13451_2	FNDC4	deCODE
14051_5	FOXC2	deCODE
9896_21	FOXG1	deCODE
14204_5	FOXJ2	deCODE
11375_4	FOXL2	deCODE
10056_5	FOXM1	deCODE
8017_23	FOXRE1	deCODE
7246_4_	FREM2	deCODE
7976_19	FRRS1L	deCODE
13025_4	FRS2	deCODE
9350_3_	FSTL4	deCODE
7099_33	FSTL5	deCODE
15324_5	FTL	deCODE
8048_9_	FTMT	deCODE
7156_2_	FUT10	deCODE
9121_28	FUT2	deCODE
4549_78	FUT5	deCODE
6991_24	FUT9	deCODE
13076_4	FXR1	deCODE
3813_3_	FYN	deCODE
8931_12	G0S2	deCODE
19266_3	G3BP1	deCODE
9831_12	G3BP2	deCODE
7065_1_	G6B	deCODE
8659_68	G6B	deCODE
19297_4	G6PD	deCODE
9385_4_	GAA	deCODE
12494_9	GABAR	deCODE
11279_4	GABBR	deCODE
13948_5	GABBR	deCODE
9930_48	GABBR	deCODE
18273_1	GAGE2I	deCODE
8696_15	GAL3ST	deCODE
10734_3	GAL3ST	deCODE
11457_5	GALE	deCODE

IRAK4_
ISLR2_(
ISM1_B
IST1_P5
ITGA2_
ITGA5_
ITGAL_
ITGB2_
ITGB5_
ITGB6_
ITGB7_
ITIH1_P
ITIH3_C
ITIH4_C
ITIH5_C
ITM2A_
ITPA_Q
IVD_P2
JAM2_P
JAM3_C
JUN_P0
KAZAL
KCNIP4
KCTD5_
KDR_P3
KEL_P2
KIAA15
KIAA20
KIF1C_(
KIF22_(
KIR2DL
KIR2DL
KIR2DS
KIR3DL
KIR3DL
KIRREL
KIT_P1(
KITLG_

STAM_(	STAM	UKB-PPP	11448_3	GALK1	deCODE	KLB_Q8
STAT2_	STAT2	UKB-PPP	7090_17	GALNT	deCODE	KLF4_C
STEAP4	STEAP4	UKB-PPP	8700_32	GALNT	deCODE	KLK10_
STK11_	STK11	UKB-PPP	10908_2	GALNT	deCODE	KLK11_
STK4_Q	STK4	UKB-PPP	8923_94	GALNT	deCODE	KLK12_
STX16_	STX16	UKB-PPP	9398_30	GALP	deCODE	KLK13_
STX5_Q	STX5	UKB-PPP	10054_3	GAN	deCODE	KLK14_
STXBP1	STXBP1	UKB-PPP	15325_1	GAP43	deCODE	KLK15_
STXBP3	STXBP3	UKB-PPP	8004_15	GAPDH	deCODE	KLK3_P
SUGP1_	SUGP1	UKB-PPP	5463_22	GAS1	deCODE	KLK4_(
SULT1A	SULT1A	UKB-PPP	17721_8	GAS7	deCODE	KLK6_(
SUSD2_	SUSD2	UKB-PPP	18188_1	GATM	deCODE	KLK7_P
SUSD4_	SUSD4	UKB-PPP	12594_5	GCA	deCODE	KLK8_(
SUSD5_	SUSD5	UKB-PPP	19258_2	GCDH	deCODE	KLKB1_
SYAP1_	SYAP1	UKB-PPP	11185_1	GCH1	deCODE	KLRB1_
SYNGA	SYNGA	UKB-PPP	12960_9	GCK	deCODE	KLRC1_
SYT1_P	SYT1	UKB-PPP	5223_59	GCKR	deCODE	KLRF1_
TAB2_C	TAB2	UKB-PPP	7143_9_	GCNT2	deCODE	KLRK1_
TACC3_	TACC3	UKB-PPP	10842_7	GCNT4	deCODE	KRT17_
TADA3_	TADA3	UKB-PPP	10956_8	GCSH	deCODE	KRT18_
TAFA5_	TAFA5	UKB-PPP	12667_2	GDA	deCODE	KRT19_
TAP1_Q	TAP1	UKB-PPP	16749_7	GDF10	deCODE	KRT5_P
TARM1_	TARM1	UKB-PPP	6517_14	GDF10	deCODE	KYNU_
TARS1_	TARS1	UKB-PPP	14587_1	GDF11	deCODE	L1CAM_
TBC1D1	TBC1D1	UKB-PPP	16755_1	GDF3	deCODE	LACRT_
TBC1D2	TBC1D2	UKB-PPP	2752_62	GDF5	deCODE	LAG3_P
TBCC_(	TBCC	UKB-PPP	16756_3	GDF7	deCODE	LAIR1_(
TBL1X_	TBL1X	UKB-PPP	3067_67	GDF9	deCODE	LAIR2_(
TBR1_C	TBR1	UKB-PPP	16810_3	GDI1	deCODE	LAMA4
TCOF1_	TCOF1	UKB-PPP	2647_66	GDI2	deCODE	LAMP1_
TCTN3_	TCTN3	UKB-PPP	12817_1	GEM	deCODE	LAP3_P
TDRKH_	TDRKH	UKB-PPP	11683_1	GGA3	deCODE	LAT_O4
TEF_Q1	TEF	UKB-PPP	17786_5	GGPS1	deCODE	LAT2_C
TET2_Q	TET2	UKB-PPP	6334_9_	GGT2	deCODE	LAYN_(
TEX101	TEX101	UKB-PPP	9730_22	GHDC	deCODE	LBP_P1
TEX33_	TEX33	UKB-PPP	16293_1	GHRH	deCODE	LCAT_P
TFAP2A	TFAP2A	UKB-PPP	9487_60	GHRH	deCODE	LCN2_P
TGFA_P	TGFA	UKB-PPP	17672_1	GIF	deCODE	LCP1_P

TGFBR	TGFBR	UKB-PPP	19302_7	GIMAP(	deCODE	LDLR_I
TGOLN	TGOLN	UKB-PPP	19260_4	GIPC1	deCODE	LDLRA
THAP12	THAP12	UKB-PPP	9937_7_	GJA1	deCODE	LECT2_
THBD_I	THBD	UKB-PPP	11678_1	GJD2	deCODE	LEFTY2
THOP1_	THOP1	UKB-PPP	6416_8_	GKN2	deCODE	LEG1_C
THRAP	THRAP	UKB-PPP	7808_5_	GLCE	deCODE	LEP_P4
THY1_F	THY1	UKB-PPP	9265_10	GLIPR1	deCODE	LEPR_P
TIA1_P	TIA1	UKB-PPP	15522_2	GLIPR2	deCODE	LGALS1
TIMM1(	TIMM1(	UKB-PPP	12486_8	GLRX2	deCODE	LGALS3
TJAP1_(	TJAP1	UKB-PPP	16596_2	GLRX3	deCODE	LGALS3
TJP3_O	TJP3	UKB-PPP	8955_60	GLT8D1	deCODE	LGALS4
TMED1_	TMED1	UKB-PPP	12513_8	GLTP	deCODE	LGALS7
TMED8_	TMED8	UKB-PPP	7948_12	GLTPD2	deCODE	LGALS8
TMOD4	TMOD4	UKB-PPP	19238_1	GLUL	deCODE	LGALS9
TMSB1(	TMSB1(	UKB-PPP	19506_6	GLYAT	deCODE	LGMN_
TNFAIP	TNFAIP	UKB-PPP	18337_4	GMDS	deCODE	LHB_P0
TNFAIP	TNFAIP	UKB-PPP	12804_5	GMEB2	deCODE	LIF_P15
TNFRSF	TNFRSF	UKB-PPP	17784_2	GMFB	deCODE	LIFR_P4
TNFRSF	TNFRSF	UKB-PPP	17703_4	GMNN	deCODE	LILRA2
TNFSF1	TNFSF1	UKB-PPP	18184_2	GMPS	deCODE	LILRA3
TNIP1_(	TNIP1	UKB-PPP	19271_6	GNAI1	deCODE	LILRA4
TNN_Q	TNN	UKB-PPP	12650_4	GNAI3	deCODE	LILRA5
TNPO1_	TNPO1	UKB-PPP	18282_1	GNG11	deCODE	LILRA6
TOMM2	TOMM2	UKB-PPP	10917_4	GNGT2	deCODE	LILRB1
TOP1M	TOP1M	UKB-PPP	14006_3	GNMT	deCODE	LILRB2
TOP2B_	TOP2B	UKB-PPP	13954_9	GNPNA	deCODE	LILRB4
TP53BP	TP53BP	UKB-PPP	10666_7	GNPTG	deCODE	LILRB5
TP53INI	TP53INI	UKB-PPP	5627_53	GNRH1	deCODE	LMNB1
TP73_O	TP73	UKB-PPP	10708_3	GNRH2	deCODE	LMOD1
TPBGL_	TPBGL	UKB-PPP	3616_3_	GNS	deCODE	LMOD1
TPD52L	TPD52L	UKB-PPP	19174_1	GOLGA	deCODE	LMOD1
TPM3_F	TPM3	UKB-PPP	17456_5	GOLM1	deCODE	LMOD1
TPPP3_(	TPPP3	UKB-PPP	8983_7_	GOLM1	deCODE	LONP1_
TPR_P1	TPR	UKB-PPP	7247_1_	GORAB	deCODE	LPCAT2
TPSD1_	TPSD1	UKB-PPP	7805_52	GOSR1	deCODE	LPO_P2
TRAF2_	TRAF2	UKB-PPP	10426_2	GOSR2	deCODE	LRCH4_
TRAF3_	TRAF3	UKB-PPP	3315_15	GPC2	deCODE	LRFN2_
TRAF3I	TRAF3I	UKB-PPP	4842_62	GPC3	deCODE	LRIG1_(

TREH_(	TREH	UKB-PPP
TRIM21	TRIM21	UKB-PPP
TRIM24	TRIM24	UKB-PPP
TRIM25	TRIM25	UKB-PPP
TRIM26	TRIM26	UKB-PPP
TRIM40	TRIM40	UKB-PPP
TRIM5_	TRIM5	UKB-PPP
TRIM58	TRIM58	UKB-PPP
TRPV3_	TRPV3	UKB-PPP
TSC1_Q	TSC1	UKB-PPP
TSC22D	TSC22D	UKB-PPP
TSHB_F	TSHB	UKB-PPP
TSNAX_	TSNAX	UKB-PPP
TSPAN1	TSPAN1	UKB-PPP
TSPAN1	TSPAN1	UKB-PPP
TSPAN7	TSPAN7	UKB-PPP
TSPAN8	TSPAN8	UKB-PPP
TSPYL1	TSPYL1	UKB-PPP
TTF2_Q	TTF2	UKB-PPP
TTR_P0	TTR	UKB-PPP
TUBB3_	TUBB3	UKB-PPP
TWF2_(	TWF2	UKB-PPP
TXK_P4	TXK	UKB-PPP
TXNDC	TXNDC	UKB-PPP
TXNL1_	TXNL1	UKB-PPP
TYRP1_	TYRP1	UKB-PPP
UBAC1_	UBAC1	UKB-PPP
UBE2Z_	UBE2Z	UKB-PPP
UBQLN	UBQLN	UKB-PPP
UBXN1_	UBXN1	UKB-PPP
UFD1_(	UFD1	UKB-PPP
UHRF2_	UHRF2	UKB-PPP
UNC79_	UNC79	UKB-PPP
UNG_P1	UNG	UKB-PPP
UPB1_C	UPB1	UKB-PPP
UPK3A_	UPK3A	UKB-PPP
UPK3BI	UPK3BI	UKB-PPP
USP47_(	USP47	UKB-PPP

18892_4	GPC4	deCODE
5350_14	GPC6	deCODE
12786_6	GPCPD1	deCODE
12420_1	GPD1L	deCODE
16599_3	GPN1	deCODE
12963_1	GPR107	deCODE
11465_4	GPR135	deCODE
12766_3	GPR142	deCODE
13540_1	GPR26	deCODE
18893_2	GPR56	deCODE
10767_5	GPR64	deCODE
12503_5	GPR68	deCODE
16015_1	GPT	deCODE
15591_2	GPX1	deCODE
18894_1	GPX2	deCODE
9475_22	GPX5	deCODE
8345_27	GPX7	deCODE
8336_26	GRAMI	deCODE
8842_16	GRAMI	deCODE
12820_1	GRAP	deCODE
11358_1	GRB10	deCODE
13628_5	GRB14	deCODE
5464_52	GRB2	deCODE
11281_6	GRB7	deCODE
18878_1	GREM1	deCODE
5598_3_	GREM2	deCODE
10760_1	GRIA4	deCODE
13493_5	GRID1	deCODE
12758_4	GRID2	deCODE
12849_2	GSKIP	deCODE
15526_3	GSS	deCODE
14645_2	GSTA4	deCODE
13474_4	GSTK1	deCODE
15395_1	GSTM1	deCODE
9748_31	GSTM3	deCODE
12436_8	GSTO1	deCODE
19230_1	GSTT1	deCODE
16872_2	GSTZ1	deCODE

LRIG3_(
LRP1_Q
LRP11_(
LRP2_P
LRPAP1
LRRC25
LRRC37
LRRC59
LRRN1_
LRTM1_
LRTM2_
LSM1_(
LTA_P0
LTA4H_
LTBR_F
LY75_O
LY9_Q9
LYN_P0
LYPD1_
LYPD3_
LYSMD
LYVE1_
LYZL2_
MAD1L
MAGEA
MAMD(
MAN1A
MAN2B
MANF_
MANSC
MANSC
MAP1L(
MAP2K
MAP2K
MAPK1
MAPK9_
MAPKA
MAPRE

VAMP5_	VAMP5	UKB-PPP	13609_1	GTF2I	deCODE	MAPT_
VASH1_	VASH1	UKB-PPP	10008_4	GUCA1	deCODE	MARCO
VCPKM	VCPKM	UKB-PPP	6223_5_	GUCA2	deCODE	MASP1_
VEGFD_	VEGFD	UKB-PPP	19180_3	GULP1	deCODE	MATN2
VGF_O	VGF	UKB-PPP	8229_1_	GXYLT	deCODE	MATN3
VIPR1_	VIPR1	UKB-PPP	9545_15	GZMK	deCODE	MAX_P
VMO1_	VMO1	UKB-PPP	5704_74	GZMM	deCODE	MB_P0
VNN1_	VNN1	UKB-PPP	12709_6	H1FX	deCODE	MBL2_
VPS37A	VPS37A	UKB-PPP	4163_5_	H2AFZ	deCODE	MCEE_
VPS53_	VPS53	UKB-PPP	7161_25	H6PD	deCODE	MCEM
VSIG10	VSIG10	UKB-PPP	5861_78	HAAO	deCODE	MCTS1_
VSIG4_	VSIG4	UKB-PPP	10815_2	HABP4	deCODE	MDGA1
VSIR_Q	VSIR	UKB-PPP	3504_58	HAMP	deCODE	MDH1_
VSTM1_	VSTM1	UKB-PPP	3196_6_	HAPLN	deCODE	MDK_P
VSTM2	VSTM2	UKB-PPP	6455_52	HAPLN	deCODE	MDM1_
VTCN1_	VTCN1	UKB-PPP	17450_5	HARS	deCODE	MEGF1
VWA5A	VWA5A	UKB-PPP	19327_3	HAT1	deCODE	MENT_
WAS_P	WAS	UKB-PPP	17137_1	HBB	deCODE	MEPE_
WASF1_	WASF1	UKB-PPP	6992_67	HBD	deCODE	MET_P
WASF3_	WASF3	UKB-PPP	13495_4	HCAR2	deCODE	METAP
WASHC	WASHC	UKB-PPP	3374_49	HCK	deCODE	METAP
WASL_	WASL	UKB-PPP	12774_1	HCN1	deCODE	MFAP3
WDR46_	WDR46	UKB-PPP	18897_3	HDAC2	deCODE	MFAP4_
WFDC1	WFDC1	UKB-PPP	18898_3	HDGFL	deCODE	MFAP5_
WNT9A	WNT9A	UKB-PPP	4553_65	HDGFR	deCODE	MFGE8_
WWP2_	WWP2	UKB-PPP	18899_8	HDGFR	deCODE	MIA_Q1
XIAP_P	XIAP	UKB-PPP	13472_3	HDHD2	deCODE	MICAL
XPNPEI	XPNPEI	UKB-PPP	19482_1	HDHD3	deCODE	MICB_
YAP1_P	YAP1	UKB-PPP	13570_4	HDLBP	deCODE	MIF_P1
YARS1_	YARS1	UKB-PPP	12669_3	HECW1	deCODE	MILR1_
YJU2_Q	YJU2	UKB-PPP	8810_26	HEPAC	deCODE	MLN_P
YOD1_	YOD1	UKB-PPP	6354_13	HEPHL	deCODE	MMP1_
YTHDF	YTHDF	UKB-PPP	12705_9	HERC1	deCODE	MMP10
ZBP1_Q	ZBP1	UKB-PPP	7860_9_	HERC4	deCODE	MMP12
ZBTB16	ZBTB16	UKB-PPP	12934_1	HERC5	deCODE	MMP13
ZBTB17	ZBTB17	UKB-PPP	9779_63	HERPU	deCODE	MMP3_
ZCCHC	ZCCHC	UKB-PPP	15470_1	HEXB	deCODE	MMP7_
ZFYVE1	ZFYVE1	UKB-PPP	14205_6	HEXIM	deCODE	MMP8_

ZHX2_(	ZHX2	UKB-PPP
ZNF830_	ZNF830	UKB-PPP
ZNRD2_	ZNRD2	UKB-PPP
ZNRF4_	ZNRF4	UKB-PPP
ZP3_P2	ZP3	UKB-PPP
ZPR1_O	ZPR1	UKB-PPP

3332_57	HFE2	deCODE
9832_33	HGD	deCODE
10833_6	HHIP	deCODE
14132_2	HHLA2	deCODE
4693_72	HIBADH	deCODE
12396_1	HIBCH	deCODE
17199_4	HIF1AN	deCODE
5900_11	HINT1	deCODE
5612_16	HINT2	deCODE
19329_3	HIPK3	deCODE
2987_37	HIST1H	deCODE
14146_9	HIST1H	deCODE
14143_8	HIST2H	deCODE
14144_3	HIST3H	deCODE
18823_5	HIST3H	deCODE
13131_5	HK1	deCODE
19518_1	HK3	deCODE
19636_2	HLA	deCODE
6962_5_	HLA	deCODE
7757_5_	HLA	deCODE
12712_9	HMG20.	deCODE
11551_1	HMG20	deCODE
16536_3	HMGA1	deCODE
19516_1	HMGA2	deCODE
2524_56	HMGB1	deCODE
6913_18	HMGB2	deCODE
12775_6	HMGB3	deCODE
5230_99	HMGCR	deCODE
13704_5	HMGCS	deCODE
9187_2_	HMGN1	deCODE
13544_9	HMHA1	deCODE
19335_2	HN1	deCODE
10041_3	HNF4A	deCODE
12466_7	HNRNP.	deCODE
5351_52	HNRNP.	deCODE
8894_80	HNRNP.	deCODE
11429_8	HNRNP	deCODE
18348_8	HNRNP.	deCODE

MMP9_
MOG_Q
MORF4
MPO_P(
MRC1_
MRPL5
MSLN_(
MSMB_
MSR1_F
MSRA_(
MST1_F
MSTN_(
MTHFD
MTHFS
MTIF3_
MUC16_
MVK_Q
MXRA8
MYBPC
MYDGF
MYL3_
MYL6B_
MYO6_(
MYOC_
MYOM2
MYOM3
NAA10_
NAAA_
NADK_
NAGK_
NAGPA
NAMPT
NAP1L4
NBL1_P
NCAM1
NCAM2
NCAN_(
NCF2_P

10852_1	HNRNP	deCODE	NCK2_
9764_79	HNRNP	deCODE	NCR1_
14309_8	HNRNP	deCODE	NCR3L
12783_2	HNRNP	deCODE	NDRG1_
13512_2	HNRNP	deCODE	NDST1_
10024_4	HOGA1	deCODE	NDUFA
19229_9	HOMER	deCODE	NEDD9_
12685_5	HOMER	deCODE	NEFL_P
19196_7	HOPX	deCODE	NEK7_
3054_3_	HP	deCODE	NELL1_
7905_30	HP	deCODE	NELL2_
17509_6	HPD	deCODE	NENF_
4995_16	HPGD	deCODE	NEO1_
19290_5	HPRT1	deCODE	NFASC_
15347_1	HPX	deCODE	NFATC
7822_11	HRASL	deCODE	NFKB1_
8008_28	HRK	deCODE	NFU1_
14636_2	HRSP12	deCODE	NGF_P
15497_9	HS3ST1	deCODE	NGFR_
8268_98	HS3ST3	deCODE	NGRN_
19370_3	HS3ST4	deCODE	NHLRC
8998_15	HS3ST4	deCODE	NID1_P
10731_1	HS3ST5	deCODE	NID2_Q
18896_2	HS6ST3	deCODE	NIT2_Q
19316_2	HSCB	deCODE	NLGN1_
4708_3_	HSD17	deCODE	NLGN2_
4217_49	HSD17	deCODE	NME1_
17397_8	HSD17	deCODE	NMI_Q
8272_22	HSD17	deCODE	NMNA
9929_16	HSD17	deCODE	NMT1_
19491_1	HSD17	deCODE	NOTCH
11616_9	HSF1	deCODE	NOTCH
19139_3	HSF2B	deCODE	NOTCH
5467_15	HSP90A	deCODE	NPDC1_
17515_6	HSPA1	deCODE	NPL_Q
18901_2	HSPA1	deCODE	NPM1_
12041_3	HSPA1	deCODE	NPPB_P
16588_1	HSPA5	deCODE	NPTN_

13672_3	HSPA6	deCODE	NPTX1_
5903_91	HSPA8	deCODE	NPTX2_
13492_4	HSPA9	deCODE	NPTXR_
18236_3	HSPC15	deCODE	NRCAM
2682_68	HSPD1	deCODE	NRGN_
17150_8	HSPE1	deCODE	NRP1_C
17704_7	HSPH1	deCODE	NRP2_C
10630_5	HTATIP	deCODE	NRXN3_
7753_21	HTATIP	deCODE	NSFL1C
10608_9	HTN1	deCODE	NT5C_C
10603_1	HTN3	deCODE	NT5C3A
13556_2	HTR2A	deCODE	NT5E_P
13561_5	HTR6	deCODE	NTF3_P
13547_5	HTR7	deCODE	NTF4_P
15594_4	HTRA1	deCODE	NTRK2_
19639_5	IAPP	deCODE	NTRK3_
12815_9	IARS	deCODE	NUDC_
3415_61	IBSP	deCODE	NUDT2_
6380_23	ICT1	deCODE	NUDT5_
15402_2	ID1	deCODE	NXPH1_
15403_5	ID2	deCODE	NXPH3_
3197_70	IDE	deCODE	OBP2B_
18338_2	IDH1	deCODE	OGN_P2
19275_6	IDH3G	deCODE	OLFM4_
17712_7	IDI1	deCODE	OLR1_P
9786_31	IER3	deCODE	OMD_Q
12893_1	IFI16	deCODE	OMG_P
13940_1	IFI16	deCODE	OMP_P4
9853_3_	IFIT2	deCODE	OPHN1_
18389_1	IFNA1	deCODE	OPTC_C
14128_1	IFNA10	deCODE	OSCAR_
7180_11	IFNA14	deCODE	OSM_P1
6421_52	IFNA16	deCODE	OSMR_
3497_13	IFNA2	deCODE	OSTN_F
15404_3	IFNA21	deCODE	OXT_P0
15405_2	IFNA4	deCODE	P4HB_P
6210_10	IFNA5	deCODE	PAEP_P
5714_88	IFNA6	deCODE	PAFAH1

14129_1	IFNA7	deCODE	PAFAH:
6214_84	IFNA8	deCODE	PAK4_C
14127_2	IFNB1	deCODE	PALM_(
5713_9_	IFNL3	deCODE	PAM_P1
2952_75	IGF1	deCODE	PAPPA_
8406_17	IGF1	deCODE	PARK7_
15295_8	IGF2	deCODE	PARP1_
6605_17	IGFALS	deCODE	PBK_Q9
19581_1	IGFBP5	deCODE	PCBD1_
6961_14	IGFL3	deCODE	PCDH9_
7244_16	IGFLR1	deCODE	PCNA_I
13230_1	IGHG2	deCODE	PCOLCI
13231_9	IGHG4	deCODE	PCSK7_
15306_2	IGJ	deCODE	PCSK9_
6485_59	IGLL1	deCODE	PDAP1_
10700_1	IGSF11	deCODE	PDCD1_
9060_27	IGSF9B	deCODE	PDCD1I
19606_2	IHH	deCODE	PDCD5_
7207_4_	IK	deCODE	PDCD6_
18216_2	IL11RA	deCODE	PDCL2_
3499_77	IL17B	deCODE	PDE4D_
5468_67	IL17RC	deCODE	PDE5A_
3376_49	IL17RD	deCODE	PDGFA_
17356_3	IL1F10	deCODE	PDGFB_
18375_2	IL1F5	deCODE	PDGFC_
5082_51	IL1RAP	deCODE	PDGFRA
7124_18	IL21	deCODE	PDGFRI
5087_5_	IL22RA:	deCODE	PDIA3_
5088_17	IL23R	deCODE	PDIA4_
16760_2	IL26	deCODE	PDIA5_(
5132_71	IL27RA	deCODE	PDRG1_
14149_9	IL36B	deCODE	PDZK1_
2723_9_	IL37	deCODE	PEAR1_
12665_1	ILF2	deCODE	PEBP1_
12759_4	ILF3	deCODE	PECAM
10053_5	ILK	deCODE	PECR_C
12581_3	IMPA2	deCODE	PENK_I
9231_23	IMPAD:	deCODE	PF4_P02

5229_90	IMPDH1	deCODE	PFDN2_
5250_53	IMPDH2	deCODE	PGD_P5
11436_6	INA	deCODE	PGF_P4
17671_5	ING4	deCODE	PGLYRI
13738_8	INHBA	deCODE	PGLYRI
19622_7	INHBA	deCODE	PGM2_(
12641_3	INPP5B	deCODE	PGR_P0
11370_2	INPP5E	deCODE	PI3_P19
4883_56	INS	deCODE	PIGR_P(
10663_4	INSIG1	deCODE	PILRA_
5754_76	INSL6	deCODE	PKD2_C
6352_8_	INSRR	deCODE	PKLR_F
11934_9	INTS3	deCODE	PLA2G1
7991_54	IQCF1	deCODE	PLA2G1
13439_6	IQCF3	deCODE	PLA2G2
17462_1	IRF1	deCODE	PLA2G7
12801_3	IRF2	deCODE	PLAT_P
17151_8	IRF3	deCODE	PLAU_F
19564_6	IRF4	deCODE	PLAUR_
9999_1_	IRF6	deCODE	PLCB1_
12439_6	IRF9	deCODE	PLG_P0
7201_5_	ISCU	deCODE	PLIN3_(
14151_4	ISG15	deCODE	PLSCR3
11549_6	ISL1	deCODE	PLTP_P
9816_37	ISOC1	deCODE	PLXDC1
12551_3	ITCH	deCODE	PLXDC2
9326_33	ITIH2	deCODE	PLXNA4
18830_1	ITLN1	deCODE	PLXNB2
8086_49	ITM2B	deCODE	PLXNB3
10560_1	ITM2C	deCODE	PMM2_(
9523_34	ITM2C	deCODE	PMVK_
13473_5	ITPKA	deCODE	PNLIP_I
12507_1	ITPKC	deCODE	PNLIPR
9221_6_	ITPRIPI	deCODE	PNLIPR
14070_5	ITSN1	deCODE	PODXL2
5092_51	JAG1	deCODE	POMC_I
7754_11	JAG1	deCODE	PON1_F
5093_47	JAG2	deCODE	POSTN_

11816_8	JAK2	deCODE	PPBP_P
9068_17	JAKMIF	deCODE	PPCDC_
12623_8	JARID2	deCODE	PPIB_P2
18322_1	JMJD6	deCODE	PPIE_Q9
6940_18	JPH1	deCODE	PPIF_P3
9089_77	JPH3	deCODE	PPM1A_
9111_40	JPH4	deCODE	PPME1_
9038_12	JTB	deCODE	PPP1CC
19602_3	JUND	deCODE	PPP1R1
16768_3	JUP	deCODE	PPP1R2_
19492_5	KAAG1	deCODE	PPP2R5
6603_18	KAL1	deCODE	PPP3R1_
19249_1	KARS	deCODE	PPT1_P5
19369_1	KAT2A	deCODE	PPY_P0
12368_1	KAT2B	deCODE	PQBP1_
13042_7	KCNA1	deCODE	PRC1_C
10015_1	KCNAB	deCODE	PRCP_P
8067_21	KCNE3	deCODE	PRDX1_
8756_41	KCNE5	deCODE	PRDX3_
8908_14	KCNE5	deCODE	PRDX5_
12834_3	KCNF1	deCODE	PRDX6_
13525_1	KCNG4	deCODE	PRG2_P
13650_1	KCNIP1	deCODE	PRG3_C
10513_1	KCNIP3	deCODE	PRKAR
8905_20	KCNMF	deCODE	PRKCQ_
13539_1	KCNN1	deCODE	PRKG1_
6611_8_	KDELC	deCODE	PRL_P0
8296_11	KDELC	deCODE	PRND_(
19483_1	KDSR	deCODE	PROC_F
8485_7_	KEAP1	deCODE	PROK1_
12568_1	KEAP1	deCODE	PROS1_
10758_2	KERA	deCODE	PRSS2_
14603_5	KIAA00	deCODE	PRSS22_
13698_2	KIAA03	deCODE	PRSS27_
9075_12	KIAA10	deCODE	PRSS8_
8068_43	KIAA11	deCODE	PRTN3_
8093_13	KIAA11	deCODE	PSAP_P
12956_4	KIAA12	deCODE	PSAPL1

10637_5	KIAA13	deCODE	PSG1_P
9097_5_	KIAA13	deCODE	PSIP1_C
8363_18	KIAA13	deCODE	PSMA1_
7892_13	KIAA14	deCODE	PSMD5_
9981_18	KIAA14	deCODE	PSMD9_
10432_3	KIAA16	deCODE	PSME1_
11672_1	KIF16B	deCODE	PSME2_
5228_25	KIF23	deCODE	PSMG3_
13961_1	KIF3A	deCODE	PSPN_C
13476_1	KIN	deCODE	PTEN_F
17200_5	KIR2DL	deCODE	PTGDS_
5095_21	KIR2DL	deCODE	PTGES2
8000_17	KIR2DL	deCODE	PTGR1_
10428_1	KIR2DS	deCODE	PTH_P0
7944_1_	KIR3DL	deCODE	PTH1R_
8045_3_	KIR3DL	deCODE	PTK7_C
5097_14	KIR3DS	deCODE	PTN_P2
4557_61	KIRREL	deCODE	PTP4A3
6606_61	KISS1	deCODE	PTPN1_
15384_1	KL	deCODE	PTPN6_
12656_1	KLC1	deCODE	PTPN9_
12695_6	KLHL12	deCODE	PTPRH_
12463_7	KLHL13	deCODE	PTPRR_
12625_1	KLHL7	deCODE	PTPRS_
14039_3	KLK5	deCODE	PTS_Q0
3201_49	KLK5	deCODE	PTTG1_
5758_49	KLK9	deCODE	PTX3_P
7795_14	KLRC3	deCODE	PVR_P1
11571_7	KLRC3	deCODE	PXDNL_
10552_8	KLRC4	deCODE	PYDC1_
10693_4	KLRG2	deCODE	PYY_P1
10762_2	KLRG2	deCODE	PZP_P20
11402_1	KMT2C	deCODE	QDPR_F
15343_3	KNG1	deCODE	QPCT_C
19631_1	KNG1	deCODE	RAB27E
7784_1_	KNG1	deCODE	RAB2B_
19587_1	KPNA1	deCODE	RAB39E
2860_19	KPNA2	deCODE	RAB6B_

12698_7	KPNA4	deCODE	RABEPI
13969_2	KPNA6	deCODE	RAC3_F
3887_90	KPNB1	deCODE	RAD23E
17331_1	KREME	deCODE	RAD51_
3202_28	KREME	deCODE	RALB_F
9931_20	KRT1	deCODE	RANBP
18905_5	KRT16	deCODE	RARRE
12975_1	KRT20	deCODE	RARRE
11383_4	KRT7	deCODE	RASA1_
11566_4	KRT72	deCODE	RBKS_C
14615_4	KRTAP	deCODE	RBM17_
5739_75	KRTDA	deCODE	RBM19_
12708_9	L3MBT	deCODE	RBP1_P
6407_63	LAD1	deCODE	RBP5_P
9580_5_	LAMC2	deCODE	RBP7_Q
12490_9	LAMTO	deCODE	REG1A_
13986_6	LANCL	deCODE	REG1B_
12628_3	LANCL	deCODE	REG3A_
7935_26	LARGE	deCODE	REG3G_
3452_17	LCK	deCODE	REG4_C
4560_34	LCK	deCODE	RELB_C
4237_70	LCMT1	deCODE	RELT_C
11708_2	LCN1	deCODE	REN_P0
13007_6	LCN10	deCODE	RET_P0
5643_2_	LCN8	deCODE	RETN_C
4304_18	LCORL	deCODE	REXO2_
9017_58	LCT	deCODE	RGMA_
10890_1	LCTL	deCODE	RGMB_
15414_3	LDHA	deCODE	RGS10_
3890_8_	LDHB	deCODE	RGS8_P
9828_86	LDHC	deCODE	RHOC_
9004_24	LDLRA	deCODE	RLN2_F
8378_3_	LDOC1	deCODE	RNASE
5708_1_	LEAP2	deCODE	RNASE
9928_12	LECT1	deCODE	RNASE
8040_9_	LEMD1	deCODE	RNASE
6273_58	LEPRE1	deCODE	RNASE
5751_14	LETMD	deCODE	RNASE

19147_6	LGALS1	deCODE		RNF149
3033_57	LGALS2	deCODE		RNF41_
8003_57	LGI3	deCODE		RNF43_
16304_6	LGR4	deCODE		ROBO1_
16296_4	LGR5	deCODE		ROBO2_
14634_1	LHCGR	deCODE		ROBO4_
15615_8	LILRB3	deCODE		ROR1_(
11334_7	LILRB3	deCODE		RPA2_P
11543_8	LIMA1	deCODE		RPE_Q9
18910_4	LIN7A	deCODE		RPS10_
15612_5	LIN7B	deCODE		RRAS_F
12986_1	LIN7C	deCODE		RRM2_
6620_82	LINGO1	deCODE		RRM2B
10827_6	LINGO3	deCODE		RSPO1_
6413_79	LIPK	deCODE		RSPO3_
8097_77	LIPN	deCODE		RTN4IP
15688_3	LMAN1	deCODE		RTN4R_
7638_30	LMAN2	deCODE		RUVBL
9468_8_	LMAN2	deCODE		S100A1
8013_9_	LMAN2	deCODE		S100A1
9936_27	LNX1	deCODE		S100A1
6504_65	LOXL2	deCODE		S100A1
15427_3	LOXL3	deCODE		S100A1
7910_41	LRFN1	deCODE		S100A3_
6587_6_	LRFN5	deCODE		S100A4_
11716_2	LRIT2	deCODE		S100P_F
11534_6	LRIT3	deCODE		SAA4_P
10704_9	LRMP	deCODE		SAP18_
16610_1	LRP10	deCODE		SAT1_P
15506_3	LRP12	deCODE		SAT2_Q
11275_9	LRP1B	deCODE		SATB1_
19558_1	LRP4	deCODE		SBSN_C
3323_37	LRP8	deCODE		SCARA
6557_50	LRRC15	deCODE		SCARB
9989_12	LRRC24	deCODE		SCARF1
6917_49	LRRC3	deCODE		SCARF2
7551_33	LRRC32	deCODE		SCG3_C
18947_3	LRRC3B	deCODE		SCGB1A

9916_14	LRRC4I	deCODE	SCGB3A
11911_1	LRRC4I	deCODE	SCGN_(
9369_17	LRRC4(	deCODE	SCIN_Q
10785_8	LRRC52	deCODE	SCN2B_
8389_8_	LRRC74	deCODE	SCN4B_
10990_2	LRRK2	deCODE	SCT_P0
19360_2	LRRTM	deCODE	SELE_P
6904_14	LRRTM	deCODE	SELL_P
4453_83	LRRTM	deCODE	SELP_P
6572_10	LRRTM	deCODE	SELPLG
8646_61	LRRTM	deCODE	SEMA3(
2999_6_	LSAMP	deCODE	SEMA4(
16854_1	LSM4	deCODE	SEMA4I
9531_24	LST1	deCODE	SEMA6(
13477_6	LTB4R	deCODE	SEMA7A
13133_7	LTBP4	deCODE	SERPIN
2780_35	LTF	deCODE	SERPIN
13114_5	LUM	deCODE	SERPIN
6256_9_	LY6G6C	deCODE	SERPIN
6469_62	LY6G6I	deCODE	SERPIN
3623_84	LY86	deCODE	SERPIN
9243_10	LYG1	deCODE	SERPIN
15416_5	LYG2	deCODE	SERPIN
12428_2	LYPLAI	deCODE	SERPIN
9106_87	LYSMD	deCODE	SERPIN
4920_10	LYZ	deCODE	SERPIN
19338_3	LZIC	deCODE	SERPIN
13653_3	MACRC	deCODE	SERPIN
15313_2	MAD2L	deCODE	SERPIN
11258_4	MADCA	deCODE	SERPIN
19281_8	MAFG	deCODE	SERPIN
13610_9	MAGEA	deCODE	SERPIN
10485_5	MAGEA	deCODE	SERPIN
18192_6	MAGEA	deCODE	SERPIN
18205_1	MAGEA	deCODE	SERPIN
18326_5	MAGEA	deCODE	SERPIN
11456_2	MAGEB	deCODE	SETMA
14066_4	MAGI2	deCODE	SEZ6L_

16875_1	MAGOH	deCODE	SEZ6L2_
7071_23	MAN1B	deCODE	SF3B4_(
13427_6	MAN1C	deCODE	SFRP1_(
6382_17	MANBA	deCODE	SFRP4_(
8014_35	MANEA	deCODE	SFTPD_
11608_5	MAP1L(	deCODE	SH2D1A
3628_3_	MAP2K	deCODE	SH3BGI
6151_18	MAP2K	deCODE	SH3GL3
5242_37	MAP2K	deCODE	SH3GLI
12990_3	MAP3K	deCODE	SHBG_I
8954_30	MAP4K	deCODE	SHC1_P
8978_30	MAP4K	deCODE	SHH_Q1
3115_64	MAPK1	deCODE	SHMT1_
15418_2	MAPK1	deCODE	SIAE_Q
5004_69	MAPK1	deCODE	SIGLEC
5005_4_	MAPK1	deCODE	SIGLEC
5007_1_	MAPK1	deCODE	SIGLEC
2855_49	MAPK3	deCODE	SIGLEC
3825_18	MAPK8	deCODE	SIGLEC
3822_54	MAPKA	deCODE	SIGLEC
3821_28	MAPKA	deCODE	SIGLEC
8382_47	MAPKA	deCODE	SIRPA_
12469_1	MAPRE	deCODE	SIRPB1_
16856_7	MAPRE	deCODE	SIRT2_(
19175_1	MARCK	deCODE	SIRT5_(
9747_48	MARK3	deCODE	SIT1_Q9
18392_1	MAT2B	deCODE	SLAMF
3823_9_	MATK	deCODE	SLAMF(
7083_74	MATN4	deCODE	SLAMF7
13436_5	MAZ	deCODE	SLAMF8
14294_6	MBD1	deCODE	SLIT2_(
3891_56	MBD4	deCODE	SLITRK
7240_2_	MBTPS1	deCODE	SLITRK
8032_23	MCCD1	deCODE	SLMAP_
13934_3	MCF2L	deCODE	SMAD1_
10396_6	MCL1	deCODE	SMAD2_
9897_9_	MCM6	deCODE	SMAD3_
19372_7	MDGA2	deCODE	SMC3_(

15534_2	MDH2	deCODE	SMNDC
13228_7	MDM2	deCODE	SMOC1_
13229_2	MDM4	deCODE	SMOC2_
19108_5	MECP2	deCODE	SMPD1_
3892_21	MED1	deCODE	SMPDL3
14021_8	MED4	deCODE	SMS_P5
8933_84	MEF2C	deCODE	SMTN_I
19110_6	MEMO1	deCODE	SNAP25
18259_1	MEOX2	deCODE	SNAP29
15299_1	MESDC	deCODE	SNAPIN
12514_1	METTL	deCODE	SNCA_F
9470_15	METTL	deCODE	SNRPB2
5606_24	MFAP1	deCODE	SNX5_C
9294_45	MFAP2	deCODE	SOD1_P
11364_1	MFN1	deCODE	SOD2_P
5605_77	MFNG	deCODE	SOD3_P
3685_53	MFRP	deCODE	SORCS2
11587_5	MGA	deCODE	SORD_C
10571_1	MGAT1	deCODE	SORT1_
6909_40	MGAT2	deCODE	SOST_C
9392_43	MGAT4	deCODE	SOX2_P
7141_21	MGAT4	deCODE	SPACA5
7208_60	MGAT4	deCODE	SPARC_
6520_87	MGP	deCODE	SPARCL
2730_58	MICA	deCODE	SPINK1_
14249_6	MID2	deCODE	SPINK2_
7956_11	MINOS1	deCODE	SPINK4_
5586_66	MINPP1	deCODE	SPINK5_
13498_1	MIOX	deCODE	SPINK6_
6285_71	MLEC	deCODE	SPINK8_
17820_1	MLF1	deCODE	SPINT1_
13623_4	MLL2	deCODE	SPINT2_
11538_2	MLYCD	deCODE	SPINT3_
17783_9	MMAB	deCODE	SPOCK1
3627_71	MMEL1	deCODE	SPON1_
7225_51	MMGT1	deCODE	SPON2_
5002_76	MMP14	deCODE	SPP1_P1
5268_49	MMP16	deCODE	SPTLC1

9719_14	MMP16	deCODE	SRC_P1
2838_53	MMP17	deCODE	SRP14_
6425_87	MMP19	deCODE	SSB_P0
4160_49	MMP2	deCODE	ST13_P
15419_1	MMP20	deCODE	ST3GAI
9723_10	MMRN	deCODE	ST6GAI
9576_58	MNX1	deCODE	STAB2_
12426_1	MOB1A	deCODE	STAMB
19177_7	MOBKI	deCODE	STAT5E
19332_1	MOBKI	deCODE	STAU1_
14229_5	MOCS3	deCODE	STC1_P
14036_1	MPDZ	deCODE	STC2_C
12438_1	MPG	deCODE	STIP1_F
3473_78	MPL	deCODE	STK24_
13490_1	MPP6	deCODE	STOML
12732_1	MPP7	deCODE	STX1B_
19153_5	MPPED	deCODE	STX3_C
12686_1	MPST	deCODE	STX4_C
10615_1	MPZ	deCODE	STX6_C
16845_1	MPZL1	deCODE	STX7_C
9372_15	MPZL2	deCODE	STX8_C
7895_10	MRAP	deCODE	SUGT1_
10889_2	MRAP2	deCODE	SULT2A
18297_8	MRAS	deCODE	SUMF1_
3041_55	MRC2	deCODE	SUMF2_
11319_1	MRE11	deCODE	SUOX_I
19145_4	MREG	deCODE	SUSD1_
8021_59	MRPL1	deCODE	SV2A_C
19132_1	MRPL2	deCODE	SWAP7
8942_2_	MRPL2	deCODE	SYTL4_
7982_10	MRPL3	deCODE	TACSTI
13453_2	MRPL3	deCODE	TAGLN
6933_20	MRPL3	deCODE	TALDO
11135_5	MRPL5	deCODE	TANK_
12355_2	MRRF	deCODE	TARBP
8255_34	MRVI1	deCODE	TAX1BI
9739_4_	MSH2	deCODE	TBC1D
8080_24	MSMP	deCODE	TBCA_

5009_11	MSN	deCODE
16877_1	MSRB2	deCODE
7824_88	MSRB3	deCODE
2640_3_	MST1R	deCODE
9910_9_	MTAP	deCODE
19374_7	MTCP1	deCODE
9095_5_	MTFR1	deCODE
12575_3	MTHFD	deCODE
14107_1	MTHFS	deCODE
11167_6	MTMR1	deCODE
12536_4	MTMR6	deCODE
9028_5_	MTPAP	deCODE
11134_3	MTRF1L	deCODE
8839_4_	MTX2	deCODE
10623_1	MUC1	deCODE
9176_3_	MUC1	deCODE
10975_5	MUCL1	deCODE
9315_16	MUL1	deCODE
11547_8	MUSK	deCODE
19116_1	MUTED	deCODE
10030_8	MUTYH	deCODE
18422_4	MVD	deCODE
17460_5	MX1	deCODE
9035_2_	MXI1	deCODE
8005_1_	MXRA7	deCODE
11618_8	MYB	deCODE
10362_3	MYC	deCODE
19250_5	MYCBP	deCODE
13541_1	MYCT1	deCODE
19373_3	MYL12A	deCODE
19122_4	MYL12B	deCODE
19286_3	MYL5	deCODE
19296_5	MYL7	deCODE
12455_4	MYNN	deCODE
8843_34	MYRF	deCODE
11536_9	MYSM1	deCODE
9055_81	MYZAP	deCODE
9964_10	MYZAP	deCODE

TBCB_
TCL1A_
TCL1B_
TCN1_P
TCN2_P
TCP11_
TDGF1_
TDO2_P
TDP1_Q
TEK_Q0
TERF1_
TF_P02
TFF1_P0
TFF2_Q
TFF3_Q
TFPI_P1
TFPI2_P
TFRC_P
TG_P01
TGFB1_
TGFB2_
TGFBI_
TGFBR2
TGFBR3
TGM2_
THBS2_
THBS4_
THPO_P
THSD1_
THTPA_
TIE1_P
TIGAR_
TIGIT_
TIMD4_
TIMM8A
TIMP1_
TIMP2_
TIMP3_

14662_6	MZF1	deCODE	TIMP4_
11096_5	N6AMT	deCODE	TINAGI
7986_98	NAALA	deCODE	TK1_P0
17505_1	NAALA	deCODE	TLR1_Q
13933_2	NAB1	deCODE	TLR2_C
13680_3	NAB2	deCODE	TLR3_C
3854_24	NACA	deCODE	TLR4_C
18177_4	NAE1	deCODE	TMCO5
15509_2	NAGLU	deCODE	TMED1
11247_2	NAGS	deCODE	TMED4
18301_1	NAIF1	deCODE	TMEM1
2732_58	NANOG	deCODE	TMEM1
16863_4	NANP	deCODE	TMEM2
17802_4	NANS	deCODE	TMPRS
13636_2	NAP1L1	deCODE	TMPRS
13529_3	NAP1L2	deCODE	TMPRS
4292_5_	NAPA	deCODE	TMPRS
12655_3	NAPB	deCODE	TNC_P2
17773_2	NAPG	deCODE	TNF_P0
12632_1	NAT1	deCODE	TNF_P0
11120_4	NAT14	deCODE	TNF_P0
18261_3	NATD1	deCODE	TNF_P0
7696_3_	NBR1	deCODE	TNFAIP
17816_5	NCALD	deCODE	TNFRSF
17766_5	NCF1	deCODE	TNFRSF
14060_6	NCF4	deCODE	TNFRSF
5014_49	NCK1	deCODE	TNFRSF
13655_3	NCL	deCODE	TNFRSF
14614_4	NCMAP	deCODE	TNFRSF
14045_1	NCOA2	deCODE	TNFRSF
2734_49	NCR2	deCODE	TNFRSF
3003_29	NCR3	deCODE	TNFRSF
12730_3	NDC80	deCODE	TNFRSF
9765_4_	NDE1	deCODE	TNFRSF
6604_59	NDNF	deCODE	TNFRSF
18312_6	NDRG3	deCODE	TNFRSF
7934_11	NDRG4	deCODE	TNFRSF
7747_47	NDUFB	deCODE	TNFRSF

10677_9	NDUFB	deCODE	TNFRSI
9800_20	NDUFB	deCODE	TNFSF1
10584_7	NDUFS	deCODE	TNFSF1
7748_11	NDUFV	deCODE	TNFSF1
9900_36	NEFH	deCODE	TNFSF1
13109_8	NEGR1	deCODE	TNFSF1
7050_5_	NEGR1	deCODE	TNFSF8
14260_1	NET1	deCODE	TNNI3_
15298_1	NETO1	deCODE	TNR_Q
5639_49	NETO1	deCODE	TNXB_I
10562_4	NETO2	deCODE	TOP1_P
15426_5	NEU1	deCODE	TOR1AI
13604_2	NEURL	deCODE	TP53_P
13468_5	NEURL	deCODE	TP53I3_
11560_7	NFATC	deCODE	TPK1_Q
11154_3	NFE2L1	deCODE	TPMT_I
19209_6	NFKBIA	deCODE	TPP1_O
17387_2	NFKBIE	deCODE	TPPP2_
19123_6	NFKBID	deCODE	TPRKB_
11351_2	NHEJ1	deCODE	TPSAB1
13602_6	NHP2L1	deCODE	TPSG1_
12564_9	NIP7	deCODE	TPT1_P
12864_9	NIPAL4	deCODE	TRDMT
12738_4	NISCH	deCODE	TREM2_
17427_2	NLGN3	deCODE	TREML
8941_4_	NLGN3	deCODE	TREML
5357_60	NLGN4	deCODE	TRIAP1_
11661_1	NLRP1	deCODE	TSLP_Q
12821_6	NLRP10	deCODE	TST_Q1
12794_6	NLRP4	deCODE	TTN_Q
9321_40	NMB	deCODE	TXLNA_
4249_64	NME2	deCODE	TXN_P1
16809_1	NME4	deCODE	TXNDC
6406_3_	NMES1	deCODE	TXNDC
13988_6	NMRAL	deCODE	TXNRD
9503_1_	NMS	deCODE	TYMP_
17821_2	NMT2	deCODE	TYRO3_
8754_5_	NMU	deCODE	UBE2B_

9374_24	NNAT	deCODE	UBE2L6
19376_7	NNMT	deCODE	UGDH_
15692_3	NODAL	deCODE	ULBP2_
8778_3_	NOG	deCODE	UMOD_
18228_3	NOP16	deCODE	UNC5D_
8252_2_	NOTUM	deCODE	UROD_
2737_22	NOV	deCODE	UROS_I
12557_1	NOVA1	deCODE	USO1_C
5617_41	NPFF	deCODE	USP25_
9954_2_	NPIPB3	deCODE	USP28_
12993_2	NPLOC4	deCODE	USP8_P
6342_10	NPNT	deCODE	UXS1_C
5443_62	NPPA	deCODE	VAMP8_
6390_18	NPS	deCODE	VASN_C
9986_14	NPW	deCODE	VASP_F
9837_60	NQO1	deCODE	VAT1_C
9754_33	NQO2	deCODE	VAV3_C
5236_2_	NR1D1	deCODE	VCAM1
12885_4	NR1D2	deCODE	VCAN_
9016_12	NR1H2	deCODE	VEGFA_
8946_38	NR1H4	deCODE	VEGFB_
12931_1	NR3C2	deCODE	VEGFC_
8089_17	NR4A1	deCODE	VIM_P0
12444_3	NR5A2	deCODE	VIT_Q6
13464_8	NRAC	deCODE	VNN2_C
10531_1	NRAS	deCODE	VPS28_
12616_4	NRBP1	deCODE	VPS4B_
15446_2	NRG1	deCODE	VSIG10_
9178_30	NRG1	deCODE	VSIG2_
8060_7_	NRG2	deCODE	VSNL1_
10981_5	NRG3	deCODE	VSTM2I
14139_1	NRG4	deCODE	VTA1_C
18237_2	NRIP3	deCODE	VTI1A_
16853_5	NRK1	deCODE	VWA1_
11654_7	NRSN1	deCODE	VWC2_
5110_84	NRXN1	deCODE	VWC2L
8971_9_	NRXN1	deCODE	VWF_P(
8876_51	NRXN2	deCODE	WARS_

8038_41	NSDHL	deCODE	WFDC1_
13992_1	NSF	deCODE	WFDC2_
11288_2	NT5C2	deCODE	WFIKKI
19135_5	NT5C3L	deCODE	WFIKKI
9126_17	NT5DC3	deCODE	WIF1_Q
17856_2	NT5M	deCODE	XCL1_P
10907_1	NTM	deCODE	XG_P55
8428_10	NTM	deCODE	XRCC4_
12454_1	NTMT1	deCODE	YES1_P
6649_51	NTN1	deCODE	YWHAG
3327_27	NTN4	deCODE	YY1_P2
5637_81	NTNG1	deCODE	ZNF174_
3477_63	NTRK1	deCODE	ZNF75D
7857_22	NTS	deCODE	ZP4_Q1
10451_1	NUCB1	deCODE	16561_9
4254_6_	NUDCD	deCODE	19325_2
19206_2	NUDT1	deCODE	3341_33
13947_3	NUDT12	deCODE	9253_52
12497_2	NUDT16	deCODE	17782_2
12796_4	NUDT3	deCODE	17333_2
9482_11	NUDT9	deCODE	17403_1
9819_11	NUP210	deCODE	3280_49
9606_4_	NUP210	deCODE	10714_7
12772_8	NUP98	deCODE	2805_6_
19219_7	NUTF2	deCODE	15553_2
12453_1	NXF1	deCODE	17675_1
9511_61	NXPH2	deCODE	3858_5_
9942_2_	NXT1	deCODE	3232_28
19255_1	NXT2	deCODE	5742_14
6414_8_	OAF	deCODE	10507_1
10361_2	OAS1	deCODE	15301_2
9893_27	OAZ1	deCODE	9844_13
6526_77	OBP2A	deCODE	16318_1
16802_3	OCIAD1	deCODE	3343_1_
10011_6	OCRL	deCODE	4420_7_
13689_2	ODC1	deCODE	15455_4
6296_36	OIT3	deCODE	9956_7_
12659_1	OLA1	deCODE	7933_75

5703_26	OLFM1	deCODE	7049_2_
19377_1	OLFM2	deCODE	3795_6_
10643_1	OLFM3	deCODE	3174_2_
11143_3	OLFM3	deCODE	3175_51
8815_1_	OLFML	deCODE	4533_76
8660_5_	OLFML	deCODE	2809_25
7106_37	OOSP2	deCODE	6379_62
7736_28	OPALIN	deCODE	4546_27
15622_1	OPCML	deCODE	6409_57
18195_3	ORAOV	deCODE	9834_62
12389_4	ORC6L	deCODE	8325_37
12878_6	OSBPL1	deCODE	3554_24
16899_5	OSBPL9	deCODE	14115_3
19118_4	OSTF1	deCODE	7922_5_
19353_2	OSTM1	deCODE	4763_31
15431_3	OTC	deCODE	5792_8_
15432_1	OTOR	deCODE	4125_52
12493_4	OTUB2	deCODE	4959_2_
12480_9	OTUD5	deCODE	17342_1
17697_2	OVCA2	deCODE	15483_3
18271_4	OXSM	deCODE	2813_11
7233_73	P2RX6	deCODE	3484_60
11645_9	P4HA1	deCODE	11633_8
11348_1	P4HA2	deCODE	3581_53
4258_15	PA2G4	deCODE	9025_5_
10447_1	PABPC3	deCODE	17347_8
12603_8	PABPC4	deCODE	2849_49
8921_13	PABPN1	deCODE	18871_2
16322_1	PACAP	deCODE	9522_3_
12676_1	PACSIN	deCODE	5012_67
14674_6	PACSIN	deCODE	11368_3
19158_1	PAF	deCODE	16606_8
2642_4_	PAFAH1	deCODE	16081_3
9841_19	PAICS	deCODE	18397_5
12430_7	PAIP1	deCODE	14685_1
3387_1_	PAK3	deCODE	5360_9_
3827_22	PAK6	deCODE	5451_1_
3388_58	PAK7	deCODE	11265_8

12658_7	PANK3	deCODE	18381_1
8011_96	PAPL	deCODE	11480_1
11254_1	PAPLN	deCODE	17792_1
8343_22	PAPOL(	deCODE	17441_4
5756_66	PAPPA2	deCODE	15453_3
14007_2	PAPSS1	deCODE	9979_13
13013_4	PARK2	deCODE	14134_4
12882_7	PARP11	deCODE	4322_28
7881_24	PARP16	deCODE	7918_11
13434_1	PARVA	deCODE	18917_5
8386_11	PATE1	deCODE	15556_4
8065_24	PATE4	deCODE	4874_3_
9565_6_	PAWR	deCODE	2811_27
12398_1	PAX4	deCODE	2602_2_
12894_3	PAXIP1	deCODE	11142_1
12518_2	PBRM1	deCODE	9092_33
13990_1	PC	deCODE	10391_1
6899_37	PCBD2	deCODE	3796_79
11458_3	PCBP1	deCODE	6371_50
8780_2_	PCDH1(	deCODE	7624_19
9018_38	PCDH1(	deCODE	18404_2
6280_11	PCDH8	deCODE	15457_1
7157_22	PCDHA	deCODE	4960_72
8758_2_	PCDHA	deCODE	13605_1
7193_98	PCDHA	deCODE	17513_1
9361_7_	PCDHA	deCODE	13700_1
9941_70	PCDHB	deCODE	4961_17
9963_19	PCDHB	deCODE	17163_1
10748_2	PCDHB	deCODE	17164_1
8071_11	PCDHB	deCODE	14158_1
6457_50	PCDHG	deCODE	15486_1
6321_65	PCDHG	deCODE	12714_3
6938_21	PCDHG	deCODE	2474_54
7175_4_	PCDHG	deCODE	9849_13
11872_9	PCDHG	deCODE	7210_25
7859_21	PCDHG	deCODE	2750_3_
7983_1_	PCDHG	deCODE	7127_3_
18182_2	PCK1	deCODE	17685_9

18160_2	PCMT1	deCODE	2797_56
17769_2	PCNP	deCODE	15364_1
6081_52	PCOLCI	deCODE	4712_28
13388_5	PCSK1	deCODE	2418_55
9391_60	PCSK1N	deCODE	12370_3
15440_5	PCSK2	deCODE	8288_27
6117_4_	PCSK2	deCODE	9506_10
16867_7	PCTK1	deCODE	11510_3
6431_68	PCYOX	deCODE	10445_2
5599_88	PCYOX	deCODE	3171_57
17202_3	PDCD4	deCODE	10088_3
18174_7	PDCD6I	deCODE	12583_7
5252_33	PDE11A	deCODE	2970_60
5253_1_	PDE1A	deCODE	12425_1
5246_64	PDE2A	deCODE	13488_3
5254_69	PDE3A	deCODE	5867_60
18918_8	PDE4A	deCODE	17752_2
13491_4	PDE6D	deCODE	9815_5_
5178_5_	PDE7A	deCODE	11955_1
5201_50	PDE9A	deCODE	11333_8
17140_5	PDGFD	deCODE	12807_8
9341_1_	PDGFD	deCODE	14748_3
9713_67	PDGFRI	deCODE	13976_9
18319_7	PDHX	deCODE	9061_3_
5650_9_	PDIA6	deCODE	3875_62
7092_7_	PDILT	deCODE	18407_3
5227_60	PDK1	deCODE	3583_54
12651_2	PDK2	deCODE	3172_28
11428_3	PDLIM1	deCODE	7970_31
12387_7	PDLIM4	deCODE	2939_10
4460_8_	PDPK1	deCODE	5748_20
11098_1	PDXK	deCODE	3212_30
3897_61	PDXP	deCODE	5452_71
19638_9	PDYN	deCODE	9474_22
6219_14	PDZD11	deCODE	6451_64
12391_2	PDZD7	deCODE	18313_4
18833_7	PEA15	deCODE	19233_7
19272_9	PEF1	deCODE	13392_1

12702_1	PELI2	deCODE	7218_87
12431_1	PELO	deCODE	8990_42
4267_81	PES1	deCODE	9933_49
19303_6	PET117	deCODE	15499_1
8300_82	PEX14	deCODE	16825_2
19366_8	PEX26	deCODE	10574_1
5915_58	PEX5	deCODE	14713_4
5663_18	PF4V1	deCODE	3485_28
19242_2	PFDN1	deCODE	6897_38
4271_75	PFDN5	deCODE	13381_4
12456_5	PFKFB3	deCODE	12451_6
17384_1	PFKM	deCODE	10078_5
16923_2	PFN2	deCODE	12844_1
3896_5_	PGAM1	deCODE	19331_1
15524_3	PGAM2	deCODE	8811_24
5020_50	PGK1	deCODE	19227_1
13936_2	PGK2	deCODE	2816_50
17799_9	PGLS	deCODE	3461_58
10561_5	PGLYRI	deCODE	19365_1
9173_21	PGM1	deCODE	15514_2
18235_1	PGP	deCODE	3412_7_
7863_50	PGRMC	deCODE	4423_77
10506_5	PGRMC	deCODE	17451_1
8681_93	PGRMC	deCODE	14047_7
18921_3	PHEX	deCODE	2421_7_
11544_3	PHF3	deCODE	13032_1
15548_3	PHGDH	deCODE	11067_1
16882_2	PHPT1	deCODE	3284_75
5745_64	PI15	deCODE	14226_1
14114_1	PIANP	deCODE	5798_3_
9599_6_	PIANP	deCODE	10046_5
13513_1	PIAS3	deCODE	14250_1
10342_5	PIAS4	deCODE	19225_1
11838_1	PIEZO1	deCODE	17148_7
14028_2	PIK3C2.	deCODE	3587_53
3391_10	PIK3CG	deCODE	15667_3
8863_3_	PIK3R1	deCODE	6326_20
5359_65	PIM1	deCODE	8459_10

15474_7	PIN1	deCODE	15368_3
12718_4	PIN4	deCODE	7835_2_
6060_2_	PIP	deCODE	4328_2_
12697_3	PIP4K2A	deCODE	15370_5
18232_4	PIP4K2I	deCODE	8404_10
13634_2	PIR	deCODE	16302_1
9934_29	PITPNA	deCODE	15367_3
12484_6	PITPNB	deCODE	11607_1
12793_4	PIWIL1	deCODE	10074_1
8664_36	PKDCC	deCODE	15372_4
4240_31	PKM2	deCODE	12748_6
12562_1	PKN1	deCODE	9790_28
14067_6	PKP2	deCODE	3585_54
9518_95	PLA1A	deCODE	4535_50
9380_2_	PLA2G1	deCODE	8832_55
7865_12	PLA2G1	deCODE	13088_3
8850_5_	PLA2G2	deCODE	15644_1
16298_8	PLA2G2	deCODE	16308_1
2447_7_	PLA2G2	deCODE	8869_5_
2449_1_	PLA2G5	deCODE	5735_54
10916_4	PLA2R1	deCODE	4967_1_
6315_58	PLBD1	deCODE	15670_1
6536_54	PLBD2	deCODE	6304_8_
4563_61	PLCG1	deCODE	7810_20
10070_2	PLCG2	deCODE	16605_2
10948_1	PLD3	deCODE	3285_23
8081_55	PLD5	deCODE	9348_1_
7875_86	PLEK	deCODE	8840_61
12459_1	PLEKHA	deCODE	3186_2_
10939_1	PLEKHA	deCODE	2683_1_
12731_1	PLEKHA	deCODE	2754_50
3394_81	PLK1	deCODE	4480_59
6923_1_	PLOD2	deCODE	4900_8_
10612_1	PLOD3	deCODE	5803_24
12737_1	PLS1	deCODE	7155_46
8565_16	PLVAP	deCODE	2381_52
9005_16	PLXNA1	deCODE	2851_63
4564_2_	PLXNC1	deCODE	2888_49

19561_2	PLXND	deCODE	3060_43
6472_40	PMEL	deCODE	4969_2_
6936_7_	PMEPA	deCODE	8859_51
17408_2	PMM1	deCODE	19347_3
15422_1	PMP2	deCODE	3420_21
11312_4	PMS2	deCODE	4970_55
13657_2	PNKP	deCODE	3799_11
17204_1	PNMT	deCODE	14069_6
15434_5	PNOC	deCODE	8791_15
15435_4	PNP	deCODE	3352_80
9802_27	PNPLA2	deCODE	3356_50
17804_1	PNPO	deCODE	11390_2
5634_39	POFUT1	deCODE	3798_71
6467_65	POGLU	deCODE	12432_2
19159_9	POLD4	deCODE	9918_23
11562_9	POLE2	deCODE	16803_4
19170_2	POLE3	deCODE	17170_1
10022_2	POLH	deCODE	12534_1
13536_5	POLI	deCODE	8924_55
12851_5	POLM	deCODE	18860_2
12939_1	POLR1C	deCODE	6480_1_
19199_3	POLR2J	deCODE	4968_50
19134_6	POLR2K	deCODE	12385_4
11444_4	POLR3F	deCODE	12808_1
8253_2_	POMGN	deCODE	5340_24
6359_50	POMGN	deCODE	4904_7_
10582_3	POMK	deCODE	3593_72
2731_29	POR	deCODE	18158_4
7960_53	POTEG	deCODE	19291_2
7967_38	POTEM	deCODE	3488_64
11715_1	POU2F1	deCODE	9313_27
5021_13	PPA1	deCODE	5688_65
18307_7	PPA2	deCODE	10086_3
13548_5	PPAPDC	deCODE	15300_6
12954_7	PPARA	deCODE	12399_1
11825_2	PPARG(	deCODE	3234_23
17843_3	PPCS	deCODE	5301_7_
13975_5	PPFIA1	deCODE	4144_13

3844_2_	PPIA	deCODE	2900_53
18819_2	PPIC	deCODE	18289_1
5202_4_	PPID	deCODE	4913_78
18449_3	PPIG	deCODE	3519_3_
12449_1	PPIH	deCODE	3044_3_
9884_8_	PPIL1	deCODE	4922_13
14314_6	PPIL2	deCODE	2578_67
11315_1	PPM1D	deCODE	2468_62
7187_3_	PPM1L	deCODE	2516_57
17706_4	PPP1R1	deCODE	3508_78
12768_3	PPP1R3	deCODE	2913_1_
13039_1	PPP1R8	deCODE	3028_36
12621_5	PPP2R1	deCODE	4128_27
13665_3	PPP2R3	deCODE	2705_5_
18866_8	PPP3R2	deCODE	9168_31
7939_1_	PQLC2I	deCODE	2192_63
14197_2	PRDM1	deCODE	2890_59
12779_3	PRDM4	deCODE	3040_59
7789_18	PRDX4	deCODE	5480_49
14273_1	PREP	deCODE	4886_3_
13591_3	PRIM1	deCODE	13748_4
3466_8_	PRKAC	deCODE	13068_1
12479_5	PRKAR	deCODE	12569_2
2644_11	PRKCA	deCODE	3290_50
5475_10	PRKCB	deCODE	16914_1
5476_66	PRKCG	deCODE	8969_49
3379_29	PRKCI	deCODE	17677_4
5687_5_	PRKCSI	deCODE	5028_59
2645_54	PRKCZ	deCODE	6915_2_
6543_18	PRLH	deCODE	13116_2
5114_65	PRLR	deCODE	7100_31
12696_1	PRMT3	deCODE	5112_73
6545_58	PRNP	deCODE	5103_30
10754_1	PROK2	deCODE	3361_26
6530_63	PROL1	deCODE	3029_52
9012_1_	PRPF6	deCODE	5062_60
9581_4_	PRPF6	deCODE	5063_12
11381_5	PRPS1	deCODE	15491_2

18329_4	PRPS2	deCODE	5412_53
9478_69	PRPSAP	deCODE	5060_62
19150_2	PRPSAP	deCODE	5630_48
10621_2	PRR16	deCODE	5066_13
9607_39	PRR27	deCODE	10798_4
8306_54	PRRG1	deCODE	5623_11
9008_6_	PRRG1	deCODE	3166_92
9579_59	PRRG4	deCODE	2973_15
3049_61	PRSS1	deCODE	11513_9
18864_7	PRSS3	deCODE	18870_1
9983_97	PRSS35	deCODE	8069_85
5653_23	PRSS37	deCODE	7924_7_
8351_17	PRSS57	deCODE	3143_3_
18342_2	PSAT1	deCODE	3534_14
13055_5	PSD	deCODE	17682_1
9118_7_	PSD2	deCODE	3292_75
7846_44	PSG11	deCODE	14065_1
6405_74	PSG2	deCODE	5596_75
6444_15	PSG3	deCODE	5069_9_
5649_83	PSG4	deCODE	10938_1
9314_9_	PSG5	deCODE	11514_1
6456_17	PSG6	deCODE	3293_2_
5651_50	PSG7	deCODE	9190_7_
6238_55	PSG8	deCODE	16618_7
9335_28	PSG9	deCODE	12008_3
4280_47	PSMA2	deCODE	5807_77
18926_7	PSMA3	deCODE	7009_8_
14099_2	PSMA4	deCODE	6974_6_
18925_2	PSMA5	deCODE	8748_45
3860_7_	PSMA6	deCODE	6351_55
12460_1	PSMA7	deCODE	13726_4
12612_3	PSMB1	deCODE	5065_8_
19252_6	PSMB2	deCODE	5337_64
18339_2	PSMB3	deCODE	15462_2
18340_2	PSMB4	deCODE	14136_2
12580_7	PSMB5	deCODE	10539_3
10530_8	PSMB6	deCODE	11548_8
18942_1	PSMB9	deCODE	3879_50

18385_4	PSMD1(	deCODE	3629_60
13572_4	PSMD1:	deCODE	16818_2
13568_3	PSMD4	deCODE	8589_13
3898_5_	PSMD7	deCODE	18429_1
5204_13	PSME3	deCODE	5410_53
19168_7	PSTPIP1	deCODE	11215_6
13517_3	PTCHD:	deCODE	16613_3
17154_2	PTGES3	deCODE	3797_1_
12727_7	PTGFRN	deCODE	2643_57
18943_4	PTGR2	deCODE	2819_23
3761_4_	PTGS2	deCODE	16312_4
7257_18	PTH2	deCODE	8372_29
2962_50	PTHLH	deCODE	9962_1_
4986_59	PTK2	deCODE	18291_8
8918_64	PTK2B	deCODE	12376_8
11395_5	PTK2B	deCODE	4962_52
3832_51	PTK6	deCODE	4541_49
17812_2	PTP4A1	deCODE	7085_81
11699_1	PTP4A2	deCODE	7048_4_
3397_7_	PTPN11	deCODE	10453_7
12664_1	PTPN13	deCODE	7204_1_
3401_8_	PTPN2	deCODE	8999_19
14254_2	PTPN4	deCODE	18873_8
14688_6	PTPN7	deCODE	18874_6
9296_15	PTPRD	deCODE	15675_3
8250_2_	PTPRJ	deCODE	7140_1_
8337_65	PTPRU	deCODE	15487_1
16887_2	PTRH2	deCODE	13078_3
10575_3	PUF60	deCODE	17410_5
12537_8	PURA	deCODE	4129_72
18300_3	PURB	deCODE	3294_55
11201_1	PUS1	deCODE	2946_52
6464_40	PVRIG	deCODE	4159_13
8803_61	PVRL1	deCODE	15584_9
6245_4_	PVRL2	deCODE	6471_53
13557_3	PVRL3	deCODE	16055_3
5734_13	PVRL4	deCODE	2567_5_
13463_1	PXDN	deCODE	2960_66

18170_4	PYCR1	deCODE	14056_4
18328_3	PYCR2	deCODE	6257_56
11441_1	PYGL	deCODE	13460_4
7253_6_	QKI	deCODE	11270_1
8866_53	QPCTL	deCODE	19328_5
17789_1	QPRT	deCODE	8476_11
6463_59	QRFP	deCODE	8235_48
8397_14	QSOX2	deCODE	11104_1
17744_3	RAB11A	deCODE	3600_2_
19282_3	RAB13	deCODE	8958_51
14283_1	RAB14	deCODE	12458_7
11118_1	RAB17	deCODE	3362_61
12945_3	RAB18	deCODE	6086_15
19223_6	RAB1A	deCODE	5717_2_
19278_1	RAB1B	deCODE	19383_1
17514_4	RAB21	deCODE	12724_8
12408_3	RAB22A	deCODE	7221_56
6997_32	RAB26	deCODE	3800_71
9504_19	RAB27A	deCODE	15542_1
16857_2	RAB2A	deCODE	11094_1
13597_2	RAB31	deCODE	10955_4
17778_1	RAB32	deCODE	2966_65
13514_1	RAB35	deCODE	4500_50
17516_7	RAB3A	deCODE	11187_1
16847_3	RAB3B	deCODE	4332_6_
19586_8	RAB3C	deCODE	8242_9_
19379_1	RAB3D	deCODE	5701_81
16883_5	RAB4A	deCODE	8853_2_
17205_2	RAB5A	deCODE	9094_5_
19222_1	RAB5B	deCODE	7752_31
14287_6	RAB5C	deCODE	10781_1
12409_9	RAB7B	deCODE	3030_3_
18483_3	RABIF	deCODE	6911_10
2870_29	RAC1	deCODE	3603_60
18950_1	RAC2	deCODE	10600_2
13587_1	RACGA	deCODE	12475_4
12670_1	RAD1	deCODE	11659_3
10058_1	RAD23A	deCODE	10440_2

12554_1	RAD51I	deCODE	9585_80
18927_1	RAET1(	deCODE	17196_5
10589_7	RAET1I	deCODE	5749_53
10001_7	RAF1	deCODE	15521_4
11311_7	RAG1	deCODE	18882_7
14332_3	RALA	deCODE	6291_55
15436_4	RAMP1	deCODE	19257_1
15437_1	RAMP3	deCODE	4542_24
4282_3_	RAN	deCODE	5456_59
14037_1	RANBP:	deCODE	6609_22
13735_1	RAP1G/	deCODE	15465_7
14106_4	RAP1GI	deCODE	3489_9_
9885_41	RAP2A	deCODE	2974_61
9059_14	RAPGEI	deCODE	3296_92
10961_1	RARRE:	deCODE	3298_52
5024_67	RB1	deCODE	3299_29
15331_4	RBBP4	deCODE	6965_19
13631_1	RBBP5	deCODE	7227_75
9887_40	RBBP6	deCODE	8974_17
10064_1	RBBP9	deCODE	2201_17
11462_8	RBFOX:	deCODE	13484_6
12879_5	RBL1	deCODE	11140_5
13565_2	RBL2	deCODE	10702_1
12980_3	RBM22	deCODE	15569_1
11590_5	RBM23	deCODE	18875_1
11380_8	RBM24	deCODE	18880_8
12747_8	RBM3	deCODE	10511_1
4284_18	RBM39	deCODE	11196_3
13958_5	RBM46	deCODE	15466_3
15633_6	RBP4	deCODE	6631_17
13465_5	RCAN1	deCODE	5457_5_
17785_1	RCL	deCODE	12509_1
9322_15	RCN1	deCODE	17683_2
16858_3	RCN3	deCODE	8043_15
14334_3	RCVRN	deCODE	18382_1
12881_1	RDH16	deCODE	19310_8
18373_1	RDX	deCODE	7887_57
11431_2	RECQL	deCODE	9276_7_

13399_3	RELL1	deCODE		9267_2_
6600_70	RELL2	deCODE		15375_4
13554_7	REPIN1	deCODE		3518_54
17822_5	RERG	deCODE		5343_74
13603_7	RFESD	deCODE		15321_8
14186_1	RFFL	deCODE		9416_77
13059_3	RFK	deCODE		18241_1
7203_12	RFNG	deCODE		9394_19
12726_3	RFXAP	deCODE		8891_7_
18945_1	RGS1	deCODE		6255_74
13982_3	RGS18	deCODE		19556_1
12713_3	RGS19	deCODE		15570_9
19381_7	RGS21	deCODE		15322_3
12827_3	RGS3	deCODE		9357_4_
17855_2	RGS4	deCODE		7628_40
16850_5	RGS5	deCODE		19637_9
12538_1	RGS7	deCODE		5614_44
9855_10	RHOA	deCODE		6039_24
18166_4	RHOB	deCODE		15492_1
12442_4	RHOD	deCODE		8699_43
12540_2	RHOG	deCODE		9053_16
11439_8	RHPN2	deCODE		9282_12
11228_3	RIC3	deCODE		3187_52
8970_9_	RIPK2	deCODE		9877_28
8993_15	RIPK2	deCODE		14747_9
9767_22	RIPPLY	deCODE		17738_7
12936_3	RLBP1	deCODE		5632_6_
6027_31	RLN3	deCODE		7968_15
7096_30	RMDN1	deCODE		17751_6
9290_8_	RMDN3	deCODE		10000_2
13926_1	RMI1	deCODE		12366_1
6424_2_	RNASE1	deCODE		9207_60
8394_56	RNASE2	deCODE		12735_3
8470_21	RNASEI	deCODE		3738_54
13574_5	RND1	deCODE		13682_4
15439_2	RND3	deCODE		4697_59
9957_9_	RNF114	deCODE		10438_1
11160_5	RNF122	deCODE		10512_1

6510_56	RNF128	deCODE	11137_4
8633_18	RNF128	deCODE	4840_73
8087_25	RNF13	deCODE	2719_3_
11401_1	RNF146	deCODE	11289_3
7742_11	RNF148	deCODE	3427_63
9955_40	RNF148	deCODE	5224_20
9774_59	RNF150	deCODE	8951_16
7758_21	RNF215	deCODE	9171_11
9064_12	RNF215	deCODE	5459_33
9583_17	RNF24	deCODE	2609_59
13386_2	RNF34	deCODE	3803_10
14663_4	RNF8	deCODE	14711_2
12847_2	RNGTT	deCODE	3302_58
12814_1	RNPC3	deCODE	2889_37
12386_1	RNPEP	deCODE	15467_1
15329_1	ROBLD	deCODE	3022_4_
5117_14	ROBO3	deCODE	5671_1_
13654_1	ROCK2	deCODE	5626_20
7861_9_	ROR2	deCODE	9229_9_
12483_6	RORB	deCODE	8007_19
11827_7	RORC	deCODE	3178_5_
9032_30	ROS1	deCODE	5508_62
12333_8	RPIA	deCODE	15376_1
19183_1	RPL12	deCODE	9212_22
12478_1	RPL30	deCODE	8465_52
10949_5	RPLP2	deCODE	9275_2_
10490_3	RPN1	deCODE	9264_11
6458_6_	RPN1	deCODE	3181_50
17511_1	RPP30	deCODE	3364_76
13515_8	RPRD1A	deCODE	4971_1_
19126_4	RPRD1B	deCODE	7943_16
19166_1	RPS19	deCODE	2827_23
2846_24	RPS27A	deCODE	11204_8
4474_19	RPS27A	deCODE	2985_35
5026_66	RPS3	deCODE	4141_79
5484_63	RPS3A	deCODE	3038_9_
9758_17	RPS4X	deCODE	3516_60
19195_8	RPS5	deCODE	3487_32

12329_2	RPS6KA	deCODE	5730_60
3469_74	RPS6KA	deCODE	2436_49
12688_1	RPS6KA	deCODE	9495_10
15608_5	RPS6KB	deCODE	2979_8_
8962_48	RPS6KB	deCODE	3495_15
3864_5_	RPS7	deCODE	3447_64
19623_2	RPSA	deCODE	9188_11
8975_26	RQCD1	deCODE	11593_2
12548_7	RRAGC	deCODE	11287_1
12361_1	RRAS2	deCODE	19143_3
9913_4_	RRBP1	deCODE	8402_22
11360_3	RRM1	deCODE	8369_10
6497_10	RS1	deCODE	4355_13
4566_24	RSPO2	deCODE	11615_1
8409_3_	RSPO2	deCODE	16919_1
8464_31	RSPO4	deCODE	4978_54
9050_17	RSRP1	deCODE	2666_53
7741_11	RTF1	deCODE	17756_6
18315_3	RTP4	deCODE	5879_51
11425_3	RUFY1	deCODE	18311_4
11138_1	RUNX3	deCODE	4314_12
14135_3	RXFP1	deCODE	17366_6
5583_62	RYK	deCODE	19511_8
15318_7	S100A1(	deCODE	17758_7
11969_5	S100A2	deCODE	19392_6
15303_6	S100A5	deCODE	3538_26
13090_1	S100A6	deCODE	4122_12
7916_10	S100A7	deCODE	19237_1
5339_49	S100A9	deCODE	13984_2
18928_1	S100Z	deCODE	12382_2
15515_2	SAA1	deCODE	12687_2
18832_6	SAA2	deCODE	19579_5
11303_7	SAMHD	deCODE	5679_16
12888_1	SAP30	deCODE	5763_67
17726_3	SAR1A	deCODE	11144_1
18178_1	SARS	deCODE	9306_7_
12348_4	SARS2	deCODE	13397_8
10081_1	SATB2	deCODE	19287_5

6594_64	SAYSD1	deCODE	9444_70
3866_7_	SBDS	deCODE	9856_22
13509_5	SCAMP	deCODE	3122_6_
17800_3	SCAND	deCODE	3535_84
11292_1	SCARA1	deCODE	3607_71
15358_2	SCG5	deCODE	3365_7_
6508_68	SCGB1I	deCODE	16309_3
5001_6_	SCGB2A	deCODE	8070_88
10510_6	SCIMP	deCODE	6496_60
12604_1	SCMH1	deCODE	8380_24
7853_19	SCO1	deCODE	5349_69
16851_5	SCO2	deCODE	3305_6_
14175_7	SCP2D1	deCODE	11582_6
8989_40	SCUBE1	deCODE	9744_13
16773_2	SCUBE3	deCODE	3852_19
16612_2	SDC3	deCODE	8053_16
17806_6	SDCBP	deCODE	11438_6
19261_1	SDCBP2	deCODE	11606_2
15333_1	SDF2	deCODE	9769_48
6990_44	SDF2L1	deCODE	11442_1
6420_4_	SDHAF2	deCODE	7997_11
17777_3	SDSL	deCODE	14002_1
14689_3	SEC13	deCODE	16831_7
13713_1	SEC22A	deCODE	14246_5
7878_2_	SEC61B	deCODE	19578_1
8872_1_	SEC61G	deCODE	8794_13
13093_6	SECTM	deCODE	8327_26
9082_25	SEL1L2	deCODE	7890_68
17319_1	SELH	deCODE	15460_9
15336_7	SELM	deCODE	8346_9_
3222_11	SEMA3A	deCODE	4979_34
5667_3_	SEMA3I	deCODE	13943_3
6448_36	SEMA3(	deCODE	7084_1_
5363_51	SEMA3I	deCODE	13126_5
16915_1	SEMA4A	deCODE	9175_48
9805_51	SEMA4I	deCODE	9484_75
9932_49	SEMA4I	deCODE	16317_2
13132_1	SEMA5A	deCODE	19194_9

7945_10	SEMA6A	deCODE	19504_2
5121_3_	SEMA6I	deCODE	6525_17
7115_5_	SEMG1	deCODE	3480_7_
6373_54	SEMG2	deCODE	9995_6_
12626_6	SENP7	deCODE	14331_2
14083_2	SEPHS1	deCODE	12867_4
17357_3	SEPSEC	deCODE	19323_1
12690_3	SEPT10	deCODE	10851_7
12620_3	SEPT11	deCODE	8767_44
18316_7	SEPT3	deCODE	17787_1
17693_2	SEPT6	deCODE	12859_3
18310_2	SEPW1	deCODE	3366_51
8985_13	SERAC1	deCODE	3083_71
7842_52	SERF1A	deCODE	2977_7_
18274_2	SERF2	deCODE	7782_34
13119_2	SERPIN	deCODE	12415_1
18841_1	SERPIN	deCODE	9360_33
15444_4	SERPIN	deCODE	6495_14
5364_7_	SET	deCODE	17826_3
12647_5	SETD2	deCODE	10830_5
12777_1	SF1	deCODE	8480_29
4829_43	SFN	deCODE	19616_1
7751_12	SFRP2	deCODE	2614_28
10580_1	SFTA2	deCODE	14131_3
10672_7	SFTPB	deCODE	8772_5_
13729_2	SFXN5	deCODE	5509_7_
10042_8	SGK3	deCODE	12338_2
3868_8_	SGTA	deCODE	19567_1
18837_9	SGTB	deCODE	2677_1_
18840_2	SH2D1B	deCODE	9901_28
12704_2	SH2D3C	deCODE	12813_1
12693_2	SH3BGI	deCODE	9850_38
17490_4	SH3BGI	deCODE	14675_2
7769_29	SH3BP2	deCODE	19263_1
12499_1	SH3GL2	deCODE	17372_5
16859_1	SH3GLI	deCODE	13991_4
13240_1	SHANK	deCODE	2612_5_
13242_1	SHANK	deCODE	5888_29

14074_2	SHC2	deCODE	9757_29
11692_2	SHC4	deCODE	5656_53
10449_3	SHISA2	deCODE	4908_6_
12691_4	SHMT2	deCODE	11105_1
8326_63	SIGIRR	deCODE	10339_4
8913_22	SIGLEC	deCODE	16616_1
10037_9	SIGLEC	deCODE	13422_6
8352_26	SIGLEC	deCODE	15558_6
8248_22	SIGLEC	deCODE	16892_2
5669_26	SIRPB2	deCODE	6556_5_
9241_40	SIRPG	deCODE	15579_2
17495_1	SIRT3	deCODE	4435_66
14670_1	SKIL	deCODE	17734_1
3902_21	SKP1	deCODE	18887_7
13430_5	SLC14A	deCODE	4437_56
12818_1	SLC14A	deCODE	8932_1_
9049_2_	SLC16A	deCODE	3431_54
9969_8_	SLC22A	deCODE	5076_53
5280_68	SLC25A	deCODE	4834_61
13502_2	SLC26A	deCODE	16288_1
9127_4_	SLC26A	deCODE	15530_3
13979_3	SLC26A	deCODE	5078_82
9045_3_	SLC27A	deCODE	5813_58
9081_39	SLC30A	deCODE	2616_23
9594_30	SLC30A	deCODE	2617_56
13501_1	SLC35G	deCODE	2618_10
9000_17	SLC3A1	deCODE	12585_3
10430_3	SLC3A2	deCODE	4956_2_
12511_8	SLC41A	deCODE	8631_13
12798_4	SLC4A8	deCODE	11229_1
12826_5	SLC5A5	deCODE	13728_1
13691_1	SLC5A8	deCODE	6064_4_
13053_6	SLC6A1	deCODE	7841_84
13056_1	SLC6A1	deCODE	3805_16
11653_6	SLC6A9	deCODE	14019_7
11669_3	SLCO5A	deCODE	9753_17
15634_1	SLIT1	deCODE	3077_66
18931_4	SLIT3	deCODE	4878_3_

10565_1	SLITRK	deCODE	2190_55
7139_14	SLITRK	deCODE	5658_64
4568_17	SLITRK	deCODE	4157_2_
4413_3_	SLPI	deCODE	5316_54
8320_5_	SLTM	deCODE	4931_59
12022_1	SMAD4	deCODE	3184_25
11649_3	SMAP1	deCODE	4876_32
11180_1	SMARC	deCODE	5307_12
7925_18	SMCO2	deCODE	11516_7
10866_6	SMEK1	deCODE	15385_1
9379_24	SMIM1(	deCODE	5437_63
13452_1	SMIM1:	deCODE	15386_7
7108_7_	SMIM2‹	deCODE	4985_11
8888_33	SMIM9	deCODE	6450_8_
7838_27	SMR3A	deCODE	18194_1
11557_3	SMURF	deCODE	16593_3
13985_1	SMURF	deCODE	13479_8
12636_1	SMYD2	deCODE	8061_10
10014_3	SNAI2	deCODE	8786_6_
7762_30	SNN	deCODE	5615_62
17343_6	SNPH	deCODE	6433_57
12678_6	SNRPA	deCODE	5618_50
17823_4	SNRPD:	deCODE	9177_6_
17774_3	SNRPD:	deCODE	13102_1
5494_52	SNRPF	deCODE	5029_3_
19276_1	SNRPG	deCODE	5392_73
19274_8	SNTA1	deCODE	9459_7_
9078_20	SNTB1	deCODE	3052_8_
17765_3	SNUPN	deCODE	7206_20
6955_68	SNX1	deCODE	9568_28
8807_13	SNX1	deCODE	4987_17
12845_1	SNX17	deCODE	9254_18
19503_2	SNX3	deCODE	3291_30
3903_49	SNX4	deCODE	3309_2_
14245_1	SNX7	deCODE	3310_62
6925_26	SNX8	deCODE	3311_27
11440_5	SOCS3	deCODE	15582_2
11657_8	SOCS7	deCODE	13717_1

12976_4	SORBS3	deCODE	3313_21
15636_4	SORCS1	deCODE	5728_60
15637_3	SORCS3	deCODE	4440_15
9923_6_	SOS1	deCODE	6103_70
8237_56	SOSTD(	deCODE	6617_12
18242_8	SPA17	deCODE	15583_1
6228_58	SPACA1	deCODE	12577_1
8076_4_	SPACA3	deCODE	3367_8_
5726_49	SPAG11	deCODE	4392_54
5762_35	SPAG11	deCODE	4393_3_
8388_24	SPAST	deCODE	13724_2
11117_2	SPATA2	deCODE	3025_50
9524_46	SPATA3	deCODE	2763_66
7809_22	SPATA9	deCODE	3807_1_
10012_5	SPDEF	deCODE	7894_15
12876_3	SPEF1	deCODE	3065_65
11161_5	SPG20	deCODE	4130_71
11122_9	SPG21	deCODE	4487_1_
4294_16	SPHK1	deCODE	19584_3
4468_21	SPHK2	deCODE	15494_1
18210_1	SPIN1	deCODE	11219_9
10624_4	SPINK1	deCODE	3808_76
8587_21	SPINK1	deCODE	4988_49
10974_2	SPINK7	deCODE	5581_28
8042_88	SPINK9	deCODE	3810_50
12873_1	SPN	deCODE	13384_1
5491_12	SPOCK2	deCODE	9826_13
9906_21	SPOCK3	deCODE	17746_7
13727_4	SPOP	deCODE	9340_17
9287_6_	SPR	deCODE	11568_2
8318_13	SPRED1	deCODE	17722_5
13447_4	SPRN	deCODE	9288_7_
13942_1	SPSB1	deCODE	13122_1
5031_10	SPTAN1	deCODE	16315_1
7267_2_	SPTLC2	deCODE	8231_12
15448_4	SQSTM	deCODE	3437_80
18220_1	SRA1	deCODE	14093_1
19251_5	SRGN	deCODE	16035_8

12356_6	SRI	deCODE	7713_10
10940_2	SRL	deCODE	3434_34
18257_6	SRP19	deCODE	3435_53
11573_3	SRSF6	deCODE	4131_72
12987_1	SRSF7	deCODE	5478_50
14268_4	SRXN1	deCODE	17455_4
8298_8_	SSMEM	deCODE	15587_2
8106_15	SSR1	deCODE	15495_9
5032_64	SSRP1	deCODE	11540_3
5957_30	SST	deCODE	13740_5
13566_2	SSU72	deCODE	8036_75
6281_51	ST3GAI	deCODE	4132_27
10588_3	ST3GAI	deCODE	13112_1
6947_4_	ST3GAI	deCODE	3438_10
7867_15	ST6GAI	deCODE	9213_24
7823_22	ST6GAI	deCODE	6276_16
10705_1	ST6GAI	deCODE	4548_4_
7927_16	ST6GAI	deCODE	8244_16
7228_2_	ST6GAI	deCODE	15389_1
7920_30	ST8SIA:	deCODE	11647_6
6929_10	ST8SIA:	deCODE	17735_1
7038_45	ST8SIA4	deCODE	12661_4
6930_95	ST8SIA(	deCODE	11280_6
14599_1	STAB1	deCODE	9302_90
12401_3	STAMB	deCODE	18268_5
12510_3	STAP1	deCODE	15390_3
10085_2	STAR	deCODE	7003_4_
17383_4	STARD!	deCODE	5764_4_
10370_2	STAT1	deCODE	6593_5_
10346_5	STAT3	deCODE	3848_14
10372_1	STAT6	deCODE	15391_1
17172_1	STATH	deCODE	15326_6
12970_3	STAU2	deCODE	18891_9
8916_32	STIM1	deCODE	7818_10
9271_10	STIM1	deCODE	15589_1
3471_49	STK16	deCODE	4891_50
5249_31	STK17B	deCODE	18214_2
8399_6_	STK17B	deCODE	7016_12

17367_5	STMN1	deCODE	4374_45
10900_2	STMN2	deCODE	4880_21
8019_73	STMN3	deCODE	5822_22
6267_51	STMN4	deCODE	3034_1_
8261_51	STOM	deCODE	15398_2
17344_2	STOML	deCODE	3314_74
10336_3	STUB1	deCODE	2515_14
11350_3	STUB1	deCODE	2505_49
12842_4	STX10	deCODE	6920_1_
10418_3	STX12	deCODE	13594_1
9037_1_	STX18	deCODE	9370_69
19553_1	STX1A	deCODE	8462_18
7738_29	STX2	deCODE	10978_3
18308_3	STXBP6	deCODE	2948_58
19236_2	SUB1	deCODE	6518_85
10703_2	SUDS3	deCODE	8447_11
17468_1	SUFU	deCODE	16292_2
8305_18	SULF2	deCODE	5755_29
13944_3	SULT1A	deCODE	12711_1
12671_3	SULT1B	deCODE	15374_1
17811_7	SULT1C	deCODE	15588_1
16932_5	SULT1E	deCODE	10554_2
17209_2	SULT2B	deCODE	9883_29
8357_43	SULT4A	deCODE	13085_1
19555_1	SUMO2	deCODE	18386_3
14623_2	SUMO3	deCODE	16597_1
8852_10	SUN3	deCODE	15441_6
11260_4	SUN5	deCODE	13062_4
8009_12	SURF1	deCODE	19254_1
5752_63	SUSD3	deCODE	19446_1
12452_3	SUV420	deCODE	5757_45
11109_5	SVEP1	deCODE	3195_50
11178_2	SVEP1	deCODE	8909_77
18285_6	SVIP	deCODE	4912_17
19438_6	SYF2	deCODE	4990_87
10722_1	SYK	deCODE	7185_29
11378_3	SYK	deCODE	3194_36
7199_3_	SYN3	deCODE	8697_38

4224_7_	SYNCR:	deCODE	4991_12
9789_52	SYNE2	deCODE	13697_5
13375_4	SYNGR	deCODE	6395_58
17348_5	SYNJ2B	deCODE	4272_46
7089_42	SYT11	deCODE	8289_8_
9110_2_	SYT17	deCODE	8606_39
9577_26	SYT2	deCODE	11371_1
17355_5	SYT4	deCODE	16074_1
9099_19	SYT5	deCODE	5265_12
7121_2_	SYT7	deCODE	18295_1
7932_23	SYT8	deCODE	4992_49
8066_38	SYT9	deCODE	5897_58
12892_1	SYTL1	deCODE	8400_74
9337_43	TAC1	deCODE	7113_1_
6468_37	TAC4	deCODE	16607_7
9257_14	TACO1	deCODE	4775_34
18270_1	TAF10	deCODE	19273_3
16865_6	TAF15	deCODE	17138_8
15640_5	TAGLN	deCODE	4993_16
9201_13	TAGLN.	deCODE	18895_5
11636_3	TAGLN.	deCODE	4911_49
12378_7	TAPBP	deCODE	11273_1
6364_7_	TAPBPI	deCODE	17325_1
12046_5	TARDB	deCODE	15562_2
18186_1	TARS	deCODE	3440_7_
12498_1	TAX1BI	deCODE	4133_54
11211_7	TBCE	deCODE	3373_5_
3400_49	TBK1	deCODE	17739_1
11227_3	TBL2	deCODE	19267_1
2875_15	TBP	deCODE	17768_5
18434_1	TBPL1	deCODE	9021_1_
11146_4	TBX22	deCODE	5134_52
13978_1	TBX3	deCODE	11481_2
11202_7	TBX5	deCODE	14094_2
8098_37	TBXAS:	deCODE	18198_5
18204_1	TCEA1	deCODE	6919_3_
13481_2	TCEA2	deCODE	2859_69
19112_2	TCEAL:	deCODE	16758_9

19109_3	TCEAL8	deCODE	8953_47
19144_9	TCF4	deCODE	18225_1
19265_9	TCP1	deCODE	9116_28
11433_1	TCTN2	deCODE	15596_7
12854_3	TEAD3	deCODE	2681_23
12516_1	TEAD4	deCODE	3617_80
16079_2	TEC	deCODE	13644_3
11164_7	TENM2	deCODE	13089_6
11107_2	TENM3	deCODE	13130_1
11365_1	TENM4	deCODE	11530_3
17419_1	TES	deCODE	13496_1
12831_2	TESC	deCODE	17398_5
10557_6	TEX29	deCODE	2622_18
9952_57	TFAM	deCODE	11193_2
9043_7_	TFIP11	deCODE	19333_4
11537_1	TFR2	deCODE	17791_2
3520_58	TGFB3	deCODE	12549_3
9847_21	TGIF2	deCODE	5604_30
11242_3	TGM1	deCODE	18900_3
4471_50	TGM3	deCODE	6584_1_
18933_4	TGM4	deCODE	4996_66
3474_19	THBS1	deCODE	6986_17
8982_65	THBS3	deCODE	5465_32
18215_5	THG1L	deCODE	13524_2
9875_10	THOC1	deCODE	13972_4
12527_5	THRA	deCODE	6393_63
11174_8	THSD7A	deCODE	4124_24
9600_55	THSD7A	deCODE	11103_2
12424_1	THYN1	deCODE	19127_1
19131_1	TIAL1	deCODE	15626_2
14035_1	TIAM1	deCODE	3317_33
17696_1	TICAM2	deCODE	8309_12
17699_4	TIMELE	deCODE	4342_10
9058_1_	TIMM21	deCODE	5486_73
7743_5_	TIMM50	deCODE	2649_77
10638_1	TINF2	deCODE	6550_4_
9839_14	TIRAP	deCODE	5124_69
12001_7	TJP1	deCODE	8245_27

19114_8	TK2	deCODE	5061_27
4306_4_	TKT	deCODE	17832_1
6383_90	TLL1	deCODE	9759_13
14082_5	TLN2	deCODE	3198_4_
18935_1	TLR5	deCODE	3169_70
8922_4_	TMCC3	deCODE	13642_9
10761_5	TMED2	deCODE	9183_7_
15641_2	TMEFF1	deCODE	15346_3
8687_26	TMEM1	deCODE	5825_49
10583_1	TMEM1	deCODE	8818_13
11110_4	TMEM1	deCODE	9180_6_
8890_9_	TMEM1	deCODE	4396_54
11128_2	TMEM1	deCODE	4397_26
7173_14	TMEM1	deCODE	7192_37
13416_8	TMEM1	deCODE	7196_21
11223_1	TMEM1	deCODE	12358_6
11112_1	TMEM1	deCODE	7118_24
13421_1	TMEM1	deCODE	11952_1
14633_2	TMEM1	deCODE	9793_14
10442_1	TMEM1	deCODE	4232_19
8992_1_	TMEM2	deCODE	16057_6
11542_1	TMEM2	deCODE	2771_35
8107_12	TMEM2	deCODE	8469_41
12856_1	TMEM2	deCODE	2571_12
9509_4_	TMEM3	deCODE	2950_57
11186_1	TMEM5	deCODE	14088_3
9778_45	TMEM5	deCODE	3320_49
12367_5	TMEM5	deCODE	7815_49
9959_60	TMEM5	deCODE	6478_2_
8074_32	TMEM7	deCODE	9715_15
13485_2	TMEM8	deCODE	6984_6_
12742_1	TMEM8	deCODE	2773_50
8597_1_	TMEM9	deCODE	10344_3
9249_17	TMEM9	deCODE	8104_21
12595_1	TMOD1	deCODE	2631_50
12853_1	TMOD2	deCODE	4493_92
12861_1	TMOD3	deCODE	13733_5
8265_22	TMPO	deCODE	2632_5_

7231_37	TMPRSS	deCODE	3815_14
11239_4	TMPRSS	deCODE	3072_4_
9226_6_	TMUB2	deCODE	2633_52
10675_2	TMX2	deCODE	19568_1
5654_70	TMX3	deCODE	14054_1
14009_6	TNFAIP	deCODE	3445_53
5036_50	TNFAIP	deCODE	2774_10
14121_2	TNFRSF	deCODE	9170_24
5526_53	TNFRSF	deCODE	9255_5_
11837_7	TNFRSF	deCODE	4136_40
2968_61	TNFSF1	deCODE	14026_2
2708_54	TNFSF1	deCODE	2992_59
2839_2_	TNFSF4	deCODE	6262_14
2599_51	TNFSF9	deCODE	5661_15
9907_21	TNKS	deCODE	3073_51
6578_29	TNMD	deCODE	14079_1
5440_26	TNNI2	deCODE	2993_1_
5315_22	TNNT2	deCODE	3035_80
16807_3	TNNT3	deCODE	4851_25
9808_41	TNRC6I	deCODE	3037_62
11667_2	TNS2	deCODE	2991_9_
9927_96	TNS4	deCODE	14133_9
13963_7	TOLLIP	deCODE	14048_7
13652_2	TOM1L	deCODE	4234_8_
19356_2	TOM1L	deCODE	2994_71
10817_2	TOMM2	deCODE	5353_89
9947_22	TOPBP1	deCODE	3070_1_
10553_8	TOR1AI	deCODE	4138_25
13459_3	TOR4A	deCODE	5085_18
12717_6	TOX3	deCODE	13435_3
13022_2	TP53I11	deCODE	9366_54
6400_33	TP53I13	deCODE	2778_10
10040_6	TP63	deCODE	3620_67
17710_4	TPD52L	deCODE	3321_2_
4309_59	TPI1	deCODE	4137_57
5033_27	TPM1	deCODE	3151_6_
4472_5_	TPM2	deCODE	9343_16
9863_1_	TPM4	deCODE	2634_2_

3873_51	TPO	deCODE	4717_55
3403_1_	TPSB2	deCODE	10455_1
7928_18	TPST1	deCODE	8273_84
8024_64	TPST2	deCODE	9051_13
12373_7	TRA2B	deCODE	4556_10
11262_3	TRABD	deCODE	14150_7
9401_57	TRABD	deCODE	9117_4_
13507_5	TRADD	deCODE	13744_3
17747_4	TRAF1	deCODE	2906_55
13041_4	TRAF4	deCODE	3055_54
14337_1	TRAPP(	deCODE	11071_1
7007_24	TRAPP(	deCODE	13686_2
8671_37	TRAPP(	deCODE	4673_13
7224_11	TRAT1	deCODE	15602_4
9266_1_	TREM1	deCODE	2620_4_
17829_2	TREX2	deCODE	4140_3_
5659_11	TRH	deCODE	5089_11
12363_7	TRIB2	deCODE	5834_18
6527_1_	TRIL	deCODE	17796_1
12573_8	TRIM3	deCODE	3888_8_
11126_1	TRIO	deCODE	13676_4
17676_1	TRIP10	deCODE	16746_1
13044_5	TSG101	deCODE	15686_4
12477_4	TSN	deCODE	6408_2_
7069_9_	TSNARI	deCODE	5723_4_
10546_2	TSPEAF	deCODE	6410_26
7018_10	TSR3	deCODE	10462_1
18181_2	TSSC4	deCODE	3448_13
11179_7	TSSK1E	deCODE	18162_1
7873_32	TSSK2	deCODE	13124_2
12657_2	TSTA3	deCODE	8355_80
19277_4	TSTD1	deCODE	12434_2
9864_38	TTC1	deCODE	15603_2
8800_14	TTC17	deCODE	6932_42
9550_15	TTC17	deCODE	11617_1
9386_42	TTC9B	deCODE	12750_9
13973_6	TTL	deCODE	7755_37
5690_49	TUFT1	deCODE	7737_76

12871_1	TWF1	deCODE	11205_1
9234_8_	TWSG1	deCODE	7955_19
9573_10	TXNDC	deCODE	7145_1_
19334_6	TXNDC	deCODE	4811_33
11682_7	TXNIP	deCODE	8233_2_
15316_2	TXNL4I	deCODE	7765_15
5260_80	TYK2	deCODE	18916_2
5035_7_	TYMS	deCODE	17737_7
8263_64	TYMSO	deCODE	2997_8_
13577_2	U2AF2	deCODE	2998_53
13580_2	UAP1	deCODE	10356_2
12500_8	UBA2	deCODE	7144_23
12558_3	UBASH	deCODE	7011_8_
6641_60	UBB	deCODE	12473_4
6651_74	UBB	deCODE	3651_50
6647_55	UBC	deCODE	7070_25
17772_7	UBD	deCODE	11145_7
17743_1	UBE2A	deCODE	6538_90
12556_7	UBE2C	deCODE	9899_28
19247_1	UBE2D1	deCODE	12734_1
18842_2	UBE2D2	deCODE	7773_20
19280_2	UBE2D3	deCODE	17153_4
13475_1	UBE2D4	deCODE	17152_1
14326_4	UBE2E1	deCODE	18907_9
18213_3	UBE2F	deCODE	5096_51
17158_1	UBE2G1	deCODE	16609_1
9199_6_	UBE2G2	deCODE	2475_1_
2877_3_	UBE2I	deCODE	9377_25
6900_30	UBE2J1	deCODE	19557_3
17380_2	UBE2K	deCODE	17850_4
3874_8_	UBE2L3	deCODE	6227_1_
19111_1	UBE2M	deCODE	2831_29
3905_62	UBE2N	deCODE	3199_54
17729_2	UBE2S	deCODE	11152_4
12400_2	UBE2T	deCODE	15544_2
11626_7	UBE2V1	deCODE	6491_59
18165_1	UBE2V2	deCODE	8468_19
11226_1	UBE3A	deCODE	2833_20

11490_4	UBL4A	deCODE	3450_4_
19124_9	UBLCP1	deCODE	3378_49
19121_3	UBQLN	deCODE	2834_54
12720_7	UBQLN	deCODE	4152_58
12875_2	UBTD2	deCODE	10809_1
18435_4	UBXN2	deCODE	5629_58
9970_7_	UBXN4	deCODE	5098_79
9997_12	UBXN4	deCODE	14095_1
5019_16	UCHL1	deCODE	12923_5
17405_2	UCHL5	deCODE	5354_11
12515_4	UCK2	deCODE	15606_1
10977_5	UCMA	deCODE	11177_1
10756_3	UCN3	deCODE	4559_64
3405_6_	UFC1	deCODE	4246_40
17411_5	UFD1L	deCODE	7163_26
3836_51	UFM1	deCODE	9950_22
13939_1	UGP2	deCODE	11284_2
17702_5	UGT1A	deCODE	7130_4_
7891_45	UGT1A	deCODE	6577_64
8899_75	UGT1A	deCODE	16902_1
8907_11	UGT2A	deCODE	13959_7
3081_70	ULBP1	deCODE	15610_7
2747_3_	ULBP3	deCODE	10551_7
12437_1	ULK3	deCODE	5613_75
9114_84	UMODI	deCODE	2635_61
14052_2	UNC13	deCODE	3074_6_
13082_9	UNC45	deCODE	15413_3
7975_97	UNC5A	deCODE	2836_68
15394_7	UNC5B	deCODE	17231_1
7776_20	UNC5B	deCODE	13129_4
5139_32	UNC5C	deCODE	18183_3
13487_2	UNC93I	deCODE	16763_1
6633_43	UPK3BI	deCODE	15503_1
19448_1	UPP1	deCODE	15503_2
12957_6	UQCRB	deCODE	7154_92
11328_9	USB1	deCODE	8484_24
17725_3	USH1C	deCODE	5400_52
19189_2	USP14	deCODE	8046_9_

19161_1	USP15	deCODE	3066_12
12681_6	USP21	deCODE	5000_52
8364_74	UST	deCODE	2982_82
6020_52	UTS2	deCODE	9196_8_
6290_3_	UTS2B	deCODE	4909_68
13530_5	UTS2R	deCODE	9197_4_
17711_1	VAMP1	deCODE	3622_33
17335_1	VAMP2	deCODE	8376_25
7903_18	VAMP3	deCODE	7790_21
7732_45	VAMP4	deCODE	5837_49
19357_1	VAMP7	deCODE	15614_1
7167_10	VAPA	deCODE	6391_52
7181_17	VAPB	deCODE	8299_66
13083_1	VARS	deCODE	7787_25
5275_28	VAV1	deCODE	8766_29
19193_1	VBP1	deCODE	7059_14
8750_46	VCL	deCODE	5090_49
10023_3	VDR	deCODE	5091_28
17362_5	VHL	deCODE	6453_70
11286_7	VIMP	deCODE	7015_8_
3522_57	VIP	deCODE	3889_64
18938_3	VLDLR	deCODE	12504_2
14618_2	VOPP1	deCODE	6398_12
12508_9	VPS24	deCODE	11175_4
18189_1	VPS25	deCODE	4801_13
19293_6	VPS26A	deCODE	11252_3
14318_1	VPS29	deCODE	7200_4_
11476_4	VPS4A	deCODE	18831_6
12553_5	VRK1	deCODE	3322_52
8367_14	VSIG1	deCODE	10699_5
7242_14	VSTM4	deCODE	8601_16
8963_8_	VTI1B	deCODE	9182_3_
13125_4	VTN	deCODE	15472_1
7128_9_	VWA2	deCODE	6713_4_
9532_5_	WBP1L	deCODE	10618_1
17698_1	WBP2	deCODE	3640_14
17760_1	WDR5	deCODE	9317_4_
13429_3	WFDC1	deCODE	8897_3_

10695_1	WFDC1	deCODE	19163_2
7969_16	WFDC1	deCODE	11293_1
9345_43	WFDC1	deCODE	9368_64
6384_19	WFDC3	deCODE	8906_60
6969_14	WFDC5	deCODE	17795_1
13412_5	WFDC6	deCODE	4703_87
7147_35	WFDC8	deCODE	3204_2_
13692_1	WISP1	deCODE	2636_10
3057_55	WISP1	deCODE	16620_2
6392_7_	WISP2	deCODE	3324_51
5927_4_	WISP3	deCODE	3381_24
5493_17	WNK3	deCODE	3453_87
10044_1	WNT10A	deCODE	9327_3_
6624_94	WNT11	deCODE	13107_9
13236_2	WNT3A	deCODE	10563_1
13268_4	WNT5A	deCODE	3206_4_
4889_82	WNT7A	deCODE	6377_54
12528_4	WRNIP1	deCODE	13618_1
6274_15	WSCD2	deCODE	12576_2
18284_7	WWC1	deCODE	6119_14
17724_3	WWOX	deCODE	9077_10
11307_3	WWP1	deCODE	9251_28
15617_8	XCL2	deCODE	9223_11
11264_3	XDH	deCODE	9557_5_
3481_87	XPNPEI	deCODE	9578_26
12535_2	XRCC1	deCODE	17781_1
2835_1_	XRCC6	deCODE	2864_2_
6375_75	XXYLT	deCODE	17175_5
19270_2	YARS	deCODE	5006_71
9751_72	YBX1	deCODE	15604_1
6372_7_	YBX2	deCODE	3820_68
17474_1	YEATS4	deCODE	16885_4
9984_12	YIPF6	deCODE	5854_60
11190_1	YME1L1	deCODE	9003_99
8878_48	YTHDC	deCODE	3605_77
14156_3	YWHAI	deCODE	9125_23
14157_2	YWHAI	deCODE	3325_2_
5858_6_	YWHAZ	deCODE	19361_7

17345_1	ZADH2	deCODE	12411_6
3837_6_	ZAP70	deCODE	3042_7_
4476_22	ZAP70	deCODE	3000_66
12785_4	ZBTB33	deCODE	9388_18
11677_1	ZDHHC	deCODE	6283_60
19173_5	ZFAND:	deCODE	12488_9
18317_1	ZFAND!	deCODE	16900_2
13651_5	ZFP91	deCODE	3853_56
9102_28	ZFYVE2	deCODE	2911_27
8219_14	ZG16	deCODE	7898_29
8310_6_	ZG16B	deCODE	11168_3
12751_2	ZHX1	deCODE	5744_12
10036_2	ZHX3	deCODE	3209_69
11567_2	ZNF10	deCODE	2837_3_
12787_4	ZNF134	deCODE	3210_1_
12716_3	ZNF175	deCODE	3170_6_
11372_2	ZNF18	deCODE	8837_8_
12771_1	ZNF180	deCODE	5636_10
11565_5	ZNF23	deCODE	6440_31
9993_11	ZNF264	deCODE	4455_89
9609_25	ZNF275	deCODE	2687_2_
14692_3	ZNF276	deCODE	12891_1
12803_9	ZNF329	deCODE	5102_55
12763_6	ZNF334	deCODE	8221_19
18265_1	ZNF34	deCODE	11173_2
14616_1	ZNF382	deCODE	5631_83
10003_1	ZNF41	deCODE	4924_32
12843_6	ZNF410	deCODE	8479_4_
12811_5	ZNF415	deCODE	4496_60
14619_8	ZNF526	deCODE	4925_54
12543_7	ZNF560	deCODE	2788_55
12795_2	ZNF566	deCODE	2789_26
12760_3	ZNF774	deCODE	9172_69
8321_27	ZNF843	deCODE	2579_17
10390_2	ZNRF3	deCODE	15619_4
14122_1	ZNRF3	deCODE	18193_1
8635_28	ZPBP	deCODE	2580_83
10710_2	ZPBP2	deCODE	2637_77

5590_11	ZPLD1	deCODE
18302_2	ZWILCH	deCODE
18286_3	ZWINT	deCODE
13632_1	ZYX	deCODE

7123_25
3893_64
10620_2
15533_9
11207_3
7137_8_
4407_10
14583_4
18321_3
19129_1
7852_9_
15565_1
11391_6
10521_1
7648_9_
9248_36
18376_1
14227_2
9894_13
16558_2
13534_2
13966_3
17854_3
3173_49
13624_1
3894_15
11208_1
5011_11
19188_2
2944_66
4498_62
6507_16
15573_1
10047_1
13615_6
8360_16
7854_38
19215_7

6927_7_
19200_1
12862_1
10082_2
12703_6
6544_33
6022_57
9219_70
8900_28
7179_69
14286_2
9869_28
7770_25
5801_72
8374_5_
7153_66
9087_8_
3213_65
16060_9
17808_3
15620_4
9027_10
9772_15
5909_51
17512_2
18932_8
5196_7_
5107_7_
5106_52
11297_5
5108_72
10424_3
19496_1
8870_38
16751_1
3723_1_
7655_11
7194_36

9256_78
6521_35
15511_3
5109_24
18303_3
5542_22
15387_4
16323_8
5111_15
4250_23
12560_9
19615_2
15452_5
4145_58
4146_58
4866_59
2658_27
8887_21
16583_8
17761_2
4562_1_
6054_6_
5680_54
17224_1
4769_10
16049_4
5358_3_
16908_5
18240_6
12591_2
15430_1
7116_31
14063_1
2693_20
10892_8
7265_32
8356_88
4278_14

8867_18
12374_8
18914_1
13719_1
18306_1
5620_13
4148_49
19523_2
19545_1
10534_4
8893_29
17475_1
11313_1
10558_2
19364_1
11237_4
4459_68
5231_79
19240_2
15623_1
3004_67
12517_5
19488_1
14192_3
5255_22
16805_5
5256_86
4499_21
4149_8_
13658_3
10366_1
3459_49
4719_58
7146_16
5593_11
12593_3
16852_1
8275_31

8892_14
4276_10
2695_25
17776_1
9076_25
2697_7_
19243_2
4187_49
3078_1_
3329_14
5601_2_
18330_7
15693_9
4982_54
3216_2_
10816_1
6402_8_
8825_4_
13486_9
13745_1
15607_5
2949_6_
4910_21
2692_74
5015_15
2212_69
4158_54
2652_15
11354_2
3710_49
4150_75
4151_6_
14687_6
11407_5
15475_4
9235_3_
11342_5
19560_2

9216_10
16922_5
17794_6
12450_4
15613_1
6627_25
15471_2
10616_6
2558_51
4890_10
9204_33
4261_55
3457_57
16765_5
17165_1
2790_54
4544_4_
13996_1
4718_5_
5238_26
5248_68
12619_1
17852_5
19295_3
19117_3
19152_4
13622_1
15545_1
9244_27
4588_1_
11432_1
15310_6
11591_4
5722_78
3855_56
8358_30
5017_19
5018_68

7258_5_
9015_1_
19125_2
3817_18
13067_5
2585_2_
18231_1
2961_1_
3758_68
2247_20
2700_56
5034_79
4534_10
15535_3
15513_1
13720_9
6207_10
8814_33
15631_1
17176_1
3859_50
7880_9_
10716_3
13931_2
5918_5_
17694_3
12729_1
2696_87
3831_21
10514_5
18458_4
19617_5
19640_2
5954_62
13470_4
9525_1_
3045_72
19231_2

3005_5_
4318_12
12633_3
11988_2
6361_49
6049_64
12014_1
17727_1
6447_73
8064_12
11324_3
12835_1
3727_35
6580_29
11257_1
7849_3_
13596_3
19253_8
12403_3
14271_2
13599_1
16769_2
12522_6
2871_73
18432_3
18191_2
8398_27
3079_62
5481_16
19467_3
17391_1
11468_1
19279_4
19241_3
14208_3
13095_5
16770_3
15304_1

15476_6
11102_2
11464_9
14112_4
3396_54
3220_40
3046_31
13590_1
5483_1_
3331_8_
11634_3
11666_7
17764_1
6621_10
7211_2_
5602_62
15576_1
5644_60
5646_20
16913_8
9773_15
13411_2
14120_2
5740_17
5116_62
16297_1
2590_69
19264_6
12646_2
13011_2
17742_2
17750_8
8925_25
16614_2
6240_70
13094_7
8427_11
18180_5

5105_2_
12365_1
14011_1
5852_6_
7223_60
17828_3
17836_1
15297_3
14116_1
9926_4_
15516_1
12830_4
9859_18
12524_1
13511_2
5724_58
10419_1
5100_53
5129_12
8956_96
9925_56
7957_2_
10569_2
6252_62
15305_7
12684_5
8353_15
7911_29
3728_52
3470_1_
4831_4_
4154_57
11266_8
5628_21
7923_41
5737_61
14597_5
7202_10

7019_13
3580_25
9002_36
6551_94
2879_9_
4153_11
3449_58
3389_7_
4785_30
2706_69
7266_4_
10737_9
15417_3
19130_8
3344_60
3316_58
2925_9_
19154_4
9211_19
3024_18
4479_14
10800_1
7117_21
12462_2
19563_3
3867_49
13449_2
3221_54
17447_5
19590_4
4567_82
19113_6
18318_9
18222_3
4929_55
16043_3
5272_55
2743_5_

12334_2
9263_57
4464_10
10847_1
16792_4
2741_22
2742_68
7864_3_
3007_7_
5430_66
6247_9_
5030_52
12461_8
11194_6
7953_20
5128_53
5487_7_
8994_65
18930_2
15539_1
16916_1
7191_32
9838_4_
10364_6
10363_1
14324_5
17805_3
13118_5
15635_4
10818_3
4771_10
18179_5
12546_1
13105_7
12357_4
6975_52
8458_11
17364_8

18327_6
2794_60
5008_51
5660_51
4569_52
15447_4
11300_3
13101_6
18294_2
6494_60
3043_49
4467_49
8243_55
13405_6
19213_1
8028_22
5731_1_
7962_11
2828_82
2843_13
7926_13
5490_53
4297_62
8099_42
13113_7
7886_26
15433_4
5488_74
17510_7
13526_5
13625_1
18387_7
5657_28
6035_2_
3399_31
19187_2
16551_1
12471_4

4930_21
6231_46
5489_18
12617_2
6555_58
6966_14
7186_11
10668_5
10945_1
8274_64
10903_5
17818_2
9829_91
6941_11
6069_71
18324_6
9582_93
12880_1
13552_7
9090_9_
11563_5
14034_2
2619_72
17775_8
11347_9
14013_1
12781_2
12721_4
9960_2_
12501_1
17686_2
17210_2
18276_3
19614_8
15560_5
5584_21
8334_65
5810_25

9880_33
10073_2
3773_15
10049_1
4162_54
9185_15
9191_8_
4721_54
8323_16
3336_50
9233_71
6895_1_
8795_48
18839_2
2333_72
4156_74
3283_21
5133_17
3009_3_
18934_5
14111_1
3339_33
3340_53
5947_90
8059_1_
5621_64
13595_2
2844_53
12476_5
16299_1
9638_2_
15449_3
19148_5
2211_9_
2278_61
2480_58
6462_12
11192_1

4301_58
16324_3
11149_3
3835_11
16918_1
11101_1
6907_17
6506_54
9319_59
10499_1
7871_16
9014_18
10895_2
6547_83
3189_61
8002_27
4155_3_
5936_53
12563_2
4832_75
5534_49
8529_1_
8304_50
5138_50
2704_74
5383_14
5352_11
2665_26
5131_15
2654_19
3152_57
8368_10
5404_53
3730_81
5070_76
2605_49
14025_1
8833_20

14061_4
5939_42
3059_50
5355_69
5988_49
3421_54
5441_67
11302_2
5698_60
2876_74
10606_3
9039_47
6123_69
6168_11
17748_2
12018_8
11218_8
17691_1
12800_5
12417_4
9409_11
3404_51
3872_2_
12635_9
16300_4
11147_1
9329_28
5736_1_
18277_2
3010_53
12663_1
11352_4
15698_6
8813_16
6366_38
11212_7
13967_1
11361_7

2611_72
9865_40
19190_4
17755_5
3082_9_
9451_20
16307_2
19289_2
11248_4
13639_1
9215_11
19562_8
13450_4
8258_22
7064_2_
5682_13
9762_14
18175_6
9830_10
2967_8_
9561_21
19437_6
2597_8_
4867_15
9453_12
3132_1_
15540_6
6234_74
15480_2
17155_1
12668_7
10825_1
8018_43
13522_2
6549_60
4209_60
7952_2_
6385_63

15308_1
7995_16
3050_7_
9870_17
9316_67
11388_7
3191_50
3235_50
16070_7
4143_74
8044_90
9886_28
2878_66
7625_27
17337_1
11486_2
11596_4
7766_25

Set 3

Gene	From
A1BG	UKB
ABHD1·	UKB
ABL1	UKB
ABO	UKB
ACAA1	UKB
ACADM	UKB
ACADS	UKB
ACAN	UKB
ACE	UKB
ACE2	UKB
ACHE	UKB
ACOT1:	UKB
ACP1	UKB
ACP5	UKB
ACP6	UKB
ACRV1	UKB
ACTN2	UKB
ACVRL	UKB
ACY1	UKB
ADAM1	UKB
ADAM1	UKB
ADAM2	UKB
ADAM2	UKB
ADAM9	UKB
ADAMT	UKB
ADAMT	UKB
ADAMT	UKB
ADAMT	UKB
ADAMT	UKB
ADGRE	UKB
ADGRF	UKB
ADH1B	UKB
ADH4	UKB
ADIPOC	UKB
ADM	UKB

AFM	UKB
AFP	UKB
AGER	UKB
AGR2	UKB
AGR3	UKB
AGRN	UKB
AGRP	UKB
AGT	UKB
AHSA1	UKB
AHSG	UKB
AHSP	UKB
AIDA	UKB
AIF1	UKB
AIF1L	UKB
AIFM1	UKB
AK1	UKB
AK2	UKB
AKR1B1	UKB
AKR1B1	UKB
AKR1C4	UKB
AKT2	UKB
ALCAM	UKB
ALDH1A	UKB
ALDH2	UKB
ALDH3A	UKB
ALDH5A	UKB
ALPI	UKB
AMBP	UKB
AMIGO	UKB
AMIGO	UKB
AMN	UKB
AMY1A	UKB
AMY2A	UKB
AMY2B	UKB
ANG	UKB
ANGPT1	UKB
ANGPT2	UKB
ANGPTI	UKB

ANGPTL	UKB
ANGPTL	UKB
ANGPTL	UKB
ANK2	UKB
ANKRD	UKB
ANPEP	UKB
ANXA1	UKB
ANXA1(	UKB
ANXA1	UKB
ANXA2	UKB
ANXA3	UKB
ANXA4	UKB
ANXA5	UKB
AOC1	UKB
AP1G2	UKB
APCS	UKB
APEX1	UKB
APLP1	UKB
APOA1	UKB
APOA2	UKB
APOA4	UKB
APOB	UKB
APOC1	UKB
APOD	UKB
APOE	UKB
APOF	UKB
APOH	UKB
APOL1	UKB
APOM	UKB
APP	UKB
APRT	UKB
ARAF	UKB
AREG	UKB
ARF6	UKB
ARFIP1	UKB
ARG1	UKB
ARG2	UKB
ARHGA	UKB

ARHGA	UKB
ARHGA	UKB
ARHGA	UKB
ARHGE	UKB
ARHGE	UKB
ARID3A	UKB
ARL2BI	UKB
ARSA	UKB
ARSB	UKB
ART3	UKB
ARTN	UKB
ASAH1	UKB
ASAH2	UKB
ASGR1	UKB
ASGR2	UKB
ASPN	UKB
ASRGL1	UKB
ATOX1	UKB
ATP1B1	UKB
ATP1B2	UKB
ATP1B3	UKB
ATP1B4	UKB
ATRN	UKB
ATXN3	UKB
AXL	UKB
AZU1	UKB
B2M	UKB
B3GAT3	UKB
B4GALT	UKB
BACH1	UKB
BAG3	UKB
BAG4	UKB
BAIAP2	UKB
BAMBI	UKB
BATF	UKB
BCAM	UKB
BCAN	UKB
BCAT2	UKB

BCHE	UKB
BCL2	UKB
BCL2L1	UKB
BCL2L1	UKB
BDNF	UKB
BECN1	UKB
BGLAP	UKB
BGN	UKB
BHMT2	UKB
BID	UKB
BIRC2	UKB
BLMH	UKB
BLNK	UKB
BLVRB	UKB
BMP10	UKB
BMP4	UKB
BMP6	UKB
BMPER	UKB
BNIP3L	UKB
BOC	UKB
BOLA1	UKB
BOLA2	UKB
BPIFA2	UKB
BPIFB1	UKB
BRD1	UKB
BRD2	UKB
BRDT	UKB
BRSK2	UKB
BSG	UKB
BST1	UKB
BST2	UKB
BTC	UKB
BTD	UKB
BTLA	UKB
BTN2A1	UKB
C1GAL1	UKB
C1QBP	UKB
C1QL2	UKB

C1QTNI	UKB
C1QTNI	UKB
C1QTNI	UKB
C1R	UKB
C1RL	UKB
C1S	UKB
C2	UKB
C3	UKB
C4BPB	UKB
C5	UKB
C7	UKB
C9	UKB
CA1	UKB
CA11	UKB
CA12	UKB
CA13	UKB
CA2	UKB
CA3	UKB
CA4	UKB
CA5A	UKB
CA6	UKB
CA7	UKB
CA8	UKB
CA9	UKB
CACYB	UKB
CALB1	UKB
CALB2	UKB
CALCB	UKB
CALCO	UKB
CALY	UKB
CAMKK	UKB
CANT1	UKB
CAPG	UKB
CAPN3	UKB
CARHS	UKB
CASP10	UKB
CASP2	UKB
CASP3	UKB

CASP8	UKB
CASQ2	UKB
CAT	UKB
CBLN1	UKB
CBLN4	UKB
CBS	UKB
CCDC1:	UKB
CCDC5(	UKB
CCDC8(	UKB
CCL11	UKB
CCL13	UKB
CCL14	UKB
CCL15	UKB
CCL16	UKB
CCL17	UKB
CCL18	UKB
CCL19	UKB
CCL2	UKB
CCL20	UKB
CCL21	UKB
CCL22	UKB
CCL23	UKB
CCL24	UKB
CCL25	UKB
CCL26	UKB
CCL27	UKB
CCL28	UKB
CCL3	UKB
CCL5	UKB
CCL7	UKB
CCL8	UKB
CCS	UKB
CCT5	UKB
CD109	UKB
CD14	UKB
CD160	UKB
CD163	UKB
CD164L	UKB

CD177	UKB
CD2	UKB
CD200	UKB
CD200R	UKB
CD207	UKB
CD209	UKB
CD226	UKB
CD244	UKB
CD248	UKB
CD27	UKB
CD274	UKB
CD300A	UKB
CD300C	UKB
CD300E	UKB
CD300L	UKB
CD33	UKB
CD36	UKB
CD38	UKB
CD3E	UKB
CD3G	UKB
CD4	UKB
CD40LG	UKB
CD46	UKB
CD48	UKB
CD5	UKB
CD55	UKB
CD58	UKB
CD59	UKB
CD5L	UKB
CD63	UKB
CD69	UKB
CD7	UKB
CD70	UKB
CD72	UKB
CD74	UKB
CD79B	UKB
CD80	UKB
CD83	UKB

CD86	UKB
CD8A	UKB
CD93	UKB
CD99L2	UKB
CDC25A	UKB
CDC37	UKB
CDC42E	UKB
CDCP1	UKB
CDH1	UKB
CDH15	UKB
CDH17	UKB
CDH2	UKB
CDH3	UKB
CDH5	UKB
CDH6	UKB
CDHR1	UKB
CDHR5	UKB
CDKN1.	UKB
CDKN2	UKB
CDNF	UKB
CDON	UKB
CDSN	UKB
CEACA	UKB
CEACA	UKB
CEACA	UKB
CEACA	UKB
CEACA	UKB
CEBPA	UKB
CEBPB	UKB
CELA2A	UKB
CES1	UKB
CETN2	UKB
CETN3	UKB
CFB	UKB
CFC1	UKB
CFD	UKB
CFH	UKB
CFHR2	UKB

CFHR4	UKB
CFHR5	UKB
CFI	UKB
CFP	UKB
CGA	UKB
CGREF1	UKB
CHAD	UKB
CHCHD	UKB
CHEK2	UKB
CHGA	UKB
CHGB	UKB
CHI3L1	UKB
CHIT1	UKB
CHL1	UKB
CHP1	UKB
CHRDL	UKB
CHRDL	UKB
CILP	UKB
CINP	UKB
CIRBP	UKB
CKAP4	UKB
CKB	UKB
CKMT1	UKB
CLC	UKB
CLEC10	UKB
CLEC11	UKB
CLEC12	UKB
CLEC1E	UKB
CLEC2I	UKB
CLEC3E	UKB
CLEC4A	UKB
CLEC4C	UKB
CLEC4I	UKB
CLEC4C	UKB
CLEC4N	UKB
CLEC6A	UKB
CLEC7A	UKB
CLGN	UKB

CLIC5	UKB
CLINT1	UKB
CLMP	UKB
CLPP	UKB
CLPS	UKB
CLSTN1	UKB
CLSTN2	UKB
CLSTN3	UKB
CLTA	UKB
CLU	UKB
CNDP1	UKB
CNP	UKB
CNPY4	UKB
CNTF	UKB
CNTN1	UKB
CNTN2	UKB
CNTN4	UKB
CNTN5	UKB
CNTNA	UKB
COCH	UKB
COL15A	UKB
COL18A	UKB
COL1A1	UKB
COL28A	UKB
COL2A1	UKB
COL3A1	UKB
COL6A3	UKB
COL9A1	UKB
COLEC1	UKB
COMMI	UKB
COMMI	UKB
COMP	UKB
COMT	UKB
COPE	UKB
COX5B	UKB
CPA2	UKB
CPA4	UKB
CPB1	UKB

CPB2	UKB
CPE	UKB
CPLX2	UKB
CPM	UKB
CPOX	UKB
CPQ	UKB
CPVL	UKB
CPXM1	UKB
CR1	UKB
CR2	UKB
CRADD	UKB
CREG1	UKB
CRELD1	UKB
CRH	UKB
CRHBP	UKB
CRIM1	UKB
CRIP2	UKB
CRISP2	UKB
CRISP3	UKB
CRKL	UKB
CRLF1	UKB
CRNN	UKB
CRTAC1	UKB
CRTAM	UKB
CRYBB	UKB
CRYBB.	UKB
CRYGD	UKB
CRYZL1	UKB
CSDE1	UKB
CSF1	UKB
CSF1R	UKB
CSF2	UKB
CSF2RA	UKB
CSF2RB	UKB
CSF3	UKB
CSF3R	UKB
CSNK1I	UKB
CSNK2A	UKB

CSPG4	UKB
CSRP3	UKB
CST1	UKB
CST3	UKB
CST5	UKB
CST6	UKB
CST7	UKB
CTF1	UKB
CTHRC1	UKB
CTLA4	UKB
CTRB1	UKB
CTRC	UKB
CTRL	UKB
CTSB	UKB
CTSC	UKB
CTSD	UKB
CTSE	UKB
CTSF	UKB
CTSH	UKB
CTSL	UKB
CTSO	UKB
CTSS	UKB
CTSV	UKB
CTSZ	UKB
CUZD1	UKB
CX3CL1	UKB
CXADR	UKB
CXCL1	UKB
CXCL10	UKB
CXCL11	UKB
CXCL12	UKB
CXCL13	UKB
CXCL14	UKB
CXCL16	UKB
CXCL17	UKB
CXCL5	UKB
CXCL6	UKB
CXCL8	UKB

CXCL8	UKB
CXCL8	UKB
CXCL8	UKB
CXCL9	UKB
CYB5A	UKB
CYB5R2	UKB
CYTL1	UKB
DAG1	UKB
DAPK2	UKB
DAPP1	UKB
DBI	UKB
DBNL	UKB
DCN	UKB
DCTD	UKB
DCTN2	UKB
DCTN6	UKB
DCTPP1	UKB
DCUN1	UKB
DCUN1	UKB
DCXR	UKB
DDAH1	UKB
DDC	UKB
DDR1	UKB
DDT	UKB
DDX25	UKB
DDX58	UKB
DECR1	UKB
DEFA1	UKB
DEFB10	UKB
DEFB10	UKB
DEFB11	UKB
DEFB11	UKB
DEFB4A	UKB
DENR	UKB
DGCR6	UKB
DHPS	UKB
DIABLO	UKB
DKK1	UKB

DKK3	UKB
DKK4	UKB
DKKL1	UKB
DLG4	UKB
DLK1	UKB
DLL1	UKB
DLL4	UKB
DNAJA2	UKB
DNAJA4	UKB
DNAJB1	UKB
DNAJB1	UKB
DNAJB2	UKB
DNAJB6	UKB
DNER	UKB
DOC2B	UKB
DOCK9	UKB
DOK1	UKB
DOK2	UKB
DPEP1	UKB
DPEP2	UKB
DPP10	UKB
DPP4	UKB
DPP7	UKB
DPT	UKB
DPY30	UKB
DRAXIN	UKB
DSC2	UKB
DSCAM	UKB
DSG2	UKB
DSG3	UKB
DTD1	UKB
DTYMK	UKB
DUSP13	UKB
DUSP3	UKB
DUT	UKB
DYNLT	UKB
DYNLT	UKB
EBAG9	UKB

EBI3	UKB
ECE1	UKB
ECHS1	UKB
ECI2	UKB
ECM1	UKB
EDA2R	UKB
EDAR	UKB
EDDM3	UKB
EDF1	UKB
EDIL3	UKB
EDN1	UKB
EEF1D	UKB
EFCAB	UKB
EFEMP	UKB
EFHD1	UKB
EFNA4	UKB
EFNB2	UKB
EGF	UKB
EGFLA	UKB
EGFR	UKB
EGLN1	UKB
EHBP1	UKB
EIF1AX	UKB
EIF4B	UKB
EIF4E	UKB
EIF4EB	UKB
EIF4G3	UKB
EIF5	UKB
EIF5A	UKB
ENAH	UKB
ENDOU	UKB
ENG	UKB
ENO1	UKB
ENO2	UKB
ENO3	UKB
ENOX2	UKB
ENPEP	UKB
ENPP2	UKB

ENPP5	UKB
ENPP6	UKB
ENPP7	UKB
ENSA	UKB
ENTPD2	UKB
ENTPD5	UKB
ENTPD6	UKB
EPHA1	UKB
EPHA10	UKB
EPHA2	UKB
EPHA4	UKB
EPHB4	UKB
EPHB6	UKB
EPO	UKB
ERBB2	UKB
ERBB3	UKB
ERBB4	UKB
ERCC1	UKB
EREG	UKB
ERMAP	UKB
ERN1	UKB
ERP29	UKB
ERP44	UKB
ESAM	UKB
ESM1	UKB
EVPL	UKB
EZR	UKB
F10	UKB
F11	UKB
F13B	UKB
F2	UKB
F3	UKB
F7	UKB
F9	UKB
FABP1	UKB
FABP2	UKB
FABP3	UKB
FABP4	UKB

FABP5	UKB
FABP6	UKB
FABP9	UKB
FADD	UKB
FAM171	UKB
FAM171	UKB
FAM172	UKB
FAM20A	UKB
FAM3B	UKB
FAM3D	UKB
FAP	UKB
FAS	UKB
FASLG	UKB
FBP1	UKB
FCAMR	UKB
FCAR	UKB
FCER1A	UKB
FCER2	UKB
FCGR2A	UKB
FCGR2B	UKB
FCGR3B	UKB
FCN1	UKB
FCN2	UKB
FCRL1	UKB
FCRL3	UKB
FCRL5	UKB
FCRL6	UKB
FCRLB	UKB
FEN1	UKB
FETUB	UKB
FGF12	UKB
FGF16	UKB
FGF19	UKB
FGF2	UKB
FGF20	UKB
FGF23	UKB
FGF3	UKB
FGF5	UKB

FGF6	UKB
FGF7	UKB
FGF9	UKB
FGFBP1	UKB
FGFBP3	UKB
FGFR2	UKB
FGFR4	UKB
FGL1	UKB
FGR	UKB
FH	UKB
FHIT	UKB
FIS1	UKB
FKBP14	UKB
FKBP1E	UKB
FKBP4	UKB
FKBP7	UKB
FLRT2	UKB
FLT1	UKB
FLT3	UKB
FLT3LG	UKB
FLT4	UKB
FMR1	UKB
FN1	UKB
FOLH1	UKB
FOLR1	UKB
FOLR2	UKB
FOLR3	UKB
FOXO3	UKB
FRZB	UKB
FSHB	UKB
FST	UKB
FSTL1	UKB
FSTL3	UKB
FTCD	UKB
FURIN	UKB
FUT3	UKB
FUT8	UKB
FXN	UKB

FZD10	UKB
GABAR	UKB
GABAR	UKB
GAD1	UKB
GADD4	UKB
GAGE2/	UKB
GAL	UKB
GALNT	UKB
GALNT.	UKB
GALNT.	UKB
GAPDH	UKB
GAS6	UKB
GBP1	UKB
GBP2	UKB
GBP6	UKB
GC	UKB
GCG	UKB
GCLM	UKB
GCNT1	UKB
GDF15	UKB
GDF2	UKB
GDNF	UKB
GFAP	UKB
GFER	UKB
GFRA1	UKB
GFRA2	UKB
GFRA3	UKB
GFRAL	UKB
GGA1	UKB
GGH	UKB
GH1	UKB
GH2	UKB
GHR	UKB
GHRL	UKB
GIP	UKB
GJA8	UKB
GKN1	UKB
GLA	UKB

GLB1	UKB
GLO1	UKB
GLP1R	UKB
GLRX	UKB
GLRX5	UKB
GM2A	UKB
GMFG	UKB
GMPR	UKB
GMPR2	UKB
GNAS	UKB
GNLY	UKB
GNPDA	UKB
GOT1	UKB
GP1BA	UKB
GP5	UKB
GP6	UKB
GPC1	UKB
GPC5	UKB
GPD1	UKB
GPHA2	UKB
GPI	UKB
GPNMB	UKB
GPR101	UKB
GRAP2	UKB
GRHPR	UKB
GRN	UKB
GRP	UKB
GRPEL1	UKB
GSN	UKB
GSR	UKB
GSTA1	UKB
GSTA3	UKB
GSTM4	UKB
GSTP1	UKB
GSTT2E	UKB
GUK1	UKB
GUSB	UKB
GZMA	UKB

GZMB	UKB
GZMH	UKB
HADH	UKB
HAGH	UKB
HAO1	UKB
HAVCR	UKB
HAVCR	UKB
HBEGF	UKB
HBQ1	UKB
HBZ	UKB
HDAC8	UKB
HDGF	UKB
HEBP1	UKB
HEPAC/	UKB
HEXIM:	UKB
HGF	UKB
HGFAC	UKB
HGS	UKB
HIF1A	UKB
HK2	UKB
HMBS	UKB
HMGCS	UKB
HMOX1	UKB
HMOX2	UKB
HNF1A	UKB
HNRNP:	UKB
HPCAL:	UKB
HPGDS	UKB
HPSE	UKB
HRAS	UKB
HRC	UKB
HRG	UKB
HS3ST3	UKB
HS6ST1	UKB
HS6ST2	UKB
HSD17E	UKB
HSP90B	UKB
HSPA1/	UKB

HSPB1	UKB
HSPB6	UKB
HSPG2	UKB
HTRA2	UKB
HYAL1	UKB
ICAM1	UKB
ICAM2	UKB
ICAM3	UKB
ICAM4	UKB
ICAM5	UKB
ICOSLG	UKB
IDI2	UKB
IDO1	UKB
IDO1	UKB
IDO1	UKB
IDO1	UKB
IDS	UKB
IDUA	UKB
IFIT3	UKB
IFNAR1	UKB
IFNG	UKB
IFNGR1	UKB
IFNGR2	UKB
IFNL1	UKB
IFNL2	UKB
IFNLR1	UKB
IFNW1	UKB
IGBP1	UKB
IGDCC3	UKB
IGDCC4	UKB
IGF1R	UKB
IGF2R	UKB
IGFBP1	UKB
IGFBP2	UKB
IGFBP3	UKB
IGFBP4	UKB
IGFBP6	UKB
IGFBP7	UKB

IGFBPL	UKB
IGLON5	UKB
IGSF3	UKB
IGSF8	UKB
IL10	UKB
IL10RA	UKB
IL10RB	UKB
IL11	UKB
IL12B	UKB
IL12RB1	UKB
IL12RB2	UKB
IL13	UKB
IL13RA1	UKB
IL15	UKB
IL15RA	UKB
IL16	UKB
IL17A	UKB
IL17C	UKB
IL17D	UKB
IL17F	UKB
IL17RA	UKB
IL17RB	UKB
IL18	UKB
IL18BP	UKB
IL18R1	UKB
IL18RA	UKB
IL19	UKB
IL1A	UKB
IL1B	UKB
IL1R1	UKB
IL1R2	UKB
IL1RAP	UKB
IL1RL1	UKB
IL1RL2	UKB
IL1RN	UKB
IL2	UKB
IL20	UKB
IL20RA	UKB

IL20RB	UKB
IL21R	UKB
IL22	UKB
IL22RA	UKB
IL24	UKB
IL25	UKB
IL2RA	UKB
IL2RB	UKB
IL2RG	UKB
IL3	UKB
IL31	UKB
IL31RA	UKB
IL32	UKB
IL34	UKB
IL36A	UKB
IL36G	UKB
IL3RA	UKB
IL4	UKB
IL4R	UKB
IL5	UKB
IL5RA	UKB
IL6	UKB
IL6	UKB
IL6	UKB
IL6	UKB
IL6R	UKB
IL6ST	UKB
IL7	UKB
IL7R	UKB
IL9	UKB
IMPA1	UKB
ING1	UKB
INHBB	UKB
INHBC	UKB
INSL3	UKB
INSL4	UKB
INSL5	UKB
INSR	UKB

IRAK4	UKB
ISLR2	UKB
ISM1	UKB
IST1	UKB
ITGA2	UKB
ITGA5	UKB
ITGAL	UKB
ITGB2	UKB
ITGB5	UKB
ITGB6	UKB
ITGB7	UKB
ITIH1	UKB
ITIH3	UKB
ITIH4	UKB
ITIH5	UKB
ITM2A	UKB
ITPA	UKB
IVD	UKB
JAM2	UKB
JAM3	UKB
JUN	UKB
KAZAL	UKB
KCNIP4	UKB
KCTD5	UKB
KDR	UKB
KEL	UKB
KIAA15	UKB
KIAA20	UKB
KIF1C	UKB
KIF22	UKB
KIR2DL	UKB
KIR2DL	UKB
KIR2DS	UKB
KIR3DL	UKB
KIR3DL	UKB
KIRREL	UKB
KIT	UKB
KITLG	UKB

KLB	UKB
KLF4	UKB
KLK10	UKB
KLK11	UKB
KLK12	UKB
KLK13	UKB
KLK14	UKB
KLK15	UKB
KLK3	UKB
KLK4	UKB
KLK6	UKB
KLK7	UKB
KLK8	UKB
KLKB1	UKB
KLRB1	UKB
KLRC1	UKB
KLRF1	UKB
KLRK1	UKB
KRT17	UKB
KRT18	UKB
KRT19	UKB
KRT5	UKB
KYNU	UKB
L1CAM	UKB
LACRT	UKB
LAG3	UKB
LAIR1	UKB
LAIR2	UKB
LAMA4	UKB
LAMP1	UKB
LAP3	UKB
LAT	UKB
LAT2	UKB
LAYN	UKB
LBP	UKB
LCAT	UKB
LCN2	UKB
LCP1	UKB

LDLR	UKB
LDLRAI	UKB
LECT2	UKB
LEFTY2	UKB
LEG1	UKB
LEP	UKB
LEPR	UKB
LGALS1	UKB
LGALS3	UKB
LGALS3	UKB
LGALS4	UKB
LGALS7	UKB
LGALS8	UKB
LGALS9	UKB
LGMN	UKB
LHB	UKB
LIF	UKB
LIFR	UKB
LILRA2	UKB
LILRA3	UKB
LILRA4	UKB
LILRA5	UKB
LILRA6	UKB
LILRB1	UKB
LILRB2	UKB
LILRB4	UKB
LILRB5	UKB
LMNB1	UKB
LMOD1	UKB
LMOD1	UKB
LMOD1	UKB
LMOD1	UKB
LONP1	UKB
LPCAT2	UKB
LPO	UKB
LRCH4	UKB
LRFN2	UKB
LRIG1	UKB

LRIG3	UKB
LRP1	UKB
LRP11	UKB
LRP2	UKB
LRPAP1	UKB
LRRC25	UKB
LRRC37	UKB
LRRC59	UKB
LRRN1	UKB
LRTM1	UKB
LRTM2	UKB
LSM1	UKB
LTA	UKB
LTA4H	UKB
LTBR	UKB
LY75	UKB
LY9	UKB
LYN	UKB
LYPD1	UKB
LYPD3	UKB
LYSMD	UKB
LYVE1	UKB
LYZL2	UKB
MAD1L	UKB
MAGEA	UKB
MAMD(	UKB
MAN1A	UKB
MAN2B	UKB
MANF	UKB
MANSC	UKB
MANSC	UKB
MAP1L(	UKB
MAP2K	UKB
MAP2K	UKB
MAPK1	UKB
MAPK9	UKB
MAPKA	UKB
MAPRE	UKB

MAPT	UKB
MARCC	UKB
MASP1	UKB
MATN2	UKB
MATN3	UKB
MAX	UKB
MB	UKB
MBL2	UKB
MCEE	UKB
MCEMF	UKB
MCTS1	UKB
MDGA1	UKB
MDH1	UKB
MDK	UKB
MDM1	UKB
MEGF1(	UKB
MENT	UKB
MEPE	UKB
MET	UKB
METAP	UKB
METAP.	UKB
MFAP3I	UKB
MFAP4	UKB
MFAP5	UKB
MFGE8	UKB
MIA	UKB
MICALI	UKB
MICB	UKB
MIF	UKB
MILR1	UKB
MLN	UKB
MMP1	UKB
MMP10	UKB
MMP12	UKB
MMP13	UKB
MMP3	UKB
MMP7	UKB
MMP8	UKB

MMP9	UKB
MOG	UKB
MORF4:	UKB
MPO	UKB
MRC1	UKB
MRPL52	UKB
MSLN	UKB
MSMB	UKB
MSR1	UKB
MSRA	UKB
MST1	UKB
MSTN	UKB
MTHFD	UKB
MTHFS:	UKB
MTIF3	UKB
MUC16	UKB
MVK	UKB
MXRA8	UKB
MYBPC	UKB
MYDGF	UKB
MYL3	UKB
MYL6B	UKB
MYO6	UKB
MYOC	UKB
MYOM2	UKB
MYOM3	UKB
NAA10	UKB
NAAA	UKB
NADK	UKB
NAGK	UKB
NAGPA	UKB
NAMPT	UKB
NAP1L4	UKB
NBL1	UKB
NCAM1	UKB
NCAM2	UKB
NCAN	UKB
NCF2	UKB

NCK2	UKB
NCR1	UKB
NCR3L(	UKB
NDRG1	UKB
NDST1	UKB
NDUFA	UKB
NEDD9	UKB
NEFL	UKB
NEK7	UKB
NELL1	UKB
NELL2	UKB
NENF	UKB
NEO1	UKB
NFASC	UKB
NFATC:	UKB
NFKB1	UKB
NFU1	UKB
NGF	UKB
NGFR	UKB
NGRN	UKB
NHLRC	UKB
NID1	UKB
NID2	UKB
NIT2	UKB
NLGN1	UKB
NLGN2	UKB
NME1	UKB
NMI	UKB
NMNAT	UKB
NMT1	UKB
NOTCH	UKB
NOTCH	UKB
NOTCH	UKB
NPDC1	UKB
NPL	UKB
NPM1	UKB
NPPB	UKB
NPTN	UKB

NPTX1	UKB
NPTX2	UKB
NPTXR	UKB
NRCAM	UKB
NRGN	UKB
NRP1	UKB
NRP2	UKB
NRXN3	UKB
NSFL1C	UKB
NT5C	UKB
NT5C3A	UKB
NT5E	UKB
NTF3	UKB
NTF4	UKB
NTRK2	UKB
NTRK3	UKB
NUDC	UKB
NUDT2	UKB
NUDT5	UKB
NXPH1	UKB
NXPH3	UKB
OBP2B	UKB
OGN	UKB
OLFM4	UKB
OLR1	UKB
OMD	UKB
OMG	UKB
OMP	UKB
OPHN1	UKB
OPTC	UKB
OSCAR	UKB
OSM	UKB
OSMR	UKB
OSTN	UKB
OXT	UKB
P4HB	UKB
PAEP	UKB
PAFAH1	UKB

PAFAH:	UKB
PAK4	UKB
PALM	UKB
PAM	UKB
PAPPA	UKB
PARK7	UKB
PARP1	UKB
PBK	UKB
PCBD1	UKB
PCDH9	UKB
PCNA	UKB
PCOLCI	UKB
PCSK7	UKB
PCSK9	UKB
PDAP1	UKB
PDCD1	UKB
PDCD1I	UKB
PDCD5	UKB
PDCD6	UKB
PDCL2	UKB
PDE4D	UKB
PDE5A	UKB
PDGFA	UKB
PDGFB	UKB
PDGFC	UKB
PDGFR/	UKB
PDGFRI	UKB
PDIA3	UKB
PDIA4	UKB
PDIA5	UKB
PDRG1	UKB
PDZK1	UKB
PEAR1	UKB
PEBP1	UKB
PECAM	UKB
PECR	UKB
PENK	UKB
PF4	UKB

PFDN2	UKB
PGD	UKB
PGF	UKB
PGLYR1	UKB
PGLYR1	UKB
PGM2	UKB
PGR	UKB
PI3	UKB
PIGR	UKB
PILRA	UKB
PKD2	UKB
PKLR	UKB
PLA2G1	UKB
PLA2G1	UKB
PLA2G2	UKB
PLA2G7	UKB
PLAT	UKB
PLAU	UKB
PLAUR	UKB
PLCB1	UKB
PLG	UKB
PLIN3	UKB
PLSCR3	UKB
PLTP	UKB
PLXDC1	UKB
PLXDC2	UKB
PLXNA4	UKB
PLXNB2	UKB
PLXNB3	UKB
PMM2	UKB
PMVK	UKB
PNLIP	UKB
PNLIPR	UKB
PNLIPR	UKB
PODXL2	UKB
POMC	UKB
PON1	UKB
POSTN	UKB

PPBP	UKB
PPCDC	UKB
PPIB	UKB
PPIE	UKB
PPIF	UKB
PPM1A	UKB
PPME1	UKB
PPP1CC	UKB
PPP1R1·	UKB
PPP1R2	UKB
PPP2R5.	UKB
PPP3R1	UKB
PPT1	UKB
PPY	UKB
PQBP1	UKB
PRC1	UKB
PRCP	UKB
PRDX1	UKB
PRDX3	UKB
PRDX5	UKB
PRDX6	UKB
PRG2	UKB
PRG3	UKB
PRKAR:	UKB
PRKCQ	UKB
PRKG1	UKB
PRL	UKB
PRND	UKB
PROC	UKB
PROK1	UKB
PROS1	UKB
PRSS2	UKB
PRSS22	UKB
PRSS27	UKB
PRSS8	UKB
PRTN3	UKB
PSAP	UKB
PSAPL1	UKB

PSG1	UKB
PSIP1	UKB
PSMA1	UKB
PSMD5	UKB
PSMD9	UKB
PSME1	UKB
PSME2	UKB
PSMG3	UKB
PSPN	UKB
PTEN	UKB
PTGDS	UKB
PTGES2	UKB
PTGR1	UKB
PTH	UKB
PTH1R	UKB
PTK7	UKB
PTN	UKB
PTP4A3	UKB
PTPN1	UKB
PTPN6	UKB
PTPN9	UKB
PTPRH	UKB
PTPRR	UKB
PTPRS	UKB
PTS	UKB
PTTG1	UKB
PTX3	UKB
PVR	UKB
PXDNL	UKB
PYDC1	UKB
PYY	UKB
PZP	UKB
QDPR	UKB
QPCT	UKB
RAB27F	UKB
RAB2B	UKB
RAB39F	UKB
RAB6B	UKB

RABEPI	UKB
RAC3	UKB
RAD23I	UKB
RAD51	UKB
RALB	UKB
RANBP	UKB
RARRE	UKB
RARRE	UKB
RASA1	UKB
RBKS	UKB
RBM17	UKB
RBM19	UKB
RBP1	UKB
RBP5	UKB
RBP7	UKB
REG1A	UKB
REG1B	UKB
REG3A	UKB
REG3G	UKB
REG4	UKB
RELB	UKB
RELT	UKB
REN	UKB
RET	UKB
RETN	UKB
REXO2	UKB
RGMA	UKB
RGMB	UKB
RGS10	UKB
RGS8	UKB
RHOC	UKB
RLN2	UKB
RNASE	UKB
RNASE	UKB
RNASE	UKB
RNASE	UKB
RNASE	UKB
RNASE	UKB

RNF149	UKB
RNF41	UKB
RNF43	UKB
ROBO1	UKB
ROBO2	UKB
ROBO4	UKB
ROR1	UKB
RPA2	UKB
RPE	UKB
RPS10	UKB
RRAS	UKB
RRM2	UKB
RRM2B	UKB
RSPO1	UKB
RSPO3	UKB
RTN4IP	UKB
RTN4R	UKB
RUVBL	UKB
S100A1	UKB
S100A1	UKB
S100A1	UKB
S100A1	UKB
S100A1	UKB
S100A3	UKB
S100A4	UKB
S100P	UKB
SAA4	UKB
SAP18	UKB
SAT1	UKB
SAT2	UKB
SATB1	UKB
SBSN	UKB
SCARA	UKB
SCARB	UKB
SCARF1	UKB
SCARF	UKB
SCG3	UKB
SCGB1	UKB

SCGB3/	UKB
SCGN	UKB
SCIN	UKB
SCN2B	UKB
SCN4B	UKB
SCT	UKB
SELE	UKB
SELL	UKB
SELP	UKB
SELPLG	UKB
SEMA3(	UKB
SEMA4(	UKB
SEMA4]	UKB
SEMA6(	UKB
SEMA7/	UKB
SERPIN	UKB
SERPIN	UKB
SERPIN	UKB
SERPIN	UKB
SERPIN	UKB
SERPIN	UKB
SERPIN	UKB
SERPIN	UKB
SERPIN	UKB
SERPIN	UKB
SERPIN	UKB
SERPIN	UKB
SERPIN	UKB
SERPIN	UKB
SERPIN	UKB
SERPIN	UKB
SERPIN	UKB
SERPIN	UKB
SERPIN	UKB
SERPIN	UKB
SERPIN	UKB
SETMA	UKB
SEZ6L	UKB

SEZ6L2	UKB
SF3B4	UKB
SFRP1	UKB
SFRP4	UKB
SFTPD	UKB
SH2D1A	UKB
SH3BGI	UKB
SH3GL3	UKB
SH3GLI	UKB
SHBG	UKB
SHC1	UKB
SHH	UKB
SHMT1	UKB
SIAE	UKB
SIGLEC	UKB
SIGLEC	UKB
SIGLEC	UKB
SIGLEC	UKB
SIGLEC	UKB
SIGLEC	UKB
SIGLEC	UKB
SIRPA	UKB
SIRPB1	UKB
SIRT2	UKB
SIRT5	UKB
SIT1	UKB
SLAMF	UKB
SLAMF	UKB
SLAMF	UKB
SLAMF	UKB
SLIT2	UKB
SLITRK	UKB
SLITRK	UKB
SLMAP	UKB
SMAD1	UKB
SMAD2	UKB
SMAD3	UKB
SMC3	UKB

SMNDC	UKB
SMOC1	UKB
SMOC2	UKB
SMPD1	UKB
SMPDL:	UKB
SMS	UKB
SMTN	UKB
SNAP25	UKB
SNAP29	UKB
SNAPIN	UKB
SNCA	UKB
SNRPB2	UKB
SNX5	UKB
SOD1	UKB
SOD2	UKB
SOD3	UKB
SORCS2	UKB
SORD	UKB
SORT1	UKB
SOST	UKB
SOX2	UKB
SPACA5	UKB
SPARC	UKB
SPARCI	UKB
SPINK1	UKB
SPINK2	UKB
SPINK4	UKB
SPINK5	UKB
SPINK6	UKB
SPINK8	UKB
SPINT1	UKB
SPINT2	UKB
SPINT3	UKB
SPOCK1	UKB
SPON1	UKB
SPON2	UKB
SPP1	UKB
SPTLC1	UKB

SRC	UKB
SRP14	UKB
SSB	UKB
ST13	UKB
ST3GAI	UKB
ST6GAI	UKB
STAB2	UKB
STAMB	UKB
STAT5E	UKB
STAU1	UKB
STC1	UKB
STC2	UKB
STIP1	UKB
STK24	UKB
STOML	UKB
STX1B	UKB
STX3	UKB
STX4	UKB
STX6	UKB
STX7	UKB
STX8	UKB
SUGT1	UKB
SULT2A	UKB
SUMF1	UKB
SUMF2	UKB
SUOX	UKB
SUSD1	UKB
SV2A	UKB
SWAP7(	UKB
SYTL4	UKB
TACSTI	UKB
TAGLN	UKB
TALDO	UKB
TANK	UKB
TARBP2	UKB
TAX1BI	UKB
TBC1D5	UKB
TBCA	UKB

TBCB	UKB
TCL1A	UKB
TCL1B	UKB
TCN1	UKB
TCN2	UKB
TCP11	UKB
TDGF1	UKB
TDO2	UKB
TDP1	UKB
TEK	UKB
TERF1	UKB
TF	UKB
TFF1	UKB
TFF2	UKB
TFF3	UKB
TFPI	UKB
TFPI2	UKB
TFRC	UKB
TG	UKB
TGFB1	UKB
TGFB2	UKB
TGFBI	UKB
TGFBR2	UKB
TGFBR3	UKB
TGM2	UKB
THBS2	UKB
THBS4	UKB
THPO	UKB
THSD1	UKB
THTPA	UKB
TIE1	UKB
TIGAR	UKB
TIGIT	UKB
TIMD4	UKB
TIMM8A	UKB
TIMP1	UKB
TIMP2	UKB
TIMP3	UKB

TIMP4	UKB
TINAGI	UKB
TK1	UKB
TLR1	UKB
TLR2	UKB
TLR3	UKB
TLR4	UKB
TMCO5.	UKB
TMED1(	UKB
TMED4	UKB
TMEM1	UKB
TMEM1	UKB
TMEM2	UKB
TMPRS!	UKB
TMPRS!	UKB
TMPRS!	UKB
TMPRS!	UKB
TNC	UKB
TNF	UKB
TNF	UKB
TNF	UKB
TNF	UKB
TNFAIP	UKB
TNFRSI	UKB
TNFRSI	UKB
TNFRSI	UKB
TNFRSI	UKB
TNFRSI	UKB
TNFRSI	UKB
TNFRSI	UKB
TNFRSI	UKB
TNFRSI	UKB
TNFRSI	UKB
TNFRSI	UKB
TNFRSI	UKB
TNFRSI	UKB
TNFRSI	UKB
TNFRSI	UKB

TNFRSF	UKB
TNFSF1	UKB
TNFSF1	UKB
TNFSF1	UKB
TNFSF1	UKB
TNFSF1	UKB
TNFSF8	UKB
TNNI3	UKB
TNR	UKB
TNXB	UKB
TOP1	UKB
TOR1AI	UKB
TP53	UKB
TP53I3	UKB
TPK1	UKB
TPMT	UKB
TPP1	UKB
TPPP2	UKB
TPRKB	UKB
TPSAB1	UKB
TPSG1	UKB
TPT1	UKB
TRDMT	UKB
TREM2	UKB
TREML	UKB
TREML	UKB
TRIAP1	UKB
TSLP	UKB
TST	UKB
TTN	UKB
TXLNA	UKB
TXN	UKB
TXNDC	UKB
TXNDC	UKB
TXNRD	UKB
TYMP	UKB
TYRO3	UKB
UBE2B	UKB

UBE2L6	UKB
UGDH	UKB
ULBP2	UKB
UMOD	UKB
UNC5D	UKB
UROD	UKB
UROS	UKB
USO1	UKB
USP25	UKB
USP28	UKB
USP8	UKB
UXS1	UKB
VAMP8	UKB
VASN	UKB
VASP	UKB
VAT1	UKB
VAV3	UKB
VCAM1	UKB
VCAN	UKB
VEGFA	UKB
VEGFB	UKB
VEGFC	UKB
VIM	UKB
VIT	UKB
VNN2	UKB
VPS28	UKB
VPS4B	UKB
VSIG10	UKB
VSIG2	UKB
VSNL1	UKB
VSTM2L	UKB
VTA1	UKB
VTI1A	UKB
VWA1	UKB
VWC2	UKB
VWC2L	UKB
VWF	UKB
WARS	UKB

WFDC1	UKB
WFDC2	UKB
WFIKKI	UKB
WFIKKI	UKB
WIF1	UKB
XCL1	UKB
XG	UKB
XRCC4	UKB
YES1	UKB
YWHA(	UKB
YY1	UKB
ZNF174	UKB
ZNF75D	UKB
ZP4	UKB
A1BG	deCODE
ABHD1	deCODE
ABL1	deCODE
ABO	deCODE
ACAA1	deCODE
ACADM	deCODE
ACADS	deCODE
ACAN	deCODE
ACE	deCODE
ACE2	deCODE
ACHE	deCODE
ACOT1	deCODE
ACP1	deCODE
ACP5	deCODE
ACP6	deCODE
ACRV1	deCODE
ACRV1	deCODE
ACTN2	deCODE
ACVRL	deCODE
ACY1	deCODE
ADAM1	deCODE
ADAM1	deCODE
ADAM1	deCODE
ADAM2	deCODE

ADAM2	deCODE
ADAM9	deCODE
ADAMT	deCODE
ADAMT	deCODE
ADAMT	deCODE
ADAMT	deCODE
ADAMT	deCODE
ADGRE	deCODE
ADGRF	deCODE
ADH1B	deCODE
ADH4	deCODE
ADIPOC	deCODE
ADM	deCODE
ADM	deCODE
AFM	deCODE
AFP	deCODE
AGER	deCODE
AGR2	deCODE
AGR3	deCODE
AGRN	deCODE
AGRP	deCODE
AGT	deCODE
AHSA1	deCODE
AHSG	deCODE
AHSP	deCODE
AIDA	deCODE
AIF1	deCODE
AIF1L	deCODE
AIFM1	deCODE
AK1	deCODE
AK2	deCODE
AKR1B	deCODE
AKR1B	deCODE
AKR1C	deCODE
AKT2	deCODE
AKT2	deCODE
ALCAM	deCODE
ALDH1	deCODE

ALDH2	deCODE
ALDH3	deCODE
ALDH5	deCODE
ALPI	deCODE
AMBP	deCODE
AMIGO	deCODE
AMIGO	deCODE
AMN	deCODE
AMY1A	deCODE
AMY2A	deCODE
AMY2B	deCODE
ANG	deCODE
ANGPT	deCODE
ANGPT	deCODE
ANGPT	deCODE
ANGPT	deCODE
ANGPT	deCODE
ANGPT	deCODE
ANGPT	deCODE
ANK2	deCODE
ANKRD	deCODE
ANPEP	deCODE
ANXA1	deCODE
ANXA1	deCODE
ANXA1	deCODE
ANXA2	deCODE
ANXA2	deCODE
ANXA3	deCODE
ANXA4	deCODE
ANXA5	deCODE
AOC1	deCODE
AP1G2	deCODE
APCS	deCODE
APEX1	deCODE
APLP1	deCODE
APOA1	deCODE
APOA2	deCODE
APOA4	deCODE

APOB	deCODE
APOC1	deCODE
APOD	deCODE
APOE	deCODE
APOF	deCODE
APOH	deCODE
APOL1	deCODE
APOL1	deCODE
APOM	deCODE
APP	deCODE
APRT	deCODE
ARAF	deCODE
AREG	deCODE
ARF6	deCODE
ARFIP1	deCODE
ARG1	deCODE
ARG2	deCODE
ARHGA	deCODE
ARHGA	deCODE
ARHGA	deCODE
ARHGA	deCODE
ARHGA	deCODE
ARHGE	deCODE
ARHGE	deCODE
ARID3A	deCODE
ARL2BI	deCODE
ARSA	deCODE
ARSB	deCODE
ART3	deCODE
ARTN	deCODE
ASAH1	deCODE
ASAH2	deCODE
ASGR1	deCODE
ASGR2	deCODE
ASPN	deCODE
ASRGL1	deCODE
ATOX1	deCODE
ATP1B1	deCODE

ATP1B2	deCODE
ATP1B3	deCODE
ATP1B4	deCODE
ATRN	deCODE
ATXN3	deCODE
AXL	deCODE
AZU1	deCODE
B2M	deCODE
B3GAT3	deCODE
B4GAL7	deCODE
BACH1	deCODE
BAG3	deCODE
BAG4	deCODE
BAIAP2	deCODE
BAMBI	deCODE
BATF	deCODE
BCAM	deCODE
BCAN	deCODE
BCAT2	deCODE
BCHE	deCODE
BCL2	deCODE
BCL2L1	deCODE
BCL2L1	deCODE
BDNF	deCODE
BDNF	deCODE
BECN1	deCODE
BGLAP	deCODE
BGN	deCODE
BHMT2	deCODE
BID	deCODE
BIRC2	deCODE
BLMH	deCODE
BLNK	deCODE
BLVRB	deCODE
BMP10	deCODE
BMP4	deCODE
BMP4	deCODE
BMP6	deCODE

BMPER	deCODE
BNIP3L	deCODE
BOC	deCODE
BOLA1	deCODE
BOLA2	deCODE
BPIFA2	deCODE
BPIFB1	deCODE
BRD1	deCODE
BRD2	deCODE
BRD2	deCODE
BRDT	deCODE
BRSK2	deCODE
BSG	deCODE
BST1	deCODE
BST2	deCODE
BTC	deCODE
BTD	deCODE
BTLA	deCODE
BTN2A1	deCODE
C1GAL1	deCODE
C1QBP	deCODE
C1QL2	deCODE
C1QTNI	deCODE
C1QTNI	deCODE
C1QTNI	deCODE
C1R	deCODE
C1RL	deCODE
C1S	deCODE
C2	deCODE
C3	deCODE
C3	deCODE
C3	deCODE
C3	deCODE
C3	deCODE
C4BPB	deCODE
C5	deCODE
C5	deCODE
C7	deCODE

C9	deCODE
CA1	deCODE
CA11	deCODE
CA12	deCODE
CA13	deCODE
CA2	deCODE
CA3	deCODE
CA4	deCODE
CA5A	deCODE
CA6	deCODE
CA7	deCODE
CA8	deCODE
CA9	deCODE
CACYB	deCODE
CALB1	deCODE
CALB2	deCODE
CALCB	deCODE
CALCO	deCODE
CALY	deCODE
CAMKK	deCODE
CANT1	deCODE
CAPG	deCODE
CAPN3	deCODE
CARHS	deCODE
CASP10	deCODE
CASP2	deCODE
CASP3	deCODE
CASP8	deCODE
CASQ2	deCODE
CAT	deCODE
CBLN1	deCODE
CBLN4	deCODE
CBS	deCODE
CCDC1	deCODE
CCDC5	deCODE
CCDC8	deCODE
CCL11	deCODE
CCL13	deCODE

CCL14	deCODE
CCL15	deCODE
CCL16	deCODE
CCL17	deCODE
CCL18	deCODE
CCL19	deCODE
CCL2	deCODE
CCL20	deCODE
CCL21	deCODE
CCL22	deCODE
CCL23	deCODE
CCL23	deCODE
CCL24	deCODE
CCL25	deCODE
CCL26	deCODE
CCL27	deCODE
CCL28	deCODE
CCL3	deCODE
CCL5	deCODE
CCL7	deCODE
CCL8	deCODE
CCS	deCODE
CCT5	deCODE
CD109	deCODE
CD14	deCODE
CD14	deCODE
CD160	deCODE
CD163	deCODE
CD164L	deCODE
CD177	deCODE
CD2	deCODE
CD200	deCODE
CD200R	deCODE
CD207	deCODE
CD209	deCODE
CD226	deCODE
CD244	deCODE
CD248	deCODE

CD27	deCODE
CD274	deCODE
CD300A	deCODE
CD300C	deCODE
CD300E	deCODE
CD300L	deCODE
CD33	deCODE
CD36	deCODE
CD38	deCODE
CD3E	deCODE
CD3E	deCODE
CD3G	deCODE
CD4	deCODE
CD40LC	deCODE
CD46	deCODE
CD48	deCODE
CD5	deCODE
CD5	deCODE
CD55	deCODE
CD58	deCODE
CD59	deCODE
CD5L	deCODE
CD63	deCODE
CD69	deCODE
CD7	deCODE
CD70	deCODE
CD72	deCODE
CD74	deCODE
CD74	deCODE
CD79B	deCODE
CD80	deCODE
CD83	deCODE
CD86	deCODE
CD8A	deCODE
CD93	deCODE
CD99L2	deCODE
CDC25A	deCODE
CDC37	deCODE

CDC42I	deCODE
CDCP1	deCODE
CDCP1	deCODE
CDH1	deCODE
CDH15	deCODE
CDH15	deCODE
CDH17	deCODE
CDH2	deCODE
CDH3	deCODE
CDH5	deCODE
CDH6	deCODE
CDHR1	deCODE
CDHR5	deCODE
CDKN1.	deCODE
CDKN2	deCODE
CDNF	deCODE
CDON	deCODE
CDSN	deCODE
CEACA	deCODE
CEACA	deCODE
CEACA	deCODE
CEACA	deCODE
CEACA	deCODE
CEBPA	deCODE
CEBPB	deCODE
CELA2/	deCODE
CES1	deCODE
CETN2	deCODE
CETN3	deCODE
CFB	deCODE
CFC1	deCODE
CFD	deCODE
CFH	deCODE
CFHR2	deCODE
CFHR4	deCODE
CFHR5	deCODE
CFI	deCODE
CFP	deCODE

CGA	deCODE
CGREF1	deCODE
CHAD	deCODE
CHCHD	deCODE
CHEK2	deCODE
CHGA	deCODE
CHGB	deCODE
CHI3L1	deCODE
CHIT1	deCODE
CHL1	deCODE
CHP1	deCODE
CHRDL	deCODE
CHRDL	deCODE
CILP	deCODE
CINP	deCODE
CIRBP	deCODE
CKAP4	deCODE
CKB	deCODE
CKMT1	deCODE
CLC	deCODE
CLEC10	deCODE
CLEC11	deCODE
CLEC11	deCODE
CLEC12	deCODE
CLEC1B	deCODE
CLEC2D	deCODE
CLEC3B	deCODE
CLEC4A	deCODE
CLEC4C	deCODE
CLEC4D	deCODE
CLEC4G	deCODE
CLEC4M	deCODE
CLEC6A	deCODE
CLEC7A	deCODE
CLGN	deCODE
CLIC5	deCODE
CLINT1	deCODE
CLMP	deCODE

CLMP	deCODE
CLPP	deCODE
CLPS	deCODE
CLSTN1	deCODE
CLSTN2	deCODE
CLSTN3	deCODE
CLTA	deCODE
CLU	deCODE
CNDP1	deCODE
CNP	deCODE
CNPY4	deCODE
CNTF	deCODE
CNTN1	deCODE
CNTN2	deCODE
CNTN4	deCODE
CNTN5	deCODE
CNTNA	deCODE
COCH	deCODE
COL15A	deCODE
COL18A	deCODE
COL1A1	deCODE
COL1A1	deCODE
COL28A	deCODE
COL2A1	deCODE
COL2A1	deCODE
COL3A1	deCODE
COL6A3	deCODE
COL6A3	deCODE
COL9A1	deCODE
COL9A1	deCODE
COLEC1	deCODE
COMMI	deCODE
COMMI	deCODE
COMP	deCODE
COMT	deCODE
COPE	deCODE
COX5B	deCODE
CPA2	deCODE

CPA4	deCODE
CPB1	deCODE
CPB2	deCODE
CPE	deCODE
CPLX2	deCODE
CPM	deCODE
CPOX	deCODE
CPQ	deCODE
CPVL	deCODE
CPXM1	deCODE
CR1	deCODE
CR2	deCODE
CRADD	deCODE
CREG1	deCODE
CRELD	deCODE
CRH	deCODE
CRH	deCODE
CRHBP	deCODE
CRIM1	deCODE
CRIM1	deCODE
CRIP2	deCODE
CRISP2	deCODE
CRISP3	deCODE
CRKL	deCODE
CRLF1	deCODE
CRNN	deCODE
CRTAC	deCODE
CRTAM	deCODE
CRYBB	deCODE
CRYBB	deCODE
CRYGD	deCODE
CRYZL	deCODE
CSDE1	deCODE
CSF1	deCODE
CSF1R	deCODE
CSF2	deCODE
CSF2RA	deCODE
CSF2RE	deCODE

CSF2RB	deCODE
CSF3	deCODE
CSF3R	deCODE
CSNK1I	deCODE
CSNK2/	deCODE
CSNK2/	deCODE
CSPG4	deCODE
CSRP3	deCODE
CST1	deCODE
CST3	deCODE
CST5	deCODE
CST6	deCODE
CST7	deCODE
CTF1	deCODE
CTHRC1	deCODE
CTLA4	deCODE
CTRB1	deCODE
CTRC	deCODE
CTRL	deCODE
CTSB	deCODE
CTSC	deCODE
CTSD	deCODE
CTSE	deCODE
CTSF	deCODE
CTSH	deCODE
CTSL	deCODE
CTSO	deCODE
CTSS	deCODE
CTSV	deCODE
CTSZ	deCODE
CUZD1	deCODE
CX3CL1	deCODE
CXADR	deCODE
CXCL1	deCODE
CXCL1(	deCODE
CXCL11	deCODE
CXCL12	deCODE
CXCL13	deCODE

CXCL14	deCODE
CXCL16	deCODE
CXCL17	deCODE
CXCL5	deCODE
CXCL6	deCODE
CXCL8	deCODE
CXCL9	deCODE
CXCL9	deCODE
CYB5A	deCODE
CYB5R2	deCODE
CYTL1	deCODE
DAG1	deCODE
DAPK2	deCODE
DAPP1	deCODE
DBI	deCODE
DBNL	deCODE
DCN	deCODE
DCTD	deCODE
DCTN2	deCODE
DCTN6	deCODE
DCTPP1	deCODE
DCUN1	deCODE
DCUN1	deCODE
DCXR	deCODE
DDAH1	deCODE
DDC	deCODE
DDR1	deCODE
DDT	deCODE
DDX25	deCODE
DDX58	deCODE
DECR1	deCODE
DEFA1	deCODE
DEFB10	deCODE
DEFB10	deCODE
DEFB11	deCODE
DEFB11	deCODE
DEFB4A	deCODE
DENR	deCODE

DGCR6	deCODE
DHPS	deCODE
DIABLO	deCODE
DKK1	deCODE
DKK3	deCODE
DKK4	deCODE
DKKL1	deCODE
DLG4	deCODE
DLK1	deCODE
DLK1	deCODE
DLL1	deCODE
DLL4	deCODE
DNAJA1	deCODE
DNAJA4	deCODE
DNAJB1	deCODE
DNAJB1	deCODE
DNAJB2	deCODE
DNAJB6	deCODE
DNER	deCODE
DNER	deCODE
DOC2B	deCODE
DOCK9	deCODE
DOK1	deCODE
DOK2	deCODE
DOK2	deCODE
DPEP1	deCODE
DPEP2	deCODE
DPP10	deCODE
DPP4	deCODE
DPP7	deCODE
DPT	deCODE
DPY30	deCODE
DRAXIN	deCODE
DSC2	deCODE
DSCAM	deCODE
DSG2	deCODE
DSG3	deCODE
DTD1	deCODE

DTYMK	deCODE
DUSP13	deCODE
DUSP3	deCODE
DUT	deCODE
DYNLT	deCODE
DYNLT	deCODE
EBAG9	deCODE
EBI3	deCODE
ECE1	deCODE
ECHS1	deCODE
ECI2	deCODE
ECM1	deCODE
EDA2R	deCODE
EDAR	deCODE
EDDM3	deCODE
EDF1	deCODE
EDIL3	deCODE
EDN1	deCODE
EEF1D	deCODE
EFCAB	deCODE
EFEMP	deCODE
EFHD1	deCODE
EFNA4	deCODE
EFNB2	deCODE
EFNB2	deCODE
EGF	deCODE
EGFLA	deCODE
EGFR	deCODE
EGFR	deCODE
EGLN1	deCODE
EHBP1	deCODE
EIF1AX	deCODE
EIF4B	deCODE
EIF4E	deCODE
EIF4EB	deCODE
EIF4G3	deCODE
EIF5	deCODE
EIF5A	deCODE

ENAH	deCODE
ENDOU	deCODE
ENG	deCODE
ENO1	deCODE
ENO2	deCODE
ENO3	deCODE
ENOX2	deCODE
ENPEP	deCODE
ENPP2	deCODE
ENPP5	deCODE
ENPP6	deCODE
ENPP7	deCODE
ENSA	deCODE
ENTPD2	deCODE
ENTPD5	deCODE
ENTPD6	deCODE
EPHA1	deCODE
EPHA10	deCODE
EPHA2	deCODE
EPHA4	deCODE
EPHB4	deCODE
EPHB6	deCODE
EPO	deCODE
ERBB2	deCODE
ERBB3	deCODE
ERBB4	deCODE
ERCC1	deCODE
EREG	deCODE
ERMAP	deCODE
ERN1	deCODE
ERP29	deCODE
ERP44	deCODE
ESAM	deCODE
ESM1	deCODE
EVPL	deCODE
EZR	deCODE
F10	deCODE
F10	deCODE

F11	deCODE
F13B	deCODE
F2	deCODE
F2	deCODE
F3	deCODE
F7	deCODE
F9	deCODE
F9	deCODE
FABP1	deCODE
FABP2	deCODE
FABP3	deCODE
FABP4	deCODE
FABP5	deCODE
FABP6	deCODE
FABP9	deCODE
FADD	deCODE
FAM171	deCODE
FAM171	deCODE
FAM171	deCODE
FAM172	deCODE
FAM20A	deCODE
FAM3B	deCODE
FAM3B	deCODE
FAM3D	deCODE
FAP	deCODE
FAS	deCODE
FAS	deCODE
FASLG	deCODE
FBP1	deCODE
FCAMR	deCODE
FCAR	deCODE
FCER1A	deCODE
FCER2	deCODE
FCGR2A	deCODE
FCGR2B	deCODE
FCGR3B	deCODE
FCN1	deCODE
FCN2	deCODE

FCN2	deCODE
FCRL1	deCODE
FCRL3	deCODE
FCRL5	deCODE
FCRL6	deCODE
FCRLB	deCODE
FEN1	deCODE
FETUB	deCODE
FGF12	deCODE
FGF16	deCODE
FGF19	deCODE
FGF2	deCODE
FGF20	deCODE
FGF23	deCODE
FGF3	deCODE
FGF5	deCODE
FGF6	deCODE
FGF7	deCODE
FGF9	deCODE
FGFBP1	deCODE
FGFBP3	deCODE
FGFR2	deCODE
FGFR4	deCODE
FGL1	deCODE
FGR	deCODE
FH	deCODE
FHIT	deCODE
FIS1	deCODE
FKBP14	deCODE
FKBP1B	deCODE
FKBP4	deCODE
FKBP7	deCODE
FLRT2	deCODE
FLT1	deCODE
FLT1	deCODE
FLT3	deCODE
FLT3LG	deCODE
FLT4	deCODE

FMR1	deCODE
FN1	deCODE
FN1	deCODE
FN1	deCODE
FOLH1	deCODE
FOLR1	deCODE
FOLR2	deCODE
FOLR3	deCODE
FOXO3	deCODE
FRZB	deCODE
FSHB	deCODE
FST	deCODE
FSTL1	deCODE
FSTL3	deCODE
FTCD	deCODE
FURIN	deCODE
FUT3	deCODE
FUT8	deCODE
FXN	deCODE
FZD10	deCODE
GABAR	deCODE
GABAR	deCODE
GAD1	deCODE
GADD4	deCODE
GAGE2,	deCODE
GAL	deCODE
GALNT	deCODE
GALNT.	deCODE
GALNT.	deCODE
GAPDH	deCODE
GAS6	deCODE
GBP1	deCODE
GBP2	deCODE
GBP6	deCODE
GC	deCODE
GCG	deCODE
GCLM	deCODE
GCNT1	deCODE

GDF15	deCODE
GDF2	deCODE
GDNF	deCODE
GFAP	deCODE
GFER	deCODE
GFRA1	deCODE
GFRA2	deCODE
GFRA3	deCODE
GFRAL	deCODE
GGA1	deCODE
GGH	deCODE
GH1	deCODE
GH2	deCODE
GHR	deCODE
GHRL	deCODE
GHRL	deCODE
GIP	deCODE
GIP	deCODE
GJA8	deCODE
GKN1	deCODE
GLA	deCODE
GLB1	deCODE
GLO1	deCODE
GLP1R	deCODE
GLRX	deCODE
GLRX5	deCODE
GM2A	deCODE
GMFG	deCODE
GMPR	deCODE
GMPR2	deCODE
GNAS	deCODE
GNLY	deCODE
GNPDA	deCODE
GOT1	deCODE
GP1BA	deCODE
GP5	deCODE
GP6	deCODE
GPC1	deCODE

GPC5	deCODE
GPD1	deCODE
GPHA2	deCODE
GPI	deCODE
GPNMB	deCODE
GPNMB	deCODE
GPR101	deCODE
GRAP2	deCODE
GRAP2	deCODE
GRHPR	deCODE
GRN	deCODE
GRP	deCODE
GRP	deCODE
GRPEL1	deCODE
GSN	deCODE
GSN	deCODE
GSR	deCODE
GSTA1	deCODE
GSTA3	deCODE
GSTM4	deCODE
GSTP1	deCODE
GSTT2E	deCODE
GUK1	deCODE
GUSB	deCODE
GZMA	deCODE
GZMB	deCODE
GZMH	deCODE
HADH	deCODE
HAGH	deCODE
HAO1	deCODE
HAVCR	deCODE
HAVCR	deCODE
HAVCR	deCODE
HBEGF	deCODE
HBQ1	deCODE
HBZ	deCODE
HDAC8	deCODE
HDGF	deCODE

HDGF	deCODE
HEBP1	deCODE
HEPAC/	deCODE
HEXIM1	deCODE
HGF	deCODE
HGFAC	deCODE
HGS	deCODE
HIF1A	deCODE
HK2	deCODE
HMBS	deCODE
HMGCS	deCODE
HMOX1	deCODE
HMOX2	deCODE
HNF1A	deCODE
HNRNP	deCODE
HPCAL1	deCODE
HPGDS	deCODE
HPSE	deCODE
HRAS	deCODE
HRC	deCODE
HRG	deCODE
HS3ST3	deCODE
HS6ST1	deCODE
HS6ST2	deCODE
HSD17E	deCODE
HSP90B	deCODE
HSPA1A	deCODE
HSPB1	deCODE
HSPB6	deCODE
HSPG2	deCODE
HTRA2	deCODE
HYAL1	deCODE
ICAM1	deCODE
ICAM2	deCODE
ICAM3	deCODE
ICAM4	deCODE
ICAM5	deCODE
ICAM5	deCODE

ICOSLG	deCODE
IDI2	deCODE
IDO1	deCODE
IDS	deCODE
IDUA	deCODE
IFIT3	deCODE
IFNAR1	deCODE
IFNG	deCODE
IFNGR1	deCODE
IFNGR2	deCODE
IFNGR2	deCODE
IFNL1	deCODE
IFNL2	deCODE
IFNLR1	deCODE
IFNW1	deCODE
IGBP1	deCODE
IGDCC3	deCODE
IGDCC3	deCODE
IGDCC4	deCODE
IGF1R	deCODE
IGF2R	deCODE
IGFBP1	deCODE
IGFBP2	deCODE
IGFBP3	deCODE
IGFBP4	deCODE
IGFBP6	deCODE
IGFBP7	deCODE
IGFBPL	deCODE
IGLON5	deCODE
IGSF3	deCODE
IGSF8	deCODE
IL10	deCODE
IL10RA	deCODE
IL10RA	deCODE
IL10RB	deCODE
IL11	deCODE
IL12B	deCODE
IL12RB1	deCODE

IL12RB2	deCODE
IL13	deCODE
IL13RA1	deCODE
IL15	deCODE
IL15RA	deCODE
IL15RA	deCODE
IL16	deCODE
IL17A	deCODE
IL17C	deCODE
IL17D	deCODE
IL17F	deCODE
IL17RA	deCODE
IL17RB	deCODE
IL18	deCODE
IL18BP	deCODE
IL18R1	deCODE
IL18RAP	deCODE
IL19	deCODE
IL1A	deCODE
IL1B	deCODE
IL1R1	deCODE
IL1R2	deCODE
IL1RAP	deCODE
IL1RL1	deCODE
IL1RL2	deCODE
IL1RN	deCODE
IL2	deCODE
IL20	deCODE
IL20RA	deCODE
IL20RB	deCODE
IL21R	deCODE
IL22	deCODE
IL22RA1	deCODE
IL24	deCODE
IL25	deCODE
IL2RA	deCODE
IL2RB	deCODE
IL2RG	deCODE

IL3	deCODE
IL31	deCODE
IL31RA	deCODE
IL32	deCODE
IL34	deCODE
IL36A	deCODE
IL36G	deCODE
IL3RA	deCODE
IL4	deCODE
IL4R	deCODE
IL5	deCODE
IL5RA	deCODE
IL6	deCODE
IL6R	deCODE
IL6ST	deCODE
IL7	deCODE
IL7R	deCODE
IL9	deCODE
IMPA1	deCODE
ING1	deCODE
INHBB	deCODE
INHBB	deCODE
INHBC	deCODE
INHBC	deCODE
INSL3	deCODE
INSL4	deCODE
INSL5	deCODE
INSR	deCODE
IRAK4	deCODE
ISLR2	deCODE
ISM1	deCODE
IST1	deCODE
ITGA2	deCODE
ITGA5	deCODE
ITGAL	deCODE
ITGB2	deCODE
ITGB5	deCODE
ITGB6	deCODE

ITGB7	deCODE
ITIH1	deCODE
ITIH3	deCODE
ITIH4	deCODE
ITIH5	deCODE
ITM2A	deCODE
ITPA	deCODE
IVD	deCODE
JAM2	deCODE
JAM3	deCODE
JUN	deCODE
KAZAL	deCODE
KCNIP4	deCODE
KCTD5	deCODE
KDR	deCODE
KEL	deCODE
KIAA15	deCODE
KIAA20	deCODE
KIF1C	deCODE
KIF22	deCODE
KIR2DL	deCODE
KIR2DL	deCODE
KIR2DS	deCODE
KIR3DL	deCODE
KIR3DL	deCODE
KIRREL	deCODE
KIT	deCODE
KITLG	deCODE
KLB	deCODE
KLF4	deCODE
KLK10	deCODE
KLK11	deCODE
KLK12	deCODE
KLK13	deCODE
KLK14	deCODE
KLK15	deCODE
KLK3	deCODE
KLK4	deCODE

KLK6	deCODE
KLK7	deCODE
KLK8	deCODE
KLKB1	deCODE
KLRB1	deCODE
KLRC1	deCODE
KLRF1	deCODE
KLRK1	deCODE
KRT17	deCODE
KRT18	deCODE
KRT19	deCODE
KRT5	deCODE
KYNU	deCODE
L1CAM	deCODE
LACRT	deCODE
LAG3	deCODE
LAIR1	deCODE
LAIR2	deCODE
LAMA4	deCODE
LAMP1	deCODE
LAP3	deCODE
LAP3	deCODE
LAT	deCODE
LAT2	deCODE
LAYN	deCODE
LBP	deCODE
LCAT	deCODE
LCN2	deCODE
LCP1	deCODE
LDLR	deCODE
LDLRA	deCODE
LECT2	deCODE
LEFTY2	deCODE
LEFTY2	deCODE
LEG1	deCODE
LEP	deCODE
LEPR	deCODE
LGALS1	deCODE

LGALS3	deCODE
LGALS3	deCODE
LGALS4	deCODE
LGALS7	deCODE
LGALS8	deCODE
LGALS9	deCODE
LGMN	deCODE
LHB	deCODE
LIF	deCODE
LIFR	deCODE
LILRA2	deCODE
LILRA3	deCODE
LILRA4	deCODE
LILRA5	deCODE
LILRA5	deCODE
LILRA6	deCODE
LILRB1	deCODE
LILRB2	deCODE
LILRB4	deCODE
LILRB5	deCODE
LMNB1	deCODE
LMOD1	deCODE
LONP1	deCODE
LPCAT2	deCODE
LPO	deCODE
LRCH4	deCODE
LRFN2	deCODE
LRIG1	deCODE
LRIG3	deCODE
LRP1	deCODE
LRP1	deCODE
LRP1	deCODE
LRP11	deCODE
LRP11	deCODE
LRP2	deCODE
LRPAP1	deCODE
LRRC25	deCODE
LRRC37	deCODE

LRRC59	deCODE
LRRN1	deCODE
LRTM1	deCODE
LRTM2	deCODE
LSM1	deCODE
LTA	deCODE
LTA4H	deCODE
LTBR	deCODE
LY75	deCODE
LY9	deCODE
LYN	deCODE
LYN	deCODE
LYPD1	deCODE
LYPD3	deCODE
LYSMD	deCODE
LYVE1	deCODE
LYZL2	deCODE
MAD1L	deCODE
MAGEA	deCODE
MAMD(	deCODE
MAN1A	deCODE
MAN2B	deCODE
MANF	deCODE
MANSC	deCODE
MANSC	deCODE
MAP1L(	deCODE
MAP2K	deCODE
MAP2K	deCODE
MAPK1	deCODE
MAPK9	deCODE
MAPKA	deCODE
MAPRE	deCODE
MAPT	deCODE
MARCC	deCODE
MASP1	deCODE
MASP1	deCODE
MATN2	deCODE
MATN3	deCODE

MAX	deCODE
MB	deCODE
MBL2	deCODE
MCEE	deCODE
MCEMI	deCODE
MCTS1	deCODE
MDGA1	deCODE
MDH1	deCODE
MDK	deCODE
MDM1	deCODE
MEGF1(	deCODE
MENT	deCODE
MEPE	deCODE
MET	deCODE
METAP	deCODE
METAP.	deCODE
MFAP3I	deCODE
MFAP4	deCODE
MFAP5	deCODE
MFGE8	deCODE
MIA	deCODE
MICALI	deCODE
MICB	deCODE
MIF	deCODE
MILR1	deCODE
MLN	deCODE
MMP1	deCODE
MMP10	deCODE
MMP12	deCODE
MMP13	deCODE
MMP3	deCODE
MMP7	deCODE
MMP8	deCODE
MMP9	deCODE
MOG	deCODE
MORF4.	deCODE
MPO	deCODE
MRC1	deCODE

MRPL52	deCODE
MSLN	deCODE
MSMB	deCODE
MSR1	deCODE
MSR1	deCODE
MSRA	deCODE
MST1	deCODE
MSTN	deCODE
MTHFD	deCODE
MTHFS1	deCODE
MTIF3	deCODE
MUC16	deCODE
MVK	deCODE
MXRA8	deCODE
MYBPC	deCODE
MYDGF	deCODE
MYL3	deCODE
MYL6B	deCODE
MYO6	deCODE
MYOC	deCODE
MYOM2	deCODE
MYOM3	deCODE
NAA10	deCODE
NAAA	deCODE
NADK	deCODE
NAGK	deCODE
NAGPA	deCODE
NAMPT	deCODE
NAP1L4	deCODE
NBL1	deCODE
NCAM1	deCODE
NCAM2	deCODE
NCAN	deCODE
NCF2	deCODE
NCK2	deCODE
NCR1	deCODE
NCR3L(	deCODE
NDRG1	deCODE

NDST1	deCODE
NDUFA	deCODE
NEDD9	deCODE
NEFL	deCODE
NEK7	deCODE
NELL1	deCODE
NELL2	deCODE
NENF	deCODE
NEO1	deCODE
NFASC	deCODE
NFATC1	deCODE
NFKB1	deCODE
NFU1	deCODE
NGF	deCODE
NGFR	deCODE
NGRN	deCODE
NHLRC	deCODE
NID1	deCODE
NID2	deCODE
NIT2	deCODE
NLGN1	deCODE
NLGN2	deCODE
NLGN2	deCODE
NME1	deCODE
NMI	deCODE
NMNAT	deCODE
NMT1	deCODE
NOTCH	deCODE
NOTCH	deCODE
NOTCH	deCODE
NOTCH	deCODE
NPDC1	deCODE
NPL	deCODE
NPM1	deCODE
NPPB	deCODE
NPPB	deCODE
NPPB	deCODE
NPTN	deCODE

NPTX1	deCODE
NPTX2	deCODE
NPTXR	deCODE
NRCAM	deCODE
NRGN	deCODE
NRP1	deCODE
NRP2	deCODE
NRXN3	deCODE
NRXN3	deCODE
NSFL1C	deCODE
NT5C	deCODE
NT5C3A	deCODE
NT5E	deCODE
NTF3	deCODE
NTF4	deCODE
NTRK2	deCODE
NTRK3	deCODE
NUDC	deCODE
NUDT2	deCODE
NUDT5	deCODE
NXPH1	deCODE
NXPH3	deCODE
OBP2B	deCODE
OGN	deCODE
OLFM4	deCODE
OLR1	deCODE
OMD	deCODE
OMG	deCODE
OMP	deCODE
OPHN1	deCODE
OPTC	deCODE
OSCAR	deCODE
OSM	deCODE
OSM	deCODE
OSMR	deCODE
OSTN	deCODE
OXT	deCODE
P4HB	deCODE

PAEP	deCODE
PAFAH1	deCODE
PAFAH2	deCODE
PAK4	deCODE
PALM	deCODE
PAM	deCODE
PAPPA	deCODE
PARK7	deCODE
PARK7	deCODE
PARP1	deCODE
PARP1	deCODE
PBK	deCODE
PCBD1	deCODE
PCDH9	deCODE
PCNA	deCODE
PCOLCE	deCODE
PCSK7	deCODE
PCSK9	deCODE
PDAP1	deCODE
PDCD1	deCODE
PDCD1L	deCODE
PDCD5	deCODE
PDCD6	deCODE
PDCL2	deCODE
PDE4D	deCODE
PDE5A	deCODE
PDE5A	deCODE
PDGFA	deCODE
PDGFB	deCODE
PDGFC	deCODE
PDGFRA	deCODE
PDGFRB	deCODE
PDIA3	deCODE
PDIA4	deCODE
PDIA5	deCODE
PDRG1	deCODE
PDZK1	deCODE
PEAR1	deCODE

PEAR1	deCODE
PEBP1	deCODE
PECAM	deCODE
PECR	deCODE
PENK	deCODE
PF4	deCODE
PFDN2	deCODE
PGD	deCODE
PGF	deCODE
PGLYRI	deCODE
PGLYRI	deCODE
PGM2	deCODE
PGR	deCODE
PI3	deCODE
PIGR	deCODE
PILRA	deCODE
PILRA	deCODE
PILRA	deCODE
PKD2	deCODE
PKD2	deCODE
PKLR	deCODE
PLA2G1	deCODE
PLA2G1	deCODE
PLA2G2	deCODE
PLA2G7	deCODE
PLAT	deCODE
PLAU	deCODE
PLAUR	deCODE
PLCB1	deCODE
PLG	deCODE
PLG	deCODE
PLG	deCODE
PLIN3	deCODE
PLSCR3	deCODE
PLTP	deCODE
PLXDC1	deCODE
PLXDC2	deCODE
PLXNA4	deCODE

PLXNB2	deCODE
PLXNB3	deCODE
PMM2	deCODE
PMVK	deCODE
PNLIP	deCODE
PNLIPR	deCODE
PNLIPR	deCODE
PODXL2	deCODE
POMC	deCODE
POMC	deCODE
POMC	deCODE
PON1	deCODE
POSTN	deCODE
PPBP	deCODE
PPBP	deCODE
PPBP	deCODE
PPBP	deCODE
PPCDC	deCODE
PPIB	deCODE
PPIE	deCODE
PPIF	deCODE
PPM1A	deCODE
PPME1	deCODE
PPP1CC	deCODE
PPP1R14	deCODE
PPP1R2	deCODE
PPP2R5	deCODE
PPP3R1	deCODE
PPT1	deCODE
PPY	deCODE
PQBP1	deCODE
PRC1	deCODE
PRC1	deCODE
PRCP	deCODE
PRDX1	deCODE
PRDX3	deCODE
PRDX5	deCODE
PRDX6	deCODE

PRG2	deCODE
PRG3	deCODE
PRKAR1	deCODE
PRKCQ	deCODE
PRKG1	deCODE
PRL	deCODE
PRND	deCODE
PROC	deCODE
PROC	deCODE
PROK1	deCODE
PROS1	deCODE
PRSS2	deCODE
PRSS22	deCODE
PRSS27	deCODE
PRSS8	deCODE
PRTN3	deCODE
PSAP	deCODE
PSAPL1	deCODE
PSG1	deCODE
PSIP1	deCODE
PSMA1	deCODE
PSMA1	deCODE
PSMD5	deCODE
PSMD9	deCODE
PSME1	deCODE
PSME2	deCODE
PSMG3	deCODE
PSPN	deCODE
PTEN	deCODE
PTGDS	deCODE
PTGES2	deCODE
PTGR1	deCODE
PTH	deCODE
PTH	deCODE
PTH1R	deCODE
PTK7	deCODE
PTN	deCODE
PTP4A3	deCODE

PTPN1	deCODE
PTPN6	deCODE
PTPN9	deCODE
PTPRH	deCODE
PTPRR	deCODE
PTPRS	deCODE
PTS	deCODE
PTTG1	deCODE
PTX3	deCODE
PVR	deCODE
PXDNL	deCODE
PYDC1	deCODE
PYY	deCODE
PZP	deCODE
QDPR	deCODE
QPCT	deCODE
RAB27I	deCODE
RAB2B	deCODE
RAB39I	deCODE
RAB6B	deCODE
RABEPI	deCODE
RAC3	deCODE
RAD23I	deCODE
RAD51	deCODE
RALB	deCODE
RANBP	deCODE
RARRE:	deCODE
RARRE:	deCODE
RASA1	deCODE
RBKS	deCODE
RBM17	deCODE
RBM19	deCODE
RBP1	deCODE
RBP5	deCODE
RBP7	deCODE
REG1A	deCODE
REG1B	deCODE
REG3A	deCODE

REG3G	deCODE
REG4	deCODE
RELB	deCODE
RELT	deCODE
REN	deCODE
RET	deCODE
RETN	deCODE
REXO2	deCODE
RGMA	deCODE
RGMB	deCODE
RGS10	deCODE
RGS8	deCODE
RHOC	deCODE
RLN2	deCODE
RNASE1	deCODE
RNASE1	deCODE
RNASE3	deCODE
RNASE4	deCODE
RNASE6	deCODE
RNASE7	deCODE
RNF149	deCODE
RNF41	deCODE
RNF43	deCODE
ROBO1	deCODE
ROBO2	deCODE
ROBO4	deCODE
ROR1	deCODE
RPA2	deCODE
RPE	deCODE
RPS10	deCODE
RRAS	deCODE
RRM2	deCODE
RRM2B	deCODE
RSPO1	deCODE
RSPO1	deCODE
RSPO3	deCODE
RSPO3	deCODE
RTN4IP	deCODE

RTN4R	deCODE
RUVBL	deCODE
S100A1	deCODE
S100A1	deCODE
S100A1	deCODE
S100A1	deCODE
S100A1	deCODE
S100A3	deCODE
S100A4	deCODE
S100P	deCODE
SAA4	deCODE
SAP18	deCODE
SAT1	deCODE
SAT2	deCODE
SATB1	deCODE
SBSN	deCODE
SCARA	deCODE
SCARB	deCODE
SCARF1	deCODE
SCARF	deCODE
SCARF	deCODE
SCG3	deCODE
SCGB1	deCODE
SCGB3	deCODE
SCGN	deCODE
SCIN	deCODE
SCN2B	deCODE
SCN4B	deCODE
SCT	deCODE
SELE	deCODE
SELL	deCODE
SELP	deCODE
SELPLC	deCODE
SEMA3	deCODE
SEMA4	deCODE
SEMA4	deCODE
SEMA6	deCODE
SEMA6	deCODE

SEMA7A	deCODE
SERPIN	deCODE
SERPIN	deCODE
SERPIN	deCODE
SERPIN	deCODE
SERPIN	deCODE
SERPIN	deCODE
SERPIN	deCODE
SERPIN	deCODE
SERPIN	deCODE
SERPIN	deCODE
SERPIN	deCODE
SERPIN	deCODE
SERPIN	deCODE
SERPIN	deCODE
SERPIN	deCODE
SERPIN	deCODE
SERPIN	deCODE
SERPIN	deCODE
SERPIN	deCODE
SERPIN	deCODE
SERPIN	deCODE
SERPIN	deCODE
SETMA	deCODE
SEZ6L	deCODE
SEZ6L2	deCODE
SF3B4	deCODE
SFRP1	deCODE
SFRP4	deCODE
SFTPD	deCODE
SH2D1A	deCODE
SH3BGI	deCODE
SH3GL3	deCODE
SH3GLI	deCODE
SHBG	deCODE
SHC1	deCODE
SHC1	deCODE
SHH	deCODE

SHMT1	deCODE
SIAE	deCODE
SIGLEC	deCODE
SIGLEC	deCODE
SIGLEC	deCODE
SIGLEC	deCODE
SIGLEC	deCODE
SIGLEC	deCODE
SIGLEC	deCODE
SIRPA	deCODE
SIRPB1	deCODE
SIRT2	deCODE
SIRT5	deCODE
SIT1	deCODE
SLAMF	deCODE
SLAMF	deCODE
SLAMF	deCODE
SLAMF	deCODE
SLIT2	deCODE
SLITRK	deCODE
SLITRK	deCODE
SLMAP	deCODE
SMAD1	deCODE
SMAD2	deCODE
SMAD3	deCODE
SMC3	deCODE
SMNDC	deCODE
SMOC1	deCODE
SMOC2	deCODE
SMPD1	deCODE
SMPDL	deCODE
SMS	deCODE
SMTN	deCODE
SNAP25	deCODE
SNAP29	deCODE
SNAPIN	deCODE
SNCA	deCODE
SNRPB	deCODE

SNX5	deCODE
SOD1	deCODE
SOD2	deCODE
SOD3	deCODE
SORCS2	deCODE
SORD	deCODE
SORT1	deCODE
SOST	deCODE
SOX2	deCODE
SPACA5	deCODE
SPARC	deCODE
SPARCI	deCODE
SPINK1	deCODE
SPINK2	deCODE
SPINK4	deCODE
SPINK5	deCODE
SPINK6	deCODE
SPINK8	deCODE
SPINT1	deCODE
SPINT2	deCODE
SPINT3	deCODE
SPOCK1	deCODE
SPON1	deCODE
SPON2	deCODE
SPP1	deCODE
SPTLC1	deCODE
SRC	deCODE
SRC	deCODE
SRP14	deCODE
SSB	deCODE
SSB	deCODE
ST13	deCODE
ST3GAI	deCODE
ST6GAI	deCODE
STAB2	deCODE
STAMB	deCODE
STAT5E	deCODE
STAU1	deCODE

STC1	deCODE
STC2	deCODE
STIP1	deCODE
STK24	deCODE
STOML	deCODE
STX1B	deCODE
STX3	deCODE
STX4	deCODE
STX6	deCODE
STX7	deCODE
STX8	deCODE
SUGT1	deCODE
SULT2A	deCODE
SUMF1	deCODE
SUMF2	deCODE
SUOX	deCODE
SUSD1	deCODE
SV2A	deCODE
SWAP7(	deCODE
SYTL4	deCODE
SYTL4	deCODE
TACSTI	deCODE
TACSTI	deCODE
TAGLN	deCODE
TALDO	deCODE
TANK	deCODE
TARBP2	deCODE
TAX1BI	deCODE
TBC1D5	deCODE
TBCA	deCODE
TBCB	deCODE
TCL1A	deCODE
TCL1B	deCODE
TCN1	deCODE
TCN2	deCODE
TCN2	deCODE
TCP11	deCODE
TDGF1	deCODE

TDO2	deCODE
TDP1	deCODE
TEK	deCODE
TERF1	deCODE
TF	deCODE
TFF1	deCODE
TFF2	deCODE
TFF3	deCODE
TFF3	deCODE
TFPI	deCODE
TFPI2	deCODE
TFRC	deCODE
TFRC	deCODE
TG	deCODE
TGFB1	deCODE
TGFB2	deCODE
TGFBI	deCODE
TGFBR2	deCODE
TGFBR3	deCODE
TGM2	deCODE
THBS2	deCODE
THBS2	deCODE
THBS4	deCODE
THPO	deCODE
THPO	deCODE
THSD1	deCODE
THTPA	deCODE
TIE1	deCODE
TIGAR	deCODE
TIGIT	deCODE
TIGIT	deCODE
TIMD4	deCODE
TIMM8A	deCODE
TIMP1	deCODE
TIMP2	deCODE
TIMP3	deCODE
TIMP4	deCODE
TINAGI	deCODE

TK1	deCODE
TLR1	deCODE
TLR1	deCODE
TLR2	deCODE
TLR3	deCODE
TLR4	deCODE
TMCO5.	deCODE
TMED1(	deCODE
TMED4	deCODE
TMEM1	deCODE
TMEM1	deCODE
TMEM2	deCODE
TMPRS!	deCODE
TMPRS!	deCODE
TMPRS!	deCODE
TMPRS!	deCODE
TNC	deCODE
TNF	deCODE
TNFAIP	deCODE
TNFRSF	deCODE
TNFRSF	deCODE
TNFRSF	deCODE
TNFRSF	deCODE
TNFRSF	deCODE
TNFRSF	deCODE
TNFRSF	deCODE
TNFRSF	deCODE
TNFRSF	deCODE
TNFRSF	deCODE
TNFRSF	deCODE
TNFRSF	deCODE
TNFRSF	deCODE
TNFRSF	deCODE
TNFRSF	deCODE
TNFRSF	deCODE
TNFRSF	deCODE
TNFRSF	deCODE
TNFSF1	deCODE

TNFSF1	deCODE
TNFSF1	deCODE
TNFSF1	deCODE
TNFSF1	deCODE
TNFSF1	deCODE
TNFSF8	deCODE
TNNI3	deCODE
TNR	deCODE
TNXB	deCODE
TOP1	deCODE
TOR1AI	deCODE
TOR1AI	deCODE
TP53	deCODE
TP53	deCODE
TP53I3	deCODE
TPK1	deCODE
TPMT	deCODE
TPP1	deCODE
TPPP2	deCODE
TPRKB	deCODE
TPSAB1	deCODE
TPSG1	deCODE
TPT1	deCODE
TRDMT	deCODE
TREM2	deCODE
TREML	deCODE
TREML	deCODE
TREML	deCODE
TRIAP1	deCODE
TSLP	deCODE
TST	deCODE
TTN	deCODE
TXLNA	deCODE
TXN	deCODE
TXNDC	deCODE
TXNDC	deCODE
TXNRD	deCODE
TYMP	deCODE

TYRO3	deCODE
UBE2B	deCODE
UBE2L6	deCODE
UGDH	deCODE
ULBP2	deCODE
UMOD	deCODE
UNC5D	deCODE
UROD	deCODE
UROS	deCODE
USO1	deCODE
USP25	deCODE
USP28	deCODE
USP8	deCODE
UXS1	deCODE
VAMP8	deCODE
VASN	deCODE
VASP	deCODE
VAT1	deCODE
VAV3	deCODE
VCAM1	deCODE
VCAN	deCODE
VEGFA	deCODE
VEGFA	deCODE
VEGFA	deCODE
VEGFB	deCODE
VEGFC	deCODE
VIM	deCODE
VIT	deCODE
VNN2	deCODE
VPS28	deCODE
VPS4B	deCODE
VSIG10	deCODE
VSIG2	deCODE
VSNL1	deCODE
VSTM2	deCODE
VTA1	deCODE
VTI1A	deCODE
VWA1	deCODE

VWC2	deCODE
VWC2L	deCODE
VWF	deCODE
WARS	deCODE
WFDC1	deCODE
WFDC2	deCODE
WFIKKI	deCODE
WFIKKI	deCODE
WIF1	deCODE
XCL1	deCODE
XG	deCODE
XRCC4	deCODE
YES1	deCODE
YWHA(	deCODE
YY1	deCODE
ZNF174	deCODE
ZNF75D	deCODE
ZP4	deCODE

Supplementary Table S2： SNPs used as proxies for lifetime smoking index

SNP	CHR	BP	EFFECT	OTHER_	EAF	INFO	BETA
rs804284	15	78817929	C	T	0.34229	0.99915	0.0192164
rs113382	9	1.36E+08	C	A	0.88933	0.99384	-0.0282339
rs601177	20	61984317	C	T	0.19124	0.99802	0.0191152
rs991967	11	1.13E+08	G	A	0.61219	0.99923	-0.0152438
rs289077	2	1.46E+08	G	T	0.41318	0.9947	-0.0136979
rs122443	10	1.05E+08	G	A	0.66112	0.99848	-0.0132503
rs117830	8	27425349	C	T	0.83881	0.98513	0.01571
rs112102	1	73860028	A	G	0.38367	1	0.011702
rs621558	2	1.06E+08	A	G	0.87336	0.99528	-0.0169073
rs102262	7	32315613	A	G	0.62964	1	-0.0114099
rs611989	20	31145415	G	A	0.76172	1	-0.0128038
rs986391	5	1.67E+08	G	A	0.36656	0.99701	0.0111388
rs374236	14	1.04E+08	T	C	0.59514	0.99484	-0.0107929
rs240192	7	1.15E+08	G	C	0.50196	0.99431	0.0105718
rs780701	7	1.18E+08	A	G	0.54033	0.9976	-0.0104211
rs549845	1	44076469	G	A	0.30116	0.99818	0.0112613
rs109229	1	91193049	A	T	0.45105	0.99372	0.0101748
rs173098	11	27667236	G	A	0.74036	0.99545	-0.0112899
rs677808	3	49317338	T	C	0.26737	0.99883	0.0111141
rs804213	15	97514404	T	G	0.54108	0.98347	-0.00994136
rs175765	4	1.48E+08	G	A	0.7235	0.99303	0.0109502
rs776661	6	1.12E+08	C	A	0.18263	0.99966	0.0125839
rs192201	7	3560401	C	T	0.36442	1	0.0100328
rs755334	1	75005067	G	A	0.43766	0.99824	0.00963399
rs752860	1	66407352	G	A	0.56561	0.99508	0.00965269
rs329120	5	1.34E+08	C	T	0.58067	0.99697	0.00965704
rs126237	2	2.03E+08	A	G	0.61338	0.98686	-0.00977238
rs132965	9	1.28E+08	G	T	0.60639	0.99625	-0.00970128
rs693595	6	26255451	A	G	0.42111	1	0.0095817
rs467135	2	60136176	T	C	0.51882	0.99307	-0.00944175
rs389622	10	1.06E+08	A	G	0.58529	0.98484	0.00962696
rs326341	3	1.08E+08	G	A	0.52494	0.99185	0.00943522
rs439180	11	28674592	A	G	0.70746	0.99112	0.0103191
rs726788	4	1.12E+08	G	A	0.82859	0.99599	0.0123829
rs112282	11	46632809	G	A	0.95895	0.9904	-0.0231662
rs108798	12	75380511	T	G	0.34344	1	-0.00957988

rs889398	16	69556715	C	T	0.58801	0.99199	0.0092407
rs447334	2	1.82E+08	A	T	0.24998	0.9995	-0.0104317
rs122114	9	1.22E+08	C	G	0.58706	0.99745	0.00916504
rs317021	4	35418368	T	A	0.81429	0.98771	-0.011567
rs193327	1	49977965	T	G	0.36373	0.99701	0.00921831
rs112559	10	8802912	T	G	0.74349	0.99537	-0.0100713
rs131533	5	1.68E+08	A	G	0.88394	0.98716	-0.0137451
rs267867	2	1.04E+08	A	T	0.48553	0.9938	0.00873079
rs733355	13	1.01E+08	G	A	0.21171	0.98455	0.01074
rs766085	19	4474725	C	A	0.95301	0.90781	0.0216317
rs454359	9	3014254	T	C	0.51991	0.99221	-0.00866243
rs119487	5	13246336	T	C	0.76825	0.9931	-0.0102364
rs703981	9	82430418	G	A	0.42731	0.99383	0.00873433
rs102822	7	1.11E+08	C	T	0.36184	0.99084	0.00896219
rs283883	21	46665208	C	T	0.69946	0.99908	-0.00936489
rs624833	4	2881256	T	G	0.6947	0.99915	0.00928567
rs621355	2	44326028	C	T	0.96826	0.99121	0.0243444
rs381103	2	1.13E+08	T	C	0.72385	0.98501	-0.00954466
rs359243	2	60475509	T	C	0.39289	0.98895	-0.00871975
rs117684	7	96629103	C	A	0.66617	0.99171	0.00901238
rs677930	3	16859710	G	T	0.63292	0.99382	-0.00875253
rs351696	8	9604066	T	G	0.6122	0.97113	0.00877627
rs675960	17	50333733	G	A	0.64881	0.99184	-0.00887666
rs267563	10	63576286	G	A	0.58057	1	0.00849857
rs757424	11	17070365	G	A	0.73885	0.98801	0.00961948
rs713675	18	77576337	G	A	0.79053	0.99655	-0.0103197
rs716275	5	43161351	G	A	0.88881	1	0.0132566
rs369230	16	89645437	G	T	0.30757	0.98467	-0.00908882
rs100525	5	50812738	T	C	0.57336	0.99659	0.00839979
rs376994	2	1.66E+08	T	A	0.52785	0.99944	-0.00825646
rs715559	14	77502546	A	C	0.67428	0.99089	-0.00885162
rs707767	10	1.04E+08	C	T	0.62343	0.98799	0.00855025
rs860326	14	57342912	C	T	0.4277	0.99542	0.00833808
rs122025	6	67475273	A	G	0.51275	0.99859	-0.00823391
rs481487	20	19616429	C	T	0.76659	0.99904	0.00971214
rs984294	3	1.57E+08	C	T	0.32617	0.99049	-0.00877189
rs289480	6	52861990	T	A	0.92212	0.99494	-0.0152983
rs127086	16	24728227	A	G	0.28463	0.9963	-0.0090871

rs202645	22	41798520	A	G	0.20294	0.99979	-0.0101598
rs620980	18	50863861	G	A	0.63991	0.98162	-0.00856663
rs495752	5	1.06E+08	A	C	0.2085	0.97849	-0.0101225
rs124626	9	86761745	T	C	0.30456	0.99355	-0.00886526
rs659853	15	99204483	T	C	0.48867	0.99737	-0.00815056
rs130090	2	1.74E+08	A	G	0.32771	0.99912	0.00863272
rs175532	10	92912773	A	C	0.88461	0.98699	-0.0127323
rs729717	12	56473808	T	C	0.43136	0.99844	-0.00811649
rs245774	5	1.71E+08	A	G	0.27169	0.99554	-0.00901601
rs696277	7	99081730	A	G	0.84555	1	0.011064
rs124812	20	44761377	G	C	0.72234	1	-0.00894356
rs353433	19	18471610	C	A	0.73271	0.961	0.00918144
rs656247	13	67332812	C	G	0.65061	0.98857	0.00836914
rs775758	3	77582005	A	T	0.43286	0.99331	0.00801379
rs206288	8	91839576	G	A	0.58678	0.98179	-0.0081103
rs751962	1	99514554	C	T	0.32368	0.9985	0.00842015
rs943534	1	1.08E+08	T	A	0.34423	0.99456	0.00834773
rs348660	11	16377356	A	G	0.68619	0.98432	-0.00857198
rs348809	20	59032097	A	G	0.34764	0.99671	-0.00828381
rs105084	16	87443734	C	T	0.42585	0.99104	0.00797046
rs732205	3	1.31E+08	A	C	0.8423	0.98747	-0.0108189
rs457150	5	87756918	C	T	0.53952	0.99581	0.00787701
rs732083	17	37834367	G	A	0.33336	0.99512	0.00834823
rs494946	1	32178489	T	C	0.86955	1	-0.0116055
rs621759	2	1.61E+08	T	C	0.96594	0.97781	0.0217461
rs136233	22	31212410	A	G	0.80908	0.99502	-0.00995614
rs128316	12	84758368	C	T	0.76431	0.99792	-0.00918459
rs118612	16	746611	G	T	0.78364	0.9993	0.00945079
rs109187	1	1.62E+08	G	A	0.37225	0.99671	0.00802389
rs108239	10	74738269	A	T	0.63297	0.97851	0.00813544
rs740869	12	50015942	G	A	0.92546	0.99682	0.0148021
rs473192	7	1.33E+08	C	T	0.31561	0.99836	-0.00829239
rs284853	15	74044197	C	T	0.63148	0.99583	0.00800666
rs119323	1	7526486	G	C	0.43918	0.99267	-0.00776138
rs609524	16	75640521	T	C	0.90915	0.99346	0.0134109
rs990428	17	47031973	T	C	0.70777	0.99884	0.00842758
rs129678	18	35138245	A	G	0.33123	0.99002	0.0081887
rs225471	6	37477000	C	A	0.23636	0.99646	0.00899636

rs72674{	8	95578201	A	T	0.76457	0.99507	0.00898766
rs19312(	1	96175101	G	T	0.51032	0.99639	-0.00760874
rs57611!	16	31165795	G	A	0.48458	0.96703	0.00774344
rs61796(	4	23678196	A	T	0.91237	0.99979	-0.0134163
rs69578!	7	1.32E+08	C	T	0.50326	0.9967	-0.00758434
rs20808'	5	60388313	A	T	0.25811	0.99978	0.00863309

SE	P	phenotyp	samplesize
0.0014618	1.8e-39	Lifetime	462690
0.0022138	3e-37	Lifetime	462690
0.0017636	2.3e-27	Lifetime	462690
0.0014211	7.6e-27	Lifetime	462690
0.0014067	2.1e-22	Lifetime	462690
0.0014641	1.4e-19	Lifetime	462690
0.0018966	1.2e-16	Lifetime	462690
0.0014227	2e-16	Lifetime	462690
0.0020855	5.2e-16	Lifetime	462690
0.001437	2e-15	Lifetime	462690
0.001627	3.6e-15	Lifetime	462690
0.0014379	9.4e-15	Lifetime	462690
0.0014159	2.5e-14	Lifetime	462690
0.0013891	2.7e-14	Lifetime	462690
0.0013906	6.7e-14	Lifetime	462690
0.0015086	8.3e-14	Lifetime	462690
0.001395	3e-13	Lifetime	462690
0.0015823	9.7e-13	Lifetime	462690
0.0015662	1.3e-12	Lifetime	462690
0.0014011	1.3e-12	Lifetime	462690
0.0015516	1.7e-12	Lifetime	462690
0.0017928	2.2e-12	Lifetime	462690
0.0014377	3e-12	Lifetime	462690
0.0013964	5.2e-12	Lifetime	462690
0.0014014	5.7e-12	Lifetime	462690
0.0014051	6.3e-12	Lifetime	462690
0.0014279	7.7e-12	Lifetime	462690
0.0014189	8.1e-12	Lifetime	462690
0.0014019	8.2e-12	Lifetime	462690
0.0013899	1.1e-11	Lifetime	462690
0.0014179	1.1e-11	Lifetime	462690
0.0013925	1.2e-11	Lifetime	462690
0.0015279	1.4e-11	Lifetime	462690
0.0018387	1.6e-11	Lifetime	462690
0.0035042	3.8e-11	Lifetime	462690
0.001458	5e-11	Lifetime	462690

0.0014137	6.3e-11	Lifetime	462690
0.0015968	6.4e-11	Lifetime	462690
0.0014071	7.3e-11	Lifetime	462690
0.0017909	1.1e-10	Lifetime	462690
0.0014381	1.5e-10	Lifetime	462690
0.0015881	2.3e-10	Lifetime	462690
0.0021733	2.5e-10	Lifetime	462690
0.0013876	3.1e-10	Lifetime	462690
0.0017072	3.2e-10	Lifetime	462690
0.00344	3.2e-10	Lifetime	462690
0.0013891	4.5e-10	Lifetime	462690
0.0016452	4.9e-10	Lifetime	462690
0.001405	5.1e-10	Lifetime	462690
0.0014469	5.9e-10	Lifetime	462690
0.0015149	6.3e-10	Lifetime	462690
0.0015036	6.6e-10	Lifetime	462690
0.0039617	8e-10	Lifetime	462690
0.0015575	8.9e-10	Lifetime	462690
0.0014254	9.5e-10	Lifetime	462690
0.0014746	9.9e-10	Lifetime	462690
0.0014392	1.2e-09	Lifetime	462690
0.0014439	1.2e-09	Lifetime	462690
0.0014584	1.2e-09	Lifetime	462690
0.0014002	1.3e-09	Lifetime	462690
0.0015849	1.3e-09	Lifetime	462690
0.0017038	1.4e-09	Lifetime	462690
0.0021987	1.6e-09	Lifetime	462690
0.001511	1.8e-09	Lifetime	462690
0.0014022	2.1e-09	Lifetime	462690
0.0013856	2.5e-09	Lifetime	462690
0.0014851	2.5e-09	Lifetime	462690
0.0014358	2.6e-09	Lifetime	462690
0.0014023	2.7e-09	Lifetime	462690
0.001386	2.8e-09	Lifetime	462690
0.0016364	2.9e-09	Lifetime	462690
0.0014807	3.1e-09	Lifetime	462690
0.0025912	3.5e-09	Lifetime	462690
0.0015389	3.5e-09	Lifetime	462690

0.001725	3.9e-09	Lifetime	462690
0.0014568	4.1e-09	Lifetime	462690
0.0017221	4.2e-09	Lifetime	462690
0.0015091	4.2e-09	Lifetime	462690
0.0013894	4.5e-09	Lifetime	462690
0.0014733	4.6e-09	Lifetime	462690
0.0021815	5.3e-09	Lifetime	462690
0.0013992	6.6e-09	Lifetime	462690
0.0015597	7.4e-09	Lifetime	462690
0.0019165	7.8e-09	Lifetime	462690
0.0015494	7.8e-09	Lifetime	462690
0.0015959	8.8e-09	Lifetime	462690
0.0014611	1e-08	Lifetime	462690
0.0014027	1.1e-08	Lifetime	462690
0.00142	1.1e-08	Lifetime	462690
0.0014789	1.2e-08	Lifetime	462690
0.0014642	1.2e-08	Lifetime	462690
0.0015056	1.2e-08	Lifetime	462690
0.001456	1.3e-08	Lifetime	462690
0.001405	1.4e-08	Lifetime	462690
0.0019126	1.5e-08	Lifetime	462690
0.0013917	1.5e-08	Lifetime	462690
0.0014731	1.5e-08	Lifetime	462690
0.0020579	1.7e-08	Lifetime	462690
0.0038568	1.7e-08	Lifetime	462690
0.0017692	1.8e-08	Lifetime	462690
0.0016331	1.9e-08	Lifetime	462690
0.0016846	2e-08	Lifetime	462690
0.0014319	2.1e-08	Lifetime	462690
0.0014526	2.1e-08	Lifetime	462690
0.002642	2.1e-08	Lifetime	462690
0.0014902	2.6e-08	Lifetime	462690
0.0014389	2.6e-08	Lifetime	462690
0.0013972	2.8e-08	Lifetime	462690
0.0024193	3e-08	Lifetime	462690
0.0015229	3.1e-08	Lifetime	462690
0.0014793	3.1e-08	Lifetime	462690
0.0016309	3.5e-08	Lifetime	462690

0.001634	3.8e-08	Lifetime	462690
0.0013858	4e-08	Lifetime	462690
0.0014103	4e-08	Lifetime	462690
0.0024475	4.2e-08	Lifetime	462690
0.001387	4.5e-08	Lifetime	462690
0.0015826	4.9e-08	Lifetime	462690

Supplementary Table S3： SNPs used to proxy initiation of smoking

SNP	pval	id	chr.expo	pos.exposu	effect_al	other_al	eaf.exposu
rs10062(	2.28e-10	xIXa8m	chr5	79994811	A	C	0.635189
rs10119:	1.8e-08	xIXa8m	chr9	29740030	T	C	0.497018
rs10121!	8.01e-10	xIXa8m	chr9	11175974	A	T	0.508946
rs10233(	1.49e-18	xIXa8m	chr7	1.18E+08	G	A	0.512922
rs10259:	4.83e-13	xIXa8m	chr18	52344970	C	G	0.294235
rs10279:	6.87e-14	xIXa8m	chr7	1.34E+08	A	G	0.629225
rs10458!	2.17e-18	xIXa8m	chr1	50146578	G	A	0.190855
rs10508	3.71e-11	xIXa8m	chr16	87410128	T	C	0.550696
rs10745:	1.97e-08	xIXa8m	chr1	1.12E+08	G	A	0.672962
rs10753(	9.96e-11	xIXa8m	chr1	1.64E+08	T	C	0.348907
rs10786	7.91e-27	xIXa8m	chr10	1.03E+08	A	C	0.416501
rs10927(	2.39e-10	xIXa8m	chr1	2.44E+08	C	T	0.196819
rs10950	1.54e-12	xIXa8m	chr7	1869450	G	C	0.609344
rs10954:	4.78e-09	xIXa8m	chr7	1.26E+08	C	A	0.346918
rs11078	2.77e-15	xIXa8m	chr17	7892654	G	A	0.408549
rs11115	8.22e-09	xIXa8m	chr12	1.17E+08	T	G	0.15507
rs11162(	3.4e-09	xIXa8m	chr1	87447493	T	C	0.379722
rs11162!	6.47e-10	xIXa8m	chr1	80356597	C	A	0.306163
rs11258	3.83e-10	xIXa8m	chr10	13491053	T	C	0.335984
rs11267!	4.15e-11	xIXa8m	chr19	55368504	T	C	0.472167
rs11390	5.22e-09	xIXa8m	chr12	1.12E+08	AG	A	0.352883
rs11405(	2.56e-11	xIXa8m	chr3	1.51E+08	A	T	0.06163
rs11439:	3.34e-08	xIXa8m	chr15	53833463	TA	T	0.185885
rs11490(	1.45e-12	xIXa8m	chr16	72595157	G	C	0.058648
rs11556	7.72e-10	xIXa8m	chr15	96959765	A	G	0.333996
rs11626!	2.73e-12	xIXa8m	chr14	1.04E+08	T	C	0.156064
rs11632	1.31e-08	xIXa8m	chr15	80694671	G	A	0.44831
rs11673	3.4e-09	xIXa8m	chr19	33437095	C	T	0.11332
rs11693	9.33e-19	xIXa8m	chr2	1.62E+08	A	T	0.416501
rs11695:	6.28e-09	xIXa8m	chr2	97045684	A	G	0.115308
rs11716	1.4e-10	xIXa8m	chr3	34687261	G	A	0.267396
rs11720	5.58e-12	xIXa8m	chr3	71011489	T	C	0.506958
rs11742(	2.19e-10	xIXa8m	chr5	92100296	C	T	0.115308
rs11756	6.2e-09	xIXa8m	chr6	99893898	A	T	0.129225
rs11873:	1.63e-12	xIXa8m	chr18	45079957	T	C	0.131213
rs11926:	1.16e-08	xIXa8m	chr3	55902389	G	A	0.04672

rs12027	1.54e-13	xIXa8m	chr1	1.54E+08	C	T	0.151093
rs12079	3.88e-08	xIXa8m	chr1	1.74E+08	G	A	0.447316
rs12133	1.15e-12	xIXa8m	chr1	90749157	A	C	0.371769
rs12209	1.29e-10	xIXa8m	chr6	37404594	G	C	0.530815
rs12441	6.09e-14	xIXa8m	chr15	83253635	A	C	0.166998
rs12450	4.46e-08	xIXa8m	chr17	53783355	C	T	0.347913
rs12453	1.9e-09	xIXa8m	chr17	39613752	T	C	0.685885
rs12462	1.27e-11	xIXa8m	chr9	84146830	C	T	0.701789
rs12465	6.85e-10	xIXa8m	chr2	49917183	C	T	0.370775
rs12485	1.89e-08	xIXa8m	chr3	1.81E+08	A	C	0.12326
rs12518	1.22e-08	xIXa8m	chr5	1.1E+08	T	C	0.345924
rs12632	1.18e-16	xIXa8m	chr3	50186792	G	A	0.663022
rs12642	5.98e-11	xIXa8m	chr4	28025554	T	G	0.757455
rs12685	1.66e-10	xIXa8m	chr9	8290929	A	G	0.150099
rs12714	6.57e-10	xIXa8m	chr2	80772273	C	T	0.532803
rs12742	2.74e-10	xIXa8m	chr1	33411456	T	G	0.630219
rs12918	2.12e-13	xIXa8m	chr10	11040229	T	G	0.531809
rs12923	4.96e-12	xIXa8m	chr16	17481208	T	C	0.219682
rs12936	6.57e-11	xIXa8m	chr17	52213595	T	C	0.293241
rs12945	1.79e-08	xIXa8m	chr17	82046218	C	T	0.616302
rs13009	6.92e-09	xIXa8m	chr2	1.73E+08	G	A	0.651093
rs13030	2.42e-39	xIXa8m	chr2	1.45E+08	A	G	0.508946
rs13145	6.87e-14	xIXa8m	chr4	1.4E+08	C	G	0.360835
rs13162	1.15e-08	xIXa8m	chr5	12122586	T	A	0.33499
rs13255	4.03e-12	xIXa8m	chr8	1.43E+08	G	A	0.695825
rs13261	3.43e-13	xIXa8m	chr8	90854069	C	G	0.261431
rs13301	3.13e-11	xIXa8m	chr9	1.26E+08	A	G	0.348907
rs13377	2.16e-20	xIXa8m	chr10	61915512	A	T	0.491054
rs13398	2.09e-11	xIXa8m	chr2	2.03E+08	A	T	0.259443
rs13452	8.86e-13	xIXa8m	chr22	28385770	C	T	0.379722
rs13739	4.01e-12	xIXa8m	chr1	72435832	T	C	0.186879
rs13812	7.51e-15	xIXa8m	chr14	98131215	T	C	0.50497
rs13828	1.31e-10	xIXa8m	chr9	38268928	GA	G	0.178926
rs13924	5.6e-10	xIXa8m	chr5	30831280	T	C	0.532803
rs14185	6.39e-11	xIXa8m	chr9	1.35E+08	TCA	T	0.192843
rs14346	2.34e-08	xIXa8m	chr6	1.58E+08	T	C	0.052684
rs14356	2.86e-10	xIXa8m	chr15	36107044	A	G	0.581511
rs14390	3.74e-12	xIXa8m	chr2	59067062	C	T	0.107356

rs14662(	1.43e-14	xIXa8m	chr6	66841535	A	T	0.379722
rs14705	1.31e-08	xIXa8m	chr1	1.8E+08	T	G	0.014911
rs14852	4.07e-08	xIXa8m	chr3	3685905	C	T	0.37674
rs14906	2.84e-08	xIXa8m	chr4	1.54E+08	C	T	0.333996
rs14999	3.01e-23	xIXa8m	chr3	1.18E+08	T	C	0.835984
rs15032	3.43e-10	xIXa8m	chr4	93158357	A	G	0.454274
rs15592	9.97e-12	xIXa8m	chr5	51498387	C	T	0.348907
rs15657	7.49e-32	xIXa8m	chr8	27568560	A	T	0.199801
rs16063	3.56e-09	xIXa8m	chr6	53030432	G	T	0.728628
rs16896	5.61e-09	xIXa8m	chr6	65078002	T	A	0.166998
rs16975	5.29e-09	xIXa8m	chr18	41689686	A	C	0.10835
rs17145	1.22e-10	xIXa8m	chr3	1.59E+08	C	A	0.378728
rs17203	1.56e-08	xIXa8m	chr19	18522945	C	T	0.425447
rs17337	1.23e-10	xIXa8m	chr10	54940461	G	A	0.49503
rs17432	2.55e-08	xIXa8m	chr2	63239328	T	C	0.224652
rs17594	1.27e-09	xIXa8m	chr14	79152407	G	A	0.416501
rs17766	3.92e-08	xIXa8m	chr10	31133648	C	T	0.774354
rs19242	3.22e-10	xIXa8m	chr10	9960737	G	C	0.373757
rs19247	1.73e-08	xIXa8m	chr20	19678718	T	C	0.500994
rs19374	1.27e-18	xIXa8m	chr1	66003960	G	C	0.568588
rs19457	8.19e-10	xIXa8m	chr18	56077314	C	T	0.514911
rs19713	7.8e-13	xIXa8m	chr12	1.21E+08	T	C	0.129225
rs19942	1.25e-14	xIXa8m	chr2	1.55E+08	T	G	0.514911
rs19993	3.34e-08	xIXa8m	chr9	31996050	T	TA	0.101392
rs20228	1.12e-09	xIXa8m	chr14	27887527	A	G	0.335984
rs20721	1.16e-13	xIXa8m	chr7	78133140	C	T	0.699801
rs21351(	8.23e-09	xIXa8m	chr2	2.12E+08	C	T	0.091451
rs21634	3.06e-11	xIXa8m	chr2	2.25E+08	G	A	0.175944
rs21730	3.47e-10	xIXa8m	chr5	1.68E+08	A	T	0.170974
rs21825	2.53e-13	xIXa8m	chr10	8789196	T	C	0.260437
rs22373(	6.37e-10	xIXa8m	chr7	21443987	A	G	0.656064
rs22897	2.87e-12	xIXa8m	chr15	67184614	T	G	0.240557
rs23135(	1.99e-12	xIXa8m	chr5	1.55E+08	T	C	0.244533
rs24177(	1.47e-08	xIXa8m	chr9	1.07E+08	A	G	0.38171
rs27086	9.99e-10	xIXa8m	chr1	8387344	T	C	0.691849
rs27831	2.71e-08	xIXa8m	chr13	79596025	G	A	0.502982
rs28765	1.88e-08	xIXa8m	chr6	1.45E+08	A	G	0.407555
rs29397	6.87e-11	xIXa8m	chr11	41414747	A	G	0.489066

rs31105!	1.22e-09	xIXa8m	chr18	30228238	A	C	0.248509
rs32138	5.08e-12	xIXa8m	chr18	75472023	C	T	0.315109
rs32634	6.16e-10	xIXa8m	chr3	1.08E+08	A	G	0.462227
rs34367(	1.36e-13	xIXa8m	chr2	1.37E+08	T	C	0.170974
rs34488(	8.73e-20	xIXa8m	chr15	47392739	C	T	0.206759
rs34638	2.49e-08	xIXa8m	chr3	1.47E+08	A	G	0.360835
rs35891!	6.52e-10	xIXa8m	chr11	20107765	A	G	0.071571
rs35984	3.91e-09	xIXa8m	chr16	13668159	TA	T	0.16004
rs37624(	2.47e-08	xIXa8m	chr2	1.32E+08	C	T	0.326044
rs37812!	1.13e-12	xIXa8m	chr10	1.02E+08	A	G	0.387674
rs38012	3.64e-12	xIXa8m	chr7	97008955	C	A	0.363817
rs38149!	7.44e-09	xIXa8m	chr7	88798071	T	G	0.373757
rs38959(	1.56e-14	xIXa8m	chr1	74540343	G	A	0.548708
rs39051	4.28e-11	xIXa8m	chr1	2.37E+08	T	C	0.542744
rs39784	6.41e-11	xIXa8m	chr5	1.71E+08	A	C	0.722664
rs40426	1.55e-10	xIXa8m	chr12	1.25E+08	T	C	0.65507
rs40443	3.04e-23	xIXa8m	chr5	1.68E+08	G	A	0.668986
rs43743	3.73e-12	xIXa8m	chr2	1.81E+08	T	C	0.764414
rs44016!	9.42e-09	xIXa8m	chr6	1.57E+08	G	C	0.531809
rs44497	5.08e-09	xIXa8m	chr8	91765834	C	T	0.692843
rs44795	2.11e-08	xIXa8m	chr3	5682131	T	C	0.498012
rs45435!	2.51e-14	xIXa8m	chr9	3014254	C	T	0.49006
rs45715(	1.64e-21	xIXa8m	chr5	88461101	T	C	0.481113
rs45795(	1.23e-14	xIXa8m	chr8	64001205	A	G	0.499006
rs46564(	1.7e-28	xIXa8m	chr6	1.11E+08	A	G	0.849901
rs46598(	8.36e-09	xIXa8m	chr1	2.38E+08	T	G	0.605368
rs47227	2.88e-14	xIXa8m	chr7	3358544	A	T	0.442346
rs47516	6.35e-11	xIXa8m	chr10	1.17E+08	T	A	0.23161
rs47851	2.03e-10	xIXa8m	chr16	49732861	A	G	0.244533
rs48190	1.34e-08	xIXa8m	chr21	45075309	G	C	0.718688
rs48376	3.54e-09	xIXa8m	chr9	1.19E+08	T	C	0.457256
rs48571	3.03e-08	xIXa8m	chr3	94494239	T	C	0.406561
rs48884	6.84e-10	xIXa8m	chr16	75656381	G	A	0.039761
rs49448	2.33e-10	xIXa8m	chr11	73600660	C	T	0.84493
rs54035(	4.07e-13	xIXa8m	chr11	1.32E+08	A	C	0.370775
rs54086(	3.18e-13	xIXa8m	chr11	1.22E+08	G	A	0.562624
rs55173!	4.42e-09	xIXa8m	chr12	40020273	T	A	0.694831
rs55754	2.82e-08	xIXa8m	chr6	1.09E+08	C	T	0.356859

rs55921:	1.9e-12	xIXa8m	chr1	2.1E+08	C	T	0.240557
rs55942:	4.08e-11	xIXa8m	chr16	49592861	A	G	0.079523
rs56169(	1e-08	xIXa8m	chr1	18113497	A	G	0.441352
rs56348:	1.81e-08	xIXa8m	chr9	16749267	G	A	0.142147
rs56820:	2.25e-10	xIXa8m	chr20	55812318	T	C	0.354871
rs56987:	3.44e-12	xIXa8m	chr6	50911842	T	C	0.124254
rs57483:	2.33e-10	xIXa8m	chr11	64343196	A	G	0.365805
rs58400:	5.03e-12	xIXa8m	chr4	31182862	A	G	0.385686
rs58547:	1.73e-08	xIXa8m	chr19	18351898	A	G	0.245527
rs58866(	8.05e-09	xIXa8m	chr1	96421048	A	G	0.580517
rs60453:	1.06e-09	xIXa8m	chr10	85602822	A	T	0.150099
rs60886:	5.89e-11	xIXa8m	chr20	34821547	A	G	0.4334
rs61413:	3.59e-13	xIXa8m	chr20	32505711	A	G	0.215706
rs61533:	1.5e-10	xIXa8m	chr2	22360096	C	T	0.377734
rs61959:	2.06e-10	xIXa8m	chr13	55260794	A	G	0.203777
rs62181:	4.47e-10	xIXa8m	chr2	2E+08	A	G	0.559642
rs62254:	3.73e-09	xIXa8m	chr3	84575615	A	G	0.014911
rs62258:	7.2e-12	xIXa8m	chr3	75053282	G	C	0.127237
rs62266:	3.06e-08	xIXa8m	chr3	1.29E+08	G	C	0.072565
rs62419:	1.08e-08	xIXa8m	chr6	83647240	A	T	0.122266
rs62459:	2.7e-08	xIXa8m	chr7	71106657	C	T	0.305169
rs64382(	7.33e-09	xIXa8m	chr3	1.14E+08	A	G	0.248509
rs64640:	3.38e-14	xIXa8m	chr7	1648733	T	C	0.437376
rs64722:	1.43e-08	xIXa8m	chr8	65880397	G	T	0.375746
rs64978:	1.13e-12	xIXa8m	chr16	25340312	A	G	0.713718
rs65985:	5.76e-12	xIXa8m	chr15	98661254	C	T	0.512922
rs66480:	1.38e-08	xIXa8m	chr14	31978983	G	C	0.442346
rs66680:	1.13e-14	xIXa8m	chr3	85936174	T	G	0.430418
rs67287:	4.27e-19	xIXa8m	chr2	623976	C	T	0.83002
rs68521:	1.97e-13	xIXa8m	chr4	1.72E+08	G	C	0.40159
rs68688:	1.92e-08	xIXa8m	chr5	22193671	T	C	0.50497
rs69596:	7.29e-09	xIXa8m	chr7	1.11E+08	T	C	0.355865
rs69731(	2.7e-09	xIXa8m	chr7	1.15E+08	G	C	0.590457
rs71277:	2.19e-65	xIXa8m	chr11	1.13E+08	T	A	0.434394
rs71367:	1.7e-10	xIXa8m	chr18	79816337	A	G	0.161034
rs71491:	3.72e-10	xIXa8m	chr11	1.25E+08	G	C	0.074553
rs71627:	5.15e-12	xIXa8m	chr5	43125693	G	A	0.119284
rs71950:	2.13e-08	xIXa8m	chr16	89954453	T	C	0.444334

rs72055!	1.15e-09	xIXa8m	chr16	69572065	A	G	0.434394
rs72247	1.02e-11	xIXa8m	chr17	32330039	T	C	0.633201
rs72664!	1.85e-09	xIXa8m	chr1	31704540	C	T	0.119284
rs72789(	1.54e-13	xIXa8m	chr5	1.07E+08	T	C	0.134195
rs72836	6.74e-13	xIXa8m	chr17	46044213	C	T	0.213718
rs72904	1.59e-08	xIXa8m	chr11	46018464	C	G	0.217694
rs73335!	3.24e-13	xIXa8m	chr13	99894196	A	G	0.791252
rs74557(	8.7e-11	xIXa8m	chr17	79807926	G	A	0.49503
rs75855	1.29e-14	xIXa8m	chr2	59797722	G	C	0.516899
rs75887!	1e-08	xIXa8m	chr11	7922612	T	A	0.427435
rs76132	9.43e-09	xIXa8m	chr1	2.27E+08	T	C	0.055666
rs76293!	7.82e-09	xIXa8m	chr3	16807327	G	A	0.296223
rs76305	6.71e-16	xIXa8m	chr16	685921	C	T	0.267396
rs76608!	2.76e-13	xIXa8m	chr19	4474728	A	C	0.045726
rs76668(	1.18e-08	xIXa8m	chr4	56883982	C	T	0.393638
rs78045!	1.09e-12	xIXa8m	chr7	99521487	G	A	0.163022
rs78093(	1.2e-11	xIXa8m	chr7	70019380	A	G	0.285288
rs78175	2.02e-11	xIXa8m	chr21	39291329	C	T	0.11829
rs78297	1.3e-13	xIXa8m	chr8	58891277	C	T	0.560636
rs78303!	3.88e-09	xIXa8m	chr8	10976748	T	C	0.350895
rs79222!	1.53e-08	xIXa8m	chr6	1.65E+08	G	T	0.255467
rs79295	9.06e-10	xIXa8m	chr11	86269916	G	A	0.772366
rs79473!	7.55e-09	xIXa8m	chr11	66419411	G	A	0.577535
rs79695!	3.28e-10	xIXa8m	chr12	69261387	G	A	0.681909
rs79843	1.77e-08	xIXa8m	chr13	1E+08	A	G	0.387674
rs79860!	5.99e-09	xIXa8m	chr13	30455794	C	A	0.694831
rs80018	3.74e-08	xIXa8m	chr13	96457819	G	A	0.323062
rs80673(	4.95e-12	xIXa8m	chr17	2072363	A	G	0.574553
rs84678	4.8e-11	xIXa8m	chr6	1.01E+08	C	T	0.737575
rs85368	8.55e-13	xIXa8m	chr6	28326773	C	T	0.380716
rs88829	2.03e-09	xIXa8m	chr18	27631008	T	A	0.295229
rs91178	4.99e-08	xIXa8m	chr10	1.22E+08	G	A	0.568588
rs93233	7.25e-12	xIXa8m	chr14	58186796	G	A	0.526839
rs93753	1.41e-13	xIXa8m	chr6	98303804	A	G	0.293241
rs94020!	3.38e-09	xIXa8m	chr6	1.29E+08	T	G	0.691849
rs94232	9.64e-11	xIXa8m	chr10	1.24E+08	G	C	0.638171
rs95174(	3.78e-26	xIXa8m	chr1	43546066	A	G	0.636183
rs95290!	1.85e-13	xIXa8m	chr13	66365965	A	T	0.506958

rs953853	4.28e-08	xIXa8m	chr13	59962187	G	T	0.668986
rs954149	2.16e-08	xIXa8m	chr13	68686940	G	T	0.082505
rs962727	1.28e-09	xIXa8m	chr22	46046408	C	G	0.389662
rs967931	4.52e-23	xIXa8m	chr2	1.04E+08	G	T	0.56163
rs976322	5.38e-12	xIXa8m	chr5	60961623	G	A	0.26839
rs983577	4.64e-13	xIXa8m	chr3	85716875	T	A	0.247515
rs993700	1e-13	xIXa8m	chr4	66960176	C	T	0.767396
rs998737	1.02e-17	xIXa8m	chr8	92177786	G	T	0.569583

beta.expos	se.expos	pval.exp	samplesi	exposure	mr_keep	pval_ori	id.expos	data_sou
0.0103	0.002	2.28e-10	805431	SmkInit	TRUE	reported	DvDEdl	textfile
0.00901	0.002	1.8e-08	781945	SmkInit	TRUE	reported	DvDEdl	textfile
0.00969	0.002	8.01e-10	805431	SmkInit	TRUE	reported	DvDEdl	textfile
0.0139	0.002	1.49e-18	805431	SmkInit	TRUE	reported	DvDEdl	textfile
-0.0123	0.002	4.83e-13	805431	SmkInit	TRUE	reported	DvDEdl	textfile
-0.0122	0.002	6.87e-14	805431	SmkInit	TRUE	reported	DvDEdl	textfile
0.0171	0.002	2.17e-18	805431	SmkInit	TRUE	reported	DvDEdl	textfile
-0.0105	0.002	3.71e-11	805431	SmkInit	TRUE	reported	DvDEdl	textfile
-0.00962	0.002	1.97e-08	748334	SmkInit	TRUE	reported	DvDEdl	textfile
0.0109	0.002	9.96e-11	780334	SmkInit	TRUE	reported	DvDEdl	textfile
0.0179	0.002	7.91e-27	748334	SmkInit	TRUE	reported	DvDEdl	textfile
-0.0127	0.002	2.39e-10	805431	SmkInit	TRUE	reported	DvDEdl	textfile
-0.0113	0.002	1.54e-12	805431	SmkInit	TRUE	reported	DvDEdl	textfile
-0.00966	0.002	4.78e-09	805431	SmkInit	TRUE	reported	DvDEdl	textfile
-0.0126	0.002	2.77e-15	805431	SmkInit	TRUE	reported	DvDEdl	textfile
-0.0125	0.002	8.22e-09	805431	SmkInit	TRUE	reported	DvDEdl	textfile
-0.00971	0.002	3.4e-09	805431	SmkInit	TRUE	reported	DvDEdl	textfile
0.0108	0.002	6.47e-10	805431	SmkInit	TRUE	reported	DvDEdl	textfile
-0.0101	0.002	3.83e-10	805431	SmkInit	TRUE	reported	DvDEdl	textfile
0.0104	0.002	4.15e-11	805431	SmkInit	TRUE	reported	DvDEdl	textfile
-0.0108	0.002	5.22e-09	624475	SmkInit	TRUE	reported	DvDEdl	textfile
-0.0227	0.003	2.56e-11	805431	SmkInit	TRUE	reported	DvDEdl	textfile
0.0114	0.002	3.34e-08	697341	SmkInit	TRUE	reported	DvDEdl	textfile
-0.0209	0.003	1.45e-12	805431	SmkInit	TRUE	reported	DvDEdl	textfile
0.0102	0.002	7.72e-10	805430	SmkInit	TRUE	reported	DvDEdl	textfile
-0.0151	0.002	2.73e-12	805431	SmkInit	TRUE	reported	DvDEdl	textfile
0.00898	0.002	1.31e-08	805430	SmkInit	TRUE	reported	DvDEdl	textfile
0.015	0.003	3.4e-09	805431	SmkInit	TRUE	reported	DvDEdl	textfile
0.014	0.002	9.33e-19	805431	SmkInit	TRUE	reported	DvDEdl	textfile
0.0138	0.002	6.28e-09	799280	SmkInit	TRUE	reported	DvDEdl	textfile
0.0117	0.002	1.4e-10	805431	SmkInit	TRUE	reported	DvDEdl	textfile
0.0109	0.002	5.58e-12	805431	SmkInit	TRUE	reported	DvDEdl	textfile
0.0157	0.002	2.19e-10	805431	SmkInit	TRUE	reported	DvDEdl	textfile
-0.0137	0.002	6.2e-09	805431	SmkInit	TRUE	reported	DvDEdl	textfile
-0.0155	0.002	1.63e-12	805431	SmkInit	TRUE	reported	DvDEdl	textfile
0.0206	0.004	1.16e-08	805431	SmkInit	TRUE	reported	DvDEdl	textfile

-0.0184	0.002	1.54e-13	732296	SmkInit	TRUE	reported	DvDEdl	textfile
-0.00872	0.002	3.88e-08	805431	SmkInit	TRUE	reported	DvDEdl	textfile
0.0116	0.002	1.15e-12	805431	SmkInit	TRUE	reported	DvDEdl	textfile
-0.0101	0.002	1.29e-10	805431	SmkInit	TRUE	reported	DvDEdl	textfile
-0.015	0.002	6.09e-14	805430	SmkInit	TRUE	reported	DvDEdl	textfile
-0.0108	0.002	4.46e-08	577967	SmkInit	TRUE	reported	DvDEdl	textfile
-0.0102	0.002	1.9e-09	805431	SmkInit	TRUE	reported	DvDEdl	textfile
0.0116	0.002	1.27e-11	805431	SmkInit	TRUE	reported	DvDEdl	textfile
-0.0101	0.002	6.85e-10	805431	SmkInit	TRUE	reported	DvDEdl	textfile
0.0138	0.002	1.89e-08	805431	SmkInit	TRUE	reported	DvDEdl	textfile
0.00992	0.002	1.22e-08	748334	SmkInit	TRUE	reported	DvDEdl	textfile
-0.0138	0.002	1.18e-16	805431	SmkInit	TRUE	reported	DvDEdl	textfile
-0.0124	0.002	5.98e-11	748334	SmkInit	TRUE	reported	DvDEdl	textfile
0.0145	0.002	1.66e-10	781945	SmkInit	TRUE	reported	DvDEdl	textfile
0.0103	0.002	6.57e-10	723237	SmkInit	TRUE	reported	DvDEdl	textfile
0.0119	0.002	2.74e-10	630626	SmkInit	TRUE	reported	DvDEdl	textfile
0.0116	0.002	2.12e-13	805431	SmkInit	TRUE	reported	DvDEdl	textfile
-0.0136	0.002	4.96e-12	805431	SmkInit	TRUE	reported	DvDEdl	textfile
0.0111	0.002	6.57e-11	805431	SmkInit	TRUE	reported	DvDEdl	textfile
-0.00941	0.002	1.79e-08	804239	SmkInit	TRUE	reported	DvDEdl	textfile
-0.0097	0.002	6.92e-09	805431	SmkInit	TRUE	reported	DvDEdl	textfile
0.0207	0.002	2.42e-39	805431	SmkInit	TRUE	reported	DvDEdl	textfile
-0.0122	0.002	6.87e-14	805431	SmkInit	TRUE	reported	DvDEdl	textfile
0.00975	0.002	1.15e-08	780334	SmkInit	TRUE	reported	DvDEdl	textfile
0.0116	0.002	4.03e-12	805431	SmkInit	TRUE	reported	DvDEdl	textfile
0.0131	0.002	3.43e-13	805431	SmkInit	TRUE	reported	DvDEdl	textfile
0.0109	0.002	3.13e-11	805431	SmkInit	TRUE	reported	DvDEdl	textfile
-0.0146	0.002	2.16e-20	805431	SmkInit	TRUE	reported	DvDEdl	textfile
0.0119	0.002	2.09e-11	805431	SmkInit	TRUE	reported	DvDEdl	textfile
-0.0116	0.002	8.86e-13	805431	SmkInit	TRUE	reported	DvDEdl	textfile
-0.0139	0.002	4.01e-12	805431	SmkInit	TRUE	reported	DvDEdl	textfile
0.0123	0.002	7.51e-15	805431	SmkInit	TRUE	reported	DvDEdl	textfile
0.0154	0.002	1.31e-10	630626	SmkInit	TRUE	reported	DvDEdl	textfile
0.00981	0.002	5.6e-10	805431	SmkInit	TRUE	reported	DvDEdl	textfile
0.0152	0.002	6.39e-11	472832	SmkInit	TRUE	reported	DvDEdl	textfile
-0.02	0.004	2.34e-08	805431	SmkInit	TRUE	reported	DvDEdl	textfile
0.01	0.002	2.86e-10	805430	SmkInit	TRUE	reported	DvDEdl	textfile
0.0176	0.003	3.74e-12	805431	SmkInit	TRUE	reported	DvDEdl	textfile

0.0144	0.002	1.43e-14	603064	SmkInit	TRUE	reported	DvDEdl	textfile
0.0344	0.006	1.31e-08	804537	SmkInit	TRUE	reported	DvDEdl	textfile
-0.00907	0.002	4.07e-08	805431	SmkInit	TRUE	reported	DvDEdl	textfile
-0.00982	0.002	2.84e-08	672245	SmkInit	TRUE	reported	DvDEdl	textfile
0.0216	0.002	3.01e-23	805431	SmkInit	TRUE	reported	DvDEdl	textfile
0.00999	0.002	3.43e-10	805431	SmkInit	TRUE	reported	DvDEdl	textfile
-0.0111	0.002	9.97e-12	805431	SmkInit	TRUE	reported	DvDEdl	textfile
-0.0232	0.002	7.49e-32	805431	SmkInit	TRUE	reported	DvDEdl	textfile
-0.0105	0.002	3.56e-09	805431	SmkInit	TRUE	reported	DvDEdl	textfile
0.0118	0.002	5.61e-09	805431	SmkInit	TRUE	reported	DvDEdl	textfile
-0.0172	0.003	5.29e-09	805431	SmkInit	TRUE	reported	DvDEdl	textfile
-0.0103	0.002	1.22e-10	805431	SmkInit	TRUE	reported	DvDEdl	textfile
0.00901	0.002	1.56e-08	805431	SmkInit	TRUE	reported	DvDEdl	textfile
0.0101	0.002	1.23e-10	805431	SmkInit	TRUE	reported	DvDEdl	textfile
-0.0105	0.002	2.55e-08	805431	SmkInit	TRUE	reported	DvDEdl	textfile
0.00966	0.002	1.27e-09	805431	SmkInit	TRUE	reported	DvDEdl	textfile
-0.0106	0.002	3.92e-08	805431	SmkInit	TRUE	reported	DvDEdl	textfile
-0.011	0.002	3.22e-10	697342	SmkInit	TRUE	reported	DvDEdl	textfile
0.0116	0.002	1.73e-08	472832	SmkInit	TRUE	reported	DvDEdl	textfile
0.014	0.002	1.27e-18	805431	SmkInit	TRUE	reported	DvDEdl	textfile
-0.00968	0.002	8.19e-10	805431	SmkInit	TRUE	reported	DvDEdl	textfile
0.0156	0.002	7.8e-13	805431	SmkInit	TRUE	reported	DvDEdl	textfile
0.0122	0.002	1.25e-14	805431	SmkInit	TRUE	reported	DvDEdl	textfile
-0.0156	0.003	3.34e-08	697342	SmkInit	TRUE	reported	DvDEdl	textfile
0.0104	0.002	1.12e-09	805431	SmkInit	TRUE	reported	DvDEdl	textfile
0.0128	0.002	1.16e-13	805431	SmkInit	TRUE	reported	DvDEdl	textfile
0.0157	0.003	8.23e-09	748334	SmkInit	TRUE	reported	DvDEdl	textfile
-0.0138	0.002	3.06e-11	796935	SmkInit	TRUE	reported	DvDEdl	textfile
0.0131	0.002	3.47e-10	805431	SmkInit	TRUE	reported	DvDEdl	textfile
0.0135	0.002	2.53e-13	805431	SmkInit	TRUE	reported	DvDEdl	textfile
-0.0102	0.002	6.37e-10	805431	SmkInit	TRUE	reported	DvDEdl	textfile
-0.0128	0.002	2.87e-12	805430	SmkInit	TRUE	reported	DvDEdl	textfile
0.0128	0.002	1.99e-12	805431	SmkInit	TRUE	reported	DvDEdl	textfile
-0.0105	0.002	1.47e-08	630626	SmkInit	TRUE	reported	DvDEdl	textfile
-0.0102	0.002	9.99e-10	805431	SmkInit	TRUE	reported	DvDEdl	textfile
-0.00877	0.002	2.71e-08	805431	SmkInit	TRUE	reported	DvDEdl	textfile
0.00905	0.002	1.88e-08	805431	SmkInit	TRUE	reported	DvDEdl	textfile
-0.0103	0.002	6.87e-11	805431	SmkInit	TRUE	reported	DvDEdl	textfile

0.0111	0.002	1.22e-09	805431	SmkInit	TRUE	reported	DvDEdl	textfile
0.0115	0.002	5.08e-12	805431	SmkInit	TRUE	reported	DvDEdl	textfile
-0.0101	0.002	6.16e-10	748334	SmkInit	TRUE	reported	DvDEdl	textfile
0.0138	0.002	1.36e-13	805431	SmkInit	TRUE	reported	DvDEdl	textfile
0.0176	0.002	8.73e-20	805430	SmkInit	TRUE	reported	DvDEdl	textfile
-0.00987	0.002	2.49e-08	697342	SmkInit	TRUE	reported	DvDEdl	textfile
-0.019	0.003	6.52e-10	805431	SmkInit	TRUE	reported	DvDEdl	textfile
0.0153	0.003	3.91e-09	630626	SmkInit	TRUE	reported	DvDEdl	textfile
0.00938	0.002	2.47e-08	805431	SmkInit	TRUE	reported	DvDEdl	textfile
-0.0116	0.002	1.13e-12	805431	SmkInit	TRUE	reported	DvDEdl	textfile
-0.0115	0.002	3.64e-12	805431	SmkInit	TRUE	reported	DvDEdl	textfile
0.00962	0.002	7.44e-09	805431	SmkInit	TRUE	reported	DvDEdl	textfile
-0.0129	0.002	1.56e-14	723237	SmkInit	TRUE	reported	DvDEdl	textfile
0.0104	0.002	4.28e-11	805431	SmkInit	TRUE	reported	DvDEdl	textfile
0.0115	0.002	6.41e-11	805431	SmkInit	TRUE	reported	DvDEdl	textfile
-0.0112	0.002	1.55e-10	697342	SmkInit	TRUE	reported	DvDEdl	textfile
-0.0164	0.002	3.04e-23	805431	SmkInit	TRUE	reported	DvDEdl	textfile
0.0127	0.002	3.73e-12	805431	SmkInit	TRUE	reported	DvDEdl	textfile
-0.00951	0.002	9.42e-09	736960	SmkInit	TRUE	reported	DvDEdl	textfile
-0.00992	0.002	5.08e-09	805431	SmkInit	TRUE	reported	DvDEdl	textfile
0.00898	0.002	2.11e-08	780334	SmkInit	TRUE	reported	DvDEdl	textfile
0.012	0.002	2.51e-14	805431	SmkInit	TRUE	reported	DvDEdl	textfile
-0.0151	0.002	1.64e-21	805431	SmkInit	TRUE	reported	DvDEdl	textfile
0.0122	0.002	1.23e-14	805431	SmkInit	TRUE	reported	DvDEdl	textfile
-0.0236	0.002	1.7e-28	805431	SmkInit	TRUE	reported	DvDEdl	textfile
0.00936	0.002	8.36e-09	805431	SmkInit	TRUE	reported	DvDEdl	textfile
0.0131	0.002	2.88e-14	675199	SmkInit	TRUE	reported	DvDEdl	textfile
0.0122	0.002	6.35e-11	805431	SmkInit	TRUE	reported	DvDEdl	textfile
0.0119	0.002	2.03e-10	805431	SmkInit	TRUE	reported	DvDEdl	textfile
-0.00989	0.002	1.34e-08	805431	SmkInit	TRUE	reported	DvDEdl	textfile
-0.00949	0.002	3.54e-09	781945	SmkInit	TRUE	reported	DvDEdl	textfile
0.00881	0.002	3.03e-08	805431	SmkInit	TRUE	reported	DvDEdl	textfile
-0.0245	0.004	6.84e-10	805431	SmkInit	TRUE	reported	DvDEdl	textfile
-0.0133	0.002	2.33e-10	805431	SmkInit	TRUE	reported	DvDEdl	textfile
0.0116	0.002	4.07e-13	805431	SmkInit	TRUE	reported	DvDEdl	textfile
0.0117	0.002	3.18e-13	780334	SmkInit	TRUE	reported	DvDEdl	textfile
0.0102	0.002	4.42e-09	805431	SmkInit	TRUE	reported	DvDEdl	textfile
-0.00945	0.002	2.82e-08	805431	SmkInit	TRUE	reported	DvDEdl	textfile

-0.0136	0.002	1.9e-12	805431	SmkInit	TRUE	reported	DvDEdl	textfile
0.0173	0.003	4.08e-11	805431	SmkInit	TRUE	reported	DvDEdl	textfile
0.00906	0.002	1e-08	805431	SmkInit	TRUE	reported	DvDEdl	textfile
0.0126	0.002	1.81e-08	799280	SmkInit	TRUE	reported	DvDEdl	textfile
-0.0108	0.002	2.25e-10	744041	SmkInit	TRUE	reported	DvDEdl	textfile
-0.0179	0.003	3.44e-12	805431	SmkInit	TRUE	reported	DvDEdl	textfile
-0.0103	0.002	2.33e-10	805431	SmkInit	TRUE	reported	DvDEdl	textfile
-0.0114	0.002	5.03e-12	805431	SmkInit	TRUE	reported	DvDEdl	textfile
-0.0106	0.002	1.73e-08	732296	SmkInit	TRUE	reported	DvDEdl	textfile
0.00921	0.002	8.05e-09	805431	SmkInit	TRUE	reported	DvDEdl	textfile
0.0127	0.002	1.06e-09	805431	SmkInit	TRUE	reported	DvDEdl	textfile
-0.0108	0.002	5.89e-11	744041	SmkInit	TRUE	reported	DvDEdl	textfile
0.0131	0.002	3.59e-13	801138	SmkInit	TRUE	reported	DvDEdl	textfile
0.0104	0.002	1.5e-10	805431	SmkInit	TRUE	reported	DvDEdl	textfile
-0.0123	0.002	2.06e-10	805431	SmkInit	TRUE	reported	DvDEdl	textfile
0.00989	0.002	4.47e-10	805431	SmkInit	TRUE	reported	DvDEdl	textfile
0.031	0.005	3.73e-09	800208	SmkInit	TRUE	reported	DvDEdl	textfile
-0.0158	0.002	7.2e-12	805431	SmkInit	TRUE	reported	DvDEdl	textfile
0.0154	0.003	3.06e-08	805431	SmkInit	TRUE	reported	DvDEdl	textfile
0.0131	0.002	1.08e-08	805431	SmkInit	TRUE	reported	DvDEdl	textfile
-0.00942	0.002	2.7e-08	805431	SmkInit	TRUE	reported	DvDEdl	textfile
-0.0104	0.002	7.33e-09	805431	SmkInit	TRUE	reported	DvDEdl	textfile
-0.0121	0.002	3.38e-14	805431	SmkInit	TRUE	reported	DvDEdl	textfile
-0.00942	0.002	1.43e-08	805431	SmkInit	TRUE	reported	DvDEdl	textfile
0.0128	0.002	1.13e-12	748334	SmkInit	TRUE	reported	DvDEdl	textfile
0.011	0.002	5.76e-12	780333	SmkInit	TRUE	reported	DvDEdl	textfile
0.009	0.002	1.38e-08	805431	SmkInit	TRUE	reported	DvDEdl	textfile
-0.0125	0.002	1.13e-14	805431	SmkInit	TRUE	reported	DvDEdl	textfile
0.0187	0.002	4.27e-19	805431	SmkInit	TRUE	reported	DvDEdl	textfile
-0.0118	0.002	1.97e-13	805431	SmkInit	TRUE	reported	DvDEdl	textfile
0.0101	0.002	1.92e-08	624207	SmkInit	TRUE	reported	DvDEdl	textfile
0.00987	0.002	7.29e-09	805431	SmkInit	TRUE	reported	DvDEdl	textfile
-0.00946	0.002	2.7e-09	805431	SmkInit	TRUE	reported	DvDEdl	textfile
0.0273	0.002	2.19e-65	805431	SmkInit	TRUE	reported	DvDEdl	textfile
0.0124	0.002	1.7e-10	805431	SmkInit	TRUE	reported	DvDEdl	textfile
-0.0183	0.003	3.72e-10	805431	SmkInit	TRUE	reported	DvDEdl	textfile
-0.017	0.002	5.15e-12	805431	SmkInit	TRUE	reported	DvDEdl	textfile
-0.00883	0.002	2.13e-08	805431	SmkInit	TRUE	reported	DvDEdl	textfile

0.0102	0.002	1.15e-09	732296	SmkInit	TRUE	reported	DvDEdl	textfile
-0.011	0.002	1.02e-11	805431	SmkInit	TRUE	reported	DvDEdl	textfile
0.0143	0.002	1.85e-09	748334	SmkInit	TRUE	reported	DvDEdl	textfile
-0.0171	0.002	1.54e-13	805431	SmkInit	TRUE	reported	DvDEdl	textfile
-0.0132	0.002	6.74e-13	805431	SmkInit	TRUE	reported	DvDEdl	textfile
-0.0108	0.002	1.59e-08	805431	SmkInit	TRUE	reported	DvDEdl	textfile
-0.014	0.002	3.24e-13	805431	SmkInit	TRUE	reported	DvDEdl	textfile
-0.0104	0.002	8.7e-11	780334	SmkInit	TRUE	reported	DvDEdl	textfile
0.0126	0.002	1.29e-14	748334	SmkInit	TRUE	reported	DvDEdl	textfile
-0.0111	0.002	1e-08	557491	SmkInit	TRUE	reported	DvDEdl	textfile
0.018	0.003	9.43e-09	748334	SmkInit	TRUE	reported	DvDEdl	textfile
0.00986	0.002	7.82e-09	805431	SmkInit	TRUE	reported	DvDEdl	textfile
-0.0149	0.002	6.71e-16	799280	SmkInit	TRUE	reported	DvDEdl	textfile
-0.0267	0.004	2.76e-13	799016	SmkInit	TRUE	reported	DvDEdl	textfile
-0.0109	0.002	1.18e-08	573529	SmkInit	TRUE	reported	DvDEdl	textfile
-0.0152	0.002	1.09e-12	805431	SmkInit	TRUE	reported	DvDEdl	textfile
-0.0114	0.002	1.2e-11	805431	SmkInit	TRUE	reported	DvDEdl	textfile
0.0162	0.002	2.02e-11	805431	SmkInit	TRUE	reported	DvDEdl	textfile
-0.0117	0.002	1.3e-13	805431	SmkInit	TRUE	reported	DvDEdl	textfile
-0.0103	0.002	3.88e-09	697342	SmkInit	TRUE	reported	DvDEdl	textfile
0.0103	0.002	1.53e-08	805431	SmkInit	TRUE	reported	DvDEdl	textfile
0.0116	0.002	9.06e-10	805431	SmkInit	TRUE	reported	DvDEdl	textfile
0.00929	0.002	7.55e-09	805431	SmkInit	TRUE	reported	DvDEdl	textfile
-0.011	0.002	3.28e-10	805431	SmkInit	TRUE	reported	DvDEdl	textfile
0.00921	0.002	1.77e-08	805431	SmkInit	TRUE	reported	DvDEdl	textfile
0.00988	0.002	5.99e-09	805431	SmkInit	TRUE	reported	DvDEdl	textfile
0.0094	0.002	3.74e-08	780334	SmkInit	TRUE	reported	DvDEdl	textfile
0.011	0.002	4.95e-12	805431	SmkInit	TRUE	reported	DvDEdl	textfile
-0.0115	0.002	4.8e-11	805431	SmkInit	TRUE	reported	DvDEdl	textfile
-0.0116	0.002	8.55e-13	805431	SmkInit	TRUE	reported	DvDEdl	textfile
-0.0107	0.002	2.03e-09	748334	SmkInit	TRUE	reported	DvDEdl	textfile
-0.00878	0.002	4.99e-08	780334	SmkInit	TRUE	reported	DvDEdl	textfile
-0.0108	0.002	7.25e-12	805431	SmkInit	TRUE	reported	DvDEdl	textfile
0.013	0.002	1.41e-13	805431	SmkInit	TRUE	reported	DvDEdl	textfile
0.0101	0.002	3.38e-09	805431	SmkInit	TRUE	reported	DvDEdl	textfile
-0.0108	0.002	9.64e-11	775111	SmkInit	TRUE	reported	DvDEdl	textfile
0.0172	0.002	3.78e-26	805431	SmkInit	TRUE	reported	DvDEdl	textfile
-0.0118	0.002	1.85e-13	780334	SmkInit	TRUE	reported	DvDEdl	textfile

-0.00936	0.002	4.28e-08	805431	SmkInit	TRUE	reported	DvDEdl	textfile
-0.0165	0.003	2.16e-08	752772	SmkInit	TRUE	reported	DvDEdl	textfile
-0.00991	0.002	1.28e-09	780334	SmkInit	TRUE	reported	DvDEdl	textfile
0.0175	0.002	4.52e-23	640245	SmkInit	TRUE	reported	DvDEdl	textfile
0.0139	0.002	5.38e-12	624207	SmkInit	TRUE	reported	DvDEdl	textfile
0.0133	0.002	4.64e-13	805431	SmkInit	TRUE	reported	DvDEdl	textfile
-0.0141	0.002	1e-13	805431	SmkInit	TRUE	reported	DvDEdl	textfile
-0.0137	0.002	1.02e-17	805431	SmkInit	TRUE	reported	DvDEdl	textfile

rce.exposure

Supplementary Table S4： SNPs used as proxies for number of cigarettes sm

SNP	pval	id	chr.expo	pos.exposu	effect_al	other_all	eaf.exposu
rs10530(	1.43e-11	DPfPSg	chr16	69652773	CTTTTT	C	0.577535
rs10665:	2.18e-13	DPfPSg	chr14	1.04E+08	AAAG	A	0.296223
rs11076:	1.06e-10	DPfPSg	chr16	52063640	A	C	0.632207
rs11217(	8.02e-10	DPfPSg	chr17	29236995	T	C	0.181909
rs11663:	1.75e-08	DPfPSg	chr18	64485223	T	A	0.592445
rs11686(	3.28e-10	DPfPSg	chr2	1.48E+08	C	T	0.286282
rs11725(	5.71e-11	DPfPSg	chr4	66188051	C	T	0.313121
rs11921(	2.33e-08	DPfPSg	chr3	85385110	C	T	0.601392
rs11928:	2.71e-09	DPfPSg	chr3	48702450	C	T	0.651093
rs11940	2.94e-08	DPfPSg	chr4	98588302	A	T	0.654076
rs12510	1.63e-08	DPfPSg	chr4	95190496	T	A	0.670974
rs12660(	4.24e-08	DPfPSg	chr6	97522873	C	T	0.043738
rs13254:	4.81e-33	DPfPSg	chr8	42690703	C	G	0.777336
rs13875(	5.71e-13	DPfPSg	chr15	78719146	C	G	0.025845
rs14114	3.61e-10	DPfPSg	chr15	78542897	G	C	0.022863
rs14440:	2.15e-08	DPfPSg	chr8	1.37E+08	G	T	0.446322
rs14510	1.09e-08	DPfPSg	chr21	39272184	T	C	0.125249
rs15055	4.92e-08	DPfPSg	chr2	1.35E+08	C	A	0.011928
rs16579:	6.52e-10	DPfPSg	chr15	56820815	T	C	0.781312
rs17197	4.52e-11	DPfPSg	chr11	46498752	C	T	0.084493
rs17378(	1.69e-14	DPfPSg	chr20	32466899	G	C	0.377734
rs18577	1.2e-13	DPfPSg	chr15	78828147	T	G	0.023857
rs20169(	2.04e-14	DPfPSg	chr16	89706498	G	C	0.571571
rs20602:	2.16e-08	DPfPSg	chr5	1.67E+08	T	A	0.133201
rs20726:	6.44e-13	DPfPSg	chr1	1.55E+08	G	C	0.101392
rs21332(	2.42e-09	DPfPSg	chr1	77508845	T	C	0.450298
rs21560(	2.81e-18	DPfPSg	chr7	32294030	A	G	0.667992
rs22735(	7.09e-24	DPfPSg	chr20	63355597	C	T	0.146123
rs26550(	1.78e-08	DPfPSg	chr3	1.36E+08	T	A	0.730616
rs30253(	6.01e-22	DPfPSg	chr9	1.34E+08	C	T	0.180915
rs34370(	2.36e-08	DPfPSg	chr1	1.67E+08	T	C	0.175944
rs34406:	1.21e-26	DPfPSg	chr19	40799625	A	C	0.025845
rs58036(	3.64e-09	DPfPSg	chr3	89320598	CT	C	0.44334
rs60783	1.22e-09	DPfPSg	chr20	11879643	A	G	0.420477
rs66038(	1.55e-08	DPfPSg	chr1	18424989	A	T	0.513916
rs66993:	3.05e-08	DPfPSg	chr1	34919004	T	C	0.877734

rs683178	6.51e-10	DPfPSg	chr4	67009830	A	C	0.558648
rs72738	1e-200	DPfPSg	chr15	78427490	C	G	0.370775
rs72976	1.46e-09	DPfPSg	chr19	4029786	G	A	0.184891
rs73229	1.65e-14	DPfPSg	chr8	27584610	A	C	0.11332
rs75994	8.42e-09	DPfPSg	chr2	60491212	T	C	0.417495
rs76780	9.6e-11	DPfPSg	chr4	2995467	A	G	0.378728
rs78023	1.82e-08	DPfPSg	chr7	74908852	A	T	0.746521
rs78744	6.64e-10	DPfPSg	chr9	1.34E+08	T	C	0.213718
rs79250	7.93e-09	DPfPSg	chr12	1.1E+08	T	C	0.176938
rs79280	2.22e-12	DPfPSg	chr11	1.14E+08	A	C	0.407555
rs79338	5.23e-13	DPfPSg	chr11	16355573	T	C	0.319085
rs79442	4.59e-10	DPfPSg	chr11	43673824	G	A	0.409543
rs80292	1.24e-11	DPfPSg	chr6	26236840	G	A	0.295229
rs80625	5.53e-12	DPfPSg	chr8	63706458	A	G	0.732604
rs95222	6.5e-09	DPfPSg	chr13	1.12E+08	G	C	0.519881
rs96466	2.82e-08	DPfPSg	chr19	40967231	G	A	0.010934
rs98817	4.47e-09	DPfPSg	chr3	16805459	C	A	0.39165

ıoked per day

beta.expo	se.expos	pval.exp	samplesi	exposure	mr_keep	pval_ori	id.expos	data_sou
0.0203	0.003	1.43e-11	227321	CigDay	TRUE	reported	m6LWZ	textfile
0.023	0.003	2.18e-13	227321	CigDay	TRUE	reported	m6LWZ	textfile
-0.0164	0.003	1.06e-10	324557	CigDay	TRUE	reported	m6LWZ	textfile
0.0205	0.003	8.02e-10	326497	CigDay	TRUE	reported	m6LWZ	textfile
-0.0141	0.003	1.75e-08	326497	CigDay	TRUE	reported	m6LWZ	textfile
0.0165	0.003	3.28e-10	326497	CigDay	TRUE	reported	m6LWZ	textfile
0.0181	0.003	5.71e-11	326497	CigDay	TRUE	reported	m6LWZ	textfile
0.0141	0.003	2.33e-08	326497	CigDay	TRUE	reported	m6LWZ	textfile
-0.0166	0.003	2.71e-09	280613	CigDay	TRUE	reported	m6LWZ	textfile
-0.0146	0.003	2.94e-08	326497	CigDay	TRUE	reported	m6LWZ	textfile
-0.0155	0.003	1.63e-08	286982	CigDay	TRUE	reported	m6LWZ	textfile
-0.0303	0.006	4.24e-08	300003	CigDay	TRUE	reported	m6LWZ	textfile
0.0353	0.003	4.81e-33	326497	CigDay	TRUE	reported	m6LWZ	textfile
0.0628	0.009	5.71e-13	325689	CigDay	TRUE	reported	m6LWZ	textfile
-0.0588	0.009	3.61e-10	325625	CigDay	TRUE	reported	m6LWZ	textfile
0.014	0.002	2.15e-08	326497	CigDay	TRUE	reported	m6LWZ	textfile
0.0212	0.004	1.09e-08	326497	CigDay	TRUE	reported	m6LWZ	textfile
-0.0877	0.016	4.92e-08	282727	CigDay	TRUE	reported	m6LWZ	textfile
-0.0189	0.003	6.52e-10	326497	CigDay	TRUE	reported	m6LWZ	textfile
0.0298	0.005	4.52e-11	326497	CigDay	TRUE	reported	m6LWZ	textfile
0.0192	0.003	1.69e-14	326497	CigDay	TRUE	reported	m6LWZ	textfile
-0.054	0.007	1.2e-13	325812	CigDay	TRUE	reported	m6LWZ	textfile
-0.0193	0.003	2.04e-14	324557	CigDay	TRUE	reported	m6LWZ	textfile
0.0201	0.004	2.16e-08	326497	CigDay	TRUE	reported	m6LWZ	textfile
-0.0301	0.004	6.44e-13	324557	CigDay	TRUE	reported	m6LWZ	textfile
-0.0162	0.003	2.42e-09	280416	CigDay	TRUE	reported	m6LWZ	textfile
-0.0225	0.003	2.81e-18	326497	CigDay	TRUE	reported	m6LWZ	textfile
0.035	0.003	7.09e-24	320237	CigDay	TRUE	reported	m6LWZ	textfile
0.0161	0.003	1.78e-08	326497	CigDay	TRUE	reported	m6LWZ	textfile
-0.0307	0.003	6.01e-22	326497	CigDay	TRUE	reported	m6LWZ	textfile
-0.0192	0.003	2.36e-08	324557	CigDay	TRUE	reported	m6LWZ	textfile
-0.0796	0.007	1.21e-26	325689	CigDay	TRUE	reported	m6LWZ	textfile
-0.0175	0.003	3.64e-09	227321	CigDay	TRUE	reported	m6LWZ	textfile
0.0153	0.003	1.22e-09	326497	CigDay	TRUE	reported	m6LWZ	textfile
-0.014	0.002	1.55e-08	324557	CigDay	TRUE	reported	m6LWZ	textfile
-0.0207	0.004	3.05e-08	322348	CigDay	TRUE	reported	m6LWZ	textfile

-0.0161	0.003	6.51e-10	300003	CigDay	TRUE	reported	m6LWZ	textfile
0.0863	0.003	1e-200	326497	CigDay	TRUE	reported	m6LWZ	textfile
-0.0207	0.003	1.46e-09	319050	CigDay	TRUE	reported	m6LWZ	textfile
0.0304	0.004	1.65e-14	326497	CigDay	TRUE	reported	m6LWZ	textfile
0.0144	0.002	8.42e-09	326497	CigDay	TRUE	reported	m6LWZ	textfile
-0.018	0.003	9.6e-11	281992	CigDay	TRUE	reported	m6LWZ	textfile
-0.0223	0.004	1.82e-08	167512	CigDay	TRUE	reported	m6LWZ	textfile
-0.02	0.003	6.64e-10	300003	CigDay	TRUE	reported	m6LWZ	textfile
-0.0189	0.003	7.93e-09	326497	CigDay	TRUE	reported	m6LWZ	textfile
-0.0176	0.002	2.22e-12	326497	CigDay	TRUE	reported	m6LWZ	textfile
0.0194	0.003	5.23e-13	326497	CigDay	TRUE	reported	m6LWZ	textfile
-0.0156	0.003	4.59e-10	326497	CigDay	TRUE	reported	m6LWZ	textfile
-0.0194	0.003	1.24e-11	300003	CigDay	TRUE	reported	m6LWZ	textfile
-0.0197	0.003	5.53e-12	326497	CigDay	TRUE	reported	m6LWZ	textfile
0.0144	0.002	6.5e-09	326497	CigDay	TRUE	reported	m6LWZ	textfile
-0.0897	0.016	2.82e-08	200142	CigDay	TRUE	reported	m6LWZ	textfile
0.0147	0.003	4.47e-09	326497	CigDay	TRUE	reported	m6LWZ	textfile

ırce.exposure

Supplementary Table S5：Causal association between smoking exposur

exposure	outcome	method	nsnp	b	se	pval
Lifetime	aSAH(FinnGen)	Inverse v	117	0.91967	0.19270	1.81933
Lifetime	aSAH(FinnGen)	Weighte	117	0.99104	0.27657	0.00033
Lifetime	aSAH(FinnGen)	MR Egg	117	2.61218	0.78916	0.00124
Lifetime	aSAH(FinnGen)	Simple r	117	1.53247	0.82812	0.06678
Lifetime	aSAH(FinnGen)	Weighte	117	1.46666	0.91740	0.11260
Lifetime	aSAH(FinnGen)	Robust a	117	0.91820	0.20380	6.62612
Lifetime	aSAH(Bakker)	Inverse v	75	1.15948	0.34982	0.00091
Lifetime	aSAH(Bakker)	Weighte	75	1.15818	0.47334	0.01441
Lifetime	aSAH(Bakker)	MR Egg	75	5.46033	1.46900	0.00039
Lifetime	aSAH(Bakker)	Simple r	75	0.97523	1.21998	0.42662
Lifetime	aSAH(Bakker)	Weighte	75	1.01239	1.24096	0.41722
Lifetime	aSAH(Bakker)	Robust a	75	1.13724	0.35368	0.00130
SmkInit	aSAH(FinnGen)	Inverse v	212	0.29711	0.13052	0.02283
SmkInit	aSAH(FinnGen)	Weighte	212	0.45977	0.18328	0.01212
SmkInit	aSAH(FinnGen)	MR Egg	212	0.62316	0.57739	0.28170
SmkInit	aSAH(FinnGen)	Simple r	212	1.05195	0.61339	0.08781
SmkInit	aSAH(FinnGen)	Weighte	212	0.77583	0.53082	0.14534
SmkInit	aSAH(FinnGen)	Robust a	212	0.33236	0.13925	0.01699
CigDay	aSAH(FinnGen)	Inverse v	40	0.45275	0.13361	0.00070
CigDay	aSAH(FinnGen)	Weighte	40	0.46642	0.20037	0.01992
CigDay	aSAH(FinnGen)	MR Egg	40	0.64952	0.24688	0.01223
CigDay	aSAH(FinnGen)	Simple r	40	0.18610	0.40665	0.64973
CigDay	aSAH(FinnGen)	Weighte	40	0.47610	0.20413	0.02493
CigDay	aSAH(FinnGen)	Robust a	40	0.43730	0.13896	0.00165
Lifetime	uIAs(FinnGen)	Inverse v	118	1.09540	0.26851	4.51364
Lifetime	uIAs(FinnGen)	Weighte	118	0.92912	0.38537	0.01591
Lifetime	uIAs(FinnGen)	MR Egg	118	2.37639	1.11957	0.03591
Lifetime	uIAs(FinnGen)	Simple r	118	0.95748	1.08962	0.38135
Lifetime	uIAs(FinnGen)	Weighte	118	0.82470	1.05365	0.43537
Lifetime	uIAs(FinnGen)	Robust a	118	1.14055	0.27501	3.36557
Lifetime	uIAs(Bakker)	Inverse v	78	2.08613	0.50105	3.13426
Lifetime	uIAs(Bakker)	Weighte	78	2.36503	0.71243	0.00090
Lifetime	uIAs(Bakker)	MR Egg	78	1.81243	2.26685	0.42647
Lifetime	uIAs(Bakker)	Simple r	78	2.34684	1.94999	0.23246
Lifetime	uIAs(Bakker)	Weighte	78	2.46178	1.77165	0.16867
Lifetime	uIAs(Bakker)	Robust a	78	2.29879	0.53211	1.55934

SmkInit	uIAs(FinnGen)	Inverse v	212	0.39199	0.17079	0.02172
SmkInit	uIAs(FinnGen)	Weighte	212	0.31888	0.25038	0.20280
SmkInit	uIAs(FinnGen)	MR Egg	212	0.72772	0.75551	0.33654
SmkInit	uIAs(FinnGen)	Simple m	212	0.54166	0.82024	0.50973
SmkInit	uIAs(FinnGen)	Weighte	212	0.23489	0.76214	0.75822
SmkInit	uIAs(FinnGen)	Robust a	212	0.36430	0.17464	0.03698
CigDay	uIAs(FinnGen)	Inverse v	41	0.62949	0.19800	0.00147
CigDay	uIAs(FinnGen)	Weighte	41	0.79069	0.28742	0.00594
CigDay	uIAs(FinnGen)	MR Egg	41	0.92746	0.36738	0.01576
CigDay	uIAs(FinnGen)	Simple m	41	1.05468	0.57923	0.07611
CigDay	uIAs(FinnGen)	Weighte	41	0.85405	0.26876	0.00286
CigDay	uIAs(FinnGen)	Robust a	41	0.69926	0.19608	0.00036
Lifetime	uIAs and aSAH(Ba	Inverse v	83	1.40741	0.29070	1.28903
Lifetime	uIAs and aSAH(Ba	Weighte	83	1.56750	0.39351	6.79517
Lifetime	uIAs and aSAH(Ba	MR Egg	83	4.16896	1.22148	0.00100
Lifetime	uIAs and aSAH(Ba	Simple m	83	2.03702	1.08692	0.06447
Lifetime	uIAs and aSAH(Ba	Weighte	83	1.71269	1.01289	0.09465
Lifetime	uIAs and aSAH(Ba	Robust a	83	1.36251	0.30073	5.88143

e and intracranial aneurysm disease

lo_ci	up_ci	or	or_lci95	or_uci95
0.54198133069	1.29737693122	2.50848536555	1.71941(	3.65968446261326
0.44895573369	1.53313233347	2.69404568055	1.56667	4.63266517761081
1.06542693781	4.15894657922	13.6288212301	2.90207	64.0040638642688
-0.090647588	3.15560051931	4.62962775201	0.91333	23.4671253229135
-0.331440818	3.26477793777	4.33477003331	0.71788	26.1742980210389
0.51875195908	1.31766035255	2.50479314871	1.67992	3.73467332847759
0.47382388445	1.84514710271	3.18829245711	1.60612	6.32903073963837
0.23042202759	2.08594391	3.18414232722	1.25913	8.05218841983752
2.58109198056	8.33958402861	235.176901811	13.2115	4186.34797844566
-1.415923046	3.36640190722	2.65180205761	0.24270	28.9740878243552
-1.419896922	3.44469382444	2.75219410844	0.24173	31.3336883676286
0.44403018939	1.83046073761	3.11816742	1.55897	6.23675950647644
0.04127899648	0.55294438371	1.34596562211	1.04214	1.7383639003799
0.10054514645	0.81901180571	1.58372311361	1.10577	2.26825725095624
-0.508522074	1.75485725551	1.86482570121	0.60138	5.78262224577262
-0.150295299	2.25421458911	2.86325659111	0.86045	9.52780712790834
-0.264573507	1.81625252761	2.17241512631	0.76753	6.14877286438367
0.05942455530	0.60530373711	1.39426047071	1.06122	1.83180851268519
0.19086210774	0.71464047011	1.57263300661	1.21029	2.04345187
0.07368104470	0.85916083761	1.59427795531	1.07646	2.36117844996244
0.16563522150	1.13340707811	1.91462379151	1.18014	3.10622163167521
-0.610938553	0.98315676471	1.20455367681	0.54284	2.67288059342866
0.07600360997	0.87620696001	1.60979249401	1.07896	2.40177238867173
0.16493393645	0.70968336151	1.54853395671	1.17931	2.03334732084275
0.56911414543	1.62169633871	2.99039427151	1.76670	5.06166934408814
0.17378315822	1.68446608111	2.53229148891	1.18979	5.38957256938748
0.18201993678	4.57076325191	10.7659846501	1.19963	96.6178253843736
-1.178184277	3.09315422911	2.60513624711	0.30783	22.046508156846
-1.240450467	2.88986152681	2.28120891811	0.28925	17.9908181832401
0.60152237059	1.67959528161	3.12851617321	1.82489	5.36338487130206
1.10406858345	3.06820658091	8.05374807301	3.01641	21.5033036392538
0.96866773308	3.76140545681	10.6444283421	2.63443	43.0088305763561
-2.630598931	6.25546762561	6.12534050441	0.07203	520.852881994339
-1.475146202	6.16884124691	10.4525662481	0.22874	477.632327370593
-1.010649036	5.93421965161	11.7257268981	0.36398	377.745108338099
1.25585602351	3.34173534691	9.96217762991	3.51084	28.2681391960744

0.0572281659	0.726760092	1.479929022	1.05889	2.06836841720695
-0.171868505	0.809648480	1.375599983	0.84208	2.24711794134751
-0.753077854	2.208522176	2.07035929	0.47091	9.10225491831248
-1.066008256	2.149344198	1.718871499	0.34438	8.57923027594136
-1.258909026	1.728708289	1.264781818	0.28396	5.63337251797512
0.0219948261	0.706619585	1.439516374	1.02223	2.02712713337922
0.2414143680	1.017577056	1.876663962	1.27304	2.76648360357759
0.2273377260	1.354043114	2.204918221	1.25525	3.87305311639613
0.2073848802	1.647542126	2.528088549	1.23045	5.19419744278148
-0.080612441	2.189984879	2.871074124	0.92255	8.93507800972797
0.3272687300	1.380841043	2.349153115	1.38717	3.97824609916257
0.3149351079	1.083597076	2.012275341	1.37017	2.95529086299093
0.8376389463	1.977190074	4.085379032	2.31090	7.22241998280717
0.7962167304	2.33879508	4.794674900	2.21713	10.3687355583671
1.7748514227	6.563086201	64.64875270	5.89940	708.454758040648
-0.093351115	4.167408914	7.667793540	0.91087	64.547985923387
-0.272583137	3.697977946	5.543895455	0.76141	40.365600380666
0.7730745008	1.951960043	3.906013434	2.16641	7.04247762111428

Supplementary Table S6： Causal association between lifetime smoking index an

method	nsnp	b	se	pval	lo_ci	up_ci
Inverse	112	0.7227372647	0.07334	6.55050	0.5789900550	0.8664844744
Inverse	112	0.6722527645	0.07430	1.46242	0.5266201129	0.8178854162
Inverse	112	0.4449865528	0.06851	8.32528	0.3106944809	0.5792786246
Inverse	112	0.5490173747	0.08931	7.88468	0.3739671922	0.7240675572
Inverse	112	0.5847925778	0.09635	1.28424	0.3959433004	0.7736418553
Inverse	112	-0.442914362	0.07408	2.25244	-0.588120789	-0.297707935
Inverse	112	0.5296073161	0.08862	2.29126	0.3558982850	0.7033163473
Inverse	112	0.4764589045	0.08146	4.96080	0.3167833610	0.6361344480
Inverse	112	0.4679613637	0.08461	3.19907	0.3021069592	0.6338157683
Inverse	112	0.4001666523	0.07280	3.87336	0.2574717863	0.5428615183
Inverse	112	0.4702420426	0.08646	5.36671	0.3007764729	0.6397076122
Inverse	112	0.3734810973	0.07335	3.55104	0.2297096278	0.5172525668
Inverse	112	0.3790480556	0.07546	5.09891	0.2311284243	0.5269676870
Inverse	112	0.3684960213	0.07410	6.60637	0.2232493811	0.5137426615
Inverse	112	0.3896634034	0.07929	8.92494	0.2342412194	0.5450855874
Inverse	112	0.2852372010	0.05904	1.36234	0.1694992232	0.4009751788
Inverse	112	0.4229976607	0.09041	2.88787	0.2457931978	0.6002021236
Inverse	112	0.3325316563	0.07201	3.88147	0.1913852198	0.4736780927
Inverse	112	0.4424400230	0.09915	8.11968	0.2480918057	0.6367882403
Inverse	112	0.3420711627	0.07707	9.06240	0.1910128266	0.4931294988
Inverse	112	-0.42876376	0.09885	1.44211	-0.622517384	-0.235010137
Inverse	112	0.3549945079	0.08354	2.14535	0.1912485505	0.5187404653
Inverse	112	0.3750454814	0.08896	2.49116	0.2006718807	0.5494190821
Inverse	112	-0.323764864	0.07718	2.73535	-0.475055079	-0.17247465
Inverse	112	0.3368984377	0.08073	3.00965	0.1786518638	0.4951450116
Inverse	112	0.3357678441	0.08124	3.58344	0.1765289963	0.4950066918
Inverse	112	0.3090493565	0.07546	4.21248	0.1611465517	0.4569521613
Inverse	112	0.3329287698	0.08230	5.23200	0.1716090747	0.4942484649
Inverse	112	-0.319559214	0.07966	6.03371	-0.475694788	-0.163423641
Inverse	112	0.3319429281	0.08294	6.28028	0.1693731722	0.4945126840
Inverse	112	0.1824999115	0.04565	6.41543	0.0930075047	0.2719923184
Inverse	112	0.2786187603	0.07056	7.87181	0.1403068317	0.4169306889
Inverse	112	0.3282505544	0.08397	9.26616	0.1636653247	0.4928357841
Inverse	112	0.2922095823	0.07510	0.00010	0.1449941592	0.4394250054
Inverse	112	0.3331073231	0.08665	0.00012	0.1632697118	0.5029449343
Inverse	112	0.2857844910	0.07422	0.00011	0.1403108565	0.4312581256

Inverse v	112	0.31241380472	0.08272(	0.00015(	0.15028096914	0.47454664029
Inverse v	112	0.21389639856	0.05671	0.00016	0.10273621162	0.32505658550
Inverse v	112	0.27595795501	0.07300(	0.00015(	0.13286066117	0.41905524885
Inverse v	112	0.30583818105	0.08178(	0.00018	0.14554754708	0.46612881503
Inverse v	112	0.29436274443	0.07924	0.00020	0.13904278246	0.44968270640
Inverse v	112	0.30859585610	0.08335	0.00021	0.14521146081	0.47198025138
Inverse v	112	0.33555442875	0.09080	0.00021	0.15757252845	0.51353632905
Inverse v	112	0.27753398264	0.07526(	0.00022(	0.13002382144	0.42504414384
Inverse v	112	0.33272120130	0.09083	0.00024	0.15467689226	0.51076551046
Inverse v	112	0.26012187139	0.07110	0.00025	0.12074954592	0.39949419686
Inverse v	112	0.37868124795	0.10575	0.00034	0.17139233672	0.58597015919
Inverse v	112	-0.282230122	0.07923	0.00036	-0.43753034	-0.126929905
Inverse v	112	0.25734320343	0.07241	0.00037	0.11541507855	0.39927132830
Inverse v	112	0.20813546009	0.05923	0.00044	0.09203026694	0.32424065324
Inverse v	112	0.26369376793	0.07589	0.00051	0.11494659080	0.41244094507
Inverse v	112	0.31344924380	0.09037	0.00052	0.13630507026	0.49059341734
Inverse v	112	0.29693979059	0.08635	0.00058	0.12768240271	0.46619717847
Inverse v	112	0.26766570324	0.07832	0.00063	0.11415398439	0.42117742209
Inverse v	112	0.30400695586	0.08942(	0.00067	0.12873117072	0.47928274100
Inverse v	112	0.28229486476	0.08371(	0.00074(	0.11820980918	0.44637992034
Inverse v	112	0.30684011760	0.09096	0.00074	0.12855118518	0.48512905002
Inverse v	112	-0.273121665	0.08077	0.00072	-0.431438648	-0.114804682
Inverse v	112	0.26284033085	0.07834	0.00079	0.10927451012	0.41640615159
Inverse v	112	-0.243470786	0.07275(	0.00081	-0.386061133	-0.100880439
Inverse v	112	0.26377595602	0.07867	0.00080(	0.10957370468	0.41797820736
Inverse v	112	0.29895033318	0.08938	0.00082	0.12375426781	0.47414639856
Inverse v	112	-0.238134512	0.07165	0.00088	-0.378583774	-0.09768525
Inverse v	112	0.22138264190	0.06694	0.00094	0.09016892001	0.35259636379
Inverse v	112	0.25594583874	0.07833(	0.00108	0.10241725580	0.40947442169
Inverse v	112	-0.277321654	0.08505	0.00111	-0.444027957	-0.110615352
Inverse v	112	0.27022349670	0.08350(	0.00121	0.10656181768	0.43388517583
Inverse v	112	-0.253083172	0.07883	0.00132	-0.407592057	-0.098574288
Inverse v	112	0.25292901083	0.07877(	0.00132	0.09852660406	0.40733141760
Inverse v	112	0.25886274128	0.08102	0.00139	0.10004980445	0.41767567812
Inverse v	112	0.28234806558	0.08864	0.00144	0.10859761326	0.45609851789
Inverse v	112	0.22465715087	0.07056	0.00145	0.08635441810	0.36295988364
Inverse v	112	0.26545171978	0.08348	0.00147	0.10182267798	0.42908076158
Inverse v	112	0.23790248420	0.07544	0.00161	0.09002262775	0.38578234064

Inverse v	112	-0.238281856	0.07578	0.00166	-0.386817922	-0.089745789
Inverse v	112	-0.311789057	0.09946	0.00172	-0.506748956	-0.116829158
Inverse v	112	0.2564083259	0.08174	0.00170	0.0961967782	0.4166198736
Inverse v	112	0.2158326891	0.06903	0.00176	0.0805222065	0.3511431717
Inverse v	112	-0.247805765	0.08023	0.00201	-0.405069929	-0.090541601
Inverse v	112	0.2934332867	0.09510	0.00203	0.1070231613	0.4798434121
Inverse v	112	0.1994755895	0.06444	0.00196	0.0731704369	0.3257807422
Inverse v	112	0.2418977449	0.07844	0.00204	0.0881464309	0.3956490590
Inverse v	112	0.2497243160	0.08079	0.00199	0.0913617434	0.4080868886
Inverse v	112	0.2376881522	0.07743	0.00214	0.0859174404	0.3894588640
Inverse v	112	0.2312076958	0.07560	0.00222	0.0830290903	0.3793863012
Inverse v	112	-0.258372469	0.08453	0.00224	-0.424064551	-0.092680387
Inverse v	112	-0.246594713	0.08070	0.00224	-0.404780408	-0.088409017
Inverse v	112	0.2072016091	0.06806	0.00233	0.0737961131	0.3406071052
Inverse v	112	0.2490382118	0.08190	0.00236	0.0884992498	0.4095771738
Inverse v	112	0.2023245516	0.06661	0.00238	0.0717513646	0.3328977387
Inverse v	112	-0.230711979	0.07622	0.00247	-0.380105533	-0.081318426
Inverse v	112	0.2571745665	0.08522	0.00254	0.0901343164	0.4242148166
Inverse v	112	0.2307625495	0.07699	0.00272	0.0798495298	0.3816755692
Inverse v	112	-0.230203185	0.07691	0.00276	-0.380965175	-0.079441194
Inverse v	112	0.2699847396	0.09023	0.00277	0.0931322111	0.4468372681
Inverse v	112	-0.227865061	0.07680	0.00300	-0.378396008	-0.077334114
Inverse v	112	0.2324897682	0.07891	0.00321	0.0778123612	0.3871671752
Inverse v	112	0.2108616173	0.07189	0.00335	0.0699552243	0.3517680103
Inverse v	112	0.2515893715	0.08569	0.00332	0.0836217888	0.4195569541
Inverse v	112	-0.247166125	0.08504	0.00365	-0.413853934	-0.080478317
Inverse v	112	0.2168754546	0.07577	0.00420	0.0683635320	0.3653873773
Inverse v	112	0.2363510623	0.08250	0.00417	0.0746478893	0.3980542354
Inverse v	112	0.2126996338	0.07485	0.00448	0.0659861753	0.3594130924
Inverse v	112	0.2485935561	0.08746	0.00448	0.0771559320	0.4200311802
Inverse v	112	0.2057701507	0.07254	0.00455	0.0635907550	0.3479495465
Inverse v	112	0.2359724138	0.08382	0.00487	0.0716670742	0.4002777535
Inverse v	112	0.2516722741	0.08994	0.00514	0.0753831581	0.4279613901
Inverse v	112	0.2358242033	0.08434	0.00517	0.0705068347	0.4011415719
Inverse v	112	0.2320114913	0.08517	0.00644	0.0650751294	0.3989478533
Inverse v	112	0.2038831345	0.07503	0.00658	0.0568064796	0.3509597895
Inverse v	112	-0.194497004	0.07226	0.00711	-0.336129337	-0.052864671
Inverse v	112	0.2104278036	0.07879	0.00756	0.0559979111	0.3648576961

Inverse v	112	-0.235023924	0.08791	0.00750	-0.407330465	-0.062717384
Inverse v	112	0.1976162516	0.07456	0.00803	0.0514765853	0.3437559178
Inverse v	112	-0.225002291	0.08558	0.00856	-0.392755918	-0.057248665
Inverse v	112	0.2075549012	0.07922	0.00879	0.0522767921	0.3628330103
Inverse v	112	0.2161035343	0.08249	0.00880	0.0544051728	0.3778018957
Inverse v	112	-0.208521089	0.07970	0.00889	-0.364740351	-0.052301828
Inverse v	112	0.2405682684	0.09217	0.00905	0.0599045804	0.4212319565
Inverse v	112	-0.201074728	0.07714	0.00915	-0.352286029	-0.049863426
Inverse v	112	0.2049546809	0.07908	0.00954	0.0499575630	0.3599517988
Inverse v	112	0.2027911228	0.07854	0.00983	0.0488343645	0.3567478811
Inverse v	112	0.2019006923	0.07837	0.00999	0.0482834275	0.3555179571
Inverse v	112	0.2098574224	0.08196	0.01045	0.0492010703	0.3705137745
Inverse v	112	0.2152434107	0.08429	0.01066	0.0500258858	0.3804609357
Inverse v	112	0.2044153176	0.08007	0.01069	0.0474597118	0.3613709233
Inverse v	112	-0.233297269	0.09106	0.01041	-0.411784237	-0.0548103
Inverse v	112	0.2142797630	0.08388	0.01063	0.0498601916	0.3786993344
Inverse v	112	-0.19922946	0.07787	0.01051	-0.35185729	-0.046601631
Inverse v	112	-0.200706287	0.07953	0.01162	-0.356602679	-0.044809895
Inverse v	112	0.2134878624	0.08453	0.01155	0.0477977163	0.3791780085
Inverse v	112	-0.199060201	0.07916	0.01191	-0.354218897	-0.043901505
Inverse v	112	0.2021498891	0.08084	0.01240	0.0436927484	0.3606070299
Inverse v	112	0.2015990813	0.08056	0.01233	0.0436927812	0.3595053814
Inverse v	112	-0.199120826	0.07955	0.01231	-0.355042655	-0.043198996
Inverse v	112	0.1961320350	0.07874	0.01275	0.0417871611	0.3504769090
Inverse v	112	0.2072351813	0.08339	0.01296	0.0437725217	0.3706978408
Inverse v	112	-0.211470085	0.08514	0.01300	-0.378353714	-0.044586456
Inverse v	112	0.2171421677	0.08763	0.01322	0.0453721214	0.3889122139
Inverse v	112	0.1786315472	0.07216	0.01331	0.0371889865	0.3200741079
Inverse v	112	0.1987413569	0.08033	0.01336	0.0412885430	0.3561941709
Inverse v	112	0.1879656125	0.07656	0.01408	0.0378977435	0.3380334815
Inverse v	112	-0.197580366	0.08061	0.01425	-0.355589557	-0.039571176
Inverse v	112	0.1937994890	0.07921	0.01442	0.0385341837	0.3490647944
Inverse v	112	0.2059188675	0.08453	0.01485	0.0402300785	0.3716076565
Inverse v	112	-0.206681373	0.08489	0.01491	-0.373082738	-0.040280009
Inverse v	112	0.1904548642	0.07863	0.01543	0.0363282910	0.3445814375
Inverse v	112	0.1933580737	0.08078	0.01669	0.0350113365	0.3517048109
Inverse v	112	0.2008426414	0.08440	0.01734	0.0354002252	0.3662850577
Inverse v	112	0.1879035357	0.07897	0.01734	0.0331115629	0.3426955085

Inverse v	112	0.18875196591	0.079418	0.017469	0.03309232356	0.34441160825
Inverse v	112	-0.210469161	0.089459	0.018639	-0.385810472	-0.03512785
Inverse v	112	0.18424901732	0.078849	0.019453	0.02970432258	0.33879371207
Inverse v	112	-0.184318237	0.079101	0.019798	-0.33935804	-0.029278435
Inverse v	112	-0.184206707	0.079220	0.020058	-0.339478625	-0.02893479
Inverse v	112	-0.185237815	0.079829	0.020319	-0.341704599	-0.028771031
Inverse v	112	0.17811588433	0.076743	0.020291	0.02769831481	0.32853345384
Inverse v	112	0.18903526565	0.082008	0.021162	0.02829848466	0.34977204664
Inverse v	112	-0.244116611	0.106431	0.021810	-0.452721459	-0.035511762
Inverse v	112	0.16956805534	0.074020	0.021983	0.02447675415	0.31465935653
Inverse v	112	0.21921480635	0.096075	0.022507	0.03090696615	0.40752264655
Inverse v	112	0.18047429948	0.079048	0.022425	0.02553902749	0.33540957147
Inverse v	112	0.17670034167	0.077743	0.023034	0.02432274758	0.32907793577
Inverse v	112	0.17695706144	0.077763	0.022870	0.02454108660	0.32937303621
Inverse v	112	0.19081696898	0.083975	0.023060	0.02622481670	0.35540912120
Inverse v	112	0.14862741403	0.065330	0.022904	0.02058041094	0.27667441712
Inverse v	112	0.18555198474	0.081924	0.023517	0.02498090160	0.34612306788
Inverse v	112	0.17785865151	0.078940	0.024265	0.02312365531	0.33259364770
Inverse v	112	0.14269777274	0.063973	0.025710	0.01730902044	0.26808652504
Inverse v	112	0.18068277272	0.081164	0.026004	0.02160121612	0.33976432933
Inverse v	112	-0.176758562	0.079805	0.026769	-0.333177272	-0.020339851
Inverse v	112	0.17664746407	0.079685	0.026630	0.02046316863	0.33283175951
Inverse v	112	0.14269196749	0.065043	0.028249	0.01520703759	0.27017689740
Inverse v	112	0.16472916758	0.075290	0.028675	0.01715970784	0.31229862733
Inverse v	112	0.15752587204	0.072148	0.029008	0.01611568884	0.29893605524
Inverse v	112	0.18875409456	0.086903	0.029855	0.01842342753	0.35908476159
Inverse v	112	0.17694921170	0.081548	0.030017	0.01711384762	0.33678457591
Inverse v	112	0.19880390411	0.091678	0.030121	0.01911390884	0.37849389937
Inverse v	112	0.17582158270	0.081338	0.030648	0.01639824014	0.33524492526
Inverse v	112	-0.164411966	0.076005	0.030528	-0.313382393	-0.015441538
Inverse v	112	0.16762084510	0.077403	0.030345	0.01591015160	0.31933153860
Inverse v	112	0.17542865760	0.081524	0.031408	0.01564105219	0.33521626300
Inverse v	112	0.17763624213	0.082502	0.031310	0.01593136955	0.33934111470
Inverse v	112	0.15916982904	0.074341	0.032270	0.01345951962	0.30488013846
Inverse v	112	0.17302058792	0.080891	0.032442	0.01447252755	0.33156864829
Inverse v	112	0.17309541294	0.081359	0.033370	0.01362986522	0.33256096066
Inverse v	112	0.16364828240	0.076869	0.033260	0.01298484660	0.31431171820
Inverse v	112	0.17976156656	0.084673	0.033754	0.01380091783	0.34572221530

Inverse v	112	0.1643666023	0.07757	0.03411	0.0123156444	0.3164175603
Inverse v	112	0.1712030983	0.08097	0.03448	0.0124985808	0.3299076159
Inverse v	112	0.1331483247	0.06308	0.03479	0.0095087067	0.2567879427
Inverse v	112	0.1566558918	0.07426	0.03491	0.0110890174	0.3022227661
Inverse v	112	0.1582301534	0.07524	0.03547	0.0107493734	0.3057109334
Inverse v	112	-0.167795107	0.07973	0.03533	-0.324072448	-0.011517766
Inverse v	112	0.1830039113	0.08731	0.03608	0.0118762435	0.3541315791
Inverse v	112	0.1535025635	0.07324	0.03610	0.0099439497	0.2970611772
Inverse v	112	-0.164934895	0.07878	0.03630	-0.319354551	-0.01051524
Inverse v	112	-0.163640992	0.07829	0.03662	-0.317106109	-0.010175874
Inverse v	112	-0.173136452	0.08327	0.03761	-0.336359685	-0.009913218
Inverse v	112	0.1610043708	0.07743	0.03760	0.0092289925	0.3127797490
Inverse v	112	0.2899782661	0.13979	0.03804	0.0159856907	0.5639708416
Inverse v	112	-0.167222917	0.08064	0.03812	-0.325288375	-0.009157459
Inverse v	112	-0.164916451	0.07963	0.03836	-0.320995178	-0.008837724
Inverse v	112	-0.185435655	0.08996	0.03928	-0.361771081	-0.009100228
Inverse v	112	-0.174127847	0.08474	0.03989	-0.340219259	-0.008036436
Inverse v	112	-0.164158555	0.07971	0.03947	-0.320405416	-0.007911693
Inverse v	112	0.1735441287	0.08449	0.03998	0.0079370559	0.3391512014
Inverse v	112	0.1627400483	0.07916	0.03980	0.0075825636	0.3178975330
Inverse v	112	0.1598908084	0.07801	0.04040	0.0069881881	0.3127934286
Inverse v	112	0.1579128249	0.07715	0.04070	0.0066795301	0.3091461198
Inverse v	112	-0.174583708	0.08526	0.04061	-0.341708122	-0.007459294
Inverse v	112	-0.161947068	0.07937	0.04133	-0.317530071	-0.006364066
Inverse v	112	0.1625251403	0.07963	0.04125	0.0064468933	0.3186033872
Inverse v	112	-0.163631713	0.08046	0.04199	-0.321344743	-0.005918682
Inverse v	112	0.1757376375	0.08682	0.04296	0.0055588814	0.3459163936
Inverse v	112	-0.177976936	0.08813	0.04345	-0.350726493	-0.005227378
Inverse v	112	0.1762015637	0.08724	0.04342	0.0051984316	0.3472046959
Inverse v	112	0.1610955722	0.07977	0.04345	0.0047287647	0.3174623796
Inverse v	112	0.1772883138	0.08851	0.04519	0.0037922320	0.3507843957
Inverse v	112	0.1668955628	0.08358	0.04584	0.0030737324	0.3307173932
Inverse v	112	-0.170539188	0.08573	0.04668	-0.338576295	-0.002502082
Inverse v	112	-0.175604395	0.08862	0.04753	-0.349304331	-0.001904458
Inverse v	112	0.1460336012	0.07378	0.04780	0.0014105076	0.2906566948
Inverse v	112	0.1375394890	0.06979	0.04876	0.0007407413	0.2743382367
Inverse v	112	-0.169878671	0.08627	0.04894	-0.338972134	-0.000785207
Inverse v	112	-0.155486663	0.07895	0.04891	-0.310236821	-0.000736506

Inverse v	112	0.1409689712(	0.07159	0.04896	0.00063882359	0.2812991188
Inverse v	112	-0.158445863	0.08065	0.04947	-0.316528853	-0.000362872
Inverse v	112	-0.126040601	0.06414	0.04943(	-0.251769276	-0.000311926
Inverse v	112	-0.155438879	0.07948(	0.05051	-0.311232244	0.0003544862
Inverse v	112	-0.168795789	0.08653	0.05109	-0.338396877	0.0008052997
Inverse v	112	0.1760231897	0.09053(	0.05186	-0.001428886	0.3534752654
Inverse v	112	-0.163053038	0.08382(	0.05174	-0.327340867	0.0012347917
Inverse v	112	-0.147937777	0.07676	0.05396	-0.29840151	0.0025259558
Inverse v	112	-0.188680192	0.09823(	0.05475	-0.381212399	0.0038520146
Inverse v	112	-0.159846047	0.08370	0.05618	-0.323914388	0.0042222947
Inverse v	112	-0.150755561	0.07904	0.05650	-0.305693027	0.0041819055
Inverse v	112	0.2051431802(	0.10857	0.05882	-0.007656937	0.4179432978
Inverse v	112	-0.128613049	0.06800	0.05860	-0.261911316	0.0046852183
Inverse v	112	-0.154593848	0.08204(	0.05953	-0.315404992	0.0062172958
Inverse v	112	-0.155993156	0.08294	0.06002	-0.318574071	0.0065877595
Inverse v	112	0.1482012452(	0.07891	0.06037	-0.006466722	0.3028692121
Inverse v	112	0.1511804006	0.08102	0.06207	-0.007635873	0.3099966744
Inverse v	112	0.1520696354	0.08147	0.06197	-0.007619455	0.3117587261
Inverse v	112	-0.165317998	0.08843(	0.06157	-0.338653722	0.0080177258
Inverse v	112	0.1612917419(	0.08713	0.06415	-0.009491771	0.3320752549
Inverse v	112	0.1618223058(	0.08765	0.06487(	-0.009983446	0.3336280572
Inverse v	112	0.1455472238	0.07895	0.06525	-0.009198711	0.3002931587
Inverse v	112	-0.145106837	0.07941	0.06768(	-0.300766304	0.0105526304
Inverse v	112	0.1409058100(	0.07705	0.06744	-0.010115179	0.2919267987
Inverse v	112	0.1444497801	0.07937	0.06877	-0.011118872	0.3000184318
Inverse v	112	-0.161548523	0.08916(	0.07000	-0.336302743	0.0132056966
Inverse v	112	-0.154027914	0.08506(	0.07017	-0.320746604	0.0126907750
Inverse v	112	0.1541232848	0.08522	0.07054	-0.012923617	0.3211701861
Inverse v	112	0.1485637913	0.08236	0.07126(	-0.012867304	0.3099948868
Inverse v	112	0.1380657209	0.07666	0.07172	-0.012198724	0.2883301656
Inverse v	112	0.1430053899(	0.07962	0.07249	-0.013056709	0.2990674885
Inverse v	112	-0.14630907	0.08148	0.07257	-0.306024456	0.0134063162
Inverse v	112	0.1482735892	0.08242	0.07205	-0.013288857	0.3098360352
Inverse v	112	-0.141775575	0.07928	0.07373	-0.297166874	0.0136157235
Inverse v	112	-0.146105218	0.08219	0.07547	-0.307207502	0.0149970647
Inverse v	112	0.1353339446	0.07630	0.07613	-0.014224835	0.2848927247
Inverse v	112	0.1414086910	0.07960	0.07567	-0.014621611	0.2974389934
Inverse v	112	0.1590565729	0.08971	0.07623	-0.016779165	0.3348923110

Inverse v	112	0.1423182418	0.08000	0.07524	-0.014485308	0.2991217918
Inverse v	112	0.1483062380	0.08375	0.07659	-0.015845653	0.3124581294
Inverse v	112	-0.1518827	0.08588	0.07697	-0.320209089	0.0164436885
Inverse v	112	0.1438734772	0.08161	0.07791	-0.016082528	0.3038294825
Inverse v	112	-0.139815449	0.07971	0.07945	-0.296065511	0.0164346136
Inverse v	112	-0.150169253	0.08585	0.08027	-0.318444447	0.0181059418
Inverse v	112	0.1363116950	0.07835	0.08192	-0.01726633	0.2898897205
Inverse v	112	0.1490116262	0.08541	0.08106	-0.018403226	0.3164264783
Inverse v	112	-0.134860515	0.07768	0.08257	-0.287128189	0.0174071578
Inverse v	112	-0.137880712	0.07915	0.08151	-0.293019386	0.0172579608
Inverse v	112	-0.129715234	0.07428	0.08078	-0.275316855	0.0158863870
Inverse v	112	-0.130287669	0.07507	0.08266	-0.277433555	0.0168582158
Inverse v	112	0.1452864711	0.08376	0.08282	-0.018883498	0.3094564403
Inverse v	112	-0.137733601	0.07912	0.08174	-0.292823657	0.0173564556
Inverse v	112	-0.136305776	0.07863	0.08301	-0.290426788	0.0178152367
Inverse v	112	-0.144099956	0.08337	0.08391	-0.307510061	0.0193101486
Inverse v	112	-0.137076605	0.07945	0.08450	-0.29281736	0.0186641491
Inverse v	112	0.1316626114	0.07675	0.08628	-0.018782428	0.2821076512
Inverse v	112	-0.175778573	0.10257	0.08658	-0.376821911	0.0252647647
Inverse v	112	0.1426333546	0.08372	0.08844	-0.021459881	0.3067265903
Inverse v	112	0.1668381762	0.09798	0.08862	-0.025210055	0.3588864072
Inverse v	112	-0.134479494	0.07917	0.08939	-0.289652736	0.0206937487
Inverse v	112	-0.145869953	0.08596	0.08970	-0.314353037	0.0226131317
Inverse v	112	0.1367346530	0.08068	0.09012	-0.021399543	0.2948688493
Inverse v	112	0.1408675808	0.08330	0.09084	-0.022412162	0.3041473239
Inverse v	112	-0.137170042	0.08137	0.09185	-0.296660949	0.0223208654
Inverse v	112	-0.133210281	0.07969	0.09460	-0.289404484	0.0229839222
Inverse v	112	0.1330045835	0.07984	0.09503	-0.023201233	0.2898103999
Inverse v	112	-0.13214851	0.07973	0.09746	-0.288437101	0.0241400800
Inverse v	112	0.1323577541	0.07987	0.09749	-0.024191089	0.2889065975
Inverse v	112	-0.133621446	0.08055	0.09718	-0.29151701	0.0242741185
Inverse v	112	-0.135750783	0.08206	0.09810	-0.296603229	0.0251016643
Inverse v	112	-0.128910707	0.07869	0.10140	-0.283156582	0.0253351689
Inverse v	112	0.1302447647	0.07954	0.10156	-0.025669156	0.2861586849
Inverse v	112	0.1368560648	0.08396	0.10313	-0.027725033	0.3014371624
Inverse v	112	0.1293525770	0.07976	0.10486	-0.026980851	0.2856860056
Inverse v	112	0.1349115983	0.08327	0.10522	-0.028311188	0.2981343847
Inverse v	112	0.1284572671	0.07957	0.10644	-0.027500544	0.2844150785

Inverse v	112	0.1259974376	0.07841	0.10810	-0.027698234	0.2796931093
Inverse v	112	0.1369187425	0.08574	0.11031	-0.03114438	0.3049818649
Inverse v	112	0.1397910618	0.08794	0.11195	-0.032588367	0.3121704907
Inverse v	112	-0.112485211	0.07164	0.11638	-0.252900282	0.0279298601
Inverse v	112	0.1222627249	0.07791	0.11662	-0.030458348	0.2749837977
Inverse v	112	0.1315757156	0.08414	0.11790	-0.033352468	0.2965038994
Inverse v	112	0.1195372184	0.07647	0.11800	-0.030343992	0.2694184293
Inverse v	112	0.1102287879	0.07048	0.11784	-0.027919788	0.2483773641
Inverse v	112	0.1456650657	0.09383	0.12059	-0.038260086	0.3295902176
Inverse v	112	0.1192631422	0.07669	0.11992	-0.031055222	0.2695815062
Inverse v	112	-0.123717536	0.07971	0.12065	-0.279957338	0.0325222668
Inverse v	112	0.1211384704	0.07867	0.12362	-0.033062555	0.2753394957
Inverse v	112	0.1216617902	0.07922	0.12463	-0.033625205	0.2769487854
Inverse v	112	0.1207347300	0.07884	0.12571	-0.033809674	0.2752791338
Inverse v	112	0.1167081732	0.07653	0.12729	-0.033303919	0.2667202653
Inverse v	112	0.1205279740	0.07900	0.12712	-0.034324842	0.2753807904
Inverse v	112	-0.121083144	0.07947	0.12763	-0.276856823	0.0346905345
Inverse v	112	0.1241784271	0.08169	0.12851	-0.035948053	0.2843049076
Inverse v	112	0.1223982358	0.08095	0.13054	-0.036270366	0.2810668376
Inverse v	112	-0.118792583	0.07877	0.13153	-0.273183053	0.0355978883
Inverse v	112	0.1133965039	0.07599	0.13565	-0.035552836	0.2623458442
Inverse v	112	0.1181953425	0.07992	0.13918	-0.038456776	0.2748474607
Inverse v	112	-0.130717736	0.08860	0.14014	-0.304387355	0.0429518838
Inverse v	112	-0.095658738	0.06485	0.14019	-0.222766752	0.0314492769
Inverse v	112	-0.133369846	0.09095	0.14255	-0.311640135	0.0449004443
Inverse v	112	0.1220950676	0.08326	0.14256	-0.04110748	0.2852976158
Inverse v	112	0.1199601188	0.08208	0.14389	-0.040922355	0.2808425926
Inverse v	112	-0.120741039	0.08259	0.14377	-0.282624037	0.0411419592
Inverse v	112	0.1211394800	0.08325	0.14563	-0.042031614	0.2843105742
Inverse v	112	0.1166762714	0.08017	0.14561	-0.040475412	0.2738279546
Inverse v	112	-0.114135445	0.07849	0.14594	-0.267991118	0.0397202277
Inverse v	112	0.1222699506	0.08437	0.14729	-0.043100032	0.2876399336
Inverse v	112	-0.11482271	0.07920	0.14716	-0.270070906	0.0404254854
Inverse v	112	0.1280948293	0.08870	0.14871	-0.045764261	0.3019539193
Inverse v	112	-0.111672557	0.07745	0.14937	-0.263488419	0.0401433050
Inverse v	112	0.1225559419	0.08497	0.14920	-0.043986561	0.2890984450
Inverse v	112	-0.087674498	0.06072	0.14877	-0.206688948	0.0313399520
Inverse v	112	-0.114741805	0.07983	0.15063	-0.271212083	0.0417284722

Inverse v	112	-0.089886305	0.06283	0.15255	-0.213039605	0.0332669949
Inverse v	112	-0.109929042	0.07687	0.15270	-0.260595489	0.0407374041
Inverse v	112	-0.113626104	0.07955	0.15323	-0.269561753	0.0423095444
Inverse v	112	-0.098515196	0.06895	0.15310	-0.233670392	0.0366400000
Inverse v	112	-0.126540876	0.08930	0.15648	-0.30157356	0.0484918083
Inverse v	112	-0.123500797	0.08726	0.15701	-0.294546258	0.0475446637
Inverse v	112	0.1019438060	0.07241	0.15921	-0.039995618	0.2438832297
Inverse v	112	0.1176956353	0.08379	0.16014	-0.046541609	0.2819328799
Inverse v	112	-0.120339407	0.08564	0.15997	-0.288196461	0.0475176463
Inverse v	112	-0.114357574	0.08185	0.16238	-0.274791361	0.0460762129
Inverse v	112	0.1093282024	0.07856	0.16406	-0.044664183	0.2633205881
Inverse v	112	0.1104847987	0.07974	0.16589	-0.045809281	0.2667788781
Inverse v	112	-0.109729229	0.07918	0.16583	-0.264935871	0.0454774134
Inverse v	112	-0.114187222	0.08231	0.16537	-0.275520976	0.0471465332
Inverse v	112	-0.11000201	0.07962	0.16711	-0.266061927	0.0460579071
Inverse v	112	0.1080702016	0.07899	0.17130	-0.046765369	0.2629057717
Inverse v	112	-0.116483565	0.08524	0.17179	-0.283562456	0.0505953257
Inverse v	112	-0.108958614	0.07960	0.17108	-0.264986342	0.0470691149
Inverse v	112	0.1155026038	0.08449	0.17163	-0.050107228	0.2811124358
Inverse v	112	0.1059916011	0.07743	0.17104	-0.04577176	0.2577549621
Inverse v	112	0.1131990453	0.08255	0.17033	-0.048615509	0.2750135999
Inverse v	112	0.1085982139	0.07945	0.17168	-0.047129614	0.2643260418
Inverse v	112	-0.108820411	0.07905	0.16864	-0.263762303	0.0461214804
Inverse v	112	-0.113779842	0.08338	0.17240	-0.277213773	0.0496540895
Inverse v	112	0.1122030954	0.08230	0.17279	-0.049112616	0.2735188074
Inverse v	112	-0.121243618	0.08906	0.17341	-0.295807365	0.0533201284
Inverse v	112	0.1167353855	0.08613	0.17534	-0.05209203	0.2855628010
Inverse v	112	0.1033918663	0.07635	0.17573	-0.046273725	0.2530574575
Inverse v	112	0.1131888420	0.08370	0.17631	-0.050879761	0.2772574448
Inverse v	112	0.0890195773	0.06600	0.17745	-0.04035462	0.2183937747
Inverse v	112	0.1070223554	0.07967	0.17918	-0.049136725	0.2631814355
Inverse v	112	0.0985425187	0.07383	0.18202	-0.046182532	0.2432675696
Inverse v	112	0.1007731154	0.07567	0.18300	-0.047559468	0.2491056986
Inverse v	112	0.1148701555	0.08630	0.18318	-0.05428226	0.2840225712
Inverse v	112	-0.105018183	0.07913	0.18445	-0.260113163	0.0500767976
Inverse v	112	-0.112341954	0.08509	0.18675	-0.279121718	0.0544378098
Inverse v	112	-0.111757441	0.08545	0.19094	-0.279248159	0.0557332776
Inverse v	112	0.1198983366	0.09176	0.19135	-0.059962003	0.2997586762

Inverse v	112	0.0991414828	0.07580	0.19093	-0.049439675	0.2477226407
Inverse v	112	-0.1030973	0.07975	0.19611	-0.259413327	0.053218727
Inverse v	112	0.1198211826	0.09292	0.19725	-0.062314805	0.3019571700
Inverse v	112	0.1077397120	0.08430	0.20127	-0.057505915	0.2729853390
Inverse v	112	0.1170326508	0.09136	0.20020	-0.06203889	0.2961041912
Inverse v	112	-0.105791593	0.08283	0.20157	-0.268154864	0.056571678
Inverse v	112	-0.103338717	0.08070	0.20036	-0.261513874	0.054836440
Inverse v	112	0.1059842031	0.08312	0.20231	-0.056941143	0.2689095496
Inverse v	112	-0.101661209	0.07973	0.20228	-0.257933207	0.054610788
Inverse v	112	0.0997978015	0.07814	0.20154	-0.053358778	0.2529543805
Inverse v	112	-0.109266956	0.08583	0.20301	-0.277501897	0.058967984
Inverse v	112	-0.097231264	0.07662	0.20445	-0.247411903	0.052949375
Inverse v	112	0.1049050604	0.08258	0.20397	-0.056957848	0.2667679691
Inverse v	112	0.1008805845	0.07964	0.20526	-0.055215376	0.2569765447
Inverse v	112	-0.100631984	0.07960	0.20617	-0.256657733	0.055393764
Inverse v	112	0.0994128736	0.07904	0.20848	-0.055508055	0.2543338021
Inverse v	112	-0.102255181	0.08141	0.20912	-0.261827361	0.057316999
Inverse v	112	-0.106588623	0.08478	0.20870	-0.272771831	0.059594585
Inverse v	112	-0.12114764	0.09725	0.21290	-0.31177442	0.069479139
Inverse v	112	-0.108215019	0.08684	0.21272	-0.278424976	0.061994938
Inverse v	112	0.3240458817	0.25950	0.21176	-0.184577692	0.8326694559
Inverse v	112	0.1087312705	0.08711	0.21196	-0.062009062	0.2794716034
Inverse v	112	0.0967202810	0.07735	0.21115	-0.054888733	0.2483292950
Inverse v	112	0.0943308106	0.07586	0.21373	-0.054368128	0.2430297494
Inverse v	112	0.1078871324	0.08701	0.21500	-0.062655117	0.2784293818
Inverse v	112	0.0988147136	0.07996	0.21653	-0.057907961	0.2555373883
Inverse v	112	0.0994707068	0.08082	0.21846	-0.058955211	0.2578966251
Inverse v	112	0.0873475699	0.07111	0.21932	-0.052028882	0.2267240220
Inverse v	112	-0.093338727	0.07609	0.21995	-0.242480277	0.055802822
Inverse v	112	-0.085667851	0.06996	0.22076	-0.222792875	0.051457173
Inverse v	112	0.0963643682	0.07886	0.22176	-0.058216617	0.2509453530
Inverse v	112	-0.099327823	0.08155	0.22325	-0.259175816	0.060520169
Inverse v	112	0.0992606375	0.08148	0.22318	-0.060456685	0.2589779597
Inverse v	112	-0.081729594	0.06718	0.22382	-0.21341894	0.049959751
Inverse v	112	0.0949076102	0.07885	0.22874	-0.059644432	0.2494596527
Inverse v	112	0.0969835859	0.08050	0.22831	-0.060802893	0.2547700654
Inverse v	112	-0.096753302	0.08055	0.22972	-0.254643076	0.061136471
Inverse v	112	0.0981396736	0.08214	0.23218	-0.06285827	0.2591376174

Inverse v	112	0.0923635398	0.07733	0.23237	-0.059221182	0.2439482619
Inverse v	112	-0.094172463	0.07885	0.23235	-0.248718931	0.0603740062
Inverse v	112	-0.10741908	0.09019	0.23369	-0.284210411	0.0693722505
Inverse v	112	0.1050782137	0.08844	0.23478	-0.068266782	0.2784232096
Inverse v	112	0.0942856475	0.07966	0.23661	-0.061862683	0.2504339781
Inverse v	112	-0.094535733	0.07983	0.23636	-0.251015119	0.0619436524
Inverse v	112	0.1023752068	0.08632	0.23563	-0.066816496	0.2715669099
Inverse v	112	0.0928919553	0.07900	0.23971	-0.061967151	0.2477510612
Inverse v	112	-0.092621096	0.07943	0.24361	-0.248315074	0.0630728818
Inverse v	112	0.1053892641	0.09042	0.24384	-0.071853462	0.2826319899
Inverse v	112	-0.091912244	0.07919	0.24578	-0.247126586	0.0633020980
Inverse v	112	-0.09066034	0.07801	0.24519	-0.243568331	0.0622476516
Inverse v	112	0.0924371672	0.07963	0.24573	-0.063646001	0.2485203357
Inverse v	112	-0.091161805	0.07872	0.24687	-0.245464894	0.0631412839
Inverse v	112	0.0968109362	0.08379	0.24795	-0.067428113	0.2610499856
Inverse v	112	0.0975521224	0.08442	0.24789	-0.067923897	0.2630281422
Inverse v	112	0.0997658693	0.08703	0.25168	-0.070824072	0.2703558107
Inverse v	112	0.0925636190	0.08048	0.25009	-0.065182286	0.2503095239
Inverse v	112	-0.096385802	0.08403	0.25140	-0.261099723	0.0683281186
Inverse v	112	-0.088349966	0.07676	0.24978	-0.238813787	0.0621138552
Inverse v	112	-0.09635489	0.08391	0.25084	-0.260819383	0.0681096033
Inverse v	112	0.0885356990	0.07736	0.25244	-0.063094318	0.2401657161
Inverse v	112	-0.095919819	0.08425	0.25495	-0.261065592	0.0692259549
Inverse v	112	0.0840953692	0.07386	0.25494	-0.060689246	0.2288799839
Inverse v	112	-0.091162785	0.08000	0.25451	-0.247973192	0.0656476208
Inverse v	112	-0.086570998	0.07650	0.25779	-0.236515275	0.0633732782
Inverse v	112	-0.117888125	0.10470	0.26019	-0.323104075	0.0873278241
Inverse v	112	-0.09247253	0.08275	0.26382	-0.254675724	0.0697306634
Inverse v	112	0.0873740920	0.07819	0.26380	-0.065881414	0.2406295984
Inverse v	112	0.0871011937	0.07859	0.26774	-0.066938985	0.2411413722
Inverse v	112	0.0879414270	0.07923	0.26705	-0.067359755	0.2432426090
Inverse v	112	0.0884041020	0.07974	0.26758	-0.06788812	0.2446963242
Inverse v	112	-0.064430583	0.05830	0.26914	-0.178711498	0.0498503308
Inverse v	112	-0.097810635	0.08865	0.26991	-0.27157707	0.0759557999
Inverse v	112	0.0889279440	0.08145	0.27493	-0.070720115	0.2485760030
Inverse v	112	0.1487664400	0.13640	0.27544	-0.118589955	0.4161228354
Inverse v	112	0.0898400670	0.08233	0.27520	-0.071536744	0.2512168775
Inverse v	112	-0.093442137	0.08762	0.28626	-0.265193351	0.0783090776

Inverse v	112	0.0910573413	0.08469	0.28234	-0.07495341	0.2570680922
Inverse v	112	0.0877660357	0.08150	0.28154	-0.071976628	0.2475086994
Inverse v	112	0.0860954653	0.07986	0.28102	-0.070439243	0.2426301736
Inverse v	112	-0.085682603	0.07968	0.28228	-0.241874464	0.0705092589
Inverse v	112	0.0889173885	0.08332	0.28593	-0.074406091	0.2522408686
Inverse v	112	-0.091196837	0.08527	0.28485	-0.25833224	0.0759385662
Inverse v	112	-0.08377132	0.07826	0.28443	-0.237164348	0.0696217086
Inverse v	112	0.0889323631	0.08312	0.28466	-0.073988762	0.2518534886
Inverse v	112	0.0883024093	0.08262	0.28520	-0.073645171	0.2502499898
Inverse v	112	-0.096033155	0.08983	0.28506	-0.272106538	0.0800402276
Inverse v	112	-0.08453555	0.07946	0.28743	-0.240293355	0.0712222556
Inverse v	112	-0.089619429	0.08456	0.28924	-0.25536479	0.0761259316
Inverse v	112	0.0871804408	0.08235	0.28979	-0.07423747	0.2485983513
Inverse v	112	0.0600578080	0.05746	0.29595	-0.052569769	0.1726853846
Inverse v	112	-0.082197567	0.07883	0.29708	-0.236705127	0.0723099927
Inverse v	112	0.0824448319	0.07906	0.29705	-0.072518615	0.2374082791
Inverse v	112	-0.083077754	0.07980	0.29784	-0.239487407	0.0733318988
Inverse v	112	0.0980418577	0.09443	0.29917	-0.087049229	0.2831329440
Inverse v	112	-0.081658545	0.07930	0.30314	-0.237091061	0.0737739709
Inverse v	112	0.0786732441	0.07685	0.30599	-0.071962372	0.2293088600
Inverse v	112	-0.083012816	0.08020	0.30066	-0.240213946	0.0741883152
Inverse v	112	-0.089288718	0.08751	0.30758	-0.260810947	0.0822335112
Inverse v	112	0.0816333182	0.07974	0.30600	-0.074672863	0.2379394990
Inverse v	112	0.0861187800	0.08431	0.30706	-0.079138342	0.2513759023
Inverse v	112	-0.094836063	0.09216	0.30348	-0.275478195	0.0858060696
Inverse v	112	0.0889112777	0.08652	0.30416	-0.080684804	0.2585073600
Inverse v	112	-0.081764767	0.07953	0.30393	-0.237654796	0.0741252620
Inverse v	112	0.0853299951	0.08311	0.30456	-0.077570133	0.2482301235
Inverse v	112	-0.094326139	0.09227	0.30667	-0.275184539	0.0865322615
Inverse v	112	0.0853322874	0.08273	0.30235	-0.076828356	0.2474929305
Inverse v	112	-0.079759411	0.07704	0.30054	-0.230762551	0.0712437282
Inverse v	112	0.0775933067	0.07620	0.30857	-0.071769185	0.2269557986
Inverse v	112	0.0756447573	0.07441	0.30937	-0.07020614	0.2214956544
Inverse v	112	-0.067310435	0.06659	0.31216	-0.197844178	0.0632233080
Inverse v	112	0.0780958620	0.07757	0.31406	-0.073947645	0.2301393686
Inverse v	112	0.0851592999	0.08482	0.31540	-0.081095536	0.2514141361
Inverse v	112	0.0817555807	0.08140	0.31521	-0.077793846	0.2413050075
Inverse v	112	-0.080754208	0.08026	0.31439	-0.238080579	0.0765721620

Inverse v	112	-0.076650619	0.07672(	0.31778(	-0.22703446	0.0737332214:
Inverse v	112	0.0839967577	0.08407(	0.31778(	-0.080796769	0.2487902846
Inverse v	112	0.0874441271	0.08740	0.31707	-0.083863091	0.2587513452:
Inverse v	112	-0.092489969	0.09334	0.32177(	-0.275450923	0.0904709857(
Inverse v	112	-0.078043196	0.07941(	0.32571	-0.233687481	0.0776010884
Inverse v	112	-0.0683547	0.06964	0.32636	-0.204860009	0.0681506083(
Inverse v	112	-0.081117231	0.08184	0.32164(	-0.241538109	0.0793036477(
Inverse v	112	0.0773623017(	0.07848(	0.32425	-0.076459831	0.2311844346(
Inverse v	112	0.0788789932(	0.08032	0.32609(	-0.078557153	0.2363151390
Inverse v	112	0.0752335189(	0.07628(	0.32403(	-0.074288786	0.2247558241
Inverse v	112	0.0811902950	0.08234	0.32413	-0.080203757	0.2425843471
Inverse v	112	0.0785401642(	0.07972	0.32454(	-0.077718794	0.2347991228(
Inverse v	112	0.0827916066	0.08421(	0.32557(	-0.082276595	0.2478598079
Inverse v	112	0.0831315759	0.08476	0.32670	-0.083001465	0.2492646174(
Inverse v	112	-0.083757226	0.08460	0.32218(	-0.24958474	0.0820702868
Inverse v	112	0.0796060887	0.08158	0.32917	-0.080294699	0.2395068761(
Inverse v	112	-0.077841964	0.07981	0.32941(	-0.23427853	0.0785946023(
Inverse v	112	0.0958882088(	0.09802(	0.32798	-0.096244128	0.2880205460(
Inverse v	112	-0.085161299	0.08729(	0.32926(	-0.256251159	0.0859285614
Inverse v	112	-0.085847294	0.08856	0.33237(	-0.259431544	0.0877369557(
Inverse v	112	0.0785615427(	0.08110	0.33273(	-0.08040896	0.2375320456(
Inverse v	112	-0.098654352	0.10229(	0.33484	-0.299154869	0.1018461656
Inverse v	112	0.0821068255	0.08516	0.33500	-0.084818366	0.2490320172(
Inverse v	112	-0.080925467	0.08480	0.33994	-0.247139102	0.0852881681
Inverse v	112	-0.076580609	0.07975(	0.33692	-0.232890703	0.0797294844(
Inverse v	112	-0.088915119	0.09301(	0.33908	-0.271214889	0.0933846500(
Inverse v	112	0.0782881491	0.08218	0.34081(	-0.082800299	0.2393765975(
Inverse v	112	-0.076788046	0.08010	0.33775	-0.233789625	0.0802135325(
Inverse v	112	-0.075421845	0.07911	0.34043	-0.230489095	0.0796454062(
Inverse v	112	0.0760269937(	0.07958	0.33941(	-0.079956091	0.2320100782
Inverse v	112	-0.096125107	0.10019(	0.33737	-0.292509575	0.1002593608(
Inverse v	112	-0.080865803	0.08505	0.34171	-0.247567359	0.0858357537
Inverse v	112	-0.083111435	0.08764(	0.34299(	-0.254897756	0.0886748867(
Inverse v	112	0.0805146697	0.08563	0.34711	-0.087331208	0.2483605473(
Inverse v	112	-0.074650454	0.07941	0.34719	-0.230297262	0.0809963545
Inverse v	112	0.0696340041(	0.07465	0.35094	-0.076688425	0.2159564335(
Inverse v	112	-0.083294616	0.08947(	0.35186	-0.258656041	0.0920668095
Inverse v	112	0.0728912796	0.07856	0.35350	-0.081091572	0.2268741316(

Inverse v	112	0.07371810441	0.07977	0.35544	-0.08263901	0.23007521896
Inverse v	112	-0.072942281	0.07922	0.35718	-0.228215377	0.08233081388
Inverse v	112	0.07763375274	0.08427	0.35693	-0.087540034	0.24280753976
Inverse v	112	-0.077262642	0.08433	0.35961	-0.242565254	0.08803997047
Inverse v	112	0.07720445001	0.08489	0.36314	-0.089193022	0.24360192230
Inverse v	112	-0.072259144	0.07943	0.36299	-0.227951332	0.08343304302
Inverse v	112	-0.073511914	0.08159	0.36764	-0.233446533	0.08642270480
Inverse v	112	0.07180276477	0.07968	0.36754	-0.084378504	0.22798403346
Inverse v	112	0.07205762624	0.07977	0.36639	-0.084303707	0.22841895982
Inverse v	112	-0.079249838	0.08772	0.36630	-0.251185442	0.09268576495
Inverse v	112	0.07150264783	0.07989	0.37078	-0.085082885	0.22808818070
Inverse v	112	0.07485169024	0.08377	0.37158	-0.089342409	0.23904578973
Inverse v	112	-0.07042189	0.07953	0.37594	-0.226316265	0.08547248551
Inverse v	112	-0.067805246	0.07677	0.37714	-0.218283862	0.08267336937
Inverse v	112	-0.075968602	0.08579	0.37591	-0.244131618	0.09219441528
Inverse v	112	0.07018053402	0.07948	0.37728	-0.085616892	0.22597796021
Inverse v	112	-0.069615284	0.07869	0.37633	-0.223848677	0.08461810987
Inverse v	112	0.06822805698	0.07716	0.37662	-0.083024107	0.21948022077
Inverse v	112	-0.073878765	0.08481	0.38370	-0.240111091	0.09235356187
Inverse v	112	-0.078662268	0.08952	0.37956	-0.254125765	0.09680122809
Inverse v	112	-0.069046575	0.07896	0.38189	-0.223815542	0.08572239207
Inverse v	112	-0.069132355	0.07927	0.38316	-0.224507403	0.08624269369
Inverse v	112	-0.065545041	0.07473	0.38047	-0.212026155	0.08093607260
Inverse v	112	-0.069686753	0.07998	0.38360	-0.22645137	0.08707786507
Inverse v	112	-0.067917554	0.07783	0.38289	-0.220475517	0.08464040897
Inverse v	112	-0.066190825	0.07647	0.38677	-0.216090583	0.08370893209
Inverse v	112	-0.068043995	0.07853	0.38624	-0.221966211	0.08587822037
Inverse v	112	-0.069932532	0.08104	0.38817	-0.228773709	0.08890864540
Inverse v	112	-0.067702394	0.07863	0.38924	-0.221823506	0.08641871770
Inverse v	112	0.06327360032	0.07378	0.39114	-0.081345882	0.20789308263
Inverse v	112	-0.069187592	0.08084	0.39209	-0.227639222	0.08926403737
Inverse v	112	-0.070821112	0.08318	0.39454	-0.233856492	0.09221426918
Inverse v	112	-0.067034993	0.07958	0.39958	-0.223012391	0.08894240496
Inverse v	112	-0.067030535	0.07870	0.39440	-0.22129463	0.08723355945
Inverse v	112	0.06682675347	0.07844	0.39427	-0.08692526	0.22057876728
Inverse v	112	-0.065416053	0.07765	0.39953	-0.217610726	0.08677862055
Inverse v	112	0.06623447623	0.07831	0.39769	-0.087263863	0.21973281581
Inverse v	112	0.06542838884	0.07773	0.39997	-0.086936161	0.21779293891

Inverse v	112	-0.067048408	0.07921	0.39732	-0.222310283	0.0882134672
Inverse v	112	-0.068361907	0.08081	0.39759	-0.226756901	0.0900330866
Inverse v	112	-0.067418294	0.07947	0.39626	-0.223184961	0.0883483738
Inverse v	112	-0.067442351	0.07993	0.39882	-0.224114643	0.0892299412
Inverse v	112	0.0659674233	0.07925	0.40520	-0.089368474	0.2213033205
Inverse v	112	-0.061584735	0.07403	0.40552	-0.206701073	0.0835316035
Inverse v	112	-0.065169797	0.07858	0.40692	-0.219190579	0.0888509843
Inverse v	112	0.0690640990	0.08358	0.40867	-0.094770178	0.2328983765
Inverse v	112	0.0649902203	0.07863	0.40855	-0.089140993	0.2191214337
Inverse v	112	0.0646930337	0.07830	0.40873	-0.088793134	0.2181792016
Inverse v	112	0.0700244267	0.08430	0.40619	-0.095213273	0.2352621263
Inverse v	112	0.0723797668	0.08677	0.40419	-0.097690168	0.2424497011
Inverse v	112	0.0610600439	0.07294	0.40257	-0.08191921	0.2040392975
Inverse v	112	0.0689496800	0.08241	0.40279	-0.092579828	0.2304791880
Inverse v	112	0.0671165822	0.08113	0.40811	-0.091909301	0.2261424652
Inverse v	112	0.0713193936	0.08661	0.41027	-0.098445167	0.2410839546
Inverse v	112	-0.064399555	0.07927	0.41658	-0.21977758	0.0909784696
Inverse v	112	-0.056186929	0.06961	0.41963	-0.192641601	0.0802677437
Inverse v	112	0.0682473973	0.08458	0.41976	-0.097544078	0.2340388730
Inverse v	112	0.0727292332	0.08984	0.41823	-0.103369758	0.2488282245
Inverse v	112	0.0645126178	0.07987	0.41925	-0.092032698	0.2210579335
Inverse v	112	0.0603532320	0.07513	0.42179	-0.086904176	0.2076106406
Inverse v	112	-0.069204115	0.08681	0.42537	-0.239363889	0.1009556593
Inverse v	112	0.0631351655	0.07912	0.42494	-0.09195793	0.2182282614
Inverse v	112	0.0680173692	0.08514	0.42438	-0.098867769	0.2349025080
Inverse v	112	0.0702291458	0.08868	0.42843	-0.103599678	0.2440579698
Inverse v	112	-0.070554418	0.08975	0.43184	-0.246482928	0.1053740921
Inverse v	112	-0.066305805	0.08446	0.43246	-0.231864706	0.0992530954
Inverse v	112	-0.066440385	0.08486	0.43369	-0.232776857	0.0998960871
Inverse v	112	0.0590466516	0.07556	0.43454	-0.089053826	0.2071471290
Inverse v	112	-0.062991201	0.08085	0.43594	-0.221468438	0.0954860345
Inverse v	112	0.0595697204	0.07658	0.43666	-0.090533607	0.2096730478
Inverse v	112	0.0587293056	0.07568	0.43776	-0.089613004	0.2070716151
Inverse v	112	0.0641211553	0.08303	0.43999	-0.09863041	0.2268727204
Inverse v	112	0.0597190830	0.07745	0.44071	-0.092099847	0.2115380135
Inverse v	112	-0.091020453	0.11855	0.44265	-0.323395281	0.1413543737
Inverse v	112	-0.059679656	0.07787	0.44347	-0.212317739	0.0929584274
Inverse v	112	0.0553361870	0.07247	0.44512	-0.086705821	0.1973781953

Inverse v	112	-0.060703365	0.07964	0.44593	-0.216801271	0.0953945414
Inverse v	112	0.0631422555	0.08328	0.44838	-0.100104714	0.2263892254
Inverse v	112	-0.066264094	0.08736	0.44814	-0.237490973	0.1049627857
Inverse v	112	0.0613836331	0.08071	0.44697	-0.096824788	0.2195920543
Inverse v	112	-0.059461674	0.07860	0.44934	-0.213517981	0.0945946336
Inverse v	112	0.0588816201	0.07798	0.45023	-0.093972642	0.2117358827
Inverse v	112	-0.066350763	0.08829	0.45238	-0.239412524	0.1067109978
Inverse v	112	0.0590450689	0.07894	0.45450	-0.095688649	0.2137787868
Inverse v	112	-0.058695063	0.07840	0.45411	-0.212377286	0.0949871601
Inverse v	112	-0.061270532	0.08195	0.45469	-0.221904284	0.0993632193
Inverse v	112	-0.059429517	0.07988	0.45692	-0.216007928	0.0971488932
Inverse v	112	-0.065027342	0.08730	0.45639	-0.23615135	0.1060966651
Inverse v	112	-0.063739538	0.08597	0.45844	-0.232242209	0.1047631342
Inverse v	112	-0.057332616	0.07772	0.46072	-0.209671121	0.0950058888
Inverse v	112	-0.058310052	0.07893	0.46010	-0.213031257	0.0964111525
Inverse v	112	-0.062443036	0.08481	0.46159	-0.22867987	0.1037937988
Inverse v	112	0.0586471693	0.08051	0.46638	-0.099166766	0.2164611047
Inverse v	112	0.0582129977	0.07988	0.46616	-0.098357901	0.2147838965
Inverse v	112	-0.057183427	0.07840	0.46577	-0.210851076	0.0964842219
Inverse v	112	-0.059356427	0.08133	0.46549	-0.218763272	0.1000504184
Inverse v	112	-0.061016291	0.08467	0.47117	-0.226983351	0.1049507685
Inverse v	112	0.0551711710	0.07653	0.47101	-0.094845965	0.2051883075
Inverse v	112	-0.057732731	0.07988	0.46988	-0.214315746	0.0988502834
Inverse v	112	-0.056929437	0.07880	0.47006	-0.211394744	0.0975358694
Inverse v	112	0.0570206284	0.07927	0.47199	-0.098365291	0.2124065483
Inverse v	112	0.0639092889	0.08908	0.47311	-0.11069134	0.2385099178
Inverse v	112	0.0582009323	0.08129	0.47406	-0.101146711	0.2175485755
Inverse v	112	0.0587879967	0.08235	0.47531	-0.102622525	0.2201985189
Inverse v	112	0.0581878438	0.08182	0.47701	-0.102193275	0.2185689627
Inverse v	112	0.0629955854	0.08912	0.47967	-0.111687586	0.2376787571
Inverse v	112	0.0562663590	0.07972	0.48036	-0.100002959	0.2125356773
Inverse v	112	-0.056052599	0.07979	0.48239	-0.212450923	0.1003457253
Inverse v	112	0.0546077329	0.07838	0.48601	-0.099028393	0.2082438592
Inverse v	112	0.0585988329	0.08403	0.48559	-0.106105059	0.2233027249
Inverse v	112	-0.055728178	0.07989	0.48545	-0.212313371	0.1008570154
Inverse v	112	0.0666759223	0.09536	0.48446	-0.120244722	0.2535965666
Inverse v	112	0.0605400190	0.08807	0.49186	-0.112094212	0.2331742497
Inverse v	112	0.2550725569	0.37243	0.49341	-0.474893852	0.9850389659

Inverse v	112	0.0610553626	0.08916	0.49348	-0.11369909	0.2358098155
Inverse v	112	-0.054211386	0.07944	0.49501	-0.209927375	0.1015046020
Inverse v	112	0.0585904221	0.08586	0.49501	-0.109705568	0.2268864121
Inverse v	112	0.0562993040	0.08353	0.50033	-0.10742828	0.2200268878
Inverse v	112	0.0532160553	0.07920	0.50164	-0.102019468	0.2084515789
Inverse v	112	0.0537739268	0.08007	0.50186	-0.103171516	0.2107193694
Inverse v	112	-0.055805235	0.08271	0.49989	-0.217929335	0.1063188642
Inverse v	112	-0.055012539	0.08173	0.50090	-0.215212411	0.1051873318
Inverse v	112	-0.053428389	0.07934	0.50069	-0.208937044	0.1020802656
Inverse v	112	0.0563702014	0.08327	0.49843	-0.106839885	0.2195802877
Inverse v	112	0.0536905472	0.07951	0.49955	-0.102165937	0.2095470311
Inverse v	112	-0.057739714	0.08673	0.50562	-0.227748284	0.1122688556
Inverse v	112	-0.05740201	0.08617	0.50535	-0.22631001	0.1115059912
Inverse v	112	0.0521045827	0.07828	0.50570	-0.101343604	0.2055527694
Inverse v	112	0.0531960715	0.07993	0.50572	-0.103473677	0.2098658205
Inverse v	112	0.0508691922	0.07674	0.50741	-0.099543776	0.2012821608
Inverse v	112	-0.056445409	0.08577	0.51051	-0.224573105	0.1116822876
Inverse v	112	0.0535362115	0.08115	0.50947	-0.10553285	0.2126052730
Inverse v	112	-0.053065503	0.08045	0.50950	-0.210748121	0.1046171147
Inverse v	112	-0.057098113	0.08662	0.50980	-0.226884127	0.1126879001
Inverse v	112	0.0569962948	0.08722	0.51346	-0.113961808	0.2279543981
Inverse v	112	0.0308532708	0.04735	0.51472	-0.061968125	0.1236746669
Inverse v	112	0.0503929669	0.07689	0.51226	-0.1003287	0.2011146333
Inverse v	112	-0.058914854	0.09027	0.51401	-0.235858221	0.1180285136
Inverse v	112	0.0510868561	0.07826	0.51393	-0.10231873	0.2044924420
Inverse v	112	0.0661360130	0.10430	0.52603	-0.138301624	0.2705736502
Inverse v	112	-0.050984101	0.07966	0.52219	-0.207130416	0.1051622139
Inverse v	112	0.0552632689	0.08825	0.53121	-0.117725102	0.2282516395
Inverse v	112	0.0511102780	0.07956	0.52062	-0.104832671	0.2070532274
Inverse v	112	-0.049537702	0.07692	0.51960	-0.200316518	0.1012411141
Inverse v	112	-0.049576656	0.07874	0.52898	-0.203922502	0.1047691905
Inverse v	112	-0.053039402	0.08400	0.52778	-0.217685999	0.1116071952
Inverse v	112	0.0478523342	0.07456	0.52104	-0.0982987	0.1940033687
Inverse v	112	0.0514561829	0.08074	0.52393	-0.106797722	0.2097100873
Inverse v	112	0.0488791585	0.07730	0.52720	-0.102641959	0.2004002763
Inverse v	112	-0.054739263	0.08749	0.53153	-0.226221165	0.1167426390
Inverse v	112	-0.049862367	0.07763	0.52068	-0.202020721	0.1022959871
Inverse v	112	-0.058413188	0.09029	0.51767	-0.235386349	0.1185599737

Inverse v	112	0.0513289219(	0.07948:	0.51842	-0.104458907	0.20711675042
Inverse v	112	-0.052759599	0.08392'	0.52958!	-0.217257504	0.11173830545
Inverse v	112	-0.054043141	0.08405!	0.52026	-0.218792866	0.1107065848(
Inverse v	112	-0.050208852	0.07956!	0.52803'	-0.206165594	0.10574788952
Inverse v	112	0.0504326458'	0.07917	0.52413!	-0.10474981	0.20561510223
Inverse v	112	-0.057480028	0.08944!	0.52048'	-0.232802006	0.11784195007
Inverse v	112	0.0497254436'	0.07911	0.52965!	-0.105338899	0.20478978673
Inverse v	112	0.0493008322:	0.07884(	0.53175(	-0.105225926	0.20382759087
Inverse v	112	0.0488888265	0.07772:	0.52933!	-0.103446623	0.20122427641
Inverse v	112	-0.051873442	0.08328(	0.53336'	-0.215103808	0.11135692348
Inverse v	112	-0.059653531	0.09671!	0.53737	-0.24921651	0.12990944749
Inverse v	112	-0.050535886	0.08193(	0.53738!	-0.211132175	0.11006040214
Inverse v	112	-0.047017567	0.07679(	0.54038(	-0.197538067	0.10350293316
Inverse v	112	-0.050905371	0.08367'	0.54295(	-0.214914058	0.11310331631
Inverse v	112	-0.048615694	0.08013!	0.54408!	-0.205688186	0.10845679854
Inverse v	112	0.0481539598(	0.07969!	0.54571:	-0.10805647	0.20436438912
Inverse v	112	-0.048286917	0.07996!	0.54594!	-0.205019476	0.10844564083
Inverse v	112	-0.060114475	0.09944'	0.54552:	-0.255032504	0.13480355429
Inverse v	112	-0.046333527	0.07744'	0.54966!	-0.198130787	0.10546373293
Inverse v	112	0.0515453647!	0.08700!	0.55357(	-0.118993727	0.22208445649
Inverse v	112	-0.051876859	0.08713!	0.55162:	-0.222670582	0.11891686372
Inverse v	112	0.0460364309	0.07756!	0.55285!	-0.106000678	0.19807354007
Inverse v	112	-0.052076766	0.08755(	0.55199(	-0.223687126	0.11953359405
Inverse v	112	-0.054325327	0.09134(	0.55200	-0.233351938	0.12470128347
Inverse v	112	-0.045365325	0.07648(	0.55307:	-0.19526752	0.10453687013
Inverse v	112	0.0439534213'	0.07445	0.55494	-0.101970687	0.18987752953
Inverse v	112	0.0492591819!	0.08530!	0.56365!	-0.117947985	0.21646634906
Inverse v	112	-0.046693784	0.07966	0.55777(	-0.202829432	0.10944186336
Inverse v	112	0.0455945347:	0.07964(	0.56698:	-0.110501722	0.20169079142
Inverse v	112	-0.046778919	0.08168'	0.56687'	-0.20688657	0.11332873286
Inverse v	112	0.0461578069:	0.07869	0.55749:	-0.108076742	0.20039235586
Inverse v	112	0.0501994032(	0.08707(	0.56428(	-0.120471328	0.22087013462
Inverse v	112	-0.045677664	0.07990	0.56754:	-0.20228502	0.11092969248
Inverse v	112	0.0441062776(	0.07709!	0.56727	-0.107008504	0.19522105952
Inverse v	112	0.0454797826:	0.07964!	0.56799:	-0.110630437	0.20159000267
Inverse v	112	-0.05096002	0.08843(	0.56442!	-0.224283169	0.1223631283(
Inverse v	112	0.0468168507:	0.08146!	0.56552	-0.1128622	0.2064959012(
Inverse v	112	-0.047000263	0.08194:	0.56625	-0.207607395	0.11360686859

Inverse v	112	0.04555878027	0.078349	0.560917	-0.108007003	0.19912456323
Inverse v	112	-0.047909251	0.083055	0.564050	-0.210697453	0.1148789522
Inverse v	112	-0.046955683	0.080272	0.558580	-0.204290519	0.1103791525
Inverse v	112	-0.048881156	0.084572	0.563278	-0.214643256	0.1168809446
Inverse v	112	0.0452136235	0.079834	0.571159	-0.111261253	0.2016885003
Inverse v	112	0.0438216074	0.077239	0.570471	-0.107567579	0.1952107936
Inverse v	112	-0.04939973	0.087210	0.571121	-0.220345036	0.1215455761
Inverse v	112	-0.045101122	0.079831	0.572105	-0.201570837	0.1113685937
Inverse v	112	-0.045943187	0.082093	0.575723	-0.206846975	0.1149606008
Inverse v	112	-0.047781925	0.085618	0.576791	-0.215594902	0.1200310532
Inverse v	112	0.0503586552	0.090229	0.576765	-0.126491842	0.2272091526
Inverse v	112	-0.04515381	0.080703	0.575818	-0.203332414	0.1130247940
Inverse v	112	0.0460207136	0.082600	0.577425	-0.115875856	0.2079172831
Inverse v	112	0.0447012091	0.080368	0.578071	-0.112820665	0.2022230837
Inverse v	112	0.0499210840	0.090273	0.580265	-0.12701552	0.2268576876
Inverse v	112	-0.043574974	0.079318	0.582755	-0.199040186	0.1118902373
Inverse v	112	0.0416725461	0.076295	0.584920	-0.107865762	0.1912108543
Inverse v	112	-0.043758113	0.080694	0.587633	-0.201919436	0.1144032088
Inverse v	112	-0.046828418	0.086549	0.588464	-0.216464649	0.1228078120
Inverse v	112	0.0412078972	0.076330	0.589292	-0.108399834	0.1908156286
Inverse v	112	0.0453788135	0.084447	0.591019	-0.120138984	0.2108966112
Inverse v	112	0.0446798196	0.083170	0.591149	-0.118345587	0.2077052262
Inverse v	112	-0.046563326	0.087113	0.592987	-0.217306475	0.1241798235
Inverse v	112	-0.047542956	0.089227	0.594151	-0.222428652	0.1273427403
Inverse v	112	0.0588064206	0.110817	0.595655	-0.158396408	0.2760092496
Inverse v	112	-0.0441916	0.083279	0.595647	-0.207410639	0.1190274385
Inverse v	112	-0.041817593	0.078940	0.596295	-0.196541239	0.1129060521
Inverse v	112	0.0420192941	0.079848	0.598721	-0.114482977	0.1985215650
Inverse v	112	0.0416887183	0.078947	0.597458	-0.113047508	0.1964249448
Inverse v	112	-0.044816371	0.085033	0.598161	-0.211481445	0.1218487026
Inverse v	112	0.0462773705	0.088364	0.600481	-0.126917862	0.2194726031
Inverse v	112	-0.041971796	0.080881	0.603811	-0.200500147	0.1165565553
Inverse v	112	-0.041034129	0.079152	0.604160	-0.196173293	0.1141050350
Inverse v	112	-0.040742997	0.078911	0.605634	-0.195408941	0.1139229464
Inverse v	112	-0.040786771	0.079898	0.609715	-0.197388545	0.1158150022
Inverse v	112	-0.04310813	0.084093	0.608214	-0.207930809	0.1217145486
Inverse v	112	0.0396389049	0.077545	0.609231	-0.112349793	0.1916276026
Inverse v	112	-0.040201101	0.078650	0.609283	-0.194368051	0.1139658481

Inverse v	112	-0.041084684	0.08100	0.61202	-0.199854558	0.1176851905
Inverse v	112	0.0383287160	0.07666	0.61709	-0.11192712	0.1885845522
Inverse v	112	-0.040049583	0.07968	0.61523	-0.196229029	0.1161298628
Inverse v	112	0.0457957800	0.09100	0.61481	-0.132576609	0.2241681690
Inverse v	112	-0.040835518	0.08157	0.61667	-0.200727701	0.1190566652
Inverse v	112	-0.039732363	0.07915	0.61569	-0.194876282	0.1154115549
Inverse v	112	0.0397137285	0.07976	0.61854	-0.116618541	0.1960459979
Inverse v	112	-0.042528973	0.08545	0.61871	-0.210024369	0.1249664228
Inverse v	112	-0.043006089	0.08700	0.62110	-0.213537853	0.1275256752
Inverse v	112	-0.041503151	0.08381	0.62046	-0.205773183	0.1227668812
Inverse v	112	-0.041231086	0.08439	0.62515	-0.206640945	0.1241787732
Inverse v	112	-0.039657398	0.08094	0.62417	-0.19830775	0.1189929535
Inverse v	112	0.0391125081	0.08038	0.62658	-0.118449301	0.1966743169
Inverse v	112	-0.040262767	0.08207	0.62374	-0.201134981	0.1206094466
Inverse v	112	0.0368636964	0.07564	0.62601	-0.111396805	0.1851241979
Inverse v	112	0.0461296728	0.09596	0.63074	-0.14196817	0.2342275158
Inverse v	112	0.0430869288	0.08912	0.62876	-0.131590055	0.2177639126
Inverse v	112	-0.040192314	0.08351	0.63034	-0.203885741	0.1235011139
Inverse v	112	0.0402913509	0.08339	0.62901	-0.123170775	0.2037534769
Inverse v	112	0.0375790869	0.07817	0.63074	-0.115652946	0.1908111194
Inverse v	112	0.0370297110	0.07720	0.63148	-0.114290176	0.1883495982
Inverse v	112	0.0387024114	0.08098	0.63271	-0.120024911	0.1974297342
Inverse v	112	-0.038607846	0.08094	0.63339	-0.197261454	0.1200457619
Inverse v	112	-0.038753885	0.08395	0.64438	-0.203314949	0.1258071787
Inverse v	112	0.0370863720	0.07938	0.64035	-0.118499752	0.1926724963
Inverse v	112	0.0410025143	0.08867	0.64379	-0.132794772	0.2147998009
Inverse v	112	0.0364854900	0.07886	0.64362	-0.118087137	0.1910581170
Inverse v	112	0.0388281964	0.08390	0.64354	-0.125631634	0.2032880271
Inverse v	112	-0.040676558	0.08775	0.64299	-0.212681063	0.1313279474
Inverse v	112	0.0338708689	0.07595	0.65563	-0.114996361	0.1827380992
Inverse v	112	0.0350594415	0.07939	0.65878	-0.120553578	0.1906724609
Inverse v	112	0.0425146278	0.09776	0.66366	-0.149108681	0.2341379361
Inverse v	112	0.0374765775	0.08260	0.65004	-0.124423574	0.1993767294
Inverse v	112	-0.038293873	0.08505	0.65256	-0.20500892	0.1284211746
Inverse v	112	0.0376014546	0.08605	0.66215	-0.131070026	0.2062729351
Inverse v	112	-0.033993935	0.07682	0.65814	-0.184573856	0.1165859869
Inverse v	112	0.0361144009	0.08044	0.65346	-0.121548679	0.1937774811
Inverse v	112	-0.034528	0.07968	0.66480	-0.190715249	0.1216592486

Inverse v	112	0.03703243985	0.085383	0.664491	-0.130318675	0.20438355443
Inverse v	112	-0.036255076	0.079555	0.648591	-0.192184294	0.11967414109
Inverse v	112	-0.034642044	0.078919	0.660696	-0.189324929	0.12004084003
Inverse v	112	-0.035888699	0.079447	0.651465	-0.191606448	0.11982905024
Inverse v	112	0.03673053025	0.082646	0.656732	-0.125256746	0.19871780656
Inverse v	112	0.03620096599	0.080599	0.653327	-0.121774934	0.19417686579
Inverse v	112	-0.033507592	0.077429	0.665195	-0.185269101	0.11825391731
Inverse v	112	0.04279649644	0.098295	0.663281	-0.149862019	0.23545501221
Inverse v	112	0.03802162382	0.084721	0.653586	-0.128032058	0.20407530549
Inverse v	112	-0.035300899	0.080132	0.659554	-0.192361572	0.12175977376
Inverse v	112	0.03407731650	0.077764	0.661234	-0.118342022	0.18649665504
Inverse v	112	-0.037916083	0.083597	0.650150	-0.201768076	0.12593591018
Inverse v	112	-0.035461418	0.080361	0.659015	-0.192970368	0.12204753136
Inverse v	112	-0.040222055	0.089345	0.652575	-0.215338495	0.13489438447
Inverse v	112	0.03356932746	0.077008	0.662898	-0.1173681	0.18450675445
Inverse v	112	0.03599386017	0.079914	0.652418	-0.120638691	0.19262641172
Inverse v	112	-0.034825864	0.081135	0.667755	-0.193851452	0.12419972294
Inverse v	112	0.03542762429	0.082904	0.669140	-0.127066044	0.19792129289
Inverse v	112	-0.035030276	0.081772	0.668367	-0.195303561	0.12524300865
Inverse v	112	0.03575274619	0.083270	0.667680	-0.127469564	0.19897505672
Inverse v	112	-0.031092176	0.073420	0.671970	-0.1750085	0.11282414830
Inverse v	112	-0.052788066	0.124408	0.671337	-0.296628681	0.19105254914
Inverse v	112	-0.036584219	0.086930	0.673890	-0.20697999	0.13381155195
Inverse v	112	0.03332750880	0.079667	0.675704	-0.122821126	0.18947614316
Inverse v	112	-0.034268164	0.081822	0.675354	-0.194640184	0.12610385724
Inverse v	112	-0.032525467	0.079537	0.682590	-0.188419781	0.12336884752
Inverse v	112	-0.032137813	0.078750	0.683203	-0.186489012	0.122213386
Inverse v	112	-0.031904439	0.078975	0.686227	-0.18669609	0.12288721249
Inverse v	112	-0.033154825	0.083404	0.690984	-0.196627376	0.13031772583
Inverse v	112	0.03122377595	0.078897	0.692288	-0.123415024	0.18586257590
Inverse v	112	-0.032902283	0.081601	0.686795	-0.192841102	0.12703653515
Inverse v	112	-0.032897693	0.079892	0.680505	-0.189487305	0.12369191929
Inverse v	112	-0.034189045	0.086101	0.691308	-0.202947269	0.13456917905
Inverse v	112	-0.034432945	0.085167	0.685995	-0.201361675	0.13249578465
Inverse v	112	-0.026580396	0.066725	0.690368	-0.157362238	0.10420144610
Inverse v	112	-0.030940682	0.078810	0.694639	-0.185420949	0.12353958522
Inverse v	112	0.03125213128	0.077267	0.685867	-0.120191315	0.18269557783
Inverse v	112	-0.037266701	0.092173	0.685983	-0.217925932	0.14339252898

Inverse	112	0.0326483831	0.08212	0.69096	-0.128317559	0.1936143253
Inverse	112	-0.033070713	0.08217	0.68736	-0.194139833	0.1279984058
Inverse	112	0.0324651333	0.08240	0.69360	-0.129051524	0.1939817910
Inverse	112	0.0318129549	0.07977	0.69005	-0.124545328	0.1881712374
Inverse	112	-0.034969929	0.08764	0.68989	-0.206751688	0.1368118307
Inverse	112	0.0325269772	0.08275	0.69426	-0.129665437	0.1947193914
Inverse	112	0.0336841055	0.08315	0.68540	-0.129290771	0.1966589819
Inverse	112	-0.033255289	0.08338	0.69004	-0.196696932	0.1301863536
Inverse	112	-0.033269869	0.08380	0.69138	-0.197534255	0.1309945163
Inverse	112	0.0359203521	0.08719	0.68038	-0.134989601	0.2068303056
Inverse	112	-0.031569888	0.08129	0.69777	-0.190915548	0.1277757713
Inverse	112	-0.032456352	0.08373	0.69832	-0.196586128	0.1316734244
Inverse	112	0.0289541007	0.07848	0.71219	-0.124876758	0.1827849594
Inverse	112	-0.030701439	0.08167	0.70701	-0.19079422	0.1293913418
Inverse	112	-0.035999677	0.09690	0.71026	-0.225927384	0.1539280296
Inverse	112	0.0307234450	0.08159	0.70650	-0.129195252	0.1906421425
Inverse	112	-0.029617331	0.07954	0.70966	-0.185535035	0.1263003721
Inverse	112	-0.033591964	0.09082	0.71150	-0.211614871	0.1444309437
Inverse	112	-0.029128209	0.07569	0.70038	-0.177494377	0.1192379591
Inverse	112	0.0293165153	0.07945	0.71213	-0.12640766	0.1850406903
Inverse	112	0.0276457707	0.07354	0.70697	-0.116495679	0.1717872203
Inverse	112	0.0302412780	0.08046	0.70702	-0.127463104	0.1879456604
Inverse	112	-0.029276904	0.07766	0.70619	-0.181495728	0.1229419202
Inverse	112	0.0303031034	0.08020	0.70556	-0.126898241	0.1875044473
Inverse	112	0.0293566523	0.07970	0.71262	-0.126855391	0.1855686957
Inverse	112	0.0308191384	0.08282	0.70981	-0.131516807	0.1931550840
Inverse	112	-0.029381213	0.07609	0.69940	-0.178525275	0.1197628500
Inverse	112	-0.029026235	0.07658	0.70467	-0.179131239	0.1210787687
Inverse	112	-0.03071119	0.08011	0.70146	-0.187732645	0.1263102641
Inverse	112	0.0288284285	0.07858	0.71374	-0.125201633	0.1828584900
Inverse	112	0.0285163909	0.07897	0.71802	-0.126266793	0.1832995750
Inverse	112	0.0298402834	0.08260	0.71792	-0.132065809	0.1917463763
Inverse	112	0.0303215128	0.08361	0.71689	-0.133573377	0.1942164030
Inverse	112	-0.028560142	0.07983	0.72054	-0.18504063	0.1279203453
Inverse	112	0.0281927645	0.07860	0.71983	-0.125864696	0.1822502247
Inverse	112	-0.028068037	0.07898	0.72231	-0.182877872	0.1267417981
Inverse	112	-0.026056926	0.07390	0.72439	-0.170903676	0.1187898238
Inverse	112	0.0278472808	0.07866	0.72334	-0.126340281	0.1820348428

Inverse v	112	-0.027943426	0.07939(	0.72485(	-0.183547984	0.12766113291
Inverse v	112	0.02783546668	0.07894:	0.72438:	-0.1268896	0.18256053294
Inverse v	112	0.02778438167	0.07926(	0.72595:	-0.127581549	0.18315031202
Inverse v	112	-0.027515413	0.07888(	0.72724(	-0.182137918	0.12710709186
Inverse v	112	-0.028358211	0.08227:	0.73033	-0.189614966	0.13289854321
Inverse v	112	-0.032989141	0.09539:	0.72947:	-0.219957735	0.15397945221
Inverse v	112	0.0290926499(	0.08420:	0.72971(	-0.135942762	0.19412806136
Inverse v	112	0.0265073290(	0.07751:	0.73236:	-0.125414926	0.17842958434
Inverse v	112	0.0272503358(	0.08009:	0.73369(	-0.12974152	0.18424219138
Inverse v	112	0.02680475452	0.08212	0.74412(	-0.134159504	0.18776899463
Inverse v	112	-0.026265925	0.07961(	0.74147(	-0.182313618	0.12978176700
Inverse v	112	-0.026420488	0.08069:	0.74335:	-0.184583389	0.13174241229
Inverse v	112	-0.027465481	0.08375:	0.74297:	-0.191633582	0.13670261963
Inverse v	112	-0.027590139	0.08387(	0.74220:	-0.191987362	0.13680708253
Inverse v	112	0.02727827189	0.08284	0.74195:	-0.135097377	0.18965392080
Inverse v	112	-0.025995755	0.07862:	0.74091(	-0.180096915	0.12810540467
Inverse v	112	-0.026571334	0.08120	0.74350:	-0.185732395	0.13258972725
Inverse v	112	-0.027270106	0.08338:	0.74362:	-0.190698634	0.13615842090
Inverse v	112	0.0265583762(	0.07942:	0.73809(	-0.129114882	0.18223163496
Inverse v	112	-0.024897914	0.07691:	0.74616:	-0.175657014	0.12586118535
Inverse v	112	0.02676681307	0.08280:	0.74650:	-0.135528572	0.18906219778
Inverse v	112	-0.025588147	0.07923(	0.74675:	-0.180895799	0.12971950623
Inverse v	112	0.02602379184	0.08239:	0.75213(	-0.135475496	0.18752307942
Inverse v	112	0.02705108415	0.08544:	0.75155:	-0.14042229	0.19452445871
Inverse v	112	0.02497699764	0.07961(	0.75373:	-0.131070611	0.18102460627
Inverse v	112	-0.025735712	0.08305(	0.75666:	-0.188525499	0.13705407557
Inverse v	112	0.0251918087(	0.08120:	0.75639:	-0.133971606	0.18435522351
Inverse v	112	-0.026842212	0.08611(	0.75527(	-0.195630407	0.14194598394
Inverse v	112	0.0227819230(	0.07839:	0.77135(	-0.130873763	0.17643760910
Inverse v	112	-0.021804611	0.07695(	0.77690(	-0.172626614	0.12901739106
Inverse v	112	-0.025898052	0.08884(	0.77067:	-0.200037235	0.14824113010
Inverse v	112	0.02344196038	0.08101:	0.77230(	-0.135345463	0.18222938360
Inverse v	112	-0.024901731	0.08695:	0.77459(	-0.195338026	0.14553456423
Inverse v	112	-0.021259834	0.07523:	0.77750(	-0.168721289	0.12620162202
Inverse v	112	-0.023297635	0.08045:	0.77213:	-0.180984093	0.13438882233
Inverse v	112	-0.023766497	0.07950:	0.76499:	-0.179598072	0.13206507829
Inverse v	112	-0.024786379	0.08270:	0.76441(	-0.186893725	0.13732096663
Inverse v	112	0.02255194775	0.07865:	0.77432:	-0.131608791	0.17671268618

Inverse v	112	-0.022424607	0.07836	0.7747	-0.176011847	0.1311626325
Inverse v	112	-0.024114931	0.08056	0.76468	-0.182019032	0.1337891709
Inverse v	112	-0.025538293	0.08533	0.76474	-0.192800317	0.1417237302
Inverse v	112	-0.023462201	0.08085	0.77169	-0.181944149	0.1350197457
Inverse v	112	-0.023294334	0.07857	0.76688	-0.177307553	0.13071888401
Inverse v	112	-0.023437692	0.08019	0.77009	-0.180626058	0.1337506743
Inverse v	112	0.0197060072	0.06482	0.76114	-0.107356948	0.1467689629
Inverse v	112	0.0238496083	0.08385	0.77609	-0.140510857	0.1882100736
Inverse v	112	-0.023731333	0.08073	0.76878	-0.181962432	0.1344997659
Inverse v	112	0.0245894915	0.08010	0.75887	-0.132422161	0.1816011445
Inverse v	112	-0.023625973	0.07900	0.76491	-0.178479008	0.1312270618
Inverse v	112	-0.022953946	0.07838	0.76964	-0.176583916	0.13067602481
Inverse v	112	0.0223424933	0.07332	0.76057	-0.12136471	0.1660496969
Inverse v	112	-0.023071784	0.08185	0.77804	-0.183501569	0.1373580006
Inverse v	112	-0.026647044	0.09365	0.77601	-0.210219575	0.1569254876
Inverse v	112	0.0243772241	0.08817	0.78219	-0.148446232	0.1972006804
Inverse v	112	0.0214737600	0.07752	0.78178	-0.130475292	0.1734228120
Inverse v	112	0.0252943907	0.09170	0.78269	-0.15445712	0.20504590121
Inverse v	112	0.0227453887	0.08156	0.78035	-0.137124335	0.1826151128
Inverse v	112	0.0223669042	0.08166	0.78417	-0.137701082	0.1824348900
Inverse v	112	-0.021408054	0.08028	0.78974	-0.178769252	0.1359531443
Inverse v	112	-0.021997183	0.08416	0.79382	-0.186969289	0.14297492231
Inverse v	112	0.0216002464	0.08259	0.79369	-0.140286535	0.1834870278
Inverse v	112	0.0212006235	0.08116	0.79392	-0.137876335	0.1802775815
Inverse v	112	0.0211868588	0.08011	0.79142	-0.135835466	0.1782091836
Inverse v	112	-0.021664756	0.08061	0.78812	-0.179664987	0.1363354740
Inverse v	112	0.0205497025	0.07713	0.78992	-0.130634702	0.1717341068
Inverse v	112	0.0193315094	0.07373	0.79318	-0.125186904	0.1638499231
Inverse v	112	-0.020011413	0.07677	0.79435	-0.170487728	0.1304649011
Inverse v	112	-0.022525782	0.08565	0.79255	-0.190405031	0.1453534675
Inverse v	112	0.0196504320	0.07351	0.78925	-0.124448214	0.1637490784
Inverse v	112	0.0228798172	0.08960	0.79846	-0.152753116	0.1985127505
Inverse v	112	-0.021392498	0.08446	0.80006	-0.186945227	0.1441602316
Inverse v	112	0.0219106849	0.08644	0.79991	-0.147523415	0.1913447850
Inverse v	112	-0.02172997	0.08481	0.79780	-0.187973954	0.1445140139
Inverse v	112	0.0203973330	0.08018	0.79920	-0.136768471	0.17756313691
Inverse v	112	-0.021857831	0.08714	0.80194	-0.192653263	0.1489376014
Inverse v	112	-0.021435044	0.08929	0.81029	-0.19645543	0.1535853421

Inverse	112	-0.020091051	0.08403	0.81104	-0.18480391	0.1446218081
Inverse	112	-0.019398737	0.08037	0.80927	-0.176927887	0.1381304129
Inverse	112	-0.019544787	0.07941	0.80560	-0.175202388	0.1361128144
Inverse	112	-0.020658861	0.08697	0.81223	-0.191122022	0.1498042998
Inverse	112	0.0188163026	0.07909	0.81197	-0.136217371	0.1738499762
Inverse	112	-0.020049793	0.08234	0.80763	-0.181451626	0.1413520397
Inverse	112	-0.019427854	0.08110	0.81067	-0.17838636	0.1395306515
Inverse	112	0.0211471236	0.08525	0.80409	-0.145952472	0.1882467189
Inverse	112	-0.019720184	0.08223	0.81047	-0.18089316	0.1414527912
Inverse	112	0.0196137886	0.08227	0.81157	-0.141640791	0.1808683677
Inverse	112	0.0182260436	0.07610	0.81071	-0.13093107	0.1673831574
Inverse	112	-0.018528585	0.07864	0.81374	-0.172669109	0.1356119395
Inverse	112	0.0207034626	0.08813	0.81427	-0.152032235	0.1934391600
Inverse	112	-0.018083573	0.07789	0.81642	-0.170759395	0.1345922492
Inverse	112	-0.018383204	0.07925	0.81657	-0.173725524	0.1369591169
Inverse	112	0.0188048147	0.08273	0.82019	-0.143350184	0.1809598134
Inverse	112	-0.019079913	0.08415	0.82064	-0.184030619	0.1458707926
Inverse	112	0.0183598440	0.08322	0.82540	-0.144766007	0.1814856949
Inverse	112	0.0187453469	0.08460	0.82464	-0.147070804	0.1845614979
Inverse	112	-0.020528763	0.09234	0.82407	-0.201521414	0.1604638885
Inverse	112	0.0180807129	0.08182	0.82512	-0.142301407	0.1784628331
Inverse	112	0.0185374300	0.08478	0.82692	-0.147632608	0.1847074679
Inverse	112	-0.017560482	0.08080	0.82796	-0.175945095	0.1408241303
Inverse	112	0.0174032538	0.08119	0.83029	-0.141746309	0.1765528163
Inverse	112	0.0163241923	0.07711	0.83234	-0.134812084	0.1674604686
Inverse	112	-0.017536259	0.08282	0.83232	-0.179877179	0.1448046601
Inverse	112	-0.018125369	0.08607	0.83321	-0.186825672	0.1505749339
Inverse	112	0.0212664766	0.10159	0.83418	-0.177850272	0.2203832255
Inverse	112	-0.016489777	0.08061	0.83792	-0.174500349	0.1415207951
Inverse	112	-0.016150211	0.07888	0.83777	-0.170755872	0.1384554497
Inverse	112	-0.015535318	0.07521	0.83636	-0.162958197	0.1318875603
Inverse	112	-0.017784675	0.08654	0.83717	-0.187406674	0.1518373242
Inverse	112	-0.016508816	0.08138	0.83925	-0.176022027	0.1430043947
Inverse	112	0.0156117088	0.07745	0.84025	-0.136195746	0.1674191634
Inverse	112	0.0167877399	0.08526	0.84392	-0.150339265	0.1839147450
Inverse	112	0.0155462936	0.07867	0.84334	-0.138648501	0.1697410885
Inverse	112	-0.016751449	0.08455	0.84296	-0.182485412	0.1489825136
Inverse	112	-0.015502128	0.07997	0.84629	-0.172248156	0.1412439004

Inverse v	112	-0.01673624	0.08605(	0.84579(	-0.185395804	0.1519233245(
Inverse v	112	-0.014946046	0.07920	0.85033	-0.170193246	0.1403011531
Inverse v	112	0.0143123776	0.07731	0.85313	-0.137219391	0.1658441468
Inverse v	112	0.0149506771	0.08048	0.85263	-0.142804787	0.1727061415
Inverse v	112	0.0144664608(	0.07865	0.85408	-0.139706466	0.1686393876
Inverse v	112	0.0146131287	0.08001	0.85508	-0.142215719	0.1714419767
Inverse v	112	0.0150441589	0.08317	0.85646(	-0.147978334	0.1780666514
Inverse v	112	0.0148332623	0.08858(	0.86702(	-0.158795311	0.1884618360
Inverse v	112	0.0127869115	0.07767(	0.86923	-0.139447609	0.1650214324
Inverse v	112	-0.013285454	0.07982	0.86782	-0.169750019	0.1431791108
Inverse v	112	-0.01477993	0.08523	0.86233	-0.181840778	0.1522809173(
Inverse v	112	0.0138500482(	0.07921	0.86119	-0.141404327	0.1691044238
Inverse v	112	0.0130888592	0.07964	0.86947	-0.143024941	0.1692026599
Inverse v	112	0.0139628888(	0.08369	0.86750	-0.150079768	0.1780055461
Inverse v	112	-0.013599163	0.08257	0.86919(	-0.175455529	0.1482572015
Inverse v	112	0.0143095483	0.08419	0.86504	-0.150720102	0.1793391989
Inverse v	112	0.0142537101	0.08273	0.86321	-0.147903471	0.1764108911
Inverse v	112	-0.013983347	0.08079	0.86259	-0.172341447	0.1443747527
Inverse v	112	0.0131100941	0.07847	0.86733(	-0.140710599	0.1669307877
Inverse v	112	-0.013814232	0.07932	0.86174	-0.169284089	0.1416556260
Inverse v	112	-0.013413885	0.07564	0.85924	-0.161674793	0.1348470226(
Inverse v	112	0.0132030075	0.08115(	0.87075(	-0.145851921	0.1722579363
Inverse v	112	-0.013228642	0.08248	0.87257	-0.17489249	0.1484352063
Inverse v	112	-0.012850074	0.08219	0.87577	-0.173961597	0.1482614498
Inverse v	112	0.0127092140	0.08149	0.87606	-0.147013147	0.1724315754
Inverse v	112	0.0090628541	0.08383(	0.91391	-0.155256395	0.1733821036
Inverse v	112	0.0086565974	0.07954(	0.91333	-0.147243479	0.1645566738
Inverse v	112	0.0089956780	0.07059(	0.89860	-0.129373759	0.1473651147
Inverse v	112	0.0084308663	0.08258	0.91869	-0.153444507	0.1703062394
Inverse v	112	0.0091137813	0.07968	0.90894	-0.147073827	0.1653013896(
Inverse v	112	0.0089720692	0.07905	0.90963	-0.145970053	0.1639141917
Inverse v	112	-0.011347981	0.08228	0.89031	-0.172627549	0.1499315883
Inverse v	112	-0.012481619	0.08977	0.88942	-0.188446133	0.1634828945(
Inverse v	112	0.0078411275(	0.07693	0.91882(	-0.142950431	0.1586326859
Inverse v	112	0.0110658961	0.07995	0.88992	-0.145644124	0.1677759165
Inverse v	112	0.0098409548	0.07799	0.89959	-0.143037454	0.1627193639
Inverse v	112	-0.010551217	0.07181	0.88319	-0.1513136	0.1302111658
Inverse v	112	0.0128331270(	0.08749	0.88338	-0.158652313	0.1843185674

Inverse [illegible]	112	-0.009211677	0.08017[illegible]	0.90852[illegible]	-0.166351626	0.1479282726[illegible]
Inverse [illegible]	112	-0.010150574	0.08143[illegible]	0.90080[illegible]	-0.169759718	0.1494585697[illegible]
Inverse [illegible]	112	0.0083204489[illegible]	0.07770[illegible]	0.91472[illegible]	-0.14398191	0.1606228078[illegible]
Inverse [illegible]	112	0.0118221701[illegible]	0.08827[illegible]	0.89346[illegible]	-0.16119963	0.1848439703[illegible]
Inverse [illegible]	112	-0.008729922	0.08648[illegible]	0.91959[illegible]	-0.17824205	0.1607822061[illegible]
Inverse [illegible]	112	0.0124587699[illegible]	0.08333[illegible]	0.88115[illegible]	-0.15087719	0.1757947295[illegible]
Inverse [illegible]	112	0.0113955438[illegible]	0.07960[illegible]	0.88617[illegible]	-0.144630128	0.1674212154[illegible]
Inverse [illegible]	112	-0.011584365	0.08477[illegible]	0.89131[illegible]	-0.177749809	0.1545810787[illegible]
Inverse [illegible]	112	-0.009807682	0.08746[illegible]	0.91072[illegible]	-0.181244279	0.1616289150[illegible]
Inverse [illegible]	112	0.0134155594[illegible]	0.11615[illegible]	0.90805[illegible]	-0.214255393	0.2410865124[illegible]
Inverse [illegible]	112	0.0097387769[illegible]	0.08013[illegible]	0.90327[illegible]	-0.147326719	0.1668042727[illegible]
Inverse [illegible]	112	0.0110408558[illegible]	0.07958[illegible]	0.88966[illegible]	-0.144949791	0.1670315029[illegible]
Inverse [illegible]	112	0.0087826967[illegible]	0.08536[illegible]	0.91805[illegible]	-0.158541715	0.1761071082[illegible]
Inverse [illegible]	112	-0.009117115	0.08069[illegible]	0.91004[illegible]	-0.167279604	0.1490453740[illegible]
Inverse [illegible]	112	-0.009029516	0.07970[illegible]	0.90979[illegible]	-0.165242731	0.1471836985[illegible]
Inverse [illegible]	112	0.0085389425[illegible]	0.07746[illegible]	0.91222[illegible]	-0.143286257	0.1603641423[illegible]
Inverse [illegible]	112	0.0099632990[illegible]	0.09295[illegible]	0.91464[illegible]	-0.172230723	0.1921573214[illegible]
Inverse [illegible]	112	0.0098415243[illegible]	0.08917[illegible]	0.91212[illegible]	-0.164950179	0.1846332273[illegible]
Inverse [illegible]	112	0.0089217611[illegible]	0.07801[illegible]	0.90894[illegible]	-0.143982406	0.1618259282[illegible]
Inverse [illegible]	112	-0.009213659	0.08278[illegible]	0.91138[illegible]	-0.171479629	0.1530523105[illegible]
Inverse [illegible]	112	0.0108484018[illegible]	0.09650[illegible]	0.91049[illegible]	-0.178307156	0.2000039602[illegible]
Inverse [illegible]	112	-0.009286171	0.08166[illegible]	0.90946[illegible]	-0.169351886	0.1507795453[illegible]
Inverse [illegible]	112	-0.011836127	0.07903[illegible]	0.88095[illegible]	-0.166741776	0.1430695220[illegible]
Inverse [illegible]	112	0.0126147952[illegible]	0.08273[illegible]	0.87881[illegible]	-0.149542651	0.1747722419[illegible]
Inverse [illegible]	112	0.0096093773[illegible]	0.08541[illegible]	0.91042[illegible]	-0.157806746	0.1770255009[illegible]
Inverse [illegible]	112	-0.008412349	0.08205[illegible]	0.91833[illegible]	-0.169231837	0.1524071379[illegible]
Inverse [illegible]	112	-0.008390565	0.07947[illegible]	0.91591[illegible]	-0.164155358	0.1473742272[illegible]
Inverse [illegible]	112	-0.00967428	0.08164[illegible]	0.90567[illegible]	-0.169701251	0.1503526899[illegible]
Inverse [illegible]	112	0.0101337957[illegible]	0.07983[illegible]	0.89899[illegible]	-0.146344431	0.1666120229[illegible]
Inverse [illegible]	112	-0.010012427	0.08239[illegible]	0.90327[illegible]	-0.171500202	0.1514753466[illegible]
Inverse [illegible]	112	0.0092237455[illegible]	0.07975[illegible]	0.90792[illegible]	-0.147088372	0.1655358629[illegible]
Inverse [illegible]	112	-0.008832701	0.08177[illegible]	0.91398[illegible]	-0.16910813	0.1514427275[illegible]
Inverse [illegible]	112	-0.008076904	0.07968[illegible]	0.91926[illegible]	-0.164267929	0.1481141204[illegible]
Inverse [illegible]	112	0.0090534246[illegible]	0.08440[illegible]	0.91458[illegible]	-0.156381602	0.1744884516[illegible]
Inverse [illegible]	112	-0.0120297	0.07926[illegible]	0.87937[illegible]	-0.167392065	0.1433326653[illegible]
Inverse [illegible]	112	-0.007926144	0.07649[illegible]	0.91746[illegible]	-0.157847185	0.1419948972[illegible]
Inverse [illegible]	112	0.0102411982[illegible]	0.07654[illegible]	0.89355[illegible]	-0.139777905	0.1602603017[illegible]
Inverse [illegible]	112	0.0047296149[illegible]	0.07958[illegible]	0.95261[illegible]	-0.151254617	0.1607138463[illegible]

Inverse v	112	0.0025364182	0.08541	0.97631	-0.164883987	0.1699568231
Inverse v	112	0.0059480193	0.08211	0.94225	-0.155003634	0.1668996723
Inverse v	112	0.0073227632	0.08182	0.92868	-0.153049462	0.1676949885
Inverse v	112	0.0050304938	0.07987	0.94978	-0.151529811	0.1615907990
Inverse v	112	-0.003963532	0.07674	0.95881	-0.154393432	0.1464663676
Inverse v	112	0.0022580651	0.07976	0.97741	-0.154086045	0.1586021755
Inverse v	112	0.0021781783	0.07965	0.97818	-0.153945117	0.1583014737
Inverse v	112	-0.002943095	0.08414	0.97209	-0.167867027	0.1619808380
Inverse v	112	0.0049725408	0.07986	0.95035	-0.15155886	0.1615039419
Inverse v	112	-0.006130857	0.07776	0.93716	-0.1585588	0.1462970864
Inverse v	112	0.0072147136	0.07925	0.92747	-0.148134469	0.1625638964
Inverse v	112	0.0080922979	0.09074	0.92894	-0.169766302	0.1859508978
Inverse v	112	-0.004185238	0.08562	0.96101	-0.172008865	0.1636383899
Inverse v	112	0.0043970471	0.09312	0.96233	-0.178122353	0.1869164470
Inverse v	112	0.0079804697	0.08510	0.92529	-0.158828953	0.1747898927
Inverse v	112	-0.006236094	0.08005	0.93790	-0.163140877	0.1506686880
Inverse v	112	-0.003033955	0.08391	0.97115	-0.167497838	0.1614299289
Inverse v	112	-0.002858249	0.08532	0.97327	-0.170089442	0.164372945
Inverse v	112	-0.004135555	0.08348	0.96049	-0.16776133	0.1594902199
Inverse v	112	-0.002919171	0.07928	0.97062	-0.158309552	0.1524712088
Inverse v	112	-0.005030068	0.08189	0.95102	-0.165540478	0.1554803425
Inverse v	112	-0.003331682	0.08165	0.96745	-0.163380049	0.1567166845
Inverse v	112	0.0031661964	0.08659	0.97083	-0.166552646	0.1728850387
Inverse v	112	-0.003223522	0.08158	0.96848	-0.163130212	0.1566831683
Inverse v	112	0.0071260157	0.07980	0.92885	-0.149297993	0.1635500248
Inverse v	112	-0.004758277	0.08042	0.95281	-0.162385719	0.1528691646
Inverse v	112	0.0067100266	0.08526	0.93727	-0.160407001	0.1738270541
Inverse v	112	-0.004050724	0.08383	0.96146	-0.168370434	0.1602689849
Inverse v	112	-0.003912435	0.08309	0.96244	-0.166770183	0.1589453127
Inverse v	112	-0.007320385	0.08548	0.93175	-0.174865494	0.1602247238
Inverse v	112	0.0036226084	0.07953	0.96367	-0.152275612	0.1595208294
Inverse v	112	-0.003047559	0.07947	0.96941	-0.158827672	0.1527325535
Inverse v	112	-0.005015839	0.08311	0.95187	-0.167923898	0.1578922198
Inverse v	112	0.0023871934	0.08141	0.97660	-0.157191328	0.1619657153
Inverse v	112	0.0055103883	0.08150	0.94609	-0.15423687	0.1652576471
Inverse v	112	-0.006542596	0.07942	0.93434	-0.162211921	0.1491267291
Inverse v	112	-0.00665717	0.07976	0.93348	-0.16299088	0.1496765404
Inverse v	112	0.0048483708	0.07971	0.95149	-0.151385791	0.1610825327

Inverse	112	-0.006475736	0.08030	0.93572	-0.163871763	0.150920292
Inverse	112	-0.006418217	0.08321	0.93852	-0.169520169	0.156683735
Inverse	112	0.002140486	0.07848	0.97824	-0.151695805	0.155976779
Inverse	112	-0.004446151	0.07946	0.95537	-0.16018786	0.151295557
Inverse	112	-0.003088513	0.08401	0.97067	-0.167756668	0.161579643
Inverse	112	-0.005872481	0.08109	0.94226	-0.164810174	0.153065212
Inverse	112	0.007221277	0.07672	0.92501	-0.14315904	0.157601594
Inverse	112	-0.005038811	0.07995	0.94974	-0.161742012	0.151664389
Inverse	112	-0.003269297	0.07863	0.96683	-0.15739073	0.150852135
Inverse	112	-0.006250875	0.08838	0.94361	-0.17949051	0.166988759
Inverse	112	-0.002462824	0.08181	0.97598	-0.162815055	0.157889407
Inverse	112	-0.007715541	0.08371	0.92657	-0.171803537	0.156372455
Inverse	112	-0.003591903	0.08003	0.96420	-0.160455936	0.153272129
Inverse	112	-0.006017253	0.08226	0.94169	-0.167255134	0.155220627
Inverse	112	0.003999054	0.08089	0.96057	-0.154557821	0.162555930
Inverse	112	-0.006403689	0.07820	0.93474	-0.159693251	0.146885873
Inverse	112	-0.003403266	0.08405	0.96770	-0.168160802	0.161354270
Inverse	112	0.003975354	0.08604	0.96315	-0.16467724	0.172627948
Inverse	112	-0.00337844	0.07949	0.96610	-0.159194258	0.152437378
Inverse	112	-0.005320585	0.08529	0.95026	-0.172497035	0.161855864
Inverse	112	0.005398106	0.08004	0.94623	-0.151492903	0.162289115
Inverse	112	0.003292353	0.05712	0.95403	-0.108670953	0.115255659
Inverse	112	0.002681356	0.07916	0.97298	-0.152483335	0.157846049
Inverse	112	0.007653304	0.08605	0.92913	-0.161020195	0.176326803
Inverse	112	-0.007039051	0.07988	0.92978	-0.163614369	0.149536267
Inverse	112	-0.006981253	0.07973	0.93022	-0.163254041	0.149291535
Inverse	112	0.002851652	0.07951	0.97139	-0.15300026	0.158703564
Inverse	112	-0.003992183	0.08852	0.96402	-0.177494549	0.169510183
Inverse	112	0.001924501	0.07938	0.98065	-0.153662378	0.157511381
Inverse	112	-0.001958905	0.08328	0.98123	-0.165203581	0.161285770
Inverse	112	-0.001789121	0.08175	0.98253	-0.162021991	0.158443749
Inverse	112	-0.0017432	0.07787	0.98214	-0.154384716	0.150898315
Inverse	112	0.001484057	0.08013	0.98522	-0.155577905	0.158546020
Inverse	112	0.001529812	0.07817	0.98438	-0.151688443	0.154748067
Inverse	112	0.001626496	0.08634	0.98497	-0.167605477	0.170858470
Inverse	112	-0.001268677	0.09024	0.98878	-0.178154898	0.175617544
Inverse	112	-0.001174959	0.08681	0.98920	-0.171328002	0.168978084
Inverse	112	-0.001037775	0.08235	0.98994	-0.162448345	0.160372796

Inverse v	112	0.0008419222(	0.08240(	0.99184(	-0.160674599	0.16235844346
Inverse v	112	-0.000744698	0.08018(	0.99259(	-0.157916944	0.15642754713
Inverse v	112	0.00047032601	0.07839	0.99521	-0.153189648	0.15413029981
Inverse v	112	-0.000383769	0.08207(	0.99626(	-0.161258747	0.16049120859
Inverse v	112	6.37087050498	0.07896	0.99935(	-0.15470802	0.15483543777

d circulating protein levels(UKB-PPP unique)

or	or_lci95	or_uci95	exposure	fdr
2.060064441	1.78423554	2.37853434	ALPP_P	7.48722913703267e-20
1.95864471	1.69319980	2.26570374	LAMP3_	8.35773508200002e-17
1.56046921	1.36437231	1.78475049	SFTPA2	3.17193393951066e-08
1.73155071	1.45348946	2.06280675	NPC2_P	2.25304859805508e-07
1.79461870	1.48578507	2.16764615	CEACA	2.93578093488725e-07
0.64216219	0.55536996	0.74251817	DPP6_P	3.74130451555852e-07
1.69826529	1.42746234	2.02044209	IFI30_P	3.74130451555852e-07
1.61036184	1.37270515	1.88916408	GCHFR_	7.08775696487535e-07
1.59673570	1.35270590	1.88478879	SHISA5_	4.06282115173733e-06
1.49207333	1.29365531	1.72092479	HEG1_C	4.42725981306093e-06
1.60038150	1.35090734	1.89592645	OCLN_(	5.57650648902201e-06
1.45278310	1.25823460	1.67741273	PRAP1_	3.38237382320474e-05
1.46089323	1.26002104	1.69378841	ORM1_I	4.48312019719228e-05
1.44555888	1.25013229	1.67153549	CSTB_P	5.39363644687555e-05
1.47648373	1.26394934	1.72475599	CEACA	6.8008097233809e-05
1.33007748	1.18471142	1.49328020	VNN1_(	9.73223675715795e-05
1.52653072	1.27863512	1.82248713	FGF21_	0.000194167274001874
1.39449404	1.21092583	1.60588996	RBFOX	0.000246473450797958
1.55650048	1.28157758	1.89039960	MZB1_(	0.000488463225107264
1.40786048	1.21047497	1.63743255	CES2_O	0.000517916471852363
0.65131377	0.53659192	0.79056283	TP53BP	0.000784924918641005
1.42617282	1.21076034	1.67991041	IGLC2_	0.0011146111025054
1.45505759	1.22222367	1.73224643	PLA2G1	0.0012379996320352
0.72342032	0.62185080	0.84157962	DCC_P	0.00130271195958413
1.40059680	1.19560443	1.64073614	RIDA_P	0.00137601464364833
1.39901419	1.19306902	1.64050921	HEPH_C	0.00157533734306762
1.36212959	1.17485713	1.57925333	SLURP1	0.00178328475938184
1.39504792	1.18721363	1.63926581	LTBP3_	0.00213577908865304
0.72646918	0.62145312	0.84923134	CDH23_	0.00236543139841397
1.39367330	1.18456210	1.63969899	GAST_I	0.00236543139841397
1.20021404	1.09746997	1.31257691	PSCA_C	0.00236543139841397
1.32130351	1.15062679	1.51729734	GPR37_	0.00281171413811099
1.38853683	1.17782006	1.63695168	SFTPA1	0.00320946305703598
1.33938369	1.15603281	1.55181467	HNMT_	0.00336405707016159
1.39529703	1.17735419	1.65358380	BPIFB2_	0.00383999023470328
1.33080562	1.15063142	1.53919280	CTBS_C	0.00383999023470328

1.3667201312	1.1621607281	1.6072853538	IMMT_(	0.00475636974666044
1.2384943383	1.1081990404	1.3841089642	SGSH_F	0.00475636974666044
1.3177924563	1.1420908496	1.5205243597	TNFRSF	0.00475636974666044
1.3577625770	1.1566727296	1.5938122931	YAP1_P	0.00526414204196182
1.3422707165	1.1491732634	1.5678146490	RBP2_P	0.00567397002136741
1.3615120125	1.1562840531	1.6031657231	EFNA1_	0.00582147783684928
1.3987156584	1.1706656614	1.6711906376	AHNAK	0.00583927027131313
1.3198709710	1.1388555121	1.5296579431	TNFRSF	0.00587943855367976
1.3947583875	1.1672807427	1.6665664808	ADAM8	0.00631346130093052
1.2970881549	1.1283422790	1.4910703188	PTPRB_	0.00631346130093052
1.4603574696	1.1869563442	1.7967332576	MERTK	0.00833728082517564
0.7541001293	0.6456289378	0.8807954102	RAP1A_	0.00876575404042257
1.2934889804	1.1223391998	1.4907380431	CPXM2_	0.00885524816356536
1.2313799610	1.0963980061	1.3829800857	TREH_(	0.0101064611458212
1.3017295039	1.1218135208	1.5105003371	GPR15L	0.0114653920943994
1.3681360196	1.1460314603	1.6332851521	VSIG4_(	0.0115196205495257
1.3457342711	1.1361920938	1.5939212553	SIGLEC	0.0126120996433049
1.3069101711	1.1209247165	1.5237546021	GGT1_F	0.0133776284432245
1.3552784831	1.1373843208	1.6149156738	IGSF9_(	0.0140285807783552
1.3261697030	1.1254802226	1.5626450344	PPL_O6	0.0147051451280681
1.3591236507	1.1371796265	1.6243846221	SCGB3A	0.0147051451280681
0.7610001949	0.6495739140	0.8915402608	SYT1_P	0.0147051451280681
1.3006190336	1.1154685157	1.5165016733	NT5C1A	0.0151967429202869
0.7839023766	0.6797289706	0.9040411143	RAB6A_	0.0151967429202869
1.3018364949	1.1158023077	1.5188875734	TCTN3_	0.0151967429202869
1.3484426489	1.1317377324	1.6066421799	TGFA_F	0.0151967429202869
0.7880966754	0.6848305978	0.9069343160	CLUL1_	0.0161444305493842
1.2478008000	1.0943591272	1.4227567513	VSTM1_	0.0168478742067048
1.2916827668	1.1078456303	1.5060260424	ITGBL1	0.0190799874943192
0.7578107047	0.6414474876	0.8952830516	PON3_C	0.0192587325260258
1.3102572562	1.1124466932	1.5432416565	FGA_P0	0.0206665524656863
0.7764033039	0.6652502078	0.9061283757	LUZP2_	0.0219534539260502
1.2877918543	1.1035437627	1.5028020782	SSC5D_	0.0219534539260502
1.2954559800	1.1052259618	1.5184281350	ADGRG	0.0228504980766564
1.3262402582	1.1147137140	1.5779057890	CD302_	0.0230753001263614
1.2518934320	1.0901926438	1.4375781876	QSOX1_	0.0230753001263614
1.3040198943	1.1071871256	1.5358450666	PAMR1_	0.0230863950315088
1.2685854796	1.0941990426	1.4707645103	SMAD5_	0.0249472378881366

0.78798056	0.6792147533	0.9141635460	RTBDN_	0.0253778198156116
0.7321359507	0.6024509931	0.8897371842	CA14_Q	0.0255516849481876
1.2922802917	1.1009756907	1.5168258178	MFAP3_	0.0255516849481876
1.2408947464	1.0838529150	1.4206907141	C8B_P0	0.0259332542557231
0.7805115315	0.6669301717	0.9134363336	B4GAT1	0.0281561660772226
1.3410237123	1.1129600318	1.6158213643	CLEC14	0.0281561660772226
1.2207624097	1.0759138967	1.3851116391	ELN_P1	0.0281561660772226
1.2736639476	1.0921480346	1.4853479565	MYL1_I	0.0281561660772226
1.2836714803	1.0956652833	1.5039378306	TCOF1_	0.0281561660772226
1.2683136104	1.0897163580	1.4761817627	ARHGE	0.0291705623194375
1.2601209341	1.0865734169	1.4613874625	FAM3C_	0.0295257332043109
0.7723075177	0.6543816392	0.9114847762	KIAA03	0.0295257332043109
0.7814573440	0.6671232896	0.9153863912	TXNL1_	0.0295257332043109
1.2302305725	1.0765872811	1.4058008005	HLA-DI	0.0303008686855863
1.2827910499	1.0925334330	1.5061807977	CRELD2	0.0303350078911124
1.2242452744	1.0743881799	1.3950046361	CD101_	0.0303419465349379
0.7939681123	0.6837892431	0.9219000880	VGF_O1	0.0310364498444823
1.2932708688	1.0943212591	1.5283898821	PALM2_	0.0316533296162793
1.2595601208	1.0831240775	1.4647368024	CDHR2_	0.033329401872923
0.7943721819	0.6832016816	0.9236323335	EXTL1_	0.033329401872923
1.3099444603	1.0976068414	1.5633598700	PGA4_P	0.033329401872923
0.7962316952	0.6849591977	0.9255805523	ODAM_	0.0358121710300914
1.2617375365	1.0809198171	1.4728026868	B3GNT7	0.0379323226652212
1.2347414764	1.0724601600	1.4215786939	FNDC1_	0.0387493987674375
1.2860678327	1.0872176181	1.5212874062	GOLM2	0.0387493987674375
0.7810109369	0.6610975079	0.9226749101	TRAF3_	0.0418023467244373
1.2421893836	1.0707544912	1.4410721387	CCN3_P	0.0471393429770065
1.2666188943	1.0775046831	1.4889247802	SUSD2_	0.0471393429770065
1.2370130386	1.0682119493	1.4324884294	ADGRD	0.0493435987242748
1.2822207763	1.0802105026	1.5220090115	FUOM_	0.0493435987242748
1.2284708085	1.0656561950	1.4161607977	LY6D_C	0.0496315210426097
1.2661393818	1.0742976232	1.4922391147	NECTIN	0.0526101317241956
1.2861744555	1.0782972300	1.5341268474	POLR2F	0.0547697538718063
1.2659517405	1.0730519034	1.4935286952	WFDC1	0.0547697538718063
1.2611342209	1.0672392024	1.4902559046	SCLY_C	0.0676229177128444
1.2261548499	1.0584509587	1.4204302086	DTNB_C	0.0684466204152492
0.8232486445	0.7145306844	0.9485083643	LPL_P0	0.0732296247103831
1.2342059448	1.0575954746	1.4403090320	NFYA_I	0.0765602588238658

0.790551936(	0.665424254!	0.93920887l!	TFAP2A	0.0765602588238658
1.218494708?	1.052824534!	1.41023438O4	PINLYP	0.0806092658966988
0.798514389l	0.675193530?	0.944359211l	PNPT1_	0.0851446317703955
1.230665280(	1.053667349(	1.437395809(	S100G_l	0.0860358983412029
1.241230882?	1.055912342!	1.459073865!	SDC1_P	0.0860358983412029
0.811783914(	0.694376921!	0.949042376?	RNF168	0.0861256522199918
1.271971766?	1.061735231?	1.523837701!	LIPF_P(	0.0869921078505689
0.817851313(	0.703078992(	0.951359346?	GP2_P5	0.08717126754093
1.227469436(	1.051226484(	1.433260328l	NECTIN	0.0902061265572741
1.224816605?	1.050046411?	1.428675628?	MEGF9_	0.092109120295175
1.223726476(	1.04946806	1.426919546(	PAG1_(	0.0928682321719889
1.233502177(	1.050431540l	1.448478614!	COL24A	0.0947222101756011
1.240163729?	1.051298309?	1.462958763!	ELOA_(	0.0947222101756011
1.226807562?	1.048603954(	1.435295746!	EPS8L2_	0.0947222101756011
0.791918126l	0.662467196?	0.946664713?	MCAM_	0.0947222101756011
1.238969224l	1.051124130l	1.460383882!	NEB_P2	0.0947222101756011
0.819361860?	0.703380494?	0.954467552?	SUSD5_	0.0947222101756011
0.818152698l	0.700050587(	0.956179238!	KCNC4_	0.101418072306813
1.237988472(	1.048958446?	1.461083097!	UPB1_(	0.101418072306813
0.819500557(	0.701721345!	0.957048217?	WASL_(	0.103195887669734
1.224031463?	1.044661331?	1.434199752!	CES3_Q	0.105017543436089
1.223357442!	1.044661366(	1.432620638!	CHCHD	0.105017543436089
0.819450876(	0.701143535(	0.957720788?	RANBP	0.105017543436089
1.216687540l	1.042672533!	1.419744476l	HLA-E_	0.107164898803517
1.230271874?	1.044744671?	1.448745255!	ANGPT	0.10770846046272
0.809393493?	0.684988167(	0.956392910?	PRRT3_	0.10770846046272
1.242520735!	1.046417181?	1.475375028?	SEMA3l	0.108319153821646
1.195580148?	1.037889149?	1.377229824?	SSC4D_	0.108319153821646
1.219866415l	1.042152768(	1.427884775(	TAFA5_	0.108319153821646
1.206792016l	1.038625021?	1.402187450(	CCN5_(	0.113410618178082
0.820714180l	0.700760183?	0.961201537!	IMPG1_	0.11391441323927
1.213852867?	1.039286254?	1.417741049l	TEX33_	0.114518651411365
1.228653516?	1.041050269!	1.450063946!	CLEC5A	0.116762280221507
0.813278741(	0.688608256(	0.960520447?	RANGA	0.116762280221507
1.209799767?	1.036996227l	1.411399037?	LAMP2_	0.120024210947581
1.213317172?	1.035631449?	1.421488853?	SEPTIN	0.128931493565422
1.222432396!	1.036034272!	1.442366341?	HYOU1_	0.132186111430477
1.206717104?	1.033665851(	1.408739747?	LEO1_(	0.132186111430477

1.20774135433	1.03364596472	1.41115936068	LY96_Q	0.132232417435603
0.81020404022	0.67989937311	0.96548197100	DMP1_(	0.140163108839926
1.20231518311	1.03014989688	1.40325384100	SCRIB_	0.145329947144814
0.83167109288	0.71222739777	0.97114602577	SIRT1_(	0.146949656605924
0.83176385422	0.71214151877	0.97147981277	TMED1_	0.147918203410189
0.83090665844	0.71055807322	0.97163891455	PDE1C_	0.14792782263822
1.19496379077	1.02808547944	1.38892970455	SORBS1	0.14792782263822
1.20808355555	1.02870269055	1.41874410422	ZHX2_(	0.153095420574038
0.78339627600	0.63589523200	0.96511138211	ITGAM_	0.156785431480231
1.18479297577	1.02477876899	1.36979262055	SPRR3_	0.157045805856997
1.24509870300	1.03138954522	1.50308948499	CD22_P	0.158801964782138
1.19778533733	1.02586794255	1.39851305899	CEMIP2	0.158801964782138
1.19327346522	1.02462095844	1.38968615700	KHK_P5	0.158842177013662
1.19357984144	1.02484469700	1.39009631511	LETM1_	0.158842177013662
1.21023792077	1.02657171300	1.42676425444	MYBPC	0.158842177013662
1.16024061933	1.02079364799	1.31873694299	SNCG_(	0.158842177013662
1.20388278100	1.02529553888	1.41357657099	RALY_(	0.160958479102747
1.19465644644	1.02339307977	1.39458049233	PRUNE2	0.16509041331621
1.15338116577	1.01745968955	1.30746026311	F12_P00	0.173884961226918
1.19803506900	1.02183621144	1.40461652400	WDR46_	0.174842890476177
0.83798208099	0.71664314199	0.97986560811	HTR1B_	0.177890225835556
1.19321036944	1.02067397477	1.39491259799	RIPK4_1	0.177890225835556
1.15337447011	1.01532325299	1.31019620055	VMO1_(	0.186645253671271
1.17907374400	1.01730778133	1.36656272500	MAMD(	0.188371167821567
1.17061104300	1.01624624699	1.34842339611	ADAMT	0.189467569287882
1.20774392511	1.01859418599	1.43201817655	CCN4_(	0.193420989937054
1.19357047222	1.01726112844	1.40043734333	SCRIB_	0.193420989937054
1.21994271600	1.01929774900	1.46008389933	SIL1_Q9	0.193420989937054
1.19222532600	1.01653342922	1.39828281800	C19orf1	0.193543571158879
0.84839244133	0.73097034099	0.98467707122	CELSR2	0.193543571158879
1.18248817944	1.01603739199	1.37620751511	ZBTB17	0.193543571158879
1.19175696288	1.01576401377	1.39824274	SCRG1_	0.196174861577281
1.19439077311	1.01605895044	1.40402219611	SCRIB_	0.196174861577281
1.17253706000	1.01355050677	1.35646240555	MNDA_	0.200443858535952
1.18889058100	1.01457776100	1.39315178044	PHOSPH	0.200443858535952
1.18897954377	1.01372317522	1.39453490833	MPRIP_	0.204006857264387
1.17779989822	1.01306951577	1.36931651099	RASSF2	0.204006857264387
1.19693194055	1.01389659011	1.41301004877	TYRP1_	0.205221722795707

1.178646330	1.012391794	1.372203113	CASP1_	0.20629203527275
1.186731747	1.012577014	1.390839631	MRPL2	0.207453009912808
1.142419434	1.009554058	1.292770956	CD6_P3	0.207870627946814
1.169593078	1.011150728	1.352862565	PON2_C	0.207870627946814
1.171435773	1.010807355	1.357589815	INPP5D	0.209028813716202
0.845527059	0.723197846	0.988548309	PRKAG	0.209028813716202
1.200819104	1.011947046	1.424942666	FDX2_C	0.210540019839709
1.165910775	1.009993555	1.345897635	WNT9A	0.210540019839709
0.847948907	0.726617880	0.989539852	ENOPH	0.210657340915783
0.849046781	0.728253476	0.989875724	SLC4A1	0.211404008025345
0.841022848	0.714366112	0.990135755	SMPD3_	0.214962746058323
1.174690103	1.009271711	1.367220366	TGOLN	0.214962746058323
1.336398442	1.016114145	1.757637963	BTN3A	0.215704211969971
0.846011000	0.722319025	0.990884342	CDKL5_	0.215704211969971
0.847964547	0.725426748	0.991201213	SAG_P1	0.215990245265299
0.830742288	0.696441778	0.990941053	CCNE1_	0.219707629132598
0.840189474	0.711614277	0.991995770	ESR1_P	0.219707629132598
0.848607460	0.725854703	0.992119521	PFDN4_	0.219707629132598
1.189513177	1.007968637	1.403755579	PIK3IP1	0.219707629132598
1.17673076	1.007611384	1.374235440	PREB_C	0.219707629132598
1.173382740	1.007012662	1.367239069	GET3_C	0.220477520113248
1.171064102	1.006701887	1.362261409	MTUS1_	0.220477520113248
0.839806553	0.710555570	0.992568457	SEZ6_Q	0.220477520113248
0.850486220	0.727944790	0.99365614	CELA3	0.221801508644924
1.176477895	1.006467719	1.375205792	FDX1_P	0.221801508644924
0.849054660	0.725173209	0.994098798	MMUT_	0.224308196768705
1.192125248	1.005574360	1.413284451	CBLIF_	0.227859776110217
0.836961727	0.704176324	0.994786260	CRTAP_	0.227859776110217
1.192678435	1.005211966	1.415106362	FOS_P0	0.227859776110217
1.174797241	1.004739962	1.373637567	SCARB	0.227859776110217
1.193975283	1.003799431	1.420181095	SEL1L_	0.23587730591454
1.181630852	1.003078461	1.391966357	THOP1_	0.238209782302505
0.843210044	0.712784395	0.997501045	IGSF21_	0.24143077383089
0.838949810	0.705178489	0.998097354	GRIK2_	0.244748207707185
1.157235071	1.001411502	1.337305401	NOS1_P	0.245016980920988
1.147447016	1.000741015	1.315659731	AOC3_C	0.246535495730759
0.843767183	0.712502303	0.999215100	CHRM1	0.246535495730759
0.855998493	0.733273281	0.999263765	IFT20_C	0.246535495730759

1.15138892l	1.00063902	1.32484983	MEP1A_	0.246535495730759
0.85346916	0.72867398	0.99963719	EPN1_Q	0.246929139262187
0.88157905	0.77742408	0.99968812	PLB1_Q	0.246929139262187
0.85603939	0.73254372	1.00035454	TPBGL_	0.25105911822668
0.84468137	0.71291229	1.00080562	ITGA11	0.252813359282962
1.19246571	0.99857213	1.42400776	ADGRE	0.254448548407951
0.84954612	0.72083799	1.00123555	AP2B1_	0.254448548407951
0.86248477	0.74200335	1.00252914	TTF2_Q	0.263611356976227
0.82805128	0.68303279	1.00385944	HSD11E	0.266336929005709
0.85227498	0.72331216	1.00423122	MORF4	0.272140359955927
0.86005790	0.73661270	1.00419066	ADGRG	0.272525566548442
1.22770083	0.99237230	1.51883455	LRG1_F	0.281341210454613
0.87931415	0.76957927	1.00469621	NPPC_F	0.281341210454613
0.85676308	0.72949337	1.00623666	NPR1_P	0.283535024729516
0.85556504	0.72718521	1.00660950	SH3BP1	0.284701542532401
1.15974626	0.99355414	1.35373740	PADI2_	0.285155311908486
1.16320648	0.99239320	1.36342058	NOMO1	0.289596452200761
1.16424130	0.99240949	1.36582511	TMSB1	0.289596452200761
0.84762411	0.71272920	1.00804995	TSC1_Q	0.289596452200761
1.17502772	0.99055313	1.39385773	CEACA	0.298108523633917
1.17565131	0.99006622	1.39602380	RPL14_	0.300219080080454
1.15667235	0.99084346	1.35025458	SCRIB_	0.300763506670682
0.86492988	0.74025074	1.01060850	ART5_C	0.309437003732046
1.15131620	0.98993580	1.33900499	GALNT	0.309437003732046
1.15540366	0.98894271	1.34988368	TNFSF1	0.313173350824384
0.85082524	0.71440679	1.01329327	ARNT_I	0.317019151689579
0.85724809	0.72560709	1.01277164	PFDN6_	0.317019151689579
1.16663470	0.98715953	1.37874020	PMCH_	0.317474252847078
1.16016680	0.98721512	1.36341814	LSP1_P	0.319443488528106
1.14805099	0.98787537	1.33419773	LXN_Q	0.320223895643952
1.15373602	0.98702816	1.34860063	CCDC2	0.32029270002121
0.86389065	0.73636861	1.01349658	CNTNA	0.32029270002121
1.15983017	0.98679905	1.36320157	RBPMS	0.32029270002121
0.86781599	0.74292002	1.01370883	TP53INI	0.324145935560254
0.86406678	0.73549796	1.01511008	C1QA_I	0.328814137953841
1.14491906	0.98587585	1.32961938	ENTR1_	0.328814137953841
1.15189532	0.98548476	1.34640623	GRIN2E	0.328814137953841
1.17240427	0.98336082	1.39778985	NOS3_F	0.328814137953841

1.1529435050	0.9856190990	1.3486738710	PHLDB1	0.328814137953841
1.1598680377	0.9842792285	1.3667807121	LCN15_	0.329123446417511
0.8590890450	0.7259972228	1.0165796301	WWP2_	0.329511557308074
1.1547379986	0.9840461052	1.3550379788	SCPEP1	0.332286645128329
0.8695186915	0.7437387035	1.0165704047	ADRA2.	0.337619220732519
0.8605623117	0.7272794794	1.0182708481	KDM3A	0.339818555575013
1.1460390526	0.9828818783	1.3362801156	ACOX1_	0.340104902875516
1.1606864835	0.9817650794	1.3722153509	ATRAII	0.340104902875516
0.8738377900	0.7504155278	1.0175595453	DHRS4I	0.340104902875516
0.8712026091	0.7460076785	1.0174077398	IKZF2_(	0.340104902875516
0.8783455181	0.7593314869	1.0160132465	MRI1_C	0.340104902875516
0.8778428660	0.7577259102	1.0170011174	NAPRT_	0.340104902875516
1.1563707896	0.9812936782	1.3626842125	NME3_(	0.340104902875516
0.8713307828	0.7461537081	1.0175079541	SHPK_C	0.340104902875516
0.8725757792	0.7479442856	1.0179748746	YJU2_Q	0.340104902875516
0.8658012016	0.7352754701	1.0194977954	GNPDA	0.342568734495504
0.8719034311	0.7461584068	1.0188394130	TUBB3_	0.343743638838152
1.1407233873	0.9813928622	1.3259214494	DNMBP	0.3497080505016
0.8388036960	0.6860382391	1.0255866237	DSG4_C	0.3497080505016
1.1533068694	0.9787687437	1.3589693619	ACY3_(	0.355420783887164
1.1815630448	0.9751050649	1.4317341577	COL5A1	0.355420783887164
0.8741708045	0.7485234573	1.0209093490	UBE2Z_	0.35725341285015
0.8642700919	0.7302611735	1.0228707467	BRD3_C	0.357271744901344
1.1465238815	0.9788278024	1.3429502182	LBR_Q1	0.357665603248782
1.1512721873	0.9778371243	1.3554687343	LTO1_C	0.359286974629782
0.8718219674	0.7432959850	1.0225718398	ADAMT	0.36203244048676
0.8752810179	0.7487093033	1.0232500878	MAG_P	0.371596180582489
1.1425979618	0.9770658461	1.3361741253	RYR1_F	0.371985128035289
0.8762108590	0.7494339424	1.0244338105	NOP56_	0.377747754560002
1.1415166285	0.9760991699	1.3349670335	SLC34A	0.377747754560002
0.8749212069	0.7471293045	1.0245711333	SOX9_F	0.377747754560002
0.8730601872	0.7433388889	1.0254193637	CDC123	0.378812782911332
0.8790524550	0.7534018093	1.0256588318	SMPDL3	0.389560813649485
1.1391071624	0.9746574962	1.3313036963	TBR1_C	0.389560813649485
1.1466630914	0.9726557784	1.3518001686	ZNRD2_	0.394274337811458
1.1380913183	0.9733798801	1.3306745653	CACNA	0.399518769787277
1.1444356098	0.9720858181	1.3473428380	GAMT_	0.399573887164225
1.1370728298	0.9728741530	1.3289844491	DTX3_C	0.402864115609671

1.1342792618	0.97268184448	1.32272381844	SART1_	0.407793942302246
1.14673496369	0.96933561044	1.35660040088	LMNB2_	0.414764462564105
1.15003348788	0.96793691233	1.36638762906	CCL4_P	0.419560519309189
0.89361056206	0.77654531333	1.02832355544	PCDH17	0.434206613328605
1.13005095544	0.97000083339	1.31650934433	TADA3_	0.434206613328605
1.14062426755	0.96719759311	1.34514780522	AGXT_I	0.435105790138133
1.12697518706	0.97011176511	1.30920283533	FCRL2_	0.435105790138133
1.11653349055	0.97246636688	1.28194360044	PXN_P4	0.435105790138133
1.15680866833	0.96246258511	1.39039825106	ITPR1_(	0.440616859838212
1.12666635277	0.96942203833	1.30941635344	MTSS1_	0.440616859838212
0.88362939955	0.75581598544	1.03305689588	THAP12	0.440616859838212
1.12878120433	0.96747803711	1.31697707033	AKR7L_	0.449995145979542
1.12937207255	0.96693383877	1.31909881222	MKI67_	0.452261674707154
1.12832556188	0.96675548611	1.31689821444	THY1_F	0.454730560229839
1.12379142944	0.96724455106	1.30567515266	BCL2L1	0.457310470262774
1.12809229788	0.96625757222	1.31703209266	ELOB_(	0.457310470262774
0.88596029388	0.75816304055	1.03529926988	TSPAN7	0.457310470262774
1.13221787144	0.96469040477	1.32883804188	CAPS_(	0.459039341849263
1.13020409999	0.96437952288	1.32454213022	GPKOW	0.464834591335525
0.88799196606	0.76095347977	1.03623907899	CABP2_	0.466903390030929
1.12007595955	0.96507174211	1.29997605399	AMOT_	0.480044506128616
1.12546394088	0.96227329744	1.31632986766	MORN4	0.491010278981066
0.87746541655	0.73757510733	1.04388766577	HDAC9_	0.49155364445863
0.90877409355	0.80030149606	1.03194903077	KLK1_F	0.49155364445863
0.87514136511	0.73224498988	1.04592372711	ITGAV_	0.496795577662615
1.12986150999	0.95972597255	1.33015784522	KRT14_	0.496795577662615
1.12745188606	0.95990365899	1.32424514166	PLIN1_(	0.498388683775912
0.88626343766	0.75380313677	1.04200016555	TARS1_	0.498388683775912
1.12878234399	0.95883946711	1.32884557188	HJV_Q6	0.500955015847018
1.12375557906	0.96033277711	1.31498854511	LYPD8_	0.500955015847018
0.89213711211	0.76491457511	1.04051962499	STXBP1	0.500955015847018
1.13005912077	0.95781557266	1.33327714955	CCN2_F	0.502545736494278
0.89152418788	0.76332536799	1.04125371822	OFD1_(	0.502545736494278
1.13666078633	0.95526712966	1.35249890122	APBB1I	0.503652336599168
0.89433705406	0.76836652099	1.04095993833	PRKAR	0.503652336599168
1.13038235411	0.95696681799	1.33522316811	TPR_P1	0.503652336599168
0.91605900744	0.81327258106	1.03183621906	ZP3_P21	0.503652336599168
0.89159631977	0.76245477588	1.04261134233	HIP1_O(	0.506396756098682

0.914035100(	0.808124130(	1.033826528(	ADGRB	0.509148483411045
0.895897703(	0.770592569(	1.041578555(	GPR158	0.509148483411045
0.892591629(	0.763714116(	1.043217350(	PPM1B_	0.509148483411045
0.906181922(	0.791622698(	1.037319518(	SPAG1_	0.509148483411045
0.881138131(	0.739653415(	1.049686773(	RNF5_C	0.518441366687639
0.883820936(	0.744869489(	1.048693038(	CXCL3_	0.518688270756144
1.107321245(	0.960793649(	1.276195300(	DCBLD	0.524450247850632
1.124901678(	0.954524842(	1.325689736(	MANEA	0.524495662538146
0.886619460(	0.749614308(	1.048664706(	SSH3_Q	0.524495662538146
0.891938964(	0.759730615(	1.047154214(	DNM1_(	0.53030545800111
1.11552841	0.956318575(	1.301243815(	BRK1_(	0.534273395111826
1.116819371(	0.955224124(	1.305751684(	CDK1_I	0.535628943811893
0.896076734(	0.767255152(	1.046527366(	CTAG1/	0.535628943811893
0.892090921(	0.759176507(	1.048275605(	SLA2_C	0.535628943811893
0.895832334(	0.766391665(	1.047135045(	CHM_P	0.538050903852697
1.114125956(	0.954311282(	1.300704150(	BAX_Q(	0.540933212582275
0.890044724(	0.753096085(	1.051897131(	CD34_P	0.540933212582275
0.896767530(	0.767216428(	1.048194452(	CSPG5_	0.540933212582275
1.122437437(	0.951127431(	1.32460253	PADI4_(	0.540933212582275
1.111812538(	0.955259966(	1.294021695(	PPP1R1(	0.540933212582275
1.119854812(	0.952547304(	1.316548579(	TNFAIP	0.540933212582275
1.114714383(	0.953963742(	1.302552813(	TPPP3_(	0.540933212582275
0.896891474(	0.768156106(	1.047201617(	VTCN1_	0.540933212582275
0.892454415(	0.757892462(	1.050907513(	RAB37_	0.541109027970927
1.118740048(	0.952073904(	1.314582082(	TGFBR(	0.541109027970927
0.885818131(	0.743930721(	1.054767252(	PDP1_Q	0.54155687331262
1.123822010(	0.949241504(	1.330510630(	HIP1R_(	0.545827756571582
1.108925874(	0.954780579(	1.287957277(	VEGFD_	0.545827756571582
1.119843386(	0.950392938(	1.319506027(	DNAJA(	0.546155873568751
1.093102056(	0.960448784(	1.244076857(	GASK1/	0.548191226748264
1.112959134(	0.952050952(	1.301062756(	MOCS2_	0.552042946016457
1.103561325(	0.954867652(	1.275409839(	CALCA_	0.559278795660925
1.106025673(	0.953553765(	1.282877624(	ECHDC	0.559831409482477
1.121727778(	0.947164721(	1.328462915(	NRTN_(	0.559831409482477
0.900308152(	0.770964336(	1.051351834(	PRKD2_	0.562222563979728
0.893738587(	0.756447824(	1.055946804(	ZNF830_	0.567709469230387
0.894261142(	0.756352184(	1.057315636(	ITGB1_	0.577102289681369
1.127382232(	0.941800318(	1.349533093(	PTPRK_	0.577102289681369

1.104222517(	0.951762571	1.281104556	TPSD1_	0.577102289681369
0.902039201	0.771504073	1.054660303	ZPR1_O	0.589886273182044
1.127295253	0.939587053	1.352503297	GPA33_	0.591757864117956
1.113757809	0.944116306(	1.313880981	AKAP1	0.595982982197931
1.124156133	0.939846335	1.344610246	CD28_P	0.595982982197931
0.899612114	0.764789333	1.058202462	COQ7_(	0.595982982197931
0.901821459	0.769885193	1.056367821	DNAJC	0.595982982197931
1.111804313	0.944649666(	1.308536778(	DNM3_	0.595982982197931
0.903335541	0.772646837	1.056129477	ECSCR_	0.595982982197931
1.104947476	0.948039816	1.287824525(	PDZD2_	0.595982982197931
0.896491061	0.757674127(	1.060741279	DNLZ_(	0.596527631128837
0.907346145	0.780819005	1.054376266(	CDA_P	0.597678515897373
1.110605165(	0.944633886	1.305737439	PHYKP	0.597678515897373
1.106144543(	0.946281319	1.293014798	SKIV2L	0.598513375628692
0.904265755	0.773632957	1.056956724	SLC44A	0.599650642852415
1.104522233	0.946004403	1.2896022	NDUFS(	0.603601151902051
0.902799145	0.769643882	1.058991456(	SLC1A4	0.603601151902051
0.898895381	0.761266465(	1.061406149	SULT1A	0.603601151902051
0.885903154	0.732146667	1.071949699	ADD1_I	0.606851213967975
0.897434610	0.756975057	1.063956959	MN1_Q	0.606851213967975
1.382710746	0.831455339	2.299448831	NUCB2	0.606851213967975
1.114862713(	0.939874369	1.322430859	RCC1_F	0.606851213967975
1.101552205	0.946590466	1.281881980(	TOP2B_	0.606851213967975
1.098923221	0.947083394	1.275106557	HSDL2_	0.607695171164187
1.113922012(	0.939267355	1.321053311	HNRNP	0.609803014693511
1.103861750	0.943736804(	1.291155286	GATA3_	0.612623635213212
1.104586113	0.942748992	1.294205023	IQGAP2	0.616553626511623
1.091275908	0.949301448	1.254483610	NTproB	0.617446714239974
0.910884906	0.784679223	1.057389170	MYL4_I	0.617713213210298
0.917899060	0.800280590	1.052804097	LPA_P0	0.618467575153165
1.101160218	0.943445559	1.285239848(	MYO9B	0.619751535611891
0.905445833	0.771687336	1.06238903	GNGT1	0.620872223767262
1.104354098	0.941334542	1.295605249	PCDH1_	0.620872223767262
0.921521109	0.807817639(	1.051228785	MEP1B_	0.620947694171245
1.099557262	0.942099454	1.283331784	ERI1_Q	0.631532418125076
1.101842287	0.941008700	1.290164933	SRPX_F	0.631532418125076
0.907779925	0.775193133	1.063043979	TRIM21	0.632711193772095
1.103116850(	0.939076559	1.295812118	CEP85_(	0.635414434002295

1.0967634668	0.9424982821	1.2762782960	NBN_O(	0.635414434002295
0.9101257860	0.7797991195	1.0622337543	UNC79_	0.635414434002295
0.8981491974	0.7526082708	1.0718351261	HTR1A_	0.637492588645947
1.1107974865	0.9340112627	1.3210451579	HMGCL	0.638962094396832
1.0988735916	0.9400119574	1.2845827760	CRHR1_	0.639364769239626
0.9097952240	0.7780106087	1.0639023947	PRAME	0.639364769239626
1.1077990475	0.9353668289	1.3120186559	PTPRC_	0.639364769239626
1.0973431668	0.9399137619	1.2811409668	LYAR_(	0.646209588976358
0.9115388203	0.7801141105	1.0651044632	CTNNA	0.654266442448776
1.1111430543	0.9306672643	1.3266168635	MRPL58	0.654266442448776
0.9121851950	0.7810418180	1.0653486303	GBA_P(	0.654862447098737
0.9133278795	0.7838259148	1.0642258693	GORAS	0.654862447098737
1.0968442216	0.9383371108	1.2821268950	RICTOF	0.654862447098737
0.9128699921	0.7823407484	1.0651773210	BCL7B_	0.656054149074585
1.1016520714	0.9347949175	1.2982925598	FUS_P3	0.656054149074585
1.1024689034	0.9343315760	1.3008633277	SCAMP	0.656054149074585
1.1049121940	0.9316257768	1.3104306330	GALNT	0.658299850603023
1.0969829283	0.9368966649	1.2844229147	KLHL41	0.658299850603023
0.9081135962	0.7702041087	1.0707165728	KYAT1_	0.658299850603023
0.9154404475	0.7875615225	1.0640834893	PCDH7_	0.658299850603023
0.9081416688	0.7704200583	1.0704826305	VSTM2I	0.658299850603023
1.0925732557	0.9388549185	1.2714598342	SNED1_	0.658778414392358
0.9085368608	0.7702303968	1.0716783328	ADGRE	0.660791963432109
1.0877326250	0.9411156502	1.2571911468	HMCN2	0.660791963432109
0.9128690970	0.7803808636	1.0678503626	JMJD1C	0.660791963432109
0.9170704361	0.7893738248	1.0654244648	ITGA6_	0.666654573928127
0.8887954807	0.7238985109	1.0912543606	RNF4_P	0.671328447167053
0.9116742539	0.7751678253	1.0722193545	NDUFB	0.677638508665896
1.0913048518	0.9362418825	1.2720497788	SNX15_	0.677638508665896
1.0910070772	0.9352522642	1.2727009471	ARL13E	0.683107264687609
1.0919241629	0.9348588207	1.2753780048	CDH22_	0.683107264687609
1.0924294858	0.9343650043	1.2772333895	MYOM1	0.683107264687609
0.9376011971	0.8363471528	1.0511137652	APOBR_	0.685153893641111
0.9068206074	0.7621765400	1.0789148850	CD82_P	0.685586770868692
1.0930018961	0.9317226308	1.2821982696	BAP18_	0.694993035552151
1.1604019341	0.8881719162	1.5160720848	PILRB_(	0.694993035552151
1.0939993031	0.9309620701	1.2855888694	TOMM2	0.694993035552151
0.9107907174	0.7670576245	1.0814568611	ADAMT	0.705177535075528

1.095331811	0.927786711	1.293133176	CAMLG	0.705177535075528
1.091732665	0.930552644	1.280830504	CD276_	0.705177535075528
1.089910372	0.931984362	1.274597157	H2AP_C	0.705177535075528
0.917885519	0.785154736	1.073054504	IL13RA	0.705177535075528
1.092990359	0.928294644	1.286905975	MTR_Q	0.705177535075528
0.912838013	0.772338587	1.078896291	NINJ1_	0.705177535075528
0.919641535	0.788861629	1.072102537	SAFB2_	0.705177535075528
1.093006726	0.928682130	1.286407550	SLC12A	0.705177535075528
1.092318399	0.929001271	1.284346450	TIMM1	0.705177535075528
0.908433896	0.761773099	1.083330646	XPNPEI	0.705177535075528
0.918938986	0.786397133	1.073819861	ZFYVE	0.706534366398583
0.914279067	0.774633868	1.079098458	EXOSC	0.709275318193239
1.091093539	0.928451188	1.282226924	PEPD_P	0.709275318193239
1.061897931	0.948788123	1.188492128	AFAP1_	0.722474890890635
0.921089963	0.789223974	1.074988531	AMOTL	0.722474890890635
1.085938762	0.930048433	1.267958693	PAXX_	0.722474890890635
0.920279589	0.787031184	1.076087629	GABRA	0.722798321002129
1.103008953	0.916631970	1.327281604	GPRC5	0.72448705568256
0.921586585	0.788919444	1.076563443	ATXN2_	0.724877280444848
1.081850763	0.930565910	1.257730441	BCAT1_	0.724877280444848
0.920339352	0.786459582	1.077009603	BSND_	0.724877280444848
0.914581479	0.770426557	1.085709305	DNAJB	0.724877280444848
1.085057865	0.928047035	1.268632437	GHRHR	0.724877280444848
1.089935783	0.923912099	1.285793326	NFATC	0.724877280444848
0.909522027	0.759208986	1.089595002	NPHS1_	0.724877280444848
1.092983680	0.922484408	1.294995680	PAGR1_	0.724877280444848
0.921488697	0.788474829	1.076941696	PALM3_	0.724877280444848
1.089076397	0.925362123	1.281754860	PCDH1	0.724877280444848
0.909985932	0.759431965	1.090386544	SESTD1	0.724877280444848
1.089078893	0.926048790	1.280810307	STAM_	0.724877280444848
0.923338464	0.793927961	1.073842919	TAB2_C	0.724877280444848
1.080683062	0.930745701	1.254774403	WASHC	0.725726406960289
1.078579348	0.932201636	1.247941825	C7orf50	0.726100324637178
0.934904929	0.820497694	1.065264694	TNN_Q	0.731162527530964
1.081226302	0.928720316	1.258775431	DMD_P	0.732729740197426
1.088890513	0.922105592	1.285842487	ELAVL	0.732729740197426
1.085190535	0.925155131	1.272909223	ITGB1B	0.732729740197426
0.922420386	0.788139181	1.079580092	PLPBP_	0.732729740197426

0.92621339	0.79689332	1.07651957	CEACA	0.733803765961434
1.08762536	0.92238112	1.28247305	REPS1_	0.733803765961434
1.09138128	0.91955714	1.29531167	THBD_	0.733803765961434
0.91165835	0.75922969	1.09468974	CCER2_	0.736541546492218
0.92492447	0.79160917	1.08069147	CGN_Q	0.736541546492218
0.93392914	0.81476136	1.07052652	FGFBP2	0.736541546492218
0.92208558	0.78541887	1.08253298	HCG22_	0.736541546492218
1.08043344	0.92639012	1.26009162	LPP_Q9	0.736541546492218
1.08207337	0.92444922	1.26657339	OTUD7	0.736541546492218
1.07813588	0.92840354	1.25201696	PBXIP1	0.736541546492218
1.08457726	0.92292827	1.27453874	SMARC	0.736541546492218
1.08170680	0.92522456	1.26465470	STEAP4	0.736541546492218
1.08631540	0.92101717	1.28128029	TSPAN	0.736541546492218
1.08668478	0.92034979	1.28308151	VAMP5	0.736541546492218
0.91965449	0.77912425	1.08553210	ZCCHC	0.736541546492218
1.08286043	0.92284434	1.27062242	ATP6V1	0.736841030923943
0.92511061	0.79114142	1.08176568	REST_	0.736841030923943
1.10063601	0.90824226	1.33378470	SLC51B	0.736841030923943
0.91836414	0.77394756	1.08972847	TSNAX	0.736841030923943
0.91773436	0.77149001	1.09170091	ARMCX	0.741366216483777
1.08172992	0.92273890	1.26811563	MUC13	0.741366216483777
0.90605583	0.74144457	1.10721313	IL12A_	0.743516841588736
1.08557177	0.91867913	1.28278310	MECR_	0.743516841588736
0.92226242	0.78103204	1.08903084	ALMS1	0.744847451338363
0.92627824	0.79224015	1.08299406	ATP5P	0.744847451338363
0.91492322	0.76245263	1.09788395	CADPS	0.744847451338363
1.08143422	0.92053496	1.27045689	CDH4_	0.744847451338363
0.92608612	0.79152831	1.08351840	GIPC3_	0.744847451338363
0.92735220	0.79414509	1.08290300	MNAT1	0.744847451338363
1.07899169	0.92315688	1.26113243	NUDT1	0.744847451338363
0.90835036	0.74638809	1.10545759	TEF_Q1	0.744847451338363
0.92231745	0.78069763	1.08962734	EIF2S2	0.745381368708418
0.92024859	0.77499573	1.09272533	IZUMO	0.746751675703634
1.08384474	0.91637355	1.28192204	CCND2	0.753024139646664
0.92806783	0.79429745	1.08436694	ITPRIP_	0.753024139646664
1.07211571	0.92617838	1.24104830	SLC39A	0.759721886774224
0.92008003	0.77208854	1.09643807	SCG2_P	0.760268894273606
1.07561358	0.92210924	1.25467193	CASC3_	0.762372098623473

1.07650330	0.92068344	1.25869468	GIPR_P	0.765102520312017
0.92965448	0.79595281	1.08581495	ATP6V1	0.765968174356372
1.08072677	0.91618219	1.27482324	CD1C_F	0.765968174356372
0.92564670	0.78461254	1.09203177	TSHB_F	0.769729381088697
1.08026291	0.91466900	1.27583634	CASP4_	0.774388118159248
0.93028978	0.79616300	1.08701242	KCNH2_	0.774388118159248
0.92912507	0.79179992	1.09026709	DARS1_	0.778186779397937
1.07444340	0.91908331	1.25606527	EDEM2_	0.778186779397937
1.07471727	0.91915206	1.25661168	PNMA2_	0.778186779397937
0.92380909	0.77787810	1.09711692	RAB3G	0.778186779397937
1.07412099	0.91843615	1.25619609	MUCL3_	0.783373755416702
1.07772430	0.91453237	1.27003668	MME_P	0.783615761605772
0.93200053	0.79746585	1.08923159	AKT3_C	0.786937283356666
0.93444244	0.80389721	1.08618696	INPP5J_	0.786937283356666
0.92684530	0.78338451	1.09657799	PTPRN2	0.786937283356666
1.07270182	0.91794583	1.25354803	SSBP1_	0.786937283356666
0.93275259	0.79943609	1.08830137	UFD1_C	0.786937283356666
1.07060944	0.92032896	1.24542921	VPS37A	0.786937283356666
0.92878428	0.78654047	1.09675252	DCDC2	0.79023126804155
0.92435205	0.77559425	1.10164137	DDX1_(	0.79023126804155
0.93328321	0.79946258	1.08950383	DYNC1	0.79023126804155
0.93320315	0.79890966	1.09007084	IFIT1_P	0.79023126804155
0.93655686	0.80894353	1.08430157	PRDX2_	0.79023126804155
0.93268593	0.79735811	1.09098162	RASGR	0.79023126804155
0.93433750	0.80213727	1.08832564	TXNDC	0.79023126804155
0.93595224	0.80566231	1.08731236	BIN2_Q	0.793697290251757
0.93421937	0.80094242	1.08967362	NUBP1_	0.793697290251757
0.93245672	0.79550852	1.09298080	PRR5_P	0.795142095094353
0.93453855	0.80105673	1.09026274	TRAF2_	0.795895448058652
1.06531827	0.92187477	1.23108153	SPESP1	0.798363707283545
0.93315161	0.79641153	1.09336930	PIBF1_(	0.798860710045223
0.93162853	0.79147539	1.09659976	CYTH3_	0.799253691229317
0.93516247	0.80010493	1.09301770	FRMD7_	0.799253691229317
0.93516664	0.80148050	1.09115149	GATD3_	0.799253691229317
1.06911024	0.91674561	1.24679812	GIMAP	0.799253691229317
0.93667767	0.80443852	1.09065520	HSBP1_	0.799253691229317
1.06847722	0.91643525	1.24574384	LAMTO	0.799253691229317
1.06761628	0.91673561	1.24332959	LRRFIP	0.799253691229317

0.93514993118	0.8006668928	1.09222125060	MGLL_(	0.799253691229317
0.93392241922	0.79711453655	1.09421048688	PPP1R1:	0.799253691229317
0.93480409700	0.79996687300	1.09236860844	STX5_Q	0.799253691229317
0.93478160844	0.79922350399	1.09333202955	TOP1MT	0.799253691229317
1.06819191855	0.91450853911	1.24770182677	AMBN_	0.801344397403159
0.94027326855	0.81326271988	1.08711957155	AMPD3_	0.801344397403159
0.93690836533	0.80316863655	1.09291778233	CDAN1_	0.801344397403159
1.07150488933	0.90958195199	1.26225319822	CEP164_	0.801344397403159
1.06714858799	0.91471659577	1.24498245044	EPGN_(	0.801344397403159
1.06683149288	0.91503484333	1.24380994044	NFIC_P(	0.801344397403159
1.07253437944	0.90917901066	1.26524037811	RLN1_P	0.801344397403159
1.07506354000	0.90692985655	1.27436714833	SCN2A_	0.801344397403159
1.06296273677	0.92134639166	1.22634634499	SDK2_(	0.801344397403159
1.07138229588	0.91157643888	1.25920326055	SLC9A3	0.801344397403159
1.06942014622	0.91218788044	1.25375426900	TRIM26	0.801344397403159
1.07392417566	0.90624538300	1.27262787388	THRAP:	0.802985476540934
0.93763028955	0.80269731399	1.09524542399	MINK1_	0.813941476528969
0.94536240388	0.82477752022	1.08357714988	HDDC2_	0.814589606997859
1.07063014688	0.90706235899	1.26369361488	JCHAIN	0.814589606997859
1.07543930422	0.90179346644	1.28252170833	KLRD1_	0.814589606997859
1.06663903688	0.91207532611	1.24739569	LRRC38	0.814589606997859
1.06221168755	0.91676493900	1.23073387844	CENPF_	0.817147280425203
0.93313619355	0.78712840266	1.10622758988	ATXN2I	0.819901987332898
1.06517080388	0.91214352222	1.24387096300	DGKZ_(	0.819901987332898
1.07038390011	0.90586248277	1.26478545622	MRPL4(	0.819901987332898
1.07275397011	0.90158614988	1.27641832200	NUP50_	0.824420204049258
0.93187702722	0.78154470377	1.11112619622	ITGAX_	0.829382670830216
0.93584463411	0.79305341111	1.10434576911	NIT1_Q	0.829382670830216
0.93571869733	0.79233035700	1.10505608266	NARS1_	0.830334423342374
1.06082472888	0.91479633277	1.23016355122	ADA_P(	0.830575945725543
0.93895173500	0.80134121400	1.10019345722	EVI5_O	0.831841575332395
1.06137975822	0.91344363522	1.23327477222	LAMB1_	0.831841575332395
1.06048813377	0.91428494155	1.23007066022	GGT5_F	0.832556018858943
1.06622156944	0.90607752366	1.25467016388	CD164_	0.835392002638549
1.06153830055	0.91201408277	1.23557693344	STK11_	0.835392002638549
0.91299903677	0.72368773811	1.15183275499	PM20D1	0.837664319999466
0.94206627066	0.80870769866	1.09741611188	EIF2AK	0.837840047518139
1.05689586900	0.91694680211	1.21820467299	PRR4_Q	0.839565393566439

0.9411023623	0.8050899479	1.1000928017	TSC22D	0.83971464553418
1.0651783560	0.9047426736	1.2540636837	AP3B1_	0.840173164122542
0.9358836707	0.7886040096	1.1106692768	GPIHBP	0.840173164122542
1.0633067556	0.9077150347	1.2455685026	TRAF3I	0.840173164122542
0.9422716468	0.8077376362	1.0992131808	UNG_P1	0.840590406800387
1.0606496737	0.9103076656	1.2358214402	DUSP29	0.840884141119261
0.9358025617	0.7870901218	1.1126126605	TSPAN1	0.843509877604495
1.0608230498	0.9087469113	1.2383486854	GIPC2_(	0.843704286989315
0.9429942791	0.8086595439	1.0996447358	MPHOS	0.843704286989315
0.9405687513	0.8009920288	1.1044673906	TMED8_	0.843704286989315
0.9423019472	0.8057289141	1.1020244454	CDC27_	0.845088905472704
0.9370418420	0.7896611497	1.1119293561	SOWAH	0.845088905472704
0.9382493464	0.7927540876	1.1104475521	SCN3A_	0.846531177008312
0.9442799346	0.8108508743	1.0996653308	ANKMY	0.848009261084735
0.9433574121	0.8081308772	1.1012117369	GUCY2(	0.848009261084735
0.9394665773	0.7955831826	1.1093716776	CD300L	0.848229949256998
1.0604010327	0.9055916734	1.2416747891	CIAPIN	0.851555531347069
1.0599407366	0.9063244711	1.2395939874	LELP1_	0.851555531347069
0.9444208210	0.8098946705	1.1012922047	MORC3	0.851555531347069
0.9423708230	0.8035119092	1.1052266405	RFC4_P	0.851555531347069
0.9408079127	0.7969340503	1.1106559297	GRSF1_	0.854837428238731
1.0567214793	0.9095130201	1.2277562389	HSPA2_	0.854837428238731
0.9439021893	0.8070935083	1.1039010149	RCOR1_	0.854837428238731
0.9446607250	0.8094544768	1.1024509851	STX16_	0.854837428238731
1.05867765	0.9063177731	1.2366505411	GADD4	0.854968402056175
1.0659956968	0.8952150234	1.2693562952	SERPIN	0.855647980453468
1.0599279481	0.9038004258	1.2430258087	CERT_(	0.856013658587117
1.0605503767	0.9024675676	1.2463241250	KIFBP_(	0.856914516734781
1.0599140753	0.9028550354	1.2442948237	RAB44_	0.858632017034041
1.0650221375	0.8943236128	1.2683016944	DENND	0.86193962467487
1.0578794219	0.9048347404	1.2368102388	FHIP2A	0.86193962467487
0.9454894028	0.8085999988	1.1055530696	CACNB	0.864226642304574
1.0561262498	0.9057169912	1.2315134488	ATXN1(	0.865296612426487
1.0603497780	0.8993301602	1.2501989829	PFKFB2	0.865296612426487
0.9457961893	0.8087112311	1.1061184725	PITHD1	0.865296612426487
1.0689489995	0.8867034144	1.2886518142	VIPR1_	0.865296612426487
1.0624101133	0.8939600321	1.2626014681	MLLT1_	0.874347725896299
1.2905552562	0.6219510717	2.6779162300	PRSS53	0.87449634995758

1.0629577607	0.8925264854	1.2659335264	SLC16A	0.87449634995758
0.9472318533	0.8106431168	1.1068350120	CRX_O4	0.874502658596359
1.0603408597	0.8960979365	1.2546873426	CWC15_	0.874502658596359
1.0579142743	0.8981409350	1.2461102353	ABRAX	0.875781379642662
1.0546574848	0.9030119713	1.2317692850	ACTN4_	0.875781379642662
1.0552460123	0.9019722577	1.2345658495	BCL7A_	0.875781379642662
0.9457233112	0.8041822669	1.1121764533	FKBPL_	0.875781379642662
0.9464732790	0.8063701402	1.1109187013	MEGF1	0.875781379642662
0.9479738230	0.8114463193	1.1074723601	NUB1_(	0.875781379642662
1.0579892803	0.8986695520	1.2455538466	PALLD_	0.875781379642662
1.0551580300	0.9028797184	1.2331193687	UPK3BI	0.875781379642662
0.9438955980	0.7963246820	1.1188136195	CHMP1	0.877158609184666
0.9442144098	0.7974708391	1.1179604419	DCTN1_	0.877158609184666
1.0534859132	0.9036224912	1.2282037909	SYAP1_	0.877158609184666
1.0546364090	0.9016997575	1.2335125367	TMOD4	0.877158609184666
1.0521852503	0.9052503205	1.2229697973	CRACR	0.878753102048427
0.9451180783	0.7988571740	1.1181575517	ASPSCI	0.878800215128836
1.0549951941	0.8998449125	1.2368963186	DIPK1C	0.878800215128836
0.9483178928	0.8099780579	1.1102854170	JPT2_Q	0.878800215128836
0.9445013966	0.7970131294	1.1192825505	TET2_Q	0.878800215128836
1.0586518879	0.8922920333	1.2560280469	ATP6V1	0.879424615119831
1.0313341660	0.9399128458	1.1316476486	FUCA1_	0.879424615119831
1.0516842923	0.9045400472	1.2227649334	GIT1_Q	0.879424615119831
0.9427870405	0.7898926561	1.1252761965	GTPBP2	0.879424615119831
1.0524142979	0.9027417750	1.2269021830	SLC27A	0.879424615119831
1.0683720197	0.8708359868	1.3107161278	ADA2_(	0.879592235859884
0.9502937789	0.8129136258	1.1108907977	ADGRV	0.879592235859884
1.0568188050	0.8889403873	1.2564014459	AHNAK	0.879592235859884
1.0524389477	0.9004751851	1.2300480424	ANP32C	0.879592235859884
0.9516692774	0.8184716500	1.1065434128	APPL2_	0.879592235859884
0.9516322073	0.8155255705	1.1104542774	FES_P0	0.879592235859884
0.9483426454	0.8043779777	1.1180735897	FLI1_Q	0.879592235859884
1.0490157402	0.9063781279	1.2141003729	LHPP_C	0.879592235859884
1.0528030545	0.8987074437	1.2333204529	MTPN_	0.879592235859884
1.0500934482	0.9024500295	1.2218917545	PACS2_	0.879592235859884
0.9467319637	0.7975416936	1.1238301624	PI16_Q	0.879592235859884
0.9513603542	0.8170779972	1.1077112915	RAPGEI	0.879592235859884
0.9432601233	0.7902654720	1.1258743950	RGCC_(	0.879592235859884

1.0526690822	0.9008118140	1.2301261811	RNF31_	0.879592235859884
0.9486080311	0.8047227233	1.1182201902	SCN3B_	0.879592235859884
0.9473912343	0.8034881302	1.1170670943	SERPIN	0.879592235859884
0.9510307789	0.8136983217	1.1115416099	SNU13_	0.879592235859884
1.0517260228	0.9005498025	1.2282803506	TNPO1_	0.879592235859884
0.9441407467	0.7923104309	1.1250662806	TRIM40	0.879592235859884
1.0509825028	0.9000194548	1.2272670500	WAS_P4	0.879592235859884
1.0505363384	0.9001211384	1.2260867466	WASF3_	0.879592235859884
1.0501036000	0.9017241524	1.2228990084	XIAP_P	0.879592235859884
0.9494490195	0.8064577193	1.1177938025	ANKRA	0.88098102849856
0.9420908822	0.7794112050	1.1387252642	FOXO1_	0.885065833235605
0.9507198102	0.8096670422	1.1163454980	PRTFDC	0.885065833235605
0.9540706374	0.8207488967	1.1090490463	PSMD1_	0.888711337696956
0.9503685987	0.8066107587	1.1197476151	IMPACT	0.891451673416088
0.9525471290	0.8140868802	1.1145567570	MYH7B	0.891451673416088
1.0493321978	0.8975769092	1.2267450845	PBLD_F	0.891451673416088
0.9528603555	0.8146314506	1.1145443211	PDIA2_	0.891451673416088
0.9416567314	0.7748913105	1.1443119670	TBL1X_	0.891451673416088
0.9547234831	0.8202625666	1.1112258028	BAG6_F	0.894961974218492
1.0528969496	0.8878133700	1.2486768322	F11R_Q	0.894961974218492
0.9494457753	0.8003784651	1.1262762799	LSM8_C	0.894961974218492
1.0471125575	0.8994240381	1.2190520397	NFKBIE	0.894961974218492
0.9492559933	0.7995652581	1.1269711029	NUDT10	0.894961974218492
0.9471239310	0.7918748334	1.1328100134	PTPRF_	0.894961974218492
0.9556482957	0.8226145624	1.1101963261	SUGP1_	0.894961974218492
1.0449336821	0.9030560227	1.2091015093	CD99_P	0.895906906877056
1.0504925842	0.8887422792	1.2416813009	AARSD	0.896707808504659
0.9543795990	0.8164174845	1.1156552086	ADCYA	0.896707808504659
1.0466499447	0.8953847883	1.2234696423	BHLHE4	0.896707808504659
0.9542983519	0.8131118760	1.1200005332	CDK5R	0.896707808504659
1.0472396595	0.8975587133	1.2218820767	GRK5_F	0.896707808504659
1.0514807441	0.8865025045	1.2471614569	L3HYPI	0.896707808504659
0.9553498562	0.8168620723	1.1173163483	NMRK2	0.896707808504659
1.0450934190	0.8985180316	1.2155796722	PER3_P	0.896707808504659
1.0465298463	0.8952695459	1.2233463365	RPGR_C	0.896707808504659
0.9503166633	0.7990888255	1.1301644219	SDHB_F	0.896707808504659
1.0479300638	0.8932737449	1.2293626952	SKAP1_	0.896707808504659
0.9540871462	0.8125259754	1.1203116086	SLC13A	0.896707808504659

1.046612522	0.897621310	1.220333965	TRIM24	0.896707808504659
0.953220287	0.810019098	1.121737645	VASH1_	0.896707808504659
0.954129680	0.815225498	1.116701390	YARS1_	0.896707808504659
0.952294297	0.806829219	1.123985605	ZNRF4_	0.896707808504659
1.046251339	0.894704973	1.223466839	CEP350	0.89798536335491
1.044795954	0.898015833	1.215567193	PPP1R9	0.89798536335491
0.951800590	0.802241947	1.129240831	RP2_O7	0.89798536335491
0.955900814	0.817445670	1.117806847	NPHS2_	0.898236321320981
0.955096222	0.813144072	1.121829237	CLSPN_	0.900184267478787
0.953341664	0.806061769	1.127531864	GP1BB_	0.900184267478787
1.051648207	0.881181338	1.255092346	PIKFYV	0.900184267478787
0.955850451	0.816006944	1.119659693	PSMC3_	0.900184267478787
1.047096099	0.890585777	1.231111331	SLC9A3	0.900184267478787
1.045715363	0.893310847	1.224121059	VPS53_	0.900184267478787
1.051188137	0.880720004	1.254651302	LTB_Q0	0.902372527397387
0.957360773	0.819516959	1.118390096	BRAP_(	0.905013508862007
1.042553034	0.897748100	1.210714709	PDLIM7	0.907152389244932
0.957185459	0.817160759	1.121204113	CNST_(	0.91011538517778
0.954251115	0.805361004	1.130667099	PTPRZ1	0.910169697800397
1.042068726	0.897268766	1.210236298	MYLPF	0.910217914412554
1.046424184	0.886797177	1.234784685	CORO6	0.910625659717914
1.045692995	0.888388983	1.230850293	OGFR_(	0.910625659717914
0.954504114	0.804683316	1.132219452	SEC31A	0.912227734725603
0.953569510	0.800572123	1.135806237	EDNRB	0.9124045543158
1.060569916	0.853511375	1.317860053	SDC4_P	0.9124045543158
0.956770622	0.812685860	1.126400824	SNAP23	0.9124045543158
0.959044700	0.821567450	1.119526750	TBC1D	0.9124045543158
1.042914600	0.891827120	1.219598327	BEX3_(	0.912451096982246
1.042569895	0.893108229	1.217043972	HHEX_(	0.912451096982246
0.956173046	0.809384298	1.129583185	OGA_O	0.912451096982246
1.047364878	0.880806017	1.245419726	COX6B	0.913915986589046
0.958896825	0.818321369	1.123621055	GCC1_(	0.917081951973171
0.959796372	0.821869798	1.120869849	SEPTIN	0.917081951973171
0.960075840	0.822498235	1.120665770	CENPJ_	0.918090869081364
0.960033814	0.820871625	1.122788139	ATF4_P	0.919399085298314
0.957807816	0.812263236	1.129431657	NRN1_(	0.919399085298314
1.040435010	0.893731582	1.211219378	PRKAB	0.919399085298314
0.960596242	0.823354811	1.120713849	UBAC1	0.919399085298314

0.9597478513	0.8188498393	1.1248899296	SPRING	0.921664260928739
1.0390727365	0.8941094175	1.2075391788	AAMDC	0.923215029620992
0.9607418015	0.8218239917	1.1231417170	CACNA	0.923215029620992
1.0468605992	0.8758358346	1.2512814285	F2R_P2	0.923215029620992
0.9599870175	0.8181351784	1.1264337461	GNE_Q	0.923215029620992
0.9610466160	0.8229364641	1.1223352451	TAP1_Q	0.923215029620992
1.0405128624	0.8899245983	1.2165828644	AGBL2_	0.923232701901137
0.9583626986	0.8105644934	1.1331104057	SPRR1E	0.923232701901137
0.9579055574	0.8077215848	1.1360140353	AMDHI	0.924374850933705
0.9593463125	0.8140176883	1.1306208213	FBN2_P	0.924374850933705
0.9596073527	0.8133116214	1.1322182632	AP3S2_	0.926499212154511
0.9611186635	0.8201174227	1.1263619814	CD3D_I	0.926499212154511
1.0398874728	0.8882968505	1.2173475067	CHAC2_	0.926499212154511
0.9605370084	0.8178020365	1.1281842102	OTOA_(	0.926499212154511
1.0375515892	0.8945837031	1.2033678867	PSMG4_	0.926499212154511
1.0472101968	0.8676488730	1.2639320241	EPCAM	0.926559718800375
1.0440286471	0.8767003204	1.2432935071	KRT8_P	0.926559718800375
0.9606046839	0.8155555500	1.1314512649	MINDY	0.926559718800375
1.0411140595	0.8841126652	1.2259958799	RAB10_	0.926559718800375
1.0382941093	0.8907843202	1.2102308414	RABEP1	0.926559718800375
1.0377238522	0.8919990815	1.2072554959	TRIM5_	0.926559718800375
1.0394611058	0.8868983425	1.2182674594	AZI2_Q	0.926972728643317
0.9621279374	0.8209759576	1.1275484492	LZTFL1	0.926972728643317
0.9619874394	0.8160211959	1.1340634756	DDA1_(	0.935872477314141
1.0377826524	0.8882520358	1.2124856349	FMNL1_	0.935872477314141
1.0418547251	0.8756447802	1.2396137026	HS1BP3	0.935872477314141
1.0371592547	0.8886186176	1.2105298024	MRPL28	0.935872477314141
1.0395918627	0.8819396632	1.2254253732	PHLDB1	0.935872477314141
0.9601396294	0.80841393	1.1403416922	PSRC1_	0.935872477314141
1.0344510183	0.8913693872	1.2004999551	ASS1_P	0.936353459643157
1.0356812694	0.8864295931	1.2100630442	BABAM	0.936353459643157
1.0434313193	0.8614754841	1.2638188065	CD40_P	0.936353459643157
1.0381876798	0.8830057430	1.2206417309	CEACA	0.936353459643157
0.9624300674	0.8146400498	1.1370317908	CMIP_C	0.936353459643157
1.0383173338	0.8771563486	1.2290886197	DGKA_	0.936353459643157
0.9665773673	0.8314585293	1.1236541259	DIPK2B	0.936353459643157
1.0367744476	0.8855479444	1.2138261536	EGFL7_	0.936353459643157
0.9660612893	0.8263678636	1.1293692021	EVI2B_	0.936353459643157

1.03772668З	0.877815648(	1.22676859	HDGFL:	0.936353459643157
0.964394268(	0.825154783(	1.12712950	IGFL4_(	0.936353459643157
0.96595112	0.82751757	1.12754289	MAEA_	0.936353459643157
0.964747665(	0.82563173	1.12730412	MAP1L(	0.936353459643157
1.03741343	0.882270354(	1.21983768	MPI_P3	0.936353459643157
1.036864200(	0.88534760	1.214311034(	MYH9_	0.936353459643157
0.96704756	0.83088066	1.12552986	NFAT5_	0.936353459643157
1.04372547	0.86082674	1.26548444	PCDHB	0.936353459643157
1.03875369	0.87982517	1.22639050	PCYT2_	0.936353459643157
0.96531491	0.82500851	1.12948273	POLR2	0.936353459643157
1.03466460	0.88839215	1.20502059	PRELP_	0.936353459643157
0.96279373	0.81728445	1.13420947	PRKRA_	0.936353459643157
0.96515997	0.82450640	1.12980780	SELEN(	0.936353459643157
0.96057611	0.80626847	1.14441590	SH2B3_	0.936353459643157
1.03413913	0.88925779	1.20262510	SPART_	0.936353459643157
1.03664948	0.88635414	1.21242975	TPM3_I	0.936353459643157
0.96577357	0.82378026	1.13224198	ATG4A_	0.937288324120872
1.03606265	0.880675507(	1.21886645	FBLN2_	0.937288324120872
0.96557618	0.82258491	1.13342385	MAGEI	0.937288324120872
1.03639956	0.88032020	1.22015153	UBXN1_	0.937288324120872
0.969386215(	0.83944988	1.119435061(	CPPED1	0.938951492823356
0.94858102	0.74331996	1.21052306	GYS1_I	0.938951492823356
0.96407689	0.81303591	1.14317736	GLI2_P	0.940484254935262
1.03388909	0.88442184	1.20861628	ARNTL_	0.940719135127677
0.966312340(	0.823130780(	1.13439977	ITGB1B	0.940719135127677
0.96799779	0.82826694	1.13130162	ARHGA	0.940726761156576
0.96837311	0.82986768	1.12999520	ATG16I	0.940726761156576
0.96859913	0.82969585	1.13075687	BNIP2_(	0.940726761156576
0.96738877	0.82149668	1.139190276(	BTN1A	0.940726761156576
1.03171635	0.883896748(	1.20425675	CEP112_	0.940726761156576
0.96763310	0.82461299	1.13545850	CIT_O1	0.940726761156576
0.96763755	0.82738322	1.13166717	CREBZI	0.940726761156576
0.96638879	0.81632128	1.14404380	CRYM_	0.940726761156576
0.96615312	0.81761666	1.14167420	EHD3_(	0.940726761156576
0.97376975	0.85439450	1.10982400	GBP4_(	0.940726761156576
0.96953308	0.83075450	1.13149479	GUCA2	0.940726761156576
1.03174560	0.88675077	1.20044890	IRAG2_	0.940726761156576
0.96341915	0.80418500	1.15418276	LYPLA	0.940726761156576

1.0331871893	0.8795740181	1.2136281270	NPY_P0	0.940726761156576
0.9674701440	0.8235427380	1.1365511908	PCBP2_	0.940726761156576
1.0329978753	0.8789286780	1.2140741757	PIK3AP	0.940726761156576
1.0323243960	0.8828982408	1.2070401881	PPP1R1	0.940726761156576
0.9656344537	0.8132215574	1.1466123710	PRTG_(	0.940726761156576
1.0330617619	0.8783892583	1.2149700076	RAB33A	0.940726761156576
1.0342578388	0.8787184227	1.2173288387	RBPMS	0.940726761156576
0.9672915888	0.8214395474	1.1390406278	RWDD1	0.940726761156576
0.9672774860	0.8207520255	1.1399615301	STK4_C	0.940726761156576
1.0365732823	0.8737249972	1.2297738683	SUSD4_	0.940726761156576
0.9689232374	0.8262023593	1.1362981835	DLGAP	0.94347789559729
0.9680647030	0.8215305711	1.1407357220	MED21_	0.94347789559729
1.0293773457	0.8826056699	1.2005562120	AHCY_	0.943829578722486
0.9697650635	0.8263026068	1.1381354370	CALCO	0.943829578722486
0.9646406051	0.7977760312	1.1664069370	CD84_C	0.943829578722486
1.0312002808	0.8788023604	1.2100263576	FOXJ3_	0.943829578722486
0.9708169635	0.8306597328	1.1346229262	GAD2_(	0.943829578722486
0.9669659814	0.8092763133	1.1553819058	LARP1_	0.943829578722486
0.9712919279	0.8373657074	1.1266379801	MGMT_	0.943829578722486
1.0297504747	0.8812555212	1.2032674005	MRPS16	0.943829578722486
1.0280314610	0.8900339428	1.1874251463	MUC2_(	0.943829578722486
1.0307031900	0.8803258954	1.2067679383	PROCR_	0.943829578722486
0.9711475128	0.8340218084	1.1308187413	RNASE	0.943829578722486
1.0307669155	0.8808233001	1.2062356140	SPRED2	0.943829578722486
1.02979181	0.8808610438	1.2039028999	TJP3_O	0.943829578722486
1.0312989640	0.8767645391	1.2130709068	TP73_O	0.943829578722486
0.9710462188	0.8365029137	1.1272294975	TRIM25	0.943829578722486
0.9713909790	0.8359961769	1.1287138162	TRIM58	0.943829578722486
0.9697556072	0.8288362710	1.1346341499	VWA5A	0.943829578722486
1.0292479897	0.8823189800	1.2006444928	MAVS_	0.944220662321499
1.0289268757	0.8813796693	1.2011741959	CC2D1A	0.94660054730345
1.0302899665	0.8762833255	1.2113632476	DFFA_C	0.94660054730345
1.0307858910	0.8749632641	1.21435905	PKN3_C	0.94660054730345
0.9718438433	0.8310705160	1.1364624745	SCGB2A	0.947736185565365
1.0285939417	0.8817341411	1.1999144045	TJAP1_(	0.947736185565365
0.9723222100	0.8328698660	1.1351238888	ARID4B	0.947952474095006
0.9742796260	0.8429027612	1.1261332071	CD2AP_	0.947952474095006
1.0282386407	0.8813149009	1.1996559925	CEP20_(	0.947952474095006

0.97244338O4	0.83231193637	1.13616792759	FUT1_P	0.947952474095006
1.02822649293	0.88083091139	1.20028680560	STAT2_	0.947952474095006
1.02817396733	0.88022163200	1.20099491844	ICA1_Q	0.948305456150909
0.97285968788	0.83348638000	1.13553861822	PHACT	0.948911501202554
0.97204010855	0.82727760255	1.14213411522	NFE2_Q	0.949683998728034
0.96754906560	0.80255271699	1.16646691822	SNX18_	0.949683998728034
1.02951997499	0.87289259400	1.21425177177	TXK_P4	0.949683998728034
1.02686177311	0.88213080744	1.19533870977	NEXN_	0.951243543404489
1.02762502199	0.87832243060	1.20230697622	SEPTIN	0.951889603044013
1.02716722400	0.87445057522	1.20655476255	ACYP1_	0.954589031747633
0.97407602355	0.83333994900	1.13857988055	ATF2_P	0.954589031747633
0.97392547922	0.83145060322	1.14081442160	DAB2_I	0.954589031747633
0.97290826560	0.82560933122	1.14648715500	DXO_O	0.954589031747633
0.97278699200	0.82531729911	1.14660692660	LAMA1	0.954589031747633
1.02765373011	0.87363083599	1.20883117399	M6PR_I	0.954589031747633
0.97433922544	0.83518926499	1.13667280700	PARD3_	0.954589031747633
0.97377857788	0.83049580999	1.14178146044	PLCB2_	0.954589031747633
0.97309836599	0.82638159411	1.14586340799	RILPL2_	0.954589031747633
1.02691419299	0.87887299260	1.19989209860	TWF2_(	0.954589031747633
0.97540948222	0.83890566688	1.13412472422	EFCAB:	0.95473975597064
1.02712826199	0.87325421222	1.20811609222	IGHMB	0.95473975597064
0.97473645544	0.83452231188	1.13850899388	SKAP2_	0.95473975597064
1.02636536733	0.87330056222	1.20625808888	GLOD4_	0.959469509873824
1.02742028633	0.86899119122	1.21473319333	SSNA1_	0.959469509873824
1.02529153611	0.87715583533	1.19844466811	DHODH	0.960445399784299
0.97459262900	0.82817938544	1.14689016555	AKT1S1	0.960967914113344
1.02551180377	0.87461489799	1.20244288322	DDX4_(	0.960967914113344
0.97351483860	0.82231610044	1.15251439255	TRPV3_	0.960967914113344
1.02304341299	0.87732851860	1.19295999455	C1QTNI	0.961406963299502
0.97843139077	0.84145174277	1.13770990999	C9orf40	0.961406963299502
0.97443442588	0.81870026844	1.15979252355	CASP9_	0.961406963299502
1.02371888277	0.87341412744	1.19988939722	CMC1_(	0.961406963299502
0.97540575960	0.82255656560	1.15665771288	CYP24A	0.961406963299502
0.97896456344	0.84474430960	1.13451088760	DDI2_Q	0.961406963299502
0.97697165933	0.83444863244	1.14383748277	DTX2_(	0.961406963299502
0.97651370199	0.83560599700	1.14118258300	DUOX2	0.961406963299502
0.97551828060	0.82953188999	1.14719630111	GOLGA	0.961406963299502
1.02280816533	0.87668389499	1.19328819560	ISM2_Q	0.961406963299502

0.9778249553	0.8386080480	1.1401531922	LTBP2_	0.961406963299502
0.9761735110	0.8335854752	1.1431517840	MAP3K	0.961406963299502
0.9747850504	0.8246466256	1.1522582704	MAP4K	0.961406963299502
0.9768108959	0.8336478994	1.1445593842	MPIG6B	0.961406963299502
0.9769748840	0.8375221625	1.1396473631	NACC1_	0.961406963299502
0.9768348377	0.8347474478	1.1431077780	PCARE_	0.961406963299502
1.0199014523	0.8982050029	1.1580863712	PVALB_	0.961406963299502
1.0241362847	0.8689142311	1.2070870658	SARG_(	0.961406963299502
0.9765480400	0.8336326576	1.1439643913	SERPIN	0.961406963299502
1.0248943063	0.8759711157	1.1991358166	SRPK2_	0.961406963299502
0.9766509351	0.8365416173	1.1402266538	STXBP3	0.961406963299502
0.9773074920	0.8381284438	1.1395985198	TACC3_	0.961406963299502
1.0225939561	0.8857108728	1.1806317741	TSPAN1	0.961406963299502
0.9771923345	0.8323505701	1.1472387872	USP47_	0.961406963299502
0.9737048560	0.8104062813	1.1699084380	VSIG10	0.961406963299502
1.0246767777	0.8620463562	1.2179884426	C2CD2I	0.962993009806185
1.0217059804	0.8776781783	1.1893688782	CCN1_(	0.962993009806185
1.0256170082	0.8568802346	1.2275814111	CEP290	0.962993009806185
1.0230060375	0.8718618162	1.2003523189	GIGYF2	0.962993009806185
1.0226189188	0.8713591180	1.2001360076	EP300_(	0.963780331937548
0.9788194720	0.8362988514	1.1456282131	ATP5IF1	0.964879963013072
0.9782429904	0.8294692097	1.1537008691	C2orf69	0.964879963013072
1.0218352205	0.8691091696	1.2013993806	CNTN3_	0.964879963013072
1.0214269533	0.8712064232	1.1975497347	CREB3_	0.964879963013072
1.0214128938	0.8729862566	1.1950752853	DCLRE1	0.964879963013072
0.9785682388	0.8355500847	1.1460663046	DDX53_	0.964879963013072
1.0207623014	0.8775382789	1.1873620798	EPPK1_	0.964879963013072
1.0195195729	0.8823319755	1.1780375056	GGACT	0.964879963013072
0.9801874861	0.8432534382	1.1393579490	PDLIM5	0.964879963013072
0.9777260291	0.8266242573	1.1564482649	SYNGA	0.964879963013072
1.0198447726	0.8829839860	1.1779187128	TBC1D1	0.964879963013072
1.0231435679	0.8583416063	1.2195875777	DNAJC(	0.966669554756957
0.9788346986	0.8294891685	1.1550691723	FARSA_	0.966669554756957
1.0221524867	0.8628422344	1.2108768724	GFOD2_	0.966669554756957
0.9785044248	0.8286362894	1.1554778877	GSAP_A	0.966669554756957
1.0206067802	0.8721721360	1.1943034601	MARS1_	0.966669554756957
0.9783793206	0.8247679021	1.1606005671	OPLAH_	0.967919728057063
0.9787930539	0.8216379509	1.1660072924	BCR_P1	0.968081606405713

0.980109429	0.831267271	1.155602448	CASP7_	0.968081606405713
0.980788207	0.837840201	1.148125270	CEND1_	0.968081606405713
0.980644974	0.839287141	1.145811150	EIF2AK	0.968081606405713
0.979553071	0.826031788	1.161606893	FKBP5_	0.968081606405713
1.018994444	0.872652922	1.189877042	ID4_P4	0.968081606405713
0.980149867	0.834058591	1.151830066	KAZN_	0.968081606405713
0.980759650	0.836619125	1.149734046	PTRHD	0.968081606405713
1.021372308	0.864198776	1.207131300	RECK_	0.968081606405713
0.980472986	0.834524514	1.151946121	SLC28A	0.968081606405713
1.019807402	0.867932970	1.198257439	VSIR_Q	0.968081606405713
1.018393151	0.877278242	1.182207150	WASF1_	0.968081606405713
0.981642014	0.841415986	1.145237386	GID8_Q	0.968483879921773
1.020919266	0.858960591	1.213415560	SLIRP_	0.968483879921773
0.982078953	0.843024384	1.144070193	CEP43_	0.969211430399485
0.981784736	0.840527570	1.146781263	SCP2_P	0.969211430399485
1.018982738	0.866450598	1.198367020	CLEC1	0.972019237702684
0.981100956	0.831910331	1.157046679	PLEKH	0.972019237702684
1.018529422	0.865224726	1.198997384	MESD_	0.973620354054839
1.018922143	0.863232854	1.202690942	POF1B_	0.973620354054839
0.979680517	0.817486071	1.174055375	ST8SIA	0.973620354054839
1.018245158	0.867359788	1.195378454	UPK3A_	0.973620354054839
1.018710314	0.862748023	1.202866511	IPCEF1_	0.974403221052043
0.982592804	0.838664029	1.151222165	TSPYL1	0.974630377618671
1.017555572	0.867841392	1.193097440	RRP15_	0.976360067448074
1.016458159	0.873880112	1.182298551	ADAM	0.976762470470252
0.982616606	0.835372806	1.155813771	SNX2_	0.976762470470252
0.982037907	0.829588344	1.162502412	YTHDF	0.976779966287618
1.021494219	0.837067746	1.246554350	DBH_P	0.976920680652894
0.983645435	0.839876561	1.152024460	OGT_O	0.977299012763423
0.983979504	0.843027354	1.148498514	RABGA	0.977299012763423
0.984584732	0.849626704	1.140980020	SDCCA	0.977299012763423
0.982372539	0.829106491	1.163970871	SPRY2_	0.977299012763423
0.983626707	0.838599511	1.153734872	ELAC1_	0.977844254928137
1.015734208	0.872671793	1.182249717	IKBKG_	0.97801857594093
1.016929445	0.860416017	1.201913349	FZD8_	0.979291555061332
1.015667765	0.870533966	1.184998002	MTDH_	0.979291555061332
0.983388076	0.833196798	1.160652693	TNFAIP	0.979291555061332
0.984617411	0.841770256	1.151705515	MED18_	0.980060039979015

0.983403033	0.830775394	1.164070977	PAIP2B_	0.980060039979015
0.985165091	0.843501797	1.150620259	CNPY2_	0.9837348351291
1.014415290	0.871778944	1.180389120	CACNB	0.984978860025132
1.015062997	0.866923286	1.188516798	FYB1_C	0.984978860025132
1.014571606	0.869613459	1.183693207	TIA1_P	0.985081955545384
1.014720422	0.867434114	1.187015266	NUMB_	0.98524749995148
1.015157891	0.862449800	1.194904960	GOPC_	0.985841960284573
1.014943821	0.853170975	1.207391003	AMFR_	0.986897584501532
1.012869013	0.869838593	1.179418396	CAMSA	0.986897584501532
0.986802408	0.843875743	1.153936465	ERVV-1	0.986897584501532
0.985328756	0.833734079	1.164487315	FOSB_	0.986897584501532
1.013946404	0.868138228	1.184243795	GTF2IR	0.986897584501532
1.013174893	0.866732451	1.184360137	KIRREL	0.986897584501532
1.014060825	0.860639321	1.194831947	NECAP	0.986897584501532
0.986492887	0.839074711	1.159811163	NUDT1	0.986897584501532
1.014412420	0.860088401	1.196426501	OXCT1_	0.986897584501532
1.014355778	0.862514368	1.192928121	PTPRM_	0.986897584501532
0.986113965	0.841691731	1.155316985	SAMD9	0.986897584501532
1.013196408	0.868740689	1.181672476	SLC39A	0.986897584501532
0.986280747	0.844269021	1.152179799	SPTBN	0.986897584501532
0.986675679	0.850717818	1.144361709	YOD1_	0.986897584501532
1.013290552	0.864285676	1.187984218	NCS1_P	0.987375492842747
0.986858472	0.839547275	1.160017633	GGCT_	0.988462044561523
0.987232135	0.840329167	1.159816090	DNPH1_	0.990448204813662
1.012790319	0.863282627	1.188190516	PGLYR	0.990448204813662
1.009104046	0.856195624	1.189320461	ACRBP_	0.990669295339903
1.008694174	0.863083809	1.178870378	ACSL1_	0.990669295339903
1.009036260	0.878645502	1.158776972	ACTA2_	0.990669295339903
1.008466506	0.857748362	1.185667893	ATP2B4	0.990669295339903
1.009155438	0.863230245	1.179748629	ATP5F1	0.990669295339903
1.009012438	0.864183582	1.178113218	ATP6A	0.990669295339903
0.988716164	0.841450955	1.161754762	AXIN1_	0.990669295339903
0.987595953	0.828245117	1.177605211	BTNL1	0.990669295339903
1.007871949	0.866797034	1.171907409	CDC26_	0.990669295339903
1.011127349	0.864465290	1.182671563	CEP152	0.990669295339903
1.009889536	0.866721605	1.176706417	CEP170	0.990669295339903
0.989504251	0.859578092	1.139068890	CGB3_	0.990669295339903
1.012915824	0.853292985	1.202398807	CHMP6	0.990669295339903

0.9908306207	0.8467484429	1.1594297305	CNGB3_	0.990669295339903
0.9899007693	0.8438675588	1.1612053608	COL9A2	0.990669295339903
1.0083551600	0.8659034120	1.1742419704	CORO1	0.990669295339903
1.0118923282	0.8511221446	1.2030307170	CPTP_Q	0.990669295339903
0.9913080734	0.8367398664	1.1744291575	DRG2_F	0.990669295339903
1.0125367036	0.8599533032	1.1921933114	EIF4G1_	0.990669295339903
1.0114607203	0.8653422999	1.1822521433	EPB41L	0.990669295339903
0.9884824750	0.8371518447	1.1671689068	FGD3_C	0.990669295339903
0.9902402564	0.8342315484	1.1754239782	GLYR1_	0.990669295339903
1.0135059518	0.8071422198	1.2726311289	HLA-A_	0.990669295339903
1.0097863531	0.8630119689	1.1815229864	HMMR_	0.990669295339903
1.0111020309	0.8650657258	1.1817914946	HSD17E	0.990669295339903
1.0088213778	0.8533873642	1.1925657855	KHDC3	0.990669295339903
0.9909243200	0.8459630438	1.1607256548	KRT6C_	0.990669295339903
0.9910111273	0.8476879188	1.1585667704	LACTB2	0.990669295339903
1.0085755033	0.8665059897	1.1739382738	MCFD2_	0.990669295339903
1.0100130979	0.8417849313	1.2118611537	METAP	0.990669295339903
1.0098901114	0.8479359484	1.2027772134	MTSS2_	0.990669295339903
1.0089616786	0.8659029825	1.1756555751	MZT1_C	0.990669295339903
0.9908286564	0.8424174287	1.1653859393	NAA80_	0.990669295339903
1.0109074591	0.8366853906	1.2214075952	NAGA_	0.990669295339903
0.9907568127	0.8442117843	1.1627402982	NCLN_C	0.990669295339903
0.9882336443	0.8464181484	1.1538100141	NECTIN	0.990669295339903
1.0126946973	0.8611017099	1.1909749315	NFKB2_	0.990669295339903
1.0096556957	0.8540148076	1.1936615321	NOS2_F	0.990669295339903
0.9916229353	0.8443131378	1.1646343067	PKD1_F	0.990669295339903
0.9916445372	0.8486101734	1.1587875317	PMS1_F	0.990669295339903
0.9903723648	0.8439168983	1.1622440822	PNMA1_	0.990669295339903
1.0101853165	0.8638601113	1.1812958606	PODXL_	0.990669295339903
0.9900375300	0.8424000981	1.1635496161	PSTPIP2	0.990669295339903
1.0092664154	0.8632176899	1.1800252811	RAB11F	0.990669295339903
0.9912061922	0.8444175912	1.1635116627	REEP4_	0.990669295339903
0.9919556262	0.8485146497	1.1596452279	RTKN2_	0.990669295339903
1.0090945308	0.8552327692	1.1906369924	SCRN1_	0.990669295339903
0.9880423676	0.8458679109	1.1541136714	SHD_Q9	0.990669295339903
0.9921051850	0.8539802727	1.1525707672	SNX9_C	0.990669295339903
1.0102938187	0.8695513367	1.1738163776	TTR_P0	0.990669295339903
1.0047408171	0.8596287950	1.1743488764	ABCA2_	0.991251699113008

1.002539637(	0.847992076;	1.185253674(	ARHGE	0.991251699113008
1.005965743{	0.856412065(	1.181635708:	ATP6V1	0.991251699113008
1.007349640:	0.858087278(	1.182575856:	BANK1_	0.991251699113008
1.005043168(	0.859392262:	1.175379176(	BLOC1S	0.991251699113008
0.996044312(	0.856934808;	1.157735992;	BLOC1S	0.991251699113008
1.002260616	0.857198259{	1.171871654;	BRME1_	0.991251699113008
1.002180552:	0.857319071{	1.171519323;	CBX2_(	0.991251699113008
0.997061232(	0.845466251:	1.175837709;	CCAR2_	0.991251699113008
1.004984924	0.859367298	1.175277091(	CLIP2_(	0.991251699113008
0.993887898:	0.853372783{	1.157540026	CLNS1/	0.991251699113008
1.007240802	0.862315152	1.176523491(	COL4A	0.991251699113008
1.008125129(	0.843862002	1.204363122(	CPA1_P	0.991251699113008
0.995823508	0.841971708;	1.177788337{	DAAM1	0.991251699113008
1.004406728:	0.836840027:	1.205526555:	DAND5	0.991251699113008
1.008012398(	0.853142273;	1.190995953	DDHD2	0.991251699113008
0.993783309:	0.849471508{	1.162611407:	DDX39/	0.991251699113008
0.996970643:	0.845778445{	1.175190108	DNPEP_	0.991251699113008
0.997145832:	0.843589360{	1.178653805{	EPHX2_	0.991251699113008
0.995872984;	0.845555619(	1.172912791(	ERBIN_	0.991251699113008
0.997085085	0.853585512:	1.164708928:	ERC2_C	0.991251699113008
0.994982561{	0.847435560(	1.168218971:	ESYT2_	0.991251699113008
0.996673861;	0.849268363(	1.169664182{	FAM13/	0.991251699113008
1.003171214	0.846578246{	1.188729439:	FNTA_I	0.991251699113008
0.996781668	0.849480568;	1.169624980{	FRMD4	0.991251699113008
1.007151466	0.861312411:	1.177684267:	FXYD5_	0.991251699113008
0.995253025(	0.850113236{	1.165172523	GART_I	0.991251699113008
1.006732589:	0.851797036:	1.189849768	GAS2_C	0.991251699113008
0.995957468(	0.845040744{	1.173826570	GIMAP;	0.991251699113008
0.996095208:	0.846394104{	1.172273836:	GLT8D:	0.991251699113008
0.992706343{	0.839569940(	1.173774616:	HCLS1_	0.991251699113008
1.003629178(	0.858751565	1.172948693;	IGF2BP	0.991251699113008
0.996957080(	0.853143367(	1.165013358:	IL33_O{	0.991251699113008
0.994996719	0.845418170:	1.171039973(	INPP1_I	0.991251699113008
1.002390045(	0.854540545:	1.175819928(	INPPL1_	0.991251699113008
1.005525598	0.857068982(	1.179697025	IRAK1_	0.991251699113008
0.993478760	0.850260997	1.160820089(	KIAA15	0.991251699113008
0.993364939{	0.849598936:	1.161458497(	KIF20B_	0.991251699113008
1.004860143:	0.859516041	1.174781922;	LMOD2	0.991251699113008

0.993545186(	0.848850868(	1.162903962(	LRP2BP	0.991251699113008
0.9936023357	0.844069729(	1.1696256442	MITD1_	0.991251699113008
1.002142779(	0.8592496201	1.1687990623	MMP15_	0.991251699113008
0.9955637182	0.851983720(	1.1633404407	MSLNL_	0.991251699113008
0.996916251(	0.845559561(	1.1753660643	MYCBP	0.991251699113008
0.994144728(	0.848054671(	1.165400974(	NEDD4I	0.991251699113008
1.007247413(	0.8666162311	1.170699688(	NFX1_C	0.991251699113008
0.9949738622	0.850660636(	1.1637695975	NXPE4_	0.991251699113008
0.996736040(	0.8543701657	1.1628247043	OSBPL2	0.991251699113008
0.993768621(	0.8356958817	1.1817409823	OTUD6I	0.991251699113008
0.997540206(	0.849748330(	1.171036679(	PDXDC	0.991251699113008
0.9923141472	0.8421446067	1.169261619(	PPM1F_	0.991251699113008
0.996414539(	0.8517553543	1.1656421415	RBM25_	0.991251699113008
0.9940008142	0.8459837443	1.1679156075	RGL2_C	0.991251699113008
1.0040070617	0.856793950(	1.1765141193	RILP_Q	0.991251699113008
0.9936167711	0.852405223(	1.158221771(	SEPTIN	0.991251699113008
0.99660252	0.845217910(	1.1751011985	SLITRK	0.991251699113008
1.003983266(	0.848167414(	1.1884238683	SLK_Q(	0.991251699113008
0.996627260(	0.852830674(	1.1646695261	TARM1_	0.991251699113008
0.9946935441	0.841560783(	1.1756907707	TBCC_(	0.991251699113008
1.005412702(	0.8594239815	1.1762002493	TDRKH_	0.991251699113008
1.0032977791	0.897025532(	1.122160291(	TEX101	0.991251699113008
1.002684954(	0.8585732015	1.1709859067	TNIP1_(	0.991251699113008
1.0076826655	0.8512748791	1.1928278155	TPD52L	0.991251699113008
0.9929856655	0.8490693865	1.1612955872	UBQLN	0.991251699113008
0.9930430597	0.849375384(	1.1610114162	UHRF2_	0.991251699113008
1.0028557222	0.858129499(	1.171990475(	VCPKM	0.991251699113008
0.996015775(	0.8373655635	1.184724412(	ZBTB16	0.991251699113008
1.0019263545	0.8575615033	1.170594081(	COPB2_	0.992087436840439
0.998043012(	0.8477211061	1.175020707(	MAP2_I	0.992087436840439
0.998212478(	0.850422502(	1.171686014(	PLA2G4	0.992087436840439
0.9982583182	0.8569422782	1.1628784053	ZBP1_Q	0.992087436840439
1.001485159(	0.855920393(	1.1718058503	HARS1_	0.992168507508817
1.001530983(	0.8592559461	1.1673638273	LATS1_	0.992168507508817
1.001627819(	0.845687411(	1.1863228372	MYH4_(	0.992168507508817
0.998732127(	0.836812793(	1.191982091(	COL4A1	0.994295108368138
0.9988257311	0.8425451713	1.1840941887	DNAJC(	0.994295108368138
0.9989627637	0.8500599985	1.1739484331	ILKAP_	0.994295108368138

1.00084227б	0.85156912‸	1.17628179б	CLASP1	0.995202487348797
0.99925557	0.85392070	1.16932603	DBN1_	0.995202487348797
1.00047043	0.85796699	1.16664289	CSH1_P	0.99695780698993
0.99961630	0.85107183	1.17408745	ESPL1_	0.997141810546866
1.00006371	0.85666526	1.16746582	MELTF_	0.999356270771135

Supplementary Table S7： UKB-PPP-specific proteins that remained significant

protein names
CEACAM6_P40199_OID30362_v1_Cardiometabolic_II
MZB1_Q8WU39_OID20732_v1_Inflammation
IGLC2_P0DOY2_OID30740_v1_Inflammation_II
PPL_O60437_OID30564_v1_Inflammation_II
CCN3_P48745_OID20299_v1_Cardiometabolic
CEACAM5_P06731_OID21171_v1_Oncology
QSOX1_O00391_OID30729_v1_Inflammation_II
VSTM1_Q6UX27_OID20851_v1_Neurology
ALPP_P05187_OID21383_v1_Oncology
TP53BP1_Q12888_OID30470_v1_Inflammation_II
VSIG4_Q9Y279_OID21144_v1_Neurology
TNFRSF10C_O14798_OID20265_v1_Cardiometabolic
SFTPA1_Q8IWL2_OID21165_v1_Oncology
CD302_Q8IX05_OID21328_v1_Oncology
FAM3C_Q92520_OID20233_v1_Cardiometabolic
CLUL1_Q15846_OID20198_v1_Cardiometabolic
PON3_Q15166_OID20777_v1_Inflammation
HNMT_P50135_OID20975_v1_Neurology
CD101_Q93033_OID31480_v1_Oncology_II
LTBP3_Q9NS15_OID21191_v1_Oncology
OCLN_Q16625_OID31069_v1_Neurology_II
ADGRG1_Q9Y653_OID21294_v1_Oncology
GCHFR_P30047_OID30631_v1_Inflammation_II
AHNAK_Q09666_OID30260_v1_Cardiometabolic_II
CLEC14A_Q86T13_OID21090_v1_Neurology
DPP6_P42658_OID21354_v1_Oncology
GPR37_O15354_OID20095_v1_Cardiometabolic
HEG1_Q9ULI3_OID30579_v1_Inflammation_II
CSTB_P04080_OID20294_v1_Cardiometabolic
C8B_P07358_OID30719_v1_Inflammation_II
SSC5D_A1L4H1_OID20351_v1_Cardiometabolic
PRAP1_Q96NZ9_OID30391_v1_Cardiometabolic_II
TCOF1_Q13428_OID30316_v1_Cardiometabolic_II
B4GAT1_O43505_OID21127_v1_Neurology
EXTL1_Q92935_OID30114_v1_Cardiometabolic_II
IGSF9_Q9P2J2_OID30330_v1_Cardiometabolic_II

FGF21_Q9NSA1_OID21402_v1_Oncology
GOLM2_Q6P4E1_OID21044_v1_Neurology
TGFA_P01135_OID20600_v1_Inflammation
ITGBL1_O95965_OID30408_v1_Cardiometabolic_II
RAB6A_P20340_OID20608_v1_Inflammation
PTPRB_P23467_OID30324_v1_Cardiometabolic_II
EFNA1_P20827_OID21125_v1_Neurology
ODAM_A1E959_OID20793_v1_Neurology
PGA4_P0DJD7_OID30699_v1_Inflammation_II
HEPH_Q9BQS7_OID30337_v1_Cardiometabolic_II
BPIFB2_Q8N4F0_OID30311_v1_Cardiometabolic_II
YAP1_P46937_OID31113_v1_Neurology_II
TNFRSF11A_Q9Y6Q6_OID20646_v1_Inflammation
NT5C1A_Q9BXI3_OID31102_v1_Neurology_II
SMAD5_Q99717_OID21353_v1_Oncology
GAST_P01350_OID31083_v1_Neurology_II
PLA2G15_Q8NCC3_OID21473_v1_Oncology
NPC2_P61916_OID30418_v1_Cardiometabolic_II
SFTPA2_Q8IWL1_OID21218_v1_Oncology
LAMP3_Q9UQV4_OID20638_v1_Inflammation
IFI30_P13284_OID31505_v1_Oncology_II
CDHR2_Q9BYE9_OID21282_v1_Oncology
GGT1_P19440_OID21075_v1_Neurology
SLURP1_P55000_OID30404_v1_Cardiometabolic_II
SHISA5_Q8N114_OID30387_v1_Cardiometabolic_II
SGSH_P51688_OID30341_v1_Cardiometabolic_II
TXNL1_O43396_OID30872_v1_Neurology_II
TREH_O43280_OID30388_v1_Cardiometabolic_II
CTBS_Q01459_OID30710_v1_Inflammation_II
ADGRD1_Q6QNK2_OID30588_v1_Inflammation_II
PAMR1_Q6UXH9_OID21153_v1_Neurology
PALM2_Q8IXS6_OID31096_v1_Neurology_II
VGF_O15240_OID31067_v1_Neurology_II
ADAM8_P78325_OID21039_v1_Neurology
CRELD2_Q6UXH1_OID20751_v1_Inflammation
LY6D_Q14210_OID20708_v1_Inflammation
HLA-DRA_P01903_OID20520_v1_Inflammation
TCTN3_Q6NUS6_OID30326_v1_Cardiometabolic_II

IMMT_Q16891_OID31397_v1_Oncology_II
ORM1_P02763_OID30768_v1_Inflammation_II
RIDA_P52758_OID30635_v1_Inflammation_II
LUZP2_Q86TE4_OID31402_v1_Oncology_II
RBP2_P50120_OID21365_v1_Oncology
CES2_O00748_OID21366_v1_Oncology
KIAA0319_Q5VV43_OID31094_v1_Neurology_II
CA14_Q9ULX7_OID21401_v1_Oncology
VNN1_O95497_OID30711_v1_Inflammation_II
RTBDN_Q9BSG5_OID21343_v1_Oncology
DCC_P43146_OID30953_v1_Neurology_II
CDH23_Q9H251_OID31074_v1_Neurology_II
SYT1_P21579_OID31089_v1_Neurology_II
RBFOX3_A6NFN3_OID30984_v1_Neurology_II

ly associated with smoking exposure after validation analyses

Supplementary Table S8: Causal association between lifetime smoking

method	nsnp	b	se	pval	lo_ci
Inverse v	109	0.4601974799	0.08762	1.503184558	0.2884613716
Inverse v	109	0.4432086678	0.09813	6.299303448	0.2508542810
Inverse v	109	0.3997110229	0.09119	1.170724077	0.2209660940
Inverse v	109	0.3935397190	0.09471	3.252506739	0.2078999986
Inverse v	109	0.3505301998	0.09266	0.000155160	0.1689017033
Inverse v	109	0.3350857160	0.09023	0.000204440	0.1582245204
Inverse v	109	0.2881219054	0.08176	0.000425168	0.1278700931
Inverse v	109	0.3176500848	0.09099	0.000481648	0.1392951784
Inverse v	109	0.2889367044	0.08445	0.000623703	0.1234000729
Inverse v	109	0.1782669862	0.08485	0.035645621	0.0119594599
Inverse v	109	0.2393155736	0.08780	0.006419860	0.0672173919
Inverse v	109	0.1866773952	0.08628	0.030507769	0.0175538655
Inverse v	109	0.1862910649	0.08670	0.031678353	0.0163400803
Inverse v	109	0.2070577685	0.08498	0.014833663	0.0404879267
Inverse v	109	0.2049725567	0.08485	0.015706380	0.0386640109
Inverse v	109	0.2664525328	0.08967	0.002963731	0.0906990801
Inverse v	109	0.1695843929	0.08199	0.038621139	0.0088720477
Inverse v	109	0.2114147457	0.08284	0.010716380	0.0490310638
Inverse v	109	0.1980189634	0.08394	0.018325116	0.0334916056
Inverse v	109	0.1963900659	0.08477	0.020524312	0.0302324150
Inverse v	109	0.1732315927	0.08362	0.038311917	0.0093244671
Inverse v	109	0.1746340345	0.08235	0.033957122	0.0132241223
Inverse v	109	-0.307659029	0.10870	0.004652680	-0.520726394
Inverse v	109	0.1753228686	0.08248	0.033536086	0.0136594013
Inverse v	109	0.1901655558	0.08421	0.023939464	0.0251044011
Inverse v	109	0.2723050520	0.09094	0.002751590	0.0940548908
Inverse v	109	0.1884805559	0.08627	0.028916266	0.0193798223
Inverse v	109	0.2240030367	0.09054	0.013358479	0.0465438076
Inverse v	109	0.1742111216	0.08403	0.038153774	0.0095121141
Inverse v	109	0.2307557325	0.09734	0.017766559	0.0399558078
Inverse v	109	0.1949907579	0.08700	0.025021761	0.0244550474
Inverse v	109	0.2035861809	0.08478	0.016338238	0.0374121253
Inverse v	109	0.2126527752	0.09221	0.021105423	0.0319144451
Inverse v	109	0.2183311180	0.08491	0.010137434	0.0518933970
Inverse v	109	0.2094958464	0.08440	0.013063958	0.0440613617
Inverse v	109	0.2095335784	0.08781	0.017027273	0.0374189694

Inverse v	109	0.26112570178	0.091157	0.00417586211	0.08245742703
Inverse v	109	0.21396877008	0.084278	0.01112200388	0.04878370185
Inverse v	109	0.18768487833	0.085341	0.02786271222	0.02041494984
Inverse v	109	0.20193946504	0.086654	0.01978522257	0.03209651358
Inverse v	109	0.19154455943	0.083434	0.02169105100	0.02801202016
Inverse v	109	0.27260222577	0.095372	0.00425924400	0.08567246923
Inverse v	109	0.19877894192	0.087708	0.02342964449	0.02686999828
Inverse v	109	0.22754386523	0.083841	0.00664820455	0.06321417084
Inverse v	109	0.18531896792	0.089774	0.03899131611	0.00936152169
Inverse v	109	0.18057480739	0.090541	0.04610897999	0.00311410118
Inverse v	109	0.17554989283	0.087188	0.04406808998	0.00465960116
Inverse v	109	0.18633328188	0.091790	0.04235793477	0.00642406890
Inverse v	109	0.20849440573	0.083298	0.01231484333	0.04523016641
Inverse v	109	0.23291444390	0.087165	0.00753785977	0.06207030391
Inverse v	109	0.18946908375	0.088230	0.03175809155	0.01653805048
Inverse v	109	0.18189887897	0.088809	0.04054062000	0.00783278079
Inverse v	109	0.20825800795	0.089259	0.01963855799	0.03330998775
Inverse v	109	0.21894748500	0.086142	0.01103199833	0.05010779625
Inverse v	109	0.17120542493	0.085233	0.04457356177	0.00414718873
Inverse v	109	0.16909807602	0.083087	0.04183361044	0.00624584752
Inverse v	109	0.17469795546	0.085149	0.04020314933	0.00780444719
Inverse v	109	0.18534204114	0.082047	0.02388520255	0.02452969072
Inverse v	109	0.18587891278	0.086947	0.03253156332	0.01546086733
Inverse v	109	0.22971394098	0.093659	0.01418121000	0.04614061354
Inverse v	109	0.18124478355	0.090857	0.04606257111	0.00316361980
Inverse v	109	0.19757340533	0.085314	0.02056747888	0.03035748325
Inverse v	109	0.18520355315	0.089502	0.03852310777	0.00977774559
Inverse v	109	0.20271713933	0.091425	0.02660261022	0.02352369438
Inverse v	109	0.21803677493	0.090007	0.01541696611	0.04162237268
Inverse v	109	0.16681188595	0.083756	0.04641285055	0.00264833017
Inverse v	109	0.23891841976	0.091310	0.00888260866	0.05994917322
Inverse v	109	0.20624807193	0.083939	0.01400578955	0.04172738709
Inverse v	109	0.20818182403	0.091064	0.02224924533	0.02969491296
Inverse v	109	0.19061507036	0.085662	0.02606857400	0.02271670733
Inverse v	109	0.18566046734	0.082060	0.02367839355	0.02480944707
Inverse v	109	0.17672768179	0.089244	0.04767371176	0.00180857182
Inverse v	109	0.19161531710	0.085851	0.02561975598	0.02334549924
Inverse v	109	0.17214367989	0.083023	0.03813123211	0.00941825400

Inverse	109	0.17371108293	0.083413	0.03729463655	0.01022018016
Inverse	109	0.17620558392	0.085218	0.03867011660	0.00917638611
Inverse	109	0.18388360452	0.083333	0.02734117588	0.02055092069
Inverse	109	0.21056328522	0.087623	0.01625780004	0.03882339476
Inverse	109	0.18890496759	0.083727	0.02405897552	0.02479894484
Inverse	109	0.23263303155	0.097870	0.01745617455	0.04080753736
Inverse	109	0.16985859128	0.082849	0.04034412559	0.00747369205
Inverse	109	0.22512847450	0.102313	0.02777818960	0.02459636525
Inverse	109	0.21044356563	0.088301	0.01716059133	0.03737334880
Inverse	109	0.21928875920	0.092524	0.01778494559	0.03794102330
Inverse	109	0.20523277463	0.092925	0.02720363637	0.02309962394
Inverse	109	0.17916802984	0.088621	0.04320414133	0.00547067599
Inverse	109	0.24133345741	0.082998	0.00364115044	0.07865641511
Inverse	109	0.17660132309	0.083933	0.03537026088	0.01209412079
Inverse	109	0.18068412249	0.083860	0.03119558144	0.01631672962
Inverse	109	0.18882435497	0.086048	0.02820661659	0.02016977024
Inverse	109	0.17742361140	0.082428	0.03136134611	0.01586395561
Inverse	109	0.18443071711	0.082472	0.02533360337	0.02278520737
Inverse	109	0.17549797239	0.083598	0.03579028860	0.01164540208
Inverse	109	0.16802271610	0.083810	0.04499986059	0.00374287818
Inverse	109	0.16871988007	0.084678	0.04631805859	0.00275062458
Inverse	109	0.21387467871	0.082919	0.00989965900	0.05135306357
Inverse	109	0.21757238382	0.101744	0.03248343444	0.01815236781
Inverse	109	0.20816551621	0.096882	0.03166282955	0.01827603845
Inverse	109	0.19960850834	0.092563	0.03104890288	0.01818370786
Inverse	109	0.17509994977	0.086045	0.04185331911	0.00645130037
Inverse	109	0.24201690070	0.093839	0.00990698882	0.05809201293
Inverse	109	0.18728628473	0.088853	0.03504612477	0.01313467896
Inverse	109	0.19802116480	0.088693	0.02557204777	0.02418222057
Inverse	109	0.21578706557	0.092663	0.01987397552	0.03416673635
Inverse	109	0.18703697777	0.089272	0.03616092557	0.01206196141
Inverse	109	0.20652433800	0.087130	0.01778262677	0.03573601615
Inverse	109	0.18918596035	0.090338	0.03624301733	0.01212256068
Inverse	109	0.18232746401	0.084667	0.03128403060	0.01637826104
Inverse	109	0.23217911000	0.092973	0.01251575770	0.04995087959
Inverse	109	0.19110925935	0.094618	0.04340602300	0.00565648251
Inverse	109	0.21992393109	0.093470	0.01863683632	0.03670980312
Inverse	109	0.16744123214	0.083770	0.04562863833	0.00325134178

Inverse v	109	0.1696087798	0.08454	0.044849065	0.0038948955
Inverse v	109	0.2047837774	0.09054	0.023711715	0.0273225474
Inverse v	109	0.2132595344	0.09013	0.017978793	0.0365989073
Inverse v	109	0.1894392700	0.09064	0.036625429	0.0117760840
Inverse v	109	0.1958373379	0.09710	0.043711327	0.0055200258
Inverse v	109	0.1877376453	0.09120	0.039550629	0.0089756488
Inverse v	109	0.1908904312	0.08310	0.021615876	0.0280097238
Inverse v	109	0.1969770149	0.08714	0.023800837	0.0261722587
Inverse v	109	0.2068857783	0.08335	0.013067964	0.0435052196
Inverse v	109	0.2411822731	0.08739	0.005785535	0.0698889732
Inverse v	109	0.2107924458	0.08415	0.012250349	0.0458511237
Inverse v	109	0.1787613598	0.09009	0.047235950	0.0021783184
Inverse v	109	0.1820457856	0.08227	0.026926750	0.0207815349
Inverse v	109	0.1845831327	0.08829	0.036577143	0.0115187421
Inverse v	109	0.1700420662	0.08308	0.040700640	0.0071925292
Inverse v	109	0.1720907544	0.08515	0.043298648	0.0051791656
Inverse v	109	0.2469437940	0.08293	0.002905814	0.0843896387
Inverse v	109	0.1941889433	0.09487	0.040673014	0.0082394564
Inverse v	109	0.2258134154	0.08691	0.009372677	0.0554638130
Inverse v	109	0.1873932856	0.08384	0.025409216	0.0230664485
Inverse v	109	0.2308661081	0.09179	0.011898776	0.0509560509
Inverse v	109	0.1925280744	0.08331	0.020845012	0.0292260574
Inverse v	109	0.1639742177	0.08191	0.045304199	0.0034255049
Inverse v	109	0.2100095459	0.09812	0.032343524	0.0176768590
Inverse v	109	0.1782915808	0.08512	0.036226241	0.0114394890
Inverse v	109	0.1817161346	0.08363	0.029795643	0.0177966707
Inverse v	109	0.2745980185	0.08819	0.001848042	0.1017409412
Inverse v	109	0.2014777271	0.08910	0.023756918	0.0268250299
Inverse v	109	0.1864735816	0.08849	0.035106250	0.0130205454
Inverse v	109	0.1894708774	0.08610	0.027778936	0.0206997938
Inverse v	109	0.1786325091	0.08061	0.026695055	0.0206325478
Inverse v	109	0.2134682140	0.08605	0.013119776	0.0447935631
Inverse v	109	0.1846609901	0.08710	0.034012840	0.0139301898
Inverse v	109	0.1905755468	0.09415	0.042954119	0.0060403128
Inverse v	109	0.1839703718	0.08529	0.031011849	0.0167959641
Inverse v	109	0.2659304756	0.08604	0.001997686	0.0972810925
Inverse v	109	0.1861209426	0.08530	0.029126539	0.0189185745
Inverse v	109	0.1924857782	0.09283	0.038135255	0.0105274057

Inverse v	109	0.18276423633	0.081967	0.0257660039	0.02210826937
Inverse v	109	0.16942814014	0.083135	0.0415515780	0.00648317687
Inverse v	109	0.18835014561	0.089093	0.0345093858	0.01372656017
Inverse v	109	0.16639057636	0.082967	0.0449099832	0.00377472908
Inverse v	109	0.22017075514	0.090128	0.0145712804	0.04351953208
Inverse v	109	0.17566980946	0.085028	0.0388280269	0.00901345533
Inverse v	109	0.19197787229	0.083580	0.0216238529	0.02815931515
Inverse v	109	0.21181165810	0.089757	0.0182840533	0.03588648615
Inverse v	109	0.22534515938	0.087091	0.0096688677	0.05464575347
Inverse v	109	0.18923391503	0.082948	0.0225275353	0.02665566135
Inverse v	109	0.22244295589	0.087780	0.0112742961	0.05039306850
Inverse v	109	0.22114667325	0.085003	0.0092782246	0.05454046978
Inverse v	109	0.21524670250	0.087258	0.0136334198	0.04422067468
Inverse v	109	0.19358818520	0.090773	0.0329532768	0.01567222558
Inverse v	109	0.16956684652	0.083220	0.0415945130	0.00645413952
Inverse v	109	0.17680051882	0.086273	0.0404331492	0.00770392931
Inverse v	109	0.19776899660	0.098697	0.0450925818	0.00432194280
Inverse v	109	0.18634105636	0.088830	0.0359302077	0.01223340509
Inverse v	109	0.20825266700	0.088833	0.0190629844	0.03413853947
Inverse v	109	0.19182515584	0.089188	0.0314939113	0.01701505687
Inverse v	109	0.22053291913	0.083675	0.0083999131	0.05652796719
Inverse v	109	0.20585212203	0.088334	0.0197863510	0.03271681310
Inverse v	109	0.19233078125	0.087119	0.0272668532	0.02157732939
Inverse v	109	0.19190411811	0.096633	0.0470414394	0.00250537020
Inverse v	109	0.21214771899	0.087760	0.0156415865	0.04012463617
Inverse v	109	0.20411492655	0.088420	0.0209732226	0.03081143078
Inverse v	109	0.17276607761	0.086450	0.0456679085	0.00332399376
Inverse v	109	0.18480805838	0.089870	0.0397464234	0.00866126302
Inverse v	109	0.18326804006	0.088428	0.0382181591	0.00994881267
Inverse v	109	0.19266734973	0.087763	0.0281424106	0.02065041030
Inverse v	109	0.21447955498	0.092949	0.0210282728	0.03229787976
Inverse v	109	0.19715275746	0.089188	0.0270691117	0.02234345956
Inverse v	109	0.18044240739	0.088295	0.0409915349	0.00738289710
Inverse v	109	0.22391351282	0.090910	0.0137835906	0.04571763606
Inverse v	109	0.20119128335	0.087251	0.0211179161	0.03017775143
Inverse v	109	0.20833973827	0.086948	0.0165688156	0.03792144265
Inverse v	109	0.19692545727	0.087910	0.0250866173	0.02462078401
Inverse v	109	0.18096821261	0.089343	0.0428124334	0.00585514159

Inverse v	109	0.21480924918	0.085712	0.012204783	0.04681323493
Inverse v	109	0.20040792497	0.084127	0.017209809	0.03551813556
Inverse v	109	0.19767081529	0.089123	0.026558999	0.02298845605
Inverse v	109	0.31868675912	0.106894	0.002870188	0.10917275109
Inverse v	109	0.18449993000	0.085949	0.031824919	0.01603845789
Inverse v	109	0.16974851688	0.085154	0.046214989	0.00284623262
Inverse v	109	0.17921107094	0.084272	0.033456887	0.01403625632
Inverse v	109	0.23279073303	0.083087	0.005082767	0.06993847302
Inverse v	109	0.17134526674	0.084684	0.043037515	0.00536437777
Inverse v	109	0.17786812534	0.083909	0.034025122	0.01340646451
Inverse v	109	0.18709669774	0.089179	0.035907979	0.01230397391
Inverse v	109	0.21360733483	0.086935	0.014007125	0.04321391085
Inverse v	109	0.18547818002	0.084221	0.027646391	0.02040415042
Inverse v	109	0.20822307438	0.090410	0.021282201	0.03100735476
Inverse v	109	0.19346319108	0.086661	0.025589052	0.02360600661
Inverse v	109	0.17400739885	0.087205	0.046002460	0.00308452425
Inverse v	109	0.18728943457	0.083622	0.025110334	0.02338930821
Inverse v	109	0.21201007578	0.082037	0.009757498	0.05121656392
Inverse v	109	0.16721333753	0.083610	0.045525448	0.00332515936
Inverse v	109	0.20862789878	0.101587	0.040006809	0.00951656607
Inverse v	109	0.26951317628	0.101383	0.007852116	0.07080226346
Inverse v	109	0.21215682376	0.087391	0.015196469	0.04086969422
Inverse v	109	0.17867019760	0.090033	0.047200878	0.00220520542
Inverse v	109	0.17391439854	0.086490	0.044348618	0.00439207842
Inverse v	109	0.24765861334	0.108405	0.022339237	0.03518310646
Inverse v	109	0.17167506253	0.084250	0.041582912	0.00654377711
Inverse v	109	0.19002485787	0.088543	0.031864115	0.01647898691
Inverse v	109	0.24090719411	0.086553	0.005380286	0.07126260155
Inverse v	109	0.19486120802	0.083542	0.019676080	0.03111707234
Inverse v	109	0.22279032994	0.083288	0.007474372	0.05954560322
Inverse v	109	0.20804646658	0.084683	0.014019115	0.04206815410
Inverse v	109	0.20294845947	0.093869	0.030616743	0.01896346321
Inverse v	109	0.16870906370	0.081359	0.038115251	0.00924356116
Inverse v	109	0.17508142264	0.084163	0.037500249	0.01012271848
Inverse v	109	0.24032053359	0.082724	0.003671830	0.07817989205
Inverse v	109	0.18126207584	0.087871	0.039130800	0.00903395972
Inverse v	109	0.17552559405	0.087505	0.044868448	0.00401523808
Inverse v	109	0.18843439926	0.087341	0.030970832	0.01724531385

Inverse v	109	0.19390629069	0.08779	0.02719998 3	0.0218288797
Inverse v	109	0.1768042929	0.08284	0.0328246633	0.0144329394
Inverse v	109	-0.199125515	0.08332	0.016858727	-0.36244052
Inverse v	109	0.1756086579	0.08494	0.0387057582	0.0091148113
Inverse v	109	0.2028265267	0.08798	0.0211527290	0.0303764827
Inverse v	109	0.1853797693	0.08749	0.0341148328	0.0138867174
Inverse v	109	0.2295026066	0.08677	0.0081731171	0.0594256835
Inverse v	109	0.1958539591	0.08721	0.0247192322	0.0249214671
Inverse v	109	0.1950482102	0.09325	0.0364763215	0.0122701487
Inverse v	109	-0.21697599	0.08561	0.0112659320	-0.384780198
Inverse v	109	0.1789694065	0.08357	0.0322419103	0.0151605565
Inverse v	109	0.2493725600	0.08928	0.0052201397	0.0743821499
Inverse v	109	0.1695972380	0.08392	0.0433099178	0.0050952643
Inverse v	109	0.1662216031	0.08313	0.0455615581	0.0032782121
Inverse v	109	0.2395641545	0.10641	0.0243665607	0.0309976365
Inverse v	109	0.1962546724	0.09889	0.0471989113	0.0024239642
Inverse v	109	0.2124396921	0.08449	0.0119296911	0.0468287878
Inverse v	109	0.2119210832	0.09008	0.0186455220	0.0353609600
Inverse v	109	0.2242417692	0.08520	0.0084897649	0.0572497168
Inverse v	109	0.2609284269	0.09345	0.0052385285	0.0777543940
Inverse v	109	0.1689057609	0.08314	0.0422155316	0.0059358606
Inverse v	109	0.1804236582	0.08886	0.0423253297	0.0062478576
Inverse v	109	0.2314920577	0.08879	0.0091311181	0.0574582845
Inverse v	109	0.2101583390	0.08460	0.0129898769	0.0443360363
Inverse v	109	0.2099294822	0.09124	0.0214084303	0.0310877090
Inverse v	109	0.1870796886	0.08255	0.0234376847	0.0252791677
Inverse v	109	0.1745092572	0.08249	0.0343865123	0.0128280735
Inverse v	109	0.1892281239	0.08756	0.0306869042	0.0176092528
Inverse v	109	0.1772082471	0.08643	0.0403500215	0.0077920790
Inverse v	109	0.2011223227	0.08305	0.0154557012	0.0383321893
Inverse v	109	0.1955787394	0.08166	0.0166195004	0.0355239271
Inverse v	109	0.1772755268	0.08211	0.0308519295	0.0163381998
Inverse v	109	0.1966239424	0.08407	0.0193523295	0.0318369803
Inverse v	109	0.1798479304	0.08314	0.0305267690	0.0168930501
Inverse v	109	0.1837844808	0.09064	0.0426057169	0.0061233881
Inverse v	109	0.2134900808	0.09477	0.0242904376	0.0277232405
Inverse v	109	0.1769130962	0.08609	0.0398971623	0.0081630300
Inverse v	109	0.2145337505	0.09394	0.0223944354	0.0304015785

Inverse v	109	0.22987043451	0.08596(	0.007496279(	0.06137610175
Inverse v	109	0.20566308759	0.08885	0.02063289835	0.03151053373
Inverse v	109	0.19720367267	0.08635	0.02239233360	0.02794838157
Inverse v	109	0.25282838443	0.09335(	0.00676160311	0.06986042829
Inverse v	109	0.18838751693	0.08430(	0.02543610660	0.02315855602
Inverse v	109	0.19678746221	0.09062	0.02990169444	0.01915773977
Inverse v	109	0.18417713101	0.08837	0.03716540544	0.01095392968
Inverse v	109	0.18081531811	0.08360	0.03057028260	0.01694106657
Inverse v	109	0.20503913563	0.08230	0.01273462060	0.04371450938
Inverse v	109	0.21537951779	0.08387	0.01023341077	0.05098309741
Inverse v	109	0.18709922908	0.08435	0.02655265077	0.02176595649
Inverse v	109	0.17952711111	0.08268	0.02992136222	0.01745797163
Inverse v	109	0.22934767421	0.09081(	0.01155125411	0.05135917960
Inverse v	109	0.18186829025	0.09040	0.04424318211	0.00468095798
Inverse v	109	0.25126276089	0.09321(	0.00702896555	0.06855799077
Inverse v	109	0.23338833156	0.08346	0.00516964633	0.06979847517
Inverse v	109	0.19107168492	0.08286	0.02112585288	0.02864985856
Inverse v	109	0.17961866984	0.08805	0.04136376188	0.00703245823
Inverse v	109	0.19800459102	0.08515	0.02005871760	0.03110237498
Inverse v	109	0.21977117853	0.08326	0.00830377388	0.05657446863
Inverse v	109	0.21839502803	0.08364	0.00903041300	0.05444613973
Inverse v	109	0.19055927592	0.09361	0.04180209955	0.00706679785
Inverse v	109	0.20933033380	0.09009	0.02015879722	0.03273949735
Inverse v	109	0.20769627370	0.08517	0.01475491622	0.04074465559
Inverse v	109	0.20860119881	0.09596	0.02972103333	0.02051557010
Inverse v	109	0.20956206630	0.08939	0.01906236669	0.03435409024
Inverse v	109	0.23591523488	0.08759	0.00707408277	0.06423454932
Inverse v	109	0.18255624293	0.08500(	0.03173738011	0.01595486230
Inverse v	109	0.17455904898	0.08667	0.04401010311	0.00467987430
Inverse v	109	0.19644550941	0.08525	0.02121568577	0.02933939699
Inverse v	109	0.16360539558	0.08242(	0.04714218733	0.00206219215
Inverse v	109	0.19568184151	0.08424(	0.02019354055	0.03055888817
Inverse v	109	0.17407217597	0.08416	0.03862639133	0.00910237611
Inverse v	109	0.17193352757	0.08251	0.03719694622	0.01019884344
Inverse v	109	0.21124703589	0.09541	0.02683744112	0.02422457088
Inverse v	109	0.19319852803	0.08353	0.02073868664	0.02946446475
Inverse v	109	0.17890642952	0.08455	0.03435757400	0.01317794426
Inverse v	109	0.19003993990	0.08569	0.02658744633	0.02206943300

Inverse v	109	0.16812307412	0.083864	0.04499483760	0.00374896412
Inverse v	109	0.23390201643	0.098001	0.01699944181	0.04181916234
Inverse v	109	-0.215153109	0.084743	0.01112067888	-0.381249766
Inverse v	109	0.19240759407	0.083073	0.02055282800	0.02958261235
Inverse v	109	0.16641936515	0.083371	0.04592212460	0.00301042638
Inverse v	109	0.20001349683	0.086335	0.02052054733	0.03079525290
Inverse v	109	0.17627223313	0.088564	0.04655582200	0.00268518413
Inverse v	109	0.18926704605	0.084971	0.02591993944	0.02272224877
Inverse v	109	0.17443126964	0.082458	0.03439647855	0.01281339886
Inverse v	109	0.23344599919	0.083492	0.00517380722	0.06980050530
Inverse v	109	0.21771662389	0.090570	0.01622437999	0.04019789834
Inverse v	109	0.17803477240	0.086550	0.03968533364	0.00839617958
Inverse v	109	0.16409209111	0.082483	0.04665865933	0.00242383493
Inverse v	109	0.23831311300	0.090360	0.00836002111	0.06119400798
Inverse v	109	0.17885420257	0.084933	0.03522116555	0.01238390243
Inverse v	109	0.21256377427	0.090040	0.01824486844	0.03607343448
Inverse v	109	0.19402224574	0.090980	0.03295929888	0.01570123257
Inverse v	109	0.18985431548	0.088999	0.03290864711	0.01541443758
Inverse v	109	0.26050506114	0.096413	0.00689320400	0.07153486649
Inverse v	109	0.21286971930	0.084140	0.01141407799	0.04794310361
Inverse v	109	0.19672720764	0.083427	0.01837094044	0.03320887312
Inverse v	109	0.16703944031	0.084290	0.04752800068	0.00181781485
Inverse v	109	0.18312245982	0.084201	0.02964420711	0.01808749565
Inverse v	109	0.18849411230	0.083702	0.02432607181	0.02443624186
Inverse v	109	0.22593689028	0.086938	0.00935445888	0.05553795303
Inverse v	109	-0.286118085	0.088092	0.00116253677	-0.458780028
Inverse v	109	0.25742490113	0.083700	0.00210271477	0.09336016496
Inverse v	109	0.19568106215	0.088472	0.02698252633	0.02227478800
Inverse v	109	0.19469787859	0.097642	0.04615350144	0.00331855262
Inverse v	109	0.20026044432	0.089167	0.02471062077	0.02549262959
Inverse v	109	0.20186790611	0.083000	0.01501760688	0.03917524574
Inverse v	109	0.18616272413	0.085975	0.03036404260	0.01765145479
Inverse v	109	0.21964352342	0.085613	0.01030229255	0.05184041355
Inverse v	109	0.19110850158	0.091160	0.03605984299	0.01242165262
Inverse v	109	0.18687316581	0.094028	0.04687717260	0.00257716001
Inverse v	109	0.17900878153	0.087254	0.04021120999	0.00799012056
Inverse v	109	0.20293753382	0.088407	0.02170545944	0.02965915802
Inverse v	109	0.19524053280	0.090080	0.03021459422	0.01867176682

Inverse v	109	0.18469476008	0.084548	0.0289263169	0.01898017436
Inverse v	109	0.25654126059	0.088175	0.0036206545	0.08371791123
Inverse v	109	0.20477877293	0.087915	0.0198448326	0.03246443848
Inverse v	109	0.19206627837	0.084680	0.0233208382	0.02609312413
Inverse v	109	0.24491183176	0.086170	0.0044835510	0.07600542152
Inverse v	109	0.21838243244	0.092321	0.0180072757	0.03743325885
Inverse v	109	0.26072931267	0.086962	0.0027159060	0.09028317491
Inverse v	109	0.22074751760	0.087044	0.0112113059	0.05014111069
Inverse v	109	0.20389932670	0.088512	0.0212433687	0.03041551427
Inverse v	109	0.19112269448	0.089080	0.0319231746	0.01651399755
Inverse v	109	0.18496051751	0.084585	0.0287662308	0.01917339642
Inverse v	109	0.20416967747	0.090890	0.0246826869	0.02602486569
Inverse v	109	0.22458701657	0.086968	0.0098118273	0.05412851281
Inverse v	109	0.23801997863	0.087429	0.0064803644	0.06665871073
Inverse v	109	0.18862815789	0.084630	0.0258348253	0.02274061619
Inverse v	109	0.17885886159	0.086958	0.0397037053	0.00841922400
Inverse v	109	0.25098498939	0.093075	0.0070059200	0.06855623531
Inverse v	109	0.20530455290	0.087541	0.0190153524	0.03372353637
Inverse v	109	0.24859662310	0.091608	0.0066541725	0.06904319052
Inverse v	109	0.23197163823	0.088843	0.0090271242	0.05783911300
Inverse v	109	0.18754820964	0.089428	0.0359774719	0.01226800019
Inverse v	109	0.17265570360	0.084530	0.0410995538	0.00697582263
Inverse v	109	0.17124092751	0.083783	0.0409677132	0.00702577965
Inverse v	109	0.20525041969	0.088447	0.0203088513	0.03189347080
Inverse v	109	0.20991340440	0.088520	0.0177225157	0.03641406611
Inverse v	109	0.21849331170	0.091347	0.0167615493	0.03945261479
Inverse v	109	0.17791211549	0.085059	0.0364724300	0.01119561101
Inverse v	109	-0.186190548	0.091867	0.0426909712	-0.366251595
Inverse v	109	0.24890060240	0.095771	0.0093567309	0.06117668207
Inverse v	109	0.28523845412	0.092498	0.0020443240	0.10394182818
Inverse v	109	0.17983795933	0.084092	0.0324708297	0.01501611491
Inverse v	109	0.26297168590	0.081578	0.0012661786	0.10307760719
Inverse v	109	0.17087793308	0.085279	0.0450879160	0.00373791099
Inverse v	109	0.20866465016	0.085932	0.0151727413	0.04023628491
Inverse v	109	0.20079031744	0.087940	0.0224161658	0.02842612479
Inverse v	109	0.23055786625	0.084931	0.0066350263	0.06409180705
Inverse v	109	-0.22688557	0.085714	0.0081209398	-0.394885476
Inverse v	109	-0.173050701	0.085545	0.0430814303	-0.340718938

Inverse	109	0.2123582129	0.08346	0.010951535	0.0487644241
Inverse	109	0.2183002724	0.09220	0.017908174	0.0375752481
Inverse	109	0.1839373173	0.09286	0.047615808	0.0019297692
Inverse	109	0.1873093092	0.09151	0.040688878	0.0079334001
Inverse	109	0.1826736616	0.08904	0.040218181	0.0081476074
Inverse	109	0.2346389497	0.09372	0.012295732	0.0509422233
Inverse	109	0.1929443477	0.09039	0.032804751	0.0157706974
Inverse	109	0.2757260151	0.09277	0.002960278	0.0938776302
Inverse	109	0.208162989	0.08867	0.018897325	0.0343650207
Inverse	109	0.2028037555	0.08999	0.024226922	0.0264144285
Inverse	109	0.2782368290	0.08385	0.000906783	0.1138754098
Inverse	109	0.1902892849	0.08452	0.024360550	0.0246288607
Inverse	109	0.1947200775	0.08995	0.030421309	0.0184019576
Inverse	109	0.1972864094	0.09029	0.028896483	0.0203070988
Inverse	109	0.1908817919	0.08697	0.028190297	0.0204071956
Inverse	109	0.2073610983	0.09288	0.025591080	0.0252992979
Inverse	109	0.1848835530	0.09019	0.040385039	0.0080986307
Inverse	109	-0.21895661	0.08318	0.008487837	-0.382008043
Inverse	109	0.1880256955	0.08075	0.019897445	0.0297410500
Inverse	109	0.1692813904	0.08444	0.045008529	0.0037642267
Inverse	109	-0.220523103	0.08287	0.007789560	-0.382949224
Inverse	109	0.2038161552	0.09402	0.030180813	0.0195296051
Inverse	109	0.1776146174	0.08397	0.034415061	0.0130302674
Inverse	109	0.1801350023	0.08789	0.040421973	0.0078588410
Inverse	109	0.2320451793	0.08132	0.004328539	0.0726405588
Inverse	109	0.2452728287	0.08676	0.004698539	0.0752220117
Inverse	109	0.2063575399	0.09242	0.025565229	0.0252086370
Inverse	109	0.2042446912	0.09583	0.033069422	0.0164105185
Inverse	109	0.1861262046	0.08684	0.032091560	0.0159154286
Inverse	109	0.1844522736	0.09200	0.044972853	0.0041315775
Inverse	109	0.1705816118	0.08499	0.044749117	0.0039951120
Inverse	109	0.2061768977	0.08488	0.015146236	0.0398000174
Inverse	109	0.1782929493	0.08338	0.032508386	0.0148517186
Inverse	109	0.1781251479	0.08531	0.036813323	0.0109058005
Inverse	109	0.1908703475	0.09147	0.036935509	0.0115700118
Inverse	109	0.2537710313	0.08735	0.003672550	0.0825519248
Inverse	109	0.1693025264	0.08372	0.043153164	0.0052094933
Inverse	109	0.1880726203	0.09004	0.036743705	0.0115801098

Inverse v	109	0.17320170604	0.083183	0.03732812486	0.01016148764
Inverse v	109	0.26867327339	0.087920	0.00224402299	0.09634978458
Inverse v	109	0.25845373012	0.081113	0.00144098699	0.09947081210
Inverse v	109	0.16801122041	0.084369	0.04643971988	0.00264705815
Inverse v	109	0.26051331207	0.084692	0.00209804631	0.09451601286
Inverse v	109	0.20796932529	0.096574	0.03128321511	0.01868255212
Inverse v	109	0.21075951520	0.091044	0.02061833358	0.03231188327
Inverse v	109	0.20291488515	0.085772	0.01799486022	0.03480009362
Inverse v	109	0.18559309450	0.088171	0.03529911951	0.01277695576
Inverse v	109	0.19399522090	0.086462	0.02485301299	0.02452779567
Inverse v	109	0.24091326289	0.087050	0.00564833344	0.07029524407
Inverse v	109	0.17282771072	0.087103	0.04723787100	0.00210453010
Inverse v	109	0.17385983771	0.082683	0.03549035988	0.01180046677
Inverse v	109	0.17867311303	0.088558	0.04363613700	0.00509825651
Inverse v	109	0.19917140555	0.084017	0.01776009667	0.03449626222
Inverse v	109	0.18637877968	0.087441	0.03305027433	0.01499374481
Inverse v	109	0.17642376645	0.084889	0.03768358733	0.01004069961
Inverse v	109	0.22526491437	0.088912	0.01129116155	0.05099634163
Inverse v	109	0.16476427707	0.083180	0.04761309992	0.00173060377
Inverse v	109	0.25007108038	0.086932	0.00401972722	0.07968322605
Inverse v	109	0.20048246363	0.089131	0.02449439755	0.02578441255
Inverse v	109	0.16873902291	0.083031	0.04213052260	0.00599724715
Inverse v	109	0.20905052443	0.088121	0.01767826160	0.03633166760
Inverse v	109	0.17772319991	0.088968	0.04576049933	0.00334484350
Inverse v	109	0.20464081984	0.084149	0.01502157155	0.03970688336
Inverse v	109	0.24970087030	0.088401	0.00473357337	0.07643418153
Inverse v	109	0.21317763258	0.087618	0.01497309488	0.04144596321
Inverse v	109	0.19101211704	0.084453	0.02371281977	0.02548380546
Inverse v	109	0.22000592740	0.084964	0.00961508122	0.05347465664
Inverse v	109	0.22479061660	0.086561	0.00940738722	0.05512969887
Inverse v	109	0.22875366561	0.083939	0.00642578337	0.06423245459
Inverse v	109	0.21036462272	0.086784	0.01535111400	0.04026697136
Inverse v	109	0.18379466472	0.083312	0.02737822499	0.02050179346
Inverse v	109	0.17456505454	0.081794	0.03282719899	0.01424781744
Inverse v	109	0.19224993002	0.090062	0.03279118888	0.01572765599
Inverse v	109	0.18999964560	0.088868	0.03251877200	0.01581645605
Inverse v	109	0.23138938712	0.084917	0.00643252933	0.06495139061
Inverse v	109	0.21481795569	0.087580	0.01418076222	0.04314938352

Inverse v	109	0.22523867594	0.084402	0.0076163126	0.05980954615
Inverse v	109	0.17106542814	0.085030	0.0442554399	0.00439328362
Inverse v	109	0.23436553501	0.086099	0.0064879181	0.06561140336
Inverse v	109	0.18260302985	0.084484	0.0306653045	0.01701416933
Inverse v	109	0.19789762483	0.087951	0.0244447285	0.02551189480
Inverse v	109	0.29326045493	0.090458	0.0011871973	0.11596204819
Inverse v	109	0.23639709634	0.083877	0.0048268700	0.07199749391
Inverse v	109	0.19849566858	0.087131	0.0227196256	0.02771797719
Inverse v	109	0.19006480994	0.084960	0.0252898133	0.02353111433
Inverse v	109	0.18631199960	0.089209	0.0367546755	0.01146151125
Inverse v	109	0.23040353860	0.082471	0.0052104206	0.06875900582
Inverse v	109	0.21715490364	0.087490	0.0130693639	0.04566203736
Inverse v	109	0.16369405261	0.082018	0.0459547403	0.00293699729
Inverse v	109	0.18681923959	0.083973	0.0260979784	0.02223194041
Inverse v	109	0.19068435068	0.083175	0.0218730439	0.02766115263
Inverse v	109	0.18057412183	0.088367	0.0410083960	0.00737383500
Inverse v	109	0.17856571458	0.085843	0.0375132788	0.01031267657
Inverse v	109	0.22959791624	0.094680	0.0153155942	0.04401291407
Inverse v	109	0.17223534857	0.086167	0.0456262721	0.00334628022
Inverse v	109	0.16633365258	0.081983	0.0424719425	0.00564587821
Inverse v	109	0.18790116522	0.080728	0.0199353656	0.02967282961
Inverse v	109	0.20112467190	0.099034	0.0422704592	0.00701637846
Inverse v	109	0.20339483877	0.084340	0.0158827890	0.03808788973
Inverse v	109	0.17314556393	0.084131	0.0395858534	0.00824859944
Inverse v	109	0.19143907760	0.085333	0.0248698076	0.02418517109
Inverse v	109	0.19939497569	0.082621	0.0158058351	0.03745776188
Inverse v	109	0.20778584958	0.087295	0.0173007671	0.03668626108
Inverse v	109	0.21134915314	0.085715	0.0136742448	0.04334701973
Inverse v	109	0.20265647898	0.090698	0.0254571933	0.02488712027
Inverse v	109	0.19774879724	0.091282	0.0302849295	0.01883552733
Inverse v	109	0.21063637088	0.089164	0.0181599627	0.03587438460
Inverse v	109	0.19013085027	0.086655	0.0282281807	0.02028609811
Inverse v	109	0.17853807473	0.084820	0.0353134378	0.01227827287
Inverse v	109	0.21183232637	0.089613	0.0180863679	0.03618996240
Inverse v	109	0.19929959927	0.088731	0.0246980804	0.02538547686
Inverse v	109	0.18113840081	0.087080	0.0375136380	0.01046093516
Inverse v	109	0.19712770939	0.086400	0.0225151464	0.02778314565
Inverse v	109	0.21889826197	0.084140	0.0092794635	0.05398305051

Inverse v	109	0.1946209024	0.08936	0.0294133674	0.0194720442
Inverse v	109	0.1387788899	0.06779	0.0406469686	0.0059056219
Inverse v	109	0.2329350003	0.09824	0.0177473865	0.0403655756
Inverse v	109	0.2279841836	0.09137	0.0125903593	0.0488972158
Inverse v	109	0.1905254229	0.08921	0.0327047634	0.0156732738
Inverse v	109	0.1738232273	0.08530	0.0415876034	0.0066218128
Inverse v	109	0.2096576930	0.08572	0.0144566615	0.0416374368
Inverse v	109	0.1789644719	0.08979	0.0462499755	0.0029725372
Inverse v	109	0.1699583624	0.08168	0.0374544645	0.0098649747
Inverse v	109	-0.22172065	0.09770	0.0232500970	-0.413221333
Inverse v	109	0.2135754661	0.08389	0.0109084342	0.0491329469
Inverse v	109	0.1798974820	0.08328	0.0307702506	0.0166595357
Inverse v	109	0.1842196766	0.08279	0.0260809663	0.0219410658
Inverse v	109	0.2045328189	0.08312	0.0138744515	0.0416046650
Inverse v	109	0.1904292664	0.08438	0.0240241434	0.0250398641
Inverse v	109	0.2082459150	0.08498	0.0142656983	0.0416833433
Inverse v	109	0.2444310828	0.08153	0.0027194224	0.0846185439
Inverse v	109	0.2887458488	0.09576	0.0025674638	0.1010542215
Inverse v	109	0.1969518034	0.09226	0.0327916532	0.0161118274
Inverse v	109	0.1839437201	0.08838	0.0374295926	0.0106993535
Inverse v	109	-0.170478127	0.08568	0.0466463660	-0.338428678
Inverse v	109	0.2248722961	0.08807	0.0106754990	0.0522421983
Inverse v	109	0.2148546322	0.09430	0.0227154332	0.0300080577
Inverse v	109	0.1817813505	0.08504	0.0325565068	0.0150960986
Inverse v	109	0.1809771634	0.08184	0.0270177110	0.0205641197
Inverse v	109	0.1959152116	0.08332	0.0187144146	0.0325949932
Inverse v	109	0.1967965876	0.09880	0.0464014189	0.0031344962
Inverse v	109	0.2697573017	0.09458	0.0043462467	0.0843618372
Inverse v	109	0.1837278965	0.08778	0.0363630672	0.0116622061
Inverse v	109	0.1746314492	0.08663	0.0438292760	0.0048272791
Inverse v	109	0.1920834398	0.08935	0.0315853405	0.0169438036
Inverse v	109	0.2004148130	0.08557	0.0191846279	0.0326838977
Inverse v	109	0.2286223164	0.09904	0.0209902898	0.0344850305
Inverse v	109	0.1717640658	0.08661	0.0473481388	0.0020070073
Inverse v	109	0.2017527186	0.09092	0.0265016795	0.0235307735
Inverse v	109	0.2357595868	0.09078	0.0094089897	0.0578158198
Inverse v	109	0.1750476266	0.08835	0.0475813270	0.0018633831
Inverse v	109	0.1555894179	0.07746	0.0445874873	0.0037589975

Inverse v	109	0.1970828510	0.08353	0.01831079	0.033353413
Inverse v	109	0.1741615611	0.08198	0.03364192	0.013473043
Inverse v	109	-0.190797409	0.08960	0.03322159	-0.366416741
Inverse v	109	0.1776485040	0.08332	0.03300063	0.014337653
Inverse v	109	0.1792608270	0.08637	0.03794735	0.009969446
Inverse v	109	0.2027730337	0.08242	0.01389411	0.041213322
Inverse v	109	-0.330392094	0.10763	0.00214474	-0.54136633
Inverse v	109	0.2437621388	0.09012	0.00683366	0.067125723
Inverse v	109	0.1866697763	0.08363	0.02562250	0.022739912
Inverse v	109	0.1928876032	0.08686	0.02637473	0.022640218
Inverse v	109	0.2039641186	0.08668	0.01862174	0.034067532
Inverse v	109	0.1663701739	0.08186	0.04213124	0.005912494
Inverse v	109	0.1762034711	0.08852	0.04653907	0.002697399
Inverse v	109	0.1809133269	0.08130	0.02607481	0.021553830
Inverse v	109	0.2251232835	0.08899	0.01141413	0.050702772
Inverse v	109	0.2321162244	0.08428	0.00588675	0.066922335
Inverse v	109	0.2451226444	0.08408	0.00355651	0.080307880
Inverse v	109	0.1895194371	0.08913	0.03347891	0.014821876
Inverse v	109	0.1881713588	0.09006	0.03668685	0.011639557
Inverse v	109	0.1954234770	0.08643	0.02376356	0.026010918
Inverse v	109	0.2032755259	0.08641	0.01865074	0.033910867
Inverse v	109	0.1766026514	0.08662	0.04147723	0.006819681
Inverse v	109	0.1639585262	0.07996	0.04033623	0.007220280
Inverse v	109	0.1988714002	0.08827	0.02426407	0.025856997
Inverse v	109	0.1864099800	0.08920	0.03663719	0.011576818
Inverse v	109	0.1789720715	0.08882	0.04392624	0.004867285
Inverse v	109	0.2064212835	0.09199	0.02484398	0.026110155
Inverse v	109	0.2172708670	0.08478	0.01038964	0.051090602
Inverse v	109	0.2309335957	0.08383	0.00587433	0.066622611
Inverse v	109	-0.175327036	0.08607	0.04165982	-0.344034673
Inverse v	109	0.1994663467	0.08749	0.02262526	0.027972994
Inverse v	109	0.1761876427	0.08509	0.03840213	0.009405853
Inverse v	109	0.1840802079	0.08234	0.02539195	0.022677634
Inverse v	109	0.2006405618	0.08678	0.02078569	0.030536662
Inverse v	109	0.2365979073	0.08471	0.00522363	0.070558886
Inverse v	109	0.1755224344	0.08654	0.04254938	0.005894267
Inverse v	109	0.2251000717	0.08948	0.01188849	0.049704646
Inverse v	109	0.1672380908	0.08318	0.04437431	0.004203762

Inverse v	109	0.1961258551(	0.08738(	0.02480016733	0.0248598900]
Inverse v	109	0.1913265341(	0.08757]	0.0289147223	0.0196741033]
Inverse v	109	0.1698396910(	0.08515	0.0460984089	0.0029370709
Inverse v	109	0.2178136486]	0.08569	0.0110281131	0.0498564454
Inverse v	109	0.2235049571(	0.08355	0.0074763213	0.0597312508(
Inverse v	109	0.2005875636(	0.08900	0.0242217035	0.0261321932
Inverse v	109	0.1894423973(	0.08412	0.0243238995	0.0245616935(
Inverse v	109	0.1838585746(	0.09220	0.0461411571	0.0031440388
Inverse v	109	0.1741040346	0.08582(	0.0425032842	0.0058841305
Inverse v	109	0.2084336519(	0.08386	0.0129464322	0.0440511176
Inverse v	109	0.2072818990	0.08339(	0.0129366470	0.0438254031
Inverse v	109	0.1635627103	0.08154(	0.0448822494	0.0037312773(
Inverse v	109	0.1896087807(	0.08763	0.0304913296	0.0178465026
Inverse v	109	0.2169364565	0.08953	0.0153999204	0.0414414440(
Inverse v	109	0.2372092641	0.08765	0.0068049727	0.0654099934
Inverse v	109	0.1579618657	0.08065	0.0501672415	-0.000118138
Inverse v	109	0.1666135034	0.08471	0.0492206792	0.0005655036]
Inverse v	109	0.1641050532(	0.08366	0.0498253516	0.0001221757(
Inverse v	109	0.1611169334	0.08256	0.0510060315	-0.00070765
Inverse v	109	0.1699354633(	0.08664	0.0498471394	0.0001103336
Inverse v	109	0.1833224498	0.09359	0.0501428869	-0.000117641
Inverse v	109	0.1701497647	0.08660	0.0494440825	0.0004106823(
Inverse v	109	0.1643837019	0.08415(	0.0507828246	-0.000562972
Inverse v	109	0.1955699488	0.09957(	0.0495140527	0.0004120545]
Inverse v	109	0.1619928397	0.08260	0.0498802077	8.1768652030(
Inverse v	109	0.1717494948	0.08783	0.0505504378	-0.000414903
Inverse v	109	0.1682343699	0.08531	0.0486092835	0.0010239784
Inverse v	109	0.1589463647(	0.08127	0.0505119494	-0.000357384
Inverse v	109	0.1713571672	0.08760(	0.0504671187	-0.000351888
Inverse v	109	0.1834779251	0.09336	0.0494040498	0.0004750632
Inverse v	109	0.1705619739	0.08625	0.0480029983	0.0014958798
Inverse v	109	0.1754584291	0.08983	0.0508079758	-0.000620042
Inverse v	109	0.1739482396	0.08909	0.0508896024	-0.000676266
Inverse v	109	0.1692790369	0.08658	0.0505869098	-0.000435763
Inverse v	109	0.1744854527(	0.08825	0.0480431164	0.0014992302
Inverse v	109	0.1620015238	0.08306	0.0511418404	-0.000806758
Inverse v	109	0.2045375025]	0.10481	0.0510006699	-0.000893611
Inverse v	109	0.1636388324	0.08325(	0.0493578120	0.0004568886

Inverse \	109	0.16594911198	0.084935	0.0507215130	-0.000524186
Inverse \	109	0.16463357935	0.083574	0.0488504967	0.00082690654
Inverse \	109	0.17385117512	0.088123	0.0485156434	0.00113006972
Inverse \	109	0.16200095666	0.082513	0.0496088833	0.00027403059
Inverse \	109	0.16927324277	0.085700	0.0482479916	0.00130072834
Inverse \	109	0.17337116175	0.088670	0.0505571443	-0.000423874
Inverse \	109	0.16839698215	0.085399	0.0486243923	0.00101373566
Inverse \	109	0.21450792694	0.109203	0.0494951826	0.00046969528
Inverse \	109	0.17030177346	0.086827	0.0498352365	0.00011943102
Inverse \	109	0.16065688370	0.082360	0.0511141751	-0.000780834
Inverse \	109	0.16507510101	0.083647	0.0484419465	0.00112679452
Inverse \	109	0.17844117003	0.091166	0.0503114439	-0.000245558
Inverse \	109	0.16558498064	0.083921	0.0484847111	0.00109897232
Inverse \	109	0.17111733801	0.087362	0.0501477111	-0.000113408
Inverse \	109	0.16582406288	0.084069	0.0485561238	0.00104823643
Inverse \	109	0.18105772536	0.092113	0.0493446541	0.00051597694
Inverse \	109	0.17038068168	0.087307	0.0509966295	-0.000741401
Inverse \	109	0.17031499734	0.086606	0.0492369119	0.00056592316
Inverse \	109	0.16929689398	0.086607	0.0506107090	-0.000453312
Inverse \	109	0.17264125471	0.088603	0.0513598787	-0.001022439
Inverse \	109	0.16241180553	0.083418	0.0515409690	-0.00108878
Inverse \	109	0.17195199701	0.088380	0.0517052957	-0.001274514
Inverse \	109	0.19267979289	0.099008	0.0516429216	-0.001376374
Inverse \	109	0.16965020244	0.087157	0.0515965321	-0.001177947
Inverse \	109	0.16190585449	0.083280	0.0518836447	-0.001324337
Inverse \	109	0.16008474866	0.082327	0.0518353642	-0.001276191
Inverse \	109	0.16707197219	0.086020	0.0521086126	-0.001528121
Inverse \	109	0.16355451014	0.084308	0.0523840347	-0.001689174
Inverse \	109	0.15731731161	0.081064	0.0523006524	-0.001568531
Inverse \	109	0.16702261422	0.086113	0.0524334007	-0.001760317
Inverse \	109	0.17945014959	0.092439	0.0522242215	-0.001730381
Inverse \	109	0.17689149784	0.091174	0.0523620375	-0.001810242
Inverse \	109	0.16587689401	0.085573	0.0525726604	-0.001847138
Inverse \	109	0.17226722720	0.088914	0.0526914579	-0.002005829
Inverse \	109	0.16109492434	0.083164	0.0527380394	-0.001907818
Inverse \	109	0.17333990156	0.089602	0.0530464440	-0.002281043
Inverse \	109	0.17385287130	0.089910	0.0531606826	-0.002372445
Inverse \	109	0.17458966425	0.090304	0.0531946370	-0.002407753

Inverse v	109	0.16996696478	0.08799	0.0534068777	-0.002497538
Inverse v	109	0.1670651218(	0.08683	0.054362329(	-0.003131425
Inverse v	109	0.16349743053	0.08494	0.0542672943	-0.0029989
Inverse v	109	0.1674284581(	0.08700	0.054304682(	-0.003097459
Inverse v	109	0.1851093546	0.09597	0.0537670627	-0.003003229
Inverse v	109	0.1629682779	0.08468	0.0543148437	-0.003021942
Inverse v	109	0.1751782416	0.09118	0.0547252519	-0.003551691
Inverse v	109	0.1718900840	0.08942	0.0545724605	-0.003374314
Inverse v	109	0.1676402488(	0.08723	0.0546514708	-0.003346736
Inverse v	109	0.1773406522	0.09204	0.0540098089	-0.003059635
Inverse v	109	0.1628627907	0.08449	0.0539095803	-0.002740705
Inverse v	109	0.1676961551	0.08721	0.0545037973	-0.003243409
Inverse v	109	0.1612628662(	0.08380	0.0543209391	-0.002994471
Inverse v	109	0.1760612298	0.09136	0.0539868528	-0.003020446
Inverse v	109	0.1798131828(	0.09350	0.054483435(	-0.003462313
Inverse v	109	0.1663225413	0.08665	0.0549338325	-0.003518199
Inverse v	109	0.2607009073	0.13577	0.0548516678	-0.00542443
Inverse v	109	0.1576758084	0.08217	0.0550213203	-0.003393313
Inverse v	109	0.1651012401	0.08610	0.055188621(	-0.003669182
Inverse v	109	0.1587205076(	0.08276	0.0551438031	-0.003497499
Inverse v	109	0.1652326539(	0.08622	0.0553272985	-0.003768287
Inverse v	109	0.1688827335	0.08815	0.0554004337	-0.003903339
Inverse v	109	0.1560232090	0.08158	0.055813803(	-0.003876202
Inverse v	109	0.1590331400	0.08326	0.0561478358	-0.004172874
Inverse v	109	0.1571313129	0.08224	0.0560685577	-0.004070983
Inverse v	109	-0.166348742	0.08713	0.0562439568	-0.337129006
Inverse v	109	0.1671591246	0.08757	0.0562858921	-0.004482335
Inverse v	109	0.1653932008	0.08665	0.0563188941	-0.004457733
Inverse v	109	0.1612204877	0.08446	0.0562915581	-0.004326899
Inverse v	109	0.1628724014	0.08537	0.0564135842	-0.004454045
Inverse v	109	0.1636077583(	0.08591	0.0568670422	-0.004782687
Inverse v	109	0.1584387299	0.08315	0.0567281968	-0.004540192
Inverse v	109	0.1617908713	0.08489	0.0566753075	-0.004600679
Inverse v	109	0.1607224917	0.08444	0.0570020079	-0.00478838
Inverse v	109	0.1557481456	0.08180	0.0569148427	-0.00458384
Inverse v	109	0.1562767686(	0.08211	0.0570344994	-0.004676993
Inverse v	109	0.1556431102	0.08220	0.0583140557	-0.005480712
Inverse v	109	0.1655152385	0.08763	0.058937828(	-0.006252266

Inverse v	109	0.17355329403	0.09176	0.05857930	-0.006300623
Inverse v	109	0.1652548922	0.08702	0.05757478	-0.005315389
Inverse v	109	0.1689940684	0.08892	0.05736601	-0.005289725
Inverse v	109	0.1768172674	0.09320	0.05783047	-0.005874014
Inverse v	109	0.1674789106	0.08846	0.05832688	-0.005906329
Inverse v	109	0.1695274199	0.08960	0.05851181	-0.006107465
Inverse v	109	0.1591110267	0.08432	0.05917074	-0.00616211
Inverse v	109	0.1766400934	0.09358	0.05910368	-0.006792499
Inverse v	109	0.1682897386	0.08851	0.05725841	-0.005192704
Inverse v	109	0.1587085456	0.08385	0.05841499	-0.00565454
Inverse v	109	0.1632600639	0.08637	0.05873477	-0.006031142
Inverse v	109	0.1833795613	0.09676	0.05807505	-0.006276961
Inverse v	109	0.1656401645	0.08774	0.05905127	-0.006333967
Inverse v	109	0.1688645482	0.08887	0.05742940	-0.005329968
Inverse v	109	0.1750665146	0.09232	0.05793273	-0.005889701
Inverse v	109	0.1738202793	0.09199	0.05884261	-0.006498144
Inverse v	109	0.1689019932	0.08926	0.05847725	-0.006060943
Inverse v	109	0.1547173210	0.08182	0.05864061	-0.00565576
Inverse v	109	0.1584685314	0.08395	0.05909792	-0.006089997
Inverse v	109	0.1707216650	0.08995	0.05772070	-0.005594151
Inverse v	109	0.1684740591	0.08901	0.05841549	-0.006002816
Inverse v	109	0.1684332046	0.08883	0.05796936	-0.005691985
Inverse v	109	0.1537393352	0.08164	0.05971222	-0.006294174
Inverse v	109	0.1636045360	0.08683	0.05956724	-0.006601275
Inverse v	109	0.1640135996	0.08714	0.05982461	-0.00678997
Inverse v	109	0.1587743743	0.08433	0.05975926	-0.006530771
Inverse v	109	0.1587448600	0.08421	0.05942880	-0.006315437
Inverse v	109	0.1548723894	0.08225	0.05973809	-0.006356899
Inverse v	109	0.1622779648	0.08612	0.05953783	-0.006528264
Inverse v	109	0.1572527385	0.08381	0.06061406	-0.007014877
Inverse v	109	0.1539762155	0.08212	0.06081665	-0.006995187
Inverse v	109	0.1665954074	0.08894	0.06105464	-0.00772902
Inverse v	109	0.1668584572	0.08910	0.06113155	-0.007793137
Inverse v	109	0.1527402017	0.08216	0.06302548	-0.008297409
Inverse v	109	0.1672150782	0.09021	0.06381817	-0.00961295
Inverse v	109	0.1614182963	0.08722	0.06421819	-0.009536671
Inverse v	109	0.1526894787	0.08232	0.06363035	-0.008663584
Inverse v	109	0.1530074659	0.08213	0.06248722	-0.007981897

Inverse v	109	0.16765376652	0.090174	0.0629960679	-0.009087832
Inverse v	109	0.15906460938	0.085117	0.0616569655	-0.007766625
Inverse v	109	0.15931693517	0.085539	0.0625201132	-0.00833207
Inverse v	109	0.16174476922	0.086910	0.0627373747	-0.008599961
Inverse v	109	0.16017173060	0.085719	0.0616711625	-0.007829851
Inverse v	109	0.16055281460	0.085900	0.0616338711	-0.007824337
Inverse v	109	0.16655916007	0.089860	0.0638050650	-0.009566543
Inverse v	109	0.17882256863	0.096382	0.0635460720	-0.010086253
Inverse v	109	0.16320786917	0.088052	0.0638055321	-0.009374361
Inverse v	109	0.15337797100	0.082894	0.0642733714	-0.009095283
Inverse v	109	0.15775360323	0.085160	0.0639846455	-0.009173601
Inverse v	109	0.15842227478	0.084912	0.0620823070	-0.008006636
Inverse v	109	0.16264680649	0.087320	0.0625131950	-0.008501703
Inverse v	109	0.15417282050	0.082981	0.0631791055	-0.008469959
Inverse v	109	0.16622675490	0.089839	0.0642634351	-0.009850649
Inverse v	109	0.16827169126	0.090620	0.0633441276	-0.009355489
Inverse v	109	0.16334373271	0.088273	0.0642509516	-0.009671696
Inverse v	109	0.17498894200	0.094410	0.0638278935	-0.010066634
Inverse v	109	0.16336637860	0.088224	0.0640677776	-0.009554037
Inverse v	109	0.17284166050	0.092738	0.0623556730	-0.008925299
Inverse v	109	0.15051355699	0.080840	0.0626429988	-0.007945855
Inverse v	109	0.17194765331	0.092723	0.0636814728	-0.009791339
Inverse v	109	0.15343841543	0.082899	0.0641842146	-0.009044487
Inverse v	109	0.16610884048	0.089204	0.0625884341	-0.008732806
Inverse v	109	0.16016825281	0.086269	0.0633661041	-0.008919017
Inverse v	109	0.16450337616	0.088618	0.0634079718	-0.009187923
Inverse v	109	0.17747174151	0.095360	0.0627349391	-0.009434431
Inverse v	109	0.15830000689	0.085218	0.0632293776	-0.00872851
Inverse v	109	0.15995829674	0.086040	0.0630118551	-0.008680798
Inverse v	109	0.15559461129	0.083940	0.0637926674	-0.008929094
Inverse v	109	0.16080674706	0.087063	0.0647466746	-0.009837907
Inverse v	109	0.15271737614	0.082695	0.0647851304	-0.009366291
Inverse v	109	0.15749286669	0.085300	0.0648627765	-0.009707634
Inverse v	109	0.16850935624	0.091222	0.0647132512	-0.010286811
Inverse v	109	0.18396091747	0.099699	0.0650150381	-0.011450014
Inverse v	109	0.16305140145	0.088402	0.0651207480	-0.010216786
Inverse v	109	0.16990405440	0.092242	0.0654867723	-0.010891989
Inverse v	109	0.16141464871	0.087609	0.0654104840	-0.010299123

Inverse v	109	0.16498789216	0.089647	0.06570835111	-0.010721113
Inverse v	109	0.15494009746	0.084373	0.0663054789	-0.010432576
Inverse v	109	0.18014116973	0.098154	0.0664633208	-0.01224121
Inverse v	109	0.16794031369	0.091508	0.0664688898	-0.011415795
Inverse v	109	0.15162255019	0.082687	0.0667015914	-0.010445143
Inverse v	109	0.15658356089	0.085367	0.0666185704	-0.010735873
Inverse v	109	0.15701772676	0.085750	0.0670848116	-0.011052742
Inverse v	109	0.14780162119	0.080717	0.0670869342	-0.010405234
Inverse v	109	0.15398896556	0.084162	0.0673001434	-0.010969362
Inverse v	109	0.15776384335	0.086248	0.0673725812	-0.011282974
Inverse v	109	0.15364413030	0.084026	0.0674716063	-0.01104784
Inverse v	109	0.15950435701	0.087331	0.0677867016	-0.011665569
Inverse v	109	0.15224106763	0.083308	0.0676329682	-0.01104296
Inverse v	109	0.15580004404	0.085284	0.0677273269	-0.011358532
Inverse v	109	0.14838581082	0.081276	0.0678964805	-0.010915972
Inverse v	109	0.15859706641	0.086961	0.0681868672	-0.011846722
Inverse v	109	0.15311017561	0.083929	0.0681099373	-0.011390968
Inverse v	109	0.16452145917	0.090167	0.0680591201	-0.012207345
Inverse v	109	0.16169344872	0.088706	0.0683336296	-0.012170438
Inverse v	109	0.17544545366	0.096320	0.0685342622	-0.013342515
Inverse v	109	0.15311607501	0.084093	0.0686374444	-0.011705818
Inverse v	109	0.15839943004	0.087013	0.0686965292	-0.012146114
Inverse v	109	0.15543316511	0.085465	0.0689633482	-0.012079749
Inverse v	109	0.15664685454	0.086185	0.0691326546	-0.012276983
Inverse v	109	0.17039683608	0.093817	0.0693319415	-0.01348627
Inverse v	109	0.15602627064	0.085958	0.0695037418	-0.012452725
Inverse v	109	0.14839789837	0.081895	0.0699786982	-0.012116501
Inverse v	109	0.14911443356	0.082294	0.0699915441	-0.012182405
Inverse v	109	0.16984727431	0.093783	0.0701281719	-0.013965868
Inverse v	109	0.15361477626	0.084883	0.0703408672	-0.01275722
Inverse v	109	0.15163216839	0.084013	0.0710947962	-0.01303277
Inverse v	109	0.15138823899	0.083873	0.0710776882	-0.013001848
Inverse v	109	0.15138170957	0.083810	0.0708802356	-0.012886325
Inverse v	109	0.17205755764	0.095343	0.0711330308	-0.014813616
Inverse v	109	0.16527217068	0.091574	0.0711087491	-0.014213991
Inverse v	109	0.19287030802	0.106763	0.0708353966	-0.016384751
Inverse v	109	0.15311442721	0.084713	0.0706917829	-0.012922748
Inverse v	109	0.15291107792	0.084648	0.0708511024	-0.01299937

Inverse v	109	0.1496730452	0.08360	0.073405766	-0.014187336
Inverse v	109	0.1489127483	0.08305	0.072995712	-0.01388267
Inverse v	109	0.1586380506	0.08850	0.073069671	-0.014834053
Inverse v	109	0.1537421096	0.08562	0.072569881	-0.014083073
Inverse v	109	0.3583607447	0.19922	0.072052464	-0.032117542
Inverse v	109	0.1450300905	0.08065	0.072146234	-0.013050151
Inverse v	109	0.1488305994	0.08278	0.072216617	-0.013432206
Inverse v	109	0.1721117458	0.09566	0.072004148	-0.015393421
Inverse v	109	0.1539796570	0.08578	0.072658447	-0.01415691
Inverse v	109	0.1677144039	0.09323	0.072035085	-0.015019993
Inverse v	109	0.1496536850	0.08364	0.073576868	-0.01428292
Inverse v	109	0.1611235280	0.08974	0.072611262	-0.014784689
Inverse v	109	0.1491487425	0.08316	0.072898325	-0.013849282
Inverse v	109	0.1553765104	0.08640	0.072151401	-0.013984219
Inverse v	109	0.1515978227	0.08404	0.071254885	-0.013123078
Inverse v	109	0.1537002094	0.08588	0.073526099	-0.01463944
Inverse v	109	0.1484806532	0.08297	0.073523951	-0.014141081
Inverse v	109	0.1481130943	0.08228	0.071869007	-0.013170358
Inverse v	109	0.1501816655	0.08380	0.073135924	-0.014081224
Inverse v	109	0.1500311540	0.08369	0.073035075	-0.014009449
Inverse v	109	0.1638672256	0.09149	0.073292867	-0.015462358
Inverse v	109	0.1564346729	0.08737	0.073392906	-0.014820606
Inverse v	109	0.1561923696	0.08670	0.071631521	-0.013746595
Inverse v	109	0.1538108605	0.08545	0.071867639	-0.013676202
Inverse v	109	0.1530062980	0.08517	0.072419460	-0.013927738
Inverse v	109	0.1668998211	0.09288	0.072372576	-0.015162518
Inverse v	109	0.1564326104	0.08701	0.072203876	-0.014110676
Inverse v	109	0.1608076180	0.08998	0.073930039	-0.015563284
Inverse v	109	0.1585524546	0.08868	0.073800293	-0.015266881
Inverse v	109	0.1540701661	0.08620	0.073899783	-0.014893516
Inverse v	109	0.1480416804	0.08284	0.073956004	-0.014342368
Inverse v	109	0.1501433061	0.08411	0.074252264	-0.014714777
Inverse v	109	0.1563494696	0.08754	0.074095536	-0.015230047
Inverse v	109	0.1550636298	0.08684	0.074187419	-0.015158853
Inverse v	109	0.1474748907	0.08271	0.074596479	-0.014645648
Inverse v	109	0.1474397352	0.08268	0.074554712	-0.014618832
Inverse v	109	0.1584302170	0.08880	0.074434088	-0.015636145
Inverse v	109	0.1500227461	0.08416	0.074656063	-0.014932524

Inverse v	109	0.16684439368	0.09367(	0.07488205690	-0.01674957
Inverse v	109	0.15587437252	0.08748!	0.074807909!	-0.015604557
Inverse v	109	0.15087025832	0.08477(	0.0751379913	-0.015291913
Inverse v	109	0.1605264871!	0.09031(	0.0755069971	-0.016494335
Inverse v	109	0.1458891167!	0.08201!	0.0752743843	-0.01486221
Inverse v	109	0.1564387440	0.08791	0.0751763726	-0.015879013
Inverse v	109	0.1534805198!	0.08635(	0.0755210242	-0.015778475
Inverse v	109	0.1569465949!	0.08826	0.0753731342	-0.016047197
Inverse v	109	-0.144219244	0.08106	0.0752301067	-0.303106462
Inverse v	109	0.1473877908!	0.08301!	0.0758416796	-0.015330329
Inverse v	109	0.1520027771	0.08560!	0.0758067623	-0.015790347
Inverse v	109	0.1667845971(	0.09390(	0.0757205530	-0.017271711
Inverse v	109	0.1584089545	0.08944(	0.0765636520	-0.01690706
Inverse v	109	0.1527191094!	0.08619(	0.0764136040	-0.016213642
Inverse v	109	0.1594409661	0.09008!	0.0767447260	-0.017125661
Inverse v	109	0.1558258511	0.08803	0.0767125971	-0.016718558
Inverse v	109	0.1459351796	0.08234!	0.076366745!	-0.015467702
Inverse v	109	0.1536654493	0.08679(	0.076659942!	-0.016456377
Inverse v	109	0.1581939038	0.08926!	0.0763747927	-0.016771794
Inverse v	109	0.1640313864!	0.09261	0.0765418907	-0.01749373
Inverse v	109	0.1486825211	0.08404!	0.0768949032	-0.016053916
Inverse v	109	0.1514118597	0.08560(	0.0769451139	-0.016377145
Inverse v	109	0.1534230280	0.08678!	0.0770889512	-0.016677463
Inverse v	109	0.1529502669(	0.08652!	0.0771239941	-0.016646173
Inverse v	109	0.1511343719	0.08563	0.0775945961	-0.016715008
Inverse v	109	0.1607864069	0.09112	0.0776472807	-0.017814199
Inverse v	109	0.1551623003	0.08795	0.0777036461	-0.017223807
Inverse v	109	0.1563011192	0.08848	0.077318875!	-0.017125038
Inverse v	109	0.1647188093	0.09333	0.0775832926	-0.018210436
Inverse v	109	0.1577507526	0.08942	0.0777128650	-0.01751658
Inverse v	109	0.1637486782(	0.09289	0.0779432903	-0.0183237
Inverse v	109	0.1592608489	0.09049	0.078418889!	-0.018104411
Inverse v	109	0.1457862201	0.08276!	0.0781787078	-0.016441921
Inverse v	109	0.1585602527	0.09005	0.0782969103	-0.017952577
Inverse v	109	0.1433319018	0.08141	0.078331636!	-0.016246968
Inverse v	109	0.1444521877	0.08199!	0.0781323012	-0.016266428
Inverse v	109	0.1570040401(	0.08916	0.0782547037	-0.017751637
Inverse v	109	0.1500434806(	0.08555	0.0794644168	-0.017640898

Inverse v	109	0.14753926927	0.084068	0.0792596102	-0.017234098
Inverse v	109	0.13829361907	0.078870	0.0795297173	-0.016293013
Inverse v	109	0.15598329615	0.088982	0.0796083148	-0.018422676
Inverse v	109	0.14540896413	0.082861	0.0792862724	-0.016999679
Inverse v	109	0.16885458480	0.096315	0.0795785738	-0.019924201
Inverse v	109	0.16172713025	0.092094	0.0790705195	-0.018777575
Inverse v	109	0.16161430184	0.092101	0.0793026701	-0.018904094
Inverse v	109	0.15178710324	0.086432	0.0790667651	-0.017621351
Inverse v	109	0.15411521820	0.088024	0.0799763394	-0.018412652
Inverse v	109	0.15448592816	0.088263	0.0800689635	-0.018510032
Inverse v	109	0.14445227839	0.082639	0.0804670769	-0.017521016
Inverse v	109	0.14630979203	0.083834	0.0809451023	-0.018005192
Inverse v	109	0.15049979526	0.086239	0.0809621622	-0.018530318
Inverse v	109	0.15636437671	0.089550	0.0807925046	-0.019154262
Inverse v	109	0.14633229925	0.083795	0.0807569474	-0.017906102
Inverse v	109	0.14765943115	0.084598	0.0809117067	-0.018153043
Inverse v	109	0.14949802275	0.085721	0.0811600542	-0.018516343
Inverse v	109	0.14815314326	0.084953	0.0811698987	-0.01835516
Inverse v	109	0.14651481454	0.084050	0.0813261170	-0.018236743
Inverse v	109	0.14615620690	0.083893	0.0814796403	-0.018274961
Inverse v	109	0.16121200929	0.092568	0.0815893364	-0.020222767
Inverse v	109	0.15444836082	0.088711	0.0816805297	-0.019426288
Inverse v	109	0.14375458928	0.082667	0.0820441916	-0.018273979
Inverse v	109	0.14796692310	0.085364	0.0830324589	-0.019347358
Inverse v	109	0.14932497182	0.086091	0.0828316163	-0.019414751
Inverse v	109	0.14397594097	0.082911	0.0824754661	-0.018530737
Inverse v	109	0.14873625793	0.085765	0.0828800765	-0.019364694
Inverse v	109	0.15100990941	0.087130	0.0830692015	-0.019765618
Inverse v	109	0.15143847027	0.087355	0.0829927212	-0.019779177
Inverse v	109	0.15695762613	0.090558	0.0830565472	-0.020536818
Inverse v	109	0.15538591225	0.089660	0.0831088012	-0.020360985
Inverse v	109	0.14222091205	0.082015	0.0829056099	-0.018529772
Inverse v	109	0.15759259608	0.090750	0.0824661108	-0.020277874
Inverse v	109	0.14322930609	0.082595	0.0828983820	-0.018657351
Inverse v	109	0.14428804778	0.083419	0.0836900385	-0.019214408
Inverse v	109	0.14620619090	0.084489	0.0835460769	-0.019392689
Inverse v	109	0.15350526694	0.088670	0.0834175033	-0.020288455
Inverse v	109	0.15423736914	0.089097	0.0834337621	-0.020394413

Inverse v	109	0.151604187	0.08761	0.083579501	-0.020127253
Inverse v	109	0.163550066	0.09455	0.083677752	-0.02177211
Inverse v	109	0.157298150	0.09126	0.084800143	-0.021585721
Inverse v	109	0.143302996	0.08313	0.084746487	-0.019637111
Inverse v	109	0.155840543	0.09013	0.083828501	-0.02083188
Inverse v	109	0.142432366	0.08266	0.084889017	-0.01959194
Inverse v	109	0.151762239	0.08783	0.084015705	-0.020390755
Inverse v	109	0.145322390	0.08434	0.084908902	-0.02000002
Inverse v	109	0.144147640	0.08366	0.084921731	-0.019845095
Inverse v	109	0.164949154	0.09573	0.084884079	-0.022686208
Inverse v	109	0.143184214	0.08295	0.084334876	-0.019405432
Inverse v	109	0.147622308	0.08552	0.084320516	-0.019999164
Inverse v	109	0.155413601	0.09006	0.084425092	-0.021114123
Inverse v	109	0.141571195	0.08208	0.084600668	-0.019324381
Inverse v	109	0.153267899	0.08881	0.084403248	-0.020810372
Inverse v	109	0.144520634	0.08391	0.085043446	-0.019960648
Inverse v	109	0.150777652	0.08766	0.085444407	-0.021045318
Inverse v	109	0.145037192	0.08434	0.085510578	-0.020279048
Inverse v	109	0.164214669	0.09550	0.085540522	-0.022978353
Inverse v	109	0.147403703	0.08577	0.085713133	-0.020718696
Inverse v	109	0.153862366	0.08957	0.085843590	-0.021699591
Inverse v	109	0.154906864	0.09024	0.086059941	-0.021968857
Inverse v	109	0.148877951	0.08724	0.087928070	-0.022122796
Inverse v	109	0.142341513	0.08304	0.086521381	-0.020425614
Inverse v	109	0.159106580	0.09322	0.087863327	-0.023605467
Inverse v	109	0.138490602	0.08091	0.086964757	-0.020095937
Inverse v	109	0.161041038	0.09432	0.087752191	-0.023827695
Inverse v	109	0.147972188	0.08649	0.087141729	-0.021566762
Inverse v	109	0.158553386	0.09282	0.087625888	-0.023387121
Inverse v	109	0.148379599	0.08649	0.086266157	-0.021154437
Inverse v	109	0.150126013	0.08764	0.086724554	-0.021653441
Inverse v	109	0.147092353	0.08607	0.087459975	-0.021608192
Inverse v	109	0.149186671	0.08726	0.087344367	-0.021853359
Inverse v	109	0.143054148	0.08355	0.086872060	-0.020710023
Inverse v	109	0.159284380	0.09306	0.086974516	-0.023118897
Inverse v	109	0.143569159	0.08407	0.087719917	-0.021225776
Inverse v	109	0.141810592	0.08307	0.087822014	-0.021018188
Inverse v	109	-0.144672214	0.08479	0.087964424	-0.310861292

Inverse v	109	0.1441900138	0.08413	0.086581017	-0.020722104
Inverse v	109	0.1460688408	0.08539	0.087157490	-0.021297699
Inverse v	109	0.1504434711	0.08792	0.087088443	-0.021897877
Inverse v	109	0.1643087576	0.09585	0.086496853	-0.023563212
Inverse v	109	0.1502192634	0.08832	0.088999983	-0.022903777
Inverse v	109	0.1422512017	0.08370	0.089247623	-0.02181591
Inverse v	109	0.1460811263	0.08597	0.089290844	-0.022426031
Inverse v	109	0.1418014844	0.08342	0.089191100	-0.021718049
Inverse v	109	0.1401560284	0.08251	0.089407878	-0.02157553
Inverse v	109	0.1432712854	0.08438	0.089550441	-0.022128664
Inverse v	109	0.1436217532	0.08484	0.090514735	-0.022680908
Inverse v	109	0.1476663147	0.08722	0.090455197	-0.023288047
Inverse v	109	0.1388839730	0.08207	0.090604594	-0.021977534
Inverse v	109	0.1541336651	0.09115	0.090864947	-0.024534761
Inverse v	109	0.1399309685	0.08279	0.091024748	-0.022354226
Inverse v	109	0.1475535667	0.08734	0.091158884	-0.023642933
Inverse v	109	0.1364542927	0.08075	0.091064536	-0.021818295
Inverse v	109	0.1426424393	0.08450	0.091421784	-0.022990433
Inverse v	109	0.1534867010	0.09090	0.091331992	-0.024688869
Inverse v	109	0.1512610310	0.08965	0.091570901	-0.024460345
Inverse v	109	0.1435756384	0.08517	0.091858206	-0.023365243
Inverse v	109	0.1469101581	0.08722	0.092143066	-0.02405763
Inverse v	109	0.1441029128	0.08558	0.092222574	-0.023638897
Inverse v	109	0.1548662139	0.09198	0.092242405	-0.02541551
Inverse v	109	0.1493557389	0.08876	0.092453322	-0.024623781
Inverse v	109	0.1404731102	0.08354	0.092664648	-0.023265393
Inverse v	109	0.1442484275	0.08577	0.092611231	-0.023863144
Inverse v	109	0.1505516576	0.08968	0.093231575	-0.025239266
Inverse v	109	0.1425304338	0.08487	0.093104027	-0.023829694
Inverse v	109	0.1571147812	0.09357	0.093152002	-0.026294946
Inverse v	109	0.1403351381	0.08358	0.093178705	-0.02350005
Inverse v	109	0.1387348042	0.08277	0.093721382	-0.023500453
Inverse v	109	0.1547121407	0.09229	0.093666296	-0.026176511
Inverse v	109	0.1461780885	0.08724	0.093819862	-0.024812558
Inverse v	109	0.1481758779	0.08847	0.093983795	-0.025238163
Inverse v	109	0.1478426093	0.08847	0.094717195	-0.025567073
Inverse v	109	0.1542201480	0.09227	0.094650878	-0.026633619
Inverse v	109	0.1377357236	0.08243	0.094743022	-0.023831886

Inverse v	109	0.14700673152	0.088020	0.0948927418	-0.025514213
Inverse v	109	0.14608858486	0.087625	0.0954745115	-0.025656564
Inverse v	109	0.14489918749	0.086900	0.0954319507	-0.0254258
Inverse v	109	0.14364615572	0.086139	0.0953957600	-0.025187483
Inverse v	109	-0.140279632	0.084080	0.0952374815	-0.305077654
Inverse v	109	0.14645018421	0.087820	0.0954172479	-0.025690318
Inverse v	109	0.14413796028	0.086543	0.0958126554	-0.02548682
Inverse v	109	0.14802746969	0.088880	0.0958408305	-0.026189356
Inverse v	109	0.15924290298	0.095794	0.0964451415	-0.028514461
Inverse v	109	0.15037810157	0.090445	0.0963841026	-0.026894616
Inverse v	109	0.14986751909	0.090100	0.0962683398	-0.026741877
Inverse v	109	0.14724212833	0.088532	0.0962840835	-0.026281619
Inverse v	109	0.14514816434	0.087537	0.0972908710	-0.02642469
Inverse v	109	0.13958161602	0.084075	0.0968735891	-0.025205426
Inverse v	109	0.13834936106	0.083524	0.0976432431	-0.025359097
Inverse v	109	0.14992890054	0.090439	0.0973608852	-0.027332119
Inverse v	109	0.14036965927	0.084621	0.0971576116	-0.025488657
Inverse v	109	0.14702207408	0.088703	0.0974279726	-0.026837038
Inverse v	109	0.15069377699	0.090961	0.0975844135	-0.027590503
Inverse v	109	0.14520897071	0.087530	0.0971250399	-0.026350677
Inverse v	109	0.14831764656	0.089540	0.0976572069	-0.02719357
Inverse v	109	0.14617675150	0.088190	0.0974406790	-0.026689295
Inverse v	109	0.15730165048	0.095510	0.0995880928	-0.029910614
Inverse v	109	0.13888789779	0.084142	0.0988127899	-0.026030456
Inverse v	109	0.14829050271	0.089989	0.0993790526	-0.028088173
Inverse v	109	0.14497557473	0.087609	0.0979653220	-0.026738431
Inverse v	109	0.13712768114	0.083174	0.0992127196	-0.025893563
Inverse v	109	0.14124494005	0.085894	0.1000924557	-0.027107739
Inverse v	109	0.15067279706	0.091461	0.0994790481	-0.02859239
Inverse v	109	0.14605886491	0.088693	0.0996013934	-0.027779648
Inverse v	109	0.14496209948	0.087857	0.0989484334	-0.027238109
Inverse v	109	0.14618386793	0.088414	0.0982525387	-0.027109337
Inverse v	109	0.15035952414	0.091560	0.1005715987	-0.029109859
Inverse v	109	0.14035410957	0.084908	0.0983290708	-0.026066089
Inverse v	109	0.13730831684	0.083070	0.0983502867	-0.025510702
Inverse v	109	0.14003720314	0.085091	0.0998206652	-0.026742266
Inverse v	109	0.13342514691	0.081093	0.0999009337	-0.025517216
Inverse v	109	0.14796552629	0.090110	0.1005814809	-0.028651506

Inverse v	109	0.13601019941	0.08264	0.09981218	-0.025969194
Inverse v	109	0.14621955553	0.08846	0.09836228	-0.027172518
Inverse v	109	0.15466833883	0.09385	0.09936158	-0.029286717
Inverse v	109	0.13705919385	0.08329	0.09985376	-0.026189508
Inverse v	109	0.13377095434	0.08137	0.10020653	-0.02572691
Inverse v	109	0.14111542006	0.08567	0.09953060	-0.026804312
Inverse v	109	0.13512548335	0.08207	0.09967907	-0.025737069
Inverse v	109	0.14250320935	0.08677	0.10053854	-0.027572341
Inverse v	109	0.13874330625	0.08413	0.09912371	-0.026155191
Inverse v	109	0.14527592443	0.08791	0.09843535	-0.027034565
Inverse v	109	0.13675676143	0.08327	0.10053162	-0.026457164
Inverse v	109	0.14265033550	0.08689	0.10065819	-0.027660676
Inverse v	109	0.13230296936	0.08044	0.10004507	-0.025369585
Inverse v	109	0.13483777669	0.08225	0.10116940	-0.026387414
Inverse v	109	0.13675415661	0.08340	0.10106154	-0.02671076
Inverse v	109	0.14816512632	0.09045	0.10142072	-0.029125973
Inverse v	109	0.15296681725	0.09339	0.10144286	-0.030081743
Inverse v	109	0.13414615184	0.08185	0.10124351	-0.026286895
Inverse v	109	0.13382523098	0.08159	0.10099109	-0.026105639
Inverse v	109	0.13681323563	0.08351	0.10138946	-0.02687947
Inverse v	109	0.14078105670	0.08600	0.10164204	-0.027783517
Inverse v	109	0.13523843973	0.08259	0.10155908	-0.026650391
Inverse v	109	0.15684173280	0.09604	0.10247643	-0.031410842
Inverse v	109	0.14301836090	0.08754	0.10234184	-0.028575144
Inverse v	109	0.13963611977	0.08552	0.10252817	-0.027990302
Inverse v	109	0.14716157602	0.09013	0.10253572	-0.02950268
Inverse v	109	0.13469999514	0.08244	0.10228902	-0.026888258
Inverse v	109	0.15076650292	0.09232	0.10245426	-0.030182468
Inverse v	109	0.13227839159	0.08091	0.10209490	-0.026315086
Inverse v	109	0.15129172034	0.09266	0.10255330	-0.030339972
Inverse v	109	0.14385475431	0.08824	0.10306777	-0.029107035
Inverse v	109	0.13794471133	0.08455	0.10278835	-0.027776639
Inverse v	109	0.14002724530	0.08586	0.10294218	-0.0282712
Inverse v	109	0.14051719095	0.08617	0.10298148	-0.028389396
Inverse v	109	0.14307649472	0.08775	0.10300557	-0.028918498
Inverse v	109	0.14655949685	0.09005	0.10365640	-0.029955274
Inverse v	109	0.14854329803	0.09135	0.10393957	-0.030507365
Inverse v	109	0.14406053590	0.08856	0.10382820	-0.029530793

Inverse v	109	0.13760466758	0.084633	0.10397490159	-0.028277764
Inverse v	109	0.14257013348	0.087631	0.10375183659	-0.029187322
Inverse v	109	0.13807282231	0.084883	0.10381659199	-0.028297789
Inverse v	109	0.15278322380	0.093900	0.10374177077	-0.031272811
Inverse v	109	0.14577387557	0.089701	0.10414277411	-0.030041798
Inverse v	109	0.13880182904	0.085425	0.10419835159	-0.028631837
Inverse v	109	0.14702930879	0.090520	0.10431738988	-0.030389947
Inverse v	109	0.13237918038	0.081530	0.10447218159	-0.027433216
Inverse v	109	0.13491176841	0.083164	0.10475440444	-0.028090574
Inverse v	109	0.14612542365	0.090179	0.10515020377	-0.0306266
Inverse v	109	0.13525281380	0.083473	0.10516539244	-0.02835494
Inverse v	109	0.14052612222	0.086697	0.10504524459	-0.029401744
Inverse v	109	0.13602820697	0.083988	0.10531807244	-0.028589702
Inverse v	109	0.13881053985	0.085733	0.10542141277	-0.029224338
Inverse v	109	0.15014877960	0.092911	0.10608457199	-0.031957287
Inverse v	109	0.14626091521	0.090493	0.10603940777	-0.031106871
Inverse v	109	0.13909901722	0.086045	0.10596899060	-0.029549666
Inverse v	109	0.14904146549	0.092238	0.10613037988	-0.031745308
Inverse v	109	0.15379906780	0.095280	0.10649028377	-0.03295074
Inverse v	109	0.15582184870	0.096563	0.10659542733	-0.033440941
Inverse v	109	0.14688787303	0.091000	0.10651875399	-0.031484555
Inverse v	109	0.14193433634	0.087890	0.10633046200	-0.030330074
Inverse v	109	0.13530548356	0.083984	0.10716218411	-0.029303766
Inverse v	109	0.14627487183	0.090791	0.10715709799	-0.031676882
Inverse v	109	0.13915927258	0.086334	0.10699287599	-0.03005675
Inverse v	109	0.13321863645	0.082690	0.10719193159	-0.028865539
Inverse v	109	0.13392531745	0.083104	0.10706490022	-0.028959708
Inverse v	109	0.13250656517	0.082380	0.10776004611	-0.028971953
Inverse v	109	0.13653091447	0.084981	0.10814201033	-0.03003232
Inverse v	109	-0.139143873	0.086529	0.10782297533	-0.308741224
Inverse v	109	0.16544420699	0.102947	0.10803682011	-0.036332087
Inverse v	109	0.13129130975	0.081653	0.10785264622	-0.028748324
Inverse v	109	0.13595333974	0.084564	0.10790210699	-0.029792421
Inverse v	109	0.13982125158	0.087003	0.10803806622	-0.030705805
Inverse v	109	0.13999282795	0.086953	0.10740061159	-0.030434559
Inverse v	109	0.13984063845	0.087045	0.10815761600	-0.0307679
Inverse v	109	0.13584507862	0.084600	0.10835820088	-0.029983036
Inverse v	109	0.13344399997	0.083091	0.10827577760	-0.029415044

Inverse v	109	0.13976884839	0.087089	0.1085174058	-0.03092601
Inverse v	109	0.13265819953	0.082749	0.1089048892	-0.029530324
Inverse v	109	0.14666830576	0.091540	0.1091298072	-0.032762993
Inverse v	109	0.13612852315	0.084995	0.1092445995	-0.030462566
Inverse v	109	0.13824486840	0.086731	0.1109504664	-0.031749419
Inverse v	109	0.14643271012	0.091884	0.1110105955	-0.033660168
Inverse v	109	0.13776124750	0.086443	0.1110121293	-0.031667605
Inverse v	109	0.13205277345	0.082551	0.1096761444	-0.02974722
Inverse v	109	0.14427844494	0.090317	0.1101628648	-0.032743502
Inverse v	109	0.13448129710	0.084249	0.1104224125	-0.030640405
Inverse v	109	0.13446706300	0.084230	0.1103945379	-0.030624242
Inverse v	109	0.14176245111	0.088863	0.1106478654	-0.032409496
Inverse v	109	0.14393660832	0.090005	0.1097780096	-0.032474856
Inverse v	109	0.14860789536	0.093098	0.1104343222	-0.033865129
Inverse v	109	0.14530125172	0.091100	0.1107239844	-0.033256638
Inverse v	109	0.14574203613	0.091117	0.1097092678	-0.032847626
Inverse v	109	0.13533723199	0.084858	0.1107431304	-0.030984994
Inverse v	109	0.13162537687	0.082478	0.1105166749	-0.030032471
Inverse v	109	0.13395410065	0.083933	0.1104969051	-0.03055468
Inverse v	109	0.14137893968	0.088721	0.1110466441	-0.032516019
Inverse v	109	0.13923897896	0.087279	0.1106400985	-0.031828858
Inverse v	109	0.13737056751	0.086080	0.1105230980	-0.03134637
Inverse v	109	0.14090654047	0.088453	0.1111583426	-0.032461564
Inverse v	109	0.14885668024	0.093473	0.1112710338	-0.03435084
Inverse v	109	0.14600221193	0.091763	0.1115926654	-0.03385374
Inverse v	109	0.13721555358	0.086275	0.1117384234	-0.031885173
Inverse v	109	0.13065216556	0.082164	0.1118050658	-0.03038997
Inverse v	109	0.13082045420	0.082271	0.1118092252	-0.030430986
Inverse v	109	0.14156860879	0.089031	0.1118129606	-0.032933002
Inverse v	109	0.13856077775	0.087179	0.1119773385	-0.032311624
Inverse v	109	0.13232174950	0.083461	0.1128688218	-0.031262098
Inverse v	109	0.13139197487	0.082833	0.1126885408	-0.03096107
Inverse v	109	0.12886986527	0.081270	0.1128096332	-0.030420365
Inverse v	109	0.14111901335	0.089139	0.1133938466	-0.033594881
Inverse v	109	0.13557145405	0.085778	0.1139973844	-0.032554903
Inverse v	109	0.13175515171	0.083420	0.1142691700	-0.031761253
Inverse v	109	0.14478746230	0.091737	0.1145015865	-0.035018182
Inverse v	109	0.14361938404	0.091007	0.1145414187	-0.034755273

Inverse v	109	0.1263784396	0.080120	0.114715219	-0.030658288
Inverse v	109	0.1355139590	0.086024	0.115186528	-0.033093201
Inverse v	109	0.1454856172	0.092440	0.115526213	-0.035697474
Inverse v	109	0.1364612906	0.086742	0.115677481	-0.033553823
Inverse v	109	0.1290167600	0.082057	0.115888353	-0.031816387
Inverse v	109	0.1432689316	0.091187	0.116148150	-0.035458349
Inverse v	109	0.1315807675	0.083789	0.116328814	-0.032646856
Inverse v	109	0.1314944411	0.083830	0.116745836	-0.032812834
Inverse v	109	0.1284771130	0.081923	0.116819480	-0.03209222
Inverse v	109	0.1371497704	0.087490	0.116999021	-0.034342669
Inverse v	109	0.1324394531	0.084589	0.117426424	-0.033356471
Inverse v	109	0.1343619497	0.085800	0.117352637	-0.03380683
Inverse v	109	0.1373325056	0.087894	0.118177659	-0.034940862
Inverse v	109	0.1308102451	0.083761	0.118358221	-0.033361979
Inverse v	109	0.1391970121	0.089211	0.118687763	-0.035657327
Inverse v	109	0.1309868060	0.083928	0.118596124	-0.033513249
Inverse v	109	0.1254041606	0.080487	0.119218671	-0.032350945
Inverse v	109	0.1406766769	0.090461	0.119922886	-0.036628163
Inverse v	109	0.1353543099	0.087042	0.119938606	-0.035249614
Inverse v	109	0.1358442343	0.087385	0.120054851	-0.035430944
Inverse v	109	0.1393747038	0.089563	0.119669162	-0.03616879
Inverse v	109	-0.141685267	0.091164	0.120144523	-0.320368172
Inverse v	109	0.1442683421	0.092843	0.120213142	-0.03770583
Inverse v	109	0.1284802794	0.082544	0.119592071	-0.033307882
Inverse v	109	-0.140365158	0.090285	0.120022645	-0.317325025
Inverse v	109	0.1268726182	0.081543	0.119733504	-0.032952195
Inverse v	109	0.1375244104	0.088635	0.120762499	-0.03620022
Inverse v	109	0.1425070419	0.091890	0.120966556	-0.037610656
Inverse v	109	0.1258158878	0.081220	0.121395306	-0.033388849
Inverse v	109	0.1372241849	0.088649	0.121621104	-0.036521643
Inverse v	109	0.1434326472	0.092768	0.122071978	-0.038393646
Inverse v	109	0.1391056406	0.090031	0.122328581	-0.037356596
Inverse v	109	0.1329353409	0.086018	0.122244171	-0.035661478
Inverse v	109	0.1381249098	0.089454	0.122569238	-0.037206051
Inverse v	109	0.1403188117	0.090887	0.122619114	-0.037820763
Inverse v	109	0.1273965490	0.082575	0.122883599	-0.034452101
Inverse v	109	0.1306937626	0.084701	0.122833909	-0.035321739
Inverse v	109	0.1327394642	0.086104	0.123167220	-0.036024708

Inverse v	109	0.1361117571(	0.08829	0.1231723828	-0.036942312
Inverse v	109	0.1338546167	0.08687	0.1233925992	-0.036429666
Inverse v	109	0.1427126441(	0.09260	0.1232983131	-0.038794822
Inverse v	109	0.1405144601	0.09138	0.1241490795	-0.03860252
Inverse v	109	0.1440003261	0.09373	0.1244644687	-0.039714037
Inverse v	109	0.1337378213(	0.08707	0.124585946(	-0.036938758
Inverse v	109	0.1278214480	0.08315	0.1242568155	-0.035162122
Inverse v	109	0.1271080562	0.08277	0.1246306759	-0.035126856
Inverse v	109	0.1350693983(	0.08794	0.1245599783	-0.037294663
Inverse v	109	0.1363044312	0.08887	0.125096514(	-0.037883324
Inverse v	109	0.1279603614	0.08350	0.1254342799	-0.035710541
Inverse v	109	0.1395969479(	0.09117	0.1257406102	-0.039102729
Inverse v	109	0.1294348487	0.08457	0.1258949785	-0.036323806
Inverse v	109	0.1298990860(	0.08480	0.1256070767	-0.036327552
Inverse v	109	0.1350431138	0.08823	0.1259024984	-0.03790111
Inverse v	109	-0.125461728	0.08191	0.1256245025	-0.286017456
Inverse v	109	0.1428094678	0.09336	0.1261217023	-0.040186701
Inverse v	109	0.1247055033	0.08170	0.1269260763	-0.035431388
Inverse v	109	0.1300477120	0.08520	0.1269150412	-0.036944368
Inverse v	109	0.1279727629	0.08387	0.1270575948	-0.036416567
Inverse v	109	0.1372089097	0.09006	0.1276637297	-0.039325842
Inverse v	109	0.1353205532(	0.08894	0.1281765615	-0.039018971
Inverse v	109	0.1276334691	0.08392	0.1282965235	-0.036854133
Inverse v	109	0.1324015582(	0.08728	0.1292733505	-0.038667375
Inverse v	109	0.1351104641	0.08905	0.1292308365	-0.039439123
Inverse v	109	0.1455714605	0.09592	0.1291345777	-0.042445445
Inverse v	109	0.1265959408	0.08342	0.129162887(	-0.03692469
Inverse v	109	0.1461883946	0.09622	0.1287090961	-0.042415447
Inverse v	109	0.1293290872	0.08516	0.128854166(	-0.037587162
Inverse v	109	0.1344200952	0.08850	0.1288199854	-0.039051267
Inverse v	109	0.1298114268	0.08556	0.1292180159	-0.037886705
Inverse v	109	0.1266061909	0.08348	0.1294019481	-0.037029779
Inverse v	109	0.1343832412	0.08872	0.1298522939	-0.039508506
Inverse v	109	0.1272475607	0.08402	0.129946844(	-0.037451191
Inverse v	109	0.1297602040	0.08573	0.1301454297	-0.03827758
Inverse v	109	0.1272464205	0.08410	0.1302952968	-0.037600329
Inverse v	109	0.1237456196	0.08178	0.1302482955	-0.036546265
Inverse v	109	0.1286847871	0.08524	0.1311701701	-0.038404715

Inverse	109	0.1262247556	0.08378	0.131909588	-0.037984854
Inverse	109	0.1325551638	0.08796	0.131850330	-0.039863419
Inverse	109	0.1425210527	0.09441	0.131166817	-0.042532403
Inverse	109	0.1394580926	0.09251	0.131722743	-0.041879433
Inverse	109	0.1281921587	0.08497	0.131408218	-0.03836048
Inverse	109	0.1314456317	0.08710	0.131305046	-0.03928838
Inverse	109	0.1379366410	0.09140	0.131270104	-0.041212272
Inverse	109	0.1312423130	0.08694	0.131181673	-0.039173069
Inverse	109	0.1315049863	0.08698	0.130568829	-0.038979937
Inverse	109	0.1253995262	0.08323	0.131900646	-0.037732742
Inverse	109	0.1349553927	0.08951	0.131634718	-0.040487265
Inverse	109	0.1403928682	0.09290	0.130770303	-0.041709746
Inverse	109	0.1226999945	0.08139	0.131706228	-0.036840132
Inverse	109	0.1399463096	0.09279	0.131518063	-0.041929587
Inverse	109	0.1328204747	0.08841	0.133017090	-0.040464763
Inverse	109	0.1359235464	0.09047	0.133004717	-0.04140448
Inverse	109	0.1229656331	0.08180	0.132809298	-0.037376445
Inverse	109	0.1244926883	0.08295	0.133449644	-0.038108777
Inverse	109	0.1378854829	0.09187	0.133406337	-0.042188396
Inverse	109	0.1280012054	0.08529	0.133413653	-0.039167284
Inverse	109	0.1260458452	0.08398	0.133386176	-0.038557312
Inverse	109	0.1264194926	0.08433	0.133856604	-0.038871605
Inverse	109	0.1380392359	0.09206	0.133791455	-0.042414218
Inverse	109	0.1360448219	0.09080	0.134061520	-0.041924942
Inverse	109	0.1290852063	0.08671	0.136582286	-0.040873323
Inverse	109	0.1271421901	0.08538	0.136483881	-0.040216088
Inverse	109	0.1341236677	0.08997	0.136038657	-0.042223889
Inverse	109	0.1322160521	0.08846	0.135009259	-0.04116618
Inverse	109	0.1351008009	0.09061	0.135957844	-0.04249484
Inverse	109	0.1253880894	0.08400	0.135522318	-0.039256377
Inverse	109	0.1213035239	0.08112	0.134832802	-0.037696581
Inverse	109	0.1416142966	0.09495	0.135862640	-0.04449833
Inverse	109	0.1349971798	0.09060	0.136253431	-0.042596237
Inverse	109	0.1341107221	0.08963	0.134612023	-0.041577075
Inverse	109	0.1224943791	0.08201	0.135282556	-0.038251759
Inverse	109	0.1270319480	0.08498	0.134964291	-0.039532887
Inverse	109	0.1234402821	0.08292	0.136604370	-0.039095075
Inverse	109	0.1254174278	0.08392	0.135054040	-0.039068262

Inverse v	109	0.12473257095	0.083510	0.13530437113	-0.038959828
Inverse v	109	0.12121828464	0.081255	0.13574693999	-0.038042352
Inverse v	109	0.13036548308	0.087393	0.13577761977	-0.040926481
Inverse v	109	0.13654753510	0.091624	0.13614604477	-0.043036192
Inverse v	109	0.12732862564	0.085330	0.13564998888	-0.03991852
Inverse v	109	0.13473987689	0.090453	0.13632418477	-0.042547057
Inverse v	109	0.13960594494	0.093843	0.13684119260	-0.04432592
Inverse v	109	0.13011602590	0.087470	0.13689684911	-0.041337107
Inverse v	109	0.13556408103	0.091174	0.13705081755	-0.043137976
Inverse v	109	0.12452396833	0.083830	0.13743259888	-0.039784427
Inverse v	109	0.12685611450	0.085444	0.13763218560	-0.04061448
Inverse v	109	0.13578719789	0.091449	0.13758908099	-0.043454234
Inverse v	109	0.13278173998	0.089389	0.13742836399	-0.042420833
Inverse v	109	0.13344559733	0.089988	0.13809459591	-0.042931349
Inverse v	109	0.13369605253	0.090123	0.13794174777	-0.042943213
Inverse v	109	0.13710364349	0.092447	0.13806478555	-0.044094318
Inverse v	109	0.12695809254	0.085645	0.13824341633	-0.040907445
Inverse v	109	0.16102291476	0.108693	0.13848645211	-0.052014795
Inverse v	109	0.12108734403	0.081744	0.13853071960	-0.039132492
Inverse v	109	0.13171475092	0.088969	0.13875301688	-0.042665198
Inverse v	109	0.12256817392	0.082944	0.13948363277	-0.040002654
Inverse v	109	0.20766115927	0.140448	0.13925926200	-0.067618317
Inverse v	109	0.12334057272	0.083455	0.13942870255	-0.040232032
Inverse v	109	0.12489825281	0.084475	0.13926850799	-0.040673054
Inverse v	109	0.13372153973	0.090554	0.13975719955	-0.0437654
Inverse v	109	0.12381606972	0.083815	0.13961103711	-0.040462806
Inverse v	109	0.13062365650	0.088469	0.13981598033	-0.042777235
Inverse v	109	0.12188436793	0.082591	0.14001134555	-0.039995055
Inverse v	109	0.12767874982	0.086537	0.14010098422	-0.041934774
Inverse v	109	0.12358324341	0.083800	0.14028278755	-0.040664935
Inverse v	109	0.13009344717	0.088233	0.14036493211	-0.042842918
Inverse v	109	0.13113126805	0.089047	0.14086042988	-0.043402386
Inverse v	109	0.13074067503	0.088840	0.14114581160	-0.043398089
Inverse v	109	0.13167708181	0.089520	0.14133913144	-0.043794183
Inverse v	109	0.13287861345	0.090395	0.14157139994	-0.044297163
Inverse v	109	-0.128131563	0.087171	0.14162043633	-0.298998828
Inverse v	109	0.12572129467	0.085610	0.14195894399	-0.042074318
Inverse v	109	0.12228970579	0.083280	0.14199505155	-0.040940676

Inverse v	109	0.13708453071	0.093361	0.14201608944	-0.045903408
Inverse v	109	0.12601229028	0.085833	0.14207772900	-0.042221823
Inverse v	109	0.12716082467	0.086657	0.14226933100	-0.042688229
Inverse v	109	0.12872845531	0.087871	0.14293115860	-0.043499693
Inverse v	109	0.12624057482	0.086274	0.14340177211	-0.042857837
Inverse v	109	0.12258797264	0.083769	0.14336064522	-0.041600929
Inverse v	109	0.13468949125	0.092041	0.14337153799	-0.045712559
Inverse v	109	0.12732317642	0.087127	0.14391927377	-0.043445865
Inverse v	109	0.11993581100	0.082113	0.14412234300	-0.041006602
Inverse v	109	0.13104386014	0.089650	0.14384353311	-0.044682244
Inverse v	109	0.21210657707	0.145153	0.14394492822	-0.072394297
Inverse v	109	-0.134768786	0.092277	0.14415984555	-0.315632546
Inverse v	109	0.12425496887	0.085130	0.14443275760	-0.042612386
Inverse v	109	0.13741211090	0.094208	0.14467446760	-0.04723565
Inverse v	109	0.13289725001	0.091207	0.14509032588	-0.045868526
Inverse v	109	0.12280329643	0.084292	0.14515271699	-0.042410298
Inverse v	109	0.12965370168	0.089091	0.14558765877	-0.044964699
Inverse v	109	0.12719695841	0.087443	0.14577394599	-0.044191928
Inverse v	109	0.11943048055	0.082144	0.14597436499	-0.04157367
Inverse v	109	0.11741213720	0.080797	0.14618006500	-0.040951846
Inverse v	109	0.11845465222	0.081559	0.14640074577	-0.041402867
Inverse v	109	0.12903893624	0.088900	0.14664287199	-0.0452068
Inverse v	109	0.13146123981	0.090567	0.14663132077	-0.04605034
Inverse v	109	0.13201155878	0.091005	0.14689578855	-0.046359835
Inverse v	109	0.13043541311	0.090081	0.14762391033	-0.0461238
Inverse v	109	0.12002598358	0.082920	0.14776322599	-0.04249877
Inverse v	109	0.12240517723	0.084585	0.14786165422	-0.043381463
Inverse v	109	0.11684409244	0.080830	0.14830461833	-0.041583548
Inverse v	109	0.12301907262	0.085141	0.14849031555	-0.0438575
Inverse v	109	0.11855182456	0.082123	0.14885577833	-0.042409667
Inverse v	109	0.12769840442	0.088433	0.14873658911	-0.045630822
Inverse v	109	0.12329005906	0.085425	0.14894887700	-0.044143035
Inverse v	109	0.12957621160	0.089805	0.14906247888	-0.046442929
Inverse v	109	0.13110522814	0.090894	0.14919333566	-0.047048287
Inverse v	109	0.12059249654	0.083651	0.14941411100	-0.043364657
Inverse v	109	0.11956591879	0.083008	0.14975133222	-0.043130217
Inverse v	109	0.12306919803	0.085440	0.14974968160	-0.044393253
Inverse v	109	0.13376252877	0.092813	0.14953070922	-0.048152667

Inverse v	109	0.11932267319	0.08288	0.1499570490	-0.043124482
Inverse v	109	0.12927255095	0.08988	0.1503869767	-0.046906145
Inverse v	109	0.12346266410	0.08582	0.1502784256	-0.044753274
Inverse v	109	0.12321219846	0.08566	0.1503303183	-0.044683844
Inverse v	109	0.11577321279	0.08053	0.1505496695	-0.042070873
Inverse v	109	0.12859543693	0.08949	0.1507439858	-0.046813819
Inverse v	109	0.12544828916	0.08741	0.1512645590	-0.045886271
Inverse v	109	0.12149667177	0.08466	0.1512765364	-0.044445716
Inverse v	109	0.12280569619	0.08562	0.1515255017	-0.045026703
Inverse v	109	0.12198889533	0.08510	0.1517431088	-0.044815885
Inverse v	109	0.13565835485	0.09462	0.1516539652	-0.049797336
Inverse v	109	0.12432860323	0.08680	0.1520872954	-0.04581836
Inverse v	109	0.12852770283	0.08981	0.1524328225	-0.047514155
Inverse v	109	0.13892678519	0.09707	0.1523848610	-0.051336238
Inverse v	109	0.12168045251	0.08508	0.1526836083	-0.045084773
Inverse v	109	-0.104327529	0.07303	0.1531471910	-0.247471776
Inverse v	109	0.13762379417	0.09634	0.1531599993	-0.05121101
Inverse v	109	0.12024666394	0.08417	0.1531446612	-0.04473867
Inverse v	109	0.11955195379	0.08373	0.1533887945	-0.044577662
Inverse v	109	0.12167790206	0.08531	0.1537924309	-0.045534374
Inverse v	109	0.12014798054	0.08443	0.1547404819	-0.045342189
Inverse v	109	0.12671555279	0.08911	0.1550298123	-0.047943115
Inverse v	109	0.12025916128	0.08467	0.1555454014	-0.04570735
Inverse v	109	0.12033576519	0.08476	0.1557139189	-0.045804172
Inverse v	109	0.12881838528	0.09081	0.1560700844	-0.049186153
Inverse v	109	0.11523715792	0.08122	0.1559731247	-0.043963188
Inverse v	109	0.13176718709	0.09288	0.1560294277	-0.050294194
Inverse v	109	0.12345092591	0.08707	0.1562447363	-0.047208707
Inverse v	109	0.11842256557	0.08356	0.1564662406	-0.045373397
Inverse v	109	0.12870961442	0.09092	0.1568855825	-0.049495065
Inverse v	109	0.12451643158	0.08803	0.1572471986	-0.048032917
Inverse v	109	0.12725655707	0.08996	0.1572320599	-0.049083504
Inverse v	109	0.12511471712	0.08850	0.1574800048	-0.048360958
Inverse v	109	0.12762692975	0.09033	0.1577087124	-0.049429465
Inverse v	109	0.11553402633	0.08175	0.1576078682	-0.044707027
Inverse v	109	0.12332651495	0.08741	0.1582960082	-0.048005752
Inverse v	109	0.11607778888	0.08228	0.1583513330	-0.045205574
Inverse v	109	0.14711696523	0.10418	0.1579475295	-0.057095192

Inverse \	109	0.1157458669(	0.08201!	0.158186856	-0.045012748
Inverse \	109	0.1155662772	0.08189	0.158187705	-0.044943234
Inverse \	109	0.1326794987	0.09417	0.158871059	-0.051901211
Inverse \	109	0.1186351155	0.08423	0.159035137	-0.046472359
Inverse \	109	0.1174070852	0.08337	0.159061975	-0.046001829
Inverse \	109	0.1244568719(	0.08847	0.159517756	-0.048953437
Inverse \	109	0.1186782466	0.08489	0.162119140	-0.047711402
Inverse \	109	0.1254747493	0.08957(	0.161258432	-0.050083101
Inverse \	109	0.1220245516	0.08717(	0.161563063	-0.048830091
Inverse \	109	0.1263339291	0.09039	0.162240668	-0.050840437
Inverse \	109	0.1306751978	0.09309	0.160412078	-0.051789547
Inverse \	109	0.1144150132	0.08163	0.161035306	-0.045583372
Inverse \	109	0.1167243262	0.08357	0.162510722(	-0.047078528
Inverse \	109	0.1226855471	0.08754(	0.161074075(	-0.048894266
Inverse \	109	0.1252047852	0.08920	0.160468072	-0.049644908
Inverse \	109	0.1164706413	0.08320	0.161554581	-0.046604305
Inverse \	109	0.1149428635	0.08226(	0.162351089(	-0.046298729
Inverse \	109	0.1270436219	0.09074	0.161492863	-0.050808771
Inverse \	109	0.1191667063	0.08512	0.161525708	-0.047671613
Inverse \	109	0.1148905790	0.08224	0.162430668	-0.046308204
Inverse \	109	0.1223179340	0.08713(	0.160366473	-0.048458747
Inverse \	109	0.1210156197	0.08628	0.160784398	-0.048111687
Inverse \	109	0.1207373059	0.08641	0.162375255	-0.048642462
Inverse \	109	0.1168774031	0.08352	0.161734140	-0.046837149
Inverse \	109	0.1234124363(	0.08828	0.162159683	-0.049631366
Inverse \	109	0.1222749328	0.08709	0.160330680(	-0.048427097
Inverse \	109	0.1181544929	0.08420	0.160559730	-0.04688556
Inverse \	109	0.1262499853	0.09038	0.162472757	-0.050904513
Inverse \	109	0.1264228053	0.09042	0.162077804	-0.050807441
Inverse \	109	0.1232522898	0.08769	0.159873489	-0.048626027
Inverse \	109	0.1204796213	0.08586	0.160563232	-0.047809617
Inverse \	109	0.1195757132	0.08569(	0.162885939	-0.048378428
Inverse \	109	0.1228052271	0.08800(	0.162890109(	-0.049686748
Inverse \	109	0.1224775947(	0.08789	0.163477861	-0.049794626
Inverse \	109	0.1234147228	0.08852	0.163283810	-0.050095634
Inverse \	109	0.1120193897	0.08038	0.163471290(	-0.045540268
Inverse \	109	0.1165563931	0.08364	0.163477993	-0.047387347
Inverse \	109	0.1232255245	0.08837	0.163195378	-0.049982439

Inverse	109	0.1313092330	0.09439	0.164188680	-0.053697004
Inverse	109	0.1314100391	0.09460	0.164830805	-0.05402012
Inverse	109	0.1211695464	0.08720	0.164664831	-0.049743265
Inverse	109	0.1148722996	0.08274	0.165038491	-0.047301481
Inverse	109	0.1833238828	0.13199	0.164867703	-0.075383494
Inverse	109	0.1165916224	0.08378	0.164051363	-0.04762497
Inverse	109	0.1228385116	0.08847	0.164999316	-0.050565687
Inverse	109	0.1145533357	0.08241	0.164562307	-0.046987902
Inverse	109	0.1183356336	0.08514	0.164594922	-0.048552232
Inverse	109	0.1273423785	0.09155	0.164271961	-0.052110244
Inverse	109	0.1238659011	0.08907	0.164363290	-0.050725412
Inverse	109	0.1292688669	0.09286	0.163936736	-0.052753833
Inverse	109	0.1144076691	0.08220	0.163983442	-0.046706918
Inverse	109	0.1211472980	0.08729	0.165203379	-0.049952105
Inverse	109	0.1193680806	0.08604	0.165341035	-0.049273389
Inverse	109	0.1140848509	0.08239	0.166188172	-0.047415482
Inverse	109	0.1155295077	0.08343	0.166159920	-0.048004997
Inverse	109	0.1155252565	0.08339	0.165970802	-0.047930224
Inverse	109	0.1185901919	0.08565	0.166211202	-0.0492971
Inverse	109	0.1151268625	0.08324	0.166686077	-0.048040121
Inverse	109	0.1162219232	0.08404	0.166689416	-0.048498364
Inverse	109	0.1232630066	0.08912	0.166657314	-0.051423321
Inverse	109	0.1193998779	0.08630	0.166525722	-0.049759177
Inverse	109	0.2134627331	0.15450	0.167106105	-0.089373358
Inverse	109	-0.112530684	0.08148	0.167270366	-0.272237907
Inverse	109	0.1184052674	0.08590	0.168105784	-0.049970015
Inverse	109	0.1237630237	0.08989	0.168591113	-0.052431939
Inverse	109	0.1256611273	0.09124	0.168468136	-0.053184399
Inverse	109	0.1345522998	0.09766	0.168292105	-0.056868283
Inverse	109	0.1176488742	0.08545	0.168571789	-0.049834092
Inverse	109	0.1172460032	0.08522	0.168905237	-0.049794161
Inverse	109	0.1167435217	0.08488	0.169019740	-0.049625456
Inverse	109	0.1409638052	0.10250	0.169080898	-0.059949866
Inverse	109	0.1163917644	0.08469	0.169384194	-0.04961778
Inverse	109	0.1225626433	0.08920	0.169461251	-0.052280007
Inverse	109	0.1129313858	0.08217	0.169359393	-0.048133255
Inverse	109	0.1148048356	0.08360	0.169717220	-0.049069134
Inverse	109	0.1102289396	0.08033	0.170051185	-0.047236457

Inverse v	109	0.1149676964	0.08386	0.170410938	-0.049405503
Inverse v	109	0.1270343293	0.09264	0.170316361	-0.054550754
Inverse v	109	0.1199487385	0.08742	0.170076928	-0.051412015
Inverse v	109	0.1223608669	0.08923	0.170328927	-0.052549031
Inverse v	109	0.1173775206	0.08565	0.170569094	-0.050503185
Inverse v	109	0.1244459067	0.09090	0.171024770	-0.053734159
Inverse v	109	0.1181839311	0.08634	0.171058761	-0.051043757
Inverse v	109	0.1177312752	0.08596	0.170835862	-0.050760459
Inverse v	109	0.1239141785	0.09058	0.171355665	-0.053641752
Inverse v	109	0.1294017618	0.09470	0.171819666	-0.056218212
Inverse v	109	0.1208693870	0.08844	0.171743073	-0.052480367
Inverse v	109	-0.137709397	0.10091	0.172396373	-0.335512027
Inverse v	109	0.1222656203	0.08961	0.172436898	-0.053370506
Inverse v	109	0.1246162085	0.09132	0.172411089	-0.054385802
Inverse v	109	0.1248026139	0.09144	0.172311697	-0.054425637
Inverse v	109	0.1204165149	0.08827	0.172522077	-0.052597679
Inverse v	109	0.1160750018	0.08512	0.172673912	-0.050760309
Inverse v	109	0.1176529131	0.08630	0.172806101	-0.0515024
Inverse v	109	0.1127018179	0.08269	0.172931162	-0.049382248
Inverse v	109	0.1122287046	0.08237	0.173047116	-0.04921851
Inverse v	109	0.1259348714	0.09251	0.173449960	-0.055399276
Inverse v	109	0.1196111829	0.08790	0.173618660	-0.052685023
Inverse v	109	-0.110474344	0.08122	0.173820124	-0.269683743
Inverse v	109	0.1112636482	0.08178	0.173714691	-0.04904397
Inverse v	109	0.1212334313	0.08921	0.174197807	-0.053634698
Inverse v	109	0.1184620221	0.08726	0.174630399	-0.052580244
Inverse v	109	0.1141170470	0.08408	0.174726052	-0.050688259
Inverse v	109	0.1162010675	0.08570	0.175178033	-0.05178988
Inverse v	109	0.1192117714	0.08818	0.176432394	-0.053632836
Inverse v	109	0.1358747966	0.10056	0.176676876	-0.061240809
Inverse v	109	0.1159014507	0.08581	0.176803164	-0.05228759
Inverse v	109	0.1167171997	0.08661	0.177789393	-0.053041584
Inverse v	109	0.1200795742	0.08909	0.177722775	-0.054542771
Inverse v	109	0.1173798595	0.08708	0.177685436	-0.053301803
Inverse v	109	0.1341957890	0.09959	0.177843715	-0.061009096
Inverse v	109	0.1120518709	0.08319	0.178008468	-0.05100378
Inverse v	109	0.1137154333	0.08448	0.178306510	-0.051874686
Inverse v	109	-0.107257315	0.07967	0.178252039	-0.263423654

Inverse v	109	0.11420943465	0.084893	0.17851347772	-0.052179336
Inverse v	109	0.11519815133	0.085620	0.17847838060	-0.052617491
Inverse v	109	0.11342481875	0.084367	0.17881491433	-0.051935577
Inverse v	109	0.13409433343	0.099770	0.17896759444	-0.061468545
Inverse v	109	0.13026554681	0.097011	0.17934291460	-0.059877508
Inverse v	109	0.09657191675	0.071938	0.17945704360	-0.044426976
Inverse v	109	0.12271017740	0.091398	0.17940663299	-0.056430869
Inverse v	109	0.11801684247	0.087853	0.17916288533	-0.054176
Inverse v	109	0.11118076530	0.082910	0.17993108280	-0.051324545
Inverse v	109	0.12840438517	0.095721	0.17977656155	-0.059208905
Inverse v	109	0.11217478737	0.083659	0.17997174099	-0.051798727
Inverse v	109	0.11557755894	0.086251	0.18024340300	-0.05347543
Inverse v	109	0.12401010190	0.092520	0.18015732622	-0.057341161
Inverse v	109	0.13545920416	0.101151	0.18051271011	-0.062796761
Inverse v	109	0.11580093690	0.086559	0.18095602788	-0.053855899
Inverse v	109	0.11589836760	0.086701	0.18130216333	-0.05403596
Inverse v	109	0.11070170860	0.082864	0.18156893422	-0.051712302
Inverse v	109	0.11273060317	0.084370	0.18150173388	-0.052634613
Inverse v	109	0.12211347958	0.091488	0.18196218744	-0.057204455
Inverse v	109	0.11045909227	0.082783	0.18209471788	-0.051794113
Inverse v	109	0.11066403374	0.082985	0.18235384011	-0.051986581
Inverse v	109	0.12184382179	0.091393	0.18246745633	-0.057285032
Inverse v	109	0.11229858797	0.084290	0.18280055160	-0.052923066
Inverse v	109	0.11713287495	0.087993	0.18313860722	-0.055334453
Inverse v	109	0.11132607537	0.083678	0.18338435299	-0.052683269
Inverse v	109	0.11872754971	0.089281	0.18358098077	-0.056264405
Inverse v	109	0.11438845924	0.086078	0.18388380755	-0.054324637
Inverse v	109	0.10776865551	0.081143	0.18413159444	-0.05127063
Inverse v	109	0.11965109219	0.090227	0.18480304800	-0.057193985
Inverse v	109	0.11563650100	0.087163	0.18462160244	-0.055204378
Inverse v	109	0.15698284388	0.118379	0.18480652944	-0.075040639
Inverse v	109	0.12252052233	0.092491	0.18527932311	-0.058761994
Inverse v	109	0.11945089898	0.090164	0.18523213355	-0.0572708
Inverse v	109	0.13089207637	0.098870	0.18554465755	-0.062893987
Inverse v	109	0.11060919980	0.083605	0.18583697244	-0.053256875
Inverse v	109	0.11047888875	0.083607	0.18636640760	-0.053391117
Inverse v	109	0.11333566860	0.085784	0.18644749177	-0.054802667
Inverse v	109	0.11172490105	0.084628	0.18677644788	-0.054147602

Inverse v	109	0.12071050701	0.09142(	0.186705244	-0.05847353
Inverse v	109	0.11937110187	0.09044	0.186919233	-0.057910769
Inverse v	109	0.11313284244	0.08577	0.187193965	-0.05498912
Inverse v	109	-0.114441044	0.08674	0.187099850	-0.284470774
Inverse v	109	0.1132716107(	0.08601	0.187866223	-0.055313231
Inverse v	109	0.10954944691	0.08322	0.188054240	-0.053565052
Inverse v	109	0.11774450651	0.08943	0.188008825	-0.057554061
Inverse v	109	0.12454572341	0.09464	0.188205787	-0.060961239
Inverse v	109	0.1128920486(	0.08587	0.188635040	-0.055420552
Inverse v	109	0.10874578177	0.08275	0.188835309	-0.053458542
Inverse v	109	0.10742331755	0.08209	0.190719840	-0.053491722
Inverse v	109	0.11133297338	0.08510	0.190812617	-0.055473431
Inverse v	109	0.1098221278(	0.08398	0.190983196	-0.054783903
Inverse v	109	0.11660597891	0.08924	0.191366992	-0.058319161
Inverse v	109	0.1080326140(	0.08271	0.191521015	-0.054087513
Inverse v	109	0.10840541112	0.08315	0.192346690	-0.054576691
Inverse v	109	0.10630714218	0.08152	0.192218962	-0.053474412
Inverse v	109	0.1203736999(	0.09232	0.192290691	-0.060579335
Inverse v	109	-0.103420309	0.07927	0.192051278	-0.258804277
Inverse v	109	0.11270855618	0.08651	0.192679981	-0.056870127
Inverse v	109	0.10576105605	0.08118	0.192666415	-0.053359727
Inverse v	109	0.10827751142	0.08316	0.192936205	-0.054728151
Inverse v	109	0.12083354195	0.09284	0.193097971	-0.06114063
Inverse v	109	0.10653820282	0.08187	0.193196208	-0.053942728
Inverse v	109	0.1090601906(	0.08388	0.193558582	-0.055353378
Inverse v	109	0.10666779312	0.08205	0.193636118	-0.054167105
Inverse v	109	0.10642913623	0.08186	0.193577841	-0.054024925
Inverse v	109	0.10630172961	0.08187	0.194154793	-0.054167823
Inverse v	109	0.11876735372	0.09150	0.194315313	-0.06058443
Inverse v	109	0.12591768487	0.09712	0.194822328	-0.064448065
Inverse v	109	0.10860711733	0.08394	0.195718499	-0.055917789
Inverse v	109	0.10447951169	0.08075	0.195738542	-0.053799735
Inverse v	109	0.1058405581(	0.08186	0.196068186	-0.054618835
Inverse v	109	0.10548425732	0.08151	0.195663963	-0.054290449
Inverse v	109	0.11382183202	0.08803	0.196062050	-0.05873519
Inverse v	109	0.10909796179	0.08443	0.196321608	-0.056393542
Inverse v	109	0.11928217817	0.09229	0.196234213	-0.061622495
Inverse v	109	0.11587428512	0.08966	0.196259142	-0.059871733

Inverse v	109	0.1056454505	0.08185	0.196827733	-0.054790233
Inverse v	109	0.1189671578	0.09217	0.196802890	-0.061689162
Inverse v	109	0.1147123774	0.08883	0.196583197	-0.059397423
Inverse v	109	0.1065569866	0.08268	0.197487770	-0.055501581
Inverse v	109	0.1099197127	0.08527	0.197411997	-0.057224837
Inverse v	109	0.1154673169	0.08969	0.197957300	-0.060326648
Inverse v	109	0.1147166215	0.08909	0.197872317	-0.059901349
Inverse v	109	0.1171291296	0.09094	0.197763063	-0.061117648
Inverse v	109	0.1055317345	0.08207	0.198514911	-0.055335539
Inverse v	109	0.1194452085	0.09289	0.198522760	-0.062634249
Inverse v	109	0.1074379230	0.08376	0.199636573	-0.056744483
Inverse v	109	0.1058823298	0.08262	0.200042617	-0.056069038
Inverse v	109	0.1084725931	0.08460	0.199827444	-0.057361335
Inverse v	109	0.1058442928	0.08260	0.200093365	-0.05606716
Inverse v	109	0.1133245478	0.08845	0.200162630	-0.060056243
Inverse v	109	0.1114840100	0.08710	0.200604391	-0.059248351
Inverse v	109	0.1073027570	0.08386	0.200712123	-0.057065543
Inverse v	109	0.1074947613	0.08403	0.200843284	-0.057215621
Inverse v	109	0.1117770816	0.08742	0.201044287	-0.059571395
Inverse v	109	0.1120860289	0.08784	0.201979874	-0.06009303
Inverse v	109	0.1060429408	0.08313	0.202105664	-0.056898559
Inverse v	109	0.1222712595	0.09576	0.201675174	-0.065426811
Inverse v	109	0.1130104788	0.08856	0.201932598	-0.060570464
Inverse v	109	0.1062117751	0.08322	0.201910258	-0.056918468
Inverse v	109	0.0996331268	0.07806	0.201828809	-0.053365364
Inverse v	109	0.1093179430	0.08561	0.201646537	-0.05848489
Inverse v	109	0.1134967850	0.08882	0.201320887	-0.060594747
Inverse v	109	0.1111438508	0.08717	0.202310415	-0.059713023
Inverse v	109	0.1051668535	0.08249	0.202372665	-0.056524129
Inverse v	109	0.1211860597	0.09518	0.202947387	-0.065371306
Inverse v	109	0.1127193638	0.08853	0.202940431	-0.060801451
Inverse v	109	0.1151974872	0.09053	0.203239960	-0.062255779
Inverse v	109	0.1099322051	0.08643	0.203425356	-0.059479766
Inverse v	109	0.1129548341	0.08902	0.204492261	-0.061526268
Inverse v	109	0.1059402229	0.08352	0.204648328	-0.057761852
Inverse v	109	0.1055376920	0.08321	0.204700128	-0.05756104
Inverse v	109	0.1173465118	0.09270	0.205567459	-0.064349189
Inverse v	109	0.1112636479	0.08793	0.205759275	-0.061086443

Inverse v	109	0.11144805162	0.088064	0.20568024088	-0.061157593
Inverse v	109	0.10457281481	0.082731	0.20622964988	-0.057581099
Inverse v	109	0.10391366692	0.082253	0.20646864400	-0.057303042
Inverse v	109	0.10364560741	0.082153	0.20708787755	-0.057374691
Inverse v	109	0.10517478008	0.083521	0.20794095499	-0.058528202
Inverse v	109	0.11188168268	0.088970	0.20859629777	-0.062511557
Inverse v	109	0.10316594350	0.082171	0.20929671855	-0.057889403
Inverse v	109	0.10858565541	0.086537	0.20955833000	-0.061027936
Inverse v	109	0.10531848810	0.084150	0.21073509388	-0.059616819
Inverse v	109	0.10928078655	0.087392	0.21113130344	-0.062008357
Inverse v	109	0.09885340765	0.079121	0.21152427311	-0.056225028
Inverse v	109	0.13017811592	0.104400	0.21245627688	-0.074458539
Inverse v	109	0.11153081279	0.089563	0.21303105322	-0.0640132
Inverse v	109	0.10652092585	0.085537	0.21301692221	-0.061132594
Inverse v	109	0.11421732785	0.091869	0.21377492999	-0.065847482
Inverse v	109	0.10688207989	0.085973	0.21379448660	-0.06162583
Inverse v	109	0.10339655375	0.083195	0.21393562800	-0.059666392
Inverse v	109	0.10991288923	0.088488	0.21419584611	-0.063525212
Inverse v	109	0.10489555145	0.084502	0.21448492533	-0.060729831
Inverse v	109	0.11509916684	0.092978	0.21574745588	-0.067138148
Inverse v	109	0.10690566320	0.086399	0.21596150300	-0.062437753
Inverse v	109	0.10674211529	0.086398	0.21665657177	-0.062598275
Inverse v	109	0.09874273534	0.080017	0.21719556888	-0.058090874
Inverse v	109	0.09831574464	0.079769	0.21776509600	-0.058033142
Inverse v	109	0.10206280282	0.082914	0.21834475111	-0.060449466
Inverse v	109	0.10302551962	0.083735	0.21855811088	-0.061095689
Inverse v	109	0.11163380468	0.090883	0.21932424744	-0.066496499
Inverse v	109	0.10866373575	0.088452	0.21926260911	-0.064704142
Inverse v	109	0.10839805373	0.088162	0.21887325777	-0.064399871
Inverse v	109	0.10623199009	0.086444	0.21910529022	-0.063198304
Inverse v	109	0.09980427351	0.081210	0.21909005188	-0.059369137
Inverse v	109	0.10456465667	0.085199	0.21971049911	-0.062425495
Inverse v	109	0.10452419539	0.085179	0.21978451888	-0.062428136
Inverse v	109	0.10602738302	0.086528	0.22044212955	-0.063567516
Inverse v	109	0.10206866418	0.083398	0.22100406911	-0.061392973
Inverse v	109	-0.108149446	0.088435	0.22136263855	-0.281483895
Inverse v	109	0.10257194437	0.083887	0.22143244499	-0.061848115
Inverse v	109	0.10642525190	0.087043	0.22145582922	-0.06418019

Inverse v	109	0.10542989898	0.086253	0.22157876913	-0.063624917
Inverse v	109	0.09980980128	0.081814	0.22248065500	-0.060545843
Inverse v	109	0.10318479760	0.084533	0.22222355690	-0.062501014
Inverse v	109	0.10460022344	0.085725	0.22239603760	-0.063421026
Inverse v	109	0.10329305964	0.084643	0.22233528700	-0.062606673
Inverse v	109	0.10420524981	0.085490	0.22291142733	-0.063368147
Inverse v	109	0.10343674	0.085270	0.22514546500	-0.063704933
Inverse v	109	0.10668821535	0.088005	0.22539966377	-0.065801967
Inverse v	109	0.10511588257	0.086459	0.22406996390	-0.06434543
Inverse v	109	0.10072673875	0.083211	0.22608978222	-0.062367481
Inverse v	109	0.11414340068	0.093757	0.22344150833	-0.069621972
Inverse v	109	0.10812168857	0.089118	0.22503918400	-0.066550278
Inverse v	109	0.10494460100	0.086455	0.22480104877	-0.064507688
Inverse v	109	0.10673690780	0.087760	0.22389941060	-0.065274376
Inverse v	109	0.10853056259	0.089504	0.22529528988	-0.066898792
Inverse v	109	0.10320152953	0.085220	0.22589760233	-0.063830639
Inverse v	109	0.10175733408	0.083680	0.22397427322	-0.062255651
Inverse v	109	0.10471030907	0.086490	0.22602556690	-0.064810555
Inverse v	109	0.10323852018	0.085200	0.22565366800	-0.06376571
Inverse v	109	0.10668599356	0.087881	0.22475814022	-0.06556215
Inverse v	109	0.10863552018	0.089738	0.22605618055	-0.067251668
Inverse v	109	0.10896097190	0.089732	0.22464088355	-0.066915707
Inverse v	109	0.10720945640	0.088072	0.22349737711	-0.06541343
Inverse v	109	0.10062768637	0.083012	0.22543650799	-0.062076944
Inverse v	109	0.11172746194	0.092182	0.22550430160	-0.068950766
Inverse v	109	0.09975788850	0.082057	0.22409792155	-0.061075304
Inverse v	109	0.10079981690	0.083277	0.22612364399	-0.062424635
Inverse v	109	0.10965978844	0.090165	0.22390506055	-0.067064004
Inverse v	109	0.10330631330	0.084877	0.22353399288	-0.063045098
Inverse v	109	0.11070198488	0.091519	0.22643471655	-0.068677119
Inverse v	109	0.10194745934	0.084307	0.22657443244	-0.063295706
Inverse v	109	0.10003046412	0.082983	0.22804088177	-0.062617886
Inverse v	109	0.10104379934	0.083880	0.22835154244	-0.06336198
Inverse v	109	0.10223444940	0.084962	0.22886651400	-0.064292782
Inverse v	109	0.10462720323	0.086955	0.22888850711	-0.06580558
Inverse v	109	0.10556768299	0.087730	0.22888286477	-0.066395013
Inverse v	109	0.10212270941	0.085000	0.22961583077	-0.064490408
Inverse v	109	0.10436692680	0.086953	0.23003566611	-0.066061135

Inverse v	109	0.10190156985	0.08488	0.2299623060	-0.064474443
Inverse v	109	0.10618170652	0.08863	0.2309319054	-0.067543493
Inverse v	109	0.10110789744	0.08447	0.2313740401	-0.064472842
Inverse v	109	0.10276010465	0.08584	0.2313090164	-0.065502943
Inverse v	109	0.10706613115	0.08954	0.2318407468	-0.068447625
Inverse v	109	0.11209702616	0.09409	0.2335427105	-0.072334606
Inverse v	109	-0.107425391	0.09019	0.2336141784	-0.284197868
Inverse v	109	0.10128188284	0.08505	0.2337644093	-0.065434781
Inverse v	109	0.10440133733	0.08777	0.2342971911	-0.067646026
Inverse v	109	0.10560584405	0.08879	0.2342954113	-0.068425815
Inverse v	109	0.09977214472	0.08400	0.2349341050	-0.064870513
Inverse v	109	0.09666142511	0.08146	0.2353995989	-0.063006638
Inverse v	109	0.09923386945	0.08364	0.2354564796	-0.064703341
Inverse v	109	0.10572854235	0.08912	0.2354944902	-0.068952232
Inverse v	109	0.09757374115	0.08241	0.2364124351	-0.063950141
Inverse v	109	0.12143593192	0.10259	0.2365575053	-0.079651725
Inverse v	109	0.09962455931	0.08414	0.2364321616	-0.065301195
Inverse v	109	0.10002029648	0.08447	0.2363958856	-0.065547775
Inverse v	109	0.10439871854	0.08815	0.2363084703	-0.06838492
Inverse v	109	0.10549304980	0.08919	0.2369103568	-0.069326067
Inverse v	109	0.10813615347	0.09155	0.2375578340	-0.071310572
Inverse v	109	0.09709642964	0.08232	0.2382085910	-0.064254003
Inverse v	109	0.10047997801	0.08534	0.2390584374	-0.066795553
Inverse v	109	0.09960589590	0.08467	0.2394466719	-0.066351581
Inverse v	109	0.09866662407	0.08393	0.2397787708	-0.065842107
Inverse v	109	0.10061307278	0.08556	0.2396473026	-0.06709409
Inverse v	109	0.09534657585	0.08117	0.2401852256	-0.063764091
Inverse v	109	0.09942325306	0.08470	0.2404826750	-0.066595402
Inverse v	109	0.10467681592	0.08918	0.2404967475	-0.070119559
Inverse v	109	0.10899480321	0.09294	0.2409477172	-0.073186623
Inverse v	109	0.10180063690	0.08686	0.2412205166	-0.06845465
Inverse v	109	0.10242435442	0.08753	0.2419704307	-0.069147196
Inverse v	109	0.10062916545	0.08596	0.2417827883	-0.067868085
Inverse v	109	0.09983452453	0.08531	0.2419402548	-0.067388071
Inverse v	109	0.09629741347	0.08228	0.2418857698	-0.064981864
Inverse v	109	0.09662733991	0.08273	0.2428246072	-0.065527419
Inverse v	109	0.10624075581	0.09095	0.2427740549	-0.072027587
Inverse v	109	0.09744420320	0.08339	0.2425977140	-0.066002633

Inverse v	109	0.11175643147	0.09577	0.24328605785	-0.075970892
Inverse v	109	0.0986691444	0.08461	0.2435597280	-0.067170545
Inverse v	109	0.1050421363	0.09007	0.2435370294	-0.071500556
Inverse v	109	0.0985644653	0.08458	0.2439259053	-0.067227998
Inverse v	109	0.0960429615	0.08249	0.2443483215	-0.065652916
Inverse v	109	0.0964904443	0.08286	0.2442304800	-0.065918224
Inverse v	109	0.0984612833	0.08461	0.2445976760	-0.067393675
Inverse v	109	0.0985491909	0.08474	0.2448603458	-0.067546287
Inverse v	109	0.0960734203	0.08277	0.2457743487	-0.066163214
Inverse v	109	0.1000056835	0.08613	0.2456169608	-0.068814977
Inverse v	109	0.0983865057	0.08466	0.2451902172	-0.067550728
Inverse v	109	0.1061578834	0.09147	0.2458222625	-0.073126308
Inverse v	109	0.1033465143	0.08898	0.2454744493	-0.071061183
Inverse v	109	0.1071740965	0.09227	0.2454572206	-0.073686433
Inverse v	109	0.0988509482	0.08512	0.2455669429	-0.068002713
Inverse v	109	0.1030911412	0.08888	0.2461090679	-0.071119566
Inverse v	109	0.0944959973	0.08152	0.2463868595	-0.065283966
Inverse v	109	0.1008421997	0.08700	0.2464602868	-0.069694839
Inverse v	109	0.0957469569	0.08258	0.2462807827	-0.066111859
Inverse v	109	0.1103609261	0.09549	0.2478069253	-0.07680599
Inverse v	109	0.0949803546	0.08221	0.2479850774	-0.066162531
Inverse v	109	0.1015495623	0.08811	0.2491526670	-0.071164278
Inverse v	109	0.0977294166	0.08462	0.2481498942	-0.068135294
Inverse v	109	-0.096247938	0.08353	0.2492414945	-0.259975583
Inverse v	109	0.1024704398	0.08907	0.2500044163	-0.072123417
Inverse v	109	0.0992282912	0.08576	0.2472727871	-0.068868188
Inverse v	109	0.0957184483	0.08292	0.2484011358	-0.066819586
Inverse v	109	0.0972465828	0.08402	0.2471189520	-0.067439201
Inverse v	109	0.0963949952	0.08353	0.2485433641	-0.067341089
Inverse v	109	0.1007771311	0.08728	0.2482418352	-0.070293357
Inverse v	109	0.1028332528	0.08924	0.2491961910	-0.07207995
Inverse v	109	0.1040529609	0.09011	0.2482255070	-0.072572194
Inverse v	109	0.1041911496	0.09058	0.2500428622	-0.073348943
Inverse v	109	0.0936772617	0.08135	0.2495205948	-0.065771376
Inverse v	109	0.0991417480	0.08614	0.2497874456	-0.069703155
Inverse v	109	0.1050075907	0.09111	0.2491377869	-0.073581993
Inverse v	109	0.1107858639	0.09601	0.2485557417	-0.077399405
Inverse v	109	0.0945869928	0.08210	0.2493333609	-0.066346443

Inverse v	109	0.11151765922	0.096730	0.2489931074	-0.078085828
Inverse v	109	0.1038164407	0.090230	0.2499412038	-0.073047186
Inverse v	109	0.0950950449	0.082632	0.2498043602	-0.066863842
Inverse v	109	0.1090906030	0.09489	0.2503348146	-0.076912692
Inverse v	109	0.0999088651	0.08699	0.2508001745	-0.070606645
Inverse v	109	0.1032908931	0.08991	0.2506674684	-0.072947395
Inverse v	109	0.0961412412	0.08375	0.2510386195	-0.068026607
Inverse v	109	0.1075010575	0.09368	0.2512080533	-0.076130099
Inverse v	109	0.0948842449	0.08270	0.2512882884	-0.067222562
Inverse v	109	0.0942048312	0.08214	0.2514442520	-0.066794179
Inverse v	109	0.0941630408	0.08219	0.2519506960	-0.06693653
Inverse v	109	0.0935727323	0.08170	0.2521157770	-0.066572639
Inverse v	109	0.0932396755	0.08160	0.2532017661	-0.066701364
Inverse v	109	0.0984071791	0.08627	0.2540164713	-0.070688007
Inverse v	109	0.0979426066	0.08600	0.2547880262	-0.0706279
Inverse v	109	0.0960527438	0.08434	0.2547795156	-0.06926213
Inverse v	109	0.0968758838	0.08512	0.2550875576	-0.069963814
Inverse v	109	0.1054181135	0.09263	0.2551317343	-0.076149896
Inverse v	109	0.0961697922	0.08459	0.2555929020	-0.069630078
Inverse v	109	0.1000303022	0.08805	0.2559352659	-0.07254946
Inverse v	109	0.0938468402	0.08267	0.2563348203	-0.068200863
Inverse v	109	0.0922427102	0.08137	0.2569747865	-0.067249553
Inverse v	109	0.0962380881	0.08496	0.2573338462	-0.07028801
Inverse v	109	0.0913811860	0.08078	0.2580033347	-0.066963334
Inverse v	109	0.0957621240	0.08470	0.2582780271	-0.070269422
Inverse v	109	0.0956076069	0.08458	0.2583224332	-0.0701715
Inverse v	109	0.0997056348	0.08815	0.2580532905	-0.073081549
Inverse v	109	0.0941653487	0.08365	0.2603155979	-0.069797535
Inverse v	109	0.1036184875	0.09204	0.2602730880	-0.07678833
Inverse v	109	0.0959509283	0.08523	0.2603101575	-0.071119141
Inverse v	109	0.0959473209	0.08520	0.2601548156	-0.071062027
Inverse v	109	0.0949717706	0.08430	0.2599682554	-0.0702748
Inverse v	109	0.1062414880	0.09432	0.2600244986	-0.078635697
Inverse v	109	0.0937869153	0.08338	0.2606691605	-0.069638198
Inverse v	109	0.1007893742	0.08968	0.2610830351	-0.074990145
Inverse v	109	-0.108224502	0.09631	0.2611595293	-0.297001375
Inverse v	109	0.0961550250	0.08552	0.2608883658	-0.071473608
Inverse v	109	0.0935481202	0.08324	0.2611155330	-0.069613566

Inverse v	109	0.10728304284	0.09550	0.26131359	-0.079912099
Inverse v	109	0.1040776742	0.09278	0.26199088	-0.077782551
Inverse v	109	0.0972728744	0.08681	0.26249744	-0.072877517
Inverse v	109	0.0995013837	0.08906	0.26389605	-0.075057781
Inverse v	109	0.0942381456	0.08445	0.26448951	-0.071293055
Inverse v	109	0.0944678819	0.08480	0.26532897	-0.071758633
Inverse v	109	0.0897687673	0.08054	0.26507336	-0.068104682
Inverse v	109	0.0974051771	0.08744	0.26533560	-0.073992206
Inverse v	109	0.0954827709	0.08568	0.26513109	-0.072460001
Inverse v	109	0.0962025337	0.08645	0.26584159	-0.073257935
Inverse v	109	0.0953413749	0.08566	0.26575164	-0.072570567
Inverse v	109	0.0950624400	0.08551	0.26629504	-0.072548651
Inverse v	109	0.0939468558	0.08453	0.26641588	-0.071739136
Inverse v	109	0.0935802435	0.08419	0.26634420	-0.071434449
Inverse v	109	0.0911772089	0.08207	0.26663655	-0.069698417
Inverse v	109	0.0946412491	0.08524	0.26692907	-0.072448591
Inverse v	109	0.0953169847	0.08589	0.26712376	-0.07303438
Inverse v	109	0.1003929025	0.09057	0.26767400	-0.077127741
Inverse v	109	0.0926388329	0.08354	0.26752268	-0.071118805
Inverse v	109	0.1026763896	0.09262	0.26764774	-0.078872084
Inverse v	109	0.0976815794	0.08830	0.26863409	-0.075391596
Inverse v	109	0.0939717913	0.08493	0.26857683	-0.072508431
Inverse v	109	-0.077942573	0.07042	0.26838856	-0.215971165
Inverse v	109	0.0923238011	0.08358	0.26937700	-0.071510436
Inverse v	109	0.1020408899	0.09243	0.26961102	-0.079125428
Inverse v	109	0.0892671015	0.08092	0.26999372	-0.069346954
Inverse v	109	-0.093400834	0.08478	0.27064988	-0.259587373
Inverse v	109	0.0919637523	0.08358	0.27123563	-0.071865946
Inverse v	109	0.0915549093	0.08320	0.27116381	-0.071522012
Inverse v	109	0.0916430584	0.08332	0.27143590	-0.071683573
Inverse v	109	0.0922581366	0.08424	0.27345740	-0.072859326
Inverse v	109	0.0957121356	0.08718	0.27228179	-0.075167678
Inverse v	109	0.0961409486	0.08783	0.27370084	-0.076013049
Inverse v	109	0.0968034621	0.08826	0.27276544	-0.076199156
Inverse v	109	0.0945419674	0.08629	0.27325651	-0.074592098
Inverse v	109	0.0913349918	0.08315	0.27205292	-0.071652259
Inverse v	109	0.0967500496	0.08833	0.27337451	-0.076376821
Inverse v	109	0.0925614037	0.08425	0.27195693	-0.072581309

Inverse v	109	0.09217111817	0.08392(	0.272098921	-0.07232398
Inverse v	109	0.0996064384	0.09098(	0.27359839	-0.078714927
Inverse v	109	0.0970976474	0.08832(	0.27163591	-0.076021559
Inverse v	109	0.0951989224	0.08687	0.27317553	-0.075081673
Inverse v	109	0.0986753670	0.09009	0.27343876	-0.077920367
Inverse v	109	0.0980251006	0.08934	0.27258339	-0.077094283
Inverse v	109	-0.092356098	0.08418	0.27263958	-0.257367282
Inverse v	109	0.0976629285	0.08934	0.27434937	-0.077452918
Inverse v	109	0.0978360005	0.08948	0.27422890	-0.07754614
Inverse v	109	0.1017423982	0.09315	0.27477355	-0.080849509
Inverse v	109	0.0918824594	0.08425	0.27548300	-0.0732582
Inverse v	109	0.0967427816	0.08879	0.27594857	-0.077302032
Inverse v	109	0.0913170085	0.08384	0.27612418	-0.073026673
Inverse v	109	0.0960182800	0.08819	0.27630585	-0.076851685
Inverse v	109	0.0939700989	0.08637	0.27664791	-0.075332885
Inverse v	109	0.0892511969	0.08213	0.27720160	-0.071735337
Inverse v	109	0.0888064292	0.08173	0.27725845	-0.071396815
Inverse v	109	0.0939474139	0.08654	0.27766345	-0.075672877
Inverse v	109	0.0907048826	0.08353	0.27754443	-0.07302052
Inverse v	109	0.0950493311	0.08767	0.27830053	-0.076788105
Inverse v	109	0.0901975018	0.08325	0.27862202	-0.072977536
Inverse v	109	-0.093322035	0.08634	0.27980865	-0.262566826
Inverse v	109	0.0908170494	0.08413	0.28038908	-0.074083711
Inverse v	109	0.0923786315	0.08567	0.28094910	-0.075552964
Inverse v	109	-0.090671793	0.08419	0.28149363	-0.255687254
Inverse v	109	0.1003290463	0.09320	0.28175058	-0.082359379
Inverse v	109	0.0907756647	0.08437	0.28201657	-0.074608469
Inverse v	109	0.0955294566	0.08886	0.28235429	-0.078637787
Inverse v	109	0.0965633859	0.08980	0.28226443	-0.079456026
Inverse v	109	0.0907724396	0.08449	0.28268092	-0.074834259
Inverse v	109	0.0968401956	0.09023	0.28318562	-0.080021947
Inverse v	109	0.0965790342	0.09001	0.28332866	-0.079858556
Inverse v	109	0.0994141589	0.09260	0.28304210	-0.08209477
Inverse v	109	0.0929574594	0.08669	0.28359429	-0.07695792
Inverse v	109	0.0886865298	0.08279	0.28410270	-0.073593128
Inverse v	109	0.0884140429	0.08263	0.28467144	-0.073558186
Inverse v	109	0.0924584747	0.08641	0.28465516	-0.076917324
Inverse v	109	0.0883434724	0.08254	0.28449159	-0.073439047

Inverse v	109	0.0898789763	0.08404	0.2849098889	-0.074858505
Inverse v	109	0.0888253597	0.08311	0.2851888091	-0.074075267
Inverse v	109	0.0918399086	0.08596	0.2853713999	-0.076653081
Inverse v	109	0.0911476380	0.08539	0.2858039460	-0.076225492
Inverse v	109	0.0866467440	0.08127	0.2863981604	-0.072657795
Inverse v	109	0.0925569895	0.08691	0.2869303652	-0.077801875
Inverse v	109	0.1094781590	0.10279	0.2868568885	-0.091994812
Inverse v	109	0.0946823223	0.08898	0.2873009630	-0.079722446
Inverse v	109	0.0895790115	0.08425	0.2877205990	-0.075569171
Inverse v	109	0.0883112747	0.08308	0.2878227116	-0.074534195
Inverse v	109	0.0874313921	0.08229	0.2880653134	-0.073872733
Inverse v	109	0.0918512934	0.08654	0.2885205549	-0.07776729
Inverse v	109	0.0863552734	0.08149	0.2893313971	-0.073382377
Inverse v	109	0.0974616841	0.09201	0.2894895779	-0.082879446
Inverse v	109	0.0909436930	0.08590	0.2897462580	-0.077426262
Inverse v	109	0.0888642431	0.08394	0.2898066993	-0.07567651
Inverse v	109	0.0889841229	0.08410	0.2900581906	-0.07586453
Inverse v	109	0.1029095771	0.09729	0.2901760378	-0.087783434
Inverse v	109	0.0910706616	0.08617	0.2905833450	-0.07782721
Inverse v	109	0.0889211425	0.08412	0.2905222772	-0.075969398
Inverse v	109	0.0883458791	0.08361	0.2906948797	-0.07553655
Inverse v	109	0.0885372077	0.08388	0.2912343589	-0.07588389
Inverse v	109	0.0956581646	0.09064	0.2912606388	-0.08199683
Inverse v	109	0.0892412584	0.08466	0.2918468901	-0.076697839
Inverse v	109	0.0951856080	0.09045	0.2926576522	-0.082104269
Inverse v	109	-0.068461107	0.06501	0.2923162976	-0.195884566
Inverse v	109	0.0923877000	0.08763	0.2917788626	-0.079377812
Inverse v	109	0.0925945420	0.08798	0.2926428135	-0.07986399
Inverse v	109	0.0867516145	0.08237	0.2923054742	-0.074711492
Inverse v	109	0.0875728563	0.08323	0.2927234482	-0.075559974
Inverse v	109	0.0966629939	0.09179	0.2923130836	-0.083250123
Inverse v	109	0.0907894237	0.08643	0.2935211396	-0.078615031
Inverse v	109	0.0901664545	0.08587	0.2937430920	-0.07815297
Inverse v	109	0.0911530104	0.08693	0.2943840557	-0.0792343
Inverse v	109	0.0862473052	0.08234	0.2949270497	-0.075151292
Inverse v	109	0.0854941986	0.08168	0.2952419233	-0.074599432
Inverse v	109	0.0939181391	0.08973	0.2952885761	-0.081966877
Inverse v	109	0.0875320153	0.08368	0.2955914855	-0.076496253

Inverse v	109	0.09376108705	0.08991(	0.297060298(	-0.082475102
Inverse v	109	0.08995889521	0.08638:	0.29768663111	-0.079349945
Inverse v	109	0.09240067828	0.08871!	0.2976476769	-0.081489744
Inverse v	109	0.09142386969	0.08784(	0.2979705833	-0.080743311
Inverse v	109	0.09059004725	0.08722{	0.2990206356	-0.080378038
Inverse v	109	0.08720016048	0.08396!	0.2990502364	-0.077380367
Inverse v	109	0.09402384789	0.09052(	0.2989743835	-0.083407771
Inverse v	109	0.08734307100	0.08415:	0.2993139966	-0.077597185
Inverse v	109	0.09385466233	0.09047	0.2995850882	-0.083481653
Inverse v	109	0.10149332257	0.09798{	0.3003103168	-0.090564051
Inverse v	109	0.09518356670	0.09189(	0.3002763401	-0.084921098
Inverse v	109	0.08896883455	0.08585	0.3000569847	-0.079300037
Inverse v	109	0.09078316370	0.08763!	0.3002406923	-0.080982467
Inverse v	109	0.09994973769	0.09657{	0.3007140342	-0.089344731
Inverse v	109	0.08948857040	0.08644(	0.3005485712	-0.079935521
Inverse v	109	0.08045297442	0.07773	0.3006660841	-0.071901526
Inverse v	109	0.09056099201	0.08757(	0.3010990860	-0.081088844
Inverse v	109	-0.087993291	0.08508!	0.3010795769	-0.25476956
Inverse v	109	0.09130466101	0.08848:	0.3021200656	-0.082120615
Inverse v	109	0.08563250596	0.08311	0.3028512520	-0.077265176
Inverse v	109	0.08677742507	0.08423{	0.3029429760	-0.078329542
Inverse v	109	0.09634871064	0.09357	0.3031590750	-0.087051001
Inverse v	109	0.09004707452	0.08751!	0.3035354559	-0.08149095
Inverse v	109	0.08728433335	0.08481:	0.3034146790	-0.078949193
Inverse v	109	0.08758268070	0.08523:	0.3041518026	-0.079473514
Inverse v	109	0.09409209667	0.09152!	0.3039320594	-0.085298694
Inverse v	109	0.08329226577	0.08106(	0.3041729170	-0.07558728
Inverse v	109	0.09454326935	0.09205:	0.3043989501	-0.085881818
Inverse v	109	0.09067010265	0.08840	0.3050815260	-0.082607881
Inverse v	109	0.08532064565	0.08321:	0.3052087531	-0.077776988
Inverse v	109	0.10132966390	0.09888(	0.3055028193	-0.092488374
Inverse v	109	0.09047239605	0.08834:	0.3057814271	-0.082678146
Inverse v	109	0.08439617180	0.08247!	0.3061691397	-0.07725492
Inverse v	109	-0.09370062	0.09160:	0.3063574505	-0.273243193
Inverse v	109	0.08865339780	0.08689(	0.3076255970	-0.08166397
Inverse v	109	0.08653156969	0.08480	0.3075725534	-0.079691199
Inverse v	109	0.08724889207	0.08549	0.3074669787	-0.080315242
Inverse v	109	0.09419275900	0.09263:	0.3092252615	-0.087365963

Inverse \	109	0.0915752204	0.09005	0.309221909	-0.084936913
Inverse \	109	-0.100503011	0.09890	0.30954532	-0.294353088
Inverse \	109	0.0978730334	0.09632	0.309589609	-0.090921627
Inverse \	109	0.0892537373	0.08789	0.309913440	-0.083029788
Inverse \	109	0.0830357379	0.08197	0.311110214	-0.07764233
Inverse \	109	0.0887024416	0.08742	0.310279907	-0.082646671
Inverse \	109	0.0859383609	0.08480	0.310874604	-0.080275464
Inverse \	109	0.0841000394	0.08302	0.311057099	-0.078619639
Inverse \	109	0.0768197358	0.07574	0.310508505	-0.071645333
Inverse \	109	0.0874935383	0.08638	0.311114540	-0.081812098
Inverse \	109	0.0919949863	0.09078	0.310927466	-0.085952428
Inverse \	109	0.0828977357	0.08196	0.311861168	-0.077762438
Inverse \	109	0.0857045931	0.08492	0.312863664	-0.080739892
Inverse \	109	0.0868705719	0.08617	0.313425643	-0.08203437
Inverse \	109	0.0847393651	0.08411	0.313728778	-0.080125053
Inverse \	109	0.0884379901	0.08777	0.313666549	-0.083600152
Inverse \	109	0.0886282928	0.08800	0.313903800	-0.083864593
Inverse \	109	0.0884325545	0.08828	0.316486791	-0.084600482
Inverse \	109	0.0877415538	0.08757	0.316369406	-0.083897786
Inverse \	109	0.0832804524	0.08319	0.316801210	-0.079777542
Inverse \	109	-0.086811117	0.08680	0.317253043	-0.256940704
Inverse \	109	0.0832326062	0.08328	0.317633502	-0.080012219
Inverse \	109	0.0829772813	0.08301	0.317530375	-0.079732097
Inverse \	109	0.0888356077	0.08909	0.318721672	-0.08579065
Inverse \	109	0.0822078345	0.08236	0.318205470	-0.079217773
Inverse \	109	0.0867180417	0.08689	0.318278159	-0.083589514
Inverse \	109	-0.08458691	0.08483	0.318710368	-0.250857517
Inverse \	109	0.0755518559	0.07574	0.318570556	-0.072915848
Inverse \	109	0.0903300791	0.09056	0.318586309	-0.087184237
Inverse \	109	0.0821895040	0.08247	0.318981078	-0.079458976
Inverse \	109	0.0871997887	0.08757	0.319403849	-0.0844527
Inverse \	109	0.0944277404	0.09491	0.319793513	-0.091602679
Inverse \	109	0.0996933930	0.10024	0.319986958	-0.096789322
Inverse \	109	0.0902289872	0.09075	0.320129102	-0.087652856
Inverse \	109	0.0892065930	0.08978	0.320457389	-0.086778998
Inverse \	109	0.0810600373	0.08162	0.320679736	-0.078927629
Inverse \	109	0.0805906424	0.08130	0.321555671	-0.078758762
Inverse \	109	0.0809333357	0.08172	0.321999123	-0.079240383

Inverse v	109	0.0862693053	0.08710	0.321970286	-0.084454561
Inverse v	109	0.0809652166	0.08179	0.322248088	-0.079354061
Inverse v	109	0.0876775302	0.08865	0.322694756	-0.086093135
Inverse v	109	0.0840387208	0.08511	0.323442981	-0.082777834
Inverse v	109	0.0826531982	0.08379	0.323949066	-0.081584792
Inverse v	109	0.0860450535	0.08736	0.324668514	-0.085187228
Inverse v	109	0.0880431969	0.08954	0.325516746	-0.087472897
Inverse v	109	-0.080883236	0.08222	0.325291422	-0.242050709
Inverse v	109	0.0792628618	0.08061	0.325511999	-0.078747886
Inverse v	109	0.0830861838	0.08463	0.326255878	-0.082801241
Inverse v	109	0.0818436830	0.08337	0.326299556	-0.081577755
Inverse v	109	0.0825947468	0.08414	0.326304846	-0.082328182
Inverse v	109	0.0879294113	0.08957	0.326308675	-0.087647019
Inverse v	109	0.0829447484	0.08464	0.327131676	-0.082960372
Inverse v	109	0.0900333390	0.09197	0.327626144	-0.090234471
Inverse v	109	0.0815363590	0.08326	0.327481815	-0.081669807
Inverse v	109	0.0896193184	0.09154	0.327616695	-0.089816021
Inverse v	109	0.0894143476	0.09146	0.328280475	-0.089856439
Inverse v	109	0.0850671232	0.08700	0.328196066	-0.08545796
Inverse v	109	0.0899530931	0.09213	0.328926057	-0.090638727
Inverse v	109	0.1173492780	0.12012	0.328603833	-0.11808683
Inverse v	109	0.0835704627	0.08559	0.328903674	-0.084199684
Inverse v	109	0.0862188680	0.08837	0.329235189	-0.086986709
Inverse v	109	0.0812663222	0.08337	0.329676566	-0.082139115
Inverse v	109	-0.094087853	0.09661	0.330112601	-0.283444703
Inverse v	109	0.0813676341	0.08371	0.331061156	-0.082710619
Inverse v	109	0.0859786098	0.08842	0.330874287	-0.087330712
Inverse v	109	0.0809873714	0.08330	0.330969021	-0.082292966
Inverse v	109	0.0792524651	0.08157	0.331255864	-0.080624902
Inverse v	109	0.0906098388	0.09336	0.331781688	-0.092377838
Inverse v	109	0.0877772263	0.09045	0.331864412	-0.089520287
Inverse v	109	0.0866150327	0.08927	0.331926609	-0.088357521
Inverse v	109	0.0813493351	0.08390	0.332261066	-0.083099607
Inverse v	109	0.0898592789	0.09288	0.333347366	-0.092201268
Inverse v	109	0.0884182399	0.09140	0.333362629	-0.090728327
Inverse v	109	-0.09959385	0.10289	0.333090319	-0.301269987
Inverse v	109	0.0848075121	0.08781	0.334176665	-0.08731287
Inverse v	109	0.0812873143	0.08427	0.334792241	-0.083898922

Inverse v	109	0.0794506087(	0.08239	0.3349297117	-0.082049135
Inverse v	109	0.0868269916	0.09014	0.3354628180	-0.089861563
Inverse v	109	0.0829651977	0.08612	0.3354101109	-0.085846389
Inverse v	109	0.0863999034	0.08987	0.3363959167	-0.089759368
Inverse v	109	-0.094744307	0.09854	0.3363368186	-0.287893213
Inverse v	109	0.0867766833	0.09044	0.3373461187	-0.090499085
Inverse v	109	0.0829013287	0.08648	0.3377566356	-0.086601461
Inverse v	109	0.0845715310	0.08822	0.3377612336	-0.088347855
Inverse v	109	0.0775696336(	0.08095	0.3379732346	-0.081102923
Inverse v	109	0.0841555185	0.08789	0.3383146004	-0.088110485
Inverse v	109	0.0827423416	0.08657	0.3392185084	-0.086948152
Inverse v	109	0.0801008743	0.08371	0.3386633440	-0.083983711
Inverse v	109	0.0811659241	0.08491	0.3391576930	-0.085270647
Inverse v	109	0.1066306324	0.11153	0.3390575151	-0.111977809
Inverse v	109	0.0794691922	0.08312	0.3390453288	-0.083450201
Inverse v	109	0.0797062771	0.08343	0.3394359460	-0.083831374
Inverse v	109	0.0821596193	0.08604	0.3396319490	-0.086480113
Inverse v	109	0.0803603745	0.08422	0.34000802	-0.084714751
Inverse v	109	-0.078044059	0.08193	0.3408209504	-0.238631169
Inverse v	109	0.0836380289	0.08777	0.3406619343	-0.088402828
Inverse v	109	0.0784801512	0.08241	0.3409571997	-0.083049859
Inverse v	109	-0.081061051	0.08506	0.3406527427	-0.247797969
Inverse v	109	0.0855810517	0.08979	0.3405725767	-0.090423969
Inverse v	109	0.0801060654	0.08459	0.3436642887	-0.085697724
Inverse v	109	0.0819008957	0.08648	0.3436513325	-0.087613288
Inverse v	109	0.0868293153(	0.09201	0.3453548434	-0.093521116
Inverse v	109	0.0800954350	0.08516	0.3469915410	-0.086833802
Inverse v	109	0.0765203566	0.08136	0.3470039132	-0.082962047
Inverse v	109	0.0770052893	0.08192	0.3472674836	-0.083575557
Inverse v	109	0.0792102289	0.08437	0.3478132687	-0.086155721
Inverse v	109	0.0783763738(	0.08356	0.3482972269	-0.085413099
Inverse v	109	-0.080328383	0.08583	0.3493478219	-0.248563444
Inverse v	109	0.0823610725	0.08817	0.3502470469	-0.090453335
Inverse v	109	0.0797967521	0.08545	0.3504044452	-0.08769177
Inverse v	109	0.0791450601(	0.08472	0.3502165325	-0.086910821
Inverse v	109	0.0816136982	0.08757	0.3513720559	-0.090032921
Inverse v	109	-0.076907914	0.08262	0.3519296746	-0.238844903
Inverse v	109	0.0764757733	0.08223	0.3524081670	-0.084711388

Inverse v	109	0.0801920921	0.08628	0.352699825	-0.088930326
Inverse v	109	0.0743628095	0.08004	0.352878292	-0.082523993
Inverse v	109	0.0838303891	0.09029	0.353172094	-0.093138588
Inverse v	109	0.0847135916	0.09139	0.353970066	-0.094416568
Inverse v	109	0.0763976589	0.08255	0.354726044	-0.085402109
Inverse v	109	0.0767638251	0.08292	0.354609729	-0.08577214
Inverse v	109	0.0808015042	0.08723	0.354323989	-0.090182089
Inverse v	109	0.0776231890	0.08386	0.354692113	-0.086760492
Inverse v	109	0.0751133080	0.08124	0.355224301	-0.084131201
Inverse v	109	0.0798749437	0.08650	0.355805295	-0.089669139
Inverse v	109	0.0794662837	0.08618	0.356522397	-0.089462041
Inverse v	109	0.0826379976	0.08975	0.357223759	-0.093289118
Inverse v	109	0.0833572348	0.09054	0.357257721	-0.094113592
Inverse v	109	0.0843480296	0.09169	0.357619749	-0.095367511
Inverse v	109	0.0759915585	0.08266	0.357939224	-0.08602696
Inverse v	109	0.0876994098	0.09568	0.359370791	-0.099838736
Inverse v	109	0.0844778272	0.09225	0.359820446	-0.096340372
Inverse v	109	0.0836158387	0.09130	0.359771063	-0.095338946
Inverse v	109	0.0839129578	0.09169	0.360130962	-0.095812317
Inverse v	109	0.0749194012	0.08190	0.360348118	-0.08561598
Inverse v	109	0.0777994542	0.08518	0.361065171	-0.089156314
Inverse v	109	0.0774637280	0.08481	0.361048474	-0.0887658
Inverse v	109	0.0794127833	0.08707	0.361744475	-0.091246423
Inverse v	109	0.0731322484	0.08026	0.362215344	-0.084184229
Inverse v	109	0.0757498619	0.08320	0.362585422	-0.087323109
Inverse v	109	0.0760984816	0.08363	0.362888180	-0.087828371
Inverse v	109	0.0858332592	0.09451	0.363785395	-0.099409736
Inverse v	109	0.0766080318	0.08439	0.363998160	-0.088798673
Inverse v	109	0.0819627370	0.09033	0.364235648	-0.09509311
Inverse v	109	0.0804299537	0.08872	0.364670517	-0.093472378
Inverse v	109	-0.077652623	0.08572	0.365024764	-0.245673997
Inverse v	109	0.0793671334	0.08772	0.365590082	-0.092566619
Inverse v	109	0.1156195676	0.12776	0.365481075	-0.134791226
Inverse v	109	0.0767951436	0.08491	0.365780040	-0.089632815
Inverse v	109	-0.093671223	0.10371	0.366441162	-0.296952664
Inverse v	109	0.0768167557	0.08510	0.366755015	-0.089997022
Inverse v	109	-0.076953185	0.08535	0.367298925	-0.244253029
Inverse v	109	0.0799226596	0.08881	0.368173067	-0.094150328

Inverse	109	0.0750187764	0.08344	0.368662351	-0.088540487
Inverse	109	0.0727277755	0.08108	0.369759272	-0.086200272
Inverse	109	0.0812630988	0.09062	0.369879477	-0.096361331
Inverse	109	0.0794826165	0.08860	0.369686498	-0.094180012
Inverse	109	0.0732361213	0.08161	0.369515197	-0.086721202
Inverse	109	0.0774379904	0.08654	0.370925901	-0.092196095
Inverse	109	0.0794518569	0.08883	0.371123954	-0.094665833
Inverse	109	-0.082655475	0.09225	0.370282617	-0.263475608
Inverse	109	0.0783327137	0.08757	0.371076864	-0.093315491
Inverse	109	0.0802702276	0.08972	0.371014242	-0.095600572
Inverse	109	0.0750400230	0.08378	0.370465542	-0.089183035
Inverse	109	0.0754034596	0.08447	0.372053886	-0.090163829
Inverse	109	0.0818580710	0.09190	0.373108911	-0.098279037
Inverse	109	0.0769276539	0.08637	0.373131895	-0.092367705
Inverse	109	-0.077534931	0.08695	0.372556309	-0.247961395
Inverse	109	0.0818042903	0.09182	0.372983692	-0.098167313
Inverse	109	0.0762559894	0.08555	0.372777768	-0.091436949
Inverse	109	0.0773255760	0.08681	0.373111347	-0.092838178
Inverse	109	0.0733724356	0.08257	0.374221911	-0.088467677
Inverse	109	0.0729603906	0.08212	0.374305545	-0.087999036
Inverse	109	0.0731010669	0.08222	0.373992992	-0.088063237
Inverse	109	0.0773097086	0.08690	0.373713864	-0.093033754
Inverse	109	0.0742674400	0.08353	0.373991491	-0.089467826
Inverse	109	0.0742734396	0.08378	0.375343410	-0.089939189
Inverse	109	0.0775704143	0.08765	0.376177659	-0.094231518
Inverse	109	0.0730796513	0.08261	0.376387570	-0.088847398
Inverse	109	0.0730448600	0.08262	0.376664472	-0.088899035
Inverse	109	0.0791689203	0.08957	0.376770516	-0.096391314
Inverse	109	0.0804232235	0.09110	0.377374929	-0.098144545
Inverse	109	0.0735931497	0.08351	0.378201700	-0.090092949
Inverse	109	0.0820520256	0.09310	0.378154286	-0.100430185
Inverse	109	0.0714145178	0.08109	0.378502700	-0.08752616
Inverse	109	-0.076781376	0.08730	0.379174702	-0.247907628
Inverse	109	0.0713837976	0.08123	0.379527897	-0.087830561
Inverse	109	0.0723583777	0.08248	0.380372023	-0.089315854
Inverse	109	0.0812111572	0.09272	0.381101796	-0.100521556
Inverse	109	0.0716972510	0.08180	0.380768820	-0.088633294
Inverse	109	0.0785011407	0.08962	0.381070780	-0.09715571

Inverse v	109	0.07345408254	0.083930	0.3814785923	-0.091050126
Inverse v	109	0.07653104919	0.087575	0.3821775122	-0.095116014
Inverse v	109	-0.07587111	0.086845	0.3823157907	-0.24608748
Inverse v	109	0.07077095184	0.081090	0.3828409323	-0.08817856
Inverse v	109	0.07662571680	0.087835	0.3830045406	-0.095532619
Inverse v	109	0.07676481808	0.088322	0.3847698415	-0.096347472
Inverse v	109	0.07315323617	0.084147	0.3846566325	-0.091775294
Inverse v	109	0.07403409588	0.085204	0.3849013881	-0.092966357
Inverse v	109	0.07394152718	0.085179	0.3853595169	-0.093010979
Inverse v	109	0.07213572003	0.083318	0.3866087757	-0.091168356
Inverse v	109	0.07583069789	0.087580	0.3865781073	-0.095827148
Inverse v	109	0.07552842916	0.087299	0.3869463004	-0.095577798
Inverse v	109	0.08085281378	0.093525	0.3873136794	-0.102457363
Inverse v	109	0.07703404101	0.089093	0.3872371286	-0.097590021
Inverse v	109	0.07769392161	0.089988	0.3879318929	-0.098683979
Inverse v	109	0.08613455551	0.099793	0.3880692198	-0.109461561
Inverse v	109	0.07197950751	0.083457	0.3884303499	-0.091597493
Inverse v	109	0.07676825682	0.089088	0.3888484973	-0.097845267
Inverse v	109	-0.075425281	0.087799	0.3903067330	-0.247512608
Inverse v	109	0.07227118390	0.084115	0.3902366933	-0.092595513
Inverse v	109	0.07180668728	0.083692	0.3909035819	-0.092231087
Inverse v	109	0.07216168660	0.084164	0.3912276369	-0.092799864
Inverse v	109	0.07776324982	0.090787	0.3916977982	-0.100180024
Inverse v	109	0.07802855991	0.091204	0.3922557464	-0.10073234
Inverse v	109	0.07567133370	0.088470	0.3923989871	-0.097741702
Inverse v	109	0.06964023632	0.081520	0.3929570517	-0.090139854
Inverse v	109	0.07287216640	0.085362	0.3932802040	-0.094437373
Inverse v	109	0.07108403741	0.083561	0.3949491115	-0.09269705
Inverse v	109	0.06991671867	0.082102	0.3944465578	-0.091003653
Inverse v	109	0.07116643475	0.083552	0.3943481971	-0.092596214
Inverse v	109	-0.077472295	0.091077	0.3949823578	-0.255984796
Inverse v	109	0.07100239044	0.083460	0.3949211693	-0.092580908
Inverse v	109	0.07128862168	0.083998	0.3960531152	-0.093348045
Inverse v	109	0.07148723380	0.084437	0.3972004344	-0.094009731
Inverse v	109	0.07111295801	0.084012	0.3972990748	-0.093551944
Inverse v	109	0.07099216660	0.083934	0.3976634437	-0.093519995
Inverse v	109	0.07393961126	0.087500	0.3980981297	-0.097560666
Inverse v	109	0.07647122455	0.090600	0.3986430820	-0.101106068

Inverse \	109	0.07381620822	0.087479	0.3987772269	-0.097644513
Inverse \	109	0.0735127513	0.087131	0.3988356559	-0.097264256
Inverse \	109	0.0693850169	0.08233	0.3993641987	-0.091983665
Inverse \	109	0.0712935212	0.084744	0.4001948755	-0.09480626
Inverse \	109	-0.08926915	0.106169	0.4004491853	-0.297360875
Inverse \	109	0.0714821250	0.08508	0.4008271366	-0.09528073
Inverse \	109	0.0706099328	0.08433	0.4024266523	-0.094678805
Inverse \	109	0.0684505699	0.08181	0.4027828554	-0.09190474
Inverse \	109	0.0709609344	0.08482	0.4028487255	-0.095298545
Inverse \	109	0.0681002523	0.08136	0.4026169576	-0.09137814
Inverse \	109	0.0738963662	0.08829	0.4026575173	-0.099170389
Inverse \	109	0.0777285355	0.09300	0.4032944316	-0.104559905
Inverse \	109	0.0747447141	0.08947	0.4034983581	-0.100622123
Inverse \	109	0.0775854316	0.09293	0.4037891134	-0.104558933
Inverse \	109	0.0700271486	0.08400	0.4045290275	-0.094631936
Inverse \	109	-0.322062184	0.38642	0.4045995710	-1.079460247
Inverse \	109	0.0752089358	0.09043	0.4055899143	-0.102034253
Inverse \	109	0.0738020683	0.08870	0.4054088031	-0.100058521
Inverse \	109	0.0692156294	0.08365	0.4079933287	-0.094740981
Inverse \	109	0.0744257807	0.08998	0.4082042958	-0.10195189
Inverse \	109	0.0742326038	0.08996	0.4093179630	-0.102105876
Inverse \	109	0.1693971802	0.20519	0.4090564607	-0.232778805
Inverse \	109	-0.070250804	0.08511	0.4091565346	-0.237073075
Inverse \	109	0.0723334947	0.08755	0.4087077354	-0.099270015
Inverse \	109	-0.068666761	0.08323	0.4093782898	-0.231804671
Inverse \	109	0.0698894019	0.08479	0.4098406350	-0.09631726
Inverse \	109	0.0731253281	0.08873	0.4099040647	-0.100800374
Inverse \	109	0.0786526132	0.09555	0.4104420755	-0.108634642
Inverse \	109	0.0734951046	0.08971	0.4126893338	-0.102354079
Inverse \	109	0.0733970379	0.08973	0.4133715509	-0.102474134
Inverse \	109	0.0703684723	0.08570	0.4116171419	-0.097614007
Inverse \	109	0.0707019637	0.08621	0.4121546223	-0.098270748
Inverse \	109	0.0718199546	0.08775	0.4131160707	-0.100178175
Inverse \	109	0.0764806089	0.09321	0.4119628773	-0.106227706
Inverse \	109	0.0690282280	0.08426	0.4127047193	-0.096138653
Inverse \	109	0.0895123076	0.10937	0.4131453045	-0.124869796
Inverse \	109	0.0851837690	0.10411	0.4132687652	-0.118885405
Inverse \	109	-0.080299608	0.09799	0.4125413905	-0.272368877

Inverse v	109	0.07324276347	0.08931	0.41219770906	-0.101818415
Inverse v	109	0.0747378703	0.09141	0.4136104807	-0.104437792
Inverse v	109	0.0689022546	0.08432	0.4138789304	-0.096378165
Inverse v	109	0.0727033166	0.08923	0.4152086866	-0.102192592
Inverse v	109	-0.067683262	0.08340	0.4171023877	-0.231165666
Inverse v	109	0.0667087312	0.08225	0.4173860324	-0.094517939
Inverse v	109	0.0711084696	0.08786	0.4183458030	-0.101106479
Inverse v	109	0.0763970615	0.09434	0.4180760329	-0.108518923
Inverse v	109	0.0705722708	0.08721	0.4183911779	-0.100360743
Inverse v	109	-0.074076164	0.09147	0.4180364342	-0.253359257
Inverse v	109	0.0696971411	0.08628	0.4192582511	-0.09943114
Inverse v	109	0.0693445294	0.08623	0.4213083641	-0.099672067
Inverse v	109	0.0667204377	0.08309	0.4220100748	-0.096146332
Inverse v	109	0.0719791491	0.08972	0.4224463298	-0.103889568
Inverse v	109	0.0656431914	0.08202	0.4235256140	-0.095118391
Inverse v	109	0.0665581571	0.08326	0.4240704650	-0.09663593
Inverse v	109	0.0685537587	0.08606	0.4257276300	-0.100135943
Inverse v	109	0.0683383605	0.08598	0.4267418451	-0.100190403
Inverse v	109	0.0697224192	0.08772	0.4267295493	-0.10221499
Inverse v	109	0.0703715548	0.08860	0.4270903430	-0.103302116
Inverse v	109	0.0663706790	0.08371	0.4278619242	-0.09770273
Inverse v	109	0.0684487317	0.08634	0.4279409369	-0.100790726
Inverse v	109	0.0846899907	0.10699	0.4286462247	-0.125025999
Inverse v	109	0.0715304313	0.09053	0.4294674265	-0.105914265
Inverse v	109	0.0707301956	0.08959	0.4298526059	-0.104875928
Inverse v	109	0.0659448892	0.08354	0.4299194589	-0.097804199
Inverse v	109	0.0654275828	0.08297	0.4304177086	-0.09721261
Inverse v	109	0.0663619851	0.08422	0.4307489009	-0.098719514
Inverse v	109	0.0680703317	0.08640	0.4308267815	-0.101289451
Inverse v	109	-0.069344627	0.08818	0.4316406040	-0.242179899
Inverse v	109	0.0648747329	0.08255	0.4319625694	-0.096932819
Inverse v	109	0.0641416264	0.08172	0.4325443455	-0.096039758
Inverse v	109	0.0663009836	0.08459	0.4331759523	-0.09950043
Inverse v	109	0.0666728740	0.08524	0.4341453108	-0.100410426
Inverse v	109	0.0688846263	0.08809	0.4342568884	-0.103783276
Inverse v	109	0.0768990917	0.09834	0.4342509176	-0.115855563
Inverse v	109	0.0649975738	0.08316	0.4344595259	-0.097998809
Inverse v	109	0.0665457522	0.08527	0.4351821433	-0.100595779

Inverse v	109	0.0675281172	0.08653	0.4351643200	-0.102074217
Inverse v	109	0.0671789838	0.08616	0.4355951340	-0.10170487
Inverse v	109	0.0643637604	0.08290	0.4375169375	-0.098122197
Inverse v	109	0.0652806046	0.08414	0.4378594235	-0.099643146
Inverse v	109	0.0640171654	0.08260	0.4383706788	-0.097895278
Inverse v	109	0.0662024068	0.08555	0.4390694953	-0.101492744
Inverse v	109	0.0640067064	0.08271	0.4390349108	-0.098114336
Inverse v	109	0.0634964549	0.08220	0.4398572254	-0.097621515
Inverse v	109	0.0633236366	0.08204	0.4402308885	-0.097487174
Inverse v	109	0.0720309803	0.09343	0.4407598839	-0.111104015
Inverse v	109	0.0676786242	0.08777	0.4406720505	-0.104357665
Inverse v	109	-0.07378169	0.09568	0.4406751451	-0.261332992
Inverse v	109	0.0690549476	0.08967	0.4412631964	-0.106707162
Inverse v	109	0.0643582081	0.08362	0.4415290994	-0.099544922
Inverse v	109	0.0786004989	0.10240	0.4427526301	-0.122111273
Inverse v	109	0.0667199677	0.08691	0.4426889233	-0.10363026
Inverse v	109	0.0623808280	0.08126	0.4426866311	-0.096889855
Inverse v	109	0.0649842689	0.08468	0.4428556157	-0.100994994
Inverse v	109	-0.069586092	0.09071	0.4430209785	-0.247383531
Inverse v	109	-0.095512886	0.12451	0.4430171262	-0.339553078
Inverse v	109	0.0681074376	0.08893	0.4437792233	-0.106201605
Inverse v	109	0.0682010927	0.08904	0.4437277495	-0.10632793
Inverse v	109	0.0664063529	0.08669	0.4436975062	-0.103518591
Inverse v	109	0.0716075342	0.09354	0.4439822012	-0.111741041
Inverse v	109	0.0669446566	0.08781	0.4458803343	-0.10518094
Inverse v	109	0.0672569833	0.08822	0.4458567665	-0.105662697
Inverse v	109	0.0659295697	0.08654	0.4461830051	-0.103698924
Inverse v	109	0.0641251815	0.08432	0.4470080946	-0.10116059
Inverse v	109	0.0628404226	0.08292	0.4485957774	-0.099701185
Inverse v	109	0.0728189516	0.09614	0.4487978750	-0.115616815
Inverse v	109	-0.06433174	0.08505	0.4494629405	-0.231049217
Inverse v	109	0.0631599897	0.08372	0.4506418901	-0.100947316
Inverse v	109	0.0739307527	0.09820	0.4515762161	-0.118558929
Inverse v	109	0.0615730029	0.08176	0.4514135711	-0.098683828
Inverse v	109	0.0658354514	0.08753	0.4520039658	-0.105739081
Inverse v	109	0.0653223692	0.08696	0.4525744979	-0.105129969
Inverse v	109	0.0646085757	0.08645	0.4548907225	-0.104848295
Inverse v	109	0.0664859880	0.08893	0.4547327709	-0.107833944

Inverse v	109	-0.063797474	0.08548	0.455475289	-0.231344064
Inverse v	109	0.067288912	0.09023	0.455841674	-0.109570715
Inverse v	109	0.062073753	0.08333	0.456356181	-0.101265006
Inverse v	109	0.064826124	0.08699	0.456172477	-0.105685567
Inverse v	109	0.063294635	0.08504	0.456713914	-0.103389092
Inverse v	109	0.062524393	0.08413	0.457409347	-0.102385576
Inverse v	109	0.059952616	0.08081	0.458157266	-0.098437422
Inverse v	109	0.063659674	0.08582	0.458264711	-0.104564322
Inverse v	109	0.060841507	0.08214	0.458911563	-0.100166953
Inverse v	109	0.062446119	0.08429	0.458791886	-0.102764699
Inverse v	109	0.068137120	0.09211	0.459504598	-0.112416435
Inverse v	109	0.061675379	0.08361	0.460772434	-0.102217998
Inverse v	109	-0.067172699	0.09124	0.461642833	-0.246021431
Inverse v	109	0.073177904	0.09937	0.461488929	-0.12159289
Inverse v	109	0.062846099	0.08530	0.461309794	-0.104358633
Inverse v	109	0.061887975	0.08424	0.462583265	-0.103236537
Inverse v	109	0.067744456	0.09226	0.462804269	-0.113095142
Inverse v	109	0.061117368	0.08330	0.463150507	-0.102157958
Inverse v	109	0.066164284	0.09034	0.463950788	-0.110910591
Inverse v	109	0.061861635	0.08443	0.463794093	-0.103640063
Inverse v	109	0.060732843	0.08290	0.463818655	-0.101757867
Inverse v	109	0.063139914	0.08632	0.464511024	-0.106052902
Inverse v	109	0.064925220	0.08891	0.465272125	-0.109348437
Inverse v	109	0.064013262	0.08778	0.465882916	-0.108047905
Inverse v	109	0.063286886	0.08679	0.465888545	-0.106824003
Inverse v	109	0.073389011	0.10062	0.465788626	-0.123831522
Inverse v	109	0.065056204	0.08943	0.466977629	-0.110238282
Inverse v	109	0.062577254	0.08607	0.467243819	-0.106138479
Inverse v	109	-0.063454921	0.08732	0.467419409	-0.234604421
Inverse v	109	0.062399421	0.08630	0.469651601	-0.106750065
Inverse v	109	-0.060393354	0.08350	0.469537433	-0.224062817
Inverse v	109	0.063878180	0.08838	0.469845642	-0.109355524
Inverse v	109	0.077471732	0.10701	0.469125956	-0.132286791
Inverse v	109	0.059678848	0.08253	0.469638605	-0.102091101
Inverse v	109	0.060340617	0.08342	0.469487540	-0.103167562
Inverse v	109	0.071651089	0.09891	0.468842306	-0.122224006
Inverse v	109	0.060588043	0.08368	0.469089061	-0.103443446
Inverse v	109	0.061848237	0.08570	0.470504392	-0.106129451

Inverse \	109	0.0659363452	0.09145	0.470951961	-0.113325241
Inverse \	109	0.0636927101	0.08853	0.471888868	-0.109835405
Inverse \	109	0.0645160560	0.08971	0.472068187	-0.111326381
Inverse \	109	0.0610059282	0.08487	0.472288786	-0.105352263
Inverse \	109	0.0544163046	0.07586	0.473218962	-0.094284626
Inverse \	109	0.0622248824	0.08672	0.473089778	-0.107764557
Inverse \	109	0.0650179254	0.09085	0.474202838	-0.113049276
Inverse \	109	-0.06224744	0.08704	0.474548531	-0.23286041
Inverse \	109	0.0588664578	0.08241	0.475087135	-0.102676495
Inverse \	109	0.0669320772	0.09369	0.475022032	-0.116717693
Inverse \	109	0.0840382200	0.11785	0.475800488	-0.146954785
Inverse \	109	0.0506635329	0.07110	0.476168513	-0.088709828
Inverse \	109	0.0601557120	0.08445	0.476303814	-0.105381034
Inverse \	109	0.0668157105	0.09395	0.476979735	-0.117330193
Inverse \	109	0.0469768341	0.06613	0.477491825	-0.082643182
Inverse \	109	0.0610999799	0.08623	0.478609071	-0.107917596
Inverse \	109	0.0600917404	0.08486	0.478916966	-0.106253221
Inverse \	109	-0.063083206	0.08920	0.479436704	-0.237915696
Inverse \	109	0.0601485213	0.08509	0.479688717	-0.106646258
Inverse \	109	0.0648187111	0.09177	0.480009089	-0.115057889
Inverse \	109	0.0618812311	0.08821	0.482987100	-0.111014168
Inverse \	109	0.0632202714	0.09030	0.483872414	-0.113774264
Inverse \	109	0.0583510093	0.08338	0.484055932	-0.105079901
Inverse \	109	0.0601114360	0.08593	0.484245514	-0.108323168
Inverse \	109	0.0584232706	0.08370	0.485195476	-0.105637484
Inverse \	109	0.0585743243	0.08407	0.486017634	-0.106220859
Inverse \	109	0.0579720474	0.08328	0.486376374	-0.105262924
Inverse \	109	0.0597122063	0.08603	0.487650581	-0.1089152
Inverse \	109	0.0589097626	0.08484	0.487485979	-0.107388643
Inverse \	109	0.0624127657	0.08994	0.487762883	-0.113886513
Inverse \	109	0.0601348523	0.08678	0.488342010	-0.109956212
Inverse \	109	0.0575833069	0.08315	0.488651297	-0.105406626
Inverse \	109	0.0579793273	0.08385	0.489314992	-0.106382385
Inverse \	109	0.0626520496	0.09068	0.489632438	-0.11508592
Inverse \	109	0.0569253781	0.08243	0.489853368	-0.104648764
Inverse \	109	0.0594057391	0.08607	0.490092127	-0.109301315
Inverse \	109	0.0569406771	0.08264	0.490833100	-0.105042281
Inverse \	109	0.0575033386	0.08397	0.493506056	-0.107093495

Inverse v	109	0.05876970898	0.085924	0.4939947379	-0.109642352
Inverse v	109	0.06138789369	0.089790	0.4942073400	-0.114613532
Inverse v	109	0.05719066934	0.083700	0.4944292908	-0.10686147
Inverse v	109	0.05730506832	0.083940	0.4948348493	-0.107229772
Inverse v	109	0.05680085190	0.083490	0.4963288081	-0.106852481
Inverse v	109	0.05891223443	0.086688	0.4967672957	-0.110997323
Inverse v	109	0.05506009867	0.081109	0.4972412649	-0.103914475
Inverse v	109	0.07060523505	0.104280	0.4983619573	-0.133784849
Inverse v	109	0.06299016342	0.093100	0.4986709653	-0.119486834
Inverse v	109	0.05694686952	0.084230	0.4989864121	-0.108144494
Inverse v	109	0.05878081718	0.087178	0.5001448235	-0.112088102
Inverse v	109	0.05638586031	0.083641	0.5002257305	-0.107552147
Inverse v	109	0.05979519433	0.088863	0.5010163731	-0.114376415
Inverse v	109	0.05435238330	0.081115	0.5028175743	-0.104633648
Inverse v	109	0.06191503172	0.092591	0.5036953113	-0.119565249
Inverse v	109	0.06055388633	0.090552	0.5036760241	-0.116928693
Inverse v	109	0.06230261665	0.093212	0.5038825248	-0.120393882
Inverse v	109	0.06112150458	0.091407	0.5037051884	-0.118037002
Inverse v	109	0.05778179260	0.086321	0.5032542031	-0.11140851
Inverse v	109	0.05808273304	0.087080	0.5047990891	-0.112605962
Inverse v	109	0.05595189031	0.083948	0.5050885889	-0.108586653
Inverse v	109	0.06047084532	0.090969	0.5062190577	-0.11782967
Inverse v	109	0.05732684158	0.086573	0.5078604754	-0.11235788
Inverse v	109	0.06463441740	0.097890	0.5090773312	-0.127230255
Inverse v	109	0.05500730171	0.083500	0.5100744102	-0.108664685
Inverse v	109	0.05649599851	0.086097	0.5117047517	-0.112255293
Inverse v	109	0.05548893889	0.084611	0.5119489107	-0.11035021
Inverse v	109	0.05622070377	0.085872	0.5126617325	-0.112089653
Inverse v	109	0.05849652693	0.089309	0.5124740192	-0.116549116
Inverse v	109	0.05779070375	0.088509	0.5137995407	-0.115687735
Inverse v	109	0.05291807070	0.081092	0.5140397338	-0.106024184
Inverse v	109	0.05483256647	0.084042	0.5141169592	-0.109890207
Inverse v	109	0.06426764345	0.098627	0.5146469744	-0.129042523
Inverse v	109	0.05433319711	0.083565	0.5155738015	-0.109456091
Inverse v	109	-0.053304161	0.081948	0.5153976489	-0.213924
Inverse v	109	0.05724442922	0.088272	0.5166629708	-0.115769364
Inverse v	109	0.05475183707	0.084461	0.5168565427	-0.110804845
Inverse v	109	0.05513492272	0.085111	0.5171447894	-0.111694821

Inverse v	109	0.0544345984	0.08416	0.517796113	-0.110532411
Inverse v	109	0.0546139778	0.08457	0.518451087	-0.111155915
Inverse v	109	0.0556007927	0.08633	0.519568163	-0.113616065
Inverse v	109	0.0610481312	0.09536	0.522053942	-0.125858466
Inverse v	109	0.0563636205	0.08819	0.522788888	-0.116505941
Inverse v	109	0.0553868112	0.08690	0.523913140	-0.114947229
Inverse v	109	0.0539607310	0.08475	0.524330551	-0.112154635
Inverse v	109	0.0536048231	0.08439	0.525327695	-0.111812353
Inverse v	109	0.0536257453	0.08435	0.524966592	-0.111711828
Inverse v	109	0.0542952196	0.08546	0.525239136	-0.11321661
Inverse v	109	0.0605005451	0.09515	0.524880228	-0.125994375
Inverse v	109	0.0516209007	0.08148	0.526428339	-0.108098345
Inverse v	109	0.0545589172	0.08613	0.526468215	-0.114267068
Inverse v	109	0.0720876010	0.11399	0.527153760	-0.151349072
Inverse v	109	-0.053415851	0.08455	0.527559754	-0.219141975
Inverse v	109	0.0524219655	0.08294	0.527403288	-0.110158945
Inverse v	109	0.0568208549	0.09002	0.527929443	-0.119627478
Inverse v	109	0.0520414953	0.08255	0.528446803	-0.109768098
Inverse v	109	0.0519509894	0.08258	0.529307764	-0.109915036
Inverse v	109	0.0538633526	0.08569	0.529637243	-0.114095465
Inverse v	109	0.0507848581	0.08096	0.530475161	-0.107897371
Inverse v	109	0.0552293760	0.08800	0.530278623	-0.117257714
Inverse v	109	0.0545443856	0.08708	0.531106145	-0.116146756
Inverse v	109	0.0536558124	0.08567	0.531117625	-0.114259322
Inverse v	109	0.0511686653	0.08175	0.531400897	-0.10907348
Inverse v	109	0.0546185393	0.08761	0.533044448	-0.117114351
Inverse v	109	0.0550934380	0.08845	0.533393135	-0.118280226
Inverse v	109	0.0529996246	0.08536	0.534698395	-0.114318219
Inverse v	109	0.0536715339	0.08654	0.535159273	-0.115958826
Inverse v	109	0.0498917535	0.08053	0.535580903	-0.107955495
Inverse v	109	0.0561419804	0.09081	0.536427303	-0.121848807
Inverse v	109	0.0515828142	0.08346	0.536566437	-0.112009592
Inverse v	109	0.0522950398	0.08459	0.536473863	-0.113518474
Inverse v	109	0.0532393711	0.08629	0.537248206	-0.115889679
Inverse v	109	0.0576575422	0.09344	0.537207427	-0.125488654
Inverse v	109	0.0536723923	0.08694	0.537038416	-0.116744406
Inverse v	109	0.0511592325	0.08330	0.539148469	-0.112123463
Inverse v	109	0.0550562608	0.08965	0.539164709	-0.120671443

Inverse v	109	-0.051491126	0.083941	0.5396297829	-0.216028232
Inverse v	109	0.0498844490?	0.081295	0.5394679422	-0.109455006
Inverse v	109	0.0518861594	0.08479	0.5406010707	-0.114311187
Inverse v	109	0.0504229668(	0.08241(	0.5406641701	-0.11111278
Inverse v	109	0.0503449272?	0.08253	0.5418721567	-0.111423244
Inverse v	109	-0.06400198	0.10510?	0.5425640565	-0.270005582
Inverse v	109	0.0511428318?	0.084239	0.5437772957	-0.113966945
Inverse v	109	0.0507220800(	0.083669	0.5443710667	-0.113270972
Inverse v	109	0.0520777840?	0.08604?	0.5450356998	-0.116576896
Inverse v	109	-0.049255312	0.08137?	0.5449815363	-0.20874789
Inverse v	109	0.0532651841?	0.08791?	0.5446171089	-0.119055478
Inverse v	109	-0.047470138	0.078575	0.5457566987	-0.20147896
Inverse v	109	-0.050590075	0.08383?	0.5462254316	-0.214912747
Inverse v	109	0.0510671150?	0.08492?	0.5476373527	-0.115390288
Inverse v	109	0.0521303665?	0.08682(	0.5482428996	-0.118050078
Inverse v	109	0.0562119569?	0.09362?	0.5482371319	-0.127290261
Inverse v	109	0.0513508364?	0.085779	0.5494142332	-0.116776847
Inverse v	109	0.0506700190(	0.08486(	0.5504403822	-0.115655854
Inverse v	109	0.0487979505?	0.081969	0.5516330544	-0.111863233
Inverse v	109	0.0522659163?	0.08825(	0.553684879(	-0.120704336
Inverse v	109	0.0505669120?	0.085659	0.5549709207	-0.117324811
Inverse v	109	0.0497160974?	0.084199	0.5548864545	-0.115315531
Inverse v	109	0.0498954319(	0.08469?	0.5557659966	-0.116100271
Inverse v	109	-0.047897166	0.081619	0.5573168303	-0.207872275
Inverse v	109	0.0506585052?	0.08631?	0.557281739(	-0.118524334
Inverse v	109	0.0503779501?	0.085819	0.5571887025	-0.117828224
Inverse v	109	0.0503477701?	0.08579(	0.5572931937	-0.117802202
Inverse v	109	-0.054769028	0.09313?	0.5564809492	-0.237308708
Inverse v	109	0.0512548753?	0.08747(	0.557898415(	-0.120187783
Inverse v	109	0.0504178941(	0.08609?	0.5581296436	-0.118324218
Inverse v	109	-0.05649699	0.096445	0.5580160274	-0.245530476
Inverse v	109	0.0474032777?	0.081295	0.5598270201	-0.111935892
Inverse v	109	0.0484375467	0.08305?	0.5597413804	-0.114342649
Inverse v	109	0.0475998178	0.08165?	0.5599466407	-0.112448766
Inverse v	109	0.0495505638?	0.08491(	0.5595135951	-0.116873483
Inverse v	109	0.0506912085(	0.08707?	0.560456524(	-0.119973494
Inverse v	109	0.0483029738(	0.08332?	0.5621307835	-0.11501802
Inverse v	109	-0.049403987	0.08551	0.5634467379	-0.217011509

Inverse v	109	0.05088154395	0.08833	0.5646090559	-0.122254319
Inverse v	109	0.04758370913	0.08290	0.5659848578	-0.114904384
Inverse v	109	0.0471001991	0.08261	0.5686052061	-0.114828016
Inverse v	109	0.0550477991	0.09654	0.5685769832	-0.134190069
Inverse v	109	0.0464970216	0.08167	0.5691717264	-0.113592005
Inverse v	109	0.0492042762	0.08651	0.5695129647	-0.120355587
Inverse v	109	0.0511808148	0.09005	0.5698177333	-0.125329612
Inverse v	109	-0.048106169	0.08473	0.5702067385	-0.21418029
Inverse v	109	-0.051003828	0.08983	0.5702014805	-0.227078966
Inverse v	109	0.0477233606	0.08422	0.5709516598	-0.117348032
Inverse v	109	0.0524495967	0.09271	0.5716128844	-0.12928146
Inverse v	109	0.0478420641	0.08466	0.5720397750	-0.118108649
Inverse v	109	0.0461639062	0.08184	0.5727221510	-0.114250487
Inverse v	109	0.0494027837	0.08759	0.5727525302	-0.122279933
Inverse v	109	0.0510273681	0.09083	0.5742792624	-0.127008745
Inverse v	109	0.0466425590	0.08308	0.5745471416	-0.116208766
Inverse v	109	-0.068383513	0.12197	0.5750384052	-0.307449728
Inverse v	109	0.0467303651	0.08350	0.5757228916	-0.116930214
Inverse v	109	0.0472593407	0.08446	0.5758266799	-0.118298836
Inverse v	109	0.0479475334	0.08561	0.5754528153	-0.119857138
Inverse v	109	0.0486651689	0.08764	0.5787117296	-0.123114626
Inverse v	109	0.0497882047	0.08970	0.5788812326	-0.126034191
Inverse v	109	0.0460817032	0.08314	0.5794313292	-0.116887626
Inverse v	109	-0.047448189	0.08556	0.5792080005	-0.215151369
Inverse v	109	-0.045519203	0.08226	0.5800231792	-0.206750791
Inverse v	109	-0.060857916	0.11035	0.5813224102	-0.277161525
Inverse v	109	0.0464076260	0.08422	0.5816343171	-0.118672414
Inverse v	109	0.0477987963	0.08709	0.5831353923	-0.122907874
Inverse v	109	0.0459237060	0.08394	0.5843226415	-0.118604687
Inverse v	109	0.0532297557	0.09770	0.5858737048	-0.138263424
Inverse v	109	0.0462454181	0.08505	0.5866593051	-0.120471106
Inverse v	109	0.0485813975	0.08934	0.5866059210	-0.12653144
Inverse v	109	0.0517519860	0.09524	0.5868652738	-0.13491867
Inverse v	109	0.0458698119	0.08427	0.5862494750	-0.119311697
Inverse v	109	0.0453305276	0.08359	0.5876410431	-0.118517485
Inverse v	109	0.0460901431	0.08501	0.5877302738	-0.120543321
Inverse v	109	-0.04783866	0.08845	0.5886397251	-0.22121576
Inverse v	109	0.0454743250	0.08415	0.5889651801	-0.119477927

Inverse v	109	0.0453835979	0.08403	0.5891722428	-0.119331089
Inverse v	109	0.0453366422	0.08410	0.5898580429	-0.119511104
Inverse v	109	0.0455603697	0.08479	0.5910649967	-0.120639946
Inverse v	109	-0.050101262	0.09322	0.5909578755	-0.232813618
Inverse v	109	0.0515647517	0.09624	0.5921073928	-0.137068711
Inverse v	109	0.0472262409	0.08836	0.5930502238	-0.125976936
Inverse v	109	0.0451950897	0.08477	0.5939527680	-0.120964343
Inverse v	109	0.0440325867	0.08262	0.5940720488	-0.117905254
Inverse v	109	0.0478611631	0.08994	0.5946494777	-0.128432826
Inverse v	109	0.0442398869	0.08325	0.5951747772	-0.118947896
Inverse v	109	0.0478074893	0.09016	0.5959441786	-0.128909341
Inverse v	109	0.0536707903	0.10138	0.5965526995	-0.145048187
Inverse v	109	0.0483222462	0.09161	0.5978701630	-0.131237459
Inverse v	109	0.0440115942	0.08367	0.5989117003	-0.119996524
Inverse v	109	-0.046611227	0.08875	0.5994614418	-0.220568443
Inverse v	109	0.0469807978	0.09022	0.6025516113	-0.129850835
Inverse v	109	0.0483811039	0.09291	0.6025885271	-0.133739689
Inverse v	109	0.0427325028	0.08211	0.6027837444	-0.118211885
Inverse v	109	0.0475260673	0.09233	0.6067667999	-0.133457342
Inverse v	109	0.0454274196	0.08858	0.6080894858	-0.12820228
Inverse v	109	-0.045296949	0.08835	0.6081811502	-0.21847222
Inverse v	109	0.0431983370	0.08435	0.6085838908	-0.122139327
Inverse v	109	0.0429424106	0.08394	0.6089618621	-0.12158922
Inverse v	109	0.0463041530	0.09068	0.6096298004	-0.131439268
Inverse v	109	0.0428886072	0.08427	0.6108187709	-0.122292831
Inverse v	109	0.0502444710	0.09899	0.6117740997	-0.143786825
Inverse v	109	0.0434574135	0.08567	0.6119997730	-0.124470439
Inverse v	109	0.0468241037	0.09243	0.6124520922	-0.134343532
Inverse v	109	0.0423876087	0.08382	0.6131049316	-0.121916379
Inverse v	109	0.0453342798	0.09017	0.6151677035	-0.131417862
Inverse v	109	0.0565427057	0.11266	0.6157521123	-0.16427454
Inverse v	109	-0.048129573	0.09634	0.6173833173	-0.236962808
Inverse v	109	0.0420762497	0.08431	0.6177735795	-0.123190459
Inverse v	109	0.0421706842	0.08430	0.6169075379	-0.123059764
Inverse v	109	0.0418446162	0.08366	0.6169704001	-0.122137522
Inverse v	109	0.0418245240	0.08380	0.6177244869	-0.12243052
Inverse v	109	0.0435490063	0.08727	0.6177859352	-0.127508395
Inverse v	109	0.0438142464	0.08766	0.6172051500	-0.128001075

Inverse v	109	0.0414759567	0.08324	0.618302368	-0.121678298
Inverse v	109	0.0408943746	0.08204	0.618176786	-0.119914593
Inverse v	109	0.0439885225	0.08864	0.619727240	-0.129754088
Inverse v	109	0.0415842940	0.08409	0.620960014	-0.123242458
Inverse v	109	0.0456344570	0.09302	0.623742164	-0.136697349
Inverse v	109	0.0466367430	0.09522	0.624321284	-0.140011092
Inverse v	109	0.0436020937	0.08937	0.625651680	-0.131572678
Inverse v	109	-0.041673235	0.08538	0.625499274	-0.209024862
Inverse v	109	0.0425185162	0.08713	0.625585766	-0.128270324
Inverse v	109	0.0435043533	0.08919	0.625718155	-0.131311366
Inverse v	109	-0.04454577	0.09180	0.627501972	-0.224474587
Inverse v	109	0.0395842705	0.08160	0.627616603	-0.120357378
Inverse v	109	0.0433577160	0.08919	0.626900269	-0.131466621
Inverse v	109	0.0446658983	0.09182	0.626675383	-0.135315757
Inverse v	109	0.0422442601	0.08697	0.627157489	-0.128217684
Inverse v	109	0.0444469379	0.09147	0.627043823	-0.134843973
Inverse v	109	0.0415711637	0.08596	0.628674134	-0.126916255
Inverse v	109	0.0429494290	0.08875	0.628445598	-0.13100826
Inverse v	109	0.0405114264	0.08392	0.629307373	-0.123984273
Inverse v	109	0.0425922758	0.08861	0.630786667	-0.131101864
Inverse v	109	-0.043748914	0.09105	0.630899557	-0.22221887
Inverse v	109	0.0425645154	0.08873	0.631457507	-0.13135784
Inverse v	109	0.0404396663	0.08473	0.633201527	-0.125648985
Inverse v	109	0.0393344092	0.08265	0.634140979	-0.122662621
Inverse v	109	0.0437206206	0.09186	0.634145984	-0.136343485
Inverse v	109	0.0432064477	0.09114	0.635457977	-0.135430965
Inverse v	109	0.0400830879	0.08477	0.636323868	-0.126066234
Inverse v	109	0.0394355676	0.08334	0.636099963	-0.12392128
Inverse v	109	0.0404121310	0.08538	0.636001870	-0.126941371
Inverse v	109	0.0418242577	0.08907	0.638669331	-0.132755192
Inverse v	109	0.0387487193	0.08249	0.638547029	-0.122934132
Inverse v	109	0.0512715234	0.10930	0.639029498	-0.162971878
Inverse v	109	-0.041364811	0.08826	0.639330500	-0.21436729
Inverse v	109	-0.040676375	0.08765	0.642611002	-0.212480004
Inverse v	109	0.0430549091	0.09295	0.643219997	-0.139128517
Inverse v	109	-0.038518684	0.08322	0.643478391	-0.201634379
Inverse v	109	-0.040853646	0.08813	0.642977911	-0.213596629
Inverse v	109	-0.039934152	0.08633	0.643692481	-0.20915314

Inverse v	109	-0.038765951	0.08417	0.64512860	-0.203748905
Inverse v	109	0.04137423598	0.09013	0.64621475	-0.135289655
Inverse v	109	0.04682505348	0.10194	0.64600839	-0.152988195
Inverse v	109	0.04470037515	0.09768	0.64724171	-0.146762114
Inverse v	109	0.04001982338	0.08755	0.64761247	-0.131588207
Inverse v	109	0.04172790602	0.09148	0.64830391	-0.137581818
Inverse v	109	0.04414147025	0.09713	0.64951123	-0.146240177
Inverse v	109	-0.039294586	0.08680	0.65077412	-0.209428438
Inverse v	109	0.04312541665	0.09534	0.65103448	-0.143744017
Inverse v	109	0.03985645068	0.08839	0.65206867	-0.133397794
Inverse v	109	0.03789222357	0.08411	0.65236356	-0.126973195
Inverse v	109	0.04072206729	0.09049	0.65273110	-0.136656456
Inverse v	109	-0.039152102	0.08676	0.65181120	-0.209209835
Inverse v	109	0.03841363073	0.08534	0.65265770	-0.128871884
Inverse v	109	0.03904922700	0.08699	0.65353872	-0.131467017
Inverse v	109	0.03918246129	0.08726	0.65341164	-0.131848453
Inverse v	109	0.03645097070	0.08134	0.65409424	-0.122992957
Inverse v	109	0.03662916938	0.08236	0.65651552	-0.124802067
Inverse v	109	-0.041148388	0.09246	0.65631962	-0.22238616
Inverse v	109	0.03757595546	0.08435	0.65599436	-0.127759821
Inverse v	109	0.04050314218	0.09103	0.65638499	-0.137928918
Inverse v	109	0.04146637507	0.09355	0.65760980	-0.141907357
Inverse v	109	0.04157607279	0.09379	0.65757167	-0.142260897
Inverse v	109	0.04282077740	0.09669	0.65787657	-0.146700136
Inverse v	109	0.04006673937	0.09094	0.65954302	-0.138191909
Inverse v	109	-0.036248131	0.08243	0.66015549	-0.197827798
Inverse v	109	0.03015065775	0.06856	0.66014607	-0.104244942
Inverse v	109	0.03835899157	0.08751	0.66116242	-0.133172174
Inverse v	109	0.03626986188	0.08279	0.66134907	-0.126014609
Inverse v	109	0.03856990945	0.08802	0.66125771	-0.133956171
Inverse v	109	0.03888955045	0.08965	0.66443900	-0.13682462
Inverse v	109	-0.046447837	0.10704	0.66434385	-0.256249151
Inverse v	109	0.03659462951	0.08439	0.66458414	-0.128826634
Inverse v	109	0.03717497782	0.08569	0.66443732	-0.130791363
Inverse v	109	0.03868566110	0.08937	0.66513830	-0.136496055
Inverse v	109	-0.029798672	0.06909	0.66625104	-0.165216123
Inverse v	109	0.03624897225	0.08421	0.66687405	-0.128809187
Inverse v	109	0.03614043925	0.08478	0.66993126	-0.130044978

Inverse v	109	0.03808771271	0.089285	0.66968190022	-0.136911368
Inverse v	109	-0.035211503	0.082723	0.67036121115	-0.19734981
Inverse v	109	-0.034922171	0.082288	0.67128267771	-0.196207156
Inverse v	109	0.0367498767	0.086575	0.67121189533	-0.132937394
Inverse v	109	0.0346040030	0.082070	0.67331082822	-0.126265268
Inverse v	109	0.0317500779	0.075301	0.67328983344	-0.115841618
Inverse v	109	0.0417584698	0.099340	0.67424332311	-0.152960714
Inverse v	109	0.0366013214	0.087239	0.67481573500	-0.134388893
Inverse v	109	0.0349953596	0.083362	0.67463317333	-0.12839498
Inverse v	109	0.0338428426	0.081108	0.67649090377	-0.125128902
Inverse v	109	0.0256477608	0.061898	0.67861592955	-0.095673106
Inverse v	109	0.0350562316	0.084743	0.67911278055	-0.131041211
Inverse v	109	0.0360112789	0.087503	0.68067464855	-0.135494884
Inverse v	109	0.0372977041	0.090839	0.68137427522	-0.140747942
Inverse v	109	0.0336146484	0.082024	0.68194324933	-0.127153158
Inverse v	109	0.0357031699	0.087288	0.68252310355	-0.135383268
Inverse v	109	0.0342619238	0.084343	0.68458146233	-0.131051167
Inverse v	109	-0.033787214	0.083501	0.68575124611	-0.197451125
Inverse v	109	0.0329186428	0.081324	0.68563963244	-0.126478133
Inverse v	109	0.0373690768	0.092711	0.68689769488	-0.144345338
Inverse v	109	0.0332433276	0.082520	0.68708068955	-0.12850865
Inverse v	109	0.0345212650	0.086115	0.68851339488	-0.134264425
Inverse v	109	0.0337763794	0.084874	0.69066144677	-0.132577285
Inverse v	109	0.0355523246	0.089317	0.69059909944	-0.139510925
Inverse v	109	0.0342763754	0.086180	0.69083235455	-0.13463825
Inverse v	109	0.0337624725	0.085265	0.69212667533	-0.133357098
Inverse v	109	0.0338341270	0.085613	0.69269475011	-0.133966393
Inverse v	109	0.0345371418	0.087324	0.69246995660	-0.13661805
Inverse v	109	0.0344080515	0.087049	0.69264242200	-0.136208236
Inverse v	109	0.0337467825	0.085570	0.69332679177	-0.133984
Inverse v	109	-0.033744521	0.086010	0.69481471388	-0.202325389
Inverse v	109	0.0340895831	0.086923	0.69492482199	-0.136279867
Inverse v	109	-0.03358942	0.086270	0.69701578077	-0.202678737
Inverse v	109	0.0344998553	0.088709	0.69734557544	-0.1393717
Inverse v	109	0.0370594276	0.095998	0.69946457155	-0.151096851
Inverse v	109	0.0320926163	0.083828	0.70184024999	-0.132210846
Inverse v	109	0.0354371360	0.093323	0.70415002660	-0.147476556
Inverse v	109	0.0320078557	0.083927	0.70292560577	-0.132490419

Inverse v	109	0.03119853202	0.08215	0.70411985311	-0.129819782
Inverse v	109	0.03176888649	0.08352	0.70369338777	-0.131945403
Inverse v	109	-0.032457477	0.08526	0.70345445833	-0.199578817
Inverse v	109	-0.032261921	0.08468	0.70324396444	-0.198252664
Inverse v	109	0.03282680123	0.08696	0.70580918222	-0.137616015
Inverse v	109	0.04076121343	0.10847	0.70709871733	-0.171855743
Inverse v	109	-0.03067129	0.08174	0.70749401888	-0.190884277
Inverse v	109	0.03197001620	0.08517	0.70741438533	-0.134979284
Inverse v	109	-0.032806765	0.08722	0.70682509955	-0.20376461
Inverse v	109	0.03128395079	0.08369	0.70855359411	-0.132752026
Inverse v	109	0.03291974490	0.08799	0.70832782155	-0.139553428
Inverse v	109	0.03194132962	0.08568	0.70932252233	-0.136005818
Inverse v	109	0.03168162033	0.08493	0.70912898333	-0.134783857
Inverse v	109	0.03350346691	0.09028	0.71058018655	-0.143459256
Inverse v	109	0.03406924974	0.09223	0.71185780077	-0.146717353
Inverse v	109	0.03050127760	0.08362	0.71530242122	-0.133401307
Inverse v	109	0.03133511203	0.08598	0.71554398133	-0.137197692
Inverse v	109	0.03077367767	0.08452	0.71580901311	-0.134900876
Inverse v	109	-0.025649924	0.07072	0.71684446677	-0.16426785
Inverse v	109	0.03093378691	0.08551	0.71755214177	-0.136676997
Inverse v	109	0.03128447203	0.08671	0.71825439966	-0.138667859
Inverse v	109	0.03048481190	0.08447	0.71818051988	-0.135078031
Inverse v	109	0.03241816845	0.08970	0.71781100388	-0.143403894
Inverse v	109	-0.029080257	0.08051	0.71796741655	-0.186890312
Inverse v	109	0.03003454668	0.08347	0.71900106111	-0.13358042
Inverse v	109	0.03728010665	0.10377	0.71942471311	-0.166125692
Inverse v	109	0.03143421971	0.08795	0.72080357899	-0.140959878
Inverse v	109	-0.029624423	0.08287	0.72073914666	-0.192053967
Inverse v	109	0.03179277525	0.08915	0.72139742599	-0.142955611
Inverse v	109	-0.027523948	0.07787	0.72376636733	-0.180162355
Inverse v	109	0.03031461168	0.08578	0.72381074855	-0.137828032
Inverse v	109	0.02911433537	0.08222	0.72328698366	-0.132052151
Inverse v	109	-0.031304352	0.08873	0.72425822777	-0.205230468
Inverse v	109	0.02948133127	0.08422	0.72632783988	-0.1356073
Inverse v	109	0.03397801149	0.09706	0.72629323277	-0.156265923
Inverse v	109	0.03186807408	0.09135	0.72721882266	-0.147193
Inverse v	109	0.03045043642	0.08855	0.73096440766	-0.143125487
Inverse v	109	-0.030106224	0.08760	0.73109459333	-0.201806717

Inverse v	109	0.02992706522	0.087403	0.7320492274	-0.141383967
Inverse v	109	0.02878506752	0.084798	0.7342697533	-0.137420591
Inverse v	109	0.03076088878	0.090554	0.7340867971	-0.146726199
Inverse v	109	0.02862834340	0.084610	0.7350967665	-0.137208697
Inverse v	109	0.03092851373	0.091378	0.7350120448	-0.148173337
Inverse v	109	0.02615863257	0.077404	0.7354014848	-0.12555329
Inverse v	109	0.02890251186	0.085945	0.7366540185	-0.139551298
Inverse v	109	-0.027780236	0.082835	0.7373493479	-0.190138183
Inverse v	109	0.02858480229	0.086293	0.7404563159	-0.14055138
Inverse v	109	0.03227209409	0.098215	0.7424690045	-0.160229715
Inverse v	109	-0.028042155	0.085440	0.7427550756	-0.19550539
Inverse v	109	0.02787045422	0.085021	0.7430589079	-0.138771441
Inverse v	109	0.03054248107	0.093444	0.7437793321	-0.152608128
Inverse v	109	0.02904534274	0.089400	0.7452651454	-0.146180308
Inverse v	109	-0.028296777	0.088361	0.7487864711	-0.201484694
Inverse v	109	0.02676265338	0.084265	0.7507893460	-0.138397964
Inverse v	109	0.02673221731	0.084370	0.7513796631	-0.1386457
Inverse v	109	0.02678157557	0.084931	0.7525089974	-0.139683477
Inverse v	109	-0.028111658	0.089315	0.7529540944	-0.203169494
Inverse v	109	0.02639445060	0.084012	0.7533906085	-0.138270659
Inverse v	109	0.02723173882	0.086995	0.7542624377	-0.14327982
Inverse v	109	-0.026441218	0.084655	0.7547836106	-0.192366515
Inverse v	109	-0.025455395	0.081943	0.7560686201	-0.186063725
Inverse v	109	-0.026840348	0.086661	0.7567782661	-0.196697185
Inverse v	109	-0.028658863	0.092511	0.7567235980	-0.209981926
Inverse v	109	0.02818655769	0.091324	0.7575935898	-0.150809132
Inverse v	109	0.02596738133	0.084417	0.7583811811	-0.139490561
Inverse v	109	0.02630646733	0.085540	0.7584380427	-0.141352774
Inverse v	109	0.02825746083	0.092181	0.7591920754	-0.152418073
Inverse v	109	0.03327544780	0.108777	0.7596777642	-0.179928317
Inverse v	109	-0.02535272	0.084459	0.7640441956	-0.190894305
Inverse v	109	0.02507044299	0.083738	0.7646431169	-0.139057293
Inverse v	109	0.02571948139	0.086103	0.7651644406	-0.143042436
Inverse v	109	-0.028909696	0.096988	0.7656482366	-0.219008095
Inverse v	109	0.02529722629	0.084908	0.7657538185	-0.141124279
Inverse v	109	0.02589730970	0.087315	0.7667768169	-0.145241688
Inverse v	109	-0.025421775	0.087293	0.7708821342	-0.196517405
Inverse v	109	-0.025066567	0.086283	0.7714227851	-0.194182049

Inverse v	109	-0.025498978	0.08794	0.77185336	-0.197865878
Inverse v	109	0.02496115634	0.08625	0.77227820	-0.144093818
Inverse v	109	0.0242607439	0.08421	0.77327865	-0.140796118
Inverse v	109	0.0243752423	0.08546	0.77548048	-0.143132445
Inverse v	109	0.0249037602	0.08762	0.77624827	-0.146839337
Inverse v	109	0.0247602978	0.08732	0.77675814	-0.146394097
Inverse v	109	0.0257404733	0.09102	0.77734557	-0.152671537
Inverse v	109	0.0235456583	0.08385	0.77887872	-0.140815764
Inverse v	109	0.0270204416	0.09634	0.77911915	-0.161807633
Inverse v	109	0.0236312440	0.08442	0.77955075	-0.141843885
Inverse v	109	-0.026980794	0.09642	0.77961338	-0.215965891
Inverse v	109	0.0246336916	0.08826	0.78016553	-0.148356139
Inverse v	109	0.0232587757	0.08336	0.78023417	-0.140128079
Inverse v	109	-0.023370391	0.08392	0.78065878	-0.187867568
Inverse v	109	-0.022874736	0.08227	0.78098328	-0.184127889
Inverse v	109	0.0240242136	0.08666	0.78163130	-0.145847763
Inverse v	109	0.0260190945	0.09400	0.78193707	-0.158223101
Inverse v	109	-0.023311202	0.08434	0.78225855	-0.188628737
Inverse v	109	0.0237367684	0.08603	0.78261603	-0.144882749
Inverse v	109	0.0231985426	0.08437	0.78335951	-0.142177671
Inverse v	109	0.0223473181	0.08132	0.78348795	-0.137057667
Inverse v	109	0.0233063878	0.08543	0.78500713	-0.144144343
Inverse v	109	0.0287368569	0.10528	0.78488839	-0.177613633
Inverse v	109	0.0279321804	0.10259	0.78541499	-0.173144781
Inverse v	109	-0.025060023	0.09214	0.78564939	-0.205663239
Inverse v	109	-0.02159387	0.07941	0.78569008	-0.177247411
Inverse v	109	0.0227366623	0.08379	0.78613494	-0.141503817
Inverse v	109	0.0220512146	0.08246	0.78916487	-0.139583609
Inverse v	109	-0.022121306	0.08341	0.79087032	-0.18562405
Inverse v	109	-0.022696335	0.08549	0.79063667	-0.190257564
Inverse v	109	0.0228248683	0.08612	0.79099519	-0.145981228
Inverse v	109	0.0225088229	0.08628	0.79420459	-0.146617136
Inverse v	109	0.0209314400	0.08075	0.79549113	-0.137354501
Inverse v	109	0.0208176240	0.08072	0.79650424	-0.137409113
Inverse v	109	0.0246226038	0.09689	0.79941341	-0.165299085
Inverse v	109	0.0223586834	0.08814	0.79975834	-0.15040424
Inverse v	109	0.0208660045	0.08256	0.80049111	-0.140968181
Inverse v	109	-0.021806513	0.08692	0.80191259	-0.192174908

Inverse v	109	-0.020605793	0.08220	0.80208164	-0.181733706
Inverse v	109	0.02118529491	0.08433	0.80164699	-0.144103348
Inverse v	109	-0.022472682	0.08940	0.80154504	-0.197713598
Inverse v	109	-0.022036779	0.08764	0.80147658	-0.193818256
Inverse v	109	-0.021233837	0.08491	0.80254895	-0.18767407
Inverse v	109	-0.020211205	0.08191	0.80512328	-0.180772259
Inverse v	109	0.0210429848	0.08615	0.80703980	-0.147820135
Inverse v	109	0.0212816335	0.08780	0.80848020	-0.150806718
Inverse v	109	0.0207026685	0.08552	0.80872973	-0.146926752
Inverse v	109	0.0204644873	0.08490	0.80952847	-0.145944954
Inverse v	109	0.0211031683	0.08803	0.81054771	-0.151441032
Inverse v	109	0.0210509481	0.08759	0.81008610	-0.15063982
Inverse v	109	-0.02031186	0.08471	0.81050761	-0.186350322
Inverse v	109	0.0203884269	0.08508	0.81063041	-0.1463861
Inverse v	109	0.0198701126	0.08350	0.81191876	-0.143799513
Inverse v	109	-0.02041049	0.08599	0.81238997	-0.188961494
Inverse v	109	0.0202574931	0.08579	0.81333996	-0.14789758
Inverse v	109	-0.020281684	0.08679	0.81523245	-0.190394191
Inverse v	109	0.0204503150	0.08776	0.81575704	-0.151573939
Inverse v	109	-0.020792677	0.08928	0.81585675	-0.195793256
Inverse v	109	-0.020234272	0.08744	0.81700541	-0.19162351
Inverse v	109	0.0194746900	0.08418	0.81706409	-0.145534578
Inverse v	109	0.0186198773	0.08115	0.81852322	-0.140437817
Inverse v	109	-0.01954112	0.08523	0.81866163	-0.186598068
Inverse v	109	-0.019095882	0.08315	0.81837822	-0.182087239
Inverse v	109	-0.019331721	0.08509	0.82028207	-0.186114924
Inverse v	109	-0.018249364	0.08127	0.82234593	-0.177555291
Inverse v	109	-0.020049261	0.08914	0.82205411	-0.194775305
Inverse v	109	-0.019154611	0.08560	0.82295483	-0.186947612
Inverse v	109	-0.018929665	0.08508	0.82394182	-0.185697379
Inverse v	109	0.0203800659	0.09179	0.82430913	-0.1595471
Inverse v	109	0.0181699553	0.08252	0.82572446	-0.143569352
Inverse v	109	-0.019821699	0.08998	0.82564738	-0.196184679
Inverse v	109	0.0187553511	0.08575	0.82687089	-0.149318337
Inverse v	109	0.0185887181	0.08502	0.82694382	-0.148063043
Inverse v	109	0.0188228965	0.08685	0.82843456	-0.151418088
Inverse v	109	-0.017570372	0.08125	0.82879254	-0.176820598
Inverse v	109	0.0184969891	0.08583	0.82937884	-0.149736825

Inverse v	109	0.0187195875	0.08711	0.829861323	-0.152029151
Inverse v	109	0.0193512067	0.09041	0.830532901	-0.157869112
Inverse v	109	0.0205797276	0.09643	0.831004253	-0.16842527
Inverse v	109	0.0184824788	0.08844	0.834463284	-0.154862017
Inverse v	109	0.0191202009	0.09095	0.833503653	-0.159156599
Inverse v	109	-0.017774144	0.08484	0.834068482	-0.184072805
Inverse v	109	0.0182950544	0.08783	0.834999085	-0.15385697
Inverse v	109	-0.019903084	0.09515	0.834327480	-0.206415971
Inverse v	109	-0.018845014	0.08998	0.834124292	-0.195223155
Inverse v	109	0.0178495760	0.08566	0.834946775	-0.150056597
Inverse v	109	0.0186398904	0.09015	0.836195421	-0.158056574
Inverse v	109	0.0168063650	0.08179	0.837197522	-0.143503921
Inverse v	109	0.0172636313	0.08404	0.837259981	-0.147472462
Inverse v	109	-0.020103683	0.09783	0.837192259	-0.211859693
Inverse v	109	-0.017146272	0.08380	0.837878387	-0.181395414
Inverse v	109	0.0173188295	0.08483	0.838235980	-0.148955193
Inverse v	109	-0.017471845	0.08643	0.839810531	-0.186886711
Inverse v	109	0.0185156737	0.09206	0.840612434	-0.161936272
Inverse v	109	0.0209380956	0.10407	0.840563130	-0.183058576
Inverse v	109	0.0190816208	0.09554	0.841707192	-0.168189568
Inverse v	109	0.0182718194	0.09180	0.842243542	-0.161669633
Inverse v	109	-0.016959131	0.08591	0.843519588	-0.185353176
Inverse v	109	0.0166033652	0.08504	0.845218179	-0.1500909
Inverse v	109	0.0169519243	0.08688	0.845302179	-0.153335396
Inverse v	109	0.0187707762	0.09641	0.845640003	-0.170205414
Inverse v	109	0.0328949385	0.17042	0.846944992	-0.301136766
Inverse v	109	0.0162581381	0.08457	0.847558432	-0.149507797
Inverse v	109	0.0173208348	0.09055	0.848309821	-0.160165835
Inverse v	109	-0.017003482	0.08917	0.848777021	-0.191783135
Inverse v	109	0.0161540268	0.08547	0.850096057	-0.15137288
Inverse v	109	-0.015723569	0.08485	0.852991176	-0.182035779
Inverse v	109	0.0152807746	0.08293	0.853812025	-0.147265862
Inverse v	109	0.0158809698	0.08699	0.855144618	-0.154621842
Inverse v	109	0.0156914918	0.08701	0.856895627	-0.1548613
Inverse v	109	0.0170731090	0.09529	0.857806848	-0.16969878
Inverse v	109	0.0151341940	0.08532	0.859215505	-0.152101179
Inverse v	109	0.0148226444	0.08347	0.859061547	-0.148789241
Inverse v	109	-0.015206496	0.08686	0.861033087	-0.185461619

Inverse v	109	0.014770219	0.08433	0.86096808	-0.150522142
Inverse v	109	0.014924928	0.08477	0.86024832	-0.151229685
Inverse v	109	0.014944401	0.08503	0.86050290	-0.151733783
Inverse v	109	0.015831243	0.09110	0.86204823	-0.162736375
Inverse v	109	0.014740010	0.08480	0.86201033	-0.151472978
Inverse v	109	0.015076783	0.08706	0.86251692	-0.155566542
Inverse v	109	0.016663880	0.09647	0.86286926	-0.172432133
Inverse v	109	0.014109302	0.08388	0.86642221	-0.150298862
Inverse v	109	0.014250889	0.08471	0.86641542	-0.151798605
Inverse v	109	-0.01418464	0.08541	0.86809777	-0.181590096
Inverse v	109	0.014013744	0.08448	0.86825879	-0.151578845
Inverse v	109	0.013870959	0.08337	0.86786229	-0.149538053
Inverse v	109	-0.01386524	0.08304	0.86740725	-0.176641093
Inverse v	109	-0.013505609	0.08270	0.87028479	-0.175609225
Inverse v	109	0.013647577	0.08430	0.87139131	-0.151581947
Inverse v	109	0.013713616	0.08552	0.87260295	-0.153908298
Inverse v	109	0.014099557	0.08783	0.87246914	-0.158057347
Inverse v	109	-0.013491571	0.08579	0.87504061	-0.181643827
Inverse v	109	-0.013750981	0.08728	0.87482349	-0.18483665
Inverse v	109	0.031342379	0.20284	0.87720383	-0.366231563
Inverse v	109	-0.01306358	0.08496	0.87780604	-0.17959656
Inverse v	109	0.012858506	0.08360	0.87776089	-0.150999187
Inverse v	109	-0.014567022	0.09481	0.87789022	-0.200394715
Inverse v	109	-0.013029667	0.08627	0.87995270	-0.182123794
Inverse v	109	0.013673938	0.09034	0.87969415	-0.163397025
Inverse v	109	-0.012458164	0.08232	0.87971918	-0.173819262
Inverse v	109	0.017202349	0.11447	0.88054665	-0.207161784
Inverse v	109	-0.013302291	0.08915	0.88138911	-0.188040942
Inverse v	109	0.012986172	0.08797	0.88265099	-0.159447891
Inverse v	109	0.012540781	0.08502	0.88273624	-0.154101198
Inverse v	109	-0.012547307	0.08518	0.88290448	-0.179517298
Inverse v	109	0.012289993	0.08475	0.88471098	-0.153836978
Inverse v	109	0.012761610	0.08833	0.88512992	-0.160373877
Inverse v	109	0.012004794	0.08295	0.88493336	-0.150582851
Inverse v	109	0.012509206	0.08577	0.88404573	-0.155603972
Inverse v	109	-0.012900677	0.08869	0.88435507	-0.186741959
Inverse v	109	0.013046664	0.09134	0.88642860	-0.165994199
Inverse v	109	-0.013753235	0.09612	0.88622446	-0.202149493

Inverse v	109	-0.011191456	0.07809	0.886053106	-0.164263506
Inverse v	109	0.0115328709	0.08034	0.885859006	-0.145938819
Inverse v	109	-0.012164298	0.08654	0.888227680	-0.181801452
Inverse v	109	0.0115606772	0.08235	0.888359259	-0.149849961
Inverse v	109	-0.013964622	0.10052	0.889514626	-0.210991501
Inverse v	109	0.0118787198	0.08688	0.891248001	-0.15840656
Inverse v	109	0.0114291989	0.08468	0.892648119	-0.154562203
Inverse v	109	0.0109498518	0.08270	0.894673717	-0.151156636
Inverse v	109	0.0114630429	0.08679	0.894928160	-0.158654319
Inverse v	109	0.0110519550	0.08435	0.895757632	-0.154277341
Inverse v	109	0.0108012850	0.08377	0.897413384	-0.153400922
Inverse v	109	-0.010880919	0.08347	0.896289119	-0.174490562
Inverse v	109	-0.012354793	0.09591	0.897508794	-0.200349392
Inverse v	109	0.0110153217	0.08476	0.896605018	-0.155124174
Inverse v	109	0.0107746647	0.08388	0.897799818	-0.153645637
Inverse v	109	0.0106411137	0.08285	0.897809013	-0.151755904
Inverse v	109	-0.010741747	0.08442	0.898758043	-0.176219689
Inverse v	109	0.0111641637	0.08901	0.900194149	-0.16330915
Inverse v	109	-0.010558527	0.08451	0.900573553	-0.176199928
Inverse v	109	0.0104661990	0.08453	0.901471628	-0.155231143
Inverse v	109	0.0109963692	0.09064	0.903446878	-0.166674005
Inverse v	109	-0.010232946	0.08475	0.903903102	-0.176357334
Inverse v	109	-0.010325472	0.08683	0.905342654	-0.180513557
Inverse v	109	-0.010256542	0.08693	0.906082444	-0.180646388
Inverse v	109	-0.01008147	0.08541	0.906043767	-0.177493615
Inverse v	109	-0.010124856	0.08634	0.906650832	-0.179355938
Inverse v	109	-0.012719972	0.10885	0.906977182	-0.22607622
Inverse v	109	-0.010288539	0.09137	0.910351150	-0.189385521
Inverse v	109	-0.009239787	0.08262	0.910962238	-0.171189231
Inverse v	109	-0.009853763	0.08933	0.912173878	-0.184957212
Inverse v	109	-0.008998014	0.08256	0.913217067	-0.170824449
Inverse v	109	-0.009226478	0.08568	0.914249577	-0.177167649
Inverse v	109	0.0096557030	0.09024	0.914797824	-0.167233549
Inverse v	109	-0.008730268	0.08340	0.916630422	-0.172194792
Inverse v	109	0.0087668302	0.08463	0.917495347	-0.157109391
Inverse v	109	0.0086144351	0.08415	0.918470188	-0.156334162
Inverse v	109	0.0088035749	0.08662	0.919051262	-0.160980923
Inverse v	109	-0.008265652	0.08313	0.920801823	-0.171211396

Inverse v	109	0.00785064471	0.08139(	0.9231583181	-0.151675394
Inverse v	109	-0.008407586	0.08982	0.925426709(	-0.184463394
Inverse v	109	0.0086816112(	0.09237	0.925126955	-0.172382378
Inverse v	109	-0.008244386	0.08812	0.925461341(	-0.180963225
Inverse v	109	0.0076193152	0.08450(	0.928158228(	-0.158013714
Inverse v	109	0.0076016082	0.08437	0.928211981	-0.157770566
Inverse v	109	-0.007377271	0.08488(	0.9307398171	-0.173742191
Inverse v	109	0.0069764783(	0.08134	0.931651432(	-0.152453792
Inverse v	109	-0.007319854	0.08594	0.932125070	-0.175767276
Inverse v	109	-0.006915867	0.08093	0.931903704	-0.16554794
Inverse v	109	0.0080613570	0.09407	0.931715492	-0.1763344
Inverse v	109	-0.007014655	0.08355	0.933091591	-0.170776147
Inverse v	109	-0.006832964	0.08212	0.933690641	-0.167797283
Inverse v	109	0.0066949876	0.08558(	0.937649572(	-0.161055482
Inverse v	109	0.0066104467	0.08525	0.938198806	-0.160496742
Inverse v	109	-0.006640553	0.08623	0.938621198	-0.175666328
Inverse v	109	0.0063068356	0.08429	0.940360798	-0.158916284
Inverse v	109	0.0060252435	0.08391	0.942757915	-0.15844307
Inverse v	109	0.0066434323	0.09210	0.942501153	-0.173888112
Inverse v	109	-0.006753995	0.09670	0.944320011	-0.196295709
Inverse v	109	-0.007457266	0.10675	0.944309999	-0.216697642
Inverse v	109	-0.005718111	0.08385(	0.945635088	-0.170077151
Inverse v	109	-0.005712396	0.09086	0.949872495	-0.18380765
Inverse v	109	0.0053133006	0.08700	0.951302367	-0.165209625
Inverse v	109	0.0053951899	0.08784	0.951027258(	-0.166781953
Inverse v	109	0.0054796502	0.09148	0.952240306	-0.173839986
Inverse v	109	-0.005167569	0.08520	0.9516409031	-0.172175884
Inverse v	109	-0.005523655	0.09178	0.9520103471	-0.185416119
Inverse v	109	0.0057371319	0.09527	0.951985653	-0.181011564
Inverse v	109	0.0049432591	0.08705	0.954718984	-0.165688565
Inverse v	109	-0.004760072	0.08328	0.954424884	-0.168007201
Inverse v	109	-0.004156777	0.08059	0.958866203	-0.162122009
Inverse v	109	-0.004478918	0.08521(	0.958080230	-0.171491977
Inverse v	109	-0.004398115	0.08462	0.958549239	-0.170255449
Inverse v	109	0.0046855845	0.08955(	0.958271059	-0.170833537
Inverse v	109	-0.004390848	0.08323(	0.957926732(	-0.167521981
Inverse v	109	0.0045940300(	0.08988	0.959239520	-0.171588014
Inverse v	109	-0.003989244	0.08241	0.961393866	-0.165522093

Inverse v	109	-0.004177072	0.08972	0.962867168	-0.18003144
Inverse v	109	-0.004347594	0.09618	0.963947707	-0.192870615
Inverse v	109	0.0037143528	0.08419	0.964812798	-0.161312155
Inverse v	109	-0.003998642	0.09083	0.964889282	-0.182043197
Inverse v	109	-0.003857402	0.08642	0.964400107	-0.173251444
Inverse v	109	-0.003875574	0.08570	0.963933847	-0.171865928
Inverse v	109	0.0035731192	0.08638	0.967007420	-0.165745046
Inverse v	109	-0.003498901	0.08561	0.967400112	-0.171298475
Inverse v	109	0.0034180423	0.08481	0.967852850	-0.162813545
Inverse v	109	-0.003334228	0.08508	0.968742064	-0.170105035
Inverse v	109	0.0032789267	0.08607	0.969612261	-0.165424703
Inverse v	109	0.0033890483	0.08973	0.969872552	-0.172487655
Inverse v	109	0.0038261371	0.10763	0.971644615	-0.207148223
Inverse v	109	-0.002588246	0.08449	0.975564340	-0.168207077
Inverse v	109	-0.002517255	0.08188	0.975474801	-0.163005182
Inverse v	109	-0.002230343	0.07618	0.976644811	-0.151551862
Inverse v	109	0.0024855292	0.08469	0.976586784	-0.163508171
Inverse v	109	0.0024238012	0.08134	0.976229828	-0.157015941
Inverse v	109	0.0022947311	0.08687	0.978927867	-0.167987348
Inverse v	109	0.0024421022	0.10163	0.980830439	-0.196765552
Inverse v	109	0.0022758972	0.10479	0.982673958	-0.203131128
Inverse v	109	0.0014756217	0.08559	0.986244930	-0.166283787
Inverse v	109	0.0012842133	0.08210	0.987521442	-0.159651059
Inverse v	109	0.0013761878	0.08475	0.987044720	-0.164738478
Inverse v	109	0.0013189598	0.08542	0.987681322	-0.166116093
Inverse v	109	0.0010567843	0.08374	0.989931230	-0.163075706
Inverse v	109	0.0010025041	0.08939	0.991052481	-0.174212211
Inverse v	109	-0.001051744	0.09370	0.991045195	-0.184722898
Inverse v	109	0.0007958058	0.08063	0.992125533	-0.157247107
Inverse v	109	-0.000716076	0.07810	0.992685200	-0.153806085
Inverse v	109	-0.000560334	0.08274	0.994596797	-0.162736936
Inverse v	109	0.0004390353	0.08422	0.995841128	-0.164649956
Inverse v	109	-0.000489063	0.09331	0.995818114	-0.183377894
Inverse v	109	0.0005127398	0.08550	0.995215284	-0.167072025
Inverse v	109	-0.000394771	0.08333	0.996220451	-0.163737522
Inverse v	109	-0.000338614	0.08725	0.996903513	-0.171352199
Inverse v	109	-0.000321028	0.10050	0.997451387	-0.197306676
Inverse v	109	0.0002480387	0.09255	0.997861805	-0.181164743

index and circulating protein levels(deCODE unique)

up_ci	or	or_lci95	or_uci95	exposure
0.63193358828	1.58438683870	1.33437280390	1.88124461730	6715_63_ALPPL2_PP
0.63556305454	1.55769734170	1.28512280390	1.88808493700	13231_90_IGHG4_IgG
0.57845595180	1.49139365670	1.24728113960	1.78328282920	9266_1_TREM1_sTRE
0.57917943940	1.48221815480	1.23109005270	1.78457347910	14260_112_NET1_AR
0.53215869632	1.41982013740	1.18400374950	1.70260374880	8464_31_RSPO4_RSP
0.51194691158	1.39806021620	1.17142917500	1.66853652780	2731_29_POR_NADP
0.44837371781	1.33391990630	1.13640536620	1.56576374010	12768_3_PPP1R3B_PI
0.49600499133	1.37389543030	1.14946334670	1.64214775430	13671_40_ELANE_El
0.45447333585	1.33500722570	1.13133694770	1.57534348750	5264_65_CALR_calret
0.34457451246	1.19514436570	1.01203126020	1.41138926330	10008_43_GUCA1A_(
0.41141375542	1.27037937160	1.06952795960	1.50894956340	10011_65_OCRL_OCI
0.35580092494	1.20523840660	1.01770884000	1.42732337540	10014_31_SNAI2_SLU
0.35624204946	1.20477287640	1.01647430960	1.42795314170	10030_8_MUTYH_Mu
0.37362761035	1.23005362810	1.04131873750	1.45299596900	10044_12_WNT10A_V
0.37128110257	1.22749137810	1.03942119080	1.44959049970	10081_17_SATB2_SA
0.44220598551	1.30532562770	1.09493946660	1.55613624890	10342_55_PIAS4_PIA
0.33029673804	1.18481233250	1.00891152100	1.39138094280	10396_6_MCL1_Mcl_
0.37379842770	1.23542463590	1.05025297510	1.45324418710	10426_21_GOSR2_GC
0.36254632127	1.21898550970	1.03405876340	1.43698378230	10449_31_SHISA2_TI
0.36254771686	1.21700152360	1.03069405490	1.43698578780	10527_22_DHX9_DH
0.33713871834	1.18914146970	1.00936807540	1.40093338540	10530_8_PSMB6_PSE
0.33604394671	1.19081034140	1.01331194780	1.39940052250	10546_2_TSPEAR_TS
-0.094591664	0.73516594550	0.59408884870	0.90974433980	10565_19_SLITRK3_S
0.33698633600	1.19163089480	1.01375311720	1.40071992410	10582_36_POMK_SG
0.35522671063	1.20944981260	1.02542217010	1.42650402120	10630_5_HTATIP2_H
0.45055521321	1.31298746970	1.09862004840	1.56918317490	10672_75_SFTPB_SP_
0.35758128951	1.20741360570	1.01956883010	1.42986679480	10675_223_TMX2_TN
0.40146226589	1.25107481860	1.04764397280	1.49400773770	10695_12_WFDC10B_
0.33891012916	1.19030683880	1.00955749800	1.40341721320	10734_339_GAL3ST2
0.42155565724	1.25955153440	1.04076477950	1.52433104870	10754_113_PROK2_P
0.36552646844	1.21529975450	1.02475652460	1.44127259290	10758_2_KERA_KER
0.36976023661	1.22579079290	1.03812076850	1.44738754250	10772_21_CSGALNA
0.39339110535	1.23695507520	1.03242917210	1.48199789330	10814_7_CD1A_CD1/
0.38476883906	1.24399890990	1.05326345550	1.46927464320	10817_26_TOMM20L
0.37493033118	1.23305625350	1.04504647880	1.45489005060	10848_137_BTNL3_B
0.38164818747	1.23310278000	1.03812787350	1.46469669580	10900_272_STMN2_S

0.43979397653	1.29839086532	1.08595244022	1.55238735731	11098_1_PDXK_PDX
0.37915383831	1.23858397333	1.04999321455	1.46104778355	11118_107_RAB17_R
0.35495480682	1.20645327558	1.02062476022	1.42611620211	11120_49_NAT14_NA
0.37178241651	1.22377392477	1.03261716200	1.45031738188	11158_40_CCDC64_B
0.35507709870	1.21111879877	1.02840804559	1.42629061511	11164_7_TENM2_TE
0.45953198230	1.31337771311	1.08944944160	1.58333278388	11214_40_DNAJB9_D
0.37068788556	1.21991226460	1.02723425188	1.44873083300	11228_37_RIC3_RIC3
0.39187355962	1.25551251188	1.06525496133	1.47975059933	11260_47_SUN5_SPA
0.36127641415	1.20360228944	1.00940547788	1.43516010460	11262_39_TRABD_TI
0.35803551361	1.19790573033	1.00311895500	1.43051642233	11263_57_CASQ1_CA
0.34644018451	1.19190145455	1.00467047399	1.41402491077	11281_6_GRB7_GRB
0.36624249486	1.20482373933	1.00644474744	1.44230495177	11283_13_DCAF5_DC
0.37175864504	1.23182203899	1.04626864811	1.45028290600	11292_13_SCARA3_S
0.40375858390	1.26227347955	1.06403714800	1.49744239660	11303_7_SAMHD1_S
0.36240011703	1.20860775788	1.01667556100	1.43677370455	11311_79_RAG1_RAG
0.35596497716	1.19949289388	1.00786353722	1.42755755022	11315_148_PPM1D_P
0.38320602816	1.23153087333	1.03387097699	1.46698023811	11320_29_CHFR_CHI
0.38778717375	1.24476590600	1.05138442555	1.47371610544	11363_58_AQP4_AQI
0.33826366113	1.18673450888	1.00415580022	1.40251024211	11372_2_ZNF18_ZNF
0.33195030452	1.18423627844	1.00626539355	1.39368358700	11377_19_ADH7_ADI
0.34159146374	1.19088646155	1.00783498122	1.40718529388	11380_84_RBM24_RI
0.34615439156	1.20363006077	1.02483301866	1.41362085011	11396_39_DNAI1_DN
0.35629695822	1.20427642900	1.01558100488	1.42803155100	11429_80_HNRNPC_I
0.41328726841	1.25824002755	1.04722165411	1.51177924999	11439_88_RHPN2_RH
0.35932594731	1.19870856755	1.00316862933	1.43236360055	11456_2_MAGEB10_I
0.36478932741	1.21844250188	1.03082297000	1.44021056333	11459_81_ELL_ELL
0.36062936071	1.20346338399	1.00982570399	1.43423177977	11556_19_ARFGAP1_
0.38191058427	1.22472599244	1.02380255888	1.46508107799	11570_94_BCAP29_B
0.39445117719	1.24363280122	1.04250073	1.48356975055	11601_26_DHX8_DHI
0.33097544172	1.18153198160	1.00265184011	1.39232559877	11643_73_DTX3L_DT
0.41788766630	1.26987493560	1.06178257811	1.51875005800	11654_77_NRSN1_NI
0.37076875677	1.22905806099	1.04261021100	1.44884799833	11656_110_EVL_EVL
0.38666873511	1.23143705400	1.03014020355	1.47206876577	11671_19_BRPF1_BR
0.35851343339	1.20999360000	1.02297669666	1.43120025788	11672_17_KIF16B_KI
0.34651148761	1.20401338911	1.02511976233	1.41412573860	11678_105_GJD2_CX
0.35164679176	1.19330608999	1.00181020822	1.42140638260	11692_21_SHC4_SHC
0.35988513496	1.21120449760	1.02362013844	1.43316478455	11872_9_PCDHGB1_I
0.33486910579	1.18784849099	1.00946274533	1.39775741499	12001_7_TJP1_ZO1

0.33720198569	1.18971178881	1.01027258455	1.40102202155	12046_51_TARDBP_T
0.34323478173	1.19268322299	1.00921861822	1.40949964788	12350_86_ETS2_ETS2
0.34721628836	1.20187592206	1.02076354499	1.41512276688	12352_70_ARRDC3_A
0.38230317569	1.23437316833	1.03958687099	1.46565636911	12386_11_RNPEP_AM
0.35301099033	1.20792615488	1.02510899633	1.42334678644	12392_30_ARL1_ARL
0.42445852574	1.26191831011	1.04165160722	1.52876241006	12400_25_UBE2T_UE
0.33224349052	1.18513725077	1.00750169	1.39409225544	12430_78_PAIP1_PAI
0.42566058376	1.25248361806	1.02490135122	1.53060117606	12436_84_GSTO1_GS
0.38351378247	1.23422539855	1.03808051466	1.46743177711	12439_67_IRF9_ISGF
0.40063649510	1.24519078506	1.03866997377	1.49277453906	12452_32_SUV420H2_
0.38736592532	1.22781083499	1.02336848644	1.47309543555	12455_48_MYNN_MY
0.35286538370	1.19622172822	1.00548566744	1.42313955277	12456_5_PFKFB3_F26
0.40401049971	1.27294543706	1.08183255688	1.49781967355	12463_7_KLHL13_KL
0.34110852540	1.19315531488	1.01216755033	1.40650587411	12478_15_RPL30_RL3
0.34505151537	1.19803668666	1.01645057444	1.41206266077	12480_9_OTUD5_OTU
0.35747893970	1.20782878477	1.02037455455	1.42972045588	12484_67_PITPNB_PI
0.33898326718	1.19413683599	1.01599045622	1.40351986011	12507_16_ITPKC_IP3
0.34607622686	1.20253366344	1.02304677306	1.41351035911	12516_13_TEAD4_TE
0.33935054270	1.19183957206	1.01171347377	1.40403543333	12521_3_CDKN2C_CI
0.33230255401	1.18296348266	1.00374989155	1.39417459788	12530_14_CKS1B_CK
0.33468913556	1.18378848977	1.00275441106	1.39750588288	12533_135_CYTH2_C
0.37639629386	1.23846743877	1.05269449577	1.45702442922	12553_5_VRK1_VRK
0.41699239982	1.24305540333	1.01831812344	1.51739098044	12572_236_EFS_EFS
0.39805499398	1.23141697211	1.01844406733	1.48892590966	12604_16_SCMH1_SC
0.38103330881	1.22092468277	1.01835003811	1.46379636199	12618_50_AKR1C1_A
0.34374859918	1.19136528733	1.00647215488	1.41022405944	12625_138_KLHL7_K
0.42594178848	1.27381572106	1.05981250766	1.53103164888	12664_19_PTPN13_PT
0.36143789050	1.20597248711	1.01322131777	1.43539186777	12671_35_SULT1B1_
0.37186010915	1.21898819322	1.02447698166	1.45043006522	12681_63_USP21_UB
0.39740739480	1.24083813377	1.03475712399	1.48796199455	12683_156_DPYSL5_
0.36201199414	1.20567186722	1.01213500022	1.43621616806	12700_9_ACLY_ACL
0.37731265996	1.22939765506	1.03638222222	1.45836020899	12705_9_HERC1_HEI
0.36624936002	1.20826562106	1.01219633677	1.44231485344	12716_3_ZNF175_ZN
0.34827666698	1.20000708866	1.01651312006	1.41662412866	12730_3_NDC80_KNT
0.41440734041	1.26134562822	1.05121945877	1.51347350022	12759_47_ILF3_DRBI
0.37656203619	1.21059171333	1.00567251066	1.45726593988	12772_8_NUP98_NUI
0.40313805907	1.24598194655	1.03739192922	1.49651348466	12775_6_HMGB3_HM
0.33163112250	1.18227580833	1.00325663311	1.39323881922	12790_10_CCDC51_C

0.33532266416	1.18484122688	1.00390249044	1.39839152322	12814_17_RNPC3_RE
0.38224500744	1.22725967500	1.02769923100	1.46557111699	12827_37_RGS3_RGS
0.38992016150	1.23770583688	1.03727689322	1.47686287860	12831_21_TESC_TES
0.36710245612	1.20857172522	1.01184569500	1.44354581160	12873_11_SPN_LEUK
0.38615465000	1.21632903860	1.00553528922	1.47131219160	12875_28_UBTD2_UI
0.36649964186	1.20651693844	1.00901605077	1.44267588388	12894_3_PAXIP1_PA
0.35377113867	1.21032683088	1.02840568444	1.42442915244	12925_105_AXIN2_A
0.36778177125	1.21771605111	1.02651775988	1.44452676733	12931_16_NR3C2_MC
0.37026633709	1.22984208911	1.04446544600	1.44812025144	12933_17_ENOX1_EN
0.41247557312	1.27275300288	1.07238911077	1.51055264377	12934_1_HERC5_HEI
0.37573376802	1.23465607055	1.04691853800	1.45605943255	12939_1_POLR1C_RI
0.35534440119	1.19573535960	1.00218069277	1.42667191711	12945_33_RAB18_RA
0.34331003640	1.19966912	1.02099897460	1.40960572322	12957_62_UQCRB_U
0.35764752325	1.20271696222	1.01158533833	1.42996150344	12970_35_STAU2_ST
0.33289160329	1.18535471377	1.00721845760	1.39499607733	12975_11_KRT20_Ke
0.33900234318	1.18778562511	1.00519260060	1.40354663399	12976_49_SORBS3_V
0.40949794933	1.28010716111	1.08805275877	1.50606147611	12986_12_LIN7C_LIN
0.38013843019	1.21432570000	1.00827349422	1.46248702777	12988_49_EWSR1_EV
0.39616301784	1.25334178922	1.05703076560	1.48611156055	12990_39_MAP3K3_N
0.35172012285	1.20610153422	1.02333453633	1.42151061977	13007_66_LCN10_LC
0.41077616529	1.25969056588	1.05227664577	1.50798777877	13013_41_PARK2_PR
0.35583009137	1.21231053811	1.02965732988	1.42736500600	13039_1_PPP1R8_PP1
0.32452293062	1.17818393855	1.00343137860	1.38337052488	13041_47_TRAF4_TR
0.40234223290	1.23368983660	1.01783401944	1.49532299388	13059_33_RFK_RIFK
0.34514367259	1.19517376011	1.01150517011	1.41219279855	13073_14_ANP32A_A
0.34563559840	1.19927371322	1.01795597511	1.41288766344	13083_18_VARS_SYV
0.44745509584	1.31600156033	1.10709663155	1.56432605560	13093_6_SECTM1_SE
0.37613042441	1.22320899244	1.02718805988	1.45663710244	13097_11_BCL2L2_A
0.35992661792	1.20499278777	1.01310568188	1.43322423777	13098_93_FIGF_VEG
0.35824196090	1.20860992560	1.02091552055	1.43081177911	13114_50_LUM_Lumi
0.33663247040	1.19558129822	1.02084687033	1.40022434533	13119_26_SERPINA10
0.38214286497	1.23796414788	1.04581194355	1.46542142755	13131_5_HK1_HXK1
0.35539179057	1.20281060644	1.01402766699	1.42673952788	13378_80_C5orf63_YI
0.37511078096	1.20994577788	1.00605859222	1.45515260888	13386_248_RNF34_RI
0.35114477949	1.20198021000	1.01693780933	1.42069299822	13429_3_WFDC10A_
0.43457985878	1.30464435099	1.10217014188	1.54431409260	13431_74_FAM159A_
0.35332331071	1.20456793511	1.01909866460	1.42379139600	13441_30_DIXDC1_D
0.37444415077	1.21225926311	1.01058301388	1.45418288344	13451_2_FNDC4_FNI

0.3434202033	1.200531333	1.0223544681	1.4097610237	13476_16_KIN_KIN17
0.3323731034	1.1846272168	1.0065042381	1.3942729594	13485_20_TMEM87B_
0.3629737310	1.2072561568	1.0138212019	1.4375980945	13490_1_MPP6_MPP6
0.3290064236	1.1810342957	1.0037818623	1.3895867817	13494_6_CERS5_CER
0.3968219782	1.2462895227	1.0444803949	1.4870911718	13495_48_HCAR2_HC
0.3423261635	1.1920443918	1.0090541988	1.4082195325	13502_2_SLC26A11_S
0.3557964294	1.2116437057	1.0285595364	1.4273169589	13506_10_DUS2_DUS
0.3877368300	1.2359150885	1.0365381783	1.4736419149	13513_174_PIAS3_PIA
0.3960445653	1.2527550417	1.0561664051	1.4859355372	13514_121_RAB35_R
0.3518121687	1.2083235644	1.0270141012	1.4216414698	13530_5_UTS2R_UR2
0.3944928432	1.2491245624	1.0516843991	1.4836315663	13539_131_KCNN1_K
0.3877528767	1.2475063929	1.0560552138	1.4736655622	13548_53_PPAPDC1A
0.3862727303	1.2401678117	1.0452129814	1.4714859349	13549_15_ADAM29_A
0.3715041448	1.2135964030	1.0157956789	1.4499138557	13580_2_UAP1_Sperm
0.3326795535	1.1847915435	1.0064750123	1.3947003000	13604_27_NEURL1_N
0.3458971083	1.1933930099	1.0077336809	1.4132571959	13609_11_GTF2I_GTI
0.3912160503	1.2186808418	1.0043312958	1.4787779694	13614_6_CREBBP_CI
0.3604487076	1.2048331063	1.0123085392	1.4339727047	13623_4_MLL2_MLL
0.3823667945	1.2315242958	1.0347279474	1.4657496155	13631_1_RBBP5_RB_
0.3666352548	1.2114586819	1.0171606374	1.4428715426	13645_14_DHX38_PR
0.3845378710	1.2467409657	1.0581562080	1.4689353270	13650_11_KCNIP1_K
0.3789874309	1.2285715119	1.0332578927	1.4608046747	13653_335_MACROD
0.3630842331	1.2120713811	1.0218118033	1.4377569608	13655_34_NCL_Nucle
0.3813028660	1.2115543452	1.0025085112	1.4641909919	13666_222_CA10_Car
0.3841708018	1.2363305010	1.0409405049	1.4683962249	13681_173_CSNK2A2
0.3774184223	1.2264390957	1.0312910158	1.4585144567	13698_28_KIAA0319I
0.3422081614	1.1885880352	1.0033295243	1.4080533694	13711_10_CLTC_CLH
0.3609548537	1.2029875145	1.0086988802	1.4346986881	13735_1_RAP1GAP_I
0.3565872674	1.2011363175	1.0099984660	1.4284461819	13941_82_FARS2_SY
0.3646842891	1.2124793948	1.0208651053	1.4400592940	13946_8_DDX23_DD
0.3966612301	1.2392167849	1.0328251171	1.4868521441	13947_371_NUDT12_
0.3719620553	1.2179300744	1.0225949441	1.4505779380	13955_33_DAPK1_DA
0.3535019176	1.1977471381	1.0074102178	1.4240457178	13961_18_KIF3A_KIF
0.4021093895	1.2509628225	1.0467787966	1.4949748584	13985_12_SMURF2_S
0.3722048152	1.2228586619	1.0306377150	1.4509301235	13994_1_FBL_FBRL
0.3787580338	1.2316315308	1.0386496361	1.4604696088	14024_196_EDA_EDA
0.3692301305	1.2176532701	1.0249263783	1.4466204769	14028_22_PIK3C2A_I
0.3560812836	1.1983770854	1.0058723164	1.4277235941	14043_12_EEA1_EEA

0.38280526343	1.2396254148	1.0479262747	1.4663924420	14045_12_NCOA2_NC
0.36529771439	1.2219011004	1.0361564392	1.4409429337	14051_54_FOXC2_FC
0.37235317453	1.2185611960	1.0232547270	1.4511453984	14060_67_NCF4_NCF
0.52820076715	1.3753204508	1.1153550124	1.6958782820	14100_63_C1QC_C1Q
0.35296140211	1.2026168971	1.0161677643	1.4232762069	14122_132_ZNRF3_Z
0.33665080114	1.1850068040	1.0028502869	1.4002500126	14149_9_IL36B_IL_1
0.34438588555	1.1962732160	1.0141352270	1.4111230624	14151_4_ISG15_UCR
0.39564299305	1.2621173322	1.0724421951	1.4853389465	14197_2_PRDM1_PR
0.33732615572	1.1869004755	1.0053787918	1.4011959973	14229_5_MOCS3_MC
0.34232978617	1.1946677644	1.0134967341	1.4082246339	14245_195_SNX7_SN
0.36188942157	1.2057438721	1.0123799791	1.4360401381	14599_18_STAB1_ST
0.38400075881	1.2381363862	1.0441612283	1.4681465557	14645_253_GSTA4_G
0.35055220962	1.2037939327	1.0206137381	1.4198513877	14692_3_ZNF276_ZN
0.38543879400	1.2314878523	1.0314930902	1.4702593208	15315_64_CUTC_CU
0.36332037556	1.2134447201	1.0238868337	1.4380965164	15318_75_S100A10_C
0.34493027346	1.19006437	1.0030892862	1.4118914699	15365_41_BAI3_BAI3
0.35118956092	1.2059762857	1.0236649831	1.4207566203	15403_53_ID2_ID2
0.37280358764	1.2361603403	1.0525508131	1.4517991605	15412_40_BIRC7_KIA
0.33110151570	1.1820064047	1.0033306938	1.3925011458	15416_54_LYG2_LYG
0.40773923148	1.2319864895	1.0095619925	1.5034150667	15426_5_NEU1_NEU
0.46822408909	1.3093268841	1.0733689606	1.5971552676	15468_14_CFHR1_FH
0.38344395331	1.2363417576	1.0417163550	1.4673293111	15470_11_HEXB_Hex
0.35513518978	1.1956263586	1.0022076386	1.4263734723	15585_304_FBLN5_fi
0.34343671866	1.1899536996	1.0044017377	1.4097843066	15591_28_GPX1_Glut
0.46013412021	1.2810225335	1.0358093548	1.5842864555	15594_47_HTRA1_HT
0.33680634795	1.1872919748	1.0065652343	1.4004678340	15668_19_BMP8B_BM
0.36357072883	1.2092796574	1.0166155143	1.4384565936	15688_30_LMAN1_M
0.41055178666	1.2724029433	1.0738631869	1.5076494565	16015_19_GPT_ALT
0.35860534371	1.2151423227	1.0316062693	1.4313318059	16021_30_CDH8_Cad
0.38603505667	1.2495585512	1.0613541610	1.4711362430	16296_43_LGR5_LGF
0.37402477905	1.2312703811	1.0429655587	1.4535731681	16320_139_CD320_CI
0.38693345573	1.2250093289	1.0191444116	1.4724585043	16536_3_HMGA1_HM
0.32817456624	1.1837756855	1.0092864148	1.3884313243	16585_16_B3GNT4_B
0.34004012680	1.1913432149	1.0101741265	1.4050039677	16588_10_HSPA5_BiF
0.40246117514	1.2716566930	1.0813171615	1.4955008614	16754_40_CASK_CSK
0.35349019195	1.1987292961	1.0090748891	1.4240290199	16755_195_GDF3_GD
0.34703595002	1.1918724931	1.0040233099	1.4148675889	16785_45_DEFA5_HD
0.35962348467	1.2073578768	1.0173948727	1.4327898456	16850_5_RGS5_RGS5

0.36598370162	1.21398251611	1.02206887282	1.44193174133	16854_17_LSM4_LSM
0.33917564653	1.19339751400	1.01453759722	1.40378989433	16877_19_MSRB2_M
-0.035810509	0.81944703400	0.69597570888	0.96482310100	16907_3_CADM2_Ne
0.34210250455	1.19197149888	1.00915647777	1.40790460677	16926_44_ALPL_Alka
0.37527657065	1.22485996933	1.03084255533	1.45539387822	17202_37_PDCD4_PD
0.35687282130	1.20367547244	1.01398358588	1.42885413844	17204_17_PNMT_PNI
0.39957952965	1.25797414622	1.06122689144	1.49119756144	17326_44_DUSP23_D
0.36678645112	1.21634925560	1.02523460277	1.44308971600	17329_2_BDH2_BDH
0.37782627177	1.21536957833	1.01234573588	1.45910943233	17331_138_KREMEN
-0.049171781	0.80494929600	0.68060021677	0.95201757699	17346_61_FDCSP_FD
0.34277825648	1.19598415422	1.01527606077	1.40885632255	17355_56_SYT4_SYT
0.42436297004	1.28322002044	1.07721838588	1.52861633500	17365_7_CCBL1_KA7
0.33409921171	1.18482755177	1.00510826733	1.39668170388	17367_5_STMN1_Stat
0.32916499407	1.18083474944	1.00328359144	1.38980714660	17393_13_ACADS_SC
0.44813067249	1.27069520288	1.03148306599	1.56538323488	17396_23_ADH1A_AI
0.39008538053	1.21683676060	1.00242690444	1.47710690460	17404_5_ARL5B_ARI
0.37805059647	1.23669152911	1.04794257322	1.45943678333	17432_25_AK4_KAD4
0.38848120644	1.23605033611	1.03599359360	1.47473926760	17435_43_ETFA_ETF
0.39123382151	1.25137352644	1.05892020760	1.47880424922	17449_23_CD9_CD9
0.44410245978	1.29813475060	1.08085716111	1.55909022144	17460_51_MX1_Mx1
0.33187566130	1.18400855388	1.00595351277	1.39357956199	17462_19_IRF1_IRF1
0.35459945874	1.19772468155	1.00626741622	1.42560952444	17505_125_NAALAD
0.40552583084	1.26047931544	1.05914108722	1.50009108688	17515_6_HSPA13_ST
0.37598064178	1.23387341488	1.04533356599	1.45641893988	17516_7_RAB3A_Rat
0.38877125550	1.23359106688	1.03157597844	1.47516707633	17673_34_CA5B_Cart
0.34888020957	1.20572336377	1.02560139533	1.41747937977	17702_53_UGT1A1_U
0.33619044104	1.19066176460	1.01291070622	1.39960554177	17710_40_TPD52L1_7
0.36084699507	1.20831656700	1.01776520988	1.43454395177	17760_128_WDR5_W
0.34662441519	1.19387968922	1.00782251633	1.41428544144	17766_5_NCF1_NCF_
0.36391245624	1.22277433588	1.03907634560	1.43894823777	17771_35_CRCP_RPC
0.35563355183	1.21601453855	1.03616244022	1.42708449999	17789_1_QPRT_NAD
0.33821285385	1.19396001588	1.01647239811	1.40243898611	17797_1_CUL1_CUL1
0.36141090457	1.21728618500	1.03234919844	1.43535313288	17800_33_SCAND1_S
0.34280281067	1.19703531660	1.01703654460	1.40889091622	17812_2_PTP4A1_TP4
0.36144557359	1.20175679355	1.00614217433	1.43540289600	17817_22_DDIT4_DD
0.39925692113	1.23799121833	1.02811110555	1.49071656600	17822_57_RERG_REI
0.34566316240	1.19352736655	1.00819643844	1.41292660888	17823_40_SNRPD2_S
0.39866592256	1.23928394688	1.03086842555	1.48983581499	18170_46_PYCR1_P5

0.39836476728	1.2584369493	1.0632987475	1.4893872105	18177_49_NAE1_ULA
0.37981564146	1.2283392915	1.0320122464	1.4620150296	18178_13_SARS_SYS
0.36645896376	1.2179920871	1.0283426016	1.4426171997	18182_24_PCK1_PCK
0.43579634058	1.2876622749	1.0723584999	1.5461938657	18187_16_ASPA_ACY
0.35361647784	1.2073012744	1.0234287974	1.4242088660	18192_69_MAGEA5_
0.37441718465	1.2174852515	1.0193424267	1.4541436703	18202_22_CD37_CD3
0.35740033234	1.2022287562	1.0110141436	1.4296080736	18205_123_MAGEA6_
0.34468956965	1.1981938741	1.0170853802	1.41155166	18208_3_CCNB2_CCI
0.36636376188	1.2275731058	1.0446840648	1.4424798664	18210_12_SPIN1_SPI
0.37977593817	1.2403325359	1.0523051064	1.4619569839	18220_141_SRA1_SR
0.35243250168	1.2057469242	1.0220045629	1.4225236345	18227_3_CDK2AP2_C
0.34159625060	1.1966513461	1.0176112527	1.4071920298	18228_30_NOP16_NC
0.40733616883	1.2577792604	1.0527009340	1.5028092183	18242_8_SPA17_Sper
0.35905562253	1.1994562034	1.0046919307	1.4319764495	18255_6_CYB5R1_NI
0.43396753100	1.2856478579	1.0709627291	1.5433687556	18259_15_MEOX2_M
0.39697818795	1.2628717970	1.0722920660	1.4873234881	18270_10_TAF10_TA
0.35349351128	1.2105462268	1.0290642133	1.4240337467	18273_14_GAGE2D_C
0.35220488144	1.1967609150	1.0070572440	1.4221998762	18274_2_SERF2_SER
0.36490680707	1.2189679901	1.0315911076	1.4403797686	18282_1_GNG11_GB(
0.38296788843	1.2457916341	1.0582054149	1.4666309334	18285_6_SVIP_SVIP
0.38234391634	1.2440784164	1.0559556008	1.4657160821	18286_3_ZWINT_ZW
0.37405175400	1.2099260910	1.0070918265	1.4536123787	18290_6_ANXA8_Anı
0.38592117024	1.2328521840	1.0332813316	1.4709687101	18297_8_MRAS_RAS
0.37464789181	1.2308392745	1.0415861084	1.4544791904	18302_204_ZWILCH_
0.39668682751	1.2319535959	1.0207274609	1.4868902040	18304_19_BCCIP_BC
0.38477004236	1.2331379090	1.0349510079	1.4692764112	18323_39_CRMP1_DI
0.40759592044	1.2660669873	1.0663424793	1.5031996261	18326_50_MAGEA8_
0.34915762357	1.2002816564	1.0160828207	1.4178726628	18375_28_IL1F5_IL_1
0.34443822365	1.1907210511	1.0046908420	1.4111969198	18389_11_IFNA1_IFN
0.36355162184	1.2170690002	1.0297740373	1.4384291093	18399_1_AKAP7_AK
0.32514859900	1.1777494782	1.0020643199	1.3842363268	18410_26_ARL14_AR
0.36080479486	1.2161399185	1.0310306037	1.4344834150	18414_26_ARL5A_AI
0.33904197584	1.1901414623	1.0091439287	1.4036022613	18449_33_PPIG_PPIG
0.33366821169	1.1875988880	1.0102510289	1.3960798637	18837_9_SGTB_SGTI
0.39826950090	1.2352174604	1.0245203695	1.4892453287	18840_205_SH2D1B_
0.35693259131	1.2131236086	1.0299028369	1.4289395436	18842_24_UBE2D2_U
0.34463491477	1.1959088371	1.0132651560	1.4114745171	18859_7_EIF4EBP3_4
0.35801044680	1.2092978959	1.0223147643	1.4304805643	18866_8_PPP3R2_CA

0.3324971841	1.183082208	1.003756000	1.394445972	18883_4_DSTN_DES
0.4259848705	1.263520681	1.042705901	1.531097610	18893_26_GPR56_GP
-0.049056452	0.806417961	0.683007276	0.952127379	18896_23_HS6ST3_H
0.3552325757	1.212164487	1.030024524	1.426512387	18904_23_CAMK4_K
0.3298283039	1.181068296	1.003014962	1.390729325	18927_14_RAET1G_
0.3692317407	1.221419243	1.031274331	1.446622806	18938_3_VLDLR_VL
0.3498592821	1.192762723	1.002688792	1.418867874	18943_4_PTGR2_PT
0.3558118433	1.208363598	1.02298237	1.427338959	19111_10_UBE2M_U
0.3360491404	1.190568911	1.012895842	1.399407790	19118_47_OSTF1_OS
0.3970914930	1.262944626	1.072294242	1.487492019	19123_6_NFKBID_IK
0.3952353494	1.243234714	1.041016769	1.484733581	19126_4_RPRD1B_C
0.3476733652	1.194866868	1.008431526	1.415769734	19168_71_PSTPIP1_P
0.3257603473	1.178322823	1.002426774	1.385083390	19176_27_FAM49B_
0.4154322181	1.269106504	1.063105145	1.515025420	19199_3_POLR2J_RP
0.3453245027	1.195846380	1.012460900	1.412448188	19208_8_ACVR2A_A
0.3890541140	1.236844989	1.036731975	1.475584399	19247_1_UBE2D1_U
0.3723432589	1.214123291	1.015825144	1.451131009	19280_29_UBE2D3_
0.3642941933	1.209073441	1.015533852	1.439497642	19281_86_MAFG_MA
0.4494752557	1.297585281	1.074155602	1.567489438	19294_26_CRIPT_CR
0.3777963349	1.237223454	1.049110963	1.459065751	19302_7_GIMAP6_GI
0.3602455421	1.217411894	1.033766442	1.433681400	19303_64_PET117_PT
0.3322610657	1.181800874	1.001819468	1.394116757	19316_2_HSCB_HSC
0.3481574239	1.200961468	1.018252065	1.416455216	19353_25_OSTM1_O
0.3525519828	1.207429974	1.024737253	1.42269361	19356_20_TOM1L2_T
0.3963358275	1.253496554	1.057109136	1.486368397	19366_8_PEX26_PEX
-0.113456143	0.751173908	0.632054263	0.892743349	19372_7_MDGA2_MI
0.4214896373	1.293594659	1.097857073	1.524230415	19483_16_KDSR_KD
0.3690873362	1.216138970	1.022524723	1.446413922	19491_11_HSD17B8_
0.3860772045	1.214943870	1.003324065	1.471198249	19506_6_GLYAT_GL
0.3750282590	1.221720907	1.025820345	1.455032531	19518_12_HK3_HXK
0.3645605664	1.223686355	1.039952714	1.439881137	19587_12_KPNA1_IM
0.3546739934	1.204618264	1.017808162	1.425715785	19635_69_FGL2_FGL
0.3874466333	1.245632612	1.053207651	1.473214330	19636_23_HLA_G_HI
0.3697953505	1.210590795	1.012499121	1.447438366	2431_17_CTSG_Cath
0.3711691716	1.205474379	1.002580483	1.449428254	2447_7_PLA2G2E_GI
0.3500274425	1.196031247	1.008022126	1.419106491	2599_51_TNFSF9_4_
0.3762159096	1.224995945	1.030103371	1.456761628	2640_3_MST1R_MSP
0.3718092987	1.215603343	1.018847174	1.450356370	2734_49_NCR2_NKp

0.35040934579	1.2028512258	1.0191614428	1.4196485568	2770_51_CCL1_I_309
0.42936460995	1.2924520920	1.08732212911	1.53628107560	2780_35_LTF_Lactofe
0.37709310737	1.22725353332	1.03299715755	1.45804005733	2806_49_ACVR1B_A
0.35803943260	1.21175082711	1.02643653000	1.43052202855	2826_53_EDA_EDA
0.41381824201	1.27750867255	1.07896842377	1.51258217800	2853_68_CHEK1_CHI
0.39933160603	1.24406274660	1.03814270799	1.49082790411	3057_55_WISP1_WIS
0.43117545042	1.29787629922	1.09448417022	1.53906555559	3179_51_CTSA_Cathe
0.39135392450	1.24700854311	1.05141945244	1.47898186877	3327_27_NTN4_NET4
0.37738313913	1.22617470411	1.03088279144	1.45846299655	3342_76_ABL2_ABL2
0.36573139141	1.21060797778	1.01665110733	1.44156797311	3346_72_AURKB_AU
0.35074763860	1.20317093500	1.01935838633	1.42012889499	3374_49_HCK_HCK
0.38231448925	1.22650624622	1.02636646944	1.46567295100	3388_58_PAK7_PAK7
0.39504552034	1.25180563444	1.05562025411	1.48445176211	3394_81_PLK1_PLK_
0.40938124653	1.26873454011	1.06893060133	1.50588572477	3401_8_PTPN2_TCPT
0.35451569960	1.20759183555	1.02300116	1.42549012160	3413_50_BCL2A1_BF
0.34929849917	1.19585195160	1.00845476533	1.41807242060	3418_12_CAMK1D_C
0.43341374347	1.28529079111	1.07096084900	1.54251429399	3474_19_THBS1_Thro
0.37688556956	1.22789896822	1.03429862122	1.45773749022	3497_13_IFNA2_IFN_
0.42815005568	1.28222470899	1.07148248600	1.53441631155	3520_58_TGFB3_TGF
0.40610416346	1.26108396177	1.05954451500	1.50095888933	3627_71_MMEL1_MM
0.36282841909	1.20628840277	1.01234356077	1.43738920955	3809_1_FGFR3_FGFF
0.33833558468	1.18845685332	1.00700021033	1.40261111922	3837_6_ZAP70_ZAP7
0.33545607536	1.18677664177	1.00705051833	1.39857809688	3890_8_LDHB_LDH_
0.37860736857	1.22783249999	1.03240751799	1.46024958322	4126_22_BPI_BPI
0.38341274281	1.23357123355	1.03708517944	1.46728351588	4194_26_ANP32B_AN
0.39753400862	1.24420069500	1.04024121	1.48815040300	4280_47_PSMA2_PSA
0.34462861998	1.19472031922	1.01125851633	1.41146563211	4284_18_RBM39_RBI
-0.006129501	0.83011540322	0.69332834160	0.99388924599	4324_33_CST2_CYTI
0.43662452286	1.28261453800	1.06308672599	1.54747492655	4359_87_DYRK3_DY
0.46653508006	1.33007915311	1.10953590933	1.59445993488	4413_3_SLPI_SLPI
0.34465980374	1.19702338099	1.01512942322	1.41150965	4429_51_CHST6_CHS
0.42286576461	1.30078988777	1.10857743933	1.52632939477	4453_83_LRRTM3_LI
0.33801795518	1.18634592655	1.00374490560	1.40216567933	4469_78_CHST15_ST
0.37709301541	1.23203176755	1.04105673111	1.45803992322	4540_11_CBX5_CBX5
0.37315451010	1.22236843560	1.02883400260	1.45230871888	4553_65_HDGFRP2_F
0.39702392545	1.25930233622	1.06619027800	1.48739151611	4560_34_LCK_LCK
-0.058885665	0.79701197888	0.67375719622	0.94281455977	4568_17_SLITRK5_SI
-0.005382464	0.84109496911	0.71125878760	0.99463199522	4842_62_GPC3_Glypi

0.37595200172	1.23659076855	1.04997297333	1.45637722855	4920_10_LYZ_Lysozy
0.39902529681	1.24396053860	1.03829012355	1.49037131977	4984_83_ESD_Esteras
0.36594486546	1.20194047999	1.00193163244	1.44187574333	4997_19_EIF4A3_IF4.
0.36668521838	1.20600025444	1.00796495300	1.44294363544	5009_11_MSN_Moesi
0.35719971589	1.20042260021	1.00818088955	1.42932129955	5094_62_AMICA1_JA
0.41833567620	1.26445215555	1.05226209533	1.51943062555	5121_3_SEMA6B_SE
0.37011799807	1.21281529577	1.01589571111	1.44790545460	5223_59_GCKR_GCK
0.45757440004	1.31748684311	1.09842532377	1.58023631144	5249_31_STK17B_DR
0.38196095740	1.23141386021	1.03496232055	1.46515488055	5261_13_ABL2_ABL2
0.37919308257	1.22483207811	1.02676638155	1.46110512244	5262_57_BCAR3_BC.
0.44259824816	1.32079896360	1.12061249899	1.55674678288	5268_49_MMP16_MM
0.35594970913	1.20959946559	1.02493465644	1.42753575444	5280_68_SLC25A18_
0.37103819748	1.21497084122	1.01857231700	1.44923842955	5315_22_TNNT2_Trop
0.37426572008	1.21809286400	1.02051469077	1.45392343577	5339_49_S100A9_calg
0.36135638814	1.21031637444	1.02061684622	1.43527488460	5347_59_CCNB1_Cyc
0.38942289883	1.23042679677	1.02562204111	1.47612867244	5468_67_IL17RC_IL_
0.36166847525	1.20307833711	1.00813151333	1.43572288533	5639_49_NETO1_NET
-0.055905177	0.80335657511	0.68248956399	0.94562879933	5648_28_CTRB2_CTR
0.34631034098	1.20686452599	1.03018773233	1.41384132	5651_50_PSG7_PSG7
0.33479855400	1.18445338588	1.00377132033	1.39765880400	5663_18_PF4V1_PF4V
-0.058096983	0.80209910760	0.68184752011	0.94355843411	5689_1_DEFB112_DE
0.38810270541	1.22607272577	1.01972155533	1.47418118299	5691_2_CRISPLD2_C
0.34219896749	1.19436494511	1.01311553133	1.40804042399	5719_66_FAM189A2_
0.35241116365	1.19737900122	1.00788980288	1.42249328100	5726_49_SPAG11A_S
0.39144979985	1.26117670677	1.07534394455	1.47912367344	5734_13_PVRL4_nect
0.41532364568	1.27796993255	1.07812348044	1.51486093955	5739_75_KRTDAP_KT
0.38750644287	1.22919261099	1.02552906155	1.47330244588	5745_64_PI15_PI15
0.39207886390	1.22659825455	1.01654591077	1.48005443	5754_76_INSL6_INSL
0.35633698057	1.20457427360	1.01604275377	1.42808870533	5756_66_PAPPA2_PA
0.36477296968	1.20255958600	1.00414012422	1.44018700499	5762_35_SPAG11B_S
0.33716811171	1.18599443922	1.00400310311	1.40097456411	5981_6_C21orf33_ES1
0.37255377816	1.22897058699	1.04060265099	1.45143653260	6020_52_UTS2_Urote
0.34173418002	1.19517539588	1.01496255344	1.40738613644	6210_100_IFNA5_IFN
0.34534449526	1.19497486044	1.01096548555	1.41247642733	6230_56_CER1_CER1
0.37017068321	1.21030252322	1.01163720333	1.44798173977	6245_4_PVRL2_Necti
0.42499013790	1.28887665811	1.08605506511	1.52957533477	6315_58_PLBD1_PLB
0.33339555953	1.18447842077	1.00522308633	1.39569927	6321_65_PCDHGA10_
0.36456513088	1.20692115922	1.01164741888	1.43988770922	6360_7_DEFB128_DE

0.3362419244	1.1891059307	1.0102132908	1.399677600	6362_6_C2orf40_AUC
0.4409967622	1.3082276384	1.1011441598	1.554255669	6416_8_GKN2_GKN2
0.4174366481	1.2949262323	1.1045862297	1.518065228	6420_4_SDHAF2_SDI
0.3333753826	1.1829498837	1.0026505647	1.395671110	6421_52_IFNA16_IFN
0.4265106112	1.2975959874	1.0991267631	1.531902782	6439_59_C14orf93_CI
0.3972560984	1.2311754030	1.0188581629	1.487736888	6448_36_SEMA3C_Se
0.3892071472	1.2346154131	1.0328395804	1.475810229	6456_17_PSG6_PSG6
0.3710296766	1.2249682008	1.0354127025	1.449226080	6468_37_TAC4_TKN
0.3584092333	1.2039322741	1.0128589298	1.431051134	6473_55_BPIFA1_PLU
0.3634626462	1.2140904807	1.0248310765	1.438301129	6504_65_LOXL2_Lys
0.4115312817	1.2724106653	1.0728248796	1.509126915	6517_14_GDF10_BMI
0.3435508913	1.1886612938	1.0021067461	1.409945274	6528_95_EXTL2_EXT
0.3359192086	1.1898887760	1.0118703669	1.399225974	6531_29_FAM162A_I
0.3522479695	1.1956298444	1.0051112747	1.422261157	6545_58_PRNP_PRIO
0.3638465488	1.2203911297	1.0350981594	1.438853403	6553_68_CFAP45_CC
0.3577638145	1.2048785574	1.0151067149	1.430127805	6558_5_COLEC10_CC
0.3428068332	1.1929434809	1.0100912765	1.408896583	6572_10_LRRTM4_LI
0.3995334871	1.2526545184	1.0523190435	1.491128904	6578_29_TNMD_TNM
0.3277979503	1.1791151414	1.0017321021	1.387908517	6587_6_LRFN5_LRFN
0.4204589347	1.2841166889	1.0829439649	1.522660196	6611_8_KDELC1_KD
0.3751805147	1.2219921827	1.0261197061	1.455254085	6624_94_WNT11_WN
0.3314807986	1.1838111510	1.0060152666	1.393029398	6633_43_UPK3BL_UI
0.3817693812	1.2325072686	1.0369997286	1.464874218	6913_189_HMGB2_H
0.3521015563	1.1944946392	1.0033504437	1.422052934	6921_24_B4GALT3_I
0.3695747563	1.2270842414	1.0405057399	1.447119105	6923_1_PLOD2_PLOI
0.4229675591	1.2836413840	1.0794311415	1.526484774	6951_26_ASIC4_ASIC
0.3849093019	1.2376044705	1.0423168368	1.469481036	6955_68_SNX1_SNX1
0.3565404286	1.2104741193	1.0258112936	1.428379276	6961_14_IGFL3_IGFL
0.3865371981	1.2460841160	1.0549302559	1.471875147	6962_5_HLA_DRB3_I
0.3944515344	1.2520605281	1.0566776556	1.483570280	6990_44_SDF2L1_SD
0.3932748766	1.2570323505	1.0663402456	1.481825652	6992_67_HBD_HBD
0.3804622740	1.2341279690	1.0410886779	1.462960721	6994_19_C3orf18_CC
0.3470875359	1.2017690321	1.0207133988	1.414940578	7018_10_TSR3_TSR3
0.3348822916	1.1907282021	1.0143498013	1.397775845	7045_4_BNIP3_BNIP
0.3687722040	1.2119733876	1.0158519865	1.445958182	7046_6_C20orf173_C
0.3641828351	1.2092491691	1.0159421982	1.439337351	7054_87_CLEC2D_CI
0.3978273836	1.2603490079	1.0671071517	1.488587053	7060_2_ERO1L_ERO
0.3864865278	1.2396362077	1.0440938536	1.471800568	7066_199_FAM209B_

0.39066780572	1.25262165111	1.06163433349	1.47796745955	7069_9_TSNARE1_TS
0.33773757266	1.18656838144	1.00440294822	1.40177259166	7071_23_MAN1B1_M
0.40311966667	1.26410648299	1.06781168855	1.49648596044	7081_2_BTN3A1_BT3
0.34819189036	1.20033781522	1.01715973466	1.41650403711	7082_2_B3GNT6_B3C
0.37028335487	1.21883760877	1.02584010833	1.44814489544	7089_42_SYT11_SYT
0.47055886167	1.34079196088	1.12295325322	1.60088861855	7090_17_GALNT1_GA
0.40079669877	1.26667720311	1.07465265088	1.49301370611	7099_33_FSTL5_FSTI
0.36927335997	1.21956674499	1.02810569422	1.44668301488	7124_18_IL21_IL_21
0.35659850555	1.20932797155	1.02381015544	1.42846223500	7132_55_C1QL4_C1Q
0.36116248794	1.20479809822	1.01152744660	1.43499661155	7142_5_CPN1_CBPN
0.39204807150	1.25910800611	1.07117803033	1.48000885599	7148_42_FAM173A_F
0.38864776992	1.24253656066	1.04672059877	1.47498492577	7189_55_CHST3_CHS
0.32445110794	1.17785389866	1.00294131444	1.38327117100	7203_125_RFNG_RFN
0.35140653877	1.20540937500	1.02248091166	1.42106492644	7242_14_VSTM4_CJ0
0.35370754874	1.21007743166	1.02804727422	1.42433857599	7247_1_GORAB_GOF
0.35377440866	1.19790490911	1.00740108866	1.42443381033	7249_307_BCL2L10_I
0.34681875259	1.19550144255	1.01036603544	1.41456031677	7251_64_C1QTNF3_C
0.41518291841	1.25809404900	1.04499584999	1.51464777233	7267_2_SPTLC2_LCE
0.34112441692	1.18795738433	1.00335188522	1.40652822588	7747_47_NDUFB11_N
0.32702142695	1.18096706877	1.00566184622	1.38683119	7754_11_JAG1_JAG1
0.34612950084	1.20671424411	1.03011745488	1.41358566455	7762_30_SNN_SNN
0.39523296534	1.22277720822	1.00704105099	1.48473004122	7779_86_CHST11_CH
0.36870178782	1.22555626988	1.03882253077	1.44585636744	7784_1_KNG1_Kininc
0.33804252841	1.18903917377	1.00828271288	1.40220013544	7789_182_PRDX4_PR
0.35869298423	1.21099105444	1.02448000444	1.43145725411	7797_11_CCDC167_C
0.36133218950	1.22066400333	1.03816814588	1.43524015344	7805_52_GOSR1_GOS
0.37888543808	1.23094953299	1.03736750722	1.46065569066	7821_6_C1D_C1D
0.37935128655	1.23534360388	1.04430022477	1.46133629333	7823_22_ST6GALNA(
0.38042583769	1.22465170233	1.02519938977	1.46290741799	7838_27_SMR3A_SM
0.37666206716	1.21865622555	1.01901403488	1.45741171888	7842_52_SERF1A_SE
0.38539835716	1.23446338666	1.03652563477	1.47019986944	7850_1_COX4I2_COX
0.35997560243	1.20940783866	1.02049325944	1.43329444522	7851_30_ANKRD46_A
0.34479787658	1.19546839955	1.01235396033	1.41170455222	7867_154_ST6GALNA
0.38747469033	1.23594063300	1.03685279088	1.47325566555	7881_244_PARP16_PA
0.37321372167	1.22054758622	1.02571043199	1.45239471499	7888_58_COA3_CCD
0.35181586646	1.19858105244	1.01051584200	1.42164672677	7891_45_UGT1A6_UC
0.36647227312	1.21789956799	1.02817269655	1.44263640011	7910_41_LRFN1_LRF
0.38381347344	1.24470463644	1.05546671233	1.46787161911	7917_17_CPD_CBPD

0.36976976062	1.21485035233	1.0196628610	1.4474013275	7934_11_NDRG4_ND
0.27165215783	1.14887004511	1.0059230945	1.3121305076	7956_11_MINOS1_M
0.42550442511	1.26229942760	1.0411913387	1.5303621781	7994_41_ERO1LB_EF
0.40707115157	1.25606545891	1.0501124102	1.5024110007	8008_28_HRK_HRK
0.36537757211	1.20988513211	1.0157967438	1.4410580087	8011_96_PAPL_PAPL
0.34102464190	1.18984521511	1.0066437855	1.4063878965	8017_23_FOXRED1_F
0.37767794934	1.23325583571	1.0425164321	1.4588930297	8024_64_TPST2_TPST
0.35495640669	1.19597825260	1.0029769595	1.4261184837	8055_33_DGCR2_IDD
0.33005175020	1.18525549911	1.0099137940	1.3910401132	8066_38_SYT9_SYT9
-0.030219967	0.80113913141	0.6615158513	0.9702320914	8080_24_MSMP_PSM
0.37801798527	1.23809692911	1.0503599836	1.4593891901	8094_20_CISD2_CISD
0.34313542840	1.19709463311	1.0167990796	1.4093596163	8107_12_TMEM234_T
0.34649828742	1.20227990688	1.0221835411	1.4141070720	8249_124_ALK_ALK
0.36746097283	1.22695172241	1.0424822675	1.4440634397	8261_51_STOM_STO
0.35581866864	1.20976879931	1.0253559946	1.4273487017	8321_27_ZNF843_ZN
0.37480848667	1.23151598060	1.0425642915	1.4547127910	8336_267_GRAMD1C
0.40424362181	1.27689465931	1.0883018482	1.4981688891	8340_9_DEFB110_DE
0.47643747608	1.33475245641	1.1063366273	1.6103273415	8351_17_PRSS57_PRS
0.37779177954	1.21768535100	1.0162423227	1.4590591052	8357_43_SULT4A1_S
0.35718808678	1.20194817571	1.0107567962	1.4293046778	8363_18_KIAA1324L_
-0.002527575	0.84326153431	0.7128896221	0.9974756163	8364_74_UST_UST
0.39750239396	1.25216279991	1.0536308992	1.4881033564	8367_142_VSIG1_VSI
0.39970120669	1.23968167411	1.0304628371	1.4913790170	8378_3_LDOC1_LDO
0.34846660242	1.1993519275	1.01521062	1.4168932213	8386_11_PATE1_PAT
0.34139020712	1.19838781191	1.0207770181	1.4069021169	8388_24_SPAST_Spas
0.35923543009	1.21642376241	1.0331320290	1.4322339528	8389_8_LRRC74A_CI
0.39045867911	1.21749636171	1.0031394139	1.4776584095	8429_16_AXIN2_AXI
0.45515276630	1.30964656321	1.0880225096	1.5764141876	8587_21_SPINK14_IS
0.35579358695	1.20168879491	1.0117304748	1.4273129018	8646_61_LRRTM4_LI
0.34443561935	1.19080726281	1.0048389492	1.4111932447	8653_132_DNAJC4_D
0.367223076	1.21177162271	1.0170881640	1.4437199425	8654_13_ADAM10_A
0.36814572833	1.22190951701	1.0332238832	1.4450526087	8664_36_PKDCC_PKI
0.42275960228	1.25686725121	1.0350865335	1.5261673647	8671_378_TRAPPC5_
0.34152112433	1.18739765251	1.0020090227	1.4070863167	8696_15_GAL3ST1_G
0.37997466365	1.22354541061	1.0238098065	1.4622475409	8700_325_GALNT11_
0.41370335391	1.26586994181	1.0595198352	1.5124084102	8775_61_FAM24B_FA
0.34823187022	1.19130295301	1.0018651202	1.4165606699	8776_10_ERLIN1_ER
0.30741983842	1.16834640251	1.0037660714	1.3599117914	8780_2_PCDH10_PCI

0.3608122888(	1.2178449362	1.0339158742	1.434494165(	8785_1_ARMC5_ARM
0.3348500789	1.1902478481	1.0135642138	1.3977308202	8800_14_TTC17_TTC
-0.015178076	0.8262999724	0.6932138506	0.9849365299	8841_65_CILP2_CILP
0.3409593541(	1.1944054187	1.0144409311	1.4062960795	8842_16_GRAMD1C_
0.3485522071	1.1963327393	1.0100193073	1.4170145193	8863_3_PIK3R1_P85A
0.3643327450	1.2247944490	1.0420743795	1.4395531386	8878_48_YTHDC1_Y
-0.119417858	0.7186419031	0.5819525696	0.8874369011	8885_6_CACNA2D3_
0.4203985541	1.2760407738	1.0694299221	1.5225682606	8889_5_CCPG1_CCP(
0.3505996402(	1.2052292241	1.0230004352	1.4199187337	8894_80_HNRNPAB_
0.3631349876	1.2127464770	1.0228984537	1.4378299353	8907_11_UGT2A1_UI
0.3738607049	1.2262541530	1.0346544770	1.4533346940	8913_22_SIGLEC11_S
0.3268278530	1.1810101999	1.0059300080	1.3865627640	8927_6_C7orf73_CG0
0.3497095427	1.1926807100	1.0027010406	1.4186554300	8929_7_BDP1_BDP1
0.3402728233	1.1983113134	1.0217877922	1.4053309453	8941_4_NLGN3_NLG
0.3995437944(	1.2524771164	1.0520101605	1.4911442741	8946_38_NR1H4_NR1
0.3973101137(	1.2612663102	1.0692124346	1.4878172511	8957_72_ERLEC1_XT
0.4099374079	1.2777780159	1.0836206424	1.5067234732	8998_15_HS3ST4_HS
0.3642169976(	1.2086686168	1.01493227	1.4393865237	9012_1_PRPF6_PRP6
0.3647031604	1.2070403346	1.0117075604	1.4400864700	9016_12_NR1H2_NR1
0.3648360355	1.21582575	1.0263521547	1.4402778344	9022_49_DEFB132_D
0.3726401846	1.2254100539	1.0344923954	1.4515619516	9055_81_MYZAP_MY
0.3463856211	1.1931568997	1.0068429887	1.4139477588	9059_14_RAPGEF5_F
0.3206967722	1.1781654511	1.0072464094	1.3780876430	9068_17_JAKMIP3_JI
0.3718858028	1.2200250608	1.0261941897	1.4504673326	9082_25_SEL1L2_SE
0.3612431416	1.2049161506	1.0116440890	1.4351123540	9107_59_ARMC10_A
0.3530768571	1.19598734	1.0048791504	1.4234405408	9123_18_DOS_DOS
0.3867324113	1.2292709665	1.0264540119	1.4721625045	9176_3_MUC1_MUC1
0.3834511318	1.2426806577	1.0524182403	1.4673398445	9221_6_ITPRIPL1_IPI
0.3952445797(	1.2597755821	1.0688920146	1.4847472856	9241_40_SIRPG_SIRI
-0.006619398	0.8391825333	0.7089043476	0.9934024621	9296_15_PTPRD_PTP
0.3709596986	1.2207511264	1.0283679128	1.4491246704	9310_2_CD8B_CD8B
0.3429694316	1.1926618320	1.0094502280	1.4091256865	9372_157_MPZL2_MI
0.3454827816	1.2021122381	1.0229367264	1.4126717671	9374_24_NNAT_NNA
0.3707444612	1.2221853927	1.0310076885	1.4488128	9412_52_ACVR2B_A(
0.4026369278(	1.2669315914	1.0731077596	1.4957637228	9457_3_CAV2_CAV2
0.3451506015	1.1918687272	1.0059116727	1.4122025835	9523_34_ITM2C_ITM
0.4004954972	1.2524480446	1.0509606454	1.4925640758	9547_29_EMID1_EMI
0.3302724194	1.1820356636	1.00421261	1.3913471068	9576_58_MNX1_MN

0.36739182018	1.2166800210	1.0251714737	1.4439635823	9581_4_PRPF6_PRP6
0.36297896502	1.2108547729	1.0198689139	1.4376056189	9609_25_ZNF275_ZN
0.33674231104	1.1851148515	1.0029413883	1.4003781552	9748_31_GSTM3_GST
0.38577085173	1.2433553450	1.0511201925	1.4707476129	9786_310_IER3_IEX1
0.38727866352	1.2504518389	1.0615512171	1.4729668962	9789_52_SYNE2_Nesp
0.37504293395	1.2221206208	1.0264766327	1.4550538844	9796_4_CEL_CEL
0.35432310122	1.2085755048	1.0248658167	1.4252156010	9823_2_DHFR_DYR
0.36457311047	1.2018458396	1.0031489865	1.4398991990	9835_16_ALDH1A3_A
0.34232393886	1.1901793793	1.0059014760	1.4082163996	9872_23_CTNNA2_CT
0.37281618611	1.2317472032	1.0450357733	1.4518174510	9896_21_FOXG1_FOX
0.37073839487	1.2303293515	1.0447999202	1.4488040092	9898_161_ARID1A_A
0.32339414338	1.1776992067	1.0037382471	1.3818098748	9930_48_GABBR2_G
0.36137105886	1.2087766084	1.0180067030	1.4352959413	9941_70_PCDHB1_PC
0.39243146915	1.24226516	1.0423121264	1.4805763966	9954_2_NPIPB3_NPP
0.40900853476	1.2677063754	1.0675966423	1.5053245680	9964_10_MYZAP_MY
0.31604186982	1.1711215340	0.9998818686	1.3716876867	10022_207_POLH_PO
0.332661503	1.1812976096	1.0005656635	1.3946751257	10627_87_APLP2_AP
0.32808793069	1.1783380967	1.0001221831	1.3883110420	10975_59_MUCL1_M
0.32294151707	1.1748223367	0.9992926001	1.3811845728	11122_97_SPG21_SPG
0.33976059294	1.1852283581	1.0001103397	1.4046112764	11544_39_PHF3_PHF
0.36676254072	1.2012016729	0.9998823659	1.4430552115	11989_35_EMC1_EM
0.33988884726	1.1854823815	1.0004107666	1.4047914354	12482_5_BPTF_BPTF
0.32933037608	1.1786664849	0.9994371862	1.3900370147	12620_3_SEPT11_Sep
0.39072784324	1.2160038490	1.0004121394	1.4780561956	12634_79_BCAR3_BC
0.32390391089	1.17585182	1.0000817719	1.3825144561	12644_63_ADSS_PUR
0.34391389295	1.1873803510	0.9995851827	1.4104571799	12690_33_SEPT10_SE
0.33544476151	1.1832138879	1.0010245029	1.3985622735	13042_7_KCNA10_KC
0.31825011325	1.1722750696	0.9996426800	1.3747200539	13230_174_IGHG2_Ig
0.34306622219	1.1869146003	0.9996481742	1.4092620832	13423_94_FAM213A_
0.36648078711	1.2013884446	1.0004751761	1.4426486828	13515_8_RPRD1A_RP
0.33962806801	1.1859711489	1.0014969992	1.4044251427	13540_1_GPR26_GPR
0.35153689990	1.1917924437	0.9993801505	1.4212501901	13680_3_NAB2_NAB
0.34857274558	1.1899939696	0.9993239622	1.4170436228	13933_276_NAB1_NA
0.33899383642	1.1844505982	0.9995643323	1.4035346943	14135_3_RXFP1_RXF
0.34747167515	1.1906334217	1.0015003546	1.4154842166	14186_13_RFFL_RFF
0.32480980603	1.1758620331	0.9991935669	1.3837674367	14314_6_PPIL2_PPIL
0.40996861611	1.2269574689	0.9991067880	1.5067704960	14337_1_TRAPPC3_T
0.32682077627	1.1777888591	1.0004569930	1.3865529517	14708_59_C8G_CO8C

0.33242241002	1.1805130262	0.9994759512	1.3943417080	16587_1_CSN2_Beta_
0.32844025216	1.1789610438	1.0008272485	1.3888002600	19219_71_NUTF2_NT
0.34657228052	1.1898784691	1.0011307084	1.4142117100	2943_5_CYP3A4_Cyto
0.32372788272	1.1758613662	1.0002740681	1.3822711160	3376_49_IL17RD_IL_
0.33724575719	1.1844437353	1.0013015746	1.4010833477	3472_40_BIRC5_Surv
0.34716619719	1.1893074486	0.9995762161	1.4150518834	4163_5_H2AFZ_Histo
0.33578022863	1.1834063085	1.0010142496	1.3990315239	5335_73_ANXA6_ann
0.42854615860	1.2392519444	1.0004698056	1.5350242187	5491_12_SPOCK2_Te
0.34048411590	1.1856625988	1.0001194381	1.4056279127	5611_56_DEFB108B_
0.32209460103	1.1742819844	0.9992194711	1.3800153206	5927_4_WISP3_WISP
0.32902340750	1.1794816956	1.0011274295	1.3896103825	6969_14_WFDC5_WF
0.35712789774	1.1953525586	0.9997544724	1.4292186520	7763_25_CDH11_Cad
0.33007098895	1.1800832426	1.0010995764	1.3910668753	7796_10_CD79A_CD7
0.34234808391	1.1866299776	0.9998865985	1.4082504015	8269_327_ARSK_ARS
0.33059988929	1.1803654133	1.0010487860	1.3918028056	8406_17_IGF1_IGF_I
0.36159947378	1.1984843602	1.0005161100	1.4356238218	8686_342_C11orf73_H
0.34150276424	1.1857561610	0.9992588738	1.4070604827	8754_5_NMU_NMU
0.34006407153	1.1856782780	1.0005660833	1.4050376105	9049_2_SLC16A3_MC
0.33904709989	1.1844717492	0.9995467908	1.4036094535	9271_101_STIM1_STI
0.34630494847	1.1884396814	0.9989780834	1.4138336965	7751_121_SFRP2_SAI
0.32591239141	1.1763445667	0.9989118121	1.3852939999	12030_82_DES_Desm
0.34517850826	1.1876208225	0.9987262976	1.4122419940	13926_1_RMI1_RMI1
0.38673595958	1.2124944819	0.9986245729	1.4721677281	18398_1_AKR1D1_AI
0.34047835224	1.1848903070	0.9988227461	1.4056198111	8399_6_STK17B_DRA
0.32513604596	1.1757495445	0.9986765395	1.3842189505	12991_49_CUL9_CUI
0.32144568818	1.1736103286	0.9987246231	1.3791201014	6382_17_MANBA_MA
0.33567206564	1.1818393218	0.9984730457	1.3988802087	11109_56_SVEP1_SV
0.32879819438	1.1776895494	0.9983122517	1.3892974592	13672_3_HSPA6_HSP
0.31620315381	1.1703669260	0.9984326989	1.3719089358	2732_58_NANOG_NA
0.33580554528	1.1817809900	0.9982412316	1.3990669431	7993_23_ASTL_ASTI
0.36063067999	1.1965592530	0.9982711154	1.4342336718	8933_84_MEF2C_ME
0.35559323782	1.1935015886	0.9981913953	1.4270269696	9783_75_DNAJC11_D
0.33360092583	1.1804277750	0.9981545671	1.3959859304	6527_1_TRIL_TRIL
0.34654028320	1.1879952554	0.9979961815	1.4141664598	15678_71_DKK2_DKI
0.32409766654	1.1747964803	0.9980940008	1.3827823520	17453_34_CP_Cerulop
0.34896084630	1.1892702712	0.9977215564	1.4175936852	12704_26_SH2D3C_S
0.35007818799	1.1898804873	0.9976303666	1.4191785069	5024_67_RB1_Rb
0.35158708180	1.1907575059	0.9975951430	1.4213215130	9955_40_RNF148_RN

0.34243146738	1.18526569511	0.99750557844	1.40836783122	13536_56_POLI_POLI
0.33726166813	1.18183122577	0.99687347322	1.40110564044	11645_9_P4HA1_P4H
0.32999376125	1.17762232933	0.99700559200	1.39095945066	13430_50_SLC14A1_U
0.33795437525	1.18226070599	0.99690733311	1.40207653255	16748_1_BMP3_BMP
0.37322193816	1.20335002488	0.99700127633	1.45240664855	16749_79_GDF10_BM
0.32895849776	1.17699935222	0.99698261955	1.38952018622	17209_27_SULT2B1_
0.35390817457	1.19145856522	0.99645460855	1.42462436377	18935_14_TLR5_TLR
0.34715448253	1.18754729566	0.99663137211	1.41503530677	19115_13_CMBL_CM
0.33862723379	1.18251112433	0.99665885800	1.40302024911	2524_56_HMGB1_HM
0.35774093980	1.19403777555	0.99694504066	1.43009509177	3873_51_TPO_TMA
0.32846628610	1.17687520044	0.99726304766	1.38883641644	7228_2_ST6GALNAC
0.33863571903	1.18257723600	0.99676184533	1.40303215411	7925_18_SMCO2_CL0
0.32552020399	1.17499379444	0.99701000744	1.38475081155	8784_7_FAM175A_F1
0.35514290560	1.19251107377	0.99698411111	1.42638447800	9290_8_RMDN3_RMI
0.36308867902	1.19699372322	0.99654367344	1.43776335300	9487_60_GHRH_Grov
0.33616328202	1.18095394688	0.99648798233	1.39956753033	19596_18_BRF1_BRF
0.52682624513	1.29783943311	0.99459025511	1.69354886133	8778_3_NOG_Noggin
0.31874493013	1.17078657400	0.99661243755	1.37540045699	11356_19_DGCR14_D
0.33387166276	1.17951252677	0.99633754088	1.39636392655	10425_3_B4GALT5_B
0.32093851408	1.17201033299	0.99650861022	1.37842082488	15329_167_ROBLD3_
0.33423359499	1.17966754100	0.99623880399	1.39686940711	7878_2_SEC61B_SC6
0.34166880609	1.18398128944	0.99610426900	1.40729413300	9111_40_JPH4_JPH4
0.31592262037	1.16885333077	0.99613130055	1.37152412355	9916_146_LRRC4B_L
0.32223915452	1.17237679877	0.99583581999	1.38021482111	12842_43_STX10_ST
0.31833360896	1.17014925955	0.99593729211	1.37483484199	6079_59_ACPL2_ACI
0.00443152154	0.84675088500	0.71381674755	1.00444135522	6470_19_FBLN1_fibu
0.33880058425	1.18194232655	0.99552769577	1.40326348444	8635_283_ZPBP_ZPB
0.33524413432	1.17985694833	0.99555218844	1.39828171211	9032_30_ROS1_ROS1
0.32676787491	1.17494400100	0.99568244811	1.38647960311	9942_2_NXT1_NXT1
0.33019884796	1.17688651100	0.99555585944	1.39124474711	10550_37_BMPR1B_I
0.33199820388	1.17775226100	0.99522873166	1.39375034511	11587_5_MGA_MGA
0.32141765212	1.17168013311	0.99547009899	1.37908143699	5082_51_IL1RAPL2_I
0.32818242160	1.17561436100	0.99540988800	1.38844223100	5634_39_POFUT1_OI
0.32623336325	1.17435902933	0.99522306633	1.38573871166	7183_102_C19orf80_T
0.31608013106	1.16853186611	0.99542664999	1.37174017022	8485_7_KEAP1_KEA
0.31723053050	1.16914974233	0.99533392699	1.37331912733	9544_24_C7orf69_CG
0.31676693244	1.16840913544	0.99453427988	1.37268260688	10553_8_TOR1AIP2_
0.33728274281	1.18000094400	0.99376723900	1.40113516866	11096_57_N6AMT1_I

0.3534072110	1.189524079	0.993719184	1.423910857	11185_145_GCH1_GC
0.3358251729	1.179693775	0.994698713	1.399094403	11286_78_VIMP_SEL
0.3432778619	1.184113115	0.994724240	1.409560370	11825_27_PPARGC1
0.3595085487	1.193412997	0.994143204	1.432625176	12343_14_ACAP2_CE
0.3408641508	1.182320355	0.994111078	1.406162201	12702_13_PELI2_PEL
0.3451623047	1.184744832	0.993911147	1.412219110	12963_1_GPR107_GP
0.3243841639	1.172468114	0.993856836	1.383178572	13452_113_SMIM13_
0.3600726857	1.193201574	0.993230518	1.433433600	18483_36_RABIF_MS
0.3417721812	1.183279402	0.994820754	1.407439619	19482_11_HDHD3_H
0.3230716308	1.171996313	0.994361417	1.381364295	19555_1_SUMO2_SU
0.3325512694	1.177342834	0.993987009	1.394521393	5201_50_PDE9A_PD
0.3730360838	1.201270277	0.993742697	1.452136737	6289_78_ARHGAP36_
0.3376142965	1.180148366	0.993686049	1.401599797	7231_37_TMPRSS11
0.3430590644	1.183959758	0.994684211	1.409251996	7753_21_HTATIP2_H
0.3560227297	1.191325454	0.994127609	1.427639997	7860_9_HERC4_HER
0.3541387026	1.189841707	0.993522923	1.424952817	7928_183_TPST1_TP
0.3438649297	1.184004092	0.993957387	1.410388121	8071_114_PCDHB7_P
0.3150904023	1.167327935	0.994360203	1.370383191	8347_222_DEFB129_
0.3230270596	1.171715051	0.993928509	1.381302727	9040_144_EPDR1_EP
0.3470374812	1.186160553	0.994421466	1.414869755	9363_11_CT55_CX04
0.3429509347	1.183497525	0.994015164	1.409099622	9504_19_RAB27A_R
0.3425583944	1.183449175	0.994324183	1.408546602	9936_27_LNX1_LNX
0.3137728442	1.166186863	0.993725593	1.368578820	12581_39_IMPA2_IM
0.3338103466	1.177748465	0.993420465	1.396278309	12677_164_FLII_FLII
0.3348171692	1.178230338	0.993233029	1.397684822	12960_9_GCK_HXK4
0.3240795197	1.172073467	0.993490508	1.382757259	2723_9_IL37_IL_1F7
0.3238051570	1.172038874	0.993704463	1.382377934	4436_1_ENTPD3_EN
0.3161016781	1.16750897	0.993663263	1.371769727	4545_53_DNAJC19_D
0.3310841937	1.176187134	0.993492998	1.392477025	8681_93_PGRMC2_P
0.3215203542	1.170291354	0.993009669	1.379223078	4476_22_ZAP70_ZAP
0.3149476181	1.166463142	0.993029222	1.370187536	18243_9_CMPK1_Cyt
0.3409198346	1.181276233	0.992300772	1.406240504	10012_5_SPDEF_SPD
0.3415100518	1.181587008	0.992237150	1.407070736	13043_157_ATAD2_
0.3137778121	1.165022268	0.991736919	1.368585619	10949_59_RPLP2_RL
0.3440431063	1.182008462	0.990433106	1.410639441	11690_47_ANAPC7_
0.3323732641	1.175176438	0.990508658	1.394273183	11967_23_CRABP1_
0.3140425415	1.164963176	0.991373836	1.368947972	12355_223_MRRF_R
0.3139968292	1.165333679	0.992049873	1.368885396	12391_27_PDZD7_PD

0.3443953450	1.182527097	0.990953337	1.411136410	12445_50_ANKRD27_
0.3258958436	1.172413693	0.992263457	1.385271076	12518_289_PBRM1_P
0.3269659404	1.172709560	0.991702545	1.386754244	12655_30_NAPB_SN
0.3320894991	1.175560163	0.991436913	1.393877593	12892_10_SYTL1_SY
0.3281733121	1.173712416	0.992200722	1.388429583	13123_3_FLRT3_FLR
0.3289299665	1.174159784	0.992206193	1.389480542	13473_55_ITPKA_IP3
0.3426848631	1.181233415	0.990479070	1.408724750	13659_36_ATP13A1_
0.3677313903	1.195808551	0.989964442	1.444453992	13665_35_PPP2R3A_
0.3357900993	1.177281384	0.990669441	1.399045333	14036_116_MPDZ_M
0.3158512249	1.165765521	0.990945954	1.371426206	15567_2_CD247_CD3
0.3246808074	1.170877658	0.990868348	1.383588944	17387_27_NFKBIB_I
0.3248511856	1.171660853	0.992025331	1.383824697	17397_8_HSD17B11_
0.3337953159	1.176621041	0.991534334	1.396257322	17813_21_BPHL_BP
0.3168156003	1.166692497	0.991565809	1.372749414	18190_15_CARD18_I
0.3423041588	1.180840832	0.990197709	1.408188545	18197_97_CKMT2_K
0.3458988715	1.183258047	0.990688137	1.413259687	18267_74_CARD17_C
0.3363591610	1.177441345	0.990374924	1.399841703	19180_38_GULP1_GU
0.3600445175	1.191233043	0.989983865	1.433393224	19572_10_CEBPG_CE
0.3362867947	1.177468009	0.990491457	1.399740405	3423_59_CMA1_Chym
0.3546086198	1.188677875	0.991114413	1.425622584	4563_61_PLCG1_PLC
0.3089729686	1.162431064	0.992085630	1.362025552	5246_64_PDE2A_cGM
0.3536866458	1.187615663	0.990256439	1.424308803	6390_18_NPS_NPS
0.3159213175	1.165835987	0.990996291	1.371522336	7201_5_ISCU_ISCU
0.3409504871	1.180701602	0.991305214	1.406283610	7736_28_OPALIN_OP
0.3292555221	1.173708334	0.991120639	1.389932968	7803_4_CHST1_CHST
0.3381946749	1.178807549	0.990854157	1.402413491	7960_53_POTEG_POT
0.3643779136	1.194194311	0.990609933	1.439618162	7991_54_IQCF1_IQC
0.3253285237	1.171517605	0.991309472	1.384485407	9038_12_JTB_JTB
0.3285973912	1.173461932	0.991356771	1.389018511	9121_28_FUT2_FUT2
0.3201183165	1.168352470	0.991110651	1.377290711	9963_19_PCDHB10_P
0.3314514010	1.174457979	0.990210326	1.392988446	15439_21_RND3_RN
0.3148010432	1.164995676	0.990677436	1.369986715	5627_53_GNRH1_GO
0.3246933673	1.170572407	0.990339332	1.383606322	8959_61_ADAM17_A
0.3473055238	1.183539300	0.989765916	1.415249051	9802_27_PNPLA2_PL
0.3793718488	1.201968846	0.988615288	1.461366342	10666_7_GNPTG_GN
0.3363195892	1.177097192	0.989835227	1.399786310	7244_16_IGFLR1_TM
0.3507000973	1.185191131	0.989167114	1.420061381	11457_53_GALE_GA
0.3331284208	1.175172151	0.989753730	1.395326475	2783_18_CCL3L1_LD

0.34069689702	1.17937883838	0.98933615355	1.40592703560	5714_88_IFNA6_IFNA
0.32031277125	1.16758801755	0.98962165422	1.37755855760	10974_20_SPINK7_IS
0.37252354943	1.19738638559	0.98783340888	1.45139265822	3616_3_GNS_GNS
0.34729642229	1.18286600760	0.98864911800	1.41523617077	6612_90_BRICD5_CP
0.31369024365	1.16372090722	0.98960921777	1.36846577999	12623_84_JARID2_JA
0.32390299475	1.16950848344	0.98932155088	1.38251318955	9543_131_COG8_COG
0.32508819572	1.17001635433	0.98900811499	1.38415271699	8470_213_RNASEH1_
0.30600847630	1.15928289633	0.98964871322	1.35799381722	9503_1_NMS_NMS
0.31894729298	1.16647801522	0.98909058222	1.37567881500	6369_82_DEAF1_DEA
0.32681066114	1.17088964860	0.98878043960	1.38653892660	7233_73_P2RX6_P2R
0.31833610014	1.16607584188	0.98901296377	1.37483826699	11616_9_HSF1_HSF1
0.33067428285	1.17292937260	0.98840221011	1.39190635077	10882_12_DSC1_Desr
0.31552509488	1.16444091160	0.98901779000	1.37097901600	11243_90_ADGRF1_C
0.32295862018	1.16859251260	0.98870573244	1.38120819555	17725_37_USH1C_US
0.30768759348	1.15996033533	0.98914339111	1.36027596344	7108_7_SMIM24_CSC
0.32904085503	1.17186566744	0.98822317399	1.38963462799	11134_30_MTRF1L_F
0.31761131878	1.16545337644	0.98867366388	1.37384217077	14705_1_FIGF_VEGF
0.34125026340	1.17882886599	0.98786686233	1.40670524360	18339_207_PSMB3_P
0.33555733551	1.17549983510	0.98790332211	1.39871972422	11325_8_BTG2_BTG2
0.36423342247	1.19177697988	0.98674610160	1.43941016560	13565_2_RBL2_p130
0.31793796777	1.16546025199	0.98836242877	1.37429100822	19189_2_USP14_UBP
0.32894497419	1.17163408710	0.98792735211	1.38950139500	9913_4_RRBP1_RRBI
0.32294607956	1.16816385933	0.98799291799	1.38119087444	14082_56_TLN2_TLN
0.32557069167	1.16958250822	0.98779807200	1.38482072611	10023_32_VDR_VDR
0.35427994197	1.18577531633	0.98660426244	1.42515409100	12514_16_METTL1_M
0.32450526661	1.16885690933	0.98762448900	1.38334608911	18328_36_PYCR2_P5
0.30891229744	1.15997436	0.98795660855	1.36194291933	18892_48_GPC4_GPC
0.31041127239	1.16080581677	0.98789149988	1.36398596855	8060_7_NRG2_NRG2
0.35366041617	1.18512383860	0.98613120277	1.42427144488	7738_299_STX2_STX
0.31998677218	1.16604161333	0.98732380877	1.37710954800	19206_20_NUDT1_8C
0.31629710659	1.16373210022	0.98705178899	1.37203783655	12594_5_GCA_GRAN
0.31577832568	1.16344826633	0.98708231111	1.37132623411	13743_56_CUL4B_CU
0.31564974403	1.16344066977	0.98719634833	1.37114991800	18166_4_RHOB_RHO
0.35892873164	1.18774619510	0.98529556544	1.43179475622	5586_66_MINPP1_MI
0.34475833233	1.17971415860	0.98588655088	1.41164872855	6948_82_FIG4_FIG4
0.40212536660	1.21272550255	0.98374874922	1.49499874399	9199_6_UBE2G2_UB2
0.31915160253	1.16545833144	0.98716039200	1.37595990811	9379_248_SMIM10_C
0.31882152560	1.16522136044	0.98708475711	1.37550581044	9767_22_RIPPLY1_RI

0.31353342638	1.16145443759	0.98591283011	1.36825119760	10485_56_MAGEA4_
0.31170816640	1.16057172299	0.98621325011	1.36575606130	10561_5_PGLYRP3_P
0.33211015472	1.17191369659	0.98527542911	1.39390638540	12389_4_ORC6L_OR(
0.32156729240	1.16619009880	0.98601562940	1.37928781850	12438_127_MPG_3M(
0.74883903195	1.43098174650	0.96839274820	2.11454367330	12490_92_LAMTOR3_
0.30311033209	1.15607435600	0.98703463290	1.35406385290	12564_9_NIP7_NIP7
0.31109340483	1.16047638720	0.98665760350	1.36491670500	13573_5_ARPC3_p21_
0.35961691263	1.18781055870	0.98472445210	1.43278042930	13689_2_ODC1_Ornit
0.32211622400	1.16646715710	0.98594282780	1.38004516110	14067_6_PKP2_PKP2
0.35044880103	1.18259881680	0.98509224430	1.41970457050	14121_24_TNFRSF10
0.31359029009	1.16143195180	0.98581859690	1.36832900370	14309_8_HNRNPH1_
0.33703174508	1.17483008430	0.98532406790	1.40078353100	15361_37_ANKRD1_
0.31214676743	1.16084564340	0.98624617760	1.36635521470	17154_2_PTGES3_TE
0.32473723940	1.16809767920	0.98611310640	1.38366702500	18237_29_NRIP3_NR
0.31631872302	1.16369213180	0.98696265460	1.37206749540	18422_41_MVD_ERG
0.32203985860	1.16614123620	0.98546719580	1.37993977740	19377_14_OLFM2_N(
0.31110238732	1.16007035390	0.98595843460	1.36492896540	2752_62_GDF5_BMP_
0.30939654630	1.15964403810	0.98691599210	1.36260259840	3473_78_MPL_Throm
0.31444455505	1.16204532710	0.98601745270	1.36949841860	4472_5_TPM2_Tropor
0.31407175709	1.16187043910	0.98608822660	1.36898796760	5021_13_PPA1_PPase
0.34319680949	1.17805788880	0.98465657030	1.40944612700	6027_31_RLN3_REL3
0.32768995196	1.16933437060	0.98528867840	1.38775863370	6354_13_HEPHL1_HI
0.32613133454	1.16905107130	0.98634745760	1.38559733370	7038_45_ST8SIA4_SI
0.32129792293	1.16627027810	0.98641689250	1.37891633040	8810_26_HEPACAM_
0.31994033405	1.16533231820	0.98616880430	1.37704559910	9037_1_STX18_STX1
0.34896216036	1.18163588440	0.98495185400	1.41759554800	9332_6_DEFB136_DE
0.32697589655	1.16933195890	0.98598841340	1.38676805080	9994_217_ATP4B_AT
0.33717852059	1.17445900240	0.98455719760	1.40098914680	13493_5_GRID1_GRI
0.33237179054	1.17181338960	0.98484906670	1.39427112890	13512_28_HNRNPR_
0.32303384868	1.16657273780	0.98521684350	1.38131210570	5649_83_PSG4_PSG4
0.31042572925	1.15956122640	0.98575999350	1.36400568760	7121_2_SYT7_SYT7
0.31500138956	1.16200075200	0.98539295600	1.37026121500	18195_3_ORAOV1_O
0.32792898613	1.16923474370	0.98488534370	1.38809039510	7930_3_EXOSC1_EX(
0.32528611267	1.16773226140	0.98495546410	1.38442669130	9110_2_SYT17_SYT1
0.30959542910	1.15890418520	0.98546107820	1.36287362350	11223_1_TMEM154_
0.30949830248	1.15886344400	0.98548750420	1.36274125870	12531_5_ERVV_1_EI
0.33249657927	1.17167015870	0.98448546470	1.39444512910	19311_15_CRYGS_CI
0.31497801611	1.16186067030	0.98517841350	1.37022918760	8000_17_KIR2DL5A_

0.35043835691	1.18157039111	0.98338992455	1.41968974300	13729_26_SFXN5_SF
0.32735330232	1.16867937560	0.98451656290	1.38729152390	7076_17_ECEL1_ECE
0.31703243014	1.16284577870	0.98482444141	1.37304709930	11592_1_ELAVL1_EL
0.33754730959	1.17412887210	0.98364095144	1.40150591140	12373_73_TRA2B_TR
0.30664044343	1.15706788150	0.98524768760	1.35885229590	13936_24_PGK2_PGK
0.32875650090	1.16933913110	0.98424639390	1.38923953580	5743_82_CARTPT_CA
0.32273951499	1.16588507530	0.98434535280	1.38090559880	6530_63_PROL1_PRO
0.32994038721	1.16993313190	0.98408087300	1.39088521140	9226_6_TMUB2_TMU
0.01466797381	0.86569792810	0.73852046790	1.01477607640	9590_10_COX7A2L_C
0.31010591050	1.15880324920	0.98478658250	1.36356952280	12678_66_SNRPA_SN
0.31979590156	1.16416346940	0.98433366660	1.37684672330	6237_11_FGFRL1_FG
0.35084090500	1.18149973950	0.98287659020	1.42026135140	7758_217_RNF215_R
0.33372496884	1.17164524640	0.98323506250	1.39615910350	10933_107_CALN1_C
0.32165186128	1.16499769610	0.98391709120	1.37940446820	13507_51_TRADD_TR
0.33600759368	1.17285502200	0.98302014910	1.39934965090	13509_5_SCAMP5_SC
0.32837025978	1.16862267100	0.98342042190	1.38870305800	15653_9_COL10A1_C
0.30733806140	1.15712118050	0.98465130830	1.35980058640	18910_45_LIN7A_LIN
0.32378727557	1.16610070170	0.98367828960	1.38235321550	19108_50_MECP2_ME
0.33315960190	1.17139331030	0.98336806920	1.39536998430	6273_58_LEPRE1_P3
0.34555650295	1.17825129560	0.98265839690	1.41277591490	7197_2_DNAJC15_DJ
0.31341895803	1.16030456	0.98407426140	1.36809458510	11715_1_POU2F1_PO
0.31920086434	1.16347574820	0.98375623140	1.37602769200	9026_40_BTNL8_BTN
0.32352351945	1.16581804830	0.98346083560	1.38198865950	9735_44_CD96_TACT
0.32254670673	1.16526702520	0.98349160910	1.38063937450	9974_8_DLL3_DLL3
0.31898375153	1.16315294270	0.98342391300	1.37572897120	15358_28_SCG5_7B2
0.33938701272	1.17443409100	0.98234353600	1.40408663940	17703_40_GMNN_GE
0.32754840812	1.16784748770	0.98292367430	1.38756221890	19170_25_POLE3_DP
0.32972727602	1.16917821210	0.98302076240	1.39058882980	19612_3_CD81_CD81
0.34764805477	1.17906153100	0.98195437200	1.41573390120	6219_14_PDZD11_PD
0.33301808536	1.17087432090	0.98263594320	1.39517253040	9932_49_SEMA4F_SE
0.34582105683	1.17791824130	0.98184315780	1.41314971970	10451_11_NUCB1_NU
0.33662610877	1.17264378960	0.98205848930	1.40021543750	12940_35_ALDH3B1_
0.30801436139	1.15694882920	0.98369250950	1.36072053000	13111_79_BCL6_BCL
0.33507308249	1.17182252760	0.98220761040	1.39804255390	5659_11_TRH_TRH
0.30291077131	1.15411279020	0.98388430240	1.35379366180	6430_36_FAM19A2_F
0.30517080298	1.15540645100	0.98386515630	1.35685673830	7184_13_CEACAM7_
0.33175971754	1.17000034080	0.98240499480	1.39341799430	8843_34_MYRF_MRF
0.31772785950	1.16188476100	0.98251379130	1.37400228860	10842_7_GCNT4_GC

0.31231263632	1.15897879611	0.98291355998	1.36658186933	13628_58_GRB14_GR
0.29288025106	1.14831266722	0.98383900022	1.34028228344	15615_8_LILRB3_LIL
0.33038926839	1.16880667933	0.98174598411	1.39150969388	16594_44_FAIM_FAI
0.30781760728	1.15651244560	0.98314400022	1.36045282960	17166_4_FGF8_FGF_
0.35763337077	1.18394796244	0.98027297411	1.42994126611	18863_176_CHST2_C
0.34223183551	1.17553942844	0.98139762533	1.40808670411	2838_53_MMP17_MM
0.34213269749	1.17540680160	0.98127346799	1.40794711611	7740_33_FATE1_FAT
0.32119555796	1.16391241688	0.98253299655	1.37877518499	7976_19_FRRS1L_FR
0.32664308853	1.16662529555	0.98175582511	1.38630660011	8219_14_ZG16_ZG16
0.32748188842	1.16705785522	0.98166022644	1.38746992188	5638_23_COLGALT1_
0.30642557250	1.15540655577	0.98263158477	1.35856034944	5715_4_ABHD14A_A
0.31062477577	1.15755473377	0.98215593322	1.36427721522	11258_41_MADCAM1
0.31952990804	1.16241506711	0.98164031322	1.37648053977	12511_83_SLC41A2_S
0.33188301551	1.16925217377	0.98102801511	1.39358981055	12848_9_ARHGEF2_A
0.31057070085	1.15758078733	0.98225325933	1.36420344400	18868_7_CBX3_CBX3
0.31347190537	1.15911806960	0.98201073099	1.36816702400	9754_33_NQO2_Quin
0.31751238850	1.16125117477	0.98165403122	1.37370626299	18160_2_PCMT1_PIM
0.31466144695	1.15969048155	0.98181226955	1.36979548400	3432_21_EPHA3_EPH
0.31126637189	1.15779208288	0.98192854033	1.36515281111	6899_37_PCBD2_PHS
0.31058737481	1.15737696422	0.98189101344	1.36422619099	5494_52_SNRPF_RUX
0.34264678532	1.17493403933	0.97998034199	1.40867111077	9384_17_CAMP_Cath
0.32832300927	1.16701401288	0.98076118677	1.38863744260	9984_12_YIPF6_YIPF
0.30578315734	1.15460072233	0.98189197799	1.35768786999	11227_31_TBL2_TBL
0.31528120389	1.15947454399	0.98083860122	1.37064468733	10638_1_TINF2_TINF
0.31806469442	1.16105023655	0.98077250177	1.37446517855	12453_161_NXF1_NX
0.30648261876	1.15485632344	0.98163990166	1.35863785244	12466_7_HNRNPA1_H
0.31683721004	1.16036691122	0.98082159700	1.37277907900	13932_45_ARHGEF7_
0.32178543677	1.16300818277	0.98042844122	1.37958873511	14631_22_DUSP16_D
0.32265611705	1.16350670933	0.98041514800	1.38079043899	17772_7_UBD_UBD
0.33445207058	1.16994603777	0.97967262599	1.39717462233	5617_41_NPFF_NPFF
0.33113280920	1.16810866155	0.97984490044	1.39254472277	6930_95_ST8SIA6_SI
0.30297159625	1.15283129477	0.98164084860	1.35387600877	7743_5_TIMM50_TIM
0.33546306569	1.17068915411	0.97992633988	1.39858787333	7983_1_PCDHGC5_PC
0.30511596301	1.15399438922	0.98151562011	1.35678233044	8034_6_DRGX_DRGX
0.30779050396	1.15521681822	0.98096901166	1.36041595733	10037_98_SIGLEC12_
0.31180507063	1.15743481600	0.98079413988	1.36588841533	10490_3_RPN1_RPN1
0.32729898905	1.16591392799	0.97991597077	1.38721617760	12798_46_SLC4A8_S4
0.32886915173	1.16676780855	0.97981214600	1.38939604360	13992_12_NSF_NSF

0.32333562858	1.16369953385	0.98007394777	1.38172902088	18434_141_TBPL1_TI
0.34887224242	1.17768431588	0.97846319155	1.41746808644	7696_3_NBR1_NBR1
0.33618202233	1.17034450006	0.97864558300	1.39959375899	10015_119_KCNAB2_
0.30624310540	1.15407943	0.98055444077	1.35831247955	12562_1_PKN1_PKN1
0.33251296778	1.16863984133	0.97938360455	1.39446798222	14206_28_APBB1_AP
0.30445667325	1.15307509211	0.98059873500	1.35588811255	14603_51_KIAA0040_
0.32391523276	1.16388347755	0.97981573100	1.38253010899	15497_9_HS3ST1_HS
0.31064479982	1.15641232599	0.98019865399	1.36430453388	16859_100_SH3GLB1
0.30814037616	1.15505462806	0.98035052233	1.36089201	17804_102_PNPO_PN
0.35258451816	1.17933315388	0.97756918877	1.42273989806	19516_10_HMGA2_H
0.30577386117	1.15394235499	0.98078164100	1.35767524877	5753_21_FAM19A1_F
0.31524378134	1.15907504100	0.98019949266	1.37059339533	7846_44_PSG11_PSG
0.33194132659	1.16814100655	0.97910721977	1.39367107466	8042_88_SPINK9_ISK
0.30246677182	1.15208252444	0.98086113800	1.35319271155	9819_110_NUP210_PO
0.32734617044	1.16563721006	0.97940466999	1.38728162999	9923_6_SOS1_SOS1
0.30900191747	1.15548553800	0.98023724722	1.36206498222	11462_8_RBFOX2_RI
0.32260062200	1.16273809700	0.97917458966	1.38071381400	12647_52_SETD2_SE
0.31035343405	1.15608256755	0.97992518877	1.36390708011	12709_63_H1FX_H1X
0.35140769257	1.17846726933	0.97728363900	1.42106656611	6442_6_BAGE3_BAG
0.31552610297	1.15882168911	0.97949446177	1.37098039811	5117_14_ROBO3_RO
0.32942432468	1.16633035000	0.97853415144	1.39016761288	5254_69_PDE3A_PDE
0.33178258506	1.16754921533	0.97827070100	1.39344985877	19196_73_HOPX_HO
0.31987869818	1.16053133922	0.97812011888	1.37696072633	12653_13_CSNK1G2_
0.30510864145	1.15297033633	0.97978157555	1.35677239677	12785_49_ZBTB33_K
0.34181862760	1.17246290177	0.97667096300	1.40750499	12849_25_GSKIP_GS
0.29707714304	1.14853888886	0.98010464033	1.34591912377	13056_18_SLC6A16_S
0.34590977106	1.17473317606	0.97645394322	1.41327509	13525_17_KCNG4_KC
0.31751113820	1.15948064844	0.97866413755	1.37370454533	13557_3_PVRL3_Nec
0.34049389479	1.17181448211	0.97688423822	1.40564165822	14005_2_CHD7_CHD
0.31791363552	1.15995313000	0.97906774844	1.37425756900	15313_28_MAD2L1_M
0.32190546797	1.16198065899	0.97857931211	1.37975433888	16612_28_SDC3_SDC
0.31579289988	1.15846094622	0.97862359200	1.37134622022	18886_28_DSCAML1_
0.32022670196	1.16088967377	0.97838369533	1.37743999722	19638_9_PDYN_Dync
0.30681831970	1.15379227611	0.97950295711	1.35909402500	3387_1_PAK3_PAK3
0.34168765740	1.17267138399	0.97714629744	1.40732066266	4708_3_HSD17B1_17
0.30836409432	1.15438664433	0.97899790522	1.36119650266	5351_52_HNRNPA2B
0.30463937222	1.15235836222	0.97920115500	1.35613585455	8633_18_RNF128_RN
0.02151686284	0.86530588144	0.73281551644	1.02175001988	8773_172_EMILIN3_E

0.30910213201	1.15510357331	0.97949112300	1.36220148771	8845_2_ADAMTS3_A
0.31343538088	1.15727585322	0.97892749533	1.36811705333	8874_53_CLN5_CLN5
0.32278481939	1.16234959699	0.97834014088	1.38096816133	9008_6_PRRG1_TMG
0.35218072738	1.17857815399	0.97671223260	1.42216552477	9385_4_GAA_GAA
0.32334230354	1.16208901844	0.97735652377	1.38173824388	18918_86_PDE4A_PD
0.30631831359	1.15286621411	0.97842033560	1.35841463955	15336_7_SELM_SELM
0.31458828364	1.15729007099	0.97782356322	1.36969526899	9350_3_FSTL4_FSTL4
0.30532101820	1.15234786688	0.97851608933	1.35706057422	9867_23_FBP2_F16P2
0.30188758703	1.15045328822	0.97865555660	1.35240918988	9541_15_B3GALT1_B
0.30867123482	1.15404283411	0.97811437888	1.36161464544	5667_3_SEMA3B_SEM
0.30992441407	1.15444736	0.97757437055	1.36332206222	13468_5_NEURL4_NE
0.31862067632	1.15912604855	0.97698102699	1.37522956888	7006_4_COL25A1_CO
0.29974548010	1.14899077833	0.97826221244	1.34951528533	9970_7_UBXN4_UBX
0.33280209164	1.16664681633	0.97576376933	1.39487121444	15419_15_MMP20_M
0.30221616333	1.15019439655	0.97789377811	1.35285363244	19195_85_RPS5_RS5
0.31875006683	1.15899536677	0.97663437100	1.37540752199	2737_22_NOV_NovH
0.29472688024	1.14620248688	0.97841800255	1.34275957444	8067_21_KCNE3_KCN
0.30827531157	1.15331734700	0.97727183344	1.36107565722	6458_6_RPN1_RPN1
0.33166227135	1.16589228188	0.97561340811	1.39328221777	9583_17_RNF24_RNF
0.32698240695	1.16330027599	0.97583638500	1.38677707933	8876_51_NRXN2_NR
0.31051652047	1.15439412411	0.97690561011	1.36412953300	7788_1_ATP5J_CF6
0.31787794591	1.15824989900	0.97622944833	1.37420852322	15634_139_SLIT1_SL
0.31184472275	1.15500296700	0.97663831311	1.36594257677	2835_1_XRCC6_Ku70
0.33514793745	1.16750175511	0.97490474555	1.39814720822	9315_16_MUL1_MUL
0.32333525939	1.16108595922	0.97567691077	1.38172851077	11198_37_CREB3L1_
0.30421161370	1.15081813399	0.97700315922	1.35555587999	17466_72_ACSF2_AC
0.31235999868	1.15517104911	0.97641932988	1.36664659544	18396_10_AES_AES
0.32634258145	1.16247535422	0.97507658122	1.38589006777	11160_56_RNF122_RN
0.30889056161	1.15318817633	0.97645199122	1.36191331677	12479_50_PRKAR1B_
0.34052450877	1.17012991511	0.97404775555	1.40568469111	5350_14_GPC6_GPC6
0.30417032649	1.15065936400	0.97677392555	1.35549991399	9383_24_CHI3L2_CH
0.30097006197	1.14881939755	0.97677353177	1.35116888960	13977_28_BARD1_BA
0.33560079209	1.16732188822	0.97416312422	1.39878050911	9907_216_TNKS_Tan
0.31716873475	1.15740228977	0.97549274355	1.37323426477	11220_53_C10orf105_
0.32158991877	1.15971684700	0.97507765700	1.37931902711	7879_12_CYP3A4_Cy
0.32125229145	1.15933041411	0.97475699700	1.37885340999	11538_216_MLYCD_
0.33507391550	1.16674771577	0.97371792760	1.39804371855	8993_151_RIPK2_RIP
0.29930333364	1.14767220755	0.97644985044	1.34891873388	9322_15_RCN1_RCN1

0.31952767627	1.15836176055	0.97480852377	1.37647746788	17811_78_SULT1C2_
0.31783373418	1.15729870206	0.97466976833	1.37414776844	11537_12_TFR2_TFR
0.31522417534	1.15592303299	0.97489471311	1.37056652	19119_10_DDIT3_DD
0.31247979490	1.15447553160	0.97512707460	1.36681032433	7767_1_CLPSL2_CF1
0.02451839013	0.86911516899	0.73706613599	1.02482143755	9018_38_PCDH10_PC
0.31859068651	1.15771725677	0.97463687022	1.37518832655	9035_2_MXI1_MXI1
0.31376274074	1.15504344760	0.97483522770	1.36856499333	15434_5_PNOC_PNO
0.32224429497	1.15954474822	0.97415061122	1.38022191600	3091_70_AURKA_Au
0.34700026715	1.17262274560	0.97188823933	1.41481710333	13960_15_AGAP3_A
0.32765081872	1.16227361710	0.97346382400	1.38770432733	19153_53_MPPED2_M
0.32647691495	1.16168033200	0.97361252111	1.38607625177	7950_142_BTNL9_BT
0.32076587528	1.15863446733	0.97406073733	1.37818287644	9095_5_MTFR1_MTF
0.31672101875	1.15621086688	0.97392138700	1.37261958333	11146_4_TBX22_TBX
0.30436865772	1.14979264333	0.97510957899	1.35576877860	11383_41_KRT7_Kera
0.30205781962	1.14837667833	0.97495974355	1.35263943355	19374_72_MTCP1_MT
0.32718991960	1.16175163988	0.97303802399	1.38706488299	2708_54_TNFSF18_T
0.30622797595	1.15069908688	0.97483343600	1.35829192911	4127_75_C6_C6
0.32088118643	1.15837953299	0.97351987500	1.37834180544	5606_24_MFAP1_MF
0.32897805710	1.16264057622	0.97278663833	1.38954736455	6454_38_ARHGDIA_
0.31676861845	1.15628117399	0.97399347255	1.37268492122	6510_56_RNF128_RN
0.32382886339	1.15988127011	0.97317284599	1.38241070588	6594_64_SAYSD1_SM
0.31904279762	1.15740074222	0.97366371700	1.37581020500	6599_5_APCDD1_AP
0.34451391456	1.17034859688	0.97053228200	1.41130373877	10087_10_CRYAA_A
0.30380625168	1.14899528788	0.97430541566	1.35500650044	10418_36_STX12_ST
0.32466917889	1.15984978699	0.97230263177	1.38357285511	10748_216_PCDHB2_
0.31668958026	1.15601133400	0.97361587611	1.37257643099	11370_20_INPP5E_IN
0.30014892564	1.14697458611	0.97443880000	1.35005985111	11536_9_MYSM1_MY
0.30959761890	1.15170671255	0.97325637844	1.36287660800	11573_3_SRSF6_SRS
0.32993798379	1.16261618444	0.97181250455	1.39088186855	15331_47_RBBP4_RB
0.31989737740	1.15726430833	0.97260265855	1.37698644711	15343_337_KNG1_Ki
0.31716230755	1.15599575600	0.97312950344	1.37322543877	16887_29_PTRH2_PT
0.31947707278	1.15740897888	0.97325482300	1.37640781500	17468_1_SUFU_SUFU
0.32982890738	1.16225202522	0.97130975133	1.39073016444	17785_11_RCL_RCL
0.30677430848	1.15068119399	0.97427069855	1.35903421099	19251_56_SRGN_Plat
0.30012733604	1.14718178944	0.97481194611	1.35003070411	19338_3_LZIC_LZIC
0.30681667201	1.15031659344	0.97361214244	1.35909178560	2976_58_DSG1_Desm
0.29236750993	1.14273572560	0.97480559644	1.33959524177	5643_2_LCN8_LCN8
0.32458255893	1.15947292433	0.97175505599	1.38345301533	6909_40_MGAT2_MG

0.29798959241	1.14569357859	0.97436510588	1.34714776722	6998_106_ASPH_HAA
0.31961162881	1.15745028477	0.97319333339	1.37659303144	7866_11_DNAJC30_D
0.33862339435	1.16727075855	0.97113798	1.40301486233	8858_21_BATF3_BAT
0.30030789529	1.14689603577	0.97415046322	1.35027448677	8967_6_DUSP26_DUS
0.29326881863	1.14313096044	0.97460120711	1.34080317488	9097_5_KIAA1324_K
0.30903515249	1.15155755331	0.97355173499	1.36211025122	9106_87_LYSMD4_LY
0.29598803543	1.14468041360	0.97459130644	1.34445407099	9287_6_SPR_SPRE
0.31257875989	1.15315678188	0.97280430611	1.36694559744	9391_60_PCSK1N_PC
0.30364180372	1.14882916488	0.97418389311	1.35478369000	9607_39_PRR27_CD0
0.31758641369	1.15635859388	0.97332759800	1.37380795559	9723_105_MMRN2_E
0.29997068667	1.14654922960	0.97388976066	1.34981923933	9742_59_DDX39B_BA
0.31296134703	1.15332645388	0.97271837744	1.36746867322	9873_17_ESRRA_ERI
0.28997552334	1.14145409255	0.97494951900	1.33639477711	9959_60_TMEM59L_T
0.29606296713	1.14435112888	0.97395769199	1.34455481699	16882_27_PHPT1_PH
0.30021907340	1.14654624300	0.97364281700	1.35015455811	18824_7_EIF4A2_IF4
0.32545622564	1.15970437822	0.97129409999	1.38466222033	6493_9_CPZ_CBPZ
0.33601537774	1.16528631099	0.97036620944	1.39936054300	6526_77_OBP2A_OBI
0.29457919916	1.14355994088	0.97405559733	1.34256128888	8272_22_HSD17B7_D
0.29375610114	1.14319300744	0.97423216700	1.34145668400	8882_1_ADAM17_AD
0.30050594112	1.14661398188	0.97347856799	1.35054192944	9836_20_DCK_DCK
0.30934563002	1.15117257888	0.97259889555	1.36253322155	2642_4_PAFAH1B2_F
0.29712727003	1.14480971999	0.97370159722	1.34598659233	7257_18_PTH2_TIP39
0.34509430764	1.16981045600	0.96907735355	1.41212308733	10713_151_CD300LB_
0.31461186600	1.15375098533	0.97182926400	1.36972756999	12669_30_HECW1_H
0.30726254137	1.14985531311	0.97239779722	1.35969789811	13477_65_LTB4R_LT
0.32382583164	1.15854114033	0.97092827599	1.38240651400	16807_35_TNNT3_TN
0.29628824797	1.14419346922	0.97347001322	1.34485775344	5713_9_IFNL3_IFN_la
0.33171547371	1.16272513344	0.97026847455	1.39335634500	5758_49_KLK9_kallik
0.29087186897	1.14142603844	0.97402813888	1.33759318577	5915_58_PEX5_PEX5
0.33292341239	1.16333597733	0.97011566566	1.39504045155	7199_3_SYN3_SYN3
0.31681654401	1.15471637877	0.97131249400	1.37275070944	11116_16_C11orf87_C
0.30366606165	1.14791208199	0.97260558477	1.35481655533	12426_19_MOB1A_M
0.30832569046	1.15030513888	0.97212469099	1.36114422844	16591_71_CHIA_CHI
0.30942377763	1.15086886399	0.97200979666	1.36263970433	7792_58_CCDC90B_C
0.31507148743	1.15381805922	0.97149564011	1.37035727077	8305_18_SULF2_SUL
0.32307426793	1.15784381688	0.97048893844	1.38136793844	12427_8_CDC25B_MI
0.32759396089	1.16014302860	0.96995328855	1.38762542760	15692_300_NODAL_N
0.31765186498	1.15495402255	0.97090098000	1.37389787559	3115_64_MAPK1_MK

0.30348709870	1.14752180800	0.97211831022	1.35457411500	5031_10_SPTAN1_SP
0.31432758911	1.15323395844	0.97123451377	1.36933824333	8315_5_DEFB119_DE
0.30444343333	1.14805915155	0.97209884360	1.35587016088	8903_1_COX6C_COX
0.33683925838	1.16507239160	0.96921112577	1.40051392477	9365_91_EYS_EYS
0.32158954938	1.15693454733	0.97040497144	1.37931851760	4963_19_ARPP19_AR
0.30623549468	1.14889639955	0.97177417022	1.35830214188	7143_9_GCNT2_GNT
0.32444856420	1.15838791344	0.97006718533	1.38326765233	7092_7_PDILT_PDIL
0.29219157681	1.14154108722	0.97293965711	1.33935958333	9573_108_TXNDC11_
0.29791411088	1.14443580455	0.97230029760	1.34704608633	13940_19_IFI16_IP16
0.32287744707	1.15734133669	0.96983764300	1.38109608311	10064_12_RBBP9_RB
0.29886056744	1.14482617560	0.97204328860	1.34832161044	12616_45_NRBP1_NR
0.31045398799	1.15087914260	0.97102628255	1.36404423322	5669_26_SIRPB2_SIR
0.30064611591	1.14571421022	0.97181511655	1.35073125460	5036_50_TNFAIP6_T
0.30684541796	1.14890640744	0.97119856300	1.35913085460	11201_19_PUS1_TRU
0.33225484573	1.16200711288	0.96854795122	1.39410808577	4471_50_TGM3_TGM
0.32362870127	1.15749815755	0.96937196999	1.38213402722	5026_66_RPS3_RS3
0.30774770045	1.14923788877	0.97088265655	1.36035772799	5664_57_DEFB106A_
0.32982823919	1.16072111800	0.96875328411	1.39072923511	8976_13_B4GALNT1_
0.34054887567	1.16625652477	0.96758622160	1.40571894377	10890_135_LCTL_LC
0.34508463875	1.16861799377	0.96711202588	1.41210943388	11682_7_TXNIP_TXN
0.32526030132	1.15822408755	0.96900592233	1.38439095788	17474_106_YEATS4_
0.31419874642	1.15250096860	0.97012526788	1.36916182555	8062_15_EVA1B_F17
0.29991473338	1.14488647499	0.97112142577	1.34974371455	10643_16_OLFM3_N
0.32422662569	1.15751431244	0.96881957455	1.38296068600	10815_2_HABP4_HA
0.30837529502	1.14930713855	0.97039046244	1.36121174900	17704_74_HSPH1_HS
0.29530281169	1.14249976311	0.97154709100	1.34353313460	9800_20_NDUFB8_N
0.29681034326	1.14330743133	0.97145560522	1.34556008077	9808_41_TNRC6B_T
0.29398508331	1.14168651144	0.97144371022	1.34176388888	11174_8_THSD7A_T
0.30309414867	1.14629031411	0.97041416955	1.35404193960	12580_7_PSMB5_PSE
0.03045347818	0.87010283511	0.73437078288	1.03092192855	13416_8_TMEM132D
0.36722050054	1.17991712977	0.96432000255	1.44371622422	13439_6_IQCF3_IQC
0.29133094303	1.14029991344	0.97166097799	1.33820738100	19369_17_KAT2A_G
0.30169910072	1.14562843700	0.97064699833	1.35215430322	4543_65_COL23A1_C
0.31034830810	1.15006820760	0.96976082999	1.36390008888	6280_11_PCDH8_PC
0.31042021537	1.15026554900	0.97002390888	1.36399816677	8950_4_CLCA2_CLC
0.31044917652	1.15009050400	0.96970061488	1.36403767022	9074_6_FAXDC2_CE
0.30167319285	1.14550442	0.97046199677	1.35211927222	12431_13_PELO_PEL
0.29630304376	1.14275726999	0.97101336777	1.34487765188	13132_14_SEMA5A_

0.31046370643	1.15000794119	0.96954730755	1.36405748977	5752_63_SUSD3_SUS
0.29484672283	1.14185964344	0.97090143588	1.34292050388	7825_7_ABHD12_AB
0.32609960466	1.15796980744	0.96776790000	1.38555336955	19174_141_GOLGA7_
0.30271961230	1.14582914977	0.96999674222	1.3535349	6223_5_GUCA2B_GU
0.30823915631	1.14825668777	0.96874930144	1.36102644800	10767_52_GPR64_GP
0.32652558822	1.15769702699	0.96690032444	1.38614371822	11401_181_RNF146_F
0.30719010038	1.14770150099	0.96882856199	1.35959940388	11449_22_CAPZA1_C
0.29385276671	1.14116854100	0.97069087399	1.34158636299	12348_46_SARS2_SY
0.32130039230	1.15520572499	0.96778676277	1.37891973555	12424_107_THYN1_T
0.29960299937	1.14394326377	0.96982425411	1.34932301911	12500_88_UBA2_SAE
0.29955836855	1.14392698099	0.96983992933	1.34926279911	12662_82_ECH1_ECH
0.31593439868	1.15230288777	0.96811006322	1.37154027788	12795_2_ZNF566_ZN
0.32034807224	1.15481090088	0.96804679044	1.37760718777	13025_4_FRS2_FRS2
0.33108091991	1.16021797311	0.96670187577	1.39247246655	13501_10_SLC35G2_S
0.32385914144	1.15638788116	0.96729028422	1.38245256311	18947_3_LRRC3B_LF
0.32433169849	1.15689771177	0.96768599833	1.38310600522	3761_4_PTGS2_COX_
0.30165945814	1.14492282388	0.96949012099	1.35210070144	5590_11_ZPLD1_ZPL
0.29328322448	1.14068091377	0.97041402299	1.34082249044	5727_35_B3GALTL_F
0.29846288148	1.14334033988	0.96990739599	1.34778550844	6372_7_YBX2_YBOX
0.31527389856	1.15186105111	0.96800694300	1.37063467444	8962_48_RPS6KB1_K
0.31030681612	1.14939874933	0.96867234822	1.36384349911	9398_30_GALP_GAL
0.30608750525	1.14725320455	0.96913983377	1.35810114233	9951_36_FBXL4_FBX
0.31427464521	1.15131704	0.96805965722	1.36926574711	13942_140_SPSB1_SF
0.33206420044	1.16050665377	0.96623245222	1.39384233099	12731_12_PLEKHA7_
0.32585816427	1.15719874766	0.96671288533	1.38521888144	11254_13_PAPLN_PP
0.30631628015	1.14707537800	0.96861779911	1.35841187733	14633_26_TMEM185A
0.29169430093	1.13957133022	0.97006716288	1.33869371766	15580_2_EPHA7_EPH
0.29207189410	1.13976312322	0.97002737555	1.33919929477	18264_12_CCDC25_C
0.31607021943	1.15207954433	0.96760338500	1.37172657411	19360_22_LRRTM1_L
0.30943317941	1.14861948999	0.96820481922	1.36265251566	9401_57_TRABD2A_
0.29590559665	1.14147552933	0.96922150911	1.34434324033	13447_42_SPRN_SPR
0.29374501970	1.14041470766	0.96951331555	1.34144181888	4192_10_AKR1A1_AI
0.28816009544	1.13754208088	0.97003767800	1.33397084966	5612_16_HINT2_HIN
0.31583290724	1.15156169100	0.96696316088	1.37140108533	8366_19_C1orf115_C
0.30369781093	1.14519102144	0.96796930411	1.35485957044	13652_2_TOM1L1_TM
0.29527155663	1.14082895500	0.96873783755	1.34349114300	12713_365_RGS19_R
0.32459310621	1.15579389433	0.96558786011	1.38346760700	10035_6_DUSP4_DUS
0.32199404072	1.15444462488	0.96584175522	1.37987655288	11163_7_FAM162B_F

0.28341516715	1.13471150068	0.96980691112	1.32765624606	11135_5_MRPL55_RM
0.30412111882	1.14512518006	0.96744838885	1.35543321406	3847_56_ETHE1_ETH
0.32666870840	1.15660109933	0.96493216655	1.38634211755	17319_1_SELH_SELH
0.30647640464	1.14621050788	0.96700286255	1.35862940977	12658_72_PANK3_PA
0.28984990678	1.13770919211	0.96868442911	1.33622691433	12766_33_GPR142_G
0.32199621267	1.15404011788	0.96516293300	1.37987954988	12803_9_ZNF329_ZN
0.29580839087	1.14063002999	0.96788030006	1.34421256877	12420_10_GPD1L_GP
0.29580171629	1.14053156760	0.96771966699	1.34420359677	14021_81_MED4_ME
0.28904644623	1.13709539633	0.96841727044	1.33515373999	18409_61_ARF5_ARF
0.30864220950	1.14699992233	0.96624034760	1.36157512477	18156_7_ATG3_ATG3
0.29823537713	1.14160989331	0.96719372177	1.34747891622	5864_10_ALDOA_ald
0.30253072958	1.14380674533	0.96675823522	1.35327926144	7745_3_CISD1_ZCD1
0.30960587299	1.14720953888	0.96566252233	1.36288785733	13518_5_ASAP2_ASA
0.29498246912	1.13975148744	0.96718839455	1.34310281277	19223_6_RAB1A_RA
0.31405135165	1.14935051360	0.96497090588	1.36896003331	10753_31_ADAM7_A
0.29548686143	1.13995274077	0.96704209844	1.34378043433	14216_35_DDX46_DI
0.28315926603	1.13360651960	0.96816674944	1.32731654144	10513_13_KCNIP3_C
0.31798151735	1.15105242600	0.96403453199	1.37435085933	10572_65_CST8_CST8
0.30595823354	1.14494237699	0.96536441799	1.35792558960	12041_33_HSPA1L_H
0.30711941272	1.14550344955	0.96518938399	1.35950330033	2714_78_AIMP1_EMA
0.31491819804	1.14955476188	0.96447748511	1.37014722560	3192_3_CNDP2_Gluta
0.03699763890	0.86789436877	0.72588173822	1.03769057077	4883_56_INS_Insulin
0.32624251395	1.15519405411	0.96299618400	1.38575139211	6411_58_DEFB135_D
0.29026844126	1.13709899688	0.96724071744	1.33678628844	6641_60_UBB_PolyUb
0.03659470884	0.86904084022	0.72809406800	1.03727253822	9805_51_SEMA4B_SE
0.28669743192	1.13527239555	0.96758481344	1.33202112544	9952_57_TFAM_TFA
0.31124904067	1.14742971499	0.96444717277	1.36512915155	13459_30_TOR4A_CI
0.32262474040	1.15316120144	0.96308783999	1.38074711560	13554_78_REPIN1_RE
0.28502062502	1.13407335244	0.96716240600	1.32978945477	14128_121_IFNA10_I
0.31097001328	1.14708527899	0.96413722644	1.36474829622	2500_2_ANGPT4_Ang
0.32525894087	1.15422906760	0.96233404699	1.38438907444	9993_11_ZNF264_ZN
0.31556787730	1.14924550060	0.96333255355	1.37103767111	12820_1_GRAP_GRA
0.30153215953	1.14217614399	0.96496690100	1.35192859188	9173_21_PGM1_PGM
0.31345587052	1.14811895260	0.96347758944	1.36814508599	11534_6_LRIT3_LRIT
0.31845838695	1.15064057811	0.96288550977	1.37500640177	12794_6_NLRP4_NAI
0.28924519884	1.13586735555	0.96613461560	1.33541913160	16883_57_RAB4A_RA
0.29670926427	1.13961873400	0.96529479333	1.34542407977	19238_12_GLUL_GLN
0.30150363632	1.14195244011	0.96461645955	1.35189003160	8424_269_CTNNB1_b

0.30916582572	1.14580993388	0.96373172999	1.36228825432	8916_32_STIM1_STIM
0.30413889962	1.14322660155	0.96422590922	1.35545732	10076_1_AP4M1_AP4
0.32422011018	1.15339831833	0.96194805960	1.38295167533	19145_4_MREG_MRE
0.31963144040	1.15086572111	0.96213306160	1.37662030422	11247_20_NAGS_NA(
0.32771468947	1.15488444851	0.96106422844	1.38779296388	13268_45_WNT5A_W
0.30441440081	1.14309308577	0.96373515455	1.35583079700	5260_80_TYK2_TYK2
0.29080501769	1.13635008700	0.96544888833	1.33750376899	6294_11_BAGE2_BA(
0.28934296816	1.13553971333	0.96548293155	1.33554970100	8850_5_PLA2G2C_PA
0.30743345974	1.14461621600	0.96339221733	1.35993031533	8954_30_MAP4K1_M
0.31049218618	1.14603072800	0.96282527333	1.36409633833	15440_57_PCSK2_NE
0.29163126438	1.13650795233	0.96491955722	1.33860933360	14287_6_RAB5C_RAI
0.31829662466	1.14981027200	0.96165191477	1.37478399555	14662_6_MZF1_MZF1
0.29519350302	1.13818495488	0.96432798811	1.34338628288	16836_1_CFHR3_FHF
0.29612572420	1.13871346544	0.96432437533	1.34463919988	19327_31_HAT1_Hat1
0.30798733812	1.14458613088	0.96280814799	1.36068376000	6536_54_PLBD2_LAN
0.03509399949	0.88208952299	0.75124950211	1.03571706111	9237_54_ACP2_Lysos
0.32580563716	1.15351000000	0.96061007511	1.38514612177	9114_84_UMODL1_U
0.28484239482	1.13281479388	0.96518895544	1.32955246722	17356_34_IL1F10_IL1
0.29703979262	1.13888272044	0.96372974766	1.34586885400	2761_49_FGF18_FGF
0.29236209247	1.13652204677	0.96423854022	1.33958798455	17821_20_NMT2_NM
0.31374366163	1.14706775711	0.96143738122	1.36853888255	9875_107_THOC1_TH
0.30966007726	1.14490372811	0.96173246422	1.36296173377	19376_74_NNMT_NN
0.29212107176	1.13613649733	0.96381671377	1.33926515500	6991_24_FUT9_FUT9
0.30347049107	1.14156663277	0.96207066511	1.35455161899	11551_16_HMG20B_I
0.30966005149	1.14466322166	0.96132847488	1.36296169855	12437_18_ULK3_ULK
0.33358836582	1.15670039000	0.95844275211	1.39596839699	17137_160_HBB_Beta
0.29011657187	1.13495833477	0.96374871233	1.33658328699	17195_43_BAG1_BA(
0.33479223620	1.15741421811	0.95847150388	1.39764997399	17855_28_RGS4_RGS
0.29624533692	1.13806458500	0.96311046699	1.34480005	6469_62_LY6G6D_LY
0.30789145787	1.14387325444	0.96170140400	1.36055330355	6511_17_FAM19A4_F
0.29750955862	1.13861365110	0.96282201766	1.34650124599	9075_121_KIAA1024_
0.29024216086	1.13496996822	0.96364743844	1.33675115766	5980_55_BOLA3_BOL
0.30827498840	1.14383109899	0.96126177766	1.36107521733	3182_38_ENTPD1_CD
0.29194631228	1.13569813722	0.96324143166	1.33903112611	9490_3_C4orf32_CD0
0.29779798789	1.13855532900	0.96244574833	1.34688967233	11360_39_RRM1_RRI
0.29209317027	1.13569684233	0.96309778600	1.33922778800	16802_31_OCIAD1_C
0.28403750403	1.13172794500	0.96411348833	1.32848275322	5252_33_PDE11A_PD
0.29577428949	1.13733156611	0.96232339522	1.34416673000	10761_5_TMED2_TM

0.29043436525	1.13453713332	0.96272752222	1.33700811117	11567_23_ZNF10_ZN
0.30497374713	1.14174199772	0.96092067332	1.35658938881	12329_21_RPS6KA1_
0.32757450896	1.15317735822	0.95835941088	1.38759843559	12378_71_TAPBP_TP
0.32079561778	1.14965062588	0.95898539606	1.37822386766	12987_12_SRSF7_SRS
0.29474479736	1.13677142222	0.96236596477	1.34278363306	13421_17_TMEM167I
0.30217964301	1.14047590003	0.96147339988	1.35280422677	13532_25_CNEP1R1_
0.31708555449	1.14790281806	0.95962540633	1.37312004344	13561_5_HTR6_5HT6
0.30165769537	1.14024404377	0.96158427339	1.35209831799	18385_4_PSMD10_PS
0.30198990952	1.14054359488	0.96177000506	1.35254757877	18830_1_ITLN1_Ome
0.28853179466	1.13360126606	0.96299702678	1.33446677777	19503_2_SNX3_SNX3
0.31039805009	1.14448573099	0.96032139466	1.36396793366	19570_12_FGF8_FGF
0.32249548246	1.15072579377	0.95914813688	1.38056865406	2875_15_TBP_TBP
0.28224012139	1.13054520022	0.96383020822	1.32609710606	6350_43_APOC2_Apo
0.32182220653	1.15021204199	0.95893729966	1.37963946332	8815_1_OLFML1_OL
0.30610571276	1.14204495406	0.96034300333	1.35812587011	3499_77_IL17B_IL_17
0.31325157253	1.14559430555	0.95944097711	1.36786560511	7246_4_FREM2_FREI
0.28330771145	1.13084555606	0.96331343233	1.32751359011	8450_36_ADCYAP1_I
0.28709415354	1.13257373944	0.96260822577	1.33254967118	11618_83_MYB_MYE
0.31795936189	1.14784409506	0.95868915033	1.37432041033	12528_40_WRNIP1_W
0.29516969466	1.13655437277	0.96158983733	1.34335429944	12727_7_PTGFRN_FF
0.29064900223	1.13433417099	0.96217655922	1.33729511399	8306_54_PRRG1_TM(
0.29171059057	1.13475809106	0.96187420077	1.33871552466	12536_46_MTMR6_M
0.31849268998	1.14802059311	0.95847268166	1.37505356944	8320_5_SLTM_SLTM
0.31401458584	1.14573325	0.95894175422	1.36890970311	13610_9_MAGEA10_I
0.29904373548	1.13778706677	0.95995072611	1.34856860244	10080_9_EIF2B1_EI2I
0.29450046835	1.13557847433	0.96058184644	1.34245559211	11190_129_YME1L1_
0.31047122410	1.14353422922	0.95865512466	1.36406774433	11667_29_TNS2_TEN
0.30559828406	1.14135488488	0.95966963906	1.35743689299	12793_4_PIWIL1_PIW
0.31269644167	1.14465216055	0.95839541106	1.36710647144	13027_20_CBX7_CBX
0.29003255594	1.13358830133	0.96150416999	1.33647099733	13529_39_NAP1L2_N
0.28030362920	1.12896752990	0.96300509033	1.32353161411	13541_1_MYCT1_MY
0.32772692299	1.15213218155	0.95647719766	1.38780994155	13990_1_PC_pyruvate
0.31259059631	1.14453355655	0.95829823777	1.36696177722	14029_42_COPS2_CS
0.30979851903	1.14351942555	0.95927539655	1.36315043755	15319_226_CCNA1_C
0.28324051745	1.13031276677	0.96247059966	1.32742439211	15402_2_ID1_ID_1
0.29359678323	1.13545329277	0.96123834111	1.34124298299	19261_12_SDCBP2_S
0.28597563893	1.13138243877	0.96165927533	1.33106002888	2647_66_GDI2_Rab_C
0.28990311760	1.13362155955	0.96168506055	1.33629801799	5001_6_SCGB2A1_M

0.2884249700	1.1328454567	0.9617893452	1.3343242315	6651_74_UBB_PolyU
0.2804789210	1.1288713007	0.9626721691	1.3237636388	7822_11_HRASLS2_
0.3016574473	1.1392446819	0.9598996980	1.3520979826	8394_56_RNASE2_R
0.3161312619	1.1463093664	0.9578767221	1.3718103103	8659_68_G6B_G6B
0.2945757713	1.1357902062	0.9608677274	1.3425566868	9321_400_NMB_NM
0.3120268103	1.1442391026	0.9583453680	1.3661913205	9524_46_SPATA31D4
0.3235378098	1.1498206169	0.9566421178	1.3820084088	19116_1_MUTED_M
0.3015691590	1.1389605246	0.9595056192	1.3519786134	9747_48_MARK3_M
0.3142661380	1.1451825779	0.9577792304	1.3692540986	12860_7_ARPP21_c
0.2888323636	1.1326091668	0.9609965817	1.3348679374	4224_7_SYNCRIP_H
0.2943267091	1.1352536594	0.9601992344	1.3422223483	8690_25_CAV3_CAV
0.3150286298	1.1454381160	0.9574763728	1.3702985419	9043_7_TFIP11_TFP1
0.3079843132	1.1420007180	0.9584663411	1.3606796440	9050_170_RSRP1_CA
0.3098225434	1.1427590952	0.9579771541	1.3631831868	16814_13_FHL1_SLI
0.3103353179	1.1430453411	0.9579657885	1.3638823717	18275_5_CRIP1_CRI
0.3183016048	1.1469470159	0.9568637038	1.3747908423	9060_27_IGSF9B_TU
0.2948236296	1.1353694363	0.9599179714	1.34288949	5690_49_TUFT1_TU
0.3740606245	1.1747118867	0.9493148215	1.4536252732	5088_175_IL23R_IL_
0.2813071800	1.1287234953	0.9616232932	1.3248605122	9532_5_WBP1L_WB
0.3060946998	1.1407828655	0.9582321544	1.3581109133	5716_49_CSAG1_CS
0.2851390016	1.1303961809	0.9607868893	1.3299468801	13602_6_NHP2L1_N
0.4829406357	1.2307960550	0.9346171328	1.6208336825	18819_21_PPIC_PPIC
0.2869131769	1.1312696349	0.9605665315	1.3323085333	8535_102_DMKN_De
0.2904695598	1.1330331642	0.9601429933	1.3370551680	8992_1_TMEM2_TM
0.3112084795	1.1430744745	0.9571784851	1.3650737814	12751_26_ZHX1_ZH
0.2880949454	1.1318076781	0.9603448829	1.3338839442	15395_15_GSTM1_G
0.3040245482	1.1395388426	0.9581248028	1.3553023260	15574_37_CCNA2_Cy
0.2837637910	1.1296234735	0.9607941900	1.3281191800	12746_4_CYTH4_CY
0.2972922731	1.1361879434	0.9589323263	1.3462087026	18337_4_GMDS_GM
0.2878314217	1.1315441942	0.9601507890	1.3335324806	12540_25_RHOG_RH
0.3030298125	1.1389348085	0.9580618723	1.3539548287	18315_38_RTP4_RTP
0.3056649221	1.1401174324	0.9575260174	1.3575273529	11669_39_SLCO5A1_
0.3048794392	1.1396721975	0.9575301318	1.3564614570	10042_8_SGK3_SGK
0.3071483463	1.1407398941	0.9571509354	1.3595426363	12777_11_SF1_SF01
0.3100543903	1.1421113529	0.9566696279	1.3634992733	5230_99_HMGCR_H
0.0427357019	0.8797376300	0.7415602786	1.0436620205	6373_54_SEMG2_SE
0.2935169072	1.1339660818	0.9587985220	1.3411358541	12515_45_UCK2_UC
0.2855200876	1.1300814455	0.9598860725	1.3304538007	14688_6_PTPN7_PTN

0.32007246953	1.14692509480	0.95513421559	1.37722756777	17344_23_STOML1_S
0.29424640404	1.13429610900	0.95865710433	1.34211456533	3823_9_MATK_MATK
0.29700987818	1.13559963555	0.95821008577	1.34582859377	14326_4_UBE2E1_UB
0.30095660339	1.13738123244	0.95743284822	1.35115070499	9070_1_C17orf67_CQ
0.29533898619	1.13455508088	0.95804757977	1.34358173711	19242_21_PFDN1_PF
0.28677687474	1.13041856160	0.95925251360	1.33212694911	6441_62_ADAMTS6_
0.31509154156	1.14418145088	0.95531651988	1.37038475233	9378_6_FIBCD1_FBC
0.29809221734	1.13578401700	0.95748438655	1.34728602522	11167_6_MTMR1_MT
0.28087822366	1.12742448100	0.95982279355	1.32429232660	12856_14_TMEM237_
0.30676996419	1.14001778155	0.95630130411	1.35902830699	13634_209_PIR_PIR
0.49660745119	1.23627963711	0.93016406255	1.64313738044	19558_10_LRP4_LRP4
0.04609497392	0.87391795022	0.72932739500	1.04717386044	6904_14_LRRTM2_LI
0.29112232355	1.13230453660	0.95828276200	1.33792823400	19631_13_KNG1_Kin
0.32205987176	1.14730086633	0.95386259355	1.37996739455	12760_34_ZNF774_ZI
0.31166302610	1.14213263811	0.95516753355	1.36569441211	13570_43_HDLBP_VI
0.28801689085	1.13066199388	0.95847643899	1.33377983260	18877_15_CNN2_CNI
0.30427210266	1.13843407722	0.95603122988	1.35563787855	14663_44_RNF8_RNF
0.29858584433	1.13564066977	0.95677030933	1.34795124622	11590_5_RBM23_RBI
0.28043463126	1.12685490300	0.95927866255	1.32370501099	6455_52_HAPLN4_HI
0.27577612018	1.12458281660	0.95987535100	1.31755285755	19132_1_MRPL2_RM
0.27831217098	1.12575582244	0.95944252477	1.32089847900	17150_8_HSPE1_HSP
0.30328467257	1.13773442244	0.95579980199	1.35429994166	13572_43_PSMD11_P
0.30897281939	1.14049370111	0.95499388688	1.36202534933	17419_17_TES_TES
0.31038295305	1.14112150911	0.95469836600	1.36394734199	5178_5_PDE7A_PDE7
0.30699462600	1.13932435211	0.95492373555	1.35933366299	2760_2_FAM107A_DI
0.28255073668	1.12752614833	0.95839164477	1.32650907600	17724_3_WWOX_WW
0.28819181740	1.13021194511	0.95754605200	1.33401316655	9927_96_TNS4_TENS
0.27527173274	1.12394418460	0.95926918711	1.31688846799	9847_21_TGIF2_TGIF
0.28989564534	1.13090599000	0.95709033300	1.33628803288	12332_7_EEF2K_EF2I
0.27951331571	1.12586522000	0.95847704411	1.32248602255	12387_7_PDLIM4_PD
0.30102763039	1.13621027500	0.95539460811	1.35124667655	6917_49_LRRC3_LRI
0.29072315304	1.13121249188	0.95681708944	1.33739427911	8569_147_ARL8B_AI
0.30559535250	1.13834586333	0.95461903977	1.35743291355	3419_49_CAMK2D_C
0.30925874341	1.14008774433	0.95404132855	1.36241484077	12345_4_ANAPC10_A
0.28454965009	1.12816508755	0.95756214466	1.32916330477	10058_1_RAD23A_RI
0.28226205465	1.12700753255	0.95778666177	1.32612619199	11542_11_TMEM230_
0.29053164903	1.13096267860	0.95657770644	1.33713818733	18401_18_ALKBH3_A
0.31567772447	1.14312132899	0.95298828611	1.37118828399	9937_7_GJA1_CXA1

0.2817698280	1.126733426	0.957792155	1.325473598	8229_1_GXYLT1_GX
0.3054512469	1.138000244	0.954176947	1.357237314	10624_45_SPINK13_I
0.2916786022	1.131407761	0.956233380	1.338672702	17820_170_MLF1_MI
0.2911082407	1.131124418	0.956299774	1.337909392	7141_21_MGAT4B_M
0.2736172990	1.122741219	0.958801824	1.314711564	7863_50_PGRMC1_P
0.3040046930	1.137229949	0.954265046	1.355275416	11657_86_SOCS7_SO
0.2967828497	1.133656545	0.955150583	1.345523087	10677_9_NDUFB4_N
0.2874390595	1.129185607	0.956527522	1.333009355	12754_14_CSDC2_CS
0.2906380951	1.130664707	0.955971954	1.337280528	11562_9_POLE2_DPC
0.2887936757	1.129741556	0.956173511	1.334816295	4995_16_HPGD_HPG
0.3211140460	1.145290543	0.951422223	1.378662803	8556_5_FAM134B_F1
0.2944755665	1.132387916	0.955215451	1.342422162	9550_153_TTC17_TT
0.3045695610	1.137152923	0.953596974	1.356041184	11440_58_SOCS3_SO
0.3291898086	1.149039970	0.949959204	1.389841634	9983_97_PRSS35_PR
0.2884456778	1.129393149	0.955916442	1.334351862	17140_57_PDGFD_PD
0.0388167181	0.900930168	0.780772256	1.039579930	10916_44_PLA2R1_P
0.3264585984	1.147543756	0.950078173	1.386050863	12738_43_NISCH_NI
0.2852319976	1.127774998	0.956247345	1.330070565	8888_33_SMIM9_CX
0.2836815700	1.126991794	0.956401320	1.328009985	3471_49_STK16_STK
0.2888901781	1.129390268	0.955486758	1.334945114	7147_35_WFDC8_WF
0.2856381504	1.127663711	0.955670405	1.330610887	18890_227_FGB_Fibri
0.3013742209	1.135094097	0.953188007	1.351715087	3400_49_TBK1_TBK1
0.2862256728	1.127789092	0.955321495	1.331392880	7757_5_HLA_DQA2_
0.2864757028	1.127875489	0.955229003	1.331725810	14587_16_GDF11_GD
0.3068229234	1.137483521	0.952003894	1.359100281	10708_3_GNRH2_GO
0.2744375034	1.122139530	0.956989185	1.315790339	18016_19_DNAJC4_DN
0.3138285683	1.140842685	0.950949619	1.368655085	8955_60_GLT8D1_GL
0.2941105586	1.131394481	0.953888293	1.341932257	7253_6_QKI_QKI
0.2822185286	1.125719701	0.955640581	1.326068472	13547_5_HTR7_5HT7
0.3069142938	1.137359803	0.951709854	1.359224469	14127_240_IFNB1_IF
0.2970657800	1.132600630	0.953102413	1.345903830	4862_63_BMPR2_BM
0.3035966179	1.135708354	0.952101622	1.354722475	7969_163_WFDC11_V
0.2985903918	1.133278452	0.952789808	1.347957376	10562_42_NETO2_NE
0.3046833249	1.136129067	0.951772288	1.356195461	8253_2_POMGNT1_P
0.2757750794	1.122472707	0.956277604	1.317551486	9606_4_NUP210L_P2
0.2946587820	1.131253731	0.953128304	1.342668138	13375_48_SYNGR3_S
0.2773611514	1.123083232	0.955800974	1.319642875	6497_10_RS1_XLRS1
0.3513291220	1.158489458	0.944504156	1.420954916	6997_32_RAB26_RA

0.2765044815	1.122710517	0.955985295	1.318512861	7947_19_ENTHD2_A
0.2760757887	1.122508908	0.956051751	1.317947745	9513_9_FGF22_FGF2
0.3172602084	1.141883964	0.949422654	1.373359885	12454_105_NTMT1_N
0.2837425897	1.125958998	0.954590946	1.328091022	12693_2_SH3BGRL_S
0.2808159991	1.124577135	0.955040215	1.324209925	15612_5_LIN7B_LIN7
0.2978671810	1.132533175	0.952225466	1.346982871	6604_59_NDNF_CD0
0.2850678950	1.126007563	0.953408899	1.329852315	10048_7_CBFB_PEBI
0.3010325997	1.133686542	0.951150379	1.351253391	10722_13_SYK_KSYI
0.2928791944	1.129781839	0.952342927	1.340280867	11539_4_ATP5J2_ATI
0.3035082953	1.134661001	0.950430311	1.354602827	11560_76_NFATC4_N
0.3131399423	1.139597577	0.949528677	1.367712918	12503_5_GPR68_SPR
0.2744133982	1.121217348	0.955439942	1.315758622	12756_3_BACH2_BA
0.2805271802	1.123809582	0.954012478	1.323827524	12811_55_ZNF415_ZI
0.2942653603	1.130528866	0.952281812	1.342140007	12876_39_SPEF1_SPI
0.3000544780	1.133380528	0.951567258	1.349932347	12882_7_PARP11_PA
0.2795455880	1.123524524	0.954464999	1.322528702	14037_18_RANBP3_F
0.2761844562	1.121809339	0.954756705	1.318090971	14132_21_HHLA2_HI
0.3048960149	1.135466548	0.950460408	1.356483941	15427_35_LOXL3_LC
0.2860050256	1.126557706	0.953446835	1.331099144	18897_31_HDAC2_HI
0.2760893620	1.121750687	0.954747659	1.317965634	19271_64_GNAI1_GN
0.2930946151	1.130113346	0.952696640	1.340569622	19370_30_HS3ST4_H
0.2901429262	1.128642541	0.953027340	1.336618512	19448_104_UPP1_UPI
0.2901170739	1.128328468	0.952521631	1.336583958	2514_65_EFNB3_Eph
0.2805919554	1.123981624	0.954242784	1.323913278	5609_92_FAM19A5_F
0.2964562383	1.131350934	0.951580145	1.345083695	6380_23_ICT1_ICT1
0.2929769628	1.130064751	0.952726793	1.340411910	7181_17_VAPB_VAP
0.2831945460	1.125417967	0.954196589	1.327363370	7224_11_TRAT1_TRA
0.3034044835	1.134565757	0.950369414	1.354462211	7769_29_SH3BP2_3B
0.3036530521	1.134761850	0.950461672	1.354798929	7939_1_PQLC2L_CC
0.2951306065	1.131169767	0.952537286	1.343301791	8040_9_LEMD1_LEM
0.2887688593	1.128037752	0.953315265	1.334783170	9128_34_FLRT3_FLR
0.2875298542	1.127018571	0.952773162	1.333130391	13988_67_NMRAL1_
0.2952972024	1.130664176	0.951527445	1.343525598	16810_3_GDI1_RhoG
0.2947498158	1.130293795	0.951424802	1.34279037	11430_49_DTX1_DTX
0.2969250798	1.131353521	0.951138458	1.345714474	13950_9_COL4A3BP_
0.2695790476	1.118534548	0.955481126	1.309413134	14284_23_EIF2AK4_I
0.2805001337	1.123620873	0.953717905	1.323791719	17156_72_DCLK1_DC
0.2964334884	1.131139491	0.951246128	1.345053095	7748_11_NDUFV2_N

0.3163154706	1.140320351	0.947719217	1.372063032	10575_31_PUF60_PU
0.3168401982	1.140435308	0.947413044	1.372783181	10663_42_INSIG1_IN
0.2920823575	1.128816282	0.951473670	1.339213307	11557_3_SMURF1_SM
0.2770460806	1.121730183	0.953799801	1.319227160	12568_14_KEAP1_KE
0.4420312592	1.201203394	0.927387770	1.555864374	12571_14_ARL3_ARL
0.2808082148	1.123660458	0.953491307	1.324199617	12717_65_TOX3_TOX
0.2962427100	1.130701811	0.950691478	1.344796512	14329_4_DIDO1_DID
0.2760945738	1.121372448	0.954098939	1.317972503	18909_11_EXOSC8_E
0.2852234997	1.125621844	0.952607581	1.330059262	19332_1_MOBKL3_M
0.3067950007	1.135805826	0.949224215	1.359062332	4965_27_ATP5B_ATP
0.2984572145	1.131864079	0.950539641	1.347777870	7981_230_B3GALT6_
0.3112915665	1.137996052	0.948613501	1.365187206	7982_10_MRPL32_RM
0.2755222567	1.121209114	0.954367064	1.317218421	8834_58_CANX_Caln
0.2922467009	1.128791168	0.951274984	1.339433416	11375_49_FOXL2_FO
0.2880095498	1.126784589	0.951920849	1.333770041	9028_5_MTPAP_PAP
0.2755851833	1.120847225	0.953691074	1.317301312	12885_42_NR1D2_NR
0.2790640128	1.122467635	0.953129024	1.321891959	15636_49_SORCS1_S
0.2789807366	1.122462863	0.953200295	1.321781881	17856_23_NT5M_NT5
0.2864774839	1.125908417	0.951898278	1.331728182	9751_72_YBX1_NSEI
0.2782938458	1.122015770	0.953095547	1.320874273	12341_8_DUSP6_DUS
0.2809422106	1.123245118	0.952658897	1.324377066	13125_45_VTN_Vitro
0.2979493340	1.131181890	0.949876482	1.347093534	7806_33_B4GALT7_B
0.2885589333	1.126820418	0.951458529	1.334502993	8019_73_STMN3_STM
0.5162988241	1.237957362	0.914504072	1.675813676	18271_43_OXSM_OX
0.0471765399	0.893569928	0.761673032	1.048307060	10835_25_A4GNT_A4
0.2867805495	1.125700228	0.951257947	1.332131844	9118_7_PSD2_PSD2
0.2999579861	1.131747641	0.948918903	1.349802096	15666_21_BMP2_BM
0.3045066538	1.133897856	0.948205148	1.355955882	3486_58_FGF1_b_EC
0.3259728824	1.144024489	0.944718496	1.385377800	6571_75_C17orf78_C
0.2851318401	1.124849078	0.951387254	1.329937355	9127_4_SLC26A5_S2
0.2842861674	1.124396000	0.951425244	1.328813139	18817_50_CBX1_CBX
0.2831124991	1.123831154	0.951585768	1.327254468	4267_81_PES1_PESC
0.3418774766	1.151382973	0.941811749	1.407587824	4923_79_AMH_MIS
0.2824013085	1.123435908	0.951593073	1.326310873	12718_43_PIN4_PIN4
0.2974052939	1.130389929	0.949063085	1.346360860	18878_15_GREM1_GI
0.2739960265	1.11955511	0.953006785	1.315209576	8265_225_TMPO_LA
0.2786788052	1.121654509	0.952115304	1.321382854	9450_18_CDK2AP1_C
0.2676943360	1.116533659	0.953861823	1.306947592	11162_37_EQTN_EQ

0.27934089607	1.12183719760	0.95179509540	1.32225801960	11275_94_LRP1B_LR
0.30861941273	1.13545599650	0.94691044810	1.36154408550	12815_9_IARS_SYIC
0.29130949188	1.12743905590	0.94988722220	1.33817867530	13412_5_WFDC6_WF
0.29727076520	1.13016186620	0.94880779860	1.34617974870	8409_3_RSPO2_RSPC
0.28525822634	1.12454388810	0.95075090040	1.33010545200	11681_8_AGFG1_NU
0.30262597195	1.13252075700	0.94768400670	1.35340815710	17774_38_SNRPD3_S
0.28741161950	1.12545109780	0.95023708980	1.33297277810	4468_21_SPHK2_SPH
0.28622300974	1.12494177100	0.95050632810	1.33138933490	7865_126_PLA2G16_
0.30147010941	1.13191872390	0.94777158260	1.35184470710	8864_59_CENPW_CE
0.31502173547	1.13814729640	0.94533283050	1.37028909450	12527_50_THRA_TH
0.29421914087	1.12847750890	0.94887295020	1.34207797550	12904_180_CUBN_cu
0.06009323268	0.87135187230	0.71497190770	1.06193554900	10902_53_ASPRV1_A
0.29790174639	1.13005422730	0.94802869730	1.34702943080	18392_19_MAT2B_M
0.30361821960	1.13271364380	0.94706665530	1.35475173960	18841_1_SERPINB13_
0.30403086460	1.13292480730	0.94702893020	1.35531088660	2443_10_FGF8_FGF_
0.29343070914	1.12796656800	0.94876164210	1.34102025570	5095_21_KIR2DL4_K
0.28291031275	1.12308010210	0.95050647110	1.32698614270	13242_134_SHANK3_
0.28680822640	1.12485362160	0.94980137020	1.33216871420	18405_117_AP1S2_A
0.27478588397	1.11929812860	0.95181722990	1.31624881470	13510_7_ATP2A3_AT
0.27367591949	1.11876869890	0.95197309100	1.31478863580	19197_95_ACAT1_TH
0.30726901898	1.13420829650	0.94610731450	1.35970670570	8817_29_CENPV_CE
0.29190738924	1.12705854670	0.94867877700	1.33897900800	3350_53_CAMK2A_C
0.04873505580	0.89540930270	0.76362095640	1.04994213780	5605_77_MFNG_MF
0.27157126700	1.11768954490	0.95213926270	1.31202437260	5687_5_PRKCSH_GL
0.29610156020	1.12888839950	0.94777826890	1.34460670850	19144_9_TCF4_ITF2
0.28950428795	1.12576411920	0.94877818460	1.33576516890	17721_82_GAS7_GAS
0.27892235270	1.12088331320	0.95057495790	1.32170471300	6508_68_SCGB1D2_S
0.28419201539	1.12322169280	0.94952836050	1.32868803480	16865_62_TAF15_RB
0.29205637849	1.12660848	0.94778003370	1.33917851630	6324_11_DNASE1L2_
0.33299040188	1.14553845930	0.94059670870	1.39513390770	5087_5_IL22RA2_IL_
0.28409049198	1.12288520720	0.94905588820	1.32855314870	10063_10_FANCL_FA
0.28647598345	1.12380157330	0.94834057580	1.33172618400	18308_30_STXBP6_S
0.29470191950	1.12758657490	0.94691800730	1.34272605860	18348_89_HNRNPD_
0.28806152168	1.12454651830	0.94809383210	1.33383936160	18899_82_HDGFRP3_
0.32940067400	1.14361670540	0.94081468220	1.39013473480	9402_18_DCBLD1_D
0.27510752167	1.11857088050	0.95027507870	1.31667223800	10703_203_SUDS3_S
0.27930555218	1.12043324150	0.94944783890	1.32221128660	13553_4_DCUN1D3_
0.04890902368	0.89829449860	0.76841628600	1.05012480990	3630_27_CADM3_Ne

0.28059820551	1.12098687381	0.94915863301	1.32392155261	6213_10_CGB2_CGB2
0.28301379413	1.12209576031	0.94874284531	1.32712346821	9971_5_CSMD2_CSM
0.27878521489	1.12010767461	0.94939002701	1.32152346981	12732_13_MPP7_MPI
0.32965721224	1.14350068491	0.94038252461	1.3904914	6896_3_C1orf162_CA
0.32040860206	1.13913083571	0.94187989881	1.37769057651	12695_62_KLHL12_K
0.23757080998	1.10138878661	0.95654544781	1.26816479231	2730_58_MICA_MICA
0.30185122411	1.13055671261	0.94513181991	1.35236001311	7859_21_PCDHGC3_I
0.29020968458	1.12526306341	0.94726537341	1.33670774561	8985_13_SERAC1_SR
0.27368607530	1.11759691141	0.94997031271	1.31480198861	10852_114_HNRNPDI
0.31601767580	1.13701270081	0.94250985311	1.37165450051	10880_38_FAM163B_
0.27614830201	1.11870837961	0.94951996001	1.31804331801	17857_6_ACADL_AC
0.28463054832	1.12252157271	0.94792923091	1.32927083601	7245_2_CELF2_CELF
0.30536136510	1.13202730651	0.94427186531	1.35711532851	9848_22_CCNH_Cycli
0.33371516897	1.14506248111	0.93913432331	1.39614542141	12510_3_STAP1_STA
0.28545777307	1.12277234741	0.94756864211	1.33037089671	5204_13_PSME3_PSM
0.28583269483	1.12288174511	0.94739803781	1.33086977521	7262_191_CHST14_C
0.27311571921	1.11706164731	0.94960202621	1.31405229711	11355_10_EIF5A2_IF5
0.27809581897	1.11933034821	0.94872660181	1.32061273091	4184_43_EIF4EBP2_4
0.30143141416	1.12988231301	0.94440096211	1.35179239811	18172_71_ASF1A_AS
0.27271229769	1.11679066271	0.94952434121	1.31352228701	18921_30_PHEX_PHE
0.27331464827	1.11701956291	0.94934160611	1.31431372631	3481_87_XPNPEP1_X
0.30097267561	1.12957767251	0.94432486811	1.35117242101	5250_53_IMPDH2_IM
0.27752024219	1.11884688501	0.94845297771	1.31985283551	7208_60_MGAT4C_M
0.28960020257	1.12426880691	0.94616864631	1.33589329451	8963_8_VTI1B_Vti1_r
0.27533541927	1.11775932121	0.94868044181	1.31697233861	18181_2_TSSC4_TSSC
0.29371950404	1.12606308021	0.94528916401	1.34140759141	2645_54_PRKCZ_PKC
0.28310155590	1.12118757591	0.94712458451	1.32723994401	12845_18_SNX17_SN
0.26680794076	1.11379004631	0.95002153161	1.30578963321	12626_6_SENP7_SEN
0.29649616935	1.12710352771	0.94441085001	1.34513740721	12847_27_RNGTT_M
0.28647738041	1.12258773851	0.94629172671	1.33172804441	19212_4_DMBT1_DM
0.38900632724	1.16997554141	0.92770578401	1.47551388721	5462_62_FCN3_Ficoli
0.30380303880	1.13034231701	0.94293116551	1.35500214691	10623_19_MUC1_MU
0.29617259746	1.12687791181	0.94433830831	1.34470222901	6463_59_QRFP_OX26
0.32467814019	1.13984475841	0.93904301871	1.38358525381	7638_30_LMAN2_Lec
0.27447527490	1.11695831401	0.94813642831	1.31584003931	9887_40_RBBP6_RBE
0.27434889490	1.11681277141	0.94800915701	1.31567375401	14593_152_FAM210A
0.28147400442	1.12000782131	0.94667193891	1.32508154961	7110_2_DNAJB11_DJ
0.27759740434	1.11820520121	0.94729227371	1.31995468221	11614_29_ERH_ERH

0.29989454405	1.1282982306	0.9432032066	1.3497164644	18888_37_FABP12_FI
0.2966529726	1.1267879938	0.9437341541	1.3453483457	7741_111_RTF1_RTF
0.28125480517	1.1197806774	0.9464954454	1.3247911245	13055_53_PSD_PSD1
0.0555886869	0.8918645178	0.7524123448	1.0571627694	9231_23_IMPAD1_IM
0.2818564527	1.1199360782	0.9461887255	1.3255884218	12493_42_OTUB2_OT
0.2726639459	1.1157752412	0.9478442797	1.3134587774	19166_15_RPS19_RS1
0.2930430737	1.1249566555	0.9440708518	1.3405005295	4905_63_COTL1_Coa
0.3100526854	1.1326338071	0.9408597080	1.3634969487	15525_294_ADH1C_A
0.2812046494	1.1195110736	0.9460871851	1.3247246804	11178_21_SVEP1_SV
0.2709501051	1.1148788917	0.9479452405	1.3112096460	9758_17_RPS4X_RS4
0.2683383569	1.1134054788	0.9479137879	1.3077895648	17743_14_UBE2A_UI
0.2781393774	1.1177670315	0.9460371589	1.3206702560	5345_51_CKAP2_CKA
0.2744281586	1.1160795333	0.9466897027	1.3157780433	17806_6_SDCBP_Syn
0.2915311191	1.1236765901	0.9433488189	1.3384752849	19249_18_KARS_SYK
0.2701527409	1.1140840795	0.9473491975	1.3101645511	7873_32_TSSK2_TSS
0.2713875135	1.1144994843	0.9468858881	1.3117833056	12347_29_CCM2_CCI
0.2660886967	1.1121634163	0.9479301959	1.3048507895	16851_50_SCO2_SCC
0.3013267350	1.1279182757	0.9412190942	1.3516509010	8520_8_ADAM30_AD
0.0519636596	0.9017478814	0.7719741014	1.0533374631	9297_12_B3GNT8_B3
0.2822872390	1.1193056706	0.9447167548	1.3261595901	13229_20_MDM4_MI
0.2648818394	1.1115562451	0.9480389156	1.3032769707	19260_4_GIPC1_GIPC
0.2712831741	1.1143569492	0.9467424836	1.3116464421	19290_5_HPRT1_HPF
0.3028077139	1.1284370593	0.9406909410	1.3536541506	11319_106_MRE11A_
0.2670191333	1.1124204232	0.9474863696	1.3060654355	6399_52_DEFB107A_
0.2734737589	1.1152294747	0.9461507403	1.3145228642	11646_4_CHST9_Carb
0.2675026916	1.1125645914	0.9472737987	1.3066971469	16293_1_GHRH_Grov
0.2668831970	1.1122991019	0.9474084924	1.3058879058	6912_6_EFHA2_EFHA
0.2667712823	1.1121573967	0.9472731188	1.3057417660	17793_4_DIRAS1_DII
0.2981191376	1.1261079030	0.9412142987	1.3473222951	11425_31_RUFY1_RU
0.3162834343	1.1341888035	0.9375848068	1.3720190778	8352_26_SIGLEC12_S
0.2731320241	1.1147243084	0.9456168723	1.3140737227	10045_47_CUL3_CUL
0.2627587579	1.1101326488	0.9476218633	1.3005129427	10432_3_KIAA1644_I
0.2662999510	1.1116446197	0.9468459839	1.3051264740	12396_19_HIBCH_HI
0.2652589639	1.1112486103	0.9471569654	1.3037685610	13403_5_ETV5_ETV5
0.2863788535	1.1205524605	0.9429564408	1.3315968399	14006_36_GNMT_GN
0.2745894651	1.1152715989	0.9451671001	1.3159903041	7857_22_NTS_NEUT
0.3001868515	1.1266878001	0.9402377640	1.3501110543	9044_1_CNTNAP5_C
0.2916203033	1.1228547037	0.9418853385	1.3385946612	9738_7_FAF2_FAF2

0.26608113415	1.1114277505	0.9466837101	1.3048409215	12034_28_CAP1_CA
0.29962347746	1.1263329264	0.9401750837	1.3493506509	4304_18_LCORL_tran
0.28882217770	1.1215508078	0.9423321904	1.3348543405	6936_7_PMEPA1_PM
0.26861555396	1.1124413189	0.9460105283	1.3081521304	8081_55_PLD5_PLD5
0.27706426285	1.1161884511	0.9443817132	1.3192511471	9536_16_EDDM3A_E
0.29126128201	1.1223978306	0.9414569582	1.3381141634	10435_2_AGA_ASPG
0.28933459249	1.1215555678	0.9418574438	1.3355385149	6151_18_MAP2K3_M
0.29537590757	1.1242645962	0.9407125600	1.3436313449	7932_23_SYT8_SYT8
0.26639900815	1.1113013706	0.9461676185	1.3052557624	18316_75_SEPT3_SE
0.30152466649	1.1268714994	0.9392869555	1.3519184618	8866_53_QPCTL_QP
0.27162032916	1.1134217407	0.9448354600	1.3120887448	10752_8_CDYL2_CD
0.26783369751	1.1116910559	0.9454738601	1.3071297430	18415_16_ARL6_AR
0.27430652137	1.1145743611	0.9442528161	1.3156180054	18876_77_CHST4_CH
0.26775574536	1.1116487714	0.9454756360	1.3070278534	7020_13_CHST5_CH
0.28670533881	1.1199953660	0.9417115672	1.3320316576	9058_1_TIMM21_TI
0.28221637163	1.1179358681	0.9424726755	1.3260656119	18204_1_TCEA1_TC
0.27167105667	1.1132712541	0.9445321602	1.3121553055	2839_2_TNFSF4_OX
0.27220514367	1.1134850276	0.9443904171	1.3128562978	12401_3_STAMBPL1
0.28312555834	1.1182635514	0.9421682651	1.3272718014	4123_60_FGF4_FGF_
0.28426508788	1.1186090893	0.9416769254	1.3287851289	11155_16_COL6A5_C
0.26898444034	1.1118696201	0.9446898951	1.3086347789	17778_19_RAB32_RA
0.30996932987	1.1300605999	0.9366675982	1.3633832983	18417_3_AS3MT_AS
0.28659142172	1.1196436654	0.9412274440	1.3318799250	2449_1_PLA2G5_GV
0.26934201868	1.1120573576	0.9446710866	1.3091028021	4906_35_F5_Coagula
0.25263161768	1.1047655348	0.9480335718	1.2874089307	6444_15_PSG3_PSG3
0.27712077630	1.1155169648	0.9431924917	1.3193257046	6649_51_NTN1_NET1
0.28758831692	1.1201882875	0.9412045887	1.3332083318	9719_145_MMP16_M
0.28200072462	1.1175556560	0.9420348371	1.3257796806	11143_32_OLFM3_N
0.26685783612	1.1108959518	0.9450436813	1.3058547877	6357_83_CELA3B_E
0.30774342598	1.1288349235	0.9367195887	1.3603519132	6221_1_ADPGK_AD
0.28624017833	1.1193177678	0.9410100582	1.3314121932	6400_33_TP53I13_P5
0.29265075320	1.1220950151	0.9396425155	1.3399747268	3067_67_GDF9_GDF_
0.27934417588	1.1162023950	0.9422545993	1.3222623563	3477_63_NTRK1_Trk
0.28743593654	1.1195813649	0.9403282446	1.3330051925	10006_25_ELK1_ELK
0.26964229818	1.1117554170	0.9438747021	1.3094959578	13613_23_AK5_Aden
0.26863642379	1.1113079912	0.9440642632	1.3081794316	9445_44_C19orf18_C
0.29904221260	1.1245090178	0.9376775157	1.3485665487	11334_7_LILRB3_LI
0.28361373924	1.1176895445	0.9407419153	1.3279199083	12871_10_TWF1_TW

0.28405369675	1.1178956690	0.9406749838	1.3285042652	3623_84_LY86_LY86
0.2667267283	1.1102362325	0.9440453264	1.3056835912	3312_64_FCGR1A_FC
0.2651303761	1.1095046638	0.9443078607	1.3036009231	5610_32_FAM171A1_
0.2646659058	1.1092072904	0.9442402047	1.3029955798	17345_12_ZADH2_ZA
0.2688777625	1.1109047574	0.9431516409	1.3084951841	7104_71_CABP7_CAI
0.2862749224	1.1183805290	0.9394022060	1.3314584527	11117_2_SPATA20_S
0.2642212900	1.1086753712	0.9437543180	1.3024163761	7786_83_CLEC2B_CI
0.2781992468	1.11470038	0.9407969573	1.3207493260	6622_90_APLN_APEI
0.2702537956	1.1110644148	0.9421254685	1.3102969562	11348_132_P4HA2_P4
0.2805699297	1.1154755169	0.9398750325	1.3238841182	6413_79_LIPK_LIPK
0.2539318430	1.1039044637	0.9453263873	1.2890839412	3592_4_CAMK1_CAN
0.3348147706	1.1390312448	0.9282459594	1.3976814690	14636_25_HRSP12_R
0.2870748255	1.1179881918	0.9379926179	1.3325239165	3896_5_PGAM1_Phos
0.2741744454	1.1124012041	0.9406985008	1.3154442554	9828_86_LDHC_LDH
0.2942821378	1.1209957220	0.9362736518	1.3421625250	10990_21_LRRK2_LR
0.2753899900	1.1128030248	0.9402346283	1.3170442088	11110_4_TMEM119_
0.2664594993	1.1089310726	0.9420787663	1.3053347213	14655_1_DNAJC17_D
0.2833509906	1.1161808348	0.9384504585	1.3275710451	9574_11_BIN1_BIN1
0.2705209337	1.1105946043	0.9410774555	1.3106470331	9482_110_NUDT9_NU
0.2973364821	1.1219846957	0.9350660145	1.3462682184	8328_9_ETNK1_EKI1
0.2762490793	1.1128292687	0.9394715403	1.3181761535	12356_65_SRI_Sorcin
0.2760825060	1.1126472827	0.9393207461	1.3179565988	11571_75_KLRC3_NK
0.2555763446	1.1037822988	0.9435641981	1.2912055858	8973_23_FCRL4_FCR
0.2546646311	1.1033110946	0.9436186736	1.2900289128	7178_59_DEPP_DEPP
0.2645750719	1.1074530207	0.9413413372	1.3028772290	14273_19_PREP_PPC
0.2671467285	1.1085196977	0.9407332173	1.3062320938	17343_6_SNPH_SNPH
0.2897641079	1.1181033414	0.9356661919	1.3361122726	12692_56_DOT1L_DO
0.2820316134	1.1147874241	0.9373447433	1.3258206330	17696_1_TICAM2_TI
0.2811959779	1.1144912845	0.9376299939	1.3247131931	19142_39_DNMT3L_I
0.2756622839	1.1120798380	0.9387572961	1.3174028808	6464_40_PVRIG_PVR
0.2589776843	1.1049546280	0.9423588451	1.2956048922	8446_4_ADCYAP1_P
0.2715548083	1.1102271751	0.9394830564	1.3120027785	4693_72_HIBADH_3H
0.2714765266	1.1101822548	0.9394805753	1.3119000768	7097_8_CDY1_CDY1
0.2756222815	1.1118523219	0.9384107598	1.3173501825	10024_44_HOGA1_H
0.2655303010	1.1074595119	0.9404535946	1.3041223698	8877_22_EVA1C_F17
0.0651850029	0.8974934597	0.7546630697	1.0673564702	10785_8_LRRC52_LR
0.2669920032	1.1080170146	0.9400256521	1.3060300023	2987_37_HIST1H1C_
0.2770306941	1.1122947814	0.9378359953	1.3192068623	3452_17_LCK_LCK

0.27448471478	1.11118820633	0.93835689544	1.31585246088	4566_24_RSPO2_RSP
0.26016544508	1.10496073559	0.94125061877	1.29714467511	12783_29_HNRNPM_
0.26887060940	1.10869627459	0.93941211000	1.30848582433	13240_170_SHANK1_
0.27262147264	1.11026666300	0.93854823755	1.31340299177	3003_29_NCR3_NKp3
0.26919279199	1.10881631077	0.93931285844	1.30890746355	4282_3_RAN_RAN
0.27177864694	1.10982822355	0.93859786777	1.31229648833	5493_17_WNK3_WNI
0.27057841238	1.10897563700	0.93828181477	1.31072236955	10043_31_BRD4_BRI
0.27917839819	1.11258731277	0.93631626699	1.32204317299	10362_35_MYC_c_My
0.27457719496	1.11083932988	0.93768104055	1.31597415677	10442_1_TMEM190_T
0.26382095820	1.10597438055	0.93953756144	1.30189508177	11312_40_PMS2_PMS
0.29790877359	1.12091285300	0.93274635722	1.34703889677	12363_70_TRIB2_TRI
0.28279365487	1.11418332055	0.93561587400	1.32683134833	12670_15_RAD1_RAI
0.27439688992	1.11064907988	0.93752890655	1.31573690133	12676_1_PACSIN1_PA
0.27874819204	1.11264148860	0.93681038944	1.32147454411	12712_9_HMG20A_H
0.28395991753	1.11463897422	0.93528985500	1.32837968499	13109_82_NEGR1_NI
0.27023369772	1.10871482533	0.93816387477	1.31027062222	15316_262_TXNL4B_
0.26577031920	1.10711478011	0.93964263544	1.30443542055	18215_5_THG1L_THO
0.27423117362	1.11038889411	0.93724500244	1.31551888033	2747_3_ULBP3_ULBI
0.27024275000	1.10875583811	0.93822479088	1.31028248322	4271_75_PFDN5_PFD
0.27893413665	1.11258484088	0.93654083933	1.32172028800	4292_5_NAPA_SNAA
0.28452270884	1.11475597022	0.93495987177	1.32912749599	4557_61_KIRREL3_K
0.28483765117	1.11511882855	0.93527403477	1.32954616033	5114_65_PRLR_Prola
0.27983234309	1.11316739000	0.93668013133	1.32290799900	6290_3_UTS2B_UTS2
0.26333231671	1.10586483655	0.93981057122	1.30125907722	7778_104_C9orf89_BI
0.29240568975	1.11820806488	0.93337263277	1.33964638822	7920_30_ST8SIA2_SI
0.26059108063	1.10490337588	0.94075239500	1.29769690355	9045_3_SLC27A2_S27
0.26402426844	1.10605520611	0.93948386488	1.30215979733	9257_14_TACO1_TAO
0.28638358079	1.11589836433	0.93513534711	1.33160313460	9853_3_IFIT2_IFIT2
0.26965772467	1.10883100677	0.93890113011	1.30951615899	9897_9_MCM6_MCM
0.29008108898	1.11706195559	0.93362808199	1.33653586199	5033_27_TPM1_Tropo
0.26719062508	1.10732529000	0.93866586311	1.30628943411	6424_2_RNASE13_RN
0.26267881379	1.10520458600	0.93930232611	1.30040897844	13603_7_RFESD_RFE
0.26544957840	1.10632509700	0.93860365600	1.30401710199	19110_6_MEMO1_MI
0.26876168101	1.10764312755	0.93773040855	1.30834330088	11638_42_B3GALT2_
0.27505998625	1.11029661822	0.93631288477	1.31660965099	14008_22_DCP1A_DO
0.27753037878	1.11134132088	0.93576115360	1.31986621444	9249_17_TMEM9_TM
0.26873582682	1.10751936644	0.93754510711	1.30830947511	12637_7_CRAT_CAC
0.27479498879	1.11000767188	0.93607363560	1.31626079899	12954_71_PPARA_PP

0.26827758304	1.1072744771	0.9375600748	1.3077100877	15405_23_IFNA4_IFN
0.27990690562	1.1120239201	0.9346870677	1.3230066421	12459_13_PLEKHA1_
0.26668863667	1.1063960126	0.9375615764	1.3056338565	11129_66_BMP15_BM
0.27102315210	1.1082255190	0.9365962905	1.3113054295	8225_86_EPHB2_EPH
0.28257988769	1.1130078565	0.9338423684	1.3265477456	12367_52_TMEM57_
0.29652865807	1.1186213909	0.9302195869	1.3451811097	5963_9_DMKN_Derm
0.06934708644	0.8981435298	0.7526177111	1.0718081546	8033_1_DNAJC18_DJ
0.26799854699	1.1065885261	0.9366601325	1.3073452404	8250_2_PTPRJ_PTPR
0.27644870102	1.1100458683	0.9345912356	1.3184393163	11543_84_LIMA1_LIM
0.27963750304	1.1113837316	0.9338627361	1.3226502687	9999_1_IRF6_IRF6
0.26441480280	1.1049191277	0.9371888089	1.3026684347	6586_19_ADAM11_A
0.25632948802	1.1014873745	0.9389372411	1.2921784151	8565_160_PLVAP_PL
0.26317107980	1.1043245371	0.9373454942	1.3010492831	9778_45_TMEM52B_
0.28040931693	1.1115201049	0.9333712640	1.3236715026	9934_29_PITPNA_PI
0.25909762338	1.1024927376	0.9380517686	1.2957602951	11107_25_TENM3_T
0.32252358899	1.1291170232	0.9234379005	1.3806074576	11288_26_NT5C2_5N
0.26455031380	1.1047560698	0.9367852656	1.3028449726	17436_193_CSNK1G2
0.26558836779	1.1051933493	0.9365543019	1.3041980982	19121_3_UBQLN2_U
0.27718235697	1.1100429614	0.9339009273	1.3194069521	5110_84_NRXN1_NR
0.28031216654	1.1112583810	0.9330224027	1.3235429136	9081_39_SLC30A3_Z
0.28758287855	1.1141994371	0.9311726514	1.3332010813	3881_49_DYNLL1_D
0.25844686224	1.1019666307	0.9377667737	1.2949173390	8945_7_FAM175B_F1
0.26775550937	1.1057015031	0.9353864184	1.3070275449	8035_6_C1orf198_CA
0.26556337275	1.1047354515	0.9358017963	1.3041655001	11566_48_KRT72_Ke
0.26317535560	1.1036982917	0.9362786840	1.3010548462	11444_49_POLR3F_R
0.26832023561	1.1058486760	0.9351072129	1.3077658661	12442_4_RHOD_RHO
0.25445724284	1.1000400363	0.9382263093	1.2897614036	7824_88_MSRB3_MS
0.26544190763	1.1045336979	0.9355736564	1.3040070991	13535_2_COL4A3BP_
0.27947319044	1.1103517043	0.9322823508	1.3224329584	19276_124_SNRPG_R
0.29117622903	1.1151565550	0.9294273616	1.3380003576	12801_33_IRF2_IRF2
0.27205592420	1.1071627223	0.9338358082	1.3126604087	10885_36_C6orf89_C
0.27399590520	1.1078534945	0.9331893079	1.3152094167	12361_102_RRAS2_R
0.26912641551	1.1058664722	0.9343837250	1.3088205858	18416_3_ARL9_ARL
0.26705712033	1.1049880545	0.9348323493	1.3061150499	6407_63_LAD1_LAD
0.25757669107	1.1010864932	0.9370844580	1.2937910293	8687_26_TMEM106B_
0.25878209907	1.1014498307	0.9365733662	1.2953515157	18945_11_RGS1_RGS
0.28450909877	1.1120895863	0.9305052254	1.3291094065	2999_6_LSAMP_LSA
0.26089103930	1.1023499323	0.9361283996	1.2980862173	9202_309_ATP5O_AT

0.2994837545	1.118240459	0.926843185	1.349162128	10070_22_PLCG2_PL
0.2645088334	1.103701073	0.935035722	1.302790931	10447_18_PABPC3_P
0.2815848292	1.110757412	0.930995759	1.325228409	9874_28_CDKN2B_p1
0.2643569288	1.103585545	0.934982002	1.302593046	19175_18_MARCKSL
0.2577388389	1.100806355	0.936455836	1.294000831	13958_5_RBM46_RB
0.2588991129	1.101299057	0.936207420	1.295503098	18435_40_UBXN2B_
0.2643162417	1.103471680	0.934827110	1.302540048	18317_111_ZFAND5_
0.2646446685	1.103568688	0.934684456	1.302967907	11431_235_RECQL_
0.2583100551	1.100839885	0.935978086	1.294740197	12761_12_APBB2_A
0.2688263439	1.105177199	0.933499383	1.308427904	13497_34_EIF3J_EIF
0.2643237395	1.103389168	0.934680304	1.302549814	16875_13_MAGOHB_
0.2854420743	1.111997428	0.929483421	1.330350011	17671_58_ING4_ING
0.2777542120	1.108875583	0.931404903	1.320161677	3892_21_MED1_MED
0.2880346257	1.113128029	0.928962940	1.333803487	6933_20_MRPL34_R
0.2657046098	1.103901748	0.934257938	1.304349709	7959_34_CDH7_CAD
0.2773018480	1.108592443	0.931350528	1.319564618	2975_19_CTGF_CTG
0.2542759605	1.099104763	0.936801405	1.289527613	8297_8_DNAJC10_DJ
0.2713792383	1.106102084	0.932678393	1.311772450	8839_4_MTX2_Metax
0.2576057733	1.100480559	0.936026155	1.293828656	9779_63_HERPUD2_
0.2975278419	1.116681037	0.926069503	1.346525864	10705_14_ST6GALN
0.2561232407	1.099637252	0.935978725	1.291911934	10961_15_RARRES3_
0.2742634022	1.106884776	0.931308886	1.315561278	11327_56_CLK2_CL
0.2635941277	1.102664382	0.934134081	1.301599805	11328_9_USB1_USB1
0.0674797075	0.908238801	0.771070412	1.069808550	12605_1_EXOSC3_E
0.2770642966	1.107904551	0.930416059	1.319251191	13975_56_PPFIA1_L
0.2673247705	1.104318377	0.933449711	1.306464678	14619_8_ZNF526_ZN
0.2582564822	1.100449187	0.935363939	1.294670836	15306_20_IGJ_IgJ
0.2619323665	1.102132106	0.934784552	1.299438653	15414_316_LDHA_L
0.2601310791	1.101193944	0.934876271	1.297100098	15524_30_PGAM2_P
0.2718476197	1.106030114	0.932120335	1.312387004	16298_84_PLA2G2D_
0.2777464553	1.108306586	0.930456503	1.320151437	16304_6_LGR4_LGR
0.2806781161	1.109659221	0.929998603	1.324027352	18322_15_JMJD6_JM
0.2817312424	1.109812574	0.929276508	1.325422454	19147_6_LGALS13_P
0.2531258991	1.098205255	0.936344911	1.288045430	19255_124_NXT2_N
0.2679866511	1.10422281	0.932670637	1.307329688	3197_70_IDE_IDE
0.2835971746	1.110719041	0.929059966	1.327897912	7916_10_S100A7_S10
0.2989711326	1.117155657	0.925520122	1.348470696	8106_15_SSR1_SSRA
0.2555204288	1.099204781	0.935806604	1.291133389	8804_39_COL20A1_C

0.30112114621	1.11797348631	0.92488504231	1.35137304531	8852_10_SUN3_SUN3
0.28068006786	1.10939679621	0.92955696660	1.32402993644	9341_1_PDGFD_PDG
0.25705393224	1.09976337771	0.93532254400	1.29311486544	9486_13_DEFB125_D
0.29509389848	1.11526339200	0.92597069431	1.34325248211	5654_70_TMX3_TMX
0.27042437570	1.10507020300	0.93182835900	1.31052048577	13704_5_HMGCS2_H
0.27952918168	1.10881390855	0.92964973260	1.32250700521	7895_108_MRAP_MR
0.26030908912	1.10091454755	0.93423561631	1.29733101560	12333_87_RPIA_RPIA
0.29113221381	1.11349203831	0.92669563688	1.33794146660	4254_6_NUDCD3_NU
0.25699105193	1.09953157144	0.93498708560	1.29303355655	9997_12_UBXN4_UB
0.25520384190	1.09878478831	0.93538770360	1.29072469788	5903_91_HSPA8_HSP
0.25526261132	1.09873887060	0.93525456044	1.29080055521	5653_23_PRSS37_PRS
0.25371810360	1.09809046711	0.93559495277	1.28880844260	19262_219_ACADVL_
0.25318071546	1.09772480155	0.93547452560	1.28811603822	7742_11_RNF148_RN
0.26750236558	1.10341197999	0.93175254677	1.30669672099	12667_2_GDA_GUAD
0.26651311311	1.10289948421	0.93180855388	1.30540470711	18901_26_HSPA1B_H
0.26136761740	1.10081712388	0.93308205955	1.29870500421	19230_12_GSTT1_GS
0.26371558221	1.10172362344	0.93242755977	1.30175790055	17747_45_TRAF1_TR
0.28698612331	1.11117511060	0.92667729077	1.33240572399	19602_36_JUND_jun_
0.26196966259	1.10094598011	0.93273879655	1.29948711877	15383_200_EDN3_En
0.27261006405	1.10520440777	0.93001974700	1.31338800777	17348_5_SYNJ2BP_S
0.25589454359	1.09839150377	0.93407283400	1.29161651155	8296_117_KDELC2_K
0.25173497366	1.09663095533	0.93496184955	1.28625510060	6228_58_SPACA1_sp
0.26276418625	1.10102117299	0.93212531999	1.30052000222	10977_55_UCMA_UC
0.24972570582	1.09568658600	0.93522949200	1.28367326422	12395_86_DARS2_SY
0.26179367032	1.10049725100	0.93214264611	1.29925843922	12804_5_GMEB2_GM
0.26138671442	1.10032721855	0.93223392760	1.29872980588	17769_28_PCNP_PCN
0.27249281862	1.10484564200	0.92952502500	1.31323402800	19109_32_TCEAL8_T
0.25812823254	1.09874140633	0.93258261600	1.29450480560	11626_7_UBE2V1_UI
0.28402530488	1.10917720922	0.92608585755	1.32846654700	12409_90_RAB7B_RA
0.26302099776	1.10070504922	0.93135092333	1.30085403360	18307_71_PPA2_PPas
0.26295666880	1.10070107855	0.93140411811	1.30077035377	3868_8_SGTA_SGTA
0.26021834143	1.09962781288	0.93213763311	1.29721329111	6653_58_CD47_CD47
0.29111867329	1.11209040060	0.92437661611	1.33792335022	7050_5_NEGR1_NEG
0.25721202864	1.09832568460	0.93273122299	1.29331931844	12843_6_ZNF410_ZN
0.27656889310	1.10604365599	0.92775262977	1.31859779188	17200_50_KIR2DL1_I
0.08055236972	0.89742609944	0.74304299088	1.08388560799	17711_13_VAMP1_V
0.26378365777	1.10092972244	0.93102084900	1.30184652144	7007_24_TRAPPC4_T
0.25670980633	1.09806344111	0.93275419833	1.29266994760	8065_245_PATE4_PA

0.29447818423	1.11324930711	0.92319749332	1.34242567706	8263_64_TYMSOS_C
0.28593789906	1.10968664560	0.92516558133	1.33100979577	6414_8_OAF_OAF
0.26742326576	1.10216108422	0.92971469744	1.30659336555	7861_9_ROR2_ROR2
0.27406054810	1.10461999922	0.92768988222	1.31529443844	15669_7_BRAF_BRA
0.25976934611	1.09882139433	0.93118896277	1.29663097922	11405_150_CARD9_C
0.26069439661	1.09907386244	0.93075552255	1.29783098322	14035_13_TIAM1_TIA
0.24764221649	1.09392130411	0.93416267855	1.28100152899	3201_49_KLK5_kallik
0.26880256050	1.10230691260	0.92867893160	1.30839678633	3202_28_KREMEN2_
0.26342554243	1.10018986660	0.93010294955	1.30138039377	5443_62_NPPA_ANP
0.26566300292	1.10098202744	0.92936108400	1.30429544088	13481_24_TCEA2_TC
0.26325331663	1.10003431522	0.93000011722	1.30115628177	9884_8_PPIL1_PPIL1
0.26267353089	1.09972752000	0.93002049933	1.30040210855	10910_6_CEACAM4_
0.25963284803	1.09850136544	0.93077366911	1.29645400366	11448_34_GALK1_GA
0.25859493598	1.09809871511	0.93105730744	1.29510909644	12720_71_UBQLN4_U
0.25205283486	1.09546311338	0.93267505644	1.28666401622	5359_65_PIM1_PIM1
0.26173108897	1.09926442233	0.93011356199	1.29917713233	9030_56_C17orf89_C
0.26366834959	1.10000748544	0.92956887088	1.30169641655	9957_9_RNF114_RN1
0.27791354624	1.10560522799	0.92577158870	1.32037204111	14334_3_RCVRN_RE
0.25639647139	1.09706544	0.93135123599	1.29226497244	2877_3_UBE2I_UBC9
0.28422486288	1.10813274788	0.92415813199	1.32873167966	8390_25_COX7A1_C
0.27075475507	1.10261163499	0.92738025655	1.31095352622	12513_8_GLTP_GLTP
0.26045201360	1.09852875744	0.93005790533	1.29751644922	15323_112_AGO3_AG
0.06008602005	0.92501754644	0.80575853544	1.06192788977	16828_8_COL6A1_Co
0.25615803863	1.09671988377	0.93098656144	1.29195689099	18165_181_UBE2V2_
0.28320720824	1.10742875355	0.92392403099	1.32738017744	9863_1_TPM4_Tropon
0.24788115699	1.09337265888	0.93300291499	1.28130764866	11716_28_LRIT2_LRI
0.07278570506	0.91082833611	0.77136980800	1.07550004	3323_37_LRP8_LRP8
0.25579345088	1.09632508222	0.93065564544	1.29148594511	17332_3_ADPRHL2_A
0.25463183052	1.09587694899	0.93097578488	1.28998659977	2846_24_RPS27A_Ub
0.25496968981	1.09597355377	0.93082538766	1.29042250733	17786_5_GGPS1_GGP
0.25737559970	1.09664787055	0.92973160955	1.29353088533	11636_33_TAGLN2_T
0.26659194920	1.10044224022	0.92758793711	1.30550762422	12428_2_LYPLAL1_L
0.26829494633	1.10091422544	0.92680411260	1.30773279411	12498_12_TAX1BP3_
0.26980607988	1.10164383760	0.92663164433	1.30971044699	12878_60_OSBPL11_
0.26367603320	1.09915529099	0.92812199177	1.30170641833	16753_46_COL6A2_C
0.25432224302	1.09563597299	0.93085453555	1.28958729788	16899_59_OSBPL9_O
0.26987692034	1.10158499760	0.92646702844	1.30980323060	17454_15_EGFL6_EG
0.25770411664	1.09698049811	0.92999012688	1.29395590199	17799_9_PGLS_6PGL

0.2566662164	1.096552446	0.930229471	1.292613601	18188_12_GATM_GA
0.2779278037	1.104736050	0.924303381	1.320390866	6543_182_PRLH_PRR
0.2702168538	1.101967972	0.926796225	1.310248552	7207_4_IK_RED
0.2654795179	1.099877623	0.927667717	1.304056144	7892_132_KIAA1467_
0.2752711005	1.103707941	0.925038087	1.316887635	8828_21_ESRP1_ESR
0.2731444842	1.102990470	0.925802562	1.314090096	9600_55_THSD7A_T
0.0726550867	0.911780408	0.773084221	1.075359567	9841_197_PAICS_Mu
0.2727787749	1.102591070	0.925470596	1.313609609	14341_8_FAM69C_F
0.2732181406	1.102781914	0.925384326	1.314186891	9187_2_HMGN1_HM
0.2843343052	1.107098244	0.922332483	1.328877107	12529_32_FKBP6_FK
0.2570231185	1.096235962	0.929360838	1.293075020	12747_89_RBM3_RB
0.2707875947	1.101576991	0.925610247	1.310996578	12893_159_IFI16_IP1
0.2556606902	1.095616269	0.929576034	1.291314497	13438_115_CHRD_C
0.2688882452	1.100779186	0.926027187	1.308508900	13727_44_SPOP_SPO
0.2632730826	1.098526898	0.927434706	1.301182	12846_3_FBXL5_FB
0.2502377308	1.093355269	0.930777205	1.284330705	14674_63_PACSIN3_
0.2490096732	1.092869088	0.931092347	1.282754441	9794_17_ADCK4_AD
0.2635677051	1.098501978	0.927119438	1.301565414	10075_75_ACBD6_A
0.2544302854	1.094945820	0.929581754	1.289726635	5253_1_PDE1A_PDE1
0.2668867677	1.099713103	0.926086065	1.305892568	11661_11_NLRP1_NA
0.2533725391	1.094390406	0.929621713	1.288363153	16621_77_APOA1BP_
0.0759227569	0.910900111	0.769074968	1.078879235	15522_2_GLIPR2_GA
0.2557178100	1.095068643	0.928593956	1.291388259	18265_18_ZNF34_ZN
0.2603102271	1.096780018	0.927230619	1.297332491	17336_54_CREB3L2_
0.0743436685	0.913317419	0.774384116	1.077176933	2952_75_IGF1_IGF_I
0.2830174718	1.105534630	0.920940932	1.327128349	14615_46_KRTAP2_4
0.2561597986	1.095023325	0.928106797	1.291959164	19193_18_VBP1_PFD
0.2696967005	1.100241230	0.924374684	1.309567199	18922_27_CD68_CD6
0.2725827982	1.101379390	0.923618634	1.313352197	9057_19_ADRM1_AD
0.2563791382	1.095019793	0.927897264	1.292242573	13474_40_GSTK1_GS
0.2737023387	1.101684305	0.923096086	1.314823372	12406_119_DIRAS3_
0.2730166248	1.101396625	0.923246924	1.313922088	19236_24_SUB1_TCP
0.2809230884	1.104523653	0.921184653	1.32435174	5707_55_ELK3_ELK3
0.2628728393	1.097415049	0.925928815	1.300661315	10866_60_SMEK1_P4
0.2509661880	1.092738061	0.929049620	1.285266626	5703_26_OLFM1_NO
0.2503862714	1.092440345	0.929082084	1.284521494	10605_22_APMAP_A
0.2618342730	1.09686759	0.925966406	1.299311193	12743_18_BAG5_BA
0.2501259919	1.092363254	0.929192781	1.284187203	9774_59_RNF150_RN

0.25461645813	1.0940418707	0.9278747662	1.2899667697	13377_3_CYB561D1_
0.25172598681	1.0928897771	0.9286017979	1.2862435413	19228_11_BAG2_BAG
0.26033289885	1.0961893176	0.9262111176	1.2973619050	11128_29_TMEM132
0.25852076800	1.0954307205	0.9266072405	1.2950130444	10040_63_TP63_P73L
0.24595128260	1.0905113819	0.9299189988	1.2788372703	11180_17_SMARCC1_
0.26291585433	1.0969756559	0.9251477030	1.3007172646	5236_2_NR1D1_NR1I
0.31095113002	1.1156957028	0.9121098816	1.3647225255	6626_81_CHST12_CH
0.26908709023	1.0993095735	0.9233725969	1.3087691170	10621_26_PRR16_PRI
0.25472719447	1.0937137459	0.9272155915	1.2901096238	7826_1_DCLK3_DCL
0.25115674449	1.0923280832	0.9281757344	1.2855115654	9900_36_NEFH_NFH
0.24873551735	1.0913673855	0.9287898906	1.2824028149	5957_30_SST_Somato
0.26146987688	1.0962017975	0.9251797000	1.2988378159	17514_48_RAB21_RA
0.24609292437	1.0901935763	0.9292454396	1.2790184200	11279_42_GABBR1_G
0.27780281431	1.1023692025	0.9204621059	1.3202258419	2962_50_PTHLH_PTH
0.25931364795	1.0952073356	0.9254952660	1.2960402414	10003_15_ZNF41_ZN
0.25340499612	1.0929322732	0.9271160710	1.2884049702	2972_57_BMP7_BMP
0.25383277572	1.0930633015	0.9269417709	1.2889562414	19335_2_HN1_HN1
0.29360258878	1.1083911807	0.9159592205	1.3412507696	3168_8_ADAMTS5_A
0.25996853335	1.0953464014	0.9251242648	1.2968892772	10667_78_C1orf185_C
0.25381168335	1.0929944622	0.9268445692	1.2889290546	5460_60_DDX19B_DI
0.25222830793	1.0923658833	0.9272458394	1.2868898109	6706_18_ALPPL2_PPI
0.25295830581	1.0925749041	0.9269238251	1.2878295807	11154_3_NFE2L1_NF
0.27331315936	1.1003828499	0.9212748790	1.3143117694	12557_18_NOVA1_NO
0.25518035548	1.0933444030	0.9261696640	1.2906943836	13090_17_S100A6_S1
0.27247548509	1.0998629796	0.9211759036	1.3132112652	14254_27_PTPN4_PTI
0.05896235304	0.9338297792	0.8221071282	1.0607353065	4964_67_ERAP1_ART
0.26415321165	1.0967899651	0.9236908774	1.3023277128	5532_53_FGFR1_bFG
0.26505307449	1.0970168508	0.9232419076	1.3035001565	5677_15_C2orf66_CB
0.24821472100	1.0906257504	0.9280111863	1.2817351180	6117_4_PCSK2_NEC2
0.25070568626	1.0915217858	0.9272241199	1.2849318555	6284_7_FAM150B_F1
0.27657611092	1.1014891025	0.9201209749	1.3186073093	8100_15_ADM2_ADM
0.26019387851	1.0950383918	0.9243957195	1.2971815579	8005_1_MXRA7_MXI
0.25848587904	1.0943564292	0.9248229456	1.2949678635	4973_18_BIRC3_cIAP
0.26154032102	1.0954366050	0.9238234471	1.2989293146	14249_68_MID2_TRII
0.24764590239	1.0900758764	0.9276031368	1.2810062500	19258_24_GCDH_GC
0.24558782942	1.0892552421	0.9281151849	1.2783725573	16837_20_CFLAR_FL
0.26980315560	1.0984698205	0.9213024741	1.3097066169	3351_1_CAMK2B_CA
0.25156028400	1.0914772079	0.9263563851	1.2860304247	8803_61_PVRL1_nect

0.26999727648	1.0982973171	0.9208343641	1.3099608830	8923_94_GALNT16_C
0.25926773533	1.0941293088	0.9237166179	1.2959807382	10361_25_OAS1_OAS
0.26629110105	1.0968041996	0.9217421627	1.3051149237	7945_10_SEMA6A_Se
0.26359105047	1.0957333550	0.9224304387	1.3015958004	14156_33_YWHAB_1
0.26155813266	1.0948200887	0.9227674391	1.2989524509	10560_1_ITM2C_ITM
0.25178068774	1.0911150559	0.9255377428	1.2863139019	18373_13_RDX_Radix
0.27145546650	1.0985859445	0.9199759314	1.3118724482	6081_52_PCOLCE2_P
0.25228332740	1.0912709989	0.9253370907	1.2869606168	19561_216_PLXND1_
0.27119097748	1.0984000954	0.9199079642	1.3115255183	5002_76_MMP14_MM
0.29355069664	1.1068225276	0.9134158263	1.3411811710	10583_1_TMEM108_T
0.27528823142	1.0998607344	0.9185847596	1.3169101950	12788_6_CCDC101_S
0.25723770637	1.0930465905	0.9237627196	1.2933525283	12879_5_RBL1_p107
0.26254879452	1.0950315369	0.9222098603	1.3002399112	13053_6_SLC6A14_S(
0.28924420610	1.1051153710	0.9145302525	1.3354178058	4989_7_FGG_Fibrinog
0.25891266189	1.0936148330	0.9231758698	1.2955206515	6375_75_XXYLT1_X
0.23280747452	1.0837778801	0.9306225337	1.2621384621	6947_4_ST3GAL6_SL
0.26221082773	1.0947882789	0.9221117639	1.2998005475	4706_17_EPB41_41
0.07878297812	0.9157670205	0.77509509	1.0819694855	9243_10_LYG1_LYG
0.26472993736	1.0956027418	0.9211608458	1.3030790153	8750_46_VCL_Vincul
0.24853018797	1.0894059	0.9256443623	1.2821395269	12659_13_OLA1_GTF
0.25188439185	1.0906539004	0.9246596624	1.2864473049	17728_61_CHMP2A_C
0.27974842230	1.1011429773	0.9166303453	1.3227969842	15481_45_CAMP_LL_
0.26158509916	1.0942257926	0.9217410514	1.2989874796	11436_6_INA_AINX
0.25351786020	1.0912069021	0.9240868728	1.2885503930	8768_4_BCL10_Bcl_1
0.25463887510	1.0915325093	0.9236024824	1.2899956871	12532_28_CDC34_UE
0.27348288774	1.0986609242	0.9182379708	1.3145348643	5704_74_GZMM_Gra
0.24217181128	1.0868594139	0.9271988013	1.2740130638	9739_4_MSH2_MSH2
0.27496835631	1.0991567219	0.9177026811	1.3164890156	7967_38_POTEM_PO
0.26394808670	1.0949077385	0.9207121047	1.3020606002	12861_13_TMOD3_T
0.24841827964	1.0890662150	0.9251707273	1.2819960529	9816_37_ISOC1_ISO
0.29514770148	1.1066414013	0.9116598101	1.3433247550	18173_11_AKR7A3_A
0.26362293817	1.0946912894	0.9206474135	1.3016373060	11402_17_KMT2C_K
0.24604726393	1.0880598671	0.9256538556	1.2789600207	12799_65_DNAJC27_
0.08584195200	0.9105553230	0.7609077177	1.0896341002	19553_14_STX1A_ST
0.25897076588	1.0927018578	0.9215815855	1.2955959286	10510_62_SCIMP_SC
0.25275433846	1.0903857903	0.9234014495	1.2875669323	14332_3_RALA_RAL
0.25481302657	1.0911682290	0.9228253868	1.2902203614	9518_95_PLA1A_PLA
0.27575148130	1.0987715236	0.9163416868	1.3175203948	11422_2_DLX3_DLX

0.26808735355	1.0958992076	0.9185702327	1.3074613464	8991_115_FCRL4_FC
0.09334706622	0.9043823894	0.7450133898	1.0978426932	12686_15_MPST_THT
0.28666769391	1.1028227546	0.9130892696	1.3319815143	12895_28_DGKB_DG
0.26153726238	1.0933580468	0.9203237324	1.2989253417	9234_8_TWSG1_TWS
0.24371380615	1.0865806399	0.9252953174	1.2759791008	11171_25_FLNA_filar
0.26005155391	1.0927554494	0.9206763917	1.2969969502	13436_54_MAZ_MAZ
0.25215218563	1.0897391557	0.9228620963	1.2867918536	15474_7_PIN1_PIN1
0.24681971795	1.0877377050	0.9243914599	1.2799483402	3685_53_MFRP_MFR
0.22528480453	1.0798474011	0.9308609831	1.2526794341	8014_359_MANEA_M
0.25679917443	1.0914352119	0.9214450841	1.2927854763	9201_13_TAGLN2_Tr
0.26994240107	1.0963593252	0.9176378836	1.3098890003	9889_42_AFAP1L1_A
0.24355790990	1.0864306998	0.9251841886	1.2757801960	12691_44_SHMT2_GI
0.25214907788	1.0894844396	0.9224335929	1.2867878546	13104_32_EFNB1_EF
0.25577551373	1.0907554961	0.9212402951	1.2914627797	12740_55_FEV_FEV
0.24960378328	1.0884333460	0.9230009151	1.2835167651	12796_44_NUDT3_NU
0.26047613249	1.0924665068	0.9197989621	1.2975477442	8921_139_PABPN1_P
0.26112117815	1.0926744258	0.9195557623	1.2983849917	9989_12_LRRC24_LR
0.26146559097	1.0924605686	0.9188793200	1.2988322492	12832_10_ASH2L_AS
0.25938089420	1.0917059384	0.9195252392	1.2961273982	13393_46_DERL1_DE
0.24633844735	1.0868465745	0.9233217234	1.2793324869	7106_37_OOSP2_PLA
0.08331846953	0.9168502560	0.7734140730	1.0868878941	5363_51_SEMA3E_Se
0.24647743157	1.0867945743	0.9231050667	1.2795103063	15299_102_MESDC2_
0.24568665926	1.0865171240	0.9233636855	1.2784989049	19122_47_MYL12B_M
0.26346186574	1.0929009771	0.9177863494	1.3014276650	11406_82_ACAD8_AC
0.24363344261	1.0856814285	0.9238387149	1.2758765627	13545_97_EIF1AD_EI
0.25702559758	1.0905891357	0.9198087471	1.2930782260	14070_56_ITSN1_ITS
0.08168369801	0.9188917914	0.7781332343	1.0851125322	16823_75_APOL3_AP
0.22401956025	1.0784791518	0.9296790608	1.2510954909	19141_22_DAP_DAP1
0.26784439513	1.0945355073	0.9165082254	1.3071437262	19317_114_ACPP_Pro
0.24383798414	1.08566153	0.9236159095	1.2761375592	18309_18_CS_CISY
0.25885227721	1.0911146503	0.9190151239	1.2954424243	12881_17_RDH16_RD
0.28045816008	1.0990297448	0.9124676198	1.3237361565	15637_38_SORCS3_S
0.29617610781	1.1048321169	0.9077472285	1.3447069493	10036_201_ZHX3_ZH
0.26811083049	1.0944248643	0.9160788328	1.3074920419	9947_22_TOPBP1_TO
0.26519218392	1.0933065025	0.9168797056	1.3036814983	11308_8_CREB3L4_C
0.24104770358	1.0844360012	0.9241068006	1.2725817405	12651_21_PDK2_PDK
0.23994004661	1.0839270919	0.9242628650	1.2711729369	8021_59_MRPL14_RM
0.24110705488	1.0842986102	0.9238178273	1.2726572721	12665_16_ILF2_ILF2

0.2569931722	1.090099858	0.919013413	1.293036298	4889_82_WNT7A_WN
0.2412844942	1.084333179	0.923712815	1.272883111	6238_55_PSG8_PSG8
0.2614481949	1.091636045	0.917508775	1.298809654	14616_16_ZNF382_ZN
0.2508552758	1.087671008	0.920555640	1.285124082	19620_16_DLG2_DLG
0.2468911888	1.086165059	0.921654557	1.280039822	19209_6_NFKBIA_IK
0.2572773349	1.089855429	0.918340329	1.293403783	3719_2_CDKN1B_p27
0.2635592909	1.092035293	0.916243704	1.301554463	10454_99_DNAJC16_
0.0802842369	0.922301376	0.785016369	1.083595021	19381_7_RGS21_RGS
0.2372736101	1.082488829	0.924272916	1.267787950	8396_42_FAAH2_FAA
0.2489736085	1.086635454	0.920534093	1.282708180	11338_49_BLK_BLK
0.2452651214	1.085286147	0.921661042	1.277960082	17757_86_CAB39_CA
0.2475176757	1.086101573	0.920969663	1.280842002	19293_6_VPS26A_VP
0.2635058420	1.091911042	0.916084179	1.301484898	6940_18_JPH1_JPH1
0.2488498692	1.086481777	0.920387619	1.282549468	17383_4_STARD5_ST
0.2703011493	1.094210762	0.913716919	1.310359005	10001_7_RAF1_c_Raf
0.2447425245	1.084952664	0.921576206	1.277292399	4309_59_TPI1_Triosep
0.2690546579	1.093757831	0.914099344	1.308726671	6383_90_TLL1_TLL1
0.2686851341	1.093533665	0.914062399	1.308243155	3081_70_ULBP1_ULB
0.2555922065	1.088790147	0.918091738	1.291226067	9765_4_NDE1_NDE1
0.2705449136	1.094122960	0.913347618	1.310678462	13612_7_CYTIP_CYT
0.3527853862	1.124512128	0.888618890	1.423025709	2860_19_KPNA2_Kar
0.2513406091	1.08716182	0.919247679	1.285747947	8087_250_RNF13_RN
0.2594244449	1.090044878	0.916689279	1.296183846	14684_17_CAPN2_CA
0.2446717592	1.084659727	0.921143805	1.277202014	12603_87_PABPC4_P
0.0952689962	0.910202794	0.753184770	1.099954699	13499_30_F8_Coagula
0.2454458870	1.084769621	0.920617518	1.278191114	12394_53_C16orf54_C
0.2592879316	1.089783017	0.916373989	1.296006912	15674_3_CD79A_CD7
0.2442677086	1.084357202	0.921002097	1.276686064	9178_30_NRG1_NEU
0.2391298320	1.082477575	0.922539669	1.270143431	6352_8_INSRR_INSR
0.2735975161	1.094841757	0.911760586	1.314685555	12864_9_NIPAL4_NIP
0.2650747394	1.091744882	0.914369715	1.303528397	17729_20_UBE2S_UB
0.2615875864	1.090476801	0.915433531	1.298990710	6647_55_UBC_PolyUb
0.2457982773	1.084749771	0.920259478	1.278641616	6600_70_RELL2_REL
0.2719198260	1.094020321	0.911921590	1.312481770	13934_3_MCF2L_MC
0.2675648072	1.092444930	0.913265786	1.306778315	8285_64_ADCYAP1_
0.1020822869	0.905204992	0.739877988	1.107474598	8397_147_QSOX2_QS
0.2569278941	1.088507522	0.916390339	1.292951894	17816_58_NCALD_NC
0.2464735508	1.084682496	0.919524194	1.279505340	13591_31_PRIM1_PR

0.24095035293	1.08269208277	0.92122669277	1.27245785999	6256_9_LY6G6C_LY6
0.26351554624	1.09070796188	0.91405771600	1.30149752811	17511_10_RPP30_RPI
0.25177678438	1.08650399511	0.91773519477	1.28630888100	9764_79_HNRNPF_HI
0.26255917465	1.09024223277	0.91415113300	1.30025340799	10956_82_GCSH_GCS
0.09840459991	0.90960548488	0.74984166122	1.10340913400	7157_22_PCDHA4_PC
0.26405245157	1.09065309159	0.91347516999	1.30219649677	5476_66_PRKCG_PKC
0.25240411860	1.08643460333	0.91704249988	1.28711607977	7225_51_MMGT1_MM
0.25749091746	1.08825068511	0.91544237988	1.29368006100	8102_239_C1orf186_C
0.23624219037	1.08065748	0.92209878133	1.26648100255	7853_19_SCO1_SCO1
0.25642152201	1.08779805344	0.91565970466	1.29229734499	7173_141_TMEM132C
0.25243283574	1.08626188800	0.91672462422	1.28715304255	10608_9_HTN1_HIS1
0.24418545998	1.08339634911	0.91944623266	1.27658106322	12786_61_GPCPD1_G
0.24760249525	1.08455083488	0.91826372544	1.28095064700	17783_9_MMAB_MM
0.32523907440	1.11252324885	0.89406409700	1.38436157188	4549_78_FUT5_FUT5
0.24238858595	1.08271220311	0.91993689711	1.27428926755	5242_37_MAP2K4_M
0.24324392816	1.08296892822	0.91958630922	1.27537968722	8326_63_SIGIRR_SIG
0.25079935200	1.08562908333	0.91715378777	1.28505221533	5598_3_GREM2_GRE
0.24543550034	1.08367752711	0.91877432622	1.27817783899	11226_16_UBE3A_UE
0.08254305117	0.92492367466	0.78770535911	1.08604542799	13374_4_DEFB113_D
0.25567888571	1.08723527488	0.91539205700	1.29133799422	17736_105_APTX_AP
0.24001016118	1.08164188577	0.92030526088	1.27126206777	18942_11_PSMB9_PS
0.08567586711	0.92213739244	0.78051761622	1.08945314344	7809_22_SPATA9_SP
0.26158607213	1.08934985155	0.91354378922	1.29898874344	8749_194_CD1D_CD1
0.24590985481	1.08340197311	0.91787163999	1.27878429211	10710_23_ZPBP2_ZPI
0.25141507931	1.08534824199	0.91611508111	1.28584370077	19297_4_G6PD_G6PD
0.26717974667	1.09071049622	0.91071878644	1.30627522388	11838_130_PIEZO1_F
0.24702467223	1.08339045611	0.91682945811	1.28021069800	5751_14_LETMD1_HC
0.23600276035	1.07952416566	0.92038607799	1.26617780522	8872_1_SEC61G_SC6
0.23758613543	1.08004778911	0.91982158533	1.26818422766	10827_67_LINGO3_L
0.24457617849	1.08243185666	0.91745135400	1.27707994455	17467_1_DHRS9_DHI
0.24216584662	1.08152964155	0.91813292633	1.27400546477	8980_19_CD200R1L_
0.08790667723	0.92281326000	0.77992037799	1.09188621944	10053_5_ILK_ILK1
0.25517548010	1.08584780888	0.91351696211	1.29068809100	15432_1_OTOR_Otora
0.24728527468	1.08306691433	0.91604318488	1.28054436755	19373_3_MYL12A_M
0.24520094101	1.08236131811	0.91675884788	1.27787806555	7187_3_PPM1L_PPM1
0.25326031751	1.08503657733	0.91390109822	1.28821857900	17362_5_VHL_VHL
0.08502907501	0.92597511888	0.78753701722	1.08874872166	9906_21_SPOCK3_TIC
0.23766293433	1.07947603799	0.91877741666	1.26828162655	12033_3_BUB1_BUB1

0.24931451069	1.08349517855	0.91490931633	1.28314553260	17158_17_UBE2G1_U
0.23124961168	1.07719755210	0.92078934555	1.26017375422	6576_1_ART4_ART4
0.26079936581	1.08744443577	0.91106722899	1.29796722277	8300_82_PEX14_PEX
0.26384375127	1.08840529360	0.90990364644	1.30192475633	10390_21_ZNRF3_ZN
0.23819742706	1.07939171855	0.91814301644	1.26895969500	13574_50_RND1_RNI
0.23929979031	1.07978702777	0.91780333800	1.27035932099	17357_33_SEPSECS_S
0.25178509759	1.08415567499	0.91376478360	1.28631957444	3825_18_MAPK8_MK
0.24200687008	1.08071535655	0.91689667333	1.27380294399	7795_14_KLRC3_NK(
0.23435781679	1.07800629077	0.91931063399	1.26409672633	11242_33_TGM1_TGI
0.24941902685	1.08315160422	0.91423361900	1.28327964900	15581_16_FANK1_FA
0.24839460819	1.08270905400	0.91442297522	1.28196570655	15418_25_MAPK10_M
0.25856511308	1.08614854899	0.91093009600	1.29507047311	14124_6_EFNA2_Eph
0.26082806168	1.08693002855	0.91017936733	1.29800446955	6384_19_WFDC3_WF
0.26406357067	1.08800748677	0.90903879099	1.30221097611	13738_8_INHBA_Inhi
0.23801007717	1.07895346611	0.91756949311	1.26872197788	12486_8_GLRX2_GLI
0.27523755528	1.09165993044	0.90498334788	1.31684346088	13463_1_PXDN_PXD
0.26529602674	1.08814871660	0.90815485277	1.30381688333	11202_70_TBX5_TBX
0.26257062307	1.08721114911	0.90906475877	1.30026829388	8098_37_TBXAS1_TE
0.26363823326	1.08753422844	0.90863453499	1.30165721488	18395_5_ADPRH_AD
0.23545478205	1.07779727822	0.91794667377	1.26548415733	11837_7_TNFRSF18_(
0.24475522248	1.08090586588	0.91470258166	1.27730861855	11138_16_RUNX3_RU
0.24369325589	1.08054303833	0.91505985566	1.27595287944	14670_1_SKIL_SKIL
0.25007198956	1.08265113022	0.91279275011	1.28411785644	13657_2_PNKP_PNKI
0.23044872573	1.07587281000	0.91926188559	1.25916490288	13503_19_ARL6IP5_F
0.23882283265	1.07869271833	0.91638095700	1.26975355777	12020_39_BPGM_PM
0.24002533399	1.07906883744	0.91591806166	1.27128135655	10552_88_KLRC4_NK
0.27107625398	1.08962462822	0.90537166900	1.31137506411	6938_21_PCDHGA12_
0.24201473678	1.07961881722	0.91502977511	1.27381296455	14074_2_SHC2_SHC2
0.25901858382	1.08541536333	0.90928826700	1.29565788288	17835_28_ANXA13_A
0.25433228521	1.08375293111	0.91076317444	1.28960024811	10506_53_PGRMC2_F
0.09036874998	0.92528579344	0.78217717577	1.09457783488	6367_66_FMOD_fibro
0.25130088600	1.08260170844	0.91158847977	1.28569687433	13457_33_ELF5_ELF5
0.36603036097	1.12256872944	0.87389834022	1.44199902244	5637_81_NTNG1_NTI
0.24322310247	1.07982084555	0.91426682844	1.27535312688	9064_12_RNF215_RN
0.10961021760	0.91058209111	0.74307918577	1.11584304977	14101_2_CNTFR_CN'
0.24363053377	1.07984418300	0.91393390666	1.27587285144	4460_8_PDPK1_PDPk
0.09034665995	0.92593320044	0.78328941399	1.09455365588	7128_9_VWA2_VWA
0.25399564693	1.08320328911	0.91014593188	1.28916619244	15548_35_PHGDH_SI

0.23857803957	1.0779043899	0.9152660539	1.2694427689	9855_10_RHOA_Rho_
0.2316558234(	1.0754377366	0.9174104808	1.2606857556	11653_69_SLC6A9_S(
0.2588875284	1.0846562308	0.9081358194	1.2954880910	13465_5_RCAN1_CCI
0.2531452445	1.0827267378	0.9101189156	1.2880703484	14012_17_DHX58_LG
0.2331934447	1.0759845698	0.9169326989	1.2626257040	4986_59_PTK2_FAK1
0.2470720755	1.0805152281	0.9119263082	1.2802713856	11130_158_CACNB4_
0.2535695468(	1.0826934341	0.9096768677	1.2886169955	11336_9_FANCF_FAI
0.0981646572(	0.9206682856	0.7683763647	1.1031444108	14076_74_CST4_Cyst
0.2499809186	1.0814824228	0.9109060721	1.2840009159	5023_23_ADSL_PUR8
0.2561410269	1.0835798413	0.9088269547	1.2919349127	6334_9_GGT2_GGT2
0.2392630809	1.0779272919	0.9146781402	1.2703126876	8935_22_ABCC6_MR
0.2409707484	1.0783191213	0.9137814689	1.2724838126	13587_10_RACGAP1_
0.2619951792	1.0853017632	0.9063959504	1.2995202777	11186_12_TMEM52_
0.2462230126	1.0799639424	0.9117698258	1.2791848161	13566_2_SSU72_SSU
0.0928915326	0.9253946986	0.7803900696	1.0973427029	13998_26_ADSSL1_P
0.2617758938	1.0852433964	0.9064972220	1.2992353432	18864_7_PRSS3_TRY
0.2439489277	1.0792388125	0.9126188560	1.2762791464	4547_59_FLRT1_FLR
0.2474893297(	1.0803937694	0.9113409637	1.2808056957	8045_3_KIR3DL3_KI
0.2352125482(	1.0761312519	0.9153326966	1.2651776513	15333_11_SDF2_SDF
0.2339198175(	1.0756879287	0.9157617592	1.2635431740	15534_26_MDH2_Ma
0.2342653709	1.0758392632	0.9157029686	1.2639798712	16845_15_MPZL1_MI
0.2476531710	1.0803766265	0.9111627445	1.2810155618	17744_31_RAB11A_F
0.2380027062	1.0770948252	0.9144176848	1.2687126263	6274_15_WSCD2_WS
0.2384860686	1.0771012874	0.9139867637	1.2693260224	7193_98_PCDHAC1_I
0.2493723468(	1.0806583230	0.9100720396	1.2832197469	19139_3_HSF2BP_HS
0.2350067003	1.0758162237	0.9149851918	1.2649172441	19136_22_ALDH6A1_
0.2349887554	1.0757787953	0.9149379453	1.2648945454	17693_2_SEPT6_Septi
0.2547291548	1.0823871437	0.9081085907	1.2901121529	5013_2_CLIC1_NCC2
0.2589909916(	1.0837456373	0.9065178620	1.2956221333	9545_156_GZMK_Gra
0.2372792489(	1.0763687955	0.9138462399	1.2677950987	11350_30_STUB1_CH
0.2645342363	1.0855122828	0.9044482541	1.3028240263	13577_25_U2AF2_U2
0.2303551961	1.0740263366	0.9161949032	1.2590471391	9335_28_PSG9_PSG9
0.0943448753(	0.9260922970	0.7804320298	1.0989386773	8074_32_TMEM70_T
0.2305981562(	1.0739933429	0.9159160555	1.2593530745	8931_124_G0S2_G0S
0.2340326100	1.0750405456	0.9145566611	1.2636857004	12698_72_KPNA4_IM
0.2629438703	1.0845998935	0.9043656178	1.3007537061	17408_2_PMM1_PMN
0.2320277964	1.0743300425	0.9151811141	1.2611547839	18226_148_COX5A_C
0.2541579914	1.0816645890	0.9074147015	1.2893754985	3813_3_FYN_FYN

0.2379582915(	1.076219118:	0.912971945(	1.268656278(	5467_15_HSP90AB1_
0.248178112:	1.079535708:	0.909267440'	1.281688196:	12587_65_ARL2_ARI
0.0943452606	0.926935671{	0.781853825:	1.098939100{	7139_14_SLITRK4_SI
0.2297204635	1.073335352:	0.915597373	1.258248234:	11546_7_CYGB_CYG
0.2487840524'	1.079637910	0.908888714:	1.282465058(	9579_59_PRRG4_TM(
0.2498771082(	1.079788099{	0.908148405(	1.283867630:	17837_5_CLIC2_CLIC
0.2380817660(	1.075895390:	0.912310128:	1.268812934:	18950_13_RAC2_RA(
0.2410345483:	1.076843520{	0.911224156	1.272564999(	9831_12_G3BP2_G3B
0.240894033:	1.076743843	0.911183496	1.272386197:	12771_19_ZNF180_ZI
0.235439796(	1.074801206:	0.912864011{	1.265465192:	12818_159_SLC14A2_
0.247488544:	1.078779918:	0.908621059	1.280804689:	13556_28_HTR2A_5H
0.2466346559'	1.078453886{	0.908847652(	1.279711492	8343_224_PAPOLG_I
0.2641629910(	1.084211303{	0.902616633:	1.302340448{	10557_6_TEX29_TEX
0.251658102'	1.080078842'	0.907020687:	1.286156228{	12632_14_NAT1_AR
0.2540718225(	1.080791801(	0.906028986'	1.289264399	12694_28_DIMT1_DI
0.281730672(	1.089952977(	0.896316617	1.325421698{	16773_29_SCUBE3_S
0.235556507:	1.074633321:	0.912472352:	1.265612896	18411_83_ARL15_AR
0.251381780{	1.079791813(	0.906789202{	1.285800884:	6620_82_LINGO1_LI(
0.0966620454(	0.927349018:	0.780740377(	1.101488057{	10089_7_ASMTL_AS
0.2371378810(	1.074946812{	0.911562140:	1.267615885{	2615_60_EFNA5_Eph
0.235844461:	1.074447619:	0.911894398:	1.265977386:	9846_32_ARHGDIB_I
0.237123237{	1.074829115:	0.911375880{	1.267597323:	19124_9_UBLCP1_UI
0.255706524	1.080866732:	0.904674539'	1.291373685:	11364_18_MFN1_MFI
0.256789460:	1.081153535{	0.904175011{	1.292772917'	12494_99_GABARAP
0.249084368:	1.078608013'	0.906883119:	1.282850261:	8942_2_MRPL21_RM
0.2294203269'	1.072122401	0.913803376:	1.257870644{	10637_50_KIAA1324_
0.240181706:	1.075593031	0.909884715:	1.271480164:	19120_33_DAO_OXD
0.234865125:	1.073671450(	0.911469587:	1.264738175'	11382_5_BLVRA_BII
0.230837089:	1.072418865(	0.913014376(	1.259654012	16857_2_RAB2A_RA
0.234929083:	1.073759921:	0.911561502	1.264819068:	4306_4_TKT_Transke
0.101040205:	0.925452663:	0.774153739	1.106321120{	9294_45_MFAP2_MF
0.2345856891(	1.073583792:	0.911575454(	1.264384811'	9392_43_MGAT4A_M
0.235925288(	1.073891129	0.910876419	1.266079715:	13475_10_UBE2D4_U
0.236984198:	1.074104438:	0.910273904(	1.267421090:	14144_3_HIST3H2A_
0.235777859:	1.073702502:	0.910690711	1.265893073	14205_6_HEXIM2_HI
0.2355043285	1.073572816	0.910719807(	1.265546859:	12449_16_PPIH_PPIH
0.245439888	1.076741780	0.907047313(	1.278183447(	10056_5_FOXM1_FO
0.2540485169(	1.079471127(	0.903837159'	1.289234352	15448_47_SQSTM1_S

0.24527692968	1.07660891544	0.90697126277	1.27797517344	16915_153_SEMA4A_
0.24428975895	1.07628226006	0.90731621044	1.27671421622	17377_1_AKR1C3_AI
0.23075369892	1.07184880906	0.91212004888	1.25954897292	10430_31_SLC3A2_4I
0.23739330207	1.07389639100	0.90954913388	1.26793970300	13492_44_HSPA9_HS
0.11882257512	0.91459937588	0.74277591444	1.12617008999	15515_2_SAA1_SAA
0.23824497958	1.07409895100	0.90911768244	1.26902003877	13993_20_EPB41L1_I
0.23589867099	1.07316253877	0.90966506711	1.26604601644	11493_169_DYNLL2_
0.22880588002	1.07084769166	0.91219204077	1.25709798744	18319_7_PDHX_ODP
0.23722041380	1.07353928666	0.90910148644	1.26772051000	18925_24_PSMA5_Pr
0.22757864439	1.07047262044	0.91267252788	1.25555617833	6929_10_ST8SIA3_SI
0.24696312178	1.07669522	0.90558839233	1.28013190299	8405_108_ADAM10_
0.26001697603	1.08082921211	0.90072083822	1.29695210355	3348_49_BMP1_BMP
0.25011155126	1.07760901744	0.90427467300	1.28416865933	18213_30_UBE2F_UE
0.25972979637	1.08067455233	0.90072171366	1.29657969877	9220_7_EPHB3_EPHI
0.23468623381	1.07253729888	0.90970770300	1.26451194533	13937_75_CAND1_C
0.43533587836	0.72465312677	0.33977887277	1.54548206577	9837_60_NQO1_NAD
0.25245212471	1.07810938300	0.90299862077	1.28717787066	13228_75_MDM2_MI
0.24766265778	1.07659369244	0.90478446755	1.28102771455	17698_15_WBP2_WB
0.23317223988	1.07166726722	0.90960850988	1.26259893044	17802_4_NANS_SIAS
0.25080345166	1.07726538677	0.90307299722	1.28505748355	14090_23_DEF6_DEF
0.25057108399	1.07705730400	0.90293394733	1.28475891244	16932_5_SULT1E1_S
0.57157316506	1.18459054144	0.79232881366	1.77105101666	17692_2_BTN3A3_B
0.09657146722	0.93216000133	0.78893363488	1.10138829144	19639_53_IAPP_IAPP
0.24393700438	1.07501379577	0.90549817700	1.27626392899	5004_69_MAPK11_M
0.09447114964	0.93363775299	0.79310102366	1.09907745388	7875_86_PLEK_PLEK
0.23609606387	1.07238957044	0.90817584244	1.26629594966	17205_21_RAB5A_R
0.24705103006	1.07586536466	0.90411349955	1.28024444200	2968_61_TNFSF15_T
0.26593986881	1.08182844399	0.89705810466	1.30465660577	12834_3_KCNF1_KC
0.24934428845	1.07626326799	0.90270986433	1.28318374233	10917_40_GNGT2_GI
0.24926820948	1.07615772755	0.90260149644	1.28308612277	10953_14_CLEC2A_C
0.23835095205	1.07290344366	0.90699893100	1.26915452700	11926_23_AAGAB_P
0.23967467546	1.07326130733	0.90640346355	1.27083564900	12636_113_SMYD2_S
0.24381808383	1.07446187477	0.90467621333	1.27611216399	13568_30_PSMD4_PS
0.25918892420	1.07948125788	0.89921986666	1.29587860455	13588_11_ANXA9_AI
0.23419510927	1.07146645	0.90833806333	1.26389106500	18218_48_CNRIP1_C
0.30389441106	1.09364079344	0.88261181499	1.35512596222	4337_49_CRP_CRP
0.28925294341	1.08891715755	0.88790954466	1.33542947388	8337_65_PTPRU_PTP
0.11176966127	0.92283981477	0.76157328211	1.11825525344	9337_43_TAC1_TKN

0.24830394217	1.07599171606	0.90319354299	1.28184948111	9565_6_PAWR_PAWI
0.25391353319	1.07760164255	0.90083083411	1.28906033855	13589_10_APBB3_AP
0.23418267462	1.07133148633	0.90812053144	1.26387534906	8345_27_GPX7_GPX7
0.24759922561	1.07541143299	0.90285565199	1.28094645877	9531_24_LST1_LST1
0.09579914133	0.93455643606	0.79360798144	1.10053798922	9599_6_PIANP_PIAN
0.22793540182	1.06898407122	0.90981141311	1.25600418722	4217_49_HSD17B10_
0.24332341836	1.07369768322	0.90383678811	1.27548107144	11433_11_TCTN2_TE
0.26131304577	1.07939107388	0.89716191806	1.29863413377	11911_13_LRRC4B_L
0.24150528449	1.07312212206	0.90451106333	1.27316418299	12444_39_NR5A2_NF
0.10520692966	0.92860096544	0.77618898044	1.11094047306	12764_3_ELMO1_ELI
0.23882542219	1.07218341118	0.90535229033	1.26975684588	8383_20_C11orf94_CI
0.23836112626	1.07180541411	0.90513419255	1.26916743977	10889_2_MRAP2_MR
0.22958720726	1.06899658522	0.90833108866	1.25808057622	5357_60_NLGN4X_N
0.24784786675	1.07463293688	0.90132482655	1.28126499433	7180_114_IFNA14_IF
0.22640477387	1.06784563277	0.90926527933	1.25408318266	9730_22_GHDC_GHD
0.22975224479	1.06882312199	0.90788647977	1.25828822388	14614_41_NCMAP_C
0.23724346033	1.07095819688	0.90471442033	1.26774972699	7935_26_LARGE_LA
0.23686712393	1.07072753911	0.90466515088	1.26727271633	6267_51_STMN4_STN
0.24165982826	1.07221051499	0.90283543055	1.27336095777	9910_9_MTAP_MTAI
0.24404522584	1.07290675099	0.90185446755	1.27640205555	5229_90_IMPDH1_IM
0.23044408846	1.06862275988	0.90691846306	1.25915906377	19278_19_RAB1B_RA
0.23768818981	1.07084572311	0.90412222211	1.26831365806	9728_4_CCNB1IP1_C
0.29440598104	1.08837960606	0.88247395844	1.34232875306	7897_75_DLG3_DLG3
0.24897512803	1.07415083806	0.89950176311	1.28271012911	12381_26_CBR1_CBF
0.24633631977	1.07329160811	0.90043623422	1.27932977	12627_97_ATG7_ATG
0.22969397714	1.06816784806	0.90682644422	1.25821490844	7065_1_G6B_G6B
0.22806777608	1.06761542077	0.90736307077	1.25617046088	9981_18_KIAA1467_I
0.23144348406	1.06861346933	0.90599679206	1.26041809077	11699_16_PTP4A2_TI
0.23743011446	1.07044059177	0.90367142644	1.26798637977	7903_18_VAMP3_VA
0.10349064561	0.93300508622	0.78491495944	1.10903541899	13082_9_UNC45A_UI
0.22668228494	1.06702535288	0.90761697866	1.25443125299	10939_16_PLEKHA4_
0.22432301082	1.06624339633	0.90842789811	1.25147519411	13621_31_AP2A2_AP
0.23210239776	1.06854828422	0.90528956011	1.26124887122	15325_14_GAP43_NE
0.23375617405	1.06894574111	0.90446612555	1.26333642044	11490_42_UBL4A_UI
0.24155252886	1.07131260077	0.90142063566	1.27322433411	4212_5_EPS15L1_EP1
0.26965374626	1.07993309606	0.89060385033	1.30951094906	8905_20_KCNMB3_K
0.22799395644	1.06715643522	0.90664998377	1.25607773422	3033_57_LGALS2_Ga
0.23368728392	1.06880986333	0.90429849506	1.26324939206	3222_11_SEMA3A_Se

0.23713045108	1.0698603407	0.90296253444	1.26760646760	7167_102_VAPA_VA
0.23606283829	1.06948688	0.90329610160	1.26625387668	6226_69_EFHA1_EFH
0.22684971760	1.06648027150	0.90653812090	1.25464130322	2823_7_COMMD7_CO
0.23020435513	1.06745851610	0.90516037050	1.25885723755	5202_4_PPID_PPID
0.22592960899	1.06611069890	0.90674385477	1.25348742799	13472_35_HDHD2_HI
0.23389755774	1.06844295544	0.90348773499	1.26351504822	12787_47_ZNF134_ZI
0.22612774913	1.06609954844	0.90654524688	1.25373581877	9577_26_SYT2_SYT2
0.22461442473	1.06555570833	0.90699212188	1.25183994460	5650_9_PDIA6_PDIA6
0.22413444777	1.06537157677	0.90711397560	1.25123923444	17370_186_ABHD10_
0.25516597568	1.07468863777	0.89484566660	1.29067582388	18884_22_DNAJB4_D
0.23971491389	1.07002137422	0.90090301799	1.27088678655	8975_26_RQCD1_RCI
0.11376961222	0.92887445455	0.77002446522	1.12049394700	9931_20_KRT1_Kerat
0.24481705680	1.07149508360	0.89878883444	1.27738760255	8838_10_CASC4_CAS
0.22826133823	1.06647435010	0.90524928333	1.25641363144	3902_21_SKP1_SKP1
0.27931227069	1.08177206660	0.88504988100	1.32222017000	10760_107_GRIA4_GI
0.23707019542	1.06899608288	0.90155857811	1.26753008944	10907_116_NTM_NTI
0.22165151134	1.06436760855	0.90765597400	1.24813634077	13683_18_CLCNKB_C
0.23096353214	1.06714223699	0.90393755577	1.25981329599	19266_35_G3BP1_G3
0.10821134775	0.93277982522	0.78084115900	1.11428322177	6388_21_CCDC126_C
0.14852730533	0.90890664922	0.71208849988	1.16012447499	6392_7_WISP2_WISP
0.24241648018	1.07048031222	0.89924333800	1.27432481333	11387_3_ATF6B_ATF
0.24273011588	1.07058057288	0.89912974777	1.27472454977	14129_1_IFNA7_IFNA
0.23633129692	1.06866088244	0.90165925977	1.26659385933	8255_34_MRVI1_MRV
0.25495610960	1.07423366144	0.89427580822	1.29040498322	6296_36_OIT3_OIT3
0.23907025349	1.06923630133	0.90016163233	1.27006776011	3466_8_PRKACA_PR
0.24017666348	1.06957030460	0.89972807811	1.27147375344	5484_63_RPS3A_RS3
0.23555806363	1.06815148422	0.90149667533	1.26561486533	19252_67_PSMB2_PS
0.22941095343	1.06622586211	0.90378788155	1.25785885422	15437_11_RAMP3_RA
0.22538202980	1.06485689855	0.90510783788	1.25280123211	13519_112_ARHGEF2
0.26125471840	1.07553579522	0.89081650522	1.29855839011	17706_4_PPP1R1A_PI
0.10238573748	0.93769387744	0.79370040122	1.10781071344	7975_97_UNC5A_UN
0.22726729575	1.06519724611	0.90398065660	1.25516532344	4203_50_CFL1_Cofili
0.26642043410	1.07673224222	0.88819947377	1.30528372922	12398_15_PAX4_PAX
0.22182983360	1.06350813	0.90602912400	1.24835893100	8899_75_UGT1A8_UI
0.23740998417	1.06805095644	0.89965935544	1.26796085511	15422_12_PMP2_MYI
0.23577470763	1.06750309900	0.90020751577	1.26588908288	13692_154_WISP1_W
0.23406544647	1.06674139422	0.90046111677	1.26372719599	13236_25_WNT3A_W
0.24080592012	1.06874598877	0.89777666511	1.27227408855	8236_8_C18orf32_CR

0.10374911537	0.93819498887	0.79346641555	1.10932210817	7175_4_PCDHGA2_PC
0.24414854085	1.06960445599	0.89621878577	1.27653393388	13453_2_MRPL33_RM
0.22541251229	1.06404081811	0.90369351688	1.25283942122	19183_164_RPL12_RI
0.23533781588	1.06697348760	0.89970750122	1.26533614713	3854_24_NACA_NAC
0.22997836316	1.06534068044	0.90177603177	1.25857277800	3403_1_TPSB2_TPSB
0.22743436209	1.06452042544	0.90268143222	1.25537503688	12477_42_TSN_TSN
0.21834265531	1.06178623411	0.90625240188	1.24401326233	16872_248_GSTZ1_M
0.23188367060	1.06572964199	0.90071685977	1.26097303200	6597_24_FAM174A_T
0.22184996911	1.06273046644	0.90468636500	1.24838406760	17509_6_HPD_HPPD
0.22765693837	1.06443710499	0.90233926944	1.25565448460	5007_1_MAPK14_MA
0.24869067638	1.07051208777	0.89367202360	1.28234531200	12548_75_RRAGC_RI
0.22556875652	1.06361701655	0.90283271444	1.25303518555	19438_68_SYF2_SYF2
0.11167603314	0.93503370800	0.78190546777	1.11815055822	10613_33_CASC4_CA
0.26794869800	1.07592193099	0.88550879500	1.30728007222	17456_53_GOLM1_GO
0.23005083306	1.06486294411	0.90090214600	1.25866399000	19169_88_FIBP_FIBP
0.22701248869	1.06384316188	0.90191361222	1.25484553922	11683_19_GGA3_GGA
0.24858405572	1.07009181899	0.89306568800	1.28220859488	14283_12_RAB14_RA
0.22439269487	1.06302367233	0.90288692233	1.25156240500	14083_25_SEPHS1_SE
0.24323916010	1.06840222488	0.89501876833	1.27537360611	10054_3_GAN_GAN
0.22736333431	1.06381514055	0.90154974022	1.25528587355	19224_5_EIF1AY_IF1
0.22322355489	1.06261499200	0.90324823111	1.25010000844	9339_204_FKBP2_FK
0.23233273006	1.06517586188	0.89937706799	1.26153941100	9929_16_HSD17B7_D
0.23919887825	1.06707922577	0.89641801760	1.27023113288	11365_17_TENM4_TE
0.23607443045	1.06610653800	0.89758459655	1.26626855560	11531_24_C10orf54_C
0.23339777614	1.06533242500	0.89868382488	1.26288372444	15435_4_PNP_PNP
0.27060954536	1.07614909022	0.88352868388	1.31076317688	18342_2_PSAT1_SER
0.24035069051	1.06721900522	0.89562069999	1.27169504355	9283_8_CD44_CD44
0.23129298784	1.06457669888	0.89930010511	1.26022841699	13387_55_EHF_EHF
0.10769457971	0.93851642588	0.79088364433	1.11370754455	17400_71_ACOT8_AC
0.23154890701	1.06438739860	0.89875027422	1.26055097488	11217_16_CTBP1_CT
0.10327610911	0.94139415944	0.79926492499	1.10879751599	12551_3_ITCH_ITCH
0.23711188629	1.06596253633	0.89641166460	1.26758293499	13979_3_SLC26A7_S
0.28723025548	1.08055168744	0.87608970488	1.33273104677	15431_31_OTC_OTCa
0.22144879860	1.06149559100	0.90294728811	1.24788335322	17765_3_SNUPN_SPN
0.22384879660	1.06219828799	0.90197582388	1.25088186755	19207_119_ADK_ADK
0.26552618529	1.07428045122	0.88495011233	1.30411700244	7551_33_LRRC32_LR
0.22461953359	1.06246113677	0.90172701788	1.25184634000	9369_174_LRRC4C_N
0.22982592660	1.06380088788	0.89930822422	1.25838094022	5463_22_GAS1_GAS1

0.2451979312	1.0681587216	0.8928602183	1.2778742193	5097_14_KIR3DS1_K
0.2372208255	1.0657648497	0.8959815970	1.2677210320	5014_49_NCK1_NCK
0.2403584929	1.0666427042	0.8946467058	1.2717049658	9345_436_WFDC13_V
0.2273641194	1.0629052153	0.9000074274	1.2552868591	12650_43_GNAI3_GN
0.2031172351	1.0559240967	0.9100237090	1.2252160982	15388_24_FCGR3A_F
0.2322143220	1.0642016381	0.8978389613	1.2613900435	3897_61_PDXP_PLPP
0.2430851265	1.0671781538	0.8931066506	1.2751771708	6107_3_CELA1_ELA1
0.1083655303	0.9396503509	0.7922641576	1.1144550381	18881_7_CD97_CD97
0.2204094108	1.0606335919	0.9024188630	1.2465869924	12597_68_ATG5_Auto
0.2505818480	1.0692228511	0.8898363643	1.2847727417	19272_9_PEF1_PEF1
0.3150312248	1.0876704638	0.8633330123	1.3703020977	9928_125_LECT1_LE
0.1900368939	1.0519688808	0.9151110746	1.2092942124	16760_2_IL26_IL_26
0.2256924579	1.0620019001	0.8999815338	1.2531901973	19329_31_HIPK3_HIP
0.2509616139	1.06909844	0.8892915069	1.2852607473	13516_46_EMC4_TM
0.1765968505	1.0480977287	0.9206796034	1.1931499783	10584_7_NDUFS4_NI
0.2301175556	1.0630051880	0.8977015678	1.2587479741	11465_4_GPR135_GP
0.2264367019	1.0619339643	0.8991969235	1.2541232237	9005_16_PLXNA1_PL
0.1117492847	0.9388653514	0.7882691423	1.1182324675	5861_78_HAAO_3HA
0.2269433003	1.0619942636	0.8988435757	1.2547587215	18280_29_DTD2_DTI
0.2446953111	1.0669655779	0.8913145452	1.2772320955	14624_51_CTCF_CTC
0.2347766304	1.0638359866	0.8949260693	1.2646262582	9326_33_ITIH2_ITI_h
0.2402148071	1.0652614600	0.8924593931	1.2715222530	14139_16_NRG4_NR
0.2217819196	1.0600870308	0.9002525885	1.2482991186	17777_31_SDSL_SDS
0.2285460405	1.0619548799	0.8973375583	1.2567713862	18829_4_EIF4A1_IF4
0.2224840251	1.0601636369	0.8997507631	1.2491758641	12460_18_PSMA7_PS
0.2233695073	1.06032379	0.8992260243	1.2502824769	11709_29_CPT1B_CP
0.2212070189	1.0596853744	0.9000878367	1.2475816769	14091_42_CBR3_Carb
0.2283396125	1.0615310000	0.8968064634	1.2565119802	17676_13_TRIP10_CI
0.2252081684	1.0606795234	0.8981765349	1.2525834374	19274_80_SNTA1_Al
0.2387120442	1.0644016025	0.8923592214	1.2696128915	7240_2_MBTPS1_MB
0.2302259170	1.0619797473	0.8958733625	1.2588843812	3874_8_UBE2L3_UB
0.2205732395	1.0592735111	0.8999585020	1.2467912357	12851_5_POLM_DPO
0.2223410395	1.0596930887	0.8990807873	1.2489972627	5005_4_MAPK12_MK
0.2403900196	1.0646563272	0.8912895606	1.2717450592	14294_61_MBD1_MB
0.2184995201	1.0585768144	0.9006408046	1.2442084195	17405_2_UCHL5_UC
0.2281127934	1.0612057261	0.8964602598	1.2562270116	13482_14_CNOT1_CN
0.2189236356	1.0585930098	0.9002864565	1.2447362195	3821_28_MAPKAPK5
0.2221001725	1.0591888068	0.8984416689	1.2486964560	17843_30_PPCS_PPC

0.22718176964	1.06053098188	0.89615458611	1.25505797806	7785_1_EFNB3_Ephri
0.23738931966	1.06331128559	0.89171069477	1.26793465355	15446_25_NRG1_SMI
0.22124280912	1.05885768288	0.89865015399	1.24762632888	19334_62_TXNDC12_
0.22183990870	1.05897882200	0.89831924066	1.24837150844	13963_7_TOLLIP_toll
0.22045418456	1.05844500211	0.89865823266	1.24664280799	10370_21_STAT1_ST
0.22882179153	1.06068214522	0.89494114499	1.25711798999	6415_90_CPN2_CPN2
0.21403467280	1.05660411332	0.90130237766	1.23866560200	13487_24_UNC93B1_
0.27499531948	1.07315749722	0.87477825344	1.31652451288	9312_8_AZGP1_AZG
0.24546716108	1.06501636300	0.88737569077	1.27821830766	11934_9_INTS3_INT3
0.22203823336	1.05859956500	0.89749790377	1.24861911588	19161_1_USP15_UBP
0.22964973638	1.06054276255	0.89396549388	1.25815924533	12853_112_TMOD2_T
0.22032386732	1.05800584744	0.89802969199	1.24648035944	18299_13_CAPNS1_C
0.23396680354	1.06161909860	0.89192215999	1.26360254433	12742_160_TMEM8B_
0.21333841502	1.05585660288	0.90065441833	1.23780347166	8391_12_DEFB115_D
0.24339531276	1.06387194555	0.88730610988	1.27557277466	10580_14_SFTA2_SFT
0.23803646577	1.06242484622	0.88964862899	1.26875545811	10959_125_BET1L_B
0.24499911494	1.06428436611	0.88657116388	1.27762018244	4981_6_DSC3_DSC3
0.24028001130	1.06302806911	0.88866316955	1.27160516422	7096_30_RMDN1_RM
0.22697209507	1.05948378333	0.89457323244	1.25479485244	9000_177_SLC3A1_SL
0.22877142843	1.05980267277	0.89350266444	1.25705467922	8307_47_C11orf68_CI
0.22049043343	1.05754680422	0.89710115511	1.24668799811	11239_49_TMPRSS6_
0.23877136096	1.06233662500	0.88884743688	1.26968820311	7105_7_CABLES2_CA
0.22701156322	1.05900187960	0.89372435411	1.25484437799	18813_15_DDX19A_I
0.25649908960	1.06676896099	0.88053090277	1.29239758922	15324_58_FTL_Ferriti
0.21867928885	1.05654832922	0.89703115466	1.24443210944	12408_333_RAB22A_
0.22524728973	1.05812238077	0.89381604366	1.25263244100	16890_37_ADAMTSL
0.22132808754	1.05705732477	0.89552046033	1.24773272899	11369_23_ADH5_ADI
0.22453106024	1.05783112522	0.89396410766	1.25173558999	3414_40_BMX_BMX
0.23354216991	1.06024130344	0.88998638322	1.26306609011	8660_5_OLFML3_OL
0.23126914262	1.05949322	0.89075333088	1.26019836699	10756_34_UCN3_UCI
0.21186032568	1.05434325999	0.89940289644	1.23597523900	14039_33_KLK5_kalli
0.21955533959	1.05636372911	0.89593249733	1.24552277277	9478_69_PRPSAP1_K
0.25757780968	1.06637776960	0.87893658999	1.29379247666	5440_26_TNNI2_Trop
0.21812248530	1.05583634511	0.89632152000	1.24373939800	17490_4_SH3BGRL3_
0.10731567901	0.94809159655	0.80740974577	1.11328563999	17827_53_DYNLRB2_
0.23025822289	1.05891460844	0.89068062222	1.25892505122	14066_49_MAGI2_MA
0.22030851946	1.05627845299	0.89511341744	1.24646122888	9078_207_SNTB1_SN
0.22196466612	1.05668317560	0.89431714299	1.24852726177	19222_124_RAB5B_R

0.21940160820	1.05594341388	0.89535731000	1.24533131155	5885_55_EIF4H_eIF_4
0.22038387046	1.05613284533	0.89479922566	1.24655515444	11161_5_SPG20_SPG2
0.22481765068	1.05717556733	0.89260059055	1.25209437688	16863_47_NANP_NA
0.24795472800	1.06295007400	0.88173963444	1.28140191922	19622_7_INHBA_Acti
0.22923318190	1.05798232	0.89002480944	1.25763526266	18928_10_S100Z_S10
0.22572085173	1.05694937555	0.89141318322	1.25322578066	16856_79_MAPRE2_M
0.22007609719	1.05544315522	0.89390601766	1.24617155711	17320_19_AASDHPPT
0.21902199892	1.05506758144	0.89421203833	1.24485866199	11454_87_EIF3G_EIF
0.21896331900	1.05508965600	0.89430193299	1.24478561588	12508_9_VPS24_CHM
0.22180704890	1.05579624799	0.89295721600	1.24833048788	3860_7_PSMA6_PSA6
0.24699546519	1.06236817677	0.88161980600	1.28017330744	8922_4_TMCC3_TMC
0.21134014650	1.05297648422	0.89753932355	1.23533247766	12595_11_TMOD1_TM
0.22338490224	1.05607469566	0.89201969444	1.25030172500	7776_20_UNC5B_UN
0.29552427390	1.07474948900	0.85954760233	1.34383070944	16322_10_PACAP_PA
0.11231027259	0.94798570933	0.80320767433	1.11885995833	6359_50_POMGNT2_
0.21500287643	1.05382032455	0.89569175799	1.23986546333	7234_12_COLGALT2_
0.23326918762	1.05846617444	0.88725089588	1.26272134255	18261_34_NATD1_NA
0.21385108873	1.05341945366	0.89604190511	1.23843822366	17699_43_TIMELESS
0.21381701498	1.05332411722	0.89591025211	1.23839602611	12855_16_CASS4_CT
0.22182216998	1.05534038288	0.89217278088	1.24834936411	8032_23_MCCD1_MC
0.20946708747	1.05209651899	0.89771972355	1.23302079266	12821_6_NLRP10_NA
0.22771646585	1.05678298755	0.88935596433	1.25572923288	13434_172_PARVA_P
0.22523552693	1.05605934922	0.89034455055	1.25261770666	3347_9_ADRBK1_BA
0.22157094690	1.05512137999	0.89202660377	1.24803578933	5019_16_UCHL1_PGP
0.21141081017	1.05250039866	0.89666452855	1.23541977388	16923_20_PFN2_Profi
0.22635143000	1.05613766288	0.88948347377	1.25401628677	19265_9_TCP1_TCPA
0.22846710192	1.05663934033	0.88844705199	1.25667218233	13669_6_FGFR3_FGF
0.22031746786	1.05442924933	0.89197406799	1.24647238277	14107_1_MTHFS_MT
0.22330189382	1.05513796822	0.89051188866	1.25019794388	6533_20_DNASE2B_D
0.20773900208	1.05115730611	0.89766754611	1.23089186744	4807_13_COL8A1_CO
0.23413276833	1.05774785255	0.88528220644	1.26381227533	11608_5_MAP1LC3B_
0.21517522023	1.05293638088	0.89403568211	1.24007916488	15527_90_DLD_DLDH
0.21810855381	1.05368657622	0.89268770477	1.24372207100	3391_10_PIK3CG_PK
0.22368421555	1.05468207544	0.89057346666	1.24903146322	2855_49_MAPK3_ERK
0.24080373831	1.05935215022	0.88206577255	1.27227131266	8086_49_ITM2B_ITM
0.22408919052	1.05513887400	0.88981259500	1.25118260800	9115_78_ADAMDEC1
0.21444192817	1.05249047077	0.89393388299	1.23917015800	13517_3_PTCHD3_PT
0.23078396511	1.05660058322	0.88632511977	1.25958709533	17199_43_HIF1AN_H

0.11304598052	0.94981207877	0.80571255460	1.11968341533	13629_25_ATG4B_AT
0.20922390404	1.05114962795	0.89632249255	1.23272097888	9745_20_EIF4E2_IF4I
0.21808350611	1.05325583244	0.89198033977	1.24369091900	12433_8_ARL11_ARI
0.21195871353	1.05171584332	0.89483782355	1.23609684999	16809_1_NME4_NDK
0.21211309879	1.05163377099	0.89456005133	1.23628769988	15617_8_XCL2_lymph
0.14200162185	0.93800314222	0.76337523331	1.15257851788	18832_65_SAA2_SAA
0.21625260849	1.05247320992	0.89228745011	1.24141593160	12022_12_SMAD4_SM
0.21471513210	1.05203047244	0.89290867411	1.23950875044	13044_5_TSG101_TS1
0.22073246453	1.05345768155	0.88996165955	1.24698977119	14146_92_HIST1H3A_
0.11023726538	0.95193805722	0.81159982255	1.11654295600	8948_13_ADAM19_A
0.22558584639	1.05470930022	0.88775854822	1.25305659988	8970_9_RIPK2_RIPK2
0.10653868489	0.95363895060	0.81752077800	1.11242095944	9017_58_LCT_LPH
0.11373259643	0.95066829311	0.80661181655	1.12045247188	9511_61_NXPH2_NX1
0.21752451784	1.05239352233	0.89101832244	1.24299590477	11827_7_RORC_ROR
0.22231081095	1.05351307644	0.88865154977	1.24895950788	3891_56_MBD4_MBI
0.23971417499	1.05782187260	0.88047806699	1.27088584744	3903_49_SNX4_Sortin
0.21947851970	1.05269215122	0.88978372900	1.24542709555	18338_26_IDH1_IDH
0.21699589160	1.05197570400	0.89078172988	1.24233899811	9839_148_TIRAP_Tira
0.20945913444	1.05000817577	0.89416654122	1.23301098644	14157_21_YWHAE_14
0.22523616836	1.05365588955	0.88629596699	1.25261851011	15312_14_AGO1_AGO
0.21845863504	1.05186724355	0.88929629300	1.24415755100	12612_37_PSMB1_PS
0.21474772560	1.05097268022	0.89108493488	1.23954915100	16858_384_RCN3_RC
0.21589113500	1.05116117277	0.89038593877	1.24096727388	17849_6_DRG1_DRG
0.11207794360	0.95323180688	0.81231078222	1.11860004500	10535_25_COX6A2_C
0.21984134399	1.05196359188	0.88823020155	1.24587904877	15436_40_RAMP1_RA
0.21858412484	1.05166849955	0.88884872199	1.24431368999	3866_7_SBDS_SBDS
0.21849774192	1.05163676077	0.88887185277	1.24420620711	4389_2_DHH_DHH
0.12777065152	0.94670378477	0.78874775799	1.13629236588	9102_28_ZFYVE27_Z
0.22269753405	1.05259113860	0.88675390344	1.24944260277	17427_26_NLGN3_NI
0.21916000651	1.05171050822	0.88840796788	1.24503047377	19379_154_RAB3D_R
0.13253649689	0.94506932955	0.78228944222	1.14172068455	6605_17_IGFALS_IGI
0.20674244724	1.04854477860	0.89410157499	1.22966582711	17161_1_DDOST_OST
0.21121774237	1.04962981699	0.89195227700	1.23518127700	7980_72_B3GNT2_B3
0.20764840158	1.04875087999	0.89364313099	1.23078035300	9713_67_PDGFRL_PC
0.21597461064	1.05079872322	0.88969774799	1.24107086800	9969_8_SLC22A16_S
0.22135591090	1.05199799500	0.88694394577	1.24776744560	12538_19_RGS7_RGS
0.21162396739	1.04948857477	0.89135008188	1.23568314055	16617_14_CASP14_C
0.11820353450	0.95179653833	0.80492070533	1.12547316077	9359_9_DLK2_EGFL9

0.22401740692	1.05219824660	0.88492328700	1.25109279659	3864_5_RPS7_RS7
0.21007180230	1.04873398600	0.89145137680	1.23376664400	8039_41_FAM177A1_
0.20902841450	1.04822703520	0.89151945760	1.23248001830	15506_34_LRP12_LRI
0.24428566701	1.05659111760	0.87442384810	1.27670899200	6378_2_C5orf38_CEI
0.20658604820	1.04759495890	0.89262206690	1.22947352370	10876_300_BRAT1_B
0.21876413959	1.05043490770	0.88660511530	1.24453770480	12784_10_APBB3_AP
0.22769124134	1.05251318610	0.88220606930	1.25569755800	13688_2_CAPSL_CAI
0.11796795111	0.95303259880	0.80720284130	1.12520804910	5475_10_PRKCB_PK(
0.12507130949	0.95027503260	0.79685785520	1.13322926010	9470_15_METTL24_M
0.21279475369	1.04888045350	0.88927564250	1.23713070860	18244_1_ANXA7_AN
0.23418065347	1.05384944320	0.87872660430	1.26387279460	9089_77_JPH3_JPH3
0.21379277735	1.04900496670	0.88859950190	1.23836601070	12556_7_UBE2C_UBI
0.20657829948	1.04724604700	0.89203448470	1.22946399680	11677_17_ZDHHC14_
0.22108550000	1.05064344760	0.88490062130	1.24743008120	12726_3_RFXAP_RFX
0.22906348128	1.05235169380	0.88072597060	1.25742185920	13636_20_NAP1L1_N
0.20949388438	1.04774743430	0.89028934150	1.23305383420	5093_47_JAG2_JAG2
0.17068270177	0.93390224100	0.73531983250	1.18611433730	6485_59_IGLL1_IGLI
0.21039094384	1.04783943690	0.88964727630	1.23416045300	14106_46_RAP1GDS1
0.21281751739	1.04839386500	0.88843051790	1.23715887060	18186_15_TARS_SYT
0.21575220500	1.04911561040	0.88704715290	1.24079487810	5526_53_TNFRSF18_(
0.22044496396	1.04986876320	0.88416230870	1.24663131320	10372_18_STAT6_ST
0.22561060046	1.05104846570	0.88158470400	1.25308761859	9843_5_ACTN1_ACT
0.20905103234	1.04715996380	0.88968516510	1.23250789460	10762_2_KLRG2_KLI
0.12025499183	0.95365988230	0.80641936400	1.12778439070	12641_3_INPP5B_I5P
0.11571238468	0.95550125370	0.81322228710	1.12267292770	10336_3_STUB1_CHI
0.15544569220	0.94095692490	0.75793206240	1.16817849310	19601_15_ASB9_ASB
0.21148766575	1.04750131270	0.88809868210	1.23551472630	8918_64_PTK2B_FAK
0.21850546676	1.04895957950	0.88434512990	1.24421581840	2441_2_FGF10_FGF_
0.21045209879	1.04699452850	0.88815883230	1.23423593040	5092_51_JAG1_JAG1
0.24472293547	1.05467193410	0.87086925360	1.27726737870	13399_33_RELL1_RE
0.21296194248	1.04733141350	0.88650270130	1.23733756030	11649_3_SMAP1_SM
0.22369423550	1.04978081790	0.88114644540	1.25068854480	14009_65_TNFAIP3_
0.23842264215	1.05311452300	0.87378697390	1.26924551610	18184_28_GMPS_GU
0.21105132086	1.04693810320	0.88753111680	1.23497573340	9265_10_GLIPR1_GL
0.20917854014	1.04637365810	0.88823628470	1.23266505900	18833_76_PEA15_PE
0.21272360760	1.04716880180	0.88643868480	1.23704269470	2870_29_RAC1_RAC
0.12553843982	0.95328757800	0.80154372060	1.13375874960	17829_2_TREX2_TRE
0.21042657660	1.04652413470	0.88738359510	1.23420443040	12497_29_NUDT16L1

0.21009828502	1.046429190	0.887513905	1.233799317	13076_4_FXR1_FXR1
0.21018438879	1.046380056	0.887354154	1.233905557	4976_57_CRK_CRK
0.21176068536	1.046614186	0.886353037	1.235852092	13066_42_EDC4_EDC
0.13261109272	0.951133105	0.792301230	1.141805855	8006_12_DNAJB12_D
0.24019821394	1.052917362	0.871910314	1.271501154	15633_6_RBP4_RBP
0.22042941758	1.048359164	0.881635180	1.246611932	4249_64_NME2_NDP
0.21135452251	1.046231949	0.886065553	1.235350236	3522_57_VIP_Vasoact
0.20597042762	1.045016408	0.888780256	1.228716867	5227_60_PDK1_PDK1
0.22415515284	1.049025001	0.879472637	1.251265141	9876_20_ALDOC_ald
0.20742766985	1.045233062	0.887854060	1.230508710	8068_43_KIAA1161_I
0.22452431926	1.048968698	0.879053656	1.251727152	5035_7_TYMS_TS
0.25238976757	1.055137183	0.864980611	1.287097608	3054_3_HP_Haptoglol
0.22788195139	1.049508801	0.877009496	1.255937055	5765_53_DEFB121_D
0.20801971265	1.044994470	0.886923519	1.231237440	17151_84_IRF3_IRF_
0.12734598815	0.954458392	0.802062741	1.135809926	3898_5_PSMD7_PSD
0.22381243049	1.048101883	0.878226421	1.250836378	10603_1_HTN3_HIS3
0.23050189729	1.049570574	0.874817759	1.259231856	4474_19_RPS27A_Ub
0.20367689057	1.043658681	0.888507771	1.225901988	7827_20_ADAM32_A
0.22850947642	1.048673536	0.875064796	1.256725434	18408_26_ARF4_ARF
0.21905711894	1.046475048	0.879675420	1.244902382	9449_150_C4BPA_C4
0.12787832146	0.955713641	0.803745807	1.136414717	9864_38_TTC1_TTC1
0.20853600115	1.044144966	0.885025051	1.231873278	18232_42_PIP4K2B_F
0.20747404118	1.043877776	0.885512044	1.230565772	14623_26_SUMO3_SU
0.22404757456	1.047392930	0.876832524	1.251130540	6405_74_PSG2_PSG2
0.20807004559	1.043821614	0.884889207	1.231299413	18300_39_PURB_PUF
0.24427576692	1.051528133	0.866072353	1.276696352	10612_18_PLOD3_PL
0.21138526575	1.044415515	0.882964362	1.235388216	18174_79_PDCD6IP_I
0.22799173996	1.047937664	0.874289666	1.256074950	18913_3_CDC2_p34_
0.20669159693	1.043298792	0.885222388	1.229603299	12537_88_PURA_PUF
0.22208642176	1.046377584	0.876851294	1.248679286	14318_1_VPS29_VPS
0.27735995108	1.058171803	0.848509040	1.319641291	12554_10_RAD51L3_
0.14070366200	0.953010294	0.789020632	1.151083487	11547_84_MUSK_MU
0.20734295880	1.042974002	0.884095262	1.230404477	11664_32_ARFGAP2_
0.20740113281	1.043072499	0.884210816	1.230476057	12708_91_L3MBTL2_
0.20582675426	1.042732442	0.885026649	1.228540346	13491_40_PDE6D_PD
0.20607956851	1.042711491	0.884767376	1.228850977	15363_32_APOA5_Ap
0.21460640752	1.044511180	0.880286025	1.239373992	6285_71_MLEC_K015
0.21562956768	1.044788263	0.879852433	1.240642719	7944_1_KIR3DL3_KI

0.20463021151	1.04234810011	0.88543316833	1.22707122422	12016_60_CBL_CBL
0.20170334236	1.04174206533	0.88699618911	1.22348499806	19112_2_TCEAL3_TC
0.21773113316	1.04497036122	0.87831139166	1.24325275322	4230_1_EIF4G2_IF4G
0.20641104632	1.04246103144	0.88404929155	1.22925838233	12599_10_FERMT3_U
0.22796626295	1.04669173022	0.87223416888	1.25604294955	11179_7_TSSK1B_TS
0.23328457855	1.04774134066	0.86934859211	1.26274077711	11330_15_CSNK2B_C
0.21877686602	1.04456663266	0.87671555444	1.24455354355	12574_36_EDN2_End
0.12567839111	0.95918315666	0.81137506333	1.13391743177	12675_14_DARS_SYD
0.21330735647	1.04343537677	0.87961556588	1.23776502788	8003_57_LGI3_LGI3
0.21832007223	1.04446454111	0.87694468144	1.24398516906	8079_39_EID3_EID3
0.13538304741	0.95643182332	0.79893587955	1.14497528011	12657_2_TSTA3_FCL
0.19952591917	1.04037816844	0.88660352733	1.22082385177	14178_18_CDKN3_CD
0.21818205352	1.04431139506	0.87680854133	1.24381348766	15381_45_DDR2_Disc
0.22464755375	1.04567843866	0.87344007333	1.25188141755	17384_110_PFKM_K6
0.21270620470	1.04314924744	0.87966186955	1.23702116688	3315_15_GPC2_GPC2
0.22373784901	1.04544950155	0.87385224555	1.25074309299	9386_42_TTC9B_TTC
0.21005858287	1.04244734366	0.88080743233	1.23375033444	12869_68_DDX6_DDX
0.21690711791	1.04388510322	0.87721052888	1.24222871599	14689_3_SEC13_SEC
0.20500712532	1.04134320844	0.88339373433	1.22753381155	19114_8_TK2_KITM
0.21628641594	1.04351234299	0.87712842177	1.24145790144	8076_4_SPACA3_SAC
0.13472104175	0.95719426544	0.80074008755	1.14421755099	8088_56_C1orf210_CA
0.21648687082	1.04348337499	0.87690392699	1.24170678266	8807_13_SNX1_SNX1
0.20652831791	1.04126848433	0.88192436099	1.22940254788	5228_25_KIF23_KIF2
0.20133143942	1.04011825066	0.88456204506	1.22303006506	12543_76_ZNF560_ZN
0.22378472680	1.04469044911	0.87254287522	1.25080172633	3708_62_A2M_a2_Ma
0.22184386076	1.04415343577	0.87333945166	1.24837644211	10731_10_HS3ST5_H
0.20623241008	1.04089725666	0.88155645577	1.22903881188	10833_64_HHIP_HHI
0.20279241493	1.04022347277	0.88344938366	1.22481818788	12697_30_PIP4K2A_P
0.20776563285	1.04123981306	0.88078531099	1.23092464744	19286_30_MYL5_ML
0.21640370755	1.04271121422	0.87567943922	1.24160352255	11572_4_DNM2_DYN
0.20043157084	1.03950924222	0.88432190906	1.22192999377	6214_84_IFNA8_IFNA
0.26551492518	1.05260866233	0.84961508066	1.30410231806	13983_27_CRYZ_QOR
0.13163766773	0.95947903733	0.80705190833	1.14069493377	15384_15_KL_KLOTH
0.13112725398	0.96013980506	0.80857648466	1.14011285599	14628_72_FBXO3_FB
0.22523833521	1.04399522	0.87011619666	1.25262122433	19282_3_RAB13_RAB
0.12459701198	0.96221372688	0.81739372933	1.13269189988	6434_18_AOC2_Amin
0.13188933636	0.95996961466	0.80767411155	1.14098204706	6570_1_COL13A1_CO
0.12928483514	0.96085270711	0.81127098844	1.13801422244	8908_14_KCNE5_KCN

0.12621700256	0.9619758321	0.8156671554	1.1345283371	11458_30_PCBP1_PC
0.21803812746	1.0422420770	0.8734628717	1.2436344833	16599_38_GPN1_GPN
0.24663830181	1.0479386598	0.8581398522	1.2797161580	7905_30_HP_HPT
0.23616286392	1.0457144909	0.8634993677	1.2663805410	9986_14_NPW_Neuro
0.21162785395	1.0408314067	0.8767019403	1.2356879431	3026_5_CAST_Calpas
0.22103763027	1.0426107520	0.8714630455	1.2473703685	8760_10_DCUN1D5_I
0.23452311749	1.0451301992	0.8639501773	1.2643056990	11910_27_DLX4_DLX
0.13083926643	0.9614674329	0.8110476778	1.1397845649	13575_40_DVL2_DVI
0.22999484981	1.0440688302	0.8661094294	1.2585935279	10615_18_MPZ_MYP
0.21311069508	1.0406613772	0.8751169068	1.2375216312	11378_37_SYK_KSYI
0.20275764265	1.0386192888	0.8807572805	1.2247755989	17495_141_SIRT3_SII
0.21810059061	1.0415625810	0.8722698377	1.2437121670	19135_5_NT5C3L_Cy
0.13090563143	0.9616044362	0.8112249942	1.13986021	19581_15_IGFBP5_IG
0.20569914517	1.0391609729	0.8790865836	1.2283835832	3469_74_RPS6KA3_R
0.20956547106	1.0398216697	0.8768081943	1.2331421075	11428_31_PDLIM1_PI
0.21021337565	1.0399602188	0.8764738119	1.2339413249	5275_28_VAV1_VAV
0.19589489815	1.0371234534	0.8842698907	1.2163990528	10700_10_IGSF11_IG
0.19806040614	1.0373082838	0.8826715948	1.2190360288	10924_258_CYB5D2_
0.14008938309	0.9596867131	0.8006061428	1.1503766184	10940_25_SRL_SRCA
0.20291173207	1.0382908579	0.8800647267	1.2249643384	12822_34_APBB1_AP
0.21893520195	1.0413345817	0.8711606137	1.2447506166	14052_26_UNC13A_U
0.22484010700	1.0423381127	0.8677016393	1.2521224945	12817_1_GEM_GEM
0.22541304242	1.0424524611	0.8673949263	1.2528400854	8237_56_SOSTDC1_S
0.23234169038	1.0437508143	0.8635528873	1.2615507149	9526_3_CLPSL1_COL
0.21832538750	1.0408802395	0.8709315363	1.2439917811	7732_45_VAMP4_VA
0.12533153662	0.9644009662	0.8205111345	1.1335241955	13939_14_UGP2_UGI
0.16454625712	1.0306097916	0.9010045768	1.1788580988	6457_50_PCDHGA1_I
0.20989015696	1.0391041955	0.8753143729	1.2335425564	14123_34_C10orf54_C
0.19855433319	1.0369356381	0.8816019669	1.2196382924	17672_184_GIF_Gastr
0.21109598971	1.0393233843	0.8746283980	1.2350308996	3827_22_PAK6_PAK6
0.21460372107	1.0396556478	0.8721231655	1.2393706631	12622_96_ASH1L_AS
0.16335347770	0.9546143551	0.7739491145	1.1774528194	18310_26_SEPW1_SE
0.20201589330	1.0372724559	0.8791263627	1.2238674592	18383_9_FKBP3_FKE
0.20514131868	1.0378746099	0.8774008135	1.2276985494	5870_23_BAD_BAD
0.21386737726	1.0394436947	0.8724097619	1.2384583961	6281_51_ST3GAL2_S
0.10561877801	0.9706409307	0.8477104749	1.1113981063	8971_9_NRXN1_NRX
0.20130713102	1.0369139771	0.8791417016	1.2230003354	17450_51_HARS_Hist
0.20232585636	1.0368014438	0.8780559369	1.2242468717	19277_4_TSTD1_KAT

0.21308679381	1.03882234688	0.87204751360	1.23749205322	5583_62_RYK_RYK
0.12692680369	0.96540120933	0.82090342229	1.13533391244	15295_81_IGF2_IGF_
0.12636281398	0.96568057133	0.82184196760	1.13469377633	17172_19_STATH_ST
0.20643714702	1.03743350211	0.87551990360	1.22929046722	8252_2_NOTUM_NO
0.19547327380	1.03520968777	0.88138101388	1.21588629744	12936_38_RLBP1_RL
0.17934177438	1.03225948860	0.89061626933	1.19642958322	7156_2_FUT10_FUT1
0.23647765360	1.04264261860	0.85816343499	1.26677924733	12763_69_ZNF334_ZI
0.20759153555	1.03727939733	0.87425000900	1.23071036544	15504_39_BIRC7_Liv
0.19838569919	1.03561490311	0.87950592333	1.21943263733	9004_24_LDLRAD4_(
0.19281458689	1.03442202699	0.88238315460	1.21265793000	12825_18_APPL1_DP
0.14696862786	1.02597949460	0.90876103599	1.15831762355	6364_7_TAPBPL_TPS
0.20115367468	1.03567794500	0.87718162388	1.22281267277	19177_7_MOBKL1A_
0.20751744175	1.03666753899	0.87328363077	1.23061918088	5346_24_CPNE1_CPN
0.21534335009	1.03800199233	0.86870824911	1.24028767677	8268_98_HS3ST3A1_
0.19438245522	1.034186	0.88059879111	1.21456070922	18312_68_NDRG3_NI
0.20678960747	1.03634818155	0.87338110888	1.22972381988	18905_5_KRT16_Kera
0.19957501475	1.03485562460	0.87717289099	1.22088379022	17697_2_OVCA2_OV
0.12987669658	0.96677719899	0.82082025688	1.13868797055	19202_10_BTK_BTK
0.19231541867	1.03346645599	0.88119341833	1.21205276077	3832_51_PTK6_PTK6
0.21908349127	1.03807607999	0.86558877600	1.24493521355	11441_11_PYGL_PY(
0.19499530527	1.03380206122	0.87940595560	1.21530528099	18332_17_CPLX1_CP
0.20330695475	1.03512404011	0.87435883200	1.22544856777	4240_31_PKM2_M2_I
0.20013004435	1.03435327822	0.87583524222	1.22156160500	3822_54_MAPKAPK3
0.21061557445	1.03619186500	0.86978352060	1.23443771444	8089_173_NR4A1_NF
0.20319100087	1.03487058000	0.87403203577	1.22530648055	8982_65_THBS3_TSP
0.20088204363	1.03433889377	0.87515252060	1.22248056399	16596_25_GLRX3_GI
0.20163464764	1.03441301133	0.87461945700	1.22340095400	2644_11_PRKCA_PK(
0.20569233377	1.03514047477	0.87230333877	1.22837521622	5032_64_SSRP1_SSRI
0.20502433954	1.03500685677	0.87266089388	1.22755494277	8428_102_NTM_NTR
0.20147756534	1.03432266500	0.87460405799	1.22320879444	11549_6_ISL1_ISL1
0.13483634764	0.96681847522	0.81682909755	1.14434949355	12558_3_UBASH3B_I
0.20445903341	1.03467729222	0.87259838660	1.22686119455	16756_30_GDF7_GDI
0.13549989804	0.96696844133	0.81654052311	1.14510907900	12699_28_CCT8_TCP
0.20837141043	1.03510187860	0.86990462499	1.23167053988	9598_23_CSMD1_CSI
0.22521570620	1.03775469033	0.85976442560	1.25259287917	7948_129_GLTPD2_G
0.19639607918	1.03261313777	0.87615624111	1.21700884177	18823_52_HIST3H2BI
0.21835082773	1.03607251444	0.86288266788	1.24402342899	13388_57_PCSK1_NE
0.19650613042	1.03252561644	0.87591132611	1.21714278244	14098_28_CARS_SY(

0.19221684635	1.03169030710	0.87825369355	1.21193329177	18933_4_TGM4_TGM
0.19548317560	1.03227890410	0.87638884233	1.21589833699	19296_51_MYL7_ML
0.13466386217	0.96806361355	0.81907566111	1.14415212688	4294_16_SPHK1_Sphi
0.13372882240	0.96825294300	0.82016260100	1.14308279911	8759_29_A4GALT_a1
0.20326961768	1.03337154500	0.87143324460	1.22540281339	12779_30_PRDM4_PI
0.25337817034	1.04160335459	0.84210064299	1.28837040822	12656_1_KLC1_KLC1
0.12954169718	0.96979430160	0.82622819559	1.13830657460	13978_122_TBX3_Tb:
0.19891931642	1.03248654699	0.87373401177	1.22008352122	19259_176_EIF2S1_IF
0.13815107973	0.96772553999	0.81565434560	1.148149	6060_2_PIP_PIP
0.19531992778	1.03177843660	0.87568221114	1.21569986044	12737_12_PLS1_PLSI
0.20539291780	1.03346759499	0.86974655322	1.22800747611	5599_88_PCYOX1L_I
0.19988847688	1.03245692888	0.87283755460	1.22126655111	12469_19_MAPRE1_M
0.19814709807	1.03218882500	0.87390477933	1.21914171400	4237_70_LCMT1_LCI
0.21046618997	1.03407102877	0.86635609811	1.23425332233	12621_55_PPP2R1A_I
0.21485585215	1.03465625399	0.86353801960	1.23968318644	12689_56_ARPC1B_A
0.19440386260	1.03097120722	0.87511383199	1.21458671000	12968_2_CSRP2_CSR
0.19986791596	1.03183122500	0.87179786177	1.22124144100	14618_26_VOPP1_EC
0.19644823155	1.03125208210	0.87380252200	1.21707231333	15627_83_AKT1_PKE
0.11296800094	0.97467624033	0.84851471700	1.11959610633	8382_47_MAPKAPK5
0.19854457129	1.03141720833	0.87225192022	1.21962638655	10647_18_ASCC1_AS
0.20123680332	1.03177897444	0.87051711460	1.22291432777	12980_31_RBM22_RE
0.19604765522	1.03095423160	0.87364773700	1.21658488055	18189_12_VPS25_VP!
0.20824023049	1.03294936188	0.86640406311	1.23150898000	19492_5_KAAG1_KA
0.12872979864	0.97133850500	0.82953472160	1.13738276022	9443_137_CTSK_cath
0.19364951307	1.03049013333	0.87495710233	1.21367083260	16781_2_ENGASE_EI
0.24068590573	1.03798372622	0.84693977355	1.27212140655	18343_10_DECR2_DE
0.20382831780	1.03193349244	0.86852415760	1.22608763799	11112_18_TMEM167A
0.13280512008	0.97081007855	0.82526233033	1.14202741844	4829_43_SFN_STRAT
0.20654116142	1.03230356422	0.86679254411	1.22941833777	17341_89_ACAT2_TH
0.12511445933	0.97285138477	0.83513461244	1.13327815999	15347_12_HPX_Hemc
0.19845725543	1.03077877799	0.87124850560	1.21951989844	19131_184_TIAL1_TI
0.19028082128	1.02954230088	0.87629529460	1.20958922833	2682_68_HSPD1_HSP
0.14262176372	0.96918055599	0.81445958788	1.15329350177	12575_30_MTHFD1_C
0.19456996218	1.02992020799	0.87318546544	1.21478846911	6368_9_CSTL1_CST1
0.22422194645	1.03456185800	0.85533170733	1.25134872100	8983_7_GOLM1_GOL
0.21092914770	1.03238129844	0.86312737811	1.23482486177	11969_5_S100A2_S10
0.20402636009	1.03091879277	0.86664530911	1.22633047922	19229_92_HOMER1_I
0.14159427022	0.97034245488	0.81725287355	1.15210910877	19606_28_IHH_ihh

0.2012380972	1.030379380	0.868155904	1.222915910	12706_2_CDC42BPA_
0.1949907260	1.029203361	0.871603560	1.215299715	11395_5_PTK2B_FAK
0.2082479765	1.031238893	0.863530380	1.231518519	12872_35_CNGA2_CI
0.1944653842	1.029042073	0.871788267	1.214661435	11211_7_TBCE_TBCI
0.2100303642	1.031411769	0.862281636	1.233715520	6625_31_ATAD1_AT
0.1778705549	1.026503772	0.882008761	1.194670667	12384_92_COPS7B_C
0.1973563218	1.029324242	0.869748405	1.218178026	8051_10_AGGF1_AG
0.1345777104	0.972602086	0.826844870	1.144053560	19239_5_ARF1_ARF1
0.1977209846	1.028997268	0.868879020	1.218622332	15314_49_CLIC4_CLI
0.2247739031	1.032798485	0.851948061	1.252039602	11424_4_FAH_FAAA
0.1394210802	0.972347376	0.822418910	1.149608075	8890_9_TMEM132B_
0.1945123496	1.028262468	0.870426949	1.214718484	18236_3_HSPC159_LI
0.2136930906	1.031013687	0.858466064	1.238242568	10085_25_STAR_STA
0.2042709932	1.029471272	0.864001902	1.226630517	3887_90_KPNB1_IMI
0.1448911402	0.972099827	0.817516090	1.155913730	13654_1_ROCK2_RO
0.1919232711	1.027123989	0.870752094	1.211577550	7989_5_C1orf226_CA
0.1921101342	1.027092728	0.870536405	1.211803970	11476_43_VPS4A_VP
0.1932466282	1.027143425	0.869633450	1.213181961	17773_26_NAPG_SN
0.1469461770	0.972279797	0.816139898	1.158291618	15482_12_A2ML1_A2
0.1910595604	1.026745869	0.870862952	1.210531549	17411_55_UFD1L_UF
0.1977432976	1.027605911	0.866511567	1.218649523	11696_7_CRABP2_R
0.1394840790	0.973905290	0.825004436	1.149680501	10704_91_LRMP_LRI
0.1351529347	0.974865861	0.830220687	1.144711837	3806_55_EPHA5_Eph
0.1430164887	0.973516652	0.821439339	1.153748825	13620_10_ASNA1_AS
0.1526642012	0.971747907	0.810598896	1.164933729	13632_10_ZYX_Zyxi
0.2071822471	1.028587557	0.860011831	1.230206753	12685_57_HOMER2_
0.1914253239	1.026307471	0.869801233	1.210974398	14203_3_ANXA7_AN
0.1939657089	1.026655536	0.868182985	1.214054651	15641_20_TMEFF1_T
0.2089329949	1.028660490	0.858629235	1.232362421	3196_6_HAPLN1_HPI
0.2464792123	1.033835267	0.835330088	1.279512584	3049_61_PRSS1_Tryp
0.1401888641	0.974965961	0.826219911	1.150491064	7115_5_SEMG1_Sem
0.1891981790	1.025387349	0.870178171	1.208280384	17770_42_EMC8_EM
0.1944813986	1.026053081	0.866717288	1.214680887	12499_108_SH3GL2_
0.1611887032	0.971504191	0.803315215	1.174906656	12682_5_CCBL2_KA
0.1917187318	1.025619916	0.868381383	1.211329760	13969_24_KPNA6_IM
0.1970363072	1.026235558	0.864813253	1.217788254	13427_66_MAN1C1_
0.1456738556	0.974898637	0.821587031	1.156818836	15640_54_TAGLN_T
0.1440489147	0.975244990	0.823507971	1.154940600	19270_26_YARS_SY

0.14686792129	0.97482337500	0.82047989055	1.15820097921	11565_58_ZNF23_ZN
0.19401613019	1.02527529433	0.86580651644	1.21411586677	19575_4_EPOR_EPO_
0.18931760606	1.02455743011	0.86866639899	1.20842469455	12483_62_RORB_ROI
0.19188292936	1.02467474711	0.86663927955	1.21152867421	19586_89_RAB3C_RA
0.19664685764	1.02521644921	0.86343268788	1.21731407961	6520_87_MGP_MGP
0.19591469295	1.02506937977	0.86381720811	1.21642313141	3845_51_DYNLRB1_I
0.20415248320	1.02607462011	0.85841163233	1.22648515755	2781_63_CCL4L1_LA
0.18790708088	1.02382504588	0.86864933333	1.20672138261	14153_8_EFNA3_Eph
0.21584851677	1.02738880400	0.85060481677	1.24091438701	17335_18_VAMP2_VA
0.18910637325	1.02391267433	0.86775671561	1.20816946255	4551_72_ADGRG5_G
0.16200430285	0.97337993621	0.80576278555	1.17586530088	7856_51_FAM151A_F
0.19762352269	1.02493960777	0.86212402388	1.21850356855	14268_4_SRXN1_SRX
0.18664563047	1.02353137033	0.86924689599	1.20520012311	6467_65_POGLUT1_F
0.14112678549	0.97690058121	0.82872444955	1.15157064111	10908_2_GALNT13_G
0.13837841634	0.97738490668	0.83182941511	1.14841004511	8097_77_LIPN_LIPN
0.19389619059	1.02431511999	0.86428926961	1.21397025481	11708_2_LCN1_LCN1
0.21026129010	1.02636054611	0.85365930844	1.23400044999	13022_20_TP53I11_P5
0.14200633329	0.97695840499	0.82809388898	1.15258394811	18235_16_PGP_PGP
0.19235628598	1.02402072788	0.86512372377	1.21210229511	4160_49_MMP2_MMI
0.18857475619	1.02346972188	0.86746711921	1.20752734977	3836_51_UFM1_UFM
0.18175230359	1.02259888999	0.87191994333	1.19931709055	6937_251_C1orf43_CA
0.19075711818	1.02358010399	0.86576277261	1.21016548911	12774_12_HCN1_HCN
0.23508734711	1.02915374411	0.83726585277	1.26501925961	15566_10_CNN1_Calp
0.22900914213	1.02832594144	0.84101584277	1.25735353388	18185_118_ALDOB_A
0.15554319246	0.97525137261	0.81410718999	1.16829239641	18413_24_ARL4D_AI
0.13405967086	0.97863760833	0.83757253401	1.14346104899	8960_3_ERAP2_LRAI
0.18697714188	1.02299711041	0.86805186201	1.20559972691	3844_2_PPIA_Cycloph
0.18368603861	1.02229613961	0.86972030341	1.20163849577	18894_1_GPX2_GPX2
0.14138143783	0.97812157601	0.83058579541	1.15186392861	12812_25_ACYP2_AC
0.14486489353	0.97755928899	0.82674616633	1.15588339233	7198_197_FAM20B_F
0.19163096486	1.02308734888	0.86417392511	1.21122345041	8220_15_C10orf35_CJ
0.19163478227	1.02276405799	0.86362456455	1.21122807421	3397_7_PTPN11_SHP
0.17921738113	1.02115203911	0.87166116661	1.19628076477	11126_102_TRIO_TRI
0.17904436090	1.02103582233	0.87161356499	1.19607380199	12888_18_SAP30_SAI
0.21454429289	1.02492824355	0.84764014955	1.23929701188	10041_3_HNF4A_HNI
0.19512160715	1.02261051221	0.86036011399	1.21545878599	12826_5_SLC5A5_SC
0.18270019010	1.02108522161	0.86851694655	1.20045444599	8978_30_MAP4K3_M
0.14856188284	0.97842953044	0.82516252811	1.16016458988	12628_31_LANCL2_I

0.1405221211	0.979605056	0.833823352	1.150874537	14099_20_PSMA4_PS
0.1864739376	1.021411296	0.865798265	1.204993216	17819_30_FAHD1_FA
0.1527682327	0.977777947	0.820604842	1.165054926	19150_20_PRPSAP2_
0.1497446973	0.978204256	0.823807612	1.161537661	3041_55_MRC2_MRC
0.1452063955	0.978990013	0.828884821	1.156278196	3628_3_MAP2K2_MP
0.1403498477	0.979991671	0.834625415	1.150676289	13567_1_DPYSL2_DP
0.1899061052	1.021265949	0.862586249	1.209136060	10948_14_PLD3_PLD
0.1933699853	1.021509702	0.860013907	1.213331625	11816_84_JAK2_JAK
0.1883320895	1.020918455	0.863357213	1.207234358	9126_171_NT5DC3_N
0.1868739287	1.020675320	0.864205272	1.205475299	11277_23_ATF6_ATF
0.1936473682	1.021327414	0.859468562	1.213668229	18301_10_NAIF1_NA
0.1927417161	1.021274082	0.860157454	1.212569565	3415_61_IBSP_BSP
0.1457266017	0.979893036	0.829982782	1.156879855	5020_50_PGK1_phos
0.1871629535	1.020597690	0.863824116	1.205823762	6603_18_KAL1_KAL
0.1835397388	1.020068837	0.866061364	1.201462709	9895_77_ERCC4_XP
0.1481405136	0.979796393	0.827818382	1.159675835	18419_20_ARPC5_p1
0.1884125664	1.020464068	0.862519449	1.207331517	12643_4_ARRB1_AR
0.1498308215	0.979922605	0.826633218	1.1616377	13133_73_LTBP4_LT
0.1924745691	1.020660855	0.859354340	1.212245674	18340_2_PSMB4_Prot
0.1542079013	0.979421999	0.822182198	1.166733426	6629_3_DEFB1_HBD_
0.1511549660	0.979969067	0.825617647	1.163176897	10588_39_ST3GAL5_
0.1844839578	1.019665558	0.864559994	1.202597688	17380_2_UBE2K_UB
0.1776775721	1.018794308	0.868977698	1.194440138	17784_23_GMFB_GM
0.1475158274	0.980648569	0.829777183	1.158951627	19134_66_POLR2K_
0.1438954742	0.981085288	0.833528621	1.154763399	7083_74_MATN4_MA
0.1474514820	0.980853938	0.830178182	1.158877057	5858_6_YWHAZ_14_
0.1410565629	0.981916147	0.837314702	1.151489777	12422_143_ALOX15E
0.1546767826	0.980150388	0.823019565	1.167280614	6431_68_PCYOX1_P
0.1486383895	0.981027672	0.829487190	1.160253353	15404_3_IFNA21_IF
0.1478380494	0.981248376	0.830524891	1.159325127	3839_60_AIP_AIP
0.2003072321	1.020589157	0.852529812	1.221778070	18926_7_PSMA3_PS
0.1799092622	1.018336033	0.866260721	1.197108735	15298_199_NETO1_N
0.1565412818	0.980373459	0.821860439	1.169459038	9885_41_RAP2A_RA
0.1868290389	1.018932337	0.861294889	1.20542119	12696_166_PRMT3_A
0.1852404793	1.018762563	0.862376746	1.203507824	17726_3_SAR1A_SA
0.1890638810	1.019001164	0.859488281	1.208118125	6925_26_SNX8_SNX
0.1416798527	0.982583086	0.837930097	1.152207713	8093_13_KIAA1161_
0.1867308030	1.018669118	0.860934523	1.205302777	13930_3_APOBEC3G_

0.1894683266	1.018895897	0.858963240	1.208606842	4220_39_FER_FER
0.1965715259	1.019539655	0.853961547	1.217222380	18400_52_ALKBH2_
0.2095847251	1.020792950	0.844994407	1.233165850	14204_55_FOXJ2_FO
0.1918269747	1.018654337	0.856533356	1.211460885	11416_23_FBXL4_FB
0.1973970013	1.019304162	0.852862790	1.218227582	12854_3_TEAD3_TE
0.1485245168	0.982382884	0.831875236	1.160121239	13544_9_HMHA1_HM
0.1904470791	1.018463434	0.857394645	1.209790348	13578_98_ABLIM3_A
0.1666098017	0.980293674	0.813494616	1.181293236	18284_77_WWC1_WV
0.1575331276	0.981331443	0.822651059	1.170619537	8222_49_CDHR3_CD
0.1857557487	1.018009831	0.860659264	1.204128114	9100_32_C5orf46_CE
0.1953363551	1.018814697	0.853801477	1.215719831	12423_38_A1CF_A1C
0.1771166505	1.016948386	0.866317403	1.193770338	13583_19_APAF1_Ap
0.1819997246	1.017413509	0.862886200	1.199613863	15526_33_GSS_GSHE
0.1716523268	0.980097048	0.809078209	1.187264981	6461_54_APOC3_Apo
0.1471028713	0.982999889	0.834105476	1.158473130	4258_15_PA2G4_PA2
0.1835928523	1.017469670	0.861607719	1.201526524	9840_2_CDC42_Cdc4
0.1519430210	0.982679902	0.829537708	1.164093905	18403_25_AMPD2_Al
0.1989676197	1.018688151	0.850495402	1.220142456	15394_79_UNC5B_UI
0.2249347673	1.021158835	0.832719376	1.252241026	6425_87_MMP19_MM
0.2063528095	1.019264838	0.845193598	1.229186796	8048_9_FTMT_FTMT
0.1982132714	1.018439770	0.850722208	1.219222391	16610_13_LRP10_LRI
0.1514349150	0.983183865	0.830810809	1.163502572	10589_7_RAET1L_RE
0.1832976305	1.016741967	0.860629741	1.201171860	12707_26_DPYSL3_D
0.1872392450	1.017096423	0.857841956	1.205915759	4564_2_PLXNC1_PL
0.2077469663	1.018948054	0.843491533	1.230901670	4430_44_COLEC11_C
0.3669266433	1.033441958	0.739976562	1.443292040	6472_40_PMEL_GP1
0.1820240734	1.016391020	0.861131723	1.199643073	3905_62_UBE2N_UBI
0.1948075045	1.017471710	0.852002485	1.215077067	18898_36_HDGFL1_I
0.1577761705	0.983140261	0.825485867	1.170904082	12630_8_ARFIP2_AR
0.1836809335	1.016285208	0.859527138	1.201632361	19173_5_ZFAND1_ZI
0.1505886403	0.984399400	0.833571515	1.162518346	11278_4_COL11A2_C
0.1778274108	1.015398122	0.863064491	1.194619125	5900_11_HINT1_HIN
0.1863837813	1.016007742	0.856739099	1.204884583	10496_11_CLCA1_Ca
0.1862442841	1.015815249	0.856533970	1.204716517	13563_259_ACBD7_A
0.2038449977	1.017219687	0.843918983	1.226108089	9509_4_TMEM38B_T
0.1823695670	1.015249295	0.858901373	1.200057613	7808_5_GLCE_GLCE
0.1784345299	1.014933044	0.861750717	1.195344621	8303_102_C12orf10_
0.1550486269	0.984908539	0.830720719	1.167714742	11421_10_EHD4_EHI

0.1800625813	1.014879838	0.860258681	1.197292288	16079_2_TEC_TEC
0.1810795408	1.015036860	0.859650227	1.198510506	18206_18_ADH6_AD
0.1816225865	1.015056627	0.859216988	1.199161528	9842_2_CTNNB1_b_
0.1943988611	1.015957221	0.849815191	1.214580635	6557_50_LRRC15_LR
0.1809529992	1.014849180	0.859441106	1.198358854	9380_2_PLA2G12B_s
0.1857201092	1.015191011	0.855930120	1.204085200	19250_50_MYCBP_M
0.2057598943	1.016803497	0.841615405	1.228458208	13973_62_TTL_TTL
0.1785174665	1.014209308	0.860450782	1.195443763	11264_33_XDH_XDH
0.1803003849	1.014352917	0.859161293	1.197577043	3379_29_PRKCI_KPC
0.1532208159	0.985915488	0.833943107	1.165582329	11358_15_GRB10_GR
0.1796063341	1.014112397	0.859350124	1.196746152	12993_21_NPLOC4_N
0.1772799733	1.013967608	0.861105669	1.19396532	6342_10_NPNT_Neph
0.1489106140	0.986230440	0.838080523	1.160569245	8038_41_NSDHL_NS
0.1485980067	0.986585182	0.838945758	1.160206500	17680_12_EPHB1_EP
0.1788771030	1.013741131	0.859347458	1.195873765	16768_3_JUP_PLAK
0.1813355302	1.013808079	0.857350638	1.198817351	10981_56_NRG3_NR
0.1862564628	1.014199425	0.853800817	1.204731189	19623_26_RPSA_40S_
0.1546606848	0.986599032	0.833898299	1.167261824	14291_53_AGAP2_A
0.1573346893	0.986343132	0.831240055	1.170387264	6404_20_C1QL1_C1Q
0.4289163224	1.031838724	0.693342230	1.535592534	14634_13_LHCGR_L
0.1534694002	0.987021378	0.835607260	1.165872111	15529_33_CSRP1_Cy
0.1767161999	1.012941532	0.859848397	1.193292388	5882_34_EEF1B2_EF_
0.1712606724	0.985538564	0.818407651	1.186800074	8009_121_SURF1_SU
0.1560644605	0.987054852	0.833498152	1.168901548	4394_71_FGF8_FGF_
0.1907449023	1.013767854	0.849253946	1.210150706	9361_7_PCDHAC2_P
0.1489029334	0.987619117	0.840448784	1.160560332	9580_5_LAMC2_Lam
0.2415664831	1.017351161	0.812888126	1.273242101	16305_10_CDH11_Ca
0.1614363594	0.986785793	0.828580783	1.175197665	18380_78_ALB_Albu
0.1854202358	1.013070858	0.852614394	1.203724181	12753_6_APBB2_AP
0.1791827609	1.012619746	0.857185271	1.196239349	15523_9_ALAD_HEM
0.1544226843	0.987531082	0.835673495	1.166984048	5139_32_UNC5C_UN
0.1784169644	1.012365825	0.857411786	1.195323624	13954_9_GNPNAT1_
0.1858970981	1.012843387	0.851825251	1.204298329	16867_76_PCTK1_PC
0.1745924405	1.012077141	0.860206458	1.190760811	19357_11_VAMP7_V
0.1806223850	1.012587773	0.855898082	1.197962725	5464_52_GRB2_GRB
0.1609406044	0.987182179	0.829657794	1.174615199	8298_8_SSMEM1_CG
0.1920875283	1.013132143	0.847051148	1.211776577	15444_45_SERPINB4_
0.1746430228	0.986340908	0.816972786	1.190821044	6900_30_UBE2J1_UB

0.1418805938	0.988870935	0.848518402	1.152439031	8248_222_SIGLEC14_
0.1690045613	1.011599630	0.864210574	1.184125540	8310_6_ZG16B_U773
0.1574728572	0.987909388	0.833766866	1.170548985	14114_18_PIANP_PIA
0.1729713156	1.011627760	0.860837125	1.188832003	8004_15_GAPDHS_G
0.1830622562	0.986132430	0.809780949	1.200889168	14007_22_PAPSS1_P
0.1821639999	1.011949551	0.853502711	1.199810946	19159_9_POLD4_DN
0.1774206013	1.011494761	0.856790195	1.194133241	7839_99_FAM19A3_F
0.1730563401	1.011010020	0.859713025	1.188933087	12838_28_DUSP15_D
0.1815804044	1.011528995	0.853291274	1.199110946	13464_8_NRAC_NRA
0.1763812508	1.011113253	0.857034297	1.192892763	18329_4_PRPS2_PRP
0.1750034920	1.010859829	0.857785747	1.191250376	10832_24_B4GALT6_
0.1527287248	0.989178064	0.839884780	1.165008898	13498_1_MIOX_MIO
0.1756398066	0.987721214	0.818444744	1.192008627	15559_5_ANTXR2_A
0.1771548178	1.011076213	0.856308839	1.193815902	15608_5_RPS6KB1_K
0.1751949664	1.010832920	0.857575860	1.191478492	6918_183_CCK_CCK
0.1730381312	1.010697931	0.859197982	1.188911438	7761_125_CHKB_CH
0.1547361938	0.989315739	0.838433768	1.167349966	9594_30_SLC30A5_Z
0.1856374777	1.011226715	0.849328577	1.203985709	11351_233_NHEJ1_N
0.1550828743	0.989497018	0.838450336	1.167754734	9832_33_HGD_HGD
0.1761635414	1.010521161	0.856217245	1.192633087	13713_164_SEC22A_
0.1886667429	1.011057051	0.846475513	1.207638431	9278_9_EPYC_Epiphy
0.1558914429	0.989819232	0.838318369	1.168699325	9314_9_PSG5_PSG5
0.1598626125	0.989727652	0.834841362	1.173349656	12652_37_AHCYL1_
0.1601333041	0.989795877	0.834730477	1.173667315	15339_32_CFL2_COF
0.1573306754	0.989969177	0.837366345	1.170382566	16847_39_RAB3B_R
0.1591062259	0.989926227	0.835808350	1.172462486	13944_3_SULT1A3_S
0.2006362753	0.987360584	0.797657302	1.222180153	6574_11_FAIM3_FAI
0.1688084423	0.989764206	0.827467438	1.183893333	8756_41_KCNE5_KC
0.1527096578	0.990802769	0.842662100	1.164986685	8758_2_PCDHA7_PC
0.1652496854	0.990194626	0.831139846	1.179687632	7999_23_ENTPD1_CI
0.1528284211	0.991042347	0.842969544	1.165125050	13982_33_RGS18_RG
0.1587146937	0.990815955	0.837639343	1.172003519	12457_10_ADI1_MTN
0.1865449552	1.009702469	0.846002005	1.205078795	5676_54_ASIP_ASIP
0.1547342570	0.991307730	0.841815178	1.167347705	5364_7_SET_SET
0.1746430516	1.008805371	0.854610566	1.190821079	12701_1_EIF1B_EIF1
0.1735630319	1.008651646	0.855273343	1.189535663	15303_63_S100A5_S1
0.1785880726	1.008842440	0.851308311	1.195528172	13986_6_LANCL1_L
0.1546800921	0.991768414	0.842643423	1.167284477	7215_18_CYB5R3_N

0.16737668379	1.00788154188	0.8592671584	1.18219949688	18257_64_SRP19_SRI
0.16764822232	0.99162765911	0.83155037911	1.18252055311	11241_8_ASL_ARLY
0.18974560088	1.00871940560	0.84165728000	1.20894200477	12340_17_AARS_SYA
0.16447445200	0.9917895054	0.83446604599	1.17877345388	12573_80_TRIM3_TR
0.17325234403	1.00764841600	0.85383807277	1.18916614622	10346_5_STAT3_STA
0.17297378277	1.00763057388	0.8540457064	1.18883493677	9940_35_DUSP28_DU
0.15898764802	0.99264987388	0.84051356188	1.17232346600	8403_18_FASN_Fatty_
0.16640674889	1.00700087060	0.85859856700	1.18105339622	19564_61_IRF4_IRF4
0.16112756716	0.99270687033	0.8388131724	1.17483482960	7921_65_FJX1_FJX1
0.15171620668	0.99310799299	0.84742923655	1.1638299024	7927_16_ST6GALNAC
0.19245711408	1.00809393733	0.8383375964	1.21222451522	8989_40_SCUBE1_SC
0.15674683712	0.9930098904	0.84301026299	1.16969945199	14175_78_SCP2D1_SC
0.15413135453	0.99319032722	0.8455252194	1.16664412077	2942_50_CYCS_Cytoc
0.17444545691	1.00671744911	0.85124484133	1.19058580233	13597_20_RAB31_RA
0.17371763522	1.00663234400	0.85172059877	1.1897195834	19341_36_ACBD6_AC
0.16238522276	0.99338144711	0.83889785333	1.17631329677	10428_1_KIR2DS2_K
0.17152995487	1.00632676560	0.85306777177	1.18711970222	11307_33_WWP1_WW
0.17049355698	1.00604343188	0.85347155077	1.18589001111	17350_13_CHMP2B_C
0.18717497659	1.00666554899	0.84039092211	1.20583826000	5843_60_EHMT2_NG
0.18278771829	0.99326876155	0.82176919411	1.20055952422	12758_47_GRID2_GR
0.20178310918	0.99257047000	0.80517338311	1.22358259555	6077_63_CECR1_CEC
0.15864092869	0.99429820600	0.84359972933	1.17191706933	17814_8_BPNT1_BPN
0.17238285880	0.99430388888	0.83209584166	1.18813263311	3504_58_HAMP_LEA
0.1758362265	1.00532744122	0.84771598277	1.19224278499	10464_6_ANTXR1_A
0.17757233300	1.00540977011	0.84638414266	1.19431444311	11494_4_ELL2_ELL2
0.18479928608	1.00549469099	0.84043136800	1.20297696166	13691_10_SLC5A8_SC
0.1618407455	0.99484575977	0.84183109566	1.17567299522	3405_6_UFC1_UFC1
0.17436880901	0.99449157233	0.8307585174	1.19049454999	4188_1_AKR7A2_Afla
0.19248582809	1.00575362088	0.83442570922	1.21225932355	7986_98_NAALAD2_
0.1755750831	1.00495549722	0.84731007522	1.19193147922	13948_50_GABBR2_C
0.1584870559	0.99525123877	0.8453477474	1.17173675700	9333_59_ERP27_ERP
0.15380845595	0.99585185088	0.85033744922	1.16626747377	12715_30_DCP1B_DC
0.16253414078	0.99553109722	0.84240702655	1.17648848400	14143_8_HIST2H2BE_
0.16145921996	0.99561154299	0.84344933066	1.1752245314	18216_22_IL11RA_IL
0.18020470580	1.00469657911	0.84296188400	1.19746246555	8597_1_TMEM9_TMI
0.15874028571	0.99561877799	0.84575802622	1.17203351333	9595_11_B4GALT2_E
0.18077607421	1.0046046	0.84232612800	1.19814685322	9893_27_OAZ1_Ornit
0.15754360516	0.99601870255	0.84745114022	1.1706318024	12578_13_ARF3_ARF

0.1716772959	0.995831639	0.835243951	1.187294626	19158_1_PAF_PAF
0.1841754264	0.995661842	0.824588656	1.202226706	10693_43_KLRG2_K
0.1687408605	1.003721259	0.851026377	1.183813326	12688_115_RPS6KA6
0.1740459135	0.996009342	0.833565332	1.190110206	13673_21_CCT7_TCP
0.1655366409	0.996150028	0.840926142	1.180026199	7073_69_C22orf15_C
0.1641147803	0.996131926	0.842092066	1.178349558	8318_13_SPRED1_SP
0.1728912841	1.003579510	0.847262219	1.188736863	7717_95_CFTR_CFT
0.1643006720	0.996507212	0.842570050	1.178568624	19367_34_DCI_D3D2
0.1696496292	1.003423890	0.849749614	1.184889627	12956_40_KIAA1279
0.1634365783	0.996671324	0.843576207	1.177550670	17712_7_IDI1_IDI1
0.1719825564	1.003284308	0.847533677	1.187657116	11231_12_FDX1L_A
0.1792657520	1.003394797	0.841568677	1.196338631	11381_56_PRPS1_PR
0.2148004969	1.003833466	0.812899151	1.239614565	3332_57_HFE2_RGM
0.1630305851	0.997415100	0.845178799	1.177072690	15622_13_OPCML_O
0.1579706722	0.997485910	0.849586786	1.171131847	9475_22_GPX5_GPX
0.1470911760	0.997772142	0.859373312	1.158459581	15509_2_NAGLU_NA
0.1684792291	1.002488620	0.849159560	1.183503644	16853_5_NRK1_NRK
0.1618635433	1.002426741	0.854690434	1.175699798	19388_2_CDH12_Cad
0.1725768100	1.002297366	0.845364530	1.188363095	8013_9_LMAN2L_L
0.2016497563	1.002445086	0.821383182	1.223419438	5708_1_LEAP2_LEA
0.2076829221	1.002278489	0.816171211	1.230822840	9468_8_LMAN2_Lect
0.1692350303	1.001476711	0.846805887	1.184398475	19275_68_IDH3G_ID
0.1622194861	1.001285038	0.852441188	1.176118354	13651_54_ZFP91_ZF
0.1674908534	1.001377135	0.848115476	1.182334475	17327_3_CNPY3_CN
0.1687540123	1.001319830	0.846947904	1.183828895	18402_1_AMMECR1
0.1651892743	1.001057342	0.849526871	1.179616368	3363_31_CSK_CSK
0.1762172194	1.001003006	0.840118596	1.192697107	5132_71_IL27RA_TC
0.1826194100	0.998948808	0.831334616	1.200357477	7161_25_H6PD_G6P
0.1588387191	1.000796122	0.854492881	1.172148886	13694_24_CRLF2_TS
0.1523739328	0.999284180	0.857438274	1.164595635	6606_61_KISS1_KISS
0.1616162686	0.999439823	0.849814714	1.175409113	18931_40_SLIT3_SLI
0.1655280271	1.000439131	0.848190555	1.180016034	10571_14_MGAT1_M
0.1823997669	0.999511056	0.832453517	1.200093855	12368_18_KAT2B_PC
0.1680975049	1.000512871	0.846138665	1.183051958	13929_27_CROT_OC
0.1629479794	0.999605306	0.848964827	1.176975461	12535_2_XRCC1_XR
0.1706749713	0.999661443	0.842524784	1.186105168	6406_3_NMES1_NM
0.1966646196	0.999679023	0.820938832	1.217335701	6264_9_CYR61_CYR
0.1816608204	1.000248069	0.834297902	1.199207378	9099_19_SYT5_SYT5

fdr
0.000450955367516658
0.00944895517335718
0.0117072407787644
0.024393800545416
0.093096068535657
0.102220046203026
0.180618292330875
0.180618292330875
0.207901250144216
0.245319301959184
0.245319301959184
0.245319301959184
0.245319301959184
0.245319301959184
0.245319301959184
0.245319301959184
0.245319301959184
0.245319301959184
0.245319301959184
0.245319301959184
0.245319301959184
0.245319301959184
0.245319301959184
0.245319301959184
0.245319301959184
0.245319301959184
0.245319301959184
0.245319301959184
0.245319301959184
0.245319301959184
0.245319301959184
0.245319301959184
0.245319301959184
0.245319301959184
0.245319301959184
0.245319301959184
0.245319301959184

0.245319301959184
0.245319301959184
0.245319301959184
0.245319301959184
0.245319301959184
0.245319301959184
0.245319301959184
0.245319301959184
0.245319301959184
0.245319301959184
0.245319301959184
0.245319301959184
0.245319301959184
0.245319301959184
0.245319301959184
0.245319301959184
0.245319301959184
0.245319301959184
0.245319301959184
0.245319301959184
0.245319301959184
0.245319301959184
0.245319301959184
0.245319301959184
0.245319301959184
0.245319301959184
0.245319301959184
0.245319301959184
0.245319301959184
0.245319301959184
0.245319301959184
0.245319301959184
0.245319301959184
0.245319301959184
0.245319301959184
0.245319301959184
0.245319301959184
0.245319301959184

0.245319301959184
0.245319301959184
0.245319301959184
0.245319301959184
0.245319301959184
0.245319301959184
0.245319301959184
0.245319301959184
0.245319301959184
0.245319301959184
0.245319301959184
0.245319301959184
0.245319301959184
0.245319301959184
0.245319301959184
0.245319301959184
0.245319301959184
0.245319301959184
0.245319301959184
0.245319301959184
0.245319301959184
0.245319301959184
0.245319301959184
0.245319301959184
0.245319301959184
0.245319301959184
0.245319301959184
0.245319301959184
0.245319301959184
0.245319301959184
0.245319301959184
0.245319301959184
0.245319301959184
0.245319301959184
0.245319301959184
0.245319301959184
0.245319301959184
0.245319301959184

0.245319301959184
0.245319301959184
0.245319301959184
0.245319301959184
0.245319301959184
0.245319301959184
0.245319301959184
0.245319301959184
0.245319301959184
0.245319301959184
0.245319301959184
0.245319301959184
0.245319301959184
0.245319301959184
0.245319301959184
0.245319301959184
0.245319301959184
0.245319301959184
0.245319301959184
0.245319301959184
0.245319301959184
0.245319301959184
0.245319301959184
0.245319301959184
0.245319301959184
0.245319301959184
0.245319301959184
0.245319301959184
0.245319301959184
0.245319301959184
0.245319301959184
0.245319301959184
0.245319301959184
0.245319301959184
0.245319301959184
0.245319301959184
0.245319301959184
0.245319301959184

0.245319301959184
0.245319301959184
0.245319301959184
0.245319301959184
0.245319301959184
0.245319301959184
0.245319301959184
0.245319301959184
0.245319301959184
0.245319301959184
0.245319301959184
0.245319301959184
0.245319301959184
0.245319301959184
0.245319301959184
0.245319301959184
0.245319301959184
0.245319301959184
0.245319301959184
0.245319301959184
0.245319301959184
0.245319301959184
0.245319301959184
0.245319301959184
0.245319301959184
0.245319301959184
0.245319301959184
0.245319301959184
0.245319301959184
0.245319301959184
0.245319301959184
0.245319301959184
0.245319301959184
0.245319301959184
0.245319301959184
0.245319301959184
0.245319301959184
0.245319301959184

0.245319301959184
0.245319301959184
0.245319301959184
0.245319301959184
0.245319301959184
0.245319301959184
0.245319301959184
0.245319301959184
0.245319301959184
0.245319301959184
0.245319301959184
0.245319301959184
0.245319301959184
0.245319301959184
0.245319301959184
0.245319301959184
0.245319301959184
0.245319301959184
0.245319301959184
0.245319301959184
0.245319301959184
0.245319301959184
0.245319301959184
0.245319301959184
0.245319301959184
0.245319301959184
0.245319301959184
0.245319301959184
0.245319301959184
0.245319301959184
0.245319301959184
0.245319301959184
0.245319301959184
0.245319301959184
0.245319301959184
0.245319301959184
0.245319301959184
0.245319301959184

0.245319301959184
0.245319301959184
0.245319301959184
0.245319301959184
0.245319301959184
0.245319301959184
0.245319301959184
0.245319301959184
0.245319301959184
0.245319301959184
0.245319301959184
0.245319301959184
0.245319301959184
0.245319301959184
0.245319301959184
0.245319301959184
0.245319301959184
0.245319301959184
0.245319301959184
0.245319301959184
0.245319301959184
0.245319301959184
0.245319301959184
0.245319301959184
0.245319301959184
0.245319301959184
0.245319301959184
0.245319301959184
0.245319301959184
0.245319301959184
0.245319301959184
0.245319301959184
0.245319301959184
0.245319301959184
0.245319301959184
0.245319301959184
0.245319301959184
0.245319301959184

0.245319301959184
0.245319301959184
0.245319301959184
0.245319301959184
0.245319301959184
0.245319301959184
0.245319301959184
0.245319301959184
0.245319301959184
0.245319301959184
0.245319301959184
0.245319301959184
0.245319301959184
0.245319301959184
0.245319301959184
0.245319301959184
0.245319301959184
0.245319301959184
0.245319301959184
0.245319301959184
0.245319301959184
0.245319301959184
0.245319301959184
0.245319301959184
0.245319301959184
0.245319301959184
0.245319301959184
0.245319301959184
0.245319301959184
0.245319301959184
0.245319301959184
0.245319301959184
0.245319301959184
0.245319301959184
0.245319301959184
0.245319301959184
0.245319301959184
0.245319301959184

0.245319301959184
0.245319301959184
0.245319301959184
0.245319301959184
0.245319301959184
0.245319301959184
0.245319301959184
0.245319301959184
0.245319301959184
0.245319301959184
0.245319301959184
0.245319301959184
0.245319301959184
0.245319301959184
0.245319301959184
0.245319301959184
0.245319301959184
0.245319301959184
0.245319301959184
0.245319301959184
0.245319301959184
0.245319301959184
0.245319301959184
0.245319301959184
0.245319301959184
0.245319301959184
0.245319301959184
0.245319301959184
0.245319301959184
0.245319301959184
0.245319301959184
0.245319301959184
0.245319301959184
0.245319301959184
0.245319301959184
0.245319301959184
0.245319301959184
0.245319301959184

0.245319301959184
0.245319301959184
0.245319301959184
0.245319301959184
0.245319301959184
0.245319301959184
0.245319301959184
0.245319301959184
0.245319301959184
0.245319301959184
0.245319301959184
0.245319301959184
0.245319301959184
0.245319301959184
0.245319301959184
0.245319301959184
0.245319301959184
0.245319301959184
0.245319301959184
0.245319301959184
0.245319301959184
0.245319301959184
0.245319301959184
0.245319301959184
0.245319301959184
0.245319301959184
0.245319301959184
0.245319301959184
0.245319301959184
0.245319301959184
0.245319301959184
0.245319301959184
0.245319301959184
0.245319301959184
0.245319301959184
0.245319301959184
0.245319301959184
0.245319301959184

0.245319301959184
0.245319301959184
0.245319301959184
0.245319301959184
0.245319301959184
0.245319301959184
0.245319301959184
0.245319301959184
0.245319301959184
0.245319301959184
0.245319301959184
0.245319301959184
0.245319301959184
0.245319301959184
0.245319301959184
0.245319301959184
0.245319301959184
0.245319301959184
0.245319301959184
0.245319301959184
0.245319301959184
0.245319301959184
0.245319301959184
0.245319301959184
0.245319301959184
0.245319301959184
0.245319301959184
0.245319301959184
0.245319301959184
0.245319301959184
0.245319301959184
0.245319301959184
0.245319301959184
0.245319301959184
0.245319301959184
0.245319301959184
0.245319301959184
0.245319301959184

0.245319301959184
0.245319301959184
0.245319301959184
0.245319301959184
0.245319301959184
0.245319301959184
0.245319301959184
0.245319301959184
0.245319301959184
0.245319301959184
0.245319301959184
0.245319301959184
0.245319301959184
0.245319301959184
0.245319301959184
0.245319301959184
0.245319301959184
0.245319301959184
0.245319301959184
0.245319301959184
0.245319301959184
0.245319301959184
0.245319301959184
0.245319301959184
0.245319301959184
0.245319301959184
0.245319301959184
0.245319301959184
0.245319301959184
0.245319301959184
0.245319301959184
0.245319301959184
0.245319301959184
0.245319301959184
0.245319301959184
0.245319301959184
0.245319301959184
0.245319301959184

0.245319301959184
0.245319301959184
0.245319301959184
0.245319301959184
0.245319301959184
0.245319301959184
0.245319301959184
0.245319301959184
0.245319301959184
0.245319301959184
0.245319301959184
0.245319301959184
0.245319301959184
0.245319301959184
0.245319301959184
0.245319301959184
0.245319301959184
0.245319301959184
0.245319301959184
0.245319301959184
0.245319301959184
0.245319301959184
0.245319301959184
0.245319301959184
0.245319301959184
0.245319301959184
0.245319301959184
0.245319301959184
0.245319301959184
0.245319301959184
0.245319301959184
0.245319301959184
0.245319301959184
0.245319301959184
0.245319301959184
0.245319301959184
0.245319301959184
0.245319301959184

0.245319301959184
0.245319301959184
0.245319301959184
0.245319301959184
0.245319301959184
0.245319301959184
0.245319301959184
0.245319301959184
0.245319301959184
0.245319301959184
0.245319301959184
0.245319301959184
0.245319301959184
0.245319301959184
0.245319301959184
0.245319301959184
0.245319301959184
0.245319301959184
0.245319301959184
0.245319301959184
0.245319301959184
0.245319301959184
0.245319301959184
0.245319301959184
0.245319301959184
0.245319301959184
0.245319301959184
0.245319301959184
0.245319301959184
0.245319301959184
0.245319301959184
0.245319301959184
0.245319301959184
0.245319301959184
0.245319301959184
0.245319301959184
0.245319301959184
0.245319301959184

0.245319301959184
0.245319301959184
0.245319301959184
0.245319301959184
0.245319301959184
0.245319301959184
0.245319301959184
0.245319301959184
0.245319301959184
0.245319301959184
0.245319301959184
0.245319301959184
0.245319301959184
0.245319301959184
0.245319301959184
0.245319301959184
0.245319301959184
0.245319301959184
0.245319301959184
0.245319301959184
0.245319301959184
0.245319301959184
0.245319301959184
0.245319301959184
0.245319301959184
0.245319301959184
0.245319301959184
0.245319301959184
0.245319301959184
0.245319301959184
0.245319301959184
0.245319301959184
0.245319301959184
0.245319301959184
0.245319301959184
0.245319301959184
0.245319301959184
0.245319301959184

0.245319301959184
0.245319301959184
0.245319301959184
0.245319301959184
0.245319301959184
0.245319301959184
0.245319301959184
0.245319301959184
0.245319301959184
0.245319301959184
0.245319301959184
0.245319301959184
0.245319301959184
0.245319301959184
0.245319301959184
0.245480834306773
0.245480834306773
0.245480834306773
0.245480834306773
0.245480834306773
0.245480834306773
0.245480834306773
0.245480834306773
0.245480834306773
0.245480834306773
0.245480834306773
0.245480834306773
0.245480834306773
0.245480834306773
0.245480834306773
0.245480834306773
0.245480834306773
0.245480834306773
0.245480834306773
0.245480834306773
0.245480834306773
0.245480834306773
0.245480834306773

0.245480834306773
0.245480834306773
0.245480834306773
0.245480834306773
0.245480834306773
0.245480834306773
0.245480834306773
0.245480834306773
0.245480834306773
0.245480834306773
0.245480834306773
0.245480834306773
0.245480834306773
0.245480834306773
0.245480834306773
0.245480834306773
0.245480834306773
0.245480834306773
0.245480834306773
0.246133604208739
0.246215694084624
0.246215694084624
0.246215694084624
0.246215694084624
0.246283123673888
0.246283123673888
0.246552040996134
0.246552040996134
0.246552040996134
0.246552040996134
0.246552040996134
0.246552040996134
0.246820002079426
0.246823897812369
0.246823897812369
0.247801104243497
0.247801104243497
0.247801104243497

0.248404082511138
0.24912861278506
0.24912861278506
0.24912861278506
0.24912861278506
0.24912861278506
0.24912861278506
0.24912861278506
0.24912861278506
0.24912861278506
0.24912861278506
0.24912861278506
0.24912861278506
0.24912861278506
0.24912861278506
0.249321479236736
0.249321479236736
0.249341330761658
0.249346182223109
0.249346182223109
0.249551503227764
0.249551503227764
0.251036595412505
0.251050048396958
0.251050048396958
0.251050048396958
0.251050048396958
0.251050048396958
0.251050048396958
0.251099039586811
0.251622791761373
0.251622791761373
0.251622791761373
0.251622791761373
0.251622791761373
0.251622791761373
0.252148044842618
0.252148044842618

0.252148044842618
0.252148044842618
0.252148044842618
0.252148044842618
0.252148044842618
0.252148044842618
0.252148044842618
0.252148044842618
0.252148044842618
0.252148044842618
0.252148044842618
0.252148044842618
0.252148044842618
0.252148044842618
0.252148044842618
0.252148044842618
0.252148044842618
0.252148044842618
0.252148044842618
0.252148044842618
0.252148044842618
0.252148044842618
0.252424554892077
0.252424554892077
0.252424554892077
0.252424554892077
0.252424554892077
0.252424554892077
0.252424554892077
0.25539633533893
0.255890554472711
0.256496030694275
0.256496030694275
0.257093485837039
0.257093485837039
0.257093485837039
0.257093485837039
0.257093485837039

0.257093485837039
0.257093485837039
0.257093485837039
0.257093485837039
0.257093485837039
0.257093485837039
0.257093485837039
0.257093485837039
0.257093485837039
0.257093485837039
0.257093485837039
0.257093485837039
0.257093485837039
0.257093485837039
0.257093485837039
0.257093485837039
0.257093485837039
0.257093485837039
0.257093485837039
0.257093485837039
0.257093485837039
0.257093485837039
0.257093485837039
0.257093485837039
0.257093485837039
0.257093485837039
0.257093485837039
0.257093485837039
0.257093485837039
0.257093485837039
0.258074707750582
0.258074707750582
0.258074707750582
0.258074707750582
0.258337899910533
0.258415666951312
0.259182476503952
0.259182476503952

0.2597168029374
0.261688542737658
0.261688542737658
0.261688542737658
0.261917243796065
0.261917243796065
0.262742562468067
0.262742562468067
0.263174145489459
0.263174145489459
0.263218230044235
0.263419824981252
0.263419824981252
0.263419824981252
0.263505099297064
0.263609022895029
0.263609022895029
0.263609022895029
0.263836407882572
0.2642174202545
0.2642174202545
0.2642174202545
0.264904026522573
0.26521478763145
0.26563962287676
0.265958196036535
0.267143298206906
0.267143298206906
0.267324670747481
0.267795179882252
0.268089312178766
0.268089312178766
0.268089312178766
0.268089312178766
0.268089312178766
0.268089312178766
0.268089312178766
0.268089312178766

0.268202435230917
0.268202435230917
0.268202435230917
0.268202435230917
0.268202435230917
0.268202435230917
0.268202435230917
0.268202435230917
0.268202435230917
0.268202435230917
0.268202435230917
0.268202435230917
0.268202435230917
0.268202435230917
0.268202435230917
0.268202435230917
0.268202435230917
0.268202435230917
0.268202435230917
0.268202435230917
0.268202435230917
0.268202435230917
0.268202435230917
0.268202435230917
0.268202435230917
0.268202435230917
0.268202435230917
0.268280549952887
0.268280549952887
0.268280549952887
0.268280549952887
0.268381677821454
0.268381677821454
0.268381677821454
0.268546990179733
0.268546990179733
0.268546990179733
0.268546990179733

0.268715515482884
0.268715515482884
0.268758093539904
0.268758093539904
0.268758093539904
0.268758093539904
0.268758093539904
0.268758093539904
0.268758093539904
0.268942126546091
0.268942126546091
0.268942126546091
0.269595056526455
0.269595056526455
0.269595056526455
0.269595056526455
0.269595056526455
0.269595056526455
0.269595056526455
0.269595056526455
0.269664315187513
0.269664315187513
0.269664315187513
0.269664315187513
0.269836336859512
0.269836336859512
0.269836336859512
0.269836336859512
0.269836336859512
0.269836336859512
0.27009950613941
0.27009950613941
0.27009950613941
0.27009950613941
0.27009950613941
0.27009950613941
0.27009950613941
0.271391982579087

0.271391982579087
0.271391982579087
0.271391982579087
0.271391982579087
0.271391982579087
0.271391982579087
0.271391982579087
0.271391982579087
0.272337137607218
0.272343413578437
0.273387577264087
0.273520818414861
0.273520818414861
0.273520818414861
0.273520818414861
0.273520818414861
0.27360640021238
0.27360640021238
0.273825309787048
0.274034664957262
0.274095737344156
0.274095737344156
0.275008463530373
0.27519470609182
0.27519470609182
0.27519470609182
0.27519470609182
0.27519470609182
0.27519470609182
0.27519470609182
0.27519470609182
0.27519470609182
0.27519470609182
0.27519470609182
0.275296179325607
0.275296179325607
0.275296179325607
0.275296179325607

0.275296179325607
0.275296179325607
0.275421832037819
0.275421832037819
0.275421832037819
0.275421832037819
0.275421832037819
0.275421832037819
0.275421832037819
0.275421832037819
0.275421832037819
0.275421832037819
0.275421832037819
0.275421832037819
0.275421832037819
0.275518725189895
0.276234194839515
0.276234194839515
0.276234194839515
0.276493980467965
0.276617370557043
0.277016978795205
0.277198815854815
0.277198815854815
0.277198815854815
0.277198815854815
0.277198815854815
0.277198815854815
0.277198815854815
0.277198815854815
0.277198815854815
0.277198815854815
0.277198815854815
0.277198815854815
0.277198815854815
0.277198815854815
0.277198815854815
0.277198815854815

0.277198815854815
0.277198815854815
0.277198815854815
0.277198815854815
0.280167840428559
0.280201395539125
0.280201395539125
0.280201395539125
0.280275481657944
0.280429356647908
0.282844726676272
0.282844726676272
0.282844726676272
0.283362621838819
0.283395495348471
0.283395495348471
0.283395495348471
0.283624976641737
0.283624976641737
0.283794115140821
0.284390732636798
0.284698782722956
0.284698782722956
0.284698782722956
0.285056494283798
0.285121994839076
0.285121994839076
0.285694305543403
0.285694305543403
0.285694305543403
0.285694305543403
0.286609731314499
0.286609731314499
0.286618724285269
0.286827453289195
0.288264774671866
0.288264774671866
0.288264774671866

0.288427786841848
0.288733401719717
0.288733401719717
0.288733401719717
0.288733401719717
0.288733401719717
0.289258039869996
0.289258039869996
0.289915255123001
0.289915255123001
0.289915255123001
0.289915255123001
0.290646449325815
0.290646449325815
0.290646449325815
0.290646449325815
0.290646449325815
0.290646449325815
0.290646449325815
0.290646449325815
0.290646449325815
0.290646449325815
0.291200175029572
0.291200175029572
0.291200175029572
0.291200175029572
0.291200175029572
0.291200175029572
0.291200175029572
0.291200175029572
0.291200175029572
0.291200175029572
0.291200175029572
0.291200175029572
0.291200175029572
0.291200175029572
0.291200175029572
0.291200175029572

0.291200175029572
0.291200175029572
0.291200175029572
0.291200175029572
0.291200175029572
0.291200175029572
0.291200175029572
0.291200175029572
0.291200175029572
0.291200175029572
0.291200175029572
0.291200175029572
0.291200175029572
0.291502499661032
0.291502499661032
0.291502499661032
0.291502499661032
0.291502499661032
0.291502499661032
0.291502499661032
0.291516372514899
0.291516372514899
0.291897438904913
0.291897438904913
0.291897438904913
0.291897438904913
0.291897438904913
0.291897438904913
0.291897438904913
0.291897438904913
0.291976697403884
0.291976697403884
0.291976697403884
0.291976697403884
0.291976697403884
0.292612293335159
0.292612293335159
0.292612293335159

0.292612293335159
0.292612293335159
0.292612293335159
0.292612293335159
0.292691998700846
0.292691998700846
0.292752263387326
0.292912658565824
0.293429704424137
0.293758079590503
0.293758079590503
0.293758079590503
0.29391089986884
0.293925871996071
0.294806610610908
0.294806610610908
0.294806610610908
0.294806610610908
0.295005795261296
0.295005795261296
0.295005795261296
0.295005795261296
0.295294577232242
0.295294577232242
0.295294577232242
0.295294577232242
0.295294577232242
0.295512612128986
0.295512612128986
0.295512612128986
0.295512612128986
0.295512612128986
0.295512612128986
0.295512612128986
0.295512612128986
0.295512612128986
0.295522365996485
0.295522365996485

0.295687754371202
0.296474290224991
0.296817245346114
0.296860324762954
0.296916160952944
0.296916160952944
0.296916160952944
0.296916160952944
0.296916160952944
0.296916160952944
0.296916160952944
0.296916160952944
0.296916160952944
0.296916160952944
0.296916160952944
0.296916160952944
0.296916160952944
0.296916160952944
0.296916160952944
0.296916160952944
0.296916160952944
0.296916160952944
0.296950158361955
0.296986745096936
0.29711149854946
0.29711149854946
0.29711149854946
0.29711149854946
0.29711149854946
0.297284969611458
0.298858310374075
0.298858310374075
0.298858310374075
0.29998372120422
0.301314672637359
0.301767174349732
0.301954530855504
0.301954530855504

0.302147196978411
0.303122443027868
0.303749903571097
0.303881300212471
0.304168906888136
0.304584310980157
0.304791653688998
0.305543540141759
0.305543540141759
0.305746571275453
0.306329802852952
0.306329802852952
0.308021700473343
0.308224535462083
0.308547046004706
0.308547046004706
0.309658887848332
0.30982768645557
0.30982768645557
0.30982768645557
0.30982768645557
0.30982768645557
0.30982768645557
0.30982768645557
0.30982768645557
0.30982768645557
0.310976394364033
0.311234708323343
0.312070196526681
0.312382973833102
0.313272826706189
0.313395171222382
0.313395171222382
0.313603873914818
0.313603873914818
0.313745361010003
0.313745361010003
0.313948299408683

0.313948299408683
0.313976079443372
0.313976079443372
0.315520698552711
0.315520698552711
0.315520698552711
0.315520698552711
0.315520698552711
0.315520698552711
0.31643300350757
0.316868704162148
0.316868704162148
0.316868704162148
0.316868704162148
0.316868704162148
0.316868704162148
0.317154322764224
0.318642869533444
0.318642869533444
0.318706341667141
0.319959222348747
0.320976364464781
0.321008816331873
0.321309073410226
0.321309073410226
0.321309073410226
0.321309073410226
0.321309073410226
0.321309073410226
0.321309073410226
0.321309073410226
0.321362454021703
0.322182259530803
0.322182259530803
0.322247230404418
0.322247230404418
0.322247230404418
0.32225469615484

0.32225469615484
0.32225469615484
0.32225469615484
0.32225469615484
0.32225469615484
0.32225469615484
0.32225469615484
0.32225469615484
0.32225469615484
0.32225469615484
0.32225469615484
0.32225469615484
0.32225469615484
0.32225469615484
0.324168375515591
0.324168375515591
0.324168375515591
0.324169175965829
0.324169175965829
0.324169175965829
0.324169175965829
0.3246320235078
0.3246320235078
0.324866365763741
0.325765588662782
0.325765588662782
0.325765588662782
0.325765588662782
0.325765588662782
0.325765588662782
0.325765588662782
0.325765588662782
0.325765588662782
0.325765588662782
0.325765588662782
0.325765588662782
0.325765588662782
0.325765588662782

0.325765588662782
0.325765588662782
0.325765588662782
0.325765588662782
0.325765588662782
0.325765588662782
0.325944878841483
0.325944878841483
0.326052698304233
0.326400440165032
0.326400440165032
0.326400440165032
0.326400440165032
0.326723089524542
0.326723089524542
0.326723089524542
0.326816587116149
0.326980455508803
0.326980455508803
0.327247681211024
0.327939575466642
0.327939575466642
0.327939575466642
0.327939575466642
0.327949914866801
0.327949914866801
0.327949914866801
0.328105349523445
0.328105349523445
0.328211064997557
0.328211064997557
0.32911315394964
0.329523295690567
0.329718036143818
0.329861264717616
0.329861264717616
0.329901847616482
0.329901847616482

0.329901847616482
0.329901847616482
0.330091255256831
0.331370537745009
0.331692611043222
0.331692611043222
0.331692611043222
0.332165542664903
0.332165542664903
0.332165542664903
0.332165542664903
0.332165542664903
0.332538966102677
0.332840032900229
0.333428905633505
0.333428905633505
0.334172131700754
0.334343912704333
0.334547818740924
0.334763507855714
0.335013148248389
0.335056066984046
0.335056066984046
0.335378512623884
0.336784586295011
0.336814702237718
0.336814702237718
0.337567416575813
0.337733848969779
0.338008041629038
0.338008041629038
0.338008041629038
0.33801015616207
0.338051364748806
0.33829367220642
0.33829367220642
0.33829367220642
0.33829367220642

0.338503496821661
0.338709407136574
0.338709407136574
0.338709407136574
0.338821461917919
0.33900446583217
0.339692821442136
0.339692821442136
0.339977092219705
0.339977092219705
0.339977092219705
0.340493945108138
0.340758917815026
0.340758917815026
0.341065394702004
0.34136701194164
0.34136701194164
0.34136701194164
0.341623150417971
0.342268021510386
0.344122643351633
0.344510694203844
0.345400595421884
0.345519050977959
0.345542622357943
0.345542622357943
0.345542622357943
0.345674195458339
0.345909154072178
0.34658081560956
0.346868820547271
0.346868820547271
0.347121157310247
0.347121157310247
0.347121157310247
0.347261695343265
0.347261695343265
0.347261695343265

0.347261695343265
0.347261695343265
0.348056838728392
0.348056838728392
0.348056838728392
0.348799759002842
0.348985086657335
0.348985086657335
0.348985086657335
0.348985086657335
0.348985086657335
0.348985086657335
0.348985086657335
0.348985086657335
0.348985086657335
0.348985086657335
0.348985086657335
0.348985086657335
0.348985086657335
0.348985086657335
0.348985086657335
0.348985086657335
0.348985086657335
0.348985086657335
0.348985086657335
0.348985086657335
0.348985086657335
0.348985086657335
0.348985086657335
0.348985086657335
0.348985086657335
0.349299734707593
0.349299734707593
0.349311952639468
0.349311952639468
0.349311952639468
0.349311952639468
0.349311952639468

0.349411061948335
0.349411061948335
0.349411061948335
0.349411061948335
0.349411061948335
0.349411061948335
0.349411061948335
0.349411061948335
0.349411061948335
0.349411061948335
0.349411061948335
0.349411061948335
0.349411061948335
0.349513496242982
0.349558213817882
0.350410125424417
0.350410125424417
0.350410125424417
0.350410125424417
0.350433250545278
0.350433250545278
0.350433250545278
0.350433250545278
0.351063246318479
0.351162420480758
0.352669477067354
0.352701073512282
0.352701073512282
0.352701073512282
0.352701073512282
0.352987262919578
0.352987262919578
0.352987262919578
0.353044274865894
0.353044274865894
0.353044274865894
0.353332172608091
0.353549665096602

0.353549665096602
0.353549665096602
0.353549665096602
0.353549665096602
0.353633229396023
0.353914678526592
0.353914678526592
0.353914678526592
0.354284628906186
0.35475498857571
0.35475498857571
0.354983698440123
0.354983698440123
0.354983698440123
0.354983698440123
0.354983698440123
0.355052595569498
0.355081031016799
0.355089842202499
0.355089842202499
0.355673192254321
0.355702846685617
0.355702846685617
0.355702846685617
0.356232735661569
0.35682652012362
0.35682652012362
0.357506190581135
0.359821334990301
0.360075156473464
0.360087911911972
0.361226233506798
0.361226233506798
0.361226233506798
0.361226233506798
0.361316241839568
0.361363314317049
0.361363314317049

0.361363314317049
0.361363314317049
0.361729428811602
0.361794328401133
0.361808555717573
0.361808555717573
0.361808555717573
0.361808555717573
0.362116178939565
0.362116178939565
0.362116178939565
0.362176965251809
0.362176965251809
0.362475321524492
0.363122463827535
0.36357385691307
0.363622698753785
0.363622698753785
0.364167152928753
0.364189435697662
0.364448980631351
0.364448980631351
0.36487136056798
0.365303072971502
0.36555020519286
0.365699164935598
0.366059338216073
0.366309538008712
0.366922295381202
0.366922295381202
0.366922295381202
0.367374731865153
0.367374731865153
0.367657841822176
0.367994004855529
0.368716199850115
0.368716199850115
0.368880410420423

0.368880410420423
0.368919540141541
0.368976279133646
0.368976279133646
0.369942768826464
0.369942768826464
0.369942768826464
0.369998271541679
0.370599293094086
0.37074995314159
0.37414238699118
0.37414238699118
0.374232259975407
0.374739541061552
0.374796508695524
0.375432708251945
0.375432708251945
0.375432708251945
0.375432708251945
0.375594505969172
0.375594505969172
0.375849750802488
0.375868110841695
0.375868110841695
0.375992463985779
0.375992463985779
0.375992463985779
0.376755743470083
0.376823491112222
0.37756265269024
0.378512099091976
0.378512099091976
0.378512099091976
0.378512099091976
0.378512099091976
0.378512099091976
0.378512099091976
0.378512099091976

0.378757665243658
0.378757665243658
0.378757665243658
0.379540879289873
0.379540879289873
0.37971349252604
0.37971349252604
0.37971349252604
0.380311801205266
0.380311801205266
0.38220148042454
0.382232904251041
0.382232904251041
0.382232904251041
0.382232904251041
0.382794895684983
0.382794895684983
0.382801684995659
0.382941500022669
0.383017683039038
0.383017683039038
0.383017683039038
0.383017683039038
0.383017683039038
0.383017683039038
0.383017683039038
0.383017683039038
0.383039744663124
0.383039744663124
0.383643455107257
0.383643455107257
0.383954585597594
0.384062976104279
0.385741447711964
0.385741447711964
0.385741447711964
0.387008041427086
0.387008041427086

0.387008041427086
0.387649717745932
0.38785593743502
0.388775740099746
0.390133123704276
0.391118058267278
0.392186230968184
0.392431329654644
0.39438882186812
0.394883983930758
0.395372473226127
0.396867266790474
0.397445994957832
0.397445994957832
0.39837481983202
0.39837481983202
0.398390368835827
0.398627505201439
0.398918026107154
0.401017575978515
0.401166878793082
0.402208982282677
0.402960239017429
0.403767174445943
0.404591879832311
0.404737242402359
0.404906303031914
0.404906303031914
0.404906303031914
0.404906303031914
0.404906303031914
0.405257256610826
0.405257256610826
0.406220140397891
0.407005652206499
0.407064487071044
0.407064487071044
0.407064487071044

0.407064487071044
0.407722642054583
0.407722642054583
0.407722642054583
0.407722642054583
0.408262687528747
0.408411156977579
0.408411156977579
0.408411156977579
0.408411156977579
0.408411156977579
0.408411156977579
0.408411156977579
0.408411156977579
0.408411156977579
0.408411156977579
0.408411156977579
0.408411156977579
0.408411156977579
0.408411156977579
0.408411156977579
0.408411156977579
0.408411156977579
0.408411156977579
0.408411156977579
0.408411156977579
0.408411156977579
0.408411156977579
0.408411156977579
0.408726925266995
0.408733191360631
0.411131397422773
0.41144422060145
0.411669976889327
0.411669976889327
0.411669976889327
0.412730672436617
0.412990423967494

0.412990423967494
0.414351504954497
0.414648817475638
0.414648817475638
0.415237158509024
0.41791445154717
0.41791445154717
0.417933985677433
0.418387841345239
0.418387841345239
0.419275618726308
0.419527001595994
0.419527001595994
0.419527001595994
0.420173188882703
0.420173188882703
0.420173188882703
0.420173188882703
0.420173188882703
0.420550929386656
0.421450917821766
0.422355657860631
0.423612116012194
0.424049596186413
0.424136976756389
0.424136976756389
0.424605584530824
0.424655822677736
0.424655822677736
0.425201853981314
0.425433009945995
0.425754423528797
0.425754423528797
0.425754423528797
0.425754423528797
0.426506921378464
0.426506921378464
0.426506921378464

0.427048032875509
0.427048032875509
0.427048032875509
0.427440254699643
0.427680842865695
0.427680842865695
0.427867654946421
0.428013225631154
0.428013225631154
0.428013225631154
0.428013225631154
0.428013225631154
0.428013225631154
0.428013225631154
0.428013225631154
0.428130202975926
0.428130202975926
0.428130202975926
0.428130202975926
0.428889986639672
0.428889986639672
0.428889986639672
0.428889986639672
0.428889986639672
0.428889986639672
0.428889986639672
0.428889986639672
0.428889986639672
0.428889986639672
0.428889986639672
0.428889986639672
0.428889986639672
0.428889986639672
0.428889986639672
0.428889986639672
0.428889986639672
0.428889986639672
0.428889986639672

0.428889986639672
0.428889986639672
0.428889986639672
0.429145396554036
0.429452353620781
0.429452353620781
0.429552629854906
0.429552629854906
0.429552629854906
0.429574462458906
0.430194700188919
0.430231701496242
0.431839282826461
0.432982621596518
0.433804811975134
0.433804811975134
0.433897507400575
0.433897507400575
0.434435527539989
0.434771119890875
0.435203430192251
0.436043189922594
0.436405618322985
0.437093795631602
0.437093795631602
0.437093795631602
0.437093795631602
0.438980772266698
0.438980772266698
0.438980772266698
0.438980772266698
0.438980772266698
0.438980772266698
0.439169612219124
0.439169612219124
0.439169612219124
0.439169612219124
0.439169612219124

0.439182508962215
0.440074280304277
0.440678422901271
0.442778612653725
0.443526295321348
0.443952486640307
0.443952486640307
0.443952486640307
0.443952486640307
0.444303509561898
0.444303509561898
0.44452038072792
0.44452038072792
0.44452038072792
0.444641287501661
0.444881792762112
0.444959073537392
0.445134162779902
0.445134162779902
0.445134162779902
0.445989082087742
0.445989082087742
0.445989082087742
0.446975118597075
0.447116130678085
0.447503404868035
0.448343274207306
0.448817920968665
0.448817920968665
0.448901722564336
0.448935226072711
0.448935226072711
0.448935226072711
0.448935226072711
0.448935226072711
0.448935226072711
0.448935226072711
0.448935226072711

0.448935226072711
0.448935226072711
0.448935226072711
0.448935226072711
0.448935226072711
0.448935226072711
0.448935226072711
0.449507439671674
0.449507439671674
0.449956695723079
0.450872347713133
0.451388077645253
0.451429192927704
0.451480157529961
0.451793009661046
0.452297646359855
0.452297646359855
0.452466248008758
0.452466248008758
0.453258200469396
0.453535588495074
0.455220150075755
0.455917215786672
0.456580343666311
0.45721759860581
0.457387307697071
0.457571507578404
0.457624470602878
0.457624470602878
0.457906466659584
0.458213468579072
0.458213468579072
0.458213468579072
0.458396976686095
0.458970440024955
0.459147495425048
0.459147495425048
0.459147495425048

0.459285153093176
0.459487877287954
0.459535265697554
0.459984891669877
0.460694091956591
0.461055755585198
0.461055755585198
0.461404116235245
0.461747665681232
0.461747665681232
0.461889866586127
0.462372684274718
0.463424554987094
0.463430487693823
0.463443549130092
0.463443549130092
0.463539996582578
0.463539996582578
0.463628197405947
0.463628197405947
0.463628197405947
0.464037130413531
0.464037130413531
0.464394682564793
0.464394682564793
0.464394682564793
0.464394682564793
0.464394682564793
0.464394682564793
0.464394682564793
0.464394682564793
0.465414069282181
0.465519955744623
0.466290135862202
0.466902981116134
0.466982461031211
0.466982461031211
0.467215203732058

0.469289570322897
0.469784267933517
0.469784267933517
0.469985147221686
0.470945254263219
0.470945254263219
0.470945254263219
0.471113321026228
0.471292744958437
0.471338611709484
0.471338611709484
0.471338611709484
0.471338611709484
0.471338611709484
0.471338611709484
0.471338611709484
0.471449508382824
0.471449508382824
0.472801354727994
0.473595064119815
0.473595064119815
0.473686054743413
0.473780628461156
0.473780628461156
0.474035714927777
0.474035714927777
0.474035714927777
0.474141666896801
0.474909885659099
0.474909885659099
0.475121025454737
0.4753079178783
0.475664121879151
0.475710327000323
0.476938910120332
0.476938910120332
0.476938910120332
0.478923998271338

0.478923998271338
0.478993723237795
0.478993723237795
0.479247587827613
0.479375255298827
0.479375255298827
0.479375255298827
0.479375255298827
0.479375255298827
0.479375255298827
0.479375255298827
0.480279006162558
0.481575676437231
0.482165130156082
0.482165130156082
0.482165130156082
0.482187096931959
0.485657480211905
0.485657480211905
0.485891426481791
0.486335784389272
0.486421902108545
0.486421902108545
0.486597973268931
0.486597973268931
0.486597973268931
0.486597973268931
0.486597973268931
0.486597973268931
0.486746304418083
0.48714364414479
0.487490112737488
0.487506247230824
0.487506247230824
0.487758583014002
0.487849498418783
0.488934118290853
0.489112592973987

0.489112592973987
0.489243049944544
0.489673378720006
0.490560639957449
0.491079939429062
0.491921991184614
0.492461038087133
0.492461038087133
0.492461038087133
0.492665338626599
0.492665338626599
0.492665338626599
0.492665338626599
0.493659471827814
0.49366068921228
0.49366068921228
0.49366068921228
0.494150238978215
0.494150238978215
0.494377841393136
0.494377841393136
0.494377841393136
0.49459467586943
0.495009859745523
0.49541661032118
0.496095639725486
0.496095639725486
0.496095639725486
0.496139587465582
0.496400712152448
0.496400712152448
0.496400712152448
0.496653313532019
0.497556163454181
0.497556163454181
0.497556163454181
0.498523121015927
0.499150091997107

0.499150091997107
0.499448364310036
0.499448364310036
0.500340976840238
0.500340976840238
0.501505627459116
0.501625594527751
0.501625594527751
0.501692085071856
0.501950445713142
0.50205008653754
0.50205008653754
0.50205008653754
0.50205008653754
0.50205008653754
0.502124180530444
0.502166509133231
0.502474903025616
0.502639606522073
0.502639606522073
0.502639606522073
0.502639606522073
0.502639606522073
0.506132973109265
0.506132973109265
0.508373174909828
0.510299872433362
0.510299872433362
0.510437261654203
0.510989131405296
0.511449672473203
0.512741421705301
0.513538512761742
0.513538512761742
0.513538512761742
0.51470516014678
0.515270387472615
0.515719268836499

0.51589442979387
0.515903936499529
0.516081969014497
0.516996202661027
0.51709335941102
0.51709335941102
0.51709335941102
0.51709335941102
0.517568191149962
0.518163051749463
0.518955454502837
0.51952164988922
0.51952164988922
0.519796148113377
0.520008559053593
0.521835611430993
0.521983239634397
0.521983239634397
0.522181193152349
0.522243650343539
0.52277775765169
0.52277775765169
0.523508647184967
0.523937334986126
0.524219888245713
0.524404884627702
0.525448332936909
0.52550263825673
0.525592566448025
0.525967091893577
0.526225033371536
0.526533964288344
0.526533964288344
0.526554760333399
0.527253471248215
0.527452083235385
0.527981205385804
0.528984292445088

0.529433726215405
0.530166475599221
0.530166475599221
0.530166475599221
0.530166475599221
0.53042966286631
0.53042966286631
0.53042966286631
0.53042966286631
0.53042966286631
0.53042966286631
0.531505551494964
0.531526916070093
0.531526916070093
0.531526916070093
0.531526916070093
0.531526916070093
0.531526916070093
0.53193587685678
0.53193587685678
0.53193587685678
0.53193587685678
0.53193587685678
0.533158253534493
0.534090382393323
0.534135624612102
0.534173700032854
0.534173700032854
0.534777887543914
0.535443654032136
0.535443654032136
0.535617028466291
0.536314995865861
0.53656158946758
0.537501682464955
0.537772996308178
0.537772996308178
0.537772996308178

0.538051611201427
0.53872586769745
0.53872586769745
0.539189874255473
0.539189874255473
0.541098483851171
0.541098483851171
0.541098483851171
0.541488782626852
0.542735763791051
0.542735763791051
0.542955519771909
0.542963101985809
0.542963101985809
0.543514313543808
0.543514313543808
0.543766238786281
0.544097710797428
0.545629170199559
0.545629170199559
0.546209010639401
0.546407314216652
0.546809397221507
0.547278922058029
0.547278922058029
0.547802581445923
0.547998426445764
0.549095029429724
0.549095029429724
0.549095029429724
0.549095029429724
0.549095029429724
0.550328552895507
0.551548923858805
0.551548923858805
0.55179941319773
0.552147198008318
0.552403955641823

0.552403955641823
0.552403955641823
0.552880755105022
0.553775196841461
0.553871625643733
0.554138898604038
0.555653414600563
0.555653414600563
0.555653414600563
0.555653414600563
0.555653414600563
0.5560125436136
0.556038160006005
0.55618335185931
0.556788400464209
0.556788400464209
0.557639662327378
0.557639662327378
0.560687121481365
0.560720186623132
0.561048364408096
0.561048364408096
0.561048364408096
0.561048364408096
0.561048364408096
0.561256136143223
0.561256136143223
0.561736417233988
0.562920859255966
0.562920859255966
0.562920859255966
0.562920859255966
0.562920859255966
0.562920859255966
0.562920859255966
0.562920859255966
0.562920859255966
0.562920859255966

0.562920859255966
0.562990672533099
0.563100585646584
0.56465369897704
0.566971981583142
0.567100587590448
0.567438306469865
0.567438306469865
0.567438306469865
0.567438306469865
0.568357321931318
0.570878542224308
0.57157121
0.571903876189761
0.573106378951896
0.573584939253223
0.57556687247829
0.576418521163541
0.576418521163541
0.576629626095161
0.57725845812551
0.57725845812551
0.577949965890194
0.578797070806325
0.578886165499834
0.578886165499834
0.579297050624057
0.579327810201426
0.579327810201426
0.580162102275168
0.580334844724967
0.580856327999773
0.581444231401332
0.582113791450396
0.582113791450396
0.582113791450396
0.582125313962857
0.582573150429987

0.582573150429987
0.582865924281819
0.585176465804502
0.585373560899579
0.585796007345069
0.586207603876714
0.586207603876714
0.586998076612512
0.587157927129161
0.587157927129161
0.587157927129161
0.587157927129161
0.587567505193156
0.587660735802837
0.588080944925136
0.588080944925136
0.588080944925136
0.588080944925136
0.588080944925136
0.588080944925136
0.588306526705494
0.588306526705494
0.588306526705494
0.588315637652905
0.590309357068284
0.590309357068284
0.590449499572411
0.59128054846874
0.593119141648294
0.593124944992681
0.593742325758398
0.595037707127372
0.595746987006402
0.595746987006402
0.596049185739969
0.596539320635188
0.59906592081649
0.59906592081649

0.599572561886738
0.599791677491344
0.599942394628867
0.599942394628867
0.600149691157815
0.600800368701754
0.601397258873344
0.601397258873344
0.601719708021661
0.601719708021661
0.602234074461752
0.603632010273166
0.603981029728816
0.603981029728816
0.603981029728816
0.604947600977226
0.604972901797018
0.605161813394285
0.605416426875359
0.605416426875359
0.605416426875359
0.605883945376842
0.606612940605047
0.606625710067462
0.606625710067462
0.606625710067462
0.607779995314549
0.607827580741763
0.607827580741763
0.608871243376186
0.608871243376186
0.608871243376186
0.608871243376186
0.608871243376186
0.608871243376186
0.608871243376186
0.608871243376186
0.609461647750933

0.609778111006539
0.61069623283888
0.61069623283888
0.610718257759743
0.611394007834078
0.611394007834078
0.612401427487099
0.612584162564185
0.612752109022618
0.612752109022618
0.613408450919464
0.613530031869864
0.613530031869864
0.614136999060547
0.614532594527539
0.615706352947822
0.615838362030602
0.616242550312135
0.616302421017136
0.616450029243416
0.620009114629903
0.620827583267599
0.620827583267599
0.620827583267599
0.621779765188315
0.622567422393249
0.622761043281307
0.623737702837708
0.623737702837708
0.623737702837708
0.624212199017476
0.624341521819705
0.624923362443996
0.625062687976763
0.625078734479037
0.625117509832437
0.625796558597379
0.628937200461755

0.629292659848999
0.629296273432243
0.629311783034891
0.629560877090322
0.631193906033189
0.631483850491804
0.631818634041388
0.632974543609779
0.633098982656322
0.633231487532367
0.634267621174936
0.634267621174936
0.635001740327634
0.637015508079017
0.637019626853548
0.637019626853548
0.637019626853548
0.637019626853548
0.637019626853548
0.637909548269568
0.638006638620303
0.639165476934687
0.640968206343477
0.642233807286946
0.643221198342127
0.645005989632085
0.645042726676975
0.645398740060566
0.645398740060566
0.646416964718872
0.646416964718872
0.646416964718872
0.646812284541671
0.647434660811626
0.647434660811626
0.6485025630297
0.6485025630297
0.64859296

0.649138462758385
0.649688079364916
0.650816071053015
0.653656855690718
0.654303990760687
0.655437623610585
0.655686392308221
0.655839819211903
0.655839819211903
0.655839819211903
0.655839819211903
0.656717108467412
0.656717108467412
0.657258830613125
0.657258830613125
0.657258830613125
0.657446380595807
0.657817597516715
0.658616048996043
0.658752790759718
0.659248336281522
0.659248336281522
0.659500362440911
0.659500362440911
0.659579103491003
0.661345469930517
0.661504508363268
0.662849250160719
0.663146559105263
0.663395008928288
0.663793530780581
0.663793530780581
0.663793530780581
0.663815740693684
0.663815740693684
0.663815740693684
0.665635444198886
0.665635444198886

0.665661738780302
0.665661738780302
0.666389692094754
0.666389692094754
0.667604299884556
0.668182335656106
0.669401677190122
0.669851331288579
0.669851331288579
0.669851331288579
0.669851331288579
0.670462774931665
0.670763935683645
0.672222609767585
0.672415657836444
0.672415657836444
0.673576910350276
0.674559291969102
0.675744860526524
0.677981484505936
0.679001942138496
0.679001942138496
0.679697509212503
0.680207685494088
0.680207685494088
0.680207685494088
0.680207685494088
0.680207685494088
0.680369333946171
0.680369333946171
0.680369333946171
0.681476641858415
0.681476641858415
0.681476641858415
0.681476641858415
0.681820588926848
0.683580198930058
0.68490284187575

0.686037735073412
0.687431001442077
0.690054861832255
0.690054861832255
0.690463072944468
0.690597774525363
0.690601621122775
0.690601621122775
0.690601621122775
0.691224769814728
0.691746128865247
0.691983598900254
0.692287506361509
0.692287506361509
0.693853317477085
0.693897514126639
0.694211354373811
0.694324774855598
0.694324774855598
0.694324774855598
0.697447268265163
0.697447268265163
0.697549754346542
0.697549754346542
0.697982165173435
0.699265128585047
0.699359900436618
0.700883885081123
0.702029605344931
0.703611334831253
0.703675388367654
0.703675388367654
0.703675388367654
0.703675388367654
0.70414968911827
0.70414968911827
0.704957754661457
0.705032600081086

0.705032600081086
0.705571821742123
0.706452187398961
0.706452187398961
0.707416239927637
0.708260617597905
0.708916526061274
0.708916526061274
0.709323432730295
0.709667858455609
0.710302954244752
0.710745869240504
0.712032746814338
0.712990119484391
0.713361493732447
0.716462453775174
0.716462453775174
0.716462453775174
0.72091104943982
0.722019568975314
0.722019568975314
0.722211895745821
0.72237468825413
0.722881186353353
0.724004864846833
0.724831946030558
0.724831946030558
0.725081403659103
0.725567966498307
0.727722046742132
0.728126266112142
0.728521150056922
0.728521150056922
0.728521150056922
0.728521150056922
0.728521150056922
0.728521150056922
0.728521150056922

0.728557386275722
0.728557386275722
0.729949635183663
0.7311146164084
0.734102193946348
0.734495629391095
0.73498608709439
0.73498608709439
0.73498608709439
0.73498608709439
0.735488207260478
0.735488207260478
0.735488207260478
0.735488207260478
0.735488207260478
0.735488207260478
0.736152382895228
0.736152382895228
0.736606367936655
0.73789421888707
0.73789421888707
0.738258972364088
0.740009576523976
0.740536377496286
0.740536377496286
0.741779740034838
0.741924448306129
0.741924448306129
0.741924448306129
0.744080774277615
0.744080774277615
0.744211372933922
0.744272992381259
0.747801787278586
0.747899861745458
0.747899861745458
0.747899861745458
0.747899861745458

0.749278284313798
0.749959092262975
0.749959092262975
0.750860462142617
0.751000165540373
0.751511489915494
0.752620206541434
0.753792419414088
0.753802950774885
0.754311755514416
0.754311755514416
0.754311755514416
0.754311755514416
0.754311755514416
0.754663660245362
0.754663660245362
0.755014524533062
0.756644858162982
0.756644858162982
0.756644858162982
0.756644858162982
0.757324157331134
0.757324157331134
0.757340644467993
0.758967807434653
0.759090257870495
0.759090257870495
0.759589285032446
0.759589285032446
0.759589285032446
0.762137778226614
0.762137778226614
0.762137778226614
0.762137778226614
0.762481814839717
0.763465677985245
0.763887804731832
0.766804199374902

0.766804199374902
0.767003674598237
0.767472572909859
0.767472572909859
0.76920505895965
0.76920505895965
0.769977148619656
0.770044581624617
0.770044581624617
0.771662627809238
0.773792394019062
0.774064719510053
0.775550302128501
0.776052705309763
0.776405976507183
0.776771362181148
0.778818500943136
0.779558066883839
0.779558066883839
0.780477875238534
0.780477875238534
0.781809305336206
0.783552765111592
0.783552765111592
0.783552765111592
0.784478765718999
0.784478765718999
0.784478765718999
0.784478765718999
0.78489825486159
0.786114051942725
0.786114051942725
0.788182186998724
0.788257997879233
0.790355448119384
0.792741246209374
0.793557505613513
0.793557505613513

0.793557505613513
0.793557505613513
0.793557505613513
0.793557505613513
0.795128631866317
0.795831292271294
0.795831292271294
0.795831292271294
0.795831292271294
0.7964259207295
0.7964259207295
0.79669321112217
0.79669321112217
0.797807095693931
0.798942537353744
0.802476472091951
0.802476472091951
0.802476472091951
0.80333709381259
0.803416554367921
0.803416554367921
0.803416554367921
0.803416554367921
0.803416554367921
0.803951987845432
0.804125983409087
0.805067288479187
0.805067288479187
0.805430695148476
0.807223883119514
0.807223883119514
0.807223883119514
0.807422773357322
0.809128674142437
0.809128674142437
0.809820515250846
0.813532966668376
0.813532966668376

0.814292800324288
0.816157562054726
0.816157562054726
0.816471788053162
0.816471788053162
0.816507940297407
0.817596025026054
0.818065105005037
0.821208483529474
0.823136368662451
0.823149326584083
0.823181951217361
0.823675893866028
0.825016766138004
0.828609152911147
0.830519188076059
0.830865827314528
0.831808029586796
0.831993474528396
0.832169302500725
0.832825658136741
0.83309449296488
0.834205906772619
0.834375155585961
0.834375155585961
0.834967218800562
0.835284188053689
0.835284188053689
0.835807789432157
0.836035690639313
0.840532668437822
0.840883193180827
0.841148157542445
0.841179588299677
0.841179588299677
0.841995040603765
0.846193341622192
0.84647708689059

0.84664007663743
0.846796277749458
0.847583468301026
0.849686428899314
0.850217166151778
0.85046512422438
0.850797783037128
0.852036491063609
0.852036491063609
0.852036491063609
0.852036491063609
0.852094108876931
0.852094108876931
0.852247684281073
0.852291692420251
0.852688691511207
0.852712187420369
0.852752789863479
0.852832580156479
0.853162928567777
0.853162928567777
0.854010956600827
0.854010956600827
0.854010956600827
0.854010956600827
0.854010956600827
0.854185018996122
0.857166769706254
0.858222634438203
0.858222634438203
0.858222634438203
0.861393273220379
0.862476835516521
0.863263266858168
0.866103372269477
0.866164271826492
0.866645020149197
0.866802925543736

0.866802925543736
0.866802925543736
0.866802925543736
0.866802925543736
0.866995631156946
0.869463596032021
0.871219652148189
0.872416106139549
0.872416106139549
0.872897072914637
0.872897072914637
0.872897072914637
0.872897072914637
0.872897072914637
0.873970687885184
0.874164243305568
0.874872678901749
0.876594034090852
0.876636909279716
0.876636909279716
0.877305760383649
0.877305760383649
0.878078261220774
0.878078261220774
0.878078261220774
0.879501871113266
0.881084929728926
0.881084929728926
0.881422528673173
0.882164689906014
0.882243094113064
0.883127767733866
0.883127767733866
0.883801741947141
0.883801741947141
0.885079665490041
0.885146897274307
0.885457844016031

0.88565776218933
0.886059283095726
0.886246981628732
0.888296899612178
0.888296899612178
0.888296899612178
0.888296899612178
0.888296899612178
0.888296899612178
0.888296899612178
0.889254258561105
0.889440490147978
0.889440490147978
0.889440490147978
0.88978235845503
0.889847113212206
0.891203252323666
0.89142357823154
0.89142357823154
0.892269108899171
0.892522298959779
0.893558885606831
0.894815292363382
0.894815292363382
0.894857146649253
0.895922065918601
0.896254951534873
0.896733426518379
0.896911258837507
0.897988792821965
0.900729858519198
0.901279407514562
0.902368573508089
0.903898341104752
0.904541492432328
0.905390416486633
0.905390416486633
0.906032712216698

0.906032712216698
0.906032712216698
0.906032712216698
0.906465024644298
0.906465024644298
0.906640071625376
0.906692747804663
0.90978881072417
0.90978881072417
0.910442640712138
0.910442640712138
0.910442640712138
0.910442640712138
0.912248211204134
0.91308904440523
0.913720376928135
0.913720376928135
0.91563371456781
0.91563371456781
0.917335654604425
0.917335654604425
0.917335654604425
0.917335654604425
0.918531004334675
0.918531004334675
0.918531004334675
0.918831292506469
0.919390597765237
0.920011623803981
0.920011623803981
0.920011623803981
0.920731545398704
0.920731545398704
0.920731545398704
0.920731545398704
0.920731545398704
0.920805338612583
0.920805338612583

0.920805338612583
0.920805338612583
0.922172241574528
0.922172241574528
0.923052189559436
0.924531121698188
0.925663449024955
0.927386694982116
0.927386694982116
0.92792572389456
0.928127856425262
0.928127856425262
0.928127856425262
0.928127856425262
0.928127856425262
0.928127856425262
0.928788884496116
0.929952634197081
0.93002432422971
0.930631412797304
0.932349720055474
0.932499761659775
0.933663789107414
0.933784725025557
0.933784725025557
0.934049621505462
0.934065069376026
0.937218068213383
0.937525459395667
0.938450492116196
0.939201646477563
0.939941306613213
0.940182758484289
0.941743584394388
0.942309497806625
0.942987873447246
0.943261644641234
0.944735113376493

0.946829044307344
0.948218587162971
0.948218587162971
0.948218587162971
0.950387694116479
0.950387694116479
0.952650785159927
0.952768385481173
0.952768385481173
0.952768385481173
0.952768385481173
0.953431462950791
0.953718735183731
0.957436595015982
0.957671459610136
0.957776733635957
0.959225567917004
0.961017242877779
0.961017242877779
0.961955868862077
0.961955868862077
0.962968522175729
0.966955374107885
0.967396179985604
0.967396179985604
0.967396179985604
0.967396179985604
0.967396179985604
0.967396179985604
0.969257852659165
0.969257852659165
0.971823854893826
0.971823854893826
0.971823854893826
0.971823854893826
0.971823854893826
0.971873880983937
0.973727751703813

0.974890822382472
0.975292401921576
0.975292401921576
0.975292401921576
0.975292401921576
0.975292401921576
0.977104163255619
0.977171831209916
0.977300084747475
0.977868840467767
0.978351599474666
0.978351599474666
0.979809696111736
0.983101451716089
0.983101451716089
0.983199474954084
0.983199474954084
0.983199474954084
0.985167259660498
0.986750944690776
0.988274178546525
0.991533107406794
0.991979902457611
0.991979902457611
0.991979902457611
0.993906857675193
0.994367037970357
0.994367037970357
0.99511086565995
0.995339439220086
0.996922951273166
0.997170689006622
0.997170689006622
0.997170689006622
0.997217668934321
0.997568559028335
0.997783981734554
0.997861805567669

Supplementary Table S9： deCODE-specific proteins that remained significantl

protein names
6715_63_ALPPL2_PPBN
13231_90_IGHG4_IgG4__Kappa
9266_1_TREM1_sTREM_1
14260_112_NET1_ARHG8

ly associated with smoking exposure after validation analyses

Supplementary Table S10: Causal association between lifetime smoking

method	nsnp	b	se	pval	lo_ci
Inverse v	112	0.7724001687	0.071063253	1.61625	0.6331161921
Inverse v	112	0.6589660430	0.074324649	7.57865	0.5132897303
Inverse v	112	0.6425775806	0.074288183	5.15918	0.4969727415
Inverse v	112	0.6292722864	0.073470107	1.08065	0.4852708752
Inverse v	112	0.7127427415	0.087651351	4.23780	0.5409460916
Inverse v	112	0.5834803578	0.075514953	1.10382	0.4354710491
Inverse v	112	0.6705928604	0.088089231	2.68523	0.4979379672
Inverse v	112	0.6221122636	0.083358454	8.45118	0.4587296925
Inverse v	112	0.5897626806	0.079915788	1.58525	0.4331277345
Inverse v	112	-0.618263728	0.085852653	5.95748	-0.786534929
Inverse v	112	0.5797085862	0.080673716	6.67942	0.4215881025
Inverse v	112	0.5446915561	0.078805338	4.78336	0.3902330919
Inverse v	112	0.5733854277	0.085509457	2.00689	0.4057868902
Inverse v	112	0.5603474519	0.083819402	2.30647	0.3960614222
Inverse v	112	0.5350276404	0.081175095	4.36780	0.3759244528
Inverse v	112	0.5825810857	0.088659990	4.99925	0.4088075047
Inverse v	112	0.5511473709	0.084818612	8.14201	0.3849028912
Inverse v	112	0.4633097760	0.074975020	6.42960	0.3163587368
Inverse v	112	0.4801623268	0.077959445	7.31458	0.3273618143
Inverse v	112	0.4617310377	0.077524176	2.58546	0.3097836519
Inverse v	112	0.5096351719	0.086583698	3.95556	0.3399311232
Inverse v	112	0.4825139879	0.083086148	6.34527	0.3196651365
Inverse v	112	0.4961592673	0.085956858	7.82481	0.3276838240
Inverse v	112	0.5296283991	0.092355933	9.77173	0.3486107690
Inverse v	112	0.4458724954	0.078126729	1.14956	0.2927441058
Inverse v	112	0.4471374172	0.079429567	1.80882	0.2914554650
Inverse v	112	0.4292715911	0.076612686	2.10503	0.2791107252
Inverse v	112	0.4300163345	0.077220299	2.56653	0.2786645470
Inverse v	112	0.4252479417	0.076764112	3.03040	0.2747902805
Inverse v	112	0.4719693067	0.085565871	3.47079	0.3042601987
Inverse v	112	0.4898917537	0.089136463	3.88558	0.3151842850
Inverse v	112	0.4459205698	0.081388068	4.27874	0.2863999560
Inverse v	112	-0.448222616	0.081877080	4.39156	-0.608701693
Inverse v	112	0.4813414794	0.088127407	4.71113	0.3086117615
Inverse v	112	0.5308604701	0.097412057	5.04723	0.3399328371
Inverse v	112	0.4683203674	0.086219802	5.58187	0.2993295553

Inverse v	112	0.37711080061	0.0696894309	6.256693	0.24051951589
Inverse v	112	0.40737003025	0.0755869172	7.068342	0.2592196724
Inverse v	112	0.40855431237	0.0760599277	7.809341	0.25947685407
Inverse v	112	0.30947937212	0.0576432503	7.922950	0.19649860152
Inverse v	112	0.40927173450	0.0763425310	8.276590	0.25964037356
Inverse v	112	0.38377936130	0.0716285854	8.419115	0.24338733390
Inverse v	112	-0.448670145	0.0842755212	1.015847	-0.613849637
Inverse v	112	0.45690342603	0.0859886447	1.075204	0.28836568239
Inverse v	112	0.39459923439	0.0758331427	1.955493	0.24596627456
Inverse v	112	0.47786695388	0.0921048660	2.122314	0.29734141646
Inverse v	112	0.37757265209	0.0728714619	2.202840	0.23474458667
Inverse v	112	0.45358753918	0.0877379766	2.343482	0.28162110494
Inverse v	112	0.39853943120	0.0775681684	2.777887	0.24650582096
Inverse v	112	0.39910254300	0.0776646034	2.765045	0.2468799203
Inverse v	112	-0.450852197	0.0878351823	2.852599	-0.623009154
Inverse v	112	0.40742410904	0.0795034840	2.981590	0.25159728023
Inverse v	112	0.44765317961	0.0875640311	3.182678	0.27602767858
Inverse v	112	0.43528820969	0.0853943203	3.443672	0.26791534184
Inverse v	112	0.44516826527	0.0874915074	3.616093	0.27368491072
Inverse v	112	0.42114420419	0.0828137552	3.667789	0.25882924389
Inverse v	112	-0.388567927	0.0767120350	4.078007	-0.538923516
Inverse v	112	0.45011086385	0.0888516353	4.065385	0.27596165866
Inverse v	112	0.41566274868	0.0822466591	4.329787	0.25445929683
Inverse v	112	0.45404207957	0.0900912593	4.660145	0.27746321128
Inverse v	112	-0.400123168	0.0796594025	5.089054	-0.556255597
Inverse v	112	0.44867060093	0.0894248892	5.240543	0.27339781807
Inverse v	112	0.36917531773	0.0747229664	7.788772	0.22271830340
Inverse v	112	0.41855374932	0.0851161398	8.768200	0.25172611516
Inverse v	112	0.40746945451	0.0829657167	9.047492	0.24485664975
Inverse v	112	0.45056672501	0.0918076918	9.213984	0.27062364906
Inverse v	112	0.38138095161	0.0780401801	1.023934	0.2284221986
Inverse v	112	-0.270150719	0.0553661322	1.064362	-0.378668338
Inverse v	112	-0.371835158	0.0764026835	1.134303	-0.521584418
Inverse v	112	0.39162732474	0.0805383115	1.158412	0.23377223416
Inverse v	112	0.40293155144	0.0829123432	1.175463	0.24042335868
Inverse v	112	0.41308240433	0.0851130072	1.213989	0.24626091022
Inverse v	112	0.40835697983	0.0853964090	1.736471	0.24098001690
Inverse v	112	0.40754052221	0.0854803658	1.863860	0.23999900519

Inverse	112	0.3579152139	0.075652717	2.23384	0.2096358870
Inverse	112	0.3776417942	0.080132569	2.44440	0.2205819576
Inverse	112	0.3459520907	0.073570023	2.57182	0.2017548454
Inverse	112	0.3582509407	0.076185061	2.57149	0.2089282202
Inverse	112	0.3853488843	0.082317553	2.85133	0.2240064785
Inverse	112	0.4046889874	0.086526809	2.91051	0.2350964416
Inverse	112	-0.336139802	0.071845006	2.88727	-0.476956015
Inverse	112	-0.391097472	0.083702185	2.97572	-0.555153756
Inverse	112	0.3994379430	0.085994501	3.40201	0.2308887198
Inverse	112	0.3530373458	0.077101633	4.67523	0.2019181443
Inverse	112	0.3438696779	0.075180970	4.78745	0.1965149753
Inverse	112	0.3518325422	0.077686961	5.93057	0.1995660972
Inverse	112	-0.388750026	0.086108040	6.34132	-0.557521785
Inverse	112	0.3524655920	0.078156971	6.49150	0.1992779288
Inverse	112	0.2834799471	0.063026286	6.86586	0.1599484253
Inverse	112	0.3796898110	0.085061182	8.05465	0.2129698939
Inverse	112	0.3997029412	0.089727252	8.40326	0.2238375257
Inverse	112	0.3582476791	0.080567987	8.72699	0.2003344244
Inverse	112	0.3311717230	0.074457824	8.67639	0.1852343874
Inverse	112	-0.398367065	0.089547851	8.64116	-0.573880855
Inverse	112	0.3900406165	0.088061417	9.45883	0.2174402377
Inverse	112	0.3562407876	0.080492772	9.61083	0.1984749539
Inverse	112	0.3590971359	0.081203890	9.77160	0.1999375111
Inverse	112	-0.332221743	0.075868099	1.19258	-0.480923218
Inverse	112	0.4003857633	0.091743579	1.27598	0.2205683471
Inverse	112	0.3267287310	0.074926386	1.29668	0.1798730142
Inverse	112	0.3164400006	0.072648710	1.32608	0.1740485282
Inverse	112	0.3273946988	0.075166104	1.32693	0.1800691344
Inverse	112	0.3511895676	0.080857493	1.40348	0.1927088803
Inverse	112	-0.353657703	0.081601337	1.46444	-0.513596326
Inverse	112	0.3895080584	0.090050980	1.52243	0.2130081361
Inverse	112	0.2940726827	0.067987505	1.52260	0.1608171725
Inverse	112	-0.376799994	0.087407808	1.62644	-0.548119298
Inverse	112	0.4279867589	0.099267751	1.62196	0.2334219655
Inverse	112	0.3544975406	0.082724849	1.82525	0.1923568358
Inverse	112	0.2573658452	0.060125092	1.86468	0.1395206645
Inverse	112	0.3054134171	0.071519981	1.95190	0.1652342539
Inverse	112	0.3073600162	0.072180910	2.06088	0.1658854309

Inverse v	112	0.4083557720	0.095991359	2.09901	0.2202127078
Inverse v	112	0.3632480765	0.085402635	2.10587	0.1958589109
Inverse v	112	0.2985247731	0.070501958	2.29288	0.1603409354
Inverse v	112	0.3754826260	0.088719724	2.31381	0.2015919663
Inverse v	112	0.3728775138	0.088531694	2.53347	0.1993553934
Inverse v	112	-0.316879715	0.075255497	2.54559	-0.464380489
Inverse v	112	0.3713219303	0.088332571	2.62609	0.1981900895
Inverse v	112	-0.39444627	0.093805108	2.61133	-0.578304283
Inverse v	112	-0.33784098	0.080559228	2.74444	-0.495737068
Inverse v	112	0.3155224557	0.075454942	2.89463	0.1676307687
Inverse v	112	0.3188159388	0.076356819	2.97536	0.1691565719
Inverse v	112	0.3415092118	0.081788715	2.97325	0.1812033289
Inverse v	112	0.3215474319	0.077435299	3.28911	0.1697742452
Inverse v	112	0.3323517966	0.080076574	3.31858	0.1754017113
Inverse v	112	-0.353321472	0.085417297	3.52772	-0.520739376
Inverse v	112	-0.329592568	0.079836707	3.65401	-0.486072516
Inverse v	112	0.3257160634	0.079108574	3.83287	0.1706632582
Inverse v	112	0.3740076881	0.091266045	4.16746	0.1951262390
Inverse v	112	0.3487618882	0.085298241	4.33732	0.1815773351
Inverse v	112	-0.342466426	0.083854627	4.42576	-0.506821496
Inverse v	112	0.3169672022	0.077837153	4.65743	0.1644063806
Inverse v	112	0.3569633835	0.088125871	5.10846	0.1842366750
Inverse v	112	-0.355680682	0.088094736	5.40285	-0.528346365
Inverse v	112	0.3228909112	0.080100390	5.55216	0.1658941468
Inverse v	112	0.3571676969	0.088706258	5.66350	0.1833034303
Inverse v	112	0.3611970251	0.089753300	5.71374	0.1852805556
Inverse v	112	0.3036384049	0.075786772	6.16296	0.1550963300
Inverse v	112	0.3623522896	0.090556201	6.29667	0.1848621356
Inverse v	112	0.3629352756	0.090660718	6.24847	0.1852402677
Inverse v	112	0.3286585867	0.082142043	6.30485	0.1676601821
Inverse v	112	0.2909613722	0.072774316	6.38441	0.1483237117
Inverse v	112	0.3530483937	0.088591638	6.74457	0.1794087823
Inverse v	112	0.3476785258	0.087305133	6.82407	0.1765604644
Inverse v	112	0.3542120832	0.089080913	6.99969	0.1796134921
Inverse v	112	0.3505855317	0.088137509	6.95818	0.1778360134
Inverse v	112	-0.316737594	0.079789265	7.19701	-0.473124553
Inverse v	112	0.2730992972	0.069259932	8.04328	0.1373498290
Inverse v	112	0.2871365610	0.073115534	8.59540	0.1438301137

Inverse v	112	0.3133801677	0.079825461	8.64357	0.156922635
Inverse v	112	0.3047370030	0.077870075	9.10043	0.152111655
Inverse v	112	0.3194339768	0.081772083	9.36837	0.159160692
Inverse v	112	0.3155517697	0.081061709	9.91203	0.156670819
Inverse v	112	0.3162987624	0.081383035	0.00010	0.156788013
Inverse v	112	0.3389287905	0.088355401	0.00012	0.165752204
Inverse v	112	0.3136064518	0.081799356	0.00012	0.153279713
Inverse v	112	0.3539639471	0.092713993	0.00013	0.172244519
Inverse v	112	0.3512817904	0.092087401	0.00013	0.170790483
Inverse v	112	0.2652370918	0.069568348	0.00013	0.128883128
Inverse v	112	0.3421327443	0.089848222	0.00014	0.166030228
Inverse v	112	0.3036980888	0.080040423	0.00014	0.146818859
Inverse v	112	-0.736822123	0.194431705	0.00015	-1.117908267
Inverse v	112	0.3143160090	0.082988733	0.00015	0.151658091
Inverse v	112	0.3257624026	0.086040603	0.00015	0.157122820
Inverse v	112	0.3203822589	0.084712139	0.00015	0.154346465
Inverse v	112	0.3303676221	0.087622262	0.00016	0.158627987
Inverse v	112	0.3151017925	0.083652615	0.00016	0.151142667
Inverse v	112	0.3384191189	0.089814617	0.00016	0.162382469
Inverse v	112	0.2775496143	0.074101610	0.00018	0.132310457
Inverse v	112	0.2902383629	0.077594790	0.00018	0.138152574
Inverse v	112	0.3061070186	0.082218686	0.00019	0.144958393
Inverse v	112	-0.280551873	0.075488871	0.00020	-0.42851006
Inverse v	112	-0.314583632	0.084618532	0.00020	-0.480435955
Inverse v	112	0.3070745962	0.082692681	0.00020	0.144996939
Inverse v	112	0.2962517700	0.079866157	0.00020	0.139714101
Inverse v	112	0.3193761955	0.086663647	0.00022	0.149515446
Inverse v	112	0.3258294437	0.088646363	0.00023	0.152082571
Inverse v	112	-0.29564958	0.080773546	0.00025	-0.453965731
Inverse v	112	0.2171018066	0.059425376	0.00025	0.100628068
Inverse v	112	0.2801098752	0.076933543	0.00027	0.129320129
Inverse v	112	0.3172339238	0.087129871	0.00027	0.146459376
Inverse v	112	0.2970634156	0.081689503	0.00027	0.136951989
Inverse v	112	0.3586963508	0.098957338	0.00028	0.164739968
Inverse v	112	0.2971703456	0.082131120	0.00029	0.136193350
Inverse v	112	0.2103619861	0.058243053	0.00030	0.096205600
Inverse v	112	0.2942833789	0.081453249	0.00030	0.134635010
Inverse v	112	-0.24859233	0.068915754	0.00030	-0.383667209

Inverse \	112	0.1773810667	0.0491751186	0.00030	0.0809978340
Inverse \	112	0.2860159126	0.0795187294	0.00032	0.1301592030
Inverse \	112	0.1803955388	0.0501671047	0.00032	0.0820680134
Inverse \	112	0.2805241961	0.0781046475	0.00032	0.1274390869
Inverse \	112	-0.304878478	0.0851741070	0.00034	-0.471819728
Inverse \	112	0.2671223493	0.0747669604	0.00035	0.1205791068
Inverse \	112	0.2501844308	0.0704071789	0.00038	0.1121863601
Inverse \	112	0.3279750219	0.0924053827	0.00038	0.1468604716
Inverse \	112	0.3081564957	0.0874540842	0.00042	0.1367464905
Inverse \	112	0.2619598846	0.0744045563	0.00043	0.1161269542
Inverse \	112	-0.317902337	0.0903610663	0.00043	-0.495010027
Inverse \	112	0.2574992431	0.0737667244	0.00048	0.1129164631
Inverse \	112	0.2987376049	0.0857686392	0.00049	0.1306310720
Inverse \	112	0.2449320893	0.0703895817	0.00050	0.1069685090
Inverse \	112	0.2773957179	0.0797647199	0.00050	0.1210568668
Inverse \	112	-0.315439588	0.0907707262	0.00051	-0.493350211
Inverse \	112	0.3049041220	0.0880876826	0.00053	0.1322522640
Inverse \	112	0.3122249629	0.0902169907	0.00053	0.1353996611
Inverse \	112	0.2726888129	0.0792753421	0.00058	0.1173091424
Inverse \	112	0.2808821143	0.0816932063	0.00058	0.1207634298
Inverse \	112	0.2813091224	0.0819847684	0.00060	0.1206189763
Inverse \	112	0.2611541900	0.0762667627	0.00061	0.1116713349
Inverse \	112	0.2906971836	0.0850581775	0.00063	0.1239831555
Inverse \	112	0.2730433456	0.0798773237	0.00063	0.1164837910
Inverse \	112	0.3147804883	0.0920173480	0.00062	0.1344264861
Inverse \	112	0.3156694551	0.0923701487	0.00063	0.1346239635
Inverse \	112	0.2868870378	0.0841068972	0.00064	0.1220375191
Inverse \	112	0.2340484087	0.0685935633	0.00064	0.0996050245
Inverse \	112	0.2621344023	0.0768897171	0.00065	0.1114305566
Inverse \	112	0.2574495218	0.0756452651	0.00066	0.1091848021
Inverse \	112	0.2480739454	0.0729487670	0.00067	0.1050943619
Inverse \	112	0.2552360105	0.0751479051	0.00068	0.1079461163
Inverse \	112	0.3191948271	0.0942998031	0.00071	0.1343672130
Inverse \	112	0.2796357600	0.0827164751	0.00072	0.1175114688
Inverse \	112	0.2689630991	0.0796740332	0.00073	0.1128019939
Inverse \	112	-0.333866457	0.0990275329	0.00074	-0.527960421
Inverse \	112	0.2833519516	0.0843679872	0.00078	0.1179906967
Inverse \	112	0.3335852161	0.0994383073	0.00079	0.1386861337

Inverse v	112	-0.372249692	0.111032379(	0.00080(	-0.589873156
Inverse v	112	0.2398595654:	0.071663578(	0.00081(	0.0993989512
Inverse v	112	-0.262336497	0.0787739102	0.00086	-0.416733361
Inverse v	112	0.2741606119	0.082618982(	0.00090	0.1122274072
Inverse v	112	-0.267993014	0.080817540	0.00091	-0.426395393
Inverse v	112	0.2658380164	0.080389178	0.00094	0.1082752264
Inverse v	112	0.2499510362	0.075710543	0.00096	0.1015583714
Inverse v	112	0.268112481	0.081366973	0.00098	0.1086332120
Inverse v	112	0.2861329302	0.086808247	0.00098(	0.1159887658
Inverse v	112	0.2726801678	0.083351433(	0.00106	0.1093113590
Inverse v	112	0.2382731748	0.072957924	0.00109	0.0952756428
Inverse v	112	0.2503840807(	0.076719040	0.00109	0.1000147606
Inverse v	112	0.2654214746	0.081394762(	0.00111(	0.1058877409
Inverse v	112	0.2672227296(	0.082038046	0.00112	0.1064281590
Inverse v	112	0.1885426068(	0.057956725(	0.00114	0.0749474245(
Inverse v	112	0.2733123660	0.084130004	0.00115	0.1084175572
Inverse v	112	0.2543920731	0.078819471	0.00124	0.0999059093
Inverse v	112	-0.257796521	0.079792903	0.00123	-0.414190611
Inverse v	112	0.2538896551	0.078628939	0.00124	0.0997769327
Inverse v	112	0.2415585724(	0.074839948	0.00124	0.0948722734
Inverse v	112	0.2784959811(	0.086730253	0.00132	0.1085046843
Inverse v	112	0.2852451348	0.088807123	0.00131	0.1118317326
Inverse v	112	0.2477642912(	0.077278332	0.00134	0.0962987594(
Inverse v	112	0.2608331218	0.081458699	0.00136	0.1011740712
Inverse v	112	0.2352132627(	0.073439136	0.00136(	0.0912725548(
Inverse v	112	0.2889400127	0.090304948	0.00137(	0.1119423136
Inverse v	112	0.3007997687	0.094104401	0.00139	0.1163551413
Inverse v	112	0.2784804866	0.087246391(	0.00141	0.1074775590
Inverse v	112	0.2547861934(	0.079829018	0.00141	0.0983213170(
Inverse v	112	0.2488710556	0.078261922	0.00147	0.0954776876
Inverse v	112	0.2598065960	0.081778911	0.00148	0.0995199294
Inverse v	112	0.2187225185	0.068916490	0.00150	0.0836461964
Inverse v	112	0.2473215020	0.077986517	0.00151	0.0944679269
Inverse v	112	0.2427981448(	0.076638097	0.00153	0.0925874738
Inverse v	112	-0.27195059	0.085990471	0.00156	-0.440491915
Inverse v	112	-0.270172948	0.085607728	0.00159	-0.437964096
Inverse v	112	0.2814165605	0.089321139	0.00162	0.1063471277
Inverse v	112	0.2518963158	0.080011604(	0.00164	0.0950735706

Inverse v	112	0.2603115960	0.082852367	0.00167	0.097920954
Inverse v	112	0.239198775	0.076209535	0.00169	0.089828085
Inverse v	112	0.2475502151	0.079247975	0.00178	0.092224182
Inverse v	112	0.2729134011	0.087474747	0.00180	0.101462896
Inverse v	112	0.2471740332	0.079385978	0.00184	0.091577516
Inverse v	112	0.2731456463	0.088239416	0.00196	0.100196390
Inverse v	112	0.2781520179	0.089994929	0.00199	0.101761956
Inverse v	112	0.3025744246	0.097924055	0.00200	0.110643276
Inverse v	112	0.2798900570	0.090809827	0.00205	0.101902794
Inverse v	112	0.2143463632	0.069724944	0.00211	0.077685471
Inverse v	112	-0.230526015	0.075051225	0.00212	-0.377626417
Inverse v	112	0.2966755939	0.096657154	0.00214	0.107227570
Inverse v	112	0.2506396314	0.081640272	0.00214	0.090624696
Inverse v	112	0.2511337541	0.081853891	0.00215	0.090700126
Inverse v	112	0.1797034908	0.058662359	0.00218	0.064725266
Inverse v	112	0.2309817041	0.075459147	0.00220	0.083081775
Inverse v	112	0.2520660325	0.082508673	0.00225	0.090349032
Inverse v	112	0.2701497428	0.088507947	0.00227	0.096674166
Inverse v	112	0.2098272075	0.068879372	0.00231	0.074823637
Inverse v	112	-0.27747073	0.091054221	0.00230	-0.455937004
Inverse v	112	-0.288521231	0.094900372	0.00236	-0.47452596
Inverse v	112	0.2728682420	0.089932128	0.00241	0.096601270
Inverse v	112	0.2811252625	0.092688783	0.00242	0.099455247
Inverse v	112	0.2098127776	0.069158623	0.00241	0.074261875
Inverse v	112	0.2250447868	0.074448851	0.00250	0.079125037
Inverse v	112	-0.245516397	0.081346631	0.00254	-0.404955794
Inverse v	112	0.2231827114	0.073943853	0.00254	0.078252759
Inverse v	112	0.2630536686	0.087215986	0.00256	0.092110335
Inverse v	112	0.2504856236	0.083087965	0.00257	0.087633210
Inverse v	112	0.2383374949	0.079188417	0.00261	0.083128197
Inverse v	112	0.2460396620	0.081822908	0.00263	0.085666761
Inverse v	112	0.2567719777	0.085579570	0.00269	0.089036019
Inverse v	112	0.2585916769	0.086359529	0.00275	0.089326998
Inverse v	112	0.2138716486	0.071728968	0.00286	0.073282870
Inverse v	112	0.2209878803	0.074208593	0.00290	0.075539036
Inverse v	112	0.2268216079	0.076484873	0.00302	0.076911255
Inverse v	112	0.2647434523	0.089349919	0.00304	0.089617609
Inverse v	112	-0.354241824	0.119529119	0.00304	-0.588518898

Inverse v	112	0.23019960019	0.07795844488	0.003148	0.07740104045
Inverse v	112	0.22960318625	0.07783694511	0.003179	0.07704277377
Inverse v	112	0.22822373591	0.07739435400	0.003189	0.07653080205
Inverse v	112	0.29737436483	0.10095412611	0.003222	0.09950427749
Inverse v	112	0.24010717238	0.08177423900	0.003322	0.07982966388
Inverse v	112	-0.229092142	0.07820730977	0.003397	-0.382378469
Inverse v	112	0.19043573203	0.06500965778	0.003390	0.06301680258
Inverse v	112	-0.216696508	0.07394174044	0.003382	-0.36162232
Inverse v	112	0.21953974308	0.07503968899	0.003437	0.07246195271
Inverse v	112	-0.231966437	0.07950493055	0.003526	-0.387796101
Inverse v	112	0.21833086505	0.07502063900	0.003611	0.07129041251
Inverse v	112	0.21197566017	0.07295359560	0.003665	0.06898661272
Inverse v	112	0.19260164684	0.06638011688	0.003713	0.06249661784
Inverse v	112	-0.265197326	0.09141628900	0.003719	-0.444373253
Inverse v	112	0.22803195350	0.07898253844	0.003887	0.07322617820
Inverse v	112	0.23883689283	0.08270395860	0.003878	0.07673713383
Inverse v	112	0.25712174587	0.08909483622	0.003902	0.08249586676
Inverse v	112	0.22976429110	0.07980762944	0.003989	0.07334133734
Inverse v	112	0.25875568064	0.09001525112	0.004045	0.08232578827
Inverse v	112	-0.399391569	0.13890408822	0.004036	-0.671643582
Inverse v	112	0.25330698602	0.08806843060	0.004024	0.08069286196
Inverse v	112	-0.236706514	0.08239027788	0.004060	-0.398191459
Inverse v	112	-0.234880808	0.08201290455	0.004183	-0.3956261
Inverse v	112	0.23735400247	0.08297785112	0.004230	0.07471741412
Inverse v	112	0.19573530841	0.06845264077	0.004244	0.06156813249
Inverse v	112	0.19269902289	0.06741960377	0.004260	0.06055659951
Inverse v	112	0.21846391054	0.07659309677	0.004340	0.06834144086
Inverse v	112	0.26173641634	0.09178795555	0.004350	0.08183202337
Inverse v	112	0.20167560671	0.07070481777	0.004339	0.06309416383
Inverse v	112	0.21855552190	0.07699939460	0.004533	0.06763670834
Inverse v	112	0.25072296509	0.08862433044	0.004668	0.07701927745
Inverse v	112	0.26187119364	0.09266602177	0.004713	0.08024579104
Inverse v	112	0.21172370258	0.07499950460	0.004757	0.06472467343
Inverse v	112	0.24589969903	0.08717578112	0.004791	0.07503516782
Inverse v	112	-0.270819859	0.09661989882	0.005063	-0.460194859
Inverse v	112	0.21864762825	0.07805965911	0.005093	0.06565069640
Inverse v	112	0.27152226060	0.09725917677	0.005242	0.08089427423
Inverse v	112	0.25091414199	0.08986320733	0.005235	0.07478225562

Inverse v	112	-0.211558366	0.0759598004	0.005350	-0.360439575
Inverse v	112	0.20677958798	0.0744682767	0.005490	0.06082176551
Inverse v	112	0.25628171010	0.0924314673	0.005559	0.07511603414
Inverse v	112	0.25008074222	0.0906323585	0.005792	0.07244131953
Inverse v	112	-0.158771384	0.0576200590	0.005860	-0.2717067
Inverse v	112	0.22617543861	0.0823973831	0.006052	0.06467656756
Inverse v	112	-0.251914409	0.0918758142	0.006108	-0.431991005
Inverse v	112	-0.238380849	0.0869783584	0.006131	-0.408858432
Inverse v	112	0.21385412874	0.0780704574	0.006158	0.06083603220
Inverse v	112	-0.250695655	0.0915420200	0.006170	-0.430118015
Inverse v	112	0.19883946867	0.0727516038	0.006273	0.05624632517
Inverse v	112	0.18270497520	0.0668503895	0.006275	0.05167821176
Inverse v	112	-0.192394421	0.0707621580	0.006550	-0.33108825
Inverse v	112	0.23780351167	0.0877558045	0.006731	0.06580213485
Inverse v	112	0.25144137544	0.0929202376	0.006810	0.06931770959
Inverse v	112	0.20336420610	0.0753158597	0.006930	0.05574512103
Inverse v	112	0.19882640350	0.0738272399	0.007078	0.05412501321
Inverse v	112	0.20528612601	0.0762801705	0.007119	0.05577699179
Inverse v	112	-0.188307263	0.0699944510	0.007138	-0.325496387
Inverse v	112	0.23541647480	0.0875032898	0.007137	0.06391002672
Inverse v	112	0.24308246454	0.0904244551	0.007183	0.06585053238
Inverse v	112	-0.260182409	0.0972474365	0.007462	-0.450787385
Inverse v	112	-0.225491528	0.0845011252	0.007619	-0.391113733
Inverse v	112	0.22990724935	0.0862166492	0.007661	0.06092261680
Inverse v	112	0.19577180965	0.0734347955	0.007671	0.05183961034
Inverse v	112	0.22037104322	0.0827529700	0.007744	0.05817522197
Inverse v	112	0.21902654423	0.0822256895	0.007728	0.05786419274
Inverse v	112	0.24281274445	0.0912470098	0.007789	0.06396860507
Inverse v	112	0.24995671563	0.0940692553	0.007880	0.06558097514
Inverse v	112	0.23843236031	0.0898196871	0.007941	0.06238577358
Inverse v	112	-0.191617697	0.0722080483	0.007961	-0.333145472
Inverse v	112	0.14196185613	0.0535164536	0.007985	0.03706960693
Inverse v	112	-0.216202973	0.0816852069	0.008120	-0.376305978
Inverse v	112	0.19062005769	0.0723499448	0.008421	0.04881416569
Inverse v	112	0.22070590008	0.0838448741	0.008480	0.05636994681
Inverse v	112	0.13786324953	0.0524685475	0.008600	0.03502489641
Inverse v	112	-0.212318308	0.0809055474	0.008683	-0.370893181
Inverse v	112	0.23333037351	0.0891046253	0.008828	0.05868530778

Inverse v	112	0.2057694517	0.078766000	0.00899	0.0513880904
Inverse v	112	0.2086261235	0.079908242	0.00903	0.0520059685
Inverse v	112	-0.23194672	0.089045990	0.00919	-0.406476862
Inverse v	112	0.1886265619	0.072426438	0.00920	0.0466707425
Inverse v	112	0.2351785657	0.090404825	0.00928	0.0579851076
Inverse v	112	-0.242676758	0.094022778	0.00985	-0.426961405
Inverse v	112	0.1419058229	0.054996261	0.00987	0.0341131510
Inverse v	112	0.2288943856	0.088826051	0.00996	0.0547953253
Inverse v	112	0.1226158468	0.047610373	0.01001	0.0292995156
Inverse v	112	-0.185469222	0.072266526	0.01027	-0.327111614
Inverse v	112	0.2250928041	0.087841829	0.01039	0.0529228183
Inverse v	112	0.2323157005	0.090983841	0.01066	0.0539873722
Inverse v	112	0.2321602522	0.091085798	0.01080	0.0536320880
Inverse v	112	0.2285612604	0.089969563	0.01107	0.0522209163
Inverse v	112	0.1730005205	0.068167903	0.01115	0.0393914294
Inverse v	112	0.2134783233	0.084022993	0.01106	0.0487932560
Inverse v	112	0.1076094346	0.042391532	0.01113	0.0245220302
Inverse v	112	0.1560173994	0.061454705	0.01112	0.0355661768
Inverse v	112	0.1915929987	0.075435189	0.01109	0.0437400279
Inverse v	112	0.1957437669	0.077427697	0.01146	0.0439854799
Inverse v	112	0.2023661497	0.080080958	0.01150	0.0454074702
Inverse v	112	0.2190404362	0.086714078	0.01153	0.0490808415
Inverse v	112	0.2792533929	0.110795792	0.01172	0.0620936406
Inverse v	112	0.2349767453	0.093682393	0.01213	0.0513592535
Inverse v	112	0.1946132674	0.077659970	0.01221	0.0423997262
Inverse v	112	0.2468971685	0.098677127	0.01234	0.0534899985
Inverse v	112	0.2002873634	0.080196881	0.01250	0.0431014748
Inverse v	112	0.2102843481	0.084298114	0.01261	0.0450600440
Inverse v	112	0.1810597945	0.072721420	0.01278	0.0385258113
Inverse v	112	0.2674585484	0.107396333	0.01276	0.0569617350
Inverse v	112	-0.198928197	0.080046788	0.01294	-0.355819903
Inverse v	112	0.2001346049	0.080513554	0.01292	0.0423280388
Inverse v	112	-0.218644778	0.088185074	0.01316	-0.391487524
Inverse v	112	0.2064941901	0.083444126	0.01333	0.0429437017
Inverse v	112	0.1972540874	0.079786576	0.01342	0.0408723974
Inverse v	112	0.1873056427	0.075822312	0.01349	0.0386939102
Inverse v	112	0.1965635389	0.079533124	0.01345	0.0406786142
Inverse v	112	-0.204491945	0.082768495	0.01348	-0.366718196

Inverse	112	0.2084661397	0.084481220	0.01360	0.0428829470
Inverse	112	-0.197536731	0.080026275	0.01357	-0.354388231
Inverse	112	0.2031433544	0.082370603	0.01365	0.0416969707
Inverse	112	0.1988262103	0.080692672	0.01373	0.0406685720
Inverse	112	0.2068733065	0.084058229	0.01385	0.0421191762
Inverse	112	0.2254029775	0.091826151	0.01410	0.0454237202
Inverse	112	-0.184165365	0.075124859	0.01422	-0.331410089
Inverse	112	0.2138940166	0.087384461	0.01437	0.0426204724
Inverse	112	0.2208518916	0.090528644	0.01470	0.0434157476
Inverse	112	0.1852129690	0.075943908	0.01473	0.0363629079
Inverse	112	0.3143398010	0.129620545	0.01530	0.0602835327
Inverse	112	0.2069814887	0.085570360	0.01556	0.0392635822
Inverse	112	0.1863782332	0.077174981	0.01573	0.0351152702
Inverse	112	0.2008457978	0.083186469	0.01576	0.0378003167
Inverse	112	-0.206392629	0.085652548	0.01596	-0.374271625
Inverse	112	0.1910537584	0.079311278	0.01600	0.0356036527
Inverse	112	0.1902245083	0.079008131	0.01605	0.0353685707
Inverse	112	-0.193279164	0.080341031	0.01614	-0.350747586
Inverse	112	0.2144260406	0.089333603	0.01638	0.0393321780
Inverse	112	0.2045471691	0.085675008	0.01696	0.0366241533
Inverse	112	0.2069814354	0.086833843	0.01714	0.0367871019
Inverse	112	-0.231327078	0.097135634	0.01724	-0.421712922
Inverse	112	0.2008773082	0.084561944	0.01752	0.0351358975
Inverse	112	0.1880109366	0.079289458	0.01773	0.0326035983
Inverse	112	-0.207101431	0.087353734	0.01774	-0.37831475
Inverse	112	0.2016478281	0.085416028	0.01823	0.0342324121
Inverse	112	0.1844067368	0.078119860	0.01824	0.0312918112
Inverse	112	0.2225490599	0.094439229	0.01844	0.0374481707
Inverse	112	0.1944874854	0.082564939	0.01849	0.0326602046
Inverse	112	-0.179633343	0.076295575	0.01855	-0.329172671
Inverse	112	0.2059615946	0.087766295	0.01894	0.0339396559
Inverse	112	0.2101030935	0.089754278	0.01923	0.0341847086
Inverse	112	0.1878959248	0.080339157	0.01934	0.0304311761
Inverse	112	0.1771360871	0.076012984	0.01978	0.0281506371
Inverse	112	-0.200493671	0.086142578	0.01994	-0.369333126
Inverse	112	0.1978279651	0.085175236	0.02020	0.0308845024
Inverse	112	0.2009073188	0.086683419	0.02046	0.0310078165
Inverse	112	-0.171512343	0.074366514	0.02109	-0.317270711

Inverse v	112	-0.191029755	0.0829375020	0.021262	-0.353587259
Inverse v	112	-0.195522753	0.0849433940	0.021340	-0.362011805
Inverse v	112	0.2014646536	0.0876732881	0.021567	0.0296250088
Inverse v	112	0.1707080817	0.0742453735	0.021491	0.0251871496
Inverse v	112	0.1846582848	0.0803504732	0.021552	0.0271713572
Inverse v	112	0.2438472417	0.1067624509	0.022370	0.0345928378
Inverse v	112	-0.184617604	0.0813245008	0.023199	-0.344013625
Inverse v	112	0.1770310621	0.0779275696	0.023102	0.0242930256
Inverse v	112	0.1924401038	0.0847702322	0.023199	0.0262904486
Inverse v	112	0.1984937929	0.0874370014	0.023199	0.0271172701
Inverse v	112	-0.14764608	0.0651647899	0.023467	-0.275369069
Inverse v	112	0.1605625236	0.0709236383	0.023581	0.0215521924
Inverse v	112	-0.184134002	0.0813589863	0.023621	-0.343597615
Inverse v	112	-0.182633958	0.0810476521	0.024233	-0.341487357
Inverse v	112	-0.192918829	0.0856568918	0.024307	-0.360806338
Inverse v	112	0.1815422639	0.0806565990	0.024397	0.0234553298
Inverse v	112	0.1741445735	0.0774205978	0.024491	0.0224002017
Inverse v	112	-0.206827282	0.0919834466	0.024542	-0.387114837
Inverse v	112	0.1664748792	0.0740793881	0.024624	0.0212792784
Inverse v	112	0.1922418199	0.0855702705	0.024669	0.0245240896
Inverse v	112	0.1870397788	0.0833352836	0.024805	0.0237026228
Inverse v	112	0.1648428517	0.0738491720	0.025604	0.0200984744
Inverse v	112	0.2209719156	0.0990677733	0.025713	0.0267990799
Inverse v	112	0.1577704295	0.0707139389	0.025673	0.0191711093
Inverse v	112	0.3152367577	0.1414290617	0.025817	0.0380357967
Inverse v	112	-0.185972463	0.0835512336	0.026024	-0.349732881
Inverse v	112	-0.19943198	0.0895823262	0.025997	-0.37501334
Inverse v	112	-0.148788692	0.0669565413	0.026271	-0.280023513
Inverse v	112	-0.178498828	0.0804521375	0.026507	-0.336185018
Inverse v	112	0.1757814752	0.0793096070	0.026664	0.0203346455
Inverse v	112	-0.181930933	0.0821593883	0.026803	-0.342963334
Inverse v	112	0.1871940447	0.0845291717	0.026790	0.0215168680
Inverse v	112	0.1756461439	0.0795362813	0.027217	0.0197550324
Inverse v	112	0.1940113096	0.0882052458	0.027839	0.0211290277
Inverse v	112	0.1935399012	0.0880643721	0.027969	0.0209337317
Inverse v	112	0.2041612496	0.0928636607	0.027913	0.0221484746
Inverse v	112	0.1233814377	0.0561785951	0.028075	0.0132713912
Inverse v	112	0.1910533682	0.0870614785	0.028201	0.0204128703

Inverse v	112	0.1868359656	0.085126340	0.02817	0.0199883375
Inverse v	112	0.1662892077	0.075869915	0.02839	0.0175841731
Inverse v	112	0.1753433488	0.079996493	0.02838	0.0185502215
Inverse v	112	0.1775854289	0.081251696	0.02884	0.0183321041
Inverse v	112	-0.196813038	0.090498675	0.02964	-0.374190442
Inverse v	112	0.1795642335	0.082660797	0.02983	0.0175490707
Inverse v	112	0.2024447365	0.093277294	0.02998	0.0196212402
Inverse v	112	0.2089889847	0.096384604	0.03013	0.0200751593
Inverse v	112	0.1596842254	0.073841208	0.03057	0.0149554566
Inverse v	112	-0.169406593	0.078426886	0.03076	-0.323123291
Inverse v	112	0.1860433486	0.086156632	0.03082	0.0171763491
Inverse v	112	0.1598003361	0.074053170	0.03093	0.0146561225
Inverse v	112	0.1887812441	0.087983838	0.03190	0.0163329207
Inverse v	112	0.2082417769	0.097073412	0.03193	0.0179778875
Inverse v	112	0.1806493895	0.084181387	0.03187	0.0156538709
Inverse v	112	0.1694613437	0.079066846	0.03209	0.0144903252
Inverse v	112	0.1724269444	0.080911718	0.03308	0.0138399759
Inverse v	112	0.1854039400	0.087069615	0.03322	0.0147474935
Inverse v	112	0.1721399908	0.080888606	0.03332	0.0135983220
Inverse v	112	0.1402461056	0.065925804	0.03339	0.0110315279
Inverse v	112	0.1901819141	0.089509977	0.03361	0.0147423577
Inverse v	112	0.1631677611	0.077092308	0.03430	0.0120668371
Inverse v	112	0.1634261238	0.077207980	0.03428	0.0120984826
Inverse v	112	0.1689893319	0.079813990	0.03423	0.0125539103
Inverse v	112	0.1593746071	0.075483082	0.03473	0.0114277662
Inverse v	112	-0.176510146	0.083804148	0.03518	-0.340766278
Inverse v	112	0.1544255462	0.073323511	0.03519	0.0107114632
Inverse v	112	-0.167730229	0.079954465	0.03592	-0.324440981
Inverse v	112	0.1565884204	0.074716012	0.03610	0.0101450350
Inverse v	112	0.1646528331	0.078731863	0.03650	0.0103383813
Inverse v	112	0.1405389233	0.067199292	0.03649	0.0088283109
Inverse v	112	0.1286640835	0.061507950	0.03645	0.0081085011
Inverse v	112	0.1719432305	0.082359574	0.03682	0.0105184646
Inverse v	112	0.1666291486	0.079798501	0.03678	0.0102240859
Inverse v	112	0.1619823045	0.077685454	0.03705	0.0097188140
Inverse v	112	-0.168231088	0.080936252	0.03765	-0.326866142
Inverse v	112	-0.185617394	0.089602949	0.03830	-0.361239176
Inverse v	112	0.1508829875	0.072844108	0.03832	0.0081085342

Inverse v	112	0.14689271285	0.07104220095	0.03866	0.00764999902
Inverse v	112	0.1579181062	0.0764574949	0.03888	0.0080614160
Inverse v	112	0.1695208230	0.0822454213	0.03928	0.0083197972
Inverse v	112	0.1108517769	0.0537822714	0.03929	0.0054385249
Inverse v	112	0.1741864853	0.0845885142	0.03947	0.0083929974
Inverse v	112	-0.197337322	0.0958716658	0.03955	-0.385245787
Inverse v	112	0.1433160380	0.0698671807	0.04024	0.0063763638
Inverse v	112	0.1815436399	0.0888957527	0.04113	0.0073079646
Inverse v	112	-0.161017734	0.0789662160	0.04144	-0.315791519
Inverse v	112	0.1485317953	0.0732087269	0.04247	0.0050426905
Inverse v	112	0.1940998876	0.0958360059	0.04283	0.0062613159
Inverse v	112	0.1658445867	0.0820572600	0.04327	0.0050123570
Inverse v	112	0.1628003436	0.0806580634	0.04354	0.0047105392
Inverse v	112	0.1906049008	0.0944048296	0.04348	0.0055714347
Inverse v	112	0.1411453318	0.0700908662	0.04403	0.0037672340
Inverse v	112	0.1432592836	0.0712830781	0.04446	0.0035444504
Inverse v	112	0.1498693533	0.0747263217	0.04490	0.0034057627
Inverse v	112	0.1678855213	0.0839534257	0.04552	0.0033368067
Inverse v	112	0.1690340999	0.0845821001	0.04566	0.0032531837
Inverse v	112	0.1693080986	0.0847855013	0.04583	0.0031285159
Inverse v	112	-0.172550709	0.0864456594	0.04592	-0.341984201
Inverse v	112	0.1504486618	0.0755120049	0.04632	0.0024451321
Inverse v	112	-0.155176851	0.0778868671	0.04633	-0.307835111
Inverse v	112	0.1656468900	0.0831792615	0.04643	0.0026155374
Inverse v	112	0.1652310791	0.0830796893	0.04672	0.0023948879
Inverse v	112	-0.16588625	0.0834292720	0.04677	-0.329407623
Inverse v	112	0.1682885531	0.0846954977	0.04692	0.0022853775
Inverse v	112	-0.198006186	0.0999771105	0.04764	-0.393961322
Inverse v	112	0.1691031500	0.0855244893	0.04801	0.0014751509
Inverse v	112	0.1478216154	0.0747631014	0.04801	0.0012859365
Inverse v	112	0.1712352841	0.0867636290	0.04842	0.0011785711
Inverse v	112	0.1670227592	0.0846079870	0.04837	0.0011911046
Inverse v	112	0.0973668573	0.0493794976	0.04863	0.0005830420
Inverse v	112	0.1486574502	0.0754725708	0.04887	0.0007312113
Inverse v	112	0.1555201605	0.0790749277	0.04921	0.0005333021
Inverse v	112	0.1537082661	0.0781277341	0.04913	0.0005779073
Inverse v	112	0.1678441197	0.0852887069	0.04907	0.0006782541
Inverse v	112	0.1678519505	0.0854097452	0.04938	0.0004488499

Inverse v	112	-0.152547753	0.07764955	0.04946	-0.304740882
Inverse v	112	0.16220476101	0.083116944	0.05099	-0.000704451
Inverse v	112	-0.15855235	0.08127284	0.05107	-0.317847116
Inverse v	112	0.1539750605	0.078932769	0.05109	-0.000733168
Inverse v	112	-0.163937863	0.084060532	0.05114	-0.328696506
Inverse v	112	0.1403955048	0.072166268	0.05172	-0.001050381
Inverse v	112	0.1545802093	0.079547954	0.05198	-0.001333782
Inverse v	112	0.1875279214	0.096592619	0.05220	-0.001793613
Inverse v	112	0.1659433322	0.085798301	0.05309	-0.002221339
Inverse v	112	0.1664849106	0.086074515	0.05308	-0.002221139
Inverse v	112	0.1809394568	0.093571224	0.05314	-0.002460143
Inverse v	112	0.1726709308	0.089538041	0.05379	-0.002823631
Inverse v	112	-0.153393087	0.079516194	0.05372	-0.309244828
Inverse v	112	0.1521966014	0.078984324	0.05398	-0.002612675
Inverse v	112	0.1441954050	0.074977827	0.05445	-0.002761137
Inverse v	112	0.1738630121	0.090469440	0.05463	-0.003457091
Inverse v	112	0.1231296024	0.064068363	0.05462	-0.00244439
Inverse v	112	-0.173830565	0.090595045	0.05501	-0.351396854
Inverse v	112	0.1693291271	0.088332525	0.05524	-0.003802624
Inverse v	112	-0.176318621	0.092030171	0.05538	-0.356697758
Inverse v	112	0.1678630327	0.087810178	0.05592	-0.004244917
Inverse v	112	0.1740955826	0.091178502	0.05621	-0.004614281
Inverse v	112	0.1649918570	0.086497438	0.05645	-0.004543123
Inverse v	112	0.1788340759	0.093799247	0.05657	-0.00501245
Inverse v	112	0.1510529830	0.079320644	0.05686	-0.004415481
Inverse v	112	-0.159422102	0.083753784	0.05698	-0.323579519
Inverse v	112	-0.14078146	0.074059297	0.05731	-0.285937684
Inverse v	112	0.1469312320	0.077358529	0.05751	-0.004691486
Inverse v	112	-0.151698816	0.080000805	0.05793	-0.308500394
Inverse v	112	0.1706457692	0.090131519	0.05831	-0.006012009
Inverse v	112	-0.159720119	0.084500798	0.05873	-0.325341683
Inverse v	112	0.2601744593	0.137806191	0.05902	-0.009925675
Inverse v	112	0.1874893119	0.099348258	0.05913	-0.007233274
Inverse v	112	-0.142588198	0.075597610	0.05927	-0.290759515
Inverse v	112	-0.145021837	0.076955875	0.05950	-0.295855354
Inverse v	112	0.1624270143	0.086244574	0.05965	-0.006612351
Inverse v	112	0.1507146536	0.080149860	0.06005	-0.006379073
Inverse v	112	0.1376215687	0.073349507	0.06062	-0.006143466

Inverse	112	0.1231541386	0.065695674	0.06084	-0.005609383
Inverse	112	0.1461986420	0.078209410	0.06157	-0.007091803
Inverse	112	0.1532821341	0.082018102	0.06163	-0.007473347
Inverse	112	-0.14978649	0.080118375	0.06154	-0.306818505
Inverse	112	0.1552024618	0.083028612	0.06158	-0.007533619
Inverse	112	0.1597448357	0.085564315	0.06190	-0.007961222
Inverse	112	0.1449824076	0.077767541	0.06227	-0.007441974
Inverse	112	-0.147133277	0.078957852	0.06240	-0.301890668
Inverse	112	0.1501281812	0.080800134	0.06316	-0.008240083
Inverse	112	0.1662845749	0.089601380	0.06347	-0.009334131
Inverse	112	0.1554545169	0.083882190	0.06384	-0.008954576
Inverse	112	0.1474961327	0.079663651	0.06409	-0.008644625
Inverse	112	0.1370744006	0.074196105	0.06468	-0.008349966
Inverse	112	0.0915873505	0.049678800	0.06524	-0.005783098
Inverse	112	0.1514621983	0.082628673	0.06679	-0.010490002
Inverse	112	-0.142468042	0.077820001	0.06713	-0.294995246
Inverse	112	0.1380325337	0.075396193	0.06713	-0.009744006
Inverse	112	0.1535074569	0.083909754	0.06733	-0.010955662
Inverse	112	0.1705378470	0.093311748	0.06760	-0.01235318
Inverse	112	0.0973461989	0.053295211	0.06776	-0.007112415
Inverse	112	0.1545716237	0.084772317	0.06824	-0.011582119
Inverse	112	0.1542032285	0.084761242	0.06887	-0.011928806
Inverse	112	0.1404038845	0.077293968	0.06929	-0.011092294
Inverse	112	0.1444550343	0.079591682	0.06953	-0.011544662
Inverse	112	0.1591481488	0.087862501	0.07008	-0.013062354
Inverse	112	0.1822242987	0.100745387	0.07048	-0.015236661
Inverse	112	0.1508915644	0.083394236	0.07039	-0.012561139
Inverse	112	0.1376281066	0.076310546	0.07130	-0.011940564
Inverse	112	0.1353611291	0.075030692	0.07121	-0.011699028
Inverse	112	0.1626784130	0.090243121	0.07144	-0.014198105
Inverse	112	-0.146416849	0.081283264	0.07165	-0.305732048
Inverse	112	0.1081130572	0.060056775	0.07183	-0.009598223
Inverse	112	0.1732424707	0.096613318	0.07294	-0.016119634
Inverse	112	0.1377690778	0.076833287	0.07295	-0.012824165
Inverse	112	-0.136456773	0.076181980	0.07326	-0.285773455
Inverse	112	0.1646004661	0.092355436	0.07470	-0.01641619
Inverse	112	-0.135694265	0.076180509	0.07487	-0.285008063
Inverse	112	0.1281811650	0.072213862	0.07589	-0.013358006

Inverse \	112	-0.132537748	0.074692840	0.07599	-0.278935716
Inverse \	112	0.1210533234	0.068214649	0.07596	-0.012647389
Inverse \	112	0.1617020734	0.091229680	0.07631	-0.017108101
Inverse \	112	0.1573467079	0.088750786	0.07624	-0.016604833
Inverse \	112	0.1531852147	0.086660951	0.07712	-0.016670249
Inverse \	112	0.1662206365	0.094078830	0.07725	-0.018173872
Inverse \	112	0.1510507672	0.085636897	0.07775	-0.016797553
Inverse \	112	0.1093867926	0.062315948	0.07919	-0.012752467
Inverse \	112	0.1627173927	0.092671078	0.07911	-0.01891792
Inverse \	112	0.1595934234	0.090995690	0.07945	-0.018758131
Inverse \	112	0.1335917414	0.076178581	0.07948	-0.015718279
Inverse \	112	-0.169322369	0.096720991	0.08001	-0.358895513
Inverse \	112	-0.152442476	0.087152425	0.08026	-0.32326123
Inverse \	112	0.1377971779	0.079193534	0.08185	-0.01742215
Inverse \	112	-0.141746554	0.081497163	0.08198	-0.301480993
Inverse \	112	0.1416382936	0.081571957	0.08250	-0.018242743
Inverse \	112	0.2274209178	0.131033443	0.08263	-0.029404632
Inverse \	112	0.1429478508	0.082550652	0.08333	-0.018851428
Inverse \	112	0.1373050675	0.079387605	0.08371	-0.018294639
Inverse \	112	0.1453208220	0.084162060	0.08422	-0.019636816
Inverse \	112	0.1463293008	0.084867216	0.08466	-0.020010444
Inverse \	112	0.1327912155	0.077187209	0.08536	-0.018495715
Inverse \	112	0.1450727096	0.084523434	0.08609	-0.020593222
Inverse \	112	0.1218471236	0.071076343	0.08647	-0.017462509
Inverse \	112	0.1280674841	0.074889324	0.08724	-0.018715591
Inverse \	112	0.1291180897	0.075580639	0.08757	-0.019019964
Inverse \	112	-0.130028698	0.076147994	0.08771	-0.279278767
Inverse \	112	0.1457632992	0.085467430	0.08810	-0.021752865
Inverse \	112	-0.137092471	0.080566325	0.08882	-0.295002469
Inverse \	112	0.1218570949	0.071678760	0.08912	-0.018633276
Inverse \	112	0.1407082047	0.082819939	0.08932	-0.021618876
Inverse \	112	0.1391747875	0.082041378	0.08981	-0.021626315
Inverse \	112	-0.125615879	0.074251501	0.09069	-0.271148821
Inverse \	112	-0.127965882	0.075719960	0.09103	-0.276377003
Inverse \	112	0.0686851705	0.040775313	0.09208	-0.011234445
Inverse \	112	0.1262534607	0.075498630	0.09447	-0.021723856
Inverse \	112	0.1273855555	0.076218862	0.09465	-0.022003414
Inverse \	112	0.1164753672	0.069797606	0.09516	-0.020327942

Inverse v	112	0.15329740684	0.09187265332	0.095199	-0.026772994
Inverse v	112	0.16573436529	0.09948565544	0.095730	-0.029257517
Inverse v	112	0.12485610150	0.07517719400	0.096748	-0.022491199
Inverse v	112	0.12647684353	0.07626155911	0.097224	-0.022995812
Inverse v	112	-0.138153262	0.08345674900	0.097845	-0.30172849
Inverse v	112	-0.151135187	0.09131577022	0.097907	-0.330114096
Inverse v	112	0.12798203723	0.07734560400	0.097989	-0.023615347
Inverse v	112	0.15017878360	0.09096857688	0.098761	-0.028119627
Inverse v	112	0.13101937038	0.07953340544	0.099480	-0.024866104
Inverse v	112	-0.131688819	0.07990817777	0.099352	-0.288308847
Inverse v	112	0.15796771637	0.09586407155	0.099387	-0.029925864
Inverse v	112	-0.128292121	0.07793439300	0.099731	-0.281043531
Inverse v	112	-0.132039981	0.08035784555	0.100351	-0.289541358
Inverse v	112	-0.140082388	0.08547854100	0.101254	-0.307620328
Inverse v	112	-0.134919271	0.08237668299	0.101450	-0.29637757
Inverse v	112	0.12144759432	0.07427175522	0.102011	-0.024125046
Inverse v	112	0.17238489578	0.10532877944	0.101700	-0.034059512
Inverse v	112	0.13885869434	0.08493480800	0.102073	-0.027613529
Inverse v	112	0.12609586973	0.07706555911	0.101794	-0.024952626
Inverse v	112	0.15925970385	0.09747898044	0.102303	-0.031799098
Inverse v	112	0.16384328029	0.10070735177	0.103753	-0.033543129
Inverse v	112	-0.276263377	0.17011460660	0.104379	-0.609688006
Inverse v	112	0.12870634547	0.07932570611	0.104695	-0.026772039
Inverse v	112	0.13369973947	0.08287554855	0.106688	-0.028736336
Inverse v	112	-0.133922688	0.08318626455	0.107410	-0.296967767
Inverse v	112	-0.127793894	0.07945423112	0.107748	-0.283524187
Inverse v	112	0.14466801008	0.09066684344	0.110578	-0.033039003
Inverse v	112	0.13501287075	0.08461455488	0.110573	-0.030831657
Inverse v	112	-0.135606114	0.08504375833	0.110813	-0.30229188
Inverse v	112	0.12841558590	0.08061197933	0.111158	-0.029583894
Inverse v	112	0.12825521118	0.08060331844	0.111560	-0.029727293
Inverse v	112	-0.153267038	0.09634280422	0.111643	-0.342098934
Inverse v	112	0.14522053697	0.09122494260	0.111408	-0.033580351
Inverse v	112	0.12042581167	0.07579408500	0.112093	-0.028130595
Inverse v	112	0.10412940961	0.06553494899	0.112079	-0.02431909
Inverse v	112	-0.147173848	0.09270567344	0.112390	-0.328876968
Inverse v	112	0.12355510851	0.07805392255	0.113433	-0.02943058
Inverse v	112	0.12236544729	0.07732887999	0.113557	-0.029199157

Inverse v	112	-0.119932497	0.0758260553	0.113722	-0.268551565
Inverse v	112	0.1273892783	0.0806774180	0.114331	-0.030738461
Inverse v	112	-0.258127373	0.1635656998	0.114530	-0.578716145
Inverse v	112	0.1185642630	0.0754382451	0.116020	-0.029294697
Inverse v	112	0.1436883750	0.0914866183	0.116270	-0.035625397
Inverse v	112	-0.125163532	0.0797934828	0.116742	-0.281558758
Inverse v	112	-0.132652227	0.0847012404	0.117320	-0.298666658
Inverse v	112	0.1211268274	0.0775897098	0.118495	-0.030949004
Inverse v	112	0.1238087702	0.0796325532	0.120005	-0.032271034
Inverse v	112	0.1298031730	0.0836935012	0.120917	-0.034236089
Inverse v	112	0.1353968735	0.0872449645	0.120682	-0.035603257
Inverse v	112	-0.077087014	0.0496885672	0.120805	-0.174476606
Inverse v	112	0.1329767516	0.0858353559	0.121331	-0.035260546
Inverse v	112	-0.254908765	0.1646980301	0.121680	-0.577716904
Inverse v	112	0.1251428940	0.0808471929	0.121647	-0.033317604
Inverse v	112	-0.118301503	0.0765460723	0.122220	-0.268331805
Inverse v	112	-0.131782568	0.0852217272	0.122020	-0.298817153
Inverse v	112	0.1245327681	0.0805632064	0.122158	-0.033371117
Inverse v	112	0.1210791874	0.0784139189	0.122563	-0.032612094
Inverse v	112	0.1352983606	0.0877338442	0.123038	-0.036659974
Inverse v	112	0.1339289796	0.0872040639	0.124584	-0.036990986
Inverse v	112	-0.111288527	0.0726218441	0.125414	-0.253627342
Inverse v	112	-0.132790974	0.0868094533	0.126095	-0.302937503
Inverse v	112	0.1294758131	0.0846537254	0.126140	-0.036445489
Inverse v	112	-0.100901348	0.0661351379	0.127088	-0.230526219
Inverse v	112	0.1390240174	0.0911105321	0.127038	-0.039552626
Inverse v	112	0.1254345098	0.0826740327	0.129211	-0.036606594
Inverse v	112	0.1197862672	0.0789426189	0.129169	-0.034941266
Inverse v	112	0.1325354215	0.0872873594	0.128918	-0.038547803
Inverse v	112	0.1498258163	0.0991865145	0.130904	-0.044579752
Inverse v	112	0.1202486866	0.0797747974	0.131720	-0.036109916
Inverse v	112	-0.126111162	0.0836475457	0.131644	-0.290060352
Inverse v	112	0.1312712558	0.0874441126	0.133303	-0.040119205
Inverse v	112	-0.110961041	0.0739072330	0.133263	-0.255819218
Inverse v	112	0.1296611802	0.0864016155	0.133438	-0.039685986
Inverse v	112	0.1187422821	0.0793499422	0.134539	-0.036783605
Inverse v	112	-0.108323838	0.0724414034	0.134828	-0.250308989
Inverse v	112	-0.128877327	0.0864340401	0.135948	-0.298288046

Inverse v	112	0.16346944429	0.1096198862	0.135898	-0.051385533
Inverse v	112	0.11814985805	0.0792230597	0.135867	-0.037127339
Inverse v	112	0.13554846032	0.0911356780	0.136928	-0.043077469
Inverse v	112	0.12262991936	0.0825490299	0.137400	-0.039166179
Inverse v	112	0.12391845770	0.0833758774	0.137209	-0.039498262
Inverse v	112	-0.12349074	0.0829959122	0.136773	-0.286162728
Inverse v	112	-0.116322121	0.0782305955	0.137037	-0.269654088
Inverse v	112	0.11227342749	0.0755616357	0.137318	-0.035827379
Inverse v	112	0.11535777248	0.0774726442	0.136483	-0.03648861
Inverse v	112	0.13129341376	0.0884953845	0.137910	-0.04215754
Inverse v	112	0.12245346744	0.0825900503	0.138163	-0.039423031
Inverse v	112	0.11995123839	0.0809684883	0.138485	-0.038746999
Inverse v	112	0.13144256054	0.0887672325	0.138671	-0.042541215
Inverse v	112	-0.134029813	0.0906302442	0.139170	-0.311665092
Inverse v	112	0.13054695857	0.0883210654	0.139382	-0.04256233
Inverse v	112	0.11285744512	0.0764979409	0.140131	-0.037078519
Inverse v	112	0.11897729882	0.0806246580	0.140020	-0.039047031
Inverse v	112	-0.130440091	0.0885518031	0.140741	-0.304001625
Inverse v	112	0.11662788490	0.0791671622	0.140701	-0.038539753
Inverse v	112	-0.089984543	0.0610834316	0.140712	-0.209708069
Inverse v	112	-0.103577975	0.0704853207	0.141698	-0.241729204
Inverse v	112	-0.115928939	0.0788457123	0.141473	-0.270466535
Inverse v	112	0.11736049453	0.0798633235	0.141692	-0.03917162
Inverse v	112	0.12384076000	0.0842865663	0.141750	-0.04136091
Inverse v	112	0.11388879045	0.0776047839	0.142220	-0.038216586
Inverse v	112	-0.108104724	0.0738583548	0.143282	-0.252867099
Inverse v	112	-0.128736434	0.0880284110	0.143620	-0.30127212
Inverse v	112	0.08242975119	0.0564329891	0.144107	-0.028178908
Inverse v	112	-0.105303398	0.0720971715	0.144131	-0.246613855
Inverse v	112	0.11661166788	0.0802329647	0.146109	-0.040644943
Inverse v	112	-0.11401631	0.0788024963	0.147935	-0.268469203
Inverse v	112	0.12006825599	0.0830753931	0.148375	-0.042759515
Inverse v	112	0.11637169929	0.0804658271	0.148114	-0.041341322
Inverse v	112	-0.11849537	0.0820057536	0.148467	-0.279226648
Inverse v	112	0.10078064332	0.0698224310	0.148912	-0.036071322
Inverse v	112	-0.114867284	0.0795975784	0.148992	-0.270878537
Inverse v	112	-0.106717301	0.0740779407	0.149695	-0.251910065
Inverse v	112	0.10560021863	0.0734032337	0.150254	-0.038270119

Inverse v	112	-0.11688787	0.08135921	0.15080	-0.276351937
Inverse v	112	0.122424117	0.08532563	0.15134	-0.044814117
Inverse v	112	0.125023254	0.08717998	0.15154	-0.045849516
Inverse v	112	-0.063954433	0.04465895	0.15212	-0.151485991
Inverse v	112	0.127549700	0.08908747	0.15221	-0.047061755
Inverse v	112	0.125535234	0.08769428	0.15228	-0.046345559
Inverse v	112	0.111215305	0.07800334	0.15393	-0.041671257
Inverse v	112	-0.110388104	0.07740929	0.15385	-0.262110329
Inverse v	112	0.112098670	0.07883664	0.15505	-0.042421158
Inverse v	112	0.121988271	0.08580388	0.15511	-0.046187346
Inverse v	112	0.065292264	0.04615088	0.15713	-0.025163462
Inverse v	112	0.094834164	0.06700553	0.15697	-0.036496679
Inverse v	112	0.117847551	0.08346119	0.15794	-0.045736386
Inverse v	112	-0.111778851	0.07914239	0.15783	-0.266897949
Inverse v	112	-0.163130567	0.11554758	0.15800	-0.389603828
Inverse v	112	0.115088988	0.08151099	0.15796	-0.044672571
Inverse v	112	0.129799732	0.09199636	0.15826	-0.050513137
Inverse v	112	-0.107865263	0.07663016	0.15924	-0.258060381
Inverse v	112	0.112440668	0.07994834	0.15960	-0.04425808
Inverse v	112	-0.10901777	0.07758409	0.15997	-0.261082598
Inverse v	112	-0.115218975	0.08243776	0.16221	-0.27679699
Inverse v	112	0.113225906	0.08115549	0.16296	-0.045838856
Inverse v	112	-0.115983397	0.08309808	0.16279	-0.278855646
Inverse v	112	0.111234527	0.07965998	0.16260	-0.044899043
Inverse v	112	0.111040567	0.07966013	0.16333	-0.0450933
Inverse v	112	-0.110620965	0.07950671	0.16412	-0.266454126
Inverse v	112	-0.116546827	0.08382093	0.16439	-0.28083585
Inverse v	112	0.104345088	0.07519911	0.16526	-0.043045181
Inverse v	112	-0.118414902	0.08530824	0.16511	-0.285619071
Inverse v	112	0.114715663	0.08266596	0.16522	-0.047309628
Inverse v	112	0.122127096	0.08798945	0.16514	-0.050332237
Inverse v	112	0.121756470	0.08807533	0.16684	-0.050871183
Inverse v	112	-0.106590551	0.07713867	0.16703	-0.257782357
Inverse v	112	-0.123285463	0.08920508	0.16695	-0.298127421
Inverse v	112	-0.109664877	0.07950280	0.16777	-0.265490376
Inverse v	112	0.108693956	0.07885710	0.16809	-0.045865971
Inverse v	112	-0.129462867	0.09441884	0.17032	-0.314523795
Inverse v	112	-0.108115079	0.07917212	0.17207	-0.26329245

Inverse v	112	0.1123212190	0.0822659611	0.17214	-0.048920065
Inverse v	112	-0.110484604	0.0809463301	0.17228	-0.269139411
Inverse v	112	0.0714121884	0.0522539126	0.17173	-0.03100548
Inverse v	112	0.1019857046	0.0748484311	0.17302	-0.04471722
Inverse v	112	0.1162646182	0.0853460179	0.17311	-0.051013577
Inverse v	112	-0.103450608	0.0759435660	0.17313	-0.252299997
Inverse v	112	0.1081241269	0.0794575064	0.17358	-0.047612586
Inverse v	112	0.1056620783	0.0776486164	0.17358	-0.04652921
Inverse v	112	0.1056396629	0.0778724572	0.17491	-0.046990353
Inverse v	112	0.0996238363	0.0734396015	0.17492	-0.044317783
Inverse v	112	0.2611403335	0.1924354771	0.17477	-0.116033202
Inverse v	112	-0.109273372	0.0807539847	0.17600	-0.267551182
Inverse v	112	-0.092108967	0.0681943299	0.17679	-0.225769853
Inverse v	112	0.0860126195	0.0637174369	0.17704	-0.038873557
Inverse v	112	0.1055683674	0.0784854189	0.17860	-0.048263054
Inverse v	112	0.1168675883	0.0868772318	0.17855	-0.053411786
Inverse v	112	0.0799234400	0.0594140114	0.17856	-0.036528022
Inverse v	112	-0.073582225	0.0548121378	0.17945	-0.181014015
Inverse v	112	0.0639488816	0.0476855485	0.17990	-0.029514793
Inverse v	112	0.1206500656	0.0901617047	0.18084	-0.056066876
Inverse v	112	-0.114095946	0.0853346819	0.18120	-0.281351923
Inverse v	112	0.1073415986	0.0804372244	0.18204	-0.050315361
Inverse v	112	-0.114149441	0.0855063374	0.18188	-0.281741862
Inverse v	112	0.2904848601	0.2182533111	0.18320	-0.13729163
Inverse v	112	0.1165031411	0.0876935267	0.18400	-0.055376171
Inverse v	112	0.1082514389	0.0815596654	0.18442	-0.051605505
Inverse v	112	0.1010624709	0.0762394955	0.18497	-0.04836694
Inverse v	112	0.1140182050	0.0860210197	0.18501	-0.054582994
Inverse v	112	-0.111463577	0.0841765464	0.18544	-0.276449608
Inverse v	112	-0.11748222	0.0887044519	0.18536	-0.291342946
Inverse v	112	-0.104036268	0.0786183448	0.18573	-0.258128224
Inverse v	112	-0.105325083	0.0797764525	0.18675	-0.26168693
Inverse v	112	-0.105230393	0.0798302956	0.18744	-0.261697773
Inverse v	112	-0.104715721	0.0795554744	0.18808	-0.260644451
Inverse v	112	0.1213966833	0.0927099657	0.19039	-0.06031485
Inverse v	112	-0.093420005	0.0715607754	0.19173	-0.233679124
Inverse v	112	-0.113720702	0.0870574440	0.19146	-0.284353292
Inverse v	112	-0.103194746	0.0790528122	0.19176	-0.258138258

Inverse v	112	-0.10998842	0.084350961	0.19225	-0.275316304
Inverse v	112	0.095762172	0.073497672	0.19259	-0.048293266
Inverse v	112	0.130444734	0.100313313	0.19347	-0.06616936
Inverse v	112	-0.08238003	0.063301120	0.19312	-0.206450227
Inverse v	112	0.111556656	0.085800827	0.19353	-0.056612966
Inverse v	112	0.102312283	0.078775287	0.19401	-0.05208728
Inverse v	112	-0.117495071	0.090937261	0.19634	-0.295732103
Inverse v	112	-0.103335262	0.080161564	0.19736	-0.260451928
Inverse v	112	0.107398169	0.083487856	0.19830	-0.05623803
Inverse v	112	-0.097166225	0.075845572	0.20015	-0.245823547
Inverse v	112	-0.103528769	0.081155904	0.20206	-0.262594341
Inverse v	112	-0.106229314	0.083270932	0.20205	-0.269440341
Inverse v	112	0.101455377	0.079687296	0.20295	-0.054731724
Inverse v	112	-0.092730919	0.072997078	0.20396	-0.235805194
Inverse v	112	0.110053094	0.086554859	0.20355	-0.05959443
Inverse v	112	0.111063721	0.087431391	0.20397	-0.060301806
Inverse v	112	-0.100402311	0.079285194	0.20539	-0.255801293
Inverse v	112	0.097616079	0.077067678	0.20528	-0.053436571
Inverse v	112	0.101564944	0.080365645	0.20630	-0.055951721
Inverse v	112	-0.080186722	0.063493919	0.20662	-0.204634804
Inverse v	112	-0.084491595	0.067039510	0.20755	-0.215889035
Inverse v	112	0.104853657	0.083326460	0.20826	-0.058466205
Inverse v	112	0.077431791	0.061504058	0.20804	-0.043116164
Inverse v	112	-0.092267028	0.073481095	0.20924	-0.236289974
Inverse v	111	-0.104206991	0.083523545	0.21216	-0.267913139
Inverse v	112	0.110733982	0.088858793	0.21269	-0.063429253
Inverse v	112	-0.109877881	0.088228057	0.21299	-0.282804874
Inverse v	112	-0.098305923	0.079211782	0.21458	-0.253561016
Inverse v	112	-0.107770861	0.087231650	0.21666	-0.278744896
Inverse v	112	0.098800099	0.080082985	0.21730	-0.058162552
Inverse v	112	-0.10411197	0.084540938	0.21813	-0.26981221
Inverse v	112	0.121674376	0.098767283	0.21797	-0.071909498
Inverse v	112	-0.097994493	0.079594729	0.21825	-0.254000163
Inverse v	112	0.093128040	0.075887778	0.21975	-0.055612005
Inverse v	112	0.111555123	0.090857505	0.21952	-0.066525587
Inverse v	112	-0.097418332	0.079315438	0.21935	-0.252876592
Inverse v	112	-0.096796914	0.079028326	0.22063	-0.251692434
Inverse v	112	0.107455078	0.087708525	0.22052	-0.064453631

Inverse v	112	-0.093549388	0.076662449	0.22236	-0.243807789
Inverse v	112	-0.097209365	0.079704762	0.22260	-0.253430698
Inverse v	112	0.0990643665	0.081542072	0.22440	-0.060758095
Inverse v	112	0.1198456189	0.098702882	0.22466	-0.07361203
Inverse v	112	0.1175779260	0.096992454	0.22542	-0.072527286
Inverse v	112	-0.096429257	0.079648168	0.22601	-0.252539666
Inverse v	112	-0.11389194	0.094044577	0.22587	-0.298219312
Inverse v	112	0.0836213281	0.069000318	0.22555	-0.051619297
Inverse v	112	0.0793567201	0.065718108	0.22722	-0.049450773
Inverse v	112	0.0849227135	0.070406011	0.22774	-0.053073068
Inverse v	112	0.0912684906	0.075835615	0.22878	-0.057369315
Inverse v	112	0.0980655936	0.081558590	0.22921	-0.061789243
Inverse v	112	0.0761289520	0.063559524	0.23101	-0.048447716
Inverse v	112	-0.096813829	0.080877557	0.23129	-0.255333843
Inverse v	112	0.0857401369	0.071793086	0.23237	-0.054974313
Inverse v	112	0.1247721226	0.104559910	0.23274	-0.080165301
Inverse v	112	-0.10414218	0.087453638	0.23372	-0.275551311
Inverse v	112	0.0923213775	0.077629751	0.23434	-0.059832935
Inverse v	112	0.1104150996	0.093132693	0.23579	-0.07212498
Inverse v	112	-0.100647088	0.084913360	0.23590	-0.267077274
Inverse v	112	0.0942862118	0.079665008	0.23659	-0.061857205
Inverse v	112	-0.10663019	0.090249677	0.23740	-0.283519559
Inverse v	112	0.1017470356	0.086242193	0.23808	-0.067287664
Inverse v	112	0.0919540226	0.078381969	0.24073	-0.061674638
Inverse v	112	-0.091364992	0.077784435	0.24015	-0.243822485
Inverse v	112	0.1086732942	0.092532982	0.24022	-0.072691352
Inverse v	112	0.0938158458	0.079964444	0.24070	-0.062914464
Inverse v	112	-0.109745689	0.093401318	0.23999	-0.292812273
Inverse v	112	0.1069665606	0.091255600	0.24113	-0.071894417
Inverse v	112	0.0906703635	0.077514864	0.24211	-0.061258771
Inverse v	112	0.0967246443	0.082881610	0.24320	-0.065723313
Inverse v	112	0.0936832913	0.080374734	0.24378	-0.063851188
Inverse v	112	0.0975426370	0.083800069	0.24442	-0.066705499
Inverse v	112	0.1041757542	0.090090495	0.24753	-0.072401617
Inverse v	112	0.0915704296	0.079563163	0.24976	-0.064373371
Inverse v	112	0.1016053652	0.088528664	0.25108	-0.071910817
Inverse v	112	0.0990711830	0.086481036	0.25196	-0.070431649
Inverse v	112	0.1156298395	0.100959365	0.25208	-0.082250517

Inverse v	112	0.10122117321	0.08844264332	0.25242	-0.072126407
Inverse v	112	0.08456819937	0.07433567077	0.25526	-0.061129715
Inverse v	112	0.08360338382	0.07387018444	0.25773	-0.061182178
Inverse v	112	0.09694304130	0.08583346059	0.25871	-0.071290541
Inverse v	112	0.08586079534	0.07600236699	0.25859	-0.063103844
Inverse v	112	-0.09090265	0.08062383260	0.25953	-0.248925362
Inverse v	112	0.09995336694	0.08871302799	0.25986	-0.073924168
Inverse v	112	0.09798881799	0.08701399100	0.26011	-0.072558541
Inverse v	112	0.08014841532	0.07129137277	0.26091	-0.059582675
Inverse v	112	0.09397800225	0.08416996544	0.26419	-0.07099513
Inverse v	112	0.09925618769	0.08885382944	0.26396	-0.074897318
Inverse v	112	0.10492057408	0.09401109411	0.26440	-0.07934117
Inverse v	112	0.08797033213	0.07887546900	0.26471	-0.066625587
Inverse v	112	-0.083765668	0.07535166160	0.26628	-0.231454925
Inverse v	112	0.07920772006	0.07137451200	0.26710	-0.060686324
Inverse v	112	0.09397101588	0.08485636288	0.26811	-0.072347455
Inverse v	112	0.08183728463	0.07393488099	0.26834	-0.063075082
Inverse v	112	0.09162474484	0.08287967033	0.26893	-0.070819409
Inverse v	112	-0.080234618	0.07257356888	0.26891	-0.222478813
Inverse v	112	0.12678024291	0.11484018144	0.26960	-0.098306513
Inverse v	112	0.09392236684	0.08511742311	0.26983	-0.072907783
Inverse v	112	0.09167157620	0.08325872355	0.27087	-0.071515522
Inverse v	112	0.08911365161	0.08099927844	0.27125	-0.069644934
Inverse v	112	0.08349988421	0.07599485088	0.27187	-0.065450023
Inverse v	112	0.08513065880	0.07753752022	0.27223	-0.066842881
Inverse v	112	-0.081283758	0.07433669200	0.27419	-0.226983674
Inverse v	112	0.08069033181	0.07384494699	0.27452	-0.064045764
Inverse v	112	0.08732412690	0.08006212622	0.27540	-0.06959764
Inverse v	112	0.07504962454	0.06885224544	0.27570	-0.059900776
Inverse v	112	0.09502834395	0.08723677955	0.27601	-0.075955744
Inverse v	112	0.08790560833	0.08098134344	0.27769	-0.070817825
Inverse v	112	-0.120074649	0.11062413844	0.27773	-0.336897961
Inverse v	112	-0.089779182	0.08276077460	0.27800	-0.251990301
Inverse v	112	-0.087728856	0.08109996334	0.27936	-0.246684785
Inverse v	112	0.09416313187	0.08729274788	0.28071	-0.076930654
Inverse v	112	0.08822591320	0.08180248288	0.28080	-0.072106953
Inverse v	112	0.10602707559	0.09831056333	0.28081	-0.086661629
Inverse v	112	0.07623767298	0.07056410555	0.27996	-0.062067974

Inverse v	112	0.08415545273	0.07845271755	0.283410	-0.069611874
Inverse v	112	0.08622243264	0.08042018577	0.283653	-0.071401131
Inverse v	112	-0.093195841	0.08711751555	0.284722	-0.263946172
Inverse v	112	0.06045267650	0.05657941099	0.285314	-0.050442969
Inverse v	112	-0.091494731	0.08581810200	0.286357	-0.259698211
Inverse v	112	0.07103814964	0.06667349033	0.286660	-0.059641891
Inverse v	112	-0.090385314	0.08516805144	0.288572	-0.257314695
Inverse v	112	0.09802251022	0.09239658577	0.288740	-0.083074798
Inverse v	112	0.07899519251	0.07481619822	0.291033	-0.067644556
Inverse v	112	0.07832758284	0.07419698060	0.291118	-0.067098499
Inverse v	112	0.10456306311	0.09929516001	0.292310	-0.090055451
Inverse v	112	-0.091351184	0.08697154244	0.293553	-0.261815407
Inverse v	112	-0.086383453	0.08238516622	0.294393	-0.247858379
Inverse v	112	0.09608991783	0.09167248664	0.294552	-0.083588156
Inverse v	112	0.10219254681	0.09794837911	0.296795	-0.089786276
Inverse v	112	-0.092853511	0.08918831933	0.297831	-0.267662617
Inverse v	112	-0.095945656	0.09246830911	0.299453	-0.277183542
Inverse v	112	0.08968480397	0.08659189033	0.300333	-0.080035301
Inverse v	112	-0.084602224	0.08168013455	0.300307	-0.244695288
Inverse v	112	-0.085241528	0.08251973988	0.301611	-0.246980218
Inverse v	112	-0.089109768	0.08630140233	0.301818	-0.258260516
Inverse v	112	0.09297909400	0.09004804311	0.301814	-0.083515071
Inverse v	112	0.08471198447	0.08199823533	0.301559	-0.076004557
Inverse v	112	0.08791903059	0.08552463633	0.303951	-0.079709257
Inverse v	112	-0.073251453	0.07132935988	0.304445	-0.213056998
Inverse v	112	-0.105302756	0.10257258422	0.304600	-0.306345021
Inverse v	112	0.08558898749	0.08372173160	0.306637	-0.078505607
Inverse v	112	0.09122312550	0.08940542199	0.307571	-0.084011501
Inverse v	112	0.07907521820	0.07760531377	0.308231	-0.073031197
Inverse v	112	-0.075661996	0.07429099099	0.308462	-0.221272338
Inverse v	112	-0.076971869	0.07611918433	0.311919	-0.226165471
Inverse v	112	0.08268831949	0.08177121955	0.311913	-0.077583271
Inverse v	112	0.09482675735	0.09391025088	0.312610	-0.089237334
Inverse v	112	0.11089902446	0.10981610011	0.312561	-0.104340532
Inverse v	112	0.10824161826	0.10747572699	0.313874	-0.102410807
Inverse v	112	0.07776000746	0.07725344477	0.314147	-0.073656744
Inverse v	112	0.09330472333	0.09315396233	0.316527	-0.089277043
Inverse v	112	0.08773325078	0.08783397522	0.317865	-0.084421341

Inverse v	112	0.0822450762(	0.082218060	0.31715	-0.078902322
Inverse v	112	-0.088383952	0.088452502	0.31768	-0.261750857
Inverse v	112	0.1019056530(	0.102055371	0.31802(	-0.098122874
Inverse v	112	0.0983104781	0.098648159	0.31896	-0.095039915
Inverse v	112	0.0511355029	0.051436366	0.32014	-0.049679776
Inverse v	112	0.0819635353	0.082436398	0.32009	-0.079611806
Inverse v	112	0.1060279056	0.106545436	0.31966(	-0.102801151
Inverse v	112	0.0797839198(	0.080349163	0.32072(	-0.07770044
Inverse v	112	0.0848481625	0.085763934	0.32250	-0.083249149
Inverse v	112	0.1252930620	0.126567075	0.32220(	-0.122778406
Inverse v	112	-0.07870783	0.079648306(	0.32305	-0.23481851
Inverse v	112	-0.080911536	0.081994310	0.32374	-0.241620385
Inverse v	112	0.0814263775	0.082490899	0.32359	-0.080255785
Inverse v	112	0.0699982311	0.071187245	0.32546	-0.069528771
Inverse v	112	-0.082392765	0.083830404	0.32568(	-0.246700357
Inverse v	112	0.0783545911	0.080173836	0.32841(	-0.078786129
Inverse v	112	0.0800381786	0.081878179	0.32830	-0.080443053
Inverse v	112	0.0859421410	0.088042733	0.32899	-0.086621617
Inverse v	112	-0.079473758	0.081493247	0.32945	-0.239200524
Inverse v	112	-0.077660396	0.079708177	0.32990	-0.233888424
Inverse v	112	-0.076065456	0.078460506	0.33230	-0.229848048
Inverse v	112	-0.075751291	0.078204148	0.33272	-0.229031421
Inverse v	112	0.0772241167	0.079796683	0.33316	-0.079177384
Inverse v	112	0.0896950024	0.093119686	0.33543	-0.092819583
Inverse v	112	0.0838272095	0.087018377	0.33538	-0.086728811
Inverse v	112	0.0784884861(	0.081835939	0.33751(	-0.081909956
Inverse v	112	-0.105623931	0.110229869(	0.33795	-0.321674474
Inverse v	112	-0.096626083	0.100889418	0.33819	-0.294369344
Inverse v	112	-0.076500837	0.079581498	0.33640(	-0.232480574
Inverse v	112	-0.080290236	0.083815069	0.33809(	-0.244567772
Inverse v	112	-0.085081531	0.088596316	0.33689(	-0.258730312
Inverse v	112	0.0754200286	0.078667234	0.33769	-0.078767751
Inverse v	112	-0.076776066	0.080029816(	0.33738	-0.233634506
Inverse v	112	-0.07413503	0.077578791	0.33926	-0.226189462
Inverse v	112	0.0790846116(	0.082741935(	0.33917	-0.083089581
Inverse v	112	-0.07750651	0.081309944(	0.34047	-0.236874001
Inverse v	112	0.0805887614	0.084560736	0.34057	-0.085150283
Inverse v	112	-0.074053751	0.077680369(	0.34043	-0.226307276

Inverse v	112	0.07496911555	0.0785243942	0.339717	-0.078938697
Inverse v	112	0.0756598317	0.0795768388	0.341717	-0.080310772
Inverse v	112	0.0786104939	0.0827888473	0.342351	-0.083655647
Inverse v	112	0.0746184571	0.0786311375	0.342630	-0.079498572
Inverse v	112	-0.051346392	0.0541314020	0.342849	-0.157443941
Inverse v	112	-0.076401295	0.0806579802	0.343523	-0.234490936
Inverse v	112	0.0783601116	0.0829480383	0.344817	-0.084218044
Inverse v	112	-0.082057119	0.0871568524	0.346454	-0.25288455
Inverse v	112	0.0746205657	0.0795378647	0.348153	-0.081273649
Inverse v	112	0.0781367483	0.0833186375	0.348343	-0.085167781
Inverse v	112	-0.077441869	0.0828562424	0.349960	-0.239840104
Inverse v	112	0.0844735696	0.0903458597	0.349787	-0.092604315
Inverse v	112	0.0760361331	0.0816510678	0.351733	-0.08399996
Inverse v	112	-0.078816487	0.0849763186	0.353661	-0.245370071
Inverse v	112	0.0803332889	0.0866854695	0.354071	-0.089570231
Inverse v	112	0.0737202559	0.07964152	0.354627	-0.08237713
Inverse v	112	0.0739963303	0.0800948444	0.355559	-0.082989565
Inverse v	112	-0.073451351	0.0795868927	0.356055	-0.229441661
Inverse v	112	0.0850242236	0.0920638728	0.355728	-0.095420967
Inverse v	112	-0.076555052	0.0830685014	0.356742	-0.239369315
Inverse v	112	-0.073598324	0.0799359813	0.357198	-0.230272847
Inverse v	112	-0.075842489	0.0825297072	0.358110	-0.237600715
Inverse v	112	0.0728959123	0.0797625221	0.360763	-0.083438631
Inverse v	112	0.0793534806	0.0867919101	0.360561	-0.090758663
Inverse v	112	-0.075130831	0.0824256378	0.362032	-0.236685081
Inverse v	112	0.0760277272	0.0837218718	0.363820	-0.088067142
Inverse v	112	0.0748199018	0.0824167249	0.363971	-0.086716879
Inverse v	112	0.1026499742	0.1134137399	0.365410	-0.119640956
Inverse v	112	0.0723598812	0.0800149908	0.365820	-0.084469501
Inverse v	112	0.0746009458	0.0827725985	0.367441	-0.087633347
Inverse v	112	-0.073594017	0.0818128498	0.368364	-0.233947203
Inverse v	112	0.0716152345	0.0795870627	0.368207	-0.084375408
Inverse v	112	-0.051194755	0.0570777141	0.369755	-0.163067074
Inverse v	112	-0.073465668	0.0818966705	0.369690	-0.233983142
Inverse v	112	0.0692462041	0.0771616959	0.369490	-0.08199072
Inverse v	112	0.0761322297	0.0849263720	0.370012	-0.090323459
Inverse v	112	-0.072583446	0.0810467611	0.370479	-0.231435098
Inverse v	112	-0.074261714	0.0830136111	0.371015	-0.236968392

Inverse \	112	-0.080209783	0.0899240666	0.372407	-0.256460953
Inverse \	112	0.0824802959	0.0930598596	0.375448	-0.099917029
Inverse \	112	-0.068868435	0.0776046476	0.374849	-0.220973545
Inverse \	112	0.0741282679	0.0835881518	0.375171	-0.08970451
Inverse \	112	-0.075218742	0.0846944530	0.374470	-0.24121987
Inverse \	112	0.0685309204	0.0770781294	0.373944	-0.082542213
Inverse \	112	-0.088738361	0.0997518140	0.373685	-0.284251917
Inverse \	112	0.0620122768	0.0699877472	0.375593	-0.075163708
Inverse \	112	0.0738697524	0.0830488880	0.373748	-0.088906068
Inverse \	112	0.0775559767	0.0876747545	0.376379	-0.094286542
Inverse \	112	0.0689307800	0.0779224881	0.376368	-0.083797297
Inverse \	112	0.0831804975	0.0941100985	0.376769	-0.101275296
Inverse \	112	0.0741043716	0.0840743016	0.378093	-0.09068126
Inverse \	102	-0.077697653	0.0881691909	0.378191	-0.250509267
Inverse \	112	0.0683723839	0.0778908179	0.380053	-0.084293619
Inverse \	112	-0.072862993	0.0830296920	0.380180	-0.235601189
Inverse \	112	0.0809839651	0.0921981291	0.379743	-0.099724368
Inverse \	112	-0.07056439	0.0805387273	0.380945	-0.228420296
Inverse \	112	-0.070700989	0.0807215705	0.381104	-0.228915268
Inverse \	112	-0.075392356	0.0863971151	0.382867	-0.244730702
Inverse \	112	0.0706195370	0.0808775195	0.382572	-0.087900401
Inverse \	112	0.0695452561	0.0796051194	0.382320	-0.086480778
Inverse \	112	0.0661650933	0.0760370110	0.384207	-0.082867448
Inverse \	112	0.0475495148	0.0549463893	0.386830	-0.060145408
Inverse \	112	-0.063649987	0.0735919857	0.387091	-0.207890279
Inverse \	112	0.0726104294	0.0842302837	0.388661	-0.092480927
Inverse \	112	0.0741484954	0.0859688357	0.388410	-0.094350423
Inverse \	112	-0.074091757	0.0861522477	0.389783	-0.242950163
Inverse \	112	-0.044141497	0.0514445411	0.390870	-0.144972798
Inverse \	112	0.0758057206	0.0885842279	0.392137	-0.097819366
Inverse \	112	-0.066549095	0.0779598671	0.393308	-0.219350435
Inverse \	112	-0.07039337	0.0827718416	0.395074	-0.23262618
Inverse \	112	0.0746708502	0.0882941050	0.397710	-0.098385596
Inverse \	112	-0.069018265	0.0816543112	0.397971	-0.229060715
Inverse \	112	-0.068339867	0.0807603693	0.397438	-0.226630191
Inverse \	112	0.0671350335	0.0795003440	0.398411	-0.088685641
Inverse \	112	-0.076966427	0.0912268446	0.398847	-0.255771042
Inverse \	112	0.0670617433	0.0795884943	0.399448	-0.088931706

Inverse v	112	0.06190257471	0.07359315998	0.400260	-0.082340019
Inverse v	112	0.06733528344	0.08023689337	0.401353	-0.089929028
Inverse v	112	0.07570729372	0.09044577881	0.402560	-0.101566431
Inverse v	112	-0.066937577	0.08024247944	0.404173	-0.224212837
Inverse v	112	-0.066765053	0.08038075922	0.406194	-0.224311342
Inverse v	112	0.06460246969	0.07787717022	0.406797	-0.088036784
Inverse v	112	-0.069156682	0.08362209060	0.408228	-0.233055979
Inverse v	112	0.06134170268	0.07438073844	0.409542	-0.084444545
Inverse v	112	0.06610951718	0.08016393460	0.409553	-0.091011795
Inverse v	112	0.07932514427	0.09670974860	0.412079	-0.110225963
Inverse v	112	-0.068135891	0.08300834960	0.411742	-0.230832256
Inverse v	112	0.08036541864	0.09817957337	0.413040	-0.112066546
Inverse v	112	0.06507527115	0.07972319500	0.414348	-0.091182191
Inverse v	112	-0.05939665	0.07295525800	0.415558	-0.202388956
Inverse v	112	-0.05599786	0.06897012008	0.416840	-0.191179297
Inverse v	112	0.06880288220	0.08486365311	0.417511	-0.097529878
Inverse v	112	0.07016582585	0.08645222460	0.417013	-0.099280534
Inverse v	112	0.07137532044	0.08797763224	0.417199	-0.101060839
Inverse v	112	-0.063906172	0.07879903558	0.417364	-0.218352282
Inverse v	112	0.06115001218	0.07621492227	0.422358	-0.088231236
Inverse v	112	-0.067052042	0.08387656200	0.424050	-0.231450104
Inverse v	112	-0.065495498	0.08188051360	0.423773	-0.225981304
Inverse v	112	0.06790629311	0.08495394633	0.424098	-0.098603442
Inverse v	112	-0.067366539	0.08431866000	0.424318	-0.232631113
Inverse v	112	0.06271040499	0.07861546833	0.425053	-0.091375913
Inverse v	112	0.06049190101	0.07592971260	0.425635	-0.088330336
Inverse v	112	-0.068059459	0.08575610877	0.427405	-0.236141432
Inverse v	112	-0.075416334	0.09502759799	0.427413	-0.261670426
Inverse v	112	0.04825599762	0.06100675760	0.428947	-0.071317247
Inverse v	112	0.06262389652	0.07917788022	0.428980	-0.092564749
Inverse v	112	0.06694050265	0.08483128177	0.430052	-0.09932881
Inverse v	112	-0.068051781	0.08650260633	0.431450	-0.23759689
Inverse v	112	0.07282517053	0.09269384060	0.432070	-0.108854757
Inverse v	112	0.06180906245	0.07905934800	0.434328	-0.09314726
Inverse v	112	0.07228284544	0.09256286655	0.434858	-0.109140373
Inverse v	112	0.06775595745	0.08659050544	0.433928	-0.101961433
Inverse v	112	0.03268854877	0.04184248111	0.434667	-0.049322714
Inverse v	112	-0.102936855	0.13220636337	0.436211	-0.362061328

Inverse v	112	0.0640709225	0.082555724	0.43769	-0.097738297
Inverse v	112	-0.074821838	0.096311965	0.43723	-0.263593291
Inverse v	112	0.0591180410	0.076222333	0.43798	-0.090277732
Inverse v	112	0.0613360166	0.079131836	0.43827	-0.093762382
Inverse v	112	0.0612979936	0.079194669	0.43892	-0.093923559
Inverse v	112	-0.071792514	0.092835514	0.43932	-0.253750123
Inverse v	112	-0.067198433	0.087050101	0.44014	-0.237816633
Inverse v	112	-0.068095206	0.088255954	0.44037	-0.241076875
Inverse v	112	-0.065500961	0.085386189	0.44301	-0.232857892
Inverse v	112	0.0642025423	0.083533917	0.44214	-0.099523937
Inverse v	112	0.0609604573	0.079476172	0.44306	-0.09481284
Inverse v	112	-0.063951053	0.083340740	0.44287	-0.227298905
Inverse v	112	0.0697817934	0.091302338	0.44469	-0.109170791
Inverse v	112	0.0614938187	0.080582680	0.44539	-0.096448236
Inverse v	112	0.0597290345	0.078497154	0.44671	-0.094125389
Inverse v	112	0.0628701789	0.082800916	0.44767	-0.099419617
Inverse v	112	-0.061823369	0.081476099	0.44797	-0.221516524
Inverse v	112	0.0599915213	0.079269163	0.44916	-0.095376039
Inverse v	112	0.0496988074	0.065654839	0.44906	-0.078984677
Inverse v	112	0.0599624310	0.079523939	0.45083	-0.095904491
Inverse v	112	0.0606469444	0.080212721	0.44960	-0.096569989
Inverse v	112	-0.060030951	0.079550147	0.45047	-0.21594924
Inverse v	112	0.0556547487	0.073851456	0.45108	-0.089094107
Inverse v	112	0.0581538022	0.077072493	0.45052	-0.092908285
Inverse v	112	0.0622943447	0.082910319	0.45244	-0.100209881
Inverse v	112	-0.063635578	0.085018581	0.45416	-0.230271998
Inverse v	112	-0.058781657	0.078476609	0.45383	-0.212595811
Inverse v	112	0.0625388715	0.084265202	0.45798	-0.102620925
Inverse v	112	0.0634364119	0.085606027	0.45867	-0.104351401
Inverse v	112	0.0732397605	0.099084161	0.45980	-0.120965195
Inverse v	112	-0.061142149	0.082713530	0.45978	-0.223260669
Inverse v	112	0.0576658834	0.078363772	0.46180	-0.09592711
Inverse v	112	-0.061298361	0.083460639	0.46266	-0.224881214
Inverse v	112	0.0570731919	0.077877978	0.46364	-0.095567645
Inverse v	112	-0.057726129	0.078973431	0.46480	-0.212514055
Inverse v	112	0.0570466726	0.078081100	0.46501	-0.095992285
Inverse v	112	-0.058164042	0.079634863	0.46515	-0.214248373
Inverse v	112	0.0697599859	0.095668747	0.46588	-0.117750759

Inverse v	112	0.0634544407	0.0870467488	0.466020	-0.107157187
Inverse v	112	0.0643241778	0.0883552142	0.46660	-0.108852042
Inverse v	112	-0.063115289	0.0868085132	0.46718	-0.233259975
Inverse v	112	0.0657591458	0.0907106099	0.46849	-0.11203365
Inverse v	112	0.0661987038	0.0917982360	0.47082	-0.11372584
Inverse v	112	-0.063966096	0.0887826300	0.471229	-0.237980051
Inverse v	112	-0.054982644	0.0766797821	0.473348	-0.205275017
Inverse v	112	0.0669040865	0.0934854702	0.474199	-0.116327435
Inverse v	112	-0.064966441	0.0910729312	0.47563	-0.243469387
Inverse v	112	0.0618565769	0.0871459275	0.47782	-0.108949441
Inverse v	112	0.0692665775	0.0979764685	0.47958	-0.122767301
Inverse v	112	0.0560883981	0.0795622251	0.48083	-0.099853563
Inverse v	112	-0.059113259	0.0840094003	0.481650	-0.223771684
Inverse v	112	-0.062456923	0.0890306673	0.48297	-0.236957031
Inverse v	112	0.0557297427	0.0795835121	0.483760	-0.100253941
Inverse v	112	-0.05834829	0.0835170704	0.48477	-0.222041748
Inverse v	112	-0.052049189	0.0749203828	0.487220	-0.198893139
Inverse v	112	0.0552306207	0.0796289886	0.48793	-0.100842197
Inverse v	112	0.0517302240	0.0746638404	0.488408	-0.094610903
Inverse v	112	0.0555568413	0.0802694986	0.488850	-0.101771376
Inverse v	112	-0.056553496	0.0813532768	0.48695	-0.216005918
Inverse v	112	-0.05703156	0.0826327155	0.490080	-0.218991682
Inverse v	112	0.0559862979	0.0810716314	0.489830	-0.1029141
Inverse v	112	-0.057151976	0.0826193837	0.48909	-0.219085968
Inverse v	112	0.0424920252	0.0611101141	0.48684	-0.077283799
Inverse v	112	0.0600601997	0.0865392269	0.48766	-0.109556685
Inverse v	112	-0.058593091	0.0847850009	0.489510	-0.224771693
Inverse v	112	0.0521497075	0.0754895541	0.489678	-0.095809819
Inverse v	112	0.0700248885	0.1016642690	0.490958	-0.12923708
Inverse v	112	-0.056243982	0.0817923775	0.49167	-0.216557042
Inverse v	112	0.0562660719	0.0818346690	0.49173	-0.104129879
Inverse v	112	-0.057067136	0.0830963475	0.49223	-0.219935977
Inverse v	112	0.0559595916	0.0817831858	0.49382	-0.104335453
Inverse v	112	-0.054161429	0.0791928242	0.494020	-0.209379365
Inverse v	112	0.0578567462	0.0844965647	0.493518	-0.107756521
Inverse v	112	-0.059504229	0.0873289697	0.495630	-0.23066901
Inverse v	112	0.0579967682	0.0852369204	0.496239	-0.109067596
Inverse v	112	0.0428145223	0.0629893712	0.49668	-0.080644645

Inverse v	112	0.0649354398	0.095629296	0.49711	-0.122497982
Inverse v	112	-0.055242726	0.081765353	0.49927	-0.21550282
Inverse v	112	0.0579979107	0.085792015	0.49902	-0.110154441
Inverse v	112	0.0558930461	0.083315082	0.50230	-0.107404515
Inverse v	112	-0.057130522	0.085403477	0.50352	-0.224521338
Inverse v	112	0.0500553025	0.075156244	0.50540	-0.097250936
Inverse v	112	-0.054882279	0.082738376	0.50712	-0.217049497
Inverse v	112	0.0603407297	0.091334922	0.50883	-0.118675718
Inverse v	112	-0.053728222	0.081596908	0.51024	-0.213658164
Inverse v	112	0.0491026984	0.074263050	0.50848	-0.09645288
Inverse v	112	-0.055993619	0.085201195	0.51105	-0.222987962
Inverse v	112	0.0457068728	0.069165950	0.50872	-0.089858389
Inverse v	112	-0.046648304	0.070812926	0.51005	-0.185441639
Inverse v	112	0.0541115823	0.082208792	0.51039	-0.107017651
Inverse v	112	-0.05407894	0.082275445	0.51099	-0.215338812
Inverse v	112	-0.05187383	0.078814558	0.51042	-0.206350365
Inverse v	112	0.0398714082	0.060912276	0.51274	-0.079516653
Inverse v	112	-0.056557836	0.086579505	0.51359	-0.226253666
Inverse v	112	-0.047549928	0.073222115	0.51608	-0.191065275
Inverse v	112	0.0489099176	0.075659251	0.51798	-0.099382215
Inverse v	112	0.0509488412	0.079069934	0.51934	-0.10402823
Inverse v	112	-0.057739066	0.089755960	0.52003	-0.233660748
Inverse v	112	-0.051075884	0.079767504	0.52197	-0.207420193
Inverse v	112	-0.045302563	0.070840608	0.52249	-0.184150154
Inverse v	112	-0.047316016	0.074422314	0.52492	-0.193183753
Inverse v	112	0.0499531643	0.078688331	0.52554	-0.104275966
Inverse v	112	0.0493104031	0.077864631	0.52654	-0.103304274
Inverse v	112	0.0509217984	0.080565547	0.52735	-0.106986674
Inverse v	112	-0.051756718	0.082775473	0.53179	-0.213996647
Inverse v	112	-0.05941242	0.095359271	0.53325	-0.246316592
Inverse v	112	-0.051548356	0.082393816	0.53155	-0.213040236
Inverse v	112	0.0503077424	0.080732313	0.53319	-0.107927591
Inverse v	112	0.0496718257	0.079226867	0.53068	-0.105612835
Inverse v	112	0.0504040343	0.080795150	0.53272	-0.107954461
Inverse v	112	0.0499127099	0.080097208	0.53318	-0.107077819
Inverse v	112	0.0515624196	0.082337277	0.53116	-0.109818644
Inverse v	112	-0.054358649	0.087755133	0.53562	-0.226358711
Inverse v	112	-0.054804615	0.088451741	0.53552	-0.228170029

Inverse v	112	0.0501584225	0.080899875	0.53525	-0.108405334
Inverse v	112	-0.055198218	0.089259205	0.53630	-0.230146261
Inverse v	112	0.0560531536	0.091071138	0.53823	-0.122446277
Inverse v	112	0.0550181280	0.089448768	0.53850	-0.120301459
Inverse v	112	0.0599566565	0.097336438	0.53791	-0.130822762
Inverse v	112	-0.060429963	0.098616974	0.54002	-0.253719233
Inverse v	112	-0.051583908	0.084155377	0.53990	-0.216528447
Inverse v	112	0.0503030062	0.081945679	0.53930	-0.110310526
Inverse v	112	0.0514803368	0.084380931	0.54179	-0.113906289
Inverse v	112	-0.052308245	0.085906467	0.54259	-0.220684921
Inverse v	112	0.0480521169	0.079006533	0.54305	-0.106800689
Inverse v	112	0.0490109375	0.080774106	0.54400	-0.109306312
Inverse v	112	0.0558601691	0.092990409	0.54803	-0.126401034
Inverse v	112	-0.05087449	0.084684838	0.54800	-0.216856772
Inverse v	112	0.0480902407	0.080155881	0.54853	-0.109015288
Inverse v	112	-0.056975682	0.095169779	0.54939	-0.24350845
Inverse v	112	-0.029178862	0.048697527	0.54904	-0.124626015
Inverse v	112	-0.049050035	0.082062390	0.55002	-0.209892321
Inverse v	112	0.0442912060	0.074539504	0.55237	-0.101806223
Inverse v	112	0.0510231712	0.085577216	0.55102	-0.116708173
Inverse v	112	-0.045878527	0.077123254	0.55192	-0.197040105
Inverse v	112	-0.046599044	0.078503723	0.55278	-0.200466343
Inverse v	112	-0.049444879	0.083227236	0.55244	-0.212570261
Inverse v	112	-0.047183961	0.079427371	0.55247	-0.20286161
Inverse v	112	0.0480618831	0.081597146	0.55585	-0.111868524
Inverse v	112	0.0495620806	0.084522060	0.55761	-0.116101157
Inverse v	112	0.0495575807	0.085199615	0.56079	-0.117433666
Inverse v	112	0.0825143491	0.141995489	0.56116	-0.195796811
Inverse v	112	0.0444479110	0.076323001	0.56032	-0.105145171
Inverse v	112	0.0492959191	0.085151027	0.56264	-0.117600095
Inverse v	112	0.0456996851	0.079086995	0.56337	-0.109310827
Inverse v	112	-0.044093628	0.076577505	0.56474	-0.194185538
Inverse v	112	0.0520180620	0.090467180	0.56529	-0.125297612
Inverse v	112	0.0467540158	0.081490576	0.56614	-0.112967514
Inverse v	112	0.0531586546	0.092758994	0.56658	-0.128648975
Inverse v	112	0.0508655210	0.088852500	0.56700	-0.12328538
Inverse v	112	-0.048332715	0.084579903	0.56769	-0.214109326
Inverse v	112	-0.049856789	0.087380431	0.56829	-0.221122435

Inverse	112	-0.045518814	0.080062244	0.56966	-0.202440814
Inverse	112	0.053901475	0.094950082	0.57025	-0.132200687
Inverse	112	-0.04921469	0.086529864	0.56951	-0.218813224
Inverse	112	0.042757655	0.075288923	0.57009	-0.104808634
Inverse	112	-0.044674059	0.079122599	0.57233	-0.199754353
Inverse	112	-0.049068964	0.086876245	0.57219	-0.219346405
Inverse	112	0.044945944	0.079560721	0.57212	-0.11099307
Inverse	112	0.046192203	0.081561408	0.57115	-0.113668157
Inverse	112	0.046907946	0.083209488	0.57293	-0.116182652
Inverse	112	-0.043720772	0.077924980	0.57475	-0.196453733
Inverse	112	0.056430924	0.100482666	0.57438	-0.140515101
Inverse	112	0.049154381	0.087781720	0.57550	-0.12289779
Inverse	112	-0.040396688	0.072435191	0.57705	-0.182369662
Inverse	112	0.040941424	0.073994971	0.58005	-0.104088719
Inverse	112	0.038059844	0.068685217	0.57949	-0.096563181
Inverse	112	0.045590856	0.082313452	0.57966	-0.115743511
Inverse	112	-0.046744184	0.084251354	0.57901	-0.211876838
Inverse	112	-0.046273738	0.083765571	0.58066	-0.210454258
Inverse	112	0.043895201	0.079587655	0.58126	-0.112096602
Inverse	112	0.044057198	0.080211083	0.58282	-0.113156525
Inverse	112	0.042741107	0.077775621	0.58263	-0.109699111
Inverse	112	-0.050670198	0.092992724	0.58583	-0.232935938
Inverse	112	-0.054590426	0.100241644	0.58603	-0.251064048
Inverse	112	0.046707876	0.085992623	0.58701	-0.121837665
Inverse	112	-0.042766699	0.079518810	0.59070	-0.198623568
Inverse	112	-0.046248085	0.085940388	0.59047	-0.214691246
Inverse	112	0.042733068	0.079560960	0.59119	-0.113206414
Inverse	112	0.041076505	0.076649688	0.59202	-0.109156884
Inverse	112	0.043464176	0.081194538	0.59243	-0.115677119
Inverse	112	0.047783183	0.089760919	0.59449	-0.128148218
Inverse	112	0.045315766	0.085046508	0.59414	-0.121375391
Inverse	112	0.044524159	0.084204632	0.59697	-0.12051692
Inverse	112	-0.047148487	0.089480315	0.59825	-0.222529905
Inverse	112	-0.040833369	0.077647654	0.59897	-0.193022773
Inverse	112	0.042433595	0.081623731	0.60315	-0.117548919
Inverse	112	0.044777316	0.086665705	0.60538	-0.125087468
Inverse	112	0.037862715	0.073367529	0.60580	-0.105937642
Inverse	112	-0.04088689	0.079262459	0.60596	-0.196241311

Inverse v	112	0.04110221291	0.07981284983	0.60656	-0.115330973
Inverse v	112	-0.044222749	0.0857738786	0.60615	-0.212339551
Inverse v	112	0.03765935122	0.0738161136	0.60992	-0.107020231
Inverse v	112	0.0424467734	0.0829825398	0.60899	-0.120199005
Inverse v	112	0.0410510368	0.0802922703	0.60916	-0.116321813
Inverse v	112	0.0389050553	0.0759814826	0.60862	-0.110018651
Inverse v	112	0.0380195851	0.0744281206	0.60947	-0.107859531
Inverse v	112	-0.04626871	0.0906400726	0.60972	-0.223923253
Inverse v	112	0.0395208191	0.0777716542	0.61133	-0.112911623
Inverse v	112	-0.042173928	0.0831015202	0.61180	-0.205052907
Inverse v	112	-0.040878885	0.0807419188	0.61265	-0.199133046
Inverse v	112	-0.039220095	0.0783941110	0.61686	-0.192872553
Inverse v	112	0.0409687752	0.0818727190	0.61679	-0.119501754
Inverse v	112	0.0422443426	0.0840857473	0.61538	-0.122563722
Inverse v	112	-0.047021078	0.0938714728	0.61643	-0.231009165
Inverse v	112	0.0350498422	0.0698308401	0.61572	-0.101818604
Inverse v	112	0.0381224814	0.0756615812	0.61436	-0.110174218
Inverse v	112	0.0394315820	0.0788277111	0.61691	-0.115070733
Inverse v	112	0.0394561973	0.0786626376	0.61595	-0.114722572
Inverse v	112	-0.048252301	0.0972232737	0.61967	-0.238809918
Inverse v	112	-0.045887127	0.0931576188	0.62231	-0.22847606
Inverse v	112	0.0403142101	0.0824865066	0.62502	-0.121359343
Inverse v	112	0.0327863156	0.0669344322	0.62425	-0.098405172
Inverse v	112	-0.039747557	0.0812157508	0.62455	-0.198930429
Inverse v	112	0.0392692783	0.0804651203	0.62552	-0.118442358
Inverse v	112	0.0441442930	0.0903893560	0.62528	-0.133018845
Inverse v	112	0.0366601920	0.0753314439	0.62650	-0.110989438
Inverse v	112	0.0410443603	0.0844480723	0.62694	-0.124473861
Inverse v	112	-0.040514008	0.0839672776	0.62945	-0.205089872
Inverse v	112	-0.041896458	0.0870487350	0.63030	-0.212511979
Inverse v	112	-0.041438405	0.0867371379	0.63283	-0.211443196
Inverse v	112	0.0390110268	0.0816104163	0.63263	-0.120945389
Inverse v	112	0.0371593503	0.0775880152	0.63198	-0.114913159
Inverse v	112	0.0287080410	0.0604550617	0.63488	-0.08978388
Inverse v	112	-0.037495326	0.0796507975	0.63782	-0.193610889
Inverse v	112	-0.038909701	0.0827000466	0.63800	-0.201001793
Inverse v	112	-0.041162366	0.0876220119	0.63851	-0.212901509
Inverse v	112	0.0377350689	0.0805811522	0.63957	-0.120203989

Inverse v	112	0.0370535756	0.079121318	0.63956	-0.118024209
Inverse v	112	-0.040191952	0.085922443	0.63994	-0.208599941
Inverse v	112	-0.038392794	0.082509782	0.64170	-0.200111968
Inverse v	112	-0.035916133	0.077378662	0.64253	-0.187578311
Inverse v	112	0.0345480033	0.074923980	0.64472	-0.112302999
Inverse v	112	0.0419862142	0.091241296	0.64539	-0.136846726
Inverse v	112	-0.039316901	0.085999059	0.64754	-0.207875057
Inverse v	112	-0.04669312	0.101901125	0.64679	-0.246419326
Inverse v	112	0.0370562477	0.080990599	0.64728	-0.121685327
Inverse v	112	-0.036046313	0.079557699	0.65048	-0.191979404
Inverse v	112	-0.035621226	0.078684239	0.65075	-0.189842336
Inverse v	112	-0.036315339	0.080680333	0.65262	-0.194448793
Inverse v	112	-0.036621973	0.081190650	0.65194	-0.195755649
Inverse v	112	0.0230774630	0.051201896	0.65219	-0.077278255
Inverse v	112	-0.035721965	0.079322883	0.65246	-0.191194818
Inverse v	112	-0.033620198	0.074891150	0.65348	-0.180406854
Inverse v	112	-0.052319291	0.117092872	0.65500	-0.28182132
Inverse v	112	0.0410786221	0.092096595	0.65556	-0.139430704
Inverse v	112	0.0354215328	0.079755521	0.65695	-0.120899289
Inverse v	112	-0.034482104	0.077835880	0.65775	-0.187040429
Inverse v	112	0.0358817738	0.081418055	0.65942	-0.123697615
Inverse v	112	0.0366130316	0.083322516	0.66036	-0.1266991
Inverse v	112	-0.037172389	0.084859768	0.66135	-0.203497536
Inverse v	112	0.0373638749	0.085307267	0.66139	-0.12983837
Inverse v	112	0.0335915997	0.076719450	0.66149	-0.116778523
Inverse v	112	-0.034291783	0.078267871	0.66128	-0.18769681
Inverse v	112	0.0352615262	0.082097036	0.66755	-0.125648666
Inverse v	112	0.0402770780	0.094207206	0.66898	-0.144369047
Inverse v	112	-0.036520552	0.085699322	0.67000	-0.204491223
Inverse v	112	0.0353014233	0.083256119	0.67155	-0.127880571
Inverse v	112	0.0354631277	0.083669457	0.67167	-0.128529008
Inverse v	112	0.0338254263	0.080887055	0.67581	-0.124713203
Inverse v	112	0.0369334408	0.088394834	0.67607	-0.136320434
Inverse v	112	0.0333387001	0.079711460	0.67577	-0.122895763
Inverse v	112	0.0400821603	0.096273837	0.67716	-0.148614561
Inverse v	112	0.0244371383	0.058622363	0.67678	-0.090462694
Inverse v	112	0.0312929695	0.075455450	0.67834	-0.116599713
Inverse v	112	0.0321435955	0.077461993	0.67817	-0.119681912

Inverse v	112	0.03741396595	0.0905687388	0.679533	-0.140100762
Inverse v	112	0.03038931967	0.0735445587	0.679453	-0.113758015
Inverse v	112	0.03329880100	0.0809145393	0.680683	-0.125293696
Inverse v	112	-0.033311244	0.0812110937	0.681673	-0.192484987
Inverse v	112	0.03219253709	0.0786727971	0.682390	-0.122006145
Inverse v	112	-0.034751482	0.0853362249	0.683838	-0.202010483
Inverse v	112	-0.03167509	0.0782340693	0.685568	-0.185013866
Inverse v	112	0.03409561748	0.0841595314	0.685381	-0.130857064
Inverse v	112	0.04000629605	0.0985266277	0.684709	-0.153105894
Inverse v	112	0.03002565005	0.0748296589	0.688233	-0.116640481
Inverse v	112	0.03270652003	0.0813541564	0.687664	-0.126747627
Inverse v	112	-0.032941125	0.0823773585	0.689244	-0.194400747
Inverse v	112	-0.023535356	0.0588818073	0.689373	-0.138943698
Inverse v	112	-0.03333513	0.0830317755	0.688070	-0.19607741
Inverse v	112	0.03169140934	0.0793092215	0.689450	-0.123754665
Inverse v	112	-0.02925629	0.0731201278	0.689073	-0.17257174
Inverse v	112	0.03229012262	0.0803052483	0.687610	-0.125108164
Inverse v	112	0.03469906843	0.0873846140	0.691305	-0.136574775
Inverse v	112	-0.031388468	0.0791048004	0.691517	-0.186433877
Inverse v	112	-0.034449096	0.0867023464	0.691127	-0.204385694
Inverse v	112	0.03171633178	0.0801498130	0.692310	-0.125377302
Inverse v	112	0.03263409109	0.0818144557	0.689982	-0.127722242
Inverse v	112	-0.031275326	0.0789933720	0.692161	-0.186102335
Inverse v	112	0.03102118009	0.0788474252	0.693999	-0.123519773
Inverse v	112	0.03308346296	0.0846493262	0.695923	-0.132829217
Inverse v	112	-0.030301353	0.0774883275	0.695764	-0.182178475
Inverse v	112	-0.033866402	0.0865363712	0.695535	-0.203477689
Inverse v	112	0.03297469236	0.0844458241	0.696179	-0.132539123
Inverse v	112	-0.041721139	0.1079216667	0.699061	-0.253247606
Inverse v	112	0.03125558746	0.0807292010	0.698633	-0.126973647
Inverse v	112	0.03160291818	0.0817562825	0.699089	-0.128639396
Inverse v	112	0.03131882332	0.0814602031	0.700631	-0.128343175
Inverse v	112	0.02975052335	0.0775318162	0.701185	-0.122211836
Inverse v	112	0.03167452177	0.0840896828	0.706414	-0.133141257
Inverse v	112	0.03314963349	0.0870508663	0.703340	-0.137470065
Inverse v	112	-0.034724026	0.0917031261	0.704942	-0.214462154
Inverse v	112	-0.023488991	0.0617835877	0.703810	-0.144584823
Inverse v	112	0.03390620942	0.0895882888	0.705083	-0.141686837

Inverse \	112	0.0290388687	0.0770470675	0.706249	-0.121973384
Inverse \	112	-0.028503018	0.0754974743	0.705775	-0.176478068
Inverse \	112	0.0312315409	0.0825530379	0.705192	-0.130572414
Inverse \	112	0.0295426579	0.0786067508	0.707044	-0.124526574
Inverse \	112	-0.029261786	0.0787067760	0.710054	-0.183527067
Inverse \	112	-0.029202746	0.0787023023	0.710597	-0.183459259
Inverse \	112	-0.031956955	0.0866263171	0.712197	-0.201744537
Inverse \	112	0.0265704094	0.0719537611	0.711920	-0.114458962
Inverse \	112	-0.030363709	0.0829526412	0.714337	-0.192950886
Inverse \	112	-0.027413957	0.0754754479	0.716442	-0.175345834
Inverse \	112	0.0275977419	0.0764487485	0.718101	-0.122241805
Inverse \	112	0.0239698279	0.0663408045	0.717865	-0.106058149
Inverse \	112	0.0309774688	0.0861983354	0.719315	-0.137971269
Inverse \	112	-0.028230963	0.0798605264	0.723711	-0.184757595
Inverse \	112	0.0297340918	0.0845906127	0.725208	-0.136063509
Inverse \	112	-0.029375659	0.0834529144	0.724830	-0.192943371
Inverse \	112	-0.026383243	0.0749191334	0.724721	-0.173224745
Inverse \	112	-0.027913507	0.0798830545	0.726760	-0.184484294
Inverse \	112	0.0288943429	0.0829066889	0.727452	-0.133602767
Inverse \	112	-0.026545206	0.0773424043	0.731435	-0.178136318
Inverse \	112	0.0295181699	0.0862264556	0.732100	-0.139485683
Inverse \	112	0.0292542193	0.0858769306	0.733364	-0.139064565
Inverse \	112	0.0333204897	0.0980258289	0.733920	-0.158810135
Inverse \	112	0.0268159703	0.0797915925	0.736814	-0.129575551
Inverse \	112	0.0275956299	0.0828511541	0.739077	-0.134792632
Inverse \	112	0.0301395656	0.0904637091	0.739007	-0.147169304
Inverse \	112	-0.029675081	0.0891958847	0.739364	-0.204499015
Inverse \	112	-0.025003048	0.0762579617	0.743006	-0.174468653
Inverse \	112	-0.027487061	0.0839829507	0.743440	-0.192093644
Inverse \	112	-0.026377558	0.0799667911	0.741508	-0.183112469
Inverse \	112	0.0276187389	0.0844639119	0.743670	-0.137930528
Inverse \	112	0.0262448902	0.0803649954	0.743992	-0.131270501
Inverse \	112	0.0186066676	0.0562235202	0.740689	-0.091591432
Inverse \	112	0.0275019047	0.0834082593	0.741600	-0.135978284
Inverse \	112	0.0256844186	0.0782654824	0.742782	-0.127715927
Inverse \	112	0.0307448643	0.0946259881	0.745249	-0.154722072
Inverse \	112	0.0285120108	0.0875790693	0.744759	-0.143142965
Inverse \	112	-0.02581958	0.0801008959	0.747190	-0.182817336

Inverse v	112	0.0307097896	0.095450923	0.74765	-0.156374021
Inverse v	112	0.0301790299	0.093482303	0.74682	-0.153046286
Inverse v	112	0.0288414508	0.090069773	0.74880	-0.147695306
Inverse v	112	0.0256894213	0.080641410	0.75005	-0.132367743
Inverse v	112	-0.027498989	0.087290150	0.75273	-0.198587684
Inverse v	112	-0.025597048	0.081686676	0.75400	-0.185702934
Inverse v	112	-0.024963039	0.079741856	0.75424	-0.181257079
Inverse v	112	0.0268725635	0.085667895	0.75376	-0.141036511
Inverse v	112	-0.02846995	0.092234647	0.75757	-0.209249859
Inverse v	112	-0.022951098	0.074257648	0.75726	-0.16849609
Inverse v	112	-0.028017441	0.090500853	0.75687	-0.205399113
Inverse v	112	0.0253341709	0.082314460	0.75825	-0.136002172
Inverse v	112	-0.02478773	0.081059537	0.75975	-0.183664423
Inverse v	112	0.0250298395	0.081796441	0.75960	-0.135291185
Inverse v	112	-0.025176644	0.082700836	0.76080	-0.187270283
Inverse v	112	0.0234139272	0.077891300	0.76372	-0.129253022
Inverse v	112	-0.024826017	0.082573004	0.76367	-0.186669106
Inverse v	112	0.0231782163	0.077654219	0.76533	-0.129024054
Inverse v	112	-0.024264286	0.081092205	0.76477	-0.183205009
Inverse v	112	0.0234432269	0.078696732	0.76578	-0.130802369
Inverse v	112	-0.024031169	0.080566057	0.76549	-0.181940641
Inverse v	112	0.0236009002	0.079513114	0.76660	-0.132244805
Inverse v	112	-0.023780694	0.080588973	0.76792	-0.181735082
Inverse v	112	-0.02384501	0.081062866	0.76863	-0.182728229
Inverse v	112	0.0252522949	0.085975721	0.76897	-0.143260119
Inverse v	112	0.0544440571	0.186420764	0.77024	-0.310940642
Inverse v	112	-0.021687004	0.074332640	0.77047	-0.16737898
Inverse v	112	0.0266959110	0.091258072	0.76987	-0.15216991
Inverse v	112	-0.026011612	0.088632128	0.76915	-0.199730584
Inverse v	112	-0.02370417	0.081509821	0.77119	-0.18346342
Inverse v	112	0.0211311426	0.072863869	0.77180	-0.121682042
Inverse v	112	0.0244286824	0.084577610	0.77271	-0.141343435
Inverse v	112	0.0244421465	0.085123789	0.77400	-0.142400481
Inverse v	112	-0.023834681	0.083113873	0.77428	-0.186737874
Inverse v	112	0.0251051131	0.087937993	0.77527	-0.147253355
Inverse v	112	0.0236328554	0.082679061	0.77500	-0.138418106
Inverse v	112	-0.024254521	0.084334329	0.77365	-0.189549807
Inverse v	112	0.0234525958	0.082347545	0.77579	-0.137948594

Inverse v	112	0.0240864565	0.085157507	0.77729	-0.142822258
Inverse v	112	-0.022619629	0.081263859	0.78074	-0.181896793
Inverse v	112	-0.02314572	0.083022950	0.78040	-0.185870703
Inverse v	112	-0.024159348	0.087402646	0.78222	-0.195468534
Inverse v	112	0.0262201437	0.095453448	0.78355	-0.160868615
Inverse v	112	0.0216222461	0.079989619	0.78691	-0.135157408
Inverse v	112	0.0226844079	0.084392928	0.78808	-0.142725731
Inverse v	112	0.0216386139	0.081402680	0.79037	-0.137910639
Inverse v	112	-0.023196135	0.087474304	0.79087	-0.194645771
Inverse v	112	-0.024109891	0.093356828	0.79620	-0.207089274
Inverse v	112	0.0209840642	0.082002925	0.79803	-0.13974167
Inverse v	112	-0.023389473	0.090538830	0.79614	-0.20084558
Inverse v	112	-0.021043594	0.082439996	0.79852	-0.182625987
Inverse v	112	-0.020502215	0.079393349	0.79622	-0.176113179
Inverse v	112	0.0202405384	0.079079667	0.79798	-0.134755609
Inverse v	112	0.0203300012	0.079052582	0.79704	-0.13461306
Inverse v	112	0.0225323085	0.087959611	0.79782	-0.149868529
Inverse v	112	0.0180156980	0.069298664	0.79488	-0.117809685
Inverse v	112	-0.01841964	0.072692353	0.79996	-0.160896653
Inverse v	112	-0.019266909	0.075970436	0.79979	-0.168168965
Inverse v	112	0.0207533421	0.082012362	0.80022	-0.139990889
Inverse v	112	-0.020930649	0.083436501	0.80192	-0.184466192
Inverse v	112	-0.019213666	0.076723524	0.80225	-0.169591774
Inverse v	112	0.0197614559	0.079084167	0.80268	-0.135243513
Inverse v	112	-0.022435904	0.090396820	0.80398	-0.199613673
Inverse v	112	-0.019531542	0.078899296	0.80448	-0.174174164
Inverse v	112	-0.020410289	0.082150262	0.80378	-0.181424804
Inverse v	112	-0.017016378	0.069133609	0.80557	-0.152518251
Inverse v	112	-0.02182529	0.089233719	0.80677	-0.19672338
Inverse v	112	0.0214991305	0.088291621	0.80761	-0.151552448
Inverse v	112	0.0181284216	0.074790230	0.80847	-0.128460429
Inverse v	112	0.0210579463	0.088434210	0.81178	-0.152273105
Inverse v	112	-0.013510808	0.056809351	0.81201	-0.124857137
Inverse v	112	0.0205720102	0.086617611	0.81226	-0.149198508
Inverse v	112	-0.020123665	0.084250246	0.81121	-0.185254149
Inverse v	112	-0.018638311	0.077486939	0.80991	-0.170512712
Inverse v	112	-0.019548176	0.082225875	0.81208	-0.180710892
Inverse v	112	0.0204713050	0.086805683	0.81356	-0.149667835

Inverse v	112	-0.019984867	0.08460919541	0.81327	-0.18581889
Inverse v	112	0.0184277608	0.0793827301	0.81643	-0.13716239
Inverse v	112	-0.017870439	0.0773177593	0.81721	-0.169413247
Inverse v	112	-0.017839672	0.0778964506	0.81885	-0.170516716
Inverse v	112	0.0179189293	0.0783490841	0.81909	-0.135645276
Inverse v	112	-0.021787333	0.0949081346	0.81843	-0.207807277
Inverse v	112	0.0197547553	0.0876231433	0.82162	-0.151986606
Inverse v	112	0.0189329378	0.0851064279	0.82395	-0.147875661
Inverse v	112	-0.018240486	0.0831527662	0.82636	-0.181219908
Inverse v	112	-0.017716331	0.0807516043	0.82634	-0.175989475
Inverse v	112	-0.019094327	0.0873485821	0.82696	-0.190297548
Inverse v	112	-0.019103877	0.0888740217	0.82980	-0.193296959
Inverse v	112	0.0190331903	0.0880860016	0.82892	-0.153615373
Inverse v	112	0.0163755148	0.0765977965	0.83071	-0.133756166
Inverse v	112	0.0176097196	0.0823131498	0.83059	-0.143724054
Inverse v	112	-0.017570935	0.0814628357	0.82922	-0.177238093
Inverse v	112	0.0154963193	0.0729401290	0.83175	-0.127466334
Inverse v	112	-0.017791762	0.0838194084	0.83190	-0.182077803
Inverse v	112	-0.017422942	0.0825852954	0.83291	-0.179290121
Inverse v	112	-0.017180199	0.0817833325	0.83361	-0.177475531
Inverse v	112	0.0166268670	0.0795051460	0.83434	-0.13920322
Inverse v	112	0.0167947872	0.0807957892	0.83533	-0.14156496
Inverse v	112	0.0167281814	0.0811865194	0.83675	-0.142397397
Inverse v	112	0.0164145899	0.0803612617	0.83815	-0.141093483
Inverse v	112	-0.016074999	0.0783818423	0.83750	-0.16970341
Inverse v	112	0.0160384030	0.0784534882	0.83801	-0.137730434
Inverse v	112	0.0166529381	0.0823156258	0.83967	-0.144685689
Inverse v	112	-0.015849561	0.0786805166	0.84035	-0.170063374
Inverse v	112	-0.014688941	0.0739650282	0.84258	-0.159660397
Inverse v	112	-0.014952198	0.0754566877	0.84292	-0.162847306
Inverse v	112	-0.015531516	0.0785817391	0.84332	-0.169551725
Inverse v	112	0.0153510886	0.0787246242	0.84539	-0.138949175
Inverse v	112	0.0154111815	0.0795742928	0.84643	-0.140554433
Inverse v	112	0.0162846899	0.0846706761	0.84748	-0.149669835
Inverse v	112	0.0178943296	0.0930881440	0.84756	-0.164558433
Inverse v	112	0.0157810939	0.0856782090	0.85386	-0.152148196
Inverse v	112	-0.01560027	0.0838829858	0.85246	-0.180010922
Inverse v	112	-0.01592861	0.0861963187	0.85339	-0.184873394

Inverse v	112	0.0166241353(	0.089939603:	0.85335'	-0.159657487
Inverse v	112	0.0144293403(	0.078138022	0.85349	-0.138721183
Inverse v	112	-0.015839969	0.084498932	0.85130	-0.181457876
Inverse v	112	0.0141108550(	0.077766926	0.85601	-0.138312322
Inverse v	112	-0.013832512	0.076861109	0.85717	-0.164480286
Inverse v	112	-0.015198323	0.086664906	0.86078	-0.18506154
Inverse v	112	-0.015029397	0.085469016	0.86041	-0.182548669
Inverse v	112	0.0220977762(	0.126574878	0.86140	-0.225988986
Inverse v	112	0.0143645299	0.082555172	0.86186	-0.147443608
Inverse v	112	0.0133678199	0.075709430	0.85984	-0.135022664
Inverse v	112	0.0145423763(	0.082321176	0.85978	-0.146807129
Inverse v	112	0.0152133023(	0.087249181	0.86157	-0.155795094
Inverse v	112	0.0139868626(	0.079000147	0.85947	-0.140853427
Inverse v	112	-0.012704651	0.074892653	0.86529	-0.159494252
Inverse v	112	-0.014315285	0.083692668	0.86418	-0.178352915
Inverse v	112	0.0125984270(	0.074375863	0.86549	-0.133178266
Inverse v	112	-0.013495649	0.079325181	0.86490	-0.168973004
Inverse v	112	0.0133382343(	0.077731877	0.86375	-0.139016246
Inverse v	112	-0.014817647	0.088228176(	0.86662	-0.187744872
Inverse v	112	-0.014206749	0.085962486	0.86873	-0.182693222
Inverse v	112	-0.014486156	0.087531457	0.86855	-0.186047812
Inverse v	112	0.0149293245	0.091451968	0.87032	-0.164316534
Inverse v	112	0.0125555374	0.077923420	0.87199	-0.140174367
Inverse v	112	-0.013229079	0.082469589	0.87255	-0.174869475
Inverse v	112	-0.013510808	0.085044232	0.87377	-0.180197503
Inverse v	112	0.0117020144	0.077813449	0.88046	-0.140812346
Inverse v	112	0.0125722010(	0.082465258	0.87882	-0.149059705
Inverse v	112	-0.012170048	0.079196676	0.87787	-0.167395533
Inverse v	112	0.0140144470(	0.091454248	0.87820	-0.165235879
Inverse v	112	-0.013161132	0.084174740	0.87575	-0.178143623
Inverse v	112	-0.012596373	0.081949113	0.87783	-0.173216636
Inverse v	112	-0.013841674	0.091420451	0.87965	-0.193025759
Inverse v	112	-0.01245831	0.082608004	0.88012	-0.174369998
Inverse v	112	0.0124303981	0.081369628	0.87858	-0.147054075
Inverse v	112	-0.014406345	0.092405633	0.87610	-0.195521386
Inverse v	112	-0.01196614	0.079291306	0.88004	-0.167377102
Inverse v	112	0.0122323995(	0.082838597	0.88260	-0.150131252
Inverse v	112	-0.011652844	0.080077857	0.88430	-0.168605446

Inverse v	112	0.0111212466	0.0791424149	0.888247	-0.143997887
Inverse v	112	0.0134525652	0.0958991499	0.888440	-0.174509769
Inverse v	112	-0.012346921	0.0871487473	0.887335	-0.183158466
Inverse v	112	0.0136669896	0.0958957599	0.886669	-0.1742887
Inverse v	112	-0.011096141	0.0786855491	0.887855	-0.165319817
Inverse v	112	-0.011377037	0.0799757781	0.886877	-0.168129562
Inverse v	112	0.0117454141	0.0854751647	0.890704	-0.155785909
Inverse v	112	0.0118889715	0.0857252297	0.889697	-0.156132479
Inverse v	112	0.0109947812	0.0796674997	0.890233	-0.145153518
Inverse v	112	-0.010503139	0.0793446559	0.894688	-0.166018664
Inverse v	112	0.0112530962	0.0914714152	0.902088	-0.168030878
Inverse v	112	-0.009618682	0.0837271180	0.908539	-0.173723833
Inverse v	112	0.0084874623	0.0746654575	0.909490	-0.137856834
Inverse v	112	0.0100199806	0.0822890742	0.903084	-0.151266605
Inverse v	112	0.0103130502	0.0820439408	0.899968	-0.150493074
Inverse v	112	0.0103510968	0.0806002851	0.897812	-0.147625462
Inverse v	112	0.0095167453	0.0846291393	0.910464	-0.156356368
Inverse v	112	0.0114069439	0.0899977678	0.899140	-0.164988681
Inverse v	112	0.0103344739	0.0873950747	0.905869	-0.160959873
Inverse v	112	0.0099967489	0.0833369763	0.904518	-0.153343725
Inverse v	112	0.0099360488	0.0865157397	0.908560	-0.159634801
Inverse v	112	0.0085166380	0.0666987869	0.898395	-0.122212984
Inverse v	112	-0.011124508	0.0852531580	0.896180	-0.178220698
Inverse v	112	0.0104795021	0.0911276947	0.908440	-0.16813078
Inverse v	112	-0.009998084	0.0808691280	0.901605	-0.168501576
Inverse v	112	0.0094264763	0.0833499327	0.909955	-0.153939392
Inverse v	112	0.0110138357	0.0939880260	0.906714	-0.173202695
Inverse v	112	-0.00939371	0.0776686122	0.903733	-0.16162419
Inverse v	112	0.0091829227	0.0806495453	0.909347	-0.148890186
Inverse v	112	-0.010609471	0.0855364873	0.901287	-0.178260987
Inverse v	112	0.0100602818	0.0834346320	0.904020	-0.153471597
Inverse v	112	-0.009396023	0.0774695209	0.903463	-0.161236284
Inverse v	112	0.0089133765	0.0792655527	0.910460	-0.146447107
Inverse v	112	0.0108779655	0.0912054970	0.905062	-0.167884809
Inverse v	112	0.0108728181	0.0905101297	0.904381	-0.166527036
Inverse v	112	0.0100510957	0.0833365773	0.904001	-0.153288596
Inverse v	112	0.0108892331	0.0930588014	0.906848	-0.171506018
Inverse v	112	-0.009698641	0.0788482340	0.902103	-0.16424118

Inverse v	112	0.00910179797	0.08187523334	0.911484	-0.15137366
Inverse v	112	-0.009306728	0.08508871299	0.912903	-0.176080605
Inverse v	112	0.00882322295	0.0813650026	0.913640	-0.150652182
Inverse v	112	0.0093455825(	0.0858270759	0.913290	-0.158875486
Inverse v	112	-0.008530902	0.07873663944	0.913720	-0.162854716
Inverse v	112	-0.008805833	0.0833279938	0.915838	-0.172128701
Inverse v	112	-0.006062776	0.0578696722	0.916561	-0.119487333
Inverse v	112	0.00537938612	0.0520375943	0.917665	-0.096614299
Inverse v	112	0.0064469649	0.0630508843	0.918558	-0.117132768
Inverse v	112	0.0084379375	0.0837681322	0.919765	-0.155747602
Inverse v	112	-0.008362123	0.0826224111	0.919384	-0.170302049
Inverse v	112	0.0084374250	0.0854284548	0.921324	-0.159002346
Inverse v	112	-0.008081335	0.0823566817	0.921832	-0.169500432
Inverse v	112	0.0076182825	0.0783870531	0.922577	-0.146020342
Inverse v	112	-0.007861158	0.0815323128	0.923188	-0.167664492
Inverse v	112	0.0079057194	0.0831851170	0.924284	-0.15513711
Inverse v	112	-0.00709443	0.0760678035	0.925693	-0.156187325
Inverse v	112	-0.007196899	0.0782946576	0.926761	-0.160654428
Inverse v	112	0.0080470694(	0.0870743886	0.926367	-0.162618732
Inverse v	112	-0.00569638	0.0631242547	0.928095	-0.129419919
Inverse v	112	-0.007519945	0.0842648259	0.928889	-0.172679004
Inverse v	112	0.0071142953(	0.0793134441	0.928520	-0.148340055
Inverse v	112	0.0056800063	0.0642707265	0.929577	-0.120290618
Inverse v	112	-0.007466422	0.0860960756	0.930892	-0.176214731
Inverse v	112	-0.007139817	0.0836355286	0.931968	-0.171065454
Inverse v	112	0.0076204685	0.0895062320	0.932150	-0.167811746
Inverse v	112	-0.007004353	0.0841367634	0.933653	-0.171912409
Inverse v	112	-0.00680282	0.0821776694	0.934024	-0.167871052
Inverse v	112	-0.00721355	0.0894188673	0.935703	-0.18247453
Inverse v	112	-0.006948075	0.0861371701	0.935710	-0.175776929
Inverse v	112	-0.006277806	0.0795661878	0.937111	-0.162227534
Inverse v	112	-0.006690682	0.0864085780	0.938280	-0.176051495
Inverse v	112	0.0058709460	0.0787736819	0.940589	-0.14852547
Inverse v	112	0.0073002752	0.0985948105	0.940970	-0.185945554
Inverse v	112	-0.006402051	0.0882486565	0.942167	-0.179369417
Inverse v	112	0.0056909581	0.0827673118	0.945181	-0.156532973
Inverse v	112	0.0056188032	0.0844777213	0.946970	-0.159957531
Inverse v	112	0.0077445103	0.1168796894	0.947170	-0.221339681

Inverse v	112	0.00467655242	0.0767337634	0.951402	-0.145721624
Inverse v	112	-0.00459678	0.0776203929	0.952775	-0.15673275
Inverse v	112	-0.005452828	0.0865464081	0.949762	-0.175083788
Inverse v	112	-0.005066664	0.0858793438	0.952954	-0.173390178
Inverse v	112	-0.004468049	0.0744969438	0.952174	-0.150482059
Inverse v	112	-0.005047468	0.0789488777	0.949023	-0.159787268
Inverse v	112	0.0051629373	0.0821630445	0.949895	-0.15587663
Inverse v	112	0.0048418743	0.0816757381	0.952727	-0.155242572
Inverse v	112	0.0053439314	0.0885702684	0.951888	-0.168253795
Inverse v	112	-0.004532546	0.0799410122	0.954785	-0.16121693
Inverse v	112	-0.004877715	0.0866845468	0.955120	-0.174779427
Inverse v	112	0.0036206908	0.0656236644	0.956000	-0.125001691
Inverse v	112	-0.004225647	0.0844279368	0.960082	-0.169704403
Inverse v	112	0.0037359724	0.0757167140	0.960647	-0.144668787
Inverse v	112	-0.004155797	0.0880021134	0.962334	-0.17663994
Inverse v	112	0.0035354301	0.0780916648	0.963889	-0.149524233
Inverse v	112	0.0037406046	0.0810658805	0.963190	-0.155148521
Inverse v	112	-0.003924928	0.0822650490	0.961940	-0.165164424
Inverse v	112	0.0037636750	0.0831067838	0.963878	-0.159125621
Inverse v	112	-0.00350183	0.0838373902	0.966682	-0.167823115
Inverse v	112	-0.003181317	0.0738421750	0.965635	-0.14791198
Inverse v	112	-0.003885384	0.0937623823	0.966940	-0.187659654
Inverse v	112	-0.003305517	0.0797041447	0.966919	-0.159525641
Inverse v	112	-0.003407396	0.0841171089	0.967688	-0.16827693
Inverse v	112	0.0032118041	0.0842088797	0.969575	-0.1618376
Inverse v	112	0.0030681721	0.0796741133	0.969281	-0.15309309
Inverse v	112	0.0033297350	0.0865151137	0.969299	-0.166239888
Inverse v	112	0.0032950057	0.0887162118	0.970372	-0.170588769
Inverse v	112	0.0029898865	0.0823865450	0.971050	-0.158487742
Inverse v	112	0.0032309530	0.0958374239	0.973100	-0.184610398
Inverse v	112	-0.002579066	0.0772704672	0.973373	-0.154029182
Inverse v	112	-0.003585599	0.1058272553	0.972971	-0.21100702
Inverse v	112	0.0026598397	0.0794977997	0.973309	-0.153155848
Inverse v	112	0.0025046845	0.0789784031	0.974700	-0.152292986
Inverse v	112	0.0024889013	0.0775088884	0.974383	-0.14942852
Inverse v	112	-0.00260405	0.0900321639	0.976925	-0.179067091
Inverse v	112	0.0026030427	0.0912948671	0.977253	-0.176334897
Inverse v	112	0.0023975277	0.0795812140	0.975965	-0.153581652

Inverse v	112	0.00237168305	0.0812287627	0.976707	-0.156836692
Inverse v	112	0.00169666568	0.0812852351	0.983340	-0.157622395
Inverse v	112	-0.001200141	0.0649185833	0.985250	-0.128440564
Inverse v	112	-0.000976632	0.0560828597	0.986100	-0.110899037
Inverse v	112	-0.001358986	0.0794285123	0.986349	-0.15703887
Inverse v	112	-0.001545755	0.0811646139	0.984805	-0.160628399
Inverse v	112	-0.001108537	0.0803164068	0.988987	-0.158528695
Inverse v	112	-0.000864403	0.0840300623	0.991792	-0.165563325
Inverse v	112	-0.000787126	0.0786003555	0.992009	-0.154843823
Inverse v	112	-0.00060896	0.0704423396	0.993102	-0.138675946
Inverse v	112	0.00058973915	0.0806640116	0.994160	-0.157511724
Inverse v	112	0.00036843875	0.0753090327	0.996090	-0.147237265
Inverse v	112	-0.000192417	0.0777140155	0.998024	-0.152511887

g index and circulating protein levels (UKB-PPP and deCODE)

up_ci	or	or_lci95	or_uci95	exposure	fdr
0.91168414529	2.16495628311	1.88347070055	2.48851001860	CXCL17	2.90440578344
0.80464235586	1.93279287633	1.67077857499	2.23589670044	TFF2_Q	6.80942477822
0.78818241978	1.90137551722	1.64373771244	2.19939521388	TNFRSF	3.09035313866
0.77327369766	1.87624471306	1.62461501722	2.16684826133	MMP12_	4.85484081166
0.88453939138	2.03957762844	1.71763113022	2.42186860099	PLAUR_	1.52306590477
0.73148966658	1.79226531322	1.54569098388	2.07817408944	TFF1_P0	3.30594664022
0.84324775368	1.95539625411	1.64532506	2.32390219588	PIGR_P0	6.89337995577
0.78549483469	1.86285873777	1.58206300155	2.19349208822	WFDC2_	1.89834731411
0.74639762663	1.80356034477	1.54207318411	2.10938751199	GDF15_	3.16522312855
-0.449992527	0.53887926677	0.45542012760	0.63763291633	SLITRK	1.07056048488
0.73782906989	1.78551803044	1.52438050699	2.09139032044	MSLN_0	1.09117436011
0.69915002028	1.72407651944	1.47732510633	2.01204178577	CFB_P0	7.16308327088
0.74098396527	1.77426353644	1.50048275099	2.09799885722	CD83_Q	2.77414827166
0.72463348160	1.75128088044	1.48596058577	2.06397447677	ICAM1_	2.96052464600
0.69413082797	1.70749543744	1.45633710744	2.00196826277	RNASE6	5.23262669333
0.75635466679	1.79065430355	1.50502198299	2.13049568111	CFH_P0	5.61478392266
0.71739185069	1.73524284344	1.46947161600	2.04908192360	PRSS8_0	8.60658648188
0.61026081531	1.58932560122	1.37212239822	1.84091147400	HAVCR	6.41888660411
0.63296283939	1.61633675588	1.38730333260	1.88318188722	TNFRSF	6.91805706766
0.61367842346	1.58681845155	1.36313017177	1.84721375133	ALDH3A	2.32304147211
0.67933922058	1.66468376077	1.40485082560	1.97257386522	FABP1_	3.38483733333
0.64536283939	1.62014230500	1.37666669188	1.90667872199	IGFBP4_	5.18293667677
0.66463471056	1.64240111777	1.38775012960	1.94378034922	ADM_P	6.11355818222
0.71064602935	1.69830110122	1.41709750477	2.03530570122	HGF_P1	7.31658935666
0.59900088507	1.56185231006	1.34009982228	1.82029920344	CDCP1_	8.26308664544
0.60281936937	1.56382918155	1.33837402777	1.82726327500	CFI_P05	1.25017513844
0.57943245709	1.53613817933	1.32195370944	1.78502506488	COLEC1	1.40101674100
0.58136812202	1.53728263411	1.32136401400	1.78848362155	CCL20_	1.64716597366
0.57570560284	1.52996971599	1.31625460177	1.77838491777	FUT3_F	1.87780391966
0.63967841475	1.60314817711	1.35562174133	1.89587109700	IL6_P05	2.07900848255
0.66459922240	1.63213953733	1.37051185255	1.94371136922	ACVRL	2.25238355144
0.60544118357	1.56192739744	1.33162494022	1.83206030555	RNASE4	2.39140442033
-0.287743539	0.63876246944	0.54405676344	0.74995390155	SCGB1A	2.39140442033
0.65407119741	1.61824378755	1.36153366811	1.92335527011	TREM2_	2.48997488244
0.72178810319	1.70039481822	1.40485323333	2.05811003555	ASGR1_	2.59139679699
0.63731117956	1.59730904655	1.34895410533	1.89138843211	RNASE1	2.78628511466

0.51370208533	1.4580658550	1.2719097560	1.6714676709	SFTPD_	3.0387235752
0.55552038809	1.5028601064	1.2959184510	1.7428477060	RARRE:	3.3425819650
0.55763177067	1.5046409711	1.2962517804	1.74653141	CHCHD	3.5593880421
0.42246014272	1.3627154617	1.2171336187	1.5257104077	MSMB_	3.5593880421
0.55890309544	1.5057208211	1.2964637601	1.7487532324	C1RL_C	3.6021785205
0.52417138870	1.4678215477	1.2755625970	1.6890586952	ICAM5_	3.6021785205
-0.283490652	0.6384766689	0.5412631864	0.7531501623	LRTM2_	4.2452976716
0.62544116966	1.5791763695	1.3342451248	1.8690703527	IL6_P05	4.3912337601
0.54323219422	1.4837894199	1.2788564428	1.7215623029	FABP3_	7.8089338344
0.65839249129	1.6126309148	1.3462748614	1.9316846373	HAVCR	8.2908681019
0.52040071751	1.4587394204	1.2645857354	1.6827018027	EDA2R_	8.4223491454
0.62555397342	1.5739486714	1.3252764841	1.8692812027	CCL21_	8.7734131876
0.55057304143	1.4896473754	1.2795466313	1.7342465282	CCL22_	9.9837264368
0.55132516568	1.4904864497	1.2800253985	1.7355513878	LGALS4	9.9837264368
-0.27869524	0.6370849983	0.5363281129	0.7567705017	NCAN_(	1.0051216647
0.56325093786	1.5029413815	1.2860780039	1.7563730889	LTA4H_	1.0303688037
0.61927868063	1.5646359538	1.3178843406	1.8575876445	COL6A3	1.0791082837
0.60266107754	1.5454083964	1.3072364671	1.8269740570	SIGLEC	1.1459775905
0.61665161982	1.5607527942	1.3148004574	1.8527140532	CD80_P	1.1769674700
0.58345916450	1.5237039870	1.2954125863	1.7922273295	VSIG2_(	1.1769674700
-0.238212338	0.6780271641	0.5833759095	0.7880353434	APLP1_	1.2634793246
0.62426006904	1.5684860642	1.3177973369	1.8668640957	IL6_P05	1.2634793246
0.57686620052	1.5153747205	1.2897640527	1.7804501053	SERPIN	1.3187505035
0.63062094785	1.5746642572	1.3197775653	1.8787768395	EFNA4_	1.3957134730
-0.243990739	0.6702374892	0.5733519102	0.7834948902	BCAN_(	1.4991853534
0.62394338380	1.5662286579	1.3144230420	1.8662729810	MRC1_I	1.5189124586
0.51563233207	1.4465411857	1.2494685531	1.6746971317	AMBP_I	2.2216546360
0.58538138347	1.5197620086	1.2862437063	1.7956756961	TNFRSF	2.4619479762
0.57008225927	1.5030095346	1.2774381790	1.7684125137	RBP5_P	2.5012838067
0.63050980097	1.5692012391	1.3107816636	1.8785680310	ANGPT:	2.5087165795
0.53433970461	1.4643053286	1.2566157548	1.7063211941	B2M_P6	2.7462847416
-0.1616331	0.7632644470	0.6847726878	0.8507532886	ENPP5_	2.8127334746
-0.222085899	0.6894678860	0.5935793245	0.8008465697	NPTXR_	2.9541216313
0.54948241532	1.4793862779	1.2633567099	1.7323561447	CD27_P	2.9738098971
0.56543974420	1.4962044751	1.2717874586	1.7602216598	GFRA1_	2.9750806026
0.57990389844	1.5114695724	1.2792332950	1.7858667979	CPM_P1	3.0299164769
0.57573394275	1.5043440858	1.2724956066	1.7784353177	IL10RB_	4.2745753368
0.57508203924	1.5031163539	1.2712478856	1.7772763273	TNFRSF	4.5261721258

0.50619454098	1.4303443421	1.2332289435	1.6589660402	SPINK6_	5.3522909384
0.53470163080	1.4588402842	1.2468021055	1.7069388682	AGRN_(	5.7797309781
0.49014933607	1.4133349023	1.2235480129	1.6325600018	PLAT_P	5.9250906001
0.50757366121	1.4308246276	1.2323565371	1.6612555322	PLG_P0	5.9250906001
0.54669129009	1.4701271362	1.2510791245	1.7275276634	IFNLR1_	6.4570292136
0.57428153320	1.4988362706	1.2650307644	1.7758541762	IL6_P05	6.4570292136
-0.19532359	0.7145232069	0.6206698290	0.8225684403	IL7R_P1	6.4570292136
-0.227041189	0.6763142308	0.5739840009	0.7968879587	OMG_P	6.5211956251
0.56798716628	1.4909864428	1.2597190499	1.7647114035	APCS_P	7.3655633909
0.50415654730	1.4233842998	1.2237478333	1.6555885207	ANGPT	0.0001000167
0.49122438058	1.4103948179	1.2171535480	1.6343160202	GM2A_	0.0001012124
0.50409898720	1.4216704340	1.2208729031	1.6554932276	RNF149	0.0001239214
-0.219978268	0.6779037070	0.5726263983	0.8025362388	IL12RB	0.0001309810
0.50565325521	1.4225707071	1.2205211367	1.6580683085	CRELD	0.0001325595
0.40701146906	1.3277422550	1.1734503490	1.5023213357	CAPG_I	0.0001386287
0.54640972816	1.4618310752	1.2373473990	1.7270413258	EDN1_F	0.0001608246
0.57556835681	1.4913816039	1.2508677690	1.7781408582	LGALS3	0.0001659413
0.51616093383	1.4308199608	1.2218112934	1.6755826136	CFD_P0	0.0001668341
0.47710905863	1.3925989130	1.2035004924	1.6114091725	SELE_P	0.0001668341
-0.222853276	0.6714155291	0.5633349697	0.8002322542	SEZ6L_	0.0001668341
0.562640995	1.4770407850	1.2428911493	1.7553021290	BSG_P3	0.0001789213
0.51400662142	1.4279513399	1.2195414823	1.6719767706	TNF_P0	0.0001799027
0.51825676068	1.4320358969	1.2213264364	1.6790980272	CST3_P	0.0001810265
-0.183520267	0.7173282428	0.6182123832	0.8323350063	VWC2L	0.0002186816
0.58020317960	1.4924002999	1.2467851360	1.7864013541	IL1RN_	0.0002316111
0.47358444792	1.3864253320	1.1970653432	1.6057395799	ANG_P(	0.0002330149
0.45883147311	1.3722339060	1.1901133185	1.5822240330	CCL16_	0.0002337740
0.47472026329	1.3873489541	1.1973001349	1.6075644398	LGALS1	0.0002337740
0.50967025499	1.4207566299	1.2125297509	1.6647421640	LGALS9	0.0002448606
-0.193719081	0.7021152578	0.5983398798	0.8238893176	ACAN_	0.0002530392
0.56600798070	1.4762543843	1.2373947188	1.7612221662	ADAMT	0.0002581240
0.42732819301	1.3418814318	1.1744702238	1.5331557501	CLEC7A	0.0002581240
-0.205480689	0.6860532752	0.5780359021	0.8142558182	FASLG_	0.0002706226
0.62255155240	1.5341657668	1.2629142731	1.8636772505	SPINT1_	0.0002706226
0.51663824544	1.4254642365	1.2121029616	1.6763825795	ADAMT	0.0003009151
0.37521102606	1.2935182677	1.1497225639	1.4552984881	CD300L	0.0003046216
0.44559258043	1.3571859710	1.1796694285	1.5614151858	CRHBP_	0.0003159969
0.44883460163	1.3598304410	1.1804378523	1.5664855416	ENPP7_	0.0003306623

0.59649883627	1.50434226888	1.24634180900	1.81575041900	BPIFB1_	0.00033195233
0.53063724211	1.43799254722	1.21635527888	1.70001528488	TNFRSF	0.00033195233
0.43670861089	1.34786892777	1.17391103066	1.54760505600	PGF_P4	0.00035828877
0.54937328568	1.45569380088	1.22334873888	1.73216710366	FCN1_C	0.00035844199
0.54639963417	1.45190649044	1.22061568777	1.72702389322	CCL7_P	0.00038766433
-0.169378941	0.72841836755	0.62852436200	0.84418894511	GFAP_F	0.00038766433
0.54445377106	1.44964968444	1.21919412788	1.72366660866	BMPER	0.00039325744
-0.210588257	0.67405317922	0.56084860122	0.81010755399	CA6_P2	0.00039325744
-0.179944891	0.71330870933	0.60912177744	0.83531624333	WFIKKN	0.00040758444
0.46341414288	1.37097539799	1.18249991400	1.58949148277	REG3A_	0.00042636588
0.46847530573	1.37549812588	1.18430555344	1.59755655000	ITIH4_C	0.00043118866
0.50181509465	1.40706955499	1.19865887655	1.65171657344	KRT19_	0.00043118866
0.47332061862	1.37926042544	1.18503729322	1.60531599466	SPON1_	0.00047284377
0.48930188205	1.39424325244	1.19172484988	1.63117706833	SPINK1	0.00047329399
-0.185903568	0.70235137100	0.59408113644	0.83035366411	NTRK3_	0.00049915922
-0.17311262	0.71921670566	0.61503720599	0.84104289100	AMY1A	0.00051298966
0.48076886873	1.38502205466	1.18609127522	1.61731742900	FAM20A	0.00053392900
0.55288913726	1.45354832544	1.21546441588	1.73826786455	MMP9_	0.00057607188
0.51594644129	1.41731167155	1.19910726622	1.67522325222	IL4R_P2	0.00059497488
-0.178111357	0.71001695688	0.60240729300	0.83684922955	PTPRR_	0.00060250755
0.46952802391	1.37295754122	1.17869321588	1.59923921244	RBP7_Q	0.00062927955
0.52969009215	1.42898354466	1.20230034388	1.69840587777	CD74_P	0.00068506822
-0.183014998	0.70069632977	0.58957911244	0.83275566600	OPTC_C	0.00071918066
0.47988767567	1.38111467866	1.18044814099	1.61589288799	TNF_P0	0.00073362044
0.53103196344	1.42927553488	1.20117882699	1.70068644955	SPON2_	0.00074287044
0.53711349457	1.43504617311	1.20355605722	1.71106074100	MSR1_F	0.00074402855
0.45218047989	1.35477908611	1.16777044711	1.57173558966	CFHR5_	0.00079675100
0.53984244358	1.43670498900	1.20305257066	1.71573651566	IL22_Q9	0.00079787455
0.54063028348	1.43754281211	1.20350756944	1.71708877388	TNFRSF	0.00079787455
0.48965699135	1.38910351622	1.18253469599	1.63175641733	VWC2_(	0.00079787455
0.43359903270	1.33771291011	1.15988830511	1.54280013166	PTGR1_	0.00080229344
0.52668800510	1.42340002533	1.19650975622	1.69331476122	GPD1_P	0.00084166699
0.51879658731	1.41577704088	1.19310656500	1.68000469377	LAMA4	0.00084571422
0.52881067439	1.42505738522	1.19675471866	1.69691292600	FSTL3_(	0.00085567744
0.52333505011	1.41989870100	1.19462940199	1.68764666088	TNFRSF	0.00085567744
-0.160350634	0.72852189899	0.62305246300	0.85184505100	CNTN5_	0.00087385433
0.40884876554	1.31403071800	1.14722941244	1.50508408266	SERPIN	0.00097005299
0.43044300834	1.33260618311	1.15468792622	1.53793869233	CPB2_Q	0.00102864311

0.46983807196	1.36804151744	1.16990466611	1.59973513022	CTSD_P	0.0010286431
0.45736235100	1.35626826160	1.16429022800	1.57990126	PRCP_P	0.00107588744
0.47970726105	1.37634849870	1.17252634790	1.61560138335	ANXA1	0.00110032444
0.47443271991	1.37101558722	1.16961053760	1.60710226177	EDIL3_	0.00115661822
0.47580951188	1.37204010844	1.16974761633	1.60931643111	GGH_Q	0.00117884855
0.51210537662	1.40344340322	1.18028059722	1.66880095344	PSAP_P	0.00144069833
0.47393319070	1.36835111855	1.16565098100	1.60629966760	CKAP4_	0.00144393133
0.53568337441	1.42470382090	1.18796827960	1.70861546744	BST2_Q	0.00153136566
0.53177309721	1.42088766211	1.18624218559	1.70194735299	CEACA	0.00154126733
0.40159105485	1.30374004522	1.13755716877	1.49420016188	LBP_P1	0.00154436555
0.51823525974	1.40794718200	1.18060878960	1.67906192544	CD79B_	0.00156432555
0.46057731793	1.35485994690	1.15814415744	1.58498876333	SERPIN	0.00164231677
-0.35573598	0.47863253633	0.32696299911	0.70065758333	NCAM1	0.00166336499
0.47697392636	1.36932238644	1.16376226844	1.61119143388	CLSTN3	0.00166631966
0.49440198523	1.38508623700	1.17013932177	1.63951749011	FABP4_	0.00166631966
0.48641805289	1.37765428444	1.16689510511	1.62647980860	TNFRSF	0.00168405299
0.50210725662	1.39147957311	1.17190190355	1.65219921260	SERPIN	0.00175407777
0.47906091796	1.37039880022	1.16316259122	1.61455748811	CD300E	0.00175829866
0.51445576833	1.40272829022	1.17631005811	1.67272790255	LILRA5	0.00175829866
0.42278877152	1.31989160311	1.14146263955	1.52621188255	ASGR2_	0.00190286644
0.44232415183	1.33674608088	1.14815071499	1.55632014277	UXS1_C	0.00193047366
0.46725564403	1.35812764377	1.15599147222	1.59560925922	SCARB	0.00205614988
-0.132593685	0.75536676011	0.65147903660	0.87582087860	LEPR_P	0.00208646455
-0.148731309	0.73009279822	0.61851368877	0.86180064200	TNR_Q	0.00208646455
0.46915225245	1.35944237355	1.15603603277	1.59863837688	FLT3LG	0.00209949344
0.45278943833	1.34480869677	1.14994498455	1.57269300277	VNN2_	0.00212157866
0.48923694454	1.37626897388	1.16127140833	1.63107114722	NHLRC	0.00231974144
0.49957631602	1.38517909777	1.16425636677	1.64802288188	CCL3_P	0.00239548111
-0.137333429	0.74404811160	0.63510449722	0.87167953433	DNER_	0.00252976566
0.33357554496	1.24247058744	1.10586525899	1.39595049955	XG_P55	0.00258407811
0.43089962139	1.32327519955	1.13805439011	1.53864109555	CDH2_F	0.00268220055
0.48800847126	1.37332378744	1.15772789877	1.62906865011	TNFRSF	0.00268220055
0.45717484178	1.34590064799	1.14677309000	1.57960504100	CD207_	0.00271389922
0.55265273360	1.43146207333	1.17908647877	1.73785698022	OSM_P	0.00282473500
0.45814734119	1.34604457277	1.14590343277	1.58114195322	LAIR1_	0.00288112433
0.32451837145	1.23412471511	1.10098540411	1.38336421788	LECT2_	0.00292213911
0.45393174705	1.34216419088	1.14411911700	1.57449053011	SULT2A	0.00292213911
-0.11351745	0.77989784977	0.68135813922	0.89268861860	CNTN1_	0.00294367677

0.2737642993	1.194086032	1.084368547	1.314904841	MICB_M	0.0029436767
0.4418726223	1.331113636	1.139009702	1.555617576	ADAM1	0.0030415468
0.2787230641	1.197691002	1.085529637	1.321441338	CEACA	0.0030415468
0.4336093054	1.323823573	1.135915674	1.542815980	CHRDL	0.0030754606
-0.137937228	0.737212956	0.623865968	0.871153374	TNFSF1	0.0032056139
0.4136655917	1.306200249	1.128149981	1.512351299	MYL3_	0.0032723517
0.3881825014	1.284262252	1.118721326	1.474298821	NPL_Q	0.0035045624
0.5090895721	1.388154298	1.158192351	1.663775757	LRRC2	0.0035412569
0.4795665008	1.360913949	1.146537453	1.615373987	KRT18_	0.0038828525
0.4077928150	1.299474412	1.123138450	1.503495627	GSTA1_	0.0039055452
-0.140794647	0.727673851	0.609564795	0.868667676	APOF_	0.0039244393
0.4020820231	1.293690831	1.119538406	1.494933946	ROBO2_	0.0043282039
0.4668441377	1.348155827	1.139547292	1.594952791	F9_P00	0.0044318045
0.3828956696	1.277534552	1.112899207	1.466525019	VEGFA_	0.0044661953
0.4337345691	1.319688492	1.128689095	1.543009251	ACE2_	0.0044773101
-0.137528964	0.729468138	0.610577400	0.871509106	CHGB_	0.0044979265
0.4775559799	1.356494938	1.141396216	1.612129506	SFRP1_	0.0046977452
0.4890502648	1.366462061	1.144994302	1.630766687	TGFBR	0.0046977452
0.4280684835	1.313491439	1.124466996	1.534291151	C1S_P0	0.0050542775
0.4410007989	1.324297479	1.128357944	1.554261944	TNF_P0	0.0050581665
0.4419992686	1.324863085	1.128194961	1.555814602	SERPIN	0.0051660459
0.4106370450	1.298427854	1.118145304	1.507778001	TPP1_O	0.0052760466
0.4574112116	1.337359548	1.131996803	1.579978456	CCL11_	0.0053081189
0.4296029002	1.313957197	1.123539298	1.536647200	CTSZ_	0.0053081189
0.4951344905	1.369958556	1.143880564	1.640718885	SERPIN	0.0053081189
0.4967149467	1.371176945	1.144106477	1.643314019	TIMP1_	0.0053081189
0.4517365565	1.332273708	1.129796490	1.571038014	ACP5_P	0.0053851410
0.3684917930	1.263705665	1.104734488	1.445552776	LILRB2	0.0053851410
0.4128382479	1.299701213	1.117876112	1.511100582	CD14_P	0.0053949375
0.4057142415	1.293626509	1.115368453	1.500373746	S100A1	0.0054864419
0.3910535289	1.281554693	1.110815424	1.478537655	STAB2_	0.0055160032
0.4025259046	1.290766219	1.113987718	1.495597667	S100A1	0.0055761182
0.5040224412	1.376019384	1.143812765	1.655366511	IGSF3_	0.0057898994
0.4417600513	1.322647963	1.124694528	1.555442469	AFM_P	0.0058534936
0.4251242043	1.308606851	1.119410261	1.529780413	SERPIN	0.0059311546
-0.139772492	0.716149412	0.589806700	0.869556043	BPIFA2	0.0059985615
0.4487132066	1.327572320	1.125233642	1.566295389	AREG_	0.0062583341
0.5284842986	1.395964000	1.148763485	1.696359185	ERBB2_	0.0063175256

-0.154626228	0.6891821377	0.5543976021	0.8567353413	KITLG_	0.0063369748
0.3803201796	1.2710706355	1.1045068560	1.4627528581	RELT_C	0.0064380492
-0.107939633	0.7692521289	0.6591966638	0.8976817850	FOLH1_	0.0068089269
0.4360938166	1.3154260584	1.1187672474	1.5466538898	TNFRSF	0.0070741980
-0.109590636	0.7649131245	0.6528581555	0.8962009329	IGDCC4	0.0071030147
0.4234008064	1.3045237302	1.1143544030	1.5271462634	EFEMP1	0.0073071732
0.3983437009	1.2839625474	1.1068945275	1.4893558350	CD4_P0	0.0074195658
0.4275917497	1.3074941998	1.1147533971	1.5335598770	CHI3L1	0.0075233852
0.4562770946	1.3312694094	1.1229832562	1.5781875915	CLEC6A	0.0075233852
0.4360489766	1.3134800844	1.1155096202	1.5465845393	REG4_C	0.0081471831
0.3812707068	1.2690558196	1.0999620100	1.4641439054	PLA2G2	0.0082735821
0.4007534007	1.2845186807	1.1051872312	1.4929490630	GRN_P2	0.0083044829
0.4249552082	1.3039804546	1.1116970714	1.5295219082	CFP_P2	0.0083498822
0.4280173002	1.3063313726	1.1122980149	1.5342126230	GKN1_C	0.0084216790
0.3021377891	1.2074885291	1.07782748	1.3527476077	LILRA3	0.0085099124
0.4382071749	1.3143107268	1.1145130212	1.5499257979	CHGA_	0.0086090745
0.4088782369	1.2896773530	1.1050669367	1.5051284402	IGSF8_C	0.0091212137
-0.101402431	0.7727524552	0.6608749692	0.9035693358	MOG_Q	0.0091212137
0.4080023775	1.2890295586	1.1049244181	1.5038107365	OSCAR	0.0091212137
0.3882448715	1.2732320290	1.0995184084	1.4743907763	TNFRSF	0.0091212137
0.4484872778	1.3211412958	1.1146101298	1.5659415583	HSPG2_	0.0095825512
0.4593070964	1.3300880391	1.1175996024	1.5829767547	IL17C_C	0.0095825512
0.3992298230	1.2811579164	1.1010879753	1.4906761709	FABP2_	0.0097091212
0.4204921725	1.2980110377	1.1064692294	1.5227108077	COL18A	0.0097698976
0.3791539707	1.2651785554	1.0955675667	1.4610479770	TFF3_Q	0.0097698976
0.4659377118	1.3350116424	1.1184483395	1.5935077395	DLL1_C	0.0098133922
0.4852443961	1.3509388143	1.1233947652	1.6245719995	CLEC4D	0.0098821646
0.4494834141	1.3211208256	1.1134658732	1.5675022269	CD300C	0.0099689957
0.4112510698	1.2901857411	1.1033172427	1.5087040990	OXT_P0	0.0099689957
0.4022644235	1.2825766413	1.1001842741	1.4952066482	LILRB4	0.0103388651
0.4200932626	1.2966792795	1.1046404854	1.5221035043	CGREF1	0.0104071085
0.3537988405	1.2444859069	1.0872441549	1.4244686124	SERPIN	0.0104819587
0.4001750772	1.2805907592	1.0990739118	1.4920859050	TNF_P0	0.0105280043
0.3930088157	1.2748112708	1.0970090969	1.4814314492	RTN4R_	0.0106045066
-0.103409265	0.7618919051	0.6437196877	0.9017578398	NOTCH	0.0107671961
-0.1023818	0.7632474807	0.6453489530	0.9026848406	OMD_Q	0.0109727542
0.4564859933	1.3250054339	1.1122078877	1.5785173073	ITGA5_	0.0111323025
0.4087190610	1.2864626446	1.0997397607	1.5048887932	QPCT_C	0.0111803818

0.42270223712	1.2973342679	1.1028756047	1.5260798183	IL2RA_	0.0113835531
0.38856946545	1.2702310025	1.0939861959	1.4748694323	PCSK9_	0.0114643058
0.40287624764	1.2808836804	1.0966106355	1.4961217316	FLT4_P	0.0120181659
0.44436390630	1.3137864675	1.1067888514	1.5594978934	MARCC	0.0121301436
0.40277055058	1.2804019259	1.0959017233	1.4959636043	CD99L2	0.0123476491
0.44609490185	1.3140916235	1.1053879849	1.5621997151	IL18BP_	0.0130766600
0.45454207928	1.3206869501	1.1071198978	1.5754517858	CSF1_P	0.0132285335
0.49450557272	1.3533383944	1.1169963770	1.6396873324	TNFSF1	0.0132285335
0.45787731960	1.3229843515	1.1072758331	1.58071507	LPCAT2	0.0135273353
0.35100725459	1.2390517424	1.0807826686	1.4204976310	KLK10_	0.0138440356
-0.083425613	0.7941157750	0.6854865387	0.9199595171	DDC_P2	0.0139138420
0.48612361730	1.3453787790	1.1131875543	1.6260009856	LCN2_P	0.0139165649
0.41065456652	1.2848469825	1.0948580240	1.5078044204	SIGLEC	0.0139165649
0.41156738162	1.2854820113	1.0949406124	1.5091813954	FAS_P2	0.0139263385
0.29468171497	1.1968624298	1.0668658806	1.3426989297	CHIT1_	0.0140972394
0.37888163266	1.2598361894	1.0866306648	1.4606501322	IL18R1_	0.0141569485
0.41378303251	1.2866809973	1.0945562529	1.5125289216	SIGLEC	0.0143914767
0.44362531931	1.3101606231	1.1015014088	1.5583464938	GALNT	0.0144729365
0.34483077808	1.2334649081	1.0776940688	1.4117510002	F11_P03	0.0146592702
-0.099004456	0.7576977423	0.6338537663	0.9057386722	NELL2_	0.0146592702
-0.102516501	0.7493708953	0.6221799247	0.9025632561	APOD_	0.0149043021
0.44913521338	1.3137271394	1.1014211172	1.5669565163	FURIN_	0.0151081534
0.46279527736	1.3246195187	1.1045690376	1.5885081055	LGMN_	0.0151081534
0.34536367951	1.2334471095	1.0770888321	1.4125035248	SMOC2_	0.0151081534
0.37096453649	1.2523788050	1.0823396462	1.4491316811	CCL15_	0.0155722697
-0.086077	0.7823004564	0.6670062958	0.9175235795	AMY2B	0.0157050606
0.36811266361	1.2500489510	1.0813955659	1.4450048292	CCL18_	0.0157050606
0.43399700173	1.3008965344	1.0964857968	1.5434142405	FGFR2_	0.0157566106
0.41333803690	1.2846491212	1.0915876655	1.5118560026	A1BG_	0.0157761976
0.39354679259	1.2691374480	1.0866811092	1.4822286394	C1R_P0	0.0159811429
0.40641256215	1.2789502983	1.0894432239	1.5014218545	FGF23_	0.0160730441
0.42450793562	1.2927503173	1.0931200297	1.5288379478	IGFBPL	0.0163694105
0.42785635560	1.2951048750	1.0934381501	1.5339657190	INHBB_	0.0166400760
0.35446042690	1.2384636860	1.0760348722	1.4254113331	SPINK2	0.0172877973
0.36643672416	1.2473083135	1.0784653263	1.4425851168	COL15A	0.0174413425
0.37673196010	1.2546060362	1.0799462331	1.4575135852	GSTA3_	0.0180968645
0.43986929553	1.30309663	1.0937559615	1.5525042860	CTSS_P	0.0181283122
-0.119964751	0.7017052573	0.5551489083	0.8869517002	RGMA_	0.0181283122

0.38299815993	1.25885125188	1.08047530388	1.46667533122	MMP7_	0.01867308299
0.38216359872	1.25810067922	1.08008827488	1.46545181155	AHSG_	0.01879289700
0.37991666977	1.25636638822	1.07953544177	1.46216274200	SIT1_Q	0.01879289700
0.49524445218	1.34631921906	1.10462319577	1.64089931144	CD72_P	0.01892656677
0.40038468089	1.27138540044	1.08310256044	1.49239868444	TFPI2_	0.0194473091
-0.075805815	0.79525525399	0.68223679900	0.92699619822	ARHGA	0.01969360000
0.31785466148	1.20977662122	1.06504473455	1.37417652599	CD70_P	0.01969360000
-0.071770697	0.80517429599	0.69654538966	0.93074429388	WFIKK	0.01969360000
0.36661753346	1.24550334711	1.07515189866	1.44284597322	REG1B_	0.01986171699
-0.076136774	0.79297273700	0.67855068477	0.92668945122	TCL1B_	0.0203139301
0.36537131759	1.24399859511	1.07389305255	1.44104899566	TYRO3_	0.02073199333
0.35496470762	1.23611779788	1.07142186566	1.42613032188	FAM3B	0.02097582733
0.32270667585	1.21239973400	1.06449085866	1.38086025188	LRIG1_	0.02115405233
-0.0860214	0.76705457499	0.64122603666	0.91757459511	PODXL	0.02115405233
0.38283772879	1.25612546233	1.07597387122	1.46644004977	LILRB1	0.02197017199
0.40093665183	1.26977141088	1.07975821	1.49322267255	VWF_P	0.02197017199
0.43174762498	1.29320255922	1.08599418477	1.53994642211	DSC2_	0.02198395600
0.38618724487	1.25830338166	1.07609778655	1.47136014966	KLK8_	0.02240454900
0.43518557301	1.29531729500	1.08580949622	1.54524978900	CA4_P2	0.0225081421
-0.127139556	0.67072801388	0.51086823377	0.88061076977	PLTP_P	0.0225081421
0.42592111009	1.28827869966	1.08403789611	1.53099998999	TNFSF1	0.0225081421
-0.07522157	0.78922287955	0.67153344422	0.92753794900	AMY2A	0.02255156744
-0.074135515	0.79066508533	0.67325837999	0.92854585377	MYL6B	0.02313401733
0.39999059083	1.26788987444	1.07757959900	1.49181066088	OSMR_	0.02331911377
0.32990248433	1.21620494355	1.06350295311	1.39083249388	CA5A_	0.02332294399
0.32484144626	1.21251779844	1.06242772877	1.38381122011	LCP1_P	0.02334158144
0.36858638022	1.24416411466	1.07073084	1.44568951422	C9_P02	0.02362100699
0.44164080932	1.29918405366	1.08527349399	1.55525700622	FOLR2_	0.02362100699
0.34025704960	1.22345106444	1.06512713099	1.40530877822	OGN_P	0.02362100699
0.36947433546	1.24427809933	1.06997652433	1.44697379199	GNAS_	0.02454069377
0.42442665274	1.28495405788	1.08006289700	1.52871368455	VWA1_	0.02519356522
0.44349659625	1.29935916599	1.08355336266	1.55814591166	FETUB_	0.02536137422
0.35872273174	1.23580638777	1.06686524755	1.43149983700	EPHA1_	0.02552081800
0.41676423024	1.27877130511	1.07792205833	1.51704479744	GLA_P	0.02562538000
-0.081444858	0.76275388733	0.63116064588	0.92178353711	SCG3_	0.02700243499
0.37164456009	1.24439271055	1.06785364688	1.45011746000	GUSB_	0.02708228744
0.46215024696	1.31196007655	1.08425625666	1.58748379999	CLEC10	0.02770846366
0.42704602836	1.28519973499	1.07764947	1.53272320888	ULBP2_	0.02770846366

-0.062677157	0.809322042	0.697369712	0.939246653	PLXDC	0.028196192
0.352737410	1.229711498	1.062709485	1.422957440	F7_P08	0.0288496501
0.437447386	1.292116678	1.07800923	1.548748810	ROBO1_	0.0291286501
0.427720164	1.284129095	1.075129715	1.533756821	CXADR	0.030260399
-0.045836068	0.853191389	0.762077745	0.955198536	IL1RAP	0.030525376
0.387674309	1.253795610	1.066813926	1.473549785	IGF2R_	0.031433442
-0.071837813	0.777311265	0.649215216	0.930681827	ENPP6_	0.031633779
-0.067903267	0.787902561	0.664408284	0.934350852	CST1_P	0.031659236
0.366872225	1.238441988	1.062724647	1.443213501	FBP1_P	0.0316806481
-0.071273296	0.778259194	0.650432329	0.931207362	SPOCK	0.0316806481
0.341432612	1.219986104	1.05785823	1.406961778	MYOM	0.032035700
0.313731738	1.200460190	1.053036833	1.368522565	PLXNB	0.032035700
-0.053700591	0.824981414	0.718141789	0.947715818	NPTX2_	0.033344620
0.409804888	1.268459930	1.068015373	1.506523816	L1CAM	0.0341720561
0.433565041	1.285877513	1.071776668	1.542747690	HYAL1_	0.034472425
0.350983291	1.225518728	1.057328158	1.420463591	ADH4_	0.034984953
0.343527793	1.219970164	1.055616559	1.409912708	MYBPC	0.035630192
0.354795260	1.227876342	1.057361857	1.425888688	ACY1_	0.035632598
-0.051118139	0.828360143	0.722168789	0.950166412	GPC5_P	0.035632598
0.406922922	1.265435681	1.065996483	1.502188316	IL15_P4	0.035632598
0.420314396	1.27517378	1.068067063	1.522440130	EPHB4_	0.035755899
-0.069577434	0.770910951	0.637126290	0.932787901	CSNK1	0.037044502
-0.059869323	0.798123822	0.676303233	0.941887609	EBI3_IL	0.037717217
0.398891881	1.258483279	1.062816666	1.490172495	AGR3_	0.037799203
0.339704008	1.216249337	1.053206805	1.404531800	HSPB6_	0.037799203
0.382566864	1.246539164	1.059900697	1.466042897	CLMP_	0.0379219571
0.380188895	1.244864320	1.059571088	1.462560834	FSTL1_	0.0379219571
0.421656883	1.274829882	1.066058929	1.524485359	C3_P01	0.038038617
0.434332456	1.283969839	1.067779197	1.543932072	CST7_O	0.038376369
0.414478947	1.269257850	1.064372872	1.513581878	IL18_Q	0.038563905
-0.050089923	0.825622445	0.716665931	0.951143891	LRRN1_	0.038563905
0.246854105	1.152532685	1.037765253	1.279992355	PROK1_	0.038575739
-0.056099967	0.805571776	0.686392279	0.945444617	HPGDS_	0.0391493601
0.332425949	1.209999634	1.050025201	1.394346643	SIGLEC	0.040462697
0.385041853	1.246956646	1.057989011	1.469675831	THBS2_	0.040638962
0.240701602	1.147818574	1.035645492	1.272141375	CSF2RE	0.041103758
-0.053743435	0.808707238	0.690117655	0.947675215	ST3GAI	0.041390082
0.407975439	1.262798605	1.060441475	1.503770226	TNFRSI	0.041972072

0.3601508129	1.228469949	1.052731369	1.433545595	S100A1	0.042627848
0.3652462785	1.231984302	1.053382029	1.440868819	BLNK_	0.042714281
-0.057416578	0.792988372	0.665992504	0.944200654	AIDA_	0.043296617
0.3305823813	1.207589908	1.047776963	1.391778438	HRG_P	0.043296617
0.4123720238	1.265134658	1.059699214	1.510396235	LIF_P15	0.043562361
-0.058392112	0.784525063	0.652488734	0.943280003	ART3_	0.046077820
0.2496984949	1.152468107	1.034701677	1.283638334	GC_P02	0.046077820
0.4029934459	1.257209252	1.056324389	1.496297084	PALM_	0.046412667
0.2159321779	1.130450071	1.029732969	1.241018207	CFHR2_	0.046491529
-0.04382683	0.830714403	0.721003264	0.957119687	UMOD_	0.047584458
0.3972627898	1.252438942	1.054348265	1.487746843	IL1B_P	0.048009929
0.4106440289	1.261517927	1.055471273	1.507788531	PGLYR	0.049157219
0.4106884164	1.261321842	1.055096348	1.50785546	CTHRC	0.049677927
0.4049016045	1.256790514	1.053608476	1.499154982	EPHA2_	0.050484811
0.3066096117	1.188866723	1.040177560	1.358810400	FGL1_	0.050484811
0.3781633906	1.237976662	1.050003246	1.459601408	LEP_P4	0.050484811
0.1906968390	1.113612721	1.024825167	1.210092543	LILRB5	0.050484811
0.2764686220	1.168846540	1.036206218	1.318465581	PSPN_C	0.050484811
0.3394459694	1.211177465	1.044710724	1.404169422	REG1A_	0.050484811
0.3475020538	1.216215230	1.044967181	1.415527217	LDLR_	0.051781920
0.3593248293	1.224296201	1.046454172	1.432361999	CCL14_	0.051809039
0.3890000308	1.244881613	1.050305255	1.475504596	DDAH1	0.051828594
0.4964131453	1.322142323	1.064061979	1.642818140	CSF3R_	0.052525495
0.4185942370	1.264879354	1.052701011	1.519823541	PI3_P19	0.054240146
0.3468268087	1.214841077	1.043311434	1.414571712	SELPLG	0.054452782
0.4403043384	1.280047476	1.054946440	1.553179839	MMP8_	0.054919343
0.3574732521	1.221753795	1.044043833	1.429712324	CES1_P	0.055503605
0.3755086523	1.234028903	1.046090669	1.455731687	FCAMR	0.055823554
0.3235937777	1.198486840	1.039277553	1.382085759	C1QL2_	0.056299337
0.4779553617	1.306639466	1.058615301	1.612773490	CD163_	0.056299337
-0.042036492	0.819608741	0.700598784	0.958834790	BOC_Q	0.056757681
0.3579411710	1.22156718	1.043236644	1.430381470	STC1_P	0.056757681
-0.045802032	0.803607126	0.676050484	0.955231048	NXPH3_	0.057542933
0.3700446786	1.229360591	1.043879124	1.447799298	REN_P0	0.058171534
0.3536357773	1.218053493	1.041719171	1.424236352	ANK2_	0.058312176
0.3359173753	1.205995832	1.039452269	1.399223409	FTCD_	0.058312176
0.3524484636	1.217212658	1.041517322	1.422546341	KIF1C_	0.058312176
-0.042265694	0.815061307	0.693004908	0.958615048	SCT_P0	0.058312176

0.3740493325	1.231787220	1.043815705	1.453608858	ADAM9	0.058475999
-0.04068523	0.820749993	0.701602530	0.960131302	TAGLN	0.058475999
0.3645897382	1.225248100	1.042578499	1.439923141	S100P_I	0.058563573
0.3569838487	1.219969929	1.041506863	1.429012789	CDH1_I	0.058786995
0.3716274368	1.229826750	1.043018774	1.450092629	ST6GAI	0.059127185
0.4053822348	1.252827475	1.046471177	1.499875695	CD300A	0.060047751
-0.036920641	0.831798241	0.717910701	0.963752614	SPARCI	0.060443419
0.3851675607	1.238491388	1.043541766	1.469860591	RSPO3_	0.060927322
0.3982880357	1.247138705	1.044371999	1.489272931	CD5_P0	0.062159341
0.3340630300	1.203474715	1.037032125	1.396631170	IL19_Q	0.062159341
0.5683960694	1.369354965	1.062137654	1.765433147	PCOLCI	0.064410627
0.3746993953	1.229959803	1.040044584	1.454554103	NRP1_C	0.065371534
0.3376411961	1.204877899	1.035739091	1.401637500	FEN1_P	0.065866104
0.3638912790	1.222436255	1.038523836	1.438917765	LTBR_I	0.065866104
-0.038513633	0.813513604	0.687790065	0.962218586	GPHA2_	0.066556505
0.3465038641	1.210524526	1.036245052	1.414114958	PTP4A3	0.066556505
0.3450804458	1.209521114	1.036001478	1.412103512	VSTM2I	0.066631107
-0.035810742	0.824251840	0.704161471	0.964822876	CLEC4A	0.066828545
0.3895199033	1.239150470	1.040115929	1.476271870	P4HB_P	0.067676032
0.3724701850	1.226969329	1.037303080	1.451315207	CILP_O	0.069919922
0.3771757688	1.229959737	1.037472121	1.458160586	PCBD1_	0.070489800
-0.040941234	0.793479893	0.655922313	0.959885536	ESM1_C	0.070743470
0.3666187189	1.222474775	1.035760456	1.442847683	EZR_P1	0.071736576
0.3434182750	1.206846714	1.033140919	1.409758305	ACTN2_	0.072319573
-0.035888111	0.812937189	0.685014858	0.964748231	MAPRE	0.072319573
0.3690632442	1.22341708	1.034825084	1.446379075	CXCL14	0.074018919
0.3375216625	1.202504826	1.031786546	1.401469967	FGR_P0	0.074018919
0.4076499492	1.249257106	1.038158188	1.503280844	SIGLEC	0.074658393
0.3563147662	1.214688281	1.033199403	1.428056981	CRLF1_	0.074683793
-0.030094016	0.835576524	0.719518766	0.970354300	BLMH_	0.074743293
0.3779835333	1.228706014	1.034522177	1.459338912	PARP1_	0.076142344
0.3860214784	1.233805250	1.034775721	1.471116267	CXCL16	0.077170185
0.3453606734	1.206707920	1.030898937	1.412499278	NPDC1_	0.077429578
0.3261215372	1.193793542	1.028550610	1.385583758	ADH1B_	0.079020863
-0.031654217	0.818326668	0.691195116	0.968841533	NXPH1_	0.079453028
0.3647714278	1.218752707	1.031366376	1.440184784	ACAA1_	0.080311089
0.3708068211	1.222511462	1.031493566	1.448903148	CCDC8	0.081181973
-0.025753976	0.842389870	0.728133614	0.974574829	MEPE_	0.083490185

-0.028472251	0.8261080071	0.7021647198	0.9719292641	CSNK2A	0.0839736165
-0.029033701	0.8224046310	0.6962741479	0.9713837274	FGF19_	0.0841217518
0.3733042985	1.2231930009	1.0300681950	1.4525262742	CCL19_	0.0844368736
0.3162290138	1.1861444412	1.0255070258	1.3719444139	DPT_Q0	0.0844368736
0.3421452123	1.2028073523	1.0275438647	1.4079647365	GFER_P	0.0844368736
0.4531016455	1.2761493731	1.0351981295	1.5731840855	OLR1_P	0.0873909931
-0.025221582	0.8314221559	0.7089192688	0.9750938248	DHPS_P	0.0898476621
0.3297690985	1.1936681704	1.0245905052	1.3906469889	MVK_Q	0.0898476621
0.3585897589	1.2122038951	1.0266390911	1.4313094991	PRSS22	0.0898476621
0.3698703158	1.2195644575	1.0274882893	1.4475468786	SNRPB2	0.0898476621
-0.019923092	0.8627364003	0.7592918407	0.9802740612	CRISP2	0.0906912058
0.2995728548	1.1741711842	1.0217861184	1.3492823450	CXCL9_	0.0908950616
-0.024670389	0.8318243303	0.7092142478	0.9756314382	KAZAL	0.0908950616
-0.02378056	0.8330730392	0.7107124533	0.9764999690	SMS_P5	0.0930485947
-0.025031321	0.8245489005	0.6971139902	0.9752793644	PAFAH	0.0931370264
0.3396291980	1.1990652128	1.0237325694	1.4044267297	IL2_P60	0.0932830364
0.3258889452	1.19022763	1.0226529701	1.3852615205	ADAMT	0.0934386421
-0.026539726	0.8131600854	0.6790131143	0.9738093572	KLK6_C	0.0934386421
0.3116704799	1.1811338644	1.0215072967	1.3657045918	KLRK1_	0.0935117239
0.3599595502	1.2119635584	1.0248272785	1.4332714378	RNASE	0.0935117239
0.3503769347	1.2056752444	1.0239857626	1.4196025453	SLAMF	0.0938415389
0.3095872290	1.1792077936	1.0203018087	1.3628624479	SORCS2	0.0966630197
0.4151447513	1.2472884007	1.0271614047	1.5145899637	CD59_P	0.0966674441
0.2963697498	1.1708973604	1.0193560550	1.3449673663	SEMA3	0.0966674441
0.5924377187	1.3705837689	1.0387684167	1.8083913962	GHR_P1	0.0968577776
-0.022212045	0.8302964587	0.7048763506	0.9780328262	UBE2B_	0.0972258108
-0.023850621	0.8191959402	0.6872801104	0.9764315573	UNC5D	0.0972258108
-0.017553871	0.8617511907	0.7557659710	0.9825993009	CNTN2_	0.0979474829
-0.020812639	0.8365250370	0.7144909000	0.9794024494	MATN3	0.0986207724
0.3312283050	1.1921775099	1.0205428029	1.3926771113	AKR1C	0.0989998898
-0.020898532	0.8336589168	0.7096642315	0.9793183294	HDAC8	0.0991072543
0.3528712213	1.2058612533	1.0217500251	1.4231478605	LCAT_P	0.0991072543
0.3315372554	1.1920161818	1.0199514544	1.3931080461	CCL23_	0.1004325971
0.3668935914	1.2141100139	1.0213538261	1.4432443374	CCL2_P	0.1025146957
0.3661460707	1.2135378072	1.0211543792	1.4421658855	EGLN1_	0.1025750304
0.3861740246	1.2264959095	1.0223955730	1.4713406981	GRPEL1	0.1025750304
0.2334914842	1.1313158652	1.0133598470	1.2630020724	KIR3DL	0.1027513454
0.3616938662	1.2105240539	1.0206226378	1.4357593402	DEFB4A	0.1027971365

0.35368359381	1.20542953700	1.02018944200	1.42430445640	PROS1_	0.10279713650
0.31499424240	1.18091458200	1.01773968490	1.37025142150	F10_P00	0.1030871961
0.33213647626	1.19165529990	1.01872334570	1.39394307560	SBSN_0	0.1030871961
0.33683875360	1.19433008380	1.01850116870	1.40051321780	DCXR_0	0.1044989237
-0.019435634	0.82134417870	0.68784590430	0.98075201980	TNFSF1	0.1071980679
0.34157939625	1.19669576950	1.01770396040	1.40716831270	KYNU_0	0.1076499514
0.38526823290	1.22439241910	1.01981500190	1.47000857320	PRND_0	0.1079642217
0.39790281020	1.23243142	1.02027802050	1.48869933640	GAS6_0	0.1083132839
0.30441299436	1.17314036460	1.01506784900	1.35582889010	TNFRSI	0.1096741514
-0.015689896	0.84416560150	0.72388460060	0.98443254930	FAM171	0.1101139509
0.35491034816	1.20447447150	1.01732471080	1.42605280030	LRPAP1	0.1101139509
0.30494454972	1.17327658660	1.01476405010	1.35654977980	NCF2_P	0.1102964272
0.36122956744	1.20777671530	1.01646703200	1.43509287360	CDON_0	0.1131966416
0.39850566631	1.23151088440	1.01814046250	1.48959707850	IGFBP7_	0.1131966416
0.34564490810	1.19799507600	1.01577703450	1.41290081710	SEMA7A	0.1131966416
0.32443236222	1.18466655130	1.01459581880	1.38324524080	DLL4_0	0.1135211728
0.33101391298	1.18818501390	1.01393619170	1.39237916430	XCL1_P	0.1168049870
0.35606038649	1.20370456630	1.01485677430	1.42769375900	SRP14_	0.1170628295
0.33068165967	1.18784410880	1.01369119970	1.39191661850	EPHA10	0.1171997383
0.26946068340	1.15055692250	1.01109259950	1.30925815560	SPINK4	0.1171997383
0.36562147044	1.20946959720	1.01485156230	1.44140952320	TGFB1_	0.1177388562
0.31426868516	1.17723416720	1.01213993510	1.36925758630	ENO3_F	0.1194525152
0.31475376508	1.17753836000	1.01217196530	1.36992194680	FCGR3E	0.1194525152
0.32542475362	1.18410750670	1.01263304140	1.38461864280	KRT17_	0.1194525152
0.30732144814	1.17277719510	1.01149331250	1.35977799590	NT5E_P	0.1207443882
-0.012254015	0.83819027460	0.71122511780	0.98782076010	PGR_P0	0.1218669719
0.29813962921	1.16698738820	1.01076903630	1.34734990410	SMOC1_	0.1218669719
-0.011019477	0.84558191710	0.72293137300	0.98904101450	TMED4_	0.1241342715
0.30303180591	1.16951416670	1.01019667040	1.35395752760	CLGN_0	0.1245193336
0.31896728503	1.17898374360	1.01039200700	1.37570631790	AZU1_F	0.1251732921
0.27224953573	1.15089387530	1.00886739540	1.31291457950	BTD_P4	0.1251732921
0.24921966599	1.13730801950	1.00814146400	1.28302383880	FCRL3_	0.1251732921
0.33336799639	1.18761041130	1.01057397820	1.39566080210	C1QTNI	0.1258024620
0.32303421137	1.18131609130	1.01027653040	1.38131260660	HADH_0	0.1258024620
0.31424579512	1.17583943410	1.00976619510	1.36922624430	KLK13_	0.1263689932
-0.009596034	0.84515850620	0.72118027230	0.99044986110	CLEC2I	0.1281640385
-0.009995613	0.83059132310	0.69681231770	0.99005417750	CSPG4_	0.1299587231
0.29365744090	1.16286058090	1.00814149740	1.34132434210	ECM1_0	0.1299587231

0.28613542668	1.1582296932	1.0076793350	1.3312727329	FCN2_C	0.1308659064
0.30777479643	1.1710702874	1.0080939967	1.3603945887	MMP10_	0.1313354045
0.33072184887	1.1847370160	1.0083545029	1.3919725597	DPEP2_	0.1322224653
0.21626502898	1.1172295954	1.0054533405	1.2414313507	IL6R_P(	0.1322224653
0.33997997336	1.1902775144	1.0084283173	1.4049194544	IFNL1_(	0.1325851004
-0.009428856	0.8209136746	0.6802834110	0.9906154557	DSG3_P	0.1326181629
0.28025571231	1.1540944818	1.0063967361	1.3234681961	FCRLB_	0.1346620156
0.35577931527	1.1990668627	1.0073347329	1.4272925318	FST_P1!	0.1373832488
-0.00624395	0.8512769743	0.7292114612	0.9937755033	CD200_	0.1381687196
0.29202090008	1.1601296838	1.0050554263	1.3391310052	BCHE_I	0.1413320935
0.38193845943	1.2142175622	1.0062809589	1.4651219179	DEFA1_	0.1422756169
0.32667681649	1.1803896392	1.0050249398	1.3863533582	SCARF1	0.1434652737
0.32089014799	1.1768017102	1.0047216512	1.3783541576	CRIP2_I	0.1438573802
0.37563836702	1.2099812950	1.0055869840	1.4559205296	INHBC_	0.1438573802
0.27852342968	1.1515919988	1.0037743389	1.3211775598	ATRN_(	0.1451976412
0.28297411678	1.1540289837	1.0035507394	1.3270708125	NFASC_	0.1463286301
0.29633294396	1.1616824628	1.0034115689	1.3449178645	FCGR2I	0.1475098933
0.33243423591	1.1828011974	1.0033423801	1.3943581974	ENG_P1	0.1492943208
0.33481501624	1.1841605181	1.0032584810	1.3976818128	CLSTN1	0.1494770536
0.33548768138	1.1844850209	1.0031334148	1.3986223009	CANT1_	0.1497591561
-0.003117216	0.8415156154	0.7103594273	0.9968876371	ATP1B4	0.1497850379
0.29845219153	1.1623556303	1.0024481239	1.3477711007	CD8A_I	0.1505650246
-0.002518592	0.8562637326	0.7350365075	0.9974845771	LYZL2_	0.1505650246
0.32867824258	1.1801563031	1.0026189609	1.3891308205	MAD1L	0.1506107596
0.32806727030	1.1796656833	1.0023977579	1.3882823593	DPY30_	0.1511724609
-0.002364877	0.8471425912	0.7193497334	0.9976379174	ICOSLG	0.1511724609
0.33429172872	1.1832779999	1.0022879910	1.3969506147	SCGB3A	0.1513875060
-0.002051049	0.8203647785	0.6743801391	0.9979510527	CST6_Q	0.1534395452
0.33673114909	1.1842422872	1.0014762395	1.4003625243	CALB2_	0.1540895754
0.29435729439	1.1593060756	1.0012867636	1.3422634011	SIRPB1_	0.1540895754
0.34129199718	1.1867699443	1.0011792659	1.4067639520	AIFM1_	0.1548524735
0.33285441381	1.1817811614	1.0011918143	1.3949441990	BCAM_	0.1548524735
0.19415067271	1.1022646733	1.0005832120	1.2142792279	CFHR4_	0.1552237766
0.29658368922	1.1602754691	1.0007314787	1.3452551386	APEX1_	0.1557211660
0.31050701887	1.1682654886	1.0005334443	1.3641165716	CLIC5_(	0.1559704125
0.30683862507	1.1661506315	1.0005780743	1.3591216222	PKD2_C	0.1559704125
0.33500998529	1.1827522285	1.0006784841	1.3979543441	SCARA!	0.1559704125
0.33525505126	1.1827614905	1.0004489506	1.3982969771	ALDH1/	0.1562170229

-0.000354624	0.8585178961	0.7373144007	0.9996454392	GDF2_C	0.1562170229
0.3251139726	1.1761010363	0.9992957974	1.3841883960	CYB5R	0.1604066030
0.0007424171	0.8533782886	0.7277140355	1.0007426928	DCUN1	0.1604066030
0.3086832886	1.1664617954	0.9992671011	1.3616310582	PDIA3_	0.1604066030
0.0008207801	0.8487947617	0.7198614570	1.0008211170	SEZ6L2	0.1604066030
0.2818413912	1.1507288277	0.9989501700	1.3255684565	CTSH_F	0.1619223651
0.3104942006	1.1671678921	0.9986671072	1.3640990862	IL12RB	0.1624738471
0.3768494556	1.2062639296	0.9982079949	1.4576848465	PSAPL1	0.1628718484
0.3341080039	1.1805062032	0.9977811258	1.3966939839	ANXA2	0.1649546627
0.3351909602	1.1811457130	0.9977813260	1.3982073617	COCH_	0.1649546627
0.3643390568	1.1983426256	0.9975428806	1.4395622248	PDCD1_	0.1649546627
0.3481654928	1.1884749502	0.9971803515	1.4164666454	FN1_P0	0.1663922458
0.0024586535	0.8577924683	0.7340010441	1.0024616784	IVD_P2	0.1663922458
0.3070058777	1.1643891344	0.9973907351	1.3593489579	GLB1_F	0.1666992326
0.2911519471	1.1551098007	0.9972426712	1.3379678689	HS6ST1	0.1678199565
0.3511831152	1.1898925538	0.9965488778	1.4207474626	KLF4_C	0.1678199565
0.2487035944	1.1310309957	0.9975585954	1.2823618775	MAN2B	0.1678199565
0.0037357229	0.8404392850	0.7037044306	1.0037427094	IDS_P2	0.1687037455
0.3424608780	1.1845099291	0.9962045970	1.4084092528	ISLR2_	0.1691205145
0.0040605158	0.8383508244	0.6999840302	1.0040687708	MXRA8	0.1692498088
0.3399709826	1.1827745981	0.9957640798	1.4049068232	CREG1_	0.1706084340
0.3528054467	1.1901693199	0.9953963479	1.4230542567	BAG3_	0.1712066734
0.3345268368	1.1793835150	0.9954671816	1.3972790877	BNIP3L	0.1716693655
0.3626806016	1.1958223120	0.9950000916	1.4371767539	RSPO1_	0.1717389788
0.3065214469	1.1630582787	0.9955942529	1.3586906069	S100A1	0.1723263249
0.0047353143	0.8526363832	0.7235544193	1.0047465436	DSG2_C	0.1723799609
0.0043747635	0.8686791317	0.7513094327	1.0043843467	RARRE	0.1730898335
0.2985539502	1.1582743080	0.9953195015	1.3479082552	IDI2_Q	0.1734224058
0.0051027631	0.8592470335	0.7345476625	1.0051158044	STX3_C	0.1743766500
0.3473035475	1.1860705319	0.9940060269	1.4152462547	TPK1_C	0.1752475691
0.0059014457	0.8523823216	0.7222805206	1.0059188935	DKK3_	0.1762092664
0.5302745939	1.2971563680	0.9901234216	1.6993988892	CCL17_	0.1767933115
0.3822118980	1.2062173572	0.9927928230	1.4655225936	FCER2_	0.1768128577
0.0055831193	0.8671110734	0.7476954656	1.0055987340	DKK4_	0.1769393193
0.0058116794	0.8650034034	0.7438950215	1.0058285999	DLG4_F	0.1773160033
0.3314663794	1.1763624577	0.9934094627	1.3930093117	IL1R1_F	0.1774853688
0.3078083797	1.1626648485	0.9936412305	1.3604402760	IL15RA	0.1783697105
0.2813866033	1.1475412026	0.9938753666	1.3249657411	LEG1_C	0.1797646185

0.25191766047	1.13105874733	0.99440631999	1.28649010399	KIR2DL	0.1801311032
0.29948908674	1.15742607860	0.99293328477	1.34916932299	ANXA1_	0.1812860337
0.31403761505	1.16565380333	0.99255450933	1.36894122833	FABP5_	0.1812860337
0.00724552579	0.86089176600	0.73578412977	1.00727183811	IL25_Q9	0.1812860337
0.31793854321	1.16789439122	0.99249468711	1.37429179900	MMP1_	0.1812860337
0.32745089323	1.17321147122	0.99207038499	1.38742691755	PTS_Q0	0.1817779798
0.29740678908	1.15601923300	0.99258564911	1.34636287377	CXCL13	0.1825696333
0.00762411332	0.86317892760	0.73941890288	1.00765325088	NRCAM	0.1826275454
0.30849644579	1.16198317760	0.99179377300	1.36137667099	DDT_P3	0.1845689192
0.34190328109	1.18090911100	0.99070929655	1.40762414700	BAIAP2	0.1851795622
0.31986361007	1.16818880200	0.99108539655	1.37693950577	TIMP3_	0.1859524952
0.30363689041	1.15892880299	0.99139263244	1.35477703422	CD274_	0.1863869135
0.28249876749	1.14691347644	0.99168479799	1.32644014022	MB_P02	0.1877716252
0.18895779964	1.09591250111	0.99423359144	1.20798997388	SIRPA_	0.1890991305
0.31341439819	1.16353431744	0.98956482660	1.36808834699	CSF2RA	0.1932879251
0.01005916184	0.86721526833	0.74453512711	1.01010992522	MCTS1_	0.1936596164
0.28580907351	1.14801289888	0.99030331299	1.33083833877	SCN4B_	0.1936596164
0.31797057594	1.16591648122	0.98910413260	1.37433582200	REG3G_	0.1939102428
0.35342887369	1.18594253544	0.98772280760	1.42394170360	IL13RA	0.1943851419
0.20180481291	1.10224190255	0.99291281844	1.22360915200	KIR2DS	0.1945351083
0.32072536628	1.16715787122	0.98848469577	1.37812704877	ENAH_(	0.1955972716
0.32033526299	1.16672797500	0.98814206011	1.37758954177	SUMF2_	0.1970716362
0.29190006314	1.15073847044	0.98896899855	1.33896919855	SMNDC	0.1979697380
0.30045473122	1.15540974000	0.98852172144	1.35047277011	PPT1_P	0.1983299817
0.33135865136	1.17251164000	0.98702258860	1.39285925360	SMPD1_	0.1996051068
0.37968525895	1.19988329600	0.98487882911	1.46182442088	SERPIN	0.2001062763
0.31434426764	1.16287055460	0.98751742300	1.36936108211	SOD2_P	0.2001062763
0.28719677764	1.14754870511	0.98813044122	1.33268643055	CLU_P1	0.2017890603
0.28242128618	1.14495018455	0.98836913960	1.32633736999	CTSC_P	0.2017890603
0.33955493153	1.17665823099	0.98590221233	1.40432243188	STC2_O	0.2018526204
0.01289834960	0.86379755477	0.73658396211	1.01298189211	FGFBP1	0.2021359249
0.22582433742	1.11417370360	0.99044769299	1.25335547822	LAIR2_(	0.2023232921
0.36260457579	1.18915440533	0.98400959177	1.43706749560	BDNF_I	0.2048534252
0.28836232102	1.14771048788	0.98725771388	1.33424063999	CEBPB_	0.2048534252
0.01285990908	0.87244403244	0.75143282977	1.01294295333	FGFBP3	0.2053851264
0.34561712194	1.17892200533	0.98371782177	1.41286155855	SORD_(	0.2091139547
0.01361953337	0.87310953211	0.75200819100	1.01371270177	CNTNA	0.2092582154
0.26972033640	1.13675892500	0.98673081600	1.30959815255	CCL25_	0.2113863875

0.0138602199	0.875869871	0.756588538	1.013956718	ICAM4_	0.211386387
0.2547540362	1.128685096	0.987432252	1.290144253	ITPA_Q	0.211386387
0.3405122476	1.175509973	0.983037411	1.405667456	NBL1_F	0.211636142
0.3312982488	1.170401330	0.983532267	1.392775124	SLAMF	0.211636142
0.3230406788	1.165540834	0.983467930	1.381321540	C2_P06	0.213539514
0.3506151449	1.180833608	0.981990277	1.419940749	PLAU_F	0.213588338
0.3188990869	1.163055701	0.983342739	1.375612500	F13B_P	0.214637961
0.2315260524	1.115593770	0.987328500	1.260522165	RNASE	0.217946406
0.3443527057	1.176704097	0.981259900	1.411076242	VEGFB_	0.217946406
0.3379449777	1.173033845	0.981416707	1.402063356	LAYN_	0.218075859
0.2829017620	1.142926114	0.984404608	1.326974796	NAGPA	0.218075859
0.0202507749	0.844236703	0.698447326	1.020457213	APOM_	0.219174284
0.0183762783	0.858608283	0.723784755	1.018546161	CHL1_C	0.219538888
0.2930165058	1.147742739	0.982728738	1.340464915	RUVBL	0.223554637
0.0179878858	0.867841179	0.739721885	1.018150642	CPLX2_	0.223561964
0.3015193307	1.152159829	0.981922648	1.351911248	LRCH4_	0.224625490
0.4842464679	1.255358159	0.971023477	1.622951601	PILRA_	0.224653731
0.3047471297	1.153669637	0.981325148	1.356281996	GCNT1_	0.226221201
0.2929047746	1.147178061	0.981871691	1.340315152	GLO1_C	0.226888673
0.3102784597	1.156410512	0.980554730	1.363804825	PDZK1_	0.227941368
0.3126690459	1.157577316	0.980188435	1.367069019	ATOX1_	0.228797541
0.2840781459	1.142011539	0.981674281	1.328536746	ITM2A_	0.230328079
0.3107386408	1.156123628	0.979617370	1.364432567	FCAR_F	0.231955859
0.2611567565	1.129581402	0.982689076	1.298431187	KLKB1_	0.232617867
0.2748505596	1.136629704	0.981458457	1.316333946	IFNAR1	0.234360045
0.2772561430	1.137824481	0.981159774	1.319504309	HAO1_C	0.234875017
0.0192213705	0.878070231	0.756329034	1.019407290	CXCL1_	0.234907618
0.3132794637	1.156922311	0.978482022	1.367903757	IL16_Q1	0.235600267
0.0208175260	0.871889597	0.744529749	1.021035722	GFRAL_	0.237183936
0.2623474657	1.129592665	0.981539250	1.299978161	DPP7_Q	0.237615985
0.3030352853	1.151088716	0.978613137	1.353962238	IFNG_P	0.237804908
0.2999758898	1.149324970	0.978605857	1.349826262	KDR_P3	0.238742168
0.0199170633	0.881953558	0.762503011	1.020116731	CPA2_P	0.240727218
0.0204452401	0.879883398	0.758526909	1.020655675	NOTCH	0.241270056
0.1486047858	1.071098942	0.988828425	1.160214365	CD33_P	0.243717356
0.2742307773	1.134569700	0.978510407	1.315518359	ERP44_	0.249658134
0.2767745251	1.135854868	0.978236895	1.318868965	ACE_P1	0.249785428
0.2532786761	1.123529834	0.979877277	1.288242229	CD200R	0.250473622

0.33336780728	1.16567160611	0.97358222588	1.39566053811	GABAR	0.25047362233
0.36072624794	1.18025954211	0.97116634000	1.43437074511	CA12_C	0.25150355800
0.27220340185	1.13298540644	0.97775984255	1.31285401100	SERPIN	0.25380661100
0.27594949945	1.13482317233	0.97726657611	1.31778131360	TMPRSS	0.25468256288
0.02542196580	0.87096519544	0.73953882955	1.02574785988	CALB1_	0.25557021755
0.02784372314	0.85973146660	0.71884171160	1.02823498255	GFRA3_	0.25557021755
0.27957942117	1.13653258722	0.97666131355	1.32257344899	TIMP2_	0.25557021755
0.32847719424	1.16204197822	0.97227204999	1.38885156611	SIAE_Q	0.25721002511
0.28690484504	1.13998986311	0.97544051060	1.33229743277	CBS_P3	0.25797474677
0.02493120959	0.87661373833	0.74953006622	1.02524459111	DCUN1	0.25797474677
0.34586129654	1.17112838588	0.97051748133	1.41320658555	LGALS7	0.25797474677
0.02445928943	0.87959639300	0.75499546880	1.02476087160	MSTN_(	0.25823918388
0.02546139613	0.87630595800	0.74860683100	1.02578830611	CNTF_F	0.25947111788
0.02745555249	0.86928661377	0.73519439744	1.02783592933	HMGCS	0.26142875200
0.02653902747	0.87378644855	0.74350664933	1.02689432355	FLT3_P	0.26157431455
0.26702023453	1.12913019155	0.97616363688	1.30606687377	AMN_Q	0.26166225233
0.37882930355	1.18813505333	0.96651398377	1.46057369977	CD63_P	0.26166225233
0.30533091804	1.14896173377	0.97276423899	1.35707400899	KLK7_F	0.26166225233
0.27714436563	1.13439091688	0.97535611722	1.31935682700	MAN1A	0.26166225233
0.35031850552	1.17264244699	0.96870117677	1.41951960144	CLEC4C	0.26188071699
0.36122968968	1.17802968011	0.96701320399	1.43509304900	MPO_P(	0.26521237044
0.05716125191	0.75861310311	0.54352041760	1.05882653444	CTSB_F	0.26643396955
0.28418472952	1.13735608533	0.97358315560	1.32867835422	XRCC4_	0.26686160811
0.29613581460	1.14304955544	0.97167262611	1.34465276799	STX4_Q	0.27155625377
0.02912239013	0.87465768311	0.74306796333	1.02955059355	CDC25A	0.27302389433
0.02793639925	0.88003474088	0.75312490588	1.02833027977	NCAM2	0.27348006133
0.32237502317	1.15565584044	0.96750082344	1.38040236177	DCTN6_	0.27987171611
0.30085739825	1.14455151544	0.96963879144	1.35101667044	KIF22_(	0.27987171611
0.03107965291	0.87318650111	0.73912229833	1.03156766799	PEAR1_	0.28007416160
0.28641506549	1.13702543622	0.97084942600	1.33164506044	GZMA_	0.28054964733
0.28623771542	1.13684310077	0.97071021688	1.33140891400	CLC_Q(	0.28059119860
0.03556485802	0.85790059899	0.71027793022	1.03620485211	PPY_P0	0.28059119860
0.32402142453	1.15629454788	0.96697721099	1.38267693000	TXNDC	0.28059119860
0.26898221846	1.12797705511	0.97226138599	1.30863187133	LGALS3	0.28093634060
0.23257790959	1.10974405711	0.97597423600	1.26184875260	PZP_P2(	0.28093634060
0.03452927248	0.86314390899	0.71973156160	1.03513232888	ITGB2_	0.28128976960
0.27654079663	1.13151235877	0.97099828233	1.31856074444	CSRP3_	0.28342087622
0.27393005204	1.13016704288	0.97122301899	1.31512280888	INSL4_(	0.28342087622

0.0286865719(	0.886980308(	0.764486000(	1.029101994	CTRC_(	0.2834395321
0.2855170177	1.135859097	0.969729161(	1.330449716	LILRA2	0.2845758421
0.0624613981	0.772496830	0.560617656	1.064453368	FABP9_	0.2846779561
0.2664232235	1.125879224	0.971130232	1.305287370	SERPIN	0.287982593
0.3230021470	1.154524273	0.965001718	1.381268316	TIE1_P	0.288206280(
0.0312316944	0.882352597	0.754606574	1.031724521(	CEACA	0.288962320
0.0333622040	0.875769608(	0.741806643	1.033924963	GSN_P(	0.289993646
0.2732026588(	1.128768062(	0.969525013	1.31416655	GDNF_	0.292495078
0.2798885745	1.131799416	0.968244119	1.322982390	PTK7_C	0.2958158977
0.2938424354(	1.138604253	0.966343334	1.341572502(	LAT2_C	0.2968420177
0.3063970040(	1.144991110	0.965023083(	1.358521538(	NGFR_	0.2968420177
0.0203025777	0.925809291	0.839896502	1.020510076	PNLIPR	0.2968420177
0.3012140493(	1.142223443	0.965353864	1.351498597	TIMD4_	0.297453557
0.0678993739(	0.774987200(	0.561178128	1.070257607	GAL_P	0.297510628
0.2836033921	1.133310384	0.967231314	1.327906168	TFPI_P1	0.297510628
0.0317287983	0.888428148	0.764654022	1.032237522	IGFBP3	0.2976172752
0.0352520178	0.876531560	0.741695013	1.035880736	SH3BGI	0.2976172752
0.2824366527	1.132619133(	0.967179556	1.326357751	TRIAP1	0.2976172752
0.2747704686	1.128714288	0.967913946(	1.316228524	CFC1_P	0.298032864
0.3072566955	1.144878320(	0.964003865	1.359689949	ANPEP_	0.298783351
0.3048489449	1.143311618	0.963684822	1.356420093	PF4_P0	0.302130476
0.0310502873	0.894680571	0.775980923	1.031537375	TMEM1	0.303732243
0.0373555540(	0.875648105	0.738645258(	1.038062042	ADAM2	0.304684651
0.2953971150	1.138231580	0.964210652	1.343659840	CDHR5_	0.304684651
0.0287235219(	0.904022211(	0.794115613	1.029140020	C1QTNI	0.3061367287
0.3176006605(	1.149151699	0.961219367	1.373827528(	SLIT2_(	0.3061367287
0.2874756140	1.133640924	0.964055325	1.333058083	CCL5_P	0.310004613
0.2745138003	1.127255894	0.965662131	1.315890733	ENOX2_	0.310004613
0.3036186460(	1.141719456	0.962185708	1.354752317	NAMPT	0.310004613
0.3442313848	1.161631887	0.956399322	1.410905059	KIT_P1(	0.313646224
0.2766072897	1.127777279	0.964534269	1.318648422	IL36A_(	0.314762949
0.0378380277	0.881516850	0.748218410(	1.038563000	PNLIP_	0.314762949
0.3026617167(	1.140277046	0.960674915	1.353456535	LONP1_	0.317600915
0.0338971357	0.894973615	0.774281932	1.034478190	MENT_(	0.317600915
0.2990083466	1.138442591	0.961091187	1.348520879	PRTN3_	0.317600915
0.2742681690	1.126079670(	0.963884692	1.315567549	TCP11_(	0.319798279(
0.0336613129	0.897336957(	0.778560179	1.034234265(	HBQ1_I	0.320061170
0.0405333914	0.879081798	0.742087553	1.041366081	MYOC_	0.321447404

0.37832442137	1.17758937259	0.94991237775	1.45983646822	TCL1A_	0.32144740433
0.27342705507	1.12541275086	0.96355342906	1.31446147244	VIM_P0	0.32144740433
0.31417438937	1.14516468959	0.95783718477	1.36912847710	CD5L_C	0.32191539688
0.28442601814	1.13046597999	0.96159089922	1.32899898788	ERMAP	0.32191539688
0.28733517757	1.13192356759	0.96127162459	1.33287088700	FRZB_C	0.32191539688
0.03918124759	0.88382982510	0.75114037410	1.03995895660	GHRL_(	0.32191539688
0.03700984651	0.89018842888	0.76364360200	1.03770323855	GSTM4_	0.32191539688
0.26037423349	1.11881873460	0.96480682559	1.29741553210	IL1R2_F	0.32191539688
0.26720415513	1.12227488488	0.96416907559	1.30630710840	MFGE8_	0.32191539688
0.30474436741	1.14030231260	0.95871873222	1.35627824977	PFDN2_	0.32268805333
0.28432996622	1.13026652460	0.96134394444	1.32887134100	RNASE:	0.32285978610
0.27864947550	1.12744187430	0.96199406400	1.32134409922	TIMP4_	0.32319196460
0.30542633643	1.14047239770	0.95835096599	1.35720350499	DEFB11	0.32320772233
0.04360546560	0.87456399070	0.73222671599	1.04457015477	KIRREL	0.32396428722
0.30365624691	1.13945144560	0.95833073111	1.35480325822	CRIM1_	0.32402316388
0.26279340941	1.11947233533	0.96360047122	1.30055800810	OLFM4_	0.32492494022
0.27700162863	1.12634434860	0.96170547810	1.31916851944	PQBP1_	0.32492494022
0.04312144365	0.87770907444	0.73785966755	1.04406468210	EPO_P0	0.32507922788
0.27179552297	1.12370120577	0.96219345388	1.31231863488	JUN_P0	0.32507922788
0.02973898356	0.91394531244	0.81082091544	1.03018560344	PON1_F	0.32507922788
0.03457325322	0.90160571699	0.78526879644	1.03517785577	IL17RB_	0.32574930400
0.03860865736	0.89053850377	0.76302343410	1.03936365677	MEGF1(	0.32574930400
0.27389260873	1.12452474160	0.96158566799	1.31507356722	RAB6B_	0.32574930400
0.28904243004	1.13183562310	0.95948278044	1.33514837777	VASN_(	0.32574930400
0.26599416708	1.12062749344	0.96250445310	1.30472744822	CKMT1	0.32641342444
0.03665765184	0.89753359855	0.77657108199	1.03733782933	MIA_Q1	0.32841645066
0.04379925161	0.87920566377	0.73987641044	1.04477259744	FGF12_	0.32877125199
0.19303840992	1.08592238600	0.97221441477	1.21292938111	TPSAB1	0.32910407877
0.03600705772	0.90005140699	0.78144238560	1.03666316299	VCAN_	0.32910407877
0.27386827876	1.12368298277	0.96016998455	1.31504157199	TLR2_C	0.33319527466
0.04043658247	0.89224340299	0.76454896811	1.04126527311	ARHGA	0.33686482277
0.28289602657	1.12757381260	0.95814178144	1.32696718533	DPP10_(	0.33686482277
0.27408472057	1.12341336644	0.95950157500	1.31532623277	RBM19_	0.33686482277
0.04223590682	0.88825592799	0.75636845477	1.04314053360	RBP1_P	0.33686482277
0.23763260827	1.10603399922	0.96457149622	1.26824316511	FOLR3_	0.33720283811
0.04114397012	0.89148445060	0.76270913133	1.04200211199	HNF1A_	0.33720283811
0.03847546320	0.89877972155	0.77731464300	1.03922522888	LHB_P0	0.33836799722
0.24947055669	1.11137747960	0.96245292855	1.28334577799	ARSA_F	0.33920610177

0.04257619651	0.88968494760	0.75854592322	1.04349556400	FGF20_(	0.3400241055
0.2896623527	1.1302333520	0.9561752015	1.3359763231	LPO_P2	0.3408169504
0.2958960244	1.1331748037	0.9551856914	1.3443303720	BATF_(	0.3408426360
0.0235771249	0.9380477425	0.8594299219	1.0238572626	DKKL1_	0.3412151856
0.3021611571	1.1360413292	0.9540284795	1.3527792191	MDK_P	0.3412151856
0.2974160290	1.1337551160	0.9547119955	1.3463753142	SLAMF	0.3412151856
0.2641018687	1.1176355142	0.9591850537	1.3022608492	IDUA_P	0.3440517374
0.0413341200	0.8954865256	0.7694261292	1.0422002674	NEO1_(	0.3440517374
0.2666185002	1.1186232307	0.9584660294	1.3055422872	LY9_Q9	0.3458229735
0.2901638890	1.1297408517	0.9548630560	1.3366465317	RETN_(	0.3458229735
0.1557479919	1.0674709630	0.9751504986	1.1685316860	KLK12_	0.3494806177
0.2261650083	1.0994765081	0.9641612962	1.2537825327	PSG1_P	0.3494806177
0.2814314883	1.1250725825	0.9552937582	1.3250252135	COL3A1	0.3496794000
0.0433402472	0.8942419960	0.7657512134	1.0442931522	DEFB10	0.3496794000
0.0633426943	0.8494802670	0.6773251582	1.0653918804	EDAR_(	0.3496794000
0.2748505484	1.1219732760	0.9563105542	1.3163339320	EFCAB1	0.3496794000
0.3101126007	1.1386003353	0.9507414391	1.3635786454	CD93_Q	0.3498236940
0.0423298544	0.8977485482	0.7725485838	1.0432385389	EGFR_F	0.3515564393
0.2691394169	1.1190058625	0.9567070186	1.3088376024	BCL2_P	0.3519043591
0.0430470576	0.8967144826	0.7702172987	1.0439870212	AK2_P5	0.3522975496
0.0463590407	0.8911709786	0.7582084052	1.0474504208	PDE4D_	0.3568019298
0.2722906677	1.1198848931	0.9551958742	1.3129685834	IL17A_(	0.3571292885
0.0468888506	0.8904900075	0.7566491209	1.0480055174	PLA2G1	0.3571292885
0.2673680978	1.1176569971	0.9560940011	1.3065212854	SIGLEC	0.3571292885
0.2671744351	1.1174402376	0.9559082912	1.3062682855	KEL_P2	0.3575156074
0.0452121964	0.8952780260	0.7660911467	1.0462498469	MAP1L(	0.3587922532
0.0477421968	0.8899884207	0.7551522833	1.0489002100	TACSTI	0.3589621315
0.2517353589	1.1099834316	0.9578681113	1.2862555961	ASAH1_	0.3591039836
0.0487892666	0.8883274071	0.7515488481	1.0499990575	CDH3_I	0.3591039836
0.2767409550	1.1215544933	0.9537920312	1.3188246918	MATN2	0.3591039836
0.2945864291	1.1298976984	0.9509134437	1.3425709950	SLAMF	0.3591039836
0.2943841247	1.1294790073	0.9504010907	1.3422994150	NRP2_C	0.3616339376
0.0446012547	0.8988936486	0.7727634004	1.0456108443	NTF3_P	0.3616339376
0.0515564951	0.8840112740	0.7422067608	1.0529086688	RBM17_	0.3616339376
0.0461606218	0.8961344000	0.7668298226	1.0472426075	NT5C3A	0.3628090467
0.2632538831	1.1148211138	0.9551699739	1.3011570189	KLK11_	0.3630508144
0.0555980619	0.8785672119	0.7301364858	1.0571726804	HRC_P2	0.3674393442
0.0470622911	0.8975243038	0.7685171115	1.0481872998	GABAR	0.3698787685

0.27356250294	1.11887220599	0.95225724544	1.31463952544	IL13_P3	0.36987876855
0.04817020307	0.89540011555	0.76403673288	1.04934924255	ITGB6_	0.36987876855
0.17382985724	1.07402383488	0.96947026000	1.18985310377	TDGF1_	0.36987876855
0.24868862960	1.10736764133	0.95626785677	1.28234268733	ENPP2_	0.37038224422
0.28354281336	1.12329307660	0.95026576877	1.32782572777	MILR1_	0.37038224422
0.04539878153	0.90172055955	0.77701160177	1.04644507960	PRL_P0	0.37038224422
0.26386083953	1.11418603733	0.95350311633	1.30194700411	TMPRSS	0.37046732311
0.25785336651	1.11144623133	0.95453667833	1.29414903911	TOR1AI	0.37046732311
0.25826967925	1.11142131800	0.95409660133	1.29468792200	CAMKK	0.37200467744
0.24356545531	1.10475527111	0.95664990244	1.27578982233	FAM3D	0.37200467744
0.63831386887	1.29840986311	0.89044565844	1.89328585822	NCR3LC	0.37200467744
0.04900443757	0.89648530960	0.76525116211	1.05022501111	BMP10_	0.37385096588
0.04155191983	0.91200576577	0.79790171488	1.04242728311	CLPS_P	0.37509307200
0.21089879598	1.08982008122	0.96187232360	1.23478738322	IL17RA	0.37517839888
0.25939978867	1.11134208155	0.95288309460	1.29615188811	IL3RA_	0.37714340500
0.28714696270	1.12397059299	0.94798956311	1.33262004455	LSM1_C	0.37714340500
0.19637490245	1.08320413444	0.96413107622	1.21698306977	PDCD6_	0.37714340500
0.03384956497	0.92905975055	0.83442366399	1.03442898060	SPINT3	0.37849223611
0.15741255676	1.06603790322	0.97091651433	1.17047840300	CCL24_	0.37899849866
0.29736700696	1.12823003688	0.94547590444	1.34630931360	ICAM3_	0.38054082555
0.05316003035	0.89217235111	0.75476267044	1.05459839933	GH2_P0	0.38085752300
0.26499855853	1.11331449622	0.95092949088	1.30342909699	DOCK9	0.38172660555
0.05344298055	0.89212462611	0.75446841622	1.05489684044	GLP1R_	0.38172660555
0.71826135021	1.33707562560	0.87171597060	2.05086437411	MST1_F	0.38370460399
0.28838245357	1.12356103960	0.94612917422	1.33426750199	MFAP5_	0.38493112266
0.26810838331	1.11432789560	0.94970344588	1.30748884233	MYDGF	0.38535292355
0.25049188224	1.10634575400	0.95278410800	1.28465716133	ANGPT	0.38569958700
0.28261940373	1.12077252844	0.94687992055	1.32660016660	CD46_P	0.38569958700
0.05352245354	0.89452397160	0.75847183877	1.05498067955	ENTPD2	0.38570709277
0.05637850570	0.88915632060	0.74725936433	1.05799806622	RPE_Q9	0.38570709277
0.05005568809	0.90119261244	0.77249617322	1.05132964122	ALDH2_	0.38585234600
0.05103676367	0.90003188955	0.76975197188	1.05236158122	LRFN2_	0.38751835000
0.05123698612	0.90011711177	0.76974362611	1.05257230877	IGLON5	0.38850990977
0.05121300883	0.90058050233	0.77055484100	1.05254707122	PSMA1_	0.38939138211
0.30310821621	1.12907270788	0.94146806611	1.35406098788	THPO_F	0.39370772777
0.04683911519	0.91081087511	0.79161578533	1.04795339577	ADIPOC	0.39517534277
0.05691188829	0.89250719655	0.75250074500	1.05856253455	B3GAT3	0.39517534277
0.05174876553	0.90195130460	0.77248842177	1.05311131344	NTF4_P	0.39517534277

0.05533946373	0.8958445090	0.7593319056	1.0568993327	CELA2A	0.3957408496
0.2398176099 7	1.1004973039	0.9528543065	1.2710173083	DYNLT	0.3959966209
0.3270588285 0	1.1393349722	0.9359723350	1.3868830630	CXCL8_	0.3965668370
0.0416901666 5	0.9209219140	0.8134667491	1.0425714053	MMP3_	0.3965668370
0.2797262779 5	1.1180170840	0.9449597302	1.3227676921	SPINT2_	0.3965668370
0.2567118472 9	1.1077293432	0.9492460126	1.2926725859	RNF43_	0.3970944174
0.0607419615 9	0.8891448945	0.7439867130	1.0626246808	GCG_P(	0.4013942357
0.0537814040 8	0.9018245751	0.7707032043	1.0552539027	ATP1B1	0.4030338259
0.2710343683 1	1.1133774789	0.9453140962	1.3113201374	GOT1_F	0.4044903097
0.0514910975 0	0.9074051603	0.7820602095	1.0528398133	CHRDL	0.4078016567
0.0555368030 5	0.9016500827	0.7690538074	1.0571079211	PLA2G1	0.4107679968
0.0569817141 3	0.8992184214	0.7638068459	1.0586364521	SPACA5	0.4107679968
0.2576424780 6	1.1067805290	0.9467391015	1.2938761468	COPE_(	0.4121082631
0.0503433556 2	0.9114387180	0.7899345430	1.0516321182	AGT_P(	0.4127824679
0.2797006183 6	1.1163373397	0.9421465621	1.3227337508	CD36_P	0.4127824679
0.2824292484 0	1.1174661109	0.9414803463	1.3263479305	TGFB2_	0.4127824679
0.0549966713 7	0.9044734653	0.7742958115	1.0565370978	IL20RB_	0.4147032636
0.2486687294 3	1.1025394161	0.9479660678	1.2823171687	SAA4_P	0.4147032636
0.2590816090 3	1.1069018027	0.9455847866	1.2957395445	CTSO_F	0.4160871763
0.0442613595 0	0.9229439961	0.8149448766	1.0452555066	FGFR4_	0.4162587231
0.0469058459 3	0.9189793798	0.8058247152	1.0480233287	PTPRH_	0.4176606686
0.2681735204 1	1.1105480780	0.9432101152	1.3075740111	CD160_	0.4181609944
0.1979797462 5	1.0805085290	0.9578001216	1.2189377055	PGLYR	0.4181609944
0.0517559187 0	0.9118616245	0.7895516909	1.0531186645	SOD3_F	0.4196485817
0.0594991579 1	0.9010387716	0.7649742246	1.0613048672	CBLN1_	0.4250382798
0.2848972166 8	1.1170976992	0.9385405162	1.3296253577	SERPIN	0.4256340663
0.0630491125 8	0.8959435407	0.7536668337	1.0650791467	FOXO3_	0.4257437688
0.0569491698 1	0.9063715813	0.7760323930	1.0586020000	SATB1_	0.4284579542
0.0632031739 0	0.8978333013	0.7567329240	1.0652432468	NME1_I	0.4321193909
0.2557627505 4	1.1038456182	0.9434965681	1.2914462966	GPNMB	0.4329269941
0.0615882697 9	0.9011243928	0.7635228629	1.0635243694	CD248_	0.4333847702
0.3152582513 5	1.1293862872	0.9306151143	1.3706132279	CR1_P1	0.4333847702
0.0580111778 6	0.9066538971	0.7756916757	1.0597268411	UGDH_	0.4333847702
0.2418680859 0	1.0976022637	0.9459060715	1.2736261724	DNAJB	0.4349107416
0.2896358341 9	1.1180153710	0.9356389749	1.3359408954	IL36G_(	0.4349107416
0.0580399278 5	0.9071764256	0.7765637099	1.0597573087	SAP18_	0.4349107416
0.0580986054 2	0.9077403365	0.7774838290	1.0598194945	GBP2_P	0.4356958473
0.2793637887 4	1.1134408424	0.9375795876	1.3222882899	LYVE1_	0.4356958473

0.0567090143(	0.910693039	0.783638243	1.058347801	CTRB1_	0.438618048
0.0590119687	0.907366015(	0.776133530	1.060787937(	PAFAH	0.438628997
0.2588868282(	1.104137366	0.941050857(	1.295487183	GADD4	0.441690722
0.3133032681(	1.127322800	0.929032060	1.367936319	CXCL8_	0.441718171(
0.3076831377	1.124769275	0.930040369	1.360269902	CXCL8_	0.442426409
0.0596811527	0.908074135	0.776825398	1.061498036	DOC2B_	0.442426409
0.0704354311	0.892354378	0.742138562	1.072975286	FAP_Q1	0.442426409
0.2188619528	1.087217117	0.949690348	1.244659443	KIR2DL	0.442426409
0.2081642128	1.082590434	0.951752009	1.231415367	KLK4_(	0.444317191(
0.2229184952	1.088632926	0.948310718	1.249718711	SERPIN	0.444847256
0.2399062964(	1.095563114	0.944245280	1.271130035	TLR1_Q	0.446384203(
0.2579204303	1.103035134	0.940080994	1.294235832(	HIF1A_(	0.446737221
0.2007056195	1.079101717	0.952707149	1.222264907	HSD17E	0.449758337
0.0617061842(	0.907724982	0.774657840	1.063649781	ARHGE	0.449814394
0.2264545868	1.089523164	0.946509460	1.254145653	APOH_I	0.451432467
0.3297095464	1.132890263(	0.922963766	1.390564175	ITGAL_	0.451673766(
0.0672669504	0.901097170(	0.759153478(	1.069580965	BCAT2_	0.453071555
0.2444756900	1.096717225	0.941921882(	1.276951619	MUC16_	0.453781832
0.2929551797	1.116741533	0.930414605(	1.340382712	FOLR1_	0.455823922
0.0657830986	0.904252098	0.765613906	1.067995042	GAGE2	0.455823922
0.2504296289	1.098874211	0.940017106	1.284577189(	CEACA	0.456676863
0.0702591780	0.898858017	0.753128391(	1.072786187	PAK4_(	0.457740035
0.2707817349	1.107103378	0.934926218	1.310988896	GSR_P0	0.458565718
0.2455826829(	1.096314415	0.940188739(	1.278365978	CARHS	0.461194031
0.0610925010	0.912684527	0.783626727	1.062997238(	COMMI	0.461194031
0.2900379399	1.114798079	0.929887794	1.336478193(	ESAM_(	0.461194031
0.2505461562	1.098357460	0.939023790	1.284726886	HSP90B	0.461194031
0.0733208947	0.896061984	0.746162201	1.076075788	TFRC_P	0.461194031
0.2858275381	1.112897039	0.930629149	1.330862912	ECHS1_	0.461461723(
0.2425994977	1.094908024	0.940579813	1.274558058	AHSP_(	0.462852425(
0.2591726015	1.101557011	0.936389915	1.295857452	COMP_	0.464434893
0.2512177702	1.098211877	0.938144596(	1.285590017	RBKS_(	0.465052442
0.2617907730	1.102458446	0.935470657	1.299254674	COL9A	0.465785305(
0.2807531250	1.109795489(	0.930157254	1.324126669	BCL2L1	0.471217172
0.2475142299	1.095893957	0.937654841	1.280837588	FZD10_	0.474954231
0.2751215473	1.10694655	0.930613887	1.316690705	USP28_(	0.476960436(
0.2685740148	1.104144893	0.931991440	1.308097792(	BID_P5	0.477836105
0.3135101959	1.122580260	0.921041193	1.368219412	FCRL1_	0.477836105

0.27456875392	1.10652134759	0.93041327711	1.31596304855	DDR1_(	0.47798126288
0.23026611408	1.08824705594	0.94070120844	1.25893498506	EGFLAI	0.48285466299
0.22838894526	1.08719760811	0.94065185844	1.25657396888	SERPIN	0.48701342555
0.26517662402	1.10179761488	0.93119130311	1.30366121333	RAD51_	0.48784065499
0.23482543458	1.08965463277	0.93884597522	1.26468798	SAT2_Q	0.48784065499
0.06712006233	0.91310659788	0.77963816211	1.06942386799	EIF1AX	0.48887238600
0.27383090165	1.10511938177	0.92874211977	1.31499242033	JAM3_(	0.48893323422
0.26853630434	1.10295052222	0.93001130155	1.30804846400	TP53I3_	0.48893323422
0.21987950592	1.08344785600	0.94215763722	1.24592659477	CYTL1_	0.48992641866
0.25895113444	1.09853558033	0.93146642844	1.29557049455	AGR2_(	0.49492944699
0.27340969332	1.10434918399	0.92783875388	1.31443865122	IL31_Q(	0.49492944699
0.28918231854	1.11062239466	0.92372472311	1.33533516277	TALDO	0.49492944699
0.24256625149	1.09195572555	0.93554541599	1.27451568499	BAG4_(	0.49500534711
0.06392358904	0.91964673366	0.79337845644	1.06601094077	CDH6_I	0.49741220866
0.21910176368	1.08242914099	0.94111840011	1.24495796177	PVR_P1	0.49843451366
0.26028948709	1.09852790555	0.93020763400	1.29730558555	CRTAM	0.49970452600
0.22674965131	1.08527920388	0.93887297855	1.25451576222	ENTPD5	0.49970452600
0.25406889879	1.09595348277	0.93163012088	1.28926062977	CA3_P0	0.49977108900
0.06200957726	0.92289979233	0.80053196777	1.06397253466	NEFL_P	0.49977108900
0.35186699856	1.13516752922	0.90637104699	1.42171942044	BTN2A1	0.50040442800
0.26075251619	1.09847446455	0.92968655944	1.29790641488	TNXB_I	0.50040442800
0.25485867448	1.09600480899	0.93098182688	1.29027925866	SETMA	0.50182096344
0.24787223747	1.09320489377	0.93272493988	1.28129622000	PRKCQ	0.50200227077
0.23244979190	1.08708508944	0.93664585588	1.26168709788	CPQ_Q9	0.50263128277
0.23710419852	1.08885932655	0.93534215000	1.26757319011	C5_P01(	0.50278264922
0.06441615856	0.92193204888	0.79693379266	1.06653615455	NAAA_	0.50588038511
0.22542642776	1.08403515333	0.93796207355	1.25285685522	CD7_P0	0.50597214399
0.24424589440	1.09125032600	0.93276905299	1.27665821500	BRDT_(	0.50706865755
0.21000002557	1.07793764177	0.94185798333	1.23367809155	IGFBP6_	0.50711231966
0.26601243189	1.09969002422	0.92685722466	1.30475127911	CALCO	0.50715629011
0.24662904154	1.09188505233	0.93163159677	1.27970430755	ASAH2_	0.50925747400
0.09674866218	0.88685423122	0.71398169044	1.10158346922	GPC1_P	0.50925747400
0.07243193584	0.91413301955	0.77725227677	1.07511962644	MET_P(	0.50925747400
0.07122707202	0.91600921311	0.78138695988	1.07382503388	CSDE1_	0.51122869355
0.26525691758	1.09873897006	0.92595406277	1.30376589311	AMIGO	0.51178890844
0.24855877950	1.09223484444	0.93043137799	1.28217618588	CYB5A_	0.51178890844
0.29871577979	1.11185198011	0.91698732544	1.34812640422	ERBB3_	0.51178890844
0.21454331977	1.07921904488	0.93981900155	1.23929580588	GNLY_I	0.51178890844

0.23792277917	1.08779798188	0.93275577677	1.26861122588	CLTA_I	0.5159155210
0.24384599671	1.09004876377	0.93108832844	1.27614778444	PTH1R_	0.5159155210
0.07755448915	0.91101506844	0.76801487955	1.08064111400	TST_Q1	0.5173363906
0.17134832191	1.06231732388	0.95080815277	1.18690410177	LILRA6	0.5178887683
0.07670874904	0.91256612444	0.77128431577	1.07972755899	GRP_P0	0.5192570155
0.20171819076	1.07362218333	0.94210184788	1.22350316500	CD177_	0.5192938269
0.07654406705	0.91357910277	0.77312487711	1.07954976188	ENO2_I	0.5219984969
0.27911981823	1.10298761333	0.92028230955	1.32196572999	TNC_P2	0.5219984969
0.22563494106	1.08219911933	0.93459260988	1.25311811988	DNAJA4	0.5252411991
0.22375366498	1.08147687399	0.93510308988	1.25076287477	PROC_I	0.5252411991
0.29918157703	1.11022540599	0.91388050844	1.34875450400	PDGFB_	0.5268737611
0.07911303963	0.91269713022	0.76965308322	1.08232666099	ENO1_I	0.5285735546
0.07509147276	0.91724244422	0.78047046633	1.07798275244	ATP1B2	0.5293106843
0.27576799119	1.10085804633	0.91980999677	1.31754214711	MAMD(	0.5293106843
0.29417136996	1.10759671544	0.91412653488	1.34201386488	TCN2_I	0.5328081248
0.08195559502	0.91132698999	0.76516589155	1.08540761122	AXL_P3	0.5341348861
0.08529222984	0.90851338699	0.75791537511	1.08903526888	GP1BA_	0.5365086035
0.25940490903	1.09382945866	0.92308375999	1.29615852488	ITIH3_(	0.5370143897
0.07549083998	0.91887771933	0.78294307433	1.07841334933	NENF_(	0.5370143897
0.07649716189	0.91829046455	0.78115614599	1.07949912666	ARG2_I	0.5375302192
0.08004098113	0.91474515877	0.77239398477	1.08333146299	FAM172	0.5375302192
0.26947325865	1.09743879200	0.91987722355	1.30927461999	ITGB7_	0.5375302192
0.24542852573	1.08840354444	0.92681198322	1.27816892411	WFDC1_	0.5375302192
0.25554731792	1.09189970799	0.92338477522	1.29116810699	SCGN_(	0.5407928338
0.06655409218	0.92936710844	0.80811007500	1.06881877722	CRYGD	0.5408772133
0.09573950880	0.90005198499	0.73613259411	1.10047236322	IL12B_I	0.5408772133
0.24968358151	1.08935849633	0.92449687666	1.28361919155	WIF1_Q	0.5439560809
0.26645775243	1.09551341499	0.91942068133	1.30533244100	DUT_P3	0.5450749282
0.23118163315	1.08228572600	0.92957182999	1.26008809244	EEF1D_	0.5455769829
0.06994834616	0.92712952700	0.80149837188	1.07245278355	LMOD1	0.5455769829
0.07222173179	0.92591589977	0.79758611366	1.07489365577	CDH5_I	0.5506088690
0.24295990990	1.08620320744	0.92534996644	1.27501750755	CRYBB	0.5506088690
0.27889084903	1.09946836388	0.91462847500	1.32166307511	CTSL_P	0.5507462903
0.32613858079	1.11728208300	0.90091845377	1.38560737411	SPARC_	0.5507462903
0.31889404308	1.11431695211	0.90265865744	1.37560556222	IL5RA_(	0.5523711314
0.22917675910	1.08086322844	0.92899052055	1.25756430533	PLIN3_(	0.5523711314
0.27588648961	1.09779620844	0.91459215711	1.31769828311	SERPIN	0.5560124011
0.25988784222	1.09169687388	0.91904394399	1.29678463400	CLEC11	0.5564593077

0.24339247418	1.08572186188	0.9241301874	1.2755691538	DCTN2_	0.5564593077
0.08498295168	0.91540933555	0.7697027661	1.0886985060	NELL1_	0.5564593077
0.30193418041	1.10727899833	0.9065375066	1.3524722046	VEGFC_	0.5564593077
0.29166087136	1.10330528400	0.9093366374	1.3386489664	CXCL8_	0.5575767943
0.15195078173	1.0524655	0.9515340799	1.1641029398	BST1_Q	0.5580102916
0.24353887624	1.08541622297	0.9234747645	1.2757559134	NAA10_	0.5580102916
0.31485696189	1.11185290300	0.9023063786	1.3700633256	TF_P027	0.5580102916
0.23726828000	1.08305301611	0.9252415501	1.2677811925	IL5_P05	0.5584750957
0.25294547410	1.08855177122	0.9201218711	1.2878130557	HMOX2	0.5604859265
0.37336452985	1.13348058455	0.8844596319	1.4526137644	TNFRSF	0.5604859265
0.07740284963	0.92430994044	0.7907143433	1.0804772585	BRD2_P	0.5609045648
0.07979731283	0.92227527588	0.7853542531	1.0830675215	HK2_P5	0.5610095086
0.24310854016	1.08483334655	0.9228802572	1.2752070277	PRDX3_	0.5610095086
0.20952523289	1.07250628410	0.9328332947	1.2330924892	CCL8_P	0.5632807413
0.08191482722	0.92091018664	0.7813747919	1.0853633624	ITGA2_	0.5632807413
0.23549531079	1.08150608311	0.9242375713	1.2655354468	INSL5_(	0.5669204045
0.24051941017	1.08332842699	0.9227074476	1.2719096215	ISM1_B	0.5669204045
0.25850589905	1.08974327500	0.9170240162	1.2949937890	SSB_P0	0.5673734144
0.08025300723	0.92360225655	0.7872570027	1.0835611818	NOTCH	0.5676171171
0.07856763200	0.92527860133	0.7914501175	1.0817365127	STAU1_	0.5678505008
0.07771713595	0.92675542060	0.7946543423	1.0808168911	MTHFD	0.5714434572
0.07752883965	0.92704674288	0.7953035435	1.0806133965	BIRC2_(	0.5716164806
0.23362561694	1.08028415900	0.9238760295	1.2631714936	DEFB11	0.5718197425
0.27220958839	1.09384061410	0.9113579094	1.3128621331	TNFRSF	0.5746215700
0.25438323034	1.08744097810	0.9169257218	1.2896659487	WARS_	0.5746215700
0.23888692846	1.08165090100	0.9213549172	1.2698349462	AOC1_F	0.5749594574
0.11042661230	0.89976295744	0.7249341366	1.1167543900	COL1A1	0.5749594574
0.10111717751	0.90789541999	0.7450012788	1.1064062801	CRH_P0	0.5749594574
0.07947889906	0.92635213855	0.7925651456	1.0827227128	HMOX1	0.5749594574
0.08398729973	0.92284846344	0.7830429180	1.0876150807	MMP13_	0.5749594574
0.08856724994	0.91843740010	0.7720312025	1.0926077279	NGF_P0	0.5749594574
0.22960780832	1.07833698822	0.9242545567	1.2581064942	SERPIN	0.5749594574
0.08008237495	0.92609721533	0.7916511065	1.0833763070	STX6_C	0.5749594574
0.07791940236	0.92854630410	0.7975669790	1.0810355262	DIABLO	0.5757010478
0.24125880439	1.08229589299	0.9202687047	1.2728504119	GBP1_P	0.5757010478
0.08186097978	0.92542099944	0.7890907065	1.0853049200	MAP2K	0.5757429352
0.24632780590	1.08392505310	0.9183742580	1.2793188730	NGRN_(	0.5757429352
0.07819977316	0.92862177788	0.7974730197	1.0813386595	PRDX6_	0.5757429352

0.2288769283	1.077850861	0.924096572	1.257187305	UBE2L6	0.575742935
0.2316304359	1.078595607	0.922829511	1.260653750	ENPEP_	0.577130140
0.2408766348	1.081782879	0.919747919	1.272364060	IDO1_P	0.577413070
0.2287354866	1.077472970	0.923579338	1.257009499	KLK14_	0.577413070
0.0547511556	0.949949558	0.854324705	1.056277733	LRRC37	0.577413070
0.0816883459	0.926444354	0.790973402	1.085117575	FGF7_P	0.578007887
0.2409382667	1.081512053	0.919230801	1.27244248	DBI_P0	0.579641932
0.0887703114	0.921219337	0.776557529	1.092829617	IGFBP2_	0.581849962
0.2305147806	1.07747524	0.921941368	1.259248079	MFAP3I	0.583931011
0.2414412779	1.081270510	0.918358188	1.273082694	SF3B4_	0.583931011
0.0849563662	0.925480821	0.786753649	1.088669562	DOK1_	0.585559222
0.2615514546	1.088144083	0.911554116	1.298943776	F3_P137	0.585559222
0.2360722262	1.079001561	0.919431293	1.266265764	LRP11_	0.587967162
0.0877370978	0.924209513	0.782414935	1.091701073	ERBB4_	0.590640469
0.2502368092	1.083648175	0.914324048	1.284329521	TNFRSF	0.590776093
0.2298176420	1.076505617	0.920924584	1.258370515	CD3G_	0.591155288
0.2309822254	1.076802854	0.920360751	1.259836846	ATP1B3	0.591888482
0.0825389589	0.929181348	0.794977345	1.086040983	IL21R_	0.591888482
0.2654694143	1.088743439	0.908990199	1.304042968	THBS4_	0.591888482
0.0862592109	0.926301918	0.787124132	1.090088854	CALY_	0.592483156
0.0830761995	0.929044794	0.794316845	1.086624605	LMOD1	0.592692756
0.0859157370	0.926962201	0.788517471	1.089714501	SUGT1_	0.593656894
0.2292304557	1.075618572	0.919947541	1.257631834	C1GAL	0.596953436
0.2494656245	1.082586928	0.913238082	1.283339448	COX5B_	0.596953436
0.0864234191	0.927622116	0.789239795	1.090267870	RAD23I	0.598503275
0.2401225959	1.078992491	0.915699393	1.271405009	ING1_Q	0.600602419
0.2363566826	1.077690043	0.916936662	1.266626013	NFKB1_	0.600602419
0.3249409045	1.108103476	0.887238937	1.383948858	IL34_Q	0.602433793
0.2291892632	1.075042161	0.918999683	1.257580030	CLINT1	0.602548336
0.2368352390	1.077454102	0.916096704	1.267232310	IL6ST_	0.604663610
0.0867591682	0.929048794	0.791403598	1.090633988	DDX25_	0.605074106
0.2276058776	1.074241933	0.919086158	1.255590371	SHH_Q	0.605074106
0.0606775649	0.950093617	0.849534204	1.062556253	ACRV1_	0.605567031
0.0870518061	0.929168045	0.791375156	1.090953196	AMIGO	0.605567031
0.2204831282	1.071700033	0.921280508	1.246678890	BOLA1_	0.605567031
0.2425879189	1.079105254	0.913635613	1.274543300	RRM2_	0.605567031
0.0862682054	0.929988139	0.793394186	1.090098659	BCL2L1	0.605780043
0.0884449634	0.928428678	0.789016226	1.092474124	EIF4G3_	0.606104451

0.09604138802	0.92292271299	0.77378520788	1.10080462311	IGDCC3	0.6078262630
0.26487762080	1.08597727500	0.90491249644	1.30327147277	CA9_Q1	0.6086035637
0.08323667396	0.93344948099	0.80173789011	1.08679899511	CCT5_P	0.6086035637
0.23796104551	1.07694493410	0.91420128300	1.26865977188	CD2_P0	0.6086035637
0.09078238561	0.92754057177	0.78566886222	1.09503068499	DUSP3_	0.6086035637
0.21960405414	1.07093373822	0.92077256833	1.24558344933	FABP6_	0.6086035637
0.10677519437	0.91508496399	0.75257703400	1.11268408877	HS6ST2	0.6086035637
0.19918826135	1.06397540688	0.92759162000	1.22041170006	IL17D_(	0.6086035637
0.23664557309	1.07666656311	0.91493151066	1.26699198211	PTGDS_	0.6086035637
0.24939849569	1.08064272155	0.91002196511	1.28325330211	ASPN_(	0.6087790304
0.22165885689	1.07136204699	0.91961764666	1.24814550899	BECN1_	0.6087790304
0.26763629066	1.08673794433	0.90368421833	1.30687173211	SELP_P	0.6088627211
0.23889000284	1.07691919944	0.91330877288	1.26983885022	C1QTNI	0.6100627933
0.09511396183	0.92524412944	0.77840426666	1.09978418144	DTD1_(	0.6100627933
0.22103838706	1.07076396955	0.91916133311	1.24737131255	CTSE_P	0.6116333249
0.08987520383	0.92972820099	0.79009570999	1.09403774344	DLK1_F	0.6116333249
0.26169229823	1.08435350899	0.90508685455	1.29912673733	HPSE_(	0.6116333249
0.08729151587	0.93186773488	0.79578972266	1.09121473977	LACRT_	0.6120155408
0.08751328884	0.93174045066	0.79539592633	1.09145676855	RPS10_	0.6120155408
0.09394598942	0.92737955177	0.78291534733	1.09850041366	CPB1_P	0.6132014962
0.22913947545	1.07317284566	0.91585208988	1.25751741955	IL20_Q9	0.6132014962
0.22557129035	1.07202057566	0.91715317811	1.25303836044	KCNIP4	0.6132014962
0.21519763498	1.06840308888	0.92047314955	1.24010696122	FCGR2A	0.6148008114
0.15524443801	1.04869812599	0.94162760311	1.16794341599	ACP6_(	0.6183143117
0.08059030472	0.93833337006	0.81229615744	1.08392672599	SERPIN	0.6183143117
0.23770178553	1.07531154555	0.91166659933	1.26833090177	ANXA5_	0.6197208451
0.24264741354	1.07696671833	0.90996383444	1.27461913144	MYOM2	0.6197208451
0.09476664818	0.92858648522	0.78431060055	1.09940227766	SHC1_P	0.6209587157
0.05668980351	0.95681856088	0.86504582400	1.05832747000	EDDM3	0.6221378410
0.24943080730	1.07875297433	0.90681269000	1.28329476677	CXCL5_	0.6236027778
0.08625224425	0.93561698022	0.80304025544	1.09008126033	NMT1_I	0.6249119290
0.09183943939	0.93202711599	0.79244975177	1.09618880322	STX1B_	0.6271634158
0.24772729605	1.07752942399	0.90629937122	1.28111052006	LMNB1_	0.6300924048
0.09102418457	0.93330963266	0.79528024622	1.09529549411	SCIN_Q	0.6300924048
0.08995045666	0.93394300299	0.79721554511	1.09412007600	SH3GL3	0.6300924048
0.22295570789	1.06943987866	0.91513320888	1.24976521777	FGF16_(	0.6302331955
0.10183818856	0.92592093900	0.77431923477	1.10720429899	RGMB_	0.6303680811
0.22305519227	1.06936150200	0.91490805455	1.24988955600	LYPD3_	0.6307641030

0.20614516798	1.06385869295	0.92095876233	1.22893159255	C7_P10(	0.6315000038
0.22459959515	1.06965405533	0.91399605088	1.25182138044	EFHD1_	0.6326601985
0.25298101893	1.07864680122	0.90342116177	1.28785883160	GAPDH	0.6340159445
0.09033768260	0.93525358070	0.79914502877	1.09454382977	ENSA_(	0.6359888281
0.09078123478	0.93541494811	0.79906631311	1.09502942477	PPP1R1	0.63860954
0.21724172342	1.06673488060	0.91572719199	1.24264444211	IL18RA	0.6389986788
0.09474261599	0.93318045633	0.79210923044	1.09937585699	TMPRS	0.6406875357
0.20712795001	1.06326217160	0.91902261860	1.23013995811	CDH17_	0.6416462374
0.22323082917	1.06834371277	0.91300694200	1.25010910200	GSTP1_	0.6416462374
0.26887625164	1.08255625188	0.89563173277	1.30849320711	CPXM1	0.6444796717
0.09456047454	0.93413352477	0.79387262211	1.09917563322	PKLR_F	0.6444796717
0.27279738318	1.08368299333	0.89398476455	1.31363405355	DKK1_(	0.6454201007
0.22133273350	1.06723935360	0.91285138222	1.24773852599	TARBP	0.6469019885
0.08359565544	0.94233291833	0.81677717560	1.08718920588	APOA1_	0.6482272363
0.07918357724	0.94554115960	0.82598447877	1.08240300833	CRTAC	0.6484598753
0.23513564251	1.07122503088	0.90707523955	1.26508035577	DAG1_(	0.6484598753
0.23961218617	1.07268604555	0.90548865160	1.27075623799	ECI2_O	0.6484598753
0.24381148002	1.07398423844	0.90387804000	1.27610373677	GUK1_(	0.6484598753
0.09053993826	0.93809301488	0.80384221255	1.09476522977	IL1RL2	0.6484598753
0.21053126084	1.06305837399	0.91554914388	1.23433363899	CD48_P	0.6554213788
0.09734601979	0.93514653333	0.79338228144	1.10224170500	FH_P07	0.6561963629
0.09499030926	0.93660326377	0.79773301560	1.09964819860	GMPR_	0.6561963629
0.23441602801	1.07026501255	0.90610195899	1.26417031300	LYN_P(	0.6561963629
0.09789803485	0.93485247877	0.79244584299	1.10285032700	PPIF_P3	0.6561963629
0.21679672292	1.06471845744	0.91267456022	1.24209158777	FAM171	0.6567673823
0.20931413782	1.06235899355	0.91545841800	1.23283221699	IGFBP1	0.6571018235
0.10002251397	0.93420492499	0.78966898144	1.10519580011	ADGRE	0.6587152075
0.11083775824	0.92735731588	0.76976467660	1.11721363344	SAT1_P	0.6587152075
0.16782924265	1.04943927	0.93116643511	1.18273463277	CGA_P(	0.6600079274
0.21781254173	1.06462635422	0.91159018477	1.24335396877	ZNF75D	0.6600079274
0.23320981486	1.06923185998	0.90544494011	1.26264637355	TXN_P1	0.6610817276
0.10149332706	0.93421209760	0.78852048777	1.10682253260	DGCR6	0.6626725933
0.25450509820	1.07554248399	0.89686067060	1.28982312711	CPOX_I	0.6630499602
0.21676538464	1.06375921377	0.91105932800	1.24205266333	ABL1_P	0.6650553487
0.25370606395	1.07495934844	0.89660454955	1.28879292588	APP_P0	0.6650553487
0.23747334822	1.07010412560	0.90306437911	1.26804120077	PTEN_P	0.6650553487
0.11469981179	1.03322868877	0.95187389688	1.12153671499	TLR3_C	0.6650553487
0.15618761738	0.90218393960	0.69623966733	1.16904551577	PYDC1_	0.6665571271

0.22588014163	1.0661680114	0.9068862079	1.2534254227	PTN_P2	0.6674387308
0.11394961406	0.9279087893	0.7682859450	1.1206956561	S100A14	0.6674387308
0.20851381426	1.0609004631	0.9136773923	1.2318459469	TMEM2	0.6674387308
0.21643441560	1.0632561259	0.9104990873	1.2416416503	TPPP2_	0.6674387308
0.21651954669	1.0632156985	0.9103523475	1.2417473571	IL1A_P(	0.6678585356
0.11016509502	0.9307239879	0.7758856537	1.1164623776	SPINK5	0.6679107601
0.10341976641	0.9350096460	0.7883472349	1.1089568142	SH2D1A	0.6683688152
0.10488646423	0.9341715309	0.7857812167	1.1105845121	TINAGI	0.6683688152
0.10185596970	0.9365981460	0.7922661530	1.1072239864	ABHD14	0.6701914642
0.22792902160	1.0663083495	0.9052682803	1.2559961736	BOLA2_	0.6701914642
0.21673375461	1.0628568851	0.9095431487	1.2420133777	SERPIN	0.6701914642
0.09939679873	0.9380509131	0.7966826143	1.1045044786	VPS4B_	0.6701914642
0.24873437756	1.0722741784	0.8965772774	1.2824013532	PPBP_P	0.6720875169
0.21943587321	1.0634239231	0.9080569012	1.2453739835	USP25_	0.6725839765
0.21358345795	1.0615488645	0.9101686300	1.2381068238	TBC1D5	0.6740071984
0.22515997483	1.0648885853	0.9053627228	1.2525230723	LAMP1_	0.6747822490
0.09786978562	0.9400489135	0.8013026809	1.1028191728	PGM2_(	0.6747822490
0.21535908127	1.0618275436	0.9090310394	1.2403071880	RELB_(	0.6754389413
0.17838229228	1.0509545090	0.9240540833	1.1952821810	SIGLEC	0.6754389413
0.21582935261	1.0617966551	0.9085507870	1.2408906061	IL24_Q1	0.6755029011
0.21786387793	1.0625237181	0.9079463491	1.2434177994	LRRC59	0.6755029011
0.09588733804	0.9417353856	0.8057762022	1.1006350573	PSMD5_	0.6755029011
0.20040360429	1.0572326098	0.9147594844	1.2218958210	TEK_Q(	0.6755029011
0.20921588918	1.0598779948	0.9112770745	1.2327110988	TGFBI_	0.6755029011
0.22479857016	1.0642755625	0.9046475299	1.2520704864	TXNDC	0.6769713364
0.10300084233	0.9383468916	0.7943175203	1.1084923427	CRISP3_	0.6784153239
0.09503249750	0.9429126254	0.8084828513	1.0996945918	FHIT_P4	0.6784153239
0.22769866860	1.0645358382	0.9024690116	1.2557068845	IDO1_P	0.6835555519
0.23122422505	1.0654917311	0.9009086614	1.2601417631	DSCAM	0.6840184683
0.26744471653	1.0759884855	0.8860647981	1.3066213932	CR2_P2	0.6845654141
0.10097637179	0.9406895120	0.7999063114	1.1062505027	PTPN6_	0.6845654141
0.21125887683	1.0593609865	0.9085302364	1.2352320866	LRIG3_	0.6869764668
0.10228449144	0.9405425762	0.7986110766	1.1076985576	CD69_Q	0.6876898272
0.20971402901	1.0587332983	0.9088568799	1.2333253142	CD164L	0.6885739381
0.09706179805	0.9439084216	0.8085489519	1.1019284685	ARHGA	0.6891049123
0.21008563057	1.0587052218	0.9084710246	1.2337837050	PECR_(	0.6891049123
0.09792028962	0.9434951619	0.8071478860	1.1028748710	PLXNB1	0.6891049123
0.25727073125	1.0722507951	0.8889175794	1.2933952418	CSF1R_	0.6892496422

0.23406606848	1.06551094088	0.89838444475	1.2637279820	NIT2_Q	0.6892496422
0.2375003978(	1.0664380583	0.8968631056	1.2680755012	GIP_P09	0.6895435199
0.1070293970(	0.9388352301	0.7919476602	1.1129669718	LYPD1_	0.6898400166
0.2435519413	1.0679694613	0.8940141736	1.2757725815	GRHPR_	0.6912001507
0.2461232476	1.0684389989	0.8925026108	1.2790572046	PRG3_C	0.6940752802
0.1100478584	0.9380368017	0.7882184148	1.1163314951	ACADS	0.6940980235
0.0953097291	0.9465015754	0.8144233055	1.0999995042	NUDT2_	0.6966473387
0.2501356083(	1.0691929232	0.8901836980	1.2841995529	CRYBB	0.6973293783
0.1135365037	0.9370989104	0.7839034735	1.1202327808	DRAXII	0.6988642508
0.2326625949	1.0638097589	0.8967755764	1.2619556172	PPIB_P2	0.7015131821
0.2613004557	1.0717218680	0.8844694539	1.2986177840	PDGFA_	0.7035205814
0.2120303594(	1.0576911775	0.9049699292	1.2361854143	MAPT_I	0.7047779357
0.1055451655	0.9426000049	0.7994976516	1.1113162965	MYO6_	0.7054000247
0.1120431852	0.9394535310	0.7890251911	1.1185611649	CNTN4_	0.7067677885
0.2117134265	1.0573118989	0.9046076718	1.2357936887	METAP	0.7073370675
0.1053451683(	0.9433213409	0.8008819284	1.1110940586	ANXA1	0.7082482646
0.0947947618(	0.9492821718	0.8196374759	1.0994331862	ADAM2	0.7090773138
0.2113034384	1.0567843029	0.9040756874	1.2352871318	ARAF_I	0.7090773138
0.1980713513	1.0530916054	0.9097268373	1.2190493715	CAT_P0	0.7090773138
0.2128850586	1.0571291040	0.9032360295	1.2372424327	FKBP7_	0.7090773138
0.1028989270	0.9450159288	0.8057305332	1.1083793762	GGA1_(	0.7090773138
0.1049285628	0.9445642588	0.8033284000	1.1106312672	LRP2_P	0.7090773138
0.2148866955	1.0575831925	0.9022044697	1.2397214230	MAP2K	0.7090773138
0.1047820159(	0.9444505244	0.8032526608	1.1104685194	OPHN1_	0.7090773138
0.1622678490	1.0434077354	0.9256271246	1.1761752365	PDGFRI	0.7090773138
0.2296770845	1.0619004707	0.8962313599	1.2581936541	PENK_I	0.7090773138
0.1075855108	0.9430904430	0.7986985465	1.1135860803	PMVK_	0.7090773138
0.2001092337	1.0535334526	0.9086368054	1.2215361838	SMTN_I	0.7090773138
0.2692868571	1.0725348747	0.8787656031	1.3090305920	ANGPT	0.7097518340
0.1040690777	0.9453084693	0.8052865983	1.1096771062	CDNF_(	0.7097518340
0.2166620232	1.0578791182	0.9011082544	1.2419242897	NMNAT	0.7097518340
0.1058017050	0.9445306551	0.8025701790	1.1116014297	EDF1_C	0.7099084931
0.2162546358	1.0575549488	0.9009230298	1.2414184483	AIF1L_(	0.7107814034
0.1010565060	0.9472791754	0.8110874791	1.1063391548	PTGES2	0.7107814034
0.2234700130	1.0595631983	0.8978461768	1.2504081437	SORT1_	0.7107814034
0.1116605514	0.9422315486	0.7940022294	1.1181332475	LAP3_P	0.7125190680
0.2250611322	1.0597115710	0.8966698044	1.2523992758	CCL26_	0.7128237598
0.1662736900	1.0437442857	0.9225214555	1.1808962570	IFNGR2	0.7128979361

0.25236886128	1.0670901306	0.8847076906	1.2870707002	CLEC12	0.7129443443
0.1050173675	0.9462554390	0.8061359969	1.1107299008	GZMB_	0.7149034384
0.2261502620	1.0597127817	0.8956957928	1.2537640443	MASP1_	0.7149034384
0.2191906077	1.0574845756	0.8981622790	1.2450685737	CAPN3_	0.7186676612
0.1102602939	0.9444707872	0.7988985300	1.1165686686	AKT2_F	0.7198412843
0.1973615411	1.0513292359	0.9073282960	1.2181843849	AK1_P0	0.7219433528
0.1072849395	0.9465965760	0.8048901287	1.1132514186	SV2A_C	0.7238293566
0.2393571774	1.0621984074	0.8880957475	1.2704322253	CDSN_(	0.7242659712
0.1062017185	0.9476896322	0.8076244133	1.1120461742	ECE1_P	0.7242659712
0.1946582769	1.0503282122	0.9080526839	1.2148957576	HMBS_	0.7242659712
0.1110007237	0.9455451691	0.8001244810	1.1173957155	IL2RB_	0.7242659712
0.1812721350	1.0467675300	0.9140606168	1.1987413544	KLK15_	0.7242659712
0.0921450315	0.9544230056	0.8307373169	1.0965238410	LTA_P0	0.7242659712
0.2152408161	1.0556023821	0.8985098128	1.2401605116	NEK7_C	0.7242659712
0.1071809333	0.9473573195	0.8062682202	1.1131356395	PLCB1_	0.7242659712
0.1026027052	0.9494486512	0.8135479874	1.1080510987	SNX5_C	0.7242659712
0.1592594693	1.0406769431	0.9235626399	1.1726421719	OBP2B_	0.7260843114
0.1131379937	0.9450118269	0.7975157731	1.11978645	IFNL2_(	0.7267192509
0.0959654191	0.9535628624	0.8260786640	1.1007209994	BRSK2_	0.7296645695
0.1972020505	1.0501257486	0.9053965854	1.2179901113	PSMD9_	0.7317802384
0.2059259127	1.0522690591	0.9011998559	1.2286621724	PTTG1_	0.7331243940
0.1181826161	0.9438962098	0.7916303322	1.1254496178	VAT1_C	0.7335205521
0.1052684241	0.9502065618	0.8126780964	1.1110087919	HEBP1_	0.7356727133
0.0935450291	0.9557082764	0.8318108944	1.0980600469	FCRL6_	0.7358373779
0.0985517214	0.9537859387	0.8243304873	1.1035714812	TPMT_I	0.7386719909
0.2041822948	1.0512218605	0.9009766241	1.2265217216	ADAMT	0.7389689327
0.2019250803	1.0505463930	0.9018525214	1.2237563212	PTPN1_	0.7398025377
0.2088302711	1.0522406031	0.8985376465	1.2322358348	MAPK9_	0.7403519459
0.1104832103	0.9495598493	0.8073510922	1.1168175979	CTRL_F	0.7439967229
0.1274917518	0.9423180580	0.7816747135	1.1359754985	EPHB6_	0.7439967229
0.1099435236	0.9497577220	0.8081236206	1.1162150289	IGBP1_	0.7439967229
0.2085430760	1.0515946668	0.8976925949	1.2318819935	IL22RA	0.7439967229
0.2049564860	1.0509261528	0.8997729416	1.2274716516	NDUFA	0.7439967229
0.2087625294	1.0516959318	0.8976684746	1.2321523639	NID2_Q	0.7439967229
0.2069032391	1.05117933	0.8984557531	1.2298635633	PSMG3_	0.7439967229
0.2129434834	1.0529149068	0.8959966147	1.2373147205	TCN1_F	0.7439967229
0.1176414135	0.9470923720	0.7974320025	1.1248406864	CD244_	0.7455663548
0.1185607988	0.9466700950	0.7959889073	1.1258753239	DUSP13	0.7455663548

0.20872217922	1.05143765460	0.89726383111	1.23210264723	KIR3DL	0.7455663548
0.11974982618	0.94629755655	0.79441740155	1.12721481660	LDLRA	0.7459348411
0.23455258426	1.05765390043	0.88475343535	1.26434295552	ARSB_I	0.7472495515
0.23033771482	1.05655976788	0.88665310700	1.25902512955	CPA4_C	0.7472495515
0.25073607529	1.06179052388	0.87737326435	1.28497090400	PRG2_P	0.7472495515
0.13285930696	0.94135969677	0.77590962135	1.14208930300	ADAMT	0.7476315809
0.11336063142	0.94972395735	0.80530962559	1.12003578022	LYSMD	0.7476315809
0.21091653793	1.05158968635	0.89555599911	1.23480929100	NPTN_C	0.7476315809
0.21686696277	1.05282848411	0.89234157411	1.24217883511	TBCA_C	0.7495098199
0.11606843108	0.94903628635	0.80196932455	1.12307272255	THTPA_	0.7500294991
0.20290492324	1.04922533635	0.89870477655	1.22495599788	PSME2_	0.7500879039
0.20732818670	1.05023183760	0.89645578060	1.23038630195	CD3E_P	0.7508293539
0.23812137226	1.05744980922	0.88126136000	1.26886318822	CNPY4_	0.7552276614
0.11510779292	0.95039794795	0.80504526600	1.12199437411	NAGK_	0.7552276614
0.20519576948	1.04926533755	0.89671670844	1.22776540044	HEXIM	0.7553370405
0.12955708579	0.94461704011	0.78387285211	1.13832409177	IL10_P2	0.7553590253
0.06626829134	0.97124273077	0.88282700499	1.06851335155	INSL3_I	0.7553590253
0.11179225069	0.95213348833	0.81067153344	1.11828051455	ANXA4	0.7556593736
0.19038863549	1.04528670433	0.90320455460	1.20971964633	APOL1_	0.7559793137
0.21875451513	1.05234727722	0.88984483633	1.24452572699	CD86_P	0.7559793137
0.10528305150	0.95515798100	0.82115769977	1.11102504311	DDX58_	0.7559793137
0.10726825409	0.95447002100	0.81834903299	1.11323284360	GCLM_	0.7559793137
0.11368050391	0.95175761888	0.80850350788	1.12039410611	PPP1CC	0.7559793137
0.10849368802	0.95391189899	0.81639121400	1.11459787322	TSLP_Q	0.7559793137
0.20799229045	1.04923558322	0.89416181044	1.23120367744	RAC3_F	0.7595935481
0.21522531865	1.05081082522	0.89038514977	1.24014129244	FGF6_P	0.7614308248
0.21654882734	1.05080609660	0.88919949400	1.24178371688	GLRX5_	0.7645348131
0.36082550911	1.08601425677	0.82217927577	1.43451312955	NPM1_I	0.7645348131
0.19404099355	1.04545051899	0.90019383077	1.21414605400	PSIP1_C	0.7645348131
0.21619193374	1.05053117700	0.88905151733	1.24134061122	FCRL5_	0.7659588436
0.20071019704	1.04676000611	0.89645173300	1.22227050288	CSF3_P	0.7663731238
0.10599828314	0.95686436433	0.82350509822	1.11181996760	ATXN3_	0.7676638902
0.22933373598	1.05339476877	0.88223430000	1.25776172933	IL17F_C	0.7678277211
0.20647554584	1.04786421933	0.89317967522	1.22933767144	HTRA2_	0.7684034092
0.23496628450	1.05459694855	0.87928256133	1.26486612244	ARID3A	0.7684039942
0.22501642247	1.05218138755	0.88401134711	1.25234328255	BAMBI_	0.7684039942
0.11744389553	0.95281671788	0.80726012599	1.12461853200	SUSD1_	0.7687671839
0.12140885635	0.95136566055	0.80161852822	1.12908645211	EVPL_C	0.7689889380

0.11140318553	0.95550162544	0.81673482055	1.11784551511	GRAP2_	0.7693247817
0.24000363713	1.05538061558	0.87616514266	1.27125377400	HGFAC	0.7693247817
0.12038384414	0.95197672777	0.80347177300	1.12792971777	SELL_P	0.7693247817
0.19032394522	1.04368493300	0.90049683077	1.20964139177	SMPDL	0.7693247817
0.11040623547	0.95630913177	0.81893189666	1.11673163433	C4BPB_	0.7698228155
0.12120847765	0.95211546622	0.80304349199	1.12886022999	PDGFC_	0.7698228155
0.20088495973	1.04597131833	0.89494495099	1.22248412888	PDIA5_	0.7698228155
0.20605256498	1.04727568199	0.89255409422	1.22881779500	TRDMT	0.7698228155
0.20999854408	1.04802552977	0.89031259111	1.23367626388	IL3_P08	0.7700565186
0.10901218973	0.95722120344	0.82163934500	1.11517594399	ASRGL	0.7713478780
0.25337694959	1.05805352633	0.86891054311	1.28836883544	BMP6_I	0.7713478780
0.22120655342	1.05038249822	0.88435404800	1.24758109600	CDHR1_	0.7717789294
0.10157628693	0.96040838144	0.83329324633	1.10691436	CLEC4M	0.7732771106
0.18597156753	1.04179107988	0.90114534488	1.20438801611	CXCL10	0.7749919103
0.17268287064	1.03879339722	0.90795253000	1.18848914055	GLRX_I	0.7749919103
0.20692522365	1.04664609499	0.89070365011	1.22989060166	NADK_	0.7749919103
0.11838847077	0.95433149999	0.80906433722	1.12568132077	SFRP4_	0.7749919103
0.11790678152	0.95478056666	0.81021611544	1.12513922277	IFNW1_	0.7752211915
0.19988700565	1.04487284822	0.89395789499	1.22126475433	SUMF1_	0.7754561858
0.20127092120	1.04504212766	0.89301087066	1.22295605166	COMMI	0.7763774134
0.19518132565	1.04366766199	0.89610372244	1.21553137355	PDIA4_	0.7763774134
0.13159554135	0.95059212577	0.79220432211	1.14064688144	AGER_	0.7795025654
0.14188319677	0.94687288322	0.77797254211	1.15244203177	IGF1R_	0.7795025654
0.21525341802	1.04781587288	0.88529207099	1.24017614011	MAPK1	0.7802311577
0.11309017025	0.95813489788	0.81985845600	1.11973289499	CEBPA_	0.7839674742
0.12219507538	0.95480505977	0.80679050166	1.12997451044	POSTN_	0.7839674742
0.19867255126	1.04365927200	0.89296631999	1.21978248422	CHEK2_	0.7840369775
0.19130989588	1.04193181633	0.89658974566	1.21083462666	CETN3_	0.7845303745
0.20260547144	1.04442257855	0.89076278733	1.22458923755	IL9_P15	0.7845303745
0.22371458501	1.04894320255	0.87972297888	1.25071399599	CNP_P0	0.7860938080
0.21200692363	1.04635821255	0.88570141311	1.23615644377	KRT5_P	0.7860938080
0.20956524011	1.04553023633	0.88646208811	1.23314182277	THSD1_	0.7887917942
0.12823293049	0.95394573811	0.80049106744	1.13681777133	GAD1_	0.7899047839
0.11135603380	0.95998908011	0.82446319877	1.11779280800	CA13_Q	0.7902728385
0.20241610995	1.04334677100	0.88909701688	1.22435736944	ZP4_Q1	0.7952091934
0.21464209990	1.04579495233	0.88241971611	1.23941822966	CA11_C	0.7967820748
0.18166307399	1.03858864133	0.89948073588	1.19921008077	GH1_P0	0.7967820748
0.11446753171	0.95993770255	0.82181389766	1.12127623455	OSTN_I	0.7967820748

0.19753539855	1.0419586017	0.8910711748	1.2183961936	PLSCR3	0.7967820748
0.12389405299	0.9567408204	0.8086900590	1.1318959436	PLXNA4	0.7967820748
0.18233893393	1.0383774506	0.8985074946	1.2000208527	COL28A	0.7976990510
0.20509255147	1.0433605204	0.8867439530	1.2276386794	FUT8_Q	0.7976990510
0.19842388680	1.0419052797	0.8901887028	1.2194792054	ITIH1_P	0.7976990510
0.18782876139	1.0396717676	0.8958174274	1.2066268765	SHMT1_	0.7976990510
0.18389870162	1.0387515767	0.8977536937	1.2018940670	STX7_C	0.7976990510
0.13138583192	0.9547853668	0.7993764818	1.1404077021	TG_P01	0.7976990510
0.19195326149	1.0403121571	0.8932295974	1.2116138866	VTI1A_	0.7989628705
0.12070505209	0.9587030211	0.8146042166	1.1282920759	HBZ_P0	0.7989922075
0.11737527591	0.9599453867	0.8194408628	1.1245413638	PPP2R5	0.7995182778
0.11443236241	0.9615390557	0.8245870587	1.1212368007	CD209_	0.8004327751
0.20143930461	1.0418195745	0.8873624513	1.2231619945	HSPA1A	0.8004327751
0.20705240743	1.0431493335	0.8846495314	1.2300470337	IDO1_P	0.8004327751
0.13696700837	0.9540672870	0.7937321911	1.1467903134	IL7_P13	0.8004327751
0.17191828881	1.0356713276	0.9031933721	1.1875807906	MANSC	0.8004327751
0.18641918075	1.03885847	0.8956780785	1.2049272368	PLA2G7	0.8004327751
0.19393389712	1.0402193268	0.8913030971	1.2140160303	RGS8_P	0.8004327751
0.19363496714	1.0402449324	0.8916134677	1.2136531788	YWHAQ	0.8004327751
0.14230531533	0.9528933405	0.7875645701	1.1529286015	CHAD_	0.8034378354
0.13670180599	0.9551497668	0.7957453473	1.1464862222	CKB_P1	0.8062697449
0.20198776309	1.0411378588	0.8857156268	1.2238330320	FIS1_Q	0.8075263565
0.16397780299	1.0333297093	0.9062816297	1.1781881625	ICAM2_	0.8075263565
0.11943531499	0.9610320145	0.8196069126	1.1268603507	IL20RA	0.8075263565
0.19698091412	1.0400505089	0.8883030181	1.2177207992	PTX3_P	0.8075263565
0.22130743094	1.0451331494	0.8754485941	1.2477069553	SPP1_P	0.8075263565
0.18430982212	1.0373404643	0.8949482012	1.2023882918	EIF4EB	0.8081928352
0.20656258212	1.0418983235	0.8829613404	1.2294446730	KCTD5_	0.8081928352
0.12406185649	0.9602957129	0.8145741058	1.1320858956	IRAK4_	0.8108431413
0.12871906286	0.9589690690	0.8085506308	1.1373705496	PMM2_	0.8113585004
0.12856638498	0.9594084278	0.8094152578	1.1371969115	DAPP1_	0.8128635143
0.19896744283	1.0397819491	0.8860823480	1.2201422408	EHBP1_	0.8128635143
0.18923186020	1.0378583907	0.8914435539	1.2083210815	MICALI	0.8128635143
0.14719996198	1.02912409	0.9141287254	1.1585856129	CD38_P	0.8149172071
0.11862023702	0.9631989195	0.8239784607	1.1259422459	RASA1_	0.8177550052
0.12318239022	0.9618375579	0.8179109651	1.1310907020	VTA1_C	0.8177550052
0.13057677794	0.9596732994	0.8082357372	1.1394854238	MIF_P1	0.8178308891
0.19567412730	1.0384560771	0.8867395327	1.2161305370	CSF2_P	0.8179148897

0.19213136067	1.03774861773	0.88867453800	1.21182969332	PDCD5_	0.8179148897
0.12821603772	0.96060503155	0.81171990611	1.13679856755	SWAP70	0.8179148897
0.12332638032	0.96233486722	0.81863908622	1.13125357960	RRM2B_	0.8195801860
0.11574604445	0.96472119800	0.82896419855	1.12271071733	MAPKA	0.8200505123
0.18139900532	1.03515171788	0.89377340422	1.19889344877	PAM_P1	0.8222601891
0.22081915469	1.04288010177	0.87210388655	1.24709787833	SPTLC1	0.8225372510
0.12924125511	0.96144597788	0.81230852288	1.13796463088	BTLA_(	0.8235206115
0.15303308565	0.95438023277	0.78159441344	1.16536353511	HBEGF_	0.8235206115
0.19579782268	1.03775139033	0.88542694444	1.21628097600	YY1_P2	0.8235206115
0.11988677782	0.96459561911	0.82532386688	1.12736920111	FMR1_(	0.8264381505
0.11859988346	0.96500574300	0.82708952588	1.12591932922	IMPA1_	0.8264381505
0.12181811478	0.96433615244	0.82328833500	1.12954863488	IFIT3_O	0.8264785677
0.12251170236	0.96404049966	0.82221311900	1.13033234755	KIAA15	0.8264785677
0.12343318119	1.02334580800	0.92563225599	1.13137440499	KLK3_P	0.8264785677
0.11975088658	0.96490853400	0.82597165877	1.12721601199	MAGEA	0.8264785677
0.11316645770	0.96693867999	0.83493044744	1.11981831988	CA2_P0	0.8269854286
0.17718273810	0.94902580299	0.75440846999	1.19384923499	TOP1_P	0.8283220661
0.22158794877	1.04193402144	0.86985329800	1.24805700844	VCAM1	0.8284517206
0.19174235426	1.03605634855	0.88612319788	1.21135837544	TMED10	0.8296144071
0.11807622071	0.96610562877	0.82941020333	1.12532988155	ARF6_P	0.8300502176
0.19546116276	1.03653329399	0.88364700200	1.21587157199	CETN2_	0.8312634651
0.19992516313	1.03729154411	0.88099872522	1.22131135560	CLSTN2	0.8312634651
0.12915275752	0.96351002244	0.81587221499	1.13786392811	NAP1L4	0.8312634651
0.20456611938	1.03807067999	0.87823736933	1.22699258111	OMP_P4	0.8312634651
0.18396172287	1.03416216833	0.88978223722	1.20196981422	SOD1_P	0.8312634651
0.11911324386	0.96628951633	0.82886597266	1.12649747999	SRC_P1	0.8312634651
0.19617171803	1.03589058599	0.88192464288	1.21673582288	PRDX5_	0.8382884068
0.22492320282	1.04109919999	0.86556825399	1.25222654500	FLRT2_	0.8395053837
0.13145011936	0.96413827877	0.81506189544	1.14048101833	SYTL4_	0.8401888788
0.19848341722	1.03593192	0.87995846577	1.21955180366	FCER1A	0.8411171036
0.19945526392	1.03609944400	0.87938805233	1.22073759711	HNRNP	0.8411171036
0.19236405607	1.03440401122	0.88275003599	1.21211171322	MDM1_	0.8448601158
0.21018731610	1.03762395511	0.87256298855	1.23390916933	PDGFRA	0.8448601158
0.18957316283	1.03390066222	0.88435584077	1.20873355511	TPT1_P	0.8448601158
0.22877888191	1.04089629100	0.86190126100	1.25706404866	KLRB1_	0.8450454198
0.13933697030	1.02473817233	0.91350841299	1.14951138599	MBL2_P	0.8450454198
0.17918565232	1.03178774199	0.88994135355	1.19624280888	TGM2_P	0.8453452681
0.18396910345	1.03266578088	0.88720259999	1.20197868555	VASP_P	0.8453452681

0.2149286940	1.038122679	0.869270641	1.239773490	CD40LC	0.845651758
0.1745366548	1.030855788	0.892473894	1.190694386	LRP1_Q	0.845651758
0.1918912981	1.033859411	0.882237754	1.211538813	MDH1_	0.846497157
0.1258625001	0.967237466	0.824906702	1.134126215	PPCDC_	0.8471416091
0.1863912195	1.032716322	0.885142928	1.204893546	SLITRK	0.847454221
0.1325075185	0.965845416	0.817086362	1.141687599	TPSG1_	0.848659275
0.1216636857	0.968821310	0.831092759	1.129374213	C1QBP_	0.849046863
0.1990482990	1.034683535	0.877343169	1.220240900	SNAP29	0.849046863
0.2331184864	1.040817327	0.858038855	1.262531063	SPINK8	0.849046863
0.1766917815	1.030480965	0.889905073	1.193263250	AGRP_(	0.849179576
0.1921606666	1.033247257	0.880955974	1.211865207	ARHGA	0.849179576
0.1285184980	0.967595525	0.823327891	1.137142456	CCDC1	0.849179576
0.0918729866	0.976739440	0.870277025	1.096225577	IL1RL1_	0.849179576
0.1294071504	0.967214363	0.821948605	1.138153429	PCNA_	0.849179576
0.1871374835	1.032198929	0.883596591	1.205793050	RALB_	0.849179576
0.1140591607	0.971167532	0.841497917	1.120818431	SERPIN	0.849179576
0.1896884093	1.032817105	0.882401453	1.208872865	YES1_P	0.849179576
0.2059729119	1.035308105	0.872341088	1.228719919	DPP4_P	0.8492102931
0.1236569406	0.969099035	0.829913436	1.131627589	EIF4E_	0.8492102931
0.1354875034	0.966137519	0.815147912	1.145094886	METAP	0.8492102931
0.1888099654	1.032224654	0.882163997	1.207811404	NDST1_	0.8492102931
0.1929904244	1.033172423	0.880097799	1.212871179	PPP3R1	0.8492102931
0.1235516833	0.969208688	0.830188633	1.131508483	TANK_(	0.8492102931
0.1855621335	1.031507351	0.883804165	1.203894999	NEDD9_	0.850694027
0.1989961424	1.033636806	0.875614619	1.220177258	ACOT1	0.851043466
0.1215757686	0.970153130	0.833452576	1.129274926	ANGPT	0.851043466
0.1357448860	0.966700645	0.815888407	1.145389651	CCDC5(	0.851043466
0.1984885076	1.033524382	0.875868666	1.219558011	LIFR_P	0.851043466
0.1698053275	0.959137208	0.776275647	1.185074127	BGLAP_	0.852860267
0.1894848216	1.031749172	0.880756883	1.208626778	GALNT	0.852860267
0.1918452319	1.032107592	0.879290984	1.211483003	TXLNA	0.852860267
0.1909808214	1.031814417	0.879551487	1.210436237	SNCA_	0.854162344
0.1817128831	1.030197491	0.884960881	1.199269813	PRDX1_	0.8542582811
0.1964903000	1.032181498	0.875341435	1.217123514	AHSA1_	0.855986089
0.2037693315	1.033705204	0.871560440	1.226015317	APOA2_	0.855986089
0.1450141007	0.965871934	0.806975352	1.156055871	FLT1_P	0.855986089
0.0976068407	0.976784727	0.865381504	1.102529230	LEFTY2	0.855986089
0.2094992555	1.034487576	0.867893006	1.233060457	NUDT5_	0.855986089

0.18005112107	1.0294646076	0.8851719282	1.1972785677	SLMAP_	0.8559860890
0.11947203160	0.9718993608	0.8382171629	1.1269017259	SOST_C	0.8559860890
0.19303549539	1.0317243626	0.8775929410	1.2129258460	STIP1_F	0.8559860890
0.18361188962	1.0299833715	0.8829147987	1.2015493988	CTLA4_	0.8561716790
0.12500349513	0.9711621945	0.8323293466	1.1331524135	POMC_	0.8592382067
0.12505376644	0.9712195335	0.8323857876	1.1332093800	TTN_Q8	0.8593165247
0.13783062644	0.9685482721	0.8173036922	1.1477811300	APRT_F	0.8600938377
0.16759978133	1.0269265500	0.8918485369	1.1824632720	CCS_O1	0.8600938377
0.13222346812	0.9700926381	0.8245224690	1.14136335	GJA8_P	0.8620986345
0.12051792136	0.9729583956	0.8391667577	1.1280809575	COMT_	0.8640589639
0.17743728924	1.0279820872	0.8849343608	1.1941531693	RLN2_P	0.8648989337
0.15399780477	1.0242594134	0.8993723490	1.1664883260	VSIG10_	0.8648989337
0.19992620636	1.0314622635	0.8711237199	1.2213126297	FXN_Q1	0.8657797683
0.12829566906	0.9721638072	0.8313057724	1.1368890959	PBK_Q9	0.8704878804
0.19553169276	1.0301805640	0.8727872007	1.2159573303	NRGN_	0.8705413625
0.13419205375	0.9710516121	0.8245286651	1.1436124336	TMCO5	0.8705413625
0.12045825834	0.9739617539	0.8409485949	1.1280136548	VSNL1_	0.8705413625
0.12865728006	0.9724724753	0.8315330000	1.1373002818	ST13_P5	0.8718290479
0.19139145317	1.0293158342	0.8749375493	1.2109333830	HGS_O1	0.8720693308
0.12504590663	0.9738040212	0.8368283407	1.1332004732	PXDNL_	0.8762596474
0.19852202295	1.0299581496	0.8698054761	1.2195988862	TNFSF1	0.8764717579
0.19757300341	1.0296863274	0.8701718442	1.2184420121	ARTN_	0.8774013460
0.22545111438	1.0338818346	0.8531583285	1.25288778	IL2RG_	0.8774814754
0.18320749162	1.0271787539	0.8784682167	1.2010635928	CEACA	0.8803558050
0.18998389203	1.0279799160	0.8738971111	1.2092301192	ERP29_	0.8816437940
0.20744843550	1.0305983600	0.8631478304	1.2305342633	LAT_O4	0.8816437940
0.14514885327	0.9707609010	0.8150555446	1.1562116633	SEMA4	0.8816437940
0.12446255671	0.9753069390	0.8399031820	1.1325396136	BTC_P3	0.8824779423
0.13711952262	0.9728872707	0.8252295867	1.1469652286	EBAG9_	0.8824779423
0.13035735264	0.9739672910	0.8326745007	1.1392354193	ERCC1_	0.8824779423
0.19316800633	1.0280036719	0.8711592104	1.2130865826	FKBP1B	0.8824779423
0.18376028130	1.0265923201	0.8769805183	1.2017277119	GNPDA	0.8824779423
0.12880476735	1.0187808502	0.9124778825	1.1374680315	HDGF_	0.8824779423
0.19098209301	1.0278835729	0.8728615876	1.2104377765	JAM2_P	0.8824779423
0.17908476425	1.0260171054	0.8801033572	1.1961221282	UROD_	0.8824779423
0.21621180112	1.0312223687	0.8566532319	1.2413652737	DAPK2_	0.8828040421
0.20016698666	1.0289223689	0.8666301622	1.2216067331	TDP1_C	0.8828040421
0.13117817600	0.9745108948	0.8329202861	1.1401709143	ALCAM	0.8839045293

0.2177936001	1.031186199	0.855239253	1.243330417	IFNGR1	0.883904529
0.2134043456	1.030639032	0.858090003	1.237885083	PRSS2_	0.883904529
0.2053782073	1.029261393	0.862693932	1.227989411	AFP_P0	0.884685650
0.1837465852	1.026022238	0.876018786	1.201711253	ANGPT	0.885580286
0.1435897047	0.972875665	0.819887876	1.154410362	RTN4IP	0.888162951
0.1345088375	0.974727778	0.830520277	1.143974768	CASP3_	0.888189150
0.1313310006	0.975345961	0.834220870	1.140345173	CASQ2_	0.888189150
0.1947816379	1.027236887	0.868457603	1.215045637	PGD_P5	0.888189150
0.1523099596	0.971931500	0.811192526	1.164521135	BGN_P	0.890359494
0.1225938937	0.977310275	0.844934567	1.130425254	CLEC4	0.890359494
0.1493642310	0.972371407	0.814322244	1.161095819	ROR1_	0.890359494
0.1866705142	1.025657808	0.872840736	1.205230113	CRKL_	0.890577985
0.1340889628	0.975516962	0.832215029	1.143494543	CTSV_	0.891180068
0.1853508644	1.025345715	0.873461535	1.203640680	USP8_P	0.891180068
0.1369169948	0.975137644	0.829219582	1.146732959	RANBP	0.891818458
0.1760808765	1.023690185	0.878751593	1.192534502	PPME1_	0.894076532
0.1370170730	0.975479614	0.829718239	1.146847728	RHOC_	0.894076532
0.1753804866	1.023448918	0.878952823	1.191699556	CCL13_	0.894161774
0.1346764368	0.976027725	0.832597448	1.144166514	IL4_P05	0.894161774
0.1776888231	1.023720179	0.877391156	1.194453577	SOX2_	0.894161774
0.1338783030	0.976255280	0.833650823	1.143253680	VAV3_	0.894161774
0.1794466055	1.023881605	0.876126488	1.196555012	KLRC1_	0.894539235
0.1341736935	0.976499838	0.833822205	1.143591436	COL2A	0.894983922
0.1350382090	0.976437036	0.832994509	1.144580516	CPVL_	0.894983922
0.1937647094	1.025573835	0.866528638	1.213810651	DEFB10	0.894983922
0.4198287560	1.055953401	0.732757369	1.521700951	DPEP1_	0.894983922
0.1240049712	0.978546467	0.845878979	1.132021498	GALNT	0.894983922
0.2055617323	1.027055439	0.858842342	1.228214799	NDRG1_	0.894983922
0.1477073605	0.974323776	0.818951362	1.159173626	PRKAR	0.894983922
0.1360550793	0.976574566	0.832382324	1.145744998	CDKN2	0.895242450
0.1639443277	1.021355986	0.885429852	1.178148723	HAGH_	0.895379001
0.1902007997	1.024729507	0.868191093	1.209492439	NUDC_	0.895845409
0.1912847738	1.024743304	0.867273860	1.210804208	EGF_P0	0.895924592
0.1390685112	0.976447121	0.829661183	1.149202830	GBP6_	0.895924592
0.1974635811	1.025422900	0.863075285	1.218308694	MANSC	0.895924592
0.1856838167	1.023914324	0.870734556	1.204041502	PRC1_C	0.895924592
0.1410407646	0.976037255	0.827331508	1.151471586	SIRT2_	0.895924592
0.1848537858	1.023729770	0.871143472	1.203042525	AKR1B	0.895956061

0.19099517072	1.02437887873	0.8669081416	1.2104536064	TIGAR_	0.8971091701
0.1366575346	0.97763427633	0.83368737799	1.1464354668	IST1_P5	0.8999353678
0.13957926229	0.97712008732	0.8303809540	1.1497899370	STAT5E	0.8999353678
0.14714983845	0.9761301530	0.8224492220	1.1585275419	ROBO4_	0.9010685760
0.21330890303	1.02656691599	0.8514039250	1.2377669421	PPM1A_	0.9020158631
0.1784019004	1.02185770088	0.87357839255	1.1953056185	TPRKB_	0.9053099032
0.18809454702	1.0229436550	0.8669918251	1.2069476232	ALDH5	0.9060735475
0.1811878672	1.0218744265	0.8711765371	1.1986403434	CDKN1	0.9081147510
0.14825350047	0.9770708267	0.8231261813	1.1598068706	KIAA20	0.9081147510
0.15886949255	0.9761784308	0.81294707116	1.1721849578	CDH15_	0.9116551608
0.1817097981	1.0212057778	0.8695828460	1.1992661141	CTSF_C	0.9116551608
0.15406663468	0.9768819407	0.8180387430	1.1665686181	CX3CL1	0.9116551608
0.1405387992	0.97917627773	0.8330796798	1.1508937324	DYNLT	0.9116551608
0.1351087500	0.9797065268	0.8385230746	1.14466126	FADD_(	0.9116551608
0.17523668583	1.02044676772	0.8739294661	1.1915282010	IL10RA_	0.9116551608
0.17527306223	1.02053806333	0.8740540529	1.1915715453	MTHFS	0.9116551608
0.19493314628	1.0227880784	0.8608211418	1.21522974	TXNRD	0.9116551608
0.15384108085	1.0181789596	0.8888652011	1.1663055237	VIT_Q6	0.9116551608
0.12405737389	0.9817489649	0.8513800537	1.1320808210	FGF5_P	0.9118646943
0.12963514727	0.9809175116	0.8452110113	1.1384129544	KLB_Q8	0.9118646943
0.1814975734	1.0209701902	0.8693661559	1.1990116272	NPTX1_	0.9118646943
0.14260489322	0.97928687633	0.8315480524	1.1532740452	ARFIP1	0.9129227307
0.13116444123	0.9809697397	0.8440092930	1.1401552544	CUZD1_	0.9129227307
0.1747664248	1.0199580061	0.8735031765	1.1909680035	IL32_P2	0.9129227307
0.15474186467	0.9778139089	0.8190471119	1.1673565862	BMP4_I	0.9132374389
0.1351110802	0.98065796299	0.8401505610	1.1446639268	CXCL6_	0.9132374389
0.1406042260	0.9797965913	0.8340809628	1.1509690343	GMPR2_	0.9132374389
0.11848549627	0.98312758333	0.85854322422	1.1257905458	APOE_I	0.9139012795
0.15307279929	0.9784111580	0.8214178226	1.1654098168	PTH_P0	0.9146868881
0.1945507093	1.02173190199	0.8593728085	1.2147650812	CXCL12	0.9150616627
0.16471727253	1.0182937389	0.8794483621	1.1790597190	PDCD1I	0.9154597373
0.1943889981	1.0212812294	0.8587537184	1.2145686560	CLPP_C	0.9162849813
0.0978355209	0.9865800531	0.8826229874	1.1027813857	GSTT2E	0.9162849813
0.1903425280	1.0207850726	0.8613981039	1.2096638705	LILRA4	0.9162849813
0.14500681808	0.9800774643	0.8308930867	1.1560474522	PDRG1_	0.9162849813
0.13323609013	0.9815343084	0.8432323706	1.1425197041	RAB2B_	0.9162849813
0.14161454055	0.9806416509	0.8346766356	1.1521324625	RGS10_	0.9162849813
0.19061044528	1.0206822793	0.8609939207	1.2099880037	PEBP1_	0.9165998996

0.14584915653	0.98021350722	0.83042397977	1.15702164577	SEMA4I	0.9165998996
0.17401791194	1.01859859977	0.87182863800	1.19007688222	NLGN1_	0.9192518686
0.13367236938	0.98228829044	0.84415998455	1.14301827055	APOC1_	0.9195575971
0.13483737102	0.98231851260	0.84322899455	1.14435066460	CASP8_	0.9199485384
0.17148313422	1.01808043655	0.87315230599	1.18706412188	DCTPP1	0.9199485384
0.16423261095	0.97844829660	0.81236358288	1.17848841255	SMAD3	0.9199485384
0.19149611629	1.01995117188	0.85899978655	1.21106012977	TIGIT_(	0.9222147576
0.18574153667	1.01911330233	0.86253835533	1.20411100160	TYMP_I	0.9242483943
0.14473893548	0.98192486433	0.83425187960	1.15573780877	DNAJA2	0.9258008788
0.14055681387	0.98243968077	0.83862680955	1.15091446560	STAMB	0.9258008788
0.15210889423	0.98108681500	0.82671311060	1.16428701360	RABEPI	0.9258886379
0.15508920563	0.98107744560	0.82423717277	1.16776212744	CLEC1E	0.9272008394
0.19168175362	1.01921547611	0.85760181444	1.21128496855	CXCL11	0.9272008394
0.16650719609	1.01651032855	0.87480334511	1.18117203560	DBNL_(	0.9272008394
0.17894349339	1.01776568499	0.86612671922	1.19595316300	KLRF1_	0.9272008394
0.14209622296	0.98258253355	0.83758033860	1.15268755822	SMAD2	0.9272008394
0.15845897235	1.01561700999	0.88032305260	1.17170385099	CNDP1_	0.9273744373
0.14649427857	0.98236557699	0.83353648660	1.15776830677	TP53_P(	0.9273744373
0.14444423725	0.98272795999	0.83586336277	1.15539726500	INSR_P(	0.9279242512
0.14311513285	0.98296653911	0.83738148911	1.15386264155	SNAP25	0.9281307761
0.17245695445	1.01676586260	0.87005119833	1.18822067199	FGF3_P	0.9283726944
0.17515453416	1.01693661255	0.86799878888	1.19143031900	CRNN_(	0.9288941312
0.17585375947	1.01686888088	0.86727653511	1.19226368860	IDO1_P	0.9297260251
0.17392266287	1.01655004944	0.86840812644	1.18996353399	PDCL2_	0.9297260251
0.13755341148	0.98405351388	0.84391507599	1.14746299200	RNF41_	0.9297260251
0.16980724007	1.01616770855	0.87133354199	1.18507639400	STK24_	0.9297260251
0.17799156491	1.01679237122	0.86529422222	1.19481524288	HRAS_I	0.9308455282
0.13836425161	0.98427538222	0.84361135200	1.14839377833	ITIH5_C	0.9310199085
0.13028251388	0.98541841477	0.85243322888	1.13915016360	HSPB1_	0.9325827873
0.13294291003	0.98515903100	0.84972092588	1.14218478911	NTRK2_	0.9325827873
0.13848869254	0.98458847577	0.84404309522	1.14853669444	S100A3	0.9325827873
0.16965135215	1.01546952188	0.87027225900	1.18489166933	APOB_I	0.9343019280
0.17137679553	1.01553054611	0.86887636855	1.18693789760	ZNF174	0.9348751744
0.18223921528	1.01641800822	0.86099219877	1.19990119422	EFNB2_	0.9349718014
0.20034709194	1.01805539244	0.84826818888	1.22182677077	SUOX_I	0.9349718014
0.18371038362	1.01590627300	0.85886099177	1.20166775000	AKR1B1	0.9384672350
0.14881038237	0.98452078400	0.83526108855	1.16045292600	ANXA3	0.9384672350
0.15301617499	0.98419757900	0.83120951288	1.16534382822	CD226_	0.9384672350

0.19290575784	1.0167630852	0.8524357091	1.2127684942	GP5_P4	0.9384672350
0.16757986404	1.0145339458	0.8704706964	1.1824397208	PECAM	0.9384672350
0.14977793827	0.9842848230	0.8340533782	1.1615762724	TLR4_C	0.9384672350
0.16653403170	1.0142108831	0.8708266713	1.1812037335	RET_P0	0.9402548874
0.13681526161	0.9862627173	0.8483344807	1.1466163049	ANKRD	0.9409583507
0.15466489424	0.9849165889	0.8310531396	1.1672667377	BHMT2	0.9415036073
0.15248987411	0.9850829802	0.8331440946	1.1647306681	CALCB	0.9415036073
0.27018453876	1.0223437404	0.7977268874	1.3102062122	ENDOU	0.9415036073
0.17617266809	1.0144681955	0.8629110980	1.1926439724	GP6_Q9	0.9415036073
0.16175830392	1.0134575687	0.8736961100	1.1755760748	GZMH_	0.9415036073
0.17589188158	1.0146486311	0.8634604979	1.1923091411	LAG3_P	0.9415036073
0.18622169833	1.0153296130	0.8557345177	1.2046893083	NMI_Q1	0.9415036073
0.16882715208	1.0140851364	0.8686166183	1.1839154839	PPP1R2	0.9415036073
0.13408494939	0.9873757123	0.8525748683	1.1434899543	ACP1_P	0.9425983267
0.14972234584	0.9857866919	0.8366471061	1.1615116994	CRYZL1	0.9425983267
0.15837511972	1.0126781215	0.8753090407	1.1716056040	HS3ST3	0.9425983267
0.14198170628	0.9865950089	0.8445317017	1.1525555638	LMOD1	0.9425983267
0.16569271459	1.0134275854	0.8702138909	1.1802103845	SNAPIN	0.9425983267
0.15810957843	0.9852915944	0.8288261372	1.1712945363	PYY_P1	0.9432618128
0.15427972503	0.9858936910	0.8330236696	1.1668172291	EPHA4_	0.9444131398
0.15707550058	0.9856182639	0.8302338990	1.1700839525	FGF9_P	0.9444131398
0.19417518349	1.0150413236	0.8484734083	1.2143089912	TREML	0.9455690166
0.16528544211	1.0126346891	0.8692066610	1.1797298152	ENTPD	0.9468112581
0.14841131751	0.9868580410	0.8395665982	1.1599899224	CA8_P3	0.9468506450
0.15317588694	0.9865800530	0.8351052589	1.1655299624	PTPRS_	0.9475979677
0.16421637504	1.0117707508	0.8686523023	1.1784692788	CASP10	0.9485536477
0.17420410694	1.0126515633	0.8615176765	1.1902984892	CDC37_	0.9485536477
0.14305543738	0.9879037070	0.8458649775	1.1537937632	CIRBP_	0.9485536477
0.19326477331	1.0141131097	0.8476937270	1.2132039750	DECR1_	0.9485536477
0.15182135990	0.9869250974	0.8368222280	1.1639522893	MORF4	0.9485536477
0.14802389068	0.9874826295	0.8409554139	1.1595405982	NFATC	0.9485536477
0.16534241041	0.9862536812	0.8244607360	1.1797970243	PNLIPR	0.9485536477
0.14945337810	0.9876189733	0.8399860460	1.1611993322	PTPN9_	0.9485536477
0.17191487076	1.0125079760	0.8632472964	1.1875767314	STOML	0.9485536477
0.16670869565	0.9856969295	0.8224057552	1.1814100652	TMEM1	0.9485536477
0.14344482123	0.9881051692	0.8458805680	1.1542431193	VAMP8	0.9485536477
0.17459605127	1.0123075213	0.8605950140	1.1907651113	GPR101	0.9502962289
0.14529975752	0.9884147874	0.8448421739	1.1563861537	MRPL5	0.9515504238

0.16624037993	1.0111833176	0.8658895780	1.1808569219	CA7_P4	0.9525817074
0.20141489906	1.0135434581	0.8398686498	1.2231321429	CBLN4_	0.9525817074
0.15846462340	0.9877289891	0.8326362006	1.1717104723	DNAJB1	0.9525817074
0.20162267909	1.0137608099	0.8400543391	1.2233863117	EREG_(	0.9525817074
0.14312753557	0.9889651942	0.8476225763	1.1538769526	NPPB_P	0.9525817074
0.14537548816	0.9886874367	0.8452443158	1.1564737309	SH3GLI	0.9525817074
0.17927673711	1.0118146623	0.8557423776	1.1963517732	CTF1_Q	0.9533028427
0.17991042192	1.0119599262	0.8554458543	1.1971101234	FKBP4_	0.9533028427
0.16714308073	1.0110554460	0.8648895066	1.1819233636	NLGN2_	0.9533028427
0.14501238687	0.9895518266	0.8470304247	1.1560538900	RPA2_P	0.9569974683
0.19053707018	1.0113166504	0.8453277324	1.2098992239	ACHE_I	0.9579093263
0.15448646944	0.9904274296	0.8405289917	1.16705849	ADGRF	0.9579093263
0.15483175918	1.0085235829	0.8712234119	1.1674615298	BLVRB_	0.9579093263
0.17130656606	1.0100703487	0.8596184896	1.1868545425	CACYB	0.9579093263
0.17111917428	1.01036641	0.8602836885	1.1866321566	CASP2_	0.9579093263
0.16832765569	1.0104048547	0.8627541886	1.1833242700	CDC42E	0.9579093263
0.17538985856	1.0095621735	0.8552543508	1.1917107246	DCTD_I	0.9579093263
0.18780256891	1.0114722512	0.8479033014	1.2065952724	DTYMK	0.9579093263
0.18162882049	1.0103880590	0.8513262319	1.1991690043	GMFG_	0.9579093263
0.17333722256	1.0100468833	0.8578348121	1.1892670851	ITGB5_	0.9579093263
0.17950689879	1.0099855752	0.8524550478	1.1966271593	LRTM1_	0.9579093263
0.13924626038	1.0085530077	0.8849598658	1.1494071185	LY75_O	0.9579093263
0.15597168153	0.9889371402	0.8367577322	1.1687931041	MAX_P	0.9579093263
0.18908978390	1.0105346044	0.8452432868	1.2081494199	MFAP4_	0.9579093263
0.14850540813	0.9900517305	0.8449299315	1.1600990717	NSFL1C	0.9579093263
0.17279234454	1.0094710454	0.8573239801	1.1886192561	PARK7_	0.9579093263
0.19523036674	1.0110747113	0.8409671374	1.2155909859	PCDH9_	0.9579093263
0.14283677036	0.9906502733	0.8507608691	1.1535414941	PRKG1_	0.9579093263
0.16725603166	1.0092252151	0.8616637323	1.1820568705	QDPR_I	0.9579093263
0.15704204366	0.9894466104	0.8367240212	1.1700448058	RAB27E	0.9579093263
0.17359216065	1.0101110566	0.8577251258	1.1895703133	RRAS_I	0.9579093263
0.14244423833	0.9906479820	0.8510909484	1.15308878	SCARF2	0.9579093263
0.16427386000	1.0089532189	0.8637714186	1.1785370250	SEMA6	0.9579093263
0.18964073978	1.0109373457	0.8454512176	1.2088152404	SHBG_I	0.9579093263
0.18827267257	1.0109321420	0.8465999277	1.2071626306	TGFBR3	0.9579093263
0.17339078737	1.0101017777	0.8578821049	1.1893307897	TMPRS	0.9579093263
0.19328448410	1.0109487366	0.8423951987	1.2132278884	TNFSF8	0.9579093263
0.14484389735	0.9903482387	0.8485373469	1.1558591235	UROS_I	0.9579093263

0.16957725558	1.00914334552	0.85952646833	1.18480387622	CHP1_C	0.9584184071
0.15746714988	0.99073644600	0.83855038933	1.17054230488	DNAJB1	0.9585260968
0.16829862817	1.00886222623	0.86014682099	1.18328992155	PCSK7_	0.9585260968
0.17756665141	1.00938938888	0.85310257522	1.19430765755	PDAP1_	0.9585260968
0.14579291102	0.99150538255	0.84971462955	1.15695657022	TNNI3_	0.9585260968
0.15451703470	0.99123282455	0.84187081644	1.16709415899	PLXDC2	0.9601880662
0.10736178183	0.99395556577	0.88737524777	1.11333696677	PAEP_P	0.9603853961
0.10737307104	1.00539388099	0.90790611900	1.11334953544	FSHB_P	0.9609817275
0.13002669816	1.00646779133	0.88946709222	1.13885878833	ADAM1	0.9613563704
0.17262347670	1.00847363722	0.85577515933	1.18841855355	DNAJB6	0.9614995858
0.15357780262	0.99167274200	0.84341002699	1.16599850133	PDE5A_	0.9614995858
0.17587719658	1.00847312044	0.85299435744	1.19229163211	NFU1_C	0.9625406594
0.15333776077	0.99195123077	0.84408639011	1.16571864644	PSME1_	0.9625406594
0.16125690675	1.00764737544	0.86414012499	1.17498679200	CLEC3E	0.9627590100
0.15194217470	0.99216965966	0.84563750555	1.16409292044	CRADD	0.9628382645
0.17094854881	1.00793705211	0.85629776255	1.18642970422	ERN1_C	0.9634222623
0.14199846522	0.99293067633	0.85539893811	1.15257487955	CA1_P0	0.9643251943
0.14626063001	0.99282893666	0.85158630455	1.15749782744	MCEE_C	0.9643251943
0.17871287115	1.00807953400	0.84991517188	1.19567738144	SERPIN	0.9643251943
0.11802715944	0.99431981377	0.87860494500	1.12527467277	CST5_P	0.9648640888
0.15763911410	0.99250825922	0.84140766033	1.17074361355	FKBP14	0.9648640888
0.16256864576	1.00713966200	0.86213789066	1.17652907944	IL11_P2	0.9648640888
0.13165063033	1.00569616811	0.88666271944	1.14070972	SERPIN	0.9650207983
0.16128188586	0.99256138211	0.83843792577	1.17501614255	SMC3_C	0.9658278452
0.15678581867	0.99288561044	0.84276640955	1.16974504955	MTIF3_	0.9660179821
0.18305268338	1.00764957822	0.84551299077	1.20087767277	NCR1_C	0.9660179821
0.15790370360	0.99302012055	0.84205292611	1.17105342100	B4GALT	0.9668449976
0.15426541197	0.99322026600	0.84546284811	1.16680052855	VPS28_C	0.9668449976
0.16804742957	0.99281240477	0.83320586500	1.18299271811	EIF5A_I	0.9674746423
0.16188077858	0.99307600700	0.83880507599	1.17572006199	SMAD1_	0.9674746423
0.14967192246	0.99374185844	0.85024772222	1.16145313355	EIF5_P5	0.9683668515
0.16267013125	0.99333165100	0.83857480011	1.17664848611	S100A4_	0.9690176600
0.16026736265	1.00588821388	0.86197805188	1.17382466599	APOA4_	0.9706853574
0.20054610394	1.00732698711	0.83031880166	1.22206995311	CD55_P	0.9706853574
0.16656531616	0.99361839888	0.83579708433	1.18124068744	CPE_P1	0.9713570892
0.16791488939	1.00570718244	0.85510332188	1.18283593444	DCN_P0	0.9739058223
0.17119513703	1.00563461833	0.85217997977	1.18672229999	GPI_P06	0.9748368950
0.23682870158	1.00777457600	0.80144439866	1.26722402577	HEPACA	0.9748368950

0.15507472885	1.00468750459	0.8643982974	1.1677452221	ALPI_P(	0.9757598594
0.14753919040	0.99541376922	0.8549325089	1.1589787047	AP1G2_	0.9757598594
0.16417813243	0.99456201200	0.8393866876	1.1784242119	EIF4B_I	0.9757598594
0.16325684960	0.99494614955	0.8408094851	1.1773390499	HPCAL1	0.9757598594
0.14154596048	0.99554191730	0.8602931639	1.1520534519	LMOD1	0.9757598594
0.14969233279	0.99496524930	0.8523250863	1.1614768394	MANF_	0.9757598594
0.16620250468	1.00517628830	0.8556647472	1.1808121975	NID1_P	0.9757598594
0.16492632111	1.00485361510	0.8562074599	1.1793062255	STX8_Q	0.9757598594
0.17894165759	1.00535823560	0.8451393155	1.1959509674	TNFAIP	0.9757598594
0.15215183840	0.99547771080	0.8511074206	1.1643370140	AIF1_P5	0.9768714328
0.16502399634	0.99513416122	0.8396422025	1.1794214201	PAPPA_	0.9768714328
0.13224307324	1.00362725340	0.8824954098	1.1413857258	CD109_	0.9772083921
0.16125310956	0.99578326870	0.8439142381	1.1749823304	ACADM	0.9808231085
0.15214073200	1.00374295990	0.8653088471	1.1643240825	LGALS8	0.9808426110
0.16832834524	0.99585282620	0.8380814902	1.1833250860	BRD1_(	0.9813654718
0.15659509336	1.00354168710	0.8611175703	1.1695219708	DENR_(	0.9813654718
0.16262973043	1.00374760940	0.8562879911	1.1766009495	DOK2_(	0.9813654718
0.15731456797	0.99608276420	0.8477543011	1.1703637149	REXO2_	0.9813654718
0.16665297147	1.00377076600	0.8528892112	1.1813442340	TBCB_(	0.9813654718
0.16081945471	0.99650429410	0.8455033783	1.1744729041	ARHGE	0.9822511932
0.14154934577	0.99682373770	0.8625070292	1.1520573520	CCL27_	0.9822511932
0.17988888537	0.99612215420	0.8288967713	1.1970843421	GFRA2_	0.9822511932
0.15291460654	0.99669994000	0.8525481071	1.1652254720	RAB39B	0.9822511932
0.16146213708	0.99659840210	0.8451197633	1.1752279597	TK1_P0	0.9824496606
0.16826120854	1.00321696750	0.8505793272	1.1832456441	ARG1_I	0.9826998642
0.15922943438	1.00307288380	0.8580498422	1.1726069521	SIRT5_(	0.9826998642
0.17289935810	1.00333528480	0.8468430623	1.1887464613	USO1_(	0.9826998642
0.17717878103	1.00330044020	0.8431682387	1.1938445108	TREML	0.9829535387
0.16446751483	1.00299436070	0.8534334253	1.1787652765	PRSS27_	0.9830859141
0.19107230400	1.00323617820	0.8314281469	1.2105469763	CD58_P	0.9832224885
0.14887104955	0.99742425670	0.8572470044	1.1605233295	FGF2_P	0.9832224885
0.20383582136	0.99642082130	0.8097683824	1.2260968380	MCEMP	0.9832224885
0.15847552724	1.00266338020	0.8579959946	1.1717232485	TERF1_	0.9832224885
0.15730235466	1.00250782380	0.8587366464	1.1703494210	CINP_Q	0.9834569324
0.15440632267	1.00249200120	0.8611999944	1.1669649547	F2_P007	0.9834569324
0.17385899173	0.99739933790	0.8360498061	1.1898877699	BACH1_	0.9838231880
0.18154098236	1.00260643360	0.8383371798	1.1990636761	CCL28_	0.9838231880
0.15837670726	1.00240040410	0.8576307342	1.1716074640	NRXN3_	0.9838231880

0.161580057960	1.00237449777	0.85484365030	1.17536655188	SCN2B_	0.98382318800
0.16101572649	1.00169810588	0.85417226066	1.17470344260	TIMM8A	0.98940342322
0.12604028246	0.99880057900	0.87946583277	1.13432786088	ABO_P1	0.99020645133
0.10894577345	0.99902384500	0.89502910922	1.11510188055	MDGA1	0.99020645133
0.15432089809	0.99864193688	0.85467083677	1.16686527155	NT5C_C	0.99020645133
0.15753688783	0.99845543860	0.85160847111	1.17062393899	PPIE_Q	0.99020645133
0.15631162029	0.99889207699	0.85339847533	1.16919048988	TDO2_P	0.99230103788
0.16383451940	0.99913597060	0.84741619877	1.17801935960	MSRA_	0.99422296911
0.15326957119	0.99921318399	0.85654894055	1.16563915933	TAX1BI	0.99422296911
0.13745802516	0.99939122499	0.87051007499	1.14735354499	IL31RA_	0.99476323411
0.15869120191	1.00058991300	0.85426679844	1.17197598688	NCK2_C	0.99527437933
0.14797414298	1.00036850660	0.86308917188	1.15948291522	MLN_P1	0.99665109800
0.15212705379	0.99980760177	0.85854868800	1.16430815677	ARL2BI	0.99802447211

8901e-24
5352e-16
4554e-15
1053e-15
6478e-13
4327e-12
5224e-12
5318e-11
3902e-11
1425e-10
0038e-10
2011e-10
2383e-09
8306e-09
3168e-09
8321e-09
0292e-08
8354e-08
8223e-07
5959e-07
7383e-07
7847e-07
5301e-07
2402e-07
5658e-06
4066e-06
7246e-06
0676e-06
7153e-06
7164e-06
7164e-06
2229e-06
2913e-06
3772e-06

1081e-06
4996e-06
0557e-06
0557e-06
1347e-06
1347e-06
7179e-06
1842e-06
1743e-06
9646e-06

9943e-06
2991e-06
2991e-06
2309e-05
7262e-05
3259e-05
9508e-05
7416e-05
7416e-05
6385e-05
6385e-05
3809e-05
0047e-05
7738e-05
5674e-05
1963e-05
3887e-05
2033e-05
0733e-05
7761e-05
8168e-05
3575e-05
5845e-05
0772e-05
0499e-05
6073e-05
1803e-05

40542e-05
14491e-05
17115e-05
17115e-05
51973e-05
51973e-05
51973e-05
18241e-05
94834e-05
721801493
441261162
489254557
076823529
502509081
708486214
517046616
361463559
165691397
165691397
165691397
303389476
777551417
50983911
55804079
116087948
933480137
060977304
060977304
592966511
234099754
00578335
00578335
554180976
554180976
192754221
589082626
981435492

48867435
48867435
50221018
79161584
19727629
19727629
00371454
00371454
96204181
37599872
45150808
45150808
85811496
11405457
33964755
59772111
20399398
20613623
59997785
28750141
42701724
83789771
22180556
72443778
86755186
01241726
30596451
36367846
36367846
36367846
74639254
40444073
78235758
23819959
23819959
10022675
88610665
0931448

0931448
2260134
0768316
9682215
6531597
0799779

9263479
1787548

3455744
8723889
2872332
2872332
0232132
6065143
3444494
3444494
4686973
6259442
6871238

1583276
1901174
6065589
9042603
1877367
4439871
4265531
4265531
6391382
3275586
5096721
0395318
0395318
3002495

3002495
7479118
7479118
0488667
9436365
4021392
4816335
5666404
3799489
4510413
9599394
0642988
1370498
3807893
3508572
2952693
7019367
7019367
5940575
8900834
5152292
5140582

9589131
9589131
6935472
5942168
9420917
5514224
8466908
4589165
5376396
9482054
1057193
1350216

30406648
20162544
96131772
01885226
79815142
24975511
32348462
27451896
27451896
18856089

23031286
09814898
42132454

72212591
72212591
72212591
72212591
20619255
20619255
25509047

29296813
61613633
71983878
71983878

Supplementary Table S11: Proteins that remained significantly associated with sı
protein names
PLAUR_Q03405_OID20764_v1_Inflammation
CXCL17_Q6UXB2_OID20622_v1_Inflammation
WFDC2_Q14508_OID21505_v1_Oncology
CCL21_O00585_OID20686_v1_Inflammation
TNFRSF10B_O14763_OID20981_v1_Neurology
PRSS8_Q16651_OID20763_v1_Inflammation
CRELD1_Q96HD1_OID30619_v1_Inflammation_II
SIGLEC7_Q9Y286_OID20190_v1_Cardiometabolic
PIGR_P01833_OID21102_v1_Neurology
IL10RB_Q08334_OID20696_v1_Inflammation
TIMP1_P01033_OID20418_v1_Cardiometabolic
GDF15_Q99988_OID20251_v1_Cardiometabolic
OSCAR_Q8IYS5_OID20776_v1_Inflammation
ACVRL1_P37023_OID20999_v1_Neurology
MMP12_P39900_OID21439_v1_Oncology
CD27_P26842_OID21527_v1_Oncology
ANGPT2_O15123_OID21463_v1_Oncology
SERPINB8_P50452_OID20630_v1_Inflammation
EFEMP1_Q12805_OID20414_v1_Cardiometabolic
ICAM1_P05362_OID20411_v1_Cardiometabolic
ASGR1_P07306_OID20990_v1_Neurology
SCGB1A1_P11684_OID20688_v1_Inflammation
CTSD_P07339_OID20358_v1_Cardiometabolic
CAPG_P40121_OID21431_v1_Oncology
TNFRSF1A_P19438_OID21155_v1_Neurology
HAVCR2_Q8TDQ0_OID21137_v1_Neurology
FUT3_FUT5_P21217_Q11128_OID21013_v1_Neurology
LILRA5_A6NI73_OID20209_v1_Cardiometabolic
MMP7_P09237_OID20087_v1_Cardiometabolic
EFNA4_P52798_OID20935_v1_Neurology
SFTPD_P35247_OID20214_v1_Cardiometabolic
TFF2_Q03403_OID20787_v1_Inflammation
IFNLR1_Q8IU57_OID20506_v1_Inflammation
CHRDL1_Q9BU40_OID20771_v1_Inflammation
IGFBP4_P22692_OID21158_v1_Neurology
BMPER_Q8N8U9_OID30615_v1_Inflammation_II

FSTL3_O95633_OID20782_v1_Inflammation
CFB_P00751_OID30755_v1_Inflammation_II
SPON2_Q9BUD6_OID20221_v1_Cardiometabolic
GFRA1_P56159_OID21457_v1_Oncology
CD300E_Q496F6_OID21418_v1_Oncology
CD300LF_Q8TDQ1_OID21469_v1_Oncology
TNFRSF10A_O00220_OID20967_v1_Neurology
NCAN_O14594_OID21055_v1_Neurology
SIGLEC6_O43699_OID21394_v1_Oncology
IL2RA_P01589_OID20267_v1_Cardiometabolic
CD83_Q01151_OID20565_v1_Inflammation
LILRB4_Q8NHJ6_OID20429_v1_Inflammation
RNASE6_Q93091_OID30689_v1_Inflammation_II
GLA_P06280_OID30594_v1_Inflammation_II
PGF_P49763_OID20673_v1_Inflammation
MSR1_P21757_OID21063_v1_Neurology
EDA2R_Q9HAV5_OID21451_v1_Oncology
SPON1_Q9HCB6_OID20759_v1_Inflammation
CDCP1_Q9H5V8_OID20940_v1_Neurology
VWA1_Q6PCB0_OID21421_v1_Oncology
COLEC12_Q5KU26_OID20738_v1_Inflammation
IL18BP_O95998_OID20348_v1_Cardiometabolic
TPP1_O14773_OID20750_v1_Inflammation
CTSZ_Q9UBR2_OID20365_v1_Cardiometabolic
HGF_P14210_OID20656_v1_Inflammation
HAVCR1_Q96D42_OID21422_v1_Oncology
ADM_P35318_OID21467_v1_Oncology
SPINT1_O43278_OID21109_v1_Neurology
CLEC4D_Q8WXI8_OID20609_v1_Inflammation
MRC1_P22897_OID30694_v1_Inflammation_II
APLP1_P51693_OID20164_v1_Cardiometabolic
CD207_Q9UJ71_OID21298_v1_Oncology
TGFBR2_P37173_OID21493_v1_Oncology
CRHBP_P24387_OID20747_v1_Inflammation
RNASE1_P07998_OID30672_v1_Inflammation_II
TNFRSF1B_P20333_OID21145_v1_Neurology
CSF1_P09603_OID20719_v1_Inflammation
FAS_P25445_OID20316_v1_Cardiometabolic

F9_P00740_OID20349_v1_Cardiometabolic
LILRB1_Q8NHL6_OID20323_v1_Cardiometabolic
GGH_Q92820_OID20416_v1_Cardiometabolic
NCAM1_P13591_OID20354_v1_Cardiometabolic
CD300C_Q08708_OID21010_v1_Neurology
COL6A3_P12111_OID20292_v1_Cardiometabolic
PLA2G2A_P14555_OID20274_v1_Cardiometabolic
LBP_P18428_OID20355_v1_Cardiometabolic
OMD_Q99983_OID20725_v1_Inflammation
TNFRSF12A_Q9NP84_OID21482_v1_Oncology
FAM20A_Q96MK3_OID30348_v1_Cardiometabolic_II
IL6_P05231_OID21276_v1_Oncology
MSLN_Q13421_OID21380_v1_Oncology
CLEC6A_Q6EIG7_OID21228_v1_Oncology
TNFRSF9_Q07011_OID20985_v1_Neurology
C1RL_Q9NZP8_OID30721_v1_Inflammation_II
LAIR1_Q6GTX8_OID20737_v1_Inflammation
S100A11_P31949_OID20130_v1_Cardiometabolic
CHCHD10_Q8WYQ3_OID31116_v1_Neurology_II
CD74_P04233_OID21040_v1_Neurology
CD4_P01730_OID20584_v1_Inflammation
TNFRSF13B_O14836_OID20702_v1_Inflammation
MSMB_P08118_OID20275_v1_Cardiometabolic
LRRN1_Q6UXK5_OID20438_v1_Inflammation
QPCT_Q16769_OID20320_v1_Cardiometabolic
CKAP4_Q07065_OID20617_v1_Inflammation
RARRES2_Q99969_OID20373_v1_Cardiometabolic
FOLR2_P14207_OID21062_v1_Neurology
TNFRSF4_P43489_OID20653_v1_Inflammation
ACE2_Q9BYF1_OID20105_v1_Cardiometabolic
IL6_P05231_OID20563_v1_Inflammation
BCAN_Q96GW7_OID20998_v1_Neurology
CA6_P23280_OID21096_v1_Neurology
LGALS1_P09382_OID20154_v1_Cardiometabolic
IGFBPL1_Q8WX77_OID20155_v1_Cardiometabolic
CCL20_P78556_OID20671_v1_Inflammation
FABP4_P15090_OID20245_v1_Cardiometabolic
ADAMTS15_Q8TE58_OID21275_v1_Oncology

FURIN_P09958_OID21514_v1_Oncology
GM2A_P17900_OID30413_v1_Cardiometabolic_II
IL1RN_P18510_OID20700_v1_Inflammation
CHI3L1_P36222_OID20399_v1_Cardiometabolic
PLTP_P55058_OID20238_v1_Cardiometabolic
FABP3_P05413_OID30351_v1_Cardiometabolic_II
SERPINF1_P36955_OID30757_v1_Inflammation_II
CCL18_P55774_OID20395_v1_Cardiometabolic
TNFSF13B_Q9Y275_OID20272_v1_Cardiometabolic
ACAN_P16112_OID20159_v1_Cardiometabolic
SFRP1_Q8N474_OID20984_v1_Neurology
SLITRK1_Q96PX8_OID30510_v1_Inflammation_II
IL7R_P16871_OID21136_v1_Neurology
CD80_P33681_OID30291_v1_Cardiometabolic_II
BPIFB1_Q8TDL5_OID20308_v1_Cardiometabolic
CLMP_Q9H6B4_OID21419_v1_Oncology
SELE_P16581_OID20290_v1_Cardiometabolic
TNFRSF11B_O00300_OID20735_v1_Inflammation
VWC2_Q2TAL6_OID20936_v1_Neurology
IL6_P05231_OID20911_v1_Neurology
SCARB2_Q14108_OID20943_v1_Neurology
FGF23_Q9GZV9_OID21411_v1_Oncology
LILRB2_Q8N423_OID20301_v1_Cardiometabolic
EPHB4_P54760_OID20242_v1_Cardiometabolic
PLG_P00747_OID30785_v1_Inflammation_II
UMOD_P07911_OID20237_v1_Cardiometabolic
S100A12_P80511_OID21374_v1_Oncology
SERPINA1_P01009_OID30750_v1_Inflammation_II
ADAMTSL2_Q86TH1_OID30370_v1_Cardiometabolic_II
RNASE4_P34096_OID30705_v1_Inflammation_II
CFH_P08603_OID30790_v1_Inflammation_II
LRTM2_Q8N967_OID31020_v1_Neurology_II
CLEC10A_Q8IUN9_OID20928_v1_Neurology
S100A13_Q99584_OID30529_v1_Inflammation_II
LTA4H_P09960_OID21452_v1_Oncology
LRIG1_Q96JA1_OID21429_v1_Oncology
AGRN_O00468_OID20786_v1_Inflammation
GPD1_P21695_OID31358_v1_Oncology_II

TREM2_Q9NZC2_OID20731_v1_Inflammation
LGMN_Q99538_OID20773_v1_Inflammation
IGSF3_O75054_OID21312_v1_Oncology
PLXNB2_O15031_OID20317_v1_Cardiometabolic
ASGR2_P07307_OID30679_v1_Inflammation_II
LCN2_P80188_OID20392_v1_Cardiometabolic
BSG_P35613_OID20626_v1_Inflammation
CD79B_P40259_OID20635_v1_Inflammation
PLAT_P00750_OID20247_v1_Cardiometabolic
TNFSF11_O14788_OID20592_v1_Inflammation
CEACAM8_P31997_OID20102_v1_Cardiometabolic
ST3GAL1_Q11201_OID21322_v1_Oncology
LEPR_P48357_OID20128_v1_Cardiometabolic
TFPI2_P48307_OID21512_v1_Oncology
CD99L2_Q8TCZ2_OID21122_v1_Neurology
ULBP2_Q9BZM5_OID20919_v1_Neurology
CFI_P05156_OID30772_v1_Inflammation_II
CST3_P01034_OID20400_v1_Cardiometabolic
REG3A_Q06141_OID20258_v1_Cardiometabolic
HSPB6_O14558_OID21408_v1_Oncology
FSTL1_Q12841_OID30254_v1_Cardiometabolic_II
CLSTN3_Q9BQT9_OID31092_v1_Neurology_II
SERPINA3_P01011_OID30786_v1_Inflammation_II
BLNK_Q8WV28_OID30541_v1_Inflammation_II
ERBB2_P04626_OID21520_v1_Oncology
DLL1_O00548_OID21525_v1_Oncology
SEZ6L_Q9BYH1_OID21488_v1_Oncology
CCL22_O00626_OID20765_v1_Inflammation
MMP9_P14780_OID21103_v1_Neurology
NHLRC3_Q5JS37_OID30614_v1_Inflammation_II
OGN_P20774_OID21123_v1_Neurology
CTHRC1_Q96CG8_OID30393_v1_Cardiometabolic_II
STAB2_Q8WWQ8_OID30401_v1_Cardiometabolic_II
SIT1_Q9Y3P8_OID20462_v1_Inflammation
KITLG_P21583_OID20196_v1_Cardiometabolic
IL18R1_Q13478_OID20652_v1_Inflammation
OSM_P13725_OID20574_v1_Inflammation
CCL7_P80098_OID20523_v1_Inflammation

CLEC7A_Q9BXN2_OID20636_v1_Inflammation
GSTA3_Q16772_OID21242_v1_Oncology
CD14_P08571_OID20378_v1_Cardiometabolic
INHBB_P09529_OID30625_v1_Inflammation_II
IL6_P05231_OID20101_v1_Cardiometabolic
TNFRSF6B_O95407_OID20964_v1_Neurology
VEGFA_P15692_OID20650_v1_Inflammation
RBP7_Q96R05_OID31526_v1_Oncology_II
SERPIND1_P05546_OID30767_v1_Inflammation_II
GFAP_P14136_OID21247_v1_Oncology
CST7_O76096_OID20704_v1_Inflammation
IL17C_Q9P0M4_OID20477_v1_Inflammation
CEACAM21_Q3KPI0_OID20548_v1_Inflammation
CD72_P21854_OID30641_v1_Inflammation_II
LECT2_O14960_OID30320_v1_Cardiometabolic_II
ANG_P03950_OID20403_v1_Cardiometabolic
PRCP_P42785_OID20337_v1_Cardiometabolic
RELT_Q969Z4_OID21142_v1_Neurology
IL15_P40933_OID20562_v1_Inflammation
BPIFA2_Q96DR5_OID30279_v1_Cardiometabolic_II
FBP1_P09467_OID20227_v1_Cardiometabolic
GSTA1_P08263_OID20166_v1_Cardiometabolic
THBS2_P35442_OID21104_v1_Neurology
CFHR2_P36980_OID30720_v1_Inflammation_II
DNER_Q8NFT8_OID20712_v1_Inflammation
OMG_P23515_OID21352_v1_Oncology
CGREF1_Q99674_OID20152_v1_Cardiometabolic
REG4_Q9BYZ8_OID20784_v1_Inflammation
SIGLEC9_Q9Y336_OID21390_v1_Oncology
CFD_P00746_OID30738_v1_Inflammation_II
ACP5_P13686_OID20314_v1_Cardiometabolic
IL1RAP_Q9NPH3_OID21126_v1_Neurology
ADAM12_O43184_OID30491_v1_Inflammation_II
GUSB_P08236_OID20296_v1_Cardiometabolic
RNF149_Q8NC42_OID30235_v1_Cardiometabolic_II
ITGA5_P08648_OID21139_v1_Neurology
TFF1_P04155_OID21154_v1_Neurology
TNFSF14_O43557_OID20953_v1_Neurology

ALDH3A1_P30838_OID20512_v1_Inflammation
NPTXR_O95502_OID20191_v1_Cardiometabolic
NTRK3_Q16288_OID21057_v1_Neurology
CNTN5_O94779_OID21006_v1_Neurology
WFIKKN2_Q8TEU8_OID20785_v1_Inflammation
FABP1_P07148_OID20778_v1_Inflammation
RGMA_Q96B86_OID21065_v1_Neurology
ENPP5_Q9UJA9_OID20736_v1_Inflammation
IGDCC4_Q8TDY8_OID30402_v1_Cardiometabolic_II
CHGB_P05060_OID21038_v1_Neurology
TNR_Q92752_OID20957_v1_Neurology
OXT_P01178_OID21047_v1_Neurology
VWC2L_B2RUY7_OID31002_v1_Neurology_II
CPM_P14384_OID21054_v1_Neurology
SULT2A1_Q06520_OID20508_v1_Inflammation
NELL2_Q99435_OID20706_v1_Inflammation
OPTC_Q9UBM4_OID21362_v1_Oncology
RBP5_P82980_OID21369_v1_Oncology
NOTCH3_Q9UM47_OID20253_v1_Cardiometabolic
CNTN1_Q12860_OID20307_v1_Cardiometabolic
SPINK6_Q6UWN8_OID21450_v1_Oncology
AMY1A_AMY1B_AMY1C_P0DUB6_P0DTE7_P0DTE8_OID30707_v1_Infla
APCS_P02743_OID30778_v1_Inflammation_II
PTGR1_Q14914_OID30297_v1_Cardiometabolic_II
SCG3_Q8WXD2_OID20728_v1_Inflammation
ACY1_Q03154_OID20137_v1_Cardiometabolic
CCL16_O15467_OID20334_v1_Cardiometabolic
AMY2A_P04746_OID20333_v1_Cardiometabolic
PLXDC1_Q8IUK5_OID21258_v1_Oncology
BST2_Q10589_OID21029_v1_Neurology
CST1_P01037_OID30581_v1_Inflammation_II
CDH2_P19022_OID20170_v1_Cardiometabolic
AMY2B_P19961_OID20340_v1_Cardiometabolic
ART3_Q13508_OID20346_v1_Cardiometabolic
FETUB_Q9UGM5_OID20404_v1_Cardiometabolic
SERPING1_P05155_OID30777_v1_Inflammation_II
LGALS9_O00182_OID20781_v1_Inflammation
FABP2_P12104_OID20200_v1_Cardiometabolic

CFHR5_Q9BXR6_OID30716_v1_Inflammation_II
ENPP6_Q6UWR7_OID30273_v1_Cardiometabolic_II
FCN1_O00602_OID30690_v1_Inflammation_II
NPL_Q9BXD5_OID30329_v1_Cardiometabolic_II
MOG_Q16653_OID21384_v1_Oncology
CA4_P22748_OID20241_v1_Cardiometabolic
CCL3_P10147_OID20610_v1_Inflammation
AMBP_P02760_OID21413_v1_Oncology
ANGPTL4_Q9BY76_OID20703_v1_Inflammation
TFF3_Q07654_OID20371_v1_Cardiometabolic
HYAL1_Q12794_OID20357_v1_Cardiometabolic
C1S_P09871_OID30731_v1_Inflammation_II
IL12RB2_Q99665_OID30422_v1_Inflammation_II
A1BG_P04217_OID30771_v1_Inflammation_II
ADH4_P08319_OID20117_v1_Cardiometabolic
AFM_P43652_OID30741_v1_Inflammation_II
LCP1_P13796_OID30367_v1_Cardiometabolic_II
PTPRR_Q15256_OID31099_v1_Neurology_II
EBI3_IL27_Q14213_Q8NEV9_OID21389_v1_Oncology
KRT18_P05783_OID21296_v1_Oncology
ICAM5_Q9UMF0_OID20145_v1_Cardiometabolic
LGALS3BP_Q08380_OID30765_v1_Inflammation_II
AGR3_Q8TD06_OID21243_v1_Oncology
PODXL2_Q9NZ53_OID21487_v1_Oncology
F11_P03951_OID30773_v1_Inflammation_II
IL18_Q14116_OID20694_v1_Inflammation
ANXA10_Q9UJ72_OID20886_v1_Neurology
COL18A1_P39060_OID20332_v1_Cardiometabolic
APOD_P05090_OID30746_v1_Inflammation_II
CPB2_Q96IY4_OID30734_v1_Inflammation_II
LGALS4_P56470_OID20748_v1_Inflammation
NPTX2_P47972_OID30390_v1_Cardiometabolic_II
EDIL3_O43854_OID20081_v1_Cardiometabolic
ENPP7_Q6UWV6_OID20689_v1_Inflammation
KLK8_O60259_OID21517_v1_Oncology
PSAP_P07602_OID30419_v1_Cardiometabolic_II
B2M_P61769_OID30725_v1_Inflammation_II
MYL6B_P14649_OID30936_v1_Neurology_II

APOF_Q13790_OID30701_v1_Inflammation_II
SERPINA6_P08185_OID30743_v1_Inflammation_II
ITIH4_Q14624_OID30762_v1_Inflammation_II
CSNK1D_P48730_OID30492_v1_Inflammation_II
PCSK9_Q8NBP7_OID20235_v1_Cardiometabolic
AIDA_Q96BJ3_OID30956_v1_Neurology_II
SERPINA7_P05543_OID30724_v1_Inflammation_II
EDN1_P05305_OID30224_v1_Cardiometabolic_II
GKN1_Q9NS71_OID20885_v1_Neurology
GC_P02774_OID30780_v1_Inflammation_II
GPC5_P78333_OID20944_v1_Neurology
RTN4R_Q9BZR6_OID21424_v1_Oncology
EPHA1_P21709_OID20677_v1_Inflammation
VWF_P04275_OID20256_v1_Cardiometabolic
SPINK2_P20155_OID30627_v1_Inflammation_II
LPCAT2_Q7L5N7_OID21290_v1_Oncology
FLT4_P35916_OID21491_v1_Oncology
SPOCK1_Q08629_OID20924_v1_Neurology
C1R_P00736_OID30753_v1_Inflammation_II
TNFRSF14_Q92956_OID20783_v1_Inflammation
FASLG_P48023_OID20665_v1_Inflammation
TNF_P01375_OID20848_v1_Neurology
FOLH1_Q04609_OID30430_v1_Inflammation_II
KRT19_P08727_OID20596_v1_Inflammation
WFIKKN1_Q96NZ8_OID20939_v1_Neurology
CCL15_Q16663_OID20328_v1_Cardiometabolic
C9_P02748_OID30698_v1_Inflammation_II
IGSF8_Q969P0_OID20167_v1_Cardiometabolic
CA5A_P35218_OID20075_v1_Cardiometabolic
TNF_P01375_OID21237_v1_Oncology
F7_P08709_OID20372_v1_Cardiometabolic
HPGDS_O60760_OID21266_v1_Oncology
IL1B_P01584_OID20427_v1_Inflammation

noking exposure after validation analyses (UKB-PPP and deCODE overlapping p

proteins)

Supplementary Table S12. Results of GO/KEGG enrichment analysis

GO enrichment analysis

ONTOL	ID	Descript	GeneRat	BgRatio	RichFacto	FoldEnrichme	zScore
BP	GO:000	humoral	43/403	309/188	0.139158	6.49349137	14.4090022
BP	GO:004	positive	45/403	499/188	0.090180	4.20804387	10.7482800
BP	GO:004	defense	37/403	343/188	0.107871	5.03356748	11.1568641
BP	GO:000	positive	38/403	372/188	0.102150	4.76660263	10.8584639
BP	GO:005	positive	39/403	392/188	0.099489	4.64244568	10.7849707
BP	GO:005	leukocyt	39/403	395/188	0.098734	4.60718660	10.7222368
BP	GO:000	positive	25/403	152/188	0.164473	7.67475839	12.2272313
BP	GO:000	leukocyt	40/403	433/188	0.092378	4.31062642	10.3138553
BP	GO:002	positive	35/403	337/188	0.103857	4.84625693	10.5435883
BP	GO:000	complen	17/403	65/1880	0.261538	12.2040465	13.3903497
BP	GO:005	regulatic	37/403	395/188	0.093670	4.37092062	10.0199443
BP	GO:190	positive	31/403	285/188	0.108771	5.07557354	10.2597270
BP	GO:000	regulatic	28/403	236/188	0.118644	5.53623249	10.3777255
BP	GO:009	myeloid	28/403	237/188	0.118143	5.51287286	10.3464136
BP	GO:190	regulatic	36/403	395/188	0.091139	4.25278763	9.66879818
BP	GO:005	positive	29/403	261/188	0.111111	5.18472566	10.0746662
BP	GO:001	positive	40/403	493/188	0.081135	3.78600657	9.27647199
BP	GO:004	positive	40/403	493/188	0.081135	3.78600657	9.27647199
BP	GO:007	leukocyt	33/403	344/188	0.095930	4.47634745	9.62985877
BP	GO:003	regulatic	28/403	248/188	0.112903	5.26835027	10.0132979
BP	GO:000	positive	36/403	414/188	0.086956	4.05761139	9.30946915
BP	GO:004	positive	37/403	437/188	0.084668	3.95083214	9.23632955
BP	GO:003	leukocyt	27/403	234/188	0.115384	5.38413819	9.98664587
BP	GO:007	regulatic	29/403	273/188	0.106227	4.95682563	9.74568167
BP	GO:000	adaptive	35/403	396/188	0.088383	4.12421360	9.29861665
BP	GO:005	positive	32/403	337/188	0.094955	4.43086347	9.40488708
BP	GO:005	regulatic	27/403	243/188	0.111111	5.18472566	9.71634387
BP	GO:006	cell cher	31/403	325/188	0.095384	4.45088757	9.28681167
BP	GO:003	mononu	30/403	308/188	0.097402	4.54505172	9.28308692
BP	GO:005	defense	20/403	131/188	0.152671	7.12405053	10.4088048
BP	GO:004	positive	37/403	464/188	0.079741	3.72093458	8.78234951
BP	GO:000	chemota	37/403	466/188	0.079399	3.70496490	8.75007750
BP	GO:004	T cell pr	25/403	217/188	0.115207	5.37586762	9.59451093
BP	GO:004	taxis	37/403	468/188	0.079059	3.68913172	8.71798247
BP	GO:004	regulatic	23/403	184/188	0.125	5.83281637	9.74883762

BP	GO:190	regulatic	31/403	340/188	0.091176	4.254524886	8.961847110
BP	GO:004	lymphoc	29/403	300/188	0.096666	4.510711331	9.071024393
BP	GO:000	acute-ph	13/403	48/1880	0.270833	12.63776881	11.94686032
BP	GO:190	positive	23/403	195/188	0.117948	5.503785709	9.355499262
BP	GO:190	positive	23/403	195/188	0.117948	5.503785709	9.355499262
BP	GO:000	positive	16/403	96/1880	0.166666	7.777088502	9.851415064
BP	GO:004	wound h	34/403	450/188	0.075555	3.525613454	8.024926734
BP	GO:000	acute inf	17/403	111/188	0.153153	7.146513759	9.611349270
BP	GO:000	leukocyt	35/403	483/188	0.072463	3.381342827	7.846106156
BP	GO:190	regulatic	33/403	436/188	0.075688	3.531797072	7.915429990
BP	GO:004	biologic	23/403	220/188	0.104545	4.878355515	8.562938121
BP	GO:000	regulatic	32/403	414/188	0.077294	3.606765682	7.936785157
BP	GO:003	response	24/403	241/188	0.099585	4.646891055	8.43219541
BP	GO:009	granuloc	19/403	149/188	0.127516	5.950255633	8.977525046
BP	GO:001	antimicr	21/403	185/188	0.113513	5.296827845	8.691387454
BP	GO:005	regulatic	33/403	442/188	0.074660	3.483854125	7.820068103
BP	GO:001	positive	33/403	447/188	0.073825	3.444884840	7.741847595
BP	GO:007	mononu	23/403	230/188	0.1	4.666253101	8.278794097
BP	GO:003	lymphoc	32/403	432/188	0.074074	3.456483779	7.643827325
BP	GO:004	positive	22/403	214/188	0.102803	4.797082627	8.267052397
BP	GO:007	positive	15/403	95/1880	0.157894	7.367768055	9.207829649
BP	GO:001	peptidyl	21/403	198/188	0.106060	4.949056320	8.266713280
BP	GO:000	regulatic	17/403	127/188	0.133858	6.246165569	8.778570452
BP	GO:001	peptidyl	21/403	199/188	0.105527	4.924186690	8.235591969
BP	GO:000	regulatic	22/403	219/188	0.100456	4.687560193	8.122941777
BP	GO:007	cellular	22/403	219/188	0.100456	4.687560193	8.122941777
BP	GO:005	positive	18/403	146/188	0.123287	5.752914782	8.531735919
BP	GO:004	symbion	17/403	131/188	0.129770	6.055442956	8.592535123
BP	GO:000	regulatic	21/403	204/188	0.102941	4.803495840	8.083045324
BP	GO:000	negative	26/403	312/188	0.083333	3.888544251	7.613735203
BP	GO:000	lymphoc	29/403	384/188	0.075520	3.523993227	7.395070648
BP	GO:004	symbion	17/403	136/188	0.125	5.832816377	8.370556880
BP	GO:004	negative	13/403	75/1880	0.173333	8.088172043	9.102089976
BP	GO:007	granuloc	16/403	122/188	0.131147	6.119676198	8.395454917
BP	GO:005	negative	14/403	91/1880	0.153846	7.178850925	8.743599006
BP	GO:003	negative	14/403	93/1880	0.150537	7.024467034	8.618766865
BP	GO:000	cytokine	16/403	127/188	0.125984	5.878744065	8.163752731
BP	GO:005	positive	18/403	163/188	0.110429	5.152917535	7.880358748

BP	GO:000	response	28/403	379/188	0.073878	3.447363769	7.122750455
BP	GO:003	response	27/403	357/188	0.075630	3.529098984	7.139543128
BP	GO:000	regulatic	16/403	130/188	0.123076	5.743080740	8.030581975
BP	GO:007	regulatic	17/403	148/188	0.114864	5.359885319	7.880071335
BP	GO:000	canonica	25/403	318/188	0.078616	3.668437973	7.102031312
BP	GO:002	negative	20/403	209/188	0.095693	4.465313972	7.455059177
BP	GO:005	positive	15/403	116/188	0.129310	6.033947976	8.048034264
BP	GO:007	negative	14/403	100/188	0.14	6.532754342	8.209329030
BP	GO:000	producti	21/403	231/188	0.090909	4.242048274	7.336989164
BP	GO:190	negative	14/403	101/188	0.138613	6.468073606	8.154041606
BP	GO:004	tissue re	19/403	192/188	0.098958	4.617646298	7.456133235
BP	GO:004	regulatic	24/403	300/188	0.08	3.733002481	7.061569532
BP	GO:006	antimicr	16/403	136/188	0.117647	5.489709531	7.776287381
BP	GO:003	T cell di	25/403	324/188	0.077160	3.600503936	6.987348719
BP	GO:003	zymoger	10/403	46/1880	0.217391	10.14402848	9.188757186
BP	GO:003	negative	30/403	448/188	0.066964	3.124723059	6.735744659
BP	GO:005	regulatic	17/403	156/188	0.108974	5.085019405	7.581825415
BP	GO:005	biologica	18/403	176/188	0.102272	4.772304308	7.440693466
BP	GO:005	regulatic	20/403	217/188	0.092165	4.300694102	7.237089498
BP	GO:000	complem	7/403	18/1880	0.388888	18.14653984	10.77033368
BP	GO:007	positive	19/403	198/188	0.095959	4.477717622	7.280041642
BP	GO:001	lipid loc	31/403	482/188	0.064315	3.001117140	6.586320666
BP	GO:190	regulatic	18/403	181/188	0.099447	4.640472697	7.282922951
BP	GO:000	regulatic	15/403	126/188	0.119047	5.555063216	7.591856687
BP	GO:000	negative	19/403	204/188	0.093137	4.346020045	7.110835091
BP	GO:005	negative	20/403	227/188	0.088105	4.111236212	6.978973762
BP	GO:000	myeloid	21/403	250/188	0.084	3.919652605	6.877266218
BP	GO:007	ERK1 ar	24/403	319/188	0.075235	3.510660640	6.692787137
BP	GO:007	regulatic	23/403	296/188	0.077702	3.625804774	6.738479984
BP	GO:000	myeloid	21/403	251/188	0.083665	3.904036459	6.854334272
BP	GO:001	negative	10/403	52/1880	0.192307	8.973563657	8.520476675
BP	GO:007	positive	17/403	168/188	0.101190	4.721803733	7.170760816
BP	GO:000	complem	9/403	40/1880	0.225	10.49906947	8.899834008
BP	GO:003	positive	16/403	150/188	0.106666	4.977336641	7.237448480
BP	GO:000	humoral	10/403	54/1880	0.185185	8.641209447	8.321313769
BP	GO:005	negative	17/403	173/188	0.098265	4.585335417	7.010810626
BP	GO:015	regulatic	9/403	42/1880	0.214285	9.999113789	8.640092267
BP	GO:190	negative	16/403	155/188	0.103225	4.816777395	7.061035478

BP	GO:005(	negative	15/403	136/188(	0.110294	5.14660268	7.18201788
BP	GO:004	positive	15/403	137/188(	0.109489	5.10903624	7.14326053
BP	GO:000	regulatic	18/403	197/188(	0.091370	4.26358151	6.81432570
BP	GO:000	cell killi	21/403	263/188(	0.079847	3.72590551	6.58802920
BP	GO:003	interleuk	17/403	177/188(	0.096045	4.48171201	6.88718099
BP	GO:003	regulatic	17/403	177/188(	0.096045	4.48171201	6.88718099
BP	GO:005	regulatic	12/403	87/1880	0.137931	6.43621117	7.52092668
BP	GO:003(	extracell	24/403	339/188(	0.070796	3.30354201	6.33365937
BP	GO:004	positive	14/403	122/188(	0.114754	5.35471667	7.14104292
BP	GO:004	extracell	24/403	340/188(	0.070588	3.29382571	6.31641056
BP	GO:003	regulatic	12/403	88/1880	0.136363	6.36307241	7.46245985
BP	GO:004	external	24/403	341/188(	0.070381	3.28416640	6.29922563
BP	GO:004	positive	13/403	105/188(	0.123809	5.77726574	7.26437705
BP	GO:004(	positive	6/403	15/1880	0.4	18.6650124	10.1284110
BP	GO:007	positive	6/403	15/1880	0.4	18.6650124	10.1284110
BP	GO:000	positive	21/403	270/188(	0.077777	3.62930796	6.43979697
BP	GO:004	regulatic	11/403	73/1880	0.150684	7.03134029	7.64061503
BP	GO:007	leukocyt	14/403	124/188(	0.112903	5.26835027	7.05693253
BP	GO:005(	defense	13/403	108/188(	0.120370	5.61678614	7.12050511
BP	GO:004	negative	16/403	165/188(	0.096969	4.52485149	6.72984363
BP	GO:000	heteroph	9/403	47/1880	0.191489	8.93537827	8.06061964
BP	GO:005(	positive	15/403	147/188(	0.102040	4.76148275	6.77532807
BP	GO:000	positive	14/403	130/188(	0.107692	5.02519564	6.81511983
BP	GO:000(	lipid trai	28/403	460/188(	0.060869	2.84032797	5.91371535
BP	GO:003	positive	11/403	78/1880	0.141025	6.58061334	7.30870210
BP	GO:004	regulatic	17/403	192/188(	0.088541	4.13157826	6.45432495
BP	GO:000	regulatic	20/403	260/188(	0.076923	3.58942546	6.22188243
BP	GO:009	extrinsic	19/403	237/188(	0.080168	3.74087801	6.28385923
BP	GO:005	positive	6/403	17/1880	0.352941	16.4691285	9.44267768
BP	GO:005	regulatic	23/403	334/188(	0.068862	3.21328806	6.03964068
BP	GO:001(	negative	21/403	285/188(	0.073684	3.43829175	6.13808277
BP	GO:000(	prostagl	9/403	50/1880	0.18	8.39925558	7.75281869
BP	GO:003	non-can(	14/403	133/188(	0.105263	4.91184537	6.69972862
BP	GO:002	extracell	10/403	65/1880	0.153846	7.17885092	7.38456187
BP	GO:003	positive	10/403	65/1880	0.153846	7.17885092	7.38456187
BP	GO:004	regulatic	24/403	363/188(	0.066115	3.08512601	5.93644901
BP	GO:000(	prostano	9/403	51/1880	0.176470	8.23456429	7.65588928
BP	GO:007(	response	13/403	116/188(	0.112068	5.22942157	6.76179609

BP	GO:003	interleuk	10/403	66/1880	0.151515	7.07008045	7.31035287
BP	GO:003	regulatic	10/403	66/1880	0.151515	7.07008045	7.31035287
BP	GO:190	positive	10/403	66/1880	0.151515	7.07008045	7.31035287
BP	GO:004	regulatic	18/403	220/188	0.081818	3.81784344	6.22145622
BP	GO:000	positive	14/403	136/188	0.102941	4.80349584	6.58774838
BP	GO:015	positive	6/403	18/1880	0.333333	15.5541770	9.14198108
BP	GO:003	tumor ne	13/403	118/188	0.110169	5.14078731	6.67727505
BP	GO:005	negative	12/403	100/188	0.12	5.59950372	6.82460074
BP	GO:000	regulatic	17/403	200/188	0.085	3.96631513	6.24112067
BP	GO:000	positive	7/403	28/1880	0.25	11.6656327	8.35790959
BP	GO:005	regulatic	7/403	28/1880	0.25	11.6656327	8.35790959
BP	GO:190	regulatic	24/403	372/188	0.064516	3.01048587	5.79588307
BP	GO:004	regulatic	18/403	225/188	0.08	3.73300248	6.10314321
BP	GO:003	positive	12/403	102/188	0.117647	5.48970953	6.72833837
BP	GO:000	T cell ac	14/403	140/188	0.1	4.66625310	6.44343712
BP	GO:006	regulatic	14/403	140/188	0.1	4.66625310	6.44343712
BP	GO:005	regulatic	27/403	454/188	0.059471	2.77508444	5.66581262
BP	GO:000	monocyt	9/403	54/1880	0.166666	7.77708850	7.38028191
BP	GO:001	fatty acid	13/403	121/188	0.107438	5.01332977	6.55401958
BP	GO:000	phagocy	7/403	29/1880	0.241379	11.2633695	8.18526150
BP	GO:003	chemoki	12/403	105/188	0.114285	5.33286068	6.58860851
BP	GO:003	regulatic	12/403	105/188	0.114285	5.33286068	6.58860851
BP	GO:200	positive	8/403	42/1880	0.190476	8.88810114	7.57340370
BP	GO:003	type II ir	13/403	124/188	0.104838	4.89203954	6.43477191
BP	GO:003	regulatic	13/403	124/188	0.104838	4.89203954	6.43477191
BP	GO:000	regulatic	12/403	106/188	0.113207	5.28255068	6.54321791
BP	GO:199	neutroph	12/403	107/188	0.112149	5.23318104	6.49839768
BP	GO:190	regulatic	16/403	189/188	0.084656	3.95026717	6.03245645
BP	GO:004	regulatic	6/403	21/1880	0.285714	13.3321517	8.36757469
BP	GO:004	bone ren	11/403	91/1880	0.120879	5.64052572	6.56673815
BP	GO:190	positive	12/403	112/188	0.107142	4.99955689	6.28241351
BP	GO:003	osteocla	12/403	113/188	0.106194	4.95531302	6.24075777
BP	GO:007	T cell ap	9/403	61/1880	0.147540	6.88463572	6.81237418
BP	GO:005	negative	15/403	175/188	0.085714	3.99964551	5.89966922
BP	GO:000	leukocyt	21/403	319/188	0.065830	3.07182806	5.52297031
BP	GO:000	leukocyt	12/403	114/188	0.105263	4.91184537	6.19958997
BP	GO:001	viral life	20/403	295/188	0.067796	3.16356142	5.54271003
BP	GO:003	regulatic	25/403	430/188	0.058139	2.71293784	5.31749745

BP	GO:0019	antibacte	10/403	79/18805	0.126582	5.906649495	6.467273130
BP	GO:0002	cell activ	21/403	323/1880	0.065015	3.033786846	5.456037461
BP	GO:0002	regulatic	8/403	48/18805	0.166666	7.777088502	6.957083531
BP	GO:0031	T cell co	8/403	48/18805	0.166666	7.777088502	6.957083531
BP	GO:0002	T cell ac	5/403	14/18805	0.357142	16.66518964	8.677005239
BP	GO:0032	positive	5/403	14/18805	0.357142	16.66518964	8.677005239
BP	GO:0002	antigen p	7/403	35/18805	0.2	9.332506203	7.301677760
BP	GO:0002	T cell m	13/403	137/1880	0.094890	4.427831410	5.959035499
BP	GO:0008	cell reco	14/403	158/1880	0.088607	4.134654647	5.855431297
BP	GO:0019	antigen p	12/403	117/1880	0.102564	4.785900617	6.078912028
BP	GO:0031	regenera	16/403	203/1880	0.078817	3.677834957	5.676715597
BP	GO:0002	positive	9/403	64/18805	0.14063	6.561918424	6.595737341
BP	GO:0016	protein p	17/403	227/1880	0.074889	3.494550781	5.595654992
BP	GO:0006	membran	8/403	49/18805	0.163265	7.618372410	6.864742639
BP	GO:0031	lymphoc	8/403	49/18805	0.163265	7.618372410	6.864742639
BP	GO:2000	positive	6/403	24/18805	0.25	11.66563275	7.737096638
BP	GO:0098	cell-cell	19/403	280/1880	0.067857	3.166386033	5.404815652
BP	GO:0002	lymphoc	17/403	231/1880	0.073593	3.434039079	5.508406056
BP	GO:0052	regulatic	9/403	66/18805	0.136363	6.363072411	6.458885030
BP	GO:0002	positive	13/403	141/1880	0.092198	4.302219171	5.824494225
BP	GO:0070	chemoki	10/403	83/18805	0.120481	5.621991688	6.245068654
BP	GO:0032	regulatic	5/403	15/18805	0.333333	15.55417700	8.344782527
BP	GO:0002	antigen p	7/403	37/18805	0.189189	8.828046408	7.053268026
BP	GO:0072	eosinoph	6/403	25/18805	0.24	11.19900744	7.551361307
BP	GO:1903	positive	6/403	25/18805	0.24	11.19900744	7.551361307
BP	GO:0046	regulatic	8/403	51/18805	0.156862	7.319612708	6.687652941
BP	GO:0032	interleuk	9/403	67/18805	0.134328	6.268101181	6.392563088
BP	GO:0032	regulatic	9/403	67/18805	0.134328	6.268101181	6.392563088
BP	GO:0046	regulatic	12/403	123/1880	0.097560	4.552442050	5.849422686
BP	GO:0044	multi-org	16/403	211/1880	0.075829	3.538390977	5.487296923
BP	GO:0002	positive	10/403	85/18805	0.117647	5.489709531	6.139314990
BP	GO:0070	lymphoc	10/403	85/18805	0.117647	5.489709531	6.139314990
BP	GO:0007	digestion	13/403	144/1880	0.090277	4.212589605	5.726828408
BP	GO:0008	extrinsic	10/403	86/18805	0.116279	5.425875699	6.087686050
BP	GO:0007	female p	15/403	190/1880	0.078947	3.683884027	5.502425322
BP	GO:0032	interleuk	11/403	105/1880	0.104761	4.888455630	5.912839971
BP	GO:0032	regulatic	11/403	105/1880	0.104761	4.888455630	5.912839971
BP	GO:0045	positive	15/403	192/1880	0.07813	3.645510235	5.452516682

BP	GO:200(	regulatic	9/403	70/1880!	0.128571	5.999468273(	6.201419623(
BP	GO:003	positive	10/403	88/1880!	0.113636	5.302560342	5.986807816
BP	GO:000	blood co	17/403	241/188(	0.070539	3.291547831	5.298425097
BP	GO:190	regulatic	11/403	107/188(	0.102803	4.797082627	5.828938727(
BP	GO:004	fibrinoly	6/403	27/1880!	0.222222	10.36945133	7.209688577
BP	GO:000	T cell cy	7/403	40/1880!	0.175	8.165942928(	6.713889472
BP	GO:000	regulatic	7/403	40/1880!	0.175	8.165942928(	6.713889472
BP	GO:004	vascular	11/403	108/188(	0.101851	4.752665196	5.787764668
BP	GO:190	positive	15/403	195/188(	0.076923(	3.589425462	5.378890020
BP	GO:004	multi-mu	16/403	219/188(	0.073059	3.409134686	5.306831553
BP	GO:007	tumor ne	15/403	196/188(	0.076530(	3.571112067(	5.354669316
BP	GO:190	regulatic	15/403	196/188(	0.076530(	3.571112067(	5.354669316
BP	GO:005(	positive	9/403	72/1880!	0.125	5.832816377	6.080061990
BP	GO:005	modulati	6/403	28/1880!	0.214285	9.999113789	7.051975372
BP	GO:005(	coagulat	17/403	246/188(	0.069105(	3.224646452	5.197522765
BP	GO:003	positive	9/403	73/1880!	0.123287(	5.752914782	6.021081619
BP	GO:000	tissue hc	18/403	272/188(	0.066176	3.087961611	5.133094148
BP	GO:006(	anatomi(	18/403	272/188(	0.066176	3.087961611	5.133094148
BP	GO:003	interleuk	12/403	131/188(	0.091603(	4.274430322	5.565418855
BP	GO:003	regulatic	12/403	131/188(	0.091603(	4.274430322	5.565418855
BP	GO:004	response	26/403	494/188(	0.052631	2.455922685	4.852783175(
BP	GO:007	cellular	18/403	273/188(	0.065934(	3.076650396	5.114800492
BP	GO:003	positive	16/403	223/188(	0.071748	3.347984288	5.219711759
BP	GO:000	hemosta	17/403	248/188(	0.068548	3.198641239(	5.157882653
BP	GO:199(	response	10/403	92/1880!	0.108695(	5.072014241(	5.793975096(
BP	GO:199(	cellular	10/403	92/1880!	0.108695(	5.072014241(	5.793975096(
BP	GO:001(	negative	7/403	42/1880!	0.166666(	7.777088502	6.506715143(
BP	GO:003	positive	7/403	42/1880!	0.166666(	7.777088502	6.506715143(
BP	GO:004	regulatic	7/403	42/1880!	0.166666(	7.777088502	6.506715143(
BP	GO:003	positive	9/403	74/1880!	0.121621	5.675172691	5.963183401
BP	GO:005	regulatic	5/403	18/1880!	0.277777	12.96181417	7.513628484
BP	GO:004(	alpha-be	15/403	201/188(	0.074626	3.482278434	5.235889694
BP	GO:190	positive	11/403	113/188(	0.097345	4.542370276(	5.589209927
BP	GO:000	negative	12/403	134/188(	0.089552	4.178734120	5.464720377
BP	GO:190	positive	11/403	114/188(	0.096491	4.502524922	5.550888736
BP	GO:005(	regulatic	9/403	76/1880!	0.118421(	5.525826041	5.850493342(
BP	GO:007	T cell mi	9/403	76/1880!	0.118421(	5.525826041	5.850493342(
BP	GO:200(	negative	8/403	59/1880!	0.135593	6.327122849	6.064703075(

BP	GO:0002	toleranc	6/403	30/18805	0.2	9.332506203	6.759139834
BP	GO:0050	regulatio	22/403	388/1880	0.056701	2.645813614	4.847660539
BP	GO:2000	regulatio	10/403	95/18805	0.105263	4.911845370	5.656551160
BP	GO:0000	receptor-	18/403	280/1880	0.064285	2.999734136	4.989043594
BP	GO:0001	negative	22/403	389/1880	0.056555	2.639012036	4.833975241
BP	GO:0031	positive	4/403	10/18805	0.4	18.66501240	8.268712899
BP	GO:0045	positive	13/403	159/1880	0.081761	3.815175491	5.275411313
BP	GO:0002	regulatio	5/403	19/18805	0.263157	12.27961342	7.279457057
BP	GO:0032	prostagl	5/403	19/18805	0.263157	12.27961342	7.279457057
BP	GO:0016	viral pro	23/403	421/1880	0.054631	2.549259414	4.757626201
BP	GO:0061	negative	9/403	78/18805	0.115384	5.384138194	5.741727305
BP	GO:0019	antigen p	6/403	31/18805	0.193548	9.031457616	6.622804620
BP	GO:0001	temperat	14/403	184/1880	0.076086	3.550409968	5.144729434
BP	GO:0031	neuron p	8/403	61/18805	0.131147	6.119676198	5.926815461
BP	GO:0048	homeost	20/403	339/1880	0.058997	2.752951682	4.819794371
BP	GO:0015	monocar	14/403	186/1880	0.075268	3.512233517	5.095460424
BP	GO:0031	killing o	11/403	119/1880	0.092436	4.313343203	5.365705876
BP	GO:0141	disruptio	11/403	119/1880	0.092436	4.313343203	5.365705876
BP	GO:0030	myeloid	24/403	455/1880	0.052747	2.461320317	4.669584273
BP	GO:0032	positive	7/403	46/18805	0.152173	7.100819937	6.130662558
BP	GO:0046	alpha-be	7/403	46/18805	0.152173	7.100819937	6.130662558
BP	GO:0032	positive	5/403	20/18805	0.25	11.66563275	7.062218577
BP	GO:0006	aminogly	6/403	32/18805	0.1875	8.749224565	6.492493548
BP	GO:0043	regulatio	8/403	63/18805	0.126984	5.925400764	5.794940918
BP	GO:0002	lymphoc	9/403	81/18805	0.111111	5.184725668	5.585413331
BP	GO:0006	phagocy	16/403	238/1880	0.067226	3.136976875	4.909786003
BP	GO:0001	fever ge	4/403	11/18805	0.363636	16.96819309	7.839486890
BP	GO:0033	membra	8/403	64/18805	0.125	5.832816377	5.731113801
BP	GO:0036	response	8/403	64/18805	0.125	5.832816377	5.731113801
BP	GO:0043	endothel	18/403	293/1880	0.061433	2.866640130	4.765548227
BP	GO:0032	tumor ne	14/403	191/1880	0.073298	3.420290231	4.975186987
BP	GO:0032	regulatio	14/403	191/1880	0.073298	3.420290231	4.975186987
BP	GO:0032	interleuk	5/403	21/18805	0.238095	11.11012643	6.859892743
BP	GO:0032	regulatio	5/403	21/18805	0.238095	11.11012643	6.859892743
BP	GO:0048	eosinoph	5/403	21/18805	0.238095	11.11012643	6.859892743
BP	GO:0141	disruptio	11/403	123/1880	0.089430	4.173071879	5.224754795
BP	GO:0006	icosanoi	11/403	124/1880	0.088709	4.139418074	5.190450668
BP	GO:0045	negative	6/403	34/18805	0.176470	8.234564297	6.248176162

BP	GO:200(	regulatic	6/403	34/1880!	0.17647(	8.234564297	6.24817616(
BP	GO:003(	neutroph	9/403	84/1880!	0.107142	4.999556894	5.43665947
BP	GO:004:	T-helper	7/403	49/1880!	0.142857	6.66607585	5.876996659
BP	GO:190	regulatic	17/403	270/188(	0.062962	2.938011212	4.74664774
BP	GO:000	endothel	12/403	147/188(	0.081632	3.80918620	5.060014732
BP	GO:005	positive	8/403	66/1880!	0.121212	5.656064365	5.60741719(
BP	GO:000	regulatic	22/403	410/188(	0.053658	2.503843127	4.556062771
BP	GO:003(	regulatic	5/403	22/1880!	0.227272	10.6051206	6.67078307
BP	GO:003:	regulatic	5/403	22/1880!	0.227272	10.6051206	6.67078307
BP	GO:007	cellular r	16/403	246/188(	0.065040	3.03496136	4.75435457
BP	GO:003(	negative	7/403	50/1880!	0.14	6.532754342	5.797129296
BP	GO:200(	regulatic	7/403	50/1880!	0.14	6.532754342	5.797129296
BP	GO:200(	positive	6/403	35/1880!	0.171428	7.99929103	6.13339689(
BP	GO:004!	bone res	8/403	67/1880!	0.119402	5.571645494	5.54745082
BP	GO:005(	regulatic	8/403	67/1880!	0.119402	5.571645494	5.54745082
BP	GO:014(	interleuk	4/403	12/1880!	0.333333	15.5541770	7.46320463
BP	GO:190(	negative	7/403	51/1880!	0.137254	6.40466112(	5.71941660
BP	GO:015(	neuroinf	9/403	87/1880!	0.103448	4.82715838	5.29482329
BP	GO:001(	regulatic	15/403	224/188(	0.066964	3.12472305	4.73409457
BP	GO:190:	positive	6/403	36/1880!	0.166666	7.77708850	6.02308471
BP	GO:003(	regulatic	8/403	69/1880!	0.115942	5.41014852	5.43105815
BP	GO:007	cellular r	9/403	88/1880!	0.102272	4.77230430	5.24898179
BP	GO:004:	negative	18/403	306/188(	0.058823	2.74485476	4.55397877
BP	GO:004(	negative	8/403	70/1880!	0.114285	5.33286068	5.37454901
BP	GO:190:	negative	12/403	154/188(	0.077922	3.63604137	4.86078770
BP	GO:003	negative	18/403	308/188(	0.058441	2.72703103	4.52241052(
BP	GO:000:	regulatic	4/403	13/1880!	0.307692	14.3577018	7.12954888
BP	GO:003	regulatic	4/403	13/1880!	0.307692	14.3577018	7.12954888
BP	GO:003:	positive	10/403	110/188(	0.090909	4.24204827	5.04659224
BP	GO:007:	lymphoc	10/403	110/188(	0.090909	4.24204827	5.04659224
BP	GO:004:	positive	8/403	71/1880!	0.112676	5.25774997	5.31911282
BP	GO:004	antigen p	8/403	71/1880!	0.112676	5.25774997	5.31911282
BP	GO:190(	regulatic	8/403	71/1880!	0.112676	5.25774997	5.31911282
BP	GO:003:	interleuk	10/403	111/188(	0.090090	4.20383162	5.00985533
BP	GO:003:	regulatic	10/403	111/188(	0.090090	4.20383162	5.00985533
BP	GO:004!	positive	11/403	133/188(	0.082706	3.85930707	4.89707021
BP	GO:003	ERBB si	13/403	180/188(	0.072222	3.37007168	4.72818787
BP	GO:001	immuno	14/403	205/188(	0.068292	3.18670943	4.65861336

BP	GO:003(	macroph	7/403	54/1880!	0.129629(	6.04884661З	5.498218198
BP	GO:005(	negative	7/403	54/1880!	0.129629(	6.04884661З	5.498218198
BP	GO:007:	regulatic	7/403	54/1880!	0.129629(	6.04884661З	5.498218198
BP	GO:002:	digestive	10/403	112/188(	0.089285'	4.16629741:	4.97354836:
BP	GO:003:	regulatic	12/403	157/188(	0.076433:	3.56656288(	4.77893073'
BP	GO:190:	carbohy(	16/403	258/188(	0.062015!	2.89380037:	4.53265177'
BP	GO:005:	positive	17/403	285/188(	0.059649:	2.78337904:	4.48942508'
BP	GO:007:	cellular ı	15/403	232/188(	0.064655:	3.01697398:	4.57455013'
BP	GO:001!	B cell m	14/403	208/188(	0.067307(	3.14074728(	4.59431484'
BP	GO:000!	detectior	6/403	39/1880!	0.153846:	7.17885092'	5.71609130:
BP	GO:001(	cell grov	24/403	492/188(	0.048780'	2.27622102:	4.24496222!
BP	GO:004!	positive	14/403	210/188(	0.066666(	3.11083540:	4.55209226(
BP	GO:004(	positive	7/403	56/1880!	0.125	5.83281637:	5.35982221'
BP	GO:005:	positive	16/403	263/188(	0.060836!	2.83878515!	4.44401694(
BP	GO:004(	positive	8/403	75/1880!	0.106666(	4.97733664:	5.10739239!
BP	GO:009(	detectior	5/403	26/1880!	0.192307(	8.97356365:	6.02071458(
BP	GO:003(	vascular	9/403	95/1880!	0.094736(	4.42066083:	4.94629546'
BP	GO:000'	axon gui	15/403	238/188(	0.063025:	2.94091582'	4.45932824(
BP	GO:000:	negative	7/403	57/1880!	0.122807(	5.73048626:	5.29310942!
BP	GO:009'	neuron p	15/403	239/188(	0.062761!	2.92861073:	4.44047559'
BP	GO:000:	antigen ı	6/403	41/1880!	0.146341'	6.82866307'	5.52895810(
BP	GO:005(	negative	14/403	214/188(	0.065420!	3.05268894(	4.46913821'
BP	GO:000:	T cell to	4/403	15/1880!	0.266666(	12.4433416(	6.56115402(
BP	GO:004(	negative	4/403	15/1880!	0.266666(	12.4433416(	6.56115402(
BP	GO:001!	lipid stoı	9/403	97/1880!	0.092783!	4.32951318:	4.86517041:
BP	GO:017(	nutrient	9/403	97/1880!	0.092783!	4.32951318:	4.86517041:
BP	GO:005:	positive	13/403	190/188(	0.068421(	3.19269949(	4.49541227!
BP	GO:003:	plasminc	5/403	27/1880!	0.185185:	8.64120944(	5.87982566(
BP	GO:005(	regulatic	5/403	27/1880!	0.185185:	8.64120944(	5.87982566(
BP	GO:007(	regulatic	6/403	42/1880!	0.142857:	6.66607585(	5.44002658'
BP	GO:190:	negative	10/403	120/188(	0.083333:	3.88854425'	4.69751556(
BP	GO:003:	regulatic	8/403	78/1880!	0.102564:	4.78590061:	4.95823990'
BP	GO:003(	cellular ı	7/403	60/1880!	0.116666(	5.44396195(	5.10209294:
BP	GO:007(	regulatic	7/403	60/1880!	0.116666(	5.44396195(	5.10209294:
BP	GO:000:	positive	5/403	28/1880!	0.178571'	8.33259482!	5.74604114:
BP	GO:005(	positive	5/403	28/1880!	0.178571'	8.33259482!	5.74604114:
BP	GO:190!	positive	5/403	28/1880!	0.178571'	8.33259482!	5.74604114:
BP	GO:004!	regulatic	6/403	43/1880!	0.139534(	6.51105083(	5.35394876:

BP	GO:004(	regulatic	6/403	43/1880!	0.139534{	6.511050839(	5.353948761
BP	GO:004(	carboxyl	19/403	360/188(	0.052777	2.462744692!	4.146919288
BP	GO:007(	negative	4/403	16/1880!	0.25	11.66563275	6.315967912(
BP	GO:003	negative	8/403	80/1880!	0.1	4.666253101	4.862970722(
BP	GO:001!	organic a	19/403	361/188(	0.052631!	2.455922685	4.133419540
BP	GO:003	cell-subs	19/403	361/188(	0.052631!	2.455922685	4.133419540
BP	GO:001	glial cell	7/403	61/1880!	0.114754(	5.354716674	5.041256733{
BP	GO:004(	icosanoi	7/403	61/1880!	0.114754(	5.354716674	5.041256733{
BP	GO:001	astrocyte	6/403	44/1880!	0.136363(	6.363072411	5.270564572
BP	GO:005	negative	11/403	147/188(	0.074829!	3.491754021	4.488243618
BP	GO:000!	response	22/403	453/188(	0.048565	2.266171484	4.036872754(
BP	GO:000!	response	14/403	223/188(	0.062780	2.929486252	4.289365193
BP	GO:004!	endothel	10/403	124/188(	0.080645	3.763107340	4.568290046{
BP	GO:004(	regulatic	8/403	81/1880!	0.098765	4.608645038	4.816510257
BP	GO:003	positive	7/403	62/1880!	0.112903	5.268350276	4.981744790(
BP	GO:007(	regulatic	7/403	62/1880!	0.112903	5.268350276	4.981744790(
BP	GO:007	cellular r	13/403	198/188(	0.065656!	3.063701531	4.320026729
BP	GO:190	negative	8/403	82/1880!	0.097560!	4.552442050	4.770801431
BP	GO:005(	epithelia	23/403	488/188(	0.047131	2.199258634(	3.972287953!
BP	GO:003(	lung dev	13/403	199/188(	0.065326(	3.048306046	4.298728413!
BP	GO:003(	negative	12/403	174/188(	0.068965!	3.218105587	4.349946225
BP	GO:004	regulatic	20/403	397/188(	0.050377{	2.350757230(	4.025449498
BP	GO:000{	opsoniza	4/403	17/1880!	0.235294	10.97941906	6.091644860
BP	GO:190	regulatic	4/403	17/1880!	0.235294	10.97941906	6.091644860
BP	GO:190	regulatic	4/403	17/1880!	0.235294	10.97941906	6.091644860
BP	GO:000	axonoge	22/403	459/188(	0.047930	2.236548327(	3.969098472
BP	GO:001!	prostagla	5/403	30/1880!	0.166666(	7.777088502{	5.497420313
BP	GO:004!	positive	5/403	30/1880!	0.166666(	7.777088502{	5.497420313
BP	GO:190	negative	10/403	127/188(	0.078740	3.674215040	4.474846405
BP	GO:003(	respirato	13/403	203/188(	0.064039	2.988240902!	4.214852044
BP	GO:005(	regulatic	19/403	372/188(	0.051075	2.383301315	3.987818460
BP	GO:004{	platelet-(	9/403	106/188(	0.084905(	3.961913010!	4.525443764(
BP	GO:001!	regulatic	18/403	343/188(	0.052478	2.448762560(	4.007283167
BP	GO:000(	peptide r	7/403	65/1880!	0.107692	5.025195648(	4.81065277
BP	GO:004!	regulatic	7/403	65/1880!	0.107692	5.025195648(	4.81065277
BP	GO:000	regulatic	5/403	31/1880!	0.161290	7.526214680	5.381569175
BP	GO:005(	positive	5/403	31/1880!	0.161290	7.526214680	5.381569175
BP	GO:200(	regulatic	8/403	85/1880!	0.094117(	4.391767625	4.637968153{

BP	GO:0002	regulatic	6/403	47/18805	0.127659	5.95691885	5.03515221
BP	GO:0050	regulatic	17/403	315/1880	0.053968	2.51829532	4.02150124
BP	GO:0045	positive	9/403	107/1880	0.084112	3.92488578	4.49002081
BP	GO:0042	B cell ac	16/403	287/1880	0.055749	2.60139545	4.04564370
BP	GO:0010	regulatic	4/403	18/18805	0.222222	10.3694513	5.88527588
BP	GO:0031	heat gen	4/403	18/18805	0.222222	10.3694513	5.88527588
BP	GO:0001	endothel	7/403	66/18805	0.106060	4.94905632	4.75594935
BP	GO:0031	animal o	7/403	66/18805	0.106060	4.94905632	4.75594935
BP	GO:0090	positive	7/403	66/18805	0.106060	4.94905632	4.75594935
BP	GO:1903	regulatic	7/403	66/18805	0.106060	4.94905632	4.75594935
BP	GO:0002	positive	11/403	155/1880	0.070967	3.31153445	4.27633711
BP	GO:0045	negative	14/403	233/1880	0.060085	2.80375722	4.09988654
BP	GO:0032	icosanoi	6/403	48/18805	0.125	5.83281637	4.96117281
BP	GO:1902	regulatic	6/403	48/18805	0.125	5.83281637	4.96117281
BP	GO:2000	positive	6/403	48/18805	0.125	5.83281637	4.96117281
BP	GO:0001	prostagl	5/403	32/18805	0.15625	7.29102047	5.27077380
BP	GO:0001	lymph ve	5/403	32/18805	0.15625	7.29102047	5.27077380
BP	GO:0010	positive	5/403	32/18805	0.15625	7.29102047	5.27077380
BP	GO:0046	prostano	5/403	32/18805	0.15625	7.29102047	5.27077380
BP	GO:0043	regulatic	9/403	109/1880	0.082568	3.85286953	4.42044449
BP	GO:0002	negative	7/403	67/18805	0.104477	4.87518980	4.70233856
BP	GO:0050	defense	7/403	67/18805	0.104477	4.87518980	4.70233856
BP	GO:0080	regulatic	7/403	67/18805	0.104477	4.87518980	4.70233856
BP	GO:0002	positive	10/403	133/1880	0.075187	3.50846097	4.29618408
BP	GO:0006	fat-solub	6/403	49/18805	0.122448	5.71377930	4.88925067
BP	GO:0043	T cell hc	6/403	49/18805	0.122448	5.71377930	4.88925067
BP	GO:0050	regulatic	17/403	321/1880	0.052959	2.47122438	3.93440064
BP	GO:0031	regulatic	9/403	110/1880	0.081818	3.81784344	4.38627240
BP	GO:0007	epiderma	11/403	158/1880	0.069620	3.24865722	4.20041738
BP	GO:0002	macroph	4/403	19/18805	0.210526	9.82369074	5.69449283
BP	GO:0010	positive	4/403	19/18805	0.210526	9.82369074	5.69449283
BP	GO:0032	response	4/403	19/18805	0.210526	9.82369074	5.69449283
BP	GO:0035	CD4-pos	4/403	19/18805	0.210526	9.82369074	5.69449283
BP	GO:0045	positive	4/403	19/18805	0.210526	9.82369074	5.69449283
BP	GO:0140	positive	4/403	19/18805	0.210526	9.82369074	5.69449283
BP	GO:0035	CD4-pos	10/403	134/1880	0.074626	3.48227843	4.26740866
BP	GO:0044	cellular	17/403	323/1880	0.052631	2.45592268	3.90580198
BP	GO:0033	T cell di	8/403	89/18805	0.089887	4.19438481	4.47013715

BP	GO:006(	developr	14/403	238/188(	0.058823!	2.744854765	4.008870478
BP	GO:001!	antigen p	6/403	50/1880!	0.12	5.599503722(	4.819284596
BP	GO:004:	amide m	22/403	480/188(	0.045833:	2.138699338	3.739838313(
BP	GO:004:	macroph	9/403	113/188(	0.079646(	3.716484771	4.286114230!
BP	GO:003(	hyaluron	5/403	34/1880!	0.147058	6.862136914:	5.062870771
BP	GO:190	negative	5/403	34/1880!	0.147058	6.862136914:	5.062870771
BP	GO:004:	amide tr	17/403	328/188(	0.051829	2.418484839	3.835226539
BP	GO:000	lymphan	4/403	20/1880!	0.2	9.332506203	5.517345427(
BP	GO:000:	regulatic	4/403	20/1880!	0.2	9.332506203	5.517345427(
BP	GO:004!	regulatic	4/403	20/1880!	0.2	9.332506203	5.517345427(
BP	GO:006(	mammar	4/403	20/1880!	0.2	9.332506203	5.517345427(
BP	GO:006	mammar	4/403	20/1880!	0.2	9.332506203	5.517345427(
BP	GO:007	monocyt	4/403	20/1880!	0.2	9.332506203	5.517345427(
BP	GO:007	regulatic	4/403	20/1880!	0.2	9.332506203	5.517345427(
BP	GO:007	response	12/403	188/188(	0.063829	2.978459426(	4.034561637!
BP	GO:007	cellular r	12/403	188/188(	0.063829	2.978459426(	4.034561637!
BP	GO:004(	alpha-be	10/403	138/188(	0.072463	3.381342827	4.154983784(
BP	GO:003:	unsatura	9/403	115/188(	0.078260	3.651850253!	4.221223983!
BP	GO:190:	regulatic	9/403	115/188(	0.078260	3.651850253!	4.221223983!
BP	GO:004(	regulatic	10/403	139/188(	0.071942	3.357016619!	4.127523412!
BP	GO:005(	negative	10/403	139/188(	0.071942	3.357016619!	4.127523412!
BP	GO:000:	negative	5/403	35/1880!	0.142857	6.666075859(	4.965116027(
BP	GO:003:	interleuk	5/403	35/1880!	0.142857	6.666075859(	4.965116027(
BP	GO:003:	regulatic	5/403	35/1880!	0.142857	6.666075859(	4.965116027(
BP	GO:000!	response	14/403	245/188(	0.057142	2.666430343	3.885316052
BP	GO:000:	positive	7/403	72/1880!	0.097222	4.536634960(	4.449364121(
BP	GO:000:	leukocyt	4/403	21/1880!	0.190476	8.888101146	5.352210793
BP	GO:004:	vitamin	4/403	21/1880!	0.190476	8.888101146	5.352210793
BP	GO:004	response	4/403	21/1880!	0.190476	8.888101146	5.352210793
BP	GO:009(	immunol	4/403	21/1880!	0.190476	8.888101146	5.352210793
BP	GO:190:	regulatic	4/403	21/1880!	0.190476	8.888101146	5.352210793
BP	GO:003(	monocyt	5/403	36/1880!	0.138888	6.480907085	4.871113558(
BP	GO:003:	positive	5/403	36/1880!	0.138888	6.480907085	4.871113558(
BP	GO:000	cell surf	20/403	427/188(	0.046838	2.185598642	3.667310132!
BP	GO:005(	regulatic	10/403	142/188(	0.070422	3.286093733(	4.046621722!
BP	GO:005(	regulatic	10/403	143/188(	0.069930(	3.263114057	4.020133724
BP	GO:000	activatio	3/403	10/1880!	0.3	13.99875930!	6.084513628
BP	GO:004	adhesion	3/403	10/1880!	0.3	13.99875930!	6.084513628

BP	GO:004	positive	3/403	10/1880	0.3	13.99875930	6.08451362
BP	GO:005	positive	12/403	195/188	0.061538	2.87154037	3.88766155
BP	GO:004	regulatio	18/403	369/188	0.048780	2.27622102	3.66396114
BP	GO:003	negative	4/403	22/1880	0.181818	8.48409654	5.19772590
BP	GO:003	regulatio	4/403	22/1880	0.181818	8.48409654	5.19772590
BP	GO:190	regulatio	4/403	22/1880	0.181818	8.48409654	5.19772590
BP	GO:005	positive	9/403	120/188	0.075	3.49968982	4.06513830
BP	GO:000	T cell di	8/403	97/1880	0.082474	3.84845616	4.16223750
BP	GO:004	positive	8/403	97/1880	0.082474	3.84845616	4.16223750
BP	GO:000	negative	7/403	76/1880	0.092105	4.29786469	4.26311910
BP	GO:004	cellular	7/403	76/1880	0.092105	4.29786469	4.26311910
BP	GO:000	aminogl	8/403	98/1880	0.081632	3.80918620	4.12607029
BP	GO:000	positive	6/403	56/1880	0.107142	4.99955689	4.43569799
BP	GO:003	negative	6/403	56/1880	0.107142	4.99955689	4.43569799
BP	GO:006	respirato	13/403	226/188	0.057522	2.68412789	3.76932210
BP	GO:004	positive	5/403	38/1880	0.131578	6.13980671	4.69338617
BP	GO:003	cellular	9/403	122/188	0.073770	3.44231786	4.00501295
BP	GO:001	organic	21/403	469/188	0.044776	2.08936706	3.53552157
BP	GO:001	negative	4/403	23/1880	0.173913	8.11522278	5.05273630
BP	GO:001	negative	4/403	23/1880	0.173913	8.11522278	5.05273630
BP	GO:004	glycospl	4/403	23/1880	0.173913	8.11522278	5.05273630
BP	GO:006	antifung	4/403	23/1880	0.173913	8.11522278	5.05273630
BP	GO:200	negative	4/403	23/1880	0.173913	8.11522278	5.05273630
BP	GO:004	phospha	16/403	316/188	0.050632	2.36265979	3.61509242
BP	GO:000	regulatio	8/403	100/188	0.08	3.73300248	4.05514418
BP	GO:004	negative	8/403	100/188	0.08	3.73300248	4.05514418
BP	GO:000	glycopro	5/403	39/1880	0.128205	5.98237577	4.60922657
BP	GO:007	negative	5/403	39/1880	0.128205	5.98237577	4.60922657
BP	GO:000	response	7/403	78/1880	0.089743	4.18766304	4.17475250
BP	GO:003	regulatio	9/403	124/188	0.072580	3.38679660	3.94612942
BP	GO:000	positive	3/403	11/1880	0.272727	12.7261448	5.75687919
BP	GO:000	immune	3/403	11/1880	0.272727	12.7261448	5.75687919
BP	GO:003	positive	3/403	11/1880	0.272727	12.7261448	5.75687919
BP	GO:007	response	3/403	11/1880	0.272727	12.7261448	5.75687919
BP	GO:007	cellular	3/403	11/1880	0.272727	12.7261448	5.75687919
BP	GO:007	cellular	3/403	11/1880	0.272727	12.7261448	5.75687919
BP	GO:004	gliogene	17/403	348/188	0.048850	2.27949145	3.56526312
BP	GO:190	response	6/403	58/1880	0.103448	4.82715838	4.31986001

BP	GO:2001	positive	6/403	58/1880	0.103448	4.8271583811	4.319860012
BP	GO:003	BMP sig	11/403	176/188	0.0625	2.9164081885	3.780025343
BP	GO:000	regulatic	9/403	125/188	0.072	3.3597022332	3.917138300
BP	GO:003	mainten	4/403	24/1880	0.166666	7.7770885028	4.916256717
BP	GO:003	regulatic	4/403	24/1880	0.166666	7.7770885028	4.916256717
BP	GO:003	lymph v	4/403	24/1880	0.166666	7.7770885028	4.916256717
BP	GO:004	positive	4/403	24/1880	0.166666	7.7770885028	4.916256717
BP	GO:004	positive	4/403	24/1880	0.166666	7.7770885028	4.916256717
BP	GO:006	positive	4/403	24/1880	0.166666	7.7770885028	4.916256717
BP	GO:007	hepatocy	4/403	24/1880	0.166666	7.7770885028	4.916256717
BP	GO:007	epithelia	4/403	24/1880	0.166666	7.7770885028	4.916256717
BP	GO:001	positive	9/403	126/188	0.071428	3.3330379298	3.888439911
BP	GO:006	glial cell	6/403	59/1880	0.101694	4.7453421373	4.263912925
BP	GO:004	smooth	11/403	178/188	0.061797	2.8836395572	3.736641881
BP	GO:000	nitric ox	7/403	80/1880	0.0875	4.0829714640	4.089297588
BP	GO:010	cold-ind	10/403	152/188	0.065789	3.0699033564	3.791775785
BP	GO:009	regulatic	18/403	384/188	0.04688	2.1873061414	3.478699031
BP	GO:000	leukocyt	10/403	153/188	0.065359	3.0498386285	3.767452766
BP	GO:190	positive	7/403	81/1880	0.086419	4.0325644089	4.047607183
BP	GO:005	negative	12/403	207/188	0.057971	2.7050742618	3.650400211
BP	GO:004	hepatocy	6/403	60/1880	0.1	4.6662531017	4.209208874
BP	GO:005	positive	6/403	60/1880	0.1	4.6662531017	4.209208874
BP	GO:001	regulatic	13/403	236/188	0.055084	2.5703936577	3.592654114
BP	GO:007	negative	4/403	25/1880	0.16	7.4660049627	4.787440342
BP	GO:190	regulatic	4/403	25/1880	0.16	7.4660049627	4.787440342
BP	GO:190	negative	4/403	25/1880	0.16	7.4660049627	4.787440342
BP	GO:009	dendritic	3/403	12/1880	0.25	11.66563275	5.469206521
BP	GO:200	regulatic	3/403	12/1880	0.25	11.66563275	5.469206521
BP	GO:000	positive	6/403	61/1880	0.098360	4.5897571492	4.155698006
BP	GO:000	regulatic	5/403	42/1880	0.119047	5.5550632163	4.373338019
BP	GO:005	cartilage	12/403	210/188	0.057142	2.6664303438	3.593716819
BP	GO:001	response	7/403	83/1880	0.084337	3.9353941821	3.966198865
BP	GO:200	regulatic	7/403	83/1880	0.084337	3.9353941821	3.966198865
BP	GO:001	negative	9/403	131/188	0.068702	3.2058227416	3.749149094
BP	GO:009	regulatic	16/403	329/188	0.048632	2.2693024202	3.437195317
BP	GO:000	negative	6/403	62/1880	0.096774	4.5157288081	4.103333264
BP	GO:000	embryo i	6/403	62/1880	0.096774	4.5157288081	4.103333264
BP	GO:003	collagen	6/403	62/1880	0.096774	4.5157288081	4.103333264

BP	GO:004:	CD4-pos	8/403	107/188(	0.074766:	3.488787365'	3.820561858:
BP	GO:000(	glycosan	4/403	26/1880!	0.153846	7.178850925'	4.665554763(
BP	GO:001!	glycolipi	4/403	26/1880!	0.153846	7.178850925'	4.665554763(
BP	GO:004{	astrocyte	4/403	26/1880!	0.153846	7.178850925'	4.665554763(
BP	GO:005(	positive	4/403	26/1880!	0.153846	7.178850925'	4.665554763(
BP	GO:007:	liver mo	4/403	26/1880!	0.153846	7.178850925'	4.665554763(
BP	GO:009(	positive	4/403	26/1880!	0.153846	7.178850925'	4.665554763(
BP	GO:004{	macroph	5/403	43/1880!	0.116279(	5.425875699(	4.299708423{
BP	GO:003	heteroty	6/403	63/1880!	0.095238(	4.444050573(	4.052070186
BP	GO:004:	regulatic	6/403	63/1880!	0.095238(	4.444050573(	4.052070186
BP	GO:190!	macroph	6/403	63/1880!	0.095238(	4.444050573(	4.052070186
BP	GO:004{	ephrin re	7/403	85/1880!	0.082352!	3.842796672(	3.887294735:
BP	GO:003:	detectior	3/403	13/1880!	0.230769:	10.76827638{	5.213726491!
BP	GO:003:	regulatic	3/403	13/1880!	0.230769:	10.76827638{	5.213726491!
BP	GO:003:	regulatic	3/403	13/1880!	0.230769:	10.76827638{	5.213726491!
BP	GO:003(	TRAIL-a	3/403	13/1880!	0.230769:	10.76827638{	5.213726491!
BP	GO:004	adhesior	3/403	13/1880!	0.230769:	10.76827638{	5.213726491!
BP	GO:005	negative	3/403	13/1880!	0.230769:	10.76827638{	5.213726491!
BP	GO:007(	extracell	3/403	13/1880!	0.230769:	10.76827638{	5.213726491!
BP	GO:190:	positive	3/403	13/1880!	0.230769:	10.76827638{	5.213726491!
BP	GO:000'	synapse	14/403	273/188(	0.051282(	2.392950308!	3.430843696(
BP	GO:000:	organ or	5/403	44/1880!	0.113636:	5.302560342{	4.228345316
BP	GO:005(	intestina	5/403	44/1880!	0.113636:	5.302560342{	4.228345316
BP	GO:005(	regulatic	9/403	134/188(	0.067164	3.134050590'	3.668752813
BP	GO:000!	oligosac	6/403	64/1880!	0.09375	4.374612282{	4.001866721
BP	GO:004{	positive	6/403	64/1880!	0.09375	4.374612282{	4.001866721
BP	GO:003(	positive	4/403	27/1880!	0.148148	6.912967558	4.549962759'
BP	GO:004:	peptide l	4/403	27/1880!	0.148148	6.912967558	4.549962759'
BP	GO:190(	positive	4/403	27/1880!	0.148148	6.912967558	4.549962759'
BP	GO:005	regulatic	20/403	463/188(	0.043196!	2.015660087	3.274618177(
BP	GO:003	negative	11/403	188/188(	0.058510(	2.730254474	3.528411162!
BP	GO:014	transforr	18/403	399/188(	0.045112'	2.105076587:	3.301750467
BP	GO:006(	epithelia	16/403	337/188(	0.047477'	2.215431739(	3.331813794
BP	GO:009'	response	14/403	276/188(	0.050724(	2.366939979	3.385502408!
BP	GO:000:	negative	6/403	65/1880!	0.092307(	4.307310555	3.952683065!
BP	GO:001(	vascular	6/403	65/1880!	0.092307(	4.307310555	3.952683065!
BP	GO:007	icosanoi	6/403	65/1880!	0.092307(	4.307310555	3.952683065!
BP	GO:003(	collagen	5/403	45/1880!	0.111111	5.184725668!	4.159124912{

BP	GO:003	negative	5/403	45/1880	0.111111	5.184725668	4.159124912
BP	GO:005	regulatic	5/403	45/1880	0.111111	5.184725668	4.159124912
BP	GO:004	odontog	9/403	136/188	0.066176	3.087961611	3.616400886
BP	GO:005	positive	12/403	218/188	0.055045	2.568579689	3.447270124
BP	GO:005	negative	20/403	467/188	0.042826	1.998395332	3.233181828
BP	GO:000	CD4-pos	7/403	88/1880	0.079545	3.711792240	3.773329758
BP	GO:004	nitric ox	7/403	88/1880	0.079545	3.711792240	3.773329758
BP	GO:003	positive	4/403	28/1880	0.142857	6.666075859	4.440106921
BP	GO:003	regulatic	4/403	28/1880	0.142857	6.666075859	4.440106921
BP	GO:003	positive	4/403	28/1880	0.142857	6.666075859	4.440106921
BP	GO:007	positive	4/403	28/1880	0.142857	6.666075859	4.440106921
BP	GO:006	morphog	11/403	191/188	0.057591	2.687370896	3.468586377
BP	GO:001	negative	6/403	66/1880	0.090909	4.242048274	3.904481510
BP	GO:000	immune	17/403	372/188	0.045698	2.132427492	3.264592616
BP	GO:000	negative	5/403	46/1880	0.108695	5.072014241	4.091932805
BP	GO:004	regulatic	5/403	46/1880	0.108695	5.072014241	4.091932805
BP	GO:000	epithelia	11/403	192/188	0.057291	2.673374172	3.448900129
BP	GO:000	response	3/403	14/1880	0.214285	9.999113789	4.984641696
BP	GO:003	positive	3/403	14/1880	0.214285	9.999113789	4.984641696
BP	GO:003	positive	3/403	14/1880	0.214285	9.999113789	4.984641696
BP	GO:003	immatur	3/403	14/1880	0.214285	9.999113789	4.984641696
BP	GO:003	immatur	3/403	14/1880	0.214285	9.999113789	4.984641696
BP	GO:003	positive	3/403	14/1880	0.214285	9.999113789	4.984641696
BP	GO:004	amino su	3/403	14/1880	0.214285	9.999113789	4.984641696
BP	GO:007	positive	3/403	14/1880	0.214285	9.999113789	4.984641696
BP	GO:007	commiss	3/403	14/1880	0.214285	9.999113789	4.984641696
BP	GO:200	regulatic	3/403	14/1880	0.214285	9.999113789	4.984641696
BP	GO:000	alpha-be	7/403	89/1880	0.078651	3.670086709	3.736448332
BP	GO:000	alpha-be	7/403	89/1880	0.078651	3.670086709	3.736448332
BP	GO:200	regulatic	10/403	165/188	0.060606	2.828032182	3.490181294
BP	GO:006	mesench	16/403	342/188	0.046783	2.183042386	3.267437991
BP	GO:003	muscle c	13/403	251/188	0.051792	2.416784475	3.344005668
BP	GO:007	positive	8/403	114/188	0.070175	3.274563580	3.604785015
BP	GO:200	reactive	7/403	90/1880	0.077777	3.629307968	3.700095451
BP	GO:007	cellular	16/403	344/188	0.046511	2.170350279	3.241996969
BP	GO:010	cellular	16/403	344/188	0.046511	2.170350279	3.241996969
BP	GO:004	sulfur co	5/403	47/1880	0.106382	4.964099044	4.026663066
BP	GO:004	positive	5/403	47/1880	0.106382	4.964099044	4.026663066

BP	GO:0048	lymphoc	5/403	47/18805	0.1063829	4.9640990444	4.0266630667
BP	GO:1905	regulatic	5/403	47/18805	0.1063829	4.9640990444	4.0266630667
BP	GO:0090	inflamm	4/403	29/18805	0.1379310	6.4362111748	4.3354972069
BP	GO:0051	positive	6/403	68/18805	0.0882352	4.1172821485	3.8108834927
BP	GO:0060	positive	6/403	68/18805	0.0882352	4.1172821485	3.8108834927
BP	GO:0030	B cell di	9/403	141/1880	0.0638297	2.9784594260	3.4896307321
BP	GO:0051	response	7/403	91/18805	0.0769230	3.5894254628	3.6642570239
BP	GO:0001	regulatic	5/403	48/18805	0.1041666	4.8606803143	3.9632174528
BP	GO:0002	negative	5/403	48/18805	0.1041666	4.8606803143	3.9632174528
BP	GO:0071	semapho	5/403	48/18805	0.1041666	4.8606803143	3.9632174528
BP	GO:0097	dendritic	5/403	48/18805	0.1041666	4.8606803143	3.9632174528
BP	GO:0001	response	15/403	316/1880	0.0474683	2.2149935609	3.2233386637
BP	GO:0051	regulatic	10/403	169/1880	0.0591715	2.7610965099	3.4032612511
BP	GO:1990	adaptive	10/403	169/1880	0.0591715	2.7610965099	3.4032612511
BP	GO:0032	negative	4/403	30/18805	0.1333333	6.2216708023	4.2357007918
BP	GO:0043	positive	4/403	30/18805	0.1333333	6.2216708023	4.2357007918
BP	GO:1903	positive	4/403	30/18805	0.1333333	6.2216708023	4.2357007918
BP	GO:0014	neural cr	3/403	15/18805	0.2	9.3325062034	4.7775255275
BP	GO:0060	positive	3/403	15/18805	0.2	9.3325062034	4.7775255275
BP	GO:0097	mast cell	3/403	15/18805	0.2	9.3325062034	4.7775255275
BP	GO:0006	vitamin	8/403	117/1880	0.0683760	3.1906004114	3.5173842468
BP	GO:0002	peptide s	13/403	257/1880	0.0505836	2.36036149	3.2495070139
BP	GO:0036	vascular	5/403	49/18805	0.1020408	4.7614827568	3.9015046999
BP	GO:0061	establish	5/403	49/18805	0.1020408	4.7614827568	3.9015046999
BP	GO:0045	regulatic	20/403	482/1880	0.0414937	1.9362046065	3.0813507239
BP	GO:0042	odontog	7/403	94/18805	0.0744680	3.4748693310	3.5596953594
BP	GO:0019	antigen p	4/403	31/18805	0.1290322	6.0209717441	4.1403337299
BP	GO:0070	response	4/403	31/18805	0.1290322	6.0209717441	4.1403337299
BP	GO:0000	response	11/403	201/1880	0.0547263	2.5536708516	3.2771703162
BP	GO:0071	response	10/403	173/1880	0.0578034	2.6972561281	3.3188362908
BP	GO:0001	microgli	5/403	50/18805	0.1	4.6662531017	3.8414398957
BP	GO:0097	regulatic	7/403	95/18805	0.0736842	3.4382917591	3.5257840672
BP	GO:0048	regulatic	10/403	174/1880	0.0574712	2.6817546561	3.2981037332
BP	GO:0002	dendritic	3/403	16/18805	0.1875	8.7492245657	4.5889310866
BP	GO:0003	renal soc	3/403	16/18805	0.1875	8.7492245657	4.5889310866
BP	GO:0009	oligosac	3/403	16/18805	0.1875	8.7492245657	4.5889310866
BP	GO:0030	proteogl	3/403	16/18805	0.1875	8.7492245657	4.5889310866
BP	GO:0030	hyaluron	3/403	16/18805	0.1875	8.7492245657	4.5889310866

BP	GO:006(	venous b	3/403	16/1880!	0.1875	8.74922456$5_7$	4.58893108$6_6$
BP	GO:190(	regulatic	3/403	16/1880!	0.1875	8.749224565	4.588931086
BP	GO:003	response	15/403	324/188(	0.046296	2.160302361	3.117640819
BP	GO:003	response	8/403	121/188(	0.066115	3.085126017	3.405141740
BP	GO:000	negative	4/403	32/1880!	0.125	5.832816377	4.049054055
BP	GO:000	mature E	4/403	32/1880!	0.125	5.832816377	4.049054055
BP	GO:000	negative	4/403	32/1880!	0.125	5.832816377	4.049054055
BP	GO:003	negative	4/403	32/1880!	0.125	5.832816377	4.049054055
BP	GO:003	positive	4/403	32/1880!	0.125	5.832816377	4.049054055
BP	GO:004	regulatic	4/403	32/1880!	0.125	5.832816377	4.049054055
BP	GO:007(	response	16/403	358/188(	0.044692	2.085476246	3.068626949
BP	GO:001	positive	6/403	73/1880!	0.082191	3.835276521	3.591781492
BP	GO:005(	positive	6/403	73/1880!	0.082191	3.835276521	3.591781492
BP	GO:200	positive	9/403	149/188(	0.060402	2.818542141	3.298013039
BP	GO:000	ossificat	19/403	458/188(	0.041484	1.935781854	3.000336458
BP	GO:009(	negative	11/403	206/188(	0.053398	2.491688549	3.185752995
BP	GO:000(	unsatura	5/403	52/1880!	0.096153	4.486781828	3.725942943
BP	GO:000(	cellular (	5/403	52/1880!	0.096153	4.486781828	3.725942943
BP	GO:003(	response	5/403	52/1880!	0.096153	4.486781828	3.725942943
BP	GO:003(	cellular r	5/403	52/1880!	0.096153	4.486781828	3.725942943
BP	GO:003	Kit signa	5/403	52/1880!	0.096153	4.486781828	3.725942943
BP	GO:003(	negative	6/403	74/1880!	0.081081	3.783448460	3.550288888
BP	GO:007	cellular r	6/403	74/1880!	0.081081	3.783448460	3.550288888
BP	GO:000	myeloid	7/403	98/1880!	0.071428	3.333037929	3.426714287
BP	GO:001(	regulatic	4/403	33/1880!	0.121212	5.656064365	3.961556039
BP	GO:003(	response	15/403	330/188(	0.045454	2.121024137	3.040358816
BP	GO:003	granuloc	3/403	17/1880!	0.176470	8.234564297	4.416128448
BP	GO:003	regulatic	3/403	17/1880!	0.176470	8.234564297	4.416128448
BP	GO:004	cellular r	3/403	17/1880!	0.176470	8.234564297	4.416128448
BP	GO:012(	regulatic	9/403	151/188(	0.059602	2.781210457	3.252092238
BP	GO:000	morphog	11/403	208/188(	0.052884	2.467730005	3.149934520
BP	GO:007	cellular r	6/403	75/1880!	0.08	3.733002481	3.509513361
BP	GO:007	cellular r	6/403	75/1880!	0.08	3.733002481	3.509513361
BP	GO:006(	SMAD p	7/403	99/1880!	0.070707	3.299370880	3.394542812
BP	GO:000	leukocyt	5/403	53/1880!	0.094339	4.402125567	3.670367980
BP	GO:014(	lipid exp	5/403	53/1880!	0.094339	4.402125567	3.670367980
BP	GO:000	innate in	7/403	100/188(	0.07	3.266377171	3.362780046
BP	GO:005(	leukocyt	4/403	34/1880!	0.117647	5.489709531	3.877565381

BP	GO:007	interleuk	4/403	34/1880	0.117647	5.489709531	3.877565381
BP	GO:007	regulatic	4/403	34/1880	0.117647	5.489709531	3.877565381
BP	GO:000	cell-mat	12/403	240/188	0.05	2.333126550	3.075917289
BP	GO:200	regulatic	17/403	399/188	0.042606	1.988127887	2.952330864
BP	GO:003	axon reg	5/403	54/1880	0.092592	4.320604723	3.616154484
BP	GO:005	negative	5/403	54/1880	0.092592	4.320604723	3.616154484
BP	GO:012	positive	7/403	101/188	0.069306	3.234036803	3.331416208
BP	GO:002	gland m	8/403	127/188	0.062992	2.939372032	3.245210963
BP	GO:004	positive	6/403	77/1880	0.077922	3.636041377	3.430022875
BP	GO:000	positive	3/403	18/1880	0.166666	7.777088502	4.256923279
BP	GO:000	positive	3/403	18/1880	0.166666	7.777088502	4.256923279
BP	GO:003	triglycer	3/403	18/1880	0.166666	7.777088502	4.256923279
BP	GO:003	positive	3/403	18/1880	0.166666	7.777088502	4.256923279
BP	GO:004	negative	3/403	18/1880	0.166666	7.777088502	4.256923279
BP	GO:015	negative	3/403	18/1880	0.166666	7.777088502	4.256923279
BP	GO:007	cellular	16/403	369/188	0.043360	2.023307577	2.937860396
BP	GO:004	negative	7/403	102/188	0.068627	3.202330560	3.300441849
BP	GO:001	regulatic	5/403	55/1880	0.090909	4.242048274	3.563241986
BP	GO:003	brain-de	5/403	55/1880	0.090909	4.242048274	3.563241986
BP	GO:003	platelet-	5/403	55/1880	0.090909	4.242048274	3.563241986
BP	GO:003	negative	4/403	35/1880	0.114285	5.332860687	3.796835160
BP	GO:005	positive	4/403	35/1880	0.114285	5.332860687	3.796835160
BP	GO:009	regulatic	4/403	35/1880	0.114285	5.332860687	3.796835160
BP	GO:000	respirato	6/403	78/1880	0.076923	3.589425462	3.391265107
BP	GO:004	regulatic	9/403	156/188	0.057692	2.692069097	3.140492421
BP	GO:004	mesench	13/403	275/188	0.047272	2.205865102	2.981072205
BP	GO:003	granuloc	5/403	56/1880	0.089285	4.166297412	3.511573767
BP	GO:004	positive	5/403	56/1880	0.089285	4.166297412	3.511573767
BP	GO:003	regulatic	14/403	309/188	0.045307	2.114159981	2.922355309
BP	GO:000	B cell h	4/403	36/1880	0.111111	5.184725668	3.719142401
BP	GO:003	regulatic	4/403	36/1880	0.111111	5.184725668	3.719142401
BP	GO:003	response	4/403	36/1880	0.111111	5.184725668	3.719142401
BP	GO:000	Notch si	10/403	187/188	0.053475	2.495322514	3.041125156
BP	GO:005	regulatic	13/403	278/188	0.046762	2.182060802	2.938358588
BP	GO:000	cell surf	8/403	131/188	0.061068	2.849620214	3.143725841
BP	GO:000	positive	3/403	19/1880	0.157894	7.367768055	4.109528612
BP	GO:003	negative	3/403	19/1880	0.157894	7.367768055	4.109528612
BP	GO:004	regulatic	3/403	19/1880	0.157894	7.367768055	4.109528612

BP	GO:009	detection	3/403	19/1880	0.157894	7.367768055	4.109528612
BP	GO:200	negative	3/403	19/1880	0.157894	7.367768055	4.109528612
BP	GO:000	cytokine	6/403	80/1880	0.075	3.499689826	3.315624454
BP	GO:001	regulatio	6/403	80/1880	0.075	3.499689826	3.315624454
BP	GO:190	regulatio	6/403	80/1880	0.075	3.499689826	3.315624454
BP	GO:000	regulatio	10/403	189/188	0.052910	2.468916985	3.003517043
BP	GO:000	cell surf	6/403	81/1880	0.074074	3.456483779	3.278704109
BP	GO:003	response	4/403	37/1880	0.108108	5.044597947	3.644285163
BP	GO:003	collagen	5/403	58/1880	0.086206	4.022631984	3.411760303
BP	GO:006	leukocyt	5/403	58/1880	0.086206	4.022631984	3.411760303
BP	GO:001	response	15/403	347/188	0.043227	2.017112291	2.830000097
BP	GO:000	producti	7/403	107/188	0.065420	3.052688945	3.151102901
BP	GO:001	regulatio	18/403	448/188	0.040178	1.874833835	2.773376657
BP	GO:001	peptide t	13/403	283/188	0.045936	2.143508491	2.868360961
BP	GO:001	positive	3/403	20/1880	0.15	6.999379652	3.972472277
BP	GO:003	interleuk	3/403	20/1880	0.15	6.999379652	3.972472277
BP	GO:003	regulatio	3/403	20/1880	0.15	6.999379652	3.972472277
BP	GO:000	T cell m	5/403	59/1880	0.084745	3.954451781	3.363517851
BP	GO:006	maternal	5/403	59/1880	0.084745	3.954451781	3.363517851
BP	GO:000	mature B	4/403	38/1880	0.105263	4.911845370	3.57208004
BP	GO:001	epithelia	4/403	38/1880	0.105263	4.911845370	3.57208004
BP	GO:001	macroph	4/403	38/1880	0.105263	4.911845370	3.57208004
BP	GO:001	macroph	4/403	38/1880	0.105263	4.911845370	3.57208004
BP	GO:001	regulatio	4/403	38/1880	0.105263	4.911845370	3.57208004
BP	GO:004	regulatio	4/403	38/1880	0.105263	4.911845370	3.57208004
BP	GO:002	glial cell	8/403	135/188	0.059259	2.765187023	3.046004793
BP	GO:004	response	8/403	135/188	0.059259	2.765187023	3.046004793
BP	GO:003	glycosan	6/403	83/1880	0.072289	3.373195013	3.206575603
BP	GO:006	regulatio	6/403	83/1880	0.072289	3.373195013	3.206575603
BP	GO:001	lipid cat	15/403	350/188	0.042857	1.999822757	2.794120595
BP	GO:000	JNK cas	10/403	193/188	0.051813	2.417747721	2.929720713
BP	GO:005	response	8/403	136/188	0.058823	2.744854765	3.022131387
BP	GO:000	ovarian f	5/403	60/1880	0.083333	3.888544251	3.316324803
BP	GO:000	regulatio	10/403	194/188	0.051546	2.405285103	2.911558927
BP	GO:004	cellular r	9/403	165/188	0.054545	2.545228964	2.950237571
BP	GO:009	foam cel	4/403	39/1880	0.102564	4.785900617	3.502360029
BP	GO:003	negative	3/403	21/1880	0.142857	6.666075859	3.844528842
BP	GO:004	lymph n	3/403	21/1880	0.142857	6.666075859	3.844528842

BP	GO:1903	regulatic	3/403	21/18805	0.1428571	6.6660758590	3.8445288427
BP	GO:1905	regulatic	3/403	21/18805	0.1428571	6.6660758590	3.8445288427
BP	GO:0030	negative	16/403	388/18800	0.0412371	1.9242280831	2.7222669497
CC	GO:0062	collagen	61/421	432/1988	0.1412037	6.6677663411	17.518398207
CC	GO:0009	external	46/421	401/1988	0.1147132	5.4168616463	13.142582409
CC	GO:0005	endoplas	38/421	315/1988	0.1206349	5.6964898390	12.358529357
CC	GO:0043	lysosoma	22/421	98/19880	0.2244897	10.600610790	14.013765907
CC	GO:0060	cytoplas	35/421	329/1988	0.1063829	5.0235002779	10.8241936
CC	GO:0031	vesicle l	35/421	330/1988	0.1060606	5.0082775498	10.799892461
CC	GO:0034	secretory	34/421	324/1988	0.1049382	4.9552799038	10.558148577
CC	GO:0005	vacuolar	25/421	178/1988	0.1404494	6.6321492433	11.102149059
CC	GO:0072	blood mi	20/421	145/1988	0.1379310	6.5132279465	9.8005272470
CC	GO:0042	specific	18/421	160/1988	0.1125	5.3123515439	8.0556158767
CC	GO:0070	tertiary g	18/421	164/1988	0.1097560	5.1827819940	7.9114448470
CC	GO:0031	platelet a	12/421	67/19880	0.1791044	8.4574750948	8.9935706152
CC	GO:0045	membran	24/421	291/1988	0.0824742	3.8945074319	7.3163266341
CC	GO:1904	tertiary g	11/421	55/19880	0.2	9.4441805225	9.2238092208
CC	GO:0098	membran	24/421	293/1988	0.0819112	3.8679237635	7.2743720060
CC	GO:0031	platelet a	13/421	91/19880	0.1428571	6.7458432304	8.0805440140
CC	GO:0005	Golgi lu	13/421	106/1988	0.1226415	5.7912427732	7.2749816240
CC	GO:0030	secretory	23/421	322/1988	0.0714285	3.3729216152	6.3143283687
CC	GO:0045	clathrin-	12/421	93/19880	0.1290322	6.0930196919	7.2411060580
CC	GO:0035	specific	10/421	62/19880	0.1612903	7.6162746149	7.6746311348
CC	GO:0005	primary	15/421	155/1988	0.0967741	4.5697647689	6.5625858524
CC	GO:0042	azurophi	15/421	155/1988	0.0967741	4.5697647689	6.5625858524
CC	GO:0101	ficolin-1	16/421	185/1988	0.0864864	4.0839699557	6.1986350242
CC	GO:0005	multives	9/421	67/19880	0.1343283	6.3431063211	6.4436828005
CC	GO:1904	ficolin-1	12/421	124/1988	0.0967741	4.5697647689	5.8651481774
CC	GO:0030	clathrin-	9/421	75/19880	0.12	5.6665083135	5.9554325179
CC	GO:0035	azurophi	8/421	58/19880	0.1379310	6.5132279465	6.1847801378
CC	GO:0072	perineur	4/421	10/19880	0.4	18.888361045	8.3224208584
CC	GO:0098	perisyna	4/421	11/19880	0.3636363	17.171237313	7.8909564021
CC	GO:0030	coated v	19/421	322/1988	0.0590062	2.7863265510	4.7534028721
CC	GO:0140	specializ	5/421	22/19880	0.2272727	10.732023321	6.7177646381
CC	GO:0030	clathrin-	15/421	222/1988	0.0675675	3.1906015278	4.8277888107
CC	GO:0030	endocyti	20/421	358/1988	0.0558659	2.6380392521	4.6002530223
CC	GO:0005	collagen	9/421	86/19880	0.1046511	4.9417223664	5.3882350184
CC	GO:0099	synapse-	4/421	12/19880	0.3333333	15.740300870	7.5127329457

CC	GO:003(	endocyti	14/421	207/198	0.067632	3.193684234	4.666615794
CC	GO:003	platelet (	4/421	14/1988	0.285714	13.49168646	6.877141068
CC	GO:000	basemen	9/421	97/1988	0.092783	4.381320860	4.910257088
CC	GO:004	lamellar	4/421	18/1988	0.222222	10.49353391	5.926942659
CC	GO:000	late endo	17/421	320/198	0.05313	2.508610451	4.001711098
CC	GO:003	specific	8/421	91/1988	0.087912	4.151288141	4.431746831
CC	GO:004	platelet (	4/421	21/1988	0.190476	8.994457640	5.391356136
CC	GO:190	serine-ty	4/421	21/1988	0.190476	8.994457640	5.391356136
CC	GO:009	protein c	6/421	58/1988	0.103448	4.884920959	4.358133243
CC	GO:003(	clathrin-	9/421	138/198	0.065217	3.079624083	3.605843414
CC	GO:000	semapho	3/421	14/1988	0.214285	10.11876484	5.020221441
CC	GO:003	azurophi	7/421	91/1988	0.076923	3.632377124	3.701987395
CC	GO:003(	COPII-c	7/421	95/1988	0.073684	3.479434929	3.563071571
CC	GO:009	plasma r	15/421	326/198	0.046012	2.172740917	3.140299923
CC	GO:007(	tertiary g	6/421	73/1988	0.082191	3.881170077	3.627429919
CC	GO:004	MHC cla	3/421	17/1988	0.176470	8.333100461	4.449054541
CC	GO:190	serine-ty	3/421	19/1988	0.157894	7.455931991	4.141076440
CC	GO:000	lysosoma	18/421	451/198	0.039911	1.884647997	2.795191722
CC	GO:009	lytic vac	18/421	451/198	0.039911	1.884647997	2.795191722
CC	GO:003	endosom	4/421	38/1988	0.105263	4.970621327	3.603591902
MF	GO:003(	carbohy	38/408	280/186	0.135714	6.199947478	13.11529161
MF	GO:000	cytokine	31/408	234/186	0.132478	6.052130467	11.63426803
MF	GO:000	endopep	25/408	161/186	0.155279	7.093761417	11.61703754
MF	GO:000	glycosan	30/408	242/186	0.123966	5.663283908	10.92321609
MF	GO:006	endopep	26/408	184/186	0.141304	6.455322890	11.12491847
MF	GO:003(	peptidas	25/408	169/186	0.147928	6.757962060	11.24872224
MF	GO:000	death rec	9/408	13/1863	0.692307	31.62726244	16.52511713
MF	GO:000	serine-ty	18/408	95/1863	0.189473	8.655882352	11.19128186
MF	GO:000	cytokine	29/408	275/186	0.105454	4.817566844	9.540998878
MF	GO:006	peptidas	26/408	226/186	0.115044	5.255661114	9.629050618
MF	GO:000	tumor ne	7/408	10/1863	0.7	31.97867647	14.65860281
MF	GO:000	serine-ty	22/408	194/186	0.113402	5.180639781	8.756464145
MF	GO:001	serine hy	22/408	198/186	0.111111	5.075980392	8.625751394
MF	GO:000	serine-ty	20/408	176/186	0.113636	5.191343582	8.357626372
MF	GO:001	cytokine	18/408	144/186	0.125	5.710477941	8.488746067
MF	GO:000	growth f	19/408	162/186	0.117283	5.357979302	8.333960369
MF	GO:003	carboxyl	20/408	187/186	0.106951	4.885970430	7.989563662
MF	GO:004	organic a	20/408	189/186	0.105820	4.834267040	7.925735281

MF	GO:000	heparin l	19/408	172/186	0.110465	5.046468878	7.975662043
MF	GO:014	immune	18/408	156/186	0.115384	5.271210407	8.014040321
MF	GO:000	virus rec	13/408	81/1863	0.160493	7.331971677	8.543589583
MF	GO:014	exogeno	13/408	82/1863	0.158536	7.242557388	8.475007195
MF	GO:000	extracell	18/408	168/186	0.107142	4.894695378	7.585905383
MF	GO:000	D-mann	8/408	25/1863	0.32	14.61882352	10.19328651
MF	GO:190	sulfur co	23/408	275/186	0.083636	3.820828877	7.049915887
MF	GO:000	enzyme	29/408	416/186	0.069711	3.184689621	6.741413445
MF	GO:003	pattern r	10/408	48/1863	0.208333	9.517463235	8.839017640
MF	GO:000	endopep	28/408	405/186	0.069135	3.158387799	6.569623838
MF	GO:005	extracell	11/408	67/1863	0.164179	7.500329236	7.973847167
MF	GO:001	growth f	15/408	136/186	0.110294	5.038657006	7.071472237
MF	GO:004	monosac	11/408	74/1863	0.148648	6.790838632	7.466796854
MF	GO:003	low-den	6/408	17/1863	0.352941	16.12370242	9.332412199
MF	GO:000	cytokine	12/408	96/1863	0.125	5.710477941	6.922055569
MF	GO:003	cargo re	10/408	65/1863	0.153846	7.028280542	7.283194408
MF	GO:003	sialic aci	6/408	22/1863	0.272727	12.45922459	8.045188281
MF	GO:000	fatty aci	8/408	47/1863	0.170212	7.775969962	6.957958521
MF	GO:005	transforr	6/408	24/1863	0.25	11.42095588	7.641984413
MF	GO:000	cysteine-	7/408	36/1863	0.194444	8.882965686	7.082292890
MF	GO:004	CCR ch	8/408	50/1863	0.16	7.309411764	6.682982892
MF	GO:000	complen	6/408	25/1863	0.24	10.96411764	7.457847282
MF	GO:000	scaveng	6/408	27/1863	0.222222	10.15196078	7.119082083
MF	GO:004	chemoki	9/408	72/1863	0.125	5.710477941	5.990800309
MF	GO:000	chemoki	7/408	45/1863	0.155555	7.106372549	6.135184538
MF	GO:001	transmer	9/408	78/1863	0.115384	5.271210407	5.654862791
MF	GO:001	corecept	8/408	61/1863	0.131147	5.991321118	5.841245227
MF	GO:003	monocar	9/408	79/1863	0.113924	5.204486224	5.602243525
MF	GO:007	lipoprote	6/408	32/1863	0.1875	8.565716911	6.407835987
MF	GO:007	protein-l	6/408	32/1863	0.1875	8.565716911	6.407835987
MF	GO:000	complen	4/408	11/1863	0.363636	16.61229946	7.748267230
MF	GO:000	opsonin	5/408	21/1863	0.238095	10.87710084	6.774809873
MF	GO:000	exopepti	10/408	102/186	0.098039	4.478806228	5.270296995
MF	GO:001	hydrolas	11/408	130/186	0.084615	3.865554298	4.904709958
MF	GO:003	inhibitor	4/408	13/1863	0.307692	14.05656108	7.044743280
MF	GO:002	MHC pr	6/408	37/1863	0.162162	7.408187599	5.836879787
MF	GO:000	hydrolas	9/408	91/1863	0.098901	4.518180349	5.032858737
MF	GO:009	cell-cell	7/408	55/1863	0.127272	5.814304812	5.348965783

MF	GO:000(	metalloe	4/408	14/1863!	0.285714:	13.05252100(	6.7486711141
MF	GO:007(	growth f	11/408	139/186:	0.079136(	3.615266610:	4.629797485!
MF	GO:000‹	carbonat	4/408	15/1863!	0.266666(	12.18235294:	6.481370278:
MF	GO:004:	chemoat	6/408	42/1863!	0.142857:	6.526260504:	5.363632859!
MF	GO:000‹	transmer	7/408	60/1863!	0.116666(	5.329779411:	5.025215822:
MF	GO:001!	retinol b	4/408	16/1863!	0.25	11.42095588:	6.238313797‹
MF	GO:003(	signaling	6/408	44/1863!	0.136363(	6.229612299‹	5.1954387591
MF	GO:005:	glycolipi	5/408	29/1863!	0.172413:	7.876521298:	5.543947316(
MF	GO:003(	long-cha	4/408	17/1863!	0.235294:	10.74913494(	6.015917490:
MF	GO:007(	oligosac	4/408	17/1863!	0.235294:	10.74913494(	6.015917490:
MF	GO:000:	fibronec	5/408	30/1863!	0.166666(	7.613970588:	5.423577600:
MF	GO:000‹	carboxyp	6/408	46/1863!	0.130434:	5.958759590:	5.037345328!
MF	GO:000!	tumor ne	5/408	31/1863!	0.161290:	7.368358633:	5.308636644(
MF	GO:003:	MHC cl	4/408	18/1863!	0.222222:	10.15196078‹	5.811301296‹
MF	GO:003‹	apolipop	4/408	18/1863!	0.222222:	10.15196078‹	5.811301296‹
MF	GO:000!	hormone	10/408	132/186:	0.075757!	3.460895721!	4.244598870(
MF	GO:001(	retinal b	4/408	19/1863!	0.210526:	9.617647058(	5.622120571(
MF	GO:009:	ceramid	4/408	19/1863!	0.210526:	9.617647058(	5.622120571(
MF	GO:000!	epiderm	6/408	50/1863!	0.12	5.482058823!	4.747435031!
MF	GO:003:	tumor ne	6/408	50/1863!	0.12	5.482058823!	4.747435031!
MF	GO:000!	neurotro	6/408	51/1863!	0.117647(	5.374567474(	4.679811554!
MF	GO:000:	protease	10/408	136/186:	0.073529‹	3.359104671:	4.130666000!
MF	GO:000!	integrin	11/408	161/186:	0.068322!	3.121255023:	4.043922558:
MF	GO:000:	G protei	16/408	297/186:	0.053872(	2.461081402:	3.797115280!
MF	GO:004:	alcohol l	8/408	92/1863!	0.086956!	3.972506393(	4.275671833:
MF	GO:000!	ephrin re	6/408	53/1863!	0.113207!	5.171753607:	4.549756019:
MF	GO:000!	transmer	6/408	53/1863!	0.113207!	5.171753607:	4.549756019:
MF	GO:003(	collagen	6/408	53/1863!	0.113207!	5.171753607:	4.549756019:
MF	GO:003!	chondroi	3/408	10/1863!	0.3	13.70514705(	6.011867698‹
MF	GO:009(	cell adhe	7/408	76/1863!	0.092105:	4.207720588:	4.191830710(
MF	GO:004(	activin b	3/408	11/1863!	0.272727:	12.45922459(	5.687127296:
MF	GO:000!	hyaluron	4/408	24/1863!	0.166666(	7.613970588:	4.850213427‹
MF	GO:000:	phospha	6/408	59/1863!	0.101694!	4.645812562:	4.195869552(
MF	GO:000:	lipopoly	5/408	41/1863!	0.121951:	5.571197991:	4.383422460:
MF	GO:004:	MHC pr	5/408	41/1863!	0.121951:	5.571197991:	4.383422460:
MF	GO:007(	serine-ty	3/408	12/1863!	0.25	11.42095588:	5.4019581181
MF	GO:001!	immuno	4/408	26/1863!	0.153846:	7.028280542!	4.601468255:
MF	GO:000(	metallop	11/408	184/186:	0.059782(	2.731098145:	3.530189896‹

MF	GO:0005	vascular	3/408	13/18639	0.2307692	10.5424208	5.148668510
MF	GO:0023	MHC cla	4/408	27/18639	0.148148	6.7679738562	4.486763033
MF	GO:0046	sphingol	4/408	28/18639	0.142857	6.5262605042	4.377740398
MF	GO:0033	amide bi	13/408	247/1863	0.052631	2.404411764	3.323938241
MF	GO:0072	modified	7/408	91/18639	0.076923	3.514140271	3.596550368
MF	GO:0048	receptor	4/408	30/18639	0.133333	6.091176470	4.174858399
MF	GO:0005	GPI-link	5/408	49/18639	0.102040	4.661614645	3.839330255
MF	GO:0005	hepatocy	5/408	49/18639	0.102040	4.661614645	3.839330255
MF	GO:0005	insulin r	5/408	49/18639	0.102040	4.661614645	3.839330255
MF	GO:0005	macroph	5/408	49/18639	0.102040	4.661614645	3.839330255
MF	GO:0005	platelet-	5/408	49/18639	0.102040	4.661614645	3.839330255
MF	GO:0005	stem cell	5/408	49/18639	0.102040	4.661614645	3.839330255
MF	GO:0008	boss rec	5/408	49/18639	0.102040	4.661614645	3.839330255
MF	GO:0036	placenta	5/408	49/18639	0.102040	4.661614645	3.839330255
MF	GO:0038	protein t	5/408	49/18639	0.102040	4.661614645	3.839330255
MF	GO:0060	brain-de	5/408	49/18639	0.102040	4.661614645	3.839330255
MF	GO:0097	scaffold	6/408	70/18639	0.085714	3.915756302	3.656204464
MF	GO:0005	fibroblas	5/408	50/18639	0.1	4.568382352	3.779661101
MF	GO:0005	platelet-	5/408	50/18639	0.1	4.568382352	3.779661101
MF	GO:0005	platelet-	5/408	50/18639	0.1	4.568382352	3.779661101
MF	GO:0005	long-cha	3/408	16/18639	0.1875	8.565716911	4.529077440
MF	GO:0005	insulin-l	5/408	51/18639	0.098039	4.478806228	3.721546800
MF	GO:0005	vascular	5/408	51/18639	0.098039	4.478806228	3.721546800
MF	GO:0016	intramol	5/408	53/18639	0.094339	4.309794672	3.609695023
MF	GO:0016	endonuc	3/408	18/18639	0.166666	7.613970588	4.199731266
MF	GO:0003	protein c	3/408	19/18639	0.157894	7.213235294	4.053491367
MF	GO:0015	fatty aci	3/408	19/18639	0.157894	7.213235294	4.053491367
MF	GO:0016	intramol	3/408	19/18639	0.157894	7.213235294	4.053491367
MF	GO:0034	immuno	3/408	19/18639	0.157894	7.213235294	4.053491367
MF	GO:0042	peptide l	12/408	249/1863	0.048192	2.201630049	2.855644092
MF	GO:0005	retinoid	4/408	38/18639	0.105263	4.808823529	3.515920067
MF	GO:0019	isopreno	4/408	38/18639	0.105263	4.808823529	3.515920067

KEGG enrichment analysis

category	subcateg	ID	Descript	GeneRat	BgRatio	RichFactor	FoldEnrichme
Environ	Signalin	hsa0406	Cytokin	53/253	298/9521	0.177852348	6.693012706
Organisr	Immune	hsa0461	Comple	22/253	88/9521	0.25	9.408102766
Environ	Signalin	hsa0406	Viral pro	22/253	100/9521	0.22	8.279130434

Human I	Immune	hsa0532	Rheuma	16/253	95/9521	0.1684210526	6.3380902850
Organisr	Develop	hsa0438	Osteocla	16/253	143/9521	0.1118881118	4.2106194201
Cellular	Transpoı	hsa0414	Lysoson	15/253	133/9521	0.1127819548	4.2442568872
Environı	Signal tr	hsa0466	TNF sig	14/253	119/9521	0.1176470588	4.4273424784
Environı	Signal tr	hsa0406	NF-kapp	13/253	105/9521	0.1238095238	4.6592508940
Human I	Infectiou	hsa0514	African	8/253	37/9521	0.2162162162	8.1367375280
Environı	Signal tr	hsa0401	MAPK s	22/253	300/9521	0.0733333333	2.7597101449
Human I	Infectiou	hsa0513	Pertussis	10/253	78/9521	0.1282051282	4.8246680855
Human I	Infectiou	hsa0514	Malaria	8/253	50/9521	0.16	6.0211857707
Organisr	Immune	hsa0464	Hemato	11/253	100/9521	0.11	4.1395652173
Human I	Infectiou	hsa0515	Staphylc	11/253	102/9521	0.1078431372	4.0583972719
Environı	Signal tr	hsa0415	PI3K-Al	23/253	362/9521	0.0635359116	2.3910095429
Human I	Immune	hsa0533	Graft-ve	7/253	45/9521	0.1555555555	5.8539306104
Organisr	Immune	hsa0467	Intestina	7/253	50/9521	0.14	5.2685375494
Human I	Cardiova	hsa0541	Lipid an	15/253	216/9521	0.0694444444	2.6133618796
Metabol	Energy r	hsa0091	Nitrogen	4/253	17/9521	0.2352941176	8.8546849569
Human I	Endocrir	hsa0494	Type I d	6/253	44/9521	0.1363636363	5.1316924182
Human I	Drug res	hsa0152	Antifola	5/253	30/9521	0.1666666666	6.2720685111
Cellular	Transpoı	hsa0414	Phagoso	12/253	159/9521	0.0754716981	2.8401819673
Human I	Infectiou	hsa0515	Tubercu	13/253	182/9521	0.0714285714	2.6880293619
Human I	Infectiou	hsa0516	Herpes s	13/253	183/9521	0.0710382513	2.6733406769
Human I	Cardiova	hsa0541	Fluid sh	11/253	142/9521	0.0774647887	2.9151867728
Human I	Immune	hsa0532	Inflamm	7/253	66/9521	0.1060606060	3.9913163253
Human I	Infectiou	hsa0516	Influenz	12/253	173/9521	0.0693641618	2.6103406520
NA	NA	hsa0451	Integrin	11/253	154/9521	0.0714285714	2.6880293619
Human I	Immune	hsa0533	Allograf	5/253	39/9521	0.1282051282	4.8246680855
Environı	Signalin	hsa0451	Cell adh	11/253	160/9521	0.06875	2.5872282608
Organisr	Immune	hsa0465	IL-17 sig	8/253	95/9521	0.0842105263	3.1690451425
Environı	Signal tr	hsa0401	Ras sign	14/253	238/9521	0.0588235294	2.2136712392
Human I	Endocrir	hsa0493	Alcoholi	10/253	144/9521	0.0694444444	2.6133618796
Human I	Endocrir	hsa0493	AGE-RA	8/253	101/9521	0.0792079207	2.9807850350
Organisr	Immune	hsa0461	Antigen	7/253	82/9521	0.0853658536	3.2125228959
Human I	Infectiou	hsa0514	Chagas	8/253	103/9521	0.0776699029	2.9229057139
Organisr	Immune	hsa0465	Th17 cel	8/253	109/9521	0.0733944954	2.7620118214
Organisr	Immune	hsa0466	B cell re	7/253	91/9521	0.0769230769	2.8948008513
Metabol	Glycan b	hsa0053	Glycosa	3/253	19/9521	0.1578947368	5.9419596421
Environı	Signal tr	hsa0463	JAK-ST	10/253	168/9521	0.0595238095	2.2400244682
Organisr	Endocrir	hsa0332	PPAR si	6/253	76/9521	0.0789473684	2.9709798210

Human I	Infectiou	hsa0513	Legionel	5/253	56/9521	0.0892857142	3.360036702
Human I	Immune	hsa0534	Primary	4/253	38/9521	0.1052631578	3.961306428
Metabol	Xenobio	hsa0098	Metabol	6/253	79/9521	0.0759493670	2.858157802
Metabol	Carbohy	hsa0050	Starch ar	4/253	40/9521	0.1	3.763241106
Organisr	Immune	hsa0462	C-type le	7/253	105/9521	0.0666666666	2.508827404
Human I	Cancer:	hsa0521	Prostate	7/253	106/9521	0.0660377358	2.485159221
Human I	Infectiou	hsa0517	Coronav	12/253	238/9521	0.0504201680	1.897432490
Metabol	Lipid me	hsa0059	Arachido	5/253	63/9521	0.0793650793	2.986699291
Organisr	Immune	hsa0462	Toll-like	7/253	109/9521	0.0642201834	2.416760343
Environ	Signal tr	hsa0401	Rap1 sig	11/253	212/9521	0.0518867924	1.952625102
Cellular	Cell grov	hsa0421	Apoptos	8/253	137/9521	0.0583941605	2.197513055
Environ	Signalin	hsa0451	ECM-rec	6/253	89/9521	0.0674157303	2.537016476
Human I	Infectiou	hsa0516	Human T	11/253	224/9521	0.0491071428	1.848020186
Organisr	Immune	hsa0465	Th1 and	6/253	93/9521	0.0645161290	2.427897488
Genetic	Informat	hsa0326	Virion -	2/253	12/9521	0.1666666666	6.272068511
Cellular	Cellular	hsa0451	Focal ad	10/253	203/9521	0.0492610837	1.853813353
Human I	Infectiou	hsa0513	Pathoger	10/253	203/9521	0.0492610837	1.853813353
Metabol	Xenobio	hsa0098	Drug me	5/253	73/9521	0.0684931506	2.577562401
Organisr	Digestiv	hsa0497	Choleste	4/253	51/9521	0.0784313725	2.951561652
Human I	Drug res	hsa0152	Platinum	5/253	75/9521	0.0666666666	2.508827404

pvalue	p.adjust	qvalue	geneID	Count
1.005458042(	4.00976(	2.92429!	IGHG4/'	43
3.2179564981	6.41660!	4.67958(	PLAUR/	45
5.664744274·	7.53033!	5.49182(	IGHG4/'	37
1.357256873(	1.08663!	7.92473!	CCL21/(	38
1.362377136!	1.08663!	7.92473!	CCL21/(	39
1.757476767!	1.16813(	8.51913'	TREM1/	39
2.096469277!	1.19438!	8.71059!	CXCL17	25
7.132824061(	3.55571!	2.59315(	CCL21/(	40
1.149103893'	5.09180'	3.71341!	PLAUR/	35
2.2135486191	8.82763!	6.43793!	IGHG4/(	17
5.037258725·	1.82623!	1.33186(	CCL21/(	37
1.153423462!	3.83321(	2.79553·	CCL21/(	31
2.138728862(	6.56096!	4.78486·	CXCL17	28
2.378255011·	6.77462!	4.94069(	TREM1/	28
2.571975846!	6.83802(	4.98692!	CCL21/(	36
4.268638812!	1.06395!	7.75937·	CCL21/(	29
5.0592957791	1.12091!	8.17475(	PLAUR/	40
5.0592957791	1.12091!	8.17475(	PLAUR/	40
6.429190633·	1.34945!	9.84147(	SCGB1/	33
7.374157000!	1.47040(	1.07235'	SCGB1/	28
1.042385849!	1.97424!	1.43980!	PLAUR/	36
1.112430007!	1.97424!	1.43980!	PLAUR/	37
1.138606714(	1.97424!	1.43980!	TREM1/	27
1.338047437(	2.18161(	1.59103!	SCGB1/	29
1.367609179'	2.18161(	1.59103!	IGHG4/(	35
1.922932776(	2.94948!	2.15103'	CCL21/(	32
2.808204239!	4.14782!	3.02497'	SCGB1/	27
3.839980289(	5.46922!	3.98867!	TREM1/	31
5.082493646!	6.98930!	5.09725!	SCGB1/	30
5.848938305!	7.77518!	5.67039!	HAVCR	20
6.641356364!	8.54378!	6.23092!	CXCL17	37
7.538601394!	9.39498!	6.85169!	TREM1/	37
8.251866156!	9.97225!	7.27269'	SCGB1/	25
8.550725203!	1.00294!	7.31444!	TREM1/	37
1.062675381!	1.21084!	8.83059!	SCGB1/	23

1.2406652043	1.374381	1.002320	CD27/FS	31
1.4030928143	1.512300	1.102914	SCGB1A	29
1.6224004493	1.702660	1.241743	LBP/IL6	13
3.5423187270	3.531691	2.575638	CD27/IL	23
3.5423187270	3.531691	2.575638	CD27/IL	23
2.0406448748	1.984900	1.447574	CXCL17	16
2.0993016578	1.993330	1.453720	PLAUR/	34
2.1954570778	2.036158	1.484950	LBP/IL6	17
3.4834744890	3.157294	2.302593	IGHG4/T	35
3.7463203333	3.320072	2.421305	CD27/FS	33
4.0765766892	3.506705	2.557410	TREM1/	23
4.1327777830	3.506705	2.557410	ICAM1/	32
4.5953402370	3.817961	2.784413	TNFRSF	24
4.8362871722	3.936145	2.870603	TREM1/	19
5.2409380500	4.161331	3.034830	IGHG4/V	21
5.3216628800	4.161331	3.034830	CXCL17	33
7.0941222048	5.440640	3.967825	CD27/IL	33
9.8262208519	7.393767	5.392224	CXCL17	23
1.1955439593	8.829313	6.439157	CD27/IL	32
1.3563358754	9.834668	7.172350	CCL21/T	22
1.6516759820	1.176229	8.578159	CXCL17	15
1.8319975070	1.281755	9.347754	EFEMP1	21
1.8783950873	1.291558	9.419247	CXCL17	17
2.0083553520	1.357513	9.900243	EFEMP1	21
2.0984793852	1.371923	1.000534	HAVCR	22
2.0984793852	1.371923	1.000534	TNFRSF	22
2.4161930041	1.554157	1.133430	CXCL17	18
3.0500685807	1.930741	1.408070	ICAM1/	17
3.1523281261	1.964294	1.432540	HAVCR	21
3.9989612040	2.453510	1.789333	CCL21/A	26
4.7618878485	2.877334	2.098420	IGHG4/C	29
5.4511501359	3.244654	2.366304	ICAM1/	17
6.3045772670	3.697449	2.696524	SCGB1A	13
7.6359498140	4.413357	3.218631	TREM1/	16
8.2161958528	4.680884	3.413730	SCGB1A	14
1.0973252720	6.163567	4.495047	SCGB1A	14
1.3738591075	7.609652	5.549667	TREM1/	16
1.4160045318	7.680808	5.601560	TNFRSF	18

1.425222519	7.680808	5.601560	SCGB1A	28
1.591726590	8.463740	6.172548	SCGB1A	27
1.927784229	1.011579	7.377379	FSTL3/I	16
1.982078455	1.026562	7.486647	CXCL17	17
2.564150777	1.311004	9.561064	CCL21/T	25
2.608980048	1.317039	9.605079	CCL21/S	20
2.736039665	1.363915	9.946944	LILRA5	15
2.853297925	1.396551	1.018495	SCGB1A	14
2.871545801	1.396551	1.018495	TREM1/	21
3.248711697	1.560947	1.138388	PLAUR/	14
3.399912214	1.614148	1.177187	TIMP1/A	19
3.515561465	1.649418	1.202909	CCL21/T	24
3.689255983	1.696639	1.237347	CCL21/I	16
3.704678817	1.696639	1.237347	CD27/IL	25
3.743839371	1.696639	1.237347	PLAUR/	10
3.977987933	1.782490	1.299962	PLAUR/	30
4.364973138	1.934168	1.410575	LILRA5	17
4.707859747	2.063180	1.504663	ICAM1/	18
4.899687549	2.123908	1.548951	CXCL17	20
5.116143067	2.193890	1.599989	CFB/CF	7
5.583185327	2.368690	1.727473	CXCL17	19
5.876737424	2.466992	1.799160	MSR1/P	31
7.252752136	3.012914	2.197297	PLAUR/	18
8.417769970	3.460831	2.523960	SPON2/	15
8.994176428	3.660079	2.669270	SCGB1A	19
1.033245626	4.162205	3.035468	SCGB1A	20
1.119599045	4.422288	3.225144	OSCAR/	21
1.119987898	4.422288	3.225144	CXCL17	24
1.156375118	4.521199	3.297280	CXCL17	23
1.198105388	4.638877	3.383101	HAVCR	21
1.295628261	4.942242	3.604343	TIMP1/S	10
1.301242324	4.942242	3.604343	HAVCR	17
1.324578839	4.983415	3.634370	IGHG4/	9
1.471568020	5.484685	3.999943	HAVCR	16
1.886457565	6.965919	5.080197	IGHG4/	10
1.992271938	7.289150	5.315931	SCGB1A	17
2.079628271	7.539597	5.498570	TNFRSF	9
2.319121135	8.288984	6.045099	CCL21/S	16

2.327899278	8.28898	6.04509	SCGB1	15
2.563750930	9.04799	6.59864	CD27/IL	15
2.621841801	9.11675	6.64878	SPON2/	18
2.628953307	9.11675	6.64878	TREM1/	21
2.769685275	9.44060	6.88496	HAVCR	17
2.769685275	9.44060	6.88496	HAVCR	17
3.319711412	1.12194	8.18230	TIMP1/	12
3.434569163	1.15101	8.39426	MMP12/	24
3.602196624	1.19429	8.70991	CD27/IL	14
3.623616344	1.19429	8.70991	MMP12/	24
3.770494151	1.23251	8.98867	IL6/SFR	12
3.822218095	1.23926	9.03790	MMP12/	24
3.888984904	1.25074	9.12161	HAVCR	13
3.966436453	1.25540	9.15560	CD74/C	6
3.966436453	1.25540	9.15560	CD74/C	6
4.066775150	1.27703	9.31330	SPON2/	21
4.128702121	1.28634	9.38125	FSTL3/I	11
4.407807488	1.36266	9.93779	CCL21/	14
5.412704948	1.66045	1.21095	TREM1/	13
5.456547246	1.66112	1.21144	TIMP1/	16
5.781111307	1.74659	1.27378	ICAM1/	9
6.422098618	1.92566	1.40437	HAVCR	15
7.885441676	2.33946	1.70615	IL6/CLE	14
7.919437354	2.33946	1.70615	MSR1/P	28
8.199816374	2.40447	1.75356	HAVCR	11
8.782124119	2.55643	1.86438	CD27/IL	17
9.146100665	2.64309	1.92758	ICAM1/	20
9.214847255	2.64379	1.92810	TNFRSI	19
9.457196128	2.69394	1.96467	TNFRSI	6
9.776618791	2.76518	2.01663	NET1/T	23
9.849336780	2.76613	2.01732	FSTL3/I	21
1.004268373	2.80071	2.04254	TNFRSI	9
1.041463038	2.88427	2.10348	TNFRSI	14
1.144088459	3.12508	2.27910	MMP12/	10
1.144088459	3.12508	2.27910	LBP/IL6	10
1.171657086	3.17861	2.31814	ACVRL	24
1.196612108	3.22438	2.35152	TNFRSI	9
1.239019110	3.31624	2.41851	IL6/IL1I	13

1.322936628	3.46785	2.52907	LILRA5	10
1.322936628	3.46785	2.52907	LILRA5	10
1.322936628	3.46785	2.52907	CD27/H	10
1.330443997	3.46785	2.52907	FSTL3/I	18
1.364695966	3.53403	2.57734	IL6/CLE	14
1.392998703	3.58405	2.61382	IL6/TRE	6
1.507082258	3.85272	2.80976	TNFRSF	13
1.530487406	3.88763	2.83522	TIMP1/S	12
1.549711572	3.91155	2.85266	HAVCR	17
1.581545658	3.94200	2.87487	IL6/TNF	7
1.581545658	3.94200	2.87487	TIMP1/T	7
1.801905978	4.46335	3.25509	ACVRL	24
1.837993406	4.52464	3.29978	CD27/IL	18
1.894080641	4.63410	3.37962	LILRA5	12
1.934196698	4.67349	3.40835	ICAM1/	14
1.934196698	4.67349	3.40835	PLAUR/	14
1.945337681	4.67349	3.40835	SPINT1/	27
1.976284854	4.71941	3.44183	CXCL17	9
2.006148042	4.76221	3.47304	PLA2G2	13
2.046547636	4.82936	3.52202	SPON2/	7
2.582688204	6.00141	4.37679	HAVCR	12
2.582688204	6.00141	4.37679	HAVCR	12
2.588375079	6.00141	4.37679	CCL21/	8
2.646913004	6.06660	4.42433	SCGB1A	13
2.646913004	6.06660	4.42433	SCGB1A	13
2.856890701	6.51044	4.74802	LILRB4	12
3.156445436	7.15221	5.21606	TREM1/	12
3.312982774	7.46450	5.44381	MSR1/IL	16
3.855663695	8.63841	6.29994	CD74/C	6
3.888843863	8.66408	6.31865	TPP1/IL	11
5.108734775	0.00011	8.25463	ACVRL	12
5.606865131	0.00012	9.00946	OSCAR/	12
5.646120895	0.00012	9.02269	CD27/IL	9
5.755739247	0.00012	9.14760	TNFRSF	15
5.858552582	0.00012	9.26040	ICAM1/	21
6.147171801	0.00013	9.66408	IL2RA/C	12
6.313237957	0.00013	9.87180	ICAM1/	20
6.952452740	0.00014	0.00010	PLAUR/	25

7.053101427	0.00014	0.00010	IGHG4/	10
7.097826832	0.00014	0.00010	ICAM1/	21
7.396289855	0.00015	0.00011	IL6/TNF	8
7.396289855	0.00015	0.00011	CCL21/I	8
7.526585499	0.00015	0.00011	ICAM1/	5
7.526585499	0.00015	0.00011	TNFSF1	5
7.891824456	0.00016	0.00011	CTSD/C	7
8.023282022	0.00016	0.00011	ICAM1/	13
8.030900124	0.00016	0.00011	CCL21/I	14
8.052192902	0.00016	0.00011	CCL21/I	12
8.290228225	0.00016	0.00012	ANGPT	16
8.472882787	0.00016	0.00012	CSF1/CI	9
8.583204917	0.00017	0.00012	PLAUR/	17
8.675715660	0.00017	0.00012	TIMP1/I	8
8.675715660	0.00017	0.00012	CCL21/I	8
9.056355365	0.00017	0.00012	FABP3/	6
1.061162845	0.00020	0.00015	ICAM1/	19
1.080207580	0.00021	0.00015	ICAM1/	17
1.096563864	0.00021	0.00015	TIMP1/S	9
1.098476029	0.00021	0.00015	SPON2/	13
1.103583878	0.00021	0.00015	CCL21/	10
1.109155642	0.00021	0.00015	TNFSF1	5
1.164463495	0.00022	0.00016	CTSD/C	7
1.170191569	0.00022	0.00016	CCL21/	6
1.170191569	0.00022	0.00016	CD74/C	6
1.180026122	0.00022	0.00016	IL6/SFR	8
1.243045967	0.00023	0.00016	HAVCR	9
1.243045967	0.00023	0.00016	HAVCR	9
1.346658024	0.00024	0.00018	IL2RA/	12
1.348490352	0.00024	0.00018	TIMP1/	16
1.366794418	0.00024	0.00018	SPON2/	10
1.366794418	0.00024	0.00018	CD27/IL	10
1.379907545	0.00025	0.00018	TFF2/SE	13
1.517526511	0.00027	0.00019	TNFRSF	10
1.553026682	0.00027	0.00020	TIMP1/	15
1.566326653	0.00027	0.00020	LBP/IL6	11
1.566326653	0.00027	0.00020	LBP/IL6	11
1.758674300	0.00031	0.00022	ACVRL	15

1.786646823	0.00031	0.00022	CCL21/	9
1.862315314	0.00032	0.00023	PLTP/F	10
1.874788161	0.00032	0.00023	PLAUR	17
1.874909245	0.00032	0.00023	CD27/H	11
1.886199967	0.00032	0.00023	PLAUR	6
1.995129010	0.00034	0.00025	LILRB4	7
1.995129010	0.00034	0.00025	LILRB4	7
2.048026000	0.00035	0.00025	PGF/EP	11
2.112392688	0.00036	0.00026	ACVRL	15
2.139916416	0.00036	0.00026	TIMP1/	16
2.2435394111	0.00037	0.00027	HAVCR	15
2.2435394111	0.00037	0.00027	HAVCR	15
2.251770465	0.00037	0.00027	ANGPT	9
2.357485077	0.00039	0.00028	CD74/C	6
2.440660372	0.00040	0.00029	PLAUR	17
2.520477058	0.00041	0.00030	HAVCR	9
2.526779479	0.00041	0.00030	TFF2/T	18
2.526779479	0.00041	0.00030	TFF2/T	18
2.547463207	0.00041	0.00030	HAVCR	12
2.547463207	0.00041	0.00030	HAVCR	12
2.594342220	0.00042	0.00030	TIMP1/	26
2.652915690	0.00042	0.00031	HAVCR	18
2.672395361	0.00042	0.00031	TNFRS	16
2.706519079	0.00043	0.00031	PLAUR	17
2.757498455	0.00043	0.00031	CCL21/	10
2.757498455	0.00043	0.00031	CCL21/	10
2.782555386	0.00043	0.00031	TIMP1/	7
2.782555386	0.00043	0.00031	LILRA5	7
2.782555386	0.00043	0.00031	CD74/C	7
2.815907773	0.00044	0.00032	HAVCR	9
3.000883262	0.00046	0.00034	PLAUR	5
3.013255514	0.00046	0.00034	IL2RA/	15
3.137067197	0.00048	0.00035	MSR1/P	11
3.193858599	0.00049	0.00035	HAVCR	12
3.406444383	0.00052	0.00038	HAVCR	11
3.495537056	0.00053	0.00038	PLAUR	9
3.495537056	0.00053	0.00038	CCL21/	9
3.539177785	0.00053	0.00039	CCL21/	8

3.583354011	0.00054	0.00039	HAVCR	6
3.614421049	0.00054	0.00039	PLAUR/	22
3.650475312	0.00054	0.00039	CCL21/	10
3.704598512	0.00055	0.00040	CCL21/	18
3.756819688	0.00055	0.00040	SCGB1/	22
3.939637879	0.00058	0.00042	TNFSF1	4
3.956302486	0.00058	0.00042	CCL21/	13
4.001226528	0.00058	0.00042	HAVCR	5
4.001226528	0.00058	0.00042	TNFSF1	5
4.292218050	0.00062	0.00045	ICAM1/	23
4.309103829	0.00062	0.00045	PLAUR/	9
4.363612171	0.00063	0.00046	CTSD/C	6
4.467251400	0.00064	0.00047	FABP4/	14
4.527995307	0.00065	0.00047	ADM/G	8
4.700166671	0.00067	0.00049	IL2RA/	20
5.028367881	0.00071	0.00052	PLA2G2	14
5.073435838	0.00072	0.00052	TREM1/	11
5.073435838	0.00072	0.00052	TREM1/	11
5.119204369	0.00072	0.00052	OSCAR/	24
5.128429890	0.00072	0.00052	ACE2/F	7
5.128429890	0.00072	0.00052	IL2RA/T	7
5.241483199	0.00073	0.00053	TNFSF1	5
5.274247441	0.00073	0.00053	CHI3L1/	6
5.737084206	0.00079	0.00058	HAVCR	8
5.826138265	0.00080	0.00058	IL2RA/I	9
5.864108145	0.00080	0.00059	SFTPD/	16
6.086182658	0.00083	0.00061	TNFSF1	4
6.435626482	0.00087	0.00064	TIMP1/	8
6.435626482	0.00087	0.00064	CSF1/EI	8
6.675775319	0.00090	0.00065	ACVRL	18
6.708648709	0.00090	0.00065	HAVCR	14
6.708648709	0.00090	0.00065	HAVCR	14
6.758957209	0.00090	0.00065	SCGB1/	5
6.758957209	0.00090	0.00065	SCGB1/	5
6.758957209	0.00090	0.00065	CCL21/	5
6.871430890	0.00091	0.00066	TREM1/	11
7.397955903	0.00098	0.00071	TNFRSI	11
7.549462117	0.00099	0.00072	FSTL3/I	6

7.549462117	0.00099	0.00072	FABP3/	6
7.770185378	0.00102	0.00074	TREM1/	9
7.788478988	0.00102	0.00074	HAVCR	7
7.840702043	0.00102	0.00074	ACVRL	17
7.918986328	0.00103	0.00075	ACVRL	12
8.045749007	0.00104	0.00076	LRRN1/	8
8.173099262	0.00105	0.00077	NET1/A	22
8.593765994	0.00110	0.00080	CFH/TR	5
8.593765994	0.00110	0.00080	TNFSF1	5
8.668990364	0.00111	0.00081	HAVCR	16
8.891350902	0.00112	0.00082	PLAUR/	7
8.891350902	0.00112	0.00082	CCL21/	7
8.948140670	0.00112	0.00082	CCL21/	6
8.968149420	0.00112	0.00082	TPP1/IL	8
8.968149420	0.00112	0.00082	RARRE	8
8.975020874	0.00112	0.00082	IL10RB/	4
0.000101181	0.00126	0.00092	PLAUR/	7
0.000102324	0.00127	0.00093	TNFRSF	9
0.000103716	0.00129	0.00094	CCL21/	15
0.000105454	0.00131	0.00095	CCL21/	6
0.000110767	0.00137	0.00100	PLAUR/	8
0.000111859	0.00138	0.00100	IL6/IL1	9
0.000115871	0.00142	0.00104	TIMP1/	18
0.000122754	0.00150	0.00109	CRHBP/	8
0.000123666	0.00151	0.00110	TNFRSF	12
0.000125738	0.00153	0.00111	CXCL17	18
0.000127450	0.00153	0.00112	IL2RA/I	4
0.000127450	0.00153	0.00112	TNFSF1	4
0.000127505	0.00153	0.00112	HAVCR	10
0.000127505	0.00153	0.00112	CCL21/	10
0.000135793	0.00162	0.00118	S100A1	8
0.000135793	0.00162	0.00118	CTSD/C	8
0.000135793	0.00162	0.00118	PLAUR/	8
0.000137503	0.00163	0.00119	LILRA5	10
0.000137503	0.00163	0.00119	LILRA5	10
0.000139052	0.00164	0.00120	GDF15/	11
0.000140743	0.00166	0.00121	PLAUR/	13
0.000142643	0.00167	0.00122	IGHG4/	14

0.000146476	0.00170	0.00124	CSF1/EI	7
0.000146476	0.00170	0.00124	PLAUR/	7
0.000146476	0.00170	0.00124	CXCL17	7
0.000148154	0.00172	0.00125	TFF2/SI	10
0.000148461	0.00172	0.00125	FURIN/	12
0.000150716	0.00174	0.00126	MMP12/	16
0.000150992	0.00174	0.00126	SPINT1/	17
0.000152966	0.00175	0.00128	HAVCR	15
0.000166104	0.00190	0.00138	IGHG4/	14
0.000167323	0.00191	0.00139	LBP/CL	6
0.000169726	0.00193	0.00141	NET1/A	24
0.000183535	0.00208	0.00152	TNFRSI	14
0.000184899	0.00209	0.00152	CD83/LI	7
0.000187700	0.00212	0.00154	TNFRSI	16
0.000199903	0.00224	0.00163	CD83/LI	8
0.000200041	0.00224	0.00163	LBP/CL	5
0.000201713	0.00225	0.00164	PGF/EPI	9
0.000202181	0.00225	0.00164	EFNA4/	15
0.000206952	0.00230	0.00168	FSTL3/I	7
0.000211592	0.00235	0.00171	EFNA4/	15
0.000222423	0.00246	0.00179	CTSD/C	6
0.000223178	0.00246	0.00179	MMP12/	14
0.000235175	0.00258	0.00188	IL2RA/I	4
0.000235175	0.00258	0.00188	RGMA/	4
0.000236310	0.00258	0.00188	GLA/MS	9
0.000236310	0.00258	0.00188	GLA/MS	9
0.000240256	0.00261	0.00190	CCL21/	13
0.000241218	0.00261	0.00190	PLAUR/	5
0.000241218	0.00261	0.00190	ANGPT	5
0.000254855	0.00275	0.00200	CD27/IL	6
0.000261202	0.00281	0.00205	FSTL3/I	10
0.000262763	0.00282	0.00205	ACE2/F	8
0.000286128	0.00305	0.00223	CSF1/EI	7
0.000286128	0.00305	0.00223	PLAUR/	7
0.000288532	0.00306	0.00223	IL6/IL18	5
0.000288532	0.00306	0.00223	CLSTN3	5
0.000288532	0.00306	0.00223	CXCL17	5
0.000290892	0.00306	0.00223	IL6/TNI	6

0.000290892	0.00306	0.00223	IL2RA/T	6
0.000304272	0.00320	0.00233	PLA2G2	19
0.000308283	0.00323	0.00235	RGMA/T	4
0.000313017	0.00327	0.00238	HAVCR	8
0.000314984	0.00327	0.00239	PLA2G2	19
0.000314984	0.00327	0.00239	CCL21/	19
0.000317349	0.00328	0.00239	CSF1/IL	7
0.000317349	0.00328	0.00239	CD74/L	7
0.000330808	0.00341	0.00249	IL6/TRE	6
0.000333958	0.00343	0.00250	IL6/TRE	11
0.000334341	0.00343	0.00250	SCGB1A	22
0.000340139	0.00347	0.00253	TNFRSF	14
0.000340501	0.00347	0.00253	ACVRL	10
0.000340936	0.00347	0.00253	CD83/LI	8
0.000351244	0.00356	0.00259	LILRA5	7
0.000351244	0.00356	0.00259	CD27/C	7
0.000358439	0.00362	0.00264	CRHBP/	13
0.000370851	0.00374	0.00273	HAVCR	8
0.000376163	0.00377	0.00275	ACVRL	23
0.000376209	0.00377	0.00275	MMP12/	13
0.000384633	0.00385	0.00281	ACVRL	12
0.000388406	0.00388	0.00283	TNFRSF	20
0.000396352	0.00393	0.00286	SPON2/	4
0.000396352	0.00393	0.00286	TNFRSF	4
0.000396352	0.00393	0.00286	TNFRSF	4
0.000399934	0.00395	0.00288	EFNA4/	22
0.000403907	0.00397	0.00290	TNFSF1	5
0.000403907	0.00397	0.00290	CSF1/TI	5
0.000412357	0.00405	0.00295	FSTL3/I	10
0.000454983	0.00445	0.00325	MMP12/	13
0.000456345	0.00446	0.00325	SPINT1/	19
0.000458741	0.00447	0.00326	EPHB4/	9
0.000465769	0.00453	0.00330	GDF15/	18
0.000470649	0.00455	0.00331	MMP7/T	7
0.000470649	0.00455	0.00331	GLA/CL	7
0.000473190	0.00455	0.00331	HAVCR	5
0.000473190	0.00455	0.00331	ANGPT	5
0.000473647	0.00455	0.00331	IL2RA/C	8

0.000476736(	0.00457(	0.00333.	CFH/TR	6
0.000484829	0.00463(	0.00338	LRRN1/	17
0.000491585.	0.00469(	0.00342(	CSF1/CI	9
0.000496330!	0.00472	0.00344!	CD27/IL	16
0.000501022!	0.00474(	0.00346	PLAUR/	4
0.000501022!	0.00474(	0.00346	TNFSF1	4
0.000516943	0.00485(	0.00353	ICAM1/	7
0.000516943	0.00485(	0.00353	ANGPT.	7
0.000516943	0.00485(	0.00353	PLG/PL.	7
0.000516943	0.00485(	0.00353	PLAUR/	7
0.000524432	0.00490!	0.00358(	IL6/TRE	11
0.000528159	0.00493.	0.00359	GDF15/.	14
0.000535127	0.00496.	0.00361!	PLA2G2	6
0.000535127	0.00496.	0.00361!	CCL21/I	6
0.000535127	0.00496.	0.00361!	CD83/T(	6
0.000551051	0.00506.	0.00369.	CD74/C	5
0.000551051	0.00506.	0.00369.	ACVRL	5
0.000551051	0.00506.	0.00369.	CXCL17	5
0.000551051	0.00506.	0.00369.	CD74/C	5
0.000563073	0.00516(	0.00376.	S100A1.	9
0.000566791	0.00516(	0.00376.	HAVCR	7
0.000566791	0.00516(	0.00376.	SPON2/(	7
0.000566791	0.00516(	0.00376.	GLA/CL	7
0.000594179(	0.00539	0.00393(	IL6/TRE	10
0.000598929.	0.00541.	0.00394	PLTP/F(	6
0.000598929.	0.00541.	0.00394	IL2RA/I	6
0.000599836!	0.00541.	0.00394	LRRN1/	17
0.000601886(	0.00541	0.00395	ICAM1/	9
0.000616056.	0.00552!	0.00403.	PLAUR/	11
0.000623958(	0.00552!	0.00403.	HAVCR	4
0.000623958(	0.00552!	0.00403.	MSR1/C	4
0.000623958(	0.00552!	0.00403.	TNFRSI	4
0.000623958(	0.00552!	0.00403.	IL6/IL18	4
0.000623958(	0.00552!	0.00403.	TNFSF1	4
0.000623958(	0.00552!	0.00403.	CCL21/(	4
0.000630110.	0.00557	0.00406.	IL2RA/(	10
0.000643049.	0.00567.	0.00413	TPP1/FU	17
0.000645464	0.00568.	0.00414	TNFRSI	8

0.00065138	0.00572	0.00417	TGFBR2	14
0.00066847	0.00585	0.00427	CTSD/C	6
0.00072642	0.00635	0.00463	TNFRSF	22
0.00073165	0.00638	0.00465	HAVCR	9
0.00073515	0.00638	0.00465	STAB2/	5
0.00073515	0.00638	0.00465	ACVRL	5
0.00076293	0.00652	0.00476	MMP7/C	17
0.00076684	0.00652	0.00476	ACVRL	4
0.00076684	0.00652	0.00476	CCL21/	4
0.00076684	0.00652	0.00476	CSF1/C	4
0.00076684	0.00652	0.00476	TNFSF1	4
0.00076684	0.00652	0.00476	TNFSF1	4
0.00076684	0.00652	0.00476	CLEC7A	4
0.00076684	0.00652	0.00476	CLEC7A	4
0.00076776	0.00652	0.00476	ACVRL	12
0.00076776	0.00652	0.00476	ACVRL	12
0.00079227	0.00672	0.00490	CD83/L	10
0.00083021	0.00701	0.00511	TNFRSF	9
0.00083021	0.00701	0.00511	CD74/C	9
0.00083776	0.00704	0.00513	CCL21/	10
0.00083776	0.00704	0.00513	IL6/TRE	10
0.00084277	0.00704	0.00513	LILRB4	5
0.00084277	0.00704	0.00513	SCGB1A	5
0.00084277	0.00704	0.00513	SCGB1A	5
0.00086422	0.00721	0.00525	ANGPT	14
0.00087626	0.00729	0.00532	IL6/ULE	7
0.00093137	0.00767	0.00559	LBP/SE	4
0.00093137	0.00767	0.00559	FGF23/I	4
0.00093137	0.00767	0.00559	FGF23/I	4
0.00093137	0.00767	0.00559	CD27/S	4
0.00093137	0.00767	0.00559	IL6/TRE	4
0.00096169	0.00788	0.00574	CSF1/C	5
0.00096169	0.00788	0.00574	CD83/C	5
0.00096247	0.00788	0.00574	GDF15/	20
0.00098730	0.00806	0.00588	CD74/C	10
0.00104175	0.00849	0.00619	GLA/SL	10
0.00104800	0.00849	0.00619	TNFRSF	3
0.00104800	0.00849	0.00619	ICAM1/	3

0.00104800041	0.00849	0.00619	CSF1/CI	3
0.00105576911	0.00854	0.00622	IL6/S10	12
0.00108231431	0.00873	0.00637	TNFRSF	18
0.00111925021	0.00898	0.00654	IL6/SFR	4
0.00111925021	0.00898	0.00654	ADM/U	4
0.00111925021	0.00898	0.00654	TNFRSF	4
0.00112449511	0.00900	0.00656	S100A1	9
0.00113917951	0.00908	0.00662	CLEC4I	8
0.00113917951	0.00908	0.00662	CCL21/C	8
0.00120889971	0.00960	0.00700	HAVCR	7
0.00120889971	0.00960	0.00700	CCL21/I	7
0.00121774001	0.00965	0.00704	CHI3L1/	8
0.00122640501	0.00968	0.00706	CCL21/C	6
0.00122640501	0.00968	0.00706	ACVRL	6
0.00123172691	0.00970	0.00707	MMP12/	13
0.00123635221	0.00972	0.00709	CD83/C	5
0.00126358731	0.00991	0.00723	PGF/EP	9
0.00127375851	0.00997	0.00727	PLA2G2	21
0.00133216861	0.01033	0.00753	IL6/TRE	4
0.00133216861	0.01033	0.00753	CTSZ/F	4
0.00133216861	0.01033	0.00753	GLA/GM	4
0.00133216861	0.01033	0.00753	CLEC4I	4
0.00133216861	0.01033	0.00753	LILRB4	4
0.00137618891	0.01065	0.00777	CCL21/C	16
0.00138779391	0.01070	0.00780	ICAM1/	8
0.00138779391	0.01070	0.00780	FSTL3/I	8
0.00139356241	0.01070	0.00780	MMP12/	5
0.00139356241	0.01070	0.00780	CD27/C	5
0.00140859771	0.01073	0.00782	SPON2/	7
0.00141623691	0.01073	0.00782	ACVRL	9
0.00141818241	0.01073	0.00782	LILRB4	3
0.00141818241	0.01073	0.00782	LILRB4	3
0.00141818241	0.01073	0.00782	IL7R/AI	3
0.00141818241	0.01073	0.00782	LBP/TR	3
0.00141818241	0.01073	0.00782	LBP/TR	3
0.00141818241	0.01073	0.00782	SFRP1/T	3
0.00145252281	0.01097	0.00800	SPINT1/	17
0.00147502441	0.01109	0.00809	MMP12/	6

0.001475024	0.011098	0.00809	TNFRSF	6
0.001489902	0.011189	0.008160	ACVRL	11
0.001497938	0.011228	0.008189	CCL21/	9
0.001571823	0.011608	0.008465	TFF2/SE	4
0.001571823	0.011608	0.008465	IL1RN/T	4
0.001571823	0.011608	0.008465	ACVRL	4
0.001571823	0.011608	0.008465	LILRB4	4
0.001571823	0.011608	0.008465	TGFBR2	4
0.001571823	0.011608	0.008465	IL6/GFA	4
0.001571823	0.011608	0.008465	IL6/CPE	4
0.001571823	0.011608	0.008465	IL6/CPE	4
0.001583386	0.01167	0.00851	CCL21/	9
0.001612866	0.01186	0.00865	IL6/TRE	6
0.001630452	0.01197	0.00873	IL6/ACE	11
0.001633154	0.01197	0.00873	GLA/CL	7
0.001651374	0.01208	0.00881	FABP4/	10
0.001686040	0.01231	0.00898	GDF15/	18
0.001733994	0.01264	0.00921	TREM1/	10
0.001755391	0.01274	0.00929	PLG/PL	7
0.001756190	0.01274	0.00929	CXCL17	12
0.001760281	0.01274	0.00929	HGF/EP	6
0.001760281	0.01274	0.00929	CD4/TR	6
0.001813978	0.01310	0.00955	ACVRL	13
0.001839895	0.01322	0.00964	CD27/IL	4
0.001839895	0.01322	0.00964	CCL21/	4
0.001839895	0.01322	0.00964	CTSZ/F	4
0.001860993	0.01332	0.00971	CCL21/	3
0.001860993	0.01332	0.00971	CCL21/	3
0.001917692	0.01370	0.00999	MMP12	6
0.001953745	0.01393	0.01016	CCL21/	5
0.001980932	0.01410	0.01028	TIMP1/	12
0.002021091	0.01434	0.01045	ANGPT	7
0.002021091	0.01434	0.01045	HGF/CS	7
0.002071185	0.01466	0.01069	GFAP/R	9
0.002073575	0.01466	0.01069	GDF15/	16
0.002085525	0.01466	0.01069	HAVCR	6
0.002085525	0.01466	0.01069	TIMP1/	6
0.002085525	0.01466	0.01069	OSCAR	6

0.002136507	0.01485	0.01083	CD83/IL	8
0.002138050	0.01485	0.01083	STAB2/	4
0.002138050	0.01485	0.01083	GLA/GN	4
0.002138050	0.01485	0.01083	IL6/TRE	4
0.002138050	0.01485	0.01083	PGF/VE	4
0.002138050	0.01485	0.01083	IL6/CPE	4
0.002138050	0.01485	0.01083	CCL21/I	4
0.002172541	0.01506	0.01098	CXCL17	5
0.002264215	0.01562	0.01139	IL1RN/I	6
0.002264215	0.01562	0.01139	CD83/L	6
0.002264215	0.01562	0.01139	CXCL17	6
0.002316887	0.01595	0.01163	EFNA4/	7
0.002381056	0.01617	0.01179	LBP/TR	3
0.002381056	0.01617	0.01179	TNFRSF	3
0.002381056	0.01617	0.01179	TNFRSF	3
0.002381056	0.01617	0.01179	TNFRSF	3
0.002381056	0.01617	0.01179	ICAM1/	3
0.002381056	0.01617	0.01179	PLG/PL	3
0.002381056	0.01617	0.01179	TNFRSF	3
0.002381056	0.01617	0.01179	CSF1/TI	3
0.002391367	0.01621	0.01182	LRRN1/	14
0.002408661	0.01628	0.01187	PIGR/IF	5
0.002408661	0.01628	0.01187	FABP1/I	5
0.002417005	0.01630	0.01189	CD27/IL	9
0.002454197	0.01650	0.01203	FUT3/G	6
0.002454197	0.01650	0.01203	CD74/C	6
0.002467934	0.01651	0.01204	PLG/PL	4
0.002467934	0.01651	0.01204	MMP7/F	4
0.002467934	0.01651	0.01204	PLG/PL	4
0.002500397	0.01670	0.01218	TNFRSF	20
0.002505206	0.01670	0.01218	CCL21/	11
0.002552890	0.01699	0.01239	GDF15/	18
0.002634136	0.01749	0.01275	ACVRL	16
0.002641175	0.01749	0.01275	CCL21/	14
0.002655913	0.01749	0.01275	HAVCR	6
0.002655913	0.01749	0.01275	CXCL17	6
0.002655913	0.01749	0.01275	PLA2G2	6
0.002662883	0.01749	0.01275	MMP12	5

0.002662883(	0.01749!	0.01275!	SCGB1/	5
0.002662883(	0.01749!	0.01275!	MMP12/	5
0.00267195755	0.01752!	0.01278:	ADM/C!	9
0.002697653:	0.01766!	0.01288:	ACVRL	12
0.002757843(	0.01802!	0.01314!	TNFRSI	20
0.002821845?	0.01829!	0.01334!	IL6/LGA	7
0.002821845?	0.01829!	0.01334!	GLA/CL	7
0.002831167{	0.01829!	0.01334!	IL6/OSN	4
0.002831167{	0.01829!	0.01334!	IL7R/EF	4
0.002831167{	0.01829!	0.01334!	NET1/E	4
0.002831167{	0.01829!	0.01334!	CCL21/I	4
0.002831225(	0.01829!	0.01334!	CTSZ/H	11
0.002869810{	0.01851!	0.01350!	ACVRL	6
0.002914072·	0.01877·	0.01369:	IGHG4/I	17
0.002935987	0.01882(	0.01372!	LILRB4	5
0.002935987	0.01882(	0.01372!	ACVRL	5
0.002947198!	0.01882(	0.01372!	HGF/TG	11
0.002982593:	0.01882(	0.01372!	CLEC6/	3
0.002982593:	0.01882(	0.01372!	CD80/IL	3
0.002982593:	0.01882(	0.01372!	IL1RAP	3
0.002982593:	0.01882(	0.01372!	TNFRSI	3
0.002982593:	0.01882(	0.01372!	TNFRSI	3
0.002982593:	0.01882(	0.01372!	TNF/IL1	3
0.002982593:	0.01882(	0.01372!	CHI3L1/	3
0.002982593:	0.01882(	0.01372!	CLEC7/	3
0.002982593:	0.01882(	0.01372!	NCAM1	3
0.002982593:	0.01882(	0.01372!	LILRB1	3
0.003007634:	0.01891{	0.01379'	IL6/LGA	7
0.003007634:	0.01891{	0.01379'	IL6/LGA	7
0.003012607(	0.01892(	0.01379{	ICAM1/	10
0.003044527(	0.01909(	0.01392:	CRELD	16
0.003099214!	0.01940:	0.01415(	HGF/IL(	13
0.003171356:	0.01982:	0.01445'	S100A1:	8
0.003202638:	0.01993!	0.01453!	GLA/CL	7
0.003222854'	0.01993!	0.01453!	NET1/T	16
0.003222854'	0.01993!	0.01453!	NET1/T	16
0.003228755	0.01993!	0.01453!	GUSB/H	5
0.003228755	0.01993!	0.01453!	EDN1/T	5

0.0032287551	0.019935	0.014539	CCL21/C	5
0.0032287551	0.019935	0.014539	CXCL17	5
0.0032293448	0.019935	0.014539	TIMP1/I	4
0.0033359456	0.020530	0.014972	NET1/C	6
0.0033359456	0.020530	0.014972	MMP12/	6
0.0034030762	0.020904	0.015245	CD27/IL	9
0.0034071502	0.020904	0.015245	CRHBP/	7
0.0035419675	0.021570	0.015731	LILRB1/	5
0.0035419675	0.021570	0.015731	CRHBP/	5
0.0035419675	0.021570	0.015731	NCAM1	5
0.0035419675	0.021570	0.015731	TGFBR2	5
0.0035427719	0.021570	0.015731	ACVRL	15
0.0035757796	0.021705	0.015829	LRRN1/	10
0.0035757796	0.021705	0.015829	FABP4/I	10
0.0036640274	0.022072	0.016096	HAVCR	4
0.0036640274	0.022072	0.016096	HAVCR	4
0.0036640274	0.022072	0.016096	BMPER/	4
0.0036694477	0.022072	0.016096	SFRP1/F	3
0.0036694477	0.022072	0.016096	OXT/EN	3
0.0036694477	0.022072	0.016096	PGF/KIT	3
0.0037188979	0.022335	0.016289	PLTP/FC	8
0.0037869503	0.022710	0.016562	MMP7/C	13
0.0038764037	0.023177	0.016902	EPHB4/	5
0.0038764037	0.023177	0.016902	ICAM1/	5
0.0039303112	0.023464	0.017112	MMP12/	20
0.0040806962	0.024325	0.017740	ADM/CS	7
0.0041367440	0.024580	0.017930	CD74/U	4
0.0041367440	0.024580	0.017930	TGFBR2	4
0.0041726237	0.024762	0.018059	NET1/SC	11
0.0042194547	0.025003	0.018234	SCGB1A	10
0.0042328400	0.025045	0.018265	IL6/TRE	5
0.0043262135	0.025559	0.018640	MSR1/P	7
0.0043938461	0.025921	0.018904	IL6/ACE	10
0.0044451009	0.025954	0.018928	CCL21/C	3
0.0044451009	0.025954	0.018928	UMOD/	3
0.0044451009	0.025954	0.018928	GM2A/T	3
0.0044451009	0.025954	0.018928	GUSB/H	3
0.0044451009	0.025954	0.018928	STAB2/	3

0.004445100[illegible]	0.02595[illegible]	0.01892[illegible]	ACVRL	3
0.004445100[illegible]	0.02595[illegible]	0.01892[illegible]	MMP9/I	3
0.004459722[illegible]	0.02600[illegible]	0.01896[illegible]	CTSD/A	15
0.004559461[illegible]	0.02654[illegible]	0.01935[illegible]	MRC1/C	8
0.004648985[illegible]	0.02683[illegible]	0.01956[illegible]	HAVCR	4
0.004648985[illegible]	0.02683[illegible]	0.01956[illegible]	IL6/LGA	4
0.004648985[illegible]	0.02683[illegible]	0.01956[illegible]	LILRB4	4
0.004648985[illegible]	0.02683[illegible]	0.01956[illegible]	LILRB4	4
0.004648985[illegible]	0.02683[illegible]	0.01956[illegible]	TNFRSI	4
0.004648985[illegible]	0.02683[illegible]	0.01956[illegible]	RGMA/T	4
0.004727869[illegible]	0.02724[illegible]	0.01987[illegible]	ACVRL	16
0.004746207[illegible]	0.02727[illegible]	0.01989[illegible]	SPINT1/	6
0.004746207[illegible]	0.02727[illegible]	0.01989[illegible]	SFTPD/	6
0.004890462[illegible]	0.02802[illegible]	0.02043[illegible]	FAS/TN	9
0.004890942[illegible]	0.02802[illegible]	0.02043[illegible]	CHRDL	19
0.005011111[illegible]	0.02848[illegible]	0.02077[illegible]	GDF15/	11
0.005014798[illegible]	0.02848[illegible]	0.02077[illegible]	CD74/C	5
0.005014798[illegible]	0.02848[illegible]	0.02077[illegible]	CD300C	5
0.005014798[illegible]	0.02848[illegible]	0.02077[illegible]	EPHB4/	5
0.005014798[illegible]	0.02848[illegible]	0.02077[illegible]	EPHB4/	5
0.005014798[illegible]	0.02848[illegible]	0.02077[illegible]	EPHB4/	5
0.005073923[illegible]	0.02874[illegible]	0.02096[illegible]	CHRDL	6
0.005073923[illegible]	0.02874[illegible]	0.02096[illegible]	TNFRSI	6
0.005130016[illegible]	0.02901[illegible]	0.02116[illegible]	HAVCR	7
0.005202204[illegible]	0.02938[illegible]	0.02143[illegible]	MSR1/C	4
0.005267280[illegible]	0.02971[illegible]	0.02166[illegible]	ACVRL	15
0.005312689[illegible]	0.02984[illegible]	0.02176[illegible]	CD80/IL	3
0.005312689[illegible]	0.02984[illegible]	0.02176[illegible]	CD80/IL	3
0.005312689[illegible]	0.02984[illegible]	0.02176[illegible]	FGF23/I	3
0.005331209[illegible]	0.02990[illegible]	0.02180[illegible]	FABP4/I	9
0.005381687[illegible]	0.03014[illegible]	0.02198[illegible]	CTSZ/H	11
0.005417938[illegible]	0.03023[illegible]	0.02205[illegible]	NET1/SI	6
0.005417938[illegible]	0.03023[illegible]	0.02205[illegible]	TNFRSI	6
0.005421388[illegible]	0.03023[illegible]	0.02205[illegible]	GDF15/	7
0.005441851[illegible]	0.03026[illegible]	0.02207[illegible]	IL6/TRI	5
0.005441851[illegible]	0.03026[illegible]	0.02207[illegible]	TNFSF1	5
0.005724992[illegible]	0.03179[illegible]	0.02319[illegible]	LBP/CL	7
0.005797811[illegible]	0.03205[illegible]	0.02337[illegible]	CCL21/	4

0.005797811(	0.03205	0.02337(	IL6/IL1I	4
0.005797811(	0.03205	0.02337(	RGMA/'	4
0.005803169:	0.03205	0.02337(	CCL21/	12
0.005846932!	0.03225	0.02352(	PLAUR/	17
0.005893960{	0.03242(	0.02364	RGMA/'	5
0.005893960{	0.03242(	0.02364	RGMA/'	5
0.006041137(	0.03318	0.02420	FABP4/I	7
0.006084940'	0.03337!	0.02434	HGF/TG	8
0.006156709(	0.03372(	0.02459(	HGF/IL(	6
0.006275023:	0.03405!	0.02483(	IL18R1/	3
0.006275023:	0.03405!	0.02483(	IL6/CD7	3
0.006275023:	0.03405!	0.02483(	TREM2/	3
0.006275023:	0.03405!	0.02483(	PLAUR/	3
0.006275023:	0.03405!	0.02483(	IL6/SFR	3
0.006275023:	0.03405!	0.02483(	TNFRSI	3
0.0062765341	0.03405!	0.02483(	GDF15/(	16
0.006370132!	0.03438!	0.02507	GDF15/I	7
0.006371875:	0.03438!	0.02507	MSR1/II	5
0.006371875:	0.03438!	0.02507	EPHB4/	5
0.006371875:	0.03438!	0.02507	EPHB4/	5
0.0064371711	0.03459	0.02523	HAVCR	4
0.0064371711	0.03459	0.02523	FGF23/I	4
0.0064371711	0.03459	0.02523	CCL21/I	4
0.006552384!	0.03516!	0.02564{	SFTPD/I	6
0.006567893{	0.03520!	0.02567!	HGF/IL(	9
0.0066203591	0.03543{	0.02584!	HGF/TG	13
0.006876333:	0.03671(	0.02677	PLA2G2	5
0.006876333:	0.03671(	0.02677	CCL21/!	5
0.007091250'	0.03780	0.02757	CCL21/!	14
0.007121605!	0.03781	0.02758(	CD74/TI	4
0.007121605!	0.03781	0.02758(	LBP/BP	4
0.007121605!	0.03781	0.02758(	TGFBR2	4
0.007219542:	0.03826	0.02790	ACVRL	10
0.0072243391	0.03826	0.02790	CCL21/(	13
0.007298842(	0.03853{	0.02810!	IL10RB/	8
0.007334597{	0.03853{	0.02810!	ICAM1/	3
0.007334597{	0.03853{	0.02810!	LILRA5	3
0.007334597{	0.03853{	0.02810!	CSF1/TI	3

0.007334597	0.03853	0.02810	CLEC6A	3
0.007334597	0.03853	0.02810	ITGA5/C	3
0.007398608	0.03872	0.02823	LILRB4	6
0.007398608	0.03872	0.02823	LILRB4	6
0.007398608	0.03872	0.02823	LILRB4	6
0.007756687	0.04054	0.02956	MMP12	10
0.007850065	0.04093	0.02985	LBP/BP	6
0.007852389	0.04093	0.02985	CCL21/S	4
0.007967783	0.04142	0.03021	EPHB4/	5
0.007967783	0.04142	0.03021	CCL21/I	5
0.008215386	0.04262	0.03108	CRHBP/	15
0.008218731	0.04262	0.03108	LILRB4	7
0.008326321	0.04307	0.03141	GLA/SE	18
0.008327200	0.04307	0.03141	MMP7/C	13
0.008493613	0.04376	0.03191	CXCL17	3
0.008493613	0.04376	0.03191	SCGB1A	3
0.008493613	0.04376	0.03191	SCGB1A	3
0.008556202	0.04397	0.03206	LILRB1	5
0.008556202	0.04397	0.03206	ANGPT	5
0.008630746	0.04401	0.03209	IL6/LGA	4
0.008630746	0.04401	0.03209	TFF2/SE	4
0.008630746	0.04401	0.03209	MSR1/C	4
0.008630746	0.04401	0.03209	SPON2/	4
0.008630746	0.04401	0.03209	SPON2/	4
0.008630746	0.04401	0.03209	LILRB4	4
0.008686957	0.04418	0.03222	IL6/TRE	8
0.008686957	0.04418	0.03222	CRHBP/	8
0.008811926	0.04470	0.03260	STAB2/	6
0.008811926	0.04470	0.03260	GDF15/	6
0.008850875	0.04485	0.03270	GLA/PL	15
0.008923007	0.04515	0.03293	CCL21/C	10
0.009062895	0.04580	0.03340	SCGB1A	8
0.009174013	0.04631	0.03377	KITLG/I	5
0.009234550	0.04655	0.03395	ACVRL	10
0.009335769	0.04700	0.03428	SFRP1/F	9
0.009457853	0.04756	0.03468	MSR1/C	4
0.009753986	0.04880	0.03559	FGF23/C	3
0.009753986	0.04880	0.03559	IL7R/IL	3

0.009753986(	0.04880(	0.03559	CSF1/TI	3
0.009753986(	0.04880(	0.03559	LILRB2	3
0.009898860	0.04946	0.03607	CCL21/	16
1.347060037	4.12200	3.24712	CRELD	61
6.948269402	1.06308	8.37449	PLAUR/	46
3.649903411	3.72290	2.93272	PLAUR/	38
8.690699846	6.64838	5.23729(	CTSD/N	22
4.081858373	2.28428	1.79945(	TIMP1/(	35
4.478997696	2.28428	1.79945(	TIMP1/(	35
1.535761446	6.71347	5.28856	TIMP1/(	34
7.095370516	2.71397	2.13794	CTSD/N	25
3.258836828	1.10800	8.72834(	IGHG4/(	20
8.900747888	2.72362	2.14554	PLAUR/	18
1.317458084	3.66492	2.88706	OSCAR/	18
1.457853631(	3.71571	2.92706	TIMP1/I	12
1.595601939	3.71571	2.92706	ICAM1/	24
1.746866003	3.71571	2.92706	OSCAR/	11
1.821430007	3.71571	2.92706	ICAM1/	24
6.146891012	1.17559	9.26077(	TIMP1/I	13
3.828194668	6.89075(	5.42821	NCAN/I	13
4.284567305	7.28376	5.73781	PLAUR/	23
6.166248310	9.93090	7.82310	SFTPD/(	12
6.561340943	1.00388	7.90814	OSCAR/	10
1.102028226	1.53282	1.20748	PIGR/GI	15
1.102028226	1.53282	1.20748	PIGR/GI	15
2.171741150	2.88935	2.27610	CTSD/C	16
1.134696685	0.00014	0.00011	SFTPD/(	9
1.306820269(	0.00015	0.00012(	CTSD/C	12
2.871220964	0.00032	0.00025(	CD207/(	9
2.871978736	0.00032	0.00025(	PIGR/CI	8
3.763352235	0.00041	0.00032	NCAN/I	4
5.814983808	0.00061	0.00048	NCAN/I	4
6.238289868	0.00063(	0.00050	SFTPD/(	19
8.133822262	0.00074	0.00059(	NCAN/I	5
8.279380065	0.00074	0.00059(	SFTPD/(	15
8.562700285(	0.00074	0.00059(	SFTPD/I	20
8.566723240	0.00074	0.00059(	SFTPD/I	9
8.576771713	0.00074	0.00059(	NCAN/I	4

0.000140456(	0.00119:	0.00094(	MSR1/C	14
0.000167701:	0.00138(	0.00109:	RARRE:	4
0.000217052(	0.00174'	0.00137(	TIMP1/'	9
0.000479349'	0.00376:	0.00296:	CKAP4/	4
0.000510358(	0.00390:	0.00307!	SFTPD/(	17
0.000694364:	0.00518:	0.00408:	PLAUR/	8
0.000891603!	0.00634:	0.00499!	RARRE:	4
0.000891603!	0.00634:	0.00499!	PLAUR/	4
0.001390194:	0.00966(	0.00761(	PLAUR/	6
0.002730265!	0.01856!	0.01462!	CD207/(	9
0.002884790:	0.01919(	0.01511'	TREM2/	3
0.003196298(	0.02080(	0.01639:	GLA/G(	7
0.004061727:	0.02589:	0.02039'	CTSZ/C	7
0.004251739:	0.02655:	0.02091(	IL10RB/	15
0.004486589(	0.02745'	0.02163(	CLEC4I	6
0.005141289:	0.03084'	0.02430(	CD74/B:	3
0.007100538:	0.04178:	0.03291!	PLAUR/	3
0.007952793(	0.04506!	0.03550(	PIGR/C'	18
0.007952793(	0.04506!	0.03550(	PIGR/C'	18
0.008289008:	0.04611'	0.03632!	CTSD/L	4
1.727277428!	8.84366(	6.23638(	SIGLEC	38
8.411545465:	2.15335!	1.51850!	CCL21/'	31
1.353408319!	1.96168:	1.38334(	WFDC2	25
1.532563425:	1.96168:	1.38334(	NCAN/F	30
4.006195318:	3.64790:	2.57243:	WFDC2	26
4.274887332(	3.64790:	2.57243:	WFDC2	25
6.996647608:	5.11754'	3.60879'	TNFRSI	9
2.180034239!	1.39522:	9.83883!	WFDC2	18
2.704033058!	1.53829:	1.08477!	CCL21/'	29
5.183744283:	2.65407'	1.87160:	WFDC2	26
2.594760094:	1.20774:	8.51677:	TNFRSI	7
3.064116715(	1.30735(	9.21922!	PRSS8/I	22
4.547187452:	1.79089:	1.26290:	PRSS8/I	22
1.916084973'	7.00739(	4.94148:	PRSS8/I	20
2.688250798(	9.17589(	6.47066(	ACVRL	18
2.869876854!	9.18360!	6.47610:	TIMP1/(	19
5.533507274!	1.66656:	1.17522'	SIGLEC	20
6.653057244(	1.89242!	1.33450:	SIGLEC	20

7.833613015	2.11095	1.48860	PGF/AP	19
9.760211309	2.49861	1.76197	PIGR/IL	18
2.134595463	5.20434	3.67000	ICAM1/	13
2.487023368	5.78798	4.08157	ICAM1/	13
3.143194928	6.99702	4.93416	CRELD1	18
3.842287107	8.19687	5.78028	ASGR1/	8
4.409206790	8.77563	6.18840	PGF/AP	23
4.456376560	8.77563	6.18840	WFDC2	29
7.044133237	1.33577	9.41964	ASGR1/	10
9.105327855	1.66497	1.17410	PRSS8/M	28
2.066497836	3.64843	2.57280	VWA1/I	11
3.047252367	5.20064	3.66739	ACVRL	15
5.859419638	9.67749	6.82438	ASGR1/	11
1.069802138	1.71168	1.20704	MSR1/C	6
1.225973881	1.90211	1.34133	IL10RB/	12
1.384019884	2.08417	1.46971	ASGR1/	10
5.877522959	8.59797	6.06312	SIGLEC	6
7.329106906	0.00010	7.35053	FABP4/I	8
1.021634735	0.00014	9.96929	ACVRL	6
1.103337255	0.00014	0.00010	WFDC2	7
1.181612513	0.00015	0.00010	CCL21/C	8
1.319557866	0.00016	0.00011	CFB/CF	6
2.125289946	0.00026	0.00018	MSR1/C	6
2.658102985	0.00032	0.00022	CCL21/T	9
5.062618225	0.00059	0.00041	CCL21/C	7
5.074283588	0.00059	0.00041	ACVRL	9
5.254684994	0.00059	0.00042	IL10RB/	8
5.617624388	0.00062	0.00044	FABP4/I	9
5.931176999	0.00063	0.00044	MSR1/C	6
5.931176999	0.00063	0.00044	MSR1/C	6
6.608797083	0.00069	0.00048	CFH/CF	4
7.470862708	0.00076	0.00053	CFH/CF	5
8.028610233	0.00080	0.00056	TPP1/CT	10
0.000136466	0.00131	0.00092	GLA/GN	11
0.000138292	0.00131	0.00092	LILRA5	4
0.000138736	0.00131	0.00092	LILRB1	6
0.000169899	0.00158	0.00111	GLA/GN	9
0.000187622	0.00170	0.00120	S100A1	7

0.0001902727	0.001709	0.001205	TIMP1/I	4
0.0002462506	0.002173	0.001532	EFEMP1	11
0.0002549944	0.002212	0.001560	CA6/CA	4
0.0002854868	0.002430	0.001717	PGF/HG	6
0.0003251425	0.002729	0.001924	EFEMP1	7
0.0003341415	0.002759	0.001945	RBP7/R	4
0.0003702811	0.003009	0.002122	LILRB4	6
0.0003774945	0.003019	0.002129	TPP1/TI	5
0.0004294402	0.003331	0.002349	FABP4/I	4
0.0004294402	0.003331	0.002349	LGALS1	4
0.0004449278	0.003400	0.002397	FSTL3/I	5
0.0004735876	0.003565	0.002514	CTSZ/A	6
0.0005210504	0.003860	0.002720	TNFSF1	5
0.0005426496	0.003913	0.002759	LILRA5	4
0.0005426496	0.003913	0.002759	LILRB4	4
0.0006590848	0.004674	0.003290	GDF15/	10
0.0006755533	0.004674	0.003290	RBP7/R	4
0.0006755533	0.004674	0.003290	CD300L	4
0.0007465128	0.005029	0.003540	EFEMP1	6
0.0007465128	0.005029	0.003540	TNFSF1	6
0.0008306630	0.005450	0.003847	GFRA1/	6
0.0008312506	0.005450	0.003847	TIMP1/T	10
0.0008563473	0.005549	0.003913	ICAM1/	11
0.0008975905	0.005744	0.004050	CCL21/T	16
0.0009224448	0.005830	0.004111	SCARB2	8
0.0010207477	0.006221	0.004387	EFNA4/	6
0.0010207477	0.006221	0.004387	EFNA4/	6
0.0010207477	0.006221	0.004387	OSCAR	6
0.0011142097	0.006711	0.004732	AGRN/A	3
0.0013651778	0.008127	0.005731	S100A1	7
0.0015072554	0.008870	0.006255	ACVRL	3
0.0016987113	0.009883	0.006969	NCAN/I	4
0.0017949555	0.010320	0.007281	CD300L	6
0.0019214319	0.010810	0.007623	SPON2/	5
0.0019214319	0.010810	0.007623	LILRB1	5
0.0019772002	0.011003	0.007759	TPP1/PI	3
0.0023089815	0.012711	0.008964	PIGR/UI	4
0.0024941729	0.013585	0.009580	MMP12	11

0.0025288725	0.013629	0.009611	PGF/VE	3
0.0026642794	0.014209	0.010020	CD74/C	4
0.0030553134	0.016127	0.011372	CD300L	4
0.0032327612	0.016889	0.011910	CD300L	13
0.0038254129	0.019713	0.013901	CD300L	7
0.0039512777	0.019713	0.013901	IL18BP/	4
0.0042399133	0.019713	0.013901	EPHB4/	5
0.0042399133	0.019713	0.013901	EPHB4/	5
0.0042399133	0.019713	0.013901	EPHB4/	5
0.0042399133	0.019713	0.013901	EPHB4/	5
0.0042399133	0.019713	0.013901	EPHB4/	5
0.0042399133	0.019713	0.013901	EPHB4/	5
0.0042399133	0.019713	0.013901	EPHB4/	5
0.0042399133	0.019713	0.013901	EPHB4/	5
0.0042399133	0.019713	0.013901	EPHB4/	5
0.0042399133	0.019713	0.013901	EPHB4/	5
0.0042737490	0.019713	0.013901	TREM1/	6
0.0046280689	0.020785	0.014657	EPHB4/	5
0.0046280689	0.020785	0.014657	EPHB4/	5
0.0046280689	0.020785	0.014657	EPHB4/	5
0.0047162344	0.020997	0.014807	FABP4/	3
0.0050408304	0.022059	0.015555	EPHB4/	5
0.0050408304	0.022059	0.015555	EPHB4/	5
0.0059434124	0.025788	0.018185	CRELD	5
0.0066532698	0.028625	0.020180	RNASE	3
0.0077740917	0.032360	0.022819	CRELD	3
0.0077740917	0.032360	0.022819	FABP4/	3
0.0077740917	0.032360	0.022819	CRELD	3
0.0077740917	0.032360	0.022819	IGHG4/	3
0.0090151196	0.037223	0.026249	MSR1/T	12
0.0092809557	0.037713	0.026594	RBP7/R	4
0.0092809557	0.037713	0.026594	RBP7/R	4

zScore	pvalue	p.adjust	qvalue	geneID	Count
16.496807363	1.313665	3.139660	2.461393	CXCL17	53
13.091823818	5.568884	6.654817	5.217165	PLAUR/	22
12.089733341	1.014402	8.081404	6.335564	CCL21/	22

8.639124287(	3.25790(	1.94659!	1.52606!	ICAM1/	16
6.391274271!	1.15429!	5.51753(	4.32556!	OSCAR/	16
6.225012638(	2.27039!	9.04375:	7.09001!	CTSD/G	15
6.215936699:	3.00286(	0.00010:	8.03774!	ICAM1/	14
6.229301257‹	3.83742:	0.00011‹	8.98765(	CCL21/I	13
7.186054991′	4.40661!	0.00011′	9.17401:	ICAM1/	8
5.116800404	1.52598!	0.00036‹	0.00028!	ANGPT:	22
5.603660050‹	3.77380:	0.00081!	0.00064:	IL6/CD1	10
5.881378038:	4.54543:	0.00090!	0.00070!	ICAM1/	8
5.214428649!	6.57738:	0.00120!	0.00094′	IL2RA/(	11
5.130708101‹	7.89541(	0.00134′	0.00105(	ICAM1/	11
4.458061829(	9.29882	0.00148	0.00116	ANGPT:	23
5.392287217!	0.00016‹	0.00246	0.00192!	FAS/IL6	7
4.999791711!	0.00032:	0.00455‹	0.00357(	PIGR/IL	7
3.962654427!	0.00063‹	0.00841!	0.00660(	TNFRSI	15
5.355323860!	0.00088	0.01109:	0.00869(	CA6/CA	4
4.538419153(	0.00100:	0.01160‹	0.00909′	FAS/CD	6
4.778277943	0.00105:	0.01160‹	0.00909′	GGH/IL	5
3.865990091(	0.00107(	0.01160‹	0.00909′	SFTPD/I	12
3.798841948!	0.00111(	0.01160‹	0.00909′	IL10RB/	13
3.776319234!	0.00117‹	0.01169‹	0.00916′	TNFRSI	13
3.798942849(	0.00139(	0.01328!	0.01041!	ICAM1/	11
4.028925757(	0.00176	0.01619(	0.01269′	IL6/IL1!	7
3.531564792:	0.00220	0.01949(	0.01527!	TNFRSI	12
3.489199640′	0.00265(	0.02262(	0.01773!	ICAM1/	11
3.954228866:	0.00350:	0.02837!	0.02224!	FAS/CD	5
3.345214560!	0.00356	0.02837!	0.02224!	ICAM1/	11
3.510365986(	0.00368!	0.02844!	0.02230(	IL6/CCI	8
3.132788572(	0.00446(	0.03336	0.02615:	ANGPT:	14
3.223048208!	0.00499‹	0.03617	0.02835′	TNFRSI	10
3.306422167:	0.00535(	0.03765(	0.02951′	ICAM1/	8
3.324431808:	0.00601:	0.03998′	0.03134!	CD74/C:	7
3.241775841!	0.00602:	0.03998′	0.03134!	TNFRSI	8
3.056798627!	0.00840(	0.05430(	0.04256!	IL2RA/T	8
3.000641719‹	0.01047!	0.06590‹	0.05166′	LILRA5	7
3.562494513	0.01311:	0.08035!	0.06299!	GUSB/H	3
2.679138409!	0.01410:	0.08426‹	0.06606(	IL10RB/	10
2.850189364!	0.01539!	0.08974(	0.07035!	FABP4/I	6

2.92643029180	0.016159	0.091950	0.072091	IL6/CD1	5
3.021955011	0.017697	0.098365	0.077115	CD4/TN	4
2.7399955857	0.018338	0.099612	0.078093	GSTA3/	6
2.8933929940	0.021030	0.111697	0.087567	AMY1A	4
2.5685422290	0.021614	0.112299	0.088039	CLEC4D	7
2.5403965180	0.022637	0.115114	0.090245	SPINT1/	7
2.3164973849	0.024997	0.121575	0.095311	TNFRSF	12
2.6138970517	0.025634	0.121575	0.095311	PLA2G2	5
2.4578445059	0.025907	0.121575	0.095311	LBP/IL6	7
2.3175235654	0.025942	0.121575	0.095311	ANGPT	11
2.3325569874	0.029533	0.135739	0.106415	TNFRSF	8
2.4068961517	0.030872	0.139215	0.109140	COL6A3	6
2.12199573	0.036661	0.161425	0.126552	ICAM1/	11
2.2862011517	0.037148	0.161425	0.126552	IL2RA/C	6
3.0191866804	0.038947	0.166224	0.130314	ASGR1/	2
2.0315865582	0.044310	0.179767	0.140931	PGF/HG	10
2.0315865582	0.044310	0.179767	0.140931	TNFRSF	10
2.2354438058	0.044377	0.179767	0.140931	GSTA3/	5
2.3087563159	0.045964	0.183092	0.143538	PLTP/A	4
2.1673644556	0.048899	0.191591	0.150201	FAS/ER	5

Supplementary Table S13. Associations between smoking-associated circulating

ProbeCh	Gene	Probe_bp	topSNP	topSNP_	topSNP_bp	A1	A2
4	ADH4	99140724	rs18007	4	99144358	G	T
12	FGF23	4373970	rs64895	12	4382743	C	G
14	PGF	74948730	rs65742	14	74986336	C	G
8	CTHRC	103377264	rs82759	8	103352409	G	C
14	NPC2	74485184	rs18590	14	73689254	A	G
11	YAP1	102171936	rs10791	11	102052822	A	G
19	FUT3	5847180	rs37607	19	5841345	T	G
2	INHBB	120348970	rs17050	2	120548864	A	G
11	RELT	73386936	rs11235	11	73387281	A	G
17	CCL3	36089212	rs16344	17	36082734	C	A
1	PCSK9	55052150	rs11591	1	55039974	T	G
19	IFI30	18175640	rs72594	19	18172201	C	A
20	BPIFB1	33291676	rs24249	20	33106642	C	T
5	CD74	150407303	rs79078	5	150414087	A	G
19	CEACA	41719509	rs9621	19	41727239	A	G
20	TNFRSF	63697668	rs60624	20	63704906	T	C
3	RBP2	139466816	rs29546	3	139494058	A	G
1	VWA1	1438872	rs11550	1	1434243	A	G
3	HEG1	125010854	rs64388	3	125009628	G	C
1	QSOX1	180179450	rs12371	1	180194255	G	A
8	SFRP1	41285718	rs72643	8	41309674	T	G
2	CAPG	85406592	rs6886	2	85394936	C	T
14	CLEC14	38255046	rs10151	14	38503166	A	C
19	LGALS	38807308	rs14069	19	38774589	T	A
18	COLEC	408730	rs23050	18	334742	T	C
2	DNER	229536092	rs34412	2	229727643	T	A
19	NOTCH	15180016	rs48082	19	15192918	A	G
7	RARRE	150339990	rs37351	7	150342466	T	C
17	ENPP7	79736581	rs80765	17	79730552	T	C
17	ASGR1	7176498	rs55714	17	7176997	T	C
12	CNTN1	40882427	rs11177	12	40651583	A	C
1	CA14	150261164	rs11229	1	150286426	G	C
14	RNASE	20781868	rs10459	14	20781965	A	G
19	BSG	577386	rs56101	19	586745	C	T
10	MRC1	17860256	rs72782	10	17936043	G	T
9	FCN1	134910572	rs10858	9	134966017	C	T

1	PIGR	206937494	rs29110:	1	206933133	A	G
17	CLEC10	7077422	rs90951	17	7078078	G	A
20	OXT	3072068	rs87717:	20	3069244	G	T
1	TNFRSF	7929518	rs24932	1	7934360	G	A
13	ITGBL1	101586724	rs10342	13	101456046	T	C
3	IL1RAP	190586900	rs64444	3	190628271	G	A
19	PLAUR	43658321	rs23025:	19	43652320	C	T
12	LTA4H	96022136	rs25404	12	96037229	G	C
19	LILRB2	54277498	rs38392	19	54279666	T	C
19	SSC5D	55503752	rs81030	19	55487775	G	C
19	SIGLEC	51524838	rs62617	19	51531295	A	C
6	CFB	31948868	rs64115	6	31946403	A	G
19	LILRA5	54310118	rs41413	19	54302597	T	A
7	BMPER	34030368	rs20189	7	33743596	T	C
20	CTSZ	58996962	rs14837	20	59027157	T	C
21	TFF1	42364408	rs37613	21	42366929	A	G
12	NELL2	44715062	rs38211	12	44936741	C	T
11	MMP7	102525629	rs11568	11	102530902	A	G
10	IL2RA	6036530	rs12722	10	6053965	A	C
6	HLA-DI	32442462	rs26470	6	32606583	T	C
20	BPIFA2	33171590	rs60591	20	33166430	A	T
9	CD72	35628396	rs86948	9	35615899	G	C
2	IMMT	86169702	rs17551	2	86195293	C	T
11	TPP1	6616108	rs11827	11	6626391	C	T
19	CFD	863668	rs71335:	19	859368	T	G
1	EFNA4	155066645	rs72704	1	155196150	A	G
4	ART3	76061993	rs48596	4	76079288	G	A
6	TREM2	41160846	rs14333:	6	41161469	T	C
12	CD4	6803828	rs73053	12	6787380	G	A
5	MZB1	139388774	rs11649	5	139190262	T	C
19	CXCL1	42435612	rs11668	19	42450984	G	A
7	HGF	81734724	rs57456	7	81729735	T	C
19	GDF15	18381954	rs10542	19	18389048	C	T
1	CD101	117019151	rs12130:	1	117001709	C	A
17	KRT19	41525962	rs74056	17	41535160	A	G
22	PLXNB:	50291312	rs11547	22	50283705	C	T
4	F11	186277935	rs22892	4	186286227	T	C
7	NPTX2	98623577	rs39014	7	98658349	T	C

21	TFF2	42348677	rs22534	21	42355775	A	G
1	TNFRSF	12188110	rs57460	1	12193005	A	G
19	VSTM1	54052389	rs24337	19	54042277	C	T
12	CPM	68906884	rs79781	12	68932767	T	C
3	CDCP1	45114350	rs22768	3	45146293	G	C
5	OCLN	69525198	rs25837	5	69461947	C	T
11	SERPIN	57608279	rs28362	11	57598250	C	T
2	IL1RN	113120615	rs55709	2	113109711	C	T
17	CD300E	74616812	rs17194	17	74580252	C	T
1	C8B	56951795	rs62645	1	56941811	T	C
2	CD207	70833014	rs17718	2	70833805	C	G
7	FAM3C	121372621	rs27075	7	121429150	A	T
22	IGLC2	22901206	rs28568	22	22907270	A	C
1	EXTL1	26028174	rs27368	1	26031165	A	C
6	VEGFA	43778336	rs69214	6	43957870	A	G
17	CA4	60160420	rs11715	17	60156793	T	A
6	ULBP2	149945624	rs62441	6	149979178	T	A
1	FABP3	31371052	rs61780	1	31404204	T	C
17	CCL7	34271232	rs65053	17	34190977	A	T
1	CFHR2	196951680	rs40857	1	196951018	T	C
16	PLA2G1	68253181	rs7672	16	68260897	G	C
8	TNFRSF	23207777	rs13278	8	23225458	T	G
7	GPR37	124754838	rs41410	7	124488565	C	A
4	SCARB	76196636	rs28563	4	76176220	C	A
10	PRAP1	133350026	rs14367	10	133348399	A	G
5	SPOCK	137286838	rs48357	5	137559375	G	A
1	LEPR	65531106	rs10399	1	65608299	T	C
9	ADAMT	133553842	rs10082	9	133532712	G	A
15	RGMA	93062241	rs37521	15	93072785	A	C
12	KRT18	52950888	rs49197	12	52879136	G	A
6	VNN1	132697452	rs93890	6	132694981	T	C
12	PTPRR	70779406	rs14872	12	70833617	C	T
1	TNR	175529405	rs22352	1	175599911	G	A
1	CA6	8960480	rs37659	1	8974539	G	A
21	IL10RB	33288277	rs25157	21	33289977	A	G
19	SIGLEC	51147912	rs14018	19	51142883	C	G
11	ADM	10306235	rs79264	11	10336912	T	C
7	TFPI2	93888074	rs62466	7	93949980	C	T

9	PTGR1	111574808	rs70361(	9	111564267	G	A
1	TNFRSF	2560510	rs24953(	1	2557491	A	G
9	AMBP	114069228	rs14173{	9	114069727	C	T
8	TNFRSF	23110183	rs65576	8	23103191	C	G
11	SCGB1/	62414149	rs37412	11	62419070	A	G
6	MOG	29664687	rs94615	6	29657418	G	A
4	ODAM	70200150	rs76153(	4	70195601	C	T
3	SHISA5	48486312	rs11706(	3	48502103	C	T
4	GC	71772868	rs13528	4	71752058	G	A
2	CD302	159783442	rs25561(	2	159798013	T	G
17	FAM20/	68568240	rs29522	17	68533638	A	G
3	LRIG1	66440030	rs23062	3	66384219	C	T
1	TNFRSF	1212746	rs38132(	1	1215384	A	G
17	LGALS9	27639679	rs79526	17	27644953	G	A
19	SIGLEC	51130778	rs20758(	19	51125272	G	A
15	GCHFR	40765888	rs22892	15	40773528	A	T
1	AMY1A	103660157	rs72694	1	103822256	A	G
10	FAS	88991709	rs10887{	10	89023165	A	T
13	NHLRC	39044208	rs41286	13	39039504	C	A
13	CPB2	46079110	rs65612{	13	46066702	G	A
13	LCP1	46168896	rs73233	13	46133701	A	T
19	LILRB1	54627590	rs36707(	19	54296648	G	A
19	FGF21	48756928	rs83813	19	48757420	C	A
12	LRTM2	1828510	rs41276(	12	1831348	A	G
20	PLTP	45905388	rs60739	20	45923216	C	T
1	AGRN	1038119	rs31215(	1	1032846	A	C
1	SELE	169743672	rs75157	1	169738588	G	A
10	BLNK	96230379	rs55769	10	96246079	C	A
18	CDH2	28055412	rs12454	18	28110963	A	G
11	CLMP	123132560	rs73017{	11	123192354	G	C
9	CCL21	34709570	rs10814	9	34723038	A	C
7	GUSB	65971450	rs34356	7	66771620	T	G
19	SULT2/	47878391	rs21210(	19	47873738	C	T
3	FETUB	186644555	rs50301(	3	186731139	C	T
1	AMY2A	103621604	rs78245	1	103793104	G	T
19	LILRB4	54657124	rs37015(	19	54661088	A	G
16	PPL	4921624	rs74005	16	4954971	T	G
14	RNASE	20802042	rs17254	14	20812519	A	G

8	TNFRSF	118937721	rs64698	8	119115831	C	T
5	CRHBP	76967102	rs689625	5	76914771	A	G
5	EDIL3	84162717	rs230107	5	84023794	A	G
14	RNASE4	20692888	rs125885	14	20678425	T	C
22	RTN4R	20262330	rs663353	22	20187189	A	G
5	CDHR2	176569242	rs296368	5	176515947	T	C
4	AFM	73492873	rs131327	4	73480179	C	T
3	ITIH4	52821825	rs271033	3	52803839	C	T
16	TNFRSF	3020414	rs747581	16	3020627	T	C
18	CLUL1	623661	rs129574	18	591690	C	G
2	EFEMP1	55895053	rs379167	2	55869757	G	A
9	OMD	92418426	rs101152	9	92433576	A	G
12	APOF	56361712	rs202085	12	56349583	C	T
17	ALDH3A	19743464	rs887241	17	19742625	C	A
16	MSLN	764800	rs376424	16	760143	G	A
8	ANGPT2	6531520	rs196858	8	6421990	T	C
8	PLAT	42191214	rs202092	8	42187447	A	G
1	IFNLR1	24171064	rs139958	1	24187233	C	G
22	GGT1	24611908	rs285093	22	24601879	C	T
16	UMOD	20344676	rs129177	16	20356368	T	G
1	S100A12	153374666	rs301487	1	153365467	A	G
1	BCAN	156650461	rs236571	1	156645322	G	A
6	PLG	160728168	rs117513	6	160671406	T	C
10	MSMB	46040744	rs707783	10	46073546	G	C
11	PRCP	82896760	rs251029	11	82835874	T	A
6	EDN1	12293778	rs5370	6	12296022	T	G
2	COL6A3	237369166	rs105078	2	237324109	A	C
8	RIDA	98109758	rs224219	8	98106394	C	A
8	SLURP1	142741678	rs258513	8	142732775	G	A
19	A1BG	58349335	rs126595	19	58347310	G	A
2	FABP1	88125522	rs224188	2	88124547	C	T
5	LECT2	135938631	rs31517	5	135951340	C	T
19	LAIR1	54360971	rs111949	19	54358584	A	T
22	SEZ6L	26276530	rs137203	22	26292865	T	G
6	THBS2	169234915	rs781091	6	169224606	A	C
19	ICAM1	10278854	rs503037	19	10282558	G	A
5	HAVCR	157114350	rs115961	5	157091701	C	G
12	CD27	6448336	rs25680	12	6445462	A	G

4	ENPP6	184154968	rs78293!	4	184217948	T	C
5	SPINK6	148208966	rs12717!	5	148186561	G	A
12	C1S	7029646	rs14611	12	6800807	T	C
5	CD14	140632714	rs57444	5	140637262	A	G
21	COL18A	45459442	rs99768	21	45477300	T	C
17	TNFRSF	16950967	rs34562	17	16939677	A	G
2	HNMT	137990192	rs11558	2	138002079	T	C
1	CHI3L1	203182818	rs49509	1	203186754	C	G
7	EPHB4	100815044	rs31436	7	100815383	G	A
19	ICAM5	10293365	rs28143	19	10289434	C	G
10	CDH23	71606434	rs21666	10	71713125	T	C
17	WFIKKI	50838502	rs45911	17	50832073	A	T
6	KIAA03	24595148	rs31812	6	24654215	T	C
13	TNFSF1	108279862	rs37403	13	108308037	A	T
14	SERPIN	94383720	rs17580	14	94380925	A	T
17	CCL18	36068152	rs20150	17	36064257	G	A
2	VWC2L	214495015	rs10932	2	214526257	A	G
5	HAVCR	157044266	rs65558	5	157045138	A	C
10	SFTPA2	79558130	rs17886	10	79558907	G	C
17	CCL16	35978995	rs11268	17	35980421	C	T
1	APCS	159588346	rs36126	1	159593911	G	T
17	LGALS	78975592	rs11152	17	78966679	T	C
15	SCG3	51701259	rs26061	15	51681453	C	G
1	S100A1	153625074	rs11467	1	153622972	A	C
17	IGFBP4	40450588	rs16683	17	40459730	C	T
14	ANG	20691574	rs80082	14	20688145	A	G
1	CTBS	84562046	rs79723	1	84547012	A	G
17	CD300L	74703644	rs35489	17	74704804	G	A
1	S100A1	152040206	rs29995	1	152040058	C	G
12	STAB2	103676996	rs11584	12	103640270	G	A
3	APOD	195576369	rs13982	3	195572021	C	T
6	DLL1	170294386	rs93566	6	170278382	G	A
3	HYAL1	50306136	rs11648	3	50302191	T	C
11	IL18BP	72002164	rs57436	11	71998832	A	G
16	PRSS8	31133580	rs88955	16	31111250	T	C
11	RAB6A	73718388	rs11235	11	73927813	T	C
3	PODXL	127650994	rs11489	3	127634127	G	A
5	C9	39327732	rs69676	5	39356024	G	A

22	CHCHD	23766903	rs34284	22	23824602	A	G
11	FOLR2	72219276	rs14689	11	72206309	T	C
20	BPIFB2	33015704	rs23777	20	32994638	C	T
19	HSPB6	35756322	rs58561	19	35758074	C	T
7	VGF	101164039	rs34985	7	101174620	G	A
10	GFRA1	116166864	rs11197	10	116242259	C	T
12	C1R	7086377	rs49635	12	6833659	G	A
4	SPINK2	56815801	rs28656	4	56823779	G	A
2	QPCT	37358074	rs46706	2	37372820	C	A
19	EBI3	4233526	rs81091	19	4247479	T	C
20	MMP9	46012734	rs17576	20	46011586	G	A
3	CD80	119541954	rs56078	3	119567674	T	A
8	FABP4	81480828	rs20448	8	81483590	G	T
1	NPL	182809838	rs12142	1	182794470	T	A
20	WFDC2	45475642	rs97344	20	45465735	G	C
19	ACP5	11576834	rs23057	19	11576536	T	C
15	GOLM2	44352238	rs80235	15	43883905	G	A
17	ASGR2	7108511	rs23049	17	7108760	T	C
1	RBP7	10006614	rs12203	1	9935196	A	G
17	OMG	31284776	rs72813	17	31134507	A	G
1	IGSF3	116621077	rs22848	1	116580265	C	T
11	SPON1	14115428	rs10832	11	14026933	T	C
12	VWF	6036824	rs68120	12	6032283	A	G
4	FABP2	119319694	rs17009	4	119359462	T	C
17	PLXDC	39108854	rs62076	17	39067156	T	C
19	ANGPT	8368830	rs22782	19	8366697	A	G
22	NPTXR	38831240	rs11157	22	38845257	G	A
22	CRELD	49922854	rs74510	22	49921734	G	C
12	CKAP4	106271080	rs27048	12	106256210	A	G
12	C1RL	7099412	rs10477	12	6839528	G	A
8	LY6D	142785710	rs25729	8	142786489	T	C
3	ACY1	51986268	rs15041	3	51982820	T	C
15	SPINT1	40851112	rs17658	15	40853721	T	C
12	CLSTN	7144322	rs18834	12	6780329	A	G
10	ADAM1	126200429	rs11244	10	126256024	C	T
19	CEACA	42587649	rs12459	19	41759235	G	A
19	CEACA	41761594	rs12459	19	41759235	G	A
7	VWC2	49847794	rs76960	7	49775794	G	C

3	LRRN1	3824632	rs98394	3	3766094	A	G
9	OGN	92393982	rs70263	9	92376282	T	G
19	NCAN	19232096	rs37610	19	19215154	T	G
13	GPC5	92136152	rs34270	13	91764804	C	T
6	TNF	31576950	rs22290	6	31572779	C	T
6	TNF	31576950	rs22290	6	31572779	C	T
6	TNF	31576950	rs22290	6	31572779	C	T
6	TNF	31576950	rs22290	6	31572779	C	T
6	TNF	31576950	rs22290	6	31572779	C	T
6	TNF	31576950	rs22290	6	31572779	C	T
14	SERPIN	94618220	rs61976	14	94631329	G	A
5	GM2A	151241295	rs15347	5	151259878	G	A
15	ACAN	88839394	rs42933	15	88710319	C	T
22	LGALS	37677719	rs48202	22	37675036	A	G
16	IL17C	88639520	rs17700	16	88618087	T	G
2	ALPP	232380806	rs12478	2	232421944	T	C
15	IGDCC	65402216	rs56362	15	65509382	C	T
2	TGFA	70500738	rs17639	2	70549149	C	T
2	RNF149	101289960	rs12621	2	101280863	T	C
10	PSAP	71833774	rs57681	10	71884204	T	C
4	HPGDS	94320706	rs28653	4	94349727	C	T
12	RBP5	7122312	rs11064	12	6859577	G	C
2	CCL20	227811652	rs13953	2	227783607	A	G
13	TNFSF1	42585374	rs20623	13	42478744	A	G
11	CNTN5	99689917	rs89877	11	99218310	A	G
1	FASLG	172662990	rs79419	1	172703333	A	T
22	SERPIN	20780916	rs23294	22	20700042	C	T
18	DCC	52938050	rs98074	18	51955366	A	G
8	GGH	63027144	rs74329	8	63018637	A	G
16	CCL22	57362486	rs72784	16	57350214	G	A
3	CRELD	9939603	rs98780	3	9943773	C	T
11	MMP12	102868859	rs17368	11	102877965	G	A
13	SLITRK	83879840	rs28768	13	83794650	A	T
14	LGMN	92726243	rs14865	14	92709697	A	G
19	KLK8	50999359	rs74705	19	51001552	A	G
19	RTBDN	12830453	rs72550	19	12821455	A	G
7	DPP6	154390691	rs37349	7	154892443	C	T
12	PTPRB	70576655	rs24658	12	70596274	C	T

4	CFI	109766579	rs10033	4	109737911	C	T
1	CFH	196702115	rs73155	1	196735885	A	C
7	EPHA1	143399572	rs75045	7	143412115	G	T
12	CLEC7A	10123518	rs16910	12	10118488	C	A
12	GPD1	50107648	rs14062	12	50923648	C	G
11	LUZP2	24789804	rs76794	11	25028282	A	T
2	CGREF1	27109008	rs23845	2	27101883	T	G
6	GSTA1	52797616	rs64588	6	52801179	T	C
6	GSTA3	52903168	rs64588	6	52801179	T	C
1	AMY2B	103566674	rs12080	1	103787201	A	C
20	CHGB	5918432	rs23615	20	5923248	G	A
20	CST3	23632590	rs91111	20	23632100	T	C
3	FSTL1	120421643	rs11477	3	120450401	T	C
10	TCTN3	95676983	rs61871	10	95665573	C	A
17	ERBB2	39709170	rs37643	17	39760570	T	C
10	SFTPD	79960040	rs72191	10	79946568	G	A
11	NCAM1	113119842	rs11214	11	113105212	T	C
14	SERPIN	94313818	rs27495	14	94360731	T	C
19	CEACA	41568181	rs72390	19	41577830	C	G
8	CCN3	119420440	rs22791	8	119416784	A	G
9	LCN2	128151262	rs61125	9	128140905	C	A
3	LAMP3	183143027	rs11921	3	183110457	T	G
12	ITGA5	54407264	rs17958	12	54456092	G	T
11	PGA4	61227020	rs11306	11	61452227	T	C
8	TNFRSF	23044582	rs11059	8	23027596	G	A
11	FOLH1	49176865	rs61886	11	49158908	T	G
11	B4GAT1	66346502	rs10896	11	66349640	T	C
1	CSF1	109920617	rs11577	1	109957553	C	T
8	ST3GAL	133513394	rs96433	8	133490905	T	C
9	IGFBPL	38415491	rs78572	9	38409084	A	G
1	REG4	119802798	rs79795	1	119816663	A	C
1	EFNA1	155131388	rs43901	1	155133578	G	A
11	AHNAK	62494888	rs74853	11	62529129	C	T
5	IL7R	35866149	rs68979	5	35874473	T	C
16	CA5A	87909063	rs55870	16	87893616	C	T
15	FURIN	90876076	rs20714	15	90877710	G	C
18	TNFRSF	62358288	rs62098	18	62353960	G	A
19	FSTL3	679892	rs91690	19	663929	G	A

13	F7	113113236	rs510335	13	113105441	T	G
9	FBP1	94621691	rs499206	9	94597197	G	A
12	ADGRD	131047688	rs475953	12	130971707	A	G
19	OSCAR	54098680	rs444292	19	54051696	T	C
17	CCL15	35998996	rs854628	17	36002776	A	C
3	TGFBR2	30650372	rs763774	3	30155970	T	C
7	PON3	95378124	rs149867	7	95396432	C	T
5	FLT4	180625565	rs342212	5	180630293	C	T
2	REG3A	79158378	rs147616	2	79168385	A	T
1	IGSF9	159936326	rs374761	1	159943109	T	C
16	ADGRG	57638116	rs733464	16	57648363	T	C
21	TFF3	42313538	rs117044	21	42315073	G	A
17	GFAP	44910048	rs592916	17	44914081	T	C
10	SFTPA1	79613197	rs196570	10	79557289	T	G
17	CD79B	63930538	rs129466	17	63928647	T	C
16	CES2	66939770	rs749099	16	66889149	A	G
1	CFHR5	196992344	rs727322	1	196334759	C	T
20	CST1	23749415	rs454918	20	23748236	T	C
20	LBP	38361748	rs223261	20	38369011	T	C
8	MSR1	16337684	rs413417	8	16155085	A	G
11	CTSD	1758662	rs558610	11	1762527	G	A
17	SGSH	80213820	rs750303	17	80210594	T	C
10	ADAM8	133269644	rs299531	10	133269306	T	C
21	CSTB	43774420	rs352853	21	43781951	A	G
12	TNFRSF	6335436	rs414958	12	6333477	T	C
11	PAMR1	35481062	rs617364	11	35434513	C	T
22	OSM	30264840	rs482308	22	30204116	C	G
1	IGSF8	160095142	rs124082	1	160092144	C	T
20	CST7	24954598	rs227651	20	24949198	G	A
19	TNFSF1	6665920	rs344560	19	6665009	C	T
17	CD300C	74543594	rs584403	17	74543335	T	C
6	CD83	14127087	rs125260	6	14105666	A	G
12	KITLG	88536822	rs111051	12	88970809	T	C
17	SERPIN	1769797	rs620881	17	1762959	T	C
19	BST2	17404284	rs810124	19	17377984	C	T
12	ACVRL	51915134	rs227738	12	51912437	T	C
11	IL18	112153674	rs225041	11	112214593	C	T
4	ANXA10	168140136	rs119365	4	168155041	G	A

18	SERPIN	63994904	rs38266	18	63987229	G	A
11	ADAMT	130462645	rs730448	11	130474168	A	G
16	WFIKKI	631678	rs557989	16	619708	G	T
11	LTBP3	65548744	rs40994	11	65552515	T	C
1	OPTC	203501551	rs562195	1	203503694	T	C
6	TNF	31576950	rs28577	6	31564776	T	C
6	TNF	31576950	rs28577	6	31564776	T	C
15	NTRK3	88058260	rs28735	15	87971624	C	G
2	IL18R1	102355153	rs101905	2	102377596	G	A
11	TREH	118668503	rs51414	11	118687740	A	G
1	PLA2G2	19977924	rs115731	1	19979653	C	G
6	ENPP5	46165082	rs947270	6	46148712	A	G
16	LPCAT2	55547869	rs169553	16	55532397	T	C
4	SPON2	1187947	rs288559	4	1121121	G	A
9	ORM1	114324788	rs11699	9	114322392	A	G

proteins and the risk of aSAH （UKB-PPP）

Freq	b_GWAS	se_GWAS	p_GWAS	b_eQTL
0.604374	-0.0597182	0.0174737	0.000631757	0.130044
0.336978	0.0537577	0.0180329	0.00287217	0.108306
0.542744	-0.0503729	0.0173339	0.00366033	-0.153021
0.516899	-0.0472664	0.0173039	0.00630391	-0.162078
0.0129225	0.506873	0.208099	0.0148618	-1.02753
0.468191	0.0436575	0.0174095	0.0121529	0.0623571
0.0675944	-0.0655907	0.0279432	0.0189108	-1.06768
0.450298	0.0405666	0.0174318	0.0199568	0.364573
0.198807	-0.043348	0.0190952	0.0232017	0.3988
0.239563	0.0527768	0.0238034	0.0266097	0.419583
0.0208748	0.0994042	0.0453917	0.0285299	-1.15328
0.241551	0.0444316	0.0204925	0.0301439	-0.507285
0.586481	-0.0375414	0.017421	0.0311652	-0.421503
0.101392	-0.0795139	0.0357521	0.0261463	-0.0870348
0.0328032	-0.130585	0.0612053	0.0328791	0.963008
0.706759	0.0416726	0.0197063	0.0344572	-0.309343
0.348907	0.0415183	0.0197278	0.0353297	-0.0679184
0.0119284	-0.202003	0.0986274	0.0405453	-1.49444
0.62326	0.036808	0.0179012	0.0397658	0.108951
0.0775348	0.0669742	0.0330162	0.042507	-1.08621
0.39662	-0.0351951	0.0174126	0.0432554	-0.188674
0.65507	0.0354791	0.0178035	0.0462818	-0.871962
0.422465	0.0346821	0.0175262	0.04783	0.084058
0.0308151	0.11204	0.0574373	0.0510999	0.279919
0.493042	0.0330293	0.0174582	0.0585033	-0.120466
0.348907	0.0365637	0.0194483	0.0601021	-0.280264
0.147117	0.058086	0.0306259	0.0578775	0.107855
0.257455	-0.0366273	0.0196062	0.0617419	0.215985
0.367793	0.0333718	0.0179422	0.0628912	0.950159
0.183897	0.036871	0.0198852	0.0637118	-0.19305
0.356859	-0.0320137	0.0176523	0.0697429	0.19899
0.549702	-0.0315021	0.0174155	0.0704726	0.173857
0.248509	-0.0362685	0.0201594	0.0720046	-0.444932
0.0884692	0.0551861	0.0307041	0.0722803	0.241405
0.10835	-0.0475646	0.0268017	0.0759504	0.170087
0.2833	0.031517	0.0181208	0.0819861	0.441881

0.0258449	-0.211147	0.121155	0.0813692	-0.508096
0.360835	0.0309434	0.0178354	0.0827504	0.544455
0.349901	0.0327989	0.0189503	0.0834891	0.626675
0.183897	0.0334664	0.0194603	0.085483	-0.194434
0.860835	0.0436981	0.0256787	0.0888076	-0.383042
0.83002	0.0411399	0.0246782	0.0955037	-1.4006
0.141153	-0.0466575	0.0278819	0.0942497	-0.176742
0.331014	-0.0292855	0.0176371	0.0968233	0.102159
0.173956	-0.0318471	0.0193808	0.100336	-1.16077
0.294235	0.0312635	0.0192093	0.103626	0.514434
0.0149105	-0.152945	0.0954063	0.108915	-1.75832
0.0864811	-0.0567431	0.0355535	0.110491	0.629591
0.303181	-0.0278981	0.0175686	0.112298	-0.359963
0.084493	-0.0464596	0.0296283	0.116861	-0.323609
0.0119284	-0.147676	0.0958938	0.123561	-1.69956
0.239563	-0.0346262	0.0225446	0.124563	-0.304245
0.60338	-0.0277962	0.018174	0.126154	-0.173881
0.0666004	-0.0552605	0.036344	0.128389	0.567004
0.144135	-0.0535734	0.0355295	0.131591	0.956182
0.341948	-0.0274534	0.0182787	0.133113	-0.567568
0.0477137	-0.0655776	0.0436238	0.132774	-0.350236
0.212724	-0.032094	0.021501	0.135523	-0.166377
0.508946	-0.0258401	0.0174517	0.138697	-0.115253
0.374751	0.0259431	0.0177642	0.144177	0.149894
0.223658	0.0348578	0.0239244	0.145117	-0.185468
0.0109344	-0.0708634	0.0480952	0.140644	0.237205
0.766402	0.0298735	0.0207608	0.150167	-0.283109
0.0109344	-0.14253	0.0996531	0.152643	-1.48289
0.315109	0.0273527	0.0192213	0.154725	-0.359292
0.0616302	0.0528136	0.0372807	0.156587	0.210669
0.0894632	-0.0457385	0.0324354	0.158498	0.117925
0.05666	-0.0611997	0.0442197	0.166361	-0.185387
0.140159	-0.0450582	0.0329514	0.171496	0.391818
0.0775348	-0.038994	0.028781	0.175464	-1.27797
0.647117	0.0245749	0.0180962	0.17446	0.147113
0.639165	-0.0234436	0.0175083	0.180571	-0.713462
0.388668	0.0234259	0.017592	0.182986	0.384964
0.691849	0.0248189	0.0187541	0.185707	0.343682

0.710736	-0.0240933	0.0183741	0.189767	0.124985
0.026839	0.0762573	0.0582679	0.190624	-0.391643
0.434394	0.0225457	0.0173295	0.193257	-0.768327
0.0159046	0.209629	0.161384	0.193963	-0.475495
0.187873	-0.0284033	0.0220564	0.197829	-0.302893
0.028827	-0.0895838	0.0697503	0.199019	-0.473202
0.0506958	-0.0491213	0.0388993	0.206668	-0.424659
0.437376	0.023496	0.0186372	0.207414	-0.213196
0.680915	-0.0237409	0.0189165	0.209464	-0.361265
0.290258	-0.0219503	0.0176709	0.214174	0.5192
0.302187	0.0249055	0.020137	0.216161	0.473255
0.750497	-0.0251285	0.0202442	0.214505	-0.114252
0.137177	-0.0275981	0.0223566	0.217035	0.22902
0.697813	0.0271666	0.0222443	0.221979	-0.41233
0.467197	0.0212075	0.0173864	0.222549	-0.687849
0.0457256	-0.0512478	0.0420518	0.222965	-0.825377
0.173956	0.0273599	0.0224994	0.223975	-0.666726
0.0437376	-0.0572978	0.0471685	0.224462	-0.191542
0.445328	-0.0215866	0.0178013	0.225267	0.10544
0.250497	-0.0229266	0.0190227	0.228117	-1.03731
0.683897	0.0226355	0.0188964	0.230965	-0.245434
0.502982	-0.0206365	0.0174097	0.23588	-0.462361
0.248509	0.026124	0.0221738	0.238737	-0.784611
0.134195	-0.0300109	0.0254863	0.238984	-0.31455
0.0188867	0.117374	0.100259	0.241715	-1.29767
0.379722	-0.0210532	0.01808	0.244243	-0.131286
0.151093	0.030778	0.0265008	0.245481	-0.276043
0.312127	-0.0213432	0.0185067	0.248802	0.10608
0.49006	0.0201078	0.0175592	0.252148	-0.227453
0.693837	-0.0207119	0.0178685	0.246404	0.0477177
0.276342	-0.0232155	0.020559	0.258807	-0.995641
0.0109344	-0.151107	0.132589	0.254425	-0.292396
0.435388	-0.0193907	0.0174029	0.265184	-0.375222
0.407555	-0.0215431	0.019391	0.266574	0.683173
0.431412	0.0191259	0.0173451	0.270172	0.53177
0.0675944	-0.039484	0.0358754	0.271077	-0.634584
0.494036	0.0188699	0.0173736	0.277424	0.159271
0.0636183	0.0340741	0.0313858	0.277632	0.241748

0.0337972	-0.0532227	0.0498365	0.285545	0.757723
0.482107	-0.0185715	0.0173653	0.284861	0.114794
0.0139165	0.0645919	0.0603366	0.284383	0.41237
0.76839	-0.0213873	0.0200538	0.286199	0.296985
0.342942	0.0194972	0.0183485	0.287962	-0.339217
0.0487078	0.0646295	0.0613357	0.29202	-0.450756
0.0626243	-0.0420399	0.0399584	0.292758	-0.587089
0.0188867	0.135239	0.128938	0.294237	-0.391984
0.246521	-0.0224282	0.0215115	0.297125	1.20212
0.889662	0.0316695	0.0303688	0.297026	-0.419387
0.677932	-0.0194074	0.0187334	0.300213	-0.238835
0.292247	-0.0207112	0.0200223	0.300946	-0.858309
0.0318091	-0.0599626	0.0583552	0.304163	-0.317719
0.245527	0.0219683	0.0214419	0.305574	-0.513717
0.583499	-0.0181492	0.0177218	0.305778	-0.573145
0.468191	0.0176759	0.0174215	0.310295	0.111278
0.235586	0.0229994	0.022782	0.312714	0.403289
0.581511	-0.0176961	0.0175672	0.313772	-0.256654
0.0188867	0.0811449	0.0806353	0.314261	-0.799475
0.741551	0.0222964	0.0221796	0.314769	0.674075
0.895626	0.0340885	0.0341026	0.31751	-1.47256
0.17992	-0.0188645	0.0189262	0.318891	-0.670803
0.512922	0.0172832	0.0175266	0.324078	-0.122881
0.0586481	0.028575	0.029554	0.333607	-0.369887
0.220676	-0.0218536	0.0226856	0.335384	-0.501642
0.342942	-0.0171115	0.0179211	0.339667	-0.377555
0.690855	0.0176365	0.0184439	0.338957	-0.094664
0.0298211	-0.0377991	0.0397077	0.34113	-0.149364
0.275348	0.0196711	0.0208312	0.345012	-0.101206
0.589463	-0.0168828	0.0180112	0.34858	0.33554
0.297217	0.0180457	0.0193004	0.349793	-0.103174
0.526839	-0.0162665	0.0177185	0.358591	0.122784
0.849901	-0.0220152	0.0240947	0.360878	0.377705
0.0149105	-0.107879	0.119089	0.365006	-1.56757
0.0188867	0.0415165	0.0459717	0.366478	-0.963789
0.0984095	0.0301849	0.0335135	0.367759	0.379011
0.132207	0.0260997	0.029484	0.376041	0.127738
0.682903	0.0172461	0.019765	0.382905	0.21707

0.539761	0.0150677	0.017355	0.385284	0.186665
0.667992	-0.0158119	0.0182446	0.386127	-0.588492
0.248509	-0.0196273	0.0226005	0.385149	-0.0973885
0.246521	0.0174465	0.020261	0.38919	-0.36502
0.780318	-0.0175832	0.0204526	0.389952	-0.297977
0.494036	-0.0149104	0.0173846	0.391069	-0.101995
0.975149	-0.0649923	0.0761407	0.393337	0.336036
0.393638	-0.0150509	0.0178491	0.399099	0.247734
0.0397614	-0.0346336	0.041088	0.399278	0.158736
0.272366	0.0152576	0.018214	0.402208	0.613679
0.228628	-0.0174099	0.02079	0.402356	0.226222
0.311133	0.0162214	0.0194446	0.404149	0.0692566
0.0685885	-0.0311102	0.0380556	0.413645	-0.351487
0.644135	-0.0146812	0.0183066	0.422573	0.48354
0.254473	0.0154945	0.0193832	0.42407	0.63759
0.352883	-0.0140842	0.0179347	0.432276	-0.156687
0.0228628	0.0736594	0.0940629	0.433577	-0.280448
0.0318091	-0.0364131	0.0466832	0.435389	-0.942374
0.369781	-0.0143885	0.018479	0.436191	0.254153
0.200795	-0.0159936	0.0208028	0.442002	-0.969683
0.291252	0.0152355	0.0199253	0.444491	-0.236592
0.390656	0.0144095	0.018853	0.444683	-0.231316
0.0725646	0.0240795	0.0315131	0.444801	-0.411884
0.429423	-0.0136335	0.0178893	0.445999	-0.783606
0.519881	-0.013291	0.0173869	0.444613	0.0685715
0.212724	0.0167807	0.0220597	0.446837	0.271847
0.602386	0.0132857	0.0176098	0.45058	0.091672
0.294235	-0.0139792	0.0186048	0.452425	-0.316247
0.544732	-0.0131417	0.0175265	0.453364	0.408687
0.941352	0.0452769	0.060602	0.454991	0.297273
0.347913	0.0129756	0.0173999	0.455831	-0.236988
0.641153	0.0136593	0.0183629	0.456966	-0.800073
0.159046	0.0181358	0.0251456	0.470768	0.723161
0.0178926	0.0326105	0.0454413	0.47298	-0.570654
0.0974155	-0.0176259	0.025093	0.482417	0.661309
0.428429	0.0124048	0.0177408	0.484411	0.415481
0.0516899	0.0199515	0.0293443	0.49656	-0.224706
0.196819	0.0152736	0.0225598	0.498387	0.293625

0.0218688	-0.0371047	0.0549319	0.499379	0.952949
0.60338	0.0117894	0.0174683	0.499739	-0.359092
0.0178926	0.0271135	0.040092	0.498863	-0.258273
0.248509	0.012829	0.0191049	0.5019	-0.321281
0.188867	0.014353	0.0214543	0.503494	-0.197366
0.106362	-0.0193573	0.0289816	0.504186	-0.173645
0.104374	0.0158163	0.0237647	0.505708	-1.11656
0.819085	-0.0130344	0.0195942	0.50591	0.671982
0.531809	-0.0111804	0.0173828	0.520101	0.154367
0.796223	0.0131271	0.0206384	0.524741	0.843945
0.250497	0.0158745	0.0251374	0.527707	-0.167542
0.640159	-0.0112137	0.0181241	0.536101	-0.679912
0.624254	-0.0110879	0.0179273	0.536251	0.30768
0.026839	0.0635429	0.103261	0.538317	0.746059
0.05666	-0.0559765	0.0923878	0.54459	-0.374366
0.140159	-0.0144069	0.0238003	0.544963	0.705587
0.272366	-0.01185	0.0195724	0.544885	0.0990683
0.479125	-0.0105291	0.0175226	0.547916	-0.475893
0.139165	0.0149889	0.025008	0.54893	0.940133
0.100398	-0.0211365	0.03548	0.551356	-1.33189
0.0208748	-0.0290358	0.0487818	0.551697	-0.535152
0.0178926	-0.0559206	0.0943459	0.553369	-0.480122
0.781312	0.0147777	0.025014	0.55467	0.707695
0.027833	-0.038656	0.0654734	0.554918	-0.503584
0.311133	0.0108289	0.0184131	0.556459	0.0445896
0.114314	0.0181881	0.0314218	0.562698	0.666938
0.026839	-0.0397797	0.0689324	0.563883	1.37781
0.819085	-0.0118135	0.0205619	0.565607	1.29528
0.0328032	0.0223765	0.0392012	0.568128	0.326276
0.0755467	0.0191354	0.0337684	0.570941	0.800343
0.0238569	-0.0248178	0.0439545	0.572329	-0.536629
0.446322	0.00966236	0.0174438	0.579637	0.15688
0.0536779	-0.0197036	0.0357016	0.58102	-0.548913
0.0139165	-0.0448891	0.0813389	0.581032	-0.54695
0.2833	0.0101688	0.0184939	0.582424	0.114932
0.126243	0.012342	0.0224294	0.582141	0.0729988
0.0188867	0.0213918	0.0390337	0.583668	-1.14965
0.945328	0.0220709	0.0404095	0.584942	-0.205148

0.107356	0.0118898	0.0218198	0.585816	-0.118256
0.0387674	0.025085	0.0463142	0.588075	0.321264
0.373757	-0.00988013	0.0185844	0.594977	0.265266
0.126243	0.0125414	0.0235191	0.593867	-0.0542412
0.0934394	-0.0156267	0.0298291	0.600366	-0.119677
0.148111	-0.0115674	0.0222779	0.603598	-0.303471
0.595427	-0.00879185	0.017383	0.613016	-0.0579347
0.0129225	-0.037404	0.0747331	0.616723	1.45971
0.0367793	-0.0189974	0.0382795	0.619696	-0.884485
0.315109	0.00931291	0.0189232	0.62262	-0.412969
0.380716	-0.0086684	0.0176109	0.622567	0.145118
0.115308	0.0135609	0.0279022	0.626954	0.60458
0.16501	0.0122283	0.0252054	0.627573	-0.118087
0.0437376	-0.0161804	0.0335346	0.629451	0.388365
0.0864811	-0.0127802	0.0265134	0.629785	-0.138665
0.129225	-0.0117674	0.0244938	0.630927	-0.504211
0.121272	0.0134698	0.0280543	0.631132	0.0652499
0.272366	0.00958938	0.0200756	0.63289	0.324944
0.753479	-0.00929818	0.0194782	0.633102	0.306044
0.104374	-0.0137608	0.0288108	0.632916	-0.125182
0.197813	-0.0109942	0.0230777	0.633791	0.109766
0.50497	0.00839325	0.0176892	0.635155	-0.29149
0.347913	-0.00872329	0.0185814	0.638738	0.0975401
0.323062	-0.00828006	0.0179622	0.644819	-0.212769
0.0457256	-0.0254682	0.0555366	0.646533	-0.534797
0.540755	-0.00796645	0.0174268	0.647572	0.279904
0.026839	0.0182357	0.0400977	0.649268	2.02215
0.028827	-0.0257576	0.0567319	0.649812	-0.812982
0.235586	-0.00887808	0.0196704	0.651742	0.223303
0.645129	-0.00793609	0.0175863	0.651798	-0.0914437
0.399602	0.00808349	0.0179868	0.653134	-0.44319
0.0248509	-0.0475659	0.107388	0.657814	-0.462066
0.0666004	-0.0241427	0.0548133	0.65961	-0.675826
0.0188867	0.0211869	0.0484594	0.661959	-0.192328
0.599404	0.00776279	0.017852	0.663677	0.129391
0.284294	-0.00825864	0.0189934	0.663696	-0.130572
0.284294	-0.00825864	0.0189934	0.663696	-0.120252
0.487078	-0.0073388	0.0173874	0.67297	-0.457728

0.369781	0.00748232	0.0178599	0.675257	0.81117
0.355865	0.00782917	0.0189282	0.679149	0.59708
0.101392	0.0133029	0.0323329	0.680754	-0.240654
0.285288	0.00763423	0.018693	0.68298	-0.878117
0.287276	-0.0074953	0.0184844	0.685116	-0.102519
0.287276	-0.0074953	0.0184844	0.685116	-0.102519
0.287276	-0.0074953	0.0184844	0.685116	-0.0998757
0.287276	-0.0074953	0.0184844	0.685116	-0.0998757
0.287276	-0.0074953	0.0184844	0.685116	-0.091522
0.287276	-0.0074953	0.0184844	0.685116	-0.091522
0.193837	-0.0101252	0.0250502	0.686068	-0.447313
0.67495	-0.00737717	0.0182936	0.686754	-0.694979
0.167992	-0.0107799	0.0269831	0.68952	0.133883
0.33002	0.00745386	0.0187235	0.690555	-0.28271
0.349901	-0.00676843	0.0173807	0.696965	-0.120102
0.223658	-0.00746779	0.0192677	0.698327	0.505569
0.0168986	0.0429279	0.111848	0.701124	0.964492
0.107356	0.0108415	0.0283189	0.701841	0.17467
0.434394	0.00660457	0.0173742	0.703843	0.280815
0.220676	0.00794587	0.0212347	0.708262	-0.248735
0.641153	-0.00669722	0.0180531	0.710659	0.463397
0.0506958	0.017984	0.0488678	0.712863	-0.158409
0.60835	0.00724771	0.0197761	0.714	0.0934663
0.536779	0.00646162	0.017816	0.71684	-0.111865
0.919483	-0.010204	0.0287867	0.722986	-0.79179
0.158052	-0.00910007	0.0258525	0.72484	-0.0994035
0.124254	-0.0107432	0.031685	0.734563	0.0921479
0.33002	-0.00667125	0.0201904	0.741085	0.246506
0.0705765	-0.00965378	0.0293671	0.742361	0.722608
0.109344	0.00842844	0.02567	0.742657	0.185285
0.212724	0.00772395	0.0235505	0.742932	0.832059
0.128231	-0.00721788	0.0220962	0.743928	-0.749318
0.17495	-0.0084558	0.0265866	0.750449	0.157284
0.0149105	-0.0227256	0.0715083	0.750634	-1.05518
0.0308151	-0.0237173	0.0750961	0.752135	1.05138
0.270378	-0.00575588	0.0182852	0.752925	-0.171275
0.264414	0.0060777	0.0196758	0.757405	0.260543
0.260437	0.00627647	0.0205204	0.759709	-0.366558

0.536779	0.00528318	0.0173699	0.761008	0.216076
0.188867	0.00785193	0.0259041	0.761802	0.421186
0.175944	-0.00739774	0.0244527	0.762246	0.254349
0.0705765	0.0112868	0.037485	0.763336	-1.56188
0.0129225	-0.0360437	0.120922	0.765648	-0.414673
0.026839	-0.0219211	0.0739626	0.766939	-0.590872
0.619284	0.00532284	0.0182208	0.770188	-0.56091
0.574553	0.00509065	0.0174655	0.770694	0.314756
0.574553	0.00509065	0.0174655	0.770694	0.312097
0.105368	0.00909002	0.0315483	0.773247	0.658622
0.349901	0.00504581	0.0175613	0.773863	-0.610953
0.767396	-0.00592053	0.0207559	0.775456	0.328497
0.353877	-0.00499835	0.0175754	0.776108	-0.149402
0.28827	-0.00526145	0.0188368	0.780001	0.630848
0.159046	0.00619314	0.0223215	0.781433	0.200177
0.420477	0.00490056	0.0177125	0.782033	-0.758221
0.203777	-0.00547571	0.0200572	0.784849	-0.332747
0.508946	-0.00470485	0.0173334	0.786059	-0.213858
0.779324	-0.00504624	0.019742	0.798252	-1.26315
0.22664	0.00541386	0.0216767	0.802777	-0.273837
0.0536779	0.00786441	0.0320543	0.806188	-0.140457
0.632207	-0.00451521	0.0184789	0.806964	0.232694
0.0815109	0.00645025	0.0275693	0.815012	0.092313
0.0248509	0.0151107	0.067092	0.821805	-0.200815
0.897614	-0.00785594	0.0349318	0.822062	0.537694
0.0526839	-0.0103375	0.0459612	0.822042	0.190771
0.245527	0.0045823	0.0204567	0.822757	-0.315543
0.526839	-0.00386234	0.0174226	0.824559	-0.186628
0.577535	0.00389038	0.0177716	0.82672	-0.237049
0.821074	0.00433281	0.0199084	0.827712	0.494588
0.0188867	-0.0123926	0.0578159	0.830278	0.633474
0.521869	-0.00366887	0.0173096	0.832141	0.317183
0.0258449	-0.0167668	0.0791187	0.83217	-0.473173
0.271372	-0.00378669	0.0183753	0.836733	-0.888648
0.178926	0.005038	0.0247345	0.838601	0.812228
0.323062	0.00385529	0.0196342	0.844331	0.194343
0.173956	0.00517256	0.0267949	0.846925	-0.596988
0.619284	0.00333689	0.0177614	0.850976	0.0866776

0.125249	-0.00595103	0.0318632	0.851842	-0.778851
0.955268	-0.00706758	0.0379546	0.852279	0.575477
0.94334	-0.00706102	0.0382063	0.853376	0.82369
0.470179	-0.00321106	0.0173763	0.85339	0.52103
0.934394	0.00516845	0.0281691	0.854422	-1.5711
0.342942	0.00326434	0.0179887	0.856002	0.0493714
0.026839	0.0128329	0.0720485	0.858633	-1.05099
0.124254	0.00447143	0.0264622	0.865817	-0.442515
0.0695825	-0.00809021	0.0510105	0.873985	0.406934
0.0656064	0.00611218	0.0387962	0.874815	-0.478563
0.430417	-0.0026655	0.0173594	0.877966	0.0710144
0.0109344	-0.0189648	0.128944	0.88307	-1.00049
0.0159046	-0.0099591	0.0686588	0.88467	0.277537
0.201789	-0.00290205	0.0210916	0.890562	-0.283821
0.39165	0.00243304	0.0179645	0.892267	-0.163364
0.0168986	0.00599739	0.0466943	0.897801	-0.334298
0.0208748	-0.00479862	0.0380327	0.899597	-1.58898
0.293241	-0.00270359	0.0220405	0.902373	-0.582614
0.0725646	-0.00304631	0.0250563	0.903233	-1.18791
0.0119284	0.010038	0.0894999	0.910699	-1.5469
0.0954274	-0.00428757	0.0384993	0.911325	-0.507226
0.303181	0.00195504	0.0182409	0.914647	-0.885866
0.894632	0.00317057	0.0301272	0.916186	-0.709409
0.314115	0.00181856	0.0177612	0.918447	-0.784681
0.0188867	0.00841003	0.0862196	0.922296	-0.302924
0.131213	0.0024525	0.026912	0.927389	-0.476234
0.0248509	-0.00423475	0.047618	0.929136	-0.188281
0.190855	-0.00170379	0.0197507	0.931256	0.156221
0.210736	0.00251209	0.0293812	0.931864	-0.835844
0.94831	0.00320474	0.0377126	0.932279	0.553818
0.082505	-0.00208294	0.0248917	0.933311	0.681898
0.241551	0.00172156	0.0211493	0.935124	0.199606
0.683897	-0.00130376	0.0183996	0.943511	-0.0538187
0.34493	0.00120462	0.0175534	0.945287	-0.282879
0.961233	0.00193983	0.028439	0.945618	-0.343639
0.0805169	0.0019097	0.028326	0.946249	0.568278
0.511928	0.000974919	0.0175133	0.955607	-0.29499
0.597416	0.000924563	0.0173387	0.957474	0.0503908

0.542744	-0.00080892	0.0178996	0.963954	-0.726747
0.0666004	-0.00172229	0.0408899	0.966403	0.192702
0.267396	0.000768701	0.0192809	0.968198	0.277795
0.0467197	-0.00153208	0.0388021	0.968504	0.89636
0.0109344	-0.00249262	0.0700222	0.971603	-1.29631
0.596421	-0.000630625	0.0182081	0.972371	-0.0843443
0.596421	-0.000630625	0.0182081	0.972371	-0.0843443
0.130219	-0.000876273	0.0272992	0.974393	0.27255
0.780318	0.000693573	0.0222183	0.975097	-0.897997
0.314115	0.000470114	0.0185884	0.979823	0.882864
0.21173	0.00049361	0.0196911	0.980001	0.730757
0.670974	-0.000359745	0.019353	0.985169	-0.778049
0.05666	-0.000405697	0.0559087	0.99421	-0.252858
0.131213	-0.000177506	0.0272929	0.994811	0.315157
0.0775348	8.68287e-05	0.058487	0.998815	0.695848

se_eQTL	p_eQTL	b_SMR	se_SMR	p_SMR
0.00746331	5.38397	-0.459215	0.136928	0.000797375
0.00781878	1.23653	0.49635	0.170312	0.003564136
0.00673509	2.84453	0.329189	0.114201	0.003944704
0.00682398	1.06663	0.291628	0.107467	0.006654464
0.0468091	8.37551	-0.493293	0.203766	0.01548329
0.0069075	1.75710	0.700121	0.289762	0.01568374
0.015182	0	0.0614329	0.0261865	0.01897711
0.00749325	0	0.111272	0.047869	0.02009854
0.00841222	0	-0.108696	0.0479365	0.02335963
0.0086964	0	0.125784	0.056791	0.02676978
0.0277856	0	-0.0861926	0.0394135	0.0287514
0.00877158	0	-0.0875871	0.0404248	0.03026031
0.00745807	0	0.0890656	0.0413607	0.03128835
0.0111797	6.96625	0.913587	0.427213	0.03247761
0.0186498	0	-0.135601	0.0636106	0.03302838
0.00783856	0	-0.134713	0.0637951	0.03471608
0.00772362	1.44844	-0.611297	0.298666	0.04068195
0.0327144	0	0.13517	0.0660625	0.04074778
0.00725697	6.00895	0.33784	0.165839	0.0416342
0.013405	0	-0.0616586	0.0304053	0.04257147
0.00748271	2.76689	0.186539	0.0925854	0.04392768
0.00790727	0	-0.0406888	0.0204211	0.04631725
0.00730156	1.14339	0.412597	0.211559	0.05114402
0.0194071	3.68045	0.400259	0.207061	0.05322991
0.00715573	1.35393	-0.274179	0.145834	0.06009816
0.00791054	6.06923	-0.130462	0.0694904	0.06046265
0.00984013	5.90204	0.538556	0.288174	0.06164267
0.00844675	3.27352	-0.169583	0.0910177	0.06243654
0.00797616	0	0.0351223	0.0188857	0.06292306
0.00938679	5.51947	-0.190992	0.103423	0.06479021
0.0070555	5.29291	-0.160881	0.0888927	0.07032166
0.00718448	2.28034	-0.181195	0.100451	0.07125924
0.00852085	0	0.0815147	0.0453358	0.07217393
0.012834	6.27340	0.228604	0.127768	0.0735821
0.0108576	2.61341	-0.279649	0.158584	0.07783202
0.00843269	0	0.0713246	0.0410309	0.08215537

0.024565	4.851841	0.415565	0.239294	0.08245244
0.00790221	0	0.0568337	0.0327686	0.08284836
0.00856759	0	0.052338	0.0302479	0.08357652
0.0093022	5.14149	-0.172122	0.100425	0.08654057
0.0101105	0	-0.114082	0.0671065	0.08912877
0.010261	0	-0.0293731	0.017621	0.09552832
0.00967611	1.54917	0.263986	0.158415	0.09563002
0.0077603	1.40831	-0.286666	0.174012	0.09947671
0.00927054	0	0.0274362	0.0166979	0.1003647
0.00799455	0	0.0607726	0.0373526	0.1037374
0.0335146	0	0.0869836	0.0542852	0.1090797
0.0126838	0	-0.0901269	0.0565	0.1106748
0.00784112	0	0.0775027	0.0488359	0.1125119
0.0142604	5.27233	0.143567	0.0917742	0.117735
0.0376665	0	0.0868907	0.0564556	0.1237801
0.00850111	1.64623	0.11381	0.0741684	0.1249103
0.00741571	1.39640	0.159858	0.104742	0.1269587
0.0152635	4.77401	-0.0974605	0.064152	0.128709
0.0133443	0	-0.0560285	0.0371659	0.1316766
0.00807378	0	0.0483702	0.0322127	0.1332027
0.0193388	2.63205	0.187238	0.124984	0.134107
0.00908427	6.30095	0.192899	0.129659	0.136819
0.00739874	1.03704	0.224203	0.152103	0.1404762
0.00769187	1.40636	0.173076	0.118844	0.1453012
0.00913846	1.41446	-0.187945	0.129327	0.1461522
0.0346922	8.06304	-0.298743	0.207412	0.1497723
0.00822487	1.21951	-0.105519	0.0733955	0.1505243
0.033928	0	0.0961164	0.0672379	0.1528616
0.0080763	0	-0.0761294	0.0535251	0.1549352
0.0164645	1.74020	0.250695	0.178045	0.1591181
0.012377	1.60767	-0.387861	0.278047	0.1630322
0.0147239	2.37082	0.330119	0.239963	0.1689128
0.00893373	0	-0.114998	0.0841396	0.1717038
0.0144045	0	0.0305125	0.0225235	0.175515
0.00768459	1.08842	0.167048	0.123318	0.1755417
0.00763663	0	0.0328589	0.0245424	0.1806157
0.00702899	0	0.0608522	0.0457113	0.1831137
0.00702046	0	0.0722147	0.0545881	0.185868

0.0080594	3.06332	-0.19277	0.147535	0.191348
0.0187725	1.17275	-0.194711	0.149071	0.1914953
0.00780512	0	-0.0293439	0.0225568	0.1932969
0.0307585	6.56447	-0.440865	0.340598	0.1955328
0.00869495	7.05660	0.0937734	0.0728688	0.1981365
0.0240668	4.56042	0.189314	0.147715	0.1999764
0.0168261	1.53107	0.115672	0.0917158	0.2072356
0.0075923	1.69267	-0.110208	0.0875062	0.207873
0.00780962	0	0.065716	0.0523811	0.2096331
0.0078661	0	-0.0422772	0.0340409	0.2142542
0.00806696	0	0.052626	0.0425595	0.2162623
0.00830642	4.77530	0.219939	0.177909	0.2163674
0.0115953	7.85231	-0.120505	0.097809	0.217932
0.0083239	0	-0.0658856	0.0539642	0.2221191
0.00738725	0	-0.0308316	0.0252786	0.2225898
0.0204107	0	0.0620902	0.0509717	0.2231747
0.010737	0	-0.0410362	0.0337526	0.2240631
0.0162131	3.30368	0.29914	0.247555	0.2269026
0.00731825	4.62062	-0.204729	0.169426	0.2269052
0.00907274	0	0.022102	0.0183395	0.228143
0.00821169	2.78729	-0.0922264	0.0770536	0.2313406
0.00735572	0	0.0446329	0.0376606	0.2359644
0.00850004	0	-0.0332955	0.0282632	0.2387762
0.00997192	2.19645	0.095409	0.0810811	0.2393109
0.0281617	0	-0.0904498	0.0772857	0.2418681
0.00764647	4.49270	0.160361	0.138031	0.2453256
0.00983018	1.66339	-0.111497	0.0960845	0.2458832
0.00853067	1.6846e-	-0.201199	0.175209	0.2508279
0.00726355	2.98078	-0.0884042	0.0772509	0.2524668
0.0075178	2.19128	-0.434051	0.380656	0.2541732
0.00861601	0	0.0233171	0.02065	0.2588305
0.0517596	1.61275	0.516789	0.462593	0.2639271
0.00735317	0	0.0516779	0.0463913	0.2652977
0.0072583	0	-0.0315339	0.0283857	0.2666083
0.00749023	0	0.0359665	0.0326216	0.2702288
0.0151209	0	0.0622203	0.0565532	0.2712412
0.00705196	6.03943	0.118477	0.109208	0.2779787
0.0154703	4.79730	0.140949	0.130142	0.2787894

0.0168611	0	-0.0702403	0.06579	0.2856808
0.00740937	3.86276	-0.161781	0.151634	0.2860073
0.0320336	6.3827e-	0.156636	0.146822	0.2860425
0.00737355	0	-0.0720147	0.0675483	0.2863683
0.00760275	0	-0.0574771	0.0541061	0.288098
0.0166977	1.68735	-0.14338	0.136177	0.2923871
0.0173979	1.27341	0.0716074	0.068095	0.292992
0.0232889	1.43683	-0.345012	0.329575	0.2951744
0.00830944	0	-0.0186572	0.0178951	0.2971395
0.0107109	0	-0.0755138	0.072438	0.2971982
0.00764245	2.15661	0.0812586	0.0784797	0.3004776
0.00820763	0	0.0241302	0.0233288	0.3009697
0.0190906	3.41821	0.188728	0.184019	0.3050839
0.00834852	0	-0.0427634	0.0417445	0.3056426
0.00660681	0	0.031666	0.0309224	0.3058136
0.007403	4.56671	0.158845	0.156915	0.311395
0.00966062	0	0.0570296	0.056507	0.3128559
0.00710505	9.96519	0.0689492	0.0684736	0.3139607
0.0270849	1.72335	-0.101498	0.100919	0.3145428
0.00846019	0	0.033077	0.0329064	0.3148072
0.0129523	0	-0.0231491	0.0231596	0.3175293
0.00863465	0	0.0281223	0.0282166	0.3189306
0.00765017	4.67192	-0.14065	0.142899	0.324988
0.0188574	1.15160	-0.0772533	0.0799971	0.3341935
0.00911869	0	0.0435641	0.0452296	0.3354587
0.00776603	0	0.0453219	0.0474753	0.3397597
0.00657876	6.03671	-0.186306	0.195265	0.3400231
0.0201358	1.19042	0.253067	0.268025	0.3450722
0.00745168	5.14641	-0.194367	0.206327	0.3461746
0.00700818	0	-0.0503153	0.0536885	0.3486709
0.00794441	1.45011	-0.174905	0.187551	0.3510383
0.00698419	3.48897	-0.132481	0.144503	0.3592461
0.0100817	3.49902	-0.0582868	0.0638113	0.3610201
0.034942	0	0.0688193	0.0759859	0.3651031
0.0211146	0	-0.0430763	0.0477083	0.3665729
0.0102928	7.93458	0.0796412	0.08845	0.3679024
0.0116112	3.76878	0.204322	0.231562	0.3775801
0.00796099	1.05323	0.0794495	0.0911002	0.3831478

0.00691015	1.035286	0.0807205	0.0930221	0.3855273
0.00773054	0	0.0268685	0.0310043	0.3861581
0.00872279	6.060425	0.201536	0.232766	0.3865834
0.00901944	0	-0.047796	0.0555191	0.389297
0.00854711	2.715031	0.0590086	0.068659	0.3900958
0.00641398	6.139047	0.146188	0.170693	0.3917578
0.0262031	1.198675	-0.193409	0.227086	0.3943822
0.00764338	1.885516	-0.0607543	0.0720738	0.3992592
0.0181641	2.352333	-0.218184	0.260046	0.4014587
0.00811193	0	0.0248625	0.0296818	0.4022363
0.00822244	1.235521	-0.0769594	0.0919434	0.402576
0.00774602	3.859205	0.234222	0.281981	0.4061833
0.0143282	6.886460	0.0885102	0.10833	0.4139057
0.0077249	0	-0.0303619	0.0378626	0.4226129
0.00851339	0	0.0243017	0.0304025	0.4240978
0.0078044	1.178397	0.0898875	0.114549	0.4326269
0.0225765	1.981995	-0.262649	0.336068	0.4344877
0.0214063	0	0.0386398	0.0495456	0.4354604
0.00732924	1.776098	-0.0566135	0.0727265	0.4363071
0.00935329	0	0.0164936	0.0214538	0.442013
0.00836291	4.506439	-0.0643957	0.0842487	0.4446576
0.00783623	1.652722	-0.0622936	0.0815305	0.4448363
0.01254	1.315614	-0.0584618	0.0765303	0.4449244
0.00713658	0	0.0173984	0.02283	0.4460091
0.00718733	1.420046	-0.193827	0.254371	0.4460695
0.00905546	5.343618	0.0617285	0.0811735	0.4469852
0.00761966	2.442292	0.144926	0.192473	0.4514678
0.00795324	0	0.0442034	0.0588405	0.4525071
0.00749079	0	-0.0321559	0.0428889	0.4534065
0.0166904	5.804832	0.152307	0.204039	0.4553881
0.00795681	6.240449	-0.0547521	0.073444	0.4559727
0.00684941	0	-0.0170726	0.022952	0.4569745
0.00964453	0	0.0250785	0.0347734	0.4707876
0.0262121	4.405618	-0.0571458	0.0796735	0.4732195
0.0126719	0	-0.026653	0.0379479	0.4824551
0.00762853	0	0.0298565	0.0427029	0.4844478
0.0171943	4.973911	-0.0887894	0.130766	0.4971428
0.00864012	3.852183	0.0520174	0.0768473	0.4984739

0.0236542	0	-0.0389367	0.0576522	0.4994392
0.00727519	0	-0.0328311	0.0486503	0.4997778
0.0277776	1.432849	-0.10498	0.155641	0.4999933
0.00885394	2.615074	-0.0399308	0.059475	0.5019733
0.0093962	5.918248	-0.0727228	0.108758	0.5037096
0.0123903	1.269977	0.111476	0.167091	0.5046702
0.0118907	0	-0.0141652	0.0212844	0.5057174
0.00903308	0	-0.0193969	0.02916	0.5059286
0.0072714	5.116836	-0.0724274	0.112659	0.5202938
0.00888845	0	0.0155544	0.0244552	0.5247517
0.00933287	4.645145	-0.0947494	0.150129	0.5279628
0.00792966	0	0.0164929	0.0266572	0.536113
0.0078291	0	-0.0360371	0.0582733	0.5363012
0.0265034	2.426472	0.0851714	0.138442	0.5384127
0.0176218	3.706958	0.149523	0.246885	0.5447538
0.01088	0	-0.0204183	0.0337327	0.5449811
0.00864413	2.076817	-0.119614	0.19784	0.5454439
0.00681458	0	0.0221249	0.0368218	0.5479302
0.0113707	0	0.0159434	0.0266012	0.5489405
0.0123315	0	0.0158696	0.0266392	0.5513617
0.0235983	7.464223	0.0542571	0.0911864	0.5518348
0.0283494	2.447919	0.116472	0.196624	0.5536121
0.00863854	0	0.0208815	0.0353467	0.55468
0.0187947	3.802096	0.0767618	0.130046	0.5550134
0.00780566	1.113473	0.242857	0.415129	0.5585367
0.0113394	0	0.0272711	0.0471158	0.5627181
0.0213593	0	-0.0288717	0.0500324	0.5638991
0.00956697	0	-0.00912042	0.0158746	0.5656103
0.0181519	3.069669	0.0685815	0.120208	0.5683223
0.0144	0	0.023909	0.0421946	0.5709612
0.0220963	2.760449	0.0462476	0.0819307	0.5724328
0.00739947	9.246854	0.0615908	0.11123	0.5797672
0.0155165	3.983028	0.0358957	0.0650485	0.581065
0.0475355	1.228571	0.0820717	0.148885	0.5814666
0.00781412	5.706961	0.0884767	0.161024	0.5826882
0.010337	1.642486	0.169071	0.308188	0.583282
0.0339095	5.920102	-0.0186072	0.0339571	0.5837176
0.0158249	1.966541	-0.107585	0.197152	0.5852743

0.0112612	8.531004	-0.100543	0.184761	0.5863199
0.0212574	1.328928	0.0780822	0.144255	0.5883153
0.00751973	1.370558	-0.0372461	0.0700674	0.5950202
0.00941939	8.488293	-0.231215	0.435457	0.5954385
0.0141916	3.371326	0.130574	0.249727	0.6010672
0.0101237	1.999448	0.038117	0.0734213	0.6036532
0.00770134	5.366607	0.151754	0.300722	0.6138162
0.0240298	0	-0.0256243	0.051199	0.6167342
0.0246965	6.642891	0.0214785	0.043283	0.6197296
0.00784921	0	-0.0225511	0.0458243	0.6226339
0.00771082	5.174949	-0.0597335	0.121397	0.6226843
0.0117337	0	0.0224303	0.0461534	0.6269715
0.00958377	6.934288	-0.103553	0.213613	0.6278392
0.0134428	1.585923	-0.0416629	0.0863602	0.6294995
0.0130306	1.910279	0.092166	0.191401	0.6301369
0.0109417	0	0.0233382	0.0485811	0.6309448
0.0117467	2.779843	0.206434	0.431555	0.6324014
0.00823868	0	0.0295109	0.0617863	0.6329147
0.00885068	5.301781	-0.0303818	0.0636512	0.6331357
0.0105363	1.484558	0.109926	0.230337	0.6331905
0.00904368	6.698836	-0.10016	0.210406	0.6340511
0.00715004	0	-0.0287943	0.0606896	0.6351774
0.00777039	3.837049	-0.0894329	0.190633	0.6389727
0.00799018	3.149459	0.0389157	0.0844338	0.6448687
0.0178111	4.486337	0.0476222	0.103858	0.6465713
0.00751661	1.653198	-0.0284614	0.0622646	0.6475967
0.0271952	0	0.00901798	0.0198296	0.6492724
0.0201768	0	0.0316829	0.0697869	0.6498329
0.00870418	3.749769	-0.039758	0.088102	0.6517933
0.00781182	1.190140	0.0867866	0.192461	0.6520396
0.00760364	0	-0.0182393	0.0405861	0.6531439
0.0264537	2.558616	0.102942	0.232483	0.6579157
0.0163359	0	0.0357232	0.0811102	0.6596269
0.0323959	2.906220	-0.11016	0.252645	0.662816
0.00780314	9.418861	0.0599948	0.138017	0.663786
0.00804994	3.627368	0.0632497	0.145515	0.6638091
0.00809709	6.828045	0.0686778	0.158014	0.663831
0.00739466	0	0.0160331	0.0379872	0.6729769

0.00800364	0	0.00922411	0.0220176	0.6752588
0.00727236	0	0.0131124	0.0317017	0.6791529
0.0112025	2.28560	-0.0552781	0.134379	0.6808086
0.00859842	0	-0.00869386	0.0212878	0.6829821
0.00861945	1.27292	0.0731113	0.180407	0.685288
0.00861945	1.27292	0.0731113	0.180407	0.685288
0.00855767	1.79512	0.0750463	0.185186	0.6852947
0.00855767	1.79512	0.0750463	0.185186	0.6852947
0.00854572	9.16638	0.0818962	0.202111	0.6853283
0.00854572	9.16638	0.0818962	0.202111	0.6853283
0.00980458	0	0.0226356	0.0560037	0.68608
0.00772634	0	0.010615	0.0263228	0.6867562
0.00886503	1.56208	-0.0805173	0.201613	0.6896236
0.00794113	1.36513	-0.0263657	0.0662328	0.6905735
0.0078494	7.55261	0.0563557	0.144763	0.6970571
0.00873697	0	-0.0147711	0.0381118	0.6983327
0.0255817	0	0.0445083	0.115972	0.7011372
0.0115963	2.85364	0.0620685	0.16218	0.7019329
0.00761526	1.15000	0.0235193	0.0618739	0.703859
0.00879671	6.83664	-0.0319451	0.0853783	0.7082853
0.00792451	0	-0.0144524	0.038959	0.7106629
0.0187264	2.69400	-0.113529	0.308783	0.7131227
0.00762912	1.65425	0.0775436	0.21168	0.714123
0.00738469	7.78399	-0.0577627	0.159309	0.7169168
0.013161	0	0.0128873	0.0363571	0.7229919
0.0093201	1.47603	0.0915468	0.260218	0.7249822
0.0112685	2.89868	-0.116586	0.344145	0.7347818
0.00825123	4.15002	-0.0270632	0.0819113	0.7411002
0.0150591	0	-0.0133596	0.0406414	0.7423673
0.0117466	4.73803	0.0454891	0.138573	0.7427098
0.00867344	0	0.00928294	0.028304	0.7429328
0.010568	0	0.0096326	0.0294887	0.7439299
0.00978755	4.15422	-0.0537613	0.169069	0.7504962
0.0338526	2.73201	0.0215372	0.0677723	0.7506464
0.017783	0	-0.0225583	0.0714272	0.752138
0.00843502	1.15607	0.0336061	0.106772	0.7529547
0.00813913	7.62624	0.0233271	0.075522	0.7574145
0.00799964	0	-0.0171227	0.0559826	0.7597127

0.00737671	1.32495	0.0244506	0.0803923	0.7610206
0.00968701	0	0.0186424	0.0615043	0.7618076
0.00908574	1.90591	-0.029085	0.096144	0.7622595
0.0133386	0	-0.00722642	0.024	0.7633376
0.0398184	2.13795	0.0869208	0.291728	0.7657398
0.0207643	4.09026	0.0370996	0.125182	0.7669515
0.00763461	0	-0.00948965	0.0324846	0.7701891
0.00743745	0	0.0161733	0.0554903	0.770698
0.00764699	0	0.0163111	0.0559632	0.7706984
0.0137058	0	0.0138016	0.0479013	0.773251
0.0076408	0	-0.00825892	0.0287443	0.7738645
0.00848367	0	-0.0180231	0.0631862	0.7754619
0.00789552	7.46304	0.0334557	0.117652	0.7761326
0.00771611	0	-0.00834028	0.0298597	0.7800023
0.0100316	1.36394	0.0309383	0.11152	0.7814538
0.00733973	0	-0.00646323	0.0233607	0.7820324
0.0092402	5.66796	0.0164561	0.0602794	0.7848556
0.00680137	5.17183	0.0219999	0.081054	0.7860661
0.00920185	0	0.00399497	0.0156292	0.7982528
0.00805297	1.91330	-0.0197704	0.0791613	0.8027822
0.0175678	1.29418	-0.0559916	0.228322	0.8062775
0.0075188	2.67888	-0.0194041	0.0794154	0.8069702
0.0137278	1.76157	0.0698737	0.298831	0.815122
0.0285265	1.92796	-0.0752469	0.334269	0.821895
0.0114898	0	-0.0146104	0.0649667	0.822064
0.017125	8.01858	-0.054188	0.240973	0.8220787
0.00871286	3.43697	-0.014522	0.0648314	0.8227607
0.0074675	7.46449	0.0206954	0.0933584	0.8245654
0.00740413	6.57684	-0.0164117	0.0749719	0.8267242
0.00905069	0	0.00876044	0.0402528	0.8277131
0.0277577	2.79901	-0.0195629	0.091272	0.8302847
0.00752294	0	-0.011567	0.0545736	0.8321436
0.0224177	6.84378	0.0354348	0.167217	0.8321781
0.00808145	0	0.00426118	0.0206779	0.8367326
0.0094418	0	0.00620269	0.0304527	0.8386016
0.00785385	3.51555	0.0198376	0.101032	0.8443365
0.00935611	0	-0.00866443	0.0448837	0.8469262
0.00756545	2.16819	0.0384977	0.204941	0.8509959

0.0116972	0	0.00764078	0.0409107	0.851843
0.0141736	0	-0.0122813	0.065954	0.8522805
0.0153217	0	-0.00857242	0.0463846	0.8533766
0.00760453	0	-0.00616291	0.03335	0.8533901
0.0139486	0	-0.0032897	0.0179296	0.8544221
0.00756249	6.64508	0.066118	0.364495	0.8560567
0.0231471	0	-0.0122103	0.0685535	0.8586339
0.010485	0	-0.0101046	0.0598	0.865818
0.0153062	9.79764	-0.0198809	0.125355	0.8739871
0.0166669	2.59737	-0.0127719	0.0810693	0.8748166
0.00735137	4.45760	-0.0375346	0.24448	0.8779815
0.0379244	2.25392	0.0189555	0.128883	0.8830724
0.0239523	4.79183	-0.0358839	0.247406	0.8846787
0.00988595	2.90110	0.0102249	0.0743139	0.8905636
0.00742788	3.33434	-0.0148934	0.109968	0.8922692
0.0329197	3.14917	-0.0179403	0.13969	0.8978093
0.0295918	0	0.00301994	0.0239354	0.8995969
0.00856971	0	0.00464045	0.0378304	0.9023728
0.0137197	0	0.00256443	0.0210928	0.9032329
0.0334062	0	-0.00648911	0.0578578	0.9106995
0.0122086	0	0.00845298	0.0759019	0.9113253
0.00737931	0	-0.00220693	0.020591	0.9146471
0.0122509	0	-0.00446931	0.0424681	0.9161859
0.00814402	0	-0.00231758	0.0226349	0.9184476
0.028173	5.78095	-0.0277628	0.284636	0.9222992
0.0099184	0	-0.00514978	0.0565101	0.9273891
0.0247745	2.96620	0.0224916	0.252927	0.929141
0.00913207	1.31946	-0.0109063	0.12643	0.9312568
0.00901317	0	-0.00300545	0.0351516	0.931864
0.0167576	1.62045	0.00578663	0.0680959	0.9322791
0.0125267	0	-0.00305462	0.0365036	0.9333109
0.00881485	1.58851	0.00862479	0.105956	0.935124
0.00774843	3.76444	0.024225	0.341899	0.9435137
0.00769371	6.09232	-0.00425843	0.0620528	0.9452874
0.0182846	8.46662	-0.00564496	0.0827589	0.9456187
0.0133099	0	0.0033605	0.0498454	0.9462485
0.00736184	0	-0.00330492	0.0593692	0.9556069
0.00769962	5.96624	0.0183479	0.344096	0.9574754

0.00755829	0	0.00111307	0.0246298	0.9639542
0.0173208	9.43195	-0.00893758	0.212194	0.9664031
0.00807564	2.59803	0.00276715	0.069407	0.968198
0.0162699	0	-0.00170922	0.0432885	0.9685042
0.0421197	5.35552	0.00192286	0.0540166	0.9716033
0.00775377	1.46994	0.00747679	0.215879	0.9723715
0.00775377	1.46994	0.00747679	0.215879	0.9723715
0.0106007	8.91257	-0.00321509	0.100162	0.9743932
0.00889522	0	-0.000772356	0.0247421	0.975097
0.00808011	0	0.000532487	0.0210547	0.9798231
0.00880018	0	0.000675478	0.0269462	0.980001
0.00740822	0	0.000462368	0.0248738	0.9851693
0.0179781	6.25458	0.00160445	0.221107	0.9942103
0.0115812	4.58156	-0.00056323	0.086601	0.9948108
0.0149968	0	0.000124781	0.0840514	0.9988155

p_HEIDI	nsnp_HE	or	or_lci95	or_uci95
0.09327124	20	0.631779397725	0.48306	0.826267985853925
0.1632984	20	1.642714407256	1.17649	2.29368918702359
0.4505877	20	1.389840510622	1.11110	1.73850049987832
0.1979836	20	1.338604963960	1.08436	1.6524570805984
0.01119048	19	0.610612333439	0.40955	0.910363198379255
0.135819	20	2.013996386290	1.14132	3.55393172926657
0.1266842	20	1.063359142736	1.01015	1.11936150732218
0.1388895	20	1.117698879607	1.01760	1.22764198001777
0.4662396	20	0.897003064985	0.81656	0.985367648737806
0.3532045	20	1.134037189793	1.01457	1.26756071916186
0.1487585	13	0.917417519589	0.84921	0.991097722028124
0.006302089	20	0.916139072462	0.84635	0.991680309783778
0.5750012	20	1.093152364769	1.00803	1.18546199997164
0.2565408	17	2.493249799792	1.07923	5.7599251509694
0.6689908	20	0.873190966187	0.77083	0.989135228646718
0.003293189	20	0.873966704141	0.77124	0.99037204442506
0.1147146	20	0.542646599814	0.30219	0.974421185660935
0.1863363	20	1.144731372144	1.00570	1.30297811220389
0.1949735	20	1.401916178871	1.01287	1.94038118327777
0.5890853	20	0.940203817588	0.88580	0.997937917020422
0.3989025	20	1.205071618962	1.00508	1.4448512628749
0.6150917	20	0.960127875255	0.92245	0.999336776030309
0.03581634	20	1.510736076600	0.99794	2.28702681513925
0.1929053	20	1.492211130278	0.99443	2.23915499428087
0.7526653	20	0.760195987999	0.57120	1.01172383165349
0.8346421	20	0.877689844529	0.76593	1.00575568466818
0.193149	20	1.713530736468	0.97407	3.01432836242977
0.3453678	20	0.844016698187	0.70611	1.00885062924158
0.4724577	20	1.035746372835	0.99810	1.07480394908127
0.07765889	20	0.826139197238	0.67455	1.01178599387531
0.1567958	20	0.851393380891	0.71525	1.01343818354406
0.2463038	20	0.834272659664	0.68517	1.01581267788782
0.4540904	20	1.084929165925	0.99268	1.1857468959767
0.06370375	20	1.256844230135	0.97841	1.61450640761417
0.2800565	20	0.756049068111	0.55406	1.03166668991172
0.1378172	20	1.073929766899	0.99094	1.16386360429198

0.8191998	15	1.515226601918	0.94795	2.42196995495932
0.1049455	20	1.058479770572	0.99263	1.12869280796972
0.3791363	20	1.053731843692	0.99307	1.11809224916626
0.2907871	20	0.841876457975	0.69145	1.02501884726814
0.5037867	20	0.892184793729	0.78222	1.01759982334087
0.007918918	20	0.971054096587	0.93808	1.00517741673961
0.6745927	19	1.302109966633	0.95455	1.77620559354277
0.9160014	20	0.750762441621	0.53380	1.05590426180015
0.9409518	20	1.027816038352	0.99472	1.06201079122825
0.01140992	20	1.062657238453	0.98763	1.14337417792008
0.7283987	6	1.090878789159	0.98077	1.21334692155303
0.1066335	20	0.913815214762	0.81802	1.02082701725008
0.5987776	20	1.080585150034	0.98194	1.18912894963235
0.5254083	20	1.154384151954	0.96434	1.3818793658619
0.182619	20	1.090777451227	0.97651	1.21840627892018
0.1119706	20	1.120539202208	0.96893	1.29586712283894
0.5444542	20	1.173344244278	0.95558	1.44073344395475
0.2027378	20	0.907138172805	0.79995	1.02868102152948
0.2811297	20	0.945512188409	0.87908	1.01695886006357
0.04181816	20	1.049559130203	0.98534	1.1179616725616
0.988417	20	1.205914258492	0.94390	1.54065344289781
0.0897085	20	1.212760298543	0.94060	1.56366220916252
0.02357625	20	1.251325012624	0.92874	1.68594271140201
0.2764088	20	1.188956462341	0.94189	1.50081792075484
0.7694753	20	0.828660282294	0.64311	1.06773108952542
NA	NA	0.741750014694	0.49397	1.11380771623996
0.4555178	20	0.899857375394	0.77928	1.03908049224808
0.2950901	3	1.100887199806	0.96495	1.25596309418044
0.09858454	20	0.926696284301	0.83440	1.02919793601819
0.0262964	20	1.284918124532	0.90640	1.82150521411227
0.08041365	20	0.678506649133	0.39343	1.17012563103715
0.1043131	20	1.391133663520	0.86917	2.22653480986379
0.5547457	20	0.891367926640	0.75585	1.05118238965859
0.7389804	20	1.030982777252	0.98645	1.07751618094356
0.6730383	20	1.181810990874	0.92806	1.50493736591111
0.2918712	20	1.033404715548	0.98487	1.08432969559292
0.2992923	20	1.062741829336	0.97166	1.16235294087018
0.1285342	20	1.074886097336	0.96581	1.19626879588482

0.1064026	20	0.824671626802	0.61758	1.10119791401389
0.169802	20	0.823072491637	0.61453	1.10237634134183
0.5877966	20	0.971082451781	0.92908	1.0149784979666
0.1902378	7	0.643479570451	0.33007	1.25446235700364
0.9864779	20	1.098310840480	0.95213	1.26692948918158
0.9908171	19	1.208420336902	0.90464	1.61419341745411
0.1146797	11	1.122627589888	0.93791	1.34371070274853
0.09603885	20	0.895647821173	0.75448	1.06322224618629
0.3438318	20	1.067923383853	0.96372	1.18338971007351
0.3691966	20	0.958604018705	0.89673	1.02474414213348
0.5734827	20	1.054035362167	0.96967	1.14573072359773
0.4045614	18	1.246000722225	0.87918	1.76586497294929
0.7905996	20	0.886472654971	0.73182	1.07379665081622
0.7917727	20	0.936237963788	0.84226	1.040690289
0.8996829	20	0.969638846509	0.92276	1.01889066895846
0.2362623	20	1.064058318459	0.96289	1.17585404690556
0.08950215	20	0.959794384762	0.89835	1.02543703363835
0.9761333	19	1.348698428036	0.83021	2.19097751911153
0.09171942	20	0.814868115751	0.58461	1.13580989460887
0.8812113	20	1.022348058654	0.98625	1.05976524858687
0.7416647	20	0.911898672313	0.78407	1.06056168151024
0.3230044	20	1.045643933555	0.97123	1.1257479689242
0.1367506	20	0.967252694183	0.91512	1.02234639427306
0.05543719	15	1.100108707586	0.93846	1.28959466523215
0.7785066	20	0.913520191463	0.78511	1.06293098445704
0.3208374	20	1.173934585	0.89567	1.53864438610733
0.4873691	20	0.894494074881	0.74094	1.07985699464166
0.160851	20	0.817749683173	0.58006	1.15281945285351
0.8823759	20	0.915390800974	0.78677	1.06503489512478
0.04925465	20	0.647879212802	0.30723	1.36620218139195
0.05890188	20	1.023591068818	0.98298	1.0658697153195
NA	NA	1.676635320932	0.67712	4.15150649826522
0.3372548	20	1.053036504823	0.96151	1.15327399309019
0.1262223	20	0.968958108217	0.91652	1.02439487458801
0.09973012	20	1.036621119101	0.97241	1.10506575059456
0.9339128	14	1.064196761452	0.95253	1.18894287502474
0.3547401	20	1.125780980841	0.90885	1.39448431463312
0.4699857	5	1.151365926825	0.89214	1.48590991205566

0.09318718	20	0.932169792588	0.819395	1.06046564563499
0.3921705	20	0.850627471559	0.631925	1.14501946859341
NA	NA	1.169569812997	0.877099	1.55956528661254
0.1393095	20	0.930517217107	0.815128	1.06224008711567
0.1810709	20	0.944143511025	0.849145	1.04976975167069
0.4045853	20	0.866424764921	0.663459	1.13148046358618
0.6982043	20	1.074233517185	0.940015	1.22761541413277
0.2230621	13	0.708211854874	0.371213	1.35114853848573
0.745653	20	0.981515768184	0.947680	1.0165526986713
0.002578581	20	0.927266934162	0.804533	1.06872321610456
0.5567089	20	1.084651351141	0.930008	1.26500846265126
0.3502726	20	1.024423689172	0.978637	1.07235230137406
0.6738613	20	1.207712410027	0.842021	1.73222245480171
0.4699328	20	0.958138058715	0.882865	1.03982852525848
0.6273208	20	1.032172702043	0.971472	1.09666516192131
0.6545082	20	1.172156248380	0.861820	1.59424201886451
0.1899294	20	1.058687147071	0.947693	1.18268031971058
0.8520674	20	1.071381781571	0.936824	1.2252653784451
0.1924553	20	0.903482986306	0.741337	1.10109290878753
0.389412	20	1.033630125696	0.969068	1.10249252029177
0.6457551	20	0.977116784799	0.933754	1.02249295199844
0.7988342	20	1.028521464904	0.973183	1.08700568269968
0.4079507	20	0.868793336157	0.656560	1.14962067484214
0.1208421	19	0.925655355407	0.791324	1.08278997033208
0.6430686	20	1.044526946343	0.955915	1.14135267497542
0.8225349	20	1.046364630477	0.953391	1.14840438544391
0.9941086	20	0.830019570129	0.566070	1.21702992152326
0.571041	20	1.287969567904	0.761654	2.1779759887479
0.4369263	20	0.823355677279	0.549488	1.23371990702626
0.6712507	20	0.950929549141	0.855948	1.05644992512158
0.6355486	20	0.839536772974	0.581290	1.21251287217506
0.6054584	20	0.875919576420	0.659873	1.16269999221872
0.7096304	20	0.943379347437	0.832470	1.06906383826419
0.1901909	10	1.071242618116	0.923011	1.24327827852435
0.9184891	20	0.957838304216	0.872332	1.05172530994478
0.3363261	18	1.082898453996	0.910530	1.28788739806771
0.4110789	20	1.226693085042	0.779159	1.93128101291056
0.3684708	14	1.082690882259	0.905645	1.29434644489102

0.1904033	20	1.084067857252	0.90338	1.30088371722395
0.5666708	20	1.027232712779	0.96666	1.09159172368168
0.6869121	10	1.223280274360	0.77516	1.93045815354143
0.3614302	20	0.953328246204	0.85503	1.06292169873252
0.1975884	20	1.060784363446	0.92722	1.21358676084648
0.5394993	20	1.157413761385	0.82830	1.61728089633299
0.4462581	12	0.824144829962	0.52808	1.28618382649859
0.9799187	20	0.941054428446	0.81707	1.08384006216118
0.3606271	20	0.803977496196	0.48293	1.33844187826703
0.8716797	20	1.025174149387	0.96723	1.08658399761263
0.2776131	20	0.925927445497	0.77323	1.10876819402534
0.1393331	20	1.263925052426	0.72726	2.19658692945151
0.1049773	20	1.092545396516	0.88354	1.35098911236451
0.9827791	20	0.970094392852	0.90070	1.04482436132139
0.5428708	20	1.024599392901	0.96532	1.08750991371617
0.527376	20	1.094051196022	0.87404	1.36944224978476
0.1982583	20	0.769011773077	0.39798	1.48593511330148
0.6072437	9	1.039396025765	0.94320	1.14539456511553
0.998342	20	0.944959225536	0.81942	1.08973052401344
0.07879143	20	1.016630370330	0.97476	1.06029062810314
0.3992855	13	0.937633904378	0.79491	1.10597992506543
0.6961588	20	0.939606977680	0.80084	1.10241825448709
0.2278365	20	0.943214270496	0.81183	1.09585796702767
0.09838109	13	1.017550633754	0.97302	1.06411684327681
0.763766	20	0.823800409412	0.50037	1.35627254335898
0.3386666	20	1.063673518161	0.90721	1.24710960776454
0.8364009	19	1.155954026504	0.79269	1.68568680472223
0.9563542	20	1.045194925917	0.93134	1.17296036538514
0.9784029	20	0.968355603672	0.89028	1.05327709217449
0.09122926	20	1.164517688490	0.78066	1.73711108946337
0.6409077	20	0.946719810706	0.81979	1.09329726078587
0.8788208	20	0.983072310994	0.93982	1.02830654694677
0.006226787	20	1.025395610920	0.95783	1.09771897093242
0.334192	4	0.944456357590	0.80790	1.10408204365657
0.9283192	20	0.973699056482	0.90390	1.04888205139206
0.2445069	20	1.030306674337	0.94758	1.12025258701411
0.769871	11	0.915038260341	0.70815	1.18235943111915
0.7108545	20	1.053394071410	0.90610	1.22462920453345

0.2838668	20	0.961811589893	0.85904	1.07687315176096
0.5195373	20	0.967701990653	0.87969	1.06451946205735
0.6686384	14	0.900342529256	0.66362	1.22149602803578
0.05669742	20	0.960855928082	0.85512	1.07965354715412
0.03987409	20	0.929858551094	0.75134	1.15078334494282
0.6411934	20	1.117926913437	0.80571	1.55111551627601
0.7397183	20	0.985934654403	0.94565	1.02793529659844
0.6330268	20	0.980790009426	0.92630	1.03847854031084
0.02065819	20	0.930133271884	0.74584	1.1599585132047
0.5596119	20	1.015675999329	0.96814	1.06554519887566
0.4348989	20	0.909600851882	0.67773	1.22079640896342
0.104439	20	1.016629658689	0.96487	1.07115875595749
0.05334652	20	0.964604505992	0.86049	1.08131572981865
0.3574284	18	1.088903688796	0.83012	1.42834684060043
0.2063994	20	1.161280179948	0.71578	1.88403847891848
0.3966196	20	0.979788741944	0.91710	1.04675802450302
0.9155384	20	0.887262854088	0.60207	1.30754639490191
0.7099594	20	1.022371470692	0.95118	1.09888455894729
0.01692026	20	1.016071174150	0.96445	1.0704528165604
0.08287385	20	1.015996190864	0.96430	1.07045354446857
0.4834403	14	1.055756002133	0.88296	1.26235931715255
0.9373661	18	1.123526051297	0.76421	1.65178255312894
0.03512507	20	1.021101043994	0.95275	1.09435049496591
0.7299909	20	1.079784841007	0.83683	1.39326785117525
0.8486641	13	1.274886302341	0.56507	2.87631465201726
0.04169088	20	1.027646359931	0.93699	1.12706630667653
0.9855345	20	0.971541105194	0.88079	1.07164173460868
0.5659863	20	0.990921044875	0.96056	1.02223744248967
0.8040853	20	1.070987906908	0.84617	1.35552547014314
0.1207869	20	1.024197111712	0.94290	1.11250075754795
0.447728	20	1.047333698679	0.89195	1.22977567167635
0.3284761	20	1.063527060401	0.85519	1.32260278241153
0.7906194	20	1.036547728924	0.91247	1.17749671841989
0.7420379	11	1.085533639801	0.81079	1.45337189267793
0.7721763	20	1.092508796858	0.79681	1.49792937831239
0.6054435	20	1.184204214438	0.64727	2.16651412086443
0.935089	13	0.981564845200	0.91836	1.04911685103624
0.7838145	20	0.898000189190	0.61017	1.3215865147471

0.2834335	20	0.904346224689	0.62959	1.29899197520747
0.3995464	20	1.081211530605	0.81492	1.43450809571069
0.004334512	20	0.963439003832	0.83981	1.10526597128271
0.01165281	12	0.793568830394	0.33799	1.86317257466549
0.7159567	20	1.139482258460	0.69845	1.85900039273367
0.3551291	20	1.038852771542	0.89961	1.19964148301394
0.860122	5	1.163873888236	0.64554	2.09838734758735
0.3855176	14	0.974701216074	0.88163	1.07758857091214
0.9488631	19	1.021710823318	0.93860	1.1121701314122
0.9081254	20	0.977701275381	0.89371	1.06957837418416
0.6870628	20	0.942015547278	0.74254	1.19506983140701
0.9324863	20	1.022683750622	0.93423	1.11950985941647
0.3662263	20	0.901628235179	0.59319	1.37043537318326
0.06474416	20	0.959193070062	0.80983	1.13610198523001
0.4654766	16	1.096546833747	0.75353	1.59569912007662
0.6034441	20	1.023612666817	0.93064	1.12587122255861
0.4804715	20	1.229286598624	0.52760	2.86417915138517
0.023285	20	1.029950661872	0.91247	1.16254555871043
0.5913719	20	0.970075088166	0.85629	1.09897129073478
0.690092	20	1.116195468937	0.71067	1.75310152628863
0.329563	20	0.904692655630	0.59896	1.36647681563023
0.2764913	20	0.971616305386	0.86265	1.09434642836703
0.7040392	20	0.914449622635	0.62934	1.32870898122504
0.3426597	17	1.039682834692	0.88110	1.22679606779584
0.1580017	17	1.048774353517	0.85561	1.28554644888019
0.9821773	20	0.971939810298	0.86027	1.09809539060147
0.8965756	20	1.009058764487	0.97059	1.0490489543908
0.3093012	20	1.032190145909	0.90023	1.1834870691829
0.2737193	20	0.961021978332	0.80861	1.14216081493279
0.8966198	5	1.090663907204	0.74793	1.59043912921951
0.7224637	20	0.981926029342	0.90684	1.06322788553204
0.09292632	16	1.108427118438	0.70277	1.74823844829825
0.128015	20	1.036368939862	0.88404	1.21494546620098
0.8791611	9	0.895690813300	0.54588	1.46964988653806
0.02844296	20	1.061831025009	0.81016	1.39167508678384
0.5188927	20	1.065292809638	0.80094	1.41688259124035
0.3051059	20	1.071091048010	0.78581	1.45992525616174
0.05942861	20	1.016162319822	0.94325	1.09470838419887

0.2278933	20	1.009266783209	0.96663	1.05377463239602
0.2490309	20	1.013198744498	0.95215	1.07815121035932
0.8649438	20	0.946221967030	0.72712	1.23134213356997
0.02444907	20	0.991343822320	0.95083	1.0335817818792
0.6095619	20	1.075850272386	0.75541	1.53220675533058
0.6095619	20	1.075850272386	0.75541	1.53220675533058
0.731775	20	1.077934058076	0.74982	1.54962173622018
0.731775	20	1.077934058076	0.74982	1.54962173622018
0.6515442	20	1.085343145363	0.73034	1.61289993396626
0.6515442	20	1.085343145363	0.73034	1.61289993396626
0.410098	20	1.022893729150	0.91655	1.14156810964339
0.07205729	20	1.010671538989	0.95985	1.06418333988771
0.9862963	20	0.922638941791	0.62146	1.36977183202149
0.3297748	20	0.973978840410	0.85540	1.1089909945482
0.34129	20	1.057973938145	0.79661	1.4050756995253
0.9851366	20	0.985337457535	0.91441	1.06176012679551
0.7454846	20	1.045513654437	0.83293	1.31234212167394
0.9464033	20	1.064035228644	0.77429	1.46219875584807
0.2777828	20	1.023798059858	0.90687	1.15579930547821
0.005242056	20	0.968559754550	0.81931	1.14499053202704
0.9720173	20	0.985651534628	0.91318	1.06386365615818
NA	NA	0.892678308364	0.48736	1.63507010319188
0.4687421	20	1.080629346870	0.71365	1.6362980438819
0.0424407	20	0.943873902140	0.69072	1.2897945472606
0.111394	20	1.012970699128	0.94329	1.08778902197048
0.9612444	20	1.095868062117	0.65804	1.82498921969396
0.1984123	20	0.889953557679	0.45334	1.74706668225018
0.01698363	20	0.973299727037	0.82893	1.14280177891303
0.8498074	20	0.986729243378	0.91117	1.06854460890737
0.4116375	20	1.046539597270	0.79762	1.37312914113602
0.1935302	20	1.009326160120	0.95485	1.06690163532927
0.3204542	20	1.009679142814	0.95297	1.06975585763029
0.3158275	20	0.947658285539	0.68035	1.3199765086993
0.2260814	9	1.021770799504	0.89467	1.16692362782887
0.7558788	11	0.977694235957	0.84996	1.12461303995209
0.9900118	20	1.034177164102	0.83889	1.27491463062989
0.5220678	20	1.023601304780	0.88276	1.18690635458428
0.341847	20	0.983023060305	0.88086	1.0970263444498

0.337365	20	1.024751967109	0.87536	1.19963759618183
0.8054514	20	1.018817254417	0.90311	1.14934340598156
0.3201055	20	0.971333897576	0.80450	1.17275682750483
0.8069933	20	0.992799627791	0.94717	1.04061676433208
0.4907935	15	1.090810284122	0.61577	1.9322935785163
0.7724993	15	1.037796380210	0.81199	1.32638383738538
0.3815956	20	0.990555234636	0.92945	1.05567478172534
0.7056009	20	1.016304795766	0.91156	1.13307399406213
0.7215353	20	1.016444852217	0.91085	1.13428100224156
0.9341898	20	1.013897281761	0.92303	1.11370038153484
0.5659598	20	0.991775091183	0.93744	1.04925449580291
0.2115677	20	0.982138344700	0.86773	1.11162382530461
0.5272038	20	1.034021635553	0.82107	1.30219801828669
0.3716248	20	0.991694403644	0.93532	1.0514653177271
0.866737	20	1.031421863194	0.82891	1.28340602386472
0.1175086	20	0.993557611745	0.94909	1.04010715552055
0.9983167	20	1.016592247406	0.90330	1.14408332174667
0.1155946	20	1.022243682246	0.87208	1.19825429087191
0.07197079	20	1.004002960529	0.97371	1.03523473903306
0.03760412	20	0.980423752760	0.83951	1.14497837229216
0.7217596	6	0.945547078452	0.60440	1.47922680173333
0.9153572	20	0.980782947763	0.83940	1.14596844613515
0.6449445	8	1.072372732024	0.59700	1.92626416251204
0.118892	16	0.927514454736	0.48170	1.78589616059538
0.3620368	20	0.985495813988	0.86766	1.11932332879099
0.4597103	12	0.947254006007	0.59067	1.51910155824576
0.4448523	20	0.985582935669	0.86797	1.11912546334898
0.5437449	20	1.020911034770	0.85019	1.22590318229488
0.5451631	20	0.983722238229	0.84928	1.13943579587958
0.2038914	20	1.008798924954	0.93226	1.09161246412146
0.5466175	13	0.980627211797	0.81999	1.17272514004345
0.1728065	20	0.988499640553	0.88822	1.10009578798561
0.1316156	9	1.036070094155	0.74653	1.43789482953434
0.7595084	20	1.004270271736	0.96438	1.04580807489153
0.7747984	20	1.006221966516	0.94792	1.06810919946367
0.8831255	20	1.020035672781	0.83678	1.2434133755084
0.6643625	20	0.991372997998	0.90788	1.08253728313873
0.8280392	20	1.039248338084	0.69545	1.55298993

0.9261453	20	1.007670045248	0.93002	1.09179786185862
0.9633044	20	0.987793807378	0.86801	1.12410654735569
0.6919245	20	0.991464218424	0.90530	1.085826443
0.1390248	20	0.993856041777	0.93096	1.06099069546141
0.08387846	20	0.996715705134	0.96229	1.03236503024921
0.6881879	18	1.068352775355	0.52293	2.18262482351261
0.2836535	18	0.987863943228	0.86366	1.12992873006922
0.1375389	20	0.989946279952	0.88045	1.11304933783095
0.6588266	20	0.980315421920	0.76676	1.25334364989623
0.1161004	18	0.987309314592	0.84225	1.15733960594655
0.7795261	20	0.963161091770	0.59647	1.55526539018784
0.4304828	16	1.019136296043	0.79163	1.31201769831464
0.9989953	9	0.964752294722	0.59404	1.56679457447941
0.1232737	20	1.010277352912	0.87333	1.1686861206515
0.4483605	20	0.985216958133	0.79419	1.22218944820791
0.9911445	20	0.982219669122	0.74696	1.29156169183871
0.9335418	14	1.003024504612	0.95705	1.05120100416116
0.6874019	20	1.004651233561	0.93285	1.08197495585876
0.9745618	20	1.002567720963	0.96196	1.04488446343688
0.06608426	4	0.993531898806	0.88701	1.11283651865897
0.117152	20	1.008488807313	0.86908	1.17025386511037
0.1067008	20	0.997795503479	0.95832	1.03888853985929
0.9766638	20	0.995540662503	0.91602	1.08195345939388
0.3430726	20	0.997685103515	0.95439	1.04294331238512
NA	NA	0.972619044679	0.55674	1.69914258149248
0.5119898	18	0.994863457384	0.89055	1.11138836829997
0.2315075	20	1.022746443058	0.62297	1.67905060902054
0.6816405	20	0.989152958065	0.77204	1.26730994440793
0.222602	20	0.996999061843	0.93062	1.0681110195233
0.7604034	20	1.005803404884	0.88013	1.14941669734991
0.5477082	20	0.996950040605	0.92811	1.07089252468929
0.6654825	20	1.008662090660	0.81950	1.24147296543246
0.737239	6	1.024520809141	0.52419	2.00240115911876
0.979956	20	0.995750624256	0.88171	1.12452987339462
0.7884057	20	0.994370942848	0.84547	1.169483834
0.375889	20	1.003366152810	0.90997	1.10634023679773
0.2219826	20	0.996700535236	0.88721	1.11969767070841
0.7294514	20	1.018517256912	0.51888	1.99925789659354

NA	NA	1.001113689692	0.95393	1.0506273668709
0.3422963	14	0.991102241444	0.65387	1.50224801067394
0.449411	20	1.002770982093	0.87522	1.14889989329341
0.5297779	20	0.998292239884	0.91708	1.08668984908853
0.4741963	19	1.001924709880	0.90127	1.11381983007856
0.9467546	20	1.007504810986	0.65991	1.53817958569756
0.9467546	20	1.007504810986	0.65991	1.53817958569756
0.3757089	20	0.996790072867	0.81911	1.2130070354666
0.3687202	20	0.999227942190	0.95192	1.04887919424124
0.02109253	20	1.000532628796	0.96008	1.0426856068631
0.09884996	20	1.000675706186	0.94919	1.05494647389882
0.04022868	20	1.000462474908	0.95285	1.05044618919753
0.539	19	1.001605737818	0.64936	1.54492315307091
0.3310038	20	0.999436928584	0.84341	1.18432705831283
0.003097541	20	1.000124788785	0.84821	1.17923453048507

Supplementary Table S14. Smoking-related circulating proteins significan

ProbeCh	Gene	Probe_bp	topSNP	topSNP_	topSNP_bp	A1	A2
4	ADH4	99140724	rs180075	4	99144358	G	T
12	FGF23	4373970	rs648953	12	4382743	C	G
14	PGF	74948730	rs657420	14	74986336	C	G
8	CTHRC	1.03E+08	rs827592	8	1.03E+08	G	C
14	NPC2	74485184	rs185905	14	73689254	A	G
11	YAP1	1.02E+08	rs107915	11	1.02E+08	A	G
19	FUT3	5847180	rs376077	19	5841345	T	G
2	INHBB	1.2E+08	rs170502	2	1.21E+08	A	G
11	RELT	73386936	rs112357	11	73387281	A	G
17	CCL3	36089212	rs163448	17	36082734	C	A
1	PCSK9	55052150	rs115911	1	55039974	T	G
19	IFI30	18175640	rs725944	19	18172201	C	A
20	BPIFB1	33291676	rs242496	20	33106642	C	T
5	CD74	1.5E+08	rs790782	5	1.5E+08	A	G
19	CEACA	41719509	rs9621	19	41727239	A	G
20	TNFRSF	63697668	rs606249	20	63704906	T	C
3	RBP2	1.39E+08	rs295469	3	1.39E+08	A	G
1	VWA1	1438872	rs115503	1	1434243	A	G
3	HEG1	1.25E+08	rs643886	3	1.25E+08	G	C
1	QSOX1	1.8E+08	rs12371	1	1.8E+08	G	A
8	SFRP1	41285718	rs726438	8	41309674	T	G
2	CAPG	85406592	rs6886	2	85394936	C	T

tly associated with aSAH risk (UKB-PPP)

Freq	b_GWAS	se_GWAS	p_GWAS	b_eQTL	se_eQTL	p_eQTL
0.604374	-0.059718	0.017474	0.00063176	0.130044	0.0074633	5.38397
0.336978	0.0537577	0.018033	0.00287217	0.108306	0.0078188	1.23653
0.542744	-0.050373	0.017334	0.00366033	-0.153021	0.0067351	2.84453
0.516899	-0.047266	0.017304	0.00630391	-0.162078	0.006824	1.06663
0.012923	0.506873	0.208099	0.0148618	-1.02753	0.0468091	8.37551
0.468191	0.0436575	0.01741	0.0121529	0.0623571	0.0069075	1.75710
0.067594	-0.065591	0.027943	0.0189108	-1.06768	0.015182	0
0.450298	0.0405666	0.017432	0.0199568	0.364573	0.0074933	0
0.198807	-0.043348	0.019095	0.0232017	0.3988	0.0084122	0
0.239563	0.0527768	0.023803	0.0266097	0.419583	0.0086964	0
0.020875	0.0994042	0.045392	0.0285299	-1.15328	0.0277856	0
0.241551	0.0444316	0.020493	0.0301439	-0.507285	0.0087716	0
0.586481	-0.037541	0.017421	0.0311652	-0.421503	0.0074581	0
0.101392	-0.079514	0.035752	0.0261463	-0.087035	0.0111797	6.96625
0.032803	-0.130585	0.061205	0.0328791	0.963008	0.0186498	0
0.706759	0.0416726	0.019706	0.0344572	-0.309343	0.0078386	0
0.348907	0.0415183	0.019728	0.0353297	-0.067918	0.0077236	1.44844
0.011928	-0.202003	0.098627	0.0405453	-1.49444	0.0327144	0
0.62326	0.036808	0.017901	0.0397658	0.108951	0.007257	6.00895
0.077535	0.0669742	0.033016	0.042507	-1.08621	0.013405	0
0.39662	-0.035195	0.017413	0.0432554	-0.188674	0.0074827	2.76689
0.65507	0.0354791	0.017804	0.0462818	-0.871962	0.0079073	0

b_SMR	se_SMR	p_SMR	p_HEIDI	nsnp_HI	or	or_lci95
-0.459215	0.136928	0.00079738	0.09327124	20	0.631779	0.483069
0.49635	0.170312	0.00356414	0.1632984	20	1.642714	1.176493
0.329189	0.114201	0.0039447	0.4505877	20	1.389840	1.111105
0.291628	0.107467	0.00665446	0.1979836	20	1.338604	1.084362
-0.493293	0.203766	0.01548329	0.01119048	19	0.610613	0.409558
0.700121	0.289762	0.01568374	0.135819	20	2.013990	1.141322
0.0614329	0.026187	0.01897711	0.1266842	20	1.063359	1.010158
0.111272	0.047869	0.02009854	0.1388895	20	1.117698	1.017601
-0.108696	0.047937	0.02335963	0.4662396	20	0.897003	0.816562
0.125784	0.056791	0.02676978	0.3532045	20	1.134037	1.014578
-0.086193	0.039414	0.0287514	0.1487585	13	0.917417	0.849214
-0.087587	0.040425	0.03026031	0.00630209	20	0.916139	0.846352
0.0890656	0.041361	0.03128835	0.5750012	20	1.093152	1.008030
0.913587	0.427213	0.03247761	0.2565408	17	2.493249	1.079231
-0.135601	0.063611	0.03302838	0.6689908	20	0.873190	0.770837
-0.134713	0.063795	0.03471608	0.00329319	20	0.873960	0.771243
-0.611297	0.298666	0.04068195	0.1147146	20	0.542640	0.302195
0.13517	0.066063	0.04074778	0.1863363	20	1.144731	1.005703
0.33784	0.165839	0.0416342	0.1949735	20	1.401910	1.012877
-0.061659	0.030405	0.04257147	0.5890853	20	0.940203	0.885809
0.186539	0.092585	0.04392768	0.3989025	20	1.205071	1.005084
-0.040689	0.020421	0.04631725	0.6150917	20	0.960127	0.922457

or_uci95
0.826267985853925
2.29368918702359
1.73850049987832
1.6524570805984
0.910363198379255
3.55393172926657
1.11936150732218
1.22764198001777
0.985367648737806
1.26756071916186
0.991097722028124
0.991680309783778
1.18546199997164
5.7599251509694
0.989135228646718
0.99037204442506
0.974421185660935
1.30297811220389
1.94038118327777
0.997937917020422
1.4448512628749
0.999336776030309

Supplementary Table S15. Associations between smoking-associated circulating pr

ProbeCh	Gene	Probe_bp	topSNP	topSNP_	topSNP_b	A1	A2	Freq
6	TREM1	4.1E+07	rs442408	6	4.1E+07	G	A	0.73062

oteins and the risk of aSAH （deCODE）

b_GWAS	se_GWAS	p_GWAS	b_eQTL	se_eQTL	p_eQTL	b_SMR
0.00089975	0.01956	0.9633	-0.3225	0.00891	4.64308	-0.00278993

se_SMR	p_SMR	p_HEIDI	nsnp_HI	or	or_lci95	or_uci95
0.06065	0.9633	0.77469	20	0.99721	0.88544	1.1230909467421

.5

Supplementary Table S16. Associations between smoking-associated circul

ProbeCh	Gene	Probe_bp	topSNP	topSNP_	topSNP_bp	A1	A2
21	TFF2	42348677	rs225344	21	42355775	A	G
19	CXCL17	42435612	rs116685	19	42450984	G	A
19	IFI30	18175640	rs725944	19	18172201	C	A
14	NPC2	74485184	rs185905	14	73689254	A	G
16	PLA2G1	68253181	rs7672	16	68260897	G	C
10	IL2RA	6036530	rs127224	10	6053965	A	C
7	GPR37	1.25E+08	rs414100	7	1.24E+08	C	A
17	ERBB2	39709170	rs376435	17	39760570	T	C
8	ANGPT2	6531520	rs196858	8	6421990	T	C
2	INHBB	1.2E+08	rs170502	2	1.21E+08	A	G
12	CD27	6448336	rs25680	12	6445462	A	G
1	TNFRSF	2560510	rs249536	1	2557491	A	G
4	ADH4	99140724	rs180075	4	99144358	G	T
11	RELT	73386936	rs112357	11	73387281	A	G
5	CD74	1.5E+08	rs790782	5	1.5E+08	A	G
11	YAP1	1.02E+08	rs107915	11	1.02E+08	A	G
3	HEG1	1.25E+08	rs643886	3	1.25E+08	G	C
22	RTN4R	20262330	rs663353	22	20187189	A	G
9	PTGR1	1.12E+08	rs703610	9	1.12E+08	G	A
11	MMP7	1.03E+08	rs115688	11	1.03E+08	A	G
12	STAB2	1.04E+08	rs115840	12	1.04E+08	G	A
4	AFM	73492873	rs131327	4	73480179	C	T
5	OCLN	69525198	rs258370	5	69461947	C	T
17	CCL3	36089212	rs163448	17	36082734	C	A
2	EFEMP1	55895053	rs379167	2	55869757	G	A
17	ENPP7	79736581	rs807653	17	79730552	T	C
1	AMY1A	1.04E+08	rs726944	1	1.04E+08	A	G
22	PLXNB2	50291312	rs115477	22	50283705	C	T
21	IL10RB	33288277	rs251571	21	33289977	A	G
11	ADM	10306235	rs792649	11	10336912	T	C
1	TNR	1.76E+08	rs223525	1	1.76E+08	G	A
20	OXT	3072068	rs877172	20	3069244	G	T
18	CDH2	28055412	rs124541	18	28110963	A	G
4	GC	71772868	rs135284	4	71752058	G	A
8	PLAT	42191214	rs202092	8	42187447	A	G
14	CLEC14	38255046	rs101512	14	38503166	A	C

14	RNASE(	20781868	rs10459:	14	20781965	A	G
1	TNFRSI	7929518	rs249321	1	7934360	G	A
19	FUT3	5847180	rs37607:	19	5841345	T	G
14	PGF	74948730	rs657420	14	74986336	C	G
19	SSC5D	55503752	rs810301	19	55487775	G	C
1	AGRN	1038119	rs312156	1	1032846	A	C
1	TNFRSI	1212746	rs381320	1	1215384	A	G
6	KIAA03	24595148	rs318123	6	24654215	T	C
1	APCS	1.6E+08	rs361262	1	1.6E+08	G	T
19	CEACA	41719509	rs9621	19	41727239	A	G
19	ICAM5	10293365	rs281439	19	10289434	C	G
19	PLAUR	43658321	rs230252	19	43652320	C	T
10	FAS	88991709	rs108878	10	89023165	A	T
3	LRRN1	3824632	rs983947	3	3766094	A	G
1	CA6	8960480	rs376596	1	8974539	G	A
17	ASGR1	7176498	rs557149	17	7176997	T	C
5	HAVCR	1.57E+08	rs115961	5	1.57E+08	C	G
21	COL18A	45459442	rs997683	21	45477300	T	C
2	VWC2L	2.14E+08	rs109325	2	2.15E+08	A	G
7	BMPER	34030368	rs201897	7	33743596	T	C
1	OPTC	2.04E+08	rs562195	1	2.04E+08	T	C
18	COLEC1	408730	rs230502	18	334742	T	C
3	LRIG1	66440030	rs230627	3	66384219	C	T
14	LGMN	92726243	rs148659	14	92709697	A	G
7	HGF	81734724	rs574568	7	81729735	T	C
12	FGF23	4373970	rs648953	12	4382743	C	G
1	CTBS	84562046	rs797231	1	84547012	A	G
1	S100A1:	1.53E+08	rs301487	1	1.53E+08	A	G
2	IMMT	86169702	rs175518	2	86195293	C	T
6	ULBP2	1.5E+08	rs624418	6	1.5E+08	T	A
17	CCL7	34271232	rs650539	17	34190977	A	T
17	CLEC10	7077422	rs90951	17	7078078	G	A
1	LEPR	65531106	rs103996	1	65608299	T	C
19	LGALS4	38807308	rs140695	19	38774589	T	A
17	FAM20A	68568240	rs295227	17	68533638	A	G
12	RBP5	7122312	rs110644	12	6859577	G	C
8	TNFRSI	23110183	rs655761	8	23103191	C	G
1	CA14	1.5E+08	rs112296	1	1.5E+08	G	C

7	NPTX2	98623577	rs390143	7	98658349	T	C
15	RGMA	93062241	rs375210	15	93072785	A	C
8	TNFRSF	23207777	rs132780	8	23225458	T	G
20	TNFRSF	63697668	rs606249	20	63704906	T	C
6	HLA-DF	32442462	rs264707	6	32606583	T	C
17	WFIKKI	50838502	rs459118	17	50832073	A	T
16	ADGRG	57638116	rs733464	16	57648363	T	C
4	SPON2	1187947	rs288559	4	1121121	G	A
1	FASLG	1.73E+08	rs794194	1	1.73E+08	A	T
21	TFF1	42364408	rs376137	21	42366929	A	G
14	ANG	20691574	rs800822	14	20688145	A	G
1	TNFRSF	12188110	rs574602	1	12193005	A	G
5	HAVCR	1.57E+08	rs655582	5	1.57E+08	A	C
9	FCN1	1.35E+08	rs108583	9	1.35E+08	C	T
19	ANGPT	8368830	rs227823	19	8366697	A	G
17	CCL16	35978995	rs112689	17	35980421	C	T
3	CRELD	9939603	rs987804	3	9943773	C	T
7	RARRE	1.5E+08	rs373516	7	1.5E+08	T	C
12	CPM	68906884	rs797819	12	68932767	T	C
6	THBS2	1.69E+08	rs781091	6	1.69E+08	A	C
12	CD4	6803828	rs730537	12	6787380	G	A
3	CDCP1	45114350	rs227686	3	45146293	G	C
1	EXTL1	26028174	rs273683	1	26031165	A	C
19	ICAM1	10278854	rs503037	19	10282558	G	A
2	CAPG	85406592	rs6886	2	85394936	C	T
13	TNFSF1	42585374	rs206230	13	42478744	A	G
22	LGALS	37677719	rs482029	22	37675036	A	G
8	CTHRC	1.03E+08	rs827592	8	1.03E+08	G	C
12	NELL2	44715062	rs382115	12	44936741	C	T
1	S100A1	1.52E+08	rs299955	1	1.52E+08	C	G
6	GSTA1	52797616	rs645887	6	52801179	T	C
6	GSTA3	52903168	rs645887	6	52801179	T	C
14	RNASE	20802042	rs172543	14	20812519	A	G
15	ACAN	88839394	rs429334	15	88710319	C	T
1	CD101	1.17E+08	rs121302	1	1.17E+08	C	A
1	IFNLR1	24171064	rs139958	1	24187233	C	G
20	BPIFA2	33171590	rs605913	20	33166430	A	T
7	TFPI2	93888074	rs624666	7	93949980	C	T

2	IL18R1	1.02E+08	rs101905	2	1.02E+08	G	A
3	CD80	1.2E+08	rs560789	3	1.2E+08	T	A
17	LGALS9	27639679	rs795262	17	27644953	G	A
11	CLMP	1.23E+08	rs730178	11	1.23E+08	G	C
1	CFHR2	1.97E+08	rs408574	1	1.97E+08	T	C
12	APOF	56361712	rs202085	12	56349583	C	T
3	FETUB	1.87E+08	rs503010	3	1.87E+08	C	T
1	VWA1	1438872	rs115503	1	1434243	A	G
8	RIDA	98109758	rs224219	8	98106394	C	A
18	SERPIN	63994904	rs382661	18	63987229	G	A
19	BSG	577386	rs561011	19	586745	C	T
9	ADAMT	1.34E+08	rs100825	9	1.34E+08	G	A
12	CKAP4	1.06E+08	rs270489	12	1.06E+08	A	G
20	BPIFB1	33291676	rs242496	20	33106642	C	T
22	OSM	30264840	rs482308	22	30204116	C	G
5	EDIL3	84162717	rs230107	5	84023794	A	G
20	CTSZ	58996962	rs148370	20	59027157	T	C
13	TNFSF1	1.08E+08	rs374039	13	1.08E+08	A	T
9	AMBP	1.14E+08	rs141738	9	1.14E+08	C	T
3	IL1RAP	1.91E+08	rs644444	3	1.91E+08	G	A
8	FABP4	81480828	rs204482	8	81483590	G	T
18	CLUL1	623661	rs129574	18	591690	C	G
11	NCAM1	1.13E+08	rs112144	11	1.13E+08	T	C
12	LRTM2	1828510	rs412766	12	1831348	A	G
7	GUSB	65971450	rs343565	7	66771620	T	G
12	CLSTN3	7144322	rs188343	12	6780329	A	G
10	PSAP	71833774	rs576810	10	71884204	T	C
1	QSOX1	1.8E+08	rs12371	1	1.8E+08	G	A
10	MRC1	17860256	rs727825	10	17936043	G	T
11	SCGB1A	62414149	rs374124	11	62419070	A	G
3	ITIH4	52821825	rs271033	3	52803839	C	T
12	PTPRR	70779406	rs148726	12	70833617	C	T
4	HPGDS	94320706	rs286535	4	94349727	C	T
16	UMOD	20344676	rs129177	16	20356368	T	G
5	LECT2	1.36E+08	rs31517	5	1.36E+08	C	T
20	LBP	38361748	rs223261	20	38369011	T	C
12	KITLG	88536822	rs111051	12	88970809	T	C
9	FBP1	94621691	rs499206	9	94597197	G	A

5	CRHBP	76967102	rs68962	5	76914771	A	G
19	LILRB2	54277498	rs38392	19	54279666	T	C
17	KRT19	41525962	rs74056	17	41535160	A	G
19	NOTCH	15180016	rs48082	19	15192918	A	G
11	LUZP2	24789804	rs76794	11	25028282	A	T
17	GFAP	44910048	rs59291	17	44914081	T	C
8	MSR1	16337684	rs41341	8	16155085	A	G
9	CD72	35628396	rs86948	9	35615899	G	C
9	OMD	92418426	rs10115	9	92433576	A	G
6	VEGFA	43778336	rs69214	6	43957870	A	G
17	CD300C	74543594	rs58440	17	74543335	T	C
10	ADAM8	1.33E+08	rs29953	10	1.33E+08	T	C
10	CDH23	71606434	rs21666	10	71713125	T	C
7	FAM3C	1.21E+08	rs27075	7	1.21E+08	A	T
13	ITGBL1	1.02E+08	rs10342	13	1.01E+08	T	C
1	FABP3	31371052	rs61780	1	31404204	T	C
3	LAMP3	1.83E+08	rs11921	3	1.83E+08	T	G
3	TGFBR2	30650372	rs76377	3	30155970	T	C
6	TNF	31576950	rs22290	6	31572779	C	T
6	TNF	31576950	rs22290	6	31572779	C	T
6	TNF	31576950	rs22290	6	31572779	C	T
6	TNF	31576950	rs22290	6	31572779	C	T
6	TNF	31576950	rs22290	6	31572779	C	T
6	TNF	31576950	rs22290	6	31572779	C	T
15	GOLM2	44352238	rs80235	15	43883905	G	A
2	IL1RN	1.13E+08	rs55709	2	1.13E+08	C	T
1	CHI3L1	2.03E+08	rs49509	1	2.03E+08	C	G
11	IL18BP	72002164	rs57436	11	71998832	A	G
13	NHLRC	39044208	rs41286	13	39039504	C	A
19	SIGLEC	51147912	rs14018	19	51142883	C	G
6	VNN1	1.33E+08	rs93890	6	1.33E+08	T	C
6	CFB	31948868	rs64115	6	31946403	A	G
3	SHISA5	48486312	rs11706	3	48502103	C	T
1	PCSK9	55052150	rs11591	1	55039974	T	G
1	CFHR5	1.97E+08	rs72732	1	1.96E+08	C	T
2	DNER	2.3E+08	rs34412	2	2.3E+08	T	A
22	NPTXR	38831240	rs11157	22	38845257	G	A
6	EDN1	12293778	rs5370	6	12296022	T	G

17	ALDH3A	19743464	rs88724	17	19742625	C	A
1	PIGR	2.07E+08	rs29110	1	2.07E+08	A	G
11	FOLR2	72219276	rs14689	11	72206309	T	C
16	TNFRSF	3020414	rs74758	16	3020627	T	C
10	SFTPD	79960040	rs72191	10	79946568	G	A
2	COL6A3	2.37E+08	rs10507	2	2.37E+08	A	C
16	LPCAT2	55547869	rs16955	16	55532397	T	C
11	RAB6A	73718388	rs11235	11	73927813	T	C
8	SLURP1	1.43E+08	rs25851	8	1.43E+08	G	A
6	MOG	29664687	rs94615	6	29657418	G	A
20	PLTP	45905388	rs60739	20	45923216	C	T
12	CNTN1	40882427	rs11177	12	40651583	A	C
2	HNMT	1.38E+08	rs11558	2	1.38E+08	T	C
1	EFNA1	1.55E+08	rs43901	1	1.55E+08	G	A
11	ADAMT	1.3E+08	rs73044	11	1.3E+08	A	G
17	CD79B	63930538	rs12946	17	63928647	T	C
22	SERPIN	20780916	rs23294	22	20700042	C	T
5	IL7R	35866149	rs68979	5	35874473	T	C
12	KRT18	52950888	rs49197	12	52879136	G	A
5	CD14	1.41E+08	rs57444	5	1.41E+08	A	G
6	TREM2	41160846	rs14333	6	41161469	T	C
17	CD300E	74616812	rs17194	17	74580252	C	T
22	IGLC2	22901206	rs28568	22	22907270	A	C
4	ART3	76061993	rs48596	4	76079288	G	A
7	VGF	1.01E+08	rs34985	7	1.01E+08	G	A
19	RTBDN	12830453	rs72550	19	12821455	A	G
11	CNTN5	99689917	rs89877	11	99218310	A	G
11	LTBP3	65548744	rs40994	11	65552515	T	C
22	SEZ6L	26276530	rs13720	22	26292865	T	G
1	BCAN	1.57E+08	rs23657	1	1.57E+08	G	A
1	PLA2G2	19977924	rs11573	1	19979653	C	G
4	F11	1.86E+08	rs22892	4	1.86E+08	T	C
11	CTSD	1758662	rs55861	11	1762527	G	A
15	SPINT1	40851112	rs17658	15	40853721	T	C
19	ACP5	11576834	rs23057	19	11576536	T	C
14	RNASE4	20692888	rs12588	14	20678425	T	C
11	FOLH1	49176865	rs61886	11	49158908	T	G
11	AHNAK	62494888	rs74853	11	62529129	C	T

6	DLL1	1.7E+08	rs93566	6	1.7E+08	G	A
1	SELE	1.7E+08	rs75157	1	1.7E+08	G	A
17	OMG	31284776	rs72813	17	31134507	A	G
17	IGFBP4	40450588	rs16683	17	40459730	C	T
17	CCL15	35998996	rs85462	17	36002776	A	C
19	BST2	17404284	rs81012	19	17377984	C	T
16	WFIKK	631678	rs55798	16	619708	G	T
16	PPL	4921624	rs74005	16	4954971	T	G
4	ODAM	70200150	rs76153	4	70195601	C	T
2	CD207	70833014	rs17718	2	70833805	C	G
8	TNFRSF	23044582	rs11059	8	23027596	G	A
17	SGSH	80213820	rs75030	17	80210594	T	C
12	LTA4H	96022136	rs25404	12	96037229	G	C
13	CPB2	46079110	rs65612	13	46066702	G	A
12	C1RL	7099412	rs10477	12	6839528	G	A
19	OSCAR	54098680	rs44429	19	54051696	T	C
5	SPINK6	1.48E+08	rs12717	5	1.48E+08	G	A
7	EPHA1	1.43E+08	rs75045	7	1.43E+08	G	T
10	MSMB	46040744	rs70778	10	46073546	G	C
19	HSPB6	35756322	rs58561	19	35758074	C	T
10	SFTPA2	79558130	rs17886	10	79558907	G	C
20	WFDC2	45475642	rs97344	20	45465735	G	C
1	AMY2A	1.04E+08	rs78245	1	1.04E+08	G	T
6	TNF	31576950	rs28577	6	31564776	T	C
6	TNF	31576950	rs28577	6	31564776	T	C
14	SERPIN	94618220	rs61976	14	94631329	G	A
4	SCARB	76196636	rs28563	4	76176220	C	A
3	PODXL	1.28E+08	rs11489	3	1.28E+08	G	A
8	SFRP1	41285718	rs72643	8	41309674	T	G
17	CCL18	36068152	rs20150	17	36064257	G	A
19	KLK8	50999359	rs74705	19	51001552	A	G
17	PLXDC	39108854	rs62076	17	39067156	T	C
12	ADGRD	1.31E+08	rs47595	12	1.31E+08	A	G
20	CST1	23749415	rs45491	20	23748236	T	C
17	CA4	60160420	rs11715	17	60156793	T	A
9	OGN	92393982	rs70263	9	92376282	T	G
2	REG3A	79158378	rs14761	2	79168385	A	T
12	ACVRL	51915134	rs22773	12	51912437	T	C

17	TNFRSF	16950967	rs34562	17	16939677	A	G
19	SULT2A	47878391	rs21210	19	47873738	C	T
1	IGSF8	1.6E+08	rs124082	1	1.6E+08	C	T
17	ASGR2	7108511	rs230497	17	7108760	T	C
19	FGF21	48756928	rs838131	19	48757420	C	A
2	QPCT	37358074	rs467069	2	37372820	C	A
3	RBP2	1.39E+08	rs295469	3	1.39E+08	A	G
3	HYAL1	50306136	rs116482	3	50302191	T	C
2	TGFA	70500738	rs176392	2	70549149	C	T
19	LILRB4	54657124	rs370156	19	54661088	A	G
10	GFRA1	1.16E+08	rs111976	10	1.16E+08	C	T
16	CCL22	57362486	rs727848	16	57350214	G	A
1	IGSF9	1.6E+08	rs374761	1	1.6E+08	T	C
9	IGFBPL	38415491	rs785724	9	38409084	A	G
11	PRCP	82896760	rs251029	11	82835874	T	A
11	SPON1	14115428	rs108321	11	14026933	T	C
14	SERPIN	94313818	rs274952	14	94360731	T	C
18	TNFRSF	62358288	rs620983	18	62353960	G	A
17	LGALS3	78975592	rs111526	17	78966679	T	C
8	GGH	63027144	rs743291	8	63018637	A	G
14	SERPIN	94383720	rs17580	14	94380925	A	T
12	PTPRB	70576655	rs246581	12	70596274	C	T
2	RNF149	1.01E+08	rs126216	2	1.01E+08	T	C
8	LY6D	1.43E+08	rs257292	8	1.43E+08	T	C
15	NTRK3	88058260	rs287354	15	87971624	C	G
12	CLEC7A	10123518	rs169105	12	10118488	C	A
6	CD83	14127087	rs125260	6	14105666	A	G
19	LILRA5	54310118	rs414135	19	54302597	T	A
10	SFTPA1	79613197	rs196570	10	79557289	T	G
11	MMP12	1.03E+08	rs173688	11	1.03E+08	G	A
5	CDHR2	1.77E+08	rs296368	5	1.77E+08	T	C
5	FLT4	1.81E+08	rs342212	5	1.81E+08	C	T
16	PRSS8	31133580	rs889555	16	31111250	T	C
9	LCN2	1.28E+08	rs611250	9	1.28E+08	C	A
20	CST7	24954598	rs227651	20	24949198	G	A
20	MMP9	46012734	rs17576	20	46011586	G	A
21	TFF3	42313538	rs117044	21	42315073	G	A
2	FABP1	88125522	rs224188	2	88124547	C	T

19	FSTL3	679892	rs91690	19	663929	G	A
19	SIGLEC	51524838	rs62617	19	51531295	A	C
3	FSTL1	1.2E+08	rs11477	3	1.2E+08	T	C
22	CRELD	49922854	rs74510	22	49921734	G	C
21	CSTB	43774420	rs35285	21	43781951	A	G
6	PLG	1.61E+08	rs11751	6	1.61E+08	T	C
4	ANXA1	1.68E+08	rs11936	4	1.68E+08	G	A
4	SPINK2	56815801	rs28656	4	56823779	G	A
11	IL18	1.12E+08	rs22504	11	1.12E+08	C	T
4	FABP2	1.19E+08	rs17009	4	1.19E+08	T	C
5	C9	39327732	rs69676	5	39356024	G	A
19	GDF15	18381954	rs10542	19	18389048	C	T
7	VWC2	49847794	rs76960	7	49775794	G	C
10	BLNK	96230379	rs55769	10	96246079	C	A
12	VWF	6036824	rs68120	12	6032283	A	G
17	SERPIN	1769797	rs62088	17	1762959	T	C
11	PGA4	61227020	rs11306	11	61452227	T	C
13	F7	1.13E+08	rs51033	13	1.13E+08	T	G
2	CCL20	2.28E+08	rs13953	2	2.28E+08	A	G
5	MZB1	1.39E+08	rs11649	5	1.39E+08	T	C
19	CEACA	42587649	rs12459	19	41759235	G	A
19	CEACA	41761594	rs12459	19	41759235	G	A
15	IGDCC	65402216	rs56362	15	65509382	C	T
11	B4GAT	66346502	rs10896	11	66349640	T	C
19	LAIR1	54360971	rs11194	19	54358584	A	T
16	CES2	66939770	rs74909	16	66889149	A	G
19	SIGLEC	51130778	rs20758	19	51125272	G	A
12	GPD1	50107648	rs14062	12	50923648	C	G
2	CGREF	27109008	rs23845	2	27101883	T	G
13	SLITRK	83879840	rs28768	13	83794650	A	T
15	FURIN	90876076	rs20714	15	90877710	G	C
18	DCC	52938050	rs98074	18	51955366	A	G
8	ST3GAI	1.34E+08	rs96433	8	1.33E+08	T	C
16	CA5A	87909063	rs55870	16	87893616	C	T
10	PRAP1	1.33E+08	rs14367	10	1.33E+08	A	G
1	AMY2B	1.04E+08	rs12080	1	1.04E+08	A	C
7	EPHB4	1.01E+08	rs31436	7	1.01E+08	G	A
4	CFI	1.1E+08	rs10033	4	1.1E+08	C	T

1	CSF1	1.1E+08	rs11577	1	1.1E+08	C		T
1	IGSF3	1.17E+08	rs22848	1	1.17E+08	C		T
20	CST3	23632590	rs91111	20	23632100	T		C
1	CFH	1.97E+08	rs73155	1	1.97E+08	A		C
11	TPP1	6616108	rs11827	11	6626391	C		T
19	LILRB1	54627590	rs36707	19	54296648	G		A
1	S100A1	1.54E+08	rs11467	1	1.54E+08	A		C
8	TNFRSF	1.19E+08	rs64698	8	1.19E+08	C		T
12	TNFRSF	6335436	rs41495	12	6333477	T		C
15	SCG3	51701259	rs26061	15	51681453	C		G
9	CCL21	34709570	rs10814	9	34723038	A		C
3	APOD	1.96E+08	rs13982	3	1.96E+08	C		T
11	TREH	1.19E+08	rs51414	11	1.19E+08	A		G
17	CD300L	74703644	rs35489	17	74704804	G		A
20	BPIFB2	33015704	rs23777	20	32994638	C		T
12	C1S	7029646	rs14611	12	6800807	T		C
15	GCHFR	40765888	rs22892	15	40773528	A		T
22	CHCHD	23766903	rs34284	22	23824602	A		G
2	ALPP	2.32E+08	rs12478	2	2.32E+08	T		C
16	MSLN	764800	rs37642	16	760143	G		A
8	CCN3	1.19E+08	rs22791	8	1.19E+08	A		G
19	VSTM1	54052389	rs24337	19	54042277	C		T
20	CHGB	5918432	rs23615	20	5923248	G		A
5	GM2A	1.51E+08	rs15347	5	1.51E+08	G		A
19	CEACA	41568181	rs72390	19	41577830	C		G
1	C8B	56951795	rs62645	1	56941811	T		C
2	CD302	1.6E+08	rs25561	2	1.6E+08	T		G
11	PAMR1	35481062	rs61736	11	35434513	C		T
13	GPC5	92136152	rs34270	13	91764804	C		T
7	DPP6	1.54E+08	rs37349	7	1.55E+08	C		T
7	PON3	95378124	rs14986	7	95396432	C		T
1	RBP7	10006614	rs12203	1	9935196	A		G
16	IL17C	88639520	rs17700	16	88618087	T		G
3	ACY1	51986268	rs15041	3	51982820	T		C
1	NPL	1.83E+08	rs12142	1	1.83E+08	T		A
19	TNFSF1	6665920	rs34456	19	6665009	C		T
5	SPOCK	1.37E+08	rs48357	5	1.38E+08	G		A
6	ENPP5	46165082	rs94727	6	46148712	A		G

9	ORM1	1.14E+08	rs11699	9	1.14E+08	A	G
19	NCAN	19232096	rs37610	19	19215154	T	G
19	EBI3	4233526	rs81091	19	4247479	T	C
4	ENPP6	1.84E+08	rs78293	4	1.84E+08	T	C
19	CFD	863668	rs71335	19	859368	T	G
12	ITGA5	54407264	rs17958	12	54456092	G	T
13	LCP1	46168896	rs73233	13	46133701	A	T
10	TCTN3	95676983	rs61871	10	95665573	C	A
11	SERPIN	57608279	rs28362	11	57598250	C	T
12	C1R	7086377	rs49635	12	6833659	G	A
19	A1BG	58349335	rs12659	19	58347310	G	A
1	REG4	1.2E+08	rs79795	1	1.2E+08	A	C
1	EFNA4	1.55E+08	rs72704	1	1.55E+08	A	G
22	GGT1	24611908	rs28509	22	24601879	C	T
10	ADAM1	1.26E+08	rs11244	10	1.26E+08	C	T

lating proteins and the risk of uIAs （UKB-PPP）

Freq	b_GWAS	se_GWAS	p_GWAS	b_eQTL	se_eQTL	p_eQTL
0.710736	-0.0784915	0.025969	0.0025068	0.124985	0.0080594	3.06332
0.089463	-0.128364	0.047766	0.0072015	0.117925	0.012377	1.60767
0.241551	0.0729479	0.029319	0.0128449	-0.507285	0.0087716	0
0.012923	0.722455	0.294132	0.0140404	-1.02753	0.0468091	8.37551
0.683897	0.0660283	0.027038	0.0146023	-0.245434	0.0082117	2.78729
0.144135	-0.123775	0.050938	0.0151022	0.956182	0.0133443	0
0.248509	0.0756717	0.031684	0.0169239	-0.784611	0.0085	0
0.159046	0.0753722	0.031883	0.0180788	0.200177	0.0100316	1.36394
0.352883	-0.0601161	0.025664	0.0191571	-0.156687	0.0078044	1.17839
0.450298	0.0579008	0.024951	0.0203109	0.364573	0.0074933	0
0.196819	0.075116	0.032346	0.0202172	0.293625	0.0086401	3.85218
0.482107	-0.0577024	0.024818	0.0200701	0.114794	0.0074094	3.86276
0.604374	-0.0578317	0.02508	0.0211145	0.130044	0.0074633	5.38397
0.198807	-0.0621756	0.027308	0.022795	0.3988	0.0084122	0
0.101392	-0.116523	0.051306	0.0231393	-0.087035	0.0111797	6.96625
0.468191	0.0538781	0.024874	0.0303082	0.0623571	0.0069075	1.75710
0.62326	0.054052	0.025642	0.035034	0.108951	0.007257	6.00895
0.780318	-0.0604716	0.029234	0.0385887	-0.297977	0.0085471	2.71503
0.033797	-0.145836	0.071467	0.0412905	0.757723	0.0168611	0
0.0666	-0.106254	0.052378	0.0424991	0.567004	0.0152635	4.77401
0.075547	0.0949437	0.048208	0.0489024	0.800343	0.0144	0
0.975149	-0.21812	0.109514	0.0464034	0.336036	0.0262031	1.19867
0.028827	-0.195807	0.099784	0.0497267	-0.473202	0.0240668	4.56042
0.239563	0.0664371	0.034058	0.051094	0.419583	0.0086964	0
0.228628	-0.0580574	0.029747	0.0509742	0.226222	0.0082224	1.23552
0.367793	0.0474649	0.025655	0.0642954	0.950159	0.0079762	0
0.235586	0.0602434	0.032574	0.0643917	0.403289	0.0096606	0
0.639165	-0.0462246	0.025015	0.064616	-0.713462	0.0076366	0
0.431412	0.0450661	0.0248	0.0691911	0.53177	0.0074902	0
0.494036	0.0450432	0.024852	0.0699117	0.159271	0.007052	6.03943
0.435388	-0.0444128	0.02487	0.0741327	-0.375222	0.0073532	0
0.349901	0.0481278	0.027054	0.0752437	0.626675	0.0085676	0
0.275348	0.0535446	0.029854	0.072887	-0.101206	0.0074517	5.14641
0.246521	-0.0543834	0.030784	0.0772912	1.20212	0.0083094	0
0.022863	0.238489	0.134067	0.075261	-0.280448	0.0225765	1.98199
0.422465	0.0440751	0.025056	0.0785652	0.084058	0.0073016	1.14339

0.248509	-0.049808	0.028868	0.0844656	-0.444932	0.0085209	0
0.183897	0.0479897	0.027812	0.0844384	-0.194434	0.0093022	5.14149
0.067594	-0.0686603	0.039975	0.0858717	-1.06768	0.015182	0
0.542744	-0.0426814	0.024838	0.0857275	-0.153021	0.0067351	2.84453
0.294235	0.0469165	0.027487	0.0878517	0.514434	0.0079946	0
0.342942	-0.0436341	0.025597	0.0882551	-0.377555	0.007766	0
0.031809	-0.142549	0.08418	0.0903816	-0.317719	0.0190906	3.41821
0.624254	-0.0427693	0.025608	0.0948943	0.30768	0.0078291	0
0.020875	-0.116156	0.069933	0.0967253	-0.535152	0.0235983	7.46422
0.032803	-0.146362	0.088407	0.0978115	0.963008	0.0186498	0
0.796223	-0.0488317	0.02954	0.098315	0.843945	0.0088885	0
0.141153	-0.0661918	0.039899	0.0971181	-0.176742	0.0096761	1.54917
0.581511	-0.0410954	0.025134	0.102036	-0.256654	0.0071051	9.96519
0.369781	0.0415169	0.025536	0.103986	0.81117	0.0080036	0
0.407555	-0.0450353	0.027709	0.104098	0.683173	0.0072583	0
0.183897	0.0461942	0.028398	0.103804	-0.19305	0.0093868	5.51947
0.05169	0.0684487	0.041932	0.1026	-0.224706	0.0171943	4.97391
0.188867	0.0495881	0.030641	0.105585	-0.197366	0.0093962	5.91824
0.272366	-0.0452262	0.027925	0.105329	0.0990683	0.0086441	2.07681
0.084493	-0.0676239	0.042093	0.108153	-0.323609	0.0142604	5.27233
0.010934	-0.160749	0.10045	0.109535	-1.29631	0.0421197	5.35552
0.493042	0.0399852	0.024973	0.109342	-0.120466	0.0071557	1.35393
0.292247	-0.0456011	0.028657	0.111549	-0.858309	0.0082076	0
0.014911	0.156317	0.102174	0.126041	-1.05518	0.0338526	2.73201
0.05666	-0.0975607	0.063825	0.126371	-0.185387	0.0147239	2.37082
0.336978	0.0395415	0.025913	0.127021	0.108306	0.0078188	1.23653
0.026839	-0.149147	0.098392	0.129557	1.37781	0.0213593	0
0.291252	0.0428176	0.028492	0.132889	-0.236592	0.0083629	4.50643
0.508946	-0.0371418	0.024948	0.136541	-0.115253	0.0073987	1.03704
0.173956	0.0474056	0.032087	0.139568	-0.666726	0.010737	0
0.445328	-0.0374226	0.025461	0.14161	0.10544	0.0073183	4.62062
0.360835	0.0369756	0.025502	0.147083	0.544455	0.0079022	0
0.151093	0.0548946	0.037883	0.147318	-0.276043	0.0098302	1.66339
0.030815	0.11953	0.082506	0.147411	0.279919	0.0194071	3.68045
0.677932	-0.0385494	0.026788	0.15014	-0.238835	0.0076425	2.15661
0.050696	0.103111	0.070773	0.145135	-0.158409	0.0187264	2.69400
0.76839	-0.0411474	0.028691	0.151525	0.296985	0.0073736	0
0.549702	-0.0356597	0.024902	0.152148	0.173857	0.0071845	2.28034

0.691849	0.0380576	0.026848	0.156337	0.343682	0.0070205	0
0.49006	0.0352702	0.025091	0.159811	-0.227453	0.0072636	2.98078
0.502982	-0.0348534	0.024895	0.161515	-0.462361	0.0073557	0
0.706759	0.039504	0.028242	0.161887	-0.309343	0.0078386	0
0.341948	-0.0364995	0.026121	0.162316	-0.567568	0.0080738	0
0.640159	-0.036002	0.025875	0.164108	-0.679912	0.0079297	0
0.430417	-0.0348169	0.024846	0.161114	0.0710144	0.0073514	4.45760
0.131213	-0.0537075	0.038933	0.16774	0.315157	0.0115812	4.58156
0.158052	-0.0513058	0.036985	0.165374	-0.099404	0.0093201	1.47603
0.239563	-0.0444847	0.032314	0.168623	-0.304245	0.0085011	1.64623
0.114314	0.0601154	0.044979	0.181376	0.666938	0.0113394	0
0.026839	0.111082	0.083278	0.182247	-0.391643	0.0187725	1.17275
0.479125	0.0329931	0.025051	0.18782	-0.475893	0.0068146	0
0.2833	0.034031	0.025913	0.18909	0.441881	0.0084327	0
0.540755	-0.0321854	0.024918	0.196468	0.279904	0.0075166	1.65319
0.100398	0.0648444	0.050689	0.200802	-1.33189	0.0123315	0
0.212724	-0.0430903	0.033749	0.201682	0.832059	0.0086734	0
0.257455	-0.0358122	0.028078	0.202142	0.215985	0.0084468	3.27352
0.015905	0.29655	0.232088	0.20134	-0.475495	0.0307585	6.56447
0.097416	-0.0452913	0.035773	0.205488	0.661309	0.0126719	0
0.315109	0.0347939	0.027502	0.205815	-0.359292	0.0080763	0
0.187873	-0.0399818	0.031609	0.205905	-0.302893	0.008695	7.05660
0.697813	0.0402341	0.031896	0.207155	-0.41233	0.0083239	0
0.428429	-0.0319421	0.025361	0.207856	0.415481	0.0076285	0
0.65507	0.0315208	0.025461	0.215705	-0.871962	0.0079073	0
0.536779	0.0313756	0.025474	0.218065	-0.111865	0.0073847	7.78399
0.33002	0.0325261	0.026761	0.224205	-0.28271	0.0079411	1.36513
0.516899	-0.030154	0.024795	0.223927	-0.162078	0.006824	1.06663
0.60338	-0.0315595	0.026003	0.224875	-0.173881	0.0074157	1.39640
0.032803	0.0667426	0.055871	0.232249	0.326276	0.0181519	3.06966
0.574553	0.0297043	0.024958	0.233985	0.314756	0.0074375	0
0.574553	0.0297043	0.024958	0.233985	0.312097	0.007647	0
0.682903	0.0336334	0.028314	0.234876	0.21707	0.007961	1.05323
0.167992	-0.0456321	0.03859	0.237007	0.133883	0.008865	1.56208
0.077535	-0.0484364	0.041199	0.239723	-1.27797	0.0144045	0
0.031809	-0.0786168	0.066939	0.240213	-0.942374	0.0214063	0
0.047714	-0.0723079	0.062244	0.24536	-0.350236	0.0193388	2.63205
0.063618	0.05176	0.044697	0.246857	0.241748	0.0154703	4.79730

0.780318	0.0365022	0.031849	0.251758	-0.897997	0.0088952	0
0.115308	0.0452483	0.039925	0.257069	0.60458	0.0117337	0
0.245527	0.0346688	0.030627	0.257648	-0.513717	0.0083485	0
0.589463	-0.0288493	0.025759	0.262725	0.33554	0.0070082	0
0.250497	-0.0303107	0.027182	0.264797	-1.03731	0.0090727	0
0.068589	-0.0602861	0.054439	0.268117	-0.351487	0.0143282	6.88646(
0.014911	-0.189045	0.171061	0.269103	-1.56757	0.034942	0
0.011928	-0.155676	0.143103	0.276657	-1.49444	0.0327144	0
0.294235	-0.0286843	0.026619	0.281209	-0.316247	0.0079532	0
0.542744	0.0275556	0.025584	0.281453	-0.726747	0.0075583	0
0.088469	0.0473042	0.04397	0.282007	0.241405	0.012834	6.27340
0.312127	-0.0284327	0.026475	0.282854	0.10608	0.0085307	1.6846e-
0.235586	-0.0299487	0.02814	0.287208	0.223303	0.0087042	3.74976
0.586481	-0.0264998	0.024917	0.28754	-0.421503	0.0074581	0
0.024851	0.0724388	0.068303	0.288896	-0.188281	0.0247745	2.96620
0.248509	-0.0335526	0.032306	0.299002	-0.097389	0.0087228	6.06042
0.011928	-0.142457	0.138181	0.302565	-1.69956	0.0376665	0
0.026839	0.150848	0.147422	0.306195	0.746059	0.0265034	2.42647
0.013917	0.0858733	0.084997	0.312345	0.41237	0.0320336	6.3827e-
0.83002	0.0352581	0.035186	0.316325	-1.4006	0.010261	0
0.16501	0.0363398	0.036194	0.315365	-0.118087	0.0095838	6.93428
0.272366	0.0258867	0.026057	0.320483	0.613679	0.0081119	0
0.203777	-0.0282161	0.028699	0.325526	-0.332747	0.0092402	5.66796
0.058648	0.0415376	0.042212	0.325105	-0.369887	0.0188574	1.15160
0.526839	-0.0246391	0.025333	0.330752	0.122784	0.0069842	3.48897
0.018887	0.0681139	0.06947	0.326849	-0.192328	0.0323959	2.90622(
0.220676	0.0290052	0.030337	0.339023	-0.248735	0.0087967	6.83664
0.077535	0.0439433	0.047273	0.352597	-1.08621	0.013405	0
0.10835	-0.0356816	0.038352	0.352172	0.170087	0.0108576	2.61341
0.342942	-0.0242326	0.026205	0.355113	-0.339217	0.0076028	0
0.393638	-0.0235375	0.02551	0.356174	0.247734	0.0076434	1.88551
0.010934	-0.175343	0.187826	0.350543	-0.292396	0.0517596	1.61275
0.641153	-0.0236779	0.025814	0.359019	0.463397	0.0079245	0
0.200795	-0.0272488	0.029738	0.359505	-0.969683	0.0093533	0
0.641153	0.0240612	0.026262	0.359555	-0.800073	0.0068494	0
0.072565	-0.0326983	0.035732	0.360142	-1.18791	0.0137197	0
0.683897	-0.0240667	0.02631	0.360332	-0.053819	0.0077484	3.76444
0.955268	0.0489387	0.054136	0.365998	0.575477	0.0141736	0

0.667992	-0.0234352	0.026044	0.368206	-0.588492	0.0077305	0
0.173956	-0.0248957	0.027737	0.369415	-1.16077	0.0092705	0
0.647117	0.0231476	0.025857	0.370679	0.147113	0.0076846	1.08842
0.147117	0.0391513	0.043807	0.371465	0.107855	0.0098401	5.90204
0.026839	-0.093457	0.105115	0.373951	-0.590872	0.0207643	4.09026
0.015905	-0.0876222	0.0985	0.373697	0.277537	0.0239523	4.79183
0.011928	0.111424	0.126414	0.378091	-1.5469	0.0334062	0
0.212724	-0.0270666	0.030689	0.377791	-0.166377	0.0090843	6.30095
0.311133	0.0245784	0.027811	0.376816	0.0692566	0.007746	3.85920
0.467197	0.0218219	0.024857	0.379998	-0.687849	0.0073873	0
0.082505	0.0310797	0.035593	0.38255	0.681898	0.0125267	0
0.894632	-0.0375225	0.043086	0.383823	-0.709409	0.0122509	0
0.250497	0.0311457	0.036065	0.38781	-0.167542	0.0093329	4.64514
0.750497	-0.0249191	0.028992	0.390052	-0.114252	0.0083064	4.77530
0.860835	0.0312899	0.036726	0.394217	-0.383042	0.0101105	0
0.043738	-0.0576217	0.067625	0.394171	-0.191542	0.0162131	3.30368
0.632207	-0.0223011	0.026461	0.399345	0.232694	0.0075188	2.67888
0.342942	0.0216531	0.025702	0.399531	0.0493714	0.0075625	6.64508
0.287276	-0.0220316	0.026392	0.40384	-0.102519	0.0086195	1.27292
0.287276	-0.0220316	0.026392	0.40384	-0.102519	0.0086195	1.27292
0.287276	-0.0220316	0.026392	0.40384	-0.099876	0.0085577	1.79512
0.287276	-0.0220316	0.026392	0.40384	-0.099876	0.0085577	1.79512
0.287276	-0.0220316	0.026392	0.40384	-0.091522	0.0085457	9.16638
0.287276	-0.0220316	0.026392	0.40384	-0.091522	0.0085457	9.16638
0.121272	0.033348	0.040033	0.404835	0.0652499	0.0117467	2.77984
0.437376	0.0218678	0.026649	0.41188	-0.213196	0.0075923	1.69267
0.819085	-0.0223065	0.027992	0.425517	0.671982	0.0090331	0
0.013917	0.0925156	0.116981	0.429027	-0.54695	0.0475355	1.22857
0.018887	0.0900118	0.116933	0.441435	-0.799475	0.0270849	1.72335
0.067594	-0.0396082	0.051615	0.442855	-0.634584	0.0151209	0
0.276342	-0.022344	0.029387	0.447057	-0.995641	0.008616	0
0.086481	-0.0387692	0.051207	0.448987	0.629591	0.0126838	0
0.018887	0.141326	0.186927	0.449618	-0.391984	0.0232889	1.43683
0.020875	0.0487539	0.064629	0.450631	-1.15328	0.0277856	0
0.020875	0.0406629	0.054219	0.453271	-1.58898	0.0295918	0
0.348907	0.0207846	0.027841	0.455333	-0.280264	0.0079105	6.06923
0.026839	-0.0423041	0.056971	0.457751	2.02215	0.0271952	0
0.212724	-0.0232608	0.031589	0.461516	0.271847	0.0090555	5.34361

0.644135	-0.019023	0.026168	0.467244	0.48354	0.0077249	0
0.025845	-0.128361	0.176907	0.468092	-0.508096	0.024565	4.85184
0.038767	0.0478588	0.06657	0.472185	0.321264	0.0212574	1.32892
0.039761	-0.0418914	0.058488	0.473839	0.158736	0.0181641	2.35233
0.420477	0.0180526	0.025348	0.476347	-0.758221	0.0073397	0
0.602386	0.0178428	0.025166	0.478325	0.091672	0.0076197	2.44229
0.05666	-0.0572441	0.080782	0.478557	-0.252858	0.0179781	6.25458
0.126243	-0.022534	0.032081	0.482421	0.0729988	0.010337	1.64248
0.544732	0.0172334	0.025043	0.491353	0.408687	0.0074908	0
0.048708	0.0608516	0.088883	0.493579	-0.450756	0.0166977	1.68735
0.220676	0.0217024	0.032435	0.503426	-0.501642	0.0091187	0
0.356859	-0.0168674	0.025211	0.503458	0.19899	0.0070555	5.29291
0.104374	0.0225488	0.033944	0.506502	-1.11656	0.0118907	0
0.521869	-0.0164314	0.024756	0.506858	0.317183	0.0075229	0
0.0666	-0.038569	0.058827	0.512058	0.192702	0.0173208	9.43195
0.39165	-0.0165753	0.025692	0.518832	-0.163364	0.0074279	3.33434
0.124254	-0.0293013	0.045427	0.51891	0.0921479	0.0112685	2.89868
0.271372	-0.0167825	0.026275	0.523006	-0.888648	0.0080815	0
0.693837	-0.0162509	0.025543	0.524627	0.0477177	0.0075178	2.19128
0.248509	0.0169774	0.027271	0.53359	-0.321281	0.0088539	2.61507
0.010934	-0.088162	0.142274	0.53548	-1.48289	0.033928	0
0.680915	-0.0167515	0.027059	0.535868	-0.361265	0.0078096	0
0.137177	-0.0197564	0.031909	0.535818	0.22902	0.0115953	7.85231
0.766402	0.0183183	0.029707	0.537478	-0.283109	0.0082249	1.21951
0.093439	-0.0263963	0.042732	0.536761	-0.119677	0.0141916	3.37132
0.270378	0.0159824	0.026106	0.540393	-0.171275	0.008435	1.15607
0.919483	-0.0251295	0.041255	0.542439	-0.79179	0.013161	0
0.04672	0.0337225	0.055384	0.542604	0.89636	0.0162699	0
0.017893	-0.0392786	0.064685	0.543701	-0.570654	0.0262121	4.40561
0.390656	0.0162084	0.026977	0.547959	-0.231316	0.0078362	1.65272
0.21173	0.016843	0.028229	0.550734	0.730757	0.0088002	0
0.388668	-0.014985	0.02517	0.551614	0.384964	0.007029	0
0.095427	0.0327227	0.055196	0.553281	-0.507226	0.0122086	0
0.0666	0.0462458	0.078086	0.553688	-0.675826	0.0163359	0
0.129225	-0.0206626	0.03496	0.554499	-0.504211	0.0109417	0
0.246521	0.0169937	0.028982	0.557639	-0.36502	0.0090194	0
0.052684	0.0386503	0.066009	0.558193	0.190771	0.017125	8.01858
0.025845	-0.0673586	0.11552	0.559832	-0.473173	0.0224177	6.84378

0.446322	0.0144736	0.024958	0.561971	0.15688	0.0073995	9.24685
0.690855	0.0150679	0.026369	0.567717	-0.094664	0.0065788	6.03671
0.104374	-0.0235726	0.041477	0.569815	-0.125182	0.0105363	1.48455
0.311133	-0.0145882	0.026312	0.579275	0.0445896	0.0078057	1.11347
0.934394	-0.0216797	0.04019	0.589587	-1.5711	0.0139486	0
0.961233	0.0218161	0.040461	0.589758	-0.343639	0.0182846	8.46662
0.267396	0.0148297	0.027552	0.590413	0.277795	0.0080756	2.59803
0.132207	-0.0227353	0.042307	0.590998	0.127738	0.0116112	3.76878
0.062624	-0.0299474	0.057372	0.601682	-0.587089	0.0173979	1.27341
0.302187	-0.0150074	0.028787	0.602134	0.473255	0.008067	0
0.897614	0.0260586	0.049993	0.602196	0.537694	0.0114898	0
0.303181	-0.0135003	0.026102	0.605004	-0.885866	0.0073793	0
0.331014	-0.0130397	0.025217	0.605091	0.102159	0.0077603	1.40831
0.741551	0.016263	0.031749	0.608485	0.674075	0.0084602	0
0.645129	0.0128805	0.025141	0.608414	-0.091444	0.0078118	1.19014
0.470179	0.0126761	0.024836	0.609771	0.52103	0.0076045	0
0.60338	-0.0127069	0.024976	0.610912	-0.359092	0.0072752	0
0.175944	-0.0177232	0.035031	0.612903	0.254349	0.0090857	1.90591
0.429423	0.0128365	0.025589	0.615925	-0.783606	0.0071366	0
0.126243	-0.0166797	0.033587	0.619462	-0.054241	0.0094194	8.48829
0.139165	-0.0175728	0.035815	0.62367	0.940133	0.0113707	0
0.086481	-0.0181155	0.037734	0.631168	-0.138665	0.0130306	1.91027
0.018887	0.03087	0.065476	0.637305	-0.963789	0.0211146	0
0.596421	-0.0121379	0.026034	0.641052	-0.084344	0.0077538	1.46994
0.596421	-0.0121379	0.026034	0.641052	-0.084344	0.0077538	1.46994
0.193837	-0.0166913	0.035968	0.642609	-0.447313	0.0098046	0
0.134195	-0.016851	0.036433	0.64371	-0.31455	0.0099719	2.19645
0.018887	-0.0254116	0.05552	0.647168	-1.14965	0.0339095	5.92010
0.39662	-0.0113656	0.024879	0.647785	-0.188674	0.0074827	2.76689
0.140159	-0.0154895	0.034022	0.648911	0.705587	0.01088	0
0.030815	0.0490841	0.109595	0.654247	1.05138	0.017783	0
0.045726	0.0356923	0.079907	0.655111	-0.534797	0.0178111	4.48633
0.94334	-0.0243376	0.054549	0.655482	0.82369	0.0153217	0
0.293241	-0.0139983	0.03143	0.656047	-0.582614	0.0085697	0
0.045726	-0.026579	0.060266	0.659194	-0.825377	0.0204107	0
0.355865	0.0118314	0.027066	0.662021	0.59708	0.0072724	0
0.069583	-0.032161	0.073717	0.662637	0.406934	0.0153062	9.79764
0.080517	-0.0173917	0.040611	0.668466	0.568278	0.0133099	0

0.106362	-0.017708	0.041432	0.669086	-0.173645	0.0123903	1.26997
0.849901	-0.0146753	0.034356	0.669263	0.377705	0.0100817	3.49902
0.190855	-0.0119627	0.028208	0.6715	0.156221	0.0091321	1.31946
0.272366	0.0121759	0.028726	0.671672	0.324944	0.0082387	0
0.512922	0.0106222	0.025074	0.671828	-0.122881	0.0076502	4.67192
0.036779	-0.0231461	0.054773	0.6726	-0.884485	0.0246965	6.64289
0.348907	0.0118994	0.028222	0.673288	-0.067918	0.0077236	1.44844
0.053678	0.0215048	0.051113	0.673952	-0.548913	0.0155165	3.98302
0.107356	-0.0169635	0.040575	0.675892	0.17467	0.0115963	2.85364
0.09841	-0.0200815	0.048074	0.676153	0.379011	0.0102928	7.93458
0.148111	-0.0132791	0.031853	0.67676	-0.303471	0.0101237	1.99944
0.109344	0.0150051	0.036745	0.683015	0.185285	0.0117466	4.73803
0.065606	0.022575	0.05543	0.683809	-0.478563	0.0166669	2.59737
0.821074	-0.0115545	0.028408	0.684203	0.494588	0.0090507	0
0.519881	-0.0101188	0.024884	0.684271	0.0685715	0.0071873	1.42004
0.50497	0.0102397	0.025322	0.68593	-0.29149	0.00715	0
0.508946	0.00999323	0.024795	0.686924	-0.213858	0.0068014	5.17183
0.173956	0.0152603	0.038399	0.691059	-0.596988	0.0093561	0
0.017893	0.0537675	0.135411	0.691317	-0.480122	0.0283494	2.44791
0.070577	0.0166257	0.042085	0.692803	0.722608	0.0150591	0
0.05666	0.0513373	0.131284	0.695769	-0.374366	0.0176218	3.70695
0.260437	0.011007	0.02935	0.707639	-0.366558	0.0079996	0
0.434394	-0.00931391	0.024843	0.707731	0.280815	0.0076153	1.15000
0.399602	-0.00937192	0.025735	0.715731	-0.44319	0.0076036	0
0.130219	-0.0141723	0.038939	0.715886	0.27255	0.0106007	8.91257
0.070577	-0.0191354	0.054086	0.723492	-1.56188	0.0133386	0
0.241551	0.0106899	0.030251	0.723804	0.199606	0.0088149	1.58851
0.303181	0.00885071	0.02514	0.724798	-0.359963	0.0078411	0
0.201789	0.0105726	0.030096	0.725367	-0.283821	0.009886	2.90110
0.128231	0.010927	0.03143	0.728097	-0.749318	0.010568	0
0.494036	-0.00855695	0.024863	0.730727	-0.101995	0.006414	6.13904
0.124254	0.0126465	0.037846	0.738261	-0.442515	0.010485	0
0.2833	0.00881293	0.026445	0.738945	0.114932	0.0078141	5.70696
0.053678	0.0149812	0.045471	0.7418	-0.140457	0.0175678	1.29418
0.210736	-0.0138667	0.042153	0.742186	-0.835844	0.0090132	0
0.380716	0.00813016	0.025173	0.746717	0.145118	0.0077108	5.17494
0.010934	0.0602042	0.187652	0.74834	-1.00049	0.0379244	2.25392
0.347913	-0.0077877	0.024872	0.754197	-0.236988	0.0079568	6.24044

0.619284	-0.00770824	0.025375	0.761297	0.0866776	0.0075655	2.16819
0.014911	-0.0402944	0.134677	0.764793	-1.75832	0.0335146	0
0.353877	-0.00709339	0.025112	0.77758	-0.149402	0.0078955	7.46304
0.028827	-0.0227271	0.081208	0.779582	-0.812982	0.0201768	0
0.314115	0.00698655	0.025408	0.783338	-0.784681	0.008144	0
0.072565	0.0123234	0.045158	0.784932	-0.411884	0.01254	1.31561
0.597416	-0.00672108	0.024801	0.78639	0.0503908	0.0076996	5.96624
0.012923	-0.0285379	0.107889	0.791385	1.45971	0.0240298	0
0.511928	0.00654558	0.025049	0.793849	-0.29499	0.0073618	0
0.323062	0.00671717	0.025706	0.793858	-0.212769	0.0079902	3.14945
0.945328	0.0150382	0.05785	0.794899	-0.205148	0.0158249	1.96654
0.140159	-0.0122516	0.047233	0.795336	0.391818	0.0089337	0
0.487078	0.00642462	0.024854	0.796025	-0.457728	0.0073947	0
0.029821	-0.0146499	0.056651	0.795944	-0.149364	0.0201358	1.19042
0.347913	0.00682285	0.026565	0.797302	0.0975401	0.0077704	3.83704
0.34493	-0.00630254	0.025082	0.8016	-0.282879	0.0076937	6.09232
0.024851	-0.0241078	0.096556	0.802838	-0.200815	0.0285265	1.92796
0.125249	0.0113276	0.045667	0.804097	-0.778851	0.0116972	0
0.60835	-0.00698684	0.0283	0.804999	0.0934663	0.0076291	1.65425
0.06163	0.013045	0.052934	0.805341	0.210669	0.0164645	1.74020
0.284294	0.00663889	0.027109	0.806539	-0.130572	0.0080499	3.62736
0.284294	0.00663889	0.027109	0.806539	-0.120252	0.0080971	6.82804
0.016899	0.039289	0.163025	0.809555	0.964492	0.0255817	0
0.245527	-0.00695147	0.029233	0.812038	-0.315543	0.0087129	3.43697
0.159046	-0.00811712	0.035861	0.820929	0.723161	0.0096445	0
0.016899	-0.0149587	0.067225	0.823912	-0.334298	0.0329197	3.14917
0.583499	0.00557071	0.025338	0.825982	-0.573145	0.0066068	0
0.012923	0.0378812	0.172867	0.826545	-0.414673	0.0398184	2.13795
0.619284	0.00558741	0.026072	0.83031	-0.56091	0.0076346	0
0.17495	-0.00812263	0.038191	0.831573	0.157284	0.0097876	4.15422
0.323062	-0.00588871	0.028078	0.833879	0.194343	0.0078539	3.51555
0.33002	0.00588211	0.028883	0.838623	0.246506	0.0082512	4.15002
0.577535	0.00490135	0.025382	0.846878	-0.237049	0.0074041	6.57684
0.178926	-0.00638256	0.035486	0.85726	0.812228	0.0094418	0
0.018887	0.0252611	0.14493	0.86163	-1.29767	0.0281617	0
0.105368	0.00785223	0.045075	0.861704	0.658622	0.0137058	0
0.531809	0.00433279	0.024885	0.861775	0.154367	0.0072714	5.11683
0.536779	-0.00429821	0.024835	0.862596	0.216076	0.0073767	1.32495

0.526839	0.00416138	0.024898	0.867263	-0.186628	0.0074675	7.46449
0.197813	0.00552195	0.033047	0.867296	0.109766	0.0090437	6.69883
0.767396	0.00488671	0.029653	0.869106	0.328497	0.0084837	0
0.188867	0.00596718	0.037058	0.872076	0.421186	0.009687	0
0.374751	0.00405024	0.02538	0.873206	0.149894	0.0076919	1.40636
0.17992	-0.00420435	0.027076	0.876601	-0.670803	0.0086347	0
0.027833	0.0142966	0.094798	0.880124	-0.503584	0.0187947	3.80209
0.539761	0.00371808	0.024812	0.880883	0.186665	0.0069102	1.03528
0.018887	0.0184708	0.124598	0.882151	-0.302924	0.028173	5.78095
0.781312	-0.00526072	0.035786	0.883129	0.707695	0.0086385	0
0.297217	0.00398991	0.027617	0.885127	-0.103174	0.0079444	1.45011
0.023857	-0.00878993	0.063126	0.889257	-0.536629	0.0220963	2.76044
0.314115	-0.00360914	0.026571	0.891954	0.882864	0.0080801	0
0.819085	0.00392331	0.02939	0.893806	1.29528	0.009567	0
0.373757	-0.0034808	0.026566	0.895755	0.265266	0.0075197	1.37055
0.017893	-0.00735751	0.057247	0.897736	-0.258273	0.0277776	1.43284
0.468191	-0.00307243	0.024907	0.901825	0.111278	0.007403	4.56671
0.107356	-0.00379842	0.031122	0.902859	-0.118256	0.0112612	8.53100
0.223658	-0.00333557	0.027498	0.903453	0.505569	0.008737	0
0.254473	-0.00333332	0.02772	0.904284	0.63759	0.0085134	0
0.22664	0.00370848	0.031066	0.904978	-0.273837	0.008053	1.91330
0.434394	0.0029476	0.024761	0.905243	-0.768327	0.0078051	0
0.349901	-0.00298294	0.025123	0.905488	-0.610953	0.0076408	0
0.67495	-0.0029261	0.026171	0.910976	-0.694979	0.0077263	0
0.779324	0.00314112	0.028234	0.911414	-1.26315	0.0092019	0
0.290258	-0.00273596	0.025249	0.913709	0.5192	0.0078661	0
0.889662	-0.00435709	0.043554	0.920313	-0.419387	0.0107109	0
0.131213	0.00372493	0.038427	0.922777	-0.476234	0.0099184	0
0.285288	0.00249129	0.026714	0.925698	-0.878117	0.0085984	0
0.264414	0.00258461	0.028097	0.926707	0.260543	0.0081391	7.62624
0.026839	-0.00891913	0.103285	0.931185	-1.05099	0.0231471	0
0.753479	-0.00211472	0.02785	0.939473	0.306044	0.0088507	5.30178
0.349901	-0.00181618	0.024849	0.941736	-0.120102	0.0078494	7.55261
0.024851	-0.0107553	0.154769	0.944597	-0.462066	0.0264537	2.55861
0.043738	0.00331825	0.047904	0.944776	0.388365	0.0134428	1.58592
0.94831	0.00360406	0.054161	0.946945	0.553818	0.0167576	1.62045
0.379722	0.00165861	0.025846	0.948832	-0.131286	0.0076465	4.49270
0.670974	-0.00174521	0.027711	0.949783	-0.778049	0.0074082	0

0.077535	-0.00498986	0.083448	0.952318	0.695848	0.0149968	0
0.101392	0.00277132	0.046445	0.952419	-0.240654	0.0112025	2.285609
0.315109	0.00129107	0.027114	0.962022	-0.412969	0.0078492	0
0.021869	-0.00342686	0.077751	0.964845	0.952949	0.0236542	0
0.223658	0.00124286	0.034259	0.97106	-0.185468	0.0091385	1.414461
0.081511	-0.00135342	0.039514	0.972677	0.092313	0.0137278	1.761571
0.895626	0.00157259	0.048747	0.974264	-1.47256	0.0129523	0
0.28827	0.000746502	0.026951	0.977903	0.630848	0.0077161	0
0.050696	0.0012892	0.055707	0.981536	-0.424659	0.0168261	1.531075
0.595427	0.00056697	0.024858	0.981803	-0.057935	0.0077013	5.366607
0.941352	-0.00196455	0.087017	0.981988	0.297273	0.0166904	5.804832
0.018887	-0.00164783	0.08252	0.984068	0.633474	0.0277577	2.799012
0.010934	-0.00107629	0.068023	0.987376	0.237205	0.0346922	8.063048
0.369781	-0.000313933	0.026394	0.99051	0.254153	0.0073292	1.776098
0.599404	3.20365e-05	0.025534	0.998999	0.129391	0.0078031	9.418861

b_SMR	se_SMR	p_SMR	p_HEIDI	nsnp_HI	or
-0.628007	0.211686	0.00301017	0.00051292	20	0.5336543149
-1.08852	0.420854	0.00969669	0.2470769	20	0.3367144625
-0.143801	0.05785	0.01292814	0.6802994	20	0.8660600768
-0.703099	0.288038	0.0146469	0.2063568	19	0.4950487680
-0.269027	0.11053	0.0149338	0.233109	20	0.7641226240
-0.129447	0.053303	0.01516004	0.05070193	20	0.8785811519
-0.0964449	0.040395	0.0169608	0.06088152	20	0.9080599303
0.376528	0.16039	0.01889588	0.7074262	20	1.4572163409
0.38367	0.164901	0.01998265	0.2156055	20	1.4676610336
0.158818	0.068518	0.02045381	0.09094131	20	1.1721246005
0.255823	0.110416	0.02050967	0.1527912	20	1.2915241079
-0.50266	0.218615	0.02148825	0.9468437	20	0.6049194320
-0.444709	0.194536	0.02225456	0.4501713	20	0.6410107830
-0.155907	0.068554	0.02295261	0.6279111	20	0.8556387610
1.33881	0.614064	0.02923938	0.08155582	17	3.8145015465
0.864025	0.410218	0.0351818	0.10757	20	2.3726915835
0.496113	0.23766	0.03684401	0.8548069	20	1.6423251300
0.20294	0.09828	0.03893065	0.4795861	20	1.2249989661
-0.192466	0.094416	0.04149996	0.00300552	20	0.8249223650
-0.187395	0.092515	0.04280845	0.08409285	20	0.8291161708
0.118629	0.060272	0.04904366	0.07281723	20	1.1259521125
-0.649097	0.329807	0.04905488	0.1536098	12	0.5225173970
0.413792	0.211917	0.0508658	0.8525928	19	1.5125424853
0.158341	0.081238	0.05128338	0.9213096	20	1.1715656304
-0.256639	0.131826	0.05155784	0.1124805	20	0.7736474500
0.0499547	0.027004	0.06432801	0.7019386	20	1.0512234748
0.14938	0.080849	0.06465491	0.1987604	20	1.1611141287
0.0647892	0.035068	0.06466735	0.02263104	20	1.0669340909
0.0847474	0.046652	0.06928158	0.1146507	20	1.0884420914
0.282809	0.156536	0.07081353	0.6935464	20	1.3268517089
0.118364	0.066322	0.07431025	0.2003374	20	1.1256537747
0.0767987	0.043183	0.07532939	0.1880499	20	1.0798246858
-0.529065	0.297545	0.07538749	0.4672546	20	0.5891555726
-0.0452396	0.02561	0.07731233	0.8548062	20	0.9557684522
-0.850386	0.482923	0.07825273	0.01293602	20	0.4272499816
0.524342	0.301537	0.08205318	0.0397929	20	1.6893468923

0.111945	0.064918	0.08463463	0.8947643	20	1.1184513441
-0.246817	0.143528	0.08549652	0.5532547	20	0.7812836553
0.0643079	0.037452	0.08596501	0.4581298	20	1.0664206991
0.278925	0.162783	0.08662363	0.9688421	20	1.3217082120
0.0912002	0.053451	0.08796406	0.00398375	20	1.0954883
0.11557	0.067838	0.08845027	0.7217644	20	1.1225130877
0.448664	0.266318	0.09204745	0.1069082	20	1.5662183194
-0.139006	0.083306	0.09519238	0.4758295	20	0.8702228071
0.217052	0.13103	0.09761766	0.4787487	14	1.2424087057
-0.151984	0.09185	0.097984	0.1282447	20	0.8590020246
-0.0578612	0.035007	0.09836524	0.6055924	20	0.9437809351
0.374511	0.226675	0.09849549	0.4523099	19	1.4542800977
0.16012	0.098029	0.1023878	0.3216914	20	1.1736517007
0.0511815	0.031484	0.1040301	0.4866419	20	1.0525139072
-0.0659208	0.040565	0.1041501	0.3583903	20	0.9362050087
-0.239286	0.14756	0.1048852	0.2102471	20	0.7871897139
-0.304614	0.188058	0.1052773	0.5338988	11	0.7374079589
-0.251249	0.15571	0.1066195	0.07516337	20	0.7778286681
-0.456515	0.284678	0.1087976	0.5404978	20	0.6334875070
0.208968	0.130398	0.1090363	0.6272533	20	1.2324055609
0.124005	0.077594	0.1100141	0.3415359	19	1.1320215310
-0.331921	0.208236	0.1109442	0.6523447	20	0.7175440065
0.053129	0.033392	0.1115909	0.5154216	20	1.0545656753
-0.148143	0.096947	0.1264951	0.5283495	9	0.8623077961
0.526254	0.346807	0.129159	0.3077974	20	1.6925800134
0.365091	0.240701	0.129322	0.6073262	20	1.4406451008
-0.108249	0.071431	0.1296625	0.6738726	20	0.8974041149
-0.180977	0.120596	0.1334361	0.3328513	13	0.8344545509
0.322263	0.217445	0.1383283	0.09742585	20	1.3802477333
-0.0711021	0.04814	0.1396795	0.3370978	20	0.9313667947
-0.354918	0.242724	0.1436784	0.3529205	20	0.7012309417
0.0679131	0.04685	0.1471732	0.02424264	20	1.0702722977
-0.198862	0.137417	0.1478571	0.5359851	20	0.8196629990
0.427016	0.296233	0.1494473	0.2883903	20	1.5326771843
0.161406	0.112281	0.1505716	0.110208	20	1.1751619877
-0.650916	0.453352	0.1510633	NA	NA	0.5215678017
-0.13855	0.096668	0.1517839	0.4546675	20	0.8706197191
-0.205109	0.143485	0.1528653	0.5099851	20	0.8145585246

0.110735	0.078153	0.1565104	0.04279621	20	1.11709883б4
-0.155066	0.110423	0.1602314	0.9765129	20	0.8563586559
0.0753814	0.053857	0.1616192	0.215826	20	1.0782953343
-0.127703	0.091355	0.1621506	0.00963642	20	0.8801147344
0.0643086	0.046032	0.1623991	0.1188198	20	1.0664214456
0.052951	0.038061	0.1641636	0.6352766	20	1.0543779793
-0.490279	0.353528	0.1654966	0.7310892	20	0.6124554952
-0.170415	0.123692	0.1682855	0.3941411	20	0.8433147683
0.516137	0.375198	0.168934	0.4944124	20	1.6755425109
0.146213	0.106289	0.1689389	0.00544133	20	1.1574426970
0.0901364	0.067458	0.1814887	0.1699186	20	1.0943235392
-0.283631	0.213071	0.1831381	0.7530735	20	0.7530444668
-0.0693288	0.052649	0.1878994	0.9125488	20	0.9330198527
0.0770139	0.058661	0.1892295	0.02863537	20	1.0800570890
-0.114987	0.089075	0.1967376	0.3613235	20	0.8913777317
-0.048686	0.03806	0.2008336	0.08973577	20	0.9524801615
-0.0517876	0.040565	0.2017226	0.223636	20	0.9495305257
-0.165809	0.13016	0.202703	0.4523258	20	0.8472080354
-0.623666	0.489762	0.2028741	0.5239362	7	0.5359759437
-0.0684873	0.05411	0.2056221	0.843526	20	0.9338053193
-0.0968402	0.076575	0.2059976	0.03901337	20	0.9077010451
0.132	0.104424	0.2062036	0.8842317	20	1.1411083192
-0.0975774	0.07738	0.2073023	0.8940493	20	0.9070321345
-0.0768798	0.061057	0.2079778	0.6253886	20	0.9260011522
-0.0361493	0.029201	0.2157348	0.7772795	20	0.9644962834
-0.280477	0.228469	0.2195823	0.09454619	20	0.7554233185
-0.115051	0.094715	0.2244758	0.1458121	20	0.8913206853
0.186046	0.15318	0.2245337	0.01799595	20	1.2044776650
0.181501	0.149747	0.2254949	0.3787692	20	1.1990157357
0.204559	0.171616	0.2332782	0.4510284	20	1.2269838457
0.0943725	0.079326	0.2341714	0.7733231	20	1.0989690356
0.0951765	0.080004	0.2341853	0.7781325	20	1.0998529620
0.154943	0.130559	0.2353195	0.4418016	14	1.1675914064
-0.340836	0.289115	0.2384411	0.9138019	20	0.7111755314
0.037901	0.03224	0.2397637	0.4482348	20	1.0386284035
0.0834242	0.071058	0.2403804	0.444259	9	1.0870028173
0.206455	0.178084	0.2463293	0.8070552	20	1.2293124139
0.214107	0.185398	0.2481507	0.3326998	5	1.2387551945

-0.0406485	0.035469	0.251788	0.1032619	20	0.960166569
0.0748425	0.066053	0.2571849	0.9108099	20	1.077714397
-0.0674862	0.059629	0.2577282	0.3461467	20	0.934740619
-0.0859787	0.07679	0.2628554	0.3634645	20	0.917613776
0.0292205	0.026205	0.2648207	0.3218946	20	1.029651607
0.171517	0.155039	0.2686046	0.07986422	20	1.187104323
0.120597	0.109158	0.2692476	0.2135676	10	1.128170168
0.10417	0.095784	0.2767932	0.3106904	20	1.109789103
0.0907022	0.084201	0.2813855	0.7996732	20	1.094942882
-0.0379164	0.035206	0.2814838	NA	NA	0.962793427
0.195954	0.182441	0.2827919	0.06911256	20	1.216470946
-0.268031	0.250509	0.2846435	0.4349858	20	0.764884069
-0.134117	0.126126	0.2876219	0.09288606	20	0.874487743
0.0628698	0.059125	0.2876264	0.638487	20	1.064888181
-0.384738	0.366288	0.2935483	0.1557471	20	0.680628937
0.344523	0.333158	0.3010833	0.3217994	10	1.411316561
0.0838199	0.081325	0.3026928	0.5203791	20	1.087433029
0.202193	0.197731	0.306514	0.2530731	18	1.224084233
0.208243	0.206751	0.3138309	NA	NA	1.231512390
-0.0251736	0.025123	0.3163384	0.01621444	20	0.975140612
-0.307738	0.307519	0.3169663	0.7690897	20	0.735107891
0.0421828	0.042464	0.3205236	0.4761632	20	1.043085137
0.0847975	0.086281	0.3257056	0.8166802	20	1.088496623
-0.112298	0.114265	0.325712	0.1359944	19	0.893777872
-0.20067	0.206639	0.3314904	0.254163	20	0.818182387
-0.354155	0.366099	0.3333564	0.9483	9	0.701766185
-0.116611	0.122035	0.3392978	0.1507635	20	0.889931309
-0.0404556	0.043524	0.3526283	0.6314875	20	0.960351803
-0.209784	0.225879	0.3530203	0.5787954	20	0.810759351
0.0714369	0.077269	0.355216	0.5264675	20	1.074050375
-0.0950112	0.103015	0.3563683	0.4403286	20	0.909362749
0.599676	0.651081	0.3570254	NA	NA	1.821528529
-0.0510964	0.055714	0.359078	0.422623	20	0.950187068
0.0281007	0.030669	0.3595242	0.2193253	20	1.028499249
-0.0300738	0.032825	0.3595685	0.1102077	20	0.970373917
0.0275259	0.030082	0.3601694	0.4539987	20	1.027908237
0.447181	0.493087	0.3644589	0.9069719	6	1.563897339
0.0850402	0.094095	0.3661175	0.2329394	20	1.088760834

0.0398225	0.044258	0.3682399	0.4819183	20	1.0406260466
0.0214476	0.023896	0.3694258	0.6338288	20	1.0216792529
0.157346	0.175958	0.3712015	0.5587497	20	1.1704005
0.362999	0.40751	0.3730503	0.5680065	20	1.4376344216
0.158168	0.177985	0.3741862	0.2255005	15	1.1713629671
-0.315714	0.355951	0.3751009	0.6122218	9	0.7292679909
-0.0720305	0.081736	0.3781762	0.5808853	4	0.9305025150
0.162682	0.184667	0.3783438	0.4482598	20	1.1766624515
0.354889	0.403514	0.3791329	0.3146337	20	1.4260223570
-0.0317248	0.036139	0.3800186	0.6672493	20	0.9687731517
0.0455782	0.052203	0.3826103	0.341827	20	1.0466328481
0.0528926	0.060742	0.3838764	0.6459511	20	1.0543164054
-0.185898	0.215509	0.3883579	0.4664067	20	0.8303582872
0.218106	0.254247	0.3909742	0.2384045	18	1.2437188948
-0.0816879	0.095903	0.394337	0.5105782	20	0.9215595325
0.300831	0.353973	0.3953978	0.9438622	19	1.3509810064
-0.0958387	0.113758	0.3995199	0.4628613	20	0.9086105631
0.438576	0.524908	0.4034196	0.5481207	18	1.5504977369
0.214903	0.258068	0.4049926	0.6248129	20	1.2397416361
0.214903	0.258068	0.4049926	0.6248129	20	1.2397416361
0.22059	0.264923	0.405037	0.8748352	20	1.2468121327
0.22059	0.264923	0.405037	0.8748352	20	1.2468121327
0.240725	0.289241	0.4052611	0.7777938	20	1.2721711401
0.240725	0.289241	0.4052611	0.7777938	20	1.2721711401
0.511081	0.620391	0.4100504	0.2465185	20	1.6670923480
-0.102571	0.125051	0.4120798	0.9156694	20	0.9025140689
-0.0331951	0.041658	0.4255441	0.05521153	20	0.9673498112
-0.169148	0.214383	0.4301129	0.8118519	11	0.8443839253
-0.112589	0.146312	0.4415901	0.3705682	20	0.8935178204
0.062416	0.08135	0.4429323	0.9760632	14	1.0644050451
0.0224418	0.029517	0.4470681	0.1658702	20	1.0226955115
-0.0615784	0.081344	0.44904	0.04069608	20	0.9402792249
-0.36054	0.477355	0.4500765	0.2394955	13	0.6972996825
-0.0422741	0.056049	0.4507058	0.5519794	13	0.9586069903
-0.0255906	0.034125	0.4533154	0.9458985	14	0.9747340640
-0.0741608	0.09936	0.4554326	0.7227371	20	0.9285223754
-0.0209204	0.028175	0.4577736	0.4428035	20	0.9792969135
-0.0855658	0.116237	0.4616507	0.5873669	20	0.9179927371

-0.0393411	0.05412	0.4672741	0.8812603	20	0.961422711
0.252631	0.348391	0.468367	0.6356996	15	1.287408135
0.14897	0.207446	0.4726853	0.656275	20	1.160638169
-0.263906	0.369693	0.4753189	0.1696077	20	0.768045732
-0.0238092	0.033432	0.4763574	0.7696435	20	0.976472002
0.194637	0.275001	0.4790877	0.8156477	19	1.214869908
0.226388	0.319881	0.4791144	0.7059658	19	1.254062147
-0.30869	0.441637	0.4845726	0.09294379	20	0.734408401
0.0421677	0.061281	0.4913878	0.7138514	20	1.043069386
-0.134999	0.197249	0.493718	0.7807743	20	0.873716785
-0.0432627	0.064662	0.5034592	0.8357106	20	0.957659779
-0.0847651	0.126728	0.5035774	0.4981277	20	0.918728068
-0.0201949	0.030401	0.5065115	0.3939143	20	0.980007651
-0.0518042	0.078059	0.5069098	0.5690807	20	0.949514763
-0.200148	0.305804	0.5127898	0.316421	14	0.818609589
0.101462	0.157338	0.5190119	0.5157723	20	1.106787859
-0.317981	0.494506	0.520206	0.2744199	20	0.727616612
0.0188854	0.029568	0.523012	0.9705817	20	1.019064857
-0.340563	0.537966	0.5266961	0.1590762	20	0.711369708
-0.0528428	0.084896	0.5336504	0.04092097	20	0.948529109
0.0594528	0.095953	0.5355204	0.7927047	3	1.061255668
0.046369	0.074907	0.5359047	0.3685665	20	1.047460852
-0.086265	0.139396	0.5360177	0.8957064	20	0.917351100
-0.0647041	0.104948	0.5375425	0.2906062	20	0.937344782
0.220563	0.358018	0.5378504	0.6286329	20	1.246778469
-0.0933143	0.15249	0.5405785	0.7248833	20	0.910907157
0.0317376	0.052106	0.5424605	0.3796465	20	1.032246608
0.0376216	0.061792	0.5426281	0.3485104	20	1.038338251
0.0688309	0.113397	0.5438575	0.8684399	4	1.071255044
-0.0700704	0.116649	0.5480423	0.2235026	20	0.932328181
0.0230487	0.03863	0.5507432	0.4516335	20	1.023316373
-0.0389257	0.065387	0.5516367	0.8757411	20	0.961822169
-0.0645131	0.10883	0.5533221	0.05700824	20	0.937523832
-0.0684286	0.115553	0.5537277	0.2184626	20	0.933860135
0.0409801	0.069342	0.5545315	0.9783798	20	1.041831372
-0.0465555	0.079407	0.5576793	0.4373073	20	0.954511583
0.202601	0.346491	0.558736	0.7975738	12	1.224583761
0.142355	0.244232	0.5599822	0.4852559	9	1.152985885

0.092259	0.159151	0.5621191	0.6257682	20	1.096648817
-0.159172	0.278776	0.5680212	0.977205	20	0.852849656
0.188307	0.331716	0.5702558	0.3039827	20	1.207204070
-0.327166	0.592854	0.581052	0.7846184	13	0.720964053
0.0137991	0.025581	0.5895915	0.6114929	20	1.013894747
-0.0634855	0.117792	0.5899117	0.8489243	20	0.938487727
0.0533836	0.099195	0.5904594	0.09657804	20	1.054834201
-0.177984	0.331594	0.5914386	0.3729459	20	0.836955814
0.05101	0.097735	0.6017252	0.8272484	20	1.052333416
-0.031711	0.060829	0.6021493	0.660398	20	0.968786520
0.0484636	0.092983	0.6022194	0.2451972	20	1.049657163
0.0152397	0.029465	0.6050079	0.5832427	20	1.015356416
-0.127641	0.247035	0.6053705	0.1284777	20	0.880169303
0.0241264	0.047101	0.6084928	0.7124601	20	1.024419796
-0.140857	0.275192	0.6087556	0.9261137	5	0.868613514
0.0243289	0.047668	0.6097823	0.6514057	20	1.024627262
0.0353862	0.069556	0.610932	0.04660979	20	1.036019742
-0.0696806	0.137749	0.6129614	0.2318479	20	0.932691674
-0.0163813	0.032656	0.6159277	0.7749735	13	0.983752143
0.30751	0.621512	0.6207577	0.1758854	12	1.360034408
-0.0186918	0.038096	0.6236768	0.5227491	20	0.981481808
0.130642	0.272401	0.6315153	0.4788211	16	1.139559745
-0.0320298	0.067939	0.6373217	0.8624254	20	0.968477721
0.143909	0.308949	0.6413577	0.4981759	20	1.154779018
0.143909	0.308949	0.6413577	0.4981759	20	1.154779018
0.0373146	0.080414	0.6426249	0.2441564	20	1.038019530
0.0535718	0.115839	0.6437458	0.3338233	15	1.055032740
0.0221038	0.048298	0.6471979	0.9103076	13	1.022349898
0.0602394	0.131882	0.6478395	0.07992411	20	1.062090780
-0.0219526	0.04822	0.6489186	0.510176	20	0.978286604
0.0466854	0.104242	0.6542577	0.7956404	11	1.047792321
-0.0667399	0.149432	0.6551461	0.2415319	17	0.935438477
-0.029547	0.066227	0.6554925	0.9188469	20	0.970885244
0.0240267	0.053948	0.6560529	0.7829085	20	1.024317666
0.0322023	0.073021	0.6592127	0.559162	20	1.032726404
0.0198154	0.045332	0.6620252	0.1606317	20	1.020013028
-0.0790325	0.181177	0.66268	0.3281222	20	0.924009893
-0.0306042	0.071466	0.6684803	0.07846426	20	0.969859367

0.101978	0.23871	0.6692295	0.7261619	20	1.1073591095
-0.0388539	0.090965	0.6692838	0.9295467	20	0.9618912311
-0.0765755	0.18062	0.6715949	0.5970398	20	0.9262829772
0.0374708	0.088409	0.6716876	0.1301119	20	1.0381816817
-0.086443	0.204119	0.6719352	0.615843	20	0.9171878270
0.026169	0.061931	0.6726221	0.707513	19	1.0265144147
-0.175201	0.416002	0.6736421	0.6599046	20	0.8392883068
-0.0391771	0.093124	0.6739742	0.2355639	20	0.9615803981
-0.0971174	0.232386	0.6760095	0.6109645	20	0.9074494653
-0.0529839	0.12685	0.676173	0.1346052	18	0.9483952815
0.0437574	0.104972	0.6767896	0.7754612	20	1.0447288729
0.0809839	0.198384	0.683114	0.4869432	20	1.0843534383
-0.0471725	0.115837	0.6838384	0.7692233	18	0.9539228317
-0.0233619	0.057439	0.6842106	0.1488459	20	0.9769088764
-0.147566	0.363216	0.6845407	0.1395946	20	0.8628054912
-0.0351288	0.086875	0.6859459	0.2147873	20	0.9654810542
-0.0467283	0.115951	0.6869487	0.00176617	20	0.9543466583
-0.0255622	0.064322	0.6910656	0.6642555	20	0.9747617468
-0.111987	0.282112	0.6913971	0.5552949	18	0.8940558801
0.0230079	0.058242	0.692812	0.8984628	20	1.0232746233
-0.137131	0.350743	0.6958166	0.3353077	20	0.8718560055
-0.030028	0.080071	0.707648	0.5484688	20	0.9704183614
-0.0331674	0.088474	0.7077457	0.39539	20	0.9673766071
0.0211465	0.058069	0.7157373	0.6127438	20	1.0213716716
-0.0519989	0.142884	0.7159139	0.6418245	20	0.9493299111
0.0122515	0.034629	0.7234936	0.3233231	20	1.0123268570
0.053555	0.151571	0.7238381	0.04059219	20	1.0550150159
-0.0245878	0.069843	0.7248065	0.9597287	20	0.9757120176
-0.0372509	0.106047	0.7253861	0.2370442	20	0.9634343793
-0.0145826	0.041946	0.7280992	0.4154434	20	0.9855232111
0.0838958	0.243828	0.7307877	0.7890279	20	1.0875155687
-0.0285787	0.085527	0.7382684	0.1440733	20	0.9718258084
0.0766795	0.230154	0.7390092	0.5525859	20	1.0796959783
-0.10666	0.324007	0.7420111	0.9025261	6	0.8988312236
0.0165901	0.050432	0.7421878	0.5688421	20	1.0167284798
0.0560245	0.173491	0.7467529	0.9889854	20	1.0576235951
-0.0601747	0.187574	0.748358	0.6338009	16	0.9416000216
0.0328612	0.104957	0.7542103	0.3628552	20	1.0334070923

-0.08893	0.292849	0.7613776	0.5873919	20	0.914909615
0.0229164	0.076595	0.7647966	0.2867927	6	1.023180998
0.0474786	0.168099	0.7776037	0.9760392	20	1.048623760
0.0279552	0.099892	0.7795882	0.2155021	20	1.028349613
-0.00890368	0.03238	0.7833376	0.9314562	20	0.991135840
-0.0299196	0.109641	0.7849396	0.8780585	20	0.970523560
-0.133379	0.492595	0.7865692	0.8273359	20	0.875133353
-0.0195504	0.073912	0.7913876	0.2455322	14	0.980639469
-0.0221892	0.084915	0.7938529	0.269364	20	0.978055169
-0.0315702	0.120824	0.7938675	0.4084994	17	0.968922935
-0.0733042	0.282046	0.7949397	0.8426295	20	0.929318088
-0.0312686	0.12055	0.7953393	0.8053136	20	0.969215206
-0.0140359	0.054299	0.7960271	0.2263645	20	0.986062143
0.0980819	0.379509	0.7960643	0.2168399	20	1.103053121
0.0699492	0.272401	0.7973431	0.4406668	20	1.072453699
0.02228	0.088669	0.8016042	0.7357903	20	1.022530052
0.12005	0.481124	0.802959	0.3975543	16	1.127553227
-0.014544	0.058634	0.8040983	0.937555	20	0.985561253
-0.0747525	0.302848	0.8050382	0.5230256	20	0.927973131
0.0619218	0.25131	0.805376	0.08694798	20	1.063879146
-0.0508447	0.207643	0.8065605	0.4595653	20	0.950426260
-0.0552081	0.225468	0.8065647	0.1827366	20	0.946288204
0.0407354	0.16903	0.8095591	0.8407435	20	1.041576467
0.0220302	0.092645	0.8120419	0.1309698	20	1.022274656
-0.0112245	0.049589	0.8209299	0.06737116	20	0.988838259
0.0447466	0.201142	0.8239536	0.9424181	20	1.045762830
-0.00971955	0.044209	0.8259825	0.6128957	20	0.990327532
-0.091352	0.416968	0.8265827	0.8484815	15	0.912696385
-0.00996133	0.046483	0.830311	0.7155802	20	0.990088119
-0.0516431	0.242837	0.8315877	0.6901601	20	0.949667742
-0.0303006	0.14448	0.8338844	0.7457033	20	0.970153861
0.0238619	0.117171	0.8386273	0.04617591	20	1.024148873
-0.0206765	0.107077	0.8468808	0.8368877	20	0.979535793
-0.00785809	0.043689	0.8572605	0.8453551	20	0.992172704
-0.0194665	0.111686	0.8616318	0.3750119	20	0.980721748
0.0119222	0.068438	0.8617051	0.2198145	20	1.011993552
0.0280681	0.16121	0.86178	0.1173102	20	1.028465720
-0.0198921	0.114938	0.8625983	0.1482568	20	0.980304442

-0.0222977	0.133413	0.8672656	0.6963657	20	0.977949056
0.0503066	0.301094	0.8673079	0.03945132	20	1.051593465
0.014876	0.090271	0.8691071	0.5906918	20	1.014987198
0.0141676	0.087986	0.8720771	0.7195302	20	1.014268436
0.0270207	0.169322	0.8732106	0.3644307	20	1.027389069
0.00626764	0.040364	0.8766012	0.387195	20	1.006287322
-0.0283897	0.188249	0.8801259	0.9763323	20	0.972009500
0.0199185	0.132925	0.8808847	0.3566026	20	1.020118197
-0.060975	0.411357	0.882162	NA	NA	0.940846760
-0.0074336	0.050567	0.8831289	0.6751643	20	0.992593960
-0.0386717	0.267691	0.8851341	0.6620988	20	0.962066503
0.0163799	0.117636	0.8892588	0.0474283	20	1.016514786
-0.00408799	0.030096	0.8919541	0.2082621	20	0.995920354
0.00302893	0.02269	0.893806	0.5161692	20	1.003033521
-0.0131219	0.100148	0.8957554	0.03239692	20	0.986963816
0.0284873	0.221673	0.8977451	0.8063846	14	1.028896943
-0.0276104	0.223833	0.901828	0.2675091	20	0.972767283
0.0321203	0.263191	0.9028658	0.1277004	20	1.032641724
-0.00659766	0.054391	0.9034529	0.9578769	20	0.993424056
-0.005228	0.043476	0.9042841	0.9835392	20	0.994785642
-0.0135427	0.113446	0.9049782	0.02801152	20	0.986548589
-0.00383639	0.032228	0.9052433	0.9140624	20	0.996170959
0.00488244	0.041122	0.9054882	0.2310719	20	1.004894378
0.00421034	0.037657	0.9109759	0.02043384	20	1.004219215
-0.00248674	0.022352	0.9114144	0.05155928	20	0.997516349
-0.00526957	0.04863	0.9137096	0.3097254	20	0.994744289
0.0103892	0.103851	0.9203134	0.3834924	20	1.010443355
-0.00782164	0.080689	0.9227774	0.4368581	18	0.992208869
-0.00283708	0.030422	0.9256985	0.2153893	20	0.997166940
0.00992009	0.107841	0.9267075	0.3027791	20	1.009969457
0.00848641	0.098274	0.9311847	0.116986	18	1.008522521
-0.00690986	0.091001	0.9394734	0.7821311	20	0.993113958
0.015122	0.206903	0.9417367	0.8826718	20	1.015236915
0.0232765	0.334953	0.944598	0.2054602	16	1.023549511
0.00854415	0.123349	0.9447763	0.2313554	20	1.008580755
0.00650766	0.097796	0.9469451	0.4765502	20	1.006528880
-0.0126336	0.196866	0.9488322	0.1142668	20	0.987445868
0.00224306	0.035616	0.9497831	0.2731039	20	1.002245577

-0.00717091	0.119923	0.9523182	0.00421305	20	0.9928547396
-0.0115158	0.192996	0.9524196	0.4329412	20	0.9885502530
-0.00312631	0.065657	0.9620226	0.4543886	20	0.9968785718
-0.00359606	0.08159	0.9648448	0.02023185	20	0.9964103980
-0.00670121	0.184715	0.9710602	0.06099691	20	0.9933211930
-0.0146612	0.428051	0.972677	0.7420265	8	0.9854457520
-0.00106793	0.033104	0.9742645	0.4650466	20	0.9989326400
0.00118333	0.042722	0.9779028	0.3892432	20	1.0011840304
-0.00303585	0.13118	0.9815365	0.7803145	11	0.9969687535
-0.00978636	0.429071	0.9818032	0.8221715	5	0.9902613705
-0.00660857	0.292717	0.981988	0.08890693	20	0.9934132185
-0.00260126	0.130265	0.9840681	0.9921716	13	0.9974021203
-0.00453738	0.286771	0.9873761	NA	NA	0.9954728983
-0.00123521	0.103849	0.9905099	0.7928668	20	0.9987655525
0.0002476	0.197336	0.9989989	0.1246228	20	1.0002476256

or_lci95	or_uci95
0.35242	0.808073353942024
0.14757	0.768245327495847
0.77322	0.970043262464902
0.28149	0.870624490203997
0.61528	0.948960408899095
0.79142	0.975335364047893
0.83893	0.982877198369934
1.06413	1.99549551909651
1.06232	2.02764690376418
1.02482	1.34059359336604
1.04019	1.6035795674018
0.39410	0.928509561918071
0.43779	0.9385506643713
0.74805	0.978689001969836
1.14481	12.7098265706739
1.06183	5.30183125133552
1.03076	2.61673301799432
1.01036	1.48522903992307
0.68555	0.99261597287778
0.69161	0.99395236898485
1.00049	1.2671406481775
0.27375	0.997328295372454
0.99843	2.29136868943043
0.99911	1.37378167528201
0.59748	1.00174147460873
0.99703	1.10836121332164
0.99095	1.36048959052622
0.99606	1.14284606105427
0.99333	1.19265952927166
0.97628	1.80330223657831
0.98844	1.28191408003627
0.99219	1.17519840422341
0.32881	1.05561464586859
0.90897	1.00496790733382
0.16580	1.1009165718687
0.93550	3.05064950375784

0.98482	1.27021283875525
0.58970	1.03509983396214
0.99094	1.14764706963574
0.96066	1.81844125349736
0.98652	1.21648354422209
0.98275	1.28214146048101
0.92930	2.63965250077469
0.73912	1.02457036436237
0.96101	1.60619945265175
0.71748	1.02843807165146
0.88119	1.01081113045323
0.93260	2.26776326521313
0.96848	1.42227488867027
0.98952	1.11950959521218
0.86465	1.01367971464049
0.58948	1.05119919189219
0.51006	1.06607073598406
0.57324	1.05542401907767
0.36258	1.10677887257323
0.95445	1.59129488838024
0.97231	1.31796521569716
0.47708	1.07920165558968
0.98775	1.1258932628294
0.71308	1.04276298221934
0.85771	3.34008321049455
0.89881	2.30911644494634
0.78016	1.03226575867169
0.65879	1.05695397194147
0.90128	2.11373219969866
0.84750	1.02352474226605
0.43576	1.12842295
0.97637	1.17320451199373
0.62612	1.07301808701753
0.85761	2.73910898620875
0.94302	1.46444562809951
0.21449	1.26827019391621
0.72034	1.05223815944614
0.61487	1.0790937839837

0.958444	1.30201634308471
0.689702	1.06328490159287
0.970271	1.19834555818732
0.735828	1.05269421829784
0.974419	1.16711037388895
0.978583	1.13604297308116
0.306298	1.22462647606263
0.661760	1.07467825595999
0.803120	3.4956700708039
0.939770	1.42552396375365
0.958791	1.24901355029535
0.495964	1.14337928195381
0.841541	1.03444251835955
0.962749	1.2116579835099
0.748582	1.06141210489909
0.884012	1.02625082737899
0.876959	1.02810736680854
0.656440	1.09341365940804
0.205235	1.39971342606453
0.839840	1.03828372339697
0.781197	1.05468949703283
0.929906	1.40027835239808
0.779391	1.05557664864768
0.821558	1.0437209
0.910844	1.02130831177979
0.482740	1.18213513516527
0.740305	1.07314094603741
0.892091	1.62625333244242
0.894041	1.60802243053955
0.876508	1.71759723881863
0.940723	1.28383419845399
0.940229	1.28657588934866
0.903975	1.50808199281364
0.403531	1.25336182351091
0.975020	1.10637862747002
0.945682	1.24944205541517
0.867109	1.74281154563311
0.861333	1.78155589354422

0.89568	1.02929214487517
0.94684	1.22667368381779
0.83163	1.05062545683208
0.78939	1.06665621217116
0.97810	1.08391826187493
0.87602	1.60864697906427
0.91087	1.39730681450303
0.91982	1.33897826725697
0.92836	1.29141170373974
0.89859	1.03157521401872
0.85075	1.73939675398079
0.46812	1.24977888041709
0.68295	1.11973265956789
0.94836	1.19572657838267
0.33198	1.39540748979751
0.73457	2.71152899341383
0.92720	1.27534571643381
0.83080	1.80352982768127
0.82119	1.84683794908139
0.92828	1.02435964010787
0.40233	1.34312533791063
0.95978	1.13361523410575
0.91914	1.28905455168967
0.71443	1.11813419630192
0.54570	1.22671815890511
0.34242	1.43820964792919
0.70061	1.13040683624107
0.88182	1.04587226856125
0.52073	1.26230427443694
0.92310	1.2496762677855
0.74310	1.11282096353883
0.50842	6.52600366649624
0.85189	1.05982357119282
0.96849	1.09221851214312
0.90990	1.03485674240284
0.96905	1.09033592409038
0.59495	4.11085698936592
0.90539	1.30926564016158

0.95416	1.1349272727408
0.97493	1.07066844888232
0.82900	1.65239157849798
0.64679	3.19542020901545
0.82639	1.66033368960266
0.36299	1.46513876775464
0.79276	1.09217538347005
0.81933	1.68983234521644
0.64661	3.14488222147612
0.90252	1.03988200293499
0.94484	1.15939217880268
0.93597	1.18761456017515
0.54427	1.2668070995695
0.75561	2.04711220594294
0.76364	1.11213499628841
0.67505	2.70369155019337
0.72701	1.13556120218848
0.55419	4.3379230775105
0.74758	2.05590528721057
0.74758	2.05590528721057
0.74181	2.0955982636873
0.74181	2.0955982636873
0.72166	2.24260325840005
0.72166	2.24260325840005
0.49416	5.6240236517347
0.70633	1.15318647675416
0.89150	1.04964851866339
0.55469	1.28536494253626
0.67074	1.19027279458593
0.90752	1.24839957627503
0.96520	1.08360596498649
0.80170	1.10280271270661
0.27358	1.77726523851348
0.85887	1.06991729596942
0.91167	1.04215948474875
0.76421	1.12815529897994
0.92668	1.03489751656174
0.73096	1.1528748817453

0.86466	1.06901160935717
0.65036	2.54842968582142
0.77288	1.74292399546163
0.37213	1.58517098740542
0.91453	1.04259931047477
0.70867	2.0826454988225
0.66993	2.34750898601179
0.30903	1.74528614124093
0.92501	1.17618794748893
0.59356	1.28609312801321
0.84366	1.08705826895853
0.71666	1.17776877513584
0.92331	1.04017758356036
0.81481	1.10648758015053
0.44954	1.49067321490441
0.81308	1.50658346303227
0.27603	1.91793820982266
0.96168	1.07986806971179
0.24783	2.04183795383592
0.80313	1.12025122478779
0.87931	1.28084685418556
0.90443	1.21311023521688
0.69803	1.20556840365122
0.76307	1.15141771642923
0.61807	2.51501377330518
0.67557	1.22822016363157
0.93203	1.14323888359862
0.91989	1.17202582277902
0.85776	1.33788367705405
0.74178	1.17182415323988
0.94869	1.10380608413669
0.84612	1.09333603416745
0.75743	1.16043356706865
0.74459	1.17123093856442
0.90943	1.1935005361242
0.81693	1.11525338699013
0.62093	2.4150581367423
0.71438	1.86088042033652

0.80277	1.4980959872828
0.49382	1.47289369
0.63011	2.31282647773355
0.22556	2.30441728565477
0.96431	1.06602594489699
0.74500	1.18221147991744
0.86845	1.28121009236878
0.43696	1.60310157951358
0.86887	1.2745212271858
0.85990	1.0914577979598
0.87478	1.25949329845697
0.95837	1.07572096295553
0.54235	1.42838952024166
0.93408	1.12349521767149
0.50649	1.48961741718925
0.93323	1.12497181723958
0.90398	1.18734076582656
0.71200	1.22177832395315
0.92275	1.04877713487029
0.40226	4.59823953288315
0.91086	1.0575733042758
0.66813	1.94361173248039
0.84773	1.10642204261916
0.63025	2.11583395553952
0.63025	2.11583395553952
0.88665	1.21522062904643
0.84074	1.32394542913503
0.93001	1.12385742798896
0.82016	1.37537733647811
0.89006	1.07525476048453
0.85416	1.28530972444442
0.69793	1.25375972876913
0.85269	1.10545686719933
0.92153	1.13856031645679
0.89501	1.19163136587842
0.93329	1.11478986
0.64782	1.31794594191478
0.84309	1.11568566265874

0.69357	1.76800112406744
0.80481	1.14962627580771
0.65012	1.31974653602823
0.87300	1.23460740394598
0.61476	1.36838366944584
0.90917	1.1589953635948
0.37136	1.89678987914025
0.80115	1.15412786095171
0.57545	1.4309794787853
0.73962	1.21609158825953
0.85045	1.28338679858648
0.73502	1.59970068520699
0.76017	1.19705936480076
0.87289	1.09331977856814
0.42338	1.75828228843729
0.81431	1.1447030060522
0.76033	1.19785883636498
0.85930	1.10573350480295
0.51431	1.55418690779605
0.91288	1.14701352762889
0.43841	1.73381690211421
0.82947	1.13531637433615
0.81336	1.15055065706267
0.91149	1.14449299521164
0.71745	1.25615282927154
0.94589	1.08342177196335
0.78386	1.41996774987553
0.85088	1.11885435447517
0.78262	1.18601769456941
0.90774	1.06997017686864
0.67434	1.75382423353899
0.82183	1.1491868576826
0.68768	1.69516713382855
0.47629	1.69620553819455
0.92103	1.12236404134244
0.75275	1.48596866609515
0.65192	1.35998047088299
0.84125	1.26944134770953

0.51534	1.62426278163331
0.88054	1.18891768396998
0.75427	1.45783526465813
0.84549	1.25074920529099
0.93018	1.05607764284224
0.78284	1.20319047765607
0.33324	2.29815631659407
0.84838	1.13350785408788
0.82809	1.15516593924944
0.76461	1.22782564899608
0.53466	1.61527619398486
0.76525	1.22753660375302
0.88651	1.09679285627315
0.52426	2.32081760606159
0.62879	1.82915691772237
0.85940	1.21661357379815
0.43913	2.89519666106216
0.87856	1.10558967592083
0.51256	1.6800601225383
0.65008	1.74105177674138
0.63265	1.42780111641693
0.60827	1.47212830465998
0.74784	1.45068259378949
0.85252	1.22582418273575
0.89725	1.08977421000632
0.70504	1.55113189614235
0.90812	1.07996551492356
0.40308	2.06660110604851
0.90387	1.08452745849425
0.59001	1.52854670808357
0.73089	1.28772899765957
0.81400	1.2885493619499
0.79409	1.20827584263782
0.91074	1.08087741651661
0.78791	1.22071659590331
0.88495	1.15726686249449
0.74983	1.41063463659112
0.78257	1.22799944773788

0.75292	1.27022211642371
0.58284	1.89733608150977
0.85039	1.21143616862564
0.85360	1.20516942654396
0.73723	1.43174190735404
0.92974	1.08913118018186
0.67209	1.40576036295986
0.78614	1.3237273403295
0.42011	2.10704126637404
0.89893	1.09601218701145
0.56930	1.62580432084508
0.80719	1.28011057382338
0.93887	1.05643493842925
0.95940	1.04864821242663
0.81106	1.20101637803116
0.66631	1.58877992673703
0.62730	1.50847963596524
0.61647	1.72974614370521
0.89296	1.1051803164891
0.91352	1.08326992587603
0.78986	1.23221265525392
0.93519	1.06112504236436
0.92707	1.08924083507227
0.93276	1.08114218392161
0.95475	1.04218825291622
0.90430	1.09422355985945
0.82435	1.23854482219135
0.84707	1.16221608884242
0.93944	1.05843314344851
0.81754	1.24768327290338
0.83182	1.22275173821699
0.83087	1.18702705010633
0.67678	1.52295401536469
0.53087	1.97345217059527
0.79197	1.28442120146623
0.83096	1.21919016603312
0.67133	1.45240929457924
0.93466	1.07470931890922

0.78488	1.25593230587852
0.67720	1.44304629248306
0.87650	1.13378545921248
0.84915	1.16920022652673
0.69160	1.42666590921302
0.42586	2.28032678625318
0.93617	1.06589511529745
0.92076	1.08862871218409
0.77093	1.28927100985503
0.42708	2.29605584294108
0.55971	1.76317603831633
0.77265	1.2875203252074
0.56744	1.7463602763945
0.81482	1.22422602746056
0.67940	1.47259500649185

Supplementary Table S17： Smoking-related circulating proteins significant

ProbeCh	Gene	Probe_bp	topSNP	topSNP_	topSNP_bp	A1	A2
21	TFF2	42348677	rs225344	21	42355775	A	G
9	PTGR1	1.12E+08	rs703610	9	1.12E+08	G	A
10	IL2RA	6036530	rs127224	10	6053965	A	C
7	GPR37	1.25E+08	rs414100	7	1.24E+08	C	A
12	STAB2	1.04E+08	rs115840	12	1.04E+08	G	A
5	CD74	1.5E+08	rs790782	5	1.5E+08	A	G
11	MMP7	1.03E+08	rs115688	11	1.03E+08	A	G
2	INHBB	1.2E+08	rs170502	2	1.21E+08	A	G
11	YAP1	1.02E+08	rs107915	11	1.02E+08	A	G
12	CD27	6448336	rs25680	12	6445462	A	G
4	AFM	73492873	rs131327	4	73480179	C	T
14	NPC2	74485184	rs185905	14	73689254	A	G
8	ANGPT2	6531520	rs196858	8	6421990	T	C
16	PLA2G1	68253181	rs7672	16	68260897	G	C
19	CXCL17	42435612	rs116685	19	42450984	G	A
4	ADH4	99140724	rs180075	4	99144358	G	T
22	RTN4R	20262330	rs663353	22	20187189	A	G
11	RELT	73386936	rs112357	11	73387281	A	G
19	IFI30	18175640	rs725944	19	18172201	C	A
17	ERBB2	39709170	rs376435	17	39760570	T	C
3	HEG1	1.25E+08	rs643886	3	1.25E+08	G	C
1	TNFRSF	2560510	rs249536	1	2557491	A	G

ly associated with uIAs risk (UKB-PPP)

Freq	b_GWAS	se_GWAS	p_GWAS	b_eQTL	se_eQTL	p_eQTL
0.710736	-0.078492	0.025969	0.0025068	0.124985	0.0080594	3.06332
0.033797	-0.145836	0.071467	0.0412905	0.757723	0.0168611	0
0.144135	-0.123775	0.050938	0.0151022	0.956182	0.0133443	0
0.248509	0.0756717	0.031684	0.0169239	-0.784611	0.0085	0
0.075547	0.0949437	0.048208	0.0489024	0.800343	0.0144	0
0.101392	-0.116523	0.051306	0.0231393	-0.087035	0.0111797	6.96625
0.0666	-0.106254	0.052378	0.0424991	0.567004	0.0152635	4.77401
0.450298	0.0579008	0.024951	0.0203109	0.364573	0.0074933	0
0.468191	0.0538781	0.024874	0.0303082	0.0623571	0.0069075	1.75710
0.196819	0.075116	0.032346	0.0202172	0.293625	0.0086401	3.85218
0.975149	-0.21812	0.109514	0.0464034	0.336036	0.0262031	1.19867
0.012923	0.722455	0.294132	0.0140404	-1.02753	0.0468091	8.37551
0.352883	-0.060116	0.025664	0.0191571	-0.156687	0.0078044	1.17839
0.683897	0.0660283	0.027038	0.0146023	-0.245434	0.0082117	2.78729
0.089463	-0.128364	0.047766	0.0072015	0.117925	0.012377	1.60767
0.604374	-0.057832	0.02508	0.0211145	0.130044	0.0074633	5.38397
0.780318	-0.060472	0.029234	0.0385887	-0.297977	0.0085471	2.71503
0.198807	-0.062176	0.027308	0.022795	0.3988	0.0084122	0
0.241551	0.0729479	0.029319	0.0128449	-0.507285	0.0087716	0
0.159046	0.0753722	0.031883	0.0180788	0.200177	0.0100316	1.36394
0.62326	0.054052	0.025642	0.035034	0.108951	0.007257	6.00895
0.482107	-0.057702	0.024818	0.0200701	0.114794	0.0074094	3.86276

b_SMR	se_SMR	p_SMR	p_HEIDI	nsnp_HI	or	or_lci95
-0.628007	0.211686	0.00301017	0.00051292	20	0.53365	0.35242
-0.192466	0.094416	0.04149996	0.00300552	20	0.82492	0.68555
-0.129447	0.053303	0.01516004	0.05070193	20	0.87858	0.79142
-0.096445	0.040395	0.0169608	0.06088152	20	0.90805	0.83893
0.118629	0.060272	0.04904366	0.07281723	20	1.12595	1.00049
1.33881	0.614064	0.02923938	0.08155582	17	3.81450	1.14481
-0.187395	0.092515	0.04280845	0.08409285	20	0.82911	0.69161
0.158818	0.068518	0.02045381	0.09094131	20	1.17212	1.02482
0.864025	0.410218	0.0351818	0.10757	20	2.37269	1.06183
0.255823	0.110416	0.02050967	0.1527912	20	1.29152	1.04019
-0.649097	0.329807	0.04905488	0.1536098	12	0.52251	0.27375
-0.703099	0.288038	0.0146469	0.2063568	19	0.49504	0.28149
0.38367	0.164901	0.01998265	0.2156055	20	1.46766	1.06232
-0.269027	0.11053	0.0149338	0.233109	20	0.76412	0.61528
-1.08852	0.420854	0.00969669	0.2470769	20	0.33671	0.14757
-0.444709	0.194536	0.02225456	0.4501713	20	0.64101	0.43779
0.20294	0.09828	0.03893065	0.4795861	20	1.22499	1.01036
-0.155907	0.068554	0.02295261	0.6279111	20	0.85563	0.74805
-0.143801	0.05785	0.01292814	0.6802994	20	0.86606	0.77322
0.376528	0.16039	0.01889588	0.7074262	20	1.45721	1.06413
0.496113	0.23766	0.03684401	0.8548069	20	1.64232	1.03076
-0.50266	0.218615	0.02148825	0.9468437	20	0.60491	0.39410

or_uci95
0.808073353942024
0.99261597287778
0.975335364047893
0.982877198369934
1.2671406481775
12.7098265706739
0.99395236898485
1.34059359336604
5.30183125133552
1.6035795674018
0.997328295372454
0.870624490203997
2.02764690376418
0.948960408899095
0.768245327495847
0.9385506643713
1.48522903992307
0.978689001969836
0.970043262464902
1.99549551909651
2.61673301799432
0.928509561918071

Supplementary Table S18： Associations between smoking-associated circulating p

probeID	ProbeCh	Gene	Probe_bp	topSNP	topSNP_	topSNP_b	A1	A2
9266_1	6	TREM1	4.1E+07	rs44240	6	4.1E+07	G	A

ɔroteins and the risk of uIAs（deCODE）

Freq	b_GWAS	se_GWAS	p_GWAS	b_eQTL	se_eQTL	p_eQTL	b_SMR
0.73062	-0.028114	0.028014	0.31559	-0.3225	0.00891	4.64308⁴	0.087174

se_SMR	p_SMR	p_HEIDI	nsnp_HI	or	or_lci95	or_uci95
0.086898	0.315772	0.325522	20	1.09108	0.92021	1.2936832726425

Supplementary Table S19: Comprehensive mediation analysis of lifetime smokin

exposure	method.x	nsnp.x	b.x	se.x	pval.x	lo_ci.x	up_ci.x	or.x
Lifetime	Inverse v	117	0.919679	0.192702	1.81933	0.54198	1.297370	2.508485
Lifetime	Inverse v	117	0.919679	0.192702	1.81933	0.54198	1.297370	2.508485
Lifetime	Inverse v	117	0.919679	0.192702	1.81933	0.54198	1.297370	2.508485
Lifetime	Inverse v	117	0.919679	0.192702	1.81933	0.54198	1.297370	2.508485
Lifetime	Inverse v	117	0.919679	0.192702	1.81933	0.54198	1.297370	2.508485
Lifetime	Inverse v	117	0.919679	0.192702	1.81933	0.54198	1.297370	2.508485
Lifetime	Inverse v	117	0.919679	0.192702	1.81933	0.54198	1.297370	2.508485
Lifetime	Inverse v	117	0.919679	0.192702	1.81933	0.54198	1.297370	2.508485
Lifetime	Inverse v	117	0.919679	0.192702	1.81933	0.54198	1.297370	2.508485
Lifetime	Inverse v	117	0.919679	0.192702	1.81933	0.54198	1.297370	2.508485
Lifetime	Inverse v	117	0.919679	0.192702	1.81933	0.54198	1.297370	2.508485
Lifetime	Inverse v	117	0.919679	0.192702	1.81933	0.54198	1.297370	2.508485
Lifetime	Inverse v	117	0.919679	0.192702	1.81933	0.54198	1.297370	2.508485
Lifetime	Inverse v	117	0.919679	0.192702	1.81933	0.54198	1.297370	2.508485
Lifetime	Inverse v	117	0.919679	0.192702	1.81933	0.54198	1.297370	2.508485
Lifetime	Inverse v	117	0.919679	0.192702	1.81933	0.54198	1.297370	2.508485
Lifetime	Inverse v	117	0.919679	0.192702	1.81933	0.54198	1.297370	2.508485
Lifetime	Inverse v	117	0.919679	0.192702	1.81933	0.54198	1.297370	2.508485
Lifetime	Inverse v	117	0.919679	0.192702	1.81933	0.54198	1.297370	2.508485

g index influencing aSAH risk via modulation of plasma protein expression

or_lci95	or_uci95	Gene	method.y	nsnp.y	b.y	se.y	pval.y	lo_ci.y
1.71941	3.65968	PGF	Inverse	112	0.29852	0.07050	2.29288	0.16034
1.71941	3.65968	HEG1	Inverse	112	0.40016	0.07280	3.87336	0.25747
1.71941	3.65968	SFRP1	Inverse	112	0.30490	0.08808	0.00053	0.13225
1.71941	3.65968	CEACA	Inverse	112	0.38966	0.07929	8.92494	0.23424
1.71941	3.65968	VWA1	Inverse	112	0.25072	0.08862	0.00466	0.07701
1.71941	3.65968	RELT	Inverse	112	0.23985	0.07166	0.00081	0.09939
1.71941	3.65968	ADH4	Inverse	112	0.20336	0.07531	0.00693	0.05574
1.71941	3.65968	PCSK9	Inverse	112	0.23919	0.07620	0.00169	0.08982
1.71941	3.65968	CTHRC	Inverse	112	0.23216	0.09108	0.01080	0.05363
1.71941	3.65968	CAPG	Inverse	112	0.28347	0.06302	6.86586	0.15994
1.71941	3.65968	FUT3	Inverse	112	0.42524	0.07676	3.03040	0.27479
1.71941	3.65968	QSOX1	Inverse	112	0.22465	0.07056	0.00145	0.08635
1.71941	3.65968	CCL3	Inverse	112	0.32582	0.08864	0.00023	0.15208
1.71941	3.65968	BPIFB1	Inverse	112	0.40835	0.09599	2.09901	0.22021
1.71941	3.65968	INHBB	Inverse	112	0.25859	0.08635	0.00275	0.08932
1.71941	3.65968	CD74	Inverse	112	0.35696	0.08812	5.10846	0.18423
1.71941	3.65968	FGF23	Inverse	112	0.24603	0.08182	0.00263	0.08566
1.71941	3.65968	RBP2	Inverse	112	0.29436	0.07924	0.00020	0.13904
1.71941	3.65968	YAP1	Inverse	112	0.30583	0.08178	0.00018	0.14554

up_ci.y	or.y	or_lci95	or_uci95.y	outcome	fdr	beta_all	beta1
0.43670	1.34786	1.17391	1.54760505609	PGF_P4	0.00035	0.91967	0.29852
0.54286	1.49207	1.29365	1.72092427957	HEG1_C	4.42725	0.91967	0.40016
0.47755	1.35649	1.14139	1.61212950660	SFRP1_	0.00469	0.91967	0.30490
0.54508	1.47648	1.26394	1.72475599353	CEACA	6.80080	0.91967	0.38966
0.42442	1.28495	1.08006	1.52871368454	VWA1_	0.02519	0.91967	0.25072
0.38032	1.27107	1.10450	1.46275285816	RELT_C	0.00643	0.91967	0.23985
0.35098	1.22551	1.05732	1.42046359145	ADH4_I	0.03498	0.91967	0.20336
0.38856	1.27023	1.09398	1.47486943238	PCSK9_	0.01146	0.91967	0.23919
0.41068	1.26132	1.05509	1.50785546	CTHRC	0.04967	0.91967	0.23216
0.40701	1.32774	1.17345	1.50232133578	CAPG_I	0.00013	0.91967	0.28347
0.57570	1.52996	1.31625	1.77838491777	FUT3_F	1.87780	0.91967	0.42524
0.36295	1.25189	1.09019	1.43757818768	QSOX1_	0.02307	0.91967	0.22465
0.49957	1.38517	1.16425	1.64802288188	CCL3_P	0.00239	0.91967	0.32582
0.59649	1.50434	1.24634	1.81575041902	BPIFB1_	0.00033	0.91967	0.40835
0.42785	1.29510	1.09343	1.53396571966	INHBB_	0.01664	0.91967	0.25859
0.52969	1.42898	1.20230	1.69840587774	CD74_P	0.00068	0.91967	0.35696
0.40641	1.27895	1.08944	1.50142185453	FGF23_	0.01607	0.91967	0.24603
0.44968	1.34227	1.14917	1.56781464902	RBP2_P	0.00567	0.91967	0.29436
0.46612	1.35776	1.15667	1.59381229311	YAP1_P	0.00526	0.91967	0.30583

beta2	beta12	beta_dir	se	Z
0.329189	0.0982710715567	0.82140	0.02722	3.6094967252705
0.33784	0.1351923018199	0.78448	0.04278	3.1594381027275
0.186539	0.0568765100169	0.86280	0.02692	2.1125112690462
-0.135601	-0.052838747	0.97251	0.03108	-1.699796472
0.13517	0.0338902231920	0.88578	0.02223	1.5241366710321
-0.108696	-0.026071775	0.94575	0.01757	-1.483078089
-0.459215	-0.093387894	1.01306	0.06471	-1.442998069
-0.0861926	-0.020617164	0.94029	0.01957	-1.053030359
0.291628	0.0677044300462	0.85197	0.08713	0.7769690689442
-0.0406888	-0.011534459	0.93121	0.01793	-0.642983475
0.0614329	0.0261242142788	0.89355	0.06484	0.4028825822022
-0.0616586	-0.013852045	0.93353	0.04079	-0.33951645
0.125784	0.0409841307464	0.87869	0.20491	0.2000061911564
0.0890656	0.0363704518501	0.88330	0.18673	0.1947712378115
0.111272	0.0287740130741	0.89090	0.39093	0.0736031355534
0.913587	0.3261171067158	0.59356	0.03190	10.221354886433
0.49635	0.1221217862679	0.79755	0.02202	5.5451300938069
-0.611297	-0.179943063	1.09962	0.02390	-7.528496516
0.700121	0.2141237331612	0.70555	0.02645	8.0931811659182

P	lci	uci	beta12_p
0.000306792	0.0449087014227	0.151633441690	0.1068536495445
0.001580737	0.0513239356891	0.219060667950	0.1469994232376
0.03464262	0.0041061597392	0.109646860294	0.0618438628214
0.089169215	-0.113766009	0.008088514517	-0.057453459
0.127474574	-0.00969172	0.077472166149	0.0368500513384
0.138053665	-0.060527601	0.008384050033	-0.028348773
0.149021015	-0.220235099	0.033459311264	-0.101543996
0.292327079	-0.058991787	0.017757457845	-0.02241778
0.437177019	-0.103088319	0.238497178682	0.0736174473949
0.520234823	-0.046694833	0.023625915042	-0.01254183
0.687034605	-0.100968547	0.153216975913	0.0284057921925
0.734220707	-0.093818742	0.066114651364	-0.015061824
0.841475739	-0.360647918	0.442616179227	0.0445635106475
0.84557204	-0.329628586	0.402369489630	0.0395468926328
0.941326176	-0.737457822	0.795005848188	0.0312870131596
1.59E-24	0.2635823903044	0.388651823127	0.3545987896486
2.94E-08	0.0789562162928	0.165287356243	0.1327873843737
5.13E-14	-0.226790186	-0.13309594	-0.195658525
5.81E-16	0.1622674224730	0.265980043849	0.2328243905345

lci_p	uci_p
0.04883083665734	0.164876462431727
0.05580635023614	0.238192496239208
0.00446477429011	0.119222951352708
-0.123701849	0.00879493102033655
-0.010538153	0.0842382560814576
-0.065813824	0.00911627735209505
-0.239469497	0.0363815053936088
-0.064143879	0.0193083187899165
-0.112091614	0.259326509262818
-0.050772961	0.02568930211245
-0.109786711	0.166598295807609
-0.102012473	0.071888824196159
-0.392145375	0.481272396328502
-0.358416947	0.437510731807898
-0.801864256	0.864438282217052
0.28660255671927	0.422595022577935
0.08585191686088	0.179722851886653
-0.246597077	-0.144719974
0.17643916992818	0.289209611140965

Supplementary Table S20: Comprehensive mediation analysis of lifetime smokin

exposure	method.x	nsnp.x	b.x	se.x	pval.x	lo_ci.x	up_ci.x	or.x
Lifetime	Inverse v	118	1.09540	0.26851	4.51364	0.56911	1.62169	2.99039
Lifetime	Inverse v	118	1.09540	0.26851	4.51364	0.56911	1.62169	2.99039
Lifetime	Inverse v	118	1.09540	0.26851	4.51364	0.56911	1.62169	2.99039
Lifetime	Inverse v	118	1.09540	0.26851	4.51364	0.56911	1.62169	2.99039
Lifetime	Inverse v	118	1.09540	0.26851	4.51364	0.56911	1.62169	2.99039
Lifetime	Inverse v	118	1.09540	0.26851	4.51364	0.56911	1.62169	2.99039
Lifetime	Inverse v	118	1.09540	0.26851	4.51364	0.56911	1.62169	2.99039
Lifetime	Inverse v	118	1.09540	0.26851	4.51364	0.56911	1.62169	2.99039
Lifetime	Inverse v	118	1.09540	0.26851	4.51364	0.56911	1.62169	2.99039
Lifetime	Inverse v	118	1.09540	0.26851	4.51364	0.56911	1.62169	2.99039
Lifetime	Inverse v	118	1.09540	0.26851	4.51364	0.56911	1.62169	2.99039
Lifetime	Inverse v	118	1.09540	0.26851	4.51364	0.56911	1.62169	2.99039
Lifetime	Inverse v	118	1.09540	0.26851	4.51364	0.56911	1.62169	2.99039
Lifetime	Inverse v	118	1.09540	0.26851	4.51364	0.56911	1.62169	2.99039
Lifetime	Inverse v	118	1.09540	0.26851	4.51364	0.56911	1.62169	2.99039
Lifetime	Inverse v	118	1.09540	0.26851	4.51364	0.56911	1.62169	2.99039
Lifetime	Inverse v	118	1.09540	0.26851	4.51364	0.56911	1.62169	2.99039
Lifetime	Inverse v	118	1.09540	0.26851	4.51364	0.56911	1.62169	2.99039
Lifetime	Inverse v	118	1.09540	0.26851	4.51364	0.56911	1.62169	2.99039
Lifetime	Inverse v	118	1.09540	0.26851	4.51364	0.56911	1.62169	2.99039

ıg index influencing uIAs risk via modulation of plasma protein expression

or_lci95	or_uci95	outcome	Gene	method.y	nsnp.y	b.y	se.y	pval.y
1.76670	5.06166	IAs	CXCL17	Inverse v	112	0.772400	0.07106	1.61625
1.76670	5.06166	IAs	CD74	Inverse v	112	0.35696	0.08812	5.108460
1.76670	5.06166	IAs	AFM	Inverse v	112	0.27963	0.082710	0.00072
1.76670	5.06166	IAs	ADH4	Inverse v	112	0.203364	0.07531	0.006930
1.76670	5.06166	IAs	ANGPT	Inverse v	112	0.450560	0.09180	9.213984
1.76670	5.06166	IAs	NPC2	Inverse v	112	0.54901	0.08931	7.884684
1.76670	5.06166	IAs	PLA2G1	Inverse v	112	0.37504	0.088960	2.49116
1.76670	5.06166	IAs	RTN4R	Inverse v	112	0.242798	0.076638	0.001534
1.76670	5.06166	IAs	YAP1	Inverse v	112	0.305838	0.081780	0.000184
1.76670	5.06166	IAs	RELT	Inverse v	112	0.23985	0.07166	0.000810
1.76670	5.06166	IAs	TNFRSF	Inverse v	112	0.274160	0.082618	0.00090
1.76670	5.06166	IAs	INHBB	Inverse v	112	0.25859	0.08635	0.002750
1.76670	5.06166	IAs	HEG1	Inverse v	112	0.400160	0.07280	3.873368
1.76670	5.06166	IAs	GPR37	Inverse v	112	0.278618	0.07056	7.87181
1.76670	5.06166	IAs	IL2RA	Inverse v	112	0.26031	0.08285	0.001678
1.76670	5.06166	IAs	STAB2	Inverse v	112	0.24807	0.072948	0.00067
1.76670	5.06166	IAs	IFI30	Inverse v	112	0.52960	0.08862	2.29126
1.76670	5.06166	IAs	ERBB2	Inverse v	112	0.33358	0.099438	0.000794
1.76670	5.06166	IAs	CD27	Inverse v	112	0.39162	0.080538	1.15841
1.76670	5.06166	IAs	MMP7	Inverse v	112	0.23019	0.077958	0.003148

lo_ci.y	up_ci.y	or.y	or_lci95	or_uci95	outcome	fdr	beta_all	beta1
0.63311	0.91168	2.16495	1.88347	2.48851	CXCL1	2.90440	1.09540	0.77240
0.18423	0.52969	1.42898	1.20230	1.69840	CD74_P	0.00068	1.09540	0.35696
0.11751	0.44176	1.32264	1.12469	1.55544	AFM_P	0.00585	1.09540	0.27963
0.05574	0.35098	1.22551	1.05732	1.42046	ADH4_I	0.03498	1.09540	0.20336
0.27062	0.63050	1.56920	1.31078	1.87856	ANGPT	2.50871	1.09540	0.45056
0.37396	0.72406	1.73155	1.45348	2.06280	NPC2_P	2.25304	1.09540	0.54901
0.20067	0.54941	1.45505	1.22222	1.73224	PLA2G1	0.00123	1.09540	0.37504
0.09258	0.39300	1.27481	1.09700	1.48143	RTN4R_	0.01060	1.09540	0.24279
0.14554	0.46612	1.35776	1.15667	1.59381	YAP1_P	0.00526	1.09540	0.30583
0.09939	0.38032	1.27107	1.10450	1.46275	RELT_C	0.00643	1.09540	0.23985
0.11222	0.43609	1.31542	1.11876	1.54665	TNFRSF	0.00707	1.09540	0.27416
0.08932	0.42785	1.29510	1.09343	1.53396	INHBB_	0.01664	1.09540	0.25859
0.25747	0.54286	1.49207	1.29365	1.72092	HEG1_C	4.42725	1.09540	0.40016
0.14030	0.41693	1.32130	1.15062	1.51729	GPR37_	0.00281	1.09540	0.27861
0.09792	0.42270	1.29733	1.10287	1.52607	IL2RA_	0.01138	1.09540	0.26031
0.10509	0.39105	1.28155	1.11081	1.47853	STAB2_	0.00551	1.09540	0.24807
0.35589	0.70331	1.69826	1.42746	2.02044	IFI30_P	3.74130	1.09540	0.52960
0.13868	0.52848	1.39596	1.14876	1.69635	ERBB2_	0.00631	1.09540	0.33358
0.23377	0.54948	1.47938	1.26335	1.73235	CD27_P	2.97380	1.09540	0.39162
0.07740	0.38299	1.25885	1.08047	1.46667	MMP7_	0.01867	1.09540	0.23019

beta2	beta12	beta_dir	se	Z	P
-1.08852	-0.840773032	1.93617	0.05595	-15.02519708	5.02E-51
1.33881	0.4779061475	0.61749	0.03591	13.305221056	2.16E-40
-0.649097	-0.181510733	1.27691	0.02345	-7.738257012	1.01E-14
-0.444709	-0.090437893	1.18584	0.01679	-5.383543662	7.30E-08
0.38367	0.1728689353	0.92253	0.04197	4.1179969070	3.82E-05
-0.703099	-0.386013567	1.48141	0.09944	-3.881819664	0.00010368
-0.269027	-0.100897361	1.19630	0.03887	-2.595560391	0.00944368
0.20294	0.0492734555	1.04613	0.02038	2.4174381034	0.01563019
0.864025	0.2642518343	0.83115	0.11269	2.3447466885	0.01904001
-0.155907	-0.037395785	1.13280	0.02024	-1.847516681	0.06467229
-0.50266	-0.137809573	1.23321	0.12006	-1.147817929	0.25104373
0.158818	0.0410690129	1.05433	0.03718	1.1043748973	0.26943055
0.496113	0.1985278783	0.89687	0.21605	0.9188977568	0.35814905
-0.096445	-0.026871358	1.12227	0.03441	-0.780781321	0.43493112
-0.129447	-0.033696555	1.12910	0.06684	-0.504117625	0.61417872
0.118629	0.0294287640	1.06597	0.06304	0.4667939075	0.64064734
-0.143801	-0.076158062	1.17156	0.20788	-0.366342846	0.71410926
0.376528	0.1256041742	0.96980	0.35598	0.3528331706	0.72421351
0.255823	0.1001872770	0.99521	0.82271	0.1217756900	0.90307667
-0.187395	-0.043138254	1.13854	0.45845	-0.094093952	0.92503453

lci	uci	beta12_p	lci_p	uci_p
-0.950449806	-0.731096258	-0.767545196	-0.867669579	-0.667420814
0.4075055097	0.548306785	0.436282509	0.372013474	0.500551544169789
-0.227485044	-0.135536422	-0.165701903	-0.207672043	-0.123731763
-0.123363839	-0.057511946	-0.082561128	-0.112619362	-0.052502895
0.090590312	0.255147557	0.157812769	0.082700273	0.232925266534262
-0.580918704	-0.191108431	-0.35239339	-0.5303231	-0.174463681
-0.177088548	-0.024706174	-0.09210962	-0.161664871	-0.022554369
0.0093237365	0.089223174	0.044981942	0.008511677	0.0814522069285221
0.043360768	0.485142900	0.241236598	0.039584225	0.442888970845414
-0.077068358	0.002276787	-0.034138768	-0.070356024	0.00207848836514796
-0.373131532	0.097512385	-0.125806932	-0.340633327	0.0890194623360876
-0.031818614	0.113956639	0.037492072	-0.029047345	0.104031490234779
-0.22493016	0.621985916	0.181236925	-0.205339678	0.567813529538136
-0.09432669	0.040583972	-0.024530975	-0.086111227	0.0370492774945512
-0.164708138	0.097315028	-0.030761725	-0.150362744	0.088839293682302
-0.094138353	0.152995881	0.026865641	-0.085939294	0.139670576319803
-0.483617365	0.331301241	-0.069525011	-0.441496303	0.302446280674036
-0.572131192	0.823339540	0.114664572	-0.522300944	0.751630089884329
-1.512343624	1.712718178	0.091461381	-1.380624782	1.56354754586009
-0.941718622	0.855442113	-0.039381092	-0.859698845	0.780936662309365

Figure S1. Mediation proportions of eight candidate plasma proteins in the association between lifetime smoking index and risk of aneurysmal subarachnoid haemorrhage and unruptured intracranial aneurysms

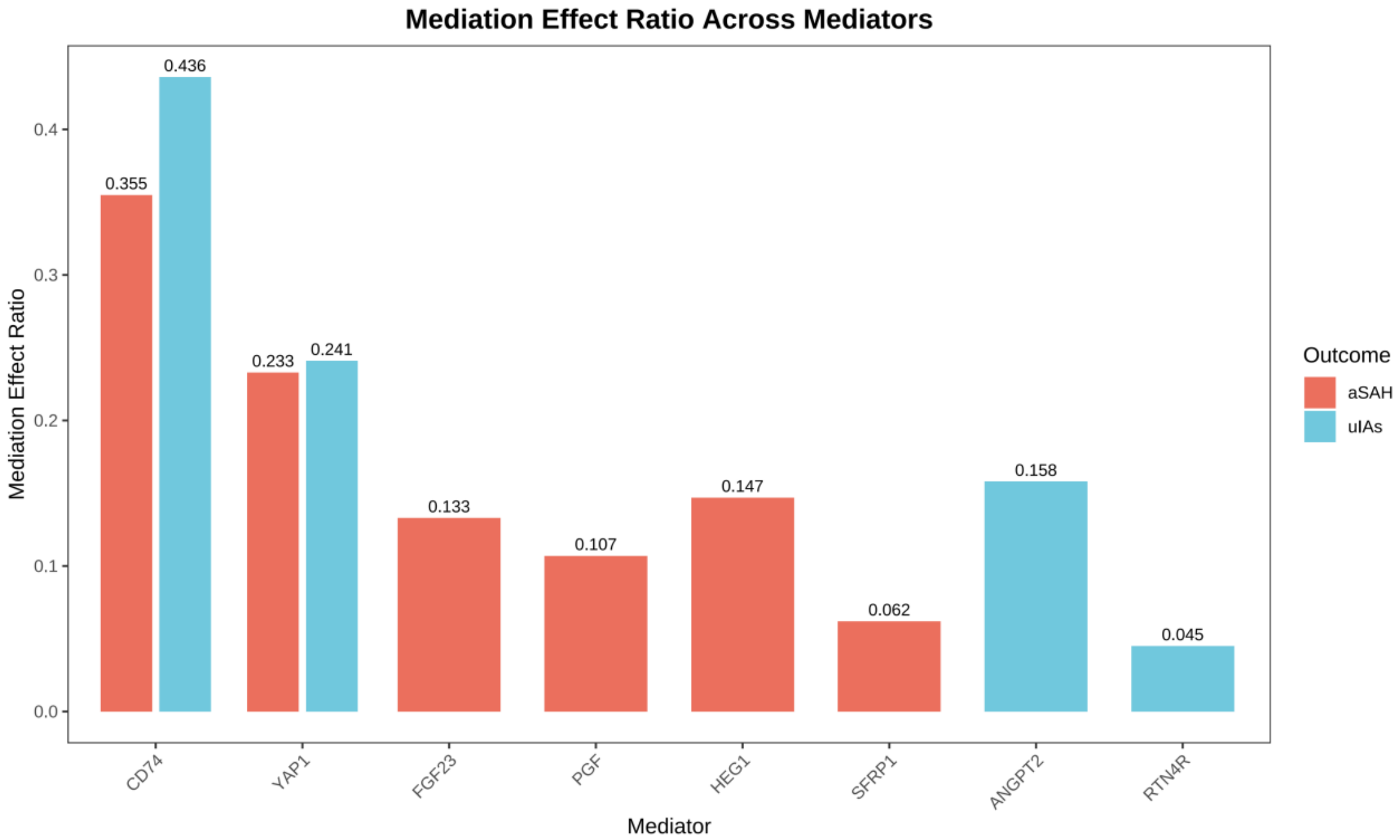

Note: Red bars represent the proportion of the effect of the lifetime smoking index on the risk of aneurysmal subarachnoid haemorrhage that is mediated by plasma proteins, whereas blue bars represent the corresponding proportion for the risk of unruptured intracranial aneurysms.

Figure S2. Protein –protein interaction network of eight candidate plasma proteins at the highest confidence threshold 0.900

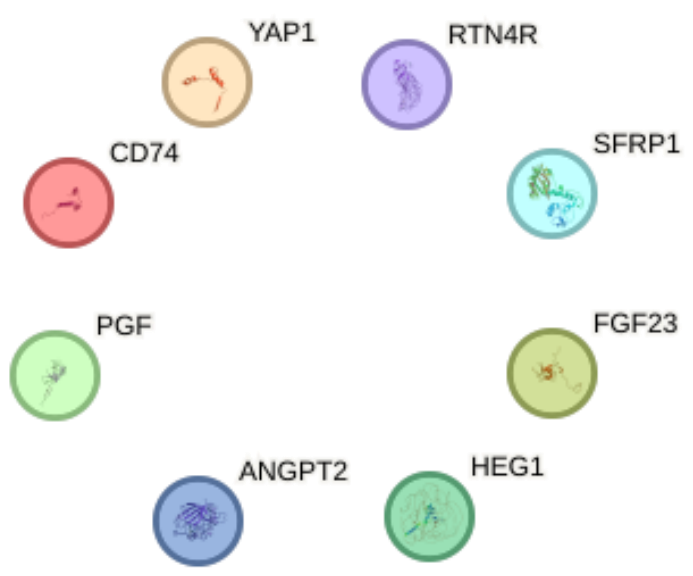

Note: The absence of connecting lines among the eight plasma proteins indicates that there is currently no strong evidence to support their biological interdependence or functional relevance.

Figure S3. Gene ontology enrichment analysis results of the eight candidate plasma proteins

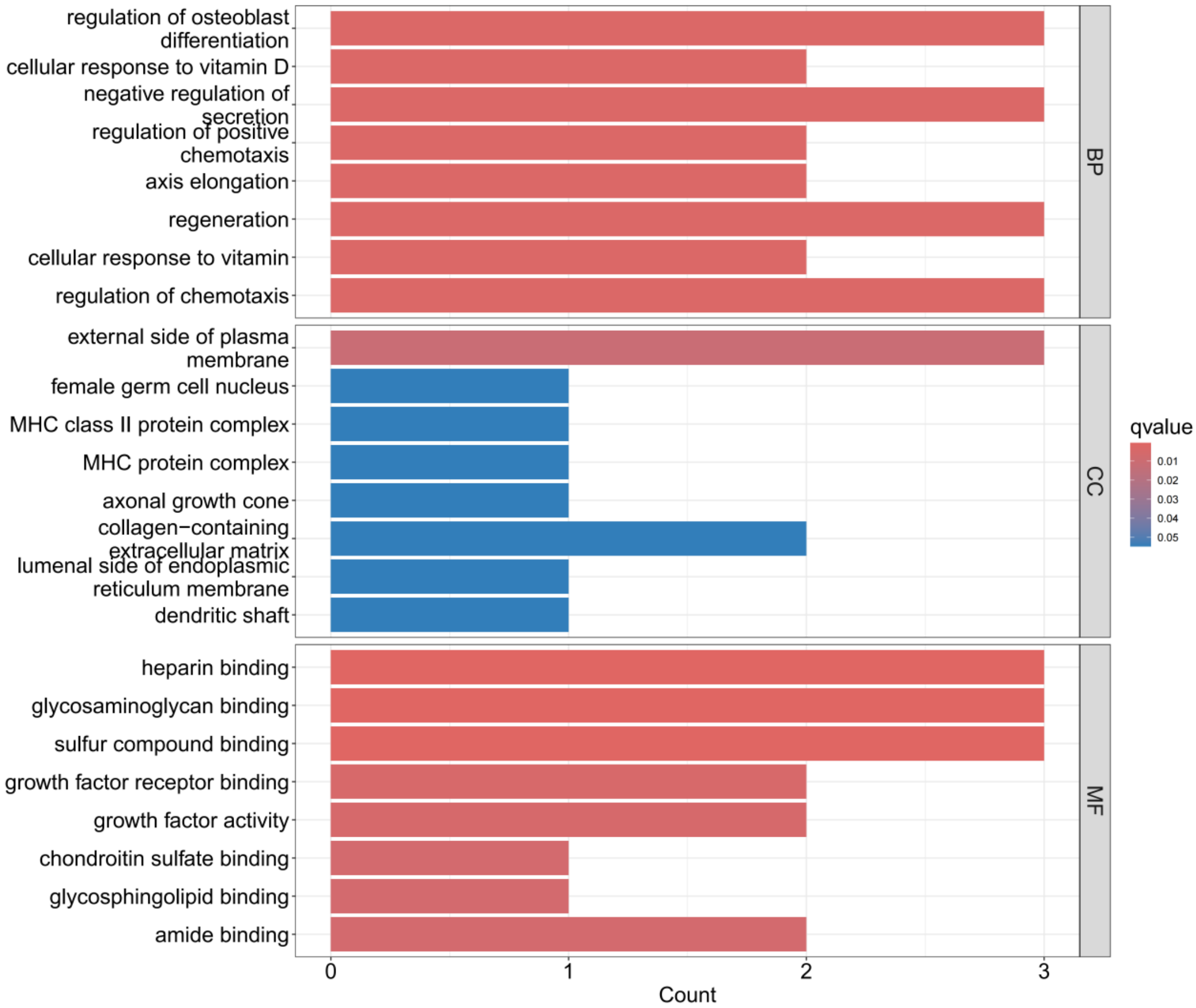

Note: BP are biological process; CC are cellular component; MF are molecular function; Count are number of proteins enriched in the given term; Red indicates higher significance; Blue indicates lower significance.

Figure S4. Kyoto Encyclopedia of genes and genomes enrichment analysis results of the eight candidate plasma proteins

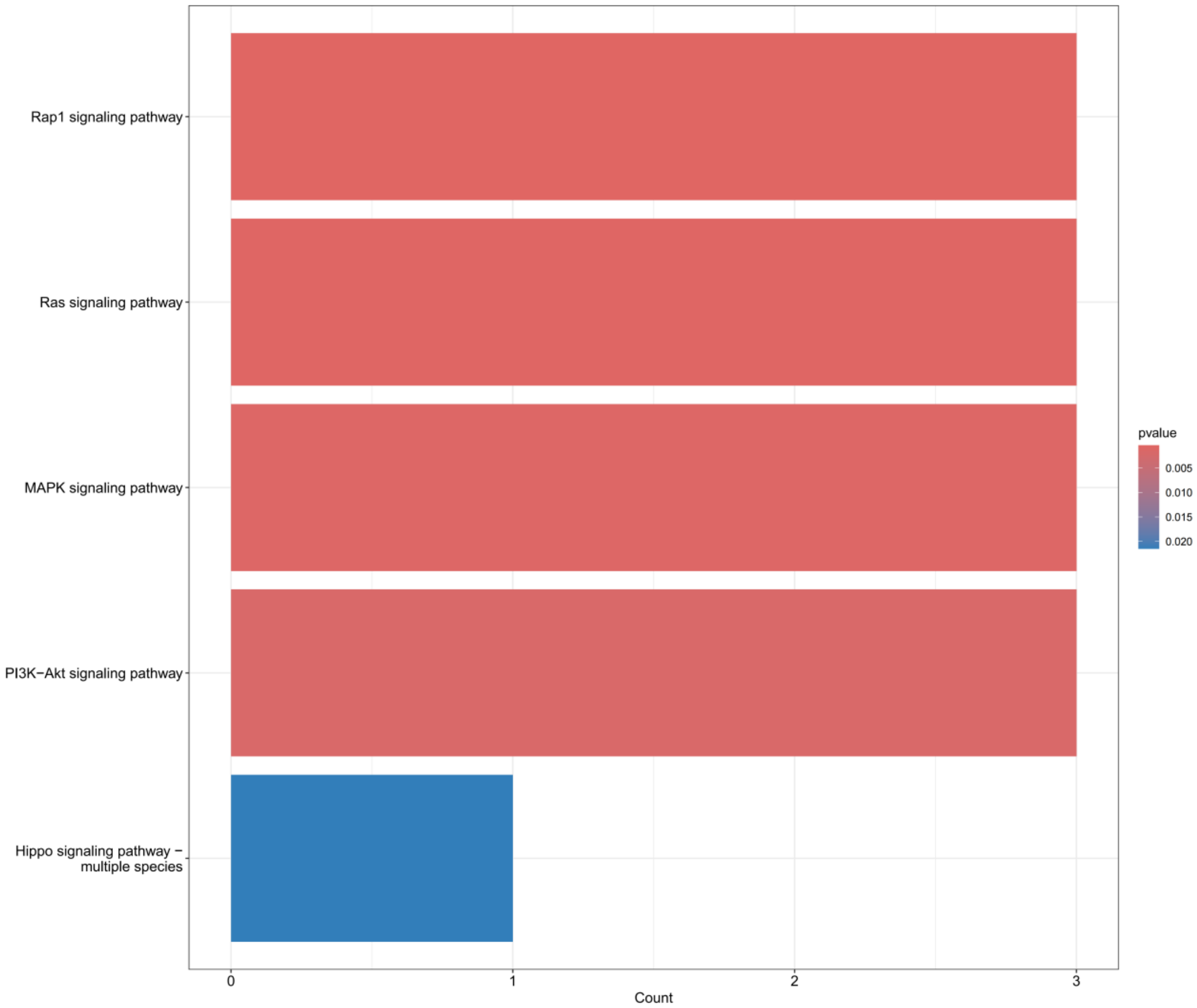

Note: Shown are the significantly enriched pathways with corresponding protein counts and *P* values; Red indicates higher significance; Blue indicates lower significance.

Figure S5. Phenome-Wide Association study from the AstraZeneca online portal. (A) Phenome-Wide Association study of CD74 across the full disease spectrum (binary traits); (B) Phenome-Wide Association study of YAP1 across the full disease spectrum (binary traits); (C) Phenome-Wide Association study of FGF23 across the full disease spectrum (binary traits); (D) Phenome-Wide Association study of PGF across the full disease spectrum (binary traits); (E) Phenome-Wide Association study of HEG1 across the full disease spectrum (binary traits); (F) Phenome-Wide Association study of SFRP1 across the full disease spectrum (binary traits); (G) Phenome-Wide Association study of ANGPT2 across the full disease spectrum (binary traits); (H) Phenome-Wide Association study of RTN4R across the full disease spectrum (binary traits)

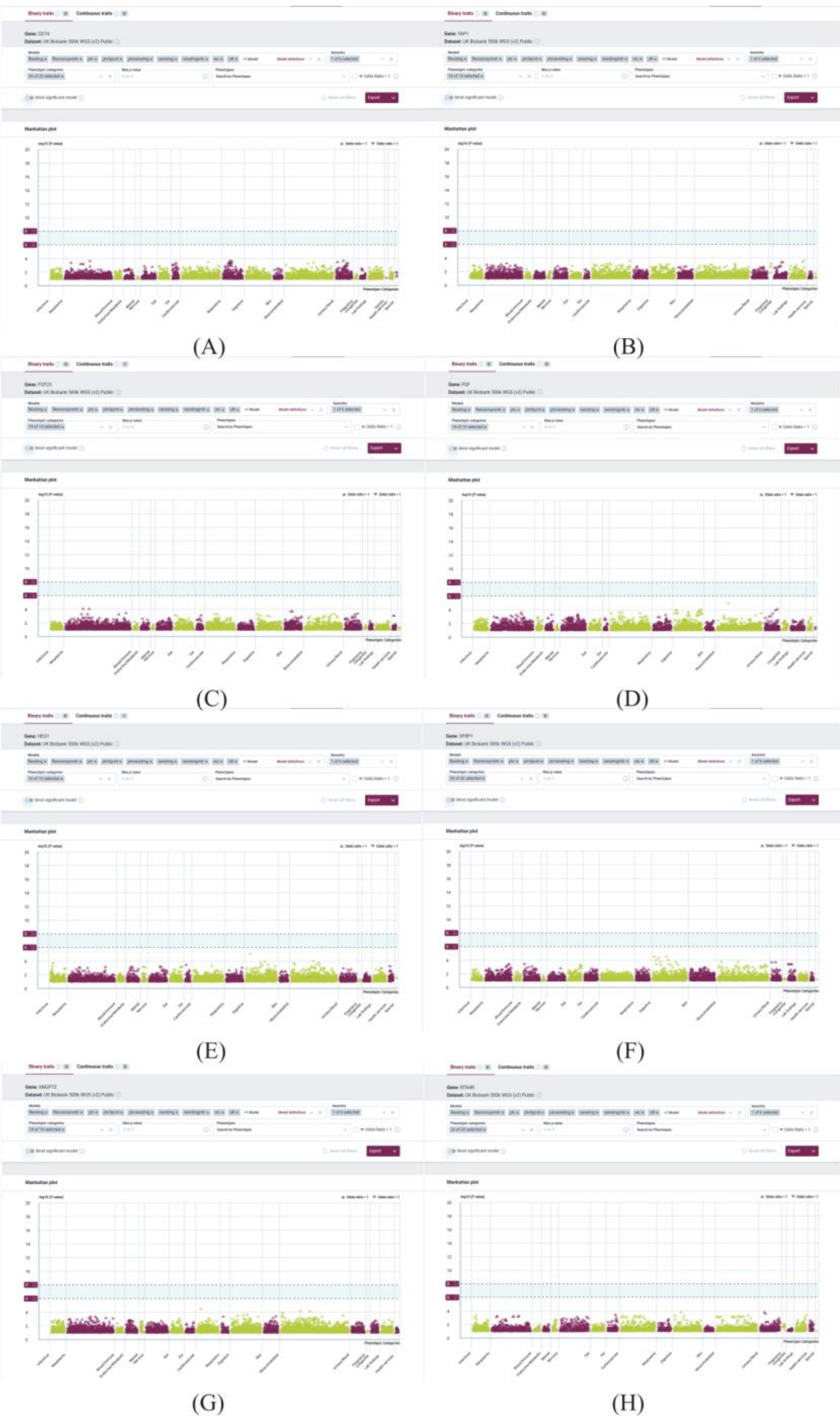

Figure S6. Phenome-Wide Association study from the AstraZeneca online portal. (A) Phenome-Wide Association study of CD74 across the full disease spectrum (continuous traits); (B) Phenome-Wide Association study of YAP1 across the full disease spectrum (continuous traits); (C) Phenome-Wide Association study of FGF23 across the full disease spectrum (continuous traits); (D) Phenome-Wide Association study of PGF across the full disease spectrum (continuous traits); (E) Phenome-Wide Association study of HEG1 across the full disease spectrum (continuous traits); (F) Phenome-Wide Association study of SFRP1 across the full disease spectrum (continuous traits); (G) Phenome-Wide Association study of ANGPT2 across the full disease spectrum (continuous traits); (H) Phenome-Wide Association study of RTN4R across the full disease spectrum (continuous traits)

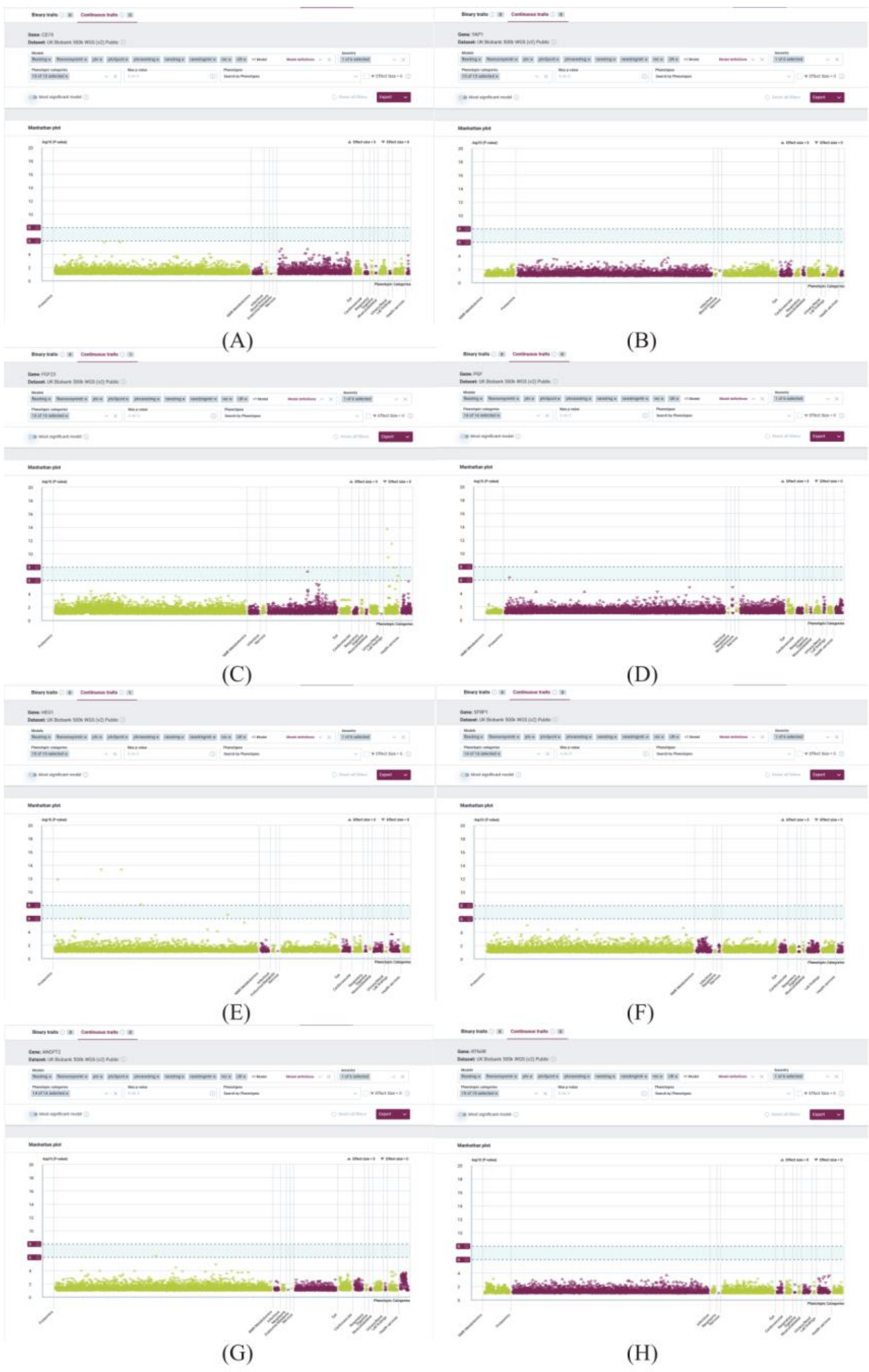

(A) (B) (C) (D) (E) (F) (G) (H)
